# Retinal pigment epithelium degeneration caused by aggregation of PRPF31 and the role of HSP70 family of proteins

**DOI:** 10.1186/s10020-019-0124-z

**Published:** 2019-12-31

**Authors:** Lourdes Valdés-Sánchez, Sofia M. Calado, Berta de la Cerda, Ana Aramburu, Ana Belén García-Delgado, Simone Massalini, Adoración Montero-Sánchez, Vaibhav Bhatia, Eduardo Rodríguez-Bocanegra, Andrea Diez-Lloret, Daniel Rodríguez-Martínez, Christina Chakarova, Shom S. Bhattacharya, Francisco J. Díaz-Corrales

**Affiliations:** 1 Regeneration and Cell Therapy Department, Andalusian Molecular Biology and Regenerative Medicine Centre-CABIMER (Junta de Andalucía), CSIC, Universidad de Sevilla, Universidad Pablo de Olavide, Avda. Americo Vespucio 24, 41092 Seville, Spain; 2 Present Address: Center for Biomedical Research (CBMR), University of Algarve, 8800-139 Faro, Portugal; 3 Present Address: Clinique de l’Oeil, Avenue Bois de la Chapelle 15, 1213 Onex, Switzerland; 4 Present Address: Center for Molecular and Cellular Bioengineering (CMCB) DFG-Research Center for Regenerative Therapies Dresden (CRTD) Cluster of Excellence, Technische Universität Dresden, Fetscherstraße, 105 01307 Dresden, Germany; 5 Present Address: Universitätsklinikum Tübingen, Forschungsinstitut für Augenheilkunde, Elfriede-Aulhorn-Str. 7, 72076 Tübingen, Germany; 6 Institute of Ophthalmology, University College London, 11-43 Bath Street, London, EC1V 9EL UK

**Keywords:** *HSP70*, *PRPF31*, Retinal degeneration, Retinal pigment epithelium, Retinitis pigmentosa

## Abstract

**Background:**

Mutations in pre-mRNA splicing factor *PRPF31* can lead to retinitis pigmentosa (RP). Although the exact disease mechanism remains unknown, it has been hypothesized that haploinsufficiency might be involved in the pathophysiology of the disease.

**Methods:**

In this study, we have analyzed a mouse model containing the p.A216P mutation in *Prpf31* gene.

**Results:**

We found that mutant Prpf31 protein produces cytoplasmic aggregates in the retinal pigment epithelium and decreasing the protein levels of this splicing factor in the nucleus. Additionally, normal protein was recruited in insoluble aggregates when the mutant protein was overexpressed in vitro. In response to protein aggregation, *Hspa4l* is overexpressed. This member of the HSP70 family of chaperones might contribute to the correct folding and solubilization of the mutant protein, allowing its translocation to the nucleus.

**Conclusions:**

Our data suggests that a mechanism haploinsufficiency and dominant-negative is involved in retinal degeneration due to mutations in *PRPF31.* HSP70 over-expression might be a new therapeutic target for the treatment of retinal degeneration due to *PRPF31* mutations.

## Background

Retinitis pigmentosa (RP) is one of a diverse group of retinal dystrophies and one of the commonest causes of inherited blindness in adults, affecting around 1:4000 individuals worldwide (Verbakel et al., 2018). RP initially presents a progressive impairment and cell death of rod photoreceptors, followed by loss of cones and retinal pigment epithelium (RPE). Clinically, RP is characterized by night blindness, which usually starts during adolescence and progresses with constriction of the visual field and a marked reduction in the amplitude of electroretinogram (ERG) waves. So far, mutations in more than 80 genes have been implicated in non-syndromic RP (Verbakel et al., 2018). Many of these genes encode for retinal-specific proteins; however, some are ubiquitously expressed, such as splicing factors *PRPF3*, *PRPF4*, *PRPF6*, *PRPF8* and *PRPF31 (Liu & Zack,* 2013*;* *Ruzickova & Stanek,* 2017*)*.

Pre-mRNA splicing is a general cellular function crucial for the expression of eukaryotic transcripts. It is catalyzed by the spliceosome, a large ribonucleoprotein complex composed of five small nuclear ribonucleoprotein complexes (Ruzickova & Stanek, 2017). In humans, *PRPF31* encodes the homolog of *S. cerevisiae* pre-mRNA processing factor 31, also known as PRPF31 protein (Vithana et al., 2001). PRPF31 is required for the U4/U6-U5 tri-snRNP formation and spliceosome activity (Makarova et al., 2002; Schaffert et al., 2004). Mutations in *PRPF31* have been described as the second most common cause of autosomal dominant RP (adRP) known as RP11 (Vithana et al., 2001; Al-Maghtheh et al., 1998; Rose et al., 2016) and, although PRPF31 is necessary for pre-mRNA splicing in every cell, adRP is the only clinical entity associated with these mutations.

Curiously, within the *PRPF31*-affected families, it is common to find asymptomatic carriers due to overexpression of the WT allele inherited from the normal parent. Therefore, the differential expression of the WT allele explains the incomplete penetrance associated with this RP locus (Rose et al., 2016; Vithana et al., 2003). It has recently been described that the expression level of *PRPF31* is regulated by the number of copies of a minisatellite repeat element-MSR1 located 200 bp upstream of the promoter. High-expressing WT alleles are found in asymptomatic carriers and low-expressing alleles are associated with the disease, where the amount of WT PRPF31 protein produced is beneath its threshold for normal function (Rose et al., 2016).

Although haploinsufficiency contributes to the physiopathology of the disease, it is still not clear how retinal degeneration occurs in patients carrying *PRPF31* mutations. To explore disease mechanisms, two animal models were previously generated (Bujakowska et al., 2009). One was a heterozygous knockout (KO) mouse (*Prpf31*^*+/−*^) and the second a knock-in (KI) mouse carrying the point mutation p.A216P (*Prpf31*^*A216P/+*^). This mutation was previously identified in RP11 patients with a severe retinal phenotype (Vithana et al., 2001). However, both heterozygous mouse models did not show any sign of photoreceptor degeneration and, as expected, the homozygous mutant mice were found to be embryonic lethal (Bujakowska et al., 2009). Based on these results, it was speculated that Prpf31 is essential for survival and the presence of one WT *Prpf31* allele is enough to maintain retinal function with no dominant-negative effect of the p.A216P mutation in mice.

More recently, it has been published that three splicing-factor mouse models (*Prpf3*^*T494M/+*^*, Prpf8*^*H2309P/+*^ and *Prpf31*^*+/−*^*)* develop late-onset morphological changes and dysfunction in the RPE rather than photoreceptor degeneration (Farkas et al., 2014; Graziotto et al., 2011). Therefore, in this work, we decided to study the effect of the p.A216P mutation on RPE. We found mislocalization and aggregation of the mutant Prpf31 protein with concomitant depletion of normal protein. These results indicate mixed haploinsufficiency and dominant-negative mechanisms involved in retinal degeneration due to mutations in *PRPF31.* Also, this work postulates HSP70 modulation as a new therapeutic target for the treatment of RP due to *PRPF31* mutations.

## Methods

### Animal handling and eye samples

Eight to sixteen-month old C57BL/6 J *Prpf31*^*+/+*^ (WT) and C57BL/6 J *Prpf31*^*A216P/+*^ (KI) mice were housed in the Biological Resources Unit of CABIMER and kept in a temperature-controlled environment (21 ± 1 °C), with a relative humidity of 55 ± 5%, a light/dark cycle 08:00–20:00 and given standard mouse chow and water ad libitum. Mouse genotyping was performed as previously described (Bujakowska et al., 2009). Due to homozygous *Prpf31*^*A216P/A216P*^ mice are not viable, we use *Prpf31*^*A216P/+*^ and *Prpf31*^*+/+*^ mice to obtain a similar proportion of KI and WT in each litter. The WT mice used as controls in each experiment belonged to the same litter of the *Prpf31*^*A216P/+*^ mice. The *rd8* mutants were discarded in these mice using the specific primers: forward 5′-GCC CCT GTT TGC ATG GAG GAA ACT TGG AAG ACA GTC ACA GTT CTT CTG-3′ and reverse 5′-GCC CCA TTT GCA CAC TGA TGA C-3’ (Mattapallil et al., 2012). A group of WT CD-1 mice were also used for the immunohistochemistry experiments.

All experiments described in this work were performed in compliance with the Spanish and European Laboratory Animal Science Association-FELASA Guide for the Care and Use of Laboratory Animals, the European Union Council Directive 2010/63/EU for the use of animals and the Association for Research in Vision and Ophthalmology-ARVO for the use of animals in ophthalmic and vision research. Animal manipulation and experimental methods have been approved by the Ethics Committee for Animal Experimentation of CABIMER, Seville, Spain. All efforts were made to minimize the number of animals used and their suffering. Pig and cow eye samples were obtained from a local slaughterhouse. Human eye sample for Western blotting was obtained from a deceased healthy donor, in a procedure approved by the Ethics Committee of University Hospital Virgen Macarena, Seville, Spain.

### Immunohistochemistry and immunofluorescence experiments

Immunohistochemistry was performed to evaluate distribution of Prpf31 protein in retinal sections of WT CD-1 mice. The animals were euthanized by cervical dislocation and the eyes excised quickly and fixed in ice-cold 4% paraformaldehyde (PFA) in PBS, overnight, at 4 °C. The fixed eyes were then cryoprotected in 30% sucrose in PBS, and embedded in optimal cutting temperature compound for cryotome sections. Serial sections of 18 μm thick were mounted in five parallel series and processed for immunohistochemistry. Briefly, the retinal sections were kept in 3% H_2_O_2_ in PBS for 30 min. Samples were then washed in 0.2% Triton X-100/PBS (PBS-T) and blocked in 1% BSA/PBS-T at room temperature for 1 h. Incubation with the primary antibody goat anti-PRPF31 (1:100; OriGene Technologies Inc., Maryland, USA, TA302582) and mouse anti-Rhodopsin (1:1000; Abcam, Cambridge, UK, ab190307) were performed overnight at 4 °C. After incubation, samples were washed 3 times in PBS-T, and incubated with appropriate biotinylated anti-goat IgG (1:500; Vector Laboratories, California, USA, BA9500) and anti-mouse IgG (1:250; Chemicon International, California, USA, AP124B) antibodies for 1 h at room temperature. The retinal sections were incubated for 1 h in avidin-biotin-peroxidase complex (1:500; Vector Laboratories). The immuno-reactive signals were visualized by 0.02% 3,3′-diaminobenzidine, 0.4% nickel ammonium sulphate and 0.005% H_2_O_2_ in 50 nM Tris-HCl buffer. Standard haematoxylin staining was performed in order to observe cell nuclei in the retinal samples. Finally, samples were dehydrated and mounted with Eukitt mounting medium (Sigma-Aldrich, Missouri, USA).

Immunofluorescence experiments were performed on eyecup sections obtained from WT and *Prpf31*^*A216P/+*^ mice. Serial sections of 18 μm thick were mounted in five parallel series and processed for immunofluorescence. After 4% PFA fixation and cryopreservation, retinal sections were incubated overnight at 4 °C with the primary antibodies: goat anti-PRPF31 (1:100; OriGene Technologies Inc., TA302582), mouse anti-RPE65 (1:100; Abcam, ab78036), rabbit anti-Laminin (1:250; Sigma-Aldrich, L9393), mouse anti-HSPA4L (1:100; Santa Cruz Biotechnology, California, USA, SC-137007) and rabbit anti-HSP27 (1:1000 Enzo Life Sciences, New York, USA, ADI-SPA-803). After incubation, samples were washed 3 times in 0.2% PBS-T, and incubated with appropriate AlexaFluor® secondary antibodies (Molecular Probes, Oregon, USA) at room temperature for 1 h. After 3 washes, sections were mounted with Vectashield mounting medium containing DAPI (Vector Laboratories). Sections of all analysed cases were processed in parallel following an identical protocol without the incubation step with the primary antibody, to be used as controls for immunoreaction specificity. To detect cholesterol accumulation, retinal sections were incubated with Filipin III (Sigma-Aldrich) for 2 h at room temperature. Whole mount of the RPE was performed as usually, and F-actin was stained with TRITC-phalloidin (Sigma-Aldrich) according to the manufacturer’s instructions.

Immunofluorescence experiments were also performed in cells grown on glass coverslips. Cells were fixed in 4% PFA and then permeabilized and blocked with 2% donkey serum/PBS-T for 1 h at room temperature. Incubation with primary antibodies: goat anti-PRPF31 (1:100; OriGene Technologies Inc., TA302582) and mouse anti-HSP70 (1:100; Santa Cruz Biotechnology, SC-24) was performed for 1 h at room temperature. Cells were washed three times with PBS-T and incubated with AlexaFluor® secondary antibodies (Molecular Probes). Coverslips were mounted on glass slides with Vectashield mounting medium containing DAPI (Vector Laboratories). Confocal images of retinal sections and cell coverslips were captured by a spectral confocal microscope TCS SP5 (Leica, Wetzlar, Germany) with an HCX PL APO Lambda blue 63 1.4 OIL objective, at 22 °C. MetaMorph Microscopy Automation and Image Analysis Software were used to analyse the images, and quantification of colocalization signal was obtained using Mander’s overlap coefficient. Adobe Photoshop CS5.1 software was used for digital amplification of the images.

### Lipofuscin staining

Retinal sections were incubated with carbol-fuchsine solution (4 g of fuchsine; 8 g of phenol, 20 mL of absolute ethanol and 100 mL of distilled water), for 1 h at room temperature. After 3 washes with distilled water, slides were cleared with alcohol acid solution (1% hydrochloric acid in 70% ethanol). The slides were then washed with tap water for 5 min and counterstained with 2% picric acid. Finally, slides were dehydrated with rising alcohol solutions and cleared with xylene.

### Transmission electron microscopy (TEM) images

Mice were anesthetized by subcutaneous injection of ketamine hydrochloride/xylazine solution (80/12 mg/kg body weight) and perfused using a fixation solution containing 2.5% of PFA and 2.5% of glutaraldehyde in PBS. Eyes were enucleated and fixed overnight at 4 °C in the same fixation solution. TEM was performed by Nanoimaging Service in BIONAND (Malaga, Spain), using a FEI Tecnai G2 29 TWIN Transmission Electron Microscope.

### Dissection of mouse neuroretina and RPE for protein and mRNA extractions

The animals were euthanized by cervical dislocation and the eyes quickly excised. The cornea was cut towards corneal limbus, using a small spring scissor. Then, the back of the eye was gently pressed to remove the lens. Four cuts were made perpendicular to the corneal limbus and towards the optic nerve head. The eye was opened in four petals, and finally the neuroretina was carefully separated from the underlying RPE-choroid using curved forceps. Samples were collected in separate microcentrifuge tubes for subsequent protein or mRNA extraction.

### Western blot

Proteins were extracted in ice-cold RIPA buffer containing protease inhibitor cocktail. Soluble/insoluble fractioning was performed as previously described (Diaz-Corrales et al., 2005). Briefly, cell lysates were incubated on ice for 60 min and the homogenates were centrifuged (19,200×*g*, 20 min at 4 °C). The supernatants (detergent-soluble fraction) were collected and the pellets (detergent-insoluble fraction) were re-suspended in resuspension buffer (60 mM Tris-HCl, 2% SDS, 2.5% 2-mercaptoethanol) and sonicated for 20 min, at 4 °C. Nuclear and cytosolic fractions were collected using the Ne-Per Nuclear and Cytoplasmic extraction reagents (Thermo Fisher Scientific). Protein content was measured by *DC*™ protein assay (Bio-Rad, California, USA) and samples stored at − 80 °C. Thirty micrograms of each extract were separated in a denaturing 10% SDS–PAGE gel and the proteins transferred to a PVDF membrane (Amersham Biosciences, Little Chalfont, UK), and blocked using Superblock Blocking buffer (Thermo Fisher Scientific, Massachusetts, USA) containing 0.1% of Tween-20 (Sigma-Aldrich) for 1 h at room temperature. The primary antibodies: anti-PRF31 (1:3000, Santa Cruz Biotechnology, SC-68347), mouse anti-Rhodopsin, (1:1000, Abcam, ab190307), mouse anti-RPE65, (1:5000, Abcam, ab78036), mouse anti-HSPA4L (1:500, Santa Cruz Biotechnology, SC-137007), mouse anti-HSP70 (1:2000, Santa Cruz Biotechnology, SC-24), mouse anti-GAPDH (1:1000, Abcam, ab9484) and mouse anti-γ-Tubulin (1:2000, Sigma-Aldrich, T-5192) were incubated overnight at 4 °C. The primary antibody mouse anti-FLAG® M2 (1:1500, Sigma, F3165) was incubated for 1 h at room temperature. The membrane was probed with the appropriate anti-HRP-conjugated secondary antibodies for 1 h at room temperature, and the immune-reactive bands were detected by chemiluminescence using ECL plus (Amersham Biosciences). Immunoreactive bands were quantified by densitometric analysis using ImageJ software, and normalized with GAPDH or γ-tubulin immunoreactive bands.

### Microarrays for gene expression analysis and alternative splicing

Eight-month old WT and *Prpf31*^*A216P/+*^ mice were sacrificed by cervical dislocation and total RNA from RPE was extracted using High Pure RNA tissue kit (Roche, Mannheim, Germany), according to manufacturer’s instructions. Quality of isolated RNA was evaluated by RNA 6000 Nano assay on a 2100 Bioanalyzer (Agilent Technologies, California, USA). The RNA extracted from RPE/Choroid samples (100 ng) was used to produce end-labeled biotinylated ssDNA. The labeled ssDNA was hybridized using oligonucleotide microarray GeneChip® MTA 1.0 (Affymetrix, California, USA), according to manufacturer’s instructions. The arrays were scanned using the GeneChip® Scanner 3000 7G (Affymetrix) and analyzed with the GeneChip® Command Console Software (Affymetrix). The raw array data were pre-processed and normalized using the Signal Space Transformation-SST Robust Microarray Analysis-RMA (Irizarry et al., 2003). Genes differentially expressed (fold change linear < − 2 or > 2 and ANOVA *p*-value < 0.05) were selected for further analysis. Gene ontology was evaluated through the Database for Annotation, Visualization and Integrated Discovery (DAVID) v6.8 (Sherman & Lempicki, 2009). For alternative splicing analysis, the data was normalized by Robust Multiarray Average-RMA and applying Detection Above the Background-DABG method. The splicing index was determined to evaluate the difference of expression of a given exon between *Prpf31*^*A216P/+*^ and WT mice, excluding the influence of gene level expression. Exons differentially expressed (splicing index = fold change linear < − 2 or > 2 and ANOVA *p*-value < 0.05) were selected for further analysis.

### RT-PCR and quantitative RT-PCR (qPCR)

Total RNA from neuroretina and RPE samples was extracted using High Pure RNA tissue kit (Roche) according to the manufacturer’s instructions. After spectrophotometric quantification of RNA using *NanoDrop1000* (Thermo Fisher Scientific), reverse transcription was carried out using cDNAQuantiTect® reverse transcription kit (Qiagen, Hilden, Germany), according to manufacturer’s instructions. cDNA amplification was performed by using 1 μg of RNA as template. Approximately 100 ng of cDNA was used for qPCR. Specific primers for *Prpf31* (Mm01329809_m1, Thermo Fisher Scientific), *Recoverin* (Mm00501325_m1, Thermo Fisher Scientific), *Rpe65* (Mm00504133_m1, Thermo Fisher Scientific) and *Hspa4l* (Mm00495441_m1, Thermo Fisher Scientific) were used. The qPCR was performed using TaqMan® Gene Expression Real Time qPCR assays (Life-Technologies, California, USA) according to the manufacturer’s instructions, using a Thermal Cycler C100 (Bio-Rad). The average cycle threshold (CT) of fluorescence units was used to analyze the mRNA levels. *Prpf31, Recoverin, Rpe65 and Hspa4l* mRNA levels were normalized by *Gapdh* RNA levels. Quantification was calculated as: mRNA levels (percent of control) = 2^Δ(CT)^ with Δ (CT) = CT_(*Prpf31/Recoverin/Rpe65/Hspa4l*)_- CT_(*Gapdh)*_.

### Funduscopy

Mouse retinas were evaluated in vivo using an advanced retinal-imaging microscope (MICRON III, Phoenix Research Laboratories, Inc., California, USA). The animals were anaesthetized by subcutaneous injection of ketamine hydrochloride/xylazine solution (80/12 mg/kg body weight) and pupils were dilated with one drop of 10% phenylephrine and 1% tropicamide. Additionally, eyes were locally anaesthetized with 0.1% tetracaine and 0.4% oxybuprocaine and a generous amount of 1% methylcellulose was placed on the mouse corneas to keep the eye moist. Correct alignment of the eye and dilatation of the pupils were checked before placing the camera lens in contact with the cornea to visualize the retina. Finally, images of the central and the peripheral regions of the retina were repeatedly captured with a three-separate charge-coupled device camera. A short wavelength excitation filter (486.5 nm transmission band Tavg N 90% 451.5) and a long wavelength emission filter (transmission band Tavg N 93% 504.7–900 nm) were used to detect autofluorescence signal.

### ERG recordings

ERG is used to measure the electrical response of retinal cells (photoreceptors, RPE cells, etc) to light stimuli. Whole field ERG was recorded in a Ganzfeld Color Dome (Diagnosys LCC). To assess scotopic vision, mice were dark adapted overnight. Anesthesia and pupil dilation of mice were performed as described above. A ring electrode made of gold wire (active electrode) was placed on the surface of the cornea which was previously treated with a wetting agent (1% methyl cellulose). Needle electrodes made of stainless steel were used as reference (forehead) and ground electrodes (tail). The narrow band filter was adjusted to frequencies of 0.312 to 300 Hz. A single flash white (6500 K) was used as stimulus divided in 6 stages of progressive intensity at 0.01, 0.05, 0.2, 1, 3 and 10 cd (cd).s/m^2^. Fifteen responses were recorded at each stage with an interval of 15 s between each stimulus. To evaluate photopic vision, mice were adapted to light for 10 min with a background illumination of 30 cd/m^2^. The intensity of the stimulus was 3, 5, 10, 15 and 20 cd.s/m^2^. The amplitude and frequency of a- and b-waves were evaluated. To measure the c-wave the narrow and broad band filters were adjusted to 0.1 Hz and 30 Hz, respectively. The c-wave value was measured at the maximum peak of the c-wave. A single green flash of 64 cd/m^2^ during 200 ms was used was used as stimulius and the recording was extended until 4 s.

### Plasmids

pEGFP-N1 (Clontech, Michigan, USA) containing the CMV promoter was used as backbone. Human *PRPF31* was amplified with specific primers containing NheI (5′) and BamHI (3′) restriction sites. *PRPF31*^*A216P*^ was obtained by using a GeneArt® Site-Directed Mutagenesis System kit (Invitrogen, California, USA). The amplified fragments were cloned in pEGFP-N1. The resulting constructs (*PRPF31-*GFP and *A216P-*GFP) were confirmed by restriction enzyme digestion and sequencing. The pcDNA3.1-PRPF31-C(K) DYK plasmid (*PRPF31*-Flag) was acquired from GenScript (New Jersey, USA).

### Cell culture

Human RPE cell line ARPE-19 (ATCC® CRL-2302™) was kept in culture at 37 °C in a humid chamber with 5% CO_2_ and grown in Dulbecco’s modified Eagle’s medium F12 (DMEM/F12; Sigma-Aldrich) supplemented with 1% penicillin/streptomycin (Sigma-Aldrich), 1% glutamine (Sigma-Aldrich), and 10% fetal bovine serum (Sigma-Aldrich). Culture medium was changed every 2 days. Transfection was performed using Lipofectamine 2000 (Invitrogen) with a 3:1 (μL of Lipofectamine 2000/μg of DNA) ratio, according to manufacturer’s instructions. Briefly, 7.5 × 10^5^ cells were seeded in a 6-cm culture dish (Orange Scientific, Belgium), and 24 h after seeding, cells were transfected with 1 μg of DNA. Twenty-four-hour post-transfection, cells were either fixed or collected for protein isolation, depending on the experiment. Cells were also transfected with the *PRPF31-*GFP or *A216P-*GFP plasmids alone and co-transfected with the *PRPF31*-Flag plasmid.

### Fluorescence recovery after bleaching (FRAP) assay

ARPE-19 cells transfected either with *PRPF31-*GFP or *A216P-*GFP constructs were used for FRAP experiments. FRAP assay was performed using a laser scanning confocal microscope TCS SP5 (Leica) equipped with an environmental control system for temperature (37 °C), humidity and CO_2_ concentration (5%). Briefly, transfected cells from each condition were selected and imaged before bleaching. Photo-bleaching was applied in a circular region of interest-ROI of the same diameter positioned in the cell nucleus of the selected cells using an Argon laser (488 nm). Pre-bleached images were recorded for 3 s (1 s/frame) and the selected area was bleached for 1 s with a pulse of 488-nm laser, at maximum intensity. After bleaching, a series of images were captured every second for 200 s. Normalization was performed using the values before bleaching and the first time point after bleaching.

### Statistical analysis

The SSPS software was used for statistical analysis. All experimental measurements were expressed as the means ± SEM or quartiles in boxplot diagrams. Normal distribution of samples was evaluated by Kolmogorov-Smirnov. The samples of both eyes were pooled in one sample for protein and mRNA extractions. Statistically significant differences between groups were estimated by *t*-test, one-way ANOVA or the nonparametric Mann-Whitney *U*-test**.** A *p* value < 0.05 was considered statistically significant.

## Results

### Prpf31^A216P/+^ KI mice display RPE degeneration with drusen-like deposits

**Fig. 1:**
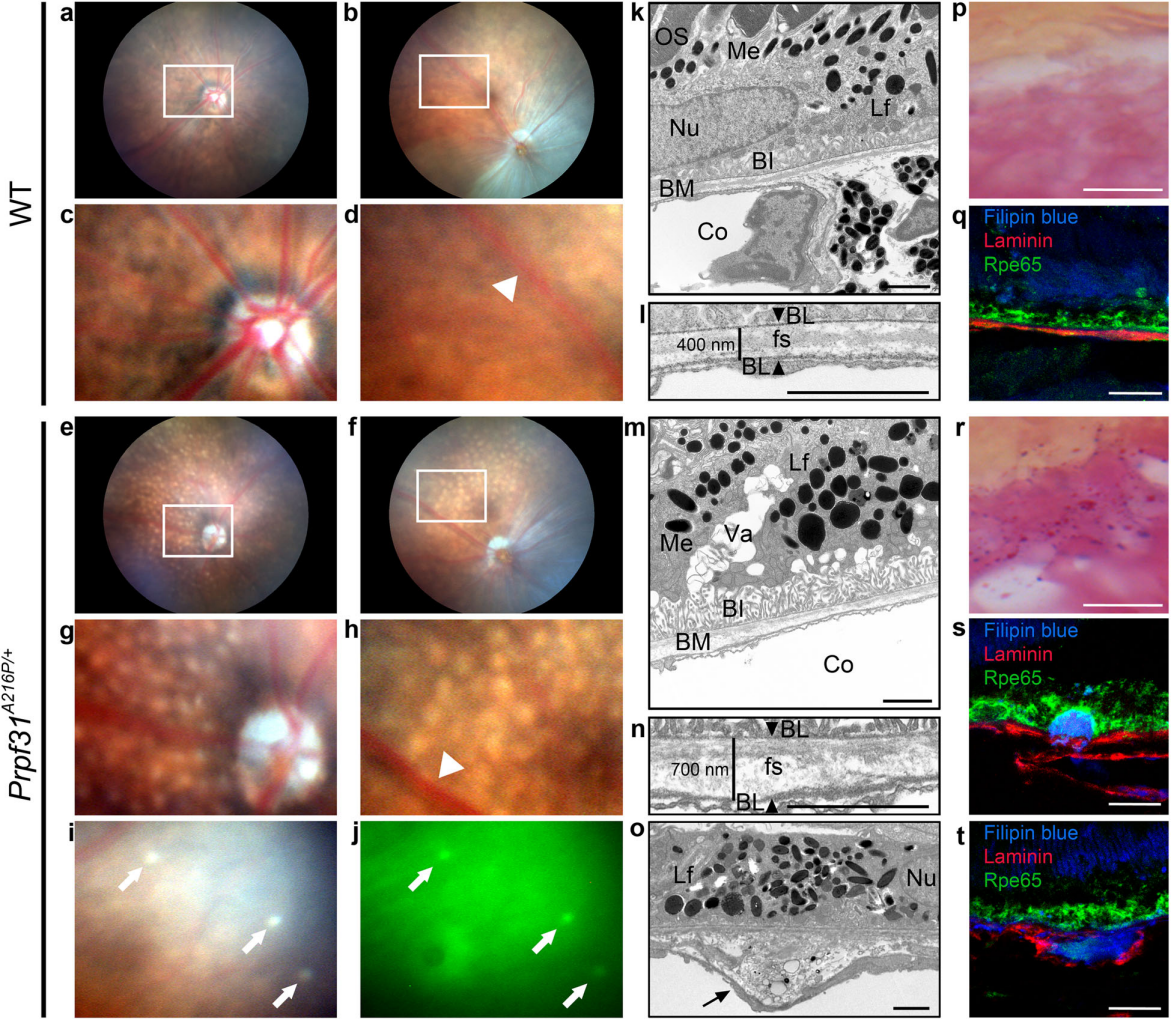
*Prpf31*^*A216P/+*^ mice exhibit degenerative phenotype of the RPE with drusen-like deposits. Funduscopy of WT (**a**-**d**) and *Prpf31*^*A216P/+*^ mice (**e**-**j**) are shown. Numerous white-yellowish round lesions were observed in the retina of *Prpf31*^*A216P/+*^ mice (**e**-**h**). These lesions were distributed in the central (**g**) and peripheral retina (**h**) and most of them showed autofluorescence (**i**, **j**; white arrows). The optic nerve head (**g**) and retinal vessels (**h**; white arrowhead) did not show differences when compared to WT mice (**c**, **d**; white arrowhead). TEM images of RPE of 8-month-old WT (**k, l**) and *Prpf31*^*A216P/+*^ mice (**m**-**o**) and the amplified images of Bruch’s membrane (BM) are displayed (**l, n, o**). Photoreceptor outer segments (OS) were observed in contact with the RPE microvilli in WT mice (**k**). Accumulation of lipofuscin granules (Lf), large vacuoles (Va) and atrophy of basal infoldings (BI) were observed in the RPE of *Prpf31*^*A216P/+*^ mice (**m**). The distance between both basal laminae (BL) was measured (**l, n**; arrowheads), and thickening of the BM was detected in *Prpf31*^*A216P/+*^ *mice* (**n**). In addition, the homogeneity of the fundamental substance (fs) was lost and amorphous electrodense material was accumulated within the BM of these mice (**o**; black arrow). The morphology of melanin granules (Me), nuclei (Nu) and choroid (Co) was normal. Lipofuscin staining in dark magenta (**p, r**) showed large accumulation of lipofuscin granules in the RPE of *Prpf31*^*A216P/+*^ mice (**r**). Filipin blue dye was used to stain free cholesterol (**q, s, t**; blue). *Prpf31*^*A216P/+*^ mice showed free cholesterol accumulation (**s, t**; blue) between the RPE and BM (**s**) or within the BM (**t**). Anti-Laminin antibodies were used to stain the BL (**q, s, t**; red) and the RPE was visualized by anti-Rpe65 antibody (**q,s, t**; green). Scale bars represent 2 μm (**k-o**) or 12.5 μm (**p**-**t**)

To better understand the role of Prpf31 in retinal degeneration, we have used heterozygous *Prpf31*^*A216P/+*^ KI mice (Bujakowska et al., 2009), a mouse model which carries the point-mutation p.A216P in the *Prpf31* gene, known to be responsible for adRP in humans (Vithana et al., 2001). However, in mice p.A216P does not produce a photoreceptor cell death phenotype as it does in humans. On the other hand, it is known that *Prpf31*^*+/−*^ KO mice display an RPE-degenerative phenotype. For this reason, we decided to characterize, in detail, the RPE-degenerative phenotype of aged KI mice. We started by studying funduscopy images in 8 to 16-month-old mutant mice and their WT-littermates to evaluate the appearance of the retina. Ophthalmoscopic images of the central and peripheral regions of the retina showed normal appearance in the WT mice (Fig. 1a-d). The homogeneity in the surface of the central (Fig. 1a) and peripheral (Fig. 1b) retina is clearly shown, as well as the normal size of the optic nerve head (Fig. 1c) and the normal thickness of the blood vessels (Fig. 1d; white arrowhead). In contrast, small round, white-yellowish, non-confluent, scattered lesions were observed throughout the retina of *Prpf31*^A216P/+^ mice like drusenoid deposits (Fig. 1e-h). Most of these lesions were autofluorescent (Fig. 1i-j; white arrows). The number of drusen-like deposits begins to be observed since the 8th month, and their number progressively increases in a non-homogeneous way during the retinal degenerative process. In the mutant mice evaluated we did not observe any of the typical fundus features of RP, such as black pigment accumulation in the form of bone spicules, vascular sharpening or optic nerve head atrophy.

TEM of 8-month-old WT (Fig. 1k, l) and KI retinas (Fig. 1m-o) was also performed to evaluate the morphology of RPE cells and the Bruch’s membrane in detail. In the WT mice, the normal expected morphology for RPE was observed, with the presence of photoreceptor outer segments (OS) in contact with RPE apical microvilli (Mv) (Fig. 1k; OS and Additional file 1: Figure S1a), melanin and lipofuscin granules in the cytoplasm (Fig. 1k; Me, Lf) as well as basal infoldings of the RPE membrane (Fig. 1 k; BI) in contact with the Bruch’s membrane (Fig. 1 k; BM). Bruch’s membrane presented a well-defined fundamental structure (Fig. 1l; fs) between each basal lamina, one corresponding to the RPE and the other to the endothelium of a choroidal vessel (Fig. 1l; BL, arrowheads). The thickness of the Bruch’s membrane measured between both basal laminae was 400 nm (Fig. 1 l). In contrast, *Prpf31*^A216P/+^ TEM images showed, accumulation of lipofuscin granules (Fig. 1m, o; Lf), vacuolization of the RPE (Fig. 1m; Va), atrophy of basal infoldings (Fig. 1m; BI) and thickening of Bruch’s membrane (Fig. 1 m; BM), to an approximate size of 700 nm (Fig. 1n). Additionally, the homogeneity of the fundamental membrane structure was lost (Fig. 1n; fs) and we also found accumulation of amorphous electrodense material within the Bruch’s membrane (Fig. 1o; black arrow). The Mv and the end of OS in mutant mice were also observed. Mv were shorter and disorganized when compared with the Mv of WT mice (Additional file 1: Figure S1). Despite all these alterations, the *Prpf31*^*A216P/+*^ mice did not show any damage of the photoreceptor.

In addition, specific staining methods were used to visualize lipofuscin granules (Fig. 1p, r). Large accumulation of lipofuscin granules was observed in the RPE of the mutant mice (Fig. 1r; dark magenta) compared to WT (Fig. 1p). To evaluate the composition and localization of the amorphous material observed in the Bruch’s membrane, Filipin blue staining was used to detect free cholesterol (Fig. 1q, s, t; blue). Immunofluorescence for laminin (Fig. 1 q, s, t; red) and Rpe65 (Fig. 1q, s, t; green) are shown as markers of basal lamina and RPE, respectively. In the KI mice, accumulation of free cholesterol was observed between the RPE and the Bruch’s membrane (Fig. 1s) or between both basal laminae (Fig. 1t). Localization of these deposits is similar to the basal linear deposits and basal lamellar deposits described by Curcio and colleague in age-related macular degeneration (AMD) (Curcio & Millican, 1999). RPE atrophy, lipofuscin accumulation and thickening of Bruch’s membrane are also features described in AMD (Curcio & Millican, 1999; Ding et al., 2009).

Finally, we have monitored ERG responses in the KI mice and found that a- and b-waves, corresponding to photoreceptor electrical activity, were not affected (Additional file 1: Figure S2a-c”). This is similar to what was previously reported for all splicing factor-mutant mouse models (Bujakowska et al., 2009; Farkas et al., 2014). Surprisingly, a defective c-wave was observed, reflecting, at a functional level, the specific degenerative changes found in the RPE layer (Additional file 1: Figure S2d-d”). Therefore, the *Prpf31*^*A216P/+*^ mice display RPE degeneration with drusen-like deposits.

### PRPF31 is highly expressed in the RPE

**Fig. 2:**
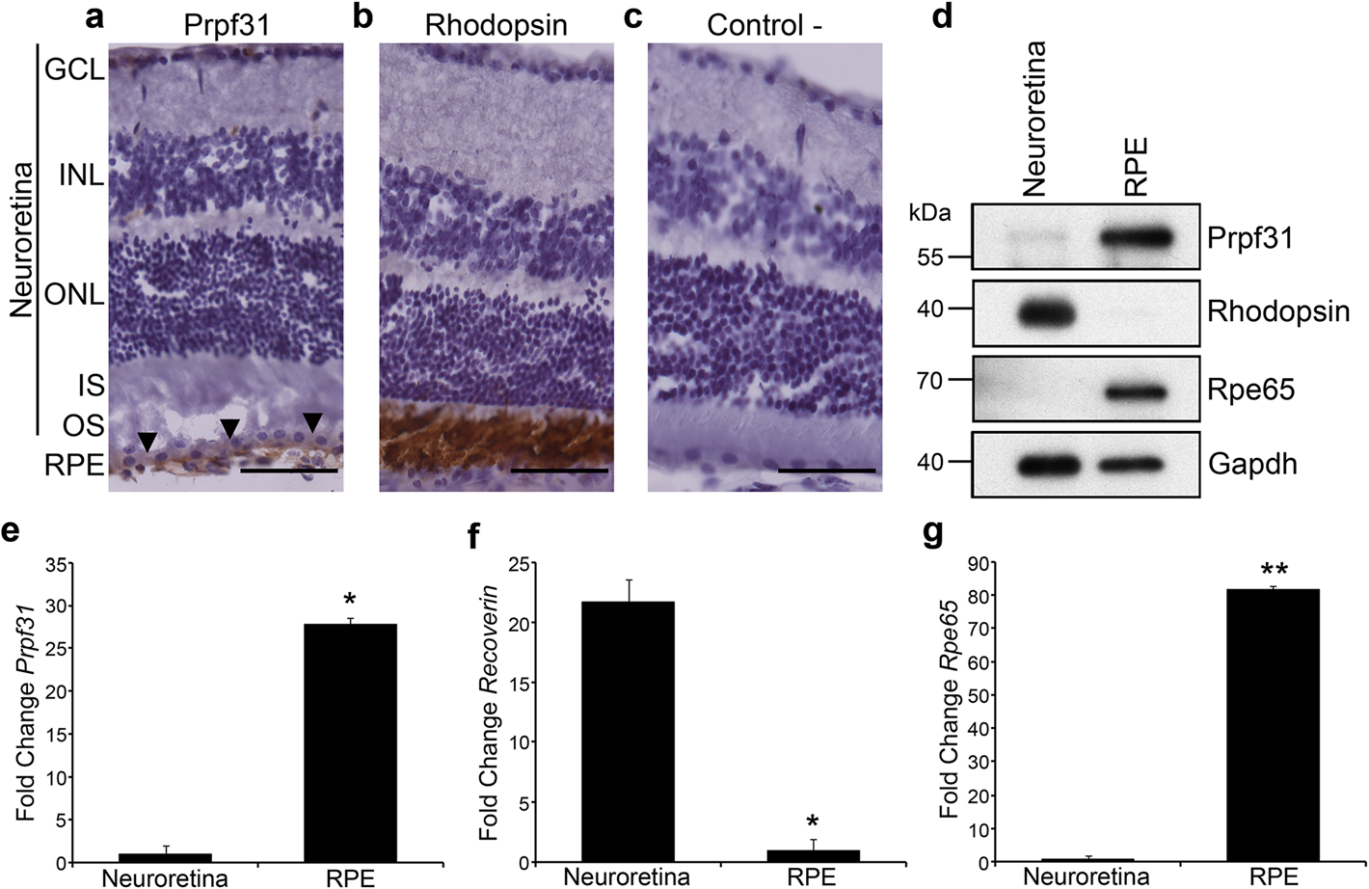
*Prpf31* protein and its mRNA are highly expressed in the RPE of mouse retinas. Immunohistochemical staining showed strong Prpf31 signal in the RPE of CD-1 mouse retinas (**a**; arrowheads). Anti-Rhodopsin antibodies were used as positive control for immunohistochemical staining (**b**). A negative control without primary antibodies is also present (**c**). Western blot (**d**) and qPCR (**e**) analysis of Prpf31 protein and mRNA expression in the neuroretina and RPE samples showed that it is mainly expressed in the RPE (**d, e**). Anti-Rhodopsin and anti-Rpe65 antibodies were used as controls for the neuroretina/RPE tissue fractions, and anti-Gapdh antibody was used as a loading control (**d**). For qPCR, *Recoverin* (**f**) and *Rpe65* (**g**) mRNA expression levels were used as controls for the two different tissue fractions. The bars in the graphs **e**-**g** represent means of fold change ± SEM (*n* = 4 replicates of 3 samples in each group). Statistically significant differences were determined by *t-*test (*p < 0.05, **p < 0.01). RPE = retinal pigment epithelium, OS = outer segment, IS = inner segment, ONL = outer nuclear layer, INL = inner nuclear layer, GCL = ganglion cell layer. Scale bars represent 50 μm

We have analysed expression of Prpf31 in retinal samples of WT CD-1 mice to examine its distribution in the different retinal layers. Immunohistochemistry (Fig. 2) shows Prpf31 to be highly expressed in the RPE cell layer (Fig. 2a; arrowheads) compared to neuroretina, where almost no Prpf31 signal was detected. Rhodopsin, the photo-pigment expressed in rod-photoreceptors, was used as a positive control for the immunohistochemical staining (Fig. 2b) and, as expected, a clear signal was displayed in the photoreceptor OS. A negative control, without primary antibody, was performed to discard non-specific binding of secondary antibody (Fig. 2c).

To compare differential expression of Prpf31 protein in different layers of the mouse retina, neuroretina and RPE were manually dissected and protein and mRNA samples were obtained from each fraction. Immunoblotting results indicate high expression of Prpf31 protein compared to the neuroretina (Fig. 2d). Antibodies against Rhodopsin and Rpe65, an enzyme of the visual cycle cascade expressed in the RPE, were used as fraction-specific markers (Fig. 2d). This result was further confirmed by qPCR (Fig. 2e), in which it is possible to observe that *Prpf31* expression level is much higher in the RPE, when compared to its expression in the neuroretina. *Recoverin* and *Rpe65* mRNAs were used as markers for neuroretina and RPE fractions, respectively (Fig. 2f-g).

To confirm whether this differential distribution of PRPF31 along the retinal cell types is common to other vertebrates, fractions of RPE and neuroretina were obtained from C57BL/6 J mice, pig, cow and human eye samples. The immunoblot for PRPF31 (Additional file 1: Figure S3) showed that protein level is comparatively higher in the RPE than in the rest of the retinal layers in several vertebrates, including humans.

### Mutant PRPF31 protein is aggregated in the cytoplasm of RPE cells

**Fig. 3:**
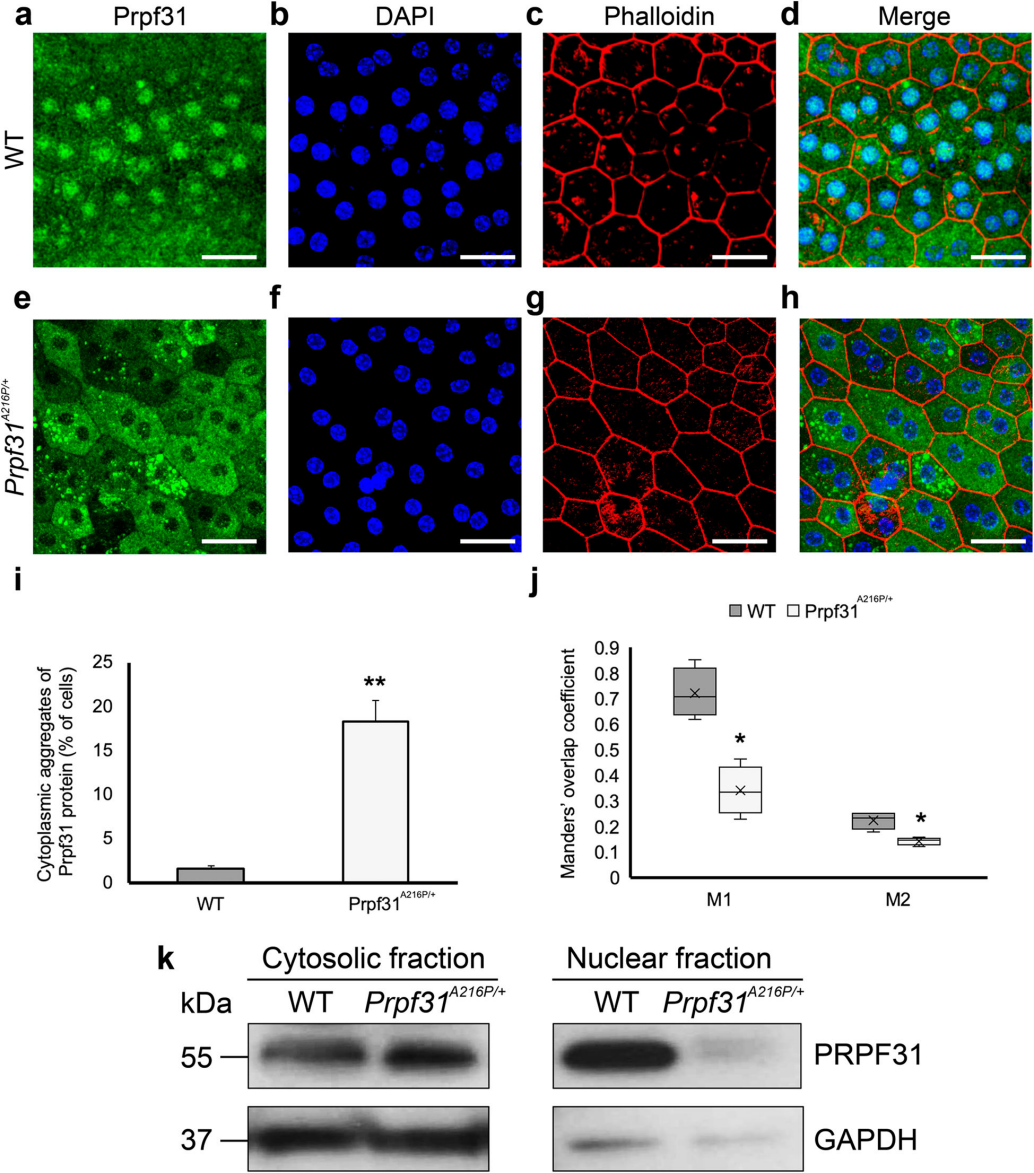
Large cytoplasmic aggregates of Prpf31 protein were observed in the RPE of *Prpf31*^*A216P/+*^ mice*.* Whole-mount of the RPE layer from WT (**a**-**d**) and *Prpf31*^*A216P/+*^ mice (**e**-**h**) were immunostained with anti-Prpf31 antibodies (**a, e**). Cell nuclei were stained with DAPI (**b, f**), and TRITC-phalloidin was used to visualize the F-actin microfilaments (**c, g**). Prpf31 signal was mainly localized in the nuclei of WT RPE cells (**a**), while large protein aggregates stained for PRPF31 were observed in the cytoplasm of RPE cells of mutant *Prpf31*^*A216P/+*^ mice (**e**). The bars in graph **i** represent the percentage of RPE cells with cytoplasmic aggregates of Prpf31 protein ± SEM in WT and *Prpf31*^*A216P/+*^ whole mount RPE samples (*n* = 1200 cells were counted from 4 mice in each group). The boxplot **j** represents the Manders’ overlap coefficient of DAPI+Prpf31/DAPI colocalization (M1) and Prpf31 + DAPI/Prpf31 colocalization (M2) in WT and *Prpf31*^*A216P/+*^ whole-mount RPE samples (*n* = 4 in each group). Expression of Prpf31 protein was evaluated in the cytosolic and nuclear fractions by Western blot (**k**). Statistically significant differences were determined by *t-*test or Mann-Whitney *U*-test (*p < 0.01, **p < 0.001). Scale bars represent 25 μm

Next, we were interested in the distribution of the Prpf31 protein histologically in the RPE of the aged-KI mice. Immunofluorescence analysis of Prpf31 protein did not show clear differences between WT and KI mice in retinal sagittal sections. However, RPE whole-mount evaluation showed large cytoplasmic aggregates of Prpf31 protein in *Prpf31*^*A216P/+*^, which were almost not present in the WT samples (Fig. 3e-h). Additionally, a difference in the Prpf31 distribution within the RPE cell was observed, with a weaker staining in the KI nuclei compared to the WT-littermates (Fig. 3e-h). In the WT RPE cells, most of Prpf31 is localized inside the nucleus with some homogeneous cytoplasmic staining (Fig. 3a-d) but in the *Prpf31*^*A216P/+*^ RPE cells, most of the Prpf31 staining is shown in the cytoplasm forming rounded clumps of Prpf31 protein resembling aggregates (Fig. 3e-h). Cells that were observed harbouring protein aggregates formed clusters in the RPE layer of the KI mice. The number of cells with cytoplasmic aggregates was counted in both KI and WT mice, providing a statistically significant difference, with *Prpf31*^*A216P/+*^ mice having 18.4 ± 2.3% of RPE cells with cytosolic aggregates compared to 1.7 ± 0.2% cells in the WT mice (Fig. 3i).

To quantify the amount of Prpf31 signal in the RPE nuclei, the Manders’ overlap coefficient for DAPI+Prpf31/DAPI colocalization (M1) and for Prpf31 + DAPI/Prpf31 colocalization (M2) were calculated (Fig. 3j). Both coefficients were significantly lower in the mutant mice (Fig. 3j), corresponding to the diminished amount of Prpf31 protein in the nucleus of RPE cells as observed by histology. Besides, Western blot of cytosolic and nuclear fractions clearly showed a decrease of Prpf31 protein in the nuclear fractions of mutant mice (Fig. 3k). Therefore, these results show that not only Prpf31 protein in the *Prpf31*^*A216P/+*^ mice is aggregated in the cytoplasm of the RPE cells, but also its concentration in the nuclei is decreased when compared to the WT. The antibody used to visualize Prpf31 recognized both mutant and normal Prpf31 protein, thus we were unable to determine whether the aggregates are composed solely of the mutated protein or if the WT protein is also present in the aggregates.

### Differential gene expression and alternative splicing were affected in Prpf31^A216P/+^ mice

**Fig. 4:**
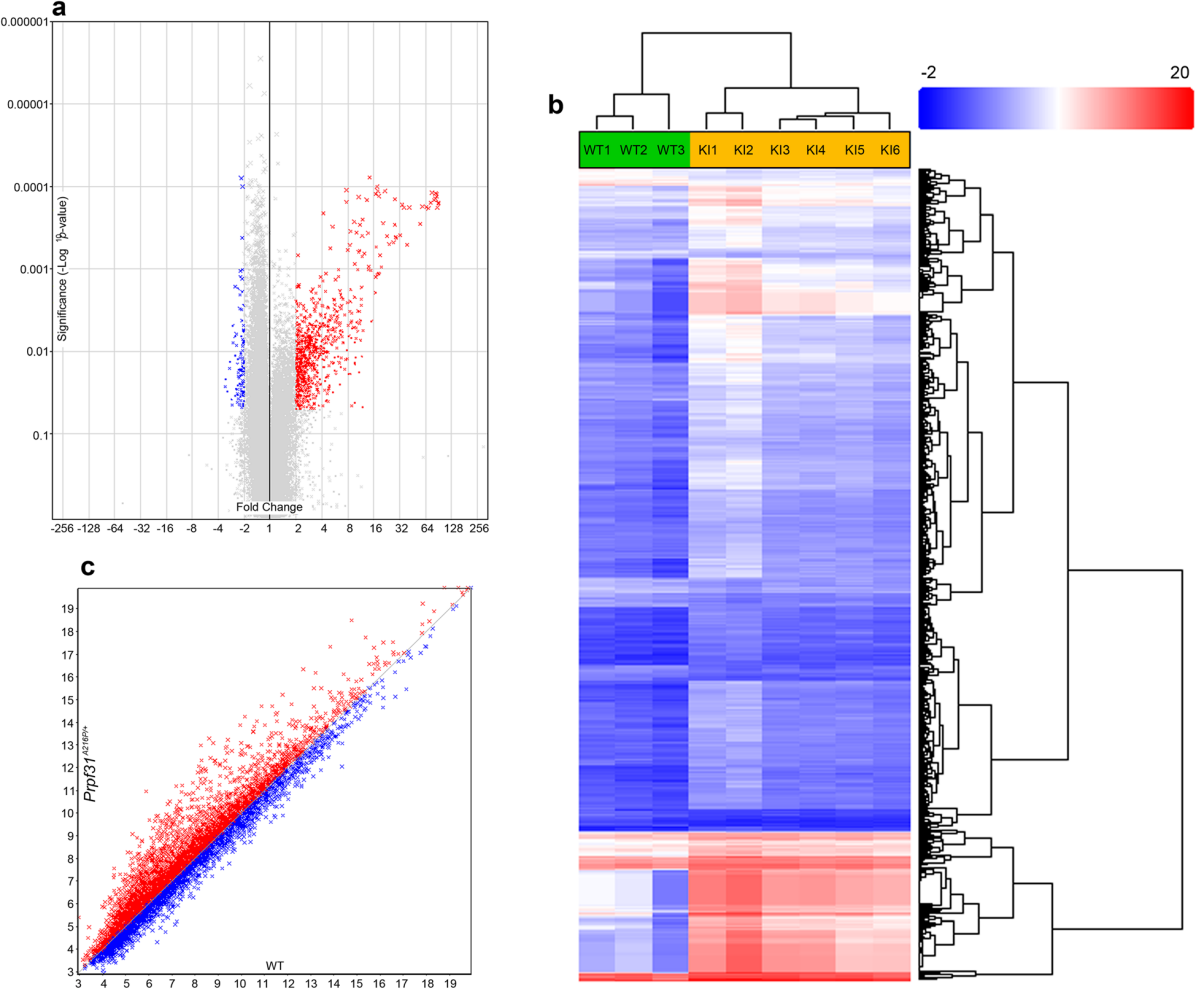
Differences in the level of gene expression and alternative splicing in the RPE of *Prpf31*^*A216P/+*^ mice compared to WT controls. Volcano plot (**a**) showing the genes that are upregulated (red) or downregulated (blue) in *Prpf31*^*A216P/+*^ KI *mice* with a significance ANOVA *p* value < 0.05 and fold change < −2 or > 2 when compared with WT mice. Hierarchically clustered (**b**) genes (rows) and WT or KI mice (columns) with dendrograms and flat clusters; red in the heatmap denotes upregulation while blue denotes downregulation. Scatter plot (**c**) presents the genes that are alternatively spliced in *Prpf31*^*A216P/+*^ mice with lower (red) or higher (blue) splicing index when compared with WT mice (WT *n* = 3 and *Prpf31*^*A216P/+*^ *n* = 6)

Considering that the mutant Prpf31 protein is aggregated in the cytoplasm of RPE cells in *Prpf31*^*A216P/+*^ mice, we decided to perform transcriptomic analysis using a GeneChip™ Mouse Transcriptome Array (MTA) 1.0 to evaluate differential gene expression in RPE samples of six *Prpf31*^*A216P/+*^ and three WT-littermates. The number of genes evaluated was 65,956 and, from these, a total of 1033 (1.6%) genes were differentially expressed in the *Prpf31*^*A216P/+*^ mice. Most of these genes were upregulated (922, 89.3%) and the rest were downregulated (Table 1; Additional file 2, Gene expression). The gene-level differential expression analysis is graphically displayed in the volcano plot (Fig. 4a). Each point on the plot represents the statistical result of a single gene. Horizontal axis represents fold change in log2 scale and vertical axis represents *p*-value in log10 scale. Threshold of fold change was either < − 2 (Fig. 4a; blue) or > 2 (Fig. 4 a; red) and ANOVA *p*-value < 0.05. Gray dots correspond to the genes without statistically significant change. Hierarchical clustering of 1033 genes differentially expressed in *Prpf31*^*A216P/+*^ vs WT mice is shown in Fig. 4b with an expression profile clearly different for WT and *Prpf31*^*A216P/+*^ clusters (Fig. 4b).

**Table 1:** Summary of gene level differential expression analysis in RPE samples in two different conditions (*Prpf31*^*A216P/+*^ vs WT mice). Default filter criteria, fold change < − 2 or > 2 and ANOVA *p*-value < 0.05

Type of genes	Number of genes evaluated (%)	Number of genes differentially expressed (%)	Number of genes up-regulated (%)	Number of genes down-regulated (%)
Non-coding	36,703 (55.7)	562 (0.9)	485 (47.0)	77 (7.5)
Complex	15,644 (23.7)	280 (0.4)	264 (25.6)	16 (1.5)
Coding	10,692 (16.2)	151 (0.2)	134 (12.9)	17 (1.6)
Pseudogene	2711 (4.1)	40 (0.1)	39 (3.8)	1 (0.1)
Ribosomal	155 (0.2)	0 (0.0)	0 (0.0)	0 (0.0)
Unassigned	51(0.1)	0 (0.0)	0 (0.0)	0 (0.0)
Total	65,956 (100.0)	1033 (1.6)	922 (89.3)	111 (10.7)

MTA 1.0, genome version mm10

Gene ontology was evaluated through the informatic tool DAVID v6.8 (Sherman & Lempicki, 2009) using the list of 1033 genes which were differentially expressed in *Prpf31*^*A216P/+*^ mice, showing that the largest group of genes (123 genes) affected by the mutation belongs to *Protein Binding* (Additional file 1: Table S1). This molecular function is defined as: interacting selectively and non-covalently with any protein or protein complex (GO:0005515), including the subcategory chaperone binding (GO:0051087); a class of proteins that bind to nascent or unfolded polypeptides to ensure correct folding or transport. Because the most relevant change observed in the RPE of the mutant mice was the cytoplasmic aggregation of Prpf31 protein, we decided to look for candidate genes related to molecular chaperones involved in protein folding. We found that *heat shock protein family A (Hsp70) member 4 like* gene (*Hspa4l*), which encodes the chaperone heat shock 70 kDa protein 4 L (Hspa4l), was upregulated in *Prpf31*^*A216P/+*^ mice (fold change 2.26; *p*-value 0.009). Other chaperones involved in the unfolded protein response were not highlighted with a different gene expression level. Lists of candidate genes found to be differentially expressed which might also be involved in RPE degeneration are shown in Additional file 1: Table S2.

Alternative splicing analysis was also performed through the GeneChip™ MTA 1.0 in the same RPE samples of six *Prpf31*^*A216P/+*^ and three WT-littermates. A total of 65,770 genes were evaluated and 92.6% (60871) of these genes were expressed in both mice (*Prpf31*^*A216P/+*^ and WT mice). From these 60,871 genes, 6700 (11%) genes have, at least, one differentially expressed probe selection region or junction to indicate alternative splicing (Table 2; Additional file 3, Splicing). The scatter plot (Fig. 4c) displays the number of genes that are alternatively spliced in *Prpf31*^*A216P/+*^ mice with lower < − 2 (Fig. 4c; red) or higher > 2 (Fig. 4 c; blue) splicing index, when compared to WT mice.

**Table 2:** Summary of alternative splicing analysis in RPE samples in two different conditions (*Prpf31*^*A216P/+*^ vs WT mice). Default filter criteria, splicing index < −2 or > 2 and ANOVA *p*-value < 0.05

Type of genes	Number of genes evaluated (%)	Number of genes expressed in both conditions (%)	Genes with at least one differentially expressed PSR or junction to indicate alternative splining (%)
Non-coding	36,581 (55.6)	33,587 (51.1)	929 (1.5)
Complex	15,644 (23.8)	15,056 (22.9)	4344 (7.1)
Coding	10,628 (16.2)	9450 (14.4)	1381 (2.3)
Pseudogene	2711 (4.1)	2601 (3.9)	29 (0.1)
Ribosomal	155 (0.2)	155 (0.3)	16 (0.0)
Unassigned	51(0.1)	22 (0.0)	1 (0.0)
Total	65,770 (100.0)	60,871 (92.6)	6700 (11.0)

MTA 1.0, genome version mm10. *PSR* probe selection region

Functional categories of alternative spliced genes in the RPE of *Prpf31*^*A216P/+*^ mice are listed in the Additional file 1: Table S3. We observed that several splicing factors, including *Prpf31*, present a different splicing index (*Prpf31* splicing index − 2.33, *p*-value 0.04; *Prpf18* splicing index − 2.62, *p*-value 0.01; *Prpf39* splicing index 2.25, *p*-value 0.03). Apart from the aforementioned splicing factors, others genes of different pathways involved in retinal degeneration were also affected by the mutation, such as inflammation, oxidative stress, retinol metabolism (*Abca4*), ciliogenesis (*Bbs1, Bbs4, Bbs5, Bbs7, Bbs9*) and cellular apoptosis (Additional file 1: Table S4; Additional file 3, Splicing). The number of candidate genes with modified splicing index that can be involved in RPE degeneration are detailed in Additional file 1: Table S4. These results suggest that normal splicing of different genes, including splicing factors, is affected in the RPE of *Prpf31*^*A216P/+*^ mice.

### Increased Hspa4l expression and colocalization with mutant Prpf31 protein in the RPE of Prpf31^A216P/+^ mice

**Fig. 5:**
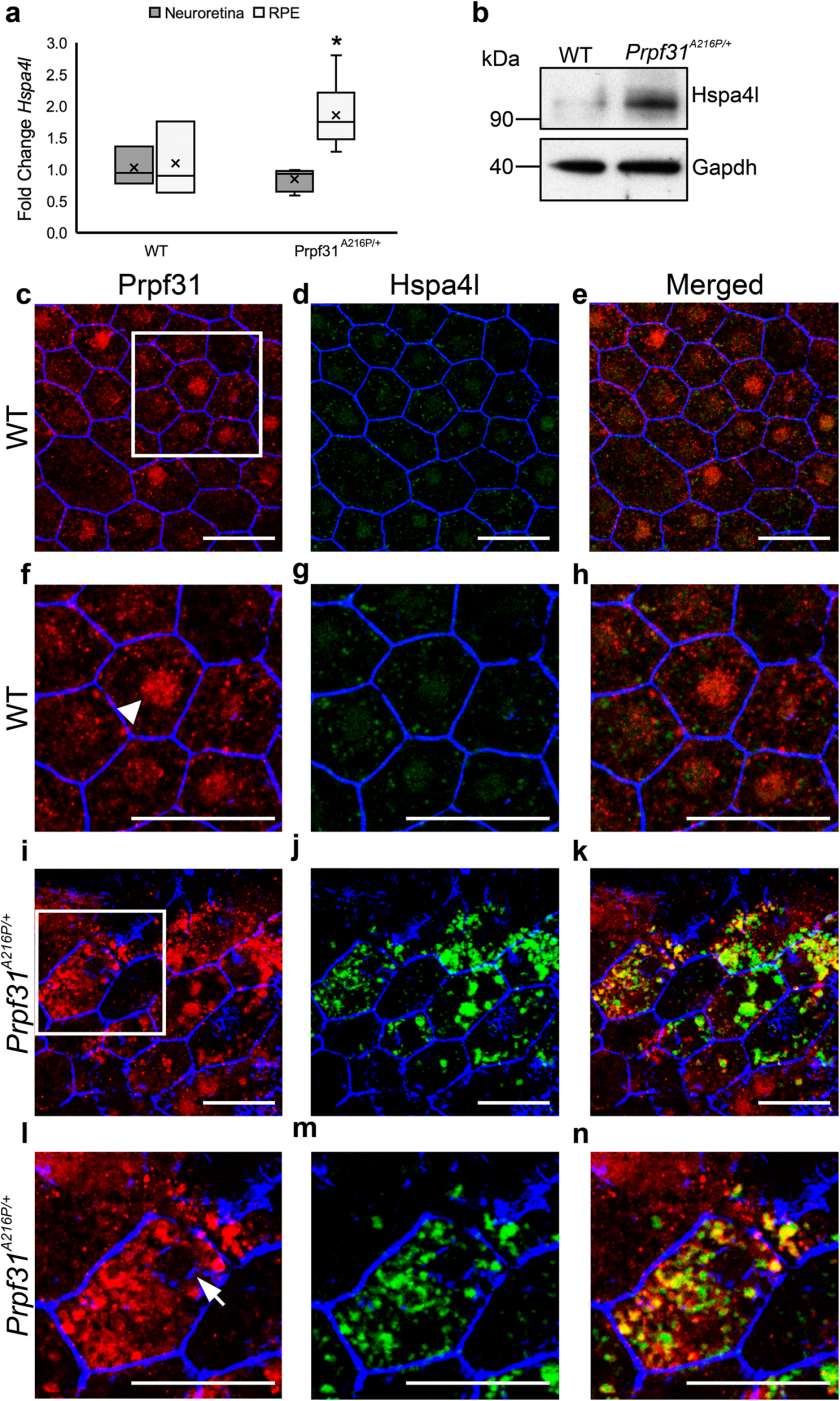
*Hspa4l* is highly expressed in the RPE of *Prpf31*^*A216P/+*^ mice. Analysis of *Hspa4l* expression by qPCR in the neuroretina and RPE samples show that *Hspa4l* mRNA is overexpressed in the RPE of *Prpf31*^*A216P/+*^ mice (**a**). The boxplot **a** represents the fold change of *Hspa4l* expression in the neuroretina and RPE of WT and *Prpf31*^*A216P/+*^ mice (WT *n* = 3 and *Prpf31*^*A216P/+*^ *n* = 6). Statistically significant differences were determined by Mann-Whitney *U*-test (*p < 0.05). Western blot of RPE samples showed higher expression of Hspa4l protein in *Prpf31*^*A216P/+*^ mice compared to WT (**b**). Anti-Gapdh antibodies were used as loading control (**b**). Whole-mount of the RPE obtained from WT (**c-h**) and *Prpf31*^*A216P/+*^ mice (**i-n**) were immunostained with anti-Prpf31 (**c, f, i, l**) and anti-Hspa4l antibodies (**d, g, j, m**). TRITC-phalloidin was used to stain F-actin microfilaments (**c-n**; blue). Magnified images are (**f-h and l-n**) and merged are shown (**e, h, k, n**) Prpf31 signal was mainly distributed in the nuclei of RPE cells in WT mice (**c, f**; arrowhead), while Prpf31 protein aggregates were observed in the cytoplasm (**i, l**) colocalizing with Hspa4l signal in mutant *Prpf31*^*A216P/+*^ mice as shown in the merged images (**k, n**). Prpf31 signal was very low in the nuclei of *Prpf31*^*A216P/+*^ mice (**l**; arrow). Scale bars represent 25 μm

Analysis of transcriptomic data show that a member of the heat shock protein 70 (HSP70) family, *Hspa4l*, was overexpressed in the RPE of KI mice. The HSP70 family is a ubiquitous and conserved family of molecular chaperons assisting in protein folding to prevent aggregation and to protect cells from stress (Mashaghi et al., 2016; Mayer & Bukau, 2005). We have analyzed mRNA expression of *Hspa4l* by qPCR in both RPE and neuroretina of the KI mice and we found that *Hspa4l* is overexpressed in the RPE of the mutant mice, when compared to its expression in the WT mice (Fig. 5a). No differences of *Hspa4l* expression in the neuroretina extracts were observed (Fig. 5a). This result was corroborated by Western blot in which we observed that Hspa4l was more abundant in the RPE of the mutant mice (Fig. 5b). RPE whole-mount immunofluorescence to localize Hspa4l and Prpf31 was also performed. As previously mentioned, Prpf31 protein predominantly localizes in RPE nuclei of WT samples (Fig. 5c, f; arrowhead) and in cytoplasmic aggregates in the case of KI tissue and low expression in the nucleus (Fig. 5i, l; arrow). As expected, Hspa4l staining is stronger in the mutant RPE cells, where the chaperone colocalizes with Prpf31 protein aggregates (Fig. 5i-n). The hexagonal shape of the RPE can be seen with phalloidin staining (Fig. 5c-n; blue). Negative controls for RPE autofluorescence and secondary antibodies nonspecific bindings are shown in the Additional file 1: Figure S4. The small heat shock protein, Hsp27, also colocalized with the Prpf31 aggregates in the RPE of mutant mice (Additional file 1: Figure S4), but transcriptomic data did not show differential expression of its gene (*Hspb1)*.

### The p.A216P mutant protein produces insoluble cytoplasmic aggregates, recruits endogenous PRPF31 protein in the insoluble fraction and increases the expression of HSP70

**Fig. 6:**
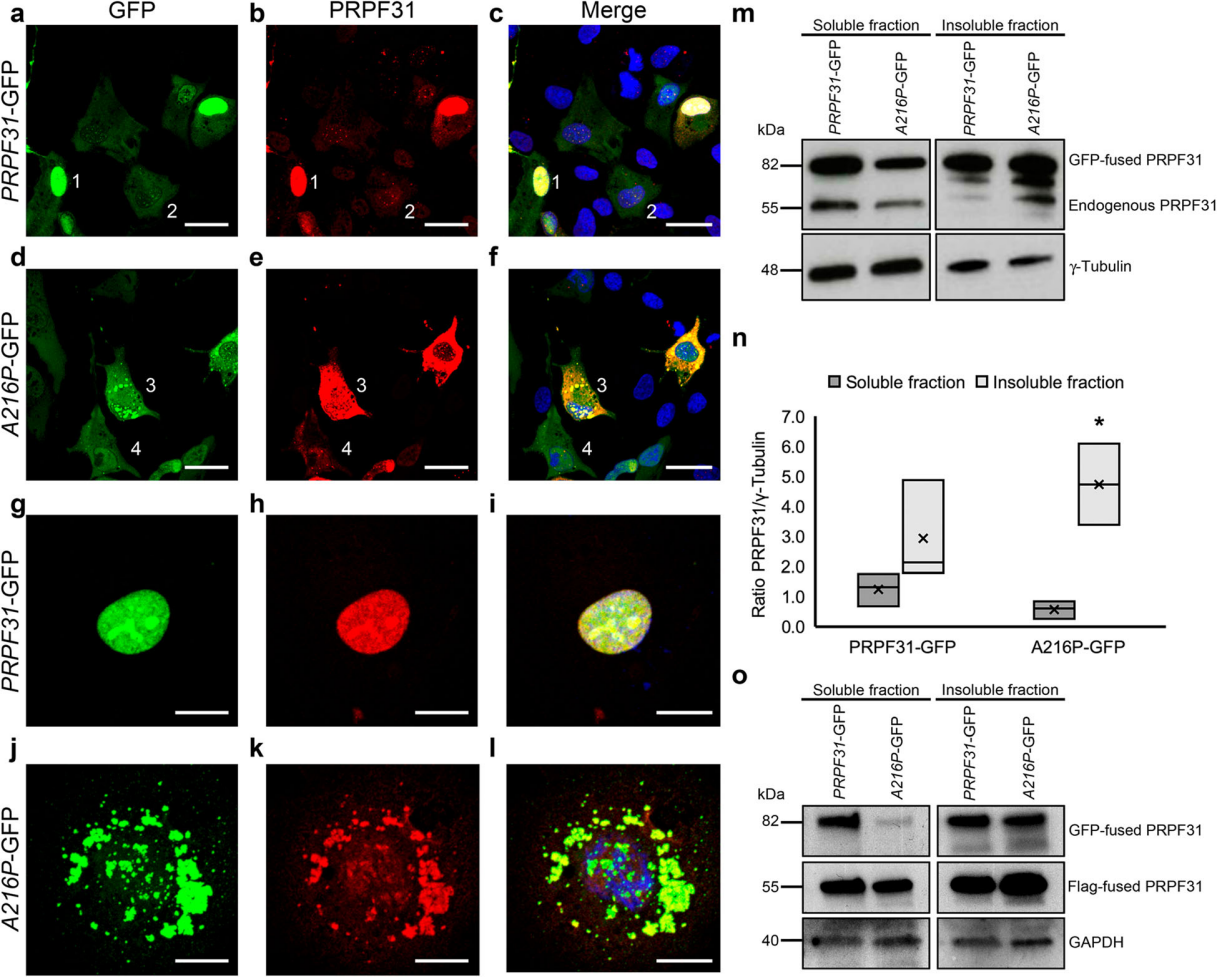
Overexpression of *A216P-*GFP induces aggregation of PRPF31 protein. The ARPE-19 cell line was transfected with *PRPF31*-GFP (**a**-**c, g**-**i**) and *A216P*-GFP (**d**-**f, j**-**l**). The GFP-tagged proteins (green) and immunostained cells with anti-PRPF31 antibodies (red) are shown. PRPF31-*GFP* was found mainly in the cell nucleus (**a**-**c; 1, g**-**i**) and to a lesser extent, in the cytoplasm (**a**-**c; 2**). *A216P-*GFP transfected cells present PRPF31 aggregation in the cytoplasm (**d**-**f; 3, j**-**l**), and a very minor signal in the nucleus (**d**-**f**; **4**). Images correspond to a maximum projection of a Z-stack. Western blot analysis of soluble and insoluble fractions of the transfected cells show a decrease in the concentration of endogenous PRPF31 in the detergent-soluble fraction and an increase in the detergent-insoluble fraction of the cells transfected with *A216P-*GFP. Anti-γ-tubulin antibody was used as loading control (**m**). Densitometry quantification of the blots (**n**) shows a significant increment of *A216P-*GFP protein in the detergent-insoluble fraction when compare with the among of *A216P-*GFP protein present in the soluble fraction (**n**). The boxplot **n** represents the ratio PRPF31/γ-tubulin in the soluble and insoluble fractions of *PRPF31*-GFP and *A216P*-GFP transfected ARPE-19 cells (*n* = 3 in each group). Statistically significant differences were determined by Mann-Whitney *U*-test (*p < 0.05). Western blot analysis of soluble and insoluble fractions of *PRPF31*-GFP and *A216P*-GFP transfected ARPE-19 cells and co-transfected with WT PRPF31 tagged to Flag showed a decrease in the concentration of WT PRPF31-Flag in the detergent-soluble fraction and an increase in the detergent-insoluble fraction of the cells co-expressing the mutant *A216P-*GFP protein. Anti-GAPDH antibody was used as loading control (**o**). Scale bars (**a**-**f**) represent 25 μm and (**g**-**l**) 10 μm

To better understand the role of p.A216P mutation in human-derived RPE cells*,* we have cloned the human WT *PRPF31* and the *A216P* mutant genes in pEGFP-N1 plasmid and the GFP-tagged PRPF31 proteins were overexpressed in ARPE-19 cells. We observed that WT *PRPF31*-GFP was mainly found in the nucleus of the transfected cells (Fig. 6a-c and g-i), while the mutant *A216P-*GFP was mostly aggregated in the cytoplasm of the transfected cells (Fig. 6d-f and j-l), similar to our previous observation in the RPE of the WT and *Prpf31*^*A216P/+*^ mice, as shown in Fig. 3.

Western blot analysis of detergent-soluble and detergent-insoluble fractions of the cell lysates was conducted to estimate the concentration of PRPF31 in both fractions. Western blotting and densitometry analysis of the immunoblots (Fig. 6m, n) show less mutant protein *A216P-*GFP in the soluble fraction compared to *PRPF31*-GFP protein, and the opposite in the detergent-insoluble fraction (Fig. 6m, n). Additionally, it is possible to observe a depletion of the endogenous PRPF31 protein in the soluble fraction and highest levels of expression in the insoluble fraction (Fig. 6m), suggesting that the cytoplasmic insoluble aggregates of *A216P-*GFP protein recruit the endogenous WT PRPF31 protein. To evaluate whether the WT PRPF31 protein increased in the insoluble fraction in cells co-transfected with the mutant protein PRPF31 (A216P), ARPE-19 cells were co-transfected with WT PRPF31 tagged to Flag (PRPF31-Flag) and *PRPF31-*GFP or *A216P*-GFP. After 24 h of incubation, the protein extracts of soluble and insoluble fractions were analyzed by Western blotting (Fig. 6o). It was observed that the WT protein tagged to GFP was expressed in both the soluble and insoluble fraction, while the mutant protein was expressed mostly in the insoluble fraction (Fig. 6o). Besides, it was found that the WT protein tagged to Flag decreased in the soluble fraction and increased in the insoluble fraction only in those cells expressing the PRPF31 protein carrying the p.A216P mutation. In this way, we confirm our hypothesis that the mutant PRPF31 protein recruits the WT protein in the insoluble fraction.

**Fig. 7:**
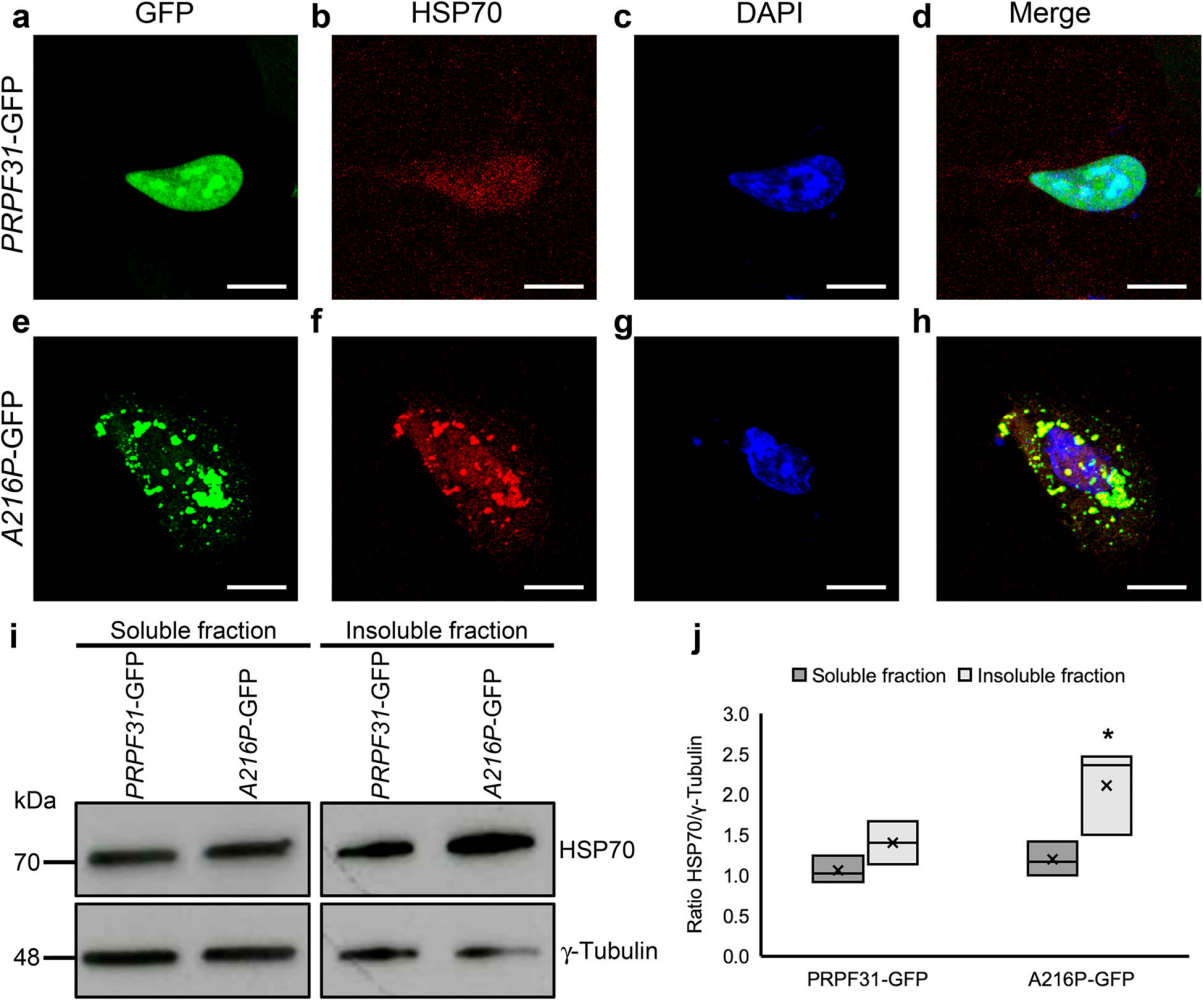
Overexpression of A216P-GFP induces HSP70 activation in ARPE-19 cells. Immunostaining of cultured ARPE-19 cells transfected with *PRPF31*-GFP (**a**-**d**) or*A216P*-GFP (**e**-**h**) displays PRPF31 aggregation in the cytoplasm of the cells overexpressing *A216P*-GFP (green) and colocalization of HSP70 (red) in the aggregates (**e**-**h**). Images correspond to a maximum projection of a Z-stack. Western blot analysis (**i**) and densitometry quantification (**j**) of the soluble and insoluble fraction of the transfected cells showing an increment of HSP70 concentration in the detergent-insoluble fraction of the cells transfected with *A216P*-GFP. Anti-γ-tubulin antibody was used as loading control (**i**). The boxplot **j** represents the ratio HSP70/γ-tubulin in soluble and insoluble fraction of *PRPF31*-GFP and *A216P*-GFP transfected ARPE-19 cells (*n* = 3 in each group). Statistically significant differences were determined by Mann-Whitney *U*-test (*p < 0.05). Scale bars represent 10 μm

HSP70 plays an important role in retinal dystrophies, including RP (Furukawa & Koriyama, 2016) so we have also evaluated the distribution and expression of HSP70 protein in the ARPE-19 cells overexpressing either *PRPF31-*GFP or *A216P*-GFP by immunofluorescence and Western blotting. In the immunofluorescence staining, *PRPF31-*GFP transfected cells show a weak labeling of HSP70 in the nucleus (Fig. 7a-d). However, in *A216P*-GFP transfected cells, HSP70 staining is increased, and its signal colocalizes with the cytoplasmic aggregates of mutant PRPF31 protein (Fig. 7e-h), similar to the distribution observed for Hspa4l in the RPE of *Prpf31*^*A216P/+*^ mice. Additionally, Western blot analysis of the soluble and insoluble fractions (Fig. 7i) and its densitometry analysis (Fig. 7j) show an increase of HSP70 in the insoluble fraction of the cells transfected with *A216P*-GFP. Therefore, protein aggregation of mutant PRPF31 seems to activate the response of chaperones.

### P.A216P mutation affects the mobility of PRPF31 protein to the nucleus in human RPE derived live cells

**Fig. 8:**
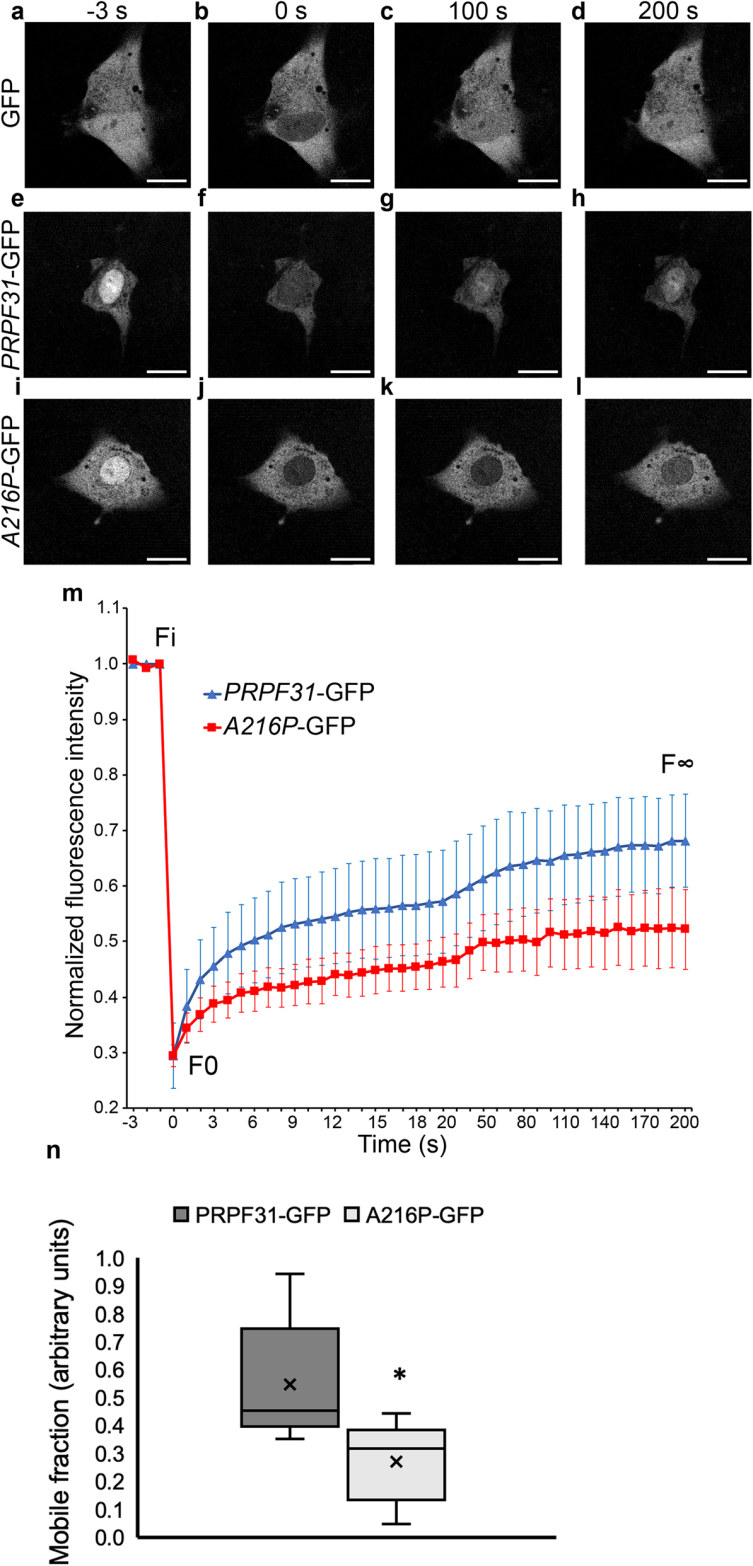
Mobility of PRPF31 protein to the nucleus is affected by the p.A216P mutation. ARPE-19 cells transfected with the pEGFP-N1 plasmid alone or carrying the open reading frame of human WT *PRPF31* (*PRPF31*-GFP) or carrying the point mutation p.A216P (*A216P*-GFP) were imaged − 3 s pre-photobleaching (**a, e, i**) and 0 s (**b, f, j**), 100 s (**c, g, k**) and 200 s (**d, h, l**) post-photobleaching. The recovery curve (**m**) indicates that the cells transfected with mutant *A216P*-GFP have a smaller mf than those transfected with *PRPF31*-GFP (**n**). The boxplot **n** represents the normalized fluorescence intensity and mobile fraction in *PRPF31*-GFP and *A216P*-GFP transfected ARPE-19 cells (*n* = 5 in each group). Statistically significant differences were determined by Mann-Whitney *U*-test (*p < 0.05). Fi = fluorescence before bleaching, F0 = fluorescence just after bleaching, F∞ = fluorescence in the bleached region after full recovery. Scale bars represent 10 μm

FRAP experiments provide qualitative and quantitative information about the mobility of a fluorescently tagged protein in a defined compartment (Phair & Misteli, 2001; Reits & Neefjes, 2001). Different parameters can be evaluated by FRAP, such as the mobile fraction (mf) of a fluorescent-tagged protein and its half-life (τ_1/2_). The mf can be affected by different circumstances, such as interaction of the fluorescent-tagged protein with other proteins, cytoplasmic organelles or membranes. Considering that p.A216P mutation induces protein aggregation and expression of HSP70, which colocalizes with the cytoplasmic aggregates of PRPF31 protein, we decided to investigate whether the translocation of PRPF31 protein from the cytoplasm to the nucleus was affected in living cells. To test this hypothesis, we have performed FRAP assay in ARPE-19 cells (Fig. 8). The cell line was transfected with the plasmids pEGFP-N1, *PRPF31*-GFP and *A216P*-GFP and FRAP was performed as described in the Methods section of this manuscript. Pre- and post-photobleached cells are displayed in Fig. 8a-l, showing that the amount of PRPF31 protein recovered in the nucleus 200 s after bleaching is affected by the mutation (Fig. 8l) but not in pEGFP-N1 (Fig. 8d) or *PRPF31*-GFP (Fig. 8h) transfected cells. A slower recovery curve for *A216P*-GFP were clearly observed when compared to the control *PRPF31-*GFP (Fig. 8m).

The changes produced in the recovery curve were due to changes in the size of mf and not due to changes in the τ_1/2_, which is defined as the time when the recovery of fluorescence intensity is half of the plateau (τ_1/2_ *PRPF31*-GFP *=* 7.40 ± 3.08 s; τ_1/2_ *A216P*-GFP = 7.00 ± 2.66 s; *p* = 0.46). However, the mf significantly decreased in *A216P*-GFP transfected cells (mf *PRPF31*-GFP *=* 0.55 ± 0.10; mf *A216P*-GFP = 0.27 ± 0.15; *p* < 0.05) (Fig. 8n). The mf was determined by comparing the fluorescence in the bleached region after full recovery (F∞) with the fluorescence before bleaching (Fi) and just after bleaching (F0), being defined as mf = (F∞ – F0)/(Fi – F0). The fluorescence intensity was normalized to the Fi and F0 in the Fig. 8m. The *A216P*-GFP transfected cells showed less F∞ than the control (Fig. 8m). These results suggest that p.A216P mutation decreases the amount of PRPF31 protein that moves to the nucleus but without affecting the diffusion time.

## Discussion

*PRPF31* is a ubiquitously expressed gene encoding for a component of the spliceosome complex involved in pre-mRNA processing. Mutations in this gene are associated with non-syndromic adRP, but the mechanism by which retinal degeneration occurs, is still unknown. Previously, two mutant mouse models (*Prprf31*^*+/−*^ and *Prpf31*^*A216P/+*^) were generated to study the role of PRPF31 in the pathogenesis of adRP, but neither of these models presented evidences of RP-like photoreceptor degeneration, leading to conclude that the presence of one copy of WT-*Prpf31* allele is sufficient to maintain the normal retina, and that the p.A216P mutation does not exert a dominant-negative effect (Bujakowska et al., 2009).

It has previously been described that the *Prprf31*^*+/−*^ KO mice (Farkas et al., 2014; Graziotto et al., 2011), as well as other splicing-factor mouse models that do not present photoreceptor-degenerative phenotype, have an RPE-degenerative phenotype. In this study, a deep phenotypic characterization of 8 to 16 month-old *Prpf31*^*A216P/+*^ mice was performed to understand how the p.A216P mutation affects RPE function and we found evidence that supports a combination of haploinsufficiency and dominant-negative effect. Fundus analysis showed a severe RPE degeneration, with the presence of white-yellowish and autofluorescent spots in mutant mice. Concomitant functional impairment was detected in ERG c-wave. TEM images accordingly showed some typical features of degenerative RPE such as vacuolization, atrophy of basal infoldings, thickening of Bruch’s membrane and accumulation of amorphous electrodense material within this membrane. Two previous studies (Farkas et al., 2014; Graziotto et al., 2011) reported that two-year-old *Prpf3*^*T494M/+*^ and *Prpf8*^*H2309P/+*^ and the one-year-old *Prpf31*^*+/−*^ mice had similar features to the ones that we have found in 8-month-old *Prpf31*^*A216P/+*^ mice. The earlier onset of RPE degeneration in *Prpf31*^*A216P/+*^ might indicate a possible toxic effect of the p.A216P mutant protein.

In addition to the RPE atrophy, we have also observed accumulation of lipofuscin granules and drusen-like deposits of free cholesterol between the RPE and the Bruch’s membrane or between both basal laminae. These deposits are similar to the basal linear deposits and basal lamellar deposits described in AMD patients (Curcio & Millican, 1999). RPE atrophy, lipofuscin accumulation and thickening of Bruch’s membrane are characteristic histological hallmarks found in human AMD patients, and these features have been also described in animal models of RPE degeneration (Curcio & Millican, 1999; Pennesi et al., 2012). Therefore, the *Prpf31*^*A216P/+*^ mice display a primary RPE degeneration phenotype with drusen-like deposits. Although RP phenotype is predominantly associated with *PRPF31* mutations in humans, some affected individuals developed juvenile macular degeneration apart from the typical RP phenotype, as described in two Chinese families carrying different mutations in the *PRPF31* gene (Lu et al., 2013; Xi et al., 2005). These results suggest that mutations in the PRPF31 gene could produce not only a characteristic RP phenotype in humans but also a phenotype with an early macular degeneration. Similar results have been observed with mutations in another gene such as *RDS,* which encodes the photoreceptor glycoprotein peripherin. Mutations in *RDS* can produce both a clear RP phenotype and also macular dystrophy (Wells et al., 1993). Although no single nucleotide polymorphism in *PRPF31* gene has been described in AMD, it has been suggested that alterations in normal mRNA splicing could contribute to the pathophysiology of age-related diseases such as AMD (Li et al., 2017). In the *Prpf31* mutant mice, we observed that the splicing of several genes is compromised. Among these genes, the *ABCA4* gene stands out. *ABCA4* is a gene expressed mainly in photoreceptors but also RPE having a major function in retinol metabolism (Lenis et al., 2018). Mutations in this gene have been found to cause Stargardt’s disease, a hereditary juvenile macular degeneration, and AMD (Baum et al., 2003). Besides, it has been reported that *ABCA4* splicing can be also affected by aging and in AMD cases (Li et al., 2017; Baum et al., 2003; Meshorer & Soreq, 2002). For these reasons, we suggest that primary RPE degeneration phenotype with drusen-like deposits present in these *Prpf31* mutant mice could be due to splicing defects in genes that could be associated with macular degenerative diseases such as *ABCA4*.

It is known that the RPE maintains structural integrity and function of photoreceptor cells and defends the retina from free radicals and photo-oxidative damage (Simo et al., 2010; Strauss, 2005). A failure in any of the RPE roles might induce retinal degeneration and loss of visual function. Two classical examples of RP due to mutations in RPE-specific genes are *MERTK* and *RPE65*, which cause RP (Gal et al., 2000) and Leber’s congenital amaurosis (Gu et al., 1997), respectively. Thus, p.A216P *PRPF31* mutation might affect photoreceptors due to a primary defect in the RPE as *MERTK* or *RPE65* mutations do.

As stated before, in humans, mutations in PRPF31 are associated with photoreceptor-cell degeneration and loss. The reason why *Prpf31*^*A216P/+*^ mice have RPE-degeneration rather than photoreceptor-cell loss is not clear, but we have found that gene expression level for *Prpf31* and the corresponding protein amount is much higher in the RPE than in the neuroretina, although the nuclei in the neuroretina greatly outnumber the nuclei in the RPE monolayer. This was also observed in mouse, pig, cow and human samples. Yuan and co-workers have studied the expression of *Prpf31* in different mouse tissues including the retina (Yuan et al., 2005). Although no differences were observed either in the expression level or pattern of Prpf31 protein among different tissues, they did not separate the neuroretina from the RPE as we have done in this study and in fact, their in situ hybridization results clearly show a much higher expression of *Prpf31* in the RPE layer. The differential expression of *Prpf31* could give us clues to understand why the RPE specifically degenerates in these mice. The elevated requirement of Prpf31 protein level in the RPE could be related to high or specific splicing demands in an epithelium that fulfils many different roles: light absorption, transport of ions, water, and metabolic end-products from the subretinal space to blood, maintenance of photoreceptors and re-isomerization of all-*trans*-retinal into 11-*cis*-retinal. Thus, defects in splicing might have a great impact on the normal function and survival of RPE cells.

To explore the role of *Prpf31* p.A216P mutation in the RPE, the distribution pattern of the protein was analysed by immunofluorescence staining of whole-mount RPE samples and Western blot of cytosolic and nuclear fractions from WT and *Prpf31*^*A216P/+*^ mutant mice. As expected, we found Prpf31 preferentially localized in the nucleus of RPE cells in WT mice, although it was also possible to observe a cytoplasmic fraction. On the other hand, in the mutant mice almost no Prpf31 was found within the nucleus, and most of the Prpf31 staining was present in the cytoplasm forming rounded clumps of Prpf31 protein resembling aggregates. The distribution of RPE cells having Prpf31 aggregates was not uniform, and we do not have an explanation for this finding. Nevertheless, our results are in accordance with the ones published by Deery and co-workers (Deery et al., 2002), in which they found that overexpression of A194E and A216P mutations in COS-7 cells induced mislocalization of the mutant PRPF31 proteins, distributed throughout the cytoplasm and with less intense staining in the nucleus when compared with WT-PRPF31 transfected cells. Also, Huranová and colleagues described similar results overexpressing A216P in HeLa cells. They found mislocalization of the mutant protein causing depletion of PRPF31 from Cajal bodies, where splicing takes place, and described a possible negative effect due to an abnormal interaction of A216P with its partner, PRPF6 (Huranova et al., 2009). It is important to note, that the Prpf31 protein detected in these mice corresponds to the endogenous WT and mutant forms of the protein. Thus, the aggregation that is found in the RPE of these mutant mice is not an artefact resulting from Prpf31 overexpression. In *Prpf31*^*A216P/+*^ mice, aggregation of the mutant protein completely depletes Prpf31 protein in the nucleus and, in this way, a combination of a dominant-negative effect and haploinsufficiency might contribute to RPE degeneration.

Although the exact mechanism underlying RPE degeneration in the mutant mice is not well understood yet, our transcriptomic analysis show that 1.6% of evaluated genes are differentially expressed in the mutant RPE when compared to WT controls. As expected, alternative splicing was also affected in the RPE of these mutant mice. Around 11% of evaluated genes have, at least, one differentially expressed probe selection region or junction, indicative of alternative splicing. From the pool of affected genes, we selected *Hspa4l* for a detailed study*,* a member of the HSP70 family of chaperons that was upregulated in the mutant RPE. The HSP70 family is a ubiquitous and conserved family of molecular chaperons, part of the cellular machinery for protein folding that prevents aggregation and protects cells from stress (Mashaghi et al., 2016; Furukawa & Koriyama, 2016). Several reports have shown that HSP70 and also small heat shock proteins play an important role in retinal dystrophies, including RP (Saliba et al., 2002), glaucoma (Nagashima et al., 2011; Park et al., 2001; Schallenberg et al., 2012) and AMD (Alge et al., 2002; Lee et al., 2011; Nakata et al., 2005). Our qPCR analysis showed increased expression of *Hspa4l* in the mutant RPE when compared with its WT control. We further confirmed this finding by Western blot analysis depicting a higher amount of protein in the mutant RPE. Moreover, immunofluorescence staining showed colocalization of Hspa4l signal with aggregates of Prpf31 protein in the RPE cytoplasm of mutant mice. This suggests that Hspa4l can be acting as a chaperone for the mutant protein in response to its aggregation. However, the aggregation of Prpf31 in the RPE of mutant mice could also activate other chaperones belonging to the family of small heat shock proteins such as Hsp27.

In addition, several other genes and signalling pathways were found to have an altered splicing in the RPE of mutant mice, including those associated with inflammation, oxidative stress, retinol metabolism and cellular apoptosis Most of these pathways are commonly affected in RPE degenerative diseases such as AMD and mutations of some genes including in this pathways such as *ABCA4* cause macular degeneration (Lenis et al., 2018; Makarev et al., 2014). In line with these results, our histological findings in the mutant RPE correlate with some macular degenerative features. Apart from that, another group of genes affected by the A216P mutation in RPE are involved in ciliogenesis. This result is in agreement with the most recent results of Buskin and co-workers in which they show that iPS-derived RPE cells from RP11 patients present shorter Mv and primary cilia, loss of polarity, reduced barrier function and defective phagocytic capacity, when compared to iPS-derived RPE cells from healthy donors, suggesting that these distorted cellular characteristics result from alternative splicing in RP11 (Buskin et al., 2018). We observed that one group of proteins affected by differential splicing in *Prpf31*^*A216P/+*^ mice are some splicing factors, including Prpf31. Four main causes are known to induce protein aggregation: mutations, errors in protein synthesis including splicing defects, environmental factors such as oxidative stress and aging. Therefore, if the Prpf31-protein level in the RPE nucleus is beneath its threshold for normal function, the mRNA splicing of *Prpf31* gene could be affected producing an aberrant protein prone to aggregation, reinforcing the dominant-negative effect and haploinsufficiency.

To explore the role of Hsp70 family in the RPE affected by a p.A216P mutation, we overexpressed both WT *PRPF31* and mutant *A216P* tagged to GFP in a human-derived RPE cell line (ARPE-19). Immunofluorescence staining results are in line with the in vivo results, with WT *PRPF31*-GFP protein being located mainly in the nucleus, and mutant *A216P*-GFP protein being mostly aggregated in the cytoplasm. By Western blot analysis we found that cells overexpressing the mutant variant have a significant decrease in the soluble fraction of PRPF31 and an increase in the insoluble PRPF31 protein. Moreover, the overexpression of the mutant protein leads to a depletion of the soluble endogenous PRPF31 protein and WT PRPF31 tagged to Flag as well, suggesting a dominant-negative effect. These results are in accordance with one of the mechanisms proposed by Yin and coworkers in which they report that mutations in *PRPF31* can induce retinal degeneration (Yin et al., 2011). They proposed three mechanisms: i) haploinsufficiency due to loss of function of the mutant protein or degradation of mutant mRNA by nonsense-mediated mRNA decay, thus compromising the splicing machinery; ii) mutant proteins with dominant-negative activity that may interfere with splicing and potentially with other cellular activities, leading to degeneration of the affected tissue; and iii) mutations might promote proteins forming insoluble and cytotoxic aggregates that can affect the tissue by loss-of-function and dominant-negative effects (Yin et al., 2011). We also observed that Hsp70 was upregulated in the cells overexpressing *A216P*-GFP and colocalized with PRPF31 mutant protein. These results corroborate our in vivo findings of Hspa4l upregulation in the RPE of *Prpf31*^*A216P/+*^ mice and its colocalization with PRPF31 protein aggregates.

Considering that PRPF31 plays its role in the nucleus and p.A216P induces PRPF31 aggregation in the cytoplasm of RPE cells, we decided to analyze how PRPF31 translocation to the nucleus was affected using FRAP assay. PRPF31 contains a classical nuclear localization sequence (NLS) between residues 351 and 364. In a previous study published by Wilkie, et al. (Wilkie et al., 2006) demonstrated that p.A216P mutations did no affect the interaction with importin β1. However, FRAP studies in the green monkey kidney fibroblast-like COS-7 cells of WT *PRPF31-GFP* and mutant *A216P-GFP* transfected cells indicated the presence of two-component recovery processes, a fast component for free diffusion within an unbounded compartment and a slow component for passive diffusion through the nuclear pores. The kinetics of both components were not affected by the p.A216P mutation (Wilkie et al., 2006). Therefore, seems that the mutation p.A216P did not affect the NLS and its interaction with importin β1 but the total amount of nuclear PRPF31 is affected probably because there is lest amount of cytoplasmic PRPF31 that can cross the nuclear pores through passive diffusion. We conclude in our FRAP experiments that p.A216P mutation decreases the amount of PRPF31 protein that moves to the nucleus but without affecting the diffusion time. We found that translocation of mf of PRPF31 to the nucleus significantly decreases in ARPE-19 cells transfected with A216P-GFP. Our results are partially in line with the studies by Wilkie and co-workers who showed a similar FRAP pattern in COS-7 cells, although they could not find a statistically significant delay in mutant protein translocation to the nucleus when compared with the WT protein (Wilkie et al., 2006). This can be an artifact caused by the cell line, since it has been shown that impaired splicing due to *PRPF31* mutations is limited to retinal cells (Buskin et al., 2018). These authors have reported that alternative spliced transcripts due to RP11 are only present in retina and RPE of the *Prpf31*^+/−^ mutant mice but not in other tissues such as brain or muscle suggesting that a specific and highly regulated splicing program takes place in the retinal cells. One of the most interesting findings of these authors (Buskin et al., 2018) with respect to our study is the fact that some alternatively spliced forms of PRPF31 were found in RPE but not in the neuroretina of the mutant KO mouse, supporting our finding that lack of PRPF31 in the nuclei of RPE cells could affect its own splicing.

## Conclusion

Our findings of a substantial higher amount of PRPF31 in the RPE compared to the neuroretina, exhibiting a degenerative phenotype of the RPE with drusen-like deposits, correlates with the growing amount of data supporting a specific splicing program in retinal cell types that is severely distorted by PRPF31 mutations. Our results suggest that p.A216P mutation promotes a damaging alternative splicing of PRPF31, generating a dominant-negative effect in which aberrant splicing of other genes and PRPF31 mutated protein aggregation are involved. Depletion of functional PRPF31 protein from the nucleus will also contribute to deficient splicing, thus mixing haploinsufficiency with a dominant-negative effect. In response to protein aggregation of PRPF31, HSPA4L chaperon is activated and recruited to the aggregates, to fold the aggregated protein and facilitate its translocation to the nucleus. A pharmacological or genetic modulation of HSP70 family of proteins could be a promising therapeutic target for RP caused by *PRPF31* mutations.

## Supplementary information

**Additional file 1: Figure S1.** TEM images of RPE of 8-month-old WT (**a**) and *Prpf31*^*A216P/+*^ mice (**b**). Photoreceptor outer segments (OS) were observed in contact with the RPE microvilli (Mv) in WT mice (**a**). In *Prpf31*^*A216P/+*^ mice OS and Mv are also observed; however, they looked shorter and disorganized (**b**). RPE nuclei (Nu), melanin granules (Me), basal infoldings (BI), Bruch’s membrane (BM) and choroid (Co) are also displayed. Scale bars represent 5 μm. **Figure S2.** ERG recordings of WT and *Prpf31*^*A216P/+*^ *mice.* Rod response and quantification of b-wave amplitude in dark adapted mice (**a-a”**). Cone-rod response and quantification of a-wave in dark adapted mice (**b-b”**). Cone response and quantification of b-wave in light adapted mice (**c-c”**). RPE response and quantification of c-wave in dark adapted mice (**d-d”**). The boxplots **a”-d”** represent the a, b and c-wave amplitude in dark or light adapted WT and *Prpf31*^*A216P/+*^ mice (*n* = 6 in each group). Statistically significant differences were determined by Mann-Whitney *U*-test (*p < 0.05). Vertical scale bars represent 300 μV. Horizontal scale bars represent 100 ms (**a-c’**) and 1 s (**d-d’**). **Figure S3.** Western blot analyses of PRPF31 expression in the neuroretina and RPE of C57BL/6 mice, pig, cow and human samples. The amount of PRPF31 protein is higher in the RPE than in the neuroretina in all the cases. Anti-γ-tubulin antibody was used as loading control. **Figure S4.** Whole-mount of the RPE obtained from WT (**a-i**) and *Prpf31*^*A216P/+*^ mice (**j-l**). Negative controls of RPE autofluorescence (**a-c**) and secondary antibodies nonspecific bindings are shown (**d-f**). The RPE of WT *Prpf31*^*A216P/+*^ mice were immunostained with anti-Prpf31 (**g, j**) and anti-Hsp27 antibodies (**h, k**). TRITC-phalloidin was used to stain F-actin microfilaments (**a-l; blue**). Merged images are shown (**c, f, i, l**). Prpf31 protein aggregates were observed in the cytoplasm colocalizing with Hsp27 signal in mutant *Prpf31*^*A216P/+*^ mice (**j-l**). Z-stack of a ARPE-19 cell transfected with *PRPF31-*GFP (**m-o**). In the Z-stacks is possible to observe that *PRPF31*-GFP signal is in the nucleus and not in the top of the nucleus. Scale bars represent 25 μm. **Table S1.** Gene ontology of differentially expressed genes in *Prpf31A216P/+* mice. The top 20 terms of 84 are listed. **Table S2.** Candidate genes differentially expressed in the RPE of *Prpf31A216P/+* vs WT mice. Default filter criteria, fold change < -2 or > 2 and *p*-value < 0.05. **Table S3.** Functional categories of genes having alternative splicing in *Prpf31A216P/+* mice. The top 40 terms of 174 are listed. **Table S4.** Number of candidate genes showing alternative splicing in the RPE of *Prpf31A216P/+* vs WT mice. Default filter criteria, splicing index < -2 or > 2 and ANOVA *p* value < 0.05.

**Additional file 2. **Results of transcriptome array (MTA) 1.0 to evaluate differential gene expression in RPE samples of six *Prpf31*^*A216P/+*^ and three WT-littermates.

**Additional file 3. **Results of alternative splicing analysis (MTA) 1.0 in RPE samples of six *Prpf31*^*A216P/+*^ and three WT-littermates.

## Data Availability

All data generated or analyzed during this study are included in this published article, in the supplementary files.

## Abbreviations

adRP: Autosomal dominant retinitis pigmentosa
AMD: Age-related macular degeneration
cd: Candela
CT: Average cycle threshold
DAVID: Database for Annotation, Visualization and Integrated Discovery
ERG: Electroretionogram
F0: Fluorescence just after bleaching
F∞: Fluorescence in the bleached region after full recovery
Fi: Fluorescence before bleaching
FRAP: Fluorescence recovery after bleaching
HSP70: Heat shock protein 70
Hspa4l: Heat shock 70 kDa protein 4 L
*Hspa4l*: *Heat shock protein family A (Hsp70) member 4 like* gene
KI: Knock-in
KO: Knockout
mf: Mobile fraction
MTA 1.0: Mouse Transcriptome Array
Mv: Microvilli
NLS: Nuclear localization sequence
OS: Outer segments
PBS-T: 0.2% Triton X-100/PBS
PFA: Paraformaldehyde
qPCR: Quantitative RT-PCR
RP: Retinitis pigmentosa
RPE: Retinal pigment epithelium
TEM: Transmission electron microscopy
τ_1/2_: Half-life
