# Supplementary material for: Retinal pigment epithelium degeneration caused by aggregation of PRPF31 and the role of HSP70 family of proteins
Source: Mol Med. 2019 Dec 31;26:1. doi: 10.1186/s10020-019-0124-z (PMC6938640; doi:10.1186/s10020-019-0124-z)
Supplement: Supplementary file 1 — Additional file 1: Figure S1. TEM images of RPE of 8-month-old WT (a) and Prpf31A216P/+ mice (b). Photoreceptor outer segments (OS) were observed in contact with the RPE microvilli (Mv) in WT mice (a). In Prpf31A216P/+ mice OS and Mv are also observed; however, they looked shorter and disorganized (b). RPE nuclei (Nu), melanin granules (Me), basal infoldings (BI), Bruch’s membrane (BM) and choroid (Co) are also displayed. Scale bars represent 5 μm. Figure S2. ERG recordings of WT and Prpf31A216P/+ mice. Rod response and quantification of b-wave amplitude in dark adapted mice (a-a”). Cone-rod response and quantification of a-wave in dark adapted mice (b-b”). Cone response and quantification of b-wave in light adapted mice (c-c”). RPE response and quantification of c-wave in dark adapted mice (d-d”). The boxplots a”-d” represent the a, b and c-wave amplitude in dark or light adapted WT and Prpf31A216P/+ mice (n = 6 in each group). Statistically significant differences were determined by Mann-Whitney U-test (*p < 0.05). Vertical scale bars represent 300 μV. Horizontal scale bars represent 100 ms (a-c’) and 1 s (d-d’). Figure S3. Western blot analyses of PRPF31 expression in the neuroretina and RPE of C57BL/6 mice, pig, cow and human samples. The amount of PRPF31 protein is higher in the RPE than in the neuroretina in all the cases. Anti-γ-tubulin antibody was used as loading control. Figure S4. Whole-mount of the RPE obtained from WT (a-i) and Prpf31A216P/+ mice (j-l). Negative controls of RPE autofluorescence (a-c) and secondary antibodies nonspecific bindings are shown (d-f). The RPE of WT Prpf31A216P/+ mice were immunostained with anti-Prpf31 (g, j) and anti-Hsp27 antibodies (h, k). TRITC-phalloidin was used to stain F-actin microfilaments (a-l; blue). Merged images are shown (c, f, i, l). Prpf31 protein aggregates were observed in the cytoplasm colocalizing with Hsp27 signal in mutant Prpf31A216P/+ mice (j-l). Z-stack of a ARPE-19 cell transfected with PRPF31-GFP ( [file 10020_2019_124_MOESM1_ESM.pdf]

Supplementary Figure 1

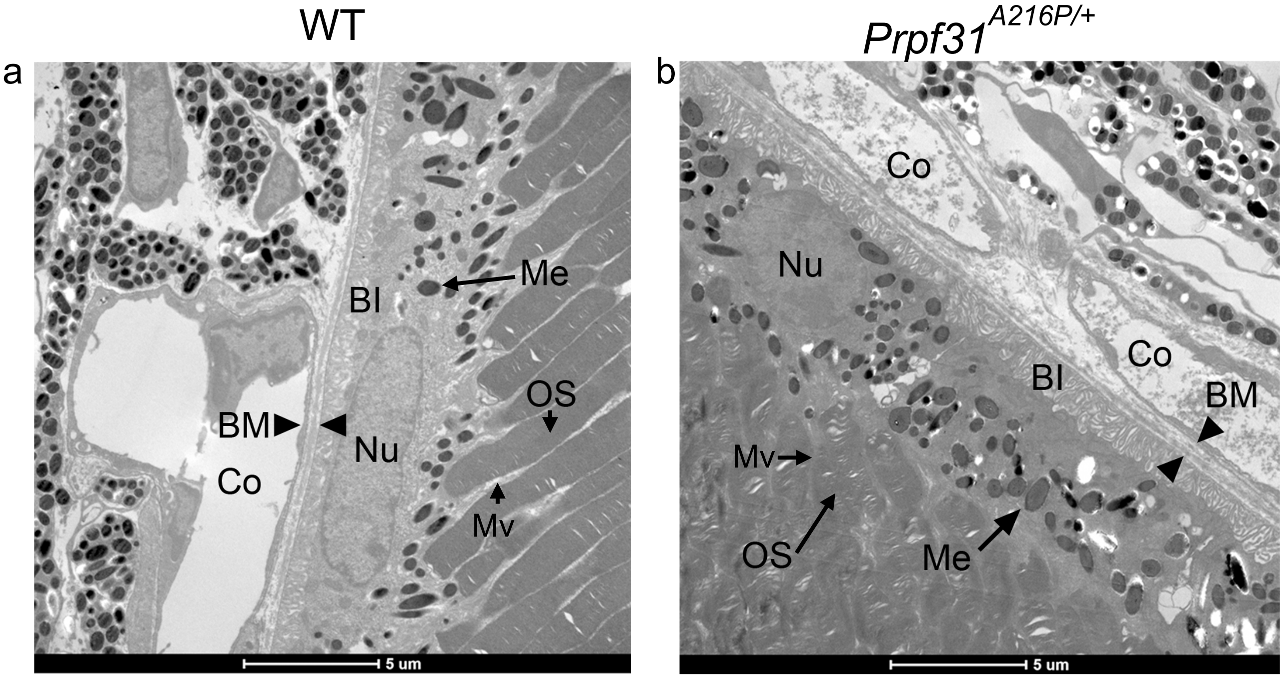

Supplementary Figure 2

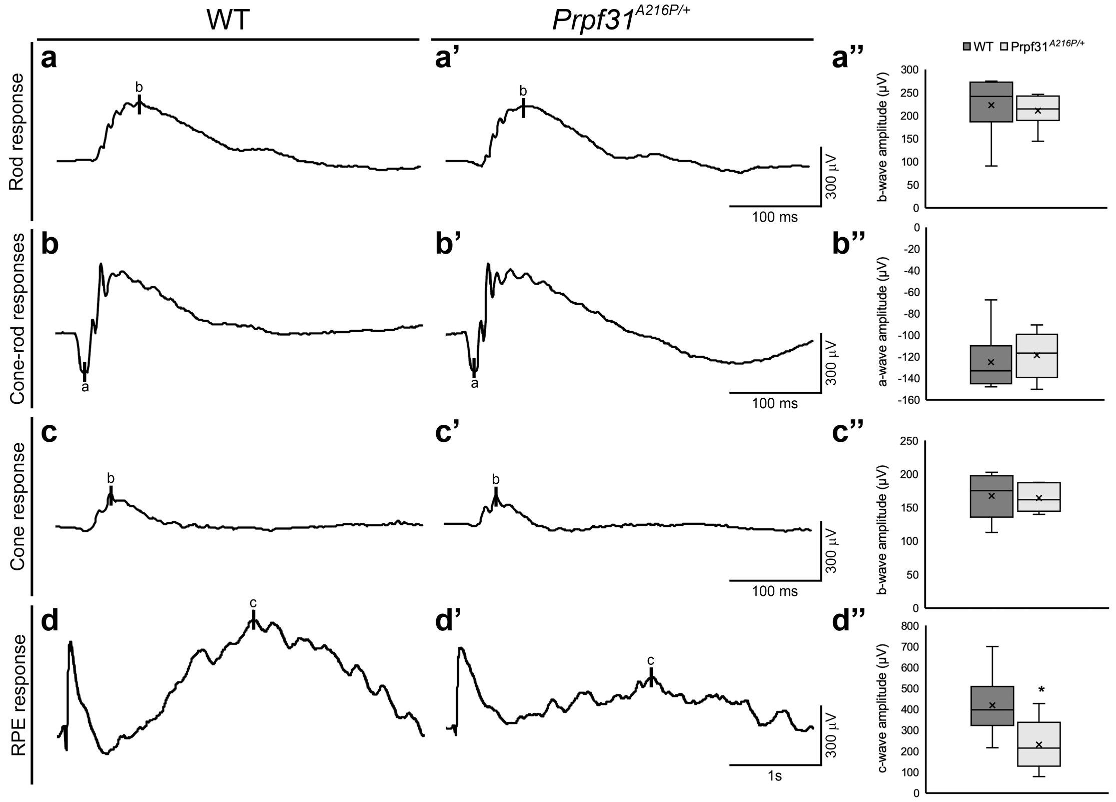

Supplementary Figure 3

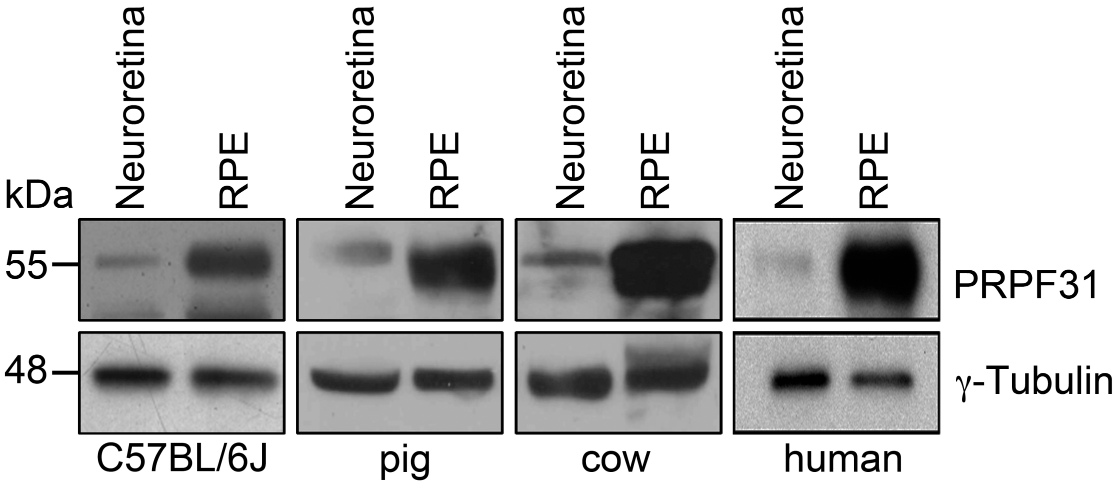

Supplementary Figure 4

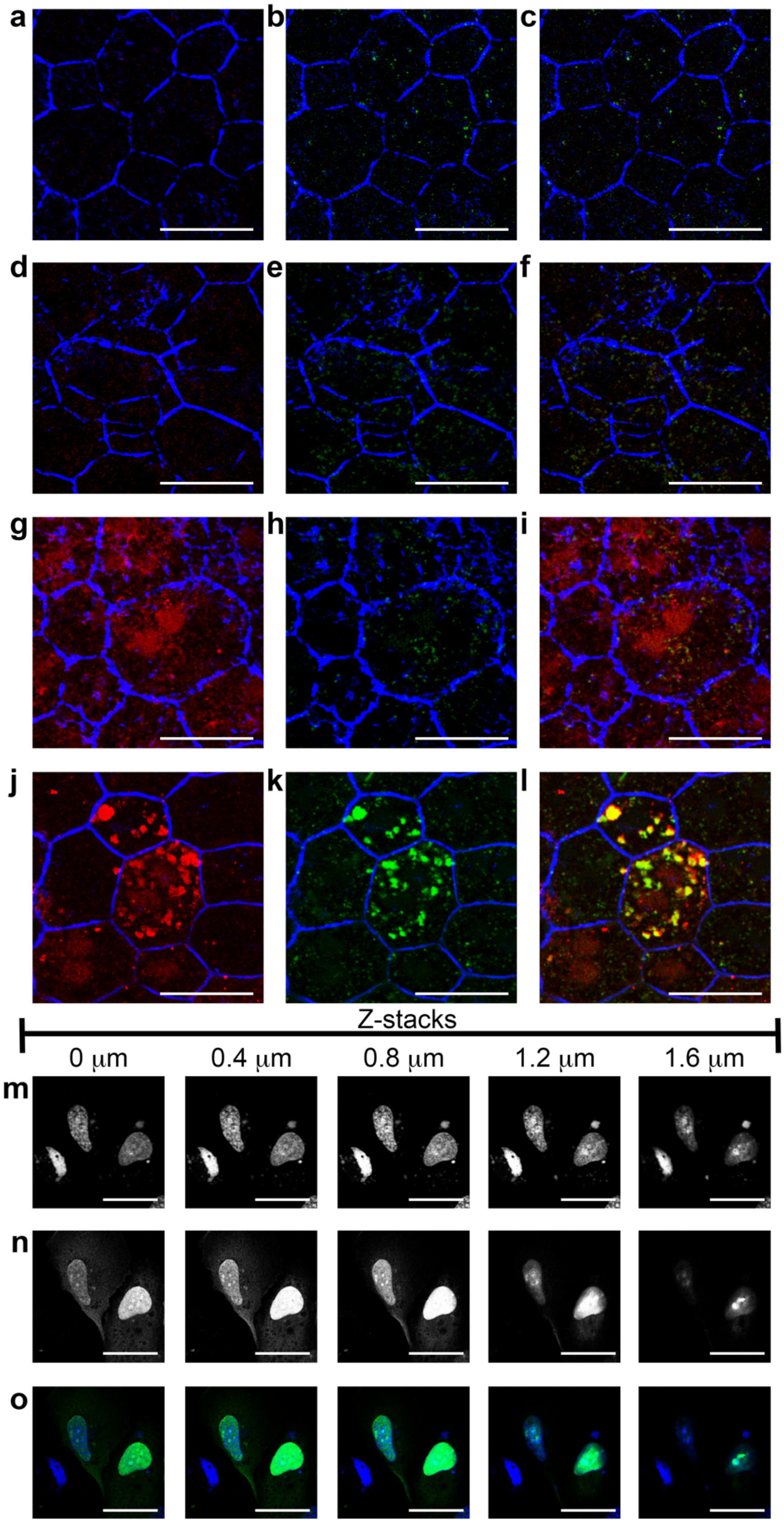

Supplementary Table 1: Gene ontology of differentially expressed genes in *Prpf31*$^{A216P/+}$ mice. The top 20 terms of 84 are listed.

**Term**	**Count (number of genes)**	***p*-Value**
Protein binding	123	<0.001
Nucleotide binding	51	0.089
Calcium ion binding	31	<0.001
Signal transducer activity	27	0.002
Protein domain specific binding	23	<0.001
Protein heterodimerization activity	22	0.004
Protein complex binding	20	<0.001
Ion channel activity	17	<0.001
Histone binding	14	<0.001
Voltage-gated ion channel activity	13	<0.001
Syntaxin binding	12	<0.001
Microtubule binding	12	0.004
Gtpase activity	12	0.005
Chloride channel activity	11	<0.001
Symporter activity	11	<0.001
Lyase activity	11	<0.001
Protein C-terminus binding	11	0.014
Extracellular ligand-gated ion channel activity	10	<0.001
Voltage-gated potassium channel activity	10	<0.001
Ion channelbinding	10	0.001

DAVID v6.8, GOTERM_MF_DIRECT category.

Supplementary Table 2: Candidate genes differentially expressed in the RPE of *Prpf31*$^{A216P/+}$ vs WT mice. Default filter criteria, fold change < -2 or > 2 and *p*-value < 0.05.

Functional Categories	Signaling Pathways and Diseases	Up-regulated	Down-regulated
Cell adhesion and migration	PI3K-Akt-mTOR-signaling pathway	*Akt3, Vtn, Fgf11, Gnb1, Gngt1, Gng3, Ppp2r2b*	
	Focal adhesion	*Vtn, Shc3, Akt3*	
	Integrin-mediated cell adhesion	*Mapk10, Akt3, Shc3*	
	α6-β4 Integrin signaling pathway	*Prkca*	
Cell proliferation, differentiation and migration	MAPK signaling pathway	*Rasgrf2, Ppp3cc, Mapk10, Akt3*	*Il1a*
	Wnt signaling pathway	*Mapk10, Prkd1, Prkca, Akt3*	
Cellular apoptosis	Apoptosis modulation by HSP70	*Mapk10*	
	FAS pathway and stress induction of HSP regulation		*Il1a*
Cilium	Ciliogenesis	*Ahi1, Bbs4, Bbs7, Bbs9, Fam161a, Rp1, Rp1l1, Rpgrip1*	
Inflammation	Chemokine signaling pathway	*Shc3, Gnb1, Gnb3, Gnb5, Gngt, Grk1, Adcy1, Akt3*	
	Toll-like receptor signaling	*Mapk10, Akt3*	
	TNF-α NF-kβ signaling pathway	*Tnfaip3*	
	B cell receptor signaling pathway	*Prkd1*	
	T cell receptor signaling pathway	*Unc119*	
	IL-1 signaling pathway		*Il1a, Il1f8, Il1f9*
	IL-3 signaling pathway	*Dnm1, Prkca, Kcnip3*	
	IL-5 signaling pathway	*Unc119*	
	IL-6 signaling pathway	*Ppp2r2b*	
	Inflammatory response pathway	*Vnt*	
mRNA Processing	mRNA processing	*Snrpn, Elavl3, Pcbp3, Pcbp4*	*Oas2*
Oxidative stress	Oxidative stress	*Mapk10*	
	Keap1-Nrf2 pathway	*Prkca*	
Pluripotency	Wnt signaling pathway	*Prkca, Prkd1, Mapk10, Ppp2r2b*	
Protein metabolism	Proteasome degradation	*Uchl1*	
	Protein processing	*Hspa4l*	
Retinol metabolism	Retinol metabolism	*Rdh8, Rdh12*	
Voltage-gated channels	K^+ channel	*Kcnip3, Kcnma1, Kcnb1, Kcnab2*	*Kcnj13*
	Ca^{2+} channel	*Cacna1f*	

Transcriptome Analysis Console Software and DAVID v6.8.

Supplementary Table 3: Functional categories of genes having alternative splicing in *Prpf31*$^{A216P/+}$ mice. The top 40 terms of 174 are listed.

Term	Count (number of genes)	*p*-Value
Phosphoprotein	2676	<0.001
Membrane	2195	0.066
Alternative splicing	1929	<0.001
Cytoplasm	1499	<0.001
Nucleus	1394	<0.001
Glycoprotein	1117	<0.001
Coiledcoil	1113	<0.001
Metal-binding	1107	<0.001
Acetylation	908	<0.001
Disulfide bond	831	0.005
Transport	668	<0.001
Zinc	652	<0.001
Nucleotide-binding	647	<0.001
Transferase	558	<0.001
Transcription	553	<0.001
Transcription regulation	539	<0.001
Ubl conjugation	536	<0.001
ATP-binding	530	<0.001
Hydrolase	517	<0.001
Zinc-finger	482	<0.001
DNA-binding	451	<0.001
Cytoskeleton	406	<0.001
Calcium	375	<0.001
Methylation	371	<0.001
Isopeptide bond	346	<0.001
Cell projection	309	<0.001
Cell junction	308	<0.001
Developmental protein	303	<0.001
Ion transport	275	<0.001
Endoplasmic reticulum	268	0.068
Golgi apparatus	265	<0.001
Kinase	263	<0.001
Lipoprotein	253	<0.001
Differentiation	240	<0.001
Synapse	214	<0.001
Cell adhesion	211	<0.001
RNA-binding	205	<0.001
Cell cycle	198	<0.001
Activator	194	<0.001
Magnesium	191	<0.001

DAVID v6.8, UP_KEYWORDS category.

Supplementary Table 4: Number of candidate genes showing alternative splicing in the RPE of *Prpf31*$^{A216P/+}$ vs WT mice. Default filter criteria, splicing index < -2 or > 2 and ANOVA *p* value < 0.05.

**Functional Categories**	**Signaling Pathways and Diseases**	**High splicing index**	**Low splicing index**	**Total number of genes showing alternative splining**
Angiogenesis	VEGF signaling pathway	1	0	1
Cell adhesion and migration	PI3K-Akt-mTOR-signaling pathway	47	69	116
	Focal adhesion	29	51	80
	Integrin-mediated cell adhesion	20	22	42
	α6-β4 Integrin signaling pathway	10	17	27
Cell proliferation, differentiation and migration	MAPK signaling pathway	29	21	50
	Wnt signaling pathway	18	13	31
Cellular apoptosis	Apoptosis modulation by HSP70	7	13	20
	FAS pathway and stress induction of HSP regulation	3	4	7
Cilium	Ciliogenesis	2	5	7
Inflammation	Chemokine signaling pathway	37	26	63
	Toll-like receptor signaling	10	17	27
	TNF-α NF-kβ signaling pathway	34	27	61
	B cell receptor signaling pathway	25	20	45
	T cell receptor signaling pathway	21	21	42
	IL-1 signaling pathway	2	7	9
	IL-3 signaling pathway	15	15	30
	IL-5 signaling pathway	9	13	22
	IL-6 signaling pathway	16	13	29
	Inflammatory response pathway	3	9	12
mRNA Processing	mRNA processing	72	30	102
Oxidative stress	Oxidative Stress	2	0	2
	Keap1-Nrf2 Pathway	2	0	2
Protein metabolism	Proteasome degradation	3	4	7
Pluripotency	Wnt signaling pathway	16	13	29
Retinol metabolism	Retinol metabolism	5	5	10

Transcriptome Analysis Console Software and DAVID v6.8
