# Supplementary material for: Retinal pigment epithelium degeneration caused by aggregation of PRPF31 and the role of HSP70 family of proteins
Source: Mol Med. 2019 Dec 31;26:1. doi: 10.1186/s10020-019-0124-z (PMC6938640; doi:10.1186/s10020-019-0124-z)
Supplement: Supplementary file 2 — Additional file 2. Results of transcriptome array (MTA) 1.0 to evaluate differential gene expression in RPE samples of six Prpf31A216P/+ and three WT-littermates. [file 10020_2019_124_MOESM2_ESM.pdf]

Transcript Cluster ID	Prpf31 A216P Bi-weight	Prpf31 WT Bi-weight Avg Si	Fold Change (linear) (Prpf31	ANOVA p-value (Prpf31 A2:	FDR p-value (Prpf31	Gene Symbol
TC0700003295.mm.1	14,48	7,96	91,24	0,00016	0,094374	Gm26032
TC0700003298.mm.1	14,48	7,96	91,24	0,00016	0,094374	Gm22046
TC0700003303.mm.1	14,48	7,96	91,24	0,00016	0,094374	Gm25471
TC0700003306.mm.1	14,48	7,96	91,24	0,00016	0,094374	Gm25210
TC0700003300.mm.1	14,66	8,16	90,08	0,000156	0,094374	Snord116; Ipw
TC0700003307.mm.1	14,66	8,16	90,08	0,000156	0,094374	Snord116; Ipw
TC0700003314.mm.1	14,66	8,16	90,08	0,000156	0,094374	Snord116; Ipw
TC0700003316.mm.1	14,66	8,16	90,08	0,000156	0,094374	Snord116; Ipw
TC0700003319.mm.1	14,66	8,16	90,08	0,000156	0,094374	Snord116; Ipw
TC0700003321.mm.1	14,66	8,16	90,08	0,000156	0,094374	Snord116; Ipw
TC0700003322.mm.1	14,66	8,16	90,08	0,000156	0,094374	Snord116; Ipw
TC0700003323.mm.1	14,66	8,16	90,08	0,000156	0,094374	Snord116; Ipw
TC0700003325.mm.1	14,66	8,16	90,08	0,000156	0,094374	Snord116; Ipw
TC0700003326.mm.1	14,66	8,16	90,08	0,000156	0,094374	Snord116; Ipw
TC0700003328.mm.1	14,66	8,16	90,08	0,000156	0,094374	Snord116; Ipw
TC0700003330.mm.1	14,66	8,16	90,08	0,000156	0,094374	Snord116; Ipw
TC0700003331.mm.1	14,66	8,16	90,08	0,000156	0,094374	Snord116; Ipw
TC0700003333.mm.1	14,66	8,16	90,08	0,000156	0,094374	Snord116; Ipw
TC0700003334.mm.1	14,66	8,16	90,08	0,000156	0,094374	Snord116; Ipw
TC0700003335.mm.1	14,66	8,16	90,08	0,000156	0,094374	Snord116; Ipw
TC0700003336.mm.1	14,66	8,16	90,08	0,000156	0,094374	Snord116; Ipw
TC0700003339.mm.1	14,66	8,16	90,08	0,000156	0,094374	Snord116; Ipw
TC0700003340.mm.1	14,66	8,16	90,08	0,000156	0,094374	Snord116; Ipw
TC0700003273.mm.1	14,55	8,1	87,56	0,00018	0,094374	Snord116; Ipw
TC0700003275.mm.1	14,55	8,1	87,56	0,00018	0,094374	Snord116; Ipw
TC0700003277.mm.1	14,55	8,1	87,56	0,00018	0,094374	Snord116; Ipw
TC0700003279.mm.1	14,55	8,1	87,56	0,00018	0,094374	Snord116; Ipw
TC0700003280.mm.1	14,55	8,1	87,56	0,00018	0,094374	Snord116; Ipw
TC0700003283.mm.1	14,55	8,1	87,56	0,00018	0,094374	Snord116; Ipw
TC0700003285.mm.1	14,55	8,1	87,56	0,00018	0,094374	Snord116; Ipw
TC0700003289.mm.1	14,55	8,1	87,56	0,00018	0,094374	Snord116l1; Snord116; Ipw
TC0700003299.mm.1	14,55	8,1	87,56	0,00018	0,094374	Snord116; Ipw
TC0700003329.mm.1	14,55	8,1	87,56	0,00018	0,094374	Snord116l2; Snord116; Ipw
TC0700003337.mm.1	14,55	8,1	87,56	0,00018	0,094374	Snord116l1; Snord116; Ipw
TC0700003286.mm.1	14,65	8,22	86,26	0,000123	0,094374	Gm23089
TC0700003287.mm.1	14,65	8,22	86,26	0,000123	0,094374	Gm24711
TC0700003288.mm.1	14,65	8,22	86,26	0,000123	0,094374	Gm26188
TC0700003327.mm.1	14,23	7,8	86,16	0,000133	0,094374	Gm22188
TC0700003301.mm.1	14,37	7,98	83,93	0,000141	0,094374	Gm26502
TC0700003302.mm.1	14,37	7,98	83,93	0,000141	0,094374	Gm23313
TC0700003305.mm.1	14,37	7,98	83,93	0,000141	0,094374	Gm23446
TC0700003338.mm.1	14,32	7,95	83,14	0,000121	0,094374	Gm22173
TC0700003272.mm.1	14,18	7,84	81,18	0,000146	0,094374	Gm23862
TC0700003310.mm.1	12,68	6,43	75,94	0,000119	0,094374	Gm25157
TC0700003313.mm.1	12,68	6,43	75,94	0,000119	0,094374	Gm22941
TC0700003318.mm.1	12,68	6,43	75,94	0,000119	0,094374	Gm22258
TC0700003308.mm.1	13,1	6,9	73,53	0,000157	0,094374	Gm22851

TC0700003309.mm.1	13,76	7,65	68,98	0,000183	0,094396	Gm25074
TC0700003311.mm.1	13,61	7,58	65,17	0,000178	0,094374	Gm22631
TC0700003284.mm.1	14,39	8,52	58,51	0,000174	0,094374	Gm22584
TC0700003293.mm.1	13,72	7,94	54,87	0,000282	0,117638	Gm23953
TC0700003294.mm.1	13,72	7,94	54,87	0,000282	0,117638	Gm26332
TC0700003296.mm.1	13,72	7,94	54,87	0,000282	0,117638	Gm22863
TC0700003297.mm.1	13,72	7,94	54,87	0,000282	0,117638	Gm24618
TC0700003278.mm.1	13,91	8,54	41,45	0,00018	0,094374	Gm24264
TC0700003274.mm.1	12,04	6,75	38,98	0,000502	0,13338	Gm22131
TC0700003312.mm.1	13,99	8,83	35,76	0,00018	0,094374	Gm25474
TC0700003315.mm.1	13,99	8,83	35,76	0,00018	0,094374	Gm22047
TC0700003320.mm.1	13,99	8,83	35,76	0,00018	0,094374	Gm23619
TC0700003324.mm.1	13,82	8,72	34,18	0,000165	0,094374	Gm25615
TC0700003247.mm.1	12,08	7,06	32,53	0,000384	0,120143	Gm25194
TC0700003205.mm.1	12,32	7,46	29,08	0,00042	0,120143	Gm26283
TC0700003230.mm.1	12,32	7,46	29,08	0,00042	0,120143	Gm23682
TC0700003231.mm.1	12,32	7,46	29,08	0,00042	0,120143	Gm24654
TC0700003234.mm.1	12,32	7,46	29,08	0,00042	0,120143	Gm25585
TC0700003235.mm.1	12,32	7,46	29,08	0,00042	0,120143	Gm25840
TC0700003237.mm.1	12,32	7,46	29,08	0,00042	0,120143	Gm24040
TC0700003238.mm.1	12,32	7,46	29,08	0,00042	0,120143	Gm22050
TC0700003239.mm.1	12,32	7,46	29,08	0,00042	0,120143	Gm23286
TC0700003240.mm.1	12,32	7,46	29,08	0,00042	0,120143	Gm24021
TC0700003241.mm.1	12,32	7,46	29,08	0,00042	0,120143	Gm22274
TC0700003242.mm.1	12,32	7,46	29,08	0,00042	0,120143	Gm24652
TC0700003243.mm.1	12,32	7,46	29,08	0,00042	0,120143	Gm23618
TC0700003244.mm.1	12,32	7,46	29,08	0,00042	0,120143	Gm23688
TC0700003245.mm.1	12,32	7,46	29,08	0,00042	0,120143	Gm24570
TC0700003248.mm.1	12,32	7,46	29,08	0,00042	0,120143	Gm22640
TC0700003249.mm.1	12,32	7,46	29,08	0,00042	0,120143	Gm24952
TC0700003251.mm.1	12,32	7,46	29,08	0,00042	0,120143	Gm23944
TC0700003252.mm.1	12,32	7,46	29,08	0,00042	0,120143	Gm22629
TC0700003253.mm.1	12,32	7,46	29,08	0,00042	0,120143	Gm26374
TC0700003254.mm.1	12,32	7,46	29,08	0,00042	0,120143	Gm26284
TC0700003255.mm.1	12,32	7,46	29,08	0,00042	0,120143	Gm25452
TC0700003256.mm.1	12,32	7,46	29,08	0,00042	0,120143	Gm25984
TC0700003261.mm.1	12,32	7,46	29,08	0,00042	0,120143	Gm25017
TC0700003236.mm.1	12,33	7,48	28,84	0,000405	0,120143	Gm25089
TC0700003226.mm.1	12,76	7,96	27,96	0,000143	0,094374	Gm23305
TC0700003228.mm.1	12,76	7,96	27,96	0,000143	0,094374	Gm25085
TC0700003282.mm.1	12,99	8,19	27,87	0,000288	0,117638	Gm23767
TC0700003220.mm.1	14,26	9,5	27,19	0,00047	0,130248	Gm22252
TC0700003317.mm.1	10,46	5,86	24,25	0,000494	0,13338	Gm26097
TC0700003281.mm.1	10,66	6,16	22,69	0,000397	0,120143	Gm23141
TC0700003172.mm.1	11,37	6,95	21,52	0,000114	0,094374	Gm25988
TC0700003173.mm.1	11,37	6,95	21,52	0,000114	0,094374	Gm25221
TC0700003175.mm.1	11,37	6,95	21,52	0,000114	0,094374	Gm23471
TC0700003213.mm.1	11,37	6,95	21,52	0,000114	0,094374	Gm23449

TC0700003160.mm.1	13,24	8,88	20,54	0,000278	0,117638	Gm24799
TC0700003198.mm.1	14,96	10,68	19,47	0,000938	0,163425	Gm24926
TC0700003200.mm.1	14,96	10,68	19,47	0,000938	0,163425	Gm22632
TC0700003156.mm.1	14,32	10,09	18,74	0,000676	0,14969	Gm24862
TC0700003214.mm.1	14,32	10,09	18,74	0,000676	0,14969	Gm23310
TC0700003215.mm.1	14,32	10,09	18,74	0,000676	0,14969	Gm25461
TC0700003203.mm.1	14,9	10,69	18,62	0,00115	0,165893	Gm26366
TC0700003157.mm.1	14,83	10,64	18,3	0,001105	0,163425	Gm24866
TC0700003162.mm.1	14,83	10,64	18,3	0,001105	0,163425	Gm25463
TC0700003165.mm.1	14,83	10,64	18,3	0,001105	0,163425	Gm24528
TC0700003166.mm.1	14,83	10,64	18,3	0,001105	0,163425	Gm23356
TC0700003168.mm.1	14,83	10,64	18,3	0,001105	0,163425	Gm24872
TC0700003188.mm.1	14,83	10,64	18,3	0,001105	0,163425	Gm24027
TC0700003189.mm.1	14,83	10,64	18,3	0,001105	0,163425	Gm22524
TC0700003190.mm.1	14,83	10,64	18,3	0,001105	0,163425	Gm22630
TC0700003192.mm.1	14,83	10,64	18,3	0,001105	0,163425	Gm25741
TC0700003193.mm.1	14,83	10,64	18,3	0,001105	0,163425	Gm26059
TC0700003195.mm.1	14,83	10,64	18,3	0,001105	0,163425	Gm22511
TC0700003206.mm.1	14,83	10,64	18,3	0,001105	0,163425	Gm25098
TC0700003207.mm.1	14,83	10,64	18,3	0,001105	0,163425	Gm24702
TC0700003209.mm.1	14,83	10,64	18,3	0,001105	0,163425	Gm25462
TC0700003211.mm.1	14,83	10,64	18,3	0,001105	0,163425	Gm25523
TC0700003217.mm.1	14,83	10,64	18,3	0,001105	0,163425	Gm22909
TC0700003221.mm.1	14,83	10,64	18,3	0,001105	0,163425	Gm26392
TC0700003222.mm.1	14,83	10,64	18,3	0,001105	0,163425	Gm24219
TC0700003223.mm.1	14,83	10,64	18,3	0,001105	0,163425	Gm25230
TC0700003224.mm.1	14,83	10,64	18,3	0,001105	0,163425	Gm22627
TC0700003225.mm.1	14,83	10,64	18,3	0,001105	0,163425	Gm24585
TC0700003227.mm.1	14,83	10,64	18,3	0,001105	0,163425	Gm24206
TC0700003229.mm.1	14,83	10,64	18,3	0,001105	0,163425	Gm25209
TC0700003232.mm.1	14,83	10,64	18,3	0,001105	0,163425	Gm26334
TC0700003233.mm.1	14,83	10,64	18,3	0,001105	0,163425	Gm25742
TC0700003257.mm.1	14,83	10,64	18,3	0,001105	0,163425	Gm25146
TC0700003258.mm.1	14,83	10,64	18,3	0,001105	0,163425	Gm24759
TC0700003262.mm.1	14,83	10,64	18,3	0,001105	0,163425	Gm23575
TC0700003264.mm.1	14,83	10,64	18,3	0,001105	0,163425	Gm22449
TC0700003266.mm.1	14,83	10,64	18,3	0,001105	0,163425	Gm22912
TC0700003267.mm.1	14,83	10,64	18,3	0,001105	0,163425	Gm25710
TC0700003268.mm.1	14,83	10,64	18,3	0,001105	0,163425	Gm26434
TC0700003269.mm.1	14,83	10,64	18,3	0,001105	0,163425	Gm23560
TC0700003186.mm.1	10,25	6,06	18,29	0,000129	0,094374	Gm22510
TC0700003202.mm.1	10,25	6,06	18,29	0,000129	0,094374	Gm26390
TC0700003204.mm.1	10,25	6,06	18,29	0,000129	0,094374	Gm26336
TC0700003212.mm.1	14,83	10,66	17,96	0,001144	0,165465	Gm22255
TC0700003218.mm.1	11,4	7,25	17,82	0,000114	0,094374	Gm25823
TC0700003219.mm.1	11,4	7,25	17,82	0,000114	0,094374	Gm25647
TC0700003199.mm.1	9,39	5,26	17,53	0,000101	0,094374	Gm26200
TC0700003197.mm.1	14,78	10,66	17,38	0,001165	0,165963	Gm22834

TC0700003291.mm.1	11,47	7,38	17,07	0,000539	0,138648	Gm25944
TC0700003304.mm.1	11,8	7,72	16,96	0,000538	0,138648	Gm22776
TC0700003208.mm.1	14,79	10,72	16,81	0,001246	0,171857	Gm26432
TC0700003135.mm.1	11,4	7,33	16,8	0,000123	0,094374	Gm26488
TC0700003136.mm.1	11,4	7,33	16,8	0,000123	0,094374	Gm24220
TC0700004651.mm.1	13	8,94	16,69	0,001934	0,195767	
TC1400000923.mm.1	11,75	7,84	15,06	0,004308	0,238857	Mir124a-1; Mir3078; A930011O12Rik
TC0700003259.mm.1	12,99	9,11	14,79	0,001047	0,163425	Gm24100
TC0700003151.mm.1	9,42	5,58	14,37	0,000078	0,094374	Gm25646
TC0700003167.mm.1	14,64	10,84	13,89	0,000924	0,163425	Gm25449
TC0700003263.mm.1	13,04	9,25	13,82	0,001011	0,163425	Gm22391
TC0700003265.mm.1	13,04	9,25	13,82	0,001011	0,163425	Gm24137
TC0700003210.mm.1	10,85	7,07	13,71	0,000365	0,120143	Gm24495
TC0700003177.mm.1	10,72	6,97	13,46	0,000146	0,094374	Gm24418
TC0700003180.mm.1	10,72	6,97	13,46	0,000146	0,094374	Gm24639
TC0700003183.mm.1	10,72	6,97	13,46	0,000146	0,094374	Gm23076
TC0700003196.mm.1	10,72	6,97	13,46	0,000146	0,094374	Gm25077
TC1400000920.mm.1	9,51	5,87	12,47	0,000646	0,146104	
TC0100000828.mm.1	14,04	10,46	11,98	0,005615	0,247078	Sag
TC0700003139.mm.1	10,16	6,59	11,88	0,00025	0,113809	Gm24969
TC1400000922.mm.1	13,44	9,87	11,85	0,047047	0,3242	
TC0700003290.mm.1	8,98	5,43	11,72	0,000703	0,150708	Gm24518
TC0700003132.mm.1	9,46	5,91	11,7	0,000395	0,120143	Gm24306
TC0500002578.mm.1	11,82	8,27	11,7	0,011455	0,263089	Cnga1
TC0500001103.mm.1	13,7	10,18	11,54	0,00231	0,203813	Pde6b
TC0400001696.mm.1	10,4	6,88	11,51	0,001663	0,185899	
TC1400000965.mm.1	10,3	6,78	11,42	0,00276	0,216023	Nefl
TC0900002745.mm.1	10,8	7,3	11,32	0,007273	0,25369	Impg1
TC0600001335.mm.1	11,2	7,7	11,25	0,004195	0,238857	Cacna2d4
TC0700003185.mm.1	9,4	5,92	11,14	0,000289	0,117638	Gm25874
TC0300000112.mm.1	10,8	7,35	10,96	0,003255	0,222675	2610100L16Rik
TC0700003260.mm.1	13,65	10,2	10,93	0,000595	0,141747	Gm22253
TC0200001692.mm.1	9,81	6,36	10,89	0,00458	0,240696	
TC0X00000700.mm.1	11,21	7,77	10,82	0,017801	0,2792	Opn1mw
TC0700003174.mm.1	9,48	6,05	10,74	0,000134	0,094374	Gm26389
TC0700003178.mm.1	9,48	6,05	10,74	0,000134	0,094374	Gm23687
TC0700003179.mm.1	9,48	6,05	10,74	0,000134	0,094374	Gm22393
TC0700003181.mm.1	9,48	6,05	10,74	0,000134	0,094374	Gm24566
TC0700003182.mm.1	9,48	6,05	10,74	0,000134	0,094374	Gm22111
TC0700003342.mm.1	8,97	5,55	10,68	0,000615	0,142861	
TC1800000627.mm.1	14,7	11,3	10,52	0,005677	0,247208	Pde6a
TC0700003170.mm.1	8,89	5,49	10,51	0,003223	0,222303	Gm23145
TC0700003201.mm.1	8,65	5,26	10,45	0,001015	0,163425	Gm23254
TC0700003137.mm.1	12,13	8,79	10,16	0,004431	0,239131	
TC0700003143.mm.1	9,98	6,63	10,14	0,000361	0,120143	Gm24657
TC0700003145.mm.1	9,98	6,63	10,14	0,000361	0,120143	Gm23922
TC0700003148.mm.1	9,98	6,63	10,14	0,000361	0,120143	Gm22996
TC0700003191.mm.1	13,12	9,79	10,06	0,006929	0,253558	

TC1700000905.mm.1	15,49	12,17	9,94	0,00515	0,247078	Prph2
TC0200002077.mm.1	15,83	12,53	9,87	0,011718	0,263089	Snap25
TC1300001857.mm.1	11,1	7,83	9,71	0,007742	0,25437	Mak
TC1100000861.mm.1	11,04	7,76	9,71	0,014257	0,271572	Rcvrn
TC0700004650.mm.1	9,9	6,62	9,67	0,000863	0,161778	Ipw; Snord116
TC1400000921.mm.1	11,7	8,43	9,61	0,041912	0,317818	
TC0800002920.mm.1	9,72	6,46	9,57	0,002186	0,199997	Calb2
TC1100004206.mm.1	13,1	9,84	9,56	0,013566	0,269523	Pde6g
TC0700003164.mm.1	8,47	5,23	9,45	0,000266	0,116988	Gm25229
TC1900000714.mm.1	8,49	5,26	9,41	0,005057	0,246422	Ina
TC0700003140.mm.1	13,02	9,86	8,97	0,005239	0,247078	
TC1300001332.mm.1	11,5	8,35	8,88	0,044998	0,322212	
TC0100001340.mm.1	8,5	5,35	8,86	0,003396	0,226338	
TC1200001854.mm.1	9,04	5,9	8,81	0,004256	0,238857	
TC0400004175.mm.1	10,22	7,08	8,8	0,006866	0,253558	Samd11
TC0100001339.mm.1	13,94	10,82	8,7	0,004473	0,239488	Pdc
TC0700003131.mm.1	9,76	6,65	8,62	0,000346	0,120143	Gm25648
TC1700002348.mm.1	10,54	7,44	8,6	0,018732	0,281329	Tubb4a
TC0700003276.mm.1	11,2	8,1	8,59	0,001763	0,188761	Gm26365
TC1100003136.mm.1	9,94	6,85	8,53	0,002468	0,209744	Aipl1
TC0700003194.mm.1	10,08	7	8,46	0,000506	0,133573	Gm25175
TC0800000127.mm.1	11,62	8,54	8,46	0,003283	0,222955	Grk1
TC1100003643.mm.1	12,69	9,66	8,18	0,006471	0,252353	Gm11594
TC0600001980.mm.1	10,87	7,84	8,15	0,018647	0,281198	Opn1sw
TC0700003158.mm.1	6,61	3,59	8,12	0,004824	0,244766	Gm22348
TC0700003176.mm.1	8,46	5,46	7,98	0,000587	0,141747	Gm25120
TC0300000113.mm.1	7,46	4,47	7,95	0,002556	0,210008	Mir124a-2
TC1200000905.mm.1	8,19	5,21	7,89	0,012082	0,263852	
TC0500001439.mm.1	9,16	6,2	7,77	0,009619	0,260471	Gm16338
TC0700003154.mm.1	7,56	4,61	7,74	0,000154	0,094374	Gm23660
TC0700003161.mm.1	7,47	4,53	7,69	0,00011	0,094374	Gm24088
TC0800000973.mm.1	11,85	8,94	7,49	0,012362	0,265476	Rtbdn
TC0X00001636.mm.1	14,88	11,99	7,41	0,016265	0,275809	Rs1
TC0100002640.mm.1	9,67	6,79	7,34	0,003565	0,229354	Scg2
TC0400000280.mm.1	9,85	7,02	7,12	0,007781	0,25437	Gabrr1
TC0900002767.mm.1	13,55	10,74	7,04	0,00233	0,203813	Elovl4
TC0700001765.mm.1	9,4	6,59	7,01	0,007856	0,25437	Cacng3
TC0X00000957.mm.1	9,36	6,57	6,89	0,005755	0,247208	Arr3
TC0600001266.mm.1	9,33	6,56	6,85	0,006595	0,252462	Slc6a11
TC0200005183.mm.1	10,9	8,14	6,77	0,002188	0,199997	Kcnb1; Gm14290
TC0500001534.mm.1	8,03	5,27	6,76	0,002775	0,216023	
TC0700003163.mm.1	10,11	7,35	6,74	0,003013	0,21946	
TC1200000718.mm.1	10,35	7,6	6,72	0,005558	0,247078	Rdh12
TC0X00003147.mm.1	13,17	10,42	6,7	0,011756	0,263089	Gm25107
TC0700000832.mm.1	11,9	9,18	6,63	0,015397	0,275022	Slc17a7
TC0700003146.mm.1	8,08	5,36	6,61	0,002004	0,199095	Gm25314
TC1600000745.mm.1	10,16	7,45	6,58	0,0062	0,250355	Impg2
TC1400000292.mm.1	9,25	6,54	6,55	0,004511	0,239729	Lrtm1

TC0700003341.mm.1	9,78	7,08	6,51	0,001825	0,191141	Gm22289
TC0X00000817.mm.1	9,37	6,67	6,49	0,024759	0,29108	
TC1700000913.mm.1	11,83	9,13	6,48	0,002895	0,217961	Guca1b
TC0100003878.mm.1	9,48	6,78	6,48	0,005615	0,247078	Mpp4
TC1200001812.mm.1	10,38	7,69	6,44	0,013686	0,269523	
TC0600000006.mm.1	10,03	7,36	6,37	0,004825	0,244766	Gngt1
TC1400000365.mm.1	15,69	13,03	6,31	0,013369	0,269523	Rbp3
TC1400000913.mm.1	8,81	6,16	6,3	0,00123	0,171167	Rp1l1
TC0900002681.mm.1	9,28	6,64	6,26	0,006737	0,253558	Scg3
TC0700003169.mm.1	8,02	5,38	6,25	0,001017	0,163425	Gm26337
TC0700002617.mm.1	14,25	11,61	6,2	0,01143	0,263089	Atp1a3
TC0800002238.mm.1	9,57	6,96	6,1	0,004481	0,239488	Wdr17
TC0200004813.mm.1	11,13	8,53	6,06	0,003727	0,230617	Napb
TC0700003171.mm.1	7,9	5,31	6,03	0,003284	0,222955	Gm22130
TC0300001809.mm.1	8,12	5,55	5,96	0,003861	0,234	
TC1900000904.mm.1	8,46	5,89	5,95	0,009023	0,260471	Cabp4
TC0800000144.mm.1	12,89	10,32	5,91	0,003239	0,222303	
TC1900001077.mm.1	12,3	9,74	5,89	0,004188	0,238857	Rom1
TC0300000096.mm.1	11,32	8,76	5,88	0,005168	0,247078	Car2
TC0900001327.mm.1	9,78	7,22	5,88	0,017469	0,278371	
TC1700002360.mm.1	9,28	6,73	5,88	0,018796	0,281433	
TC1800001477.mm.1	12,75	10,2	5,86	0,007172	0,253558	Cplx4
TC1200001150.mm.1	9,07	6,54	5,79	0,001336	0,173731	Gm23600
TC1400000393.mm.1	10,06	7,54	5,75	0,004357	0,238857	Lrit2
TC1400000604.mm.1	10,47	7,95	5,72	0,007472	0,25437	Rpgrip1
TC1300001330.mm.1	11,45	8,95	5,68	0,011702	0,263089	Hcn1
TC0900001012.mm.1	12,16	9,66	5,66	0,006535	0,252353	Gnb5
TC1400001745.mm.1	12,18	9,68	5,65	0,005694	0,247208	Cdhr1
TC1700002359.mm.1	8,68	6,19	5,64	0,002705	0,215508	2610034M16Rik
TC0400000278.mm.1	9,05	6,56	5,62	0,016782	0,277334	Gabrr2
TC1700000597.mm.1	8,65	6,16	5,59	0,002351	0,204548	
TC0100003791.mm.1	9,03	6,55	5,58	0,003066	0,220198	Prox1
TC1400001456.mm.1	9,66	7,19	5,52	0,002339	0,203813	Cadps
TC1900001231.mm.1	10,43	7,96	5,52	0,003623	0,230005	Rorb
TC0400001058.mm.1	9,98	7,52	5,51	0,013448	0,269523	Sgip1
TC0500000947.mm.1	8,84	6,39	5,45	0,006966	0,253558	Prdm8
TC0900000494.mm.1	8,31	5,88	5,42	0,013884	0,269879	
TC1700002204.mm.1	12,83	10,4	5,38	0,005131	0,247078	Guca1a
TC1000002832.mm.1	8,27	5,84	5,38	0,031063	0,299432	
TC0700002413.mm.1	11,91	9,49	5,36	0,005472	0,247078	Crx
TC0700003246.mm.1	7,21	4,8	5,33	0,000305	0,119572	Gm26467
TC0700003250.mm.1	7,21	4,8	5,33	0,000305	0,119572	Gm25087
TC1600000585.mm.1	6,4	3,99	5,3	0,005406	0,247078	
TC0900001379.mm.1	12,6	10,2	5,26	0,007652	0,25437	
TC1900000417.mm.1	8,34	5,95	5,25	0,008282	0,25767	Kcnv2
TC0400000279.mm.1	8,84	6,46	5,19	0,013327	0,269523	
TC1000002828.mm.1	12,63	10,26	5,17	0,00399	0,234922	Syt1
TC1200000910.mm.1	8,63	6,28	5,11	0,008048	0,254946	

TC0800002433.mm.1	8,84	6,49	5,09	0,006293	0,250859	Nxnl1
TC1200001011.mm.1	7,98	5,63	5,08	0,00366	0,230005	C030009J22Rik
TC0700003144.mm.1	6,87	4,55	4,99	0,000559	0,139196	Gm26230
TC0700003153.mm.1	6,87	4,55	4,99	0,000559	0,139196	Gm26498
TC0700002855.mm.1	11,05	8,73	4,98	0,004193	0,238857	Rgs9bp
TC1000001260.mm.1	9,36	7,06	4,93	0,005431	0,247078	Lin7a
TC1300001336.mm.1	8,42	6,12	4,91	0,025552	0,291354	
TC1400002167.mm.1	8,56	6,28	4,87	0,027071	0,29362	
TC1000000852.mm.1	15,81	13,53	4,85	0,027914	0,295218	Reep6
TC1200000817.mm.1	9,11	6,83	4,84	0,004113	0,238149	Vsx2
TC0300000202.mm.1	8,95	6,68	4,83	0,014727	0,272794	Samd7
TC0200003300.mm.1	10,72	8,45	4,8	0,00719	0,253558	Dnm1
TC0800001613.mm.1	9,37	7,11	4,77	0,005005	0,245732	Pcp2
TC1100003358.mm.1	7,1	4,85	4,74	0,005737	0,247208	
TC0900002312.mm.1	9,09	6,85	4,73	0,006658	0,252926	Elmod1
TC1200000922.mm.1	6,95	4,71	4,71	0,002957	0,21851	
TC0900002810.mm.1	12,7	10,46	4,71	0,028962	0,297259	Gm26135
TC0X00000059.mm.1	8,93	6,69	4,7	0,010286	0,26248	Cacna1f
TC1100001162.mm.1	10,64	8,41	4,69	0,004345	0,238857	Vtn
TC1200000899.mm.1	8,43	6,2	4,69	0,004822	0,244766	Nrxn3
TC0900002783.mm.1	9,14	6,91	4,69	0,006977	0,253558	Snap91
TC1200001140.mm.1	8,82	6,6	4,69	0,019237	0,281806	
TC0X00002320.mm.1	10,41	8,18	4,69	0,04007	0,315155	Cdr1
TC0600001268.mm.1	9,08	6,86	4,68	0,00546	0,247078	Slc6a1
TC0800001959.mm.1	7,07	4,85	4,68	0,010043	0,262117	Chrna6
TC0900002869.mm.1	9,14	6,91	4,67	0,002855	0,216977	Trpc1
TC0900000180.mm.1	7,87	5,66	4,64	0,032091	0,301599	Rdh8
TC1000002838.mm.1	8,57	6,37	4,61	0,020295	0,283915	
TC0500002752.mm.1	9,24	7,04	4,57	0,009947	0,2619	Ppef2
TC0600002814.mm.1	8,73	6,53	4,57	0,038627	0,313282	
TC1500002214.mm.1	9,25	7,07	4,53	0,011535	0,263089	Faim2
TC0200002427.mm.1	7,77	5,59	4,52	0,004937	0,245203	Snhg11; Gm25187
TC1300002418.mm.1	8,33	6,15	4,51	0,007891	0,25437	Rasgrf2
TC0100003586.mm.1	8,58	6,42	4,46	0,020408	0,283915	Ackr1
TSUnmapped00000040	10,93	8,78	4,45	0,006577	0,252353	Rorb
TC0700003292.mm.1	7,97	5,83	4,43	0,001126	0,164535	Gm23047
TC0200003313.mm.1	13,34	11,19	4,43	0,009964	0,2619	Stxbp1
TC1500002033.mm.1	7,93	5,79	4,41	0,039624	0,314731	Mlc1
TC1300001338.mm.1	6,78	4,64	4,4	0,00188	0,19434	
TC0500000191.mm.1	7,47	5,33	4,39	0,003541	0,229283	
TC1200000918.mm.1	7,48	5,34	4,38	0,022559	0,287259	
TC1400000392.mm.1	9,08	6,95	4,37	0,01024	0,26248	Lrit1
TC0X00000060.mm.1	7,85	5,74	4,35	0,003854	0,234	Syp
TC0300001686.mm.1	9,85	7,74	4,32	0,007106	0,253558	Gm10742
TC1400002086.mm.1	8,53	6,42	4,32	0,007971	0,254513	Nrl
TC0700002045.mm.1	9,25	7,15	4,29	0,028915	0,297221	Drd4
TC1000002742.mm.1	8,49	6,4	4,26	0,009173	0,260471	
TC0900002375.mm.1	10,02	7,93	4,25	0,004819	0,244766	Cplx3

TC1200000921.mm.1	7,3	5,21	4,25	0,012629	0,266964	
TC0500001971.mm.1	10,14	8,05	4,25	0,015419	0,275022	Gm26954
TC1500000303.mm.1	9,31	7,25	4,17	0,004547	0,239729	Rims2
TC1000003207.mm.1	7,58	5,52	4,17	0,00984	0,2619	Neurod4
TC0200005504.mm.1	8,95	6,89	4,17	0,015267	0,275022	Serinc4
TC0700003149.mm.1	8,63	6,58	4,15	0,010645	0,262531	
TC0900000596.mm.1	7,11	5,05	4,15	0,015164	0,275022	
TC0700003147.mm.1	6,49	4,44	4,14	0,000209	0,102997	Gm26096
TC0700003150.mm.1	6,49	4,44	4,14	0,000209	0,102997	Gm26499
TC0700003216.mm.1	6,25	4,2	4,12	0,001382	0,174934	Gm24310
TC1200001147.mm.1	9,45	7,41	4,12	0,045366	0,322304	Gm24564
TC0200002543.mm.1	9,4	7,37	4,08	0,00283	0,216977	Slc12a5; Gm11459
TC1100003986.mm.1	12,41	10,38	4,08	0,009028	0,260471	Rgs9
TC1200001010.mm.1	8,56	6,54	4,06	0,007179	0,253558	Slc24a4
TC1900000163.mm.1	9,27	7,26	4,04	0,004551	0,239748	LOC102308570; 1810009A15Rik; Gm21743
TC1200000914.mm.1	6,4	4,4	4,02	0,004925	0,245203	
TC0200005014.mm.1	13,16	11,15	4,02	0,012276	0,265369	
TC0700003155.mm.1	6,48	4,48	4,01	0,002772	0,216023	Gm23933
TC0300001130.mm.1	8,14	6,16	3,96	0,007532	0,25437	Gnat2
TC1600001871.mm.1	8,3	6,32	3,95	0,008719	0,260471	
TC1100002553.mm.1	7,25	5,28	3,91	0,003641	0,230005	Gabrg2
TC0700000165.mm.1	7,63	5,66	3,91	0,009571	0,260471	Cabp5
TC1000000224.mm.1	11,87	9,91	3,91	0,010175	0,26248	Epb4.1l2
TC1900000029.mm.1	6,57	4,62	3,88	0,004984	0,245355	Gpr152
TC0X00001921.mm.1	8,71	6,76	3,87	0,001252	0,171857	
TC0600003121.mm.1	9	7,05	3,87	0,008441	0,259938	Gnb3
TC0X00000560.mm.1	18,53	16,58	3,86	0,025066	0,291354	
TC0300002207.mm.1	8,13	6,19	3,84	0,003487	0,228365	Gria2; Gm25749
TC1100002427.mm.1	6,86	4,91	3,84	0,024595	0,290793	
TC0600000261.mm.1	8,65	6,72	3,83	0,00899	0,260471	BB283400
TC1700002502.mm.1	8,83	6,89	3,83	0,010682	0,262531	BC027072
TC1400000847.mm.1	10,42	8,49	3,81	0,006494	0,252353	
TC0900002427.mm.1	8,74	6,82	3,8	0,004541	0,239729	Nr2e3; Gm15507
TC1500001060.mm.1	8,37	6,44	3,8	0,021562	0,285405	Ankrd33
TC1600001859.mm.1	9,36	7,45	3,76	0,00233	0,203813	
TC1000002840.mm.1	7,67	5,76	3,76	0,004954	0,245203	
TC1300001653.mm.1	8,07	6,16	3,76	0,005298	0,247078	Scgn
TC0400003514.mm.1	9,38	7,47	3,75	0,011184	0,263089	Maneal
TC0700003138.mm.1	7,52	5,63	3,72	0,001604	0,18303	Gm24653
TC0700003141.mm.1	7,52	5,63	3,72	0,001604	0,18303	Gm24417
TC0100002606.mm.1	7,9	6,01	3,7	0,005252	0,247078	Ptprn
TC0900002156.mm.1	9,97	8,08	3,7	0,006387	0,251977	C2cd2l
TC1400001568.mm.1	8,13	6,25	3,68	0,005233	0,247078	A830039N20Rik
TC0400003364.mm.1	15,58	13,69	3,68	0,044313	0,321542	Snord55
TC1500000305.mm.1	6,85	4,97	3,67	0,006234	0,250355	
TC1100001154.mm.1	9,26	7,38	3,67	0,011406	0,263089	BC030499
TC0600000933.mm.1	7,18	5,31	3,65	0,031557	0,300123	
TC1400002280.mm.1	12,92	11,07	3,6	0,012042	0,263852	Dpysl2

TC0500003389.mm.1	14,04	12,19	3,6	0,029663	0,297452	Gm25492
TC1100000937.mm.1	9,17	7,32	3,58	0,009162	0,260471	Dlg4
TC1200000901.mm.1	7,27	5,43	3,58	0,018223	0,280415	
TC1200000916.mm.1	6,31	4,48	3,57	0,005712	0,247208	
TC1200000926.mm.1	6,32	4,49	3,57	0,023598	0,289432	
TC0700003270.mm.1	7,13	5,3	3,56	0,002553	0,210008	Gm25870
TC1700000389.mm.1	9,13	7,3	3,56	0,010213	0,26248	Fbxl16
TC1900001072.mm.1	9,53	7,7	3,56	0,028286	0,296148	Gng3
TC0100001812.mm.1	8,08	6,25	3,55	0,005804	0,247436	Ush2a
TC0800002741.mm.1	8,7	6,88	3,53	0,007207	0,25365	Cngb1
TC1000002708.mm.1	6,31	4,49	3,53	0,014732	0,272794	Gm4792
TC1200001850.mm.1	7,07	5,25	3,53	0,020209	0,283915	
TC0700001818.mm.1	9,31	7,5	3,52	0,017808	0,2792	Fam57b
TC1600001860.mm.1	9,54	7,73	3,51	0,025785	0,2919	
TC0300003247.mm.1	9,46	7,66	3,48	0,016964	0,277648	Amy1
TC1200000920.mm.1	7,98	6,19	3,48	0,028707	0,296716	
TC0400001695.mm.1	7,11	5,32	3,47	0,008921	0,260471	Lactbl1
TC0200002836.mm.1	11,86	10,07	3,46	0,009384	0,260471	Pcmtd2
TC1100002099.mm.1	11,47	9,68	3,46	0,012439	0,265589	Osbp2
TC0700003823.mm.1	11,83	10,04	3,46	0,03421	0,305243	Plekhb1
TC0200003637.mm.1	6,25	4,46	3,45	0,004242	0,238857	
TC0100003832.mm.1	6,99	5,2	3,44	0,007256	0,25369	Syt14
TC0800000683.mm.1	7,38	5,6	3,44	0,008806	0,260471	
TC0400001139.mm.1	8,14	6,36	3,43	0,006158	0,250088	Slc1a7
TC0900001380.mm.1	6,77	4,99	3,43	0,007701	0,25437	
TC0100002862.mm.1	6,88	5,12	3,4	0,001956	0,196621	
TC1500000139.mm.1	7,51	5,75	3,4	0,002901	0,217961	Cdh12
TC0200004495.mm.1	6,42	4,65	3,4	0,01008	0,26248	
TC0900002501.mm.1	12,22	10,46	3,39	0,002738	0,216023	Slc24a1
TC1700001756.mm.1	8,48	6,73	3,38	0,004874	0,245203	Tulp1
TC0700000412.mm.1	8,2	6,44	3,38	0,00918	0,260471	Tmem145
TC0100001904.mm.1	7,68	5,93	3,37	0,007009	0,253558	Rp1
TC0200001204.mm.1	10,66	8,91	3,37	0,007781	0,25437	Fam171b
TC1100002557.mm.1	8,22	6,46	3,37	0,011529	0,263089	Gabra1
TC1900000546.mm.1	8,33	6,59	3,36	0,004953	0,245203	Pde6c
TC1200000919.mm.1	6,67	4,92	3,36	0,025021	0,291354	
TC1400001725.mm.1	8,41	6,67	3,35	0,009613	0,260471	Sncg
TC1000002830.mm.1	6,91	5,17	3,35	0,016398	0,275809	
TC0700003426.mm.1	8,26	6,52	3,34	0,015853	0,275467	Gm7546
TC0200003603.mm.1	8,73	7	3,33	0,003228	0,222303	Pla2r1
TC0300002219.mm.1	8,14	6,41	3,33	0,006489	0,252353	Gucy1b3
TC1200000389.mm.1	8,01	6,28	3,32	0,009213	0,260471	Nrcam
TC1200001127.mm.1	8,42	6,68	3,32	0,011715	0,263089	Meg3; Mir770; Mir1906-1; Mir1906-2
TC1200002047.mm.1	7,09	5,36	3,32	0,036071	0,30933	
TC0400003702.mm.1	8,86	7,13	3,31	0,01027	0,26248	Trnp1
TC1400002168.mm.1	8,06	6,33	3,3	0,009591	0,260471	Atp8a2
TC0900002153.mm.1	9,19	7,47	3,3	0,013929	0,270237	Abcg4
TC0900002784.mm.1	6,37	4,65	3,29	0,016353	0,275809	

TC0500002558.mm.1	8,77	7,05	3,29	0,019284	0,281912	Gabra2
TC0100001381.mm.1	8,59	6,88	3,27	0,008916	0,260471	Rgs16
TC1000002408.mm.1	11,22	9,51	3,27	0,0208	0,283915	Pcbp3
TC0400001061.mm.1	8,14	6,43	3,26	0,003069	0,220198	
TC1000001813.mm.1	9,09	7,39	3,25	0,008309	0,257947	Tnfaip3
TC1700001103.mm.1	7,5	5,8	3,25	0,014425	0,272181	
TC0900002275.mm.1	7,17	5,47	3,25	0,043607	0,320281	
TC0900000542.mm.1	7,87	6,17	3,24	0,008743	0,260471	Dscaml1
TC1100003034.mm.1	7,65	5,95	3,24	0,009462	0,260471	Gucy2e
TC0200000258.mm.1	7,52	5,83	3,24	0,015294	0,275022	
TC0200004556.mm.1	9,35	7,65	3,24	0,042301	0,318465	Kcnip3
TC0200000259.mm.1	8,59	6,9	3,24	0,048393	0,325724	
TC0200001524.mm.1	9,3	7,61	3,23	0,008412	0,25964	Slc1a2
TC0500002865.mm.1	8,77	7,07	3,23	0,014869	0,273486	Mapk10
TC0100003831.mm.1	7,12	5,44	3,21	0,002558	0,210008	
TC1600000586.mm.1	6,11	4,44	3,2	0,005319	0,247078	
TC1800001088.mm.1	7,91	6,23	3,2	0,008893	0,260471	Celf4
TC1600001849.mm.1	9,81	8,13	3,2	0,009312	0,260471	Cadm2
TC1300001337.mm.1	7,45	5,78	3,19	0,016045	0,275726	
TC1000002837.mm.1	7,43	5,76	3,19	0,033097	0,304188	
TC0900000593.mm.1	7,44	5,78	3,17	0,036504	0,309778	Drd2
TC1000001374.mm.1	7,99	6,33	3,16	0,008239	0,257054	Mdm1
TC0200004225.mm.1	7,85	6,19	3,16	0,009929	0,2619	A930006I01Rik
TC1200000917.mm.1	7,1	5,45	3,15	0,01378	0,269528	
TC1500000503.mm.1	8,96	7,31	3,14	0,002243	0,201026	Khdrbs3
TC0100003587.mm.1	8,06	6,41	3,14	0,011143	0,263089	Cadm3
TC0900003112.mm.1	9,44	7,79	3,14	0,024345	0,290731	Klhl18
TC0300001567.mm.1	6,78	5,13	3,13	0,001743	0,188761	
TC0300001805.mm.1	8,48	6,84	3,12	0,00583	0,2479	Pex5l
TC1100003511.mm.1	9,37	7,73	3,11	0,004964	0,245203	Dgke
TC0700000913.mm.1	6,57	4,93	3,11	0,012993	0,268831	Slc17a6
TC1300001199.mm.1	8,87	7,24	3,11	0,016724	0,277334	
TC0400004218.mm.1	8,74	7,11	3,1	0,02431	0,290731	Ccdc24; Gm17114
TC0600000765.mm.1	8,71	7,08	3,09	0,005819	0,247596	Ndnf
TSUnmapped00000036	7,83	6,2	3,09	0,010579	0,262531	Ache
TC1000000136.mm.1	6,48	4,86	3,08	0,00677	0,253558	Gm20139
TC1200001711.mm.1	6,55	4,92	3,08	0,048825	0,326603	
TC0700003347.mm.1	6,97	5,35	3,07	0,004726	0,24395	Snord107
TC0400000138.mm.1	6,78	5,16	3,07	0,005783	0,247208	Calb1
TC0700001853.mm.1	12,78	11,16	3,06	0,002315	0,203813	Snora30
TC0X00001317.mm.1	6,89	5,27	3,06	0,007188	0,253558	Arxes1; Arxes2
TC0600003383.mm.1	8,37	6,76	3,05	0,007448	0,25437	St8sia1
TC0500001923.mm.1	7,69	6,08	3,05	0,008873	0,260471	
TC1600001866.mm.1	7,33	5,72	3,05	0,010315	0,26248	
TC1700001038.mm.1	10,5	8,89	3,05	0,014225	0,271237	Gm5815
TC0X00002593.mm.1	7,5	5,89	3,04	0,021973	0,286198	Mir1906-2; Gm27000
TC1600001870.mm.1	9,55	7,95	3,03	0,046818	0,323758	
TC0400004094.mm.1	8,01	6,41	3,02	0,006823	0,253558	Rnf207

TC0500003582.mm.1	6,93	5,34	3,02	0,007244	0,25369	Tmem130
TC0500001588.mm.1	6,83	5,23	3,02	0,014331	0,271614	Gm19359
TC0300001640.mm.1	7,83	6,24	3	0,001876	0,194218	
TC1200001849.mm.1	6,49	4,91	2,98	0,013746	0,269523	
TC0900003041.mm.1	19,93	18,35	2,98	0,048768	0,326556	Gnat1
TC0200000342.mm.1	7,57	6	2,97	0,01365	0,269523	Gpr158
TC0X00001931.mm.1	8	6,43	2,97	0,026809	0,293001	Syn1
TC0200002980.mm.1	8,37	6,8	2,96	0,007051	0,253558	C1ql3
TC0X00003096.mm.1	9,99	8,43	2,96	0,008152	0,255909	Gucy2f
TC1200000669.mm.1	8,01	6,45	2,96	0,014314	0,271614	
TC1600000171.mm.1	7,02	5,46	2,96	0,02228	0,286246	2900011O08Rik
TC1600001853.mm.1	8,07	6,51	2,94	0,007731	0,25437	
TC0600003116.mm.1	8,8	7,24	2,94	0,015461	0,275022	Eno2
TC0500000190.mm.1	8,2	6,64	2,94	0,021373	0,284394	
TC0500000640.mm.1	7,62	6,07	2,93	0,003572	0,229354	Uchl1
TC0200004493.mm.1	8,17	6,62	2,93	0,010067	0,262447	Frmd5
TC0600002580.mm.1	8,04	6,49	2,92	0,013888	0,269879	Vax2os
TC0700000557.mm.1	10,6	9,06	2,92	0,032252	0,301924	Clip3
TC0100003604.mm.1	7,02	5,49	2,91	0,006452	0,252353	Rgs7
TC1000002839.mm.1	7,03	5,5	2,91	0,009852	0,2619	
TC1200002045.mm.1	8,49	6,95	2,91	0,012079	0,263852	Dpf3
TC1100002123.mm.1	8,27	6,73	2,91	0,037734	0,311925	Gm10278
TC1200001930.mm.1	7,14	5,6	2,9	0,006379	0,251977	
TC1800001590.mm.1	14,7	13,17	2,89	0,004653	0,242814	Scarna17
TC0700003142.mm.1	6,62	5,09	2,89	0,026295	0,29242	Gm25121
TC0X00000562.mm.1	6,69	5,16	2,88	0,003782	0,232467	C030023E24Rik
TC0300001641.mm.1	8,57	7,04	2,88	0,006982	0,253558	Fabp12
TC1100003882.mm.1	10,41	8,89	2,88	0,007117	0,253558	Nsf
TC0100002147.mm.1	7,52	5,99	2,88	0,011087	0,263089	
TC0300001857.mm.1	10,41	8,89	2,88	0,02056	0,283915	Bbs7
TC1000000696.mm.1	7,75	6,23	2,88	0,029716	0,297452	Pcdh15
TC1100000235.mm.1	8,96	7,44	2,87	0,004532	0,239729	Fam161a
TC0600003060.mm.1	7,69	6,17	2,87	0,008192	0,256194	Iqsec3
TC0600000250.mm.1	7,07	5,54	2,87	0,008864	0,260471	Strip2
TC1900001144.mm.1	11,98	10,45	2,87	0,010498	0,26248	Stx3
TC0200003301.mm.1	7,94	6,42	2,87	0,033964	0,304873	
TC1600001857.mm.1	7,93	6,41	2,86	0,007574	0,25437	
TC1400001567.mm.1	9,33	7,81	2,86	0,011661	0,263089	Kcnma1
TC1400000908.mm.1	7,05	5,53	2,86	0,024209	0,290301	
TC0200002437.mm.1	7,05	5,54	2,85	0,010799	0,263089	Slc32a1
TC1200000927.mm.1	6,82	5,31	2,84	0,006505	0,252353	
TC0900001695.mm.1	7,63	6,13	2,84	0,00924	0,260471	Gria4; Gm23811
TC0700003159.mm.1	6,32	4,82	2,83	0,027174	0,293695	Gm26466
TC1200000852.mm.1	6,68	5,19	2,82	0,005252	0,247078	Esrrb
TC0700003184.mm.1	7,89	6,4	2,82	0,006597	0,252462	Gm25088
TC0900002228.mm.1	6,46	4,96	2,82	0,008997	0,260471	
TC0900003040.mm.1	13,11	11,62	2,82	0,021126	0,284155	Slc38a3
TC1100001958.mm.1	7,74	6,25	2,81	0,012301	0,265387	

TC1100001407.mm.1	9,71	8,22	2,81	0,030354	0,298161	Car10
TC0200002042.mm.1	7,84	6,35	2,8	0,007452	0,25437	Chgb
TC0100003080.mm.1	8,03	6,54	2,8	0,014337	0,271614	Nfasc
TC0500000422.mm.1	7,68	6,2	2,8	0,014607	0,272794	Crmp1
TC0700002067.mm.1	7	5,51	2,8	0,044256	0,321437	
TC0700003130.mm.1	5,51	4,02	2,79	0,013572	0,269523	Gm22496
TC0600002804.mm.1	7,44	5,96	2,79	0,019622	0,282547	
TC1300001335.mm.1	7,48	6	2,79	0,02259	0,287259	
TC1600000573.mm.1	6,42	4,95	2,78	0,005389	0,247078	
TC0300001242.mm.1	12,52	11,05	2,78	0,005584	0,247078	Abca4
TC1100001158.mm.1	10,55	9,08	2,78	0,007718	0,25437	Unc119
TC1400002028.mm.1	9,52	8,05	2,78	0,012734	0,267554	Snord8
TC1100000027.mm.1	14,09	12,61	2,78	0,014117	0,27069	Gm11953
TC0800001433.mm.1	8,03	6,55	2,78	0,040827	0,316167	Gm15898
TC1700000929.mm.1	6,54	5,07	2,77	0,005801	0,247436	
TC1200000907.mm.1	5,92	4,45	2,77	0,008798	0,260471	
TC0500003687.mm.1	7,1	5,63	2,77	0,012582	0,266408	
TC1000000263.mm.1	6,19	4,72	2,77	0,01943	0,282198	
TC0300001808.mm.1	6,74	5,27	2,77	0,027228	0,293789	Gm19445
TC0500001922.mm.1	6,35	4,87	2,77	0,030017	0,297587	
TC1100002788.mm.1	7,49	6,02	2,76	0,00797	0,254513	Glra1
TC0400000485.mm.1	8,09	6,63	2,76	0,008571	0,260471	Frmpd1
TC1200000915.mm.1	7,5	6,03	2,76	0,013649	0,269523	
TCX_GL456233_randon	10,53	9,06	2,76	0,025329	0,291354	
TC0900001299.mm.1	8,65	7,2	2,74	0,006632	0,252691	Rab6b
TC1800001263.mm.1	6,8	5,35	2,74	0,006913	0,253558	
TC0700004652.mm.1	9,23	7,77	2,74	0,016334	0,275809	Snrpn; Snurf
TC1200001018.mm.1	8,16	6,71	2,74	0,021246	0,284181	Chga
TC0100001950.mm.1	6,61	5,15	2,74	0,034483	0,305819	Ppp1r42
TC1500000049.mm.1	6,39	4,94	2,74	0,0347	0,30634	
TC1500000190.mm.1	5,74	4,29	2,74	0,038846	0,313835	
TC0900000223.mm.1	9,84	8,39	2,73	0,008422	0,259771	Bbs9
TC0600000526.mm.1	8,54	7,09	2,73	0,024786	0,29108	
TC1900000600.mm.1	12,97	11,52	2,73	0,039343	0,314581	Pgam1
TC0800002393.mm.1	8,32	6,88	2,72	0,023387	0,289289	Tmem59l
TC0X00000557.mm.1	7,02	5,57	2,72	0,034535	0,305831	C230004F18Rik
TC1300000573.mm.1	8,75	7,31	2,72	0,039151	0,314192	Gm25394
TC1000000031.mm.1	6,87	5,43	2,71	0,00666	0,252926	Gm10097
TC0300000609.mm.1	7,11	5,67	2,71	0,00953	0,260471	Fstl5
TC0500002820.mm.1	6,28	4,84	2,71	0,027669	0,294745	
TC0200005008.mm.1	7,35	5,91	2,71	0,031604	0,300241	
TC1100001156.mm.1	13,28	11,85	2,7	0,008613	0,260471	Aldoc
TC1200000866.mm.1	7,56	6,12	2,7	0,013813	0,269837	Tmem63c
TC1400002763.mm.1	5,7	4,27	2,69	0,021952	0,286198	
TC1000001826.mm.1	6,87	5,45	2,68	0,006078	0,24932	4930405J17Rik
TC0200000250.mm.1	8,92	7,5	2,68	0,015005	0,274299	Cacnb2
TC0400001374.mm.1	11,41	9,99	2,67	0,004924	0,245203	Gm12892
TC1500001171.mm.1	7,75	6,33	2,67	0,011758	0,263089	Egflam

TC0900001349.mm.1	10,29	8,87	2,67	0,012451	0,265605	Pcbp4
TC0600003316.mm.1	6,2	4,79	2,67	0,01302	0,268831	Gsg1
TC0500000103.mm.1	6,36	4,95	2,66	0,008425	0,259771	
TC1600001861.mm.1	8,03	6,62	2,66	0,012025	0,263852	
TC1100003975.mm.1	8,35	6,94	2,66	0,046625	0,323677	
TC0700001284.mm.1	7,03	5,62	2,65	0,003943	0,234419	Dlg2
TC0200002211.mm.1	16,1	14,69	2,64	0,007476	0,25437	Gm14130
TC0600001463.mm.1	7,34	5,95	2,64	0,009194	0,260471	Ano2
TC1100003974.mm.1	6,04	4,64	2,64	0,01807	0,280198	
TC1100001551.mm.1	6,87	5,48	2,63	0,007264	0,25369	B230217C12Rik
TC0200004594.mm.1	7,25	5,86	2,63	0,015686	0,275467	AI847159
TC0400003171.mm.1	9,15	7,76	2,63	0,023862	0,289432	Wdr78
TC1400001951.mm.1	7,13	5,73	2,62	0,003228	0,222303	
TC0200000929.mm.1	7,78	6,39	2,62	0,008789	0,260471	Slc4a10
TC0700001402.mm.1	7,97	6,59	2,61	0,011987	0,263852	Pgm2l1
TC1400001773.mm.1	5,46	4,07	2,61	0,034941	0,307011	
TC0800001728.mm.1	8,56	7,18	2,6	0,00681	0,253558	Grtp1
TC1200002016.mm.1	16,19	14,81	2,6	0,009031	0,260471	Gm3695
TC0800001675.mm.1	6,08	4,7	2,6	0,009256	0,260471	
TC1000002829.mm.1	6,18	4,8	2,59	0,029688	0,297452	
TC0500002719.mm.1	6,81	5,44	2,58	0,007621	0,25437	Adamts3
TC0100002086.mm.1	7,02	5,65	2,58	0,011231	0,263089	
TC0300000043.mm.1	7,82	6,45	2,58	0,014004	0,270237	Stmn2
TC0200004644.mm.1	6,98	5,61	2,58	0,024291	0,29066	
TC1300000596.mm.1	7,21	5,84	2,58	0,036939	0,310066	Nxnl2
TC0900002405.mm.1	9,32	7,96	2,57	0,012404	0,265577	Bbs4
TC0900001424.mm.1	7,05	5,69	2,57	0,012422	0,265577	Celsr3; Gm23156
TC1100001831.mm.1	7,9	6,54	2,57	0,013371	0,269523	
TC1600001993.mm.1	7,3	5,93	2,57	0,014611	0,272794	Grik1
TC0500000188.mm.1	9,28	7,92	2,57	0,014818	0,273244	Lhfpl3
TC1600001808.mm.1	8,02	6,66	2,57	0,022995	0,287421	
TC0700002475.mm.1	6,6	5,23	2,57	0,028424	0,296309	Ppm1n
TC1300001041.mm.1	12,21	10,86	2,55	0,007755	0,25437	Gm2076
TC0200001023.mm.1	7,52	6,17	2,55	0,013067	0,268831	Gad1
TC1400000909.mm.1	5,92	4,57	2,55	0,017079	0,277705	
TC0600001185.mm.1	6,59	5,24	2,55	0,017722	0,278995	
TC0200004365.mm.1	7,47	6,12	2,55	0,022476	0,286915	
TC0600000766.mm.1	6,55	5,21	2,54	0,00385	0,234	
TC1000002836.mm.1	7,56	6,22	2,54	0,00595	0,249013	
TC0X00000561.mm.1	6,39	5,05	2,54	0,007496	0,25437	
TC0600002550.mm.1	9,05	7,7	2,54	0,007968	0,254513	Hk2
TC1300001395.mm.1	11,2	9,85	2,54	0,008771	0,260471	Pfkp
TC1900000229.mm.1	15,65	14,3	2,54	0,011166	0,263089	Gm25443
TC1100000549.mm.1	7,07	5,72	2,54	0,025165	0,291354	Grm6
TC1600001855.mm.1	6,11	4,77	2,53	0,004354	0,238857	
TC0300001206.mm.1	6,95	5,6	2,53	0,006796	0,253558	4833424O15Rik
TC0700003388.mm.1	7,96	6,62	2,53	0,011439	0,263089	
TC0900002682.mm.1	16,41	15,07	2,53	0,014708	0,272794	Gm3671

TC0300000871.mm.1	7,72	6,38	2,53	0,020173	0,283915	Celf3
TC0200005004.mm.1	10,34	9	2,53	0,023142	0,287936	Gm14278
TC0900001940.mm.1	7,9	6,56	2,53	0,040178	0,315423	Glb1l3
TC0500002947.mm.1	8,07	6,73	2,52	0,009375	0,260471	Cplx1
TC0500000214.mm.1	6,52	5,18	2,52	0,018319	0,280415	Asic3
TC1300002525.mm.1	7,72	6,39	2,52	0,031227	0,299554	Cartpt
TC0X00001920.mm.1	9,25	7,92	2,51	0,015112	0,275022	
TC0100002096.mm.1	5,28	3,95	2,51	0,021622	0,285544	
TC1500001172.mm.1	5,32	3,99	2,51	0,025884	0,292119	
TC0600001669.mm.1	7	5,68	2,5	0,003802	0,23326	
TC1200002113.mm.1	6,67	5,35	2,5	0,011069	0,263089	Zdhhc22
TC1800000300.mm.1	8,75	7,43	2,5	0,014521	0,27237	Reep2
TC0100003382.mm.1	8,53	7,21	2,5	0,02296	0,287421	Dnm3
TC0200001395.mm.1	6	4,69	2,49	0,009809	0,2619	
TC0200000801.mm.1	7,47	6,15	2,49	0,014655	0,272794	Kif5c
TC1300002722.mm.1	6,57	5,26	2,49	0,020241	0,283915	Isl1
TC1200001931.mm.1	8,08	6,76	2,49	0,020878	0,283915	Kcnh5
TC1300002501.mm.1	17,17	15,86	2,48	0,023228	0,288497	Gm2445
TC1000000963.mm.1	5,99	4,69	2,47	0,003016	0,21946	
TC1200002044.mm.1	5,63	4,33	2,47	0,010522	0,262531	
TC0500003532.mm.1	7,38	6,08	2,47	0,02542	0,291354	Mmd2
TC0100002099.mm.1	7,36	6,06	2,47	0,034147	0,305243	
TC1900000027.mm.1	8,7	7,4	2,46	0,011654	0,263089	Pitpnm1
TC0900000890.mm.1	6,69	5,39	2,46	0,016375	0,275809	Gm22962
TC0300000616.mm.1	16,99	15,69	2,46	0,016397	0,275809	Gm10291
TC1600000565.mm.1	8,46	7,17	2,46	0,021005	0,284071	Gm22500
TC0600000557.mm.1	7,06	5,76	2,46	0,026639	0,293001	
TC0900002860.mm.1	6,88	5,58	2,46	0,039135	0,314192	Gm16262
TC0900001939.mm.1	7,69	6,4	2,45	0,007437	0,25437	Glb1l2
TC0X00002407.mm.1	7,76	6,47	2,45	0,019426	0,282198	Gabra3
TCX_GL456233_randon	7,52	6,23	2,45	0,020294	0,283915	Spry3
TC0200005006.mm.1	10,96	9,67	2,45	0,020672	0,283915	Ndrg3
TC0900001956.mm.1	6,97	5,68	2,45	0,046364	0,323556	
TC0600001974.mm.1	8,06	6,77	2,44	0,006424	0,252353	Impdh1
TC1600001869.mm.1	7,99	6,71	2,44	0,017414	0,278371	
TC1500000307.mm.1	6,45	5,17	2,43	0,002773	0,216023	
TC1100003644.mm.1	7,51	6,23	2,43	0,009182	0,260471	Gpr179
TC1200001704.mm.1	7,66	6,38	2,43	0,014797	0,273232	Prkd1
TC1200002046.mm.1	6,34	5,06	2,43	0,02141	0,284424	
TC1000000299.mm.1	8,78	7,5	2,43	0,022839	0,287286	Tspyl4
TC1600000564.mm.1	8,5	7,23	2,43	0,037034	0,310448	Igsf11
TC0700000023.mm.1	8,53	7,25	2,42	0,013364	0,269523	Ttyh1
TC0500003118.mm.1	7,17	5,89	2,42	0,015244	0,275022	
TC0900002369.mm.1	11,1	9,83	2,42	0,021525	0,285137	Scamp5
TC1300002520.mm.1	11,48	10,21	2,42	0,030439	0,298164	Map1b
TC1200000909.mm.1	6,34	5,07	2,42	0,041858	0,317735	
TC0500002320.mm.1	12,87	11,61	2,41	0,009454	0,260471	Prom1
TC0X00002589.mm.1	14,63	13,36	2,41	0,016565	0,27632	Gm8864

TC0200004199.mm.1	11,07	9,81	2,41	0,020285	0,283915	Gm13810
TC1200001929.mm.1	6,38	5,11	2,41	0,023594	0,289432	
TC0100003183.mm.1	9,42	8,15	2,4	0,006457	0,252353	Crb1
TC1200000912.mm.1	6,1	4,84	2,4	0,00994	0,2619	
TC1800001264.mm.1	6,73	5,47	2,4	0,022562	0,287259	
TC1200000911.mm.1	6,07	4,81	2,39	0,008718	0,260471	
TC1200001842.mm.1	6,64	5,38	2,39	0,029117	0,297285	
TC1300000126.mm.1	14,21	12,96	2,38	0,009479	0,260471	Gm18859
TC1900001298.mm.1	14,94	13,69	2,38	0,010778	0,263089	Gm8825
TC0600001922.mm.1	9,96	8,71	2,38	0,013634	0,269523	Fam3c
TC1000000342.mm.1	14,44	13,18	2,38	0,014864	0,273486	Gm8055
TC0500001783.mm.1	8,5	7,25	2,38	0,029259	0,297353	Wasf3
TC1600002002.mm.1	5,72	4,47	2,38	0,030291	0,298056	
TC0300001573.mm.1	7,83	6,59	2,37	0,004276	0,238857	
TC1000002831.mm.1	6,08	4,83	2,37	0,006349	0,251655	
TC0400002606.mm.1	7,06	5,82	2,37	0,007522	0,25437	Gabbr2
TC0600002815.mm.1	6,14	4,89	2,37	0,011925	0,263453	A930015G24Rik
TC0X00001419.mm.1	6,53	5,29	2,37	0,016882	0,277375	A730046J19Rik
TC0700003825.mm.1	17,35	16,1	2,37	0,018886	0,281433	Gm3200
TC1100001598.mm.1	15,53	14,28	2,37	0,022931	0,287421	Gm11557
TC1400000359.mm.1	7,4	6,16	2,36	0,00888	0,260471	Gm626
TC0500001260.mm.1	8,8	7,57	2,36	0,020939	0,283915	Msi1
TC0900001960.mm.1	6,86	5,63	2,35	0,005257	0,247078	
TC0X00003062.mm.1	10,7	9,47	2,35	0,007866	0,25437	Gm6322
TC1200000554.mm.1	4,28	3,04	2,35	0,021616	0,285544	
TC0900001487.mm.1	6,46	5,24	2,34	0,007072	0,253558	Trank1
TC1100001397.mm.1	7,88	6,66	2,34	0,010975	0,263089	Mmd
TC0X00001316.mm.1	7,8	6,58	2,34	0,01684	0,277334	Arxes2; Arxes1
TC0700003349.mm.1	5,8	4,57	2,34	0,025585	0,291354	
TC1200001163.mm.1	5,08	3,85	2,34	0,040054	0,315138	Gm22205
TC0700003107.mm.1	6,52	5,3	2,34	0,044192	0,32136	Gabrg3
TC0200002072.mm.1	6,66	5,44	2,33	0,011286	0,263089	Lamp5
TC0600001394.mm.1	10,62	9,4	2,33	0,013159	0,269523	Necap1
TC1800000546.mm.1	16,15	14,93	2,33	0,017557	0,278824	Gm5507
TC1100003966.mm.1	9,96	8,75	2,33	0,017835	0,2792	Prkca
TC0900001477.mm.1	7,91	6,69	2,33	0,01882	0,281433	Lrrc2
TC0600002805.mm.1	7,55	6,33	2,32	0,01924	0,281806	
TC1500000306.mm.1	7,46	6,25	2,32	0,023154	0,288035	
TC1600001863.mm.1	6,17	4,96	2,32	0,044598	0,32191	
TC1100000109.mm.1	7,74	6,53	2,31	0,003587	0,229561	Adcy1
TC0600002354.mm.1	10,17	8,97	2,31	0,012069	0,263852	Vopp1
TC1400002335.mm.1	10,04	8,83	2,31	0,0125	0,266152	Ppp3cc
TC1300002116.mm.1	9,68	8,47	2,31	0,034407	0,305819	Agtpbp1; A230056J06Rik
TC0400000774.mm.1	6,83	5,63	2,3	0,009934	0,2619	Frmd3
TC1300000135.mm.1	10,59	9,39	2,3	0,02807	0,295801	Amph
TC1500002060.mm.1	5,87	4,67	2,29	0,006509	0,252353	
TC1500000917.mm.1	16,01	14,82	2,29	0,007498	0,25437	Gm4335
TC0300000307.mm.1	9,96	8,76	2,29	0,009724	0,261145	Hspa4l

TC1100000713.mm.1	7,63	6,43	2,29	0,014542	0,272469	Trim17; Hist3h2a	
TC1000001025.mm.1	10,92	9,72	2,29	0,019244	0,281806	Ric8b	
TC1000002256.mm.1	9,59	8,4	2,28	0,005761	0,247208	Hk1	
TC0400000432.mm.1	9,69	8,5	2,28	0,008047	0,254946	Unc13b	
TC1100002630.mm.1	15,73	14,54	2,28	0,009485	0,260471	Gm12182	
TC1100003999.mm.1	9,45	8,27	2,28	0,012582	0,266408	Abca8a	
TC0600000556.mm.1	11,42	10,23	2,28	0,013317	0,269523	Atp6v0e2	
TC0100000551.mm.1	9,02	7,83	2,28	0,013984	0,270237	Map2; A730034C02	
TC1300001334.mm.1	6,43	5,24	2,28	0,015318	0,275022		
TC0X00003339.mm.1	6,3	5,1	2,28	0,020532	0,283915	Glra2	
TC1800001622.mm.1	5,71	4,52	2,28	0,029105	0,297268		
TC0X00000175.mm.1	6,51	5,32	2,28	0,029776	0,297452	Gm14505	
TC0400001630.mm.1	9,75	8,56	2,27	0,00571	0,247208	E130218I03Rik	
TC1100003472.mm.1	7,97	6,79	2,27	0,009857	0,2619	Ppm1e	
TC0300002765.mm.1	7,59	6,41	2,27	0,01277	0,267802	Ntng1	
TC0200002259.mm.1	16,25	15,07	2,27	0,014037	0,27033	Gm14148	
TC0300001562.mm.1	8,66	7,48	2,27	0,015947	0,275633	Negr1	
TC1400000907.mm.1	6,44	5,26	2,27	0,049216	0,326995		
TC1100002025.mm.1	7,09	5,91	2,26	0,009309	0,260471	Fscn2	
TC1100002957.mm.1	18,45	17,27	2,26	0,010467	0,26248	Gm12286	
TC1000002870.mm.1	8,75	7,58	2,26	0,013006	0,268831	Zdhhc17	
TC0500001918.mm.1	6,41	5,23	2,26	0,026843	0,293001	Zfp804b	
TC0200002839.mm.1	8,28	7,11	2,26	0,037209	0,310867		
TC1000000407.mm.1	11,06	9,89	2,25	0,024843	0,291092	Gm9034	
TC1300001169.mm.1	8,2	7,04	2,24	0,010212	0,26248	Sgtb	
TC0600000064.mm.1	4,97	3,81	2,24	0,01212	0,263852		
TC1100001769.mm.1	6,83	5,66	2,24	0,013341	0,269523	Kcnh6	
TC0500000814.mm.1	10,21	9,05	2,24	0,015626	0,275467	Rufy3	
TC0300001022.mm.1	6,71	5,55	2,24	0,033682	0,304791	Nhlh2	
TC1800001111.mm.1	8,7	7,53	2,24	0,046443	0,323595	Syt4	
TC1000000352.mm.1	10,62	9,46	2,23	0,002621	0,212518		
TC1300000483.mm.1	6,65	5,5	2,23	0,00778	0,25437		
TC0700002065.mm.1	7,65	6,49	2,23	0,008807	0,260471	Brsk2; Mir3104	
TC0200003028.mm.1	5,96	4,81	2,23	0,009558	0,260471		
TC0400002712.mm.1	6,08	4,93	2,22	0,013952	0,270237	Frrs1l	
TC0700003134.mm.1	6,38	5,23	2,22	0,016203	0,275809	Gm25156	
TC1500001206.mm.1	14,26	13,11	2,22	0,016657	0,277084	Gm8174	
TC1400000281.mm.1	7,74	6,59	2,22	0,021266	0,284224	Erc2	
TC1800000472.mm.1	5,96	4,81	2,22	0,035297	0,307361		
TC0400000585.mm.1	7,73	6,59	2,21	0,010382	0,26248	Fsd1l	
TC1200002048.mm.1	6,75	5,61	2,21	0,014719	0,272794		
TC1500000747.mm.1	7,17	6,02	2,21	0,015131	0,275022		sept-03
TC0700003383.mm.1	6,44	5,3	2,21	0,017182	0,278145		
TC1800001563.mm.1	6,05	4,9	2,21	0,04455	0,321903		
TC1300002370.mm.1	6,65	5,52	2,2	0,004665	0,243233	Gm26803	
TC1400002559.mm.1	7,73	6,6	2,2	0,0101	0,26248		
TC0100002081.mm.1	7,02	5,88	2,2	0,010222	0,26248	Bai3	
TC0900001243.mm.1	8,07	6,93	2,2	0,01562	0,275467	Faim	

TC0400002969.mm.1	12,58	11,45	2,2	0,017658	0,278927	Gm12419
TC0600000501.mm.1	5,92	4,78	2,2	0,033461	0,304293	
TC1200001705.mm.1	5,83	4,7	2,19	0,004965	0,245203	
TC0400002077.mm.1	18,1	16,97	2,19	0,00566	0,247208	Gnb1
TC0X00000808.mm.1	9,36	8,23	2,19	0,019218	0,281806	Dmd
TC0500003117.mm.1	7,03	5,9	2,19	0,028872	0,297221	Srrm4
TC1200000933.mm.1	6,97	5,83	2,19	0,03792	0,312029	
TC0500003182.mm.1	7,43	6,3	2,19	0,045792	0,322616	Rph3a
TC1400002552.mm.1	7,07	5,94	2,19	0,049335	0,327118	
TC1200002096.mm.1	6,53	5,41	2,18	0,015876	0,275467	
TC0600000888.mm.1	5,73	4,61	2,18	0,016122	0,275809	
TC0200003759.mm.1	8,15	7,03	2,18	0,018218	0,280415	Chn1
TC1100002256.mm.1	15,49	14,37	2,18	0,019184	0,281806	Gm12017
TC1700002660.mm.1	11,39	10,27	2,18	0,02811	0,29591	Prepl
TC0800002218.mm.1	6,79	5,67	2,18	0,034924	0,307011	
TC0300000494.mm.1	7,53	6,41	2,17	0,007478	0,25437	Arhgef26
TC0600000714.mm.1	10,5	9,38	2,17	0,010415	0,26248	Herc3
TC0100001262.mm.1	8,08	6,96	2,17	0,011562	0,263089	Kif21b
TC0300001807.mm.1	6,37	5,25	2,17	0,018948	0,281527	
TC0500002927.mm.1	9,41	8,3	2,17	0,02033	0,283915	Glmn
TC0800000684.mm.1	6,53	5,41	2,17	0,02561	0,291354	
TC0100002097.mm.1	6,03	4,91	2,17	0,033232	0,304188	
TC1300002041.mm.1	7,24	6,12	2,17	0,034466	0,305819	Sncb
TC1800001107.mm.1	8,87	7,76	2,17	0,041152	0,316857	Rit2
TC0300002504.mm.1	9,34	8,23	2,16	0,001531	0,179977	Hist2h4; Hist1h4m; Hist1h4b; Hist1h4a; Hist4h4;
TC1000002448.mm.1	12,14	11,03	2,16	0,001619	0,184076	Agpat3
TC0600003123.mm.1	6,84	5,73	2,16	0,005701	0,247208	Gpr162
TC0900000751.mm.1	13,12	12,02	2,16	0,008012	0,254675	Pkm
TC1800001749.mm.1	7,45	6,34	2,16	0,009155	0,260471	Ppp2r2b
TC0100003627.mm.1	12,33	11,21	2,16	0,009276	0,260471	Akt3
TC0500000248.mm.1	7,25	6,14	2,16	0,010475	0,26248	Dpp6
TC0600001845.mm.1	6,82	5,7	2,16	0,015329	0,275022	Gm16055
TC0200001189.mm.1	6,25	5,14	2,16	0,018326	0,280415	Zfp804a
TC0400004097.mm.1	6,97	5,86	2,16	0,02462	0,290852	Kcnab2; Gm16334
TC1100003233.mm.1	7,59	6,48	2,16	0,025513	0,291354	Doc2b
TC0700003346.mm.1	7,91	6,8	2,16	0,030092	0,297587	Snord64
TC0700003387.mm.1	8,66	7,55	2,16	0,03429	0,305337	A330076H08Rik; Mir344g
TC1600001868.mm.1	8,36	7,25	2,16	0,046229	0,323218	
TC0500000104.mm.1	5,07	3,97	2,15	0,002812	0,216199	
TC0100002827.mm.1	11,8	10,69	2,15	0,012369	0,265476	Kif1a
TC0600001713.mm.1	8,77	7,67	2,15	0,013249	0,269523	Gm15706
TC0400000844.mm.1	14,26	13,16	2,15	0,018101	0,280198	Gm12416
TC1000002316.mm.1	7,56	6,46	2,15	0,018833	0,281433	Zfp365
TC0800002159.mm.1	12,2	11,09	2,15	0,022169	0,286246	
TC1200000913.mm.1	6	4,9	2,15	0,022683	0,287259	
TC1600001435.mm.1	5,29	4,18	2,15	0,032438	0,302689	
TC1300001661.mm.1	7,29	6,19	2,15	0,043629	0,320281	Gm11344; Gm11345; Gm11342
TC0100001860.mm.1	7,16	6,05	2,15	0,044057	0,321118	Rd3

TC0200003828.mm.1	7,45	6,35	2,14	0,00533	0,247078	Cerkl; Neurod1
TC0300002065.mm.1	18,39	17,29	2,14	0,00613	0,2499	Gm8349
TC1400001462.mm.1	4,73	3,63	2,14	0,010274	0,26248	
TC1200001443.mm.1	13,53	12,44	2,14	0,011825	0,263089	Gm4929
TC1300002006.mm.1	9,65	8,55	2,14	0,01472	0,272794	Diras2
TC0200004786.mm.1	17,18	16,08	2,14	0,017938	0,279624	Gm14111
TC0X00000559.mm.1	6,99	5,89	2,14	0,026891	0,293001	
TC0900002257.mm.1	8,39	7,29	2,14	0,043907	0,320692	
TC1200002250.mm.1	5,61	4,51	2,13	0,000676	0,14969	
TC0700001607.mm.1	6,91	5,82	2,13	0,005355	0,247078	Tub; BC049265
TC0200002583.mm.1	6,24	5,14	2,13	0,006873	0,253558	
TC0500002315.mm.1	10,65	9,56	2,13	0,009284	0,260471	Fbxl5
TC1000000853.mm.1	8,78	7,69	2,13	0,011328	0,263089	Plk5
TC1200002195.mm.1	10,24	9,14	2,13	0,011398	0,263089	Eml5
TC0400000777.mm.1	10,19	9,1	2,13	0,011927	0,263453	Kdm4c
TC1600000939.mm.1	6,8	5,71	2,13	0,017678	0,278927	Gm25908
TC0300002901.mm.1	10,18	9,1	2,13	0,018231	0,280415	Ank2; Gm4392
TC0600000260.mm.1	5,48	4,39	2,13	0,019473	0,282461	
TC0500001526.mm.1	11,76	10,67	2,12	0,006211	0,250355	Rabgef1
TC1400002186.mm.1	8,31	7,23	2,12	0,00643	0,252353	Ebpl
TC0200003674.mm.1	6,43	5,35	2,12	0,012178	0,264711	Scn1a
TC0500000992.mm.1	11,13	10,04	2,12	0,018109	0,280198	Cds1
TC0X00001652.mm.1	6,24	5,17	2,11	0,001623	0,184196	Gm16459
TC1000000704.mm.1	7,58	6,51	2,11	0,00473	0,24395	Gnaz
TC0600002083.mm.1	5,86	4,78	2,11	0,008184	0,256194	
TC1600000589.mm.1	6,07	4,99	2,11	0,012234	0,265149	
TC0800001143.mm.1	8,16	7,08	2,11	0,018151	0,280372	Ndrg4
TC0X00001176.mm.1	15,96	14,89	2,1	0,005517	0,247078	Gm5863
TC1800001265.mm.1	7,69	6,61	2,1	0,017112	0,277853	
TC1000002843.mm.1	6,27	5,2	2,1	0,019546	0,282547	
TC0500002321.mm.1	9,7	8,63	2,1	0,021078	0,284155	
TC1200001142.mm.1	7,19	6,13	2,1	0,023683	0,289432	Rian; AF357355; AF357359; AF357425; DQ267100;
TC0900001892.mm.1	6,78	5,71	2,1	0,024322	0,290731	Elavl3
TC1000003067.mm.1	6,11	5,04	2,1	0,025158	0,291354	
TC0100000555.mm.1	7,91	6,84	2,1	0,028036	0,295551	
TC1500000229.mm.1	7,53	6,46	2,1	0,030389	0,298161	
TC0900002809.mm.1	6,94	5,87	2,1	0,035727	0,308433	
TC0X00000825.mm.1	6,14	5,07	2,1	0,044772	0,321918	Gm14770
TC1000000160.mm.1	9,37	8,31	2,09	0,009797	0,2619	Ahi1
TC0200000850.mm.1	6,98	5,92	2,09	0,01611	0,275809	Galnt13
TC0100002696.mm.1	7,02	5,95	2,09	0,01689	0,277375	Dner
TC0900000493.mm.1	5,32	4,26	2,09	0,022263	0,286246	Gm3898
TC1000001261.mm.1	6,13	5,07	2,09	0,025956	0,292119	
TC0500000189.mm.1	6,37	5,31	2,09	0,033472	0,304293	Gm21846
TC0400004035.mm.1	12,79	11,73	2,08	0,003843	0,234	Kif1b
TC1000002126.mm.1	4,98	3,92	2,08	0,005387	0,247078	
TC0100002321.mm.1	7,54	6,49	2,08	0,006235	0,250355	Gm5976
TC1900000942.mm.1	7,09	6,03	2,08	0,006349	0,251655	

TC1500002042.mm.1	7,7	6,64	2,08	0,016932	0,277516	Dennd6b
TC0200004539.mm.1	6,53	5,47	2,08	0,019082	0,281691	Hdc
TC0200003245.mm.1	7,5	6,44	2,08	0,020008	0,283915	Ntng2; 6530402F18Rik
TC0900001385.mm.1	6,63	5,58	2,08	0,044229	0,321416	Camkv
TC0X00002771.mm.1	5,76	4,71	2,07	0,004445	0,239131	Nap1l2
TC1200001380.mm.1	12,93	11,88	2,07	0,013537	0,269523	Gm5652
TC0900002207.mm.1	15,52	14,47	2,07	0,014819	0,273244	Gm7286
TC0300000605.mm.1	6,67	5,62	2,07	0,015292	0,275022	Serpini1
TC0300000928.mm.1	8,94	7,9	2,07	0,017069	0,277705	Sv2a
TC0900003298.mm.1	7,93	6,87	2,07	0,021805	0,286107	
TC1100003297.mm.1	6,16	5,12	2,07	0,022686	0,287259	Sarm1
TC1500000524.mm.1	8,31	7,26	2,07	0,03065	0,298781	Ptp4a3
TC0300001565.mm.1	5,81	4,76	2,07	0,033062	0,304133	
TC1300001098.mm.1	13,81	12,76	2,07	0,033491	0,304293	Fam169a
TC1500001320.mm.1	7,58	6,54	2,06	0,008315	0,257947	Ankrd33b
TC0700003389.mm.1	4,5	3,46	2,06	0,008578	0,260471	Mir344-2
TC1800001562.mm.1	5,22	4,18	2,06	0,016994	0,277705	
TC1900001599.mm.1	7,34	6,3	2,06	0,020001	0,283915	Psd
TC0700004626.mm.1	7,29	6,24	2,06	0,035082	0,307135	Sez6l2
TC1200001911.mm.1	7,89	6,86	2,05	0,002629	0,212576	4930447C04Rik; Six6os1
TC1400001146.mm.1	8,31	7,28	2,05	0,003219	0,222303	Gm25133
TC1700002532.mm.1	8,17	7,14	2,05	0,009201	0,260471	
TC1500000064.mm.1	9,88	8,84	2,05	0,010682	0,262531	Nadk2; Nadkd1
TC0500000096.mm.1	8,26	7,22	2,05	0,011138	0,263089	Pclo
TC0X00000873.mm.1	13,27	12,24	2,05	0,019941	0,283759	Gm14777
TC0800000765.mm.1	7,98	6,94	2,05	0,023103	0,287831	Rab3a
TC1700001602.mm.1	8,5	7,47	2,05	0,025482	0,291354	Paqr4
TC1400000529.mm.1	17,24	16,2	2,05	0,036089	0,30933	Gm3534
TC0600001092.mm.1	15,45	14,43	2,04	0,001645	0,184786	Slc6a6
TC1400002553.mm.1	7,05	6,02	2,04	0,004749	0,244138	
TC1200001843.mm.1	6,6	5,57	2,04	0,00744	0,25437	Mdga2
TC1600000584.mm.1	5,74	4,71	2,04	0,009604	0,260471	
TC1300002453.mm.1	7,78	6,75	2,04	0,012527	0,266152	Pde8b
TC1200000432.mm.1	7,32	6,28	2,04	0,022802	0,287259	Akap6
TC0600001732.mm.1	9,65	8,62	2,04	0,033339	0,304188	
TC1200001153.mm.1	5,3	4,27	2,04	0,039137	0,314192	
TC1000001026.mm.1	7,87	6,85	2,04	0,039509	0,314658	
TC0500001785.mm.1	9,61	8,57	2,04	0,039972	0,315004	
TC0500003276.mm.1	9,18	8,15	2,04	0,040677	0,315825	
TC1100003356.mm.1	7,08	6,06	2,03	0,006162	0,250096	1700071K01Rik
TC0200004364.mm.1	8,65	7,63	2,03	0,010656	0,262531	Scg5
TC0800002145.mm.1	8,35	7,33	2,03	0,033997	0,30488	Fam149a
TC0700004240.mm.1	6,97	5,95	2,03	0,039787	0,314936	
TC0100000157.mm.1	5,57	4,55	2,02	0,007622	0,25437	Gm7784
TC1300001997.mm.1	6,74	5,73	2,02	0,011664	0,263089	Shc3
TC1400001059.mm.1	5,8	4,79	2,02	0,012293	0,265387	
TC1100003064.mm.1	7,56	6,55	2,02	0,0137	0,269523	Fgf11
TC0900001581.mm.1	10,59	9,58	2,02	0,014058	0,270397	

TC0200000347.mm.1	6,41	5,4	2,02	0,015217	0,275022	Gad2
TC1100004116.mm.1	6,85	5,84	2,02	0,015711	0,275467	
TC0700003424.mm.1	5,63	4,62	2,02	0,039162	0,314192	
TC0100002057.mm.1	6,06	5,05	2,01	0,002436	0,209054	
TC0200004108.mm.1	8,66	7,66	2,01	0,012288	0,265387	Madd
TC0100000564.mm.1	5,44	4,43	2,01	0,016126	0,275809	
TC1800001564.mm.1	6,02	5,01	2,01	0,026206	0,292165	
TC0800002157.mm.1	15,31	14,3	2,01	0,02657	0,293001	Gm10313
TC0700000038.mm.1	7,28	6,27	2,01	0,029009	0,297268	Brsk1
TC1200001851.mm.1	5,92	4,91	2,01	0,038147	0,312312	
TC0300003248.mm.1	10,48	9,47	2,01	0,049713	0,327707	Rnpc3
TC0200000776.mm.1	4,69	5,7	-2,01	0,013533	0,269523	Gm13482
TC0300000232.mm.1	4,74	5,75	-2,01	0,015828	0,275467	
TC1600001297.mm.1	6,69	7,71	-2,02	0,005578	0,247078	
TC0X00003352.mm.1	6,3	7,32	-2,02	0,007372	0,25437	
TC0400003324.mm.1	3,89	4,91	-2,02	0,007572	0,25437	
TC0700001346.mm.1	6,16	7,17	-2,02	0,007813	0,25437	
TC0200000203.mm.1	6,75	7,76	-2,02	0,012912	0,268831	Gm24806
TC1600000488.mm.1	7,05	8,06	-2,02	0,017388	0,278371	
TC1300001823.mm.1	3,78	4,79	-2,02	0,026838	0,293001	
TC0600003305.mm.1	7,57	8,58	-2,02	0,031125	0,299464	Mansc1
TC0300000692.mm.1	10,98	12,01	-2,03	0,005451	0,247078	
TC1600000210.mm.1	10,98	12,01	-2,03	0,005451	0,247078	
TC1500001140.mm.1	7,47	8,49	-2,03	0,015852	0,275467	
TC1800000601.mm.1	6,57	7,6	-2,03	0,018772	0,281433	
TC0800001206.mm.1	7,75	8,77	-2,03	0,030692	0,298801	Gm24629
TC0900001464.mm.1	6,74	7,76	-2,03	0,03957	0,314668	
TC0700000659.mm.1	4,58	5,6	-2,04	0,002716	0,215508	
TC1000003037.mm.1	3,7	4,72	-2,04	0,005774	0,247208	
TC1500001768.mm.1	3,91	4,94	-2,04	0,023142	0,287936	Mir1942
TC1500001141.mm.1	6,52	7,56	-2,05	0,004831	0,244766	
TC0500003695.mm.1	7,36	8,4	-2,05	0,033799	0,304873	
TC0900000022.mm.1	10,33	11,36	-2,05	0,0425	0,31869	
TC1600001785.mm.1	3,88	4,92	-2,06	0,007678	0,25437	Olfr181
TC0700003509.mm.1	3,77	4,81	-2,06	0,008021	0,254814	
TC0100003684.mm.1	9,84	10,88	-2,06	0,030154	0,297733	
TC0X00001737.mm.1	6,02	7,06	-2,06	0,031608	0,300241	Gm14368
TC0800001691.mm.1	5,21	6,25	-2,06	0,034455	0,305819	Gm2814
TC0X00000015.mm.1	5,64	6,69	-2,07	0,023993	0,290042	Gm14358
TC1400002869.mm.1	8,22	9,27	-2,07	0,031075	0,299432	Phf11d; Phf11c
TC1500001959.mm.1	8,48	9,52	-2,07	0,045633	0,322616	
TC0200000277.mm.1	6,86	7,91	-2,08	0,0001	0,094374	Gm20038
TC0X00001702.mm.1	5,62	6,68	-2,08	0,01187	0,263182	
TC0500001199.mm.1	4,91	5,97	-2,08	0,012392	0,265577	
TC1000002116.mm.1	4,87	5,93	-2,08	0,022154	0,286246	Gm23481
TC0X00000005.mm.1	5,39	6,45	-2,08	0,03277	0,303482	Gm14360
TC0200003734.mm.1	7,04	8,1	-2,08	0,044692	0,32191	
TC0100000345.mm.1	9,93	11	-2,09	0,007176	0,253558	

TC0400000515.mm.1	9,93	11	-2,09	0,007176	0,253558	
TC0X00001560.mm.1	9,93	11	-2,09	0,007176	0,253558	
TC0X00001703.mm.1	5,8	6,86	-2,09	0,00867	0,260471	
TC1900000504.mm.1	6,63	7,69	-2,09	0,009839	0,2619	Ifit1
TC1600001786.mm.1	4,17	5,24	-2,09	0,013641	0,269523	Olfr186
TC0X00000544.mm.1	6,53	7,59	-2,09	0,032934	0,303834	Gm26487
TC0X00001271.mm.1	7,33	8,4	-2,1	0,001009	0,163425	Srpx2
TC0100002046.mm.1	7,77	8,83	-2,1	0,017288	0,278371	
TC1600001728.mm.1	8,21	9,29	-2,11	0,000418	0,120143	
TC1900001245.mm.1	3,91	4,99	-2,11	0,001867	0,193869	
TC0600003317.mm.1	11,32	12,41	-2,12	0,001301	0,173117	Pbp2
TC1400002580.mm.1	6,27	7,35	-2,12	0,004209	0,238857	
TC0100001357.mm.1	4,74	5,83	-2,12	0,014939	0,273837	
TC1600001911.mm.1	6,09	7,18	-2,13	0,025272	0,291354	
TC1900001323.mm.1	7,32	8,41	-2,13	0,033671	0,304791	Glis3
TC1400001449.mm.1	5,57	6,66	-2,13	0,047316	0,324405	
TC1800001451.mm.1	4,44	5,53	-2,14	0,003097	0,220443	
TC1700002642.mm.1	4,69	5,8	-2,15	0,019139	0,281806	
TC1100000989.mm.1	7,78	8,89	-2,16	0,000078	0,094374	Xaf1
TC1100003507.mm.1	10,16	11,27	-2,16	0,030312	0,298107	Gm15698
TC1300000274.mm.1	8,43	9,54	-2,17	0,006507	0,252353	
TC0300000557.mm.1	8,36	9,48	-2,17	0,010283	0,26248	
TC1000001289.mm.1	7,47	8,59	-2,17	0,016297	0,275809	
TC0600001174.mm.1	7,55	8,68	-2,18	0,010252	0,26248	Gm24784
TC0200001854.mm.1	10,35	11,47	-2,18	0,025982	0,292119	AA467197; Mir147
TC0300002817.mm.1	8	9,13	-2,19	0,010784	0,263089	
TC1100000889.mm.1	6,29	7,42	-2,19	0,028579	0,296378	Gm25819
TC1600001726.mm.1	8,64	9,78	-2,2	0,001239	0,171667	
TC1200000544.mm.1	4,4	5,54	-2,2	0,016224	0,275809	
TC1300002390.mm.1	6,69	7,83	-2,2	0,01674	0,277334	Gm26120
TC1700002380.mm.1	7,89	9,03	-2,2	0,016763	0,277334	
TC0700003612.mm.1	7,81	8,94	-2,2	0,01944	0,282198	
TC0900001686.mm.1	9,99	11,12	-2,2	0,03289	0,303834	
TC0100002109.mm.1	3,92	5,05	-2,2	0,038943	0,313875	Gm25586
TC0900000047.mm.1	6,22	7,36	-2,21	0,00105	0,163425	Mmp3
TC0X00001690.mm.1	4,31	5,45	-2,21	0,034676	0,306222	Gm15228
TC0200000279.mm.1	5,8	6,95	-2,22	0,002054	0,199997	
TC0600000588.mm.1	11,42	12,58	-2,23	0,01181	0,263089	Npy
TC0200000089.mm.1	6,7	7,86	-2,23	0,044158	0,321348	Gm22677
TC0X00002983.mm.1	10,26	11,43	-2,25	0,003046	0,219911	Tspan6
TC0200004599.mm.1	10,06	11,25	-2,27	0,024452	0,290793	Il1a
TC0400001092.mm.1	8,33	9,53	-2,29	0,020811	0,283915	
TC0700004112.mm.1	8,57	9,77	-2,29	0,043518	0,320126	
TC1400001730.mm.1	6,98	8,19	-2,33	0,016399	0,275809	Ldb3
TC0X00000943.mm.1	6,17	7,4	-2,34	0,019677	0,282547	
TC0700003533.mm.1	7,19	8,42	-2,34	0,037276	0,31112	
TC1600001729.mm.1	7,15	8,39	-2,35	0,001711	0,187978	
TC0700003970.mm.1	4,15	5,38	-2,35	0,012293	0,265387	Olfr684

TC1500001716.mm.1	7,53	8,77	-2,36	0,018007	0,279787	2300005B03Rik
TC0X00001739.mm.1	5,31	6,55	-2,36	0,033514	0,304293	
TC0X00002806.mm.1	5,17	6,43	-2,39	0,013939	0,270237	Gm22090
TC1800001536.mm.1	8,26	9,52	-2,39	0,025534	0,291354	
TC0700003534.mm.1	11,24	12,5	-2,4	0,04135	0,317166	
TC0700004358.mm.1	10,06	11,33	-2,41	0,028441	0,296342	Cox6a2
TC0700004515.mm.1	11,49	12,77	-2,42	0,032104	0,301614	Ifitm3
TC1600001644.mm.1	5,78	7,07	-2,45	0,008434	0,259938	
TC0900002735.mm.1	10,04	11,35	-2,48	0,006151	0,250088	Col12a1
TC0300000095.mm.1	13,07	14,37	-2,48	0,028202	0,295974	Car3
TC1800000600.mm.1	8,2	9,55	-2,54	0,011069	0,263089	
TC0200000373.mm.1	6,73	8,07	-2,54	0,033878	0,304873	Il1f8
TC1400002641.mm.1	10,65	12	-2,55	0,00163	0,184356	Ednrb
TC0100002762.mm.1	16,57	17,93	-2,56	0,006554	0,252353	Kcnj13
TC0300001670.mm.1	9,43	10,87	-2,7	0,007898	0,25437	
TC1000001125.mm.1	8,54	9,98	-2,72	0,004852	0,245082	Mir1931
TC1100000066.mm.1	2,39	3,86	-2,77	0,04444	0,321542	Mir3079
TC1000000286.mm.1	8,79	10,28	-2,82	0,037619	0,311925	Trdn
TC0200004369.mm.1	9,9	11,45	-2,93	0,013185	0,269523	Actc1
TC1000001422.mm.1	5,93	7,49	-2,94	0,015819	0,275467	
TC1000001429.mm.1	5,93	7,49	-2,94	0,015819	0,275467	
TC1000001436.mm.1	5,93	7,49	-2,94	0,015819	0,275467	
TC1000001444.mm.1	5,93	7,49	-2,94	0,015819	0,275467	
TC0200000376.mm.1	10,51	12,14	-3,11	0,030223	0,297844	Il1f9
TC0500003175.mm.1	5,45	7,16	-3,28	0,021643	0,285648	Oas2
TC0600001175.mm.1	7,49	9,24	-3,35	0,026344	0,29242	
