# Supplementary material for: Retinal pigment epithelium degeneration caused by aggregation of PRPF31 and the role of HSP70 family of proteins
Source: Mol Med. 2019 Dec 31;26:1. doi: 10.1186/s10020-019-0124-z (PMC6938640; doi:10.1186/s10020-019-0124-z)
Supplement: Supplementary file 3 — Additional file 3. Results of alternative splicing analysis (MTA) 1.0 in RPE samples of six Prpf31A216P/+ and three WT-littermates. [file 10020_2019_124_MOESM3_ESM.pdf]

Transcript Cluster ID	Gene Fold Change (	Gene Symbol	PSR/Junction ID	Splicing Index (linea	ANOVA p-value (Pr	FDR p-value (Prpf31	Splicing Event Estin	Splicing Event Scor	Comment
TC0300001915.mm.1	-1,49	C430002E04Rik	JUC0300007708.mm.1	50,3	0,001061	0,316361			
TC1400000505.mm.1	-1,08		JUC1400002405.mm.1	37,46	0,020545	0,478319			
TC1200000089.mm.1	1,07	Smc6	JUC1200000514.mm.1	33,28	0,039894	0,54093			
TC0300001064.mm.1	1,27	Ptpn22	JUC0300004475.mm.1	26,4	0,008608	0,40841			
TC0300001064.mm.1	1,27	Ptpn22	JUC0300004470.mm.1	-2,06	0,008958	0,411476			
TC0300001064.mm.1	1,27	Ptpn22	JUC0300004471.mm.1	-2,12	0,035785	0,529153			
TC0900002601.mm.1	-1,06	Lipc	JUC0900012161.mm.1	24,11	0,00121	0,322251			
TC1000001090.mm.1	1,55	Anks1b	JUC1000004455.mm.1	23,37	0,001083	0,317191			
TC1000001090.mm.1	1,55	Anks1b	PSR1000008316.mm.1	11,4	0,000278	0,28803	Cassette Exon	0,38	
TC1000001090.mm.1	1,55	Anks1b	JUC1000004460.mm.1	9,7	0,001667	0,335182			
TC1000001090.mm.1	1,55	Anks1b	PSR1000008314.mm.1	6,28	0,001612	0,332831	Cassette Exon	0,28	
TC1000001090.mm.1	1,55	Anks1b	JUC1000004454.mm.1	5,72	0,025794	0,498519			
TC1000001090.mm.1	1,55	Anks1b	PSR1000008315.mm.1	5,08	0,004055	0,361816	Cassette Exon	0,3	
TC1000001090.mm.1	1,55	Anks1b	PSR1000008308.mm.1	4,79	0,001115	0,317328	Cassette Exon	0,4	
TC1000001090.mm.1	1,55	Anks1b	JUC1000004453.mm.1	4,7	0,001544	0,330631			
TC1000001090.mm.1	1,55	Anks1b	PSR1000008318.mm.1	4,69	0,002634	0,349612	Cassette Exon	0,36	
TC1000001090.mm.1	1,55	Anks1b	JUC1000004463.mm.1	4,63	0,000853	0,311909			
TC1000001090.mm.1	1,55	Anks1b	JUC1000004471.mm.1	4	0,043295	0,549297			
TC1000001090.mm.1	1,55	Anks1b	JUC1000004451.mm.1	3,88	0,001372	0,32572			
TC1000001090.mm.1	1,55	Anks1b	PSR1000008306.mm.1	3,67	0,013945	0,446234	Cassette Exon	0,38	
TC1000001090.mm.1	1,55	Anks1b	PSR1000008311.mm.1	3,23	0,013996	0,446353	Cassette Exon	0,48	
TC1000001090.mm.1	1,55	Anks1b	PSR1000008319.mm.1	3,22	0,009654	0,417445	Cassette Exon	0,34	
TC1000001090.mm.1	1,55	Anks1b	PSR1000008395.mm.1	2,9	0,035621	0,528956			
TC1000001090.mm.1	1,55	Anks1b	JUC1000004458.mm.1	2,9	0,002757	0,350151			
TC1000001090.mm.1	1,55	Anks1b	PSR1000008367.mm.1	2,68	0,01642	0,458966	Alternative 5' Donc	0,23	
TC1000001090.mm.1	1,55	Anks1b	PSR1000008320.mm.1	2,66	0,018788	0,470789	Cassette Exon	0,3	
TC1000001090.mm.1	1,55	Anks1b	PSR1000008321.mm.1	2,63	0,04609	0,555702	Cassette Exon	0,22	
TC1000001090.mm.1	1,55	Anks1b	PSR1000008322.mm.1	2,35	0,034725	0,526521	Cassette Exon	0,27	
TC1000001090.mm.1	1,55	Anks1b	JUC1000004462.mm.1	2,34	0,039116	0,538475			
TC1000001090.mm.1	1,55	Anks1b	JUC1000004491.mm.1	-2,31	0,033144	0,521912			
TC1000001090.mm.1	1,55	Anks1b	JUC1000004496.mm.1	-3,55	0,015131	0,452674			
TC0400000432.mm.1	1,76	Unc13b	JUC0400001371.mm.1	22,06	0,00841	0,406611			
TC0400000432.mm.1	1,76	Unc13b	PSR0400002837.mm.1	8,69	0,001521	0,330287	Cassette Exon	0,51	
TC0400000432.mm.1	1,76	Unc13b	JUC0400001376.mm.1	5,28	0,002068	0,342			
TC0400000432.mm.1	1,76	Unc13b	JUC0400001356.mm.1	2,8	0,046584	0,556769			
TC0400000432.mm.1	1,76	Unc13b	JUC0400001366.mm.1	2,54	0,002505	0,349237			
TC0400000432.mm.1	1,76	Unc13b	JUC0400001328.mm.1	2,43	0,007259	0,39826			
TC0400000432.mm.1	1,76	Unc13b	PSR0400002773.mm.1	2,38	0,006576	0,39213	Cassette Exon	0,33	
TC0400000432.mm.1	1,76	Unc13b	PSR0400002772.mm.1	2,18	0,008702	0,40901	Cassette Exon	0,25	
TC0400000432.mm.1	1,76	Unc13b	JUC0400001375.mm.1	2,09	0,012529	0,43545			
TC0400000432.mm.1	1,76	Unc13b	PSR0400002796.mm.1	-2,07	0,038022	0,535471	Alternative 3' Acce	0,17	
TC0400000432.mm.1	1,76	Unc13b	JUC0400001361.mm.1	-2,07	0,008768	0,409781			
TC0400000432.mm.1	1,76	Unc13b	PSR0400002770.mm.1	-2,24	0,015339	0,453763	Cassette Exon	0,12	
TC0400000432.mm.1	1,76	Unc13b	JUC0400001348.mm.1	-2,26	0,007265	0,39826			
TC0400000432.mm.1	1,76	Unc13b	JUC0400001364.mm.1	-3,05	0,048134	0,559938			
TC0400000432.mm.1	1,76	Unc13b	PSR0400002816.mm.1	-3,77	0,000456	0,298954	Alternative 3' Acce	0,24	
TC0400000432.mm.1	1,76	Unc13b	JUC0400001377.mm.1	-3,89	0,036527	0,531352			

TC1600000301.mm.1	1,32	Vps8	JUC1600001621.mm.1	20,46	0,001059	0,316361		
TC1600000301.mm.1	1,32	Vps8	JUC1600001573.mm.1	3,36	0,010032	0,41878		
TC1600000301.mm.1	1,32	Vps8	JUC1600001608.mm.1	2,55	0,002274	0,345991		
TC1600000301.mm.1	1,32	Vps8	JUC1600001592.mm.1	2,29	0,030508	0,51337		
TC1600000301.mm.1	1,32	Vps8	PSR1600003022.mm.1	2,2	0,020194	0,477145	Cassette Exon	0,28
TC1700000602.mm.1	-2,48	Rgl2	JUC1700003070.mm.1	19,87	0,008864	0,410539		
TC1700000602.mm.1	-2,48	Rgl2	JUC1700003073.mm.1	4,15	0,004219	0,363236		
TC1700000602.mm.1	-2,48	Rgl2	PSR1700005580.mm.1	2,02	0,027632	0,50464	Cassette Exon	0,19
TC0100003183.mm.1	2,06	Crb1	JUC0100014675.mm.1	19,29	0,00023	0,28803		
TC0100003183.mm.1	2,06	Crb1	PSR0100025790.mm.1	6,46	0,001645	0,33384	Cassette Exon	0,27
TC0100003183.mm.1	2,06	Crb1	PSR0100025800.mm.1	3,19	0,002177	0,344453	Cassette Exon	0,34
TC0100003183.mm.1	2,06	Crb1	PSR0100025791.mm.1	2,74	0,009491	0,416578	Cassette Exon	0,27
TC0100003183.mm.1	2,06	Crb1	PSR0100025799.mm.1	2,73	0,027945	0,505612	Cassette Exon	0,34
TC0100003183.mm.1	2,06	Crb1	PSR0100025789.mm.1	2,66	0,030101	0,511957	Cassette Exon	0,27
TC0100003183.mm.1	2,06	Crb1	JUC0100014663.mm.1	2,59	0,021033	0,480239		
TC0100003183.mm.1	2,06	Crb1	PSR0100025798.mm.1	2,56	0,025204	0,49668	Cassette Exon	0,34
TC0100003183.mm.1	2,06	Crb1	PSR0100025794.mm.1	2,4	0,007043	0,396259	Cassette Exon	0,18
TC0100003183.mm.1	2,06	Crb1	PSR0100025797.mm.1	2,3	0,000861	0,311909	Cassette Exon	0,12
TC0100003183.mm.1	2,06	Crb1	JUC0100014664.mm.1	2,26	0,016642	0,459985		
TC0100003183.mm.1	2,06	Crb1	JUC0100014667.mm.1	2,22	0,002202	0,345178		
TC0100003183.mm.1	2,06	Crb1	PSR0100025796.mm.1	2,19	0,012828	0,437596	Cassette Exon	0,12
TC0100003183.mm.1	2,06	Crb1	PSR0100025802.mm.1	2,12	0,015966	0,456885	Cassette Exon	0,34
TC0100003183.mm.1	2,06	Crb1	PSR0100025793.mm.1	2,11	0,009432	0,416082	Cassette Exon	0,18
TC0100003183.mm.1	2,06	Crb1	PSR0100025811.mm.1	-2,1	0,022728	0,48778	Cassette Exon	0,21
TC0100003183.mm.1	2,06	Crb1	JUC0100014672.mm.1	-3,46	0,023353	0,490143		
TC0100003183.mm.1	2,06	Crb1	PSR0100025788.mm.1	-3,77	0,007173	0,397855	Alternative 5' Donc	0,44
TC0500003055.mm.1	1,99	Svop	JUC0500014857.mm.1	19,28	0,001892	0,338712		
TC0500003055.mm.1	1,99	Svop	PSR0500027189.mm.1	2,49	0,003597	0,356223	Cassette Exon	0,17
TC0500003055.mm.1	1,99	Svop	PSR0500027191.mm.1	2,25	0,020715	0,479166	Cassette Exon	0,11
TC0500003055.mm.1	1,99	Svop	JUC0500014866.mm.1	-2,13	0,046417	0,556519		
TC0500003055.mm.1	1,99	Svop	PSR0500027171.mm.1	-2,21	0,022049	0,485327	Cassette Exon	0,07
TC0500003055.mm.1	1,99	Svop	PSR0500027194.mm.1	-2,31	0,038249	0,535883	Cassette Exon	0,07
TC0500003055.mm.1	1,99	Svop	PSR0500027196.mm.1	-2,48	0,035063	0,527574	Alternative 5' Donc	0,2
TC0500003055.mm.1	1,99	Svop	JUC0500014856.mm.1	-2,54	0,007815	0,403089		
TC1600000866.mm.1	1,03	2810055G20Rik	PSR1600007110.mm.1	19,22	0,029078	0,509063	Alternative 5' Donc	0,48
TC1600000866.mm.1	1,03	2810055G20Rik	PSR1600007118.mm.1	-2,03	0,005406	0,379052	Cassette Exon	0,1
TC1600000866.mm.1	1,03	2810055G20Rik	PSR1600007130.mm.1	-2,39	0,043933	0,550715	Cassette Exon	0,08
TC1600000866.mm.1	1,03	2810055G20Rik	PSR1600007111.mm.1	-2,66	0,026084	0,499688	Alternative 3' Acce	0,24
TC1600000866.mm.1	1,03	2810055G20Rik	PSR1600007134.mm.1	-3,65	0,026167	0,50003	Alternative 5' Donc	0,42
TC1600000866.mm.1	1,03	2810055G20Rik	PSR1600007107.mm.1	-3,72	0,028427	0,507218	Cassette Exon	0,25
TC0300000238.mm.1	1,26	Sox2ot; Mir1897	JUC0300000774.mm.1	19,14	0,031763	0,517489		
TC0300000238.mm.1	1,26	Sox2ot; Mir1897	PSR0300001526.mm.1	-2,13	0,047095	0,557857	Cassette Exon	0,11
TC0400003814.mm.1	1,26	Alpl	JUC0400016346.mm.1	19,08	0,002326	0,346856		
TC0400003814.mm.1	1,26	Alpl	PSR0400031453.mm.1	6,58	0,014552	0,44963	Cassette Exon	0,42
TC0400003814.mm.1	1,26	Alpl	JUC0400016347.mm.1	2,39	0,00518	0,376851		
TC0700003152.mm.1	-2,06		PSR0700028330.mm.1	19,06	0,000443	0,297771	Cassette Exon	0,29
TC0700003152.mm.1	-2,06		PSR0700028329.mm.1	7,7	0,0006	0,304044	Cassette Exon	0,29
TC0900001445.mm.1	1,2	Map4; Mtap4	JUC0900006720.mm.1	18,59	0,001513	0,330268		

TC0900001445.mm.1	1,2	Map4; Mtap4	PSR0900012034.mm.1	7,75	0,002643	0,349612	Alternative 3' Acce	0,34
TC0900001445.mm.1	1,2	Map4; Mtap4	JUC0900006717.mm.1	6,92	0,003037	0,353792		
TC0900001445.mm.1	1,2	Map4; Mtap4	JUC0900006711.mm.1	4,86	0,006882	0,394341		
TC0900001445.mm.1	1,2	Map4; Mtap4	PSR0900012021.mm.1	3,59	0,003054	0,353892	Alternative 3' Acce	0,49
TC0900001445.mm.1	1,2	Map4; Mtap4	PSR0900012033.mm.1	2,83	0,003682	0,357266	Cassette Exon	0,51
TC0900001445.mm.1	1,2	Map4; Mtap4	JUC0900006714.mm.1	-2,55	0,044517	0,551825		
TC0900001445.mm.1	1,2	Map4; Mtap4	JUC0900006715.mm.1	-4,26	0,024828	0,495379		
TC0500003212.mm.1	1,4	Atp2a2	JUC0500015649.mm.1	18,38	0,004411	0,366761		
TC1000001266.mm.1	-1,11	Ppp1r12a	JUC1000005141.mm.1	17,86	0,000047	0,24627		
TC1000001266.mm.1	-1,11	Ppp1r12a	JUC1000005147.mm.1	2,74	0,021906	0,484824		
TC0200001472.mm.1	1,39	Lrrc4c; Palld	JUC0200005652.mm.1	17,78	0,016313	0,45857		
TC0200001472.mm.1	1,39	Lrrc4c; Palld	PSR0200011257.mm.1	4,58	0,004135	0,3623	Cassette Exon	0,5
TC0200001472.mm.1	1,39	Lrrc4c; Palld	PSR0200011259.mm.1	2,12	0,03622	0,530202	Cassette Exon	0,27
TC0900000959.mm.1	-1,26	Zfp280d	PSR0900007346.mm.1	17,04	0,029516	0,510172	Alternative 3' Acce	0,62
TC0900000959.mm.1	-1,26	Zfp280d	JUC0900004012.mm.1	6,55	0,023686	0,491379		
TC0900000959.mm.1	-1,26	Zfp280d	JUC0900004023.mm.1	-2,46	0,039839	0,540922		
TC0900000888.mm.1	1,07	Vps13c	JUC0900003709.mm.1	17,01	0,004012	0,361242		
TC0900000888.mm.1	1,07	Vps13c	JUC0900003704.mm.1	7,51	0,005228	0,377399		
TC0900000888.mm.1	1,07	Vps13c	PSR0900006845.mm.1	7,39	0,027944	0,505612	Cassette Exon	0,6
TC0900000888.mm.1	1,07	Vps13c	PSR0900006846.mm.1	4,7	0,041648	0,545276	Cassette Exon	0,6
TC0800002553.mm.1	-1,23	Tecr	JUC0800010565.mm.1	16,85	0,002548	0,349501		
TC1300000972.mm.1	1,42	Mef2c	JUC1300003317.mm.1	16,79	0,000247	0,28803		
TC1300000972.mm.1	1,42	Mef2c	JUC1300003318.mm.1	14,62	0,004973	0,373851		
TC1300000972.mm.1	1,42	Mef2c	JUC1300003319.mm.1	10,97	0,021084	0,480371		
TC1300000972.mm.1	1,42	Mef2c	PSR1300006261.mm.1	7,87	0,000963	0,313363	Cassette Exon	0,48
TC1300000972.mm.1	1,42	Mef2c	PSR1300006266.mm.1	3,72	0,00239	0,348564	Mutually Exclusive	0,44
TC1300000972.mm.1	1,42	Mef2c	PSR1300006267.mm.1	-2,33	0,004387	0,366156	Mutually Exclusive	0,44
TC1300000972.mm.1	1,42	Mef2c	PSR1300006253.mm.1	-2,55	0,030972	0,514659	Alternative 3' Acce	0,11
TC1300000972.mm.1	1,42	Mef2c	JUC1300003305.mm.1	-2,85	0,011115	0,426		
TC1300000972.mm.1	1,42	Mef2c	JUC1300003306.mm.1	-3,01	0,022938	0,488667		
TC1300000972.mm.1	1,42	Mef2c	JUC1300003309.mm.1	-3,82	0,003951	0,359922		
TC0200001023.mm.1	3,39	Gad1	JUC0200004207.mm.1	16,55	0,001181	0,319954		
TC0200001023.mm.1	3,39	Gad1	JUC0200004212.mm.1	7,43	0,000742	0,307808		
TC0200001023.mm.1	3,39	Gad1	JUC0200004199.mm.1	6,84	0,000301	0,288663		
TC0200001023.mm.1	3,39	Gad1	JUC0200004211.mm.1	6,29	0,003018	0,353447		
TC0200001023.mm.1	3,39	Gad1	JUC0200004208.mm.1	5,73	0,004031	0,361636		
TC0200001023.mm.1	3,39	Gad1	PSR0200008200.mm.1	5,04	0,000433	0,297771	Cassette Exon	0,47
TC0200001023.mm.1	3,39	Gad1	JUC0200004201.mm.1	4,57	0,000082	0,252815		
TC0200001023.mm.1	3,39	Gad1	PSR0200008201.mm.1	3,9	0,002109	0,342919	Cassette Exon	0,24
TC0200001023.mm.1	3,39	Gad1	PSR0200008205.mm.1	3,82	0,000331	0,28927	Cassette Exon	0,4
TC0200001023.mm.1	3,39	Gad1	JUC0200004213.mm.1	3,56	0,022178	0,485756		
TC0200001023.mm.1	3,39	Gad1	PSR0200008198.mm.1	3,55	0,000385	0,294337	Cassette Exon	0,35
TC0200001023.mm.1	3,39	Gad1	PSR0200008204.mm.1	3,42	0,000005	0,179072	Cassette Exon	0,47
TC0200001023.mm.1	3,39	Gad1	PSR0200008207.mm.1	3,21	0,000327	0,28927	Cassette Exon	0,19
TC0200001023.mm.1	3,39	Gad1	PSR0200008199.mm.1	3,09	0,00131	0,323877	Cassette Exon	0,46
TC0200001023.mm.1	3,39	Gad1	PSR0200008196.mm.1	2,98	0,010742	0,423772	Cassette Exon	0,11
TC0200001023.mm.1	3,39	Gad1	PSR0200008190.mm.1	2,84	0,004752	0,371039	Cassette Exon	0,34
TC0200001023.mm.1	3,39	Gad1	PSR0200008206.mm.1	2,5	0,000132	0,270905	Cassette Exon	0,15

TC0200001023.mm.1	3,39	Gad1	PSR0200008192.mm.1	2,1	0,006111	0,387239	Mutually Exclusive	0,22
TC0200001023.mm.1	3,39	Gad1	PSR0200008187.mm.1	-2,42	0,007717	0,402552	Cassette Exon	0,2
TC0200001023.mm.1	3,39	Gad1	JUC0200004210.mm.1	-2,79	0,006852	0,393893		
TC0200001023.mm.1	3,39	Gad1	PSR0200008180.mm.1	-2,96	0,014161	0,447466	Cassette Exon	0,2
TC0200001023.mm.1	3,39	Gad1	PSR0200008186.mm.1	-2,97	0,038607	0,536883	Cassette Exon	0,38
TC0200001023.mm.1	3,39	Gad1	PSR0200008182.mm.1	-3,38	0,019123	0,472425	Cassette Exon	0,15
TC0200001023.mm.1	3,39	Gad1	PSR0200008184.mm.1	-3,65	0,04467	0,55216	Cassette Exon	0,31
TC0200001023.mm.1	3,39	Gad1	PSR0200008195.mm.1	-3,75	0,007679	0,402502	Alternative 5' Donc	0,3
TC0200001023.mm.1	3,39	Gad1	PSR0200008193.mm.1	-4,29	0,032123	0,518586	Mutually Exclusive	0,22
TC0200001023.mm.1	3,39	Gad1	PSR0200008191.mm.1	-4,6	0,002286	0,34605	Alternative 5' Donc	0,52
TC0200001023.mm.1	3,39	Gad1	JUC0200004216.mm.1	-5,31	0,037478	0,533968		
TC0200001023.mm.1	3,39	Gad1	PSR0200008183.mm.1	-6,48	0,008079	0,404784	Cassette Exon	0,31
TC0200001023.mm.1	3,39	Gad1	JUC0200004217.mm.1	-7,2	0,006684	0,393187		
TC0200001023.mm.1	3,39	Gad1	PSR0200008174.mm.1	-8,49	0,006767	0,393343	Cassette Exon	0,28
TC1300001438.mm.1	1,59	Ryr2	JUC1300004723.mm.1	16,52	0,047861	0,559385		
TC1300001438.mm.1	1,59	Ryr2	JUC1300004752.mm.1	7,73	0,04629	0,556186		
TC1300001438.mm.1	1,59	Ryr2	JUC1300004696.mm.1	5,69	0,001001	0,316361		
TC1300001438.mm.1	1,59	Ryr2	PSR1300009057.mm.1	4,39	0,000466	0,298999	Cassette Exon	0,24
TC1300001438.mm.1	1,59	Ryr2	JUC1300004710.mm.1	3,32	0,003379	0,354303		
TC1300001438.mm.1	1,59	Ryr2	JUC1300004716.mm.1	3,19	0,01612	0,457756		
TC1300001438.mm.1	1,59	Ryr2	JUC1300004726.mm.1	3,14	0,006717	0,393343		
TC1300001438.mm.1	1,59	Ryr2	JUC1300004763.mm.1	2,97	0,000142	0,272178		
TC1300001438.mm.1	1,59	Ryr2	JUC1300004750.mm.1	2,69	0,005315	0,378059		
TC1300001438.mm.1	1,59	Ryr2	PSR1300009056.mm.1	2,67	0,006369	0,389488	Cassette Exon	0,2
TC1300001438.mm.1	1,59	Ryr2	PSR1300009073.mm.1	2,61	0,009231	0,414336	Cassette Exon	0,15
TC1300001438.mm.1	1,59	Ryr2	JUC1300004751.mm.1	2,54	0,028253	0,506565		
TC1300001438.mm.1	1,59	Ryr2	JUC1300004742.mm.1	2,54	0,004655	0,370181		
TC1300001438.mm.1	1,59	Ryr2	JUC1300004674.mm.1	2,45	0,025061	0,496166		
TC1300001438.mm.1	1,59	Ryr2	JUC1300004686.mm.1	2,26	0,001459	0,328511		
TC1300001438.mm.1	1,59	Ryr2	PSR1300009065.mm.1	2,23	0,044369	0,551441	Cassette Exon	0,16
TC1300001438.mm.1	1,59	Ryr2	PSR1300009092.mm.1	2,22	0,024556	0,494116	Cassette Exon	0,16
TC1300001438.mm.1	1,59	Ryr2	JUC1300004762.mm.1	2,07	0,002529	0,349501		
TC1300001438.mm.1	1,59	Ryr2	PSR1300009027.mm.1	2,03	0,001886	0,338712	Cassette Exon	0,15
TC1300001438.mm.1	1,59	Ryr2	JUC1300004711.mm.1	2,03	0,003117	0,353892		
TC1300001438.mm.1	1,59	Ryr2	JUC1300004777.mm.1	-2,19	0,02706	0,502979		
TC1300001438.mm.1	1,59	Ryr2	PSR1300009079.mm.1	-2,44	0,001929	0,340171	Cassette Exon	0,17
TC1300001438.mm.1	1,59	Ryr2	JUC1300004673.mm.1	-2,44	0,047272	0,558224		
TC1300001438.mm.1	1,59	Ryr2	PSR1300009114.mm.1	-2,81	0,013483	0,443132	Cassette Exon	0,15
TC1300001438.mm.1	1,59	Ryr2	PSR1300009080.mm.1	-2,88	0,002253	0,34578	Cassette Exon	0,17
TC1300001438.mm.1	1,59	Ryr2	JUC1300004735.mm.1	-3,02	0,006989	0,395722		
TC1300001438.mm.1	1,59	Ryr2	JUC1300004684.mm.1	-3,14	0,013768	0,444954		
TC1300001438.mm.1	1,59	Ryr2	PSR1300009045.mm.1	-3,27	0,018363	0,468484	Cassette Exon	0,26
TC1300001438.mm.1	1,59	Ryr2	JUC1300004715.mm.1	-3,28	0,011942	0,431753		
TC1300001438.mm.1	1,59	Ryr2	JUC1300004773.mm.1	-4,26	0,003618	0,356223		
TC1300001438.mm.1	1,59	Ryr2	JUC1300004772.mm.1	-5,59	0,035739	0,529051		
TC1400000281.mm.1	2,63	Erc2	JUC1400001370.mm.1	16,51	0,002482	0,349135		
TC1400000281.mm.1	2,63	Erc2	JUC1400001371.mm.1	3,35	0,007258	0,39826		
TC1400000281.mm.1	2,63	Erc2	JUC1400001364.mm.1	2,01	0,020755	0,479262		

TC1400000281.mm.1	2,63	Erc2	PSR1400002308.mm.1	-2,18	0,031472	0,516922	Cassette Exon	0,15
TC1400000281.mm.1	2,63	Erc2	JUC1400001375.mm.1	-2,36	0,043762	0,55037		
TC1400000281.mm.1	2,63	Erc2	JUC1400001382.mm.1	-2,69	0,003541	0,3562		
TC1400000281.mm.1	2,63	Erc2	PSR1400002311.mm.1	-2,7	0,036179	0,529944	Alternative 5' Donc	0,31
TC1400000281.mm.1	2,63	Erc2	JUC1400001386.mm.1	-2,92	0,000805	0,311765		
TC1400000281.mm.1	2,63	Erc2	PSR1400002306.mm.1	-4,07	0,0377	0,534513	Cassette Exon	0,34
TC0600001271.mm.1	-1,22	Atg7	PSR0600010028.mm.1	16,15	0,00291	0,352289	Alternative 5' Donc	0,48
TC1100001634.mm.1	1,37	Atp6v0a1	JUC1100007879.mm.1	16,03	0,002528	0,349501		
TC1100001634.mm.1	1,37	Atp6v0a1	JUC1100007880.mm.1	5,65	0,011529	0,428455		
TC1100001634.mm.1	1,37	Atp6v0a1	JUC1100007882.mm.1	-2,04	0,01901	0,471738		
TC1100001634.mm.1	1,37	Atp6v0a1	PSR1100015173.mm.1	-2,38	0,028077	0,506011	Alternative 3' Acce	0,23
TC0X00003429.mm.1	-1,79	L1cam	JUC0X00007735.mm.1	15,84	0,002127	0,342919		
TC0X00003429.mm.1	-1,79	L1cam	JUC0X00007734.mm.1	9,59	0,002214	0,34564		
TC0X00003429.mm.1	-1,79	L1cam	JUC0X00007733.mm.1	3,52	0,009283	0,414799		
TC1400001643.mm.1	1,86	Cacna2d3	PSR1400012365.mm.1	15,83	0,000902	0,313363	Cassette Exon	0,29
TC1400001643.mm.1	1,86	Cacna2d3	PSR1400012359.mm.1	5,78	0,004883	0,372195	Cassette Exon	0,44
TC1400001643.mm.1	1,86	Cacna2d3	PSR1400012367.mm.1	5,68	0,009543	0,416659	Cassette Exon	0,3
TC1400001643.mm.1	1,86	Cacna2d3	PSR1400012366.mm.1	5,13	0,001384	0,3259	Cassette Exon	0,3
TC1400001643.mm.1	1,86	Cacna2d3	PSR1400012364.mm.1	4,46	0,009748	0,417839	Cassette Exon	0,41
TC1400001643.mm.1	1,86	Cacna2d3	JUC1400006675.mm.1	4,18	0,001513	0,330268		
TC1400001643.mm.1	1,86	Cacna2d3	PSR1400012363.mm.1	3,67	0,004966	0,373851	Cassette Exon	0,39
TC1400001643.mm.1	1,86	Cacna2d3	PSR1400012362.mm.1	3,61	0,003158	0,353892	Cassette Exon	0,31
TC1400001643.mm.1	1,86	Cacna2d3	JUC1400006673.mm.1	2,85	0,029623	0,510558		
TC1400001643.mm.1	1,86	Cacna2d3	PSR1400012357.mm.1	2,71	0,007135	0,397369	Alternative 3' Acce	0,19
TC1400001643.mm.1	1,86	Cacna2d3	JUC1400006672.mm.1	2,69	0,013221	0,44107		
TC1400001643.mm.1	1,86	Cacna2d3	JUC1400006709.mm.1	2,05	0,021414	0,482126		
TC1400001643.mm.1	1,86	Cacna2d3	PSR1400012387.mm.1	-2,02	0,01159	0,429241	Cassette Exon	0,1
TC1400001643.mm.1	1,86	Cacna2d3	PSR1400012400.mm.1	-2,24	0,016931	0,4609	Cassette Exon	0,12
TC1400001643.mm.1	1,86	Cacna2d3	PSR1400012374.mm.1	-2,3	0,0002	0,28803	Cassette Exon	0,11
TC1400001643.mm.1	1,86	Cacna2d3	JUC1400006706.mm.1	-2,35	0,020423	0,477971		
TC1400001643.mm.1	1,86	Cacna2d3	PSR1400012373.mm.1	-2,4	0,035985	0,529491	Cassette Exon	0,13
TC1400001643.mm.1	1,86	Cacna2d3	PSR1400012398.mm.1	-2,56	0,022901	0,488524	Alternative 3' Acce	0,11
TC1400001643.mm.1	1,86	Cacna2d3	PSR1400012372.mm.1	-2,58	0,001235	0,322251	Cassette Exon	0,16
TC1400001643.mm.1	1,86	Cacna2d3	PSR1400012371.mm.1	-2,94	0,006582	0,39213	Cassette Exon	0,21
TC1400001643.mm.1	1,86	Cacna2d3	PSR1400012375.mm.1	-3,03	0,007182	0,397855	Cassette Exon	0,18
TC0500003182.mm.1	2,33	Rph3a	JUC0500015416.mm.1	15,69	0,009248	0,414515		
TC0500003182.mm.1	2,33	Rph3a	JUC0500015411.mm.1	8,1	0,041405	0,544627		
TC0500003182.mm.1	2,33	Rph3a	PSR0500028284.mm.1	6,9	0,028458	0,507299	Cassette Exon	0,28
TC0500003182.mm.1	2,33	Rph3a	JUC0500015410.mm.1	5,19	0,006225	0,387962		
TC0500003182.mm.1	2,33	Rph3a	PSR0500028286.mm.1	3,56	0,004302	0,364072	Cassette Exon	0,24
TC0500003182.mm.1	2,33	Rph3a	JUC0500015414.mm.1	3,03	0,00855	0,408183		
TC0500003182.mm.1	2,33	Rph3a	PSR0500028304.mm.1	2,62	0,00314	0,353892	Cassette Exon	0,23
TC0500003182.mm.1	2,33	Rph3a	PSR0500028307.mm.1	-2,24	0,035793	0,529153	Cassette Exon	0,1
TC0500003182.mm.1	2,33	Rph3a	JUC0500015407.mm.1	-2,47	0,016093	0,45756		
TC0500003182.mm.1	2,33	Rph3a	JUC0500015399.mm.1	-2,64	0,010425	0,421539		
TC0500003182.mm.1	2,33	Rph3a	JUC0500015413.mm.1	-3,83	0,049797	0,563698		
TC0500003182.mm.1	2,33	Rph3a	PSR0500028298.mm.1	-4,27	0,023257	0,490032	Cassette Exon	0,28
TC0500003182.mm.1	2,33	Rph3a	PSR0500028296.mm.1	-4,96	0,017061	0,461973	Cassette Exon	0,34

TC0200002130.mm.1	1,9	Pcsk2	JUC0200008473.mm.1	15,36	0,002563	0,349501		
TC0200002130.mm.1	1,9	Pcsk2	JUC0200008478.mm.1	6,04	0,006337	0,389463		
TC0200002130.mm.1	1,9	Pcsk2	PSR0200016814.mm.1	2,82	0,001127	0,317328	Cassette Exon	0,21
TC0200002130.mm.1	1,9	Pcsk2	PSR0200016815.mm.1	2,69	0,006019	0,386586	Cassette Exon	0,23
TC0200002130.mm.1	1,9	Pcsk2	PSR0200016823.mm.1	-2,04	0,002316	0,346468	Cassette Exon	0,1
TC0200002130.mm.1	1,9	Pcsk2	PSR0200016806.mm.1	-2,57	0,012724	0,436578	Alternative 3' Acce	0,29
TC0200002130.mm.1	1,9	Pcsk2	PSR0200016805.mm.1	-3,05	0,002279	0,345991	Alternative 3' Acce	0,29
TC0200002130.mm.1	1,9	Pcsk2	PSR0200016810.mm.1	-3,28	0,007466	0,400381		
TC0400004035.mm.1	1,93	Kif1b	JUC0400017464.mm.1	15,33	0,000877	0,311909		
TC0400004035.mm.1	1,93	Kif1b	JUC0400017463.mm.1	13	0,002019	0,34123		
TC0400004035.mm.1	1,93	Kif1b	JUC0400017473.mm.1	5,4	0,007219	0,39826		
TC0400004035.mm.1	1,93	Kif1b	JUC0400017474.mm.1	4,45	0,047738	0,559019		
TC0400004035.mm.1	1,93	Kif1b	JUC0400017472.mm.1	3,01	0,040352	0,541949		
TC0400004035.mm.1	1,93	Kif1b	PSR0400033487.mm.1	2,53	0,002017	0,34123	Alternative 5' Donc	0,17
TC0400004035.mm.1	1,93	Kif1b	PSR0400033488.mm.1	-2,27	0,023598	0,490995	Cassette Exon	0,24
TC0400004035.mm.1	1,93	Kif1b	PSR0400033492.mm.1	-2,27	0,032284	0,519116	Alternative 3' Acce	0,18
TC0400004035.mm.1	1,93	Kif1b	PSR0400033483.mm.1	-2,29	0,026471	0,501252	Alternative 5' Donc	0,18
TC0400004035.mm.1	1,93	Kif1b	PSR0400033505.mm.1	-2,36	0,017265	0,462888	Alternative 5' Donc	0,21
TC0400004035.mm.1	1,93	Kif1b	PSR0400033476.mm.1	-2,65	0,001921	0,34017	Cassette Exon	0,31
TC0400004035.mm.1	1,93	Kif1b	PSR0400033474.mm.1	-2,7	0,00723	0,39826	Cassette Exon	0,31
TC0400004035.mm.1	1,93	Kif1b	JUC0400017476.mm.1	-3,04	0,029836	0,511132		
TC0400004035.mm.1	1,93	Kif1b	PSR0400033475.mm.1	-3,36	0,005307	0,378059	Cassette Exon	0,31
TC0400004035.mm.1	1,93	Kif1b	PSR0400033463.mm.1	-3,83	0,005566	0,380931	Alternative 5' Donc	0,42
TC0400004035.mm.1	1,93	Kif1b	JUC0400017477.mm.1	-3,92	0,002723	0,349612		
TC0400004035.mm.1	1,93	Kif1b	JUC0400017460.mm.1	-5,84	0,018289	0,468049		
TC0900000948.mm.1	-1,62		JUC0900003964.mm.1	14,76	0,021631	0,483559		
TC0X00001873.mm.1	1,04	Cask	JUC0X00006080.mm.1	14,6	0,001903	0,338712		
TC0X00001873.mm.1	1,04	Cask	JUC0X00006078.mm.1	5,11	0,010184	0,419711		
TC0X00001873.mm.1	1,04	Cask	JUC0X00006075.mm.1	2,71	0,028593	0,507685		
TC0700003359.mm.1	-1,2		JUC0700015203.mm.1	14,26	0,012261	0,433888		
TC1800000179.mm.1	-1,14	Dtna; Gm19389	JUC1800000746.mm.1	14,23	0,001427	0,327447		
TC1800000179.mm.1	-1,14	Dtna; Gm19389	JUC1800000747.mm.1	6,69	0,004779	0,371044		
TC1800000179.mm.1	-1,14	Dtna; Gm19389	JUC1800000741.mm.1	2,61	0,037812	0,534771		
TC1800000179.mm.1	-1,14	Dtna; Gm19389	JUC1800000737.mm.1	2,24	0,02684	0,502454		
TC1800000179.mm.1	-1,14	Dtna; Gm19389	PSR1800001261.mm.1	2,07	0,003136	0,353892	Cassette Exon	0,02
TC1900000777.mm.1	1,21	Vti1a	JUC1900003734.mm.1	14,04	0,010552	0,422341		
TC1900000777.mm.1	1,21	Vti1a	PSR1900006860.mm.1	-2,48	0,000548	0,304044	Cassette Exon	0,14
TC1100001135.mm.1	2,75	Sez6	PSR1100010404.mm.1	13,93	0,000685	0,304511	Cassette Exon	0,4
TC1100001135.mm.1	2,75	Sez6	JUC1100005470.mm.1	4,15	0,000287	0,28803		
TC1100001135.mm.1	2,75	Sez6	JUC1100005456.mm.1	3,31	0,022348	0,486348		
TC1100001135.mm.1	2,75	Sez6	PSR1100010406.mm.1	2,76	0,000231	0,28803	Cassette Exon	0,29
TC1100001135.mm.1	2,75	Sez6	PSR1100010399.mm.1	2,58	0,008053	0,404416	Cassette Exon	0,17
TC1100001135.mm.1	2,75	Sez6	JUC1100005469.mm.1	2,47	0,038179	0,535805		
TC1100001135.mm.1	2,75	Sez6	JUC1100005468.mm.1	2,19	0,017089	0,462202		
TC1100001135.mm.1	2,75	Sez6	PSR1100010394.mm.1	-2,02	0,010819	0,424068	Cassette Exon	0,13
TC1100001135.mm.1	2,75	Sez6	PSR1100010396.mm.1	-2,07	0,017247	0,462729	Cassette Exon	0,11
TC1100001135.mm.1	2,75	Sez6	PSR1100010386.mm.1	-2,25	0,040081	0,541392	Alternative 5' Donc	0,21
TC1100001135.mm.1	2,75	Sez6	PSR1100010411.mm.1	-2,31	0,007258	0,39826	Alternative 5' Donc	0,23

TC1100001135.mm.1	2,75	Sez6	PSR1100010390.mm.1	-2,74	0,000543	0,304044	Alternative 3' Acce	0,21
TC1100001135.mm.1	2,75	Sez6	PSR1100010378.mm.1	-3,01	0,001473	0,329052	Cassette Exon	0,19
TC1100001135.mm.1	2,75	Sez6	PSR1100010395.mm.1	-3,83	0,000554	0,304044	Intron Retention	0,45
TC1100001135.mm.1	2,75	Sez6	PSR1100010392.mm.1	-3,84	0,002576	0,349501	Alternative 3' Acce	0,54
TC1100001135.mm.1	2,75	Sez6	JUC1100005460.mm.1	-3,97	0,000419	0,297771		
TC1100001135.mm.1	2,75	Sez6	PSR1100010373.mm.1	-4,65	0,021046	0,480262	Alternative 3' Acce	0,27
TC1100001135.mm.1	2,75	Sez6	JUC1100005465.mm.1	-4,71	0,000216	0,28803		
TC0600000998.mm.1	1,45	Aak1	JUC0600003976.mm.1	13,84	0,001471	0,329052		
TC0600000998.mm.1	1,45	Aak1	JUC0600003977.mm.1	7,46	0,009627	0,417315		
TC0600000998.mm.1	1,45	Aak1	JUC0600003974.mm.1	6,04	0,003344	0,354243		
TC0600000998.mm.1	1,45	Aak1	JUC0600003975.mm.1	4,54	0,004857	0,37168		
TC0600000998.mm.1	1,45	Aak1	PSR0600007804.mm.1	4,5	0,014766	0,450801	Alternative 3' Acce	0,2
TC0600000998.mm.1	1,45	Aak1	PSR0600007802.mm.1	3,72	0,000306	0,288663	Cassette Exon	0,49
TC0600000998.mm.1	1,45	Aak1	JUC0600003978.mm.1	3,37	0,017209	0,462496		
TC0600000998.mm.1	1,45	Aak1	PSR0600007798.mm.1	2,55	0,018031	0,467048	Cassette Exon	0,41
TC0600000998.mm.1	1,45	Aak1	PSR0600007799.mm.1	2,54	0,048527	0,560991	Cassette Exon	0,41
TC0600000998.mm.1	1,45	Aak1	PSR0600007800.mm.1	2,42	0,015269	0,452983	Cassette Exon	0,4
TC0600000998.mm.1	1,45	Aak1	JUC0600003973.mm.1	2,34	0,041805	0,545586		
TC0600000998.mm.1	1,45	Aak1	PSR0600007793.mm.1	-2,66	0,010239	0,420146	Cassette Exon	0,18
TC0600000998.mm.1	1,45	Aak1	JUC0600003969.mm.1	-2,72	0,035471	0,528724		
TC0300001080.mm.1	1,07	Ppm1j	JUC0300004526.mm.1	13,61	0,036348	0,530573		
TC1100000549.mm.1	3,34	Grm6	JUC1100002234.mm.1	13,54	0,000181	0,283791		
TC1100000549.mm.1	3,34	Grm6	PSR1100004286.mm.1	6,48	0,017729	0,465216	Cassette Exon	0,4
TC1100000549.mm.1	3,34	Grm6	JUC1100002237.mm.1	4,22	0,001583	0,331726		
TC1100000549.mm.1	3,34	Grm6	PSR1100004282.mm.1	3,16	0,020012	0,47638	Cassette Exon	0,23
TC1100000549.mm.1	3,34	Grm6	PSR1100004279.mm.1	3,15	0,001375	0,32572	Cassette Exon	0,2
TC1100000549.mm.1	3,34	Grm6	PSR1100004273.mm.1	-2,84	0,004454	0,367239	Cassette Exon	0,3
TC1100000549.mm.1	3,34	Grm6	PSR1100004275.mm.1	-2,88	0,004777	0,371044	Cassette Exon	0,3
TC1100000549.mm.1	3,34	Grm6	JUC1100002240.mm.1	-3,37	0,024289	0,49362		
TC1100000549.mm.1	3,34	Grm6	PSR1100004281.mm.1	-3,61	0,000078	0,25272	Alternative 3' Acce	0,55
TC1100000549.mm.1	3,34	Grm6	PSR1100004283.mm.1	-3,63	0,007007	0,395737	Alternative 3' Acce	0,56
TC1100000549.mm.1	3,34	Grm6	JUC1100002230.mm.1	-5,5	0,002789	0,351436		
TC0200003674.mm.1	2,28	Scn1a	JUC0200015929.mm.1	13,4	0,000154	0,272178		
TC0200003674.mm.1	2,28	Scn1a	JUC0200015954.mm.1	5	0,01419	0,447608		
TC0200003674.mm.1	2,28	Scn1a	PSR0200031329.mm.1	4,48	0,000489	0,298999	Cassette Exon	0,38
TC0200003674.mm.1	2,28	Scn1a	JUC0200015926.mm.1	3,25	0,024988	0,496025		
TC0200003674.mm.1	2,28	Scn1a	JUC0200015946.mm.1	3,23	0,01595	0,456846		
TC0200003674.mm.1	2,28	Scn1a	JUC0200015925.mm.1	2,74	0,010531	0,42227		
TC0200003674.mm.1	2,28	Scn1a	PSR0200031324.mm.1	2,68	0,00226	0,34578	Alternative 3' Acce	0,27
TC0200003674.mm.1	2,28	Scn1a	JUC0200015928.mm.1	2,64	0,018744	0,470733		
TC0200003674.mm.1	2,28	Scn1a	JUC0200015948.mm.1	2,55	0,021719	0,484104		
TC0200003674.mm.1	2,28	Scn1a	PSR0200031332.mm.1	2,31	0,034904	0,52699	Cassette Exon	0,06
TC0200003674.mm.1	2,28	Scn1a	PSR0200031337.mm.1	2,16	0,044064	0,55088	Alternative 5' Donc	0,05
TC0200003674.mm.1	2,28	Scn1a	PSR0200031328.mm.1	2,08	0,006345	0,389463	Cassette Exon	0,23
TC0200003674.mm.1	2,28	Scn1a	PSR0200031356.mm.1	-2,08	0,009845	0,417839	Cassette Exon	0,03
TC0200003674.mm.1	2,28	Scn1a	PSR0200031340.mm.1	-2,18	0,01619	0,45809	Cassette Exon	0,17
TC0200003674.mm.1	2,28	Scn1a	PSR0200031335.mm.1	-2,18	0,009511	0,416602	Cassette Exon	0,13
TC0200003674.mm.1	2,28	Scn1a	PSR0200031342.mm.1	-2,35	0,008144	0,405416	Alternative 3' Acce	0,23

TC0200003674.mm.1	2,28	Scn1a	PSR0200031344.mm.1	-2,4	0,007118	0,397044	Cassette Exon	0,15
TC0200003674.mm.1	2,28	Scn1a	JUC0200015947.mm.1	-2,45	0,038514	0,536557		
TC0200003674.mm.1	2,28	Scn1a	PSR0200031345.mm.1	-2,51	0,044296	0,551351	Cassette Exon	0,25
TC0200003674.mm.1	2,28	Scn1a	PSR0200031353.mm.1	-2,75	0,000134	0,270905	Cassette Exon	0,13
TC0200003674.mm.1	2,28	Scn1a	JUC0200015930.mm.1	-2,93	0,00237	0,34829		
TC0200003674.mm.1	2,28	Scn1a	PSR0200031360.mm.1	-3,03	0,010031	0,41878	Alternative 5' Donc	0,3
TC0200003674.mm.1	2,28	Scn1a	JUC0200015941.mm.1	-3,03	0,011384	0,427549		
TC0700003387.mm.1	1,58	A330076H08Rik; Mir344g	PSR0700028940.mm.1	12,77	0,042819	0,548097		
TC0700003387.mm.1	1,58	A330076H08Rik; Mir344g	PSR0700028941.mm.1	6,76	0,008126	0,405267	Alternative 3' Acce	0,37
TC0700003387.mm.1	1,58	A330076H08Rik; Mir344g	PSR0700028939.mm.1	4,62	0,001822	0,336896		
TC0700003387.mm.1	1,58	A330076H08Rik; Mir344g	PSR0700028946.mm.1	4,53	0,004387	0,366156	Alternative 3' Acce	0,25
TC0700003387.mm.1	1,58	A330076H08Rik; Mir344g	PSR0700028944.mm.1	4,13	0,004235	0,363295	Alternative 5' Donc	0,33
TC0700003387.mm.1	1,58	A330076H08Rik; Mir344g	PSR0700028938.mm.1	3,68	0,004717	0,370888		
TC0700003387.mm.1	1,58	A330076H08Rik; Mir344g	PSR0700028945.mm.1	3,6	0,005745	0,382993	Alternative 5' Donc	0,33
TC0700003387.mm.1	1,58	A330076H08Rik; Mir344g	PSR0700028947.mm.1	2,45	0,018899	0,47127	Alternative 3' Acce	0,09
TC0100000706.mm.1	-1,18	Col4a3	JUC0100003469.mm.1	12,73	0,008089	0,405012		
TC0100000706.mm.1	-1,18	Col4a3	PSR0100006007.mm.1	2,1	0,023758	0,491689	Cassette Exon	0,09
TC1900000119.mm.1	2,02	Nrxn2	JUC1900000847.mm.1	12,71	0,000151	0,272178		
TC1900000119.mm.1	2,02	Nrxn2	JUC1900000853.mm.1	6,31	0,009411	0,415853		
TC1900000119.mm.1	2,02	Nrxn2	PSR1900001657.mm.1	4,6	0,013027	0,439354	Alternative 5' Donc	0,45
TC1900000119.mm.1	2,02	Nrxn2	JUC1900000851.mm.1	4,37	0,004892	0,372195		
TC1900000119.mm.1	2,02	Nrxn2	PSR1900001634.mm.1	3,8	0,000268	0,28803	Alternative 5' Donc	0,45
TC1900000119.mm.1	2,02	Nrxn2	JUC1900000848.mm.1	2,73	0,000105	0,264747		
TC1900000119.mm.1	2,02	Nrxn2	JUC1900000850.mm.1	2,17	0,016082	0,457504		
TC1900000119.mm.1	2,02	Nrxn2	PSR1900001638.mm.1	-2,02	0,016402	0,458894	Cassette Exon	0,16
TC1900000119.mm.1	2,02	Nrxn2	PSR1900001640.mm.1	-2,04	0,012085	0,43255	Cassette Exon	0,09
TC1900000119.mm.1	2,02	Nrxn2	PSR1900001652.mm.1	-2,04	0,047855	0,559373	Alternative 3' Acce	0,09
TC1900000119.mm.1	2,02	Nrxn2	JUC1900000856.mm.1	-2,06	0,022184	0,485756		
TC1900000119.mm.1	2,02	Nrxn2	PSR1900001627.mm.1	-2,08	0,049853	0,563773		
TC1900000119.mm.1	2,02	Nrxn2	JUC1900000838.mm.1	-2,12	0,000694	0,305213		
TC1900000119.mm.1	2,02	Nrxn2	JUC1900000835.mm.1	-2,56	0,043904	0,550652		
TC1900000119.mm.1	2,02	Nrxn2	PSR1900001660.mm.1	-2,67	0,005049	0,374728	Cassette Exon	0,14
TC1900000119.mm.1	2,02	Nrxn2	PSR1900001662.mm.1	-2,97	0,014074	0,446841	Alternative 3' Acce	0,28
TC1900000119.mm.1	2,02	Nrxn2	JUC1900000846.mm.1	-2,99	0,000629	0,304044		
TC1900000119.mm.1	2,02	Nrxn2	PSR1900001650.mm.1	-3,02	0,028829	0,508414	Alternative 3' Acce	0,16
TC1900000119.mm.1	2,02	Nrxn2	PSR1900001617.mm.1	-3,04	0,021226	0,481319	Alternative 3' Acce	0,28
TC1900000119.mm.1	2,02	Nrxn2	PSR1900001615.mm.1	-3,37	0,024247	0,493309	Cassette Exon	0,22
TC1900000119.mm.1	2,02	Nrxn2	JUC1900000864.mm.1	-3,61	0,016713	0,460199		
TC1900000119.mm.1	2,02	Nrxn2	PSR1900001624.mm.1	-3,92	0,011542	0,428761	Cassette Exon	0,09
TC1900000119.mm.1	2,02	Nrxn2	JUC1900000859.mm.1	-4,41	0,006581	0,39213		
TC0800000472.mm.1	2,1	Micu3	JUC0800001732.mm.1	12,63	0,009838	0,417839		
TC0800000472.mm.1	2,1	Micu3	PSR0800003224.mm.1	-2,17	0,039616	0,540026	Cassette Exon	0,09
TC1000000517.mm.1	1,35	Ranbp2	JUC1000001888.mm.1	12,57	0,004191	0,362939		
TC0300000934.mm.1	-1,25	Hist2h3b; Hist1h3e; Hist1	PSR0300007468.mm.1	12,48	0,003408	0,355204	Alternative 3' Acce	0,48
TC0200003142.mm.1	2,24	Grin1	JUC0200013102.mm.1	12,47	0,012337	0,434446		
TC0200003142.mm.1	2,24	Grin1	JUC0200013101.mm.1	5,37	0,000696	0,305429		
TC0200003142.mm.1	2,24	Grin1	JUC0200013088.mm.1	3,2	0,010841	0,424161		
TC0200003142.mm.1	2,24	Grin1	PSR0200025921.mm.1	3,16	0,042237	0,547034	Cassette Exon	0,47

TC0200003142.mm.1	2,24	Grin1	JUC0200013104.mm.1	2,62	0,033723	0,523549		
TC0200003142.mm.1	2,24	Grin1	PSR0200025891.mm.1	-2,04	0,002651	0,349612	Cassette Exon	0,08
TC0200003142.mm.1	2,24	Grin1	PSR0200025920.mm.1	-2,2	0,038845	0,537797	Alternative 3' Acce	0,33
TC0200003142.mm.1	2,24	Grin1	PSR0200025916.mm.1	-2,31	0,003456	0,355628	Alternative 3' Acce	0,14
TC0200003142.mm.1	2,24	Grin1	JUC0200013090.mm.1	-2,35	0,025131	0,496397		
TC0200003142.mm.1	2,24	Grin1	PSR0200025884.mm.1	-2,46	0,003213	0,354243	Alternative 3' Acce	0,21
TC0200003142.mm.1	2,24	Grin1	JUC0200013100.mm.1	-2,56	0,008827	0,410293		
TC0200003142.mm.1	2,24	Grin1	PSR0200025889.mm.1	-2,58	0,000827	0,311886	Alternative 5' Donc	0,31
TC0200003142.mm.1	2,24	Grin1	JUC0200013096.mm.1	-2,6	0,045703	0,554833		
TC0200003142.mm.1	2,24	Grin1	PSR0200025886.mm.1	-2,74	0,003932	0,359818	Alternative 5' Donc	0,27
TC0200003142.mm.1	2,24	Grin1	PSR0200025912.mm.1	-3,24	0,008764	0,409781	Intron Retention	0,45
TC0200003142.mm.1	2,24	Grin1	JUC0200013108.mm.1	-3,26	0,022107	0,485497		
TC0200003142.mm.1	2,24	Grin1	PSR0200025904.mm.1	-3,33	0,007419	0,399901	Alternative 3' Acce	0,25
TC0200003142.mm.1	2,24	Grin1	JUC0200013110.mm.1	-3,42	0,022414	0,486711		
TC0200003142.mm.1	2,24	Grin1	JUC0200013094.mm.1	-3,59	0,000495	0,299933		
TC0200003142.mm.1	2,24	Grin1	JUC0200013107.mm.1	-4,54	0,000005	0,179072		
TC0200003142.mm.1	2,24	Grin1	JUC0200013105.mm.1	-5,53	0,033016	0,521478		
TC1600001807.mm.1	2,75	Arl6	JUC1600007542.mm.1	12,41	0,032314	0,519155		
TC1600001807.mm.1	2,75	Arl6	PSR1600014542.mm.1	-2,82	0,024372	0,49362	Alternative 5' Donc	0,28
TC1600001807.mm.1	2,75	Arl6	PSR1600014543.mm.1	-3,42	0,005478	0,379939	Alternative 5' Donc	0,28
TC1600001807.mm.1	2,75	Arl6	JUC1600007535.mm.1	-3,57	0,005775	0,383104		
TC1600001807.mm.1	2,75	Arl6	JUC1600007546.mm.1	-6,13	0,009574	0,417047		
TC0200000803.mm.1	-1,07	Lypd6b	JUC0200003379.mm.1	12,32	0,027765	0,505135		
TC0100002054.mm.1	2,34	Rims1	PSR0100016719.mm.1	12,24	0,000937	0,313363	Cassette Exon	0,47
TC0100002054.mm.1	2,34	Rims1	PSR0100016717.mm.1	4,59	0,00631	0,389263	Cassette Exon	0,33
TC0100002054.mm.1	2,34	Rims1	JUC0100009482.mm.1	3,93	0,006608	0,392252		
TC0100002054.mm.1	2,34	Rims1	PSR0100016718.mm.1	3,01	0,023407	0,490259	Cassette Exon	0,33
TC0100002054.mm.1	2,34	Rims1	PSR0100016720.mm.1	2,82	0,032809	0,520894	Cassette Exon	0,22
TC0100002054.mm.1	2,34	Rims1	JUC0100009521.mm.1	2,81	0,025115	0,496358		
TC0100002054.mm.1	2,34	Rims1	PSR0100016727.mm.1	2,61	0,013689	0,444581	Cassette Exon	0,13
TC0100002054.mm.1	2,34	Rims1	PSR0100016716.mm.1	2,4	0,004564	0,369273	Cassette Exon	0,33
TC0100002054.mm.1	2,34	Rims1	JUC0100009505.mm.1	2,26	0,027766	0,505135		
TC0100002054.mm.1	2,34	Rims1	JUC0100009483.mm.1	2,24	0,049157	0,562368		
TC0100002054.mm.1	2,34	Rims1	JUC0100009516.mm.1	-2,12	0,045552	0,554401		
TC0100002054.mm.1	2,34	Rims1	PSR0100016767.mm.1	-2,37	0,03282	0,520896	Cassette Exon	0,17
TC0100002054.mm.1	2,34	Rims1	JUC0100009494.mm.1	-2,4	0,007759	0,402774		
TC0100002054.mm.1	2,34	Rims1	PSR0100016766.mm.1	-2,48	0,015369	0,453849	Cassette Exon	0,17
TC0100002054.mm.1	2,34	Rims1	JUC0100009510.mm.1	-2,57	0,027776	0,50524		
TC0100002054.mm.1	2,34	Rims1	JUC0100009522.mm.1	-3,07	0,007525	0,40125		
TC1600001219.mm.1	-1,03		JUC1600005073.mm.1	12,23	0,022822	0,488298		
TC1100000859.mm.1	-1,03	Gas7	PSR1100007123.mm.1	12,17	0,008782	0,409822	Cassette Exon	0,42
TC1100000859.mm.1	-1,03	Gas7	PSR1100007124.mm.1	7,27	0,002837	0,352207	Cassette Exon	0,42
TC1100000859.mm.1	-1,03	Gas7	JUC1100003763.mm.1	4,69	0,007376	0,39943		
TC1100000859.mm.1	-1,03	Gas7	PSR1100007130.mm.1	3,78	0,018845	0,470912	Alternative 3' Acce	0,42
TC1100000859.mm.1	-1,03	Gas7	PSR1100007126.mm.1	3,39	0,01203	0,432286	Alternative 3' Acce	0,42
TC1100000859.mm.1	-1,03	Gas7	PSR1100007125.mm.1	3,05	0,015258	0,452847	Alternative 3' Acce	0,42
TC1100000859.mm.1	-1,03	Gas7	PSR1100007131.mm.1	2,46	0,005635	0,381532	Alternative 3' Acce	0,42
TC1100000859.mm.1	-1,03	Gas7	PSR1100007128.mm.1	2,35	0,045769	0,554863	Alternative 3' Acce	0,42

TC1100000859.mm.1	-1,03	Gas7	PSR1100007097.mm.1	-2,22	0,039533	0,539764	Cassette Exon	0,11
TC1100000859.mm.1	-1,03	Gas7	PSR1100007095.mm.1	-2,44	0,025179	0,496598	Alternative 3' Acce	0,16
TC1100000859.mm.1	-1,03	Gas7	JUC1100003771.mm.1	-2,88	0,014166	0,447466		
TC0100002028.mm.1	-1,35	Pkhd1	JUC0100009379.mm.1	12,07	0,004798	0,371408		
TC0500000723.mm.1	1,27	Exoc1	JUC0500003544.mm.1	12,05	0,01783	0,466005		
TC1100001397.mm.1	1,62	Mmd	JUC1100006853.mm.1	12,04	0,002224	0,345773		
TC1100001397.mm.1	1,62	Mmd	PSR1100013062.mm.1	5,54	0,011238	0,426718	Cassette Exon	0,4
TC1100001397.mm.1	1,62	Mmd	JUC1100006861.mm.1	4,52	0,02583	0,498598		
TC1100001397.mm.1	1,62	Mmd	PSR1100013061.mm.1	3,52	0,009995	0,418456	Cassette Exon	0,37
TC1100001397.mm.1	1,62	Mmd	PSR1100013079.mm.1	3,05	0,019842	0,475536	Cassette Exon	0,4
TC1100001397.mm.1	1,62	Mmd	PSR1100013065.mm.1	2,85	0,024559	0,494116	Alternative 3' Acce	0,28
TC1100001397.mm.1	1,62	Mmd	JUC1100006858.mm.1	2,69	0,008202	0,405909		
TC1100001397.mm.1	1,62	Mmd	PSR1100013066.mm.1	2,68	0,016003	0,457051	Alternative 3' Acce	0,28
TC1100001397.mm.1	1,62	Mmd	JUC1100006862.mm.1	2,68	0,012473	0,435351		
TC0700001101.mm.1	-1,83	Agbl1	JUC0700004969.mm.1	11,98	0,002677	0,349612		
TC0700001101.mm.1	-1,83	Agbl1	JUC0700004982.mm.1	2,96	0,005439	0,379743		
TC1800000945.mm.1	-1,25	Kif5b	JUC1800003862.mm.1	11,89	0,024906	0,495688		
TC1800000945.mm.1	-1,25	Kif5b	JUC1800003867.mm.1	2,29	0,016818	0,460623		
TC1900001711.mm.1	-1,19	Ablim1	JUC1900008452.mm.1	11,86	0,008271	0,405976		
TC1900001711.mm.1	-1,19	Ablim1	JUC1900008447.mm.1	8,34	0,028878	0,50858		
TC1900001711.mm.1	-1,19	Ablim1	JUC1900008432.mm.1	5,22	0,028538	0,507479		
TC1900001711.mm.1	-1,19	Ablim1	PSR1900015196.mm.1	3,06	0,020636	0,478837	Cassette Exon	0,31
TC1900001711.mm.1	-1,19	Ablim1	JUC1900008434.mm.1	2,89	0,047031	0,557783		
TC1900001711.mm.1	-1,19	Ablim1	PSR1900015197.mm.1	2,39	0,035749	0,529051	Cassette Exon	0,15
TC0700000976.mm.1	1,15	Trpm1	JUC0700004511.mm.1	11,84	0,000536	0,304044		
TC0700000976.mm.1	1,15	Trpm1	PSR0700008844.mm.1	8,25	0,001968	0,340561	Cassette Exon	0,41
TC0700000976.mm.1	1,15	Trpm1	PSR0700008915.mm.1	3,96	0,006278	0,388805	Cassette Exon	0,34
TC0700000976.mm.1	1,15	Trpm1	PSR0700008914.mm.1	3,9	0,004952	0,373423	Cassette Exon	0,34
TC0700000976.mm.1	1,15	Trpm1	PSR0700008911.mm.1	3,85	0,010062	0,418902	Cassette Exon	0,4
TC0700000976.mm.1	1,15	Trpm1	JUC0700004528.mm.1	3,46	0,046552	0,556761		
TC0700000976.mm.1	1,15	Trpm1	PSR0700008913.mm.1	3,44	0,00236	0,34829	Cassette Exon	0,34
TC0700000976.mm.1	1,15	Trpm1	JUC0700004517.mm.1	3,22	0,010129	0,419203		
TC0700000976.mm.1	1,15	Trpm1	JUC0700004539.mm.1	3,14	0,043673	0,550236		
TC0700000976.mm.1	1,15	Trpm1	JUC0700004516.mm.1	3,07	0,021659	0,483789		
TC0700000976.mm.1	1,15	Trpm1	JUC0700004531.mm.1	3,03	0,026464	0,501252		
TC0700000976.mm.1	1,15	Trpm1	JUC0700004529.mm.1	2,99	0,020807	0,479505		
TC0700000976.mm.1	1,15	Trpm1	PSR0700008917.mm.1	2,96	0,034962	0,52724	Cassette Exon	0,34
TC0700000976.mm.1	1,15	Trpm1	PSR0700008905.mm.1	2,42	0,025981	0,499207	Cassette Exon	0,22
TC0700000976.mm.1	1,15	Trpm1	PSR0700008910.mm.1	2,39	0,019501	0,474493	Cassette Exon	0,17
TC0700000976.mm.1	1,15	Trpm1	JUC0700004535.mm.1	2,25	0,012025	0,432286		
TC0700000976.mm.1	1,15	Trpm1	PSR0700008901.mm.1	2,16	0,01224	0,433659	Cassette Exon	0,12
TC0700000976.mm.1	1,15	Trpm1	PSR0700008897.mm.1	2,1	0,043779	0,550398	Alternative 5' Donc	0,11
TC0700000976.mm.1	1,15	Trpm1	JUC0700004536.mm.1	2,04	0,035879	0,52938		
TC0700000976.mm.1	1,15	Trpm1	JUC0700004515.mm.1	2,03	0,025842	0,498615		
TC0700000728.mm.1	-1,09	Gm2108	JUC0700002949.mm.1	11,79	0,004558	0,369246		
TC0200003118.mm.1	1,66	Cacna1b	JUC0200012954.mm.1	11,76	0,000303	0,288663		
TC0200003118.mm.1	1,66	Cacna1b	JUC0200012915.mm.1	8,85	0,000939	0,313363		
TC0200003118.mm.1	1,66	Cacna1b	JUC0200012921.mm.1	7,97	0,000482	0,298999		

TC0200003118.mm.1	1,66	Cacna1b	PSR0200025568.mm.1	6,98	0,000022	0,22008	Cassette Exon	0,42
TC0200003118.mm.1	1,66	Cacna1b	PSR0200025569.mm.1	5,22	0,001294	0,322675	Cassette Exon	0,55
TC0200003118.mm.1	1,66	Cacna1b	JUC0200012922.mm.1	4,8	0,005302	0,378059		
TC0200003118.mm.1	1,66	Cacna1b	PSR0200025589.mm.1	3,74	0,003551	0,356223	Cassette Exon	0,26
TC0200003118.mm.1	1,66	Cacna1b	JUC0200012917.mm.1	3,22	0,018905	0,47127		
TC0200003118.mm.1	1,66	Cacna1b	PSR0200025549.mm.1	3,19	0,005999	0,386404	Cassette Exon	0,16
TC0200003118.mm.1	1,66	Cacna1b	PSR0200025563.mm.1	2,93	0,000186	0,284485	Cassette Exon	0,28
TC0200003118.mm.1	1,66	Cacna1b	PSR0200025551.mm.1	2,29	0,015215	0,45279	Cassette Exon	0,17
TC0200003118.mm.1	1,66	Cacna1b	JUC0200012910.mm.1	2,22	0,01514	0,452699		
TC0200003118.mm.1	1,66	Cacna1b	PSR0200025591.mm.1	2,06	0,007003	0,395737	Cassette Exon	0,11
TC0200003118.mm.1	1,66	Cacna1b	PSR0200025562.mm.1	2,05	0,0011	0,317328	Cassette Exon	0,28
TC0200003118.mm.1	1,66	Cacna1b	PSR0200025543.mm.1	-2,02	0,016985	0,461441	Cassette Exon	0,1
TC0200003118.mm.1	1,66	Cacna1b	PSR0200025576.mm.1	-2,14	0,013946	0,446234	Cassette Exon	0,11
TC0200003118.mm.1	1,66	Cacna1b	PSR0200025542.mm.1	-2,21	0,012404	0,435021	Cassette Exon	0,1
TC0200003118.mm.1	1,66	Cacna1b	PSR0200025548.mm.1	-2,23	0,002409	0,348653	Alternative 3' Acce	0,25
TC0200003118.mm.1	1,66	Cacna1b	PSR0200025541.mm.1	-2,55	0,000741	0,307808	Cassette Exon	0,1
TC0200003118.mm.1	1,66	Cacna1b	JUC0200012962.mm.1	-2,59	0,033682	0,523549		
TC0200003118.mm.1	1,66	Cacna1b	JUC0200012959.mm.1	-2,68	0,021115	0,480564		
TC0200003118.mm.1	1,66	Cacna1b	PSR0200025538.mm.1	-2,9	0,011751	0,430017	Alternative 3' Acce	0,25
TC0200003118.mm.1	1,66	Cacna1b	JUC0200012967.mm.1	-3,11	0,000628	0,304044		
TC1800000484.mm.1	-1,34	Ap3s1	JUC1800001973.mm.1	11,76	0,031735	0,517489		
TC0400001058.mm.1	8,24	Sgip1	JUC0400003686.mm.1	11,63	0,000782	0,309887		
TC0400001058.mm.1	8,24	Sgip1	JUC0400003702.mm.1	3,39	0,00752	0,401141		
TC0400001058.mm.1	8,24	Sgip1	JUC0400003697.mm.1	2,24	0,002867	0,352207		
TC0400001058.mm.1	8,24	Sgip1	JUC0400003682.mm.1	2,13	0,02685	0,502454		
TC0400001058.mm.1	8,24	Sgip1	PSR0400007227.mm.1	2,03	0,02784	0,505537	Cassette Exon	0,2
TC0400001058.mm.1	8,24	Sgip1	PSR0400007210.mm.1	-2,11	0,020271	0,477245	Cassette Exon	0,14
TC0400001058.mm.1	8,24	Sgip1	PSR0400007196.mm.1	-2,12	0,003787	0,357586	Alternative 5' Donc	0,23
TC0400001058.mm.1	8,24	Sgip1	PSR0400007189.mm.1	-2,15	0,005403	0,378967	Alternative 5' Donc	0,05
TC0400001058.mm.1	8,24	Sgip1	PSR0400007208.mm.1	-2,44	0,039122	0,538475	Cassette Exon	0,16
TC0400001058.mm.1	8,24	Sgip1	PSR0400007225.mm.1	-2,5	0,005008	0,373851	Alternative 5' Donc	0,22
TC0400001058.mm.1	8,24	Sgip1	PSR0400007207.mm.1	-2,5	0,000312	0,288663	Cassette Exon	0,16
TC0400001058.mm.1	8,24	Sgip1	PSR0400007211.mm.1	-3,08	0,017459	0,463886	Alternative 3' Acce	0,29
TC0400001058.mm.1	8,24	Sgip1	PSR0400007216.mm.1	-3,22	0,009563	0,416927	Cassette Exon	0,35
TC0400001058.mm.1	8,24	Sgip1	PSR0400007213.mm.1	-3,25	0,0023	0,346364	Cassette Exon	0,34
TC0400001058.mm.1	8,24	Sgip1	PSR0400007217.mm.1	-3,29	0,013209	0,440879	Cassette Exon	0,01
TC0400001058.mm.1	8,24	Sgip1	PSR0400007199.mm.1	-3,56	0,010844	0,424161	Alternative 5' Donc	0,41
TC0400001058.mm.1	8,24	Sgip1	PSR0400007192.mm.1	-3,58	0,002037	0,341377	Cassette Exon	0,36
TC0400001058.mm.1	8,24	Sgip1	PSR0400007200.mm.1	-3,76	0,008516	0,407551	Cassette Exon	0,46
TC0400001058.mm.1	8,24	Sgip1	JUC0400003679.mm.1	-4,35	0,000734	0,307808		
TC0400001058.mm.1	8,24	Sgip1	PSR0400007177.mm.1	-4,62	0,008444	0,406611	Alternative 3' Acce	0,34
TC0400001058.mm.1	8,24	Sgip1	PSR0400007190.mm.1	-5,42	0,005186	0,376851	Cassette Exon	0,34
TC0400001058.mm.1	8,24	Sgip1	JUC0400003683.mm.1	-5,7	0,008217	0,405958		
TC0400001058.mm.1	8,24	Sgip1	PSR0400007175.mm.1	-5,74	0,007149	0,39769	Cassette Exon	0,41
TC0400001058.mm.1	8,24	Sgip1	PSR0400007176.mm.1	-5,9	0,018231	0,46765	Cassette Exon	0,21
TC0400001058.mm.1	8,24	Sgip1	JUC0400003696.mm.1	-5,92	0,000661	0,304044		
TC0400001058.mm.1	8,24	Sgip1	JUC0400003676.mm.1	-6,55	0,008437	0,406611		
TC0400001058.mm.1	8,24	Sgip1	JUC0400003711.mm.1	-6,58	0,013565	0,443703		

TC0400001058.mm.1	8,24	Sgip1	JUC0400003708.mm.1	-6,62	0,011325	0,427133		
TC0400001058.mm.1	8,24	Sgip1	JUC0400003715.mm.1	-6,8	0,011208	0,426683		
TC0400001058.mm.1	8,24	Sgip1	JUC0400003685.mm.1	-7,24	0,000081	0,25272		
TC0400001058.mm.1	8,24	Sgip1	JUC0400003707.mm.1	-10,53	0,005274	0,378059		
TC0400001058.mm.1	8,24	Sgip1	JUC0400003709.mm.1	-11,01	0,000162	0,272566		
TC0500002320.mm.1	2,67	Prom1	JUC0500011649.mm.1	11,53	0,036151	0,529835		
TC0500002320.mm.1	2,67	Prom1	JUC0500011633.mm.1	4,84	0,006042	0,386586		
TC0500002320.mm.1	2,67	Prom1	JUC0500011650.mm.1	4,58	0,028698	0,508019		
TC0500002320.mm.1	2,67	Prom1	JUC0500011634.mm.1	3,74	0,004432	0,366894		
TC0100003146.mm.1	1,06	Camsap2	JUC0100014563.mm.1	11,39	0,001129	0,317328		
TC0100003146.mm.1	1,06	Camsap2	JUC0100014546.mm.1	2,48	0,025737	0,498265		
TC1300001420.mm.1	-1,33		JUC1300004624.mm.1	11,35	0,001967	0,340561		
TC0800001666.mm.1	1,86	Fam155a	JUC0800007417.mm.1	11,33	0,00012	0,270905		
TC0800001666.mm.1	1,86	Fam155a	PSR0800013636.mm.1	2,79	0,028545	0,507497	Alternative 3' Acce	0,29
TC0800001666.mm.1	1,86	Fam155a	JUC0800007416.mm.1	2,57	0,038364	0,536296		
TC0800001666.mm.1	1,86	Fam155a	PSR0800013635.mm.1	2,24	0,026203	0,50003	Alternative 3' Acce	0,24
TC0800001666.mm.1	1,86	Fam155a	JUC0800007422.mm.1	-2,98	0,027751	0,50504		
TC0800001666.mm.1	1,86	Fam155a	PSR0800013638.mm.1	-3,51	0,006544	0,392035	Cassette Exon	0,35
TC0800001666.mm.1	1,86	Fam155a	PSR0800013640.mm.1	-4,38	0,011253	0,426718	Cassette Exon	0,48
TC0300000761.mm.1	1,25	Dcst2	JUC0300003032.mm.1	11,3	0,030278	0,512602		
TC0600000780.mm.1	1,07	E230016M11Rik	JUC0600002922.mm.1	11,22	0,002386	0,348564		
TC0600000780.mm.1	1,07	E230016M11Rik	PSR0600005843.mm.1	4,53	0,002706	0,349612	Cassette Exon	0,41
TC0600000780.mm.1	1,07	E230016M11Rik	PSR0600005842.mm.1	3,88	0,001481	0,329081	Cassette Exon	0,29
TC0600000780.mm.1	1,07	E230016M11Rik	JUC0600002930.mm.1	3,28	0,011433	0,427663		
TC0600000780.mm.1	1,07	E230016M11Rik	PSR0600005846.mm.1	3,05	0,008472	0,406734	Cassette Exon	0,32
TC0600000780.mm.1	1,07	E230016M11Rik	PSR0600005839.mm.1	3	0,032953	0,52122	Cassette Exon	0,19
TC0600000780.mm.1	1,07	E230016M11Rik	JUC0600002926.mm.1	2,5	0,021055	0,480262		
TC0100000660.mm.1	1,59	Asic4	JUC0100003270.mm.1	11,2	0,002395	0,348564		
TC0100000660.mm.1	1,59	Asic4	JUC0100003264.mm.1	4,97	0,006514	0,391382		
TC0100000660.mm.1	1,59	Asic4	JUC0100003262.mm.1	3,88	0,04475	0,552338		
TC0100000660.mm.1	1,59	Asic4	PSR0100005669.mm.1	-2,09	0,007457	0,400326	Cassette Exon	0,1
TC0900000786.mm.1	-1,07	Anp32a	JUC0900003152.mm.1	11,17	0,005389	0,378916		
TC0900000786.mm.1	-1,07	Anp32a	JUC0900003153.mm.1	9,22	0,015251	0,452847		
TC0900000786.mm.1	-1,07	Anp32a	PSR0900005891.mm.1	4,03	0,006525	0,391502	Cassette Exon	0,39
TC0900000786.mm.1	-1,07	Anp32a	PSR0900005890.mm.1	3,39	0,01184	0,430614	Cassette Exon	0,39
TC0300001624.mm.1	-1,06	Tpd52	JUC0300006855.mm.1	11,14	0,035101	0,527591		
TC0300001624.mm.1	-1,06	Tpd52	JUC0300006856.mm.1	6,89	0,018375	0,468484		
TC0300001624.mm.1	-1,06	Tpd52	JUC0300006848.mm.1	6,35	0,043095	0,548536		
TC0300001624.mm.1	-1,06	Tpd52	PSR0300012939.mm.1	4,72	0,002243	0,34578	Cassette Exon	0,56
TC0300001624.mm.1	-1,06	Tpd52	PSR0300012940.mm.1	4,51	0,019664	0,474887	Cassette Exon	0,48
TC0300001624.mm.1	-1,06	Tpd52	JUC0300006847.mm.1	4,1	0,036997	0,532502		
TC0300001624.mm.1	-1,06	Tpd52	JUC0300006849.mm.1	3,33	0,011548	0,428813		
TC0300001624.mm.1	-1,06	Tpd52	PSR0300012949.mm.1	2,92	0,004809	0,371408	Cassette Exon	0,25
TC0300001624.mm.1	-1,06	Tpd52	JUC0300006854.mm.1	2,48	0,012083	0,43255		
TC1000002056.mm.1	1,1	Cep57l1	JUC1000007965.mm.1	11,08	0,010608	0,422786		
TC1000002056.mm.1	1,1	Cep57l1	PSR1000014659.mm.1	-2,59	0,016066	0,457431	Alternative 3' Acce	0,25
TC1600000571.mm.1	1,6	Lsamp	JUC1600002825.mm.1	11,04	0,001991	0,341091		
TC1600000571.mm.1	1,6	Lsamp	PSR1600005377.mm.1	4,67	0,018689	0,47037	Cassette Exon	0,46

TC1600000571.mm.1	1,6	Lsamp	PSR1600005372.mm.1	2,7	0,005621	0,381231	Alternative 3' Acce	0,15
TC1600000571.mm.1	1,6	Lsamp	PSR1600005405.mm.1	2,61	0,044315	0,551368		
TC1600000571.mm.1	1,6	Lsamp	JUC1600002828.mm.1	2,52	0,027427	0,503961		
TC1600000571.mm.1	1,6	Lsamp	PSR1600005399.mm.1	2,46	0,043557	0,550033	Cassette Exon	0,15
TC1600000571.mm.1	1,6	Lsamp	JUC1600002830.mm.1	2,14	0,025402	0,497403		
TC1600000571.mm.1	1,6	Lsamp	PSR1600005403.mm.1	2,13	0,025729	0,498173		
TC1600000571.mm.1	1,6	Lsamp	PSR1600005396.mm.1	-2,37	0,020195	0,477145	Cassette Exon	0,16
TC1600000571.mm.1	1,6	Lsamp	PSR1600005370.mm.1	-2,4	0,012161	0,432939	Alternative 3' Acce	0,22
TC1600000571.mm.1	1,6	Lsamp	PSR1600005398.mm.1	-2,41	0,011503	0,42822	Cassette Exon	0,15
TC0500000499.mm.1	1,57	Slit2	JUC0500002653.mm.1	11,01	0,000597	0,304044		
TC0500000499.mm.1	1,57	Slit2	JUC0500002647.mm.1	7,38	0,002302	0,346364		
TC0500000499.mm.1	1,57	Slit2	JUC0500002642.mm.1	5,18	0,017071	0,462106		
TC0500000499.mm.1	1,57	Slit2	PSR0500004747.mm.1	2,95	0,013758	0,444784	Cassette Exon	0,37
TC0500000499.mm.1	1,57	Slit2	PSR0500004754.mm.1	-2,13	0,032234	0,51899	Alternative 5' Donc	0,16
TC0500000499.mm.1	1,57	Slit2	JUC0500002648.mm.1	-6,76	0,035934	0,529399		
TC0400001129.mm.1	-1,22	Ndc1	JUC0400004062.mm.1	11	0,009989	0,418456		
TC0400001129.mm.1	-1,22	Ndc1	PSR0400007849.mm.1	2,34	0,028817	0,508347	Alternative 3' Acce	0,19
TC1600000415.mm.1	1,31	Dlg1	JUC1600002019.mm.1	10,93	0,017715	0,465209		
TC1600000415.mm.1	1,31	Dlg1	JUC1600002009.mm.1	2,19	0,035375	0,528536		
TC1600000415.mm.1	1,31	Dlg1	JUC1600002022.mm.1	-2,02	0,030306	0,512738		
TC1600000415.mm.1	1,31	Dlg1	JUC1600001997.mm.1	-2,23	0,032668	0,520432		
TC1600002162.mm.1	-1,05	A630089N07Rik	JUC1600008703.mm.1	10,72	0,001702	0,335511		
TC1100001721.mm.1	-1,18	Wnt3	JUC1100008400.mm.1	10,63	0,022502	0,487114		
TC1200000840.mm.1	1,08	Ttll5	JUC1200003438.mm.1	10,57	0,01857	0,469618		
TC1200000840.mm.1	1,08	Ttll5	PSR1200006131.mm.1	2,63	0,001354	0,325427	Cassette Exon	0,19
TC1200000840.mm.1	1,08	Ttll5	JUC1200003456.mm.1	2,16	0,011296	0,427123		
TC1200000840.mm.1	1,08	Ttll5	JUC1200003489.mm.1	-2,47	0,006933	0,395269		
TC1900000054.mm.1	-1,1	Rin1	JUC1900000382.mm.1	10,55	0,02371	0,491428		
TC0700003023.mm.1	1,08	Kcnj14	PSR0700027463.mm.1	10,5	0,001388	0,325997	Cassette Exon	0,28
TC0700003023.mm.1	1,08	Kcnj14	PSR0700027464.mm.1	2,83	0,028451	0,507299	Cassette Exon	0,17
TC0700002217.mm.1	1,04	Peg3	JUC0700010670.mm.1	10,21	0,0015	0,329714		
TC0700002217.mm.1	1,04	Peg3	JUC0700010671.mm.1	6,9	0,008537	0,407916		
TC0700002217.mm.1	1,04	Peg3	PSR0700020133.mm.1	2,62	0,006303	0,389124	Cassette Exon	0,47
TC0700002217.mm.1	1,04	Peg3	PSR0700020141.mm.1	2,13	0,002177	0,344453	Alternative 5' Donc	0,12
TC1400000780.mm.1	3	Cpne6	JUC1400003284.mm.1	10,18	0,015792	0,455742		
TC1400000780.mm.1	3	Cpne6	PSR1400006149.mm.1	4,06	0,000878	0,311909	Cassette Exon	0,43
TC1400000780.mm.1	3	Cpne6	JUC1400003285.mm.1	4,05	0,003882	0,359196		
TC1400000780.mm.1	3	Cpne6	JUC1400003277.mm.1	3,1	0,002006	0,341091		
TC1400000780.mm.1	3	Cpne6	PSR1400006160.mm.1	3,02	0,000297	0,288663	Cassette Exon	0,14
TC1400000780.mm.1	3	Cpne6	JUC1400003279.mm.1	2,91	0,023359	0,490143		
TC1400000780.mm.1	3	Cpne6	JUC1400003278.mm.1	2,59	0,009681	0,417605		
TC1400000780.mm.1	3	Cpne6	PSR1400006148.mm.1	2,18	0,029627	0,510558	Cassette Exon	0,28
TC1400000780.mm.1	3	Cpne6	JUC1400003286.mm.1	-2,16	0,015605	0,455078		
TC1400000780.mm.1	3	Cpne6	PSR1400006146.mm.1	-2,17	0,001649	0,333988	Cassette Exon	0,24
TC1400000780.mm.1	3	Cpne6	PSR1400006167.mm.1	-2,71	0,002973	0,352501	Alternative 5' Donc	0,35
TC1400000780.mm.1	3	Cpne6	JUC1400003276.mm.1	-2,95	0,005735	0,382993		
TC1400000780.mm.1	3	Cpne6	PSR1400006145.mm.1	-2,96	0,003988	0,360652	Cassette Exon	0,27
TC1400000780.mm.1	3	Cpne6	JUC1400003289.mm.1	-2,97	0,044145	0,551024		

TC1400000780.mm.1	3	Cpne6	PSR1400006139.mm.1	-3,3	0,019938	0,476043	Alternative 3' Acce	0,08
TC1400000780.mm.1	3	Cpne6	JUC1400003291.mm.1	-3,54	0,006271	0,388719		
TC1400000780.mm.1	3	Cpne6	PSR1400006141.mm.1	-3,57	0,024837	0,495465	Alternative 3' Acce	0,08
TC1400000780.mm.1	3	Cpne6	PSR1400006137.mm.1	-3,65	0,008566	0,40821	Alternative 3' Acce	0,08
TC1400000780.mm.1	3	Cpne6	PSR1400006166.mm.1	-3,66	0,009504	0,416602	Intron Retention	0,45
TC1400000780.mm.1	3	Cpne6	JUC1400003275.mm.1	-3,98	0,020519	0,478264		
TC1400000780.mm.1	3	Cpne6	PSR1400006140.mm.1	-4,31	0,013516	0,443428	Alternative 3' Acce	0,08
TC1400000780.mm.1	3	Cpne6	JUC1400003290.mm.1	-4,52	0,001455	0,328414		
TC1400000780.mm.1	3	Cpne6	PSR1400006136.mm.1	-4,86	0,006706	0,393343	Alternative 3' Acce	0,08
TC1400000780.mm.1	3	Cpne6	PSR1400006150.mm.1	-4,88	0,00354	0,3562	Intron Retention	0,66
TC1400000780.mm.1	3	Cpne6	PSR1400006147.mm.1	-5,16	0,008069	0,404565	Alternative 3' Acce	0,62
TC1400000780.mm.1	3	Cpne6	PSR1400006138.mm.1	-5,18	0,005716	0,382767	Cassette Exon	0,39
TC1500000726.mm.1	1,24	L3mbtl2	JUC1500003187.mm.1	10,17	0,006393	0,389654		
TC1500000726.mm.1	1,24	L3mbtl2	JUC1500003181.mm.1	2,68	0,023149	0,489795		
TC1500000726.mm.1	1,24	L3mbtl2	JUC1500003185.mm.1	2,03	0,036306	0,530479		
TC1300002037.mm.1	-1,83	Cltb	JUC1300006572.mm.1	10,01	0,008299	0,406122		
TC1300002037.mm.1	-1,83	Cltb	JUC1300006573.mm.1	6,68	0,006258	0,388622		
TC1300002037.mm.1	-1,83	Cltb	PSR1300012732.mm.1	5,46	0,009132	0,413352	Cassette Exon	0,57
TC0500001927.mm.1	1,8	Adam22; Gm19440	JUC0500009623.mm.1	10	0,001879	0,338712		
TC0500001927.mm.1	1,8	Adam22; Gm19440	JUC0500009642.mm.1	4,35	0,004219	0,363236		
TC0500001927.mm.1	1,8	Adam22; Gm19440	JUC0500009644.mm.1	2,26	0,032571	0,520133		
TC0500001927.mm.1	1,8	Adam22; Gm19440	JUC0500009627.mm.1	2,19	0,029701	0,510949		
TC0500001927.mm.1	1,8	Adam22; Gm19440	PSR0500017749.mm.1	-2,49	0,008384	0,4065	Cassette Exon	0,21
TC0500001927.mm.1	1,8	Adam22; Gm19440	JUC0500009634.mm.1	-2,91	0,022009	0,485226		
TC0500001927.mm.1	1,8	Adam22; Gm19440	JUC0500009637.mm.1	-3,04	0,039201	0,538692		
TC0500001927.mm.1	1,8	Adam22; Gm19440	PSR0500017752.mm.1	-3,27	0,004175	0,362543	Alternative 3' Acce	0,34
TC0500001927.mm.1	1,8	Adam22; Gm19440	JUC0500009629.mm.1	-3,41	0,014237	0,447947		
TC0500001927.mm.1	1,8	Adam22; Gm19440	JUC0500009633.mm.1	-3,86	0,016651	0,459985		
TC0500001927.mm.1	1,8	Adam22; Gm19440	JUC0500009638.mm.1	-5,48	0,008252	0,405976		
TC0800002437.mm.1	1,36	Unc13a	PSR0800018618.mm.1	9,98	0,004845	0,37168	Cassette Exon	0,55
TC0800002437.mm.1	1,36	Unc13a	JUC0800010130.mm.1	9,55	0,002043	0,341377		
TC0800002437.mm.1	1,36	Unc13a	JUC0800010126.mm.1	8,71	0,00255	0,349501		
TC0800002437.mm.1	1,36	Unc13a	PSR0800018617.mm.1	8,17	0,00215	0,343679	Cassette Exon	0,55
TC0800002437.mm.1	1,36	Unc13a	JUC0800010119.mm.1	7,51	0,002513	0,349347		
TC0800002437.mm.1	1,36	Unc13a	JUC0800010129.mm.1	6,89	0,003337	0,354243		
TC0800002437.mm.1	1,36	Unc13a	JUC0800010127.mm.1	6,68	0,003931	0,359818		
TC0800002437.mm.1	1,36	Unc13a	JUC0800010122.mm.1	6,62	0,001678	0,335182		
TC0800002437.mm.1	1,36	Unc13a	JUC0800010131.mm.1	6,19	0,046662	0,556812		
TC0800002437.mm.1	1,36	Unc13a	JUC0800010147.mm.1	5,86	0,002429	0,349		
TC0800002437.mm.1	1,36	Unc13a	JUC0800010128.mm.1	5,78	0,012657	0,43631		
TC0800002437.mm.1	1,36	Unc13a	PSR0800018619.mm.1	5,62	0,001706	0,335996	Cassette Exon	0,55
TC0800002437.mm.1	1,36	Unc13a	PSR0800018612.mm.1	5,53	0,002407	0,348653	Cassette Exon	0,41
TC0800002437.mm.1	1,36	Unc13a	JUC0800010148.mm.1	4,84	0,009303	0,414883		
TC0800002437.mm.1	1,36	Unc13a	JUC0800010135.mm.1	4,56	0,005211	0,377078		
TC0800002437.mm.1	1,36	Unc13a	PSR0800018620.mm.1	4,35	0,00122	0,322251	Cassette Exon	0,55
TC0800002437.mm.1	1,36	Unc13a	JUC0800010114.mm.1	4,35	0,003167	0,353901		
TC0800002437.mm.1	1,36	Unc13a	JUC0800010111.mm.1	4,32	0,00565	0,381811		
TC0800002437.mm.1	1,36	Unc13a	PSR0800018603.mm.1	4,13	0,006378	0,389488	Cassette Exon	0,26

TC0800002437.mm.1	1,36	Unc13a	JUC0800010134.mm.1	3,74	0,039628	0,540111		
TC0800002437.mm.1	1,36	Unc13a	JUC0800010136.mm.1	3,65	0,016977	0,461419		
TC0800002437.mm.1	1,36	Unc13a	PSR0800018631.mm.1	3,58	0,002984	0,352501	Cassette Exon	0,23
TC0800002437.mm.1	1,36	Unc13a	PSR0800018595.mm.1	3,34	0,001807	0,336311	Cassette Exon	0,18
TC0800002437.mm.1	1,36	Unc13a	JUC0800010110.mm.1	3,32	0,021009	0,480143		
TC0800002437.mm.1	1,36	Unc13a	JUC0800010121.mm.1	3,32	0,043288	0,549297		
TC0800002437.mm.1	1,36	Unc13a	PSR0800018621.mm.1	3,29	0,013779	0,444974	Cassette Exon	0,48
TC0800002437.mm.1	1,36	Unc13a	PSR0800018630.mm.1	3,12	0,027813	0,505442	Cassette Exon	0,3
TC0800002437.mm.1	1,36	Unc13a	JUC0800010146.mm.1	2,99	0,006765	0,393343		
TC0800002437.mm.1	1,36	Unc13a	PSR0800018614.mm.1	2,96	0,004101	0,36228	Cassette Exon	0,19
TC0800002437.mm.1	1,36	Unc13a	PSR0800018626.mm.1	2,88	0,018518	0,469117	Cassette Exon	0,43
TC0800002437.mm.1	1,36	Unc13a	PSR0800018611.mm.1	2,67	0,022003	0,485226	Cassette Exon	0,39
TC0800002437.mm.1	1,36	Unc13a	PSR0800018606.mm.1	2,6	0,004886	0,372195	Alternative 5' Donc	0,21
TC0800002437.mm.1	1,36	Unc13a	JUC0800010125.mm.1	2,4	0,018996	0,471532		
TC0800002437.mm.1	1,36	Unc13a	PSR0800018622.mm.1	2,36	0,020965	0,479982	Cassette Exon	0,29
TC0800002437.mm.1	1,36	Unc13a	PSR0800018623.mm.1	2,33	0,018262	0,467921	Cassette Exon	0,15
TC0800002437.mm.1	1,36	Unc13a	JUC0800010109.mm.1	2,22	0,041762	0,545499		
TC0800002437.mm.1	1,36	Unc13a	PSR0800018593.mm.1	2,21	0,006037	0,386586	Cassette Exon	0,35
TC0800002437.mm.1	1,36	Unc13a	PSR0800018650.mm.1	-2,91	0,047275	0,558232	Alternative 5' Donc	0,31
TC0800002437.mm.1	1,36	Unc13a	JUC0800010151.mm.1	-3,12	0,003196	0,354243		
TC0500000011.mm.1	-1,63	Fam133b	JUC0500000023.mm.1	9,93	0,000802	0,311765		
TC0500000011.mm.1	-1,63	Fam133b	JUC0500000027.mm.1	2,11	0,011449	0,427811		
TC0100002324.mm.1	-1	Mfsd6	PSR0100018710.mm.1	9,78	0,001255	0,322251	Cassette Exon	0,44
TC0100002324.mm.1	-1	Mfsd6	JUC0100010583.mm.1	5,46	0,004544	0,368715		
TC0100001011.mm.1	-1,27	Tnfrsf11a	JUC0100004836.mm.1	9,78	0,018203	0,467586		
TC1500001460.mm.1	-1,25	Eif3e	JUC1500006266.mm.1	9,75	0,005481	0,379939		
TC1200000432.mm.1	2,52	Akap6	JUC1200001703.mm.1	9,73	0,03119	0,515739		
TC1200000432.mm.1	2,52	Akap6	JUC1200001711.mm.1	3,01	0,049383	0,562882		
TC1200000432.mm.1	2,52	Akap6	JUC1200001714.mm.1	-2,31	0,010117	0,41912		
TC1200000432.mm.1	2,52	Akap6	PSR1200003040.mm.1	-2,64	0,013247	0,441416	Cassette Exon	0,46
TC1200000432.mm.1	2,52	Akap6	JUC1200001716.mm.1	-3,45	0,025714	0,498173		
TC0600000493.mm.1	1,37	Cntnap2	JUC0600001924.mm.1	9,61	0,002811	0,352207		
TC0600000493.mm.1	1,37	Cntnap2	JUC0600001925.mm.1	5,28	0,012962	0,43903		
TC0600000493.mm.1	1,37	Cntnap2	PSR0600003830.mm.1	4,65	0,006589	0,39213	Cassette Exon	0,4
TC0600000493.mm.1	1,37	Cntnap2	PSR0600003832.mm.1	4,39	0,014833	0,451201	Cassette Exon	0,55
TC0600000493.mm.1	1,37	Cntnap2	JUC0600001939.mm.1	4,32	0,004188	0,362794		
TC0600000493.mm.1	1,37	Cntnap2	PSR0600003833.mm.1	3,95	0,004849	0,37168	Cassette Exon	0,39
TC0600000493.mm.1	1,37	Cntnap2	PSR0600003828.mm.1	3,47	0,00973	0,417839	Cassette Exon	0,27
TC0600000493.mm.1	1,37	Cntnap2	JUC0600001933.mm.1	2,78	0,005464	0,379939		
TC0600000493.mm.1	1,37	Cntnap2	PSR0600003831.mm.1	2,64	0,015417	0,454055	Cassette Exon	0,38
TC0600000493.mm.1	1,37	Cntnap2	JUC0600001930.mm.1	2,33	0,032361	0,519174		
TC0600000493.mm.1	1,37	Cntnap2	JUC0600001921.mm.1	2,23	0,030784	0,514144		
TC0600000493.mm.1	1,37	Cntnap2	JUC0600001934.mm.1	2,22	0,022884	0,488524		
TC0600000493.mm.1	1,37	Cntnap2	JUC0600001942.mm.1	-2,21	0,029851	0,511132		
TC0400002737.mm.1	-1,81	Lpar1	JUC0400011514.mm.1	9,51	0,024117	0,49322		
TC0400002737.mm.1	-1,81	Lpar1	PSR0400022163.mm.1	2,27	0,007948	0,403819	Alternative 3' Acce	0,16
TC0400002737.mm.1	-1,81	Lpar1	PSR0400022160.mm.1	2,18	0,013795	0,444974		
TC0400002737.mm.1	-1,81	Lpar1	PSR0400022151.mm.1	2,09	0,01095	0,425009	Cassette Exon	0,11

TC0700004413.mm.1	2,07	Fam53b	JUC0700019548.mm.1	9,5	0,002641	0,349612		
TC0700004413.mm.1	2,07	Fam53b	PSR0700037116.mm.1	-2,08	0,029434	0,509995	Cassette Exon	0,13
TC0700004413.mm.1	2,07	Fam53b	PSR0700037110.mm.1	-2,11	0,036027	0,529546	Alternative 3' Acce	0,18
TC0700004413.mm.1	2,07	Fam53b	PSR0700037120.mm.1	-2,25	0,045247	0,55367	Cassette Exon	0,32
TC0700004413.mm.1	2,07	Fam53b	PSR0700037131.mm.1	-2,52	0,027262	0,503682	Cassette Exon	0,14
TC0700004413.mm.1	2,07	Fam53b	JUC0700019549.mm.1	-3,7	0,02636	0,500927		
TC0700004413.mm.1	2,07	Fam53b	PSR0700037129.mm.1	-3,8	0,01735	0,46355	Alternative 5' Donc	0,31
TC1200001127.mm.1	4,72	Meg3; Mir770; Mir1906-1	PSR1200008076.mm.1	9,49	0,000968	0,313467		
TC1200001127.mm.1	4,72	Meg3; Mir770; Mir1906-1	PSR1200008071.mm.1	8,7	0,000071	0,251428		
TC1200001127.mm.1	4,72	Meg3; Mir770; Mir1906-1	PSR1200008078.mm.1	7,16	0,001364	0,32572		
TC1200001127.mm.1	4,72	Meg3; Mir770; Mir1906-1	PSR1200008070.mm.1	6,87	0,002239	0,34578		
TC1200001127.mm.1	4,72	Meg3; Mir770; Mir1906-1	PSR1200008073.mm.1	5,83	0,000649	0,304044	Intron Retention	0,29
TC1200001127.mm.1	4,72	Meg3; Mir770; Mir1906-1	PSR1200008080.mm.1	5,5	0,001015	0,316361		
TC1200001127.mm.1	4,72	Meg3; Mir770; Mir1906-1	PSR1200008072.mm.1	4,51	0,000867	0,311909	Cassette Exon	0,48
TC1200001127.mm.1	4,72	Meg3; Mir770; Mir1906-1	PSR1200008034.mm.1	4,1	0,007254	0,39826		
TC1200001127.mm.1	4,72	Meg3; Mir770; Mir1906-1	PSR1200008083.mm.1	3,97	0,005482	0,379939		
TC1200001127.mm.1	4,72	Meg3; Mir770; Mir1906-1	JUC1200004489.mm.1	3,92	0,04167	0,545356		
TC1200001127.mm.1	4,72	Meg3; Mir770; Mir1906-1	JUC1200004486.mm.1	3,86	0,024927	0,49582		
TC1200001127.mm.1	4,72	Meg3; Mir770; Mir1906-1	PSR1200008077.mm.1	3,64	0,000346	0,290082		
TC1200001127.mm.1	4,72	Meg3; Mir770; Mir1906-1	PSR1200008079.mm.1	2,95	0,007479	0,400381		
TC1200001127.mm.1	4,72	Meg3; Mir770; Mir1906-1	JUC1200004491.mm.1	2,93	0,010744	0,423772		
TC1200001127.mm.1	4,72	Meg3; Mir770; Mir1906-1	PSR1200008092.mm.1	2,74	0,005592	0,381035		
TC1200001127.mm.1	4,72	Meg3; Mir770; Mir1906-1	PSR1200008069.mm.1	2,34	0,020446	0,478018		
TC1200001127.mm.1	4,72	Meg3; Mir770; Mir1906-1	JUC1200004499.mm.1	2,24	0,016674	0,460085		
TC1200001127.mm.1	4,72	Meg3; Mir770; Mir1906-1	JUC1200004488.mm.1	2,07	0,004664	0,370181		
TC1200001127.mm.1	4,72	Meg3; Mir770; Mir1906-1	JUC1200004493.mm.1	-2,05	0,00362	0,356223		
TC1200001127.mm.1	4,72	Meg3; Mir770; Mir1906-1	PSR1200008090.mm.1	-2,36	0,002894	0,352207		
TC1200001127.mm.1	4,72	Meg3; Mir770; Mir1906-1	PSR1200008058.mm.1	-2,48	0,014579	0,449876	Intron Retention	0,09
TC1200001127.mm.1	4,72	Meg3; Mir770; Mir1906-1	PSR1200008093.mm.1	-2,58	0,003921	0,359818		
TC1200001127.mm.1	4,72	Meg3; Mir770; Mir1906-1	PSR1200008022.mm.1	-2,66	0,04036	0,541951	Alternative 3' Acce	0,3
TC1200001127.mm.1	4,72	Meg3; Mir770; Mir1906-1	PSR1200008104.mm.1	-2,67	0,049148	0,562349	Alternative 5' Donc	0,28
TC1200001127.mm.1	4,72	Meg3; Mir770; Mir1906-1	PSR1200008027.mm.1	-3,24	0,02989	0,511313	Alternative 3' Acce	0,3
TC1200001127.mm.1	4,72	Meg3; Mir770; Mir1906-1	PSR1200008052.mm.1	-3,26	0,004567	0,369396	Intron Retention	0,3
TC1200001127.mm.1	4,72	Meg3; Mir770; Mir1906-1	PSR1200008035.mm.1	-3,64	0,038353	0,53622	Cassette Exon	0,24
TC1200001127.mm.1	4,72	Meg3; Mir770; Mir1906-1	PSR1200008064.mm.1	-3,96	0,008274	0,405976	Intron Retention	0,56
TC1200001127.mm.1	4,72	Meg3; Mir770; Mir1906-1	PSR1200008032.mm.1	-3,99	0,004248	0,363417	Intron Retention	0,68
TC1200001127.mm.1	4,72	Meg3; Mir770; Mir1906-1	PSR1200008067.mm.1	-4,75	0,023126	0,489756	Intron Retention	0,65
TC1200001127.mm.1	4,72	Meg3; Mir770; Mir1906-1	PSR1200008103.mm.1	-4,76	0,035683	0,529027	Alternative 5' Donc	0,28
TC1200001127.mm.1	4,72	Meg3; Mir770; Mir1906-1	PSR1200008029.mm.1	-5,02	0,031377	0,516345	Intron Retention	0,47
TC1200001127.mm.1	4,72	Meg3; Mir770; Mir1906-1	PSR1200008055.mm.1	-5,04	0,001355	0,325439	Intron Retention	0,74
TC1200001127.mm.1	4,72	Meg3; Mir770; Mir1906-1	JUC1200004494.mm.1	-5,71	0,001668	0,335182		
TC1200001127.mm.1	4,72	Meg3; Mir770; Mir1906-1	JUC1200004502.mm.1	-8,21	0,009054	0,412827		
TC1200001127.mm.1	4,72	Meg3; Mir770; Mir1906-1	PSR1200008043.mm.1	-8,78	0,041799	0,545586		
TC1700001780.mm.1	1,86	Cpne5	JUC1700008837.mm.1	9,44	0,033957	0,524252		
TC1700001780.mm.1	1,86	Cpne5	JUC1700008850.mm.1	-2,17	0,00569	0,382387		
TC1700001780.mm.1	1,86	Cpne5	PSR1700016435.mm.1	-2,34	0,00708	0,396891	Alternative 5' Donc	0,16
TC1500001356.mm.1	-1,17	Stk3	JUC1500005889.mm.1	9,44	0,007844	0,403342		
TC1500001356.mm.1	-1,17	Stk3	JUC1500005890.mm.1	4,76	0,02473	0,494862		

TC0300003077.mm.1	-1,43	Sh3glb1	JUC0300012632.mm.1	9,41	0,00546	0,379939		
TC0300003113.mm.1	1,73	Lphn2	JUC0300012863.mm.1	9,36	0,007874	0,403545		
TC0300003113.mm.1	1,73	Lphn2	PSR0300024497.mm.1	-2,08	0,000024	0,22008	Cassette Exon	0,18
TC0300003113.mm.1	1,73	Lphn2	JUC0300012865.mm.1	-2,08	0,00284	0,352207		
TC0300003113.mm.1	1,73	Lphn2	JUC0300012861.mm.1	-2,12	0,023151	0,489814		
TC0300003113.mm.1	1,73	Lphn2	PSR0300024495.mm.1	-2,18	0,000452	0,298307	Cassette Exon	0,13
TC0300003113.mm.1	1,73	Lphn2	JUC0300012845.mm.1	-2,21	0,00545	0,379939		
TC0300003113.mm.1	1,73	Lphn2	JUC0300012838.mm.1	-2,24	0,00495	0,373403		
TC0300003113.mm.1	1,73	Lphn2	PSR0300024476.mm.1	-2,28	0,000045	0,24627	Cassette Exon	0,13
TC0300003113.mm.1	1,73	Lphn2	JUC0300012844.mm.1	-2,33	0,037368	0,533674		
TC0300003113.mm.1	1,73	Lphn2	PSR0300024480.mm.1	-2,34	0,037745	0,53468	Intron Retention	0,26
TC0300003113.mm.1	1,73	Lphn2	PSR0300024498.mm.1	-2,4	0,001026	0,316361	Cassette Exon	0,19
TC0300003113.mm.1	1,73	Lphn2	PSR0300024481.mm.1	-2,52	0,018945	0,471398	Intron Retention	0,26
TC0300003113.mm.1	1,73	Lphn2	PSR0300024504.mm.1	-2,57	0,01488	0,451706	Cassette Exon	0,21
TC0300003113.mm.1	1,73	Lphn2	JUC0300012853.mm.1	-2,62	0,00051	0,303579		
TC0300003113.mm.1	1,73	Lphn2	PSR0300024488.mm.1	-3,05	0,007326	0,399002	Alternative 5' Donc	0,26
TC0300003113.mm.1	1,73	Lphn2	JUC0300012842.mm.1	-3,77	0,010057	0,418902		
TC0700002623.mm.1	-1,14	Pou2f2; Mir6537; mmu-mi	JUC0700012037.mm.1	9,28	0,003262	0,354243		
TC0700002623.mm.1	-1,14	Pou2f2; Mir6537; mmu-mi	JUC0700012023.mm.1	-2,44	0,033029	0,521496		
TC1300002651.mm.1	4,73	Rab3c	JUC1300009622.mm.1	9,26	0,021032	0,480239		
TC1300002651.mm.1	4,73	Rab3c	PSR1300017971.mm.1	2,34	0,019371	0,473624		
TC1300002651.mm.1	4,73	Rab3c	PSR1300017972.mm.1	-2,71	0,000861	0,311909	Cassette Exon	0,02
TC1300002651.mm.1	4,73	Rab3c	JUC1300009623.mm.1	-4,11	0,014114	0,447137		
TC1300002651.mm.1	4,73	Rab3c	PSR1300017973.mm.1	-5,21	0,005524	0,380518	Alternative 5' Donc	0,21
TC1300002651.mm.1	4,73	Rab3c	JUC1300009625.mm.1	-5,95	0,022016	0,485226		
TC1300002651.mm.1	4,73	Rab3c	PSR1300017974.mm.1	-7,95	0,001504	0,329896	Cassette Exon	0,41
TC1600000054.mm.1	1,19	Rbfox1	PSR1600000485.mm.1	9,23	0,000618	0,304044	Cassette Exon	0,55
TC1600000054.mm.1	1,19	Rbfox1	JUC1600000269.mm.1	8,94	0,000085	0,255472		
TC1600000054.mm.1	1,19	Rbfox1	JUC1600000247.mm.1	7,24	0,000004	0,179072		
TC1600000054.mm.1	1,19	Rbfox1	JUC1600000248.mm.1	6,05	0,001961	0,340561		
TC1600000054.mm.1	1,19	Rbfox1	JUC1600000270.mm.1	4,86	0,005129	0,376377		
TC1600000054.mm.1	1,19	Rbfox1	JUC1600000259.mm.1	4,43	0,010085	0,418998		
TC1600000054.mm.1	1,19	Rbfox1	JUC1600000264.mm.1	3,47	0,029267	0,509825		
TC1600000054.mm.1	1,19	Rbfox1	PSR1600000498.mm.1	3,29	0,01446	0,44909	Cassette Exon	0,3
TC1600000054.mm.1	1,19	Rbfox1	JUC1600000251.mm.1	3,03	0,019598	0,474724		
TC1600000054.mm.1	1,19	Rbfox1	PSR1600000492.mm.1	2,46	0,004867	0,371692	Cassette Exon	0,38
TC1600000054.mm.1	1,19	Rbfox1	JUC1600000254.mm.1	2,39	0,017706	0,465159		
TC1600000054.mm.1	1,19	Rbfox1	JUC1600000260.mm.1	2,27	0,012454	0,43528		
TC1600000054.mm.1	1,19	Rbfox1	PSR1600000512.mm.1	-2,45	0,002177	0,344453	Cassette Exon	0,45
TC1600000054.mm.1	1,19	Rbfox1	JUC1600000272.mm.1	-2,81	0,020569	0,478426		
TC0700004626.mm.1	2,59	Sez6l2	JUC0700008200.mm.1	9,23	0,026157	0,50003		
TC0700004626.mm.1	2,59	Sez6l2	JUC0700008203.mm.1	8,83	0,007828	0,403089		
TC0700004626.mm.1	2,59	Sez6l2	JUC0700008199.mm.1	4,85	0,020758	0,479262		
TC0700004626.mm.1	2,59	Sez6l2	PSR0700015668.mm.1	3,71	0,010211	0,419922	Cassette Exon	0,46
TC0700004626.mm.1	2,59	Sez6l2	PSR0700015670.mm.1	3,71	0,001203	0,322097	Cassette Exon	0,33
TC0700004626.mm.1	2,59	Sez6l2	JUC0700008195.mm.1	3,69	0,001903	0,338712		
TC0700004626.mm.1	2,59	Sez6l2	JUC0700008192.mm.1	3,12	0,041734	0,545403		
TC0700004626.mm.1	2,59	Sez6l2	PSR0700015656.mm.1	2,65	0,030783	0,514144	Cassette Exon	0,32

TC0700004626.mm.1	2,59	Sez6l2	PSR0700015657.mm.1	2,65	0,00474	0,370933	Cassette Exon	0,27
TC0700004626.mm.1	2,59	Sez6l2	JUC0700008204.mm.1	2,65	0,023072	0,489593		
TC0700004626.mm.1	2,59	Sez6l2	PSR0700015667.mm.1	2,37	0,045111	0,553206	Cassette Exon	0,26
TC0700004626.mm.1	2,59	Sez6l2	JUC0700008194.mm.1	2,07	0,019099	0,472222		
TC0700004626.mm.1	2,59	Sez6l2	PSR0700015674.mm.1	-2,36	0,009831	0,417839	Alternative 5' Donc	0,26
TC0700004626.mm.1	2,59	Sez6l2	PSR0700015666.mm.1	-2,67	0,006952	0,395669	Alternative 3' Acce	0,38
TC0700004626.mm.1	2,59	Sez6l2	PSR0700015641.mm.1	-2,75	0,008213	0,405934	Alternative 3' Acce	0,28
TC0700004626.mm.1	2,59	Sez6l2	JUC0700008202.mm.1	-2,87	0,004092	0,36228		
TC0700004626.mm.1	2,59	Sez6l2	PSR0700015673.mm.1	-2,9	0,00387	0,358872	Intron Retention	0,3
TC0700004626.mm.1	2,59	Sez6l2	PSR0700015649.mm.1	-3,95	0,006671	0,393187	Alternative 5' Donc	0,36
TC0700004626.mm.1	2,59	Sez6l2	PSR0700015665.mm.1	-4,27	0,013147	0,440239	Alternative 3' Acce	0,38
TC0700004626.mm.1	2,59	Sez6l2	PSR0700015655.mm.1	-4,33	0,031634	0,517405	Alternative 5' Donc	0,51
TC0700004626.mm.1	2,59	Sez6l2	PSR0700015638.mm.1	-5,4	0,003899	0,35939	Alternative 3' Acce	0,41
TC1300001448.mm.1	-1,07	Lgals8	JUC1300004839.mm.1	9,22	0,033349	0,522553		
TC1300001448.mm.1	-1,07	Lgals8	PSR1300009224.mm.1	2,6	0,029854	0,511132	Alternative 3' Acce	0,23
TC1700001241.mm.1	-1,1	Eml4	JUC1700006303.mm.1	9,2	0,001027	0,316361		
TC1700001241.mm.1	-1,1	Eml4	JUC1700006279.mm.1	3,98	0,030936	0,514624		
TC0100002685.mm.1	1,34	Sphkap	JUC0100012370.mm.1	9,18	0,001113	0,317328		
TC0100002685.mm.1	1,34	Sphkap	JUC0100012371.mm.1	8,95	0,002284	0,34605		
TC0100003700.mm.1	-1,02	Enah	JUC0100017240.mm.1	9,17	0,035899	0,529399		
TC0100003700.mm.1	-1,02	Enah	JUC0100017246.mm.1	-2,18	0,015926	0,456718		
TC0400001076.mm.1	2,32	Dab1	JUC0400003773.mm.1	9,09	0,005451	0,379939		
TC0400001076.mm.1	2,32	Dab1	JUC0400003777.mm.1	3,61	0,00682	0,39387		
TC0400001076.mm.1	2,32	Dab1	PSR0400007382.mm.1	3,17	0,008302	0,406122	Cassette Exon	0,44
TC0400001076.mm.1	2,32	Dab1	PSR0400007386.mm.1	2,94	0,005713	0,38271	Cassette Exon	0,33
TC0400001076.mm.1	2,32	Dab1	JUC0400003770.mm.1	-2,14	0,019882	0,475806		
TC0400001076.mm.1	2,32	Dab1	PSR0400007390.mm.1	-2,5	0,036606	0,531472	Cassette Exon	0,14
TC0400001076.mm.1	2,32	Dab1	PSR0400007364.mm.1	-2,78	0,042832	0,548097	Cassette Exon	0,3
TC0400001076.mm.1	2,32	Dab1	JUC0400003791.mm.1	-3,14	0,020992	0,480143		
TC0400001076.mm.1	2,32	Dab1	JUC0400003768.mm.1	-4,08	0,018046	0,467148		
TC0700001916.mm.1	5,49	5430419D17Rik	JUC0700008907.mm.1	9,08	0,004053	0,361816		
TC0700001916.mm.1	5,49	5430419D17Rik	JUC0700008915.mm.1	8,38	0,011281	0,426885		
TC0700001916.mm.1	5,49	5430419D17Rik	PSR0700016812.mm.1	7,66	0,00174	0,335996	Cassette Exon	0,2
TC0700001916.mm.1	5,49	5430419D17Rik	JUC0700008914.mm.1	6,72	0,03382	0,523933		
TC0700001916.mm.1	5,49	5430419D17Rik	PSR0700016813.mm.1	6,61	0,003604	0,356223	Cassette Exon	0,33
TC0700001916.mm.1	5,49	5430419D17Rik	JUC0700008908.mm.1	6,42	0,004292	0,363973		
TC0700001916.mm.1	5,49	5430419D17Rik	PSR0700016824.mm.1	5,96	0,009606	0,417256	Cassette Exon	0,55
TC0700001916.mm.1	5,49	5430419D17Rik	JUC0700008906.mm.1	5,64	0,008405	0,406611		
TC0700001916.mm.1	5,49	5430419D17Rik	PSR0700016817.mm.1	3,55	0,047634	0,558743	Cassette Exon	0,27
TC0700001916.mm.1	5,49	5430419D17Rik	PSR0700016825.mm.1	3,46	0,041837	0,545586	Cassette Exon	0,37
TC0700001916.mm.1	5,49	5430419D17Rik	PSR0700016823.mm.1	3,11	0,039922	0,540966	Cassette Exon	0,33
TC0700001916.mm.1	5,49	5430419D17Rik	PSR0700016815.mm.1	2,86	0,020141	0,477041	Cassette Exon	0,44
TC0700001916.mm.1	5,49	5430419D17Rik	PSR0700016814.mm.1	2,84	0,009206	0,414013	Cassette Exon	0,44
TC1400001456.mm.1	7,11	Cadps	JUC1400005905.mm.1	9,06	0,000886	0,312614		
TC1400001456.mm.1	7,11	Cadps	JUC1400005906.mm.1	5,02	0,009764	0,417839		
TC1400001456.mm.1	7,11	Cadps	JUC1400005882.mm.1	3,2	0,000065	0,250119		
TC1400001456.mm.1	7,11	Cadps	PSR1400010959.mm.1	2,75	0,001913	0,339584	Cassette Exon	0,22
TC1400001456.mm.1	7,11	Cadps	PSR1400010969.mm.1	2,66	0,01164	0,42984	Cassette Exon	0,12

TC1400001456.mm.1	7,11	Cadps	JUC1400005887.mm.1	2,38	0,00395	0,359922		
TC1400001456.mm.1	7,11	Cadps	PSR1400010960.mm.1	2,19	0,000895	0,313363		
TC1400001456.mm.1	7,11	Cadps	JUC1400005895.mm.1	-2,19	0,006602	0,39213		
TC1400001456.mm.1	7,11	Cadps	JUC1400005889.mm.1	-2,71	0,001257	0,322251		
TC1400001456.mm.1	7,11	Cadps	PSR1400010977.mm.1	-2,86	0,015402	0,453964	Cassette Exon	0,28
TC1400001456.mm.1	7,11	Cadps	JUC1400005899.mm.1	-2,93	0,017986	0,466929		
TC1400001456.mm.1	7,11	Cadps	JUC1400005902.mm.1	-3,27	0,025498	0,497664		
TC1400001456.mm.1	7,11	Cadps	JUC1400005916.mm.1	-3,71	0,027126	0,503022		
TC1400001456.mm.1	7,11	Cadps	PSR1400010976.mm.1	-3,72	0,001275	0,322251	Cassette Exon	0,33
TC1400001456.mm.1	7,11	Cadps	PSR1400010956.mm.1	-4,12	0,001627	0,332831	Cassette Exon	0,75
TC1400001456.mm.1	7,11	Cadps	PSR1400010971.mm.1	-4,15	0,005953	0,385276	Cassette Exon	0,36
TC1400001456.mm.1	7,11	Cadps	JUC1400005891.mm.1	-5,25	0,018047	0,467148		
TC1400001456.mm.1	7,11	Cadps	JUC1400005886.mm.1	-5,63	0,027276	0,503732		
TC1400001456.mm.1	7,11	Cadps	JUC1400005901.mm.1	-6,12	0,003174	0,354243		
TC1400001456.mm.1	7,11	Cadps	JUC1400005890.mm.1	-6,16	0,000699	0,30566		
TC1400001456.mm.1	7,11	Cadps	PSR1400010945.mm.1	-6,9	0,001345	0,325349	Cassette Exon	0,41
TC1400001456.mm.1	7,11	Cadps	PSR1400010979.mm.1	-6,97	0,002623	0,349612	Cassette Exon	0,41
TC1400001456.mm.1	7,11	Cadps	JUC1400005914.mm.1	-7,91	0,029376	0,509995		
TC1400001456.mm.1	7,11	Cadps	JUC1400005909.mm.1	-10,93	0,007796	0,403089		
TC1400001456.mm.1	7,11	Cadps	JUC1400005913.mm.1	-12,13	0,000894	0,313363		
TC1400001456.mm.1	7,11	Cadps	JUC1400005885.mm.1	-12,47	0,033872	0,524121		
TC0800001055.mm.1	1,01	Rbl2	JUC0800004357.mm.1	9,04	0,002747	0,349833		
TC0400000727.mm.1	-1,06	Gm11482; LOC102632940	JUC0400002671.mm.1	8,98	0,010496	0,422028		
TC0700001741.mm.1	-1,04	Otoa	JUC0700007761.mm.1	8,97	0,010631	0,422841		
TC0700001741.mm.1	-1,04	Otoa	JUC0700007747.mm.1	3,12	0,005036	0,374502		
TC1600001184.mm.1	1,26	Grin2a	JUC1600004923.mm.1	8,89	0,000927	0,313363		
TC1600001184.mm.1	1,26	Grin2a	JUC1600004930.mm.1	2,97	0,031048	0,515019		
TC1600001184.mm.1	1,26	Grin2a	JUC1600004922.mm.1	2,53	0,021561	0,483034		
TC1600001184.mm.1	1,26	Grin2a	JUC1600004920.mm.1	2,2	0,003866	0,358861		
TC1600001184.mm.1	1,26	Grin2a	JUC1600004926.mm.1	-2,78	0,01033	0,42079		
TC0500000112.mm.1	-1,09	Cacna2d1	JUC0500000571.mm.1	8,87	0,000277	0,28803		
TC0500000112.mm.1	-1,09	Cacna2d1	JUC0500000541.mm.1	3,92	0,001886	0,338712		
TC0500000112.mm.1	-1,09	Cacna2d1	JUC0500000533.mm.1	2,22	0,02398	0,492734		
TC0500000112.mm.1	-1,09	Cacna2d1	JUC0500000549.mm.1	-2,43	0,039091	0,538395		
TC1800001660.mm.1	1,19	Ctdp1	JUC1800006615.mm.1	8,84	0,014144	0,447466		
TC1600001750.mm.1	-1,13	Tfg	JUC1600007385.mm.1	8,82	0,00203	0,341377		
TC1600001750.mm.1	-1,13	Tfg	PSR1600014189.mm.1	5,77	0,016984	0,461441	Cassette Exon	0,45
TC0600001634.mm.1	1,85	Ptpro	JUC0600006787.mm.1	8,73	0,006611	0,392269		
TC0600001634.mm.1	1,85	Ptpro	JUC0600006788.mm.1	6,65	0,031263	0,516001		
TC0600001634.mm.1	1,85	Ptpro	JUC0600006786.mm.1	3,97	0,009972	0,418446		
TC0600001634.mm.1	1,85	Ptpro	PSR0600012987.mm.1	3,49	0,004268	0,363868	Cassette Exon	0,23
TC0600001634.mm.1	1,85	Ptpro	JUC0600006793.mm.1	3,21	0,01643	0,459007		
TC0600001634.mm.1	1,85	Ptpro	JUC0600006794.mm.1	2,88	0,035725	0,529027		
TC0600001634.mm.1	1,85	Ptpro	JUC0600006802.mm.1	2,7	0,017448	0,463886		
TC0600001634.mm.1	1,85	Ptpro	PSR0600012985.mm.1	2,62	0,012011	0,432286	Cassette Exon	0,42
TC0600001634.mm.1	1,85	Ptpro	PSR0600012984.mm.1	2,32	0,047442	0,558383	Cassette Exon	0,39
TC0600001634.mm.1	1,85	Ptpro	PSR0600012997.mm.1	-2,02	0,020957	0,479982	Cassette Exon	0,03
TC0600001634.mm.1	1,85	Ptpro	JUC0600006790.mm.1	-2,19	0,031257	0,516001		

TC0600001634.mm.1	1,85	Ptpro	PSR0600013009.mm.1	-2,37	0,001339	0,325349	Cassette Exon	0,23
TC0600001634.mm.1	1,85	Ptpro	PSR0600013012.mm.1	-2,38	0,000425	0,297771	Cassette Exon	0,16
TC0600001634.mm.1	1,85	Ptpro	PSR0600012983.mm.1	-2,38	0,005255	0,377909	Cassette Exon	0,02
TC0600001634.mm.1	1,85	Ptpro	PSR0600013006.mm.1	-2,73	0,000965	0,313363	Cassette Exon	0,17
TC0600001634.mm.1	1,85	Ptpro	PSR0600012982.mm.1	-2,84	0,004988	0,373851	Alternative 3' Acce	0,07
TC0600001634.mm.1	1,85	Ptpro	JUC0600006809.mm.1	-3,27	0,011182	0,426414		
TC0600001634.mm.1	1,85	Ptpro	JUC0600006803.mm.1	-3,32	0,016297	0,458465		
TC0600001634.mm.1	1,85	Ptpro	JUC0600006797.mm.1	-4,11	0,026691	0,502057		
TC0600001634.mm.1	1,85	Ptpro	JUC0600006791.mm.1	-5,22	0,028984	0,508806		
TC0600001634.mm.1	1,85	Ptpro	JUC0600006795.mm.1	-5,33	0,043886	0,550573		
TC0600001634.mm.1	1,85	Ptpro	JUC0600006789.mm.1	-6,11	0,033863	0,524062		
TC0600001634.mm.1	1,85	Ptpro	JUC0600006813.mm.1	-9,04	0,008323	0,406313		
TC1700000493.mm.1	1,02	Gm16195	JUC1700002358.mm.1	8,68	0,014338	0,448173		
TC1400001621.mm.1	-1,1	Slmap	JUC1400006602.mm.1	8,66	0,000138	0,272178		
TC1400001621.mm.1	-1,1	Slmap	PSR1400012214.mm.1	2,22	0,028135	0,506248	Cassette Exon	0,24
TC0200000909.mm.1	1,07	March7	PSR0200007293.mm.1	8,55	0,015912	0,456594	Cassette Exon	0,26
TC1100001630.mm.1	-1,29	Gm11547; LOC102636037	JUC1100007831.mm.1	8,53	0,00895	0,411427		
TC0X00002482.mm.1	-1,64	Pls3	JUC0X00008121.mm.1	8,51	0,01397	0,446239		
TC0X00002482.mm.1	-1,64	Pls3	JUC0X00008122.mm.1	3,56	0,039333	0,539324		
TC0X00002482.mm.1	-1,64	Pls3	JUC0X00008115.mm.1	3,14	0,028577	0,507603		
TC0600000374.mm.1	1,3	Ttc26	JUC0600001433.mm.1	8,47	0,018991	0,471485		
TC0600000374.mm.1	1,3	Ttc26	JUC0600001432.mm.1	-2,03	0,004749	0,371039		
TC0600000374.mm.1	1,3	Ttc26	JUC0600001419.mm.1	-2,13	0,012588	0,435924		
TC0600000374.mm.1	1,3	Ttc26	PSR0600002798.mm.1	-2,32	0,01092	0,424705	Alternative 5' Donc	0,16
TC0700004492.mm.1	1,78	Caly	JUC0700019900.mm.1	8,41	0,003588	0,356223		
TC0700004492.mm.1	1,78	Caly	JUC0700019902.mm.1	4,42	0,006199	0,387873		
TC0700004492.mm.1	1,78	Caly	PSR0700037720.mm.1	4,1	0,004193	0,362939	Cassette Exon	0,41
TC0700004492.mm.1	1,78	Caly	PSR0700037726.mm.1	-2,22	0,00898	0,411895	Cassette Exon	0,1
TC0700004492.mm.1	1,78	Caly	JUC0700019899.mm.1	-2,25	0,003128	0,353892		
TC0700004492.mm.1	1,78	Caly	PSR0700037723.mm.1	-2,33	0,000532	0,304044	Alternative 3' Acce	0,09
TC0700004492.mm.1	1,78	Caly	JUC0700019903.mm.1	-3,23	0,017687	0,465014		
TC0900003043.mm.1	1,16	Rbm5	JUC0900014314.mm.1	8,4	0,029701	0,510949		
TC1300000262.mm.1	1,11	Hist1h1a	PSR1300001461.mm.1	8,39	0,045789	0,554863	Alternative 3' Acce	0,47
TC1700000650.mm.1	1,47	Ehmt2	JUC1700003571.mm.1	8,39	0,00507	0,375237		
TC1700000650.mm.1	1,47	Ehmt2	JUC1700003570.mm.1	3,17	0,00285	0,352207		
TC0200001378.mm.1	1,16	Ckap5; Snord67	JUC0200005463.mm.1	8,38	0,001039	0,316361		
TC0200001378.mm.1	1,16	Ckap5; Snord67	JUC0200005460.mm.1	-3,14	0,015293	0,453368		
TC0700003372.mm.1	1,75	A230057D06Rik; Mir344d	JUC0700015258.mm.1	8,31	0,027955	0,505663		
TC0700003372.mm.1	1,75	A230057D06Rik; Mir344d	JUC0700015265.mm.1	4,84	0,001584	0,331726		
TC0700003372.mm.1	1,75	A230057D06Rik; Mir344d	JUC0700015272.mm.1	4,78	0,000558	0,304044		
TC0700003372.mm.1	1,75	A230057D06Rik; Mir344d	PSR0700028891.mm.1	4,5	0,000759	0,308387	Cassette Exon	0,55
TC0700003372.mm.1	1,75	A230057D06Rik; Mir344d	JUC0700015268.mm.1	4,14	0,002865	0,352207		
TC0700003372.mm.1	1,75	A230057D06Rik; Mir344d	PSR0700028893.mm.1	3,12	0,004294	0,364023		
TC0700003372.mm.1	1,75	A230057D06Rik; Mir344d	JUC0700015276.mm.1	2,93	0,011065	0,425887		
TC0700003372.mm.1	1,75	A230057D06Rik; Mir344d	PSR0700028898.mm.1	2,69	0,011396	0,427549	Cassette Exon	0,21
TC0700003372.mm.1	1,75	A230057D06Rik; Mir344d	PSR0700028903.mm.1	2,5	0,014888	0,451777	Cassette Exon	0,32
TC0700003372.mm.1	1,75	A230057D06Rik; Mir344d	PSR0700028890.mm.1	2,41	0,040784	0,54318		
TC0700003372.mm.1	1,75	A230057D06Rik; Mir344d	PSR0700028914.mm.1	2,02	0,03065	0,513785	Cassette Exon	0,09

TC0700003372.mm.1	1,75	A230057D06Rik; Mir344d	PSR0700028901.mm.1	-2,11	0,013154	0,440265	Alternative 3' Acce	0,18
TC0700003372.mm.1	1,75	A230057D06Rik; Mir344d	PSR0700028918.mm.1	-2,14	0,012987	0,439245		
TC0700003372.mm.1	1,75	A230057D06Rik; Mir344d	PSR0700028865.mm.1	-2,15	0,007535	0,401261	Cassette Exon	0,1
TC0700003372.mm.1	1,75	A230057D06Rik; Mir344d	PSR0700028916.mm.1	-2,16	0,004043	0,36172	Alternative 5' Donc	0,17
TC0700003372.mm.1	1,75	A230057D06Rik; Mir344d	JUC0700015269.mm.1	-2,16	0,049859	0,563773		
TC0700003372.mm.1	1,75	A230057D06Rik; Mir344d	PSR0700028868.mm.1	-2,25	0,00294	0,352501	Alternative 3' Acce	0,15
TC0700003372.mm.1	1,75	A230057D06Rik; Mir344d	PSR0700028871.mm.1	-2,43	0,006839	0,393893	Cassette Exon	0,27
TC0700003372.mm.1	1,75	A230057D06Rik; Mir344d	PSR0700028878.mm.1	-2,44	0,005858	0,383747	Cassette Exon	0,13
TC0700003372.mm.1	1,75	A230057D06Rik; Mir344d	JUC0700015262.mm.1	-3,07	0,004936	0,373093		
TC0700003372.mm.1	1,75	A230057D06Rik; Mir344d	JUC0700015256.mm.1	-6,01	0,02309	0,489699		
TC0X00000078.mm.1	4,24	Pcsk1n	JUC0X00000287.mm.1	8,26	0,000706	0,306015		
TC0X00000078.mm.1	4,24	Pcsk1n	PSR0X00000615.mm.1	2,8	0,004192	0,362939	Cassette Exon	0,3
TC0X00000078.mm.1	4,24	Pcsk1n	PSR0X00000612.mm.1	-3,2	0,003309	0,354243	Alternative 3' Acce	0,31
TC0X00000078.mm.1	4,24	Pcsk1n	PSR0X00000616.mm.1	-3,99	0,001059	0,316361	Alternative 5' Donc	0,48
TC0600003050.mm.1	-1,15	Mical3	PSR0600023576.mm.1	8,24	0,016236	0,458312	Cassette Exon	0,29
TC1400002151.mm.1	-1,23	Zdhhc20; Gm22738	JUC1400009067.mm.1	8,24	0,009121	0,413316		
TC1400002151.mm.1	-1,23	Zdhhc20; Gm22738	JUC1400009066.mm.1	2,89	0,035415	0,528667		
TC1400000479.mm.1	2,43	Gpr137c	JUC1400002324.mm.1	8,19	0,000993	0,316361		
TC1400000479.mm.1	2,43	Gpr137c	JUC1400002325.mm.1	4,03	0,01224	0,433659		
TC1400000479.mm.1	2,43	Gpr137c	PSR1400003939.mm.1	2,1	0,002214	0,34564	Cassette Exon	0,37
TC1400000479.mm.1	2,43	Gpr137c	JUC1400002322.mm.1	-3,17	0,017508	0,463964		
TC1400000479.mm.1	2,43	Gpr137c	PSR1400003933.mm.1	-3,38	0,017114	0,46232	Alternative 3' Acce	0,24
TC1700002800.mm.1	1,81	Mapk8ip3	JUC1700008069.mm.1	8,16	0,005143	0,376442		
TC1700002800.mm.1	1,81	Mapk8ip3	JUC1700008071.mm.1	5,75	0,003811	0,358103		
TC1700002800.mm.1	1,81	Mapk8ip3	JUC1700008072.mm.1	2,09	0,027114	0,503022		
TC1700002800.mm.1	1,81	Mapk8ip3	JUC1700008054.mm.1	-2,2	0,026316	0,500721		
TC1700002800.mm.1	1,81	Mapk8ip3	PSR1700014807.mm.1	-2,66	0,012637	0,436161	Intron Retention	0,37
TC1700002800.mm.1	1,81	Mapk8ip3	PSR1700014802.mm.1	-2,68	0,008409	0,406611	Intron Retention	0,42
TC1700002800.mm.1	1,81	Mapk8ip3	PSR1700014781.mm.1	-2,86	0,001024	0,316361	Alternative 3' Acce	0,28
TC1700002800.mm.1	1,81	Mapk8ip3	JUC1700008063.mm.1	-2,86	0,004161	0,3623		
TC0X00003301.mm.1	-1,06	Reps2	JUC0X00010403.mm.1	8,15	0,00436	0,365487		
TC0200005052.mm.1	-1,63	Mafb	PSR0200043184.mm.1	8,12	0,021902	0,484824	Alternative 3' Acce	0,47
TC0500000640.mm.1	6,26	Uchl1	PSR0500005630.mm.1	8,11	0,001086	0,317191	Cassette Exon	0,42
TC0500000640.mm.1	6,26	Uchl1	JUC0500003111.mm.1	8,11	0,003675	0,357266		
TC0500000640.mm.1	6,26	Uchl1	JUC0500003108.mm.1	4,19	0,001585	0,331726		
TC0500000640.mm.1	6,26	Uchl1	PSR0500005627.mm.1	2,83	0,002816	0,352207	Cassette Exon	0,3
TC0500000640.mm.1	6,26	Uchl1	PSR0500005629.mm.1	-2,23	0,024761	0,495014		
TC0500000640.mm.1	6,26	Uchl1	PSR0500005626.mm.1	-2,71	0,000683	0,304417	Cassette Exon	0,08
TC0500000640.mm.1	6,26	Uchl1	PSR0500005622.mm.1	-2,96	0,011942	0,431753	Cassette Exon	0,36
TC0500000640.mm.1	6,26	Uchl1	JUC0500003106.mm.1	-3,82	0,009717	0,417826		
TC0500000640.mm.1	6,26	Uchl1	PSR0500005625.mm.1	-4,26	0,005542	0,380603	Alternative 3' Acce	0,21
TC0500000640.mm.1	6,26	Uchl1	PSR0500005624.mm.1	-5,01	0,00215	0,343679	Cassette Exon	0,4
TC0500000640.mm.1	6,26	Uchl1	PSR0500005620.mm.1	-5,23	0,002788	0,351436	Cassette Exon	0,36
TC0300001049.mm.1	-1,09	Trim33	JUC0300004399.mm.1	8,11	0,013812	0,445064		
TC0300001049.mm.1	-1,09	Trim33	PSR0300008491.mm.1	2,02	0,038439	0,536405	Alternative 3' Acce	0,06
TC0X00003418.mm.1	1,68	Rpgr	JUC0X00005956.mm.1	8,01	0,045531	0,554359		
TC0X00003418.mm.1	1,68	Rpgr	PSR0X00011781.mm.1	3,16	0,014732	0,450542	Cassette Exon	0,14
TC0X00003418.mm.1	1,68	Rpgr	PSR0X00011801.mm.1	-2,06	0,011127	0,426023	Cassette Exon	0,1

TC0X00003418.mm.1	1,68	Rpgr	JUC0X00005945.mm.1	-2,3	0,04942	0,563031		
TC0X00003418.mm.1	1,68	Rpgr	PSR0X00011805.mm.1	-3,19	0,023211	0,490032	Alternative 5' Donc	0,31
TC0X00003418.mm.1	1,68	Rpgr	JUC0X00005946.mm.1	-5,96	0,018521	0,469138		
TC0X00003418.mm.1	1,68	Rpgr	JUC0X00005961.mm.1	-6,52	0,048382	0,560607		
TC0800001432.mm.1	-1,34	Crispld2	JUC0800006438.mm.1	7,94	0,002357	0,34829		
TC0800002300.mm.1	-1,19	Palld	JUC0800009423.mm.1	7,94	0,023859	0,492097		
TC0800002300.mm.1	-1,19	Palld	JUC0800009413.mm.1	3,05	0,023222	0,490032		
TC0900002165.mm.1	1,02	Ccdc84	JUC0900010135.mm.1	7,92	0,008868	0,410539		
TC1200000035.mm.1	-1,12		JUC1200000239.mm.1	7,87	0,031998	0,518342		
TC0300003054.mm.1	-1,14	Pkn2	JUC0300012566.mm.1	7,87	0,033029	0,521496		
TC0200003080.mm.1	-1,25	Arhgap21	JUC0200012757.mm.1	7,86	0,002146	0,343679		
TC0200003080.mm.1	-1,25	Arhgap21	JUC0200012756.mm.1	3,24	0,009326	0,414973		
TC0200003080.mm.1	-1,25	Arhgap21	PSR0200025224.mm.1	2,31	0,032832	0,520896	Cassette Exon	0,38
TC0200003080.mm.1	-1,25	Arhgap21	JUC0200012761.mm.1	-2,55	0,013021	0,439354		
TC0200003632.mm.1	2,8	Kcnh7	JUC0200015768.mm.1	7,82	0,001445	0,328204		
TC0200003632.mm.1	2,8	Kcnh7	JUC0200015769.mm.1	3,34	0,024368	0,49362		
TC0200003632.mm.1	2,8	Kcnh7	JUC0200015763.mm.1	2,59	0,019392	0,473723		
TC0200003632.mm.1	2,8	Kcnh7	PSR0200031038.mm.1	-2,1	0,010067	0,418961	Cassette Exon	0,04
TC0200003632.mm.1	2,8	Kcnh7	PSR0200031031.mm.1	-2,18	0,016509	0,459483	Cassette Exon	0,11
TC0200003632.mm.1	2,8	Kcnh7	JUC0200015756.mm.1	-2,29	0,040109	0,541482		
TC0200003632.mm.1	2,8	Kcnh7	PSR0200031019.mm.1	-2,32	0,044504	0,551751	Cassette Exon	0,18
TC0200003632.mm.1	2,8	Kcnh7	PSR0200031021.mm.1	-2,42	0,006397	0,389654	Cassette Exon	0,14
TC0200003632.mm.1	2,8	Kcnh7	PSR0200031028.mm.1	-5,5	0,003742	0,357586	Alternative 3' Acce	0,46
TC0600001922.mm.1	2,84	Fam3c	JUC0600007871.mm.1	7,82	0,000866	0,311909		
TC0600001922.mm.1	2,84	Fam3c	PSR0600015036.mm.1	2,97	0,006891	0,394558	Cassette Exon	0,22
TC0600001922.mm.1	2,84	Fam3c	PSR0600015041.mm.1	-2,28	0,018526	0,46918	Alternative 3' Acce	0,2
TC0600001922.mm.1	2,84	Fam3c	PSR0600015034.mm.1	-2,41	0,046063	0,555645	Cassette Exon	0,17
TC0600001922.mm.1	2,84	Fam3c	PSR0600015048.mm.1	-2,49	0,035117	0,527689	Cassette Exon	0,15
TC0600001922.mm.1	2,84	Fam3c	PSR0600015047.mm.1	-2,61	0,028348	0,506843	Cassette Exon	0,15
TC0600001922.mm.1	2,84	Fam3c	PSR0600015044.mm.1	-2,69	0,02086	0,479521	Cassette Exon	0,24
TC0600001922.mm.1	2,84	Fam3c	JUC0600007868.mm.1	-2,83	0,018524	0,469156		
TC0600001922.mm.1	2,84	Fam3c	PSR0600015040.mm.1	-3,63	0,002079	0,342181	Cassette Exon	0,14
TC0400003653.mm.1	2,08	Epb4.1	JUC0400015597.mm.1	7,81	0,001893	0,338712		
TC0400003653.mm.1	2,08	Epb4.1	JUC0400015604.mm.1	2,68	0,030286	0,512627		
TC0400003653.mm.1	2,08	Epb4.1	PSR0400029896.mm.1	-2,03	0,000605	0,304044	Alternative 5' Donc	0,11
TC0400003653.mm.1	2,08	Epb4.1	JUC0400015587.mm.1	-2,05	0,049089	0,562174		
TC0400003653.mm.1	2,08	Epb4.1	JUC0400015599.mm.1	-2,07	0,014475	0,449232		
TC0400003653.mm.1	2,08	Epb4.1	PSR0400029893.mm.1	-2,17	0,016011	0,457051	Cassette Exon	0,11
TC0400003653.mm.1	2,08	Epb4.1	PSR0400029913.mm.1	-2,18	0,035639	0,528956	Alternative 3' Acce	0,19
TC0400003653.mm.1	2,08	Epb4.1	PSR0400029927.mm.1	-2,5	0,011417	0,427643	Cassette Exon	0,09
TC0400003653.mm.1	2,08	Epb4.1	PSR0400029929.mm.1	-2,79	0,002057	0,341838	Cassette Exon	0,19
TC0400003653.mm.1	2,08	Epb4.1	PSR0400029902.mm.1	-2,85	0,015741	0,455416	Cassette Exon	0,28
TC0400003653.mm.1	2,08	Epb4.1	JUC0400015572.mm.1	-2,97	0,004864	0,371692		
TC0400003653.mm.1	2,08	Epb4.1	JUC0400015573.mm.1	-3,34	0,045585	0,554548		
TC0400003653.mm.1	2,08	Epb4.1	JUC0400015570.mm.1	-3,39	0,004695	0,370496		
TC0400003653.mm.1	2,08	Epb4.1	JUC0400015603.mm.1	-3,65	0,028278	0,506588		
TC0400003653.mm.1	2,08	Epb4.1	JUC0400015576.mm.1	-4,1	0,001698	0,335395		
TC0400003653.mm.1	2,08	Epb4.1	PSR0400029899.mm.1	-4,53	0,020454	0,478018	Cassette Exon	0,43

TC0400003653.mm.1	2,08	Epb4.1	JUC0400015601.mm.1	-4,56	0,016006	0,457051		
TC0400003653.mm.1	2,08	Epb4.1	JUC0400015598.mm.1	-5,79	0,003745	0,357586		
TC0400003653.mm.1	2,08	Epb4.1	JUC0400015592.mm.1	-8,34	0,011307	0,427133		
TC1100001106.mm.1	2,16	Tusc5	JUC1100005270.mm.1	7,77	0,042732	0,548097		
TC1100001106.mm.1	2,16	Tusc5	PSR1100010030.mm.1	2,44	0,029015	0,508842	Cassette Exon	0,19
TC1100001106.mm.1	2,16	Tusc5	JUC1100005271.mm.1	2,25	0,040193	0,541714		
TC0900002640.mm.1	1,27	Tex9	JUC0900012299.mm.1	7,77	0,003247	0,354243		
TC0900002640.mm.1	1,27	Tex9	JUC0900012296.mm.1	3,34	0,043972	0,550752		
TC0900002640.mm.1	1,27	Tex9	JUC0900012313.mm.1	2,16	0,020852	0,479521		
TC0X00003146.mm.1	1,16	Lrch2	JUC0X00009976.mm.1	7,76	0,001461	0,328635		
TC0X00003146.mm.1	1,16	Lrch2	PSR0X00019711.mm.1	-2,01	0,029659	0,510834	Cassette Exon	0,08
TC0500001821.mm.1	1,26	Mtus2	JUC0500009115.mm.1	7,75	0,000558	0,304044		
TC0500001821.mm.1	1,26	Mtus2	JUC0500009108.mm.1	4,06	0,003203	0,354243		
TC0500001821.mm.1	1,26	Mtus2	JUC0500009110.mm.1	2,51	0,012618	0,436009		
TC0500001821.mm.1	1,26	Mtus2	PSR0500016807.mm.1	2,5	0,003299	0,354243	Cassette Exon	0,23
TC0500001821.mm.1	1,26	Mtus2	JUC0500009114.mm.1	2,49	0,047696	0,558944		
TC0500001821.mm.1	1,26	Mtus2	PSR0500016791.mm.1	2,11	0,022513	0,487114	Alternative 5' Donc	0,09
TC0700001996.mm.1	2,85	Gpr123	JUC0700009287.mm.1	7,73	0,002085	0,342653		
TC0700001996.mm.1	2,85	Gpr123	PSR0700017507.mm.1	-2,02	0,040984	0,543537	Alternative 3' Acce	0,28
TC0700001996.mm.1	2,85	Gpr123	PSR0700017497.mm.1	-2,76	0,017749	0,465424	Alternative 3' Acce	0,26
TC0700001996.mm.1	2,85	Gpr123	PSR0700017502.mm.1	-3,56	0,003726	0,357586	Cassette Exon	0,24
TC0700001996.mm.1	2,85	Gpr123	JUC0700009292.mm.1	-3,66	0,047409	0,558302		
TC1300002778.mm.1	-1,02		PSR1300015369.mm.1	7,72	0,024152	0,493245	Alternative 3' Acce	0,48
TC1000000812.mm.1	1,08	Bsg	JUC1000003190.mm.1	7,72	0,012048	0,432362		
TC1000000812.mm.1	1,08	Bsg	PSR1000005872.mm.1	3,53	0,019581	0,474672	Cassette Exon	0,4
TC0200002990.mm.1	-1,3	Ptpla	JUC0200012544.mm.1	7,68	0,002137	0,343459		
TC0200002990.mm.1	-1,3	Ptpla	PSR0200024760.mm.1	-2,11	0,031759	0,517489	Cassette Exon	0,45
TC0200002990.mm.1	-1,3	Ptpla	JUC0200012549.mm.1	-4,67	0,02091	0,479756		
TC0300001805.mm.1	5,77	Pex5l	JUC0300007364.mm.1	7,66	0,000003	0,179072		
TC0300001805.mm.1	5,77	Pex5l	JUC0300007363.mm.1	3,11	0,001439	0,32773		
TC0300001805.mm.1	5,77	Pex5l	PSR0300014008.mm.1	2,22	0,000027	0,229534	Cassette Exon	0,12
TC0300001805.mm.1	5,77	Pex5l	JUC0300007374.mm.1	-2,17	0,009174	0,413538		
TC0300001805.mm.1	5,77	Pex5l	PSR0300014015.mm.1	-2,42	0,02454	0,494018	Cassette Exon	0,13
TC0300001805.mm.1	5,77	Pex5l	JUC0300007366.mm.1	-2,42	0,030135	0,512017		
TC0300001805.mm.1	5,77	Pex5l	JUC0300007385.mm.1	-2,44	0,024396	0,493641		
TC0300001805.mm.1	5,77	Pex5l	PSR0300014019.mm.1	-2,55	0,021617	0,483425	Alternative 5' Donc	0,2
TC0300001805.mm.1	5,77	Pex5l	PSR0300013991.mm.1	-2,72	0,001411	0,326543	Alternative 3' Acce	0,23
TC0300001805.mm.1	5,77	Pex5l	PSR0300014005.mm.1	-2,75	0,007918	0,403819	Cassette Exon	0,2
TC0300001805.mm.1	5,77	Pex5l	PSR0300013989.mm.1	-2,96	0,022234	0,485896	Alternative 3' Acce	0,23
TC0300001805.mm.1	5,77	Pex5l	PSR0300014013.mm.1	-3,22	0,016087	0,45751	Cassette Exon	0,48
TC0300001805.mm.1	5,77	Pex5l	PSR0300014017.mm.1	-3,93	0,004278	0,363899	Alternative 5' Donc	0,25
TC0300001805.mm.1	5,77	Pex5l	JUC0300007373.mm.1	-3,94	0,007948	0,403819		
TC0300001805.mm.1	5,77	Pex5l	PSR0300014018.mm.1	-4,71	0,007882	0,403723	Alternative 5' Donc	0,25
TC0300001805.mm.1	5,77	Pex5l	PSR0300014020.mm.1	-4,78	0,009331	0,414973	Alternative 5' Donc	0,2
TC0300001805.mm.1	5,77	Pex5l	PSR0300014027.mm.1	-4,96	0,004384	0,366156	Cassette Exon	0,28
TC0300001805.mm.1	5,77	Pex5l	PSR0300013999.mm.1	-5,52	0,002592	0,349501	Alternative 5' Donc	0,36
TC0300001805.mm.1	5,77	Pex5l	PSR0300014016.mm.1	-5,55	0,006437	0,390047	Alternative 5' Donc	0,25
TC0300001805.mm.1	5,77	Pex5l	PSR0300014025.mm.1	-5,79	0,010033	0,41878	Cassette Exon	0,34

TC0300001805.mm.1	5,77	Pex5l	PSR0300014028.mm.1	-5,91	0,0055	0,379939	Cassette Exon	0,28
TC0300001805.mm.1	5,77	Pex5l	PSR0300014001.mm.1	-6,99	0,005506	0,380032	Alternative 5' Donc	0,36
TC0300001805.mm.1	5,77	Pex5l	JUC0300007384.mm.1	-8,05	0,000855	0,311909		
TC0300001805.mm.1	5,77	Pex5l	JUC0300007383.mm.1	-10,36	0,003067	0,353892		
TC0100003080.mm.1	3,24	Nfasc	JUC0100014074.mm.1	7,64	0,000529	0,304044		
TC0100003080.mm.1	3,24	Nfasc	JUC0100014046.mm.1	3,39	0,003459	0,355628		
TC0100003080.mm.1	3,24	Nfasc	JUC0100014050.mm.1	3,29	0,001988	0,341091		
TC0100003080.mm.1	3,24	Nfasc	JUC0100014075.mm.1	3,12	0,001064	0,316361		
TC0100003080.mm.1	3,24	Nfasc	JUC0100014041.mm.1	2,01	0,012837	0,437716		
TC0100003080.mm.1	3,24	Nfasc	PSR0100024756.mm.1	-2,14	0,003437	0,3554	Cassette Exon	0,36
TC0100003080.mm.1	3,24	Nfasc	PSR0100024732.mm.1	-2,24	0,009634	0,417354	Cassette Exon	0,25
TC0100003080.mm.1	3,24	Nfasc	JUC0100014047.mm.1	-2,39	0,02434	0,49362		
TC0100003080.mm.1	3,24	Nfasc	PSR0100024741.mm.1	-2,42	0,03053	0,51337	Alternative 3' Acce	0,14
TC0100003080.mm.1	3,24	Nfasc	PSR0100024749.mm.1	-2,55	0,005269	0,378003	Cassette Exon	0,27
TC0100003080.mm.1	3,24	Nfasc	PSR0100024740.mm.1	-2,93	0,009899	0,418332	Cassette Exon	0,14
TC0100003080.mm.1	3,24	Nfasc	PSR0100024765.mm.1	-2,96	0,028811	0,508347	Cassette Exon	0,09
TC0100003080.mm.1	3,24	Nfasc	PSR0100024734.mm.1	-3,07	0,008406	0,406611	Cassette Exon	0,36
TC0100003080.mm.1	3,24	Nfasc	JUC0100014068.mm.1	-3,44	0,009528	0,416602		
TC0100003080.mm.1	3,24	Nfasc	PSR0100024764.mm.1	-3,65	0,01209	0,43255	Cassette Exon	0,52
TC0100003080.mm.1	3,24	Nfasc	PSR0100024730.mm.1	-3,78	0,002703	0,349612	Cassette Exon	0,18
TC0100003080.mm.1	3,24	Nfasc	JUC0100014058.mm.1	-3,82	0,013725	0,444758		
TC0100003080.mm.1	3,24	Nfasc	JUC0100014067.mm.1	-4,2	0,027951	0,505663		
TC0100003080.mm.1	3,24	Nfasc	JUC0100014052.mm.1	-4,51	0,027322	0,503766		
TC0100003080.mm.1	3,24	Nfasc	JUC0100014070.mm.1	-4,63	0,009815	0,417839		
TC0100003080.mm.1	3,24	Nfasc	PSR0100024737.mm.1	-4,8	0,004738	0,370933	Alternative 5' Donc	0,44
TC0100003080.mm.1	3,24	Nfasc	JUC0100014064.mm.1	-5,08	0,022101	0,485486		
TC0100003080.mm.1	3,24	Nfasc	JUC0100014059.mm.1	-5,57	0,005252	0,377753		
TC0100003080.mm.1	3,24	Nfasc	JUC0100014063.mm.1	-5,64	0,006122	0,387322		
TC1200001023.mm.1	1,78	Unc79	JUC1200004021.mm.1	7,64	0,001348	0,325427		
TC1200001023.mm.1	1,78	Unc79	JUC1200004065.mm.1	6,9	0,009909	0,418378		
TC1200001023.mm.1	1,78	Unc79	PSR1200007209.mm.1	6,72	0,003166	0,353901	Cassette Exon	0,47
TC1200001023.mm.1	1,78	Unc79	PSR1200007211.mm.1	6,1	0,03286	0,521076		
TC1200001023.mm.1	1,78	Unc79	PSR1200007217.mm.1	4,95	0,004035	0,361636	Cassette Exon	0,5
TC1200001023.mm.1	1,78	Unc79	PSR1200007218.mm.1	4,81	0,032495	0,519646	Cassette Exon	0,42
TC1200001023.mm.1	1,78	Unc79	JUC1200004039.mm.1	3,93	0,00295	0,352501		
TC1200001023.mm.1	1,78	Unc79	PSR1200007215.mm.1	3,1	0,029829	0,511132	Cassette Exon	0,28
TC1200001023.mm.1	1,78	Unc79	PSR1200007207.mm.1	2,98	0,022531	0,487133	Cassette Exon	0,13
TC1200001023.mm.1	1,78	Unc79	JUC1200004020.mm.1	2,93	0,005812	0,383104		
TC1200001023.mm.1	1,78	Unc79	JUC1200004064.mm.1	2,35	0,003312	0,354243		
TC1200001023.mm.1	1,78	Unc79	PSR1200007227.mm.1	-2,06	0,031384	0,516345	Cassette Exon	0,14
TC1200001023.mm.1	1,78	Unc79	JUC1200004027.mm.1	-2,07	0,033013	0,521478		
TC1200001023.mm.1	1,78	Unc79	JUC1200004058.mm.1	-2,12	0,002703	0,349612		
TC1200001023.mm.1	1,78	Unc79	PSR1200007243.mm.1	-2,13	0,011745	0,430016	Cassette Exon	0,16
TC1200001023.mm.1	1,78	Unc79	JUC1200004042.mm.1	-2,15	0,031427	0,516564		
TC1200001023.mm.1	1,78	Unc79	PSR1200007261.mm.1	-2,18	0,009417	0,415914	Cassette Exon	0,1
TC1200001023.mm.1	1,78	Unc79	JUC1200004018.mm.1	-2,2	0,022224	0,485896		
TC1200001023.mm.1	1,78	Unc79	JUC1200004073.mm.1	-2,21	0,033359	0,522553		
TC1200001023.mm.1	1,78	Unc79	PSR1200007276.mm.1	-2,23	0,007744	0,402774	Cassette Exon	0,09

TC1200001023.mm.1	1,78	Unc79	PSR1200007260.mm.1	-2,56	0,040199	0,541714	Cassette Exon	0,13
TC1200001023.mm.1	1,78	Unc79	PSR1200007278.mm.1	-2,59	0,035638	0,528956	Alternative 5' Donc	0,1
TC1200001023.mm.1	1,78	Unc79	PSR1200007258.mm.1	-2,65	0,003258	0,354243	Cassette Exon	0,15
TC1200001023.mm.1	1,78	Unc79	JUC1200004030.mm.1	-2,65	0,000823	0,311886		
TC1200001023.mm.1	1,78	Unc79	PSR1200007210.mm.1	-2,68	0,011478	0,428022	Alternative 3' Acce	0,32
TC1200001023.mm.1	1,78	Unc79	JUC1200004048.mm.1	-2,68	0,029863	0,511132		
TC1200001023.mm.1	1,78	Unc79	PSR1200007223.mm.1	-2,78	0,045939	0,555316	Cassette Exon	0,17
TC1200001023.mm.1	1,78	Unc79	JUC1200004074.mm.1	-2,78	0,015109	0,452466		
TC1200001023.mm.1	1,78	Unc79	PSR1200007257.mm.1	-2,79	0,001009	0,316361	Cassette Exon	0,24
TC1200001023.mm.1	1,78	Unc79	JUC1200004035.mm.1	-2,92	0,006471	0,390467		
TC1200001023.mm.1	1,78	Unc79	PSR1200007281.mm.1	-3,13	0,012933	0,438537	Alternative 5' Donc	0,1
TC1200001023.mm.1	1,78	Unc79	JUC1200004025.mm.1	-3,16	0,00135	0,325427		
TC1200001023.mm.1	1,78	Unc79	PSR1200007224.mm.1	-3,25	0,005298	0,378059	Alternative 3' Acce	0,26
TC1100001816.mm.1	-1,14	Cep112	JUC1100008914.mm.1	7,62	0,014785	0,45081		
TC1100001816.mm.1	-1,14	Cep112	JUC1100008908.mm.1	4,09	0,001081	0,317191		
TC1100001816.mm.1	-1,14	Cep112	PSR1100017106.mm.1	3,83	0,007183	0,397855	Cassette Exon	0,39
TC1100001816.mm.1	-1,14	Cep112	JUC1100008909.mm.1	3,65	0,011645	0,42985		
TC1100001816.mm.1	-1,14	Cep112	PSR1100017120.mm.1	3,18	0,002552	0,349501	Cassette Exon	0,36
TC1100001816.mm.1	-1,14	Cep112	PSR1100017105.mm.1	3,09	0,008266	0,405976	Cassette Exon	0,39
TC1100001816.mm.1	-1,14	Cep112	JUC1100008906.mm.1	3	0,011913	0,431645		
TC1100001816.mm.1	-1,14	Cep112	PSR1100017122.mm.1	2,69	0,024121	0,493222	Cassette Exon	0,29
TC1100001816.mm.1	-1,14	Cep112	PSR1100017104.mm.1	2,69	0,010402	0,421477	Cassette Exon	0,25
TC1100001816.mm.1	-1,14	Cep112	JUC1100008903.mm.1	2,68	0,023229	0,490032		
TC1100001816.mm.1	-1,14	Cep112	PSR1100017096.mm.1	2,52	0,000181	0,283791	Cassette Exon	0,13
TC1100001816.mm.1	-1,14	Cep112	PSR1100017116.mm.1	2,46	0,002821	0,352207	Cassette Exon	0,18
TC1100001816.mm.1	-1,14	Cep112	PSR1100017126.mm.1	2,44	0,00797	0,403853	Cassette Exon	0,13
TC1100001816.mm.1	-1,14	Cep112	PSR1100017112.mm.1	2,43	0,000447	0,297771	Cassette Exon	0,28
TC1100001816.mm.1	-1,14	Cep112	PSR1100017125.mm.1	2,41	0,004759	0,371039	Cassette Exon	0,13
TC1100001816.mm.1	-1,14	Cep112	PSR1100017099.mm.1	2,38	0,022064	0,485335	Cassette Exon	0,17
TC1100001816.mm.1	-1,14	Cep112	PSR1100017108.mm.1	2,37	0,009545	0,416659	Cassette Exon	0,23
TC1100001816.mm.1	-1,14	Cep112	PSR1100017111.mm.1	2,34	0,004753	0,371039	Cassette Exon	0,38
TC1100001816.mm.1	-1,14	Cep112	PSR1100017100.mm.1	2,32	0,035023	0,527495	Cassette Exon	0,23
TC1100001816.mm.1	-1,14	Cep112	PSR1100017113.mm.1	2,29	0,003737	0,357586	Cassette Exon	0,17
TC1100001816.mm.1	-1,14	Cep112	PSR1100017110.mm.1	2,19	0,01072	0,423727	Cassette Exon	0,26
TC1100001816.mm.1	-1,14	Cep112	PSR1100017098.mm.1	2,19	0,020043	0,476453	Cassette Exon	0,17
TC1100001816.mm.1	-1,14	Cep112	JUC1100008922.mm.1	2,06	0,007342	0,399179		
TC1100001816.mm.1	-1,14	Cep112	PSR1100017101.mm.1	2,01	0,011662	0,429939	Cassette Exon	0,18
TC1400000795.mm.1	-1,11	Nfatc4	JUC1400003431.mm.1	7,59	0,011092	0,425947		
TC0700001446.mm.1	1,13	Trpc2; Xndc1; Xntrpc	JUC0700006482.mm.1	7,57	0,022194	0,485778		
TC0800002741.mm.1	3,13	Cngb1	JUC0800011484.mm.1	7,54	0,004351	0,365487		
TC0800002741.mm.1	3,13	Cngb1	JUC0800011465.mm.1	7,53	0,002757	0,350151		
TC0800002741.mm.1	3,13	Cngb1	PSR0800021040.mm.1	6,89	0,00005	0,24627	Cassette Exon	0,35
TC0800002741.mm.1	3,13	Cngb1	JUC0800011456.mm.1	6,82	0,00056	0,304044		
TC0800002741.mm.1	3,13	Cngb1	PSR0800021072.mm.1	6,32	0,000211	0,28803	Alternative 5' Donc	0,44
TC0800002741.mm.1	3,13	Cngb1	JUC0800011463.mm.1	5,27	0,003774	0,357586		
TC0800002741.mm.1	3,13	Cngb1	PSR0800021039.mm.1	5,06	0,000461	0,298999	Cassette Exon	0,4
TC0800002741.mm.1	3,13	Cngb1	PSR0800021033.mm.1	4,75	0,011906	0,431549	Alternative 3' Acce	0,48
TC0800002741.mm.1	3,13	Cngb1	JUC0800011466.mm.1	4,13	0,012011	0,432286		

TC0800002741.mm.1	3,13	Cngb1	JUC0800011464.mm.1	3,98	0,007169	0,397855		
TC0800002741.mm.1	3,13	Cngb1	JUC0800011462.mm.1	3,83	0,020918	0,479811		
TC0800002741.mm.1	3,13	Cngb1	JUC0800011478.mm.1	3,78	0,003658	0,357266		
TC0800002741.mm.1	3,13	Cngb1	JUC0800011458.mm.1	3,57	0,000177	0,283396		
TC0800002741.mm.1	3,13	Cngb1	JUC0800011481.mm.1	3,53	0,002239	0,34578		
TC0800002741.mm.1	3,13	Cngb1	PSR0800021043.mm.1	2,86	0,024012	0,492783	Cassette Exon	0,43
TC0800002741.mm.1	3,13	Cngb1	JUC0800011479.mm.1	2,82	0,001374	0,32572		
TC0800002741.mm.1	3,13	Cngb1	JUC0800011460.mm.1	2,54	0,015783	0,455613		
TC0800002741.mm.1	3,13	Cngb1	JUC0800011467.mm.1	2,51	0,011719	0,429969		
TC0800002741.mm.1	3,13	Cngb1	PSR0800021038.mm.1	2,46	0,00363	0,3566	Cassette Exon	0,3
TC0800002741.mm.1	3,13	Cngb1	JUC0800011461.mm.1	2,46	0,011823	0,430456		
TC0800002741.mm.1	3,13	Cngb1	PSR0800021046.mm.1	2,45	0,010288	0,420369	Cassette Exon	0,4
TC0800002741.mm.1	3,13	Cngb1	PSR0800021047.mm.1	2,39	0,000925	0,313363	Cassette Exon	0,33
TC0800002741.mm.1	3,13	Cngb1	PSR0800021068.mm.1	2,37	0,000113	0,267977	Cassette Exon	0,33
TC0800002741.mm.1	3,13	Cngb1	JUC0800011474.mm.1	2,18	0,020063	0,476514		
TC0800002741.mm.1	3,13	Cngb1	JUC0800011457.mm.1	2,15	0,00405	0,361816		
TC0800002741.mm.1	3,13	Cngb1	PSR0800021056.mm.1	-2,68	0,037023	0,532502	Cassette Exon	0,31
TC0800002741.mm.1	3,13	Cngb1	JUC0800011492.mm.1	-2,69	0,020172	0,477086		
TC0800002741.mm.1	3,13	Cngb1	PSR0800021036.mm.1	-2,73	0,006644	0,392838		
TC0800002741.mm.1	3,13	Cngb1	JUC0800011469.mm.1	-2,81	0,016382	0,458696		
TC0800002741.mm.1	3,13	Cngb1	PSR0800021063.mm.1	-2,86	0,031399	0,516434	Alternative 5' Donc	0,18
TC0800002741.mm.1	3,13	Cngb1	PSR0800021064.mm.1	-3,11	0,006454	0,390284	Cassette Exon	0,24
TC0800002741.mm.1	3,13	Cngb1	PSR0800021052.mm.1	-3,13	0,00953	0,416602	Cassette Exon	0,34
TC0800002741.mm.1	3,13	Cngb1	PSR0800021053.mm.1	-3,55	0,035326	0,528383	Cassette Exon	0,37
TC0800002741.mm.1	3,13	Cngb1	JUC0800011493.mm.1	-3,81	0,005909	0,384002		
TC0800002741.mm.1	3,13	Cngb1	PSR0800021055.mm.1	-3,9	0,004907	0,372662	Alternative 3' Acce	0,33
TC0800002741.mm.1	3,13	Cngb1	JUC0800011480.mm.1	-5,31	0,001246	0,322251		
TC0800002741.mm.1	3,13	Cngb1	JUC0800011486.mm.1	-5,34	0,001463	0,328846		
TC0800002741.mm.1	3,13	Cngb1	JUC0800011490.mm.1	-5,6	0,018383	0,468484		
TC0800002741.mm.1	3,13	Cngb1	JUC0800011488.mm.1	-6,74	0,003746	0,357586		
TC0700000296.mm.1	1,16	Rtn2	JUC0700001099.mm.1	7,54	0,003686	0,357266		
TC0700000296.mm.1	1,16	Rtn2	JUC0700001097.mm.1	3,71	0,017352	0,46355		
TC0700000296.mm.1	1,16	Rtn2	PSR0700002262.mm.1	3,48	0,002792	0,351449	Cassette Exon	0,39
TC0700000296.mm.1	1,16	Rtn2	JUC0700001106.mm.1	-2,11	0,02564	0,498009		
TC1200001583.mm.1	1,82	Sntg2	JUC1200006061.mm.1	7,52	0,000013	0,185427		
TC1200001583.mm.1	1,82	Sntg2	JUC1200006046.mm.1	5,42	0,002477	0,349135		
TC1200001583.mm.1	1,82	Sntg2	PSR1200010978.mm.1	3,85	0,000735	0,307808	Cassette Exon	0,37
TC1200001583.mm.1	1,82	Sntg2	PSR1200010979.mm.1	3,14	0,008257	0,405976	Cassette Exon	0,34
TC1200001583.mm.1	1,82	Sntg2	PSR1200010977.mm.1	2,92	0,009159	0,413447	Cassette Exon	0,21
TC1200001583.mm.1	1,82	Sntg2	JUC1200006058.mm.1	2,87	0,002667	0,349612		
TC1200001583.mm.1	1,82	Sntg2	JUC1200006055.mm.1	2,81	0,001895	0,338712		
TC1200001583.mm.1	1,82	Sntg2	PSR1200010985.mm.1	2,77	0,009069	0,412827	Cassette Exon	0,07
TC1200001583.mm.1	1,82	Sntg2	JUC1200006060.mm.1	2,53	0,0044	0,3664		
TC1200001583.mm.1	1,82	Sntg2	JUC1200006056.mm.1	2,27	0,003937	0,359818		
TC1200001583.mm.1	1,82	Sntg2	JUC1200006051.mm.1	-2,43	0,044911	0,55271		
TC1200001583.mm.1	1,82	Sntg2	PSR1200010972.mm.1	-2,64	0,007822	0,403089	Cassette Exon	0,13
TC1200001583.mm.1	1,82	Sntg2	JUC1200006065.mm.1	-2,98	0,000273	0,28803		
TC1200001583.mm.1	1,82	Sntg2	PSR1200010991.mm.1	-3,01	0,005785	0,383104	Alternative 5' Donc	0,19

TC0600001289.mm.1	1,46	Rho	JUC0600005319.mm.1	7,5	0,001243	0,322251		
TC0600001289.mm.1	1,46	Rho	PSR0600010228.mm.1	7,04	0,002091	0,342919	Cassette Exon	0,34
TC0600001289.mm.1	1,46	Rho	PSR0600010227.mm.1	2,36	0,006333	0,389463	Cassette Exon	0,34
TC0400002222.mm.1	1,15	Fam92a	JUC0400009727.mm.1	7,47	0,033434	0,522793		
TC0400002222.mm.1	1,15	Fam92a	JUC0400009728.mm.1	3,93	0,026625	0,50179		
TC0400002222.mm.1	1,15	Fam92a	JUC0400009723.mm.1	-2,68	0,030253	0,512521		
TC1100003081.mm.1	-1,46		JUC1100014867.mm.1	7,43	0,047616	0,558743		
TC0300002272.mm.1	1,21		JUC0300008957.mm.1	7,43	0,006756	0,393343		
TC0600000135.mm.1	-1,12	St7	JUC0600000484.mm.1	7,41	0,003229	0,354243		
TC0600000135.mm.1	-1,12	St7	JUC0600000466.mm.1	-2,03	0,024787	0,495079		
TC1500001711.mm.1	-1,13	Mroh4	JUC1500007354.mm.1	7,38	0,026603	0,501778		
TC1700000987.mm.1	1,73	St6gal2	JUC1700004993.mm.1	7,35	0,008636	0,408463		
TC1700000987.mm.1	1,73	St6gal2	JUC1700004990.mm.1	5,54	0,001195	0,321568		
TC1700000987.mm.1	1,73	St6gal2	PSR1700009399.mm.1	4,54	0,010071	0,418974	Cassette Exon	0,45
TC1700000987.mm.1	1,73	St6gal2	PSR1700009398.mm.1	3,59	0,010483	0,421891	Cassette Exon	0,34
TC1700000987.mm.1	1,73	St6gal2	PSR1700009402.mm.1	2,79	0,009728	0,417839	Cassette Exon	0,3
TC1700000987.mm.1	1,73	St6gal2	PSR1700009397.mm.1	2,49	0,018768	0,470771	Cassette Exon	0,33
TC1700000987.mm.1	1,73	St6gal2	JUC1700004991.mm.1	2,31	0,021757	0,484278		
TC1700000987.mm.1	1,73	St6gal2	PSR1700009390.mm.1	-2,34	0,043739	0,550304	Alternative 3' Acce	0,15
TC1100002122.mm.1	2,06	Nefh	JUC1100010791.mm.1	7,34	0,024227	0,493294		
TC1100002122.mm.1	2,06	Nefh	PSR1100020644.mm.1	-2,09	0,040231	0,541714	Alternative 5' Donc	0,15
TC0200000382.mm.1	-1,67	Il1rn	JUC0200000876.mm.1	7,34	0,004166	0,3623		
TC0700000388.mm.1	1,08	Ethe1	JUC0700001396.mm.1	7,34	0,003518	0,355749		
TC0700000388.mm.1	1,08	Ethe1	JUC0700001399.mm.1	-2,06	0,009804	0,417839		
TC0700000388.mm.1	1,08	Ethe1	JUC0700001400.mm.1	-2,24	0,000734	0,307808		
TC0200000438.mm.1	1,32	Kcnt1	JUC0200001278.mm.1	7,33	0,001827	0,337273		
TC0200000438.mm.1	1,32	Kcnt1	JUC0200001301.mm.1	4,03	0,008766	0,409781		
TC0200000438.mm.1	1,32	Kcnt1	PSR0200002715.mm.1	3,2	0,00008	0,25272	Cassette Exon	0,38
TC0200000438.mm.1	1,32	Kcnt1	PSR0200002754.mm.1	3,09	0,002607	0,349501	Cassette Exon	0,37
TC0200000438.mm.1	1,32	Kcnt1	PSR0200002742.mm.1	2,24	0,0099	0,418332	Cassette Exon	0,05
TC0200000438.mm.1	1,32	Kcnt1	PSR0200002716.mm.1	2,06	0,010853	0,424161	Cassette Exon	0,23
TC0200000438.mm.1	1,32	Kcnt1	JUC0200001277.mm.1	2,03	0,023328	0,490065		
TC0200000438.mm.1	1,32	Kcnt1	JUC0200001282.mm.1	2,01	0,017568	0,464258		
TC0200000438.mm.1	1,32	Kcnt1	JUC0200001310.mm.1	-2,09	0,008401	0,406611		
TC0200000438.mm.1	1,32	Kcnt1	JUC0200001294.mm.1	-2,46	0,005268	0,378003		
TC0200000438.mm.1	1,32	Kcnt1	PSR0200002751.mm.1	-3,16	0,03158	0,517177	Alternative 3' Acce	0,3
TC0200000438.mm.1	1,32	Kcnt1	PSR0200002708.mm.1	-3,22	0,002965	0,352501	Cassette Exon	0,22
TC1100001125.mm.1	1,21	Git1	JUC1100005361.mm.1	7,32	0,000556	0,304044		
TC1100001125.mm.1	1,21	Git1	JUC1100005360.mm.1	3,7	0,025662	0,498118		
TC1100001125.mm.1	1,21	Git1	PSR1100010209.mm.1	2,53	0,026686	0,502057	Cassette Exon	0,46
TC1800000019.mm.1	-1,41	Svil	JUC1800000115.mm.1	7,29	0,04463	0,552129		
TC0200003828.mm.1	3	Cerkl; Neurod1	PSR0200033163.mm.1	7,28	0,004232	0,363295	Alternative 5' Donc	0,32
TC0200003828.mm.1	3	Cerkl; Neurod1	PSR0200033160.mm.1	6,26	0,004938	0,373093	Cassette Exon	0,56
TC0200003828.mm.1	3	Cerkl; Neurod1	JUC0200017104.mm.1	5,69	0,003049	0,353892		
TC0200003828.mm.1	3	Cerkl; Neurod1	PSR0200033158.mm.1	5,28	0,029338	0,509981	Cassette Exon	0,56
TC0200003828.mm.1	3	Cerkl; Neurod1	PSR0200033159.mm.1	4,89	0,007272	0,398297	Cassette Exon	0,56
TC0200003828.mm.1	3	Cerkl; Neurod1	PSR0200033156.mm.1	4,44	0,026888	0,502518	Cassette Exon	0,56
TC0200003828.mm.1	3	Cerkl; Neurod1	JUC0200017068.mm.1	2,16	0,009022	0,41262		

TC0200003828.mm.1	3	Cerkl; Neurod1	PSR0200033134.mm.1	2,06	0,001053	0,316361	Alternative 3' Acce	0,15
TC0200003828.mm.1	3	Cerkl; Neurod1	PSR0200033108.mm.1	-2,31	0,007844	0,403342	Cassette Exon	0,12
TC0200003828.mm.1	3	Cerkl; Neurod1	PSR0200033137.mm.1	-2,41	0,043571	0,550033	Cassette Exon	0,19
TC0200003828.mm.1	3	Cerkl; Neurod1	PSR0200033165.mm.1	-2,73	0,010134	0,419217	Alternative 5' Donc	0,31
TC0200003828.mm.1	3	Cerkl; Neurod1	JUC0200017099.mm.1	-2,77	0,033035	0,521501		
TC0200003828.mm.1	3	Cerkl; Neurod1	JUC0200017075.mm.1	-2,88	0,023047	0,489311		
TC0200003828.mm.1	3	Cerkl; Neurod1	PSR0200033148.mm.1	-3,1	0,043629	0,550161	Cassette Exon	0,03
TC0200003828.mm.1	3	Cerkl; Neurod1	PSR0200033166.mm.1	-3,19	0,018299	0,468049	Alternative 5' Donc	0,31
TC0200003828.mm.1	3	Cerkl; Neurod1	PSR0200033154.mm.1	-3,29	0,00072	0,306015	Cassette Exon	0,26
TC0200003828.mm.1	3	Cerkl; Neurod1	JUC0200017094.mm.1	-3,36	0,02271	0,487767		
TC0200003828.mm.1	3	Cerkl; Neurod1	PSR0200033143.mm.1	-3,5	0,003235	0,354243	Cassette Exon	0,25
TC0200003828.mm.1	3	Cerkl; Neurod1	JUC0200017090.mm.1	-3,82	0,005559	0,380773		
TC0200003828.mm.1	3	Cerkl; Neurod1	JUC0200017105.mm.1	-3,91	0,000964	0,313363		
TC0200003828.mm.1	3	Cerkl; Neurod1	JUC0200017071.mm.1	-3,96	0,018123	0,467224		
TC0200003828.mm.1	3	Cerkl; Neurod1	JUC0200017101.mm.1	-4,7	0,005083	0,37538		
TC0200003828.mm.1	3	Cerkl; Neurod1	JUC0200017093.mm.1	-4,78	0,016538	0,459612		
TC0200003828.mm.1	3	Cerkl; Neurod1	JUC0200017092.mm.1	-4,87	0,006089	0,38673		
TC0200003828.mm.1	3	Cerkl; Neurod1	JUC0200017100.mm.1	-4,89	0,000864	0,311909		
TC0200003828.mm.1	3	Cerkl; Neurod1	JUC0200017091.mm.1	-5,01	0,021616	0,483425		
TC0200003828.mm.1	3	Cerkl; Neurod1	JUC0200017098.mm.1	-7,75	0,023318	0,490065		
TC0200000801.mm.1	3,42	Kif5c	JUC0200003361.mm.1	7,28	0,003218	0,354243		
TC0200000801.mm.1	3,42	Kif5c	PSR0200006637.mm.1	3,58	0,024142	0,493245	Cassette Exon	0,27
TC0200000801.mm.1	3,42	Kif5c	JUC0200003350.mm.1	2,58	0,046712	0,556856		
TC0200000801.mm.1	3,42	Kif5c	PSR0200006630.mm.1	2,25	0,021981	0,485125	Cassette Exon	0,15
TC0200000801.mm.1	3,42	Kif5c	JUC0200003360.mm.1	-2,09	0,0102	0,419711		
TC0200000801.mm.1	3,42	Kif5c	JUC0200003343.mm.1	-2,11	0,000374	0,291789		
TC0200000801.mm.1	3,42	Kif5c	PSR0200006658.mm.1	-2,21	0,022674	0,487625	Cassette Exon	0,29
TC0200000801.mm.1	3,42	Kif5c	PSR0200006649.mm.1	-2,36	0,006861	0,394108	Alternative 5' Donc	0,22
TC0200000801.mm.1	3,42	Kif5c	JUC0200003366.mm.1	-2,48	0,024155	0,493245		
TC0200000801.mm.1	3,42	Kif5c	JUC0200003370.mm.1	-2,57	0,030744	0,514065		
TC0200000801.mm.1	3,42	Kif5c	JUC0200003355.mm.1	-2,57	0,017242	0,462729		
TC0200000801.mm.1	3,42	Kif5c	PSR0200006644.mm.1	-2,61	0,002529	0,349501	Alternative 5' Donc	0,22
TC0200000801.mm.1	3,42	Kif5c	PSR0200006651.mm.1	-2,62	0,018584	0,469618	Cassette Exon	0,21
TC0200000801.mm.1	3,42	Kif5c	PSR0200006650.mm.1	-2,88	0,002024	0,341377	Cassette Exon	0,19
TC0200000801.mm.1	3,42	Kif5c	PSR0200006655.mm.1	-2,93	0,005069	0,375225	Cassette Exon	0,31
TC0200000801.mm.1	3,42	Kif5c	JUC0200003365.mm.1	-3,01	0,015467	0,454312		
TC0200000801.mm.1	3,42	Kif5c	PSR0200006625.mm.1	-3,07	0,000573	0,304044	Cassette Exon	0,24
TC0200000801.mm.1	3,42	Kif5c	JUC0200003368.mm.1	-3,2	0,045206	0,553564		
TC0200000801.mm.1	3,42	Kif5c	PSR0200006657.mm.1	-3,48	0,00287	0,352207	Cassette Exon	0,29
TC0200000801.mm.1	3,42	Kif5c	PSR0200006645.mm.1	-3,69	0,000312	0,288663	Alternative 3' Acce	0,43
TC0200000801.mm.1	3,42	Kif5c	PSR0200006626.mm.1	-3,84	0,00615	0,387873	Alternative 3' Acce	0,4
TC0200000801.mm.1	3,42	Kif5c	PSR0200006654.mm.1	-4,07	0,008994	0,41201	Cassette Exon	0,41
TC0200000801.mm.1	3,42	Kif5c	PSR0200006628.mm.1	-4,1	0,000852	0,311909	Alternative 5' Donc	0,47
TC0200000801.mm.1	3,42	Kif5c	JUC0200003371.mm.1	-5,43	0,002266	0,34578		
TC0200000801.mm.1	3,42	Kif5c	JUC0200003369.mm.1	-9,2	0,003264	0,354243		
TC1500001675.mm.1	2,89	Fam135b	JUC1500007092.mm.1	7,28	0,001245	0,322251		
TC1500001675.mm.1	2,89	Fam135b	PSR1500012580.mm.1	2,54	0,00293	0,352289	Cassette Exon	0,04
TC1500001675.mm.1	2,89	Fam135b	JUC1500007091.mm.1	2,1	0,005582	0,381035		

TC1500001675.mm.1	2,89	Fam135b	PSR1500012569.mm.1	2,02	0,040459	0,542132		
TC1500001675.mm.1	2,89	Fam135b	PSR1500012575.mm.1	-2,18	0,00161	0,332831	Cassette Exon	0,12
TC1500001675.mm.1	2,89	Fam135b	PSR1500012585.mm.1	-2,2	0,011322	0,427133	Cassette Exon	0,15
TC1500001675.mm.1	2,89	Fam135b	JUC1500007102.mm.1	-2,45	0,033766	0,523689		
TC1500001675.mm.1	2,89	Fam135b	PSR1500012568.mm.1	-2,52	0,004741	0,370933	Cassette Exon	0,28
TC1500001675.mm.1	2,89	Fam135b	JUC1500007093.mm.1	-2,56	0,034965	0,52724		
TC1500001675.mm.1	2,89	Fam135b	PSR1500012588.mm.1	-2,66	0,005034	0,374447		
TC1500001675.mm.1	2,89	Fam135b	JUC1500007099.mm.1	-3,18	0,032347	0,519174		
TC1500001675.mm.1	2,89	Fam135b	JUC1500007090.mm.1	-3,65	0,015326	0,453725		
TC1500001675.mm.1	2,89	Fam135b	JUC1500007088.mm.1	-6,23	0,003095	0,353892		
TC1000000661.mm.1	1,44	Ank3	JUC1000002510.mm.1	7,27	0,001709	0,335996		
TC1000000661.mm.1	1,44	Ank3	JUC1000002509.mm.1	3,3	0,027874	0,505612		
TC1000000661.mm.1	1,44	Ank3	JUC1000002534.mm.1	2,74	0,021323	0,481727		
TC1000000661.mm.1	1,44	Ank3	PSR1000004551.mm.1	2,53	0,010174	0,419711	Cassette Exon	0,16
TC1000000661.mm.1	1,44	Ank3	JUC1000002521.mm.1	2,44	0,035976	0,529472		
TC1000000661.mm.1	1,44	Ank3	JUC1000002519.mm.1	2,08	0,014899	0,451802		
TC1000000661.mm.1	1,44	Ank3	JUC1000002487.mm.1	2,06	0,02257	0,487325		
TC1000000661.mm.1	1,44	Ank3	PSR1000004583.mm.1	-2,04	0,000272	0,28803	Cassette Exon	0,12
TC1000000661.mm.1	1,44	Ank3	PSR1000004574.mm.1	-2,07	0,010397	0,421447	Cassette Exon	0,19
TC1000000661.mm.1	1,44	Ank3	PSR1000004581.mm.1	-2,07	0,007908	0,403819	Cassette Exon	0,12
TC1000000661.mm.1	1,44	Ank3	JUC1000002506.mm.1	-2,19	0,025407	0,497403		
TC1000000661.mm.1	1,44	Ank3	JUC1000002502.mm.1	-2,24	0,015387	0,453849		
TC1000000661.mm.1	1,44	Ank3	PSR1000004656.mm.1	-2,3	0,004768	0,371039	Alternative 5' Donc	0,21
TC1000000661.mm.1	1,44	Ank3	JUC1000002530.mm.1	-2,57	0,007196	0,397947		
TC1000000661.mm.1	1,44	Ank3	JUC1000002529.mm.1	-3,04	0,012203	0,433268		
TC1000000661.mm.1	1,44	Ank3	JUC1000002503.mm.1	-3,51	0,015008	0,452083		
TC0300000236.mm.1	1,01	Fxr1	JUC0300000752.mm.1	7,26	0,023188	0,490032		
TC1400000804.mm.1	-1,1	Atp12a	JUC1400003486.mm.1	7,21	0,00574	0,382993		
TC0100001812.mm.1	2,94	Ush2a	JUC0100008557.mm.1	7,16	0,000942	0,313363		
TC0100001812.mm.1	2,94	Ush2a	JUC0100008576.mm.1	4,66	0,034418	0,525828		
TC0100001812.mm.1	2,94	Ush2a	JUC0100008558.mm.1	4,5	0,031527	0,517029		
TC0100001812.mm.1	2,94	Ush2a	PSR0100015045.mm.1	4,21	0,026811	0,502373	Cassette Exon	0,43
TC0100001812.mm.1	2,94	Ush2a	JUC0100008594.mm.1	4,12	0,000513	0,303579		
TC0100001812.mm.1	2,94	Ush2a	JUC0100008554.mm.1	4,04	0,001527	0,330287		
TC0100001812.mm.1	2,94	Ush2a	PSR0100015073.mm.1	3,47	0,001319	0,324348	Cassette Exon	0,24
TC0100001812.mm.1	2,94	Ush2a	JUC0100008597.mm.1	3,38	0,016535	0,459612		
TC0100001812.mm.1	2,94	Ush2a	JUC0100008586.mm.1	3,37	0,004867	0,371692		
TC0100001812.mm.1	2,94	Ush2a	PSR0100015049.mm.1	3,35	0,010556	0,422341	Cassette Exon	0,16
TC0100001812.mm.1	2,94	Ush2a	PSR0100015044.mm.1	3,33	0,024328	0,49362	Cassette Exon	0,49
TC0100001812.mm.1	2,94	Ush2a	PSR0100015046.mm.1	3,1	0,047268	0,558223	Cassette Exon	0,23
TC0100001812.mm.1	2,94	Ush2a	PSR0100015033.mm.1	3,09	0,017536	0,464105	Cassette Exon	0,35
TC0100001812.mm.1	2,94	Ush2a	JUC0100008546.mm.1	2,84	0,008871	0,410539		
TC0100001812.mm.1	2,94	Ush2a	PSR0100015037.mm.1	2,82	0,000603	0,304044	Cassette Exon	0,25
TC0100001812.mm.1	2,94	Ush2a	PSR0100015043.mm.1	2,59	0,013072	0,4396	Cassette Exon	0,32
TC0100001812.mm.1	2,94	Ush2a	JUC0100008545.mm.1	2,54	0,037711	0,534583		
TC0100001812.mm.1	2,94	Ush2a	PSR0100015031.mm.1	2,53	0,016809	0,460623	Cassette Exon	0,18
TC0100001812.mm.1	2,94	Ush2a	JUC0100008550.mm.1	2,31	0,023081	0,489691		
TC0100001812.mm.1	2,94	Ush2a	JUC0100008568.mm.1	2,18	0,023616	0,491012		

TC0100001812.mm.1	2,94	Ush2a	PSR0100015042.mm.1	2,08	0,029929	0,511499	Cassette Exon	0,12
TC0100001812.mm.1	2,94	Ush2a	PSR0100015059.mm.1	2,08	0,001461	0,328635	Cassette Exon	0,11
TC0100001812.mm.1	2,94	Ush2a	PSR0100015032.mm.1	2,04	0,008172	0,405603	Cassette Exon	0,21
TC0100001812.mm.1	2,94	Ush2a	PSR0100015066.mm.1	-2,02	0,002844	0,352207	Cassette Exon	0,1
TC0100001812.mm.1	2,94	Ush2a	PSR0100015082.mm.1	-2,13	0,013711	0,444758	Cassette Exon	0,27
TC0100001812.mm.1	2,94	Ush2a	PSR0100015100.mm.1	-2,22	0,031417	0,516531	Cassette Exon	0,14
TC0100001812.mm.1	2,94	Ush2a	PSR0100015089.mm.1	-2,44	0,036259	0,530445	Cassette Exon	0,13
TC0100001812.mm.1	2,94	Ush2a	JUC0100008612.mm.1	-2,5	0,016307	0,458523		
TC0100001812.mm.1	2,94	Ush2a	PSR0100015094.mm.1	-2,56	0,000609	0,304044	Cassette Exon	0,19
TC0100001812.mm.1	2,94	Ush2a	JUC0100008581.mm.1	-2,56	0,034488	0,525929		
TC0100001812.mm.1	2,94	Ush2a	JUC0100008572.mm.1	-2,59	0,001794	0,336311		
TC0100001812.mm.1	2,94	Ush2a	JUC0100008602.mm.1	-2,61	0,018237	0,467691		
TC0100001812.mm.1	2,94	Ush2a	PSR0100015093.mm.1	-2,85	0,000252	0,28803	Cassette Exon	0,23
TC0100001812.mm.1	2,94	Ush2a	PSR0100015102.mm.1	-2,99	0,004205	0,363236	Cassette Exon	0,39
TC0100001812.mm.1	2,94	Ush2a	JUC0100008598.mm.1	-3,03	0,007974	0,403853		
TC0100001812.mm.1	2,94	Ush2a	JUC0100008569.mm.1	-3,15	0,009269	0,414688		
TC0100001812.mm.1	2,94	Ush2a	PSR0100015104.mm.1	-3,24	0,007464	0,400381	Cassette Exon	0,28
TC0100001812.mm.1	2,94	Ush2a	PSR0100015106.mm.1	-3,24	0,012333	0,434392	Alternative 5' Donc	0,26
TC0100001812.mm.1	2,94	Ush2a	JUC0100008543.mm.1	-3,4	0,031756	0,517489		
TC0100001812.mm.1	2,94	Ush2a	PSR0100015050.mm.1	-3,63	0,010577	0,422452	Alternative 5' Donc	0,29
TC0100001812.mm.1	2,94	Ush2a	PSR0100015028.mm.1	-3,74	0,002043	0,341377	Cassette Exon	0,39
TC0100001812.mm.1	2,94	Ush2a	JUC0100008547.mm.1	-3,96	0,002892	0,352207		
TC0100001812.mm.1	2,94	Ush2a	JUC0100008591.mm.1	-4,01	0,045511	0,554359		
TC0100001812.mm.1	2,94	Ush2a	PSR0100015070.mm.1	-4,02	0,004721	0,370888	Alternative 5' Donc	0,35
TC0100001812.mm.1	2,94	Ush2a	JUC0100008593.mm.1	-4,41	0,001884	0,338712		
TC0100001812.mm.1	2,94	Ush2a	JUC0100008564.mm.1	-4,41	0,02436	0,49362		
TC0100001812.mm.1	2,94	Ush2a	JUC0100008582.mm.1	-4,61	0,00604	0,386586		
TC0100001812.mm.1	2,94	Ush2a	PSR0100015098.mm.1	-4,86	0,01802	0,466939	Cassette Exon	0,28
TC0100001812.mm.1	2,94	Ush2a	JUC0100008588.mm.1	-5,94	0,011495	0,428152		
TC0100001812.mm.1	2,94	Ush2a	JUC0100008563.mm.1	-8,24	0,000729	0,306408		
TC0100001812.mm.1	2,94	Ush2a	JUC0100008615.mm.1	-10,91	0,002818	0,352207		
TC0800002595.mm.1	2,14	Mast1; Gm24197	JUC0800010794.mm.1	7,13	0,006628	0,392592		
TC0800002595.mm.1	2,14	Mast1; Gm24197	PSR0800019858.mm.1	5,3	0,002239	0,34578		
TC0800002595.mm.1	2,14	Mast1; Gm24197	JUC0800010811.mm.1	3,25	0,001308	0,323877		
TC0800002595.mm.1	2,14	Mast1; Gm24197	PSR0800019862.mm.1	3	0,005795	0,383104	Cassette Exon	0,45
TC0800002595.mm.1	2,14	Mast1; Gm24197	JUC0800010817.mm.1	2,99	0,00849	0,406966		
TC0800002595.mm.1	2,14	Mast1; Gm24197	JUC0800010799.mm.1	2,58	0,001748	0,335996		
TC0800002595.mm.1	2,14	Mast1; Gm24197	PSR0800019861.mm.1	2,45	0,014793	0,450911	Cassette Exon	0,45
TC0800002595.mm.1	2,14	Mast1; Gm24197	PSR0800019875.mm.1	2,12	0,005604	0,381115		
TC0800002595.mm.1	2,14	Mast1; Gm24197	PSR0800019851.mm.1	-2,02	0,032481	0,519641	Alternative 3' Acce	0,17
TC0800002595.mm.1	2,14	Mast1; Gm24197	PSR0800019892.mm.1	-2,11	0,003236	0,354243	Alternative 3' Acce	0,06
TC0800002595.mm.1	2,14	Mast1; Gm24197	PSR0800019894.mm.1	-2,12	0,008261	0,405976	Alternative 5' Donc	0,18
TC0800002595.mm.1	2,14	Mast1; Gm24197	PSR0800019895.mm.1	-2,21	0,001915	0,339733	Alternative 3' Acce	0,12
TC0800002595.mm.1	2,14	Mast1; Gm24197	JUC0800010801.mm.1	-2,23	0,008614	0,408434		
TC0800002595.mm.1	2,14	Mast1; Gm24197	PSR0800019865.mm.1	-2,25	0,008546	0,408059		
TC0800002595.mm.1	2,14	Mast1; Gm24197	PSR0800019884.mm.1	-2,33	0,019424	0,473956	Alternative 5' Donc	0,02
TC0800002595.mm.1	2,14	Mast1; Gm24197	PSR0800019886.mm.1	-2,49	0,006217	0,387912	Alternative 5' Donc	0,02
TC0800002595.mm.1	2,14	Mast1; Gm24197	PSR0800019855.mm.1	-2,68	0,012059	0,432426	Cassette Exon	0,28

TC0800002595.mm.1	2,14	Mast1; Gm24197	PSR0800019850.mm.1	-2,76	0,001155	0,317344	Alternative 3' Acce	0,17
TC0800002595.mm.1	2,14	Mast1; Gm24197	PSR0800019872.mm.1	-2,92	0,001348	0,325427	Intron Retention	0,44
TC0800002595.mm.1	2,14	Mast1; Gm24197	PSR0800019878.mm.1	-3,05	0,001841	0,337414	Intron Retention	0,49
TC0800002595.mm.1	2,14	Mast1; Gm24197	JUC0800010809.mm.1	-3,14	0,000994	0,316361		
TC0800002595.mm.1	2,14	Mast1; Gm24197	JUC0800010820.mm.1	-3,29	0,001665	0,335182		
TC0800002595.mm.1	2,14	Mast1; Gm24197	JUC0800010815.mm.1	-3,67	0,010407	0,42148		
TC0800002595.mm.1	2,14	Mast1; Gm24197	JUC0800010819.mm.1	-3,86	0,040648	0,54264		
TC0800002595.mm.1	2,14	Mast1; Gm24197	JUC0800010806.mm.1	-5,39	0,000314	0,288663		
TC1700000117.mm.1	-1,24	Agpat4	JUC1700000520.mm.1	7,13	0,000935	0,313363		
TC0900000737.mm.1	1,35	Nptn	JUC0900002867.mm.1	7,12	0,008236	0,405976		
TC0900000737.mm.1	1,35	Nptn	JUC0900002868.mm.1	4,76	0,033438	0,522793		
TC0900000737.mm.1	1,35	Nptn	PSR0900005431.mm.1	3,16	0,033842	0,524033	Cassette Exon	0,48
TC0900000737.mm.1	1,35	Nptn	PSR0900005449.mm.1	-2,05	0,008762	0,409779	Intron Retention	0,26
TC0900000737.mm.1	1,35	Nptn	JUC0900002881.mm.1	-3,17	0,010948	0,425002		
TC1600000374.mm.1	-1,1	Cldn16	JUC1600001849.mm.1	7,12	0,028411	0,507154		
TC1100001072.mm.1	1,63	Slc43a2	JUC1100005138.mm.1	7,07	0,039726	0,540494		
TC0600001429.mm.1	-1,2	Gm20531	JUC0600006081.mm.1	7,04	0,04779	0,559217		
TC1100001830.mm.1	-1,16	Amz2; LOC100503496	PSR1100017240.mm.1	6,99	0,00229	0,34605	Cassette Exon	0,32
TC1100001830.mm.1	-1,16	Amz2; LOC100503496	PSR1100017239.mm.1	3,81	0,004484	0,367949	Cassette Exon	0,33
TC1100001830.mm.1	-1,16	Amz2; LOC100503496	PSR1100017241.mm.1	3,5	0,015656	0,455329	Cassette Exon	0,32
TC1100001830.mm.1	-1,16	Amz2; LOC100503496	PSR1100017225.mm.1	2,08	0,035681	0,529027	Alternative 5' Donc	0,13
TC0700000165.mm.1	4,34	Cabp5	JUC0700000635.mm.1	6,96	0,000233	0,28803		
TC0700000165.mm.1	4,34	Cabp5	JUC0700000636.mm.1	3,64	0,000469	0,298999		
TC0700000165.mm.1	4,34	Cabp5	PSR0700001321.mm.1	-2,41	0,010329	0,42079	Cassette Exon	0,23
TC0700000165.mm.1	4,34	Cabp5	JUC0700000632.mm.1	-3,2	0,037253	0,53345		
TC0700000165.mm.1	4,34	Cabp5	JUC0700000637.mm.1	-9,1	0,009904	0,418332		
TC0300000137.mm.1	1,66	Cp	JUC0300000254.mm.1	6,89	0,021617	0,483425		
TC0300000137.mm.1	1,66	Cp	PSR0300000632.mm.1	-2,14	0,011276	0,426813	Alternative 5' Donc	0,07
TC0300000137.mm.1	1,66	Cp	PSR0300000587.mm.1	-2,26	0,029427	0,509995	Alternative 3' Acce	0,19
TC0300000137.mm.1	1,66	Cp	PSR0300000611.mm.1	-2,87	0,015144	0,452699	Alternative 5' Donc	0,3
TC0300000137.mm.1	1,66	Cp	PSR0300000602.mm.1	-3,46	0,00172	0,335996	Alternative 3' Acce	0,41
TC0500000050.mm.1	1,07	Abcb1a	JUC0500000221.mm.1	6,89	0,024712	0,494838		
TC0500000050.mm.1	1,07	Abcb1a	JUC0500000213.mm.1	3,71	0,006714	0,393343		
TC0500000050.mm.1	1,07	Abcb1a	JUC0500000231.mm.1	2,69	0,017585	0,464264		
TC0500000050.mm.1	1,07	Abcb1a	PSR0500000418.mm.1	2,41	0,045633	0,554672	Cassette Exon	0,31
TC0900000197.mm.1	-1,04	Dnm2	JUC0900000717.mm.1	6,88	0,033494	0,522941		
TC0900000197.mm.1	-1,04	Dnm2	JUC0900000713.mm.1	-2,16	0,008708	0,409126		
TC1900000025.mm.1	1,46	Cabp2	JUC1900000163.mm.1	6,87	0,024878	0,49558		
TC1900000025.mm.1	1,46	Cabp2	PSR1900000316.mm.1	3,18	0,019431	0,473969	Alternative 5' Donc	0,35
TC1900000025.mm.1	1,46	Cabp2	PSR1900000302.mm.1	-2,79	0,003954	0,359922	Cassette Exon	0,19
TC0400000269.mm.1	-1,09	Casp8ap2	JUC0400000763.mm.1	6,85	0,01629	0,458454		
TC1400000359.mm.1	2,7	Gm626	JUC1400001900.mm.1	6,84	0,000044	0,24627		
TC1400000359.mm.1	2,7	Gm626	JUC1400001896.mm.1	5,1	0,023742	0,491602		
TC1400000359.mm.1	2,7	Gm626	PSR1400003196.mm.1	3,65	0,003998	0,361023	Cassette Exon	0,4
TC1400000359.mm.1	2,7	Gm626	JUC1400001897.mm.1	3,44	0,009837	0,417839		
TC1400000359.mm.1	2,7	Gm626	PSR1400003198.mm.1	3,3	0,025022	0,496091	Cassette Exon	0,26
TC1400000359.mm.1	2,7	Gm626	PSR1400003213.mm.1	2,88	0,017346	0,46353	Cassette Exon	0,18
TC1400000359.mm.1	2,7	Gm626	PSR1400003195.mm.1	2,57	0,003575	0,356223	Cassette Exon	0,16

TC1400000359.mm.1	2,7	Gm626	PSR1400003188.mm.1	2,18	0,012136	0,432868		
TC1400000359.mm.1	2,7	Gm626	JUC1400001888.mm.1	2,14	0,040331	0,541841		
TC1400000359.mm.1	2,7	Gm626	PSR1400003194.mm.1	2,13	0,007382	0,39943	Cassette Exon	0,21
TC1400000359.mm.1	2,7	Gm626	JUC1400001917.mm.1	-2,09	0,015597	0,454998		
TC1400000359.mm.1	2,7	Gm626	PSR1400003210.mm.1	-2,31	0,010203	0,419711	Cassette Exon	0,25
TC1400000359.mm.1	2,7	Gm626	JUC1400001893.mm.1	-2,32	0,010369	0,421259		
TC1400000359.mm.1	2,7	Gm626	PSR1400003190.mm.1	-2,39	0,007622	0,401947	Cassette Exon	0,32
TC1400000359.mm.1	2,7	Gm626	PSR1400003189.mm.1	-2,68	0,001727	0,335996	Cassette Exon	0,33
TC1400000359.mm.1	2,7	Gm626	JUC1400001916.mm.1	-3,15	0,016491	0,459391		
TC1400000359.mm.1	2,7	Gm626	PSR1400003208.mm.1	-3,17	0,002457	0,349135	Cassette Exon	0,31
TC1400000359.mm.1	2,7	Gm626	JUC1400001914.mm.1	-3,43	0,008839	0,410293		
TC1400000359.mm.1	2,7	Gm626	JUC1400001892.mm.1	-3,48	0,017667	0,464753		
TC1400000359.mm.1	2,7	Gm626	JUC1400001911.mm.1	-3,73	0,003433	0,355303		
TC1400000359.mm.1	2,7	Gm626	JUC1400001894.mm.1	-3,94	0,042969	0,54838		
TC1400000359.mm.1	2,7	Gm626	JUC1400001889.mm.1	-4,34	0,002776	0,35099		
TC1400000359.mm.1	2,7	Gm626	JUC1400001908.mm.1	-4,87	0,000507	0,303579		
TC0900001213.mm.1	1		PSR0900009570.mm.1	6,84	0,002427	0,349	Cassette Exon	0,29
TC0600001266.mm.1	7,01	Slc6a11	JUC0600005155.mm.1	6,83	0,000112	0,267977		
TC0600001266.mm.1	7,01	Slc6a11	JUC0600005156.mm.1	3,01	0,000943	0,313363		
TC0600001266.mm.1	7,01	Slc6a11	PSR0600009968.mm.1	2,78	0,006393	0,389654	Cassette Exon	0,23
TC0600001266.mm.1	7,01	Slc6a11	JUC0600005149.mm.1	2,67	0,014612	0,449914		
TC0600001266.mm.1	7,01	Slc6a11	PSR0600009960.mm.1	2,45	0,002971	0,352501	Mutually Exclusive	0,25
TC0600001266.mm.1	7,01	Slc6a11	PSR0600009967.mm.1	2,21	0,003884	0,359203	Cassette Exon	0,34
TC0600001266.mm.1	7,01	Slc6a11	PSR0600009958.mm.1	2,01	0,004922	0,373082	Cassette Exon	0,07
TC0600001266.mm.1	7,01	Slc6a11	PSR0600009963.mm.1	-2,06	0,005985	0,386164	Cassette Exon	0,14
TC0600001266.mm.1	7,01	Slc6a11	PSR0600009956.mm.1	-2,26	0,016376	0,458693	Cassette Exon	0,16
TC0600001266.mm.1	7,01	Slc6a11	PSR0600009959.mm.1	-2,34	0,00321	0,354243	Mutually Exclusive	0,25
TC0600001266.mm.1	7,01	Slc6a11	JUC0600005152.mm.1	-2,43	0,008687	0,408815		
TC0600001266.mm.1	7,01	Slc6a11	PSR0600009964.mm.1	-2,89	0,008957	0,411476	Cassette Exon	0,36
TC0600001266.mm.1	7,01	Slc6a11	JUC0600005153.mm.1	-3,68	0,003621	0,356223		
TC0100003271.mm.1	-1,18	Smg7	JUC0100015127.mm.1	6,82	0,005894	0,383835		
TC0100003271.mm.1	-1,18	Smg7	PSR0100026555.mm.1	2,17	0,002602	0,349501	Cassette Exon	0,34
TC0100001237.mm.1	1,24	Syt2	JUC0100005858.mm.1	6,81	0,00076	0,308387		
TC0100001237.mm.1	1,24	Syt2	JUC0100005853.mm.1	-3,14	0,024104	0,493195		
TC0100001237.mm.1	1,24	Syt2	JUC0100005855.mm.1	-3,19	0,010204	0,419711		
TC0400002220.mm.1	1,35	Tmem67	JUC0400009707.mm.1	6,79	0,009907	0,418378		
TC0400002220.mm.1	1,35	Tmem67	JUC0400009691.mm.1	-2,14	0,00983	0,417839		
TC0700004661.mm.1	1,09	Prrt2; Pagr1a	JUC0700019087.mm.1	6,77	0,004357	0,365487		
TC0700004661.mm.1	1,09	Prrt2; Pagr1a	JUC0700019088.mm.1	4,94	0,012766	0,437119		
TC1000002256.mm.1	2,39	Hk1	JUC1000008784.mm.1	6,75	0,002876	0,352207		
TC1000002256.mm.1	2,39	Hk1	JUC1000008767.mm.1	3,44	0,018258	0,467907		
TC1000002256.mm.1	2,39	Hk1	PSR1000016012.mm.1	-2,26	0,00381	0,358103	Alternative 3' Acce	0,22
TC1000002256.mm.1	2,39	Hk1	PSR1000016044.mm.1	-2,29	0,020882	0,479589	Cassette Exon	0,28
TC1000002256.mm.1	2,39	Hk1	PSR1000016001.mm.1	-2,32	0,00168	0,335182	Alternative 3' Acce	0,19
TC1000002256.mm.1	2,39	Hk1	PSR1000016038.mm.1	-2,55	0,013697	0,44468	Cassette Exon	0,14
TC1000002256.mm.1	2,39	Hk1	PSR1000016042.mm.1	-2,59	0,008326	0,406313		
TC1000002256.mm.1	2,39	Hk1	PSR1000016041.mm.1	-2,84	0,002339	0,347265	Cassette Exon	0,2
TC1000002256.mm.1	2,39	Hk1	JUC1000008786.mm.1	-2,88	0,015101	0,452453		

TC1000002256.mm.1	2,39	Hk1	PSR1000016034.mm.1	-2,99	0,000677	0,304338	Alternative 5' Donc	0,19
TC1000002256.mm.1	2,39	Hk1	PSR1000016050.mm.1	-3,03	0,000135	0,270905	Cassette Exon	0,32
TC1000002256.mm.1	2,39	Hk1	PSR1000016048.mm.1	-3,13	0,003007	0,353447	Alternative 5' Donc	0,32
TC1000002256.mm.1	2,39	Hk1	PSR1000016045.mm.1	-3,22	0,000357	0,290838	Cassette Exon	0,28
TC1000002256.mm.1	2,39	Hk1	PSR1000016031.mm.1	-3,27	0,00649	0,39078	Cassette Exon	0,41
TC1000002256.mm.1	2,39	Hk1	PSR1000016035.mm.1	-3,29	0,001328	0,325252	Cassette Exon	0,21
TC1000002256.mm.1	2,39	Hk1	JUC1000008794.mm.1	-3,61	0,000713	0,306015		
TC1000002256.mm.1	2,39	Hk1	JUC1000008793.mm.1	-4,01	0,000651	0,304044		
TC1000002256.mm.1	2,39	Hk1	JUC1000008787.mm.1	-4,2	0,0081	0,405071		
TC1000002256.mm.1	2,39	Hk1	PSR1000016040.mm.1	-4,66	0,008562	0,40821	Alternative 5' Donc	0,58
TC1000002256.mm.1	2,39	Hk1	JUC1000008776.mm.1	-4,76	0,007419	0,399901		
TC1000002256.mm.1	2,39	Hk1	JUC1000008781.mm.1	-5,94	0,000476	0,298999		
TC1000002256.mm.1	2,39	Hk1	JUC1000008788.mm.1	-6,17	0,000075	0,251428		
TC1400000076.mm.1	-1,51	Pxk	JUC1400000439.mm.1	6,74	0,002165	0,344238		
TC1400000076.mm.1	-1,51	Pxk	PSR1400000712.mm.1	4,29	0,012713	0,436535	Cassette Exon	0,47
TC1400000076.mm.1	-1,51	Pxk	PSR1400000690.mm.1	2,04	0,011098	0,426	Cassette Exon	0,14
TC0500002919.mm.1	-1,27	Tgfbr3	JUC0500014070.mm.1	6,74	0,012118	0,432777		
TC0500002028.mm.1	1,31	Reln	JUC0500010057.mm.1	6,72	0,018156	0,467427		
TC0500002028.mm.1	1,31	Reln	PSR0500018462.mm.1	-2,02	0,002541	0,349501	Cassette Exon	0,15
TC0500002028.mm.1	1,31	Reln	JUC0500010088.mm.1	-2,18	0,016171	0,457969		
TC0500002028.mm.1	1,31	Reln	JUC0500010050.mm.1	-2,3	0,035144	0,527898		
TC0500002028.mm.1	1,31	Reln	JUC0500010048.mm.1	-2,38	0,018957	0,471398		
TC0500002028.mm.1	1,31	Reln	JUC0500010068.mm.1	-2,52	0,028648	0,507908		
TC0500002028.mm.1	1,31	Reln	JUC0500010116.mm.1	-2,62	0,009755	0,417839		
TC0500002028.mm.1	1,31	Reln	PSR0500018451.mm.1	-2,78	0,035688	0,529027	Alternative 3' Acce	0,09
TC0500002028.mm.1	1,31	Reln	PSR0500018454.mm.1	-2,9	0,017914	0,466466	Alternative 3' Acce	0,33
TC0500002028.mm.1	1,31	Reln	PSR0500018489.mm.1	-3,57	0,011312	0,427133	Alternative 3' Acce	0,28
TC0600003537.mm.1	-1,17	Clec9a	JUC0600006586.mm.1	6,7	0,00892	0,410901		
TC0100003604.mm.1	2,98	Rgs7	JUC0100016856.mm.1	6,69	0,004207	0,363236		
TC0100003604.mm.1	2,98	Rgs7	PSR0100029645.mm.1	6,05	0,000215	0,28803	Cassette Exon	0,44
TC0100003604.mm.1	2,98	Rgs7	JUC0100016858.mm.1	4,86	0,000132	0,270905		
TC0100003604.mm.1	2,98	Rgs7	PSR0100029647.mm.1	4,52	0,004439	0,367041	Alternative 5' Donc	0,45
TC0100003604.mm.1	2,98	Rgs7	JUC0100016857.mm.1	3,88	0,004322	0,364614		
TC0100003604.mm.1	2,98	Rgs7	PSR0100029643.mm.1	3,84	0,000951	0,313363	Cassette Exon	0,53
TC0100003604.mm.1	2,98	Rgs7	PSR0100029642.mm.1	3,68	0,006258	0,388622	Cassette Exon	0,4
TC0100003604.mm.1	2,98	Rgs7	PSR0100029640.mm.1	3,6	0,01133	0,427232	Cassette Exon	0,31
TC0100003604.mm.1	2,98	Rgs7	PSR0100029641.mm.1	2,77	0,003475	0,355628	Cassette Exon	0,24
TC0100003604.mm.1	2,98	Rgs7	PSR0100029624.mm.1	2,65	0,008425	0,406611	Cassette Exon	0,19
TC0100003604.mm.1	2,98	Rgs7	PSR0100029638.mm.1	2,64	0,013939	0,446202	Cassette Exon	0,18
TC0100003604.mm.1	2,98	Rgs7	PSR0100029634.mm.1	2,56	0,000103	0,262646	Cassette Exon	0,14
TC0100003604.mm.1	2,98	Rgs7	PSR0100029629.mm.1	2,54	0,005115	0,376246	Cassette Exon	0,24
TC0100003604.mm.1	2,98	Rgs7	PSR0100029639.mm.1	2,42	0,00142	0,327268	Cassette Exon	0,14
TC0100003604.mm.1	2,98	Rgs7	JUC0100016854.mm.1	2,42	0,004664	0,370181		
TC0100003604.mm.1	2,98	Rgs7	PSR0100029623.mm.1	2,19	0,003326	0,354243	Cassette Exon	0,17
TC0100003604.mm.1	2,98	Rgs7	JUC0100016850.mm.1	2,15	0,046313	0,556186		
TC0100003604.mm.1	2,98	Rgs7	PSR0100029648.mm.1	-2,64	0,033153	0,521956	Cassette Exon	0,18
TC0100003604.mm.1	2,98	Rgs7	JUC0100016842.mm.1	-2,82	0,011835	0,43053		
TC0100003604.mm.1	2,98	Rgs7	PSR0100029625.mm.1	-3,01	0,023242	0,490032	Cassette Exon	0,19

TC0100003604.mm.1	2,98	Rgs7	PSR0100029628.mm.1	-3,19	0,013251	0,441416	Intron Retention	0,47
TC0100003604.mm.1	2,98	Rgs7	JUC0100016846.mm.1	-3,53	0,016396	0,458812		
TC0500003012.mm.1	1,14	Miat	PSR0500026693.mm.1	6,69	0,049282	0,562691	Alternative 3' Acce	0,48
TC0300001280.mm.1	-1,18	LOC100862268; Gm16958	JUC0300005444.mm.1	6,69	0,016402	0,458894		
TC1000000224.mm.1	4,44	Epb4.1l2	JUC1000000871.mm.1	6,68	0,002856	0,352207		
TC1000000224.mm.1	4,44	Epb4.1l2	JUC1000000860.mm.1	5,3	0,000073	0,251428		
TC1000000224.mm.1	4,44	Epb4.1l2	PSR1000001627.mm.1	-2,1	0,00538	0,378761	Cassette Exon	0,24
TC1000000224.mm.1	4,44	Epb4.1l2	PSR1000001603.mm.1	-2,15	0,033574	0,523178	Cassette Exon	0,15
TC1000000224.mm.1	4,44	Epb4.1l2	PSR1000001624.mm.1	-2,37	0,002322	0,3467	Cassette Exon	0,29
TC1000000224.mm.1	4,44	Epb4.1l2	PSR1000001614.mm.1	-2,38	0,032703	0,520439	Cassette Exon	0,2
TC1000000224.mm.1	4,44	Epb4.1l2	PSR1000001598.mm.1	-2,62	0,008713	0,409266	Cassette Exon	0,09
TC1000000224.mm.1	4,44	Epb4.1l2	JUC1000000870.mm.1	-2,95	0,001482	0,329081		
TC1000000224.mm.1	4,44	Epb4.1l2	JUC1000000850.mm.1	-3,15	0,036035	0,529611		
TC1000000224.mm.1	4,44	Epb4.1l2	JUC1000000878.mm.1	-3,52	0,044133	0,551024		
TC1000000224.mm.1	4,44	Epb4.1l2	PSR1000001597.mm.1	-3,57	0,017764	0,465437	Cassette Exon	0,33
TC1000000224.mm.1	4,44	Epb4.1l2	JUC1000000875.mm.1	-3,57	0,048021	0,559606		
TC1000000224.mm.1	4,44	Epb4.1l2	PSR1000001612.mm.1	-3,67	0,030639	0,513746	Cassette Exon	0,34
TC1000000224.mm.1	4,44	Epb4.1l2	JUC1000000865.mm.1	-3,67	0,001681	0,335182		
TC1000000224.mm.1	4,44	Epb4.1l2	JUC1000000868.mm.1	-3,9	0,021431	0,482126		
TC1000000224.mm.1	4,44	Epb4.1l2	JUC1000000864.mm.1	-5,16	0,002288	0,34605		
TC1000000224.mm.1	4,44	Epb4.1l2	JUC1000000879.mm.1	-5,58	0,002886	0,352207		
TC1000000224.mm.1	4,44	Epb4.1l2	PSR1000001623.mm.1	-5,83	0,00381	0,358103	Alternative 5' Donc	0,49
TC1000000224.mm.1	4,44	Epb4.1l2	JUC1000000863.mm.1	-6,9	0,01088	0,424197		
TC1000000224.mm.1	4,44	Epb4.1l2	JUC1000000861.mm.1	-7,64	0,032287	0,519116		
TC1000000224.mm.1	4,44	Epb4.1l2	JUC1000000862.mm.1	-10,83	0,009585	0,417183		
TC0500002559.mm.1	2,14	Gabra2	PSR0500022648.mm.1	6,67	0,003478	0,355638	Alternative 3' Acce	0,42
TC0500002559.mm.1	2,14	Gabra2	PSR0500022660.mm.1	-2,6	0,002044	0,341377	Cassette Exon	0,13
TC0500002559.mm.1	2,14	Gabra2	JUC0500012304.mm.1	-2,66	0,016055	0,457283		
TC1400001067.mm.1	-1,08	Nufip1	JUC1400004584.mm.1	6,65	0,007201	0,398011		
TC0900002401.mm.1	-1,12	Neo1	JUC0900011383.mm.1	6,63	0,023527	0,490947		
TC0900002401.mm.1	-1,12	Neo1	PSR0900020177.mm.1	3,21	0,017705	0,465159	Cassette Exon	0,36
TC0900002401.mm.1	-1,12	Neo1	JUC0900011393.mm.1	2,11	0,021558	0,483034		
TC0900001800.mm.1	-1,82	Naalad2	JUC0900008516.mm.1	6,63	0,011874	0,431125		
TC0900001800.mm.1	-1,82	Naalad2	PSR0900015164.mm.1	2,02	0,006915	0,394944	Alternative 5' Donc	0,06
TC0100000356.mm.1	1,25	Dnah7c	JUC0100001628.mm.1	6,6	0,009772	0,417839		
TC0100000356.mm.1	1,25	Dnah7c	JUC0100001583.mm.1	5,33	0,017396	0,463736		
TC0100000356.mm.1	1,25	Dnah7c	JUC0100001624.mm.1	4,55	0,018587	0,469618		
TC0100000356.mm.1	1,25	Dnah7c	JUC0100001584.mm.1	3,79	0,036931	0,532349		
TC0100000356.mm.1	1,25	Dnah7c	JUC0100001619.mm.1	2,99	0,042371	0,5475		
TC0100000356.mm.1	1,25	Dnah7c	PSR0100002757.mm.1	2,77	0,047341	0,558291	Cassette Exon	0,23
TC0100000356.mm.1	1,25	Dnah7c	JUC0100001631.mm.1	2,74	0,047169	0,558007		
TC0100000356.mm.1	1,25	Dnah7c	JUC0100001618.mm.1	2,65	0,020505	0,478228		
TC0100000356.mm.1	1,25	Dnah7c	JUC0100001606.mm.1	2,61	0,003064	0,353892		
TC0100000356.mm.1	1,25	Dnah7c	PSR0100002750.mm.1	2,5	0,014771	0,450801	Cassette Exon	0,18
TC0100000356.mm.1	1,25	Dnah7c	JUC0100001597.mm.1	2,42	0,029252	0,509776		
TC0100000356.mm.1	1,25	Dnah7c	PSR0100002782.mm.1	2,41	0,027184	0,503342	Cassette Exon	0,23
TC0100000356.mm.1	1,25	Dnah7c	JUC0100001607.mm.1	2,28	0,040355	0,541949		
TC0100000356.mm.1	1,25	Dnah7c	JUC0100001603.mm.1	2,16	0,049165	0,562368		

TC0100000356.mm.1	1,25	Dnah7c	PSR0100002741.mm.1	2,14	0,0178	0,465641	Cassette Exon	0,11
TC0100000356.mm.1	1,25	Dnah7c	JUC0100001573.mm.1	-2,13	0,005539	0,380603		
TC1500002350.mm.1	-1,14	Cpt1b	JUC1500009414.mm.1	6,59	0,02087	0,479521		
TC1100000093.mm.1	-1,13	Ogdh	JUC1100000517.mm.1	6,58	0,003033	0,353543		
TC1100000093.mm.1	-1,13	Ogdh	JUC1100000515.mm.1	5,03	0,012367	0,43488		
TC1100000093.mm.1	-1,13	Ogdh	PSR1100000996.mm.1	-2,04	0,038806	0,537594	Alternative 5' Donc	0,15
TC1000000365.mm.1	-1	Mical1	JUC1000001377.mm.1	6,58	0,026095	0,499793		
TC1100003044.mm.1	-1,08	Dnah2	JUC1100014552.mm.1	6,57	0,019566	0,474636		
TC1100003044.mm.1	-1,08	Dnah2	JUC1100014595.mm.1	2,42	0,023357	0,490143		
TC0100002067.mm.1	1,08	Smap1	JUC0100009559.mm.1	6,56	0,046413	0,556519		
TC1100004102.mm.1	1,43	Wbp2	JUC1100020266.mm.1	6,55	0,007288	0,398787		
TC0600001352.mm.1	1,83	Cecr2	PSR0600010782.mm.1	6,52	0,000298	0,288663	Alternative 5' Donc	0,56
TC0600001352.mm.1	1,83	Cecr2	JUC0600005617.mm.1	3,99	0,023099	0,489699		
TC0600001352.mm.1	1,83	Cecr2	PSR0600010783.mm.1	2,71	0,007335	0,399002	Cassette Exon	0,25
TC0600001352.mm.1	1,83	Cecr2	PSR0600010768.mm.1	2,62	0,000972	0,314287	Cassette Exon	0,21
TC0600001352.mm.1	1,83	Cecr2	PSR0600010769.mm.1	2,34	0,023801	0,491893	Alternative 3' Acce	0,21
TC0600001352.mm.1	1,83	Cecr2	JUC0600005610.mm.1	2,25	0,00052	0,303623		
TC0600001352.mm.1	1,83	Cecr2	JUC0600005611.mm.1	2,22	0,003703	0,357266		
TC0600001352.mm.1	1,83	Cecr2	PSR0600010779.mm.1	2,19	0,04446	0,551693	Cassette Exon	0,13
TC0600001352.mm.1	1,83	Cecr2	PSR0600010790.mm.1	2,07	0,036094	0,529729	Cassette Exon	0,1
TC0600001352.mm.1	1,83	Cecr2	PSR0600010762.mm.1	-2,02	0,009936	0,41841	Cassette Exon	0,08
TC0600001352.mm.1	1,83	Cecr2	JUC0600005622.mm.1	-3,84	0,000778	0,309451		
TC0600001352.mm.1	1,83	Cecr2	JUC0600005616.mm.1	-6,98	0,000577	0,304044		
TC1000001066.mm.1	-2,2	Igf1	PSR1000008067.mm.1	6,52	0,001712	0,335996	Cassette Exon	0,27
TC1000001066.mm.1	-2,2	Igf1	PSR1000008079.mm.1	2,91	0,01688	0,460797	Alternative 5' Donc	0,3
TC1000001066.mm.1	-2,2	Igf1	PSR1000008074.mm.1	2,41	0,001785	0,336311	Alternative 5' Donc	0,19
TC1000001066.mm.1	-2,2	Igf1	PSR1000008070.mm.1	2,28	0,014102	0,447108	Alternative 5' Donc	0,13
TC0700001382.mm.1	1,88	Map6	JUC0700006057.mm.1	6,52	0,008038	0,40434		
TC0700001382.mm.1	1,88	Map6	PSR0700011706.mm.1	-2,14	0,005375	0,378747	Alternative 3' Acce	0,13
TC0700001382.mm.1	1,88	Map6	PSR0700011711.mm.1	-2,14	0,004724	0,370888	Alternative 5' Donc	0,13
TC1000003066.mm.1	1,02	Mon2	JUC1000012561.mm.1	6,5	0,012627	0,436053		
TC1000003066.mm.1	1,02	Mon2	JUC1000012560.mm.1	3,68	0,01239	0,434939		
TC1700001661.mm.1	2,51	Cacna1h	JUC1700008246.mm.1	6,49	0,013994	0,446353		
TC1700001661.mm.1	2,51	Cacna1h	JUC1700008232.mm.1	4,07	0,013978	0,446242		
TC1700001661.mm.1	2,51	Cacna1h	JUC1700008252.mm.1	3,18	0,003948	0,359922		
TC1700001661.mm.1	2,51	Cacna1h	JUC1700008251.mm.1	3,06	0,002041	0,341377		
TC1700001661.mm.1	2,51	Cacna1h	PSR1700015128.mm.1	2,48	0,012072	0,43255	Cassette Exon	0,09
TC1700001661.mm.1	2,51	Cacna1h	PSR1700015154.mm.1	2,41	0,022137	0,48556	Cassette Exon	0,26
TC1700001661.mm.1	2,51	Cacna1h	JUC1700008224.mm.1	2,41	0,010044	0,418873		
TC1700001661.mm.1	2,51	Cacna1h	JUC1700008248.mm.1	2,37	0,010443	0,421595		
TC1700001661.mm.1	2,51	Cacna1h	PSR1700015155.mm.1	2,36	0,040152	0,541609	Cassette Exon	0,26
TC1700001661.mm.1	2,51	Cacna1h	JUC1700008241.mm.1	2,29	0,031569	0,51716		
TC1700001661.mm.1	2,51	Cacna1h	JUC1700008243.mm.1	2,18	0,036087	0,529712		
TC1700001661.mm.1	2,51	Cacna1h	PSR1700015141.mm.1	2,03	0,003165	0,353901	Cassette Exon	0,15
TC1700001661.mm.1	2,51	Cacna1h	PSR1700015135.mm.1	-2,03	0,000991	0,316265	Cassette Exon	0,24
TC1700001661.mm.1	2,51	Cacna1h	PSR1700015144.mm.1	-2,17	0,008874	0,410574	Cassette Exon	0,09
TC1700001661.mm.1	2,51	Cacna1h	PSR1700015157.mm.1	-2,2	0,034025	0,524517		
TC1700001661.mm.1	2,51	Cacna1h	JUC1700008235.mm.1	-2,24	0,046836	0,557144		

TC1700001661.mm.1	2,51	Cacna1h	PSR1700015148.mm.1	-2,26	0,008092	0,405014	Alternative 3' Acce	0,17
TC1700001661.mm.1	2,51	Cacna1h	JUC1700008229.mm.1	-2,34	0,022379	0,486433		
TC1700001661.mm.1	2,51	Cacna1h	PSR1700015138.mm.1	-2,46	0,005578	0,381035	Cassette Exon	0,17
TC1700001661.mm.1	2,51	Cacna1h	PSR1700015124.mm.1	-2,52	0,043146	0,548676	Cassette Exon	0,14
TC1700001661.mm.1	2,51	Cacna1h	PSR1700015112.mm.1	-2,6	0,00481	0,371408	Cassette Exon	0,15
TC1700001661.mm.1	2,51	Cacna1h	PSR1700015139.mm.1	-2,65	0,000066	0,250119	Cassette Exon	0,11
TC1700001661.mm.1	2,51	Cacna1h	JUC1700008254.mm.1	-2,66	0,008159	0,40551		
TC1700001661.mm.1	2,51	Cacna1h	PSR1700015143.mm.1	-2,77	0,004298	0,364027	Alternative 3' Acce	0,09
TC1700001661.mm.1	2,51	Cacna1h	JUC1700008234.mm.1	-2,92	0,010254	0,420147		
TC1700001661.mm.1	2,51	Cacna1h	PSR1700015159.mm.1	-2,96	0,004693	0,370481		
TC1700001661.mm.1	2,51	Cacna1h	PSR1700015160.mm.1	-2,99	0,001797	0,336311	Cassette Exon	0,18
TC1700001661.mm.1	2,51	Cacna1h	PSR1700015158.mm.1	-3,13	0,002279	0,345991	Cassette Exon	0,1
TC1700001661.mm.1	2,51	Cacna1h	PSR1700015122.mm.1	-3,33	0,00035	0,290082	Alternative 5' Donc	0,38
TC1700001661.mm.1	2,51	Cacna1h	JUC1700008230.mm.1	-3,59	0,005486	0,379939		
TC1400000783.mm.1	-1,59	Fitm1	JUC1400003333.mm.1	6,48	0,01947	0,474209		
TC1300002041.mm.1	3,09	Sncb	JUC1300006599.mm.1	6,47	0,000468	0,298999		
TC1300002041.mm.1	3,09	Sncb	PSR1300012807.mm.1	3,94	0,002011	0,341091	Cassette Exon	0,39
TC1300002041.mm.1	3,09	Sncb	PSR1300012806.mm.1	2,04	0,008531	0,407721	Cassette Exon	0,11
TC1300002041.mm.1	3,09	Sncb	PSR1300012804.mm.1	-2,22	0,000932	0,313363	Cassette Exon	0,09
TC1300002041.mm.1	3,09	Sncb	PSR1300012813.mm.1	-2,34	0,020592	0,478531	Cassette Exon	0,16
TC1300002041.mm.1	3,09	Sncb	PSR1300012812.mm.1	-2,98	0,003358	0,354243	Cassette Exon	0,18
TC1300002041.mm.1	3,09	Sncb	PSR1300012814.mm.1	-3,01	0,011935	0,431703	Cassette Exon	0,16
TC1300002041.mm.1	3,09	Sncb	PSR1300012808.mm.1	-3,02	0,002894	0,352207	Alternative 5' Donc	0,32
TC1300002041.mm.1	3,09	Sncb	PSR1300012811.mm.1	-3,39	0,007047	0,396259	Cassette Exon	0,29
TC0200005430.mm.1	3,23	Kcnq2	JUC0200023843.mm.1	6,45	0,003815	0,35811		
TC0200005430.mm.1	3,23	Kcnq2	PSR0200046279.mm.1	2,62	0,003726	0,357586	Cassette Exon	0,24
TC0200005430.mm.1	3,23	Kcnq2	PSR0200046261.mm.1	-2,22	0,011021	0,425624	Cassette Exon	0,38
TC0200005430.mm.1	3,23	Kcnq2	PSR0200046268.mm.1	-2,25	0,009506	0,416602	Cassette Exon	0,41
TC0200005430.mm.1	3,23	Kcnq2	PSR0200046246.mm.1	-2,27	0,000031	0,235203	Alternative 5' Donc	0,04
TC0200005430.mm.1	3,23	Kcnq2	PSR0200046284.mm.1	-2,34	0,001434	0,327493	Alternative 5' Donc	0,37
TC0200005430.mm.1	3,23	Kcnq2	PSR0200046240.mm.1	-2,39	0,020403	0,477881	Alternative 3' Acce	0,21
TC0200005430.mm.1	3,23	Kcnq2	PSR0200046239.mm.1	-2,45	0,009142	0,413447	Alternative 3' Acce	0,21
TC0200005430.mm.1	3,23	Kcnq2	PSR0200046238.mm.1	-2,58	0,007578	0,40171	Cassette Exon	0,28
TC0200005430.mm.1	3,23	Kcnq2	PSR0200046265.mm.1	-2,59	0,006448	0,390283	Cassette Exon	0,28
TC0200005430.mm.1	3,23	Kcnq2	JUC0200023853.mm.1	-2,66	0,016216	0,458312		
TC0200005430.mm.1	3,23	Kcnq2	JUC0200023836.mm.1	-2,78	0,033123	0,52188		
TC0200005430.mm.1	3,23	Kcnq2	PSR0200046242.mm.1	-2,8	0,001748	0,335996	Alternative 3' Acce	0,09
TC0200005430.mm.1	3,23	Kcnq2	PSR0200046267.mm.1	-3,01	0,006126	0,387351	Cassette Exon	0,29
TC0200005430.mm.1	3,23	Kcnq2	PSR0200046277.mm.1	-3,08	0,000392	0,296934	Intron Retention	0,52
TC0200005430.mm.1	3,23	Kcnq2	PSR0200046253.mm.1	-3,42	0,002549	0,349501	Alternative 3' Acce	0,49
TC0200005430.mm.1	3,23	Kcnq2	PSR0200046292.mm.1	-3,42	0,018823	0,470836	Cassette Exon	0,23
TC0200005430.mm.1	3,23	Kcnq2	PSR0200046254.mm.1	-3,44	0,006267	0,388719	Alternative 3' Acce	0,51
TC0200005430.mm.1	3,23	Kcnq2	PSR0200046286.mm.1	-3,46	0,01337	0,442252	Cassette Exon	0,23
TC0200005430.mm.1	3,23	Kcnq2	PSR0200046250.mm.1	-3,52	0,008461	0,406624	Cassette Exon	0,29
TC0200005430.mm.1	3,23	Kcnq2	PSR0200046283.mm.1	-3,56	0,031481	0,516965	Alternative 5' Donc	0,37
TC0200005430.mm.1	3,23	Kcnq2	PSR0200046285.mm.1	-3,67	0,002494	0,349135	Cassette Exon	0,25
TC0200005430.mm.1	3,23	Kcnq2	PSR0200046266.mm.1	-3,75	0,001958	0,340561	Cassette Exon	0,28
TC0200005430.mm.1	3,23	Kcnq2	PSR0200046249.mm.1	-3,81	0,001043	0,316361	Cassette Exon	0,38

TC0200005430.mm.1	3,23	Kcnq2	PSR0200046243.mm.1	-3,81	0,001388	0,325997	Alternative 3' Acce	0,09
TC0200005430.mm.1	3,23	Kcnq2	PSR0200046248.mm.1	-3,85	0,004648	0,370181	Alternative 5' Donc	0,04
TC0200005430.mm.1	3,23	Kcnq2	JUC0200023833.mm.1	-3,92	0,008628	0,408463		
TC0200005430.mm.1	3,23	Kcnq2	PSR0200046282.mm.1	-4,06	0,005534	0,380557	Intron Retention	0,43
TC0200005430.mm.1	3,23	Kcnq2	JUC0200023854.mm.1	-4,11	0,007987	0,403853		
TC0200005430.mm.1	3,23	Kcnq2	JUC0200023856.mm.1	-4,18	0,014697	0,450327		
TC0200005430.mm.1	3,23	Kcnq2	PSR0200046263.mm.1	-4,21	0,004149	0,3623	Cassette Exon	0,1
TC0200005430.mm.1	3,23	Kcnq2	PSR0200046272.mm.1	-4,41	0,000637	0,304044	Alternative 3' Acce	0,34
TC0200005430.mm.1	3,23	Kcnq2	JUC0200023832.mm.1	-4,46	0,011228	0,426718		
TC0200005430.mm.1	3,23	Kcnq2	PSR0200046264.mm.1	-4,51	0,004433	0,366934	Cassette Exon	0,38
TC0200005430.mm.1	3,23	Kcnq2	JUC0200023849.mm.1	-4,56	0,000923	0,313363		
TC0200005430.mm.1	3,23	Kcnq2	JUC0200023837.mm.1	-4,72	0,005632	0,381532		
TC0200005430.mm.1	3,23	Kcnq2	PSR0200046256.mm.1	-4,8	0,002234	0,34578	Alternative 3' Acce	0,54
TC0200005430.mm.1	3,23	Kcnq2	PSR0200046269.mm.1	-4,87	0,005178	0,376851	Cassette Exon	0,41
TC0200005430.mm.1	3,23	Kcnq2	JUC0200023839.mm.1	-4,88	0,000179	0,283791		
TC0200005430.mm.1	3,23	Kcnq2	PSR0200046255.mm.1	-4,99	0,000529	0,304044	Alternative 3' Acce	0,54
TC0200005430.mm.1	3,23	Kcnq2	JUC0200023846.mm.1	-5,15	0,007766	0,402774		
TC0200005430.mm.1	3,23	Kcnq2	JUC0200023855.mm.1	-5,3	0,007655	0,402288		
TC0200005430.mm.1	3,23	Kcnq2	PSR0200046273.mm.1	-5,82	0,003369	0,354278	Intron Retention	0,38
TC1100003297.mm.1	1,73	Sarm1	PSR1100030394.mm.1	6,45	0,016136	0,457846	Cassette Exon	0,38
TC1100003297.mm.1	1,73	Sarm1	JUC1100015905.mm.1	6,42	0,020435	0,478018		
TC1100003297.mm.1	1,73	Sarm1	JUC1100015910.mm.1	4,4	0,035525	0,528764		
TC1100003297.mm.1	1,73	Sarm1	JUC1100015904.mm.1	4,35	0,007073	0,39672		
TC1100003297.mm.1	1,73	Sarm1	PSR1100030395.mm.1	3,94	0,02538	0,497403	Cassette Exon	0,38
TC1100003297.mm.1	1,73	Sarm1	PSR1100030405.mm.1	3,19	0,017312	0,463282	Cassette Exon	0,13
TC1100003297.mm.1	1,73	Sarm1	JUC1100015912.mm.1	3,14	0,011832	0,43053		
TC1100003297.mm.1	1,73	Sarm1	PSR1100030396.mm.1	2,86	0,031466	0,516906	Cassette Exon	0,38
TC1100003297.mm.1	1,73	Sarm1	JUC1100015909.mm.1	-2,6	0,009838	0,417839		
TC0200000004.mm.1	-1,1	Nmt2	JUC0200000015.mm.1	6,45	0,027377	0,503961		
TC1200001843.mm.1	2,99	Mdga2	JUC1200006944.mm.1	6,44	0,001369	0,32572		
TC1200001843.mm.1	2,99	Mdga2	PSR1200012569.mm.1	4,21	0,000274	0,28803	Cassette Exon	0,27
TC1200001843.mm.1	2,99	Mdga2	PSR1200012559.mm.1	3,82	0,000428	0,297771	Cassette Exon	0,36
TC1200001843.mm.1	2,99	Mdga2	JUC1200006945.mm.1	3,11	0,019046	0,471941		
TC1200001843.mm.1	2,99	Mdga2	PSR1200012561.mm.1	2,46	0,000419	0,297771	Cassette Exon	0,1
TC1200001843.mm.1	2,99	Mdga2	PSR1200012558.mm.1	2,33	0,00309	0,353892	Cassette Exon	0,35
TC1200001843.mm.1	2,99	Mdga2	PSR1200012563.mm.1	2,22	0,009241	0,414373	Cassette Exon	0,03
TC1200001843.mm.1	2,99	Mdga2	PSR1200012553.mm.1	-2,9	0,011295	0,427117	Alternative 3' Acce	0,3
TC1200001843.mm.1	2,99	Mdga2	PSR1200012582.mm.1	-3,05	0,024287	0,49362	Alternative 5' Donc	0,23
TC1200001843.mm.1	2,99	Mdga2	JUC1200006950.mm.1	-3,15	0,022138	0,48556		
TC1200001843.mm.1	2,99	Mdga2	JUC1200006957.mm.1	-4,27	0,040658	0,542675		
TC0900001114.mm.1	1,61	Dopey1	JUC0900004749.mm.1	6,43	0,004839	0,371624		
TC0900001114.mm.1	1,61	Dopey1	JUC0900004750.mm.1	3,39	0,044279	0,551322		
TC0900001114.mm.1	1,61	Dopey1	JUC0900004735.mm.1	3,28	0,011233	0,426718		
TC0900001114.mm.1	1,61	Dopey1	PSR0900008744.mm.1	-2,64	0,046016	0,555624	Alternative 5' Donc	0,27
TC0900001114.mm.1	1,61	Dopey1	JUC0900004773.mm.1	-2,72	0,003088	0,353892		
TC0700003126.mm.1	1,95	C230091D08Rik; Gm2496(	JUC0700015063.mm.1	6,42	0,028029	0,505851		
TC0700003126.mm.1	1,95	C230091D08Rik; Gm2496(	PSR0700028291.mm.1	5,93	0,015035	0,452111	Cassette Exon	0,29
TC0700003126.mm.1	1,95	C230091D08Rik; Gm2496(	PSR0700028274.mm.1	3,37	0,000811	0,311886	Cassette Exon	0,08

TC0700003126.mm.1	1,95	C230091D08Rik; Gm2496(	PSR0700028288.mm.1	2,06	0,008057	0,40448	Alternative 5' Donc	0,15
TC0700003126.mm.1	1,95	C230091D08Rik; Gm2496(	PSR0700028289.mm.1	-2,02	0,007779	0,402902	Alternative 5' Donc	0,18
TC0700003126.mm.1	1,95	C230091D08Rik; Gm2496(	PSR0700028280.mm.1	-2,02	0,014653	0,450098	Cassette Exon	0,11
TC0700003126.mm.1	1,95	C230091D08Rik; Gm2496(	JUC0700015068.mm.1	-2,1	0,012739	0,436731		
TC0700003126.mm.1	1,95	C230091D08Rik; Gm2496(	JUC0700015065.mm.1	-3,43	0,000835	0,311886		
TC0X00001067.mm.1	-1,27	Atp7a	JUC0X00003477.mm.1	6,4	0,005328	0,378059		
TC0X00001067.mm.1	-1,27	Atp7a	JUC0X00003468.mm.1	2,12	0,047297	0,558249		
TC1800000443.mm.1	1,23	Stk32a	JUC1800001804.mm.1	6,39	0,001584	0,331726		
TC1800000443.mm.1	1,23	Stk32a	JUC1800001805.mm.1	3,5	0,003214	0,354243		
TC0400003483.mm.1	1,28	D830031N03Rik; Macf1	JUC0400014782.mm.1	6,38	0,036321	0,530547		
TC0400003483.mm.1	1,28	D830031N03Rik; Macf1	JUC0400014752.mm.1	5,98	0,001851	0,337414		
TC0400003483.mm.1	1,28	D830031N03Rik; Macf1	JUC0400014722.mm.1	4,83	0,004185	0,362773		
TC0400003483.mm.1	1,28	D830031N03Rik; Macf1	JUC0400014745.mm.1	2,28	0,011019	0,425624		
TC0400003483.mm.1	1,28	D830031N03Rik; Macf1	JUC0400014769.mm.1	2,18	0,025444	0,497549		
TC0400003483.mm.1	1,28	D830031N03Rik; Macf1	JUC0400014826.mm.1	-2,65	0,028952	0,508766		
TC1000002418.mm.1	-1,03	Col18a1	PSR1000017505.mm.1	6,36	0,031691	0,517443	Cassette Exon	0,28
TC1000002418.mm.1	-1,03	Col18a1	JUC1000009623.mm.1	2,45	0,012186	0,433072		
TC0300000871.mm.1	3,93	Celf3	JUC0300003486.mm.1	6,34	0,001798	0,336311		
TC0300000871.mm.1	3,93	Celf3	PSR0300006680.mm.1	3,98	0,003571	0,356223	Cassette Exon	0,21
TC0300000871.mm.1	3,93	Celf3	PSR0300006679.mm.1	3,1	0,007997	0,403853	Cassette Exon	0,11
TC0300000871.mm.1	3,93	Celf3	PSR0300006668.mm.1	2,34	0,031206	0,515777	Cassette Exon	0,04
TC0300000871.mm.1	3,93	Celf3	PSR0300006671.mm.1	-2,05	0,003354	0,354243	Cassette Exon	0,21
TC0300000871.mm.1	3,93	Celf3	PSR0300006659.mm.1	-2,1	0,020507	0,478244	Alternative 3' Acce	0,22
TC0300000871.mm.1	3,93	Celf3	JUC0300003484.mm.1	-2,12	0,025324	0,497278		
TC0300000871.mm.1	3,93	Celf3	PSR0300006665.mm.1	-2,32	0,000744	0,307808	Alternative 3' Acce	0,2
TC0300000871.mm.1	3,93	Celf3	PSR0300006661.mm.1	-3,22	0,002165	0,344238	Alternative 3' Acce	0,36
TC0300000871.mm.1	3,93	Celf3	JUC0300003479.mm.1	-3,51	0,011825	0,430456		
TC0300000871.mm.1	3,93	Celf3	PSR0300006657.mm.1	-3,68	0,012623	0,436032	Alternative 3' Acce	0,22
TC0300000871.mm.1	3,93	Celf3	PSR0300006674.mm.1	-4,22	0,001568	0,331411	Alternative 5' Donc	0,22
TC0300000871.mm.1	3,93	Celf3	JUC0300003481.mm.1	-5,72	0,01582	0,45594		
TC0500001412.mm.1	1,76	Wdr66	JUC0500006869.mm.1	6,33	0,002919	0,352289		
TC0500001412.mm.1	1,76	Wdr66	PSR0500012580.mm.1	3,04	0,034103	0,524833	Alternative 3' Acce	0,36
TC0500001412.mm.1	1,76	Wdr66	PSR0500012560.mm.1	2,85	0,048473	0,560864	Cassette Exon	0,22
TC0500001412.mm.1	1,76	Wdr66	JUC0500006866.mm.1	2,78	0,031113	0,515372		
TC0500001412.mm.1	1,76	Wdr66	JUC0500006874.mm.1	2,32	0,006837	0,393893		
TC0500001412.mm.1	1,76	Wdr66	PSR0500012573.mm.1	-2,38	0,003841	0,358714	Alternative 5' Donc	0,27
TC0500001412.mm.1	1,76	Wdr66	PSR0500012553.mm.1	-2,5	0,013111	0,439759	Alternative 3' Acce	0,2
TC0500001412.mm.1	1,76	Wdr66	JUC0500006862.mm.1	-3,08	0,022041	0,48529		
TC1000001684.mm.1	1,26	Syne1	JUC1000006657.mm.1	6,31	0,002961	0,352501		
TC1000001684.mm.1	1,26	Syne1	JUC1000006658.mm.1	3,77	0,008524	0,407684		
TC1000001684.mm.1	1,26	Syne1	JUC1000006650.mm.1	2,34	0,002971	0,352501		
TC0200005400.mm.1	2,8	Hrh3	PSR0200045911.mm.1	6,3	0,001165	0,318159	Cassette Exon	0,28
TC0200005400.mm.1	2,8	Hrh3	PSR0200045908.mm.1	-2,08	0,005551	0,380756	Intron Retention	0,23
TC0200005400.mm.1	2,8	Hrh3	PSR0200045917.mm.1	-2,08	0,01311	0,439759	Alternative 5' Donc	0,17
TC0200005400.mm.1	2,8	Hrh3	JUC0200023630.mm.1	-2,16	0,006394	0,389654		
TC0200005400.mm.1	2,8	Hrh3	PSR0200045902.mm.1	-2,19	0,000285	0,28803	Alternative 3' Acce	0,2
TC0200005400.mm.1	2,8	Hrh3	PSR0200045899.mm.1	-2,67	0,004401	0,366415	Alternative 3' Acce	0,2
TC0200005400.mm.1	2,8	Hrh3	JUC0200023629.mm.1	-3,44	0,006398	0,389654		

TC0200005400.mm.1	2,8	Hrh3	PSR0200045898.mm.1	-3,65	0,000482	0,298999	Cassette Exon	0,3
TC0900000607.mm.1	-1,21	Alg9	JUC0900002281.mm.1	6,3	0,000538	0,304044		
TC0900000607.mm.1	-1,21	Alg9	JUC0900002280.mm.1	3,95	0,036903	0,532283		
TC0900000607.mm.1	-1,21	Alg9	JUC0900002260.mm.1	2,67	0,043449	0,549785		
TC0900000607.mm.1	-1,21	Alg9	JUC0900002282.mm.1	2,06	0,019924	0,475902		
TC0900003023.mm.1	1,65	Dock3; Gm22238	JUC0900014116.mm.1	6,26	0,024341	0,49362		
TC0900003023.mm.1	1,65	Dock3; Gm22238	PSR0900025239.mm.1	4,62	0,004068	0,361956	Cassette Exon	0,28
TC0900003023.mm.1	1,65	Dock3; Gm22238	JUC0900014143.mm.1	4,27	0,002182	0,344453		
TC0900003023.mm.1	1,65	Dock3; Gm22238	JUC0900014104.mm.1	3,13	0,04947	0,563286		
TC0900003023.mm.1	1,65	Dock3; Gm22238	PSR0900025221.mm.1	3,06	0,00005	0,24627	Cassette Exon	0,25
TC0900003023.mm.1	1,65	Dock3; Gm22238	PSR0900025240.mm.1	2,89	0,00336	0,354243	Cassette Exon	0,24
TC0900003023.mm.1	1,65	Dock3; Gm22238	JUC0900014097.mm.1	2,63	0,001337	0,325349		
TC0900003023.mm.1	1,65	Dock3; Gm22238	JUC0900014102.mm.1	2,62	0,003108	0,353892		
TC0900003023.mm.1	1,65	Dock3; Gm22238	PSR0900025220.mm.1	2,52	0,031147	0,515641	Cassette Exon	0,22
TC0900003023.mm.1	1,65	Dock3; Gm22238	JUC0900014135.mm.1	2,29	0,022257	0,485896		
TC0900003023.mm.1	1,65	Dock3; Gm22238	PSR0900025174.mm.1	2,09	0,00672	0,393343	Cassette Exon	0,08
TC0900003023.mm.1	1,65	Dock3; Gm22238	JUC0900014147.mm.1	2,02	0,003397	0,354856		
TC0900003023.mm.1	1,65	Dock3; Gm22238	JUC0900014100.mm.1	-2,1	0,035702	0,529027		
TC0900003023.mm.1	1,65	Dock3; Gm22238	PSR0900025230.mm.1	-2,26	0,018462	0,468677	Alternative 3' Acce	0,1
TC0900003023.mm.1	1,65	Dock3; Gm22238	PSR0900025168.mm.1	-2,39	0,004475	0,367858	Cassette Exon	0,13
TC0900003023.mm.1	1,65	Dock3; Gm22238	JUC0900014153.mm.1	-3,25	0,003671	0,357266		
TC0900003023.mm.1	1,65	Dock3; Gm22238	JUC0900014152.mm.1	-3,89	0,001453	0,328414		
TC0900003023.mm.1	1,65	Dock3; Gm22238	PSR0900025235.mm.1	-5,24	0,018673	0,470205	Alternative 3' Acce	0,59
TC0700001117.mm.1	-1,55		JUC0700005016.mm.1	6,23	0,02465	0,49468		
TC0700004225.mm.1	-1,01	Palb2	JUC0700018564.mm.1	6,21	0,000006	0,179072		
TC0600001327.mm.1	1,33	Rasgef1a; Mir7044; mmu-	JUC0600005462.mm.1	6,2	0,001129	0,317328		
TC0600001327.mm.1	1,33	Rasgef1a; Mir7044; mmu-	JUC0600005456.mm.1	4,37	0,004131	0,3623		
TC0600001327.mm.1	1,33	Rasgef1a; Mir7044; mmu-	JUC0600005459.mm.1	4,04	0,036336	0,530547		
TC0600001327.mm.1	1,33	Rasgef1a; Mir7044; mmu-	PSR0600010514.mm.1	2,78	0,002733	0,349612	Cassette Exon	0,2
TC0600001327.mm.1	1,33	Rasgef1a; Mir7044; mmu-	PSR0600010511.mm.1	-4,06	0,035197	0,528243	Alternative 3' Acce	0,51
TC0900001009.mm.1	1,38	Myo5a	JUC0900004267.mm.1	6,2	0,013259	0,441506		
TC0900001009.mm.1	1,38	Myo5a	JUC0900004269.mm.1	5,78	0,015597	0,454998		
TC0900001009.mm.1	1,38	Myo5a	JUC0900004268.mm.1	4,97	0,004264	0,363868		
TC0900001009.mm.1	1,38	Myo5a	JUC0900004282.mm.1	3,55	0,020795	0,479488		
TC0900001009.mm.1	1,38	Myo5a	JUC0900004281.mm.1	3,33	0,011587	0,429241		
TC0900001009.mm.1	1,38	Myo5a	PSR0900007912.mm.1	-2,04	0,031188	0,515739	Cassette Exon	0,29
TC0900001009.mm.1	1,38	Myo5a	PSR0900007907.mm.1	-2,48	0,006985	0,395722	Cassette Exon	0,46
TC0900001009.mm.1	1,38	Myo5a	PSR0900007879.mm.1	-2,57	0,01303	0,439367	Alternative 3' Acce	0,19
TC0900001009.mm.1	1,38	Myo5a	JUC0900004254.mm.1	-2,67	0,02576	0,498442		
TC0900001009.mm.1	1,38	Myo5a	JUC0900004283.mm.1	-2,9	0,02897	0,508766		
TC0900001009.mm.1	1,38	Myo5a	JUC0900004271.mm.1	-3,2	0,01177	0,430069		
TC1300002090.mm.1	1,64	Klhl3	PSR1300013271.mm.1	6,2	0,002704	0,349612	Cassette Exon	0,25
TC1300002090.mm.1	1,64	Klhl3	JUC1300006853.mm.1	5,95	0,007648	0,402193		
TC1300002090.mm.1	1,64	Klhl3	PSR1300013278.mm.1	2,46	0,031097	0,515253	Mutually Exclusive	0,29
TC1300002090.mm.1	1,64	Klhl3	JUC1300006848.mm.1	-2,3	0,042094	0,546488		
TC1300002090.mm.1	1,64	Klhl3	PSR1300013284.mm.1	-2,33	0,025944	0,499144	Cassette Exon	0,27
TC1300002090.mm.1	1,64	Klhl3	JUC1300006861.mm.1	-2,45	0,015165	0,452699		
TC1300002090.mm.1	1,64	Klhl3	PSR1300013279.mm.1	-2,46	0,014231	0,447947	Mutually Exclusive	0,29

TC1300002090.mm.1	1,64	Klhl3	JUC1300006860.mm.1	-2,79	0,016036	0,457219		
TC0800003186.mm.1	1,35	Pcnxl2	JUC0800013406.mm.1	6,2	0,00034	0,289576		
TC0800003186.mm.1	1,35	Pcnxl2	JUC0800013385.mm.1	3,26	0,012336	0,43443		
TC0800003186.mm.1	1,35	Pcnxl2	PSR0800024503.mm.1	2,74	0,009238	0,414373	Cassette Exon	0,2
TC0800003186.mm.1	1,35	Pcnxl2	JUC0800013396.mm.1	2,68	0,030352	0,512923		
TC0800003186.mm.1	1,35	Pcnxl2	JUC0800013399.mm.1	2,48	0,016495	0,459399		
TC0800003186.mm.1	1,35	Pcnxl2	PSR0800024521.mm.1	2,13	0,034176	0,524992	Cassette Exon	0,18
TC0800003186.mm.1	1,35	Pcnxl2	JUC0800013374.mm.1	-2,31	0,000515	0,303579		
TC0800003186.mm.1	1,35	Pcnxl2	PSR0800024524.mm.1	-2,37	0,036441	0,530977	Alternative 5' Donc	0,17
TC0800003186.mm.1	1,35	Pcnxl2	JUC0800013386.mm.1	-2,65	0,030417	0,513068		
TC0800003186.mm.1	1,35	Pcnxl2	JUC0800013373.mm.1	-3,02	0,028949	0,508766		
TC0200003201.mm.1	-1,29	Abo	JUC0200013587.mm.1	6,19	0,0082	0,405909		
TC0500001783.mm.1	2,79	Wasf3	JUC0500008993.mm.1	6,18	0,007708	0,402552		
TC0500001783.mm.1	2,79	Wasf3	JUC0500008994.mm.1	4,38	0,001743	0,335996		
TC0500001783.mm.1	2,79	Wasf3	PSR0500016517.mm.1	2,69	0,001821	0,336896	Cassette Exon	0,43
TC0500001783.mm.1	2,79	Wasf3	PSR0500016519.mm.1	-2,34	0,002265	0,34578	Cassette Exon	0,11
TC0100000023.mm.1	1,38	St18	JUC0100000098.mm.1	6,18	0,000212	0,28803		
TC0100000023.mm.1	1,38	St18	JUC0100000099.mm.1	4,82	0,015735	0,455416		
TC0100000023.mm.1	1,38	St18	PSR0100000239.mm.1	4,4	0,007053	0,396402	Alternative 5' Donc	0,23
TC0100000023.mm.1	1,38	St18	JUC0100000091.mm.1	4,06	0,018133	0,467325		
TC0100000023.mm.1	1,38	St18	PSR0100000203.mm.1	3,91	0,002649	0,349612	Cassette Exon	0,4
TC0100000023.mm.1	1,38	St18	JUC0100000110.mm.1	2,73	0,006457	0,390284		
TC0100000023.mm.1	1,38	St18	PSR0100000233.mm.1	2,65	0,009316	0,414883	Cassette Exon	0,15
TC0100000023.mm.1	1,38	St18	PSR0100000201.mm.1	2,54	0,034682	0,526294	Cassette Exon	0,12
TC0100000023.mm.1	1,38	St18	PSR0100000219.mm.1	2,5	0,019526	0,474538	Cassette Exon	0,41
TC0100000023.mm.1	1,38	St18	JUC0100000100.mm.1	2,28	0,018095	0,46716		
TC0100000023.mm.1	1,38	St18	JUC0100000093.mm.1	2,12	0,009305	0,414883		
TC0100000023.mm.1	1,38	St18	JUC0100000113.mm.1	2,03	0,009489	0,416578		
TC0100000023.mm.1	1,38	St18	PSR0100000228.mm.1	-2,09	0,001586	0,331726	Cassette Exon	0,1
TC0100000023.mm.1	1,38	St18	JUC0100000116.mm.1	-2,15	0,008658	0,408463		
TC0100000023.mm.1	1,38	St18	JUC0100000112.mm.1	-2,16	0,010795	0,424049		
TC0100000023.mm.1	1,38	St18	JUC0100000117.mm.1	-2,58	0,006742	0,393343		
TC0100000023.mm.1	1,38	St18	PSR0100000195.mm.1	-2,68	0,008791	0,409822	Alternative 3' Acce	0,27
TC0100000023.mm.1	1,38	St18	PSR0100000185.mm.1	-2,69	0,030685	0,513877	Cassette Exon	0,16
TC0100000023.mm.1	1,38	St18	JUC0100000115.mm.1	-3,1	0,017855	0,46623		
TC0100000023.mm.1	1,38	St18	JUC0100000102.mm.1	-3,21	0,023838	0,491997		
TC0100000023.mm.1	1,38	St18	JUC0100000089.mm.1	-4,07	0,003382	0,354403		
TC1600001057.mm.1	1,28	Ttc3	JUC1600004304.mm.1	6,18	0,01268	0,43631		
TC1600001057.mm.1	1,28	Ttc3	JUC1600004320.mm.1	2,61	0,032774	0,520741		
TC1600001057.mm.1	1,28	Ttc3	JUC1600004332.mm.1	2,23	0,02813	0,506228		
TC1600001057.mm.1	1,28	Ttc3	JUC1600004316.mm.1	2,1	0,027481	0,504126		
TC1600001057.mm.1	1,28	Ttc3	PSR1600008277.mm.1	-2,04	0,003731	0,357586	Alternative 3' Acce	0,08
TC1600001057.mm.1	1,28	Ttc3	PSR1600008280.mm.1	-2,25	0,049296	0,562691	Alternative 3' Acce	0,2
TC1600001057.mm.1	1,28	Ttc3	JUC1600004344.mm.1	-2,94	0,042009	0,546101		
TC0600003121.mm.1	5,88	Gnb3	JUC0600012645.mm.1	6,16	0,0013	0,323255		
TC0600003121.mm.1	5,88	Gnb3	JUC0600012650.mm.1	2,86	0,000449	0,297771		
TC0600003121.mm.1	5,88	Gnb3	PSR0600024293.mm.1	2,78	0,004282	0,363973	Cassette Exon	0,29
TC0600003121.mm.1	5,88	Gnb3	JUC0600012648.mm.1	2,77	0,003621	0,356223		

TC0600003121.mm.1	5,88	Gnb3	PSR0600024291.mm.1	2,63	0,04443	0,551566	Cassette Exon	0,24
TC0600003121.mm.1	5,88	Gnb3	PSR0600024296.mm.1	2,27	0,000513	0,303579		
TC0600003121.mm.1	5,88	Gnb3	PSR0600024297.mm.1	-2,28	0,018109	0,467161	Alternative 5' Donc	0,44
TC0600003121.mm.1	5,88	Gnb3	PSR0600024299.mm.1	-2,92	0,023272	0,49004	Cassette Exon	0,33
TC0600003121.mm.1	5,88	Gnb3	JUC0600012653.mm.1	-3,17	0,000914	0,313363		
TC0600003121.mm.1	5,88	Gnb3	PSR0600024301.mm.1	-3,68	0,002218	0,345773	Cassette Exon	0,33
TC0600003121.mm.1	5,88	Gnb3	PSR0600024287.mm.1	-3,72	0,017981	0,466929	Alternative 5' Donc	0,56
TC0600003121.mm.1	5,88	Gnb3	PSR0600024289.mm.1	-3,84	0,030627	0,513669	Cassette Exon	0,4
TC0600003121.mm.1	5,88	Gnb3	JUC0600012651.mm.1	-4,2	0,002313	0,346468		
TC0600003121.mm.1	5,88	Gnb3	PSR0600024298.mm.1	-4,25	0,003569	0,356223	Cassette Exon	0,47
TC0600003121.mm.1	5,88	Gnb3	PSR0600024295.mm.1	-5,53	0,016262	0,458329	Alternative 3' Acce	0,34
TC0600003121.mm.1	5,88	Gnb3	PSR0600024300.mm.1	-5,88	0,004346	0,365385	Cassette Exon	0,33
TC0600003121.mm.1	5,88	Gnb3	JUC0600012655.mm.1	-7,17	0,010105	0,418998		
TC0200003473.mm.1	1,3	Zeb2; Mir5129	JUC0200015019.mm.1	6,16	0,006354	0,389469		
TC0200003473.mm.1	1,3	Zeb2; Mir5129	PSR0200029758.mm.1	-2,52	0,011037	0,425682	Alternative 5' Donc	0,24
TC0200003473.mm.1	1,3	Zeb2; Mir5129	JUC0200015007.mm.1	-3,03	0,01688	0,460797		
TC0200001747.mm.1	3,27	Disp2	JUC0200006465.mm.1	6,15	0,003217	0,354243		
TC0200001747.mm.1	3,27	Disp2	PSR0200012974.mm.1	4,97	0,00034	0,289576	Cassette Exon	0,49
TC0200001747.mm.1	3,27	Disp2	JUC0200006469.mm.1	3,39	0,044217	0,55119		
TC0200001747.mm.1	3,27	Disp2	PSR0200012961.mm.1	-2,44	0,008366	0,406435	Cassette Exon	0,13
TC0200001747.mm.1	3,27	Disp2	PSR0200012973.mm.1	-3,01	0,006164	0,387873	Intron Retention	0,5
TC0200001747.mm.1	3,27	Disp2	PSR0200012969.mm.1	-3,38	0,024952	0,495859	Intron Retention	0,6
TC0200001747.mm.1	3,27	Disp2	PSR0200012978.mm.1	-3,9	0,014354	0,448293	Cassette Exon	0,2
TC0200001747.mm.1	3,27	Disp2	PSR0200012965.mm.1	-4,21	0,01676	0,460504	Intron Retention	0,69
TC0200001747.mm.1	3,27	Disp2	PSR0200012980.mm.1	-4,4	0,004369	0,36554	Cassette Exon	0,41
TC0200001747.mm.1	3,27	Disp2	PSR0200012981.mm.1	-6,38	0,005267	0,378003	Cassette Exon	0,41
TC0200001747.mm.1	3,27	Disp2	PSR0200012967.mm.1	-7	0,001508	0,330215	Intron Retention	0,75
TC0200001747.mm.1	3,27	Disp2	JUC0200006473.mm.1	-7,68	0,006925	0,395156		
TC1000000246.mm.1	-1,03	Ptprk	JUC1000000958.mm.1	6,15	0,008203	0,405909		
TC1000000246.mm.1	-1,03	Ptprk	JUC1000000937.mm.1	3,49	0,005786	0,383104		
TC1000000246.mm.1	-1,03	Ptprk	JUC1000000960.mm.1	2,29	0,04351	0,549937		
TC1000000246.mm.1	-1,03	Ptprk	JUC1000000957.mm.1	2,28	0,032401	0,519282		
TC1000000246.mm.1	-1,03	Ptprk	JUC1000000952.mm.1	-2,21	0,015594	0,454982		
TC0200001352.mm.1	1,01	Nup160	JUC0200005131.mm.1	6,14	0,011056	0,425887		
TC0900000542.mm.1	3,26	Dscaml1	JUC0900001964.mm.1	6,1	0,00048	0,298999		
TC0900000542.mm.1	3,26	Dscaml1	JUC0900001966.mm.1	5,02	0,000111	0,267977		
TC0900000542.mm.1	3,26	Dscaml1	JUC0900001945.mm.1	4,54	0,009203	0,413922		
TC0900000542.mm.1	3,26	Dscaml1	JUC0900001931.mm.1	3,56	0,034167	0,524988		
TC0900000542.mm.1	3,26	Dscaml1	PSR0900003764.mm.1	3,32	0,038813	0,537594	Cassette Exon	0,19
TC0900000542.mm.1	3,26	Dscaml1	PSR0900003794.mm.1	2,54	0,000244	0,28803	Cassette Exon	0,21
TC0900000542.mm.1	3,26	Dscaml1	PSR0900003779.mm.1	2,33	0,001377	0,32572	Cassette Exon	0,05
TC0900000542.mm.1	3,26	Dscaml1	PSR0900003761.mm.1	-2,26	0,022361	0,486366	Cassette Exon	0,33
TC0900000542.mm.1	3,26	Dscaml1	PSR0900003765.mm.1	-2,49	0,027326	0,503766	Cassette Exon	0,34
TC0900000542.mm.1	3,26	Dscaml1	PSR0900003771.mm.1	-2,54	0,000649	0,304044	Cassette Exon	0,39
TC0900000542.mm.1	3,26	Dscaml1	JUC0900001963.mm.1	-2,58	0,025776	0,498465		
TC0900000542.mm.1	3,26	Dscaml1	JUC0900001929.mm.1	-2,69	0,010931	0,424831		
TC0900000542.mm.1	3,26	Dscaml1	JUC0900001947.mm.1	-2,79	0,010173	0,419711		
TC0900000542.mm.1	3,26	Dscaml1	JUC0900001960.mm.1	-3	0,003905	0,35953		

TC0900000542.mm.1	3,26	Dscaml1	JUC0900001951.mm.1	-3,06	0,004344	0,365267		
TC0900000542.mm.1	3,26	Dscaml1	JUC0900001934.mm.1	-3,1	0,012031	0,432286		
TC0900000542.mm.1	3,26	Dscaml1	JUC0900001940.mm.1	-3,11	0,00377	0,357586		
TC0900000542.mm.1	3,26	Dscaml1	JUC0900001967.mm.1	-3,16	0,01421	0,447798		
TC0900000542.mm.1	3,26	Dscaml1	PSR0900003790.mm.1	-3,57	0,006062	0,386691	Cassette Exon	0,38
TC0900000542.mm.1	3,26	Dscaml1	PSR0900003766.mm.1	-3,68	0,0221	0,485486	Cassette Exon	0,4
TC0900000542.mm.1	3,26	Dscaml1	JUC0900001955.mm.1	-3,79	0,009519	0,416602		
TC0900000542.mm.1	3,26	Dscaml1	JUC0900001942.mm.1	-4,38	0,005616	0,381191		
TC0900000542.mm.1	3,26	Dscaml1	PSR0900003759.mm.1	-4,43	0,00223	0,345773	Cassette Exon	0,41
TC0900000542.mm.1	3,26	Dscaml1	JUC0900001950.mm.1	-4,46	0,013581	0,443703		
TC0900000542.mm.1	3,26	Dscaml1	PSR0900003760.mm.1	-5,29	0,006961	0,395722	Cassette Exon	0,29
TC0900000542.mm.1	3,26	Dscaml1	JUC0900001943.mm.1	-5,32	0,009053	0,412827		
TC0900000542.mm.1	3,26	Dscaml1	JUC0900001932.mm.1	-5,73	0,005069	0,375225		
TC0900000542.mm.1	3,26	Dscaml1	JUC0900001935.mm.1	-6,03	0,010521	0,422224		
TC0900000542.mm.1	3,26	Dscaml1	JUC0900001941.mm.1	-6,55	0,006148	0,38784		
TC0900000542.mm.1	3,26	Dscaml1	JUC0900001930.mm.1	-6,57	0,007043	0,396259		
TC0900000542.mm.1	3,26	Dscaml1	JUC0900001957.mm.1	-7,39	0,004334	0,364898		
TC0900000542.mm.1	3,26	Dscaml1	JUC0900001953.mm.1	-7,42	0,010334	0,420914		
TC0900000542.mm.1	3,26	Dscaml1	JUC0900001939.mm.1	-7,51	0,002449	0,349135		
TC0900000542.mm.1	3,26	Dscaml1	JUC0900001928.mm.1	-7,54	0,003911	0,359698		
TC0900000542.mm.1	3,26	Dscaml1	JUC0900001968.mm.1	-9,07	0,004973	0,373851		
TC0900000750.mm.1	1,44	Parp6	JUC0900002958.mm.1	6,1	0,007316	0,398848		
TC0900000750.mm.1	1,44	Parp6	JUC0900002961.mm.1	2,94	0,004194	0,362958		
TC0900000750.mm.1	1,44	Parp6	JUC0900002940.mm.1	-2,01	0,004674	0,370183		
TC1200000389.mm.1	3,4	Nrcam	JUC1200001538.mm.1	6,07	0,000236	0,28803		
TC1200000389.mm.1	3,4	Nrcam	JUC1200001539.mm.1	3,56	0,000311	0,288663		
TC1200000389.mm.1	3,4	Nrcam	PSR1200002762.mm.1	2,63	0,019032	0,471845	Cassette Exon	0,21
TC1200000389.mm.1	3,4	Nrcam	JUC1200001550.mm.1	2,62	0,035886	0,52938		
TC1200000389.mm.1	3,4	Nrcam	JUC1200001542.mm.1	2,24	0,000728	0,306408		
TC1200000389.mm.1	3,4	Nrcam	PSR1200002797.mm.1	2,18	0,004333	0,364898	Cassette Exon	0,11
TC1200000389.mm.1	3,4	Nrcam	PSR1200002788.mm.1	-2,26	0,016249	0,458312	Cassette Exon	0,18
TC1200000389.mm.1	3,4	Nrcam	PSR1200002792.mm.1	-2,37	0,010885	0,424247	Cassette Exon	0,18
TC1200000389.mm.1	3,4	Nrcam	JUC1200001566.mm.1	-2,68	0,042793	0,548097		
TC1200000389.mm.1	3,4	Nrcam	PSR1200002752.mm.1	-2,72	0,019478	0,474295	Cassette Exon	0,16
TC1200000389.mm.1	3,4	Nrcam	JUC1200001537.mm.1	-2,8	0,019274	0,473074		
TC1200000389.mm.1	3,4	Nrcam	PSR1200002787.mm.1	-2,91	0,016223	0,458312	Cassette Exon	0,11
TC1200000389.mm.1	3,4	Nrcam	PSR1200002775.mm.1	-2,93	0,006773	0,393372	Cassette Exon	0,11
TC1200000389.mm.1	3,4	Nrcam	PSR1200002781.mm.1	-3,05	0,013778	0,444974	Cassette Exon	0,17
TC1200000389.mm.1	3,4	Nrcam	JUC1200001556.mm.1	-4,22	0,004169	0,3623		
TC1200000389.mm.1	3,4	Nrcam	PSR1200002793.mm.1	-4,33	0,005503	0,379939	Cassette Exon	0,44
TC1200000389.mm.1	3,4	Nrcam	JUC1200001577.mm.1	-4,85	0,00046	0,298999		
TC1200000389.mm.1	3,4	Nrcam	PSR1200002754.mm.1	-5,21	0,002339	0,347265	Alternative 5' Dono	0,44
TC1200000389.mm.1	3,4	Nrcam	JUC1200001569.mm.1	-5,5	0,02813	0,506228		
TC1200000389.mm.1	3,4	Nrcam	JUC1200001543.mm.1	-5,86	0,000166	0,272566		
TC1200000389.mm.1	3,4	Nrcam	PSR1200002764.mm.1	-6,03	0,000516	0,303579	Intron Retention	0,75
TC1200000389.mm.1	3,4	Nrcam	JUC1200001574.mm.1	-6,04	0,02869	0,508019		
TC1200000389.mm.1	3,4	Nrcam	JUC1200001565.mm.1	-7,89	0,011875	0,431125		
TC0400004090.mm.1	-1,01	Espn	JUC0400017770.mm.1	6,07	0,003182	0,354243		

TC0800002767.mm.1	4,19	Cdh8	JUC0800011620.mm.1	6,06	0,001191	0,321162		
TC0800002767.mm.1	4,19	Cdh8	JUC0800011614.mm.1	4,39	0,000345	0,289579		
TC0800002767.mm.1	4,19	Cdh8	PSR0800021283.mm.1	2,12	0,001399	0,325997	Cassette Exon	0,11
TC0800002767.mm.1	4,19	Cdh8	PSR0800021275.mm.1	-2,06	0,026552	0,501435		
TC0800002767.mm.1	4,19	Cdh8	PSR0800021269.mm.1	-2,19	0,021587	0,483245	Cassette Exon	0,43
TC0800002767.mm.1	4,19	Cdh8	PSR0800021284.mm.1	-2,47	0,000141	0,272178	Alternative 5' Donc	0,1
TC0800002767.mm.1	4,19	Cdh8	JUC0800011611.mm.1	-3,54	0,017019	0,461709		
TC0800002767.mm.1	4,19	Cdh8	PSR0800021268.mm.1	-3,58	0,0044	0,366403	Cassette Exon	0,43
TC0800002767.mm.1	4,19	Cdh8	PSR0800021265.mm.1	-4,35	0,001471	0,329052	Alternative 3' Acce	0,32
TC0800002767.mm.1	4,19	Cdh8	PSR0800021286.mm.1	-4,56	0,002492	0,349135	Cassette Exon	0,28
TC0800002767.mm.1	4,19	Cdh8	PSR0800021272.mm.1	-4,86	0,009034	0,412736	Cassette Exon	0,53
TC0800002767.mm.1	4,19	Cdh8	PSR0800021270.mm.1	-5,15	0,003816	0,35811	Cassette Exon	0,43
TC0800002767.mm.1	4,19	Cdh8	PSR0800021287.mm.1	-6,23	0,001092	0,317225	Cassette Exon	0,28
TC0800002767.mm.1	4,19	Cdh8	JUC0800011622.mm.1	-6,51	0,005327	0,378059		
TC0800002767.mm.1	4,19	Cdh8	JUC0800011625.mm.1	-8,21	0,002616	0,349612		
TC0800002767.mm.1	4,19	Cdh8	JUC0800011623.mm.1	-8,53	0,006183	0,387873		
TC0200002171.mm.1	1,74	4930529M08Rik	PSR0200017195.mm.1	6,06	0,000002	0,179072	Alternative 5' Donc	0,29
TC0200002171.mm.1	1,74	4930529M08Rik	PSR0200017206.mm.1	4,37	0,016263	0,458329	Cassette Exon	0,32
TC0200002171.mm.1	1,74	4930529M08Rik	PSR0200017197.mm.1	3,29	0,005469	0,379939	Cassette Exon	0,29
TC0200002171.mm.1	1,74	4930529M08Rik	PSR0200017191.mm.1	3,29	0,01892	0,471305	Cassette Exon	0,22
TC0200002171.mm.1	1,74	4930529M08Rik	JUC0200008655.mm.1	2,84	0,027741	0,505023		
TC0200002171.mm.1	1,74	4930529M08Rik	PSR0200017183.mm.1	2,69	0,0189	0,47127	Mutually Exclusive	0,18
TC0200002171.mm.1	1,74	4930529M08Rik	PSR0200017201.mm.1	2,67	0,016553	0,459612	Cassette Exon	0,12
TC0200002171.mm.1	1,74	4930529M08Rik	JUC0200008653.mm.1	2,65	0,016637	0,459985		
TC0200002171.mm.1	1,74	4930529M08Rik	PSR0200017194.mm.1	2,64	0,041484	0,544745	Cassette Exon	0,29
TC0200002171.mm.1	1,74	4930529M08Rik	JUC0200008656.mm.1	2,61	0,017778	0,465534		
TC0200002171.mm.1	1,74	4930529M08Rik	PSR0200017203.mm.1	2,49	0,004641	0,370181	Cassette Exon	0,19
TC0200002171.mm.1	1,74	4930529M08Rik	PSR0200017184.mm.1	-2,03	0,021779	0,48438	Mutually Exclusive	0,18
TC0200002171.mm.1	1,74	4930529M08Rik	JUC0200008666.mm.1	-2,09	0,027334	0,503766		
TC0200002171.mm.1	1,74	4930529M08Rik	PSR0200017211.mm.1	-2,1	0,0098	0,417839	Alternative 5' Donc	0,15
TC0200002171.mm.1	1,74	4930529M08Rik	PSR0200017190.mm.1	-2,24	0,046582	0,556761	Alternative 3' Acce	0,21
TC0200002171.mm.1	1,74	4930529M08Rik	PSR0200017189.mm.1	-2,33	0,018308	0,468049	Alternative 3' Acce	0,21
TC0200002171.mm.1	1,74	4930529M08Rik	PSR0200017164.mm.1	-2,49	0,001827	0,337273	Cassette Exon	0,12
TC0200002171.mm.1	1,74	4930529M08Rik	PSR0200017173.mm.1	-2,66	0,049174	0,562376	Alternative 5' Donc	0,22
TC0600003107.mm.1	1,8	Clstn3	JUC0600012494.mm.1	6,06	0,022036	0,485268		
TC0600003107.mm.1	1,8	Clstn3	JUC0600012508.mm.1	4,96	0,023083	0,489697		
TC0600003107.mm.1	1,8	Clstn3	JUC0600012509.mm.1	4,04	0,036178	0,529944		
TC0600003107.mm.1	1,8	Clstn3	JUC0600012504.mm.1	3,63	0,012036	0,432286		
TC0600003107.mm.1	1,8	Clstn3	JUC0600012507.mm.1	2,21	0,010839	0,424161		
TC0600003107.mm.1	1,8	Clstn3	PSR0600023985.mm.1	-2,08	0,039038	0,53823	Alternative 5' Donc	0,16
TC0600003107.mm.1	1,8	Clstn3	PSR0600023986.mm.1	-2,16	0,042588	0,548047	Alternative 5' Donc	0,16
TC0600003107.mm.1	1,8	Clstn3	PSR0600023975.mm.1	-2,49	0,028018	0,505851	Alternative 3' Acce	0,2
TC0600003107.mm.1	1,8	Clstn3	JUC0600012512.mm.1	-3,37	0,017341	0,46353		
TC1100004002.mm.1	1,84	Abca6	JUC1100019666.mm.1	6,06	0,004438	0,367041		
TC1100004002.mm.1	1,84	Abca6	JUC1100019672.mm.1	3,72	0,013172	0,440567		
TC1100004002.mm.1	1,84	Abca6	PSR1100037580.mm.1	2,5	0,001106	0,317328	Cassette Exon	0,14
TC1100004002.mm.1	1,84	Abca6	JUC1100019669.mm.1	2,1	0,044219	0,55119		
TC1100004002.mm.1	1,84	Abca6	PSR1100037574.mm.1	-2,03	0,003511	0,355749		

TC1100004002.mm.1	1,84	Abca6	JUC1100019698.mm.1	-2,2	0,013098	0,439759		
TC1100004002.mm.1	1,84	Abca6	JUC1100019671.mm.1	-2,41	0,012517	0,435429		
TC1100000713.mm.1	1,44	Trim17; Hist3h2a	JUC1100002920.mm.1	6,05	0,003334	0,354243		
TC1100000713.mm.1	1,44	Trim17; Hist3h2a	PSR1100005662.mm.1	5,3	0,002726	0,349612	Alternative 5' Donc	0,35
TC1100000713.mm.1	1,44	Trim17; Hist3h2a	PSR1100005664.mm.1	4,01	0,029503	0,510123	Alternative 5' Donc	0,35
TC1100000713.mm.1	1,44	Trim17; Hist3h2a	PSR1100005663.mm.1	3,57	0,008211	0,405909	Alternative 5' Donc	0,35
TC0X00000724.mm.1	-1,37	Xlr4d-ps	JUC0X00002512.mm.1	6,05	0,007594	0,401712		
TC0X00000724.mm.1	-1,37	Xlr4d-ps	PSR0X00005016.mm.1	2,03	0,029396	0,509995	Cassette Exon	0,23
TC1500002214.mm.1	5,26	Faim2	PSR1500018132.mm.1	6,04	0,000739	0,307808	Cassette Exon	0,49
TC1500002214.mm.1	5,26	Faim2	JUC1500010345.mm.1	5,96	0,002339	0,347265		
TC1500002214.mm.1	5,26	Faim2	JUC1500010344.mm.1	5,42	0,000263	0,28803		
TC1500002214.mm.1	5,26	Faim2	JUC1500010343.mm.1	4,15	0,001328	0,325252		
TC1500002214.mm.1	5,26	Faim2	PSR1500018129.mm.1	2,73	0,002654	0,349612	Cassette Exon	0,19
TC1500002214.mm.1	5,26	Faim2	JUC1500010342.mm.1	2,64	0,011256	0,426718		
TC1500002214.mm.1	5,26	Faim2	PSR1500018134.mm.1	2,28	0,005801	0,383104	Cassette Exon	0,39
TC1500002214.mm.1	5,26	Faim2	PSR1500018128.mm.1	-3,3	0,04239	0,547599	Alternative 3' Acce	0,38
TC1500002214.mm.1	5,26	Faim2	PSR1500018139.mm.1	-3,67	0,007606	0,401767	Cassette Exon	0,38
TC1500002214.mm.1	5,26	Faim2	JUC1500010348.mm.1	-5,17	0,009024	0,412625		
TC1500002214.mm.1	5,26	Faim2	PSR1500018142.mm.1	-5,4	0,014724	0,450372	Alternative 5' Donc	0,32
TC1500002214.mm.1	5,26	Faim2	JUC1500010350.mm.1	-8,66	0,002271	0,345986		
TC1500002214.mm.1	5,26	Faim2	PSR1500018141.mm.1	-11,44	0,003187	0,354243	Alternative 5' Donc	0,32
TC0500002947.mm.1	2,63	Cplx1	JUC0500014190.mm.1	6,04	0,000005	0,179072		
TC0500002947.mm.1	2,63	Cplx1	PSR0500026050.mm.1	2,81	0,017242	0,462729	Alternative 3' Acce	0,29
TC0500002947.mm.1	2,63	Cplx1	PSR0500026055.mm.1	2,63	0,005876	0,383835	Cassette Exon	0,2
TC0500002947.mm.1	2,63	Cplx1	JUC0500014192.mm.1	2,04	0,000329	0,28927		
TC0500002947.mm.1	2,63	Cplx1	PSR0500026056.mm.1	-3,52	0,012689	0,436349	Alternative 5' Donc	0,4
TC0500002947.mm.1	2,63	Cplx1	JUC0500014193.mm.1	-3,97	0,044582	0,551982		
TC1700000995.mm.1	1,82	Fsd1	JUC1700005044.mm.1	6,02	0,045692	0,554833		
TC1700000995.mm.1	1,82	Fsd1	JUC1700005035.mm.1	4,7	0,000056	0,24627		
TC1500000747.mm.1	3,47	sept-03	JUC1500003326.mm.1	6,01	0,004461	0,36753		
TC1500000747.mm.1	3,47	sept-03	PSR1500005887.mm.1	2,18	0,003105	0,353892	Cassette Exon	0,29
TC1500000747.mm.1	3,47	sept-03	JUC1500003331.mm.1	-2,7	0,048504	0,560936		
TC1500000747.mm.1	3,47	sept-03	PSR1500005883.mm.1	-2,82	0,02298	0,488876	Cassette Exon	0,19
TC1500000747.mm.1	3,47	sept-03	PSR1500005880.mm.1	-2,86	0,022275	0,485961	Cassette Exon	0,3
TC1500000747.mm.1	3,47	sept-03	JUC1500003323.mm.1	-3,03	0,010527	0,422224		
TC1500000747.mm.1	3,47	sept-03	PSR1500005890.mm.1	-3,7	0,0023	0,346364	Alternative 5' Donc	0,45
TC1500000747.mm.1	3,47	sept-03	JUC1500003333.mm.1	-3,7	0,005857	0,383747		
TC1500000747.mm.1	3,47	sept-03	PSR1500005879.mm.1	-3,94	0,004069	0,361956	Cassette Exon	0,27
TC1500000747.mm.1	3,47	sept-03	PSR1500005881.mm.1	-4,3	0,016594	0,459691	Cassette Exon	0,35
TC0500003019.mm.1	1,64	Sez6l	JUC0500014619.mm.1	5,98	0,000319	0,288663		
TC0500003019.mm.1	1,64	Sez6l	JUC0500014618.mm.1	2,96	0,026903	0,502518		
TC0500003019.mm.1	1,64	Sez6l	PSR0500026784.mm.1	2,12	0,008794	0,409822	Cassette Exon	0,16
TC0500003019.mm.1	1,64	Sez6l	PSR0500026793.mm.1	-2,1	0,022795	0,48821	Cassette Exon	0,21
TC0500003019.mm.1	1,64	Sez6l	JUC0500014626.mm.1	-2,44	0,007713	0,402552		
TC0500003019.mm.1	1,64	Sez6l	JUC0500014631.mm.1	-3,15	0,038243	0,535883		
TC0500003019.mm.1	1,64	Sez6l	JUC0500014630.mm.1	-3,8	0,006261	0,388628		
TC0800001613.mm.1	7,23	Pcp2	JUC0800007225.mm.1	5,95	0,001094	0,317328		
TC0800001613.mm.1	7,23	Pcp2	JUC0800007221.mm.1	3,99	0,002557	0,349501		

TC0800001613.mm.1	7,23	Pcp2	PSR0800013258.mm.1	3,15	0,002181	0,344453	Cassette Exon	0,12
TC0800001613.mm.1	7,23	Pcp2	PSR0800013265.mm.1	-2,27	0,011145	0,426226	Cassette Exon	0,12
TC0800001613.mm.1	7,23	Pcp2	PSR0800013264.mm.1	-10,58	0,006622	0,392392	Cassette Exon	0,48
TC0800001613.mm.1	7,23	Pcp2	PSR0800013259.mm.1	-11,18	0,00061	0,304044	Intron Retention	0,75
TC0800001613.mm.1	7,23	Pcp2	PSR0800013261.mm.1	-12,87	0,004003	0,361169		
TC0800001613.mm.1	7,23	Pcp2	PSR0800013263.mm.1	-15,79	0,00219	0,344463	Alternative 5' Donc	0,16
TC1400002301.mm.1	-1,76	Adam7	JUC1400009779.mm.1	5,95	0,003365	0,354278		
TC1400002301.mm.1	-1,76	Adam7	PSR1400017809.mm.1	2,39	0,033018	0,521478	Cassette Exon	0,13
TC1100001769.mm.1	2,17	Kcnh6	JUC1100008685.mm.1	5,91	0,000018	0,207103		
TC1100001769.mm.1	2,17	Kcnh6	PSR1100016713.mm.1	3,1	0,002961	0,352501	Alternative 5' Donc	0,34
TC1100001769.mm.1	2,17	Kcnh6	PSR1100016714.mm.1	2,32	0,008572	0,40821	Cassette Exon	0,31
TC1100001769.mm.1	2,17	Kcnh6	PSR1100016709.mm.1	2,02	0,007827	0,403089	Cassette Exon	0,09
TC1100001769.mm.1	2,17	Kcnh6	JUC1100008689.mm.1	-2,84	0,049584	0,563357		
TC1100001769.mm.1	2,17	Kcnh6	PSR1100016718.mm.1	-3,09	0,039283	0,539034	Cassette Exon	0,33
TC1000001919.mm.1	-1,31	Lama2	JUC1000007531.mm.1	5,9	0,03021	0,512452		
TC1000001919.mm.1	-1,31	Lama2	JUC1000007554.mm.1	-2,58	0,00785	0,403345		
TC1000001919.mm.1	-1,31	Lama2	JUC1000007558.mm.1	-2,81	0,027249	0,503642		
TC1000001919.mm.1	-1,31	Lama2	JUC1000007574.mm.1	-2,94	0,001003	0,316361		
TC0700004641.mm.1	1,4	Sptbn4	JUC0700012368.mm.1	5,88	0,007262	0,39826		
TC0700004641.mm.1	1,4	Sptbn4	JUC0700012343.mm.1	2,02	0,040856	0,543295		
TC0700004641.mm.1	1,4	Sptbn4	JUC0700012351.mm.1	-2,02	0,012315	0,434392		
TC0700004641.mm.1	1,4	Sptbn4	PSR0700023264.mm.1	-2,21	0,01114	0,426226	Alternative 5' Donc	0,06
TC0700004641.mm.1	1,4	Sptbn4	JUC0700012335.mm.1	-2,79	0,040845	0,543283		
TC0400004170.mm.1	-1,02	Agrn	JUC0400018261.mm.1	5,88	0,00828	0,405992		
TC0300002901.mm.1	2,52	Ank2; Gm4392	JUC0300011946.mm.1	5,87	0,00185	0,337414		
TC0300002901.mm.1	2,52	Ank2; Gm4392	JUC0300011974.mm.1	4,56	0,001407	0,326083		
TC0300002901.mm.1	2,52	Ank2; Gm4392	PSR0300022919.mm.1	2,58	0,017461	0,463886	Cassette Exon	0,35
TC0300002901.mm.1	2,52	Ank2; Gm4392	PSR0300022920.mm.1	2,09	0,016978	0,461419	Cassette Exon	0,26
TC0300002901.mm.1	2,52	Ank2; Gm4392	JUC0300011967.mm.1	-2,19	0,013145	0,440196		
TC0300002901.mm.1	2,52	Ank2; Gm4392	JUC0300012001.mm.1	-2,26	0,014687	0,450253		
TC0300002901.mm.1	2,52	Ank2; Gm4392	PSR0300022949.mm.1	-2,45	0,042804	0,548097	Cassette Exon	0,14
TC0300002901.mm.1	2,52	Ank2; Gm4392	PSR0300022992.mm.1	-2,52	0,014979	0,452048	Alternative 5' Donc	0,13
TC0300002901.mm.1	2,52	Ank2; Gm4392	PSR0300023011.mm.1	-2,63	0,018879	0,471134	Alternative 5' Donc	0,15
TC0300002901.mm.1	2,52	Ank2; Gm4392	PSR0300022989.mm.1	-2,67	0,022344	0,486348	Alternative 5' Donc	0,13
TC0300002901.mm.1	2,52	Ank2; Gm4392	JUC0300012010.mm.1	-3,14	0,023799	0,491893		
TC0300002901.mm.1	2,52	Ank2; Gm4392	PSR0300023010.mm.1	-3,15	0,04079	0,543185	Alternative 5' Donc	0,15
TC0300002901.mm.1	2,52	Ank2; Gm4392	JUC0300011982.mm.1	-3,31	0,010768	0,423955		
TC0300002901.mm.1	2,52	Ank2; Gm4392	PSR0300022908.mm.1	-3,4	0,045543	0,554382	Cassette Exon	0,3
TC0300002901.mm.1	2,52	Ank2; Gm4392	PSR0300023012.mm.1	-3,44	0,005933	0,384945	Alternative 5' Donc	0,15
TC0300002901.mm.1	2,52	Ank2; Gm4392	JUC0300012000.mm.1	-4,13	0,023017	0,489186		
TC0300002901.mm.1	2,52	Ank2; Gm4392	JUC0300011997.mm.1	-4,36	0,000033	0,238339		
TC0300002901.mm.1	2,52	Ank2; Gm4392	JUC0300011998.mm.1	-4,94	0,010676	0,423096		
TC0300002901.mm.1	2,52	Ank2; Gm4392	PSR0300023007.mm.1	-8,14	0,005757	0,383101	Alternative 5' Donc	0,37
TC0400000015.mm.1	-1,89	Lyn; Gm11787	JUC0400000041.mm.1	5,87	0,017859	0,466232		
TC1600001540.mm.1	2,8	E130310I04Rik	JUC1600006602.mm.1	5,86	0,000234	0,28803		
TC1600001540.mm.1	2,8	E130310I04Rik	PSR1600012668.mm.1	2,25	0,02163	0,483552	Cassette Exon	0,25
TC1600001540.mm.1	2,8	E130310I04Rik	PSR1600012669.mm.1	-2,72	0,000741	0,307808	Cassette Exon	0,16
TC1600001540.mm.1	2,8	E130310I04Rik	PSR1600012665.mm.1	-3,16	0,011947	0,431753	Cassette Exon	0,2

TC0900002706.mm.1	-1,71		JUC0900012534.mm.1	5,86	0,005347	0,378286		
TC0500000096.mm.1	2,57	Pclo	PSR0500000843.mm.1	5,85	0,012412	0,435192	Cassette Exon	0,46
TC0500000096.mm.1	2,57	Pclo	JUC0500000488.mm.1	5,7	0,000739	0,307808		
TC0500000096.mm.1	2,57	Pclo	JUC0500000491.mm.1	2,15	0,000267	0,28803		
TC0500000096.mm.1	2,57	Pclo	PSR0500000853.mm.1	-2,32	0,007762	0,402774	Cassette Exon	0,39
TC0500000096.mm.1	2,57	Pclo	PSR0500000821.mm.1	-2,36	0,003817	0,35811	Cassette Exon	0,14
TC0500000096.mm.1	2,57	Pclo	PSR0500000854.mm.1	-2,43	0,006346	0,389463	Cassette Exon	0,27
TC0500000096.mm.1	2,57	Pclo	PSR0500000856.mm.1	-2,69	0,000768	0,308403	Cassette Exon	0,16
TC0500000096.mm.1	2,57	Pclo	PSR0500000822.mm.1	-2,93	0,049555	0,563325	Cassette Exon	0,17
TC0500000096.mm.1	2,57	Pclo	PSR0500000855.mm.1	-2,96	0,028778	0,508148	Cassette Exon	0,18
TC0500000096.mm.1	2,57	Pclo	JUC0500000500.mm.1	-3,05	0,001572	0,331411		
TC0500000096.mm.1	2,57	Pclo	JUC0500000476.mm.1	-3,24	0,028151	0,506347		
TC0500000096.mm.1	2,57	Pclo	JUC0500000492.mm.1	-4,05	0,012082	0,43255		
TC0500000096.mm.1	2,57	Pclo	JUC0500000497.mm.1	-4,87	0,007799	0,403089		
TC0500000096.mm.1	2,57	Pclo	JUC0500000493.mm.1	-6,28	0,018206	0,467586		
TC1200000019.mm.1	1,2		PSR1200000291.mm.1	5,85	0,001749	0,335996	Alternative 3' Acce	0,44
TC1200000019.mm.1	1,2		PSR1200000290.mm.1	5,36	0,003079	0,353892	Alternative 3' Acce	0,44
TC1200000019.mm.1	1,2		PSR1200000301.mm.1	2,75	0,031454	0,516773	Alternative 5' Donc	0,22
TC0200003666.mm.1	1,48	Scn3a	JUC0200015850.mm.1	5,85	0,000036	0,238339		
TC0200003666.mm.1	1,48	Scn3a	JUC0200015875.mm.1	5,04	0,001282	0,322251		
TC0200003666.mm.1	1,48	Scn3a	JUC0200015852.mm.1	4,29	0,001806	0,336311		
TC0200003666.mm.1	1,48	Scn3a	JUC0200015851.mm.1	4,09	0,004136	0,3623		
TC0200003666.mm.1	1,48	Scn3a	PSR0200031243.mm.1	3,56	0,046037	0,555624	Cassette Exon	0,29
TC0200003666.mm.1	1,48	Scn3a	JUC0200015876.mm.1	3,35	0,015697	0,455416		
TC0200003666.mm.1	1,48	Scn3a	PSR0200031208.mm.1	3,05	0,009684	0,417605	Cassette Exon	0,38
TC0200003666.mm.1	1,48	Scn3a	PSR0200031236.mm.1	2,83	0,006517	0,391382	Cassette Exon	0,17
TC0200003666.mm.1	1,48	Scn3a	PSR0200031213.mm.1	2,53	0,000404	0,297771	Cassette Exon	0,05
TC0200003666.mm.1	1,48	Scn3a	PSR0200031224.mm.1	-2,15	0,033643	0,523479	Alternative 5' Donc	0,14
TC0200003666.mm.1	1,48	Scn3a	PSR0200031249.mm.1	-2,44	0,026653	0,501823	Alternative 5' Donc	0,23
TC0200003666.mm.1	1,48	Scn3a	PSR0200031220.mm.1	-2,46	0,006356	0,389469	Alternative 3' Acce	0,23
TC0200003666.mm.1	1,48	Scn3a	PSR0200031221.mm.1	-2,67	0,042724	0,548097	Alternative 3' Acce	0,25
TC0X00002983.mm.1	-2,69	Tspan6	JUC0X00009352.mm.1	5,85	0,003279	0,354243		
TC0X00002983.mm.1	-2,69	Tspan6	PSR0X00018539.mm.1	2,18	0,000537	0,304044	Cassette Exon	0,11
TC0600002519.mm.1	2,18	Ctnna2	JUC0600010191.mm.1	5,84	0,034545	0,525975		
TC0600002519.mm.1	2,18	Ctnna2	PSR0600019565.mm.1	3,53	0,002161	0,344238	Cassette Exon	0,32
TC0600002519.mm.1	2,18	Ctnna2	JUC0600010194.mm.1	3,12	0,006192	0,387873		
TC0600002519.mm.1	2,18	Ctnna2	JUC0600010182.mm.1	2,34	0,044856	0,5526		
TC0600002519.mm.1	2,18	Ctnna2	PSR0600019569.mm.1	-2,01	0,031988	0,518264	Cassette Exon	0,1
TC0600002519.mm.1	2,18	Ctnna2	PSR0600019548.mm.1	-2,13	0,021978	0,485125	Cassette Exon	0,14
TC0600002519.mm.1	2,18	Ctnna2	PSR0600019573.mm.1	-2,18	0,024114	0,49322	Cassette Exon	0,1
TC0600002519.mm.1	2,18	Ctnna2	JUC0600010177.mm.1	-2,34	0,019239	0,473019		
TC1300001474.mm.1	2,7	Hecw1	JUC1300004945.mm.1	5,84	0,000162	0,272566		
TC1300001474.mm.1	2,7	Hecw1	PSR1300009432.mm.1	2,84	0,000245	0,28803	Cassette Exon	0,25
TC1300001474.mm.1	2,7	Hecw1	PSR1300009415.mm.1	2,23	0,037955	0,535303	Cassette Exon	0,2
TC1300001474.mm.1	2,7	Hecw1	JUC1300004941.mm.1	2,23	0,049188	0,562431		
TC1300001474.mm.1	2,7	Hecw1	PSR1300009411.mm.1	2,05	0,000112	0,267977	Cassette Exon	0,07
TC1300001474.mm.1	2,7	Hecw1	PSR1300009418.mm.1	-2,03	0,004608	0,369794	Cassette Exon	0,12
TC1300001474.mm.1	2,7	Hecw1	PSR1300009425.mm.1	-2,03	0,007454	0,400326	Alternative 3' Acce	0,1

TC1300001474.mm.1	2,7	Hecw1	PSR1300009403.mm.1	-2,43	0,007593	0,401712	Alternative 3' Acce	0,15
TC1300001474.mm.1	2,7	Hecw1	PSR1300009409.mm.1	-2,44	0,034343	0,525546	Cassette Exon	0,12
TC1300001474.mm.1	2,7	Hecw1	PSR1300009438.mm.1	-2,46	0,015997	0,45699	Alternative 5' Donc	0,15
TC1300001474.mm.1	2,7	Hecw1	JUC1300004942.mm.1	-2,74	0,019889	0,475806		
TC1300001474.mm.1	2,7	Hecw1	JUC1300004949.mm.1	-3,38	0,00586	0,383791		
TC0200003682.mm.1	1,08	Scn7a	JUC0200016003.mm.1	5,83	0,032472	0,519632		
TC0200003682.mm.1	1,08	Scn7a	JUC0200016015.mm.1	3,22	0,041234	0,544134		
TC1100000058.mm.1	1,27	Ap1b1	JUC1100000342.mm.1	5,82	0,026106	0,499897		
TC1100000058.mm.1	1,27	Ap1b1	JUC1100000343.mm.1	3,42	0,049986	0,564087		
TC1100000058.mm.1	1,27	Ap1b1	JUC1100000354.mm.1	-3,13	0,038422	0,536405		
TC0X00000525.mm.1	-1,86	Fhl1	JUC0X00001771.mm.1	5,81	0,046618	0,556809		
TC0X00000525.mm.1	-1,86	Fhl1	JUC0X00001772.mm.1	4,52	0,010934	0,424893		
TC0X00000525.mm.1	-1,86	Fhl1	JUC0X00001775.mm.1	2,82	0,04862	0,56119		
TC0X00000525.mm.1	-1,86	Fhl1	PSR0X00003465.mm.1	2,39	0,02007	0,476558	Cassette Exon	0,11
TC0X00000525.mm.1	-1,86	Fhl1	JUC0X00001764.mm.1	2,16	0,03715	0,533065		
TC1500000875.mm.1	1,66	Mapk8ip2	JUC1500003880.mm.1	5,8	0,006928	0,395156		
TC1500000875.mm.1	1,66	Mapk8ip2	PSR1500006924.mm.1	2,67	0,021011	0,480143	Cassette Exon	0,31
TC1500000875.mm.1	1,66	Mapk8ip2	PSR1500006928.mm.1	2,03	0,027513	0,504313	Cassette Exon	0,06
TC1500000875.mm.1	1,66	Mapk8ip2	PSR1500006934.mm.1	2,01	0,002987	0,35265	Alternative 5' Donc	0,12
TC1500000875.mm.1	1,66	Mapk8ip2	PSR1500006919.mm.1	-2,02	0,02707	0,502979	Alternative 3' Acce	0,11
TC1500000875.mm.1	1,66	Mapk8ip2	PSR1500006921.mm.1	-3,14	0,010842	0,424161	Alternative 3' Acce	0,34
TC1600001209.mm.1	-1,33	Zc3h7a	JUC1600005029.mm.1	5,79	0,044136	0,551024		
TC0500000422.mm.1	3,96	Crmp1	JUC0500002345.mm.1	5,76	0,000603	0,304044		
TC0500000422.mm.1	3,96	Crmp1	JUC0500002350.mm.1	5,37	0,008347	0,406435		
TC0500000422.mm.1	3,96	Crmp1	PSR0500004190.mm.1	2,78	0,002626	0,349612	Cassette Exon	0,23
TC0500000422.mm.1	3,96	Crmp1	PSR0500004201.mm.1	2,34	0,007253	0,39826	Cassette Exon	0,01
TC0500000422.mm.1	3,96	Crmp1	PSR0500004193.mm.1	2,2	0,000284	0,28803		
TC0500000422.mm.1	3,96	Crmp1	JUC0500002352.mm.1	2,15	0,024188	0,493294		
TC0500000422.mm.1	3,96	Crmp1	JUC0500002348.mm.1	-2,01	0,009284	0,414799		
TC0500000422.mm.1	3,96	Crmp1	PSR0500004192.mm.1	-2,14	0,009622	0,417315	Cassette Exon	0,11
TC0500000422.mm.1	3,96	Crmp1	PSR0500004189.mm.1	-2,22	0,029058	0,508962	Cassette Exon	0,09
TC0500000422.mm.1	3,96	Crmp1	JUC0500002354.mm.1	-2,99	0,012152	0,432901		
TC0500000422.mm.1	3,96	Crmp1	PSR0500004186.mm.1	-3,3	0,011355	0,427374	Cassette Exon	0,35
TC0500000422.mm.1	3,96	Crmp1	PSR0500004183.mm.1	-3,85	0,008052	0,404416	Cassette Exon	0,26
TC0500000422.mm.1	3,96	Crmp1	PSR0500004184.mm.1	-4,49	0,014909	0,451803	Cassette Exon	0,35
TC0500000422.mm.1	3,96	Crmp1	PSR0500004197.mm.1	-4,72	0,002221	0,345773	Alternative 5' Donc	0,42
TC0500000422.mm.1	3,96	Crmp1	JUC0500002346.mm.1	-5,13	0,012288	0,434238		
TC0500000422.mm.1	3,96	Crmp1	PSR0500004207.mm.1	-6,49	0,023513	0,490947	Alternative 5' Donc	0,45
TC0500000422.mm.1	3,96	Crmp1	PSR0500004185.mm.1	-7,44	0,006304	0,389124	Cassette Exon	0,35
TC0500000422.mm.1	3,96	Crmp1	PSR0500004195.mm.1	-7,55	0,015659	0,455364	Alternative 3' Acce	0,48
TC0500000422.mm.1	3,96	Crmp1	JUC0500002353.mm.1	-9	0,004117	0,3623		
TC0500000248.mm.1	4,06	Dpp6	JUC0500001085.mm.1	5,75	0,001805	0,336311		
TC0500000248.mm.1	4,06	Dpp6	PSR0500001890.mm.1	3,57	0,000375	0,291955	Cassette Exon	0,25
TC0500000248.mm.1	4,06	Dpp6	JUC0500001086.mm.1	3,05	0,00267	0,349612		
TC0500000248.mm.1	4,06	Dpp6	JUC0500001070.mm.1	3,04	0,003164	0,353901		
TC0500000248.mm.1	4,06	Dpp6	PSR0500001910.mm.1	2,98	0,001901	0,338712	Cassette Exon	0,41
TC0500000248.mm.1	4,06	Dpp6	PSR0500001905.mm.1	2,84	0,007181	0,397855	Cassette Exon	0,17
TC0500000248.mm.1	4,06	Dpp6	PSR0500001912.mm.1	2,48	0,002083	0,342452	Cassette Exon	0,02

TC0500000248.mm.1	4,06	Dpp6	JUC0500001083.mm.1	2,41	0,007902	0,403819		
TC0500000248.mm.1	4,06	Dpp6	JUC0500001090.mm.1	2,3	0,000677	0,304338		
TC0500000248.mm.1	4,06	Dpp6	PSR0500001892.mm.1	-2,2	0,008378	0,4065	Cassette Exon	0,01
TC0500000248.mm.1	4,06	Dpp6	PSR0500001895.mm.1	-2,31	0,003246	0,354243	Cassette Exon	0,09
TC0500000248.mm.1	4,06	Dpp6	PSR0500001917.mm.1	-2,4	0,035006	0,527412	Alternative 5' Donc	0,22
TC0500000248.mm.1	4,06	Dpp6	PSR0500001900.mm.1	-2,46	0,012902	0,438213	Cassette Exon	0,08
TC0500000248.mm.1	4,06	Dpp6	PSR0500001908.mm.1	-2,63	0,004408	0,366753	Cassette Exon	0,09
TC0500000248.mm.1	4,06	Dpp6	PSR0500001909.mm.1	-2,78	0,000781	0,309885	Cassette Exon	0,03
TC0500000248.mm.1	4,06	Dpp6	PSR0500001880.mm.1	-2,87	0,009778	0,417839	Cassette Exon	0,01
TC0500000248.mm.1	4,06	Dpp6	JUC0500001092.mm.1	-3,27	0,017609	0,464312		
TC0500000248.mm.1	4,06	Dpp6	PSR0500001893.mm.1	-3,53	0,008634	0,408463	Cassette Exon	0,37
TC0500000248.mm.1	4,06	Dpp6	PSR0500001885.mm.1	-3,57	0,002193	0,344674	Cassette Exon	0,25
TC0500000248.mm.1	4,06	Dpp6	PSR0500001881.mm.1	-3,67	0,008207	0,405909	Cassette Exon	0,18
TC0500000248.mm.1	4,06	Dpp6	PSR0500001891.mm.1	-3,75	0,004131	0,3623	Alternative 5' Donc	0,45
TC0500000248.mm.1	4,06	Dpp6	PSR0500001878.mm.1	-3,82	0,025306	0,49723	Cassette Exon	0,06
TC0500000248.mm.1	4,06	Dpp6	PSR0500001884.mm.1	-4,06	0,002164	0,344238	Cassette Exon	0,25
TC0500000248.mm.1	4,06	Dpp6	PSR0500001899.mm.1	-4,14	0,002031	0,341377	Cassette Exon	0,24
TC0500000248.mm.1	4,06	Dpp6	PSR0500001883.mm.1	-4,61	0,004181	0,362631	Cassette Exon	0,25
TC0500000248.mm.1	4,06	Dpp6	PSR0500001882.mm.1	-4,62	0,012077	0,43255	Cassette Exon	0,18
TC0500000248.mm.1	4,06	Dpp6	JUC0500001087.mm.1	-4,75	0,004209	0,363236		
TC0500000248.mm.1	4,06	Dpp6	PSR0500001876.mm.1	-5	0,001906	0,338854	Cassette Exon	0,28
TC0500000248.mm.1	4,06	Dpp6	JUC0500001091.mm.1	-5,24	0,002118	0,342919		
TC0500000248.mm.1	4,06	Dpp6	JUC0500001078.mm.1	-5,49	0,003727	0,357586		
TC0500000248.mm.1	4,06	Dpp6	PSR0500001906.mm.1	-5,64	0,002376	0,34829	Alternative 5' Donc	0,48
TC0500000248.mm.1	4,06	Dpp6	JUC0500001093.mm.1	-5,67	0,00118	0,319954		
TC0500000248.mm.1	4,06	Dpp6	PSR0500001879.mm.1	-5,8	0,007997	0,403853	Cassette Exon	0,07
TC0500000248.mm.1	4,06	Dpp6	JUC0500001066.mm.1	-5,83	0,004364	0,365487		
TC0500000248.mm.1	4,06	Dpp6	PSR0500001875.mm.1	-6,93	0,002399	0,348564	Cassette Exon	0,28
TC0500000248.mm.1	4,06	Dpp6	JUC0500001094.mm.1	-7,74	0,003055	0,353892		
TC0500000248.mm.1	4,06	Dpp6	JUC0500001095.mm.1	-8,6	0,003011	0,353447		
TC1100004187.mm.1	1,89	Nptx1	JUC1100020911.mm.1	5,75	0,007992	0,403853		
TC1100004187.mm.1	1,89	Nptx1	PSR1100039778.mm.1	5,23	0,008263	0,405976	Alternative 3' Acce	0,46
TC1200000636.mm.1	-1,3	Mnat1	PSR1200004387.mm.1	5,75	0,040144	0,541609	Alternative 5' Donc	0,4
TC1900000954.mm.1	-1,46	Pacs1	JUC1900004648.mm.1	5,74	0,006564	0,39213		
TC1400002723.mm.1	1,22	Uggt2	PSR1400020226.mm.1	5,73	0,033669	0,523549	Alternative 3' Acce	0,54
TC1400002723.mm.1	1,22	Uggt2	JUC1400011135.mm.1	-2,37	0,011779	0,430069		
TC0300002337.mm.1	1,25	Hcn3	JUC0300009346.mm.1	5,73	0,006175	0,387873		
TC0300002337.mm.1	1,25	Hcn3	JUC0300009351.mm.1	-2,61	0,033786	0,523767		
TC0300002212.mm.1	1,52	Glrb	JUC0300008732.mm.1	5,72	0,014713	0,45037		
TC0300002212.mm.1	1,52	Glrb	PSR0300016627.mm.1	-3,15	0,009291	0,414812	Cassette Exon	0,25
TC0500001128.mm.1	-1,01	Zfp932	JUC0500005225.mm.1	5,71	0,002907	0,352262		
TC0500001128.mm.1	-1,01	Zfp932	PSR0500009597.mm.1	2,12	0,002863	0,352207	Cassette Exon	0,28
TC1700001322.mm.1	1,03	Ppp1r21	JUC1700006587.mm.1	5,69	0,022167	0,485712		
TC1700001322.mm.1	1,03	Ppp1r21	JUC1700006589.mm.1	-2,65	0,019047	0,471941		
TC0700002065.mm.1	5,46	Brsk2; Mir3104	PSR0700018549.mm.1	5,67	0,001232	0,322251	Cassette Exon	0,11
TC0700002065.mm.1	5,46	Brsk2; Mir3104	JUC0700009868.mm.1	3,32	0,003378	0,354303		
TC0700002065.mm.1	5,46	Brsk2; Mir3104	JUC0700009887.mm.1	3,2	0,001367	0,32572		
TC0700002065.mm.1	5,46	Brsk2; Mir3104	PSR0700018558.mm.1	3,01	0,021114	0,480564		

TC0700002065.mm.1	5,46	Brsk2; Mir3104	PSR0700018541.mm.1	2,63	0,003414	0,355257	Cassette Exon	0,02
TC0700002065.mm.1	5,46	Brsk2; Mir3104	PSR0700018578.mm.1	-2,06	0,025408	0,497403		
TC0700002065.mm.1	5,46	Brsk2; Mir3104	JUC0700009879.mm.1	-2,12	0,00054	0,304044		
TC0700002065.mm.1	5,46	Brsk2; Mir3104	PSR0700018566.mm.1	-2,17	0,024636	0,49468	Cassette Exon	0,25
TC0700002065.mm.1	5,46	Brsk2; Mir3104	JUC0700009873.mm.1	-2,23	0,016641	0,459985		
TC0700002065.mm.1	5,46	Brsk2; Mir3104	JUC0700009892.mm.1	-2,37	0,014358	0,448293		
TC0700002065.mm.1	5,46	Brsk2; Mir3104	JUC0700009885.mm.1	-2,44	0,048469	0,560864		
TC0700002065.mm.1	5,46	Brsk2; Mir3104	PSR0700018582.mm.1	-2,78	0,000883	0,312378		
TC0700002065.mm.1	5,46	Brsk2; Mir3104	PSR0700018577.mm.1	-2,86	0,007786	0,402902		
TC0700002065.mm.1	5,46	Brsk2; Mir3104	PSR0700018568.mm.1	-3,22	0,003543	0,3562	Cassette Exon	0,14
TC0700002065.mm.1	5,46	Brsk2; Mir3104	PSR0700018572.mm.1	-3,3	0,033613	0,523309	Cassette Exon	0,25
TC0700002065.mm.1	5,46	Brsk2; Mir3104	PSR0700018574.mm.1	-3,34	0,00868	0,408815	Alternative 3' Acce	0,07
TC0700002065.mm.1	5,46	Brsk2; Mir3104	PSR0700018581.mm.1	-3,57	0,007133	0,397304		
TC0700002065.mm.1	5,46	Brsk2; Mir3104	PSR0700018575.mm.1	-3,75	0,00799	0,403853	Alternative 3' Acce	0,07
TC0700002065.mm.1	5,46	Brsk2; Mir3104	JUC0700009891.mm.1	-3,95	0,002598	0,349501		
TC0700002065.mm.1	5,46	Brsk2; Mir3104	JUC0700009895.mm.1	-4,04	0,044175	0,551087		
TC0700002065.mm.1	5,46	Brsk2; Mir3104	JUC0700009865.mm.1	-4,33	0,000952	0,313363		
TC0700002065.mm.1	5,46	Brsk2; Mir3104	JUC0700009875.mm.1	-4,46	0,008035	0,40434		
TC0700002065.mm.1	5,46	Brsk2; Mir3104	JUC0700009893.mm.1	-4,54	0,003281	0,354243		
TC0700002065.mm.1	5,46	Brsk2; Mir3104	JUC0700009890.mm.1	-4,59	0,020058	0,476514		
TC0700002065.mm.1	5,46	Brsk2; Mir3104	PSR0700018585.mm.1	-4,68	0,000322	0,288663		
TC0700002065.mm.1	5,46	Brsk2; Mir3104	PSR0700018589.mm.1	-4,72	0,002884	0,352207		
TC0700002065.mm.1	5,46	Brsk2; Mir3104	PSR0700018540.mm.1	-4,73	0,011409	0,427568	Cassette Exon	0,41
TC0700002065.mm.1	5,46	Brsk2; Mir3104	PSR0700018587.mm.1	-4,8	0,000615	0,304044		
TC0700002065.mm.1	5,46	Brsk2; Mir3104	PSR0700018588.mm.1	-4,86	0,002647	0,349612		
TC0700002065.mm.1	5,46	Brsk2; Mir3104	JUC0700009878.mm.1	-5,05	0,000268	0,28803		
TC0700002065.mm.1	5,46	Brsk2; Mir3104	PSR0700018586.mm.1	-5,09	0,002372	0,34829		
TC0700002065.mm.1	5,46	Brsk2; Mir3104	PSR0700018536.mm.1	-5,75	0,002145	0,343679	Cassette Exon	0,41
TC0700002065.mm.1	5,46	Brsk2; Mir3104	PSR0700018537.mm.1	-5,79	0,001804	0,336311	Cassette Exon	0,41
TC0700002065.mm.1	5,46	Brsk2; Mir3104	PSR0700018569.mm.1	-5,99	0,001863	0,337906	Cassette Exon	0,47
TC0700002065.mm.1	5,46	Brsk2; Mir3104	PSR0700018565.mm.1	-6,28	0,000302	0,288663	Cassette Exon	0,43
TC0700002065.mm.1	5,46	Brsk2; Mir3104	JUC0700009898.mm.1	-6,35	0,005816	0,383169		
TC0700002065.mm.1	5,46	Brsk2; Mir3104	PSR0700018556.mm.1	-7,11	0,000303	0,288663	Alternative 3' Acce	0,16
TC0700002065.mm.1	5,46	Brsk2; Mir3104	JUC0700009882.mm.1	-7,52	0,001515	0,330268		
TC0700002065.mm.1	5,46	Brsk2; Mir3104	JUC0700009900.mm.1	-7,78	0,003705	0,357266		
TC0700002065.mm.1	5,46	Brsk2; Mir3104	JUC0700009894.mm.1	-9,67	0,000559	0,304044		
TC0700002065.mm.1	5,46	Brsk2; Mir3104	JUC0700009867.mm.1	-10,47	0,002879	0,352207		
TC0700002065.mm.1	5,46	Brsk2; Mir3104	JUC0700009899.mm.1	-12,37	0,000769	0,308403		
TC0700004265.mm.1	-1,42	Xpo6	JUC0700018756.mm.1	5,67	0,008541	0,407996		
TC0700004265.mm.1	-1,42	Xpo6	PSR0700035510.mm.1	4,8	0,019512	0,474538	Alternative 3' Acce	0,38
TC0700004265.mm.1	-1,42	Xpo6	PSR0700035514.mm.1	3,53	0,027015	0,502979	Alternative 5' Donc	0,31
TC0700004265.mm.1	-1,42	Xpo6	PSR0700035513.mm.1	3,4	0,026762	0,502102	Alternative 5' Donc	0,31
TC0700004265.mm.1	-1,42	Xpo6	PSR0700035512.mm.1	3,37	0,018456	0,468671	Alternative 5' Donc	0,31
TC0700004265.mm.1	-1,42	Xpo6	PSR0700035509.mm.1	3,14	0,014992	0,452052	Cassette Exon	0,31
TC0700004265.mm.1	-1,42	Xpo6	JUC0700018744.mm.1	2,89	0,017989	0,466929		
TC0400002613.mm.1	1,6	Alg2	JUC0400011069.mm.1	5,67	0,029218	0,509521		
TC0400002613.mm.1	1,6	Alg2	PSR0400021341.mm.1	-2,13	0,01018	0,419711	Cassette Exon	0,05
TC0400002613.mm.1	1,6	Alg2	JUC0400011070.mm.1	-2,79	0,045465	0,554267		

TC0400001715.mm.1	1,26	Eif4g3	JUC0400007254.mm.1	5,66	0,029297	0,509874		
TC0400001715.mm.1	1,26	Eif4g3	JUC0400007293.mm.1	2,99	0,030225	0,512452		
TC1100000120.mm.1	-1,07	Abca13	PSR1100001270.mm.1	5,65	0,013546	0,44358	Cassette Exon	0,32
TC1100000120.mm.1	-1,07	Abca13	JUC1100000702.mm.1	5,07	0,000212	0,28803		
TC1100000120.mm.1	-1,07	Abca13	PSR1100001274.mm.1	4,99	0,011949	0,431753	Cassette Exon	0,42
TC1100000120.mm.1	-1,07	Abca13	PSR1100001275.mm.1	4,66	0,014742	0,450615	Cassette Exon	0,5
TC1100000120.mm.1	-1,07	Abca13	JUC1100000699.mm.1	4,5	0,030188	0,512365		
TC1100000120.mm.1	-1,07	Abca13	JUC1100000698.mm.1	4,48	0,011716	0,429969		
TC1100000120.mm.1	-1,07	Abca13	PSR1100001276.mm.1	3,95	0,019666	0,474887	Cassette Exon	0,41
TC1100000120.mm.1	-1,07	Abca13	PSR1100001272.mm.1	2,87	0,039405	0,539557	Cassette Exon	0,44
TC1100000120.mm.1	-1,07	Abca13	JUC1100000700.mm.1	2,13	0,049055	0,562009		
TC1100000120.mm.1	-1,07	Abca13	PSR1100001273.mm.1	2,04	0,049733	0,563552	Cassette Exon	0,28
TC1100000120.mm.1	-1,07	Abca13	JUC1100000695.mm.1	2,04	0,046824	0,557135		
TC1100000120.mm.1	-1,07	Abca13	JUC1100000703.mm.1	-2,24	0,028911	0,50858		
TC0900002681.mm.1	9,05	Scg3	JUC0900012436.mm.1	5,63	0,019564	0,474636		
TC0900002681.mm.1	9,05	Scg3	PSR0900022187.mm.1	-4,45	0,024924	0,4958	Cassette Exon	0,28
TC0900002681.mm.1	9,05	Scg3	PSR0900022174.mm.1	-12,72	0,00898	0,411895	Alternative 3' Acce	0,48
TC0900002681.mm.1	9,05	Scg3	JUC0900012430.mm.1	-16,03	0,001789	0,336311		
TC0300000878.mm.1	1,04	Pogz	JUC0300003530.mm.1	5,63	0,043223	0,549043		
TC0500000750.mm.1	1,9	Lphn3	JUC0500003716.mm.1	5,62	0,005772	0,383104		
TC0500000750.mm.1	1,9	Lphn3	JUC0500003696.mm.1	4	0,0126	0,435933		
TC0500000750.mm.1	1,9	Lphn3	JUC0500003710.mm.1	2,42	0,040563	0,542455		
TC0500000750.mm.1	1,9	Lphn3	PSR0500006768.mm.1	2,29	0,006424	0,389915		
TC0500000750.mm.1	1,9	Lphn3	JUC0500003685.mm.1	2,12	0,009135	0,413379		
TC0500000750.mm.1	1,9	Lphn3	PSR0500006705.mm.1	-2,03	0,030009	0,511687	Alternative 3' Acce	0,08
TC0500000750.mm.1	1,9	Lphn3	PSR0500006727.mm.1	-2,37	0,003824	0,358333	Alternative 3' Acce	0,16
TC0500000750.mm.1	1,9	Lphn3	PSR0500006717.mm.1	-2,46	0,0439	0,550649	Alternative 5' Donc	0,11
TC0500000750.mm.1	1,9	Lphn3	PSR0500006773.mm.1	-2,48	0,007154	0,397804	Alternative 5' Donc	0,14
TC0500000750.mm.1	1,9	Lphn3	PSR0500006713.mm.1	-2,6	0,006056	0,386642	Alternative 3' Acce	0,02
TC0500000750.mm.1	1,9	Lphn3	PSR0500006769.mm.1	-2,71	0,014255	0,447947	Alternative 5' Donc	0,27
TC0500000750.mm.1	1,9	Lphn3	PSR0500006750.mm.1	-3,08	0,008466	0,406626	Alternative 5' Donc	0,27
TC0500000750.mm.1	1,9	Lphn3	PSR0500006718.mm.1	-3,33	0,012595	0,435933	Alternative 5' Donc	0,11
TC0500000750.mm.1	1,9	Lphn3	JUC0500003694.mm.1	-3,73	0,024855	0,495511		
TC0500000750.mm.1	1,9	Lphn3	JUC0500003717.mm.1	-4,19	0,010509	0,422115		
TC0500000750.mm.1	1,9	Lphn3	JUC0500003726.mm.1	-5,76	0,002642	0,349612		
TC0500000750.mm.1	1,9	Lphn3	JUC0500003721.mm.1	-7,34	0,00032	0,288663		
TC0100002702.mm.1	-1,12	Slc16a14	JUC0100012450.mm.1	5,62	0,005541	0,380603		
TC0500001355.mm.1	1,29	Gm15800	JUC0500006507.mm.1	5,61	0,018097	0,46716		
TC0500001355.mm.1	1,29	Gm15800	JUC0500006501.mm.1	2,42	0,036975	0,532502		
TC0500001355.mm.1	1,29	Gm15800	JUC0500006500.mm.1	2,16	0,048362	0,56056		
TC0500001355.mm.1	1,29	Gm15800	JUC0500006483.mm.1	-2,08	0,033766	0,523689		
TC0500001355.mm.1	1,29	Gm15800	PSR0500011892.mm.1	-2,24	0,012982	0,439245	Alternative 3' Acce	0,15
TC1600000266.mm.1	-1,34	Klhl24	JUC1600001258.mm.1	5,61	0,01208	0,43255		
TC1600000266.mm.1	-1,34	Klhl24	JUC1600001256.mm.1	3,83	0,04047	0,542132		
TC0500002608.mm.1	1,15	Lnx1	JUC0500012574.mm.1	5,6	0,030901	0,514574		
TC0500002608.mm.1	1,15	Lnx1	JUC0500012576.mm.1	2,62	0,044751	0,55234		
TC0500002608.mm.1	1,15	Lnx1	JUC0500012567.mm.1	-2,2	0,024716	0,494838		
TC0600001790.mm.1	-1,09	Calcr	JUC0600007404.mm.1	5,6	0,027432	0,50398		

TC1100000324.mm.1	-1,18	Cpeb4	JUC1100001457.mm.1	5,6	0,012383	0,434939		
TC1100002000.mm.1	-1,34	Card14	JUC1100009918.mm.1	5,54	0,01021	0,419916		
TC1100002000.mm.1	-1,34	Card14	JUC1100009908.mm.1	2,59	0,004364	0,365487		
TC1100002000.mm.1	-1,34	Card14	JUC1100009925.mm.1	2,21	0,019029	0,471827		
TC0200004420.mm.1	1,37	Gm14091	JUC0200018892.mm.1	5,53	0,000717	0,306015		
TC0200004420.mm.1	1,37	Gm14091	PSR0200036913.mm.1	-3,02	0,000285	0,28803	Cassette Exon	0,19
TC1900000656.mm.1	1,54	Fam178a	JUC1900003107.mm.1	5,53	0,010897	0,424424		
TC0200004369.mm.1	-4,95	Actc1	PSR0200036505.mm.1	5,52	0,045589	0,554548	Cassette Exon	0,33
TC0200004369.mm.1	-4,95	Actc1	JUC0200018684.mm.1	2,24	0,04257	0,548012		
TC0200002398.mm.1	-1,09	Dlgap4	JUC0200009797.mm.1	5,49	0,003559	0,356223		
TC0200002398.mm.1	-1,09	Dlgap4	JUC0200009803.mm.1	4,9	0,014708	0,45037		
TC0200002398.mm.1	-1,09	Dlgap4	PSR0200019333.mm.1	3,53	0,005692	0,382434	Cassette Exon	0,29
TC0200002398.mm.1	-1,09	Dlgap4	JUC0200009804.mm.1	3,33	0,017019	0,461709		
TC0200002398.mm.1	-1,09	Dlgap4	PSR0200019341.mm.1	2,72	0,014968	0,452048	Cassette Exon	0,4
TC0200002398.mm.1	-1,09	Dlgap4	PSR0200019335.mm.1	2,6	0,042202	0,546983	Cassette Exon	0,19
TC1100000106.mm.1	-1,34	Ramp3	JUC1100000580.mm.1	5,49	0,002323	0,3467		
TC1100000106.mm.1	-1,34	Ramp3	PSR1100001104.mm.1	2,87	0,000785	0,309887	Cassette Exon	0,32
TC1300002517.mm.1	-1,06	Ptcd2	JUC1300009008.mm.1	5,49	0,008986	0,411965		
TC1300002517.mm.1	-1,06	Ptcd2	JUC1300009009.mm.1	2,69	0,017036	0,461729		
TC0900002153.mm.1	4,88	Abcg4	PSR0900017874.mm.1	5,46	0,014716	0,45037	Mutually Exclusive	0,27
TC0900002153.mm.1	4,88	Abcg4	JUC0900010067.mm.1	4,28	0,006751	0,393343		
TC0900002153.mm.1	4,88	Abcg4	JUC0900010063.mm.1	3,96	0,009532	0,416602		
TC0900002153.mm.1	4,88	Abcg4	PSR0900017860.mm.1	-2,02	0,020199	0,477145	Alternative 5' Donc	0,17
TC0900002153.mm.1	4,88	Abcg4	PSR0900017872.mm.1	-2,22	0,04331	0,549297	Mutually Exclusive	0,27
TC0900002153.mm.1	4,88	Abcg4	PSR0900017865.mm.1	-2,56	0,003355	0,354243	Alternative 5' Donc	0,25
TC0900002153.mm.1	4,88	Abcg4	PSR0900017853.mm.1	-2,97	0,000271	0,28803	Alternative 3' Acce	0,32
TC0900002153.mm.1	4,88	Abcg4	PSR0900017881.mm.1	-3,08	0,006086	0,386691	Cassette Exon	0,19
TC0900002153.mm.1	4,88	Abcg4	JUC0900010072.mm.1	-3,22	0,019648	0,474887		
TC0900002153.mm.1	4,88	Abcg4	PSR0900017885.mm.1	-3,51	0,023234	0,490032	Alternative 5' Donc	0,12
TC0900002153.mm.1	4,88	Abcg4	PSR0900017849.mm.1	-3,77	0,003153	0,353892	Cassette Exon	0,39
TC0900002153.mm.1	4,88	Abcg4	PSR0900017883.mm.1	-4,37	0,004828	0,371505	Alternative 5' Donc	0,15
TC0900002153.mm.1	4,88	Abcg4	PSR0900017878.mm.1	-4,68	0,010956	0,425117	Alternative 5' Donc	0,38
TC0900002153.mm.1	4,88	Abcg4	PSR0900017884.mm.1	-4,9	0,028701	0,508019	Alternative 5' Donc	0,15
TC0900002153.mm.1	4,88	Abcg4	JUC0900010069.mm.1	-7,47	0,008741	0,409629		
TC1300002525.mm.1	2,09	Cartpt	PSR1300016984.mm.1	5,46	0,012587	0,435924	Cassette Exon	0,28
TC1300002525.mm.1	2,09	Cartpt	PSR1300016982.mm.1	2,26	0,016493	0,459399	Alternative 3' Acce	0,2
TC0500003395.mm.1	1,19	Hip1	JUC0500016727.mm.1	5,46	0,003559	0,356223		
TC0600002481.mm.1	-1,33		JUC0600009981.mm.1	5,42	0,023257	0,490032		
TC1000000349.mm.1	1,46	Cdk19	JUC1000001270.mm.1	5,42	0,036792	0,532063		
TC1000000349.mm.1	1,46	Cdk19	JUC1000001283.mm.1	-3,54	0,004336	0,364898		
TC1300001330.mm.1	7,65	Hcn1	PSR1300008343.mm.1	5,41	0,024116	0,49322	Cassette Exon	0,49
TC1300001330.mm.1	7,65	Hcn1	JUC1300004341.mm.1	5,32	0,030246	0,512493		
TC1300001330.mm.1	7,65	Hcn1	JUC1300004344.mm.1	4,16	0,039553	0,539764		
TC1300001330.mm.1	7,65	Hcn1	JUC1300004342.mm.1	3,51	0,037429	0,533831		
TC1300001330.mm.1	7,65	Hcn1	PSR1300008345.mm.1	2,87	0,019548	0,474538	Cassette Exon	0,42
TC1300001330.mm.1	7,65	Hcn1	PSR1300008346.mm.1	2,74	0,049991	0,564097	Cassette Exon	0,33
TC1300001330.mm.1	7,65	Hcn1	PSR1300008352.mm.1	2,69	0,029146	0,509333	Alternative 3' Acce	0,19
TC1300001330.mm.1	7,65	Hcn1	JUC1300004340.mm.1	2,66	0,030482	0,513325		

TC1300001330.mm.1	7,65	Hcn1	JUC1300004343.mm.1	2,15	0,040764	0,543108		
TC1300001330.mm.1	7,65	Hcn1	PSR1300008348.mm.1	2,07	0,045038	0,553049	Cassette Exon	0,25
TC1300001330.mm.1	7,65	Hcn1	PSR1300008342.mm.1	-5,57	0,029033	0,508904	Cassette Exon	0,2
TC1300001330.mm.1	7,65	Hcn1	PSR1300008341.mm.1	-8,38	0,005889	0,383835	Cassette Exon	0,2
TC1700000464.mm.1	3,06	Pacsin1	JUC1700002103.mm.1	5,41	0,045525	0,554359		
TC1700000464.mm.1	3,06	Pacsin1	JUC1700002104.mm.1	4,61	0,043399	0,549592		
TC1700000464.mm.1	3,06	Pacsin1	PSR1700003895.mm.1	2,03	0,025758	0,498442	Cassette Exon	0,09
TC1700000464.mm.1	3,06	Pacsin1	PSR1700003891.mm.1	-3,11	0,016087	0,45751	Cassette Exon	0,24
TC1700000464.mm.1	3,06	Pacsin1	PSR1700003892.mm.1	-4,2	0,007841	0,403342	Cassette Exon	0,28
TC1700000464.mm.1	3,06	Pacsin1	PSR1700003887.mm.1	-4,44	0,002665	0,349612	Cassette Exon	0,28
TC1700000464.mm.1	3,06	Pacsin1	PSR1700003893.mm.1	-4,58	0,00426	0,363704	Alternative 3' Acce	0,48
TC1700000464.mm.1	3,06	Pacsin1	PSR1700003888.mm.1	-5,06	0,009001	0,412183	Cassette Exon	0,24
TC1000002531.mm.1	2,3	Atcay	JUC1000010397.mm.1	5,41	0,002478	0,349135		
TC1000002531.mm.1	2,3	Atcay	JUC1000010390.mm.1	4,45	0,00349	0,355638		
TC1000002531.mm.1	2,3	Atcay	JUC1000010393.mm.1	3,62	0,001229	0,322251		
TC1000002531.mm.1	2,3	Atcay	PSR1000018942.mm.1	2,35	0,002605	0,349501	Mutually Exclusive	0,16
TC1000002531.mm.1	2,3	Atcay	PSR1000018943.mm.1	-2,37	0,02048	0,478018	Mutually Exclusive	0,16
TC1000002531.mm.1	2,3	Atcay	PSR1000018934.mm.1	-2,77	0,015177	0,452699	Alternative 3' Acce	0,21
TC1000002531.mm.1	2,3	Atcay	JUC1000010387.mm.1	-3,03	0,020163	0,477086		
TC1700000672.mm.1	-1,23	Ncr3-ps	JUC1700003776.mm.1	5,41	0,009504	0,416602		
TC0700003609.mm.1	3,59	Ap3b2	JUC0700016221.mm.1	5,39	0,01215	0,432885		
TC0700003609.mm.1	3,59	Ap3b2	JUC0700016206.mm.1	5	0,003947	0,359922		
TC0700003609.mm.1	3,59	Ap3b2	JUC0700016224.mm.1	4,49	0,005565	0,380918		
TC0700003609.mm.1	3,59	Ap3b2	PSR0700030736.mm.1	4,06	0,001936	0,340349	Cassette Exon	0,4
TC0700003609.mm.1	3,59	Ap3b2	PSR0700030741.mm.1	3,32	0,002453	0,349135	Cassette Exon	0,43
TC0700003609.mm.1	3,59	Ap3b2	JUC0700016227.mm.1	3,26	0,017451	0,463886		
TC0700003609.mm.1	3,59	Ap3b2	JUC0700016212.mm.1	2,92	0,001524	0,330287		
TC0700003609.mm.1	3,59	Ap3b2	PSR0700030737.mm.1	2,7	0,006975	0,395722	Cassette Exon	0,15
TC0700003609.mm.1	3,59	Ap3b2	JUC0700016213.mm.1	2,63	0,039391	0,539461		
TC0700003609.mm.1	3,59	Ap3b2	JUC0700016217.mm.1	2,56	0,00109	0,317224		
TC0700003609.mm.1	3,59	Ap3b2	PSR0700030731.mm.1	-2,08	0,003551	0,356223	Alternative 3' Acce	0,19
TC0700003609.mm.1	3,59	Ap3b2	PSR0700030714.mm.1	-2,14	0,000998	0,316361	Cassette Exon	0,16
TC0700003609.mm.1	3,59	Ap3b2	PSR0700030719.mm.1	-2,15	0,004442	0,367059	Cassette Exon	0,2
TC0700003609.mm.1	3,59	Ap3b2	PSR0700030747.mm.1	-2,22	0,000315	0,288663		
TC0700003609.mm.1	3,59	Ap3b2	JUC0700016220.mm.1	-2,29	0,000244	0,28803		
TC0700003609.mm.1	3,59	Ap3b2	JUC0700016210.mm.1	-2,33	0,01288	0,438086		
TC0700003609.mm.1	3,59	Ap3b2	PSR0700030723.mm.1	-2,41	0,021281	0,481666	Cassette Exon	0,05
TC0700003609.mm.1	3,59	Ap3b2	PSR0700030729.mm.1	-2,43	0,047408	0,558302	Cassette Exon	0,04
TC0700003609.mm.1	3,59	Ap3b2	PSR0700030722.mm.1	-2,54	0,025991	0,499207	Cassette Exon	0,16
TC0700003609.mm.1	3,59	Ap3b2	JUC0700016207.mm.1	-2,54	0,004935	0,373093		
TC0700003609.mm.1	3,59	Ap3b2	PSR0700030744.mm.1	-2,59	0,002646	0,349612	Alternative 3' Acce	0,03
TC0700003609.mm.1	3,59	Ap3b2	PSR0700030715.mm.1	-2,71	0,000217	0,28803	Cassette Exon	0,23
TC0700003609.mm.1	3,59	Ap3b2	PSR0700030749.mm.1	-2,72	0,008757	0,409704	Alternative 5' Donc	0,15
TC0700003609.mm.1	3,59	Ap3b2	JUC0700016228.mm.1	-2,72	0,001396	0,325997		
TC0700003609.mm.1	3,59	Ap3b2	PSR0700030743.mm.1	-2,8	0,003278	0,354243	Cassette Exon	0,07
TC0700003609.mm.1	3,59	Ap3b2	PSR0700030739.mm.1	-2,81	0,003663	0,357266	Cassette Exon	0,45
TC0700003609.mm.1	3,59	Ap3b2	JUC0700016215.mm.1	-2,85	0,01127	0,426793		
TC0700003609.mm.1	3,59	Ap3b2	PSR0700030734.mm.1	-2,97	0,001322	0,324731	Alternative 3' Acce	0,17

TC0700003609.mm.1	3,59	Ap3b2	JUC0700016208.mm.1	-3,04	0,022811	0,488246		
TC0700003609.mm.1	3,59	Ap3b2	PSR0700030745.mm.1	-3,06	0,008152	0,40551	Cassette Exon	0,09
TC0700003609.mm.1	3,59	Ap3b2	PSR0700030718.mm.1	-3,15	0,003057	0,353892	Cassette Exon	0,36
TC0700003609.mm.1	3,59	Ap3b2	PSR0700030748.mm.1	-3,64	0,002224	0,345773	Alternative 5' Donc	0,23
TC0700003609.mm.1	3,59	Ap3b2	PSR0700030720.mm.1	-4,05	0,000441	0,297771	Cassette Exon	0,34
TC0700001287.mm.1	1,7	Dlg2	JUC0700005769.mm.1	5,39	0,011099	0,426		
TC0700001287.mm.1	1,7	Dlg2	JUC0700005756.mm.1	2,62	0,011481	0,428025		
TC0700001287.mm.1	1,7	Dlg2	JUC0700005751.mm.1	2,56	0,015814	0,455923		
TC0700001287.mm.1	1,7	Dlg2	PSR0700011153.mm.1	2,04	0,018782	0,470789	Cassette Exon	0,19
TC0700001287.mm.1	1,7	Dlg2	JUC0700005768.mm.1	-2,08	0,008844	0,410308		
TC0700001287.mm.1	1,7	Dlg2	JUC0700005766.mm.1	-2,12	0,038153	0,535762		
TC0700001287.mm.1	1,7	Dlg2	PSR0700011149.mm.1	-2,31	0,016534	0,459611	Alternative 5' Donc	0,2
TC0700001287.mm.1	1,7	Dlg2	PSR0700011169.mm.1	-2,54	0,002379	0,348564	Alternative 5' Donc	0,25
TC0700001287.mm.1	1,7	Dlg2	PSR0700011144.mm.1	-2,68	0,042443	0,547627	Alternative 3' Acce	0,24
TC0700001287.mm.1	1,7	Dlg2	PSR0700011137.mm.1	-2,74	0,024128	0,493245	Cassette Exon	0,17
TC0700001287.mm.1	1,7	Dlg2	JUC0700005758.mm.1	-2,83	0,041483	0,544745		
TC0700001607.mm.1	3,45	Tub; BC049265	JUC0700006842.mm.1	5,38	0,008944	0,411374		
TC0700001607.mm.1	3,45	Tub; BC049265	PSR0700013327.mm.1	4,41	0,000239	0,28803	Cassette Exon	0,52
TC0700001607.mm.1	3,45	Tub; BC049265	JUC0700006841.mm.1	3,42	0,013021	0,439354		
TC0700001607.mm.1	3,45	Tub; BC049265	PSR0700013328.mm.1	2,23	0,006813	0,39387	Cassette Exon	0,22
TC0700001607.mm.1	3,45	Tub; BC049265	PSR0700013323.mm.1	-2,41	0,001012	0,316361	Cassette Exon	0,13
TC0700001607.mm.1	3,45	Tub; BC049265	PSR0700013319.mm.1	-2,42	0,005727	0,382767	Cassette Exon	0,27
TC0700001607.mm.1	3,45	Tub; BC049265	PSR0700013318.mm.1	-2,91	0,02406	0,493026		
TC0700001607.mm.1	3,45	Tub; BC049265	PSR0700013311.mm.1	-2,92	0,038323	0,536178		
TC0700001607.mm.1	3,45	Tub; BC049265	PSR0700013316.mm.1	-3,49	0,009028	0,412682	Cassette Exon	0,37
TC0700001607.mm.1	3,45	Tub; BC049265	JUC0700006836.mm.1	-4,95	0,006744	0,393343		
TC1500002162.mm.1	-1	Col2a1	JUC1500009947.mm.1	5,38	0,037805	0,534746		
TC1500002162.mm.1	-1	Col2a1	JUC1500009954.mm.1	4,26	0,01251	0,435429		
TC1500002162.mm.1	-1	Col2a1	PSR1500017455.mm.1	3,7	0,029053	0,508954	Cassette Exon	0,34
TC1500002162.mm.1	-1	Col2a1	JUC1500009946.mm.1	3,06	0,023417	0,490343		
TC1500002162.mm.1	-1	Col2a1	PSR1500017457.mm.1	2,56	0,011387	0,427549	Cassette Exon	0,31
TC1500002162.mm.1	-1	Col2a1	PSR1500017518.mm.1	2,43	0,025581	0,497808	Cassette Exon	0,12
TC1500002162.mm.1	-1	Col2a1	JUC1500009979.mm.1	-2,11	0,01703	0,461729		
TC1300002715.mm.1	-1,53	Itga2	JUC1300009852.mm.1	5,38	0,029903	0,51142		
TC1300002715.mm.1	-1,53	Itga2	JUC1300009865.mm.1	3,02	0,034542	0,525975		
TC0600001653.mm.1	-1	Plekha5	JUC0600006922.mm.1	5,38	0,001214	0,322251		
TC0600001653.mm.1	-1	Plekha5	JUC0600006908.mm.1	3,11	0,006832	0,393893		
TC0600003538.mm.1	-1,23	Atp6v0a4	PSR0600016505.mm.1	5,37	0,008606	0,40841	Alternative 5' Donc	0,31
TC0600003538.mm.1	-1,23	Atp6v0a4	PSR0600016504.mm.1	2,04	0,037282	0,533572	Cassette Exon	0,19
TC1400000265.mm.1	-1,11	Dnah12	JUC1400001226.mm.1	5,37	0,036265	0,530445		
TC0700001252.mm.1	-1,36	Nox4	JUC0700005591.mm.1	5,37	0,010965	0,425117		
TC1600000558.mm.1	-1,35	Tmem39a	JUC1600002793.mm.1	5,34	0,010758	0,423859		
TC0900000730.mm.1	-1,07	Stoml1	JUC0900002852.mm.1	5,34	0,003991	0,360753		
TC1800001625.mm.1	1,6	Rnf165	JUC1800006512.mm.1	5,33	0,011403	0,427549		
TC1800001625.mm.1	1,6	Rnf165	PSR1800011691.mm.1	4,18	0,017794	0,465621		
TC1800001625.mm.1	1,6	Rnf165	JUC1800006515.mm.1	-2,32	0,037841	0,534803		
TC1800001625.mm.1	1,6	Rnf165	JUC1800006510.mm.1	-2,69	0,002972	0,352501		
TC0400003890.mm.1	1,1	Ddi2; Rsc1a1	JUC0400016816.mm.1	5,33	0,004675	0,370194		

TC0200005412.mm.1	-1,64		JUC0200023765.mm.1	5,32	0,000646	0,304044		
TC0600002580.mm.1	5,66	Vax2os	PSR0600020053.mm.1	5,3	0,020008	0,476356	Intron Retention	0,74
TC0600002580.mm.1	5,66	Vax2os	PSR0600020060.mm.1	3	0,030445	0,513217	Cassette Exon	0,29
TC0600002580.mm.1	5,66	Vax2os	PSR0600020059.mm.1	2,47	0,036103	0,529729		
TC0600002580.mm.1	5,66	Vax2os	PSR0600020055.mm.1	2,16	0,023882	0,492304	Cassette Exon	0,22
TC0600002580.mm.1	5,66	Vax2os	PSR0600020046.mm.1	-2,06	0,034784	0,526754	Cassette Exon	0,01
TC0600002580.mm.1	5,66	Vax2os	PSR0600020061.mm.1	-2,17	0,009404	0,415782	Alternative 5' Donc	0,27
TC0600002580.mm.1	5,66	Vax2os	PSR0600020048.mm.1	-2,33	0,028467	0,507299	Alternative 3' Acce	0,21
TC0600002580.mm.1	5,66	Vax2os	PSR0600020037.mm.1	-2,5	0,029591	0,510513	Cassette Exon	0,25
TC0600002580.mm.1	5,66	Vax2os	PSR0600020067.mm.1	-2,55	0,030858	0,514381	Cassette Exon	0,14
TC0600002580.mm.1	5,66	Vax2os	JUC0600010412.mm.1	-3,16	0,024592	0,494446		
TC0600002580.mm.1	5,66	Vax2os	JUC0600010408.mm.1	-4,01	0,012283	0,434168		
TC0600002580.mm.1	5,66	Vax2os	PSR0600020066.mm.1	-4,02	0,010109	0,418998	Alternative 5' Donc	0,48
TC0600002580.mm.1	5,66	Vax2os	JUC0600010418.mm.1	-4,76	0,003316	0,354243		
TC0600002580.mm.1	5,66	Vax2os	JUC0600010413.mm.1	-5,58	0,006024	0,386586		
TC0600002580.mm.1	5,66	Vax2os	PSR0600020065.mm.1	-6,16	0,006083	0,386691	Alternative 5' Donc	0,48
TC0600002580.mm.1	5,66	Vax2os	JUC0600010407.mm.1	-7,6	0,00391	0,359698		
TC0600002580.mm.1	5,66	Vax2os	JUC0600010405.mm.1	-8,56	0,006077	0,386691		
TC0700004095.mm.1	-1,38	Sbf2	JUC0700017805.mm.1	5,3	0,001949	0,34055		
TC0700004095.mm.1	-1,38	Sbf2	JUC0700017795.mm.1	3,68	0,027196	0,503373		
TC0700004095.mm.1	-1,38	Sbf2	JUC0700017828.mm.1	3,5	0,005416	0,379167		
TC0700004095.mm.1	-1,38	Sbf2	PSR0700033878.mm.1	3,02	0,009709	0,417826	Cassette Exon	0,18
TC0700004095.mm.1	-1,38	Sbf2	JUC0700017833.mm.1	2,44	0,016477	0,459352		
TC0700004095.mm.1	-1,38	Sbf2	PSR0700033879.mm.1	2,42	0,008185	0,40568	Cassette Exon	0,18
TC0700004095.mm.1	-1,38	Sbf2	JUC0700017838.mm.1	-3,97	0,000296	0,288663		
TC0600000925.mm.1	1,4	Dctn1	JUC0600003554.mm.1	5,3	0,012985	0,439245		
TC0600000925.mm.1	1,4	Dctn1	PSR0600006993.mm.1	-2,04	0,000049	0,24627	Cassette Exon	0,07
TC1400000261.mm.1	-1,08	Dennd6a; Fam116a	JUC1400001177.mm.1	5,3	0,017113	0,46232		
TC1400000261.mm.1	-1,08	Dennd6a; Fam116a	JUC1400001195.mm.1	-2,1	0,047624	0,558743		
TC0100003876.mm.1	-1,14	Tex30	PSR0100018283.mm.1	5,29	0,007962	0,403853	Cassette Exon	0,23
TC1400002572.mm.1	1,12	Klhl1	JUC1400010685.mm.1	5,29	0,010142	0,419347		
TC0500003238.mm.1	1,03	Clip1	JUC0500015856.mm.1	5,29	0,005067	0,375225		
TC0500003238.mm.1	1,03	Clip1	JUC0500015859.mm.1	3,93	0,002161	0,344238		
TC0500003238.mm.1	1,03	Clip1	JUC0500015858.mm.1	2,76	0,024046	0,493026		
TC1100003410.mm.1	-1,35	Aatf	JUC1100016312.mm.1	5,29	0,00651	0,391307		
TC1100002493.mm.1	3,21	Kcnip1	JUC1100012078.mm.1	5,28	0,002965	0,352501		
TC1100002493.mm.1	3,21	Kcnip1	JUC1100012079.mm.1	2,17	0,039234	0,538857		
TC1100002493.mm.1	3,21	Kcnip1	PSR1100023236.mm.1	-2,31	0,013973	0,446239	Cassette Exon	0,12
TC1100002493.mm.1	3,21	Kcnip1	PSR1100023229.mm.1	-3,21	0,008427	0,406611	Intron Retention	0,55
TC1100002493.mm.1	3,21	Kcnip1	PSR1100023241.mm.1	-3,7	0,008764	0,409781	Cassette Exon	0,27
TC1100002493.mm.1	3,21	Kcnip1	PSR1100023225.mm.1	-3,75	0,016703	0,460124	Alternative 3' Acce	0,45
TC1100002493.mm.1	3,21	Kcnip1	PSR1100023233.mm.1	-4,07	0,008421	0,406611	Alternative 5' Donc	0,39
TC1100002493.mm.1	3,21	Kcnip1	PSR1100023220.mm.1	-4,14	0,010104	0,418998	Alternative 3' Acce	0,48
TC1100002493.mm.1	3,21	Kcnip1	PSR1100023231.mm.1	-4,27	0,00621	0,387912	Intron Retention	0,74
TC1100002493.mm.1	3,21	Kcnip1	PSR1100023226.mm.1	-4,28	0,004381	0,3661	Alternative 3' Acce	0,47
TC1100002493.mm.1	3,21	Kcnip1	PSR1100023240.mm.1	-5,61	0,004774	0,371044	Cassette Exon	0,28
TC1100002493.mm.1	3,21	Kcnip1	PSR1100023235.mm.1	-5,79	0,006961	0,395722	Alternative 3' Acce	0,27
TC1100002493.mm.1	3,21	Kcnip1	PSR1100023242.mm.1	-6,5	0,003684	0,357266	Cassette Exon	0,27

TC1100002493.mm.1	3,21	Kcnip1	PSR1100023237.mm.1	-6,6	0,003643	0,356919	Alternative 5' Donc		0,27
TC0800000955.mm.1	1,62	Cacna1a	JUC0800003798.mm.1	5,28	0,00224	0,34578			
TC0800000955.mm.1	1,62	Cacna1a	JUC0800003797.mm.1	3,65	0,039514	0,539762			
TC0800000955.mm.1	1,62	Cacna1a	JUC0800003773.mm.1	3,28	0,001831	0,337331			
TC0800000955.mm.1	1,62	Cacna1a	JUC0800003779.mm.1	3,27	0,00437	0,365608			
TC0800000955.mm.1	1,62	Cacna1a	JUC0800003781.mm.1	2,87	0,01735	0,46355			
TC0800000955.mm.1	1,62	Cacna1a	PSR0800007104.mm.1	2,81	0,015082	0,452418	Cassette Exon		0,27
TC0800000955.mm.1	1,62	Cacna1a	JUC0800003783.mm.1	2,69	0,032375	0,519221			
TC0800000955.mm.1	1,62	Cacna1a	JUC0800003770.mm.1	2,55	0,003348	0,354243			
TC0800000955.mm.1	1,62	Cacna1a	PSR0800007111.mm.1	2,44	0,009293	0,414813	Cassette Exon		0,13
TC0800000955.mm.1	1,62	Cacna1a	JUC0800003796.mm.1	2,36	0,004615	0,369794			
TC0800000955.mm.1	1,62	Cacna1a	JUC0800003780.mm.1	2,35	0,017833	0,46608			
TC0800000955.mm.1	1,62	Cacna1a	PSR0800007114.mm.1	2,34	0,003342	0,354243	Cassette Exon		0,29
TC0800000955.mm.1	1,62	Cacna1a	JUC0800003771.mm.1	2,23	0,002423	0,34896			
TC0800000955.mm.1	1,62	Cacna1a	JUC0800003772.mm.1	2,01	0,047572	0,558743			
TC0800000955.mm.1	1,62	Cacna1a	PSR0800007155.mm.1	-2,14	0,049717	0,563531	Cassette Exon		0,16
TC0800000955.mm.1	1,62	Cacna1a	PSR0800007148.mm.1	-2,31	0,045024	0,553022	Cassette Exon		0,19
TC0800000955.mm.1	1,62	Cacna1a	JUC0800003820.mm.1	-2,42	0,030935	0,514624			
TC0800000955.mm.1	1,62	Cacna1a	JUC0800003813.mm.1	-2,66	0,008184	0,40568			
TC0800000955.mm.1	1,62	Cacna1a	PSR0800007152.mm.1	-2,99	0,002978	0,352501	Cassette Exon		0,2
TC0800000955.mm.1	1,62	Cacna1a	JUC0800003803.mm.1	-3,86	0,003514	0,355749			
TC0900003000.mm.1	-1,01	Col6a4; Dvwa	JUC0900013928.mm.1	5,28	0,002647	0,349612			
TC0500001628.mm.1	2,04	Actl6b	JUC0500008060.mm.1	5,26	0,000539	0,304044			
TC0500001628.mm.1	2,04	Actl6b	JUC0500008057.mm.1	4,95	0,022779	0,488083			
TC0500001628.mm.1	2,04	Actl6b	JUC0500008059.mm.1	3,88	0,017108	0,46232			
TC0500001628.mm.1	2,04	Actl6b	PSR0500014787.mm.1	2,28	0,002862	0,352207	Mutually Exclusive		0,24
TC0500001628.mm.1	2,04	Actl6b	PSR0500014789.mm.1	-2,23	0,020577	0,478502	Mutually Exclusive		0,24
TC0500001628.mm.1	2,04	Actl6b	PSR0500014769.mm.1	-2,59	0,00371	0,357512	Intron Retention		0,24
TC0500001628.mm.1	2,04	Actl6b	JUC0500008067.mm.1	-2,96	0,022815	0,488253			
TC0500001628.mm.1	2,04	Actl6b	JUC0500008066.mm.1	-3,95	0,005429	0,379581			
TC1200001643.mm.1	1,55	Tspan13	JUC1200006326.mm.1	5,26	0,03554	0,528764			
TC0900000223.mm.1	3,35	Bbs9	JUC0900000925.mm.1	5,25	0,008698	0,409002			
TC0900000223.mm.1	3,35	Bbs9	PSR0900001729.mm.1	-2,5	0,013595	0,443703	Alternative 5' Donc		0,2
TC0900000223.mm.1	3,35	Bbs9	PSR0900001727.mm.1	-2,57	0,003616	0,356223	Alternative 3' Acce		0,22
TC0900000223.mm.1	3,35	Bbs9	JUC0900000929.mm.1	-2,68	0,011686	0,429961			
TC0900000223.mm.1	3,35	Bbs9	JUC0900000918.mm.1	-2,83	0,00544	0,379743			
TC0900000223.mm.1	3,35	Bbs9	JUC0900000915.mm.1	-2,88	0,000974	0,314364			
TC0900000223.mm.1	3,35	Bbs9	PSR0900001703.mm.1	-2,99	0,008314	0,406313	Cassette Exon		0,35
TC0900000223.mm.1	3,35	Bbs9	PSR0900001697.mm.1	-3,12	0,005315	0,378059	Cassette Exon		0,44
TC0900000223.mm.1	3,35	Bbs9	JUC0900000899.mm.1	-3,19	0,012297	0,434295			
TC0900000223.mm.1	3,35	Bbs9	PSR0900001734.mm.1	-3,3	0,002391	0,348564	Intron Retention		0,38
TC0900000223.mm.1	3,35	Bbs9	JUC0900000916.mm.1	-3,34	0,000955	0,313363			
TC0900000223.mm.1	3,35	Bbs9	JUC0900000923.mm.1	-3,59	0,004018	0,361353			
TC0900000223.mm.1	3,35	Bbs9	PSR0900001723.mm.1	-4,04	0,048943	0,5618	Alternative 3' Acce		0,42
TC0900000223.mm.1	3,35	Bbs9	PSR0900001698.mm.1	-4,71	0,002728	0,349612	Cassette Exon		0,45
TC0900000223.mm.1	3,35	Bbs9	PSR0900001693.mm.1	-4,98	0,004015	0,361291	Alternative 3' Acce		0,46
TC0900000223.mm.1	3,35	Bbs9	JUC0900000926.mm.1	-5,19	0,041811	0,545586			
TC0900000223.mm.1	3,35	Bbs9	JUC0900000921.mm.1	-9,15	0,000457	0,298999			

TC1000000924.mm.1	-2,26	Gm4924	PSR1000007364.mm.1	5,25	0,005214	0,377078	Cassette Exon	0,32
TC1000000924.mm.1	-2,26	Gm4924	PSR1000007363.mm.1	3,72	0,04038	0,541974	Cassette Exon	0,29
TC1000000924.mm.1	-2,26	Gm4924	PSR1000007355.mm.1	3,32	0,019897	0,47586	Cassette Exon	0,23
TC1000000924.mm.1	-2,26	Gm4924	PSR1000007359.mm.1	3,19	0,025321	0,497278	Alternative 3' Acce	0,28
TC1000000924.mm.1	-2,26	Gm4924	PSR1000007352.mm.1	3,1	0,049222	0,562499	Cassette Exon	0,16
TC1000000924.mm.1	-2,26	Gm4924	PSR1000007357.mm.1	3,03	0,005045	0,374724	Cassette Exon	0,24
TC1000000924.mm.1	-2,26	Gm4924	PSR1000007350.mm.1	2,98	0,024463	0,493992	Cassette Exon	0,24
TC1000000924.mm.1	-2,26	Gm4924	PSR1000007360.mm.1	2,73	0,027977	0,505748	Alternative 3' Acce	0,28
TC1000000924.mm.1	-2,26	Gm4924	PSR1000007356.mm.1	2,5	0,042413	0,547627	Cassette Exon	0,1
TC1000000924.mm.1	-2,26	Gm4924	PSR1000007366.mm.1	2,38	0,04381	0,550456	Cassette Exon	0,18
TC0800001650.mm.1	1,03		PSR0800013555.mm.1	5,25	0,000241	0,28803	Cassette Exon	0,28
TC0200003171.mm.1	2,11	Camsap1	JUC0200013325.mm.1	5,25	0,003888	0,359225		
TC0200003171.mm.1	2,11	Camsap1	PSR0200026349.mm.1	2,14	0,005781	0,383104	Cassette Exon	0,25
TC0200003171.mm.1	2,11	Camsap1	PSR0200026365.mm.1	-2,13	0,019696	0,474887	Cassette Exon	0,09
TC0200003171.mm.1	2,11	Camsap1	PSR0200026362.mm.1	-2,46	0,005357	0,378582	Cassette Exon	0,14
TC0200003171.mm.1	2,11	Camsap1	PSR0200026332.mm.1	-2,57	0,037703	0,534532	Alternative 5' Donc	0,19
TC0200003171.mm.1	2,11	Camsap1	JUC0200013304.mm.1	-2,58	0,006113	0,387239		
TC0200003171.mm.1	2,11	Camsap1	JUC0200013320.mm.1	-2,59	0,023724	0,491556		
TC0400003958.mm.1	1,36	Tnfrsf8	PSR0400032839.mm.1	5,22	0,003412	0,355242	Cassette Exon	0,32
TC0400003958.mm.1	1,36	Tnfrsf8	PSR0400032835.mm.1	-2,14	0,003178	0,354243	Cassette Exon	0,12
TC1700002177.mm.1	1,3	Ttbk1	JUC1700010871.mm.1	5,22	0,002076	0,342181		
TC1700002177.mm.1	1,3	Ttbk1	JUC1700010878.mm.1	4,28	0,008583	0,40821		
TC1700002177.mm.1	1,3	Ttbk1	JUC1700010869.mm.1	-2,02	0,017608	0,464312		
TC1700002177.mm.1	1,3	Ttbk1	JUC1700010880.mm.1	-2,31	0,004229	0,363295		
TC1700002177.mm.1	1,3	Ttbk1	JUC1700010879.mm.1	-2,36	0,006243	0,388361		
TC1400000684.mm.1	-1,2	Trav6d-6; Trav11; Tnpo3;	JUC1400002983.mm.1	5,21	0,0371	0,532885		
TC1400000684.mm.1	-1,2	Trav6d-6; Trav11; Tnpo3;	PSR1400005424.mm.1	-2,19	0,029677	0,510918	Cassette Exon	0,1
TC1400000684.mm.1	-1,2	Trav6d-6; Trav11; Tnpo3;	JUC1400002988.mm.1	-2,6	0,013082	0,4396		
TC1400000684.mm.1	-1,2	Trav6d-6; Trav11; Tnpo3;	JUC1400002976.mm.1	-3,85	0,000402	0,297771		
TC1100003110.mm.1	1,26	Camta2	JUC1100015057.mm.1	5,21	0,003327	0,354243		
TC1100003110.mm.1	1,26	Camta2	JUC1100015058.mm.1	5,17	0,000842	0,311886		
TC1100003110.mm.1	1,26	Camta2	JUC1100015037.mm.1	-2,77	0,035795	0,529153		
TC1400000292.mm.1	7,17	Lrtm1	JUC1400001401.mm.1	5,2	0,00002	0,211693		
TC1400000292.mm.1	7,17	Lrtm1	PSR1400002365.mm.1	2,97	0,001247	0,322251	Cassette Exon	0,32
TC1400000292.mm.1	7,17	Lrtm1	PSR1400002372.mm.1	-2,14	0,003677	0,357266		
TC1400000292.mm.1	7,17	Lrtm1	PSR1400002367.mm.1	-2,34	0,006425	0,389922	Alternative 3' Acce	0,26
TC1400000292.mm.1	7,17	Lrtm1	PSR1400002363.mm.1	-4,18	0,000689	0,304792	Alternative 3' Acce	0,48
TC1400000292.mm.1	7,17	Lrtm1	JUC1400001403.mm.1	-5,44	0,042468	0,547627		
TC1400000292.mm.1	7,17	Lrtm1	JUC1400001402.mm.1	-6,48	0,007325	0,399002		
TC1700002438.mm.1	-1,19	Ptprm	JUC1700012121.mm.1	5,2	0,019813	0,475268		
TC0700002827.mm.1	-1,38	Pdcd2l	JUC0700013497.mm.1	5,2	0,035968	0,529457		
TC1900001726.mm.1	1,45	Hspa12a	PSR1900015284.mm.1	5,17	0,005673	0,382036	Alternative 3' Acce	0,33
TC1900001726.mm.1	1,45	Hspa12a	PSR1900015289.mm.1	3,74	0,02023	0,477145	Cassette Exon	0,31
TC1900001726.mm.1	1,45	Hspa12a	PSR1900015292.mm.1	2,95	0,011487	0,428148	Cassette Exon	0,18
TC1900001726.mm.1	1,45	Hspa12a	PSR1900015287.mm.1	2,78	0,024722	0,494838	Cassette Exon	0,25
TC1900001726.mm.1	1,45	Hspa12a	JUC1900008506.mm.1	2,3	0,011836	0,43053		
TC1900001726.mm.1	1,45	Hspa12a	PSR1900015298.mm.1	-2,02	0,024692	0,494805	Alternative 5' Donc	0,11
TC0400004071.mm.1	2,99	Camta1	PSR0400033872.mm.1	5,16	0,000166	0,272566	Alternative 3' Acce	0,42

TC0400004071.mm.1	2,99	Camta1	JUC0400017672.mm.1	3,14	0,023842	0,491997		
TC0400004071.mm.1	2,99	Camta1	JUC0400017658.mm.1	2,76	0,003316	0,354243		
TC0400004071.mm.1	2,99	Camta1	PSR0400033859.mm.1	2,47	0,001486	0,329081	Cassette Exon	0,16
TC0400004071.mm.1	2,99	Camta1	JUC0400017683.mm.1	2,04	0,025326	0,49728		
TC0400004071.mm.1	2,99	Camta1	PSR0400033898.mm.1	-2,06	0,032506	0,519769	Cassette Exon	0,5
TC0400004071.mm.1	2,99	Camta1	JUC0400017681.mm.1	-2,16	0,024726	0,494838		
TC0400004071.mm.1	2,99	Camta1	PSR0400033889.mm.1	-2,25	0,028609	0,507714	Cassette Exon	0,26
TC0400004071.mm.1	2,99	Camta1	JUC0400017651.mm.1	-2,4	0,047519	0,558686		
TC0400004071.mm.1	2,99	Camta1	PSR0400033899.mm.1	-2,42	0,012058	0,432426	Cassette Exon	0,5
TC0400004071.mm.1	2,99	Camta1	PSR0400033890.mm.1	-2,42	0,031182	0,515739	Cassette Exon	0,19
TC0400004071.mm.1	2,99	Camta1	PSR0400033893.mm.1	-2,45	0,03396	0,524252	Cassette Exon	0,28
TC0400004071.mm.1	2,99	Camta1	PSR0400033897.mm.1	-2,5	0,018733	0,470733	Cassette Exon	0,5
TC0400004071.mm.1	2,99	Camta1	JUC0400017666.mm.1	-2,61	0,019282	0,473074		
TC0400004071.mm.1	2,99	Camta1	JUC0400017682.mm.1	-2,64	0,031285	0,516126		
TC0400004071.mm.1	2,99	Camta1	PSR0400033909.mm.1	-2,66	0,015046	0,45215	Alternative 5' Donc	0,16
TC0400004071.mm.1	2,99	Camta1	PSR0400033885.mm.1	-2,77	0,027644	0,504658	Cassette Exon	0,19
TC0400004071.mm.1	2,99	Camta1	PSR0400033879.mm.1	-2,96	0,032478	0,519641	Cassette Exon	0,18
TC0400004071.mm.1	2,99	Camta1	JUC0400017691.mm.1	-3,08	0,021119	0,480595		
TC0400004071.mm.1	2,99	Camta1	PSR0400033878.mm.1	-3,23	0,033316	0,522414	Alternative 5' Donc	0,22
TC0400004071.mm.1	2,99	Camta1	PSR0400033894.mm.1	-3,39	0,029381	0,509995	Cassette Exon	0,46
TC0400004071.mm.1	2,99	Camta1	JUC0400017665.mm.1	-3,46	0,010841	0,424161		
TC0400004071.mm.1	2,99	Camta1	JUC0400017684.mm.1	-3,56	0,001354	0,325427		
TC0400004071.mm.1	2,99	Camta1	PSR0400033888.mm.1	-3,67	0,015289	0,453345	Cassette Exon	0,39
TC0400004071.mm.1	2,99	Camta1	PSR0400033881.mm.1	-3,93	0,001208	0,322251	Cassette Exon	0,46
TC0400004071.mm.1	2,99	Camta1	JUC0400017667.mm.1	-4,41	0,014517	0,449545		
TC0400004071.mm.1	2,99	Camta1	JUC0400017692.mm.1	-4,55	0,019218	0,472971		
TC0400004071.mm.1	2,99	Camta1	PSR0400033882.mm.1	-5,27	0,00687	0,394155	Cassette Exon	0,24
TC0900001792.mm.1	2,51	Fat3	JUC0900008509.mm.1	5,16	0,000168	0,272566		
TC0900001792.mm.1	2,51	Fat3	PSR0900015118.mm.1	3,06	0,039485	0,539762	Cassette Exon	0,2
TC0900001792.mm.1	2,51	Fat3	PSR0900015108.mm.1	2,27	0,004809	0,371408	Cassette Exon	0,09
TC0900001792.mm.1	2,51	Fat3	PSR0900015095.mm.1	-2,01	0,015872	0,456526	Cassette Exon	0,1
TC0900001792.mm.1	2,51	Fat3	PSR0900015101.mm.1	-2,14	0,029479	0,510123	Cassette Exon	0,12
TC0900001792.mm.1	2,51	Fat3	PSR0900015099.mm.1	-2,15	0,033915	0,52424	Cassette Exon	0,18
TC0900001792.mm.1	2,51	Fat3	JUC0900008511.mm.1	-2,35	0,001457	0,328449		
TC0900001792.mm.1	2,51	Fat3	JUC0900008506.mm.1	-2,68	0,017763	0,465437		
TC0900001792.mm.1	2,51	Fat3	PSR0900015092.mm.1	-2,7	0,014663	0,450098	Alternative 5' Donc	0,27
TC0900001792.mm.1	2,51	Fat3	PSR0900015100.mm.1	-3,44	0,008364	0,406435	Cassette Exon	0,28
TC0900001792.mm.1	2,51	Fat3	PSR0900015096.mm.1	-3,64	0,0037	0,357266	Alternative 3' Acce	0,29
TC0900001792.mm.1	2,51	Fat3	JUC0900008502.mm.1	-3,74	0,043913	0,550676		
TC1200001523.mm.1	2	Gm21067; Gm10330	PSR1200010618.mm.1	5,16	0,002126	0,342919	Cassette Exon	0,28
TC1200001523.mm.1	2	Gm21067; Gm10330	PSR1200010624.mm.1	2,3	0,023898	0,492304	Cassette Exon	0,09
TC1700002661.mm.1	-1,01	Six3os1	JUC1700013052.mm.1	5,15	0,007371	0,39943		
TC1700002661.mm.1	-1,01	Six3os1	PSR1700024208.mm.1	2,13	0,035489	0,528724	Alternative 5' Donc	0,18
TC0500002429.mm.1	-1,08	Gm15820	JUC0500011921.mm.1	5,15	0,005496	0,379939		
TC0900001202.mm.1	-1,16	Atr	JUC0900005129.mm.1	5,14	0,020063	0,476514		
TC0700004464.mm.1	1,36	Ebf3	PSR0700037437.mm.1	5,13	0,041657	0,545333		
TC0700004464.mm.1	1,36	Ebf3	PSR0700037438.mm.1	4,21	0,011828	0,430502		
TC0700004464.mm.1	1,36	Ebf3	JUC0700019721.mm.1	2,83	0,010674	0,423096		

TC0700004464.mm.1	1,36	Ebf3	JUC0700019725.mm.1	2,6	0,001788	0,336311		
TC0700004464.mm.1	1,36	Ebf3	JUC0700019737.mm.1	-2,56	0,008892	0,410717		
TC0700004464.mm.1	1,36	Ebf3	PSR0700037434.mm.1	-3,31	0,041772	0,545564	Alternative 3' Acce	0,37
TC0600001275.mm.1	2,3	Syn2	JUC0600005225.mm.1	5,12	0,003605	0,356223		
TC0600001275.mm.1	2,3	Syn2	PSR0600010079.mm.1	2,12	0,013704	0,444758	Mutually Exclusive	0,17
TC0600001275.mm.1	2,3	Syn2	PSR0600010083.mm.1	-2,4	0,008795	0,409822	Cassette Exon	0,13
TC0600001275.mm.1	2,3	Syn2	PSR0600010084.mm.1	-2,43	0,00664	0,392838	Cassette Exon	0,13
TC0600001275.mm.1	2,3	Syn2	PSR0600010064.mm.1	-2,54	0,003755	0,357586	Cassette Exon	0,13
TC0600001275.mm.1	2,3	Syn2	PSR0600010080.mm.1	-2,59	0,02782	0,505498	Mutually Exclusive	0,17
TC0600001275.mm.1	2,3	Syn2	PSR0600010073.mm.1	-2,7	0,018273	0,468043	Alternative 3' Acce	0,44
TC0600001275.mm.1	2,3	Syn2	PSR0600010086.mm.1	-2,83	0,011992	0,432286	Alternative 5' Donc	0,21
TC0600001275.mm.1	2,3	Syn2	PSR0600010065.mm.1	-2,91	0,000981	0,31497	Cassette Exon	0,14
TC0600001275.mm.1	2,3	Syn2	PSR0600010074.mm.1	-3,05	0,00377	0,357586	Alternative 3' Acce	0,44
TC0600001275.mm.1	2,3	Syn2	PSR0600010072.mm.1	-4,13	0,001401	0,325997	Alternative 3' Acce	0,44
TC0600001275.mm.1	2,3	Syn2	JUC0600005232.mm.1	-4,74	0,000666	0,304088		
TC1400001849.mm.1	-1,24	Gm3141; Gm7236; Gm811	JUC1400007713.mm.1	5,12	0,04369	0,550248		
TC0400000057.mm.1	1,59	Chd7	JUC0400000177.mm.1	5,11	0,009604	0,417256		
TC0400000057.mm.1	1,59	Chd7	PSR0400000383.mm.1	2,27	0,003292	0,354243	Cassette Exon	0,14
TC0400000057.mm.1	1,59	Chd7	PSR0400000381.mm.1	2,06	0,026804	0,502337	Cassette Exon	0,21
TC0400000057.mm.1	1,59	Chd7	PSR0400000384.mm.1	2,01	0,01301	0,439291	Cassette Exon	0,11
TC0400000057.mm.1	1,59	Chd7	PSR0400000349.mm.1	-2,1	0,00863	0,408463	Cassette Exon	0,1
TC0400000057.mm.1	1,59	Chd7	JUC0400000202.mm.1	-3,5	0,042028	0,546158		
TC0200004545.mm.1	-1,39	Sppl2a	PSR0200038889.mm.1	5,1	0,036555	0,531355	Alternative 5' Donc	0,22
TC0100000471.mm.1	1,86	Abi2	JUC0100002316.mm.1	5,09	0,001735	0,335996		
TC0100000471.mm.1	1,86	Abi2	JUC0100002315.mm.1	4,79	0,011774	0,430069		
TC0100000471.mm.1	1,86	Abi2	PSR0100003942.mm.1	2,2	0,009359	0,415092	Alternative 3' Acce	0,12
TC0100000471.mm.1	1,86	Abi2	PSR0100003930.mm.1	-2,09	0,019329	0,473349	Alternative 3' Acce	0,16
TC0100000471.mm.1	1,86	Abi2	JUC0100002311.mm.1	-2,23	0,000167	0,272566		
TC0100000471.mm.1	1,86	Abi2	JUC0100002309.mm.1	-2,37	0,017917	0,466466		
TC0100000471.mm.1	1,86	Abi2	JUC0100002319.mm.1	-2,39	0,023517	0,490947		
TC0100000471.mm.1	1,86	Abi2	JUC0100002320.mm.1	-3,65	0,001108	0,317328		
TC1400000170.mm.1	-1,08	Sec24c	JUC1400000853.mm.1	5,09	0,012139	0,432868		
TC0200004662.mm.1	-1,53	5330413P13Rik; Gm14282	PSR0200040022.mm.1	5,08	0,006587	0,39213	Alternative 5' Donc	0,33
TC0200004662.mm.1	-1,53	5330413P13Rik; Gm14282	PSR0200040021.mm.1	2,06	0,045485	0,554313	Alternative 5' Donc	0,22
TC0700000913.mm.1	3,81	Slc17a6	JUC0700004161.mm.1	5,08	0,004667	0,370181		
TC0700000913.mm.1	3,81	Slc17a6	JUC0700004165.mm.1	4,63	0,000719	0,306015		
TC0700000913.mm.1	3,81	Slc17a6	PSR0700008269.mm.1	2,7	0,001542	0,330452	Cassette Exon	0,19
TC0700000913.mm.1	3,81	Slc17a6	PSR0700008270.mm.1	2,57	0,003737	0,357586	Cassette Exon	0,31
TC0700000913.mm.1	3,81	Slc17a6	PSR0700008260.mm.1	-2,04	0,013521	0,443431	Cassette Exon	0,28
TC0700000913.mm.1	3,81	Slc17a6	PSR0700008264.mm.1	-2,92	0,004356	0,365487	Cassette Exon	0,22
TC0700000913.mm.1	3,81	Slc17a6	PSR0700008262.mm.1	-2,95	0,022159	0,48561	Cassette Exon	0,17
TC0700000913.mm.1	3,81	Slc17a6	JUC0700004164.mm.1	-3,33	0,000768	0,308403		
TC0700000913.mm.1	3,81	Slc17a6	JUC0700004156.mm.1	-3,38	0,011541	0,428761		
TC0700000913.mm.1	3,81	Slc17a6	PSR0700008259.mm.1	-3,63	0,020235	0,477145	Cassette Exon	0,28
TC0300000124.mm.1	1,29	Mtfr1	JUC0300000226.mm.1	5,08	0,039115	0,53847		
TC0300000124.mm.1	1,29	Mtfr1	PSR0300000517.mm.1	-2,07	0,011085	0,425927	Cassette Exon	0,07
TC1900001448.mm.1	2,04	Cpeb3	JUC1900006892.mm.1	5,07	0,015234	0,452839		
TC1900001448.mm.1	2,04	Cpeb3	JUC1900006889.mm.1	2,69	0,010542	0,422281		

TC1900001448.mm.1	2,04	Cpeb3	PSR1900012618.mm.1	2,23	0,013745	0,444758	Cassette Exon	0,21
TC1900001448.mm.1	2,04	Cpeb3	PSR1900012630.mm.1	-2,07	0,030407	0,513027		
TC1900001448.mm.1	2,04	Cpeb3	PSR1900012643.mm.1	-2,19	0,018414	0,468484	Cassette Exon	0,18
TC1900001448.mm.1	2,04	Cpeb3	PSR1900012622.mm.1	-2,38	0,01785	0,466223	Alternative 5' Donc	0,12
TC1900001448.mm.1	2,04	Cpeb3	PSR1900012616.mm.1	-2,58	0,015204	0,452738	Alternative 3' Acce	0,26
TC1900001448.mm.1	2,04	Cpeb3	JUC1900006902.mm.1	-2,6	0,010014	0,418611		
TC1900001448.mm.1	2,04	Cpeb3	JUC1900006898.mm.1	-2,72	0,008209	0,405909		
TC1900001448.mm.1	2,04	Cpeb3	PSR1900012608.mm.1	-3,41	0,006849	0,393893	Alternative 3' Acce	0,42
TC1900001347.mm.1	-1,3	9930021J03Rik	JUC1900006561.mm.1	5,07	0,003137	0,353892		
TC1200001308.mm.1	2,8	Ptprn2	PSR1200009458.mm.1	5,05	0,001693	0,335395	Cassette Exon	0,4
TC1200001308.mm.1	2,8	Ptprn2	JUC1200005228.mm.1	3,61	0,00634	0,389463		
TC1200001308.mm.1	2,8	Ptprn2	JUC1200005238.mm.1	2,78	0,002472	0,349135		
TC1200001308.mm.1	2,8	Ptprn2	PSR1200009468.mm.1	-2,13	0,040469	0,542132	Cassette Exon	0,09
TC1200001308.mm.1	2,8	Ptprn2	JUC1200005225.mm.1	-2,25	0,024169	0,493262		
TC1200001308.mm.1	2,8	Ptprn2	PSR1200009479.mm.1	-2,34	0,013744	0,444758	Cassette Exon	0,2
TC1200001308.mm.1	2,8	Ptprn2	PSR1200009459.mm.1	-2,4	0,031791	0,517596	Cassette Exon	0,31
TC1200001308.mm.1	2,8	Ptprn2	PSR1200009485.mm.1	-2,86	0,020318	0,477245	Cassette Exon	0,24
TC1200001308.mm.1	2,8	Ptprn2	JUC1200005229.mm.1	-3,04	0,021312	0,481714		
TC1200001308.mm.1	2,8	Ptprn2	PSR1200009481.mm.1	-3,05	0,035598	0,528887	Cassette Exon	0,35
TC1200001308.mm.1	2,8	Ptprn2	PSR1200009480.mm.1	-3,32	0,023255	0,490032	Cassette Exon	0,21
TC1200001308.mm.1	2,8	Ptprn2	PSR1200009461.mm.1	-3,45	0,024452	0,493971		
TC1200001308.mm.1	2,8	Ptprn2	JUC1200005242.mm.1	-3,48	0,004074	0,361986		
TC1200001308.mm.1	2,8	Ptprn2	PSR1200009456.mm.1	-3,68	0,010663	0,423012	Cassette Exon	0,3
TC1200001308.mm.1	2,8	Ptprn2	PSR1200009482.mm.1	-3,91	0,007437	0,399934	Cassette Exon	0,49
TC1200001308.mm.1	2,8	Ptprn2	PSR1200009478.mm.1	-4,47	0,006581	0,39213	Cassette Exon	0,42
TC1200001308.mm.1	2,8	Ptprn2	PSR1200009484.mm.1	-6,23	0,006455	0,390284	Cassette Exon	0,43
TC1200001308.mm.1	2,8	Ptprn2	JUC1200005248.mm.1	-6,68	0,01206	0,432426		
TC1200001308.mm.1	2,8	Ptprn2	PSR1200009466.mm.1	-7,25	0,000665	0,304044	Alternative 5' Donc	0,49
TC0400003275.mm.1	2,73	Elavl4	JUC0400013573.mm.1	5,05	0,001601	0,332773		
TC0400003275.mm.1	2,73	Elavl4	PSR0400026100.mm.1	-2,13	0,045211	0,553586		
TC0400003275.mm.1	2,73	Elavl4	PSR0400026119.mm.1	-2,2	0,015593	0,454982	Cassette Exon	0,03
TC0400003275.mm.1	2,73	Elavl4	PSR0400026127.mm.1	-2,48	0,016731	0,460272	Cassette Exon	0,14
TC0400003275.mm.1	2,73	Elavl4	PSR0400026126.mm.1	-3,02	0,004732	0,370888	Cassette Exon	0,19
TC0400003275.mm.1	2,73	Elavl4	PSR0400026128.mm.1	-3,06	0,019453	0,474154	Cassette Exon	0,22
TC0400003275.mm.1	2,73	Elavl4	PSR0400026122.mm.1	-3,26	0,014101	0,447108	Alternative 5' Donc	0,25
TC0400003275.mm.1	2,73	Elavl4	PSR0400026123.mm.1	-3,32	0,022068	0,485366	Cassette Exon	0,34
TC0400003275.mm.1	2,73	Elavl4	JUC0400013581.mm.1	-3,65	0,019808	0,475245		
TC0400003275.mm.1	2,73	Elavl4	PSR0400026129.mm.1	-3,75	0,00864	0,408463	Cassette Exon	0,22
TC0400003275.mm.1	2,73	Elavl4	JUC0400013588.mm.1	-3,79	0,01425	0,447947		
TC0400003275.mm.1	2,73	Elavl4	PSR0400026110.mm.1	-4,02	0,00039	0,296751	Cassette Exon	0,25
TC0400003275.mm.1	2,73	Elavl4	PSR0400026120.mm.1	-4,18	0,005451	0,379939	Alternative 5' Donc	0,29
TC0400003275.mm.1	2,73	Elavl4	PSR0400026121.mm.1	-4,69	0,009925	0,418378	Alternative 5' Donc	0,29
TC0400003275.mm.1	2,73	Elavl4	PSR0400026104.mm.1	-4,77	0,01777	0,465467	Alternative 5' Donc	0,37
TC0400003275.mm.1	2,73	Elavl4	PSR0400026115.mm.1	-5,54	0,003021	0,353447	Cassette Exon	0,28
TC1500000699.mm.1	1,09	Cacna1i	JUC1500002999.mm.1	5,05	0,007222	0,39826		
TC1500000699.mm.1	1,09	Cacna1i	JUC1500003008.mm.1	3,78	0,013095	0,439759		
TC1500000699.mm.1	1,09	Cacna1i	JUC1500003007.mm.1	2,6	0,007805	0,403089		
TC1500000699.mm.1	1,09	Cacna1i	PSR1500005296.mm.1	2,51	0,009273	0,41476	Alternative 5' Donc	0,24

TC1500000699.mm.1	1,09	Cacna1i	PSR1500005294.mm.1	2,36	0,037297	0,533573	Alternative 5' Donc	0,22
TC1500000699.mm.1	1,09	Cacna1i	PSR1500005275.mm.1	2,25	0,000034	0,238339	Cassette Exon	0,27
TC1500000699.mm.1	1,09	Cacna1i	JUC1500003004.mm.1	2,21	0,013659	0,444394		
TC1500000699.mm.1	1,09	Cacna1i	PSR1500005272.mm.1	2,06	0,021881	0,484766	Cassette Exon	0,1
TC0300002782.mm.1	1,22	Amy2a4; Amy2a2; Amy2a	JUC0300011400.mm.1	5,04	0,000841	0,311886		
TC0300002782.mm.1	1,22	Amy2a4; Amy2a2; Amy2a	PSR0300021876.mm.1	2,51	0,041717	0,545356	Cassette Exon	0,21
TC0300002782.mm.1	1,22	Amy2a4; Amy2a2; Amy2a	JUC0300011404.mm.1	2,43	0,009671	0,417554		
TC0300002783.mm.1	1,22	Amy2a3; Amy2a2; Amy2a	JUC0300011411.mm.1	5,04	0,000841	0,311886		
TC0300002783.mm.1	1,22	Amy2a3; Amy2a2; Amy2a	PSR0300021894.mm.1	2,51	0,041717	0,545356	Cassette Exon	0,21
TC0300002783.mm.1	1,22	Amy2a3; Amy2a2; Amy2a	JUC0300011415.mm.1	2,43	0,009671	0,417554		
TC0300002784.mm.1	1,22	Amy2a2; Amy2a3; Amy2a	JUC0300011422.mm.1	5,04	0,000841	0,311886		
TC0300002784.mm.1	1,22	Amy2a2; Amy2a3; Amy2a	PSR0300021912.mm.1	2,51	0,041717	0,545356	Cassette Exon	0,21
TC0300002784.mm.1	1,22	Amy2a2; Amy2a3; Amy2a	JUC0300011426.mm.1	2,43	0,009671	0,417554		
TC1600000741.mm.1	1,01	Senp7	JUC1600003376.mm.1	5,03	0,000887	0,312707		
TC1600000741.mm.1	1,01	Senp7	JUC1600003386.mm.1	2,64	0,003354	0,354243		
TC1600000741.mm.1	1,01	Senp7	PSR1600006417.mm.1	2,24	0,00459	0,369794	Cassette Exon	0,12
TC1600000741.mm.1	1,01	Senp7	JUC1600003384.mm.1	2,24	0,031397	0,516433		
TC1600000741.mm.1	1,01	Senp7	PSR1600006401.mm.1	2,05	0,039091	0,538395	Cassette Exon	0,08
TC1600000117.mm.1	-1,11	Clec16a	JUC1600000446.mm.1	5,03	0,001262	0,322251		
TC1600000117.mm.1	-1,11	Clec16a	JUC1600000447.mm.1	2,4	0,035445	0,52871		
TC1500000142.mm.1	2,56	Cdh18	JUC1500000687.mm.1	5,02	0,001519	0,330268		
TC1500000142.mm.1	2,56	Cdh18	JUC1500000692.mm.1	3,42	0,001121	0,317328		
TC1500000142.mm.1	2,56	Cdh18	PSR1500001161.mm.1	2,87	0,00836	0,406435	Alternative 3' Acce	0,28
TC1500000142.mm.1	2,56	Cdh18	PSR1500001173.mm.1	2,4	0,000459	0,298999	Cassette Exon	0,45
TC1500000142.mm.1	2,56	Cdh18	PSR1500001181.mm.1	-2,03	0,045291	0,553742	Alternative 5' Donc	0,18
TC1500000142.mm.1	2,56	Cdh18	JUC1500000685.mm.1	-2,19	0,032986	0,52136		
TC1500000142.mm.1	2,56	Cdh18	PSR1500001182.mm.1	-2,2	0,017851	0,466223	Alternative 5' Donc	0,18
TC1500000142.mm.1	2,56	Cdh18	PSR1500001177.mm.1	-2,21	0,016664	0,460023	Cassette Exon	0,14
TC1500000142.mm.1	2,56	Cdh18	PSR1500001179.mm.1	-2,3	0,008746	0,409704	Cassette Exon	0,26
TC1500000142.mm.1	2,56	Cdh18	PSR1500001160.mm.1	-2,73	0,0255	0,497664	Cassette Exon	0,18
TC1500000142.mm.1	2,56	Cdh18	PSR1500001172.mm.1	-3,53	0,003336	0,354243	Alternative 3' Acce	0,51
TC1500000142.mm.1	2,56	Cdh18	JUC1500000696.mm.1	-3,58	0,003232	0,354243		
TC1500000142.mm.1	2,56	Cdh18	JUC1500000697.mm.1	-4,05	0,001592	0,331726		
TC1500000142.mm.1	2,56	Cdh18	PSR1500001176.mm.1	-6,06	0,00043	0,297771	Alternative 5' Donc	0,45
TC1400001567.mm.1	3,05	Kcnma1	JUC1400006387.mm.1	5,02	0,008102	0,405071		
TC1400001567.mm.1	3,05	Kcnma1	PSR1400011819.mm.1	4,95	0,010894	0,424403	Cassette Exon	0,34
TC1400001567.mm.1	3,05	Kcnma1	JUC1400006385.mm.1	3,56	0,000856	0,311909		
TC1400001567.mm.1	3,05	Kcnma1	JUC1400006391.mm.1	3,56	0,000856	0,311909		
TC1400001567.mm.1	3,05	Kcnma1	PSR1400011779.mm.1	3,36	0,002262	0,34578	Alternative 3' Acce	0,27
TC1400001567.mm.1	3,05	Kcnma1	JUC1400006402.mm.1	2,97	0,027731	0,505001		
TC1400001567.mm.1	3,05	Kcnma1	JUC1400006393.mm.1	2,87	0,005413	0,379167		
TC1400001567.mm.1	3,05	Kcnma1	PSR1400011804.mm.1	2,77	0,017018	0,461709	Alternative 3' Acce	0,32
TC1400001567.mm.1	3,05	Kcnma1	PSR1400011825.mm.1	2,41	0,00309	0,353892	Cassette Exon	0,35
TC1400001567.mm.1	3,05	Kcnma1	PSR1400011813.mm.1	2,08	0,017788	0,465621	Cassette Exon	0,13
TC1400001567.mm.1	3,05	Kcnma1	PSR1400011827.mm.1	-2,02	0,046859	0,557301	Cassette Exon	0,23
TC1400001567.mm.1	3,05	Kcnma1	JUC1400006378.mm.1	-2,19	0,048259	0,560283		
TC1400001567.mm.1	3,05	Kcnma1	PSR1400011833.mm.1	-2,49	0,016412	0,458956		
TC1400001567.mm.1	3,05	Kcnma1	PSR1400011826.mm.1	-2,58	0,01492	0,451919	Cassette Exon	0,37

TC1400001567.mm.1	3,05	Kcnma1	JUC1400006364.mm.1	-2,88	0,021387	0,482022		
TC1400001567.mm.1	3,05	Kcnma1	JUC1400006372.mm.1	-2,96	0,036719	0,531762		
TC1400001567.mm.1	3,05	Kcnma1	JUC1400006369.mm.1	-2,98	0,002586	0,349501		
TC1400001567.mm.1	3,05	Kcnma1	PSR1400011797.mm.1	-3,15	0,007545	0,401384	Cassette Exon	0,38
TC1400001567.mm.1	3,05	Kcnma1	PSR1400011835.mm.1	-3,4	0,02064	0,478859	Cassette Exon	0,35
TC1400001567.mm.1	3,05	Kcnma1	JUC1400006382.mm.1	-3,49	0,027238	0,503642		
TC1400001567.mm.1	3,05	Kcnma1	PSR1400011834.mm.1	-3,55	0,017782	0,465553	Cassette Exon	0,05
TC1400001567.mm.1	3,05	Kcnma1	JUC1400006403.mm.1	-3,74	0,040341	0,541906		
TC1400001567.mm.1	3,05	Kcnma1	PSR1400011828.mm.1	-3,83	0,006753	0,393343	Cassette Exon	0,36
TC1400001567.mm.1	3,05	Kcnma1	JUC1400006370.mm.1	-3,91	0,039504	0,539762		
TC1400001567.mm.1	3,05	Kcnma1	JUC1400006392.mm.1	-5,49	0,025037	0,496091		
TC1400001567.mm.1	3,05	Kcnma1	JUC1400006373.mm.1	-6,7	0,00256	0,349501		
TC1400001567.mm.1	3,05	Kcnma1	JUC1400006397.mm.1	-7,06	0,000617	0,304044		
TC1000001571.mm.1	2,37	Tac2	JUC1000006094.mm.1	5,02	0,014774	0,450801		
TC1000001571.mm.1	2,37	Tac2	PSR1000011309.mm.1	2,49	0,016909	0,460797	Cassette Exon	0,27
TC1000001571.mm.1	2,37	Tac2	JUC1000006093.mm.1	2,08	0,032251	0,519011		
TC1000001571.mm.1	2,37	Tac2	PSR1000011302.mm.1	-3,44	0,003085	0,353892	Cassette Exon	0,36
TC1000001571.mm.1	2,37	Tac2	JUC1000006090.mm.1	-3,9	0,014093	0,446971		
TC0500002642.mm.1	1,26	Ppat	JUC0500012714.mm.1	5,02	0,024241	0,493294		
TC1000001755.mm.1	-1,3	Utrn	JUC1000006978.mm.1	5,02	0,032236	0,51899		
TC1000001755.mm.1	-1,3	Utrn	JUC1000006967.mm.1	2,84	0,007172	0,397855		
TC1000001755.mm.1	-1,3	Utrn	JUC1000006937.mm.1	2,51	0,011874	0,431125		
TC0800002920.mm.1	12,81	Calb2	PSR0800022441.mm.1	5,01	0,004528	0,368648	Cassette Exon	0,31
TC0800002920.mm.1	12,81	Calb2	JUC0800012244.mm.1	3,12	0,001373	0,32572		
TC0800002920.mm.1	12,81	Calb2	PSR0800022444.mm.1	2,64	0,031347	0,516281	Cassette Exon	0,07
TC0800002920.mm.1	12,81	Calb2	JUC0800012241.mm.1	2,29	0,022659	0,487609		
TC0800002920.mm.1	12,81	Calb2	PSR0800022447.mm.1	-2,36	0,000938	0,313363	Cassette Exon	0,03
TC0800002920.mm.1	12,81	Calb2	PSR0800022438.mm.1	-3,24	0,001375	0,32572	Cassette Exon	0,01
TC0800002920.mm.1	12,81	Calb2	JUC0800012242.mm.1	-4,06	0,003674	0,357266		
TC0800002920.mm.1	12,81	Calb2	PSR0800022448.mm.1	-6,86	0,000435	0,297771	Cassette Exon	0,28
TC0800002920.mm.1	12,81	Calb2	PSR0800022436.mm.1	-8,07	0,002858	0,352207	Cassette Exon	0,41
TC0800002920.mm.1	12,81	Calb2	JUC0800012243.mm.1	-8,64	0,001398	0,325997		
TC0800002920.mm.1	12,81	Calb2	JUC0800012236.mm.1	-22,25	0,000737	0,307808		
TC0300003029.mm.1	-1,03	Gm4862	PSR0300023899.mm.1	5	0,010075	0,418974	Alternative 5' Donc	0,47
TC0400003856.mm.1	1,52	Igsf21	JUC0400016512.mm.1	5	0,03566	0,528966		
TC0400003856.mm.1	1,52	Igsf21	PSR0400031784.mm.1	4,19	0,006169	0,387873	Cassette Exon	0,27
TC0400003856.mm.1	1,52	Igsf21	JUC0400016511.mm.1	4,12	0,003636	0,3566		
TC0600000294.mm.1	1,04	Mkln1	JUC0600001111.mm.1	4,99	0,00747	0,400381		
TC0600000294.mm.1	1,04	Mkln1	PSR0600002173.mm.1	2,51	0,000137	0,270987	Cassette Exon	0,21
TC0600000294.mm.1	1,04	Mkln1	PSR0600002170.mm.1	2,44	0,003519	0,355749	Cassette Exon	0,11
TC0600000294.mm.1	1,04	Mkln1	JUC0600001095.mm.1	2,01	0,024111	0,49322		
TC0400000913.mm.1	-1,62	Mtap	JUC0400003117.mm.1	4,99	0,024768	0,495014		
TC0X00003059.mm.1	-1,21	C330013F16Rik	JUC0X00009688.mm.1	4,99	0,003157	0,353892		
TC1600000284.mm.1	1,47	Ece2	JUC1600001421.mm.1	4,96	0,001851	0,337414		
TC1600000284.mm.1	1,47	Ece2	JUC1600001412.mm.1	4,08	0,004249	0,363417		
TC1600000284.mm.1	1,47	Ece2	JUC1600001417.mm.1	3,6	0,015332	0,453763		
TC1600000284.mm.1	1,47	Ece2	PSR1600002719.mm.1	-2,04	0,012447	0,43528	Alternative 5' Donc	0,09
TC1600000284.mm.1	1,47	Ece2	PSR1600002708.mm.1	-2,18	0,018427	0,468598	Alternative 3' Acce	0,13

TC1600000284.mm.1	1,47	Ece2	JUC1600001409.mm.1	-2,85	0,033265	0,522243		
TC1600000284.mm.1	1,47	Ece2	JUC1600001411.mm.1	-3,17	0,011402	0,427549		
TC0400002876.mm.1	1,35	Ptprd	JUC0400012285.mm.1	4,95	0,004966	0,373851		
TC0400002876.mm.1	1,35	Ptprd	JUC0400012301.mm.1	3,28	0,000034	0,238339		
TC0400002876.mm.1	1,35	Ptprd	JUC0400012310.mm.1	2,77	0,0147	0,450341		
TC0400002876.mm.1	1,35	Ptprd	JUC0400012279.mm.1	2,58	0,025852	0,498657		
TC0400002876.mm.1	1,35	Ptprd	PSR0400023573.mm.1	2,38	0,040649	0,54264	Cassette Exon	0,15
TC0400002876.mm.1	1,35	Ptprd	JUC0400012284.mm.1	2,23	0,001758	0,335996		
TC0400002876.mm.1	1,35	Ptprd	PSR0400023555.mm.1	2,07	0,036791	0,532063	Cassette Exon	0,12
TC0400002876.mm.1	1,35	Ptprd	PSR0400023571.mm.1	-2,04	0,043563	0,550033	Alternative 3' Acce	0,16
TC1400002303.mm.1	-1,62	Adam28	JUC1400009820.mm.1	4,95	0,01665	0,459985		
TC1400002303.mm.1	-1,62	Adam28	JUC1400009811.mm.1	3,05	0,001354	0,325427		
TC1400002303.mm.1	-1,62	Adam28	JUC1400009824.mm.1	2,02	0,030389	0,512923		
TC1400002303.mm.1	-1,62	Adam28	JUC1400009814.mm.1	-2,12	0,016242	0,458312		
TC0200001836.mm.1	-1,35	Eif3j2; Eif3j1	JUC0200007106.mm.1	4,95	0,007828	0,403089		
TC0400002606.mm.1	3,28	Gabbr2	JUC0400011033.mm.1	4,94	0,017415	0,46377		
TC0400002606.mm.1	3,28	Gabbr2	JUC0400011036.mm.1	4,11	0,016882	0,460797		
TC0400002606.mm.1	3,28	Gabbr2	JUC0400011028.mm.1	3,53	0,002925	0,352289		
TC0400002606.mm.1	3,28	Gabbr2	JUC0400011042.mm.1	3,14	0,005951	0,385276		
TC0400002606.mm.1	3,28	Gabbr2	PSR0400021271.mm.1	2,34	0,003141	0,353892	Alternative 3' Acce	0,21
TC0400002606.mm.1	3,28	Gabbr2	PSR0400021282.mm.1	2,17	0,027797	0,505406	Cassette Exon	0,17
TC0400002606.mm.1	3,28	Gabbr2	JUC0400011041.mm.1	-2,15	0,007357	0,39938		
TC0400002606.mm.1	3,28	Gabbr2	PSR0400021288.mm.1	-2,53	0,01143	0,427663	Cassette Exon	0,17
TC0400002606.mm.1	3,28	Gabbr2	PSR0400021293.mm.1	-3,43	0,000765	0,308403	Cassette Exon	0,15
TC0400002606.mm.1	3,28	Gabbr2	JUC0400011030.mm.1	-3,74	0,000549	0,304044		
TC0400002606.mm.1	3,28	Gabbr2	JUC0400011025.mm.1	-4,09	0,002581	0,349501		
TC0400002606.mm.1	3,28	Gabbr2	PSR0400021295.mm.1	-4,44	0,005095	0,37571	Cassette Exon	0,15
TC0400002606.mm.1	3,28	Gabbr2	PSR0400021280.mm.1	-4,91	0,001959	0,340561	Alternative 5' Donc	0,36
TC0400002606.mm.1	3,28	Gabbr2	JUC0400011034.mm.1	-5,09	0,000135	0,270905		
TC0400002606.mm.1	3,28	Gabbr2	PSR0400021273.mm.1	-6,95	0,001447	0,328226	Cassette Exon	0,41
TC0500003323.mm.1	1,81	Wbscr17	JUC0500016380.mm.1	4,94	0,023556	0,490947		
TC0500003323.mm.1	1,81	Wbscr17	PSR0500030083.mm.1	-2,21	0,021679	0,48395	Alternative 5' Donc	0,11
TC0500003323.mm.1	1,81	Wbscr17	JUC0500016377.mm.1	-2,28	0,015413	0,454055		
TC0500003323.mm.1	1,81	Wbscr17	PSR0500030074.mm.1	-2,38	0,001152	0,317344	Alternative 3' Acce	0,2
TC0100001914.mm.1	1,48	Rgs20	JUC0100008974.mm.1	4,93	0,009041	0,412806		
TC0100001914.mm.1	1,48	Rgs20	PSR0100015806.mm.1	4,26	0,009589	0,417183	Cassette Exon	0,41
TC1300002040.mm.1	1,18	Gprin1	JUC1300006593.mm.1	4,93	0,004208	0,363236		
TC0100001379.mm.1	3,75	Rgs8	PSR0100011423.mm.1	4,92	0,000154	0,272178	Alternative 5' Donc	0,33
TC0100001379.mm.1	3,75	Rgs8	PSR0100011422.mm.1	4,1	0,001235	0,322251	Alternative 5' Donc	0,46
TC0100001379.mm.1	3,75	Rgs8	PSR0100011412.mm.1	-2,04	0,022746	0,487897	Cassette Exon	0,06
TC0100001379.mm.1	3,75	Rgs8	JUC0100006589.mm.1	-2,36	0,032085	0,518469		
TC0100001379.mm.1	3,75	Rgs8	PSR0100011415.mm.1	-2,69	0,019885	0,475806	Cassette Exon	0,13
TC0100001379.mm.1	3,75	Rgs8	JUC0100006586.mm.1	-2,86	0,038205	0,535805		
TC0100001379.mm.1	3,75	Rgs8	PSR0100011401.mm.1	-3,05	0,017374	0,463612		
TC0100001379.mm.1	3,75	Rgs8	JUC0100006588.mm.1	-3,43	0,023328	0,490065		
TC0100001379.mm.1	3,75	Rgs8	PSR0100011399.mm.1	-3,58	0,01793	0,466466	Cassette Exon	0,24
TC0100001379.mm.1	3,75	Rgs8	PSR0100011398.mm.1	-3,62	0,006556	0,39213	Cassette Exon	0,26
TC0100001379.mm.1	3,75	Rgs8	PSR0100011403.mm.1	-3,64	0,030575	0,513395		

TC0100001379.mm.1	3,75	Rgs8	PSR0100011400.mm.1	-4,55	0,012389	0,434939	Cassette Exon	0,28
TC0100001379.mm.1	3,75	Rgs8	PSR0100011404.mm.1	-4,87	0,001068	0,316419	Alternative 3' Acce	0,16
TC0100001379.mm.1	3,75	Rgs8	PSR0100011408.mm.1	-5,03	0,019985	0,476227	Intron Retention	0,36
TC0100001379.mm.1	3,75	Rgs8	PSR0100011402.mm.1	-5,25	0,007972	0,403853		
TC0100001379.mm.1	3,75	Rgs8	PSR0100011397.mm.1	-6	0,006752	0,393343	Cassette Exon	0,26
TC0100001379.mm.1	3,75	Rgs8	PSR0100011406.mm.1	-6,89	0,005547	0,380675	Alternative 3' Acce	0,36
TC0100001379.mm.1	3,75	Rgs8	PSR0100011411.mm.1	-7,57	0,001503	0,329896	Alternative 3' Acce	0,29
TC0100001616.mm.1	2,01	Igsf9	PSR0100013455.mm.1	4,92	0,008133	0,405267	Cassette Exon	0,31
TC0100001616.mm.1	2,01	Igsf9	JUC0100007598.mm.1	2,05	0,008574	0,40821		
TC0100001616.mm.1	2,01	Igsf9	PSR0100013448.mm.1	-2,09	0,0279	0,505612	Intron Retention	0,23
TC0100001616.mm.1	2,01	Igsf9	PSR0100013476.mm.1	-2,46	0,040012	0,541174	Cassette Exon	0,15
TC0100001616.mm.1	2,01	Igsf9	PSR0100013468.mm.1	-2,75	0,005591	0,381035	Alternative 5' Donc	0,16
TC0100001616.mm.1	2,01	Igsf9	PSR0100013429.mm.1	-3,2	0,010843	0,424161	Cassette Exon	0,2
TC0100001616.mm.1	2,01	Igsf9	PSR0100013472.mm.1	-3,22	0,010651	0,422925	Cassette Exon	0,14
TC0100001616.mm.1	2,01	Igsf9	JUC0100007607.mm.1	-3,43	0,037021	0,532502		
TC0100001616.mm.1	2,01	Igsf9	JUC0100007596.mm.1	-3,81	0,019942	0,476043		
TC0X00000732.mm.1	-1,58	Xlr4e-ps	JUC0X00002537.mm.1	4,92	0,011609	0,429583		
TC0X00000732.mm.1	-1,58	Xlr4e-ps	PSR0X00005054.mm.1	2,59	0,039416	0,539606	Cassette Exon	0,28
TC0X00000732.mm.1	-1,58	Xlr4e-ps	PSR0X00005056.mm.1	2,35	0,01589	0,456526	Cassette Exon	0,18
TC0X00000732.mm.1	-1,58	Xlr4e-ps	JUC0X00002542.mm.1	2,04	0,005524	0,380518		
TC1900001205.mm.1	-1,07	Cep78	JUC1900005979.mm.1	4,92	0,019793	0,475212		
TC1900001205.mm.1	-1,07	Cep78	JUC1900005966.mm.1	2,61	0,003188	0,354243		
TC1900001205.mm.1	-1,07	Cep78	PSR1900010972.mm.1	2,07	0,043407	0,549603	Intron Retention	0,24
TC1000000811.mm.1	-1,13	Gzmm	JUC1000003187.mm.1	4,92	0,000565	0,304044		
TC1000000811.mm.1	-1,13	Gzmm	JUC1000003188.mm.1	2,3	0,007104	0,396897		
TC1000000332.mm.1	1,25	Rev3l	PSR1000002273.mm.1	4,91	0,015653	0,455329	Alternative 5' Donc	0,48
TC1000000332.mm.1	1,25	Rev3l	JUC1000001250.mm.1	-4,49	0,004728	0,370888		
TC1100003136.mm.1	16,28	Aipl1	JUC1100015200.mm.1	4,91	0,017837	0,466096		
TC1100003136.mm.1	16,28	Aipl1	JUC1100015199.mm.1	-2,62	0,003243	0,354243		
TC1100003136.mm.1	16,28	Aipl1	PSR1100029026.mm.1	-7,97	0,000173	0,277789	Cassette Exon	0,44
TC1100003136.mm.1	16,28	Aipl1	JUC1100015203.mm.1	-8,8	0,005584	0,381035		
TC1100003136.mm.1	16,28	Aipl1	JUC1100015202.mm.1	-19,04	0,002731	0,349612		
TC1100003136.mm.1	16,28	Aipl1	PSR1100029020.mm.1	-20,71	0,002073	0,342181	Alternative 3' Acce	0,44
TC1600001008.mm.1	1,78	Son	JUC1600004073.mm.1	4,91	0,002181	0,344453		
TC0200005041.mm.1	2,29	Gm14204	PSR0200043146.mm.1	4,9	0,021718	0,484104	Cassette Exon	0,28
TC0200005041.mm.1	2,29	Gm14204	PSR0200043144.mm.1	-2,72	0,049545	0,563325	Alternative 3' Acce	0,18
TC0200005041.mm.1	2,29	Gm14204	PSR0200043153.mm.1	-4,07	0,006359	0,389469	Cassette Exon	0,28
TC0200005041.mm.1	2,29	Gm14204	PSR0200043154.mm.1	-4,28	0,04474	0,552331	Cassette Exon	0,28
TC1500001750.mm.1	-1,54	Eef1d	JUC1500007516.mm.1	4,9	0,000633	0,304044		
TC1500001750.mm.1	-1,54	Eef1d	JUC1500007512.mm.1	2,75	0,021971	0,485125		
TC1100003997.mm.1	1,91	Abca8b	JUC1100019575.mm.1	4,89	0,002765	0,350231		
TC1100003997.mm.1	1,91	Abca8b	JUC1100019547.mm.1	2,91	0,002674	0,349612		
TC1100003997.mm.1	1,91	Abca8b	PSR1100037432.mm.1	-2,01	0,000498	0,300857	Cassette Exon	0,08
TC1100003997.mm.1	1,91	Abca8b	PSR1100037412.mm.1	-2,05	0,005943	0,385178	Alternative 3' Acce	0,17
TC1100003997.mm.1	1,91	Abca8b	JUC1100019582.mm.1	-2,06	0,008424	0,406611		
TC1100003997.mm.1	1,91	Abca8b	JUC1100019552.mm.1	-2,12	0,005052	0,374795		
TC1100003997.mm.1	1,91	Abca8b	PSR1100037458.mm.1	-2,3	0,002659	0,349612	Cassette Exon	0,14
TC1100003997.mm.1	1,91	Abca8b	JUC1100019561.mm.1	-2,72	0,024517	0,493992		

TC1100003997.mm.1	1,91	Abca8b	JUC1100019573.mm.1	-3,47	0,040916	0,543423		
TC0200001859.mm.1	-1,4	Sqrdl	JUC0200007255.mm.1	4,89	0,019651	0,474887		
TC0400001233.mm.1	-1,76	Efcab14	JUC0400004736.mm.1	4,89	0,032477	0,519641		
TC0200000850.mm.1	3,02	Galnt13	PSR0200006942.mm.1	4,88	0,014278	0,448073	Alternative 5' Donc	0,48
TC0200000850.mm.1	3,02	Galnt13	PSR0200006929.mm.1	3,37	0,000014	0,18968	Cassette Exon	0,27
TC0200000850.mm.1	3,02	Galnt13	PSR0200006937.mm.1	2,56	0,024576	0,494301		
TC0200000850.mm.1	3,02	Galnt13	JUC0200003524.mm.1	2,45	0,021887	0,484824		
TC0200000850.mm.1	3,02	Galnt13	PSR0200006946.mm.1	-2,14	0,02051	0,47825	Alternative 5' Donc	0,33
TC0200000850.mm.1	3,02	Galnt13	PSR0200006927.mm.1	-2,32	0,006016	0,386586	Alternative 3' Acce	0,21
TC0200000850.mm.1	3,02	Galnt13	PSR0200006932.mm.1	-2,5	0,011308	0,427133	Cassette Exon	0,14
TC0200000850.mm.1	3,02	Galnt13	PSR0200006924.mm.1	-3,69	0,010633	0,422841		
TC0200000850.mm.1	3,02	Galnt13	PSR0200006918.mm.1	-3,76	0,002798	0,352011	Alternative 3' Acce	0,15
TC0200000850.mm.1	3,02	Galnt13	PSR0200006926.mm.1	-4,06	0,017346	0,46353	Cassette Exon	0,28
TC0200000850.mm.1	3,02	Galnt13	PSR0200006921.mm.1	-4,18	0,003127	0,353892	Alternative 3' Acce	0,15
TC0200000850.mm.1	3,02	Galnt13	PSR0200006947.mm.1	-4,49	0,000453	0,298307	Alternative 5' Donc	0,33
TC0200000850.mm.1	3,02	Galnt13	JUC0200003519.mm.1	-4,98	0,012636	0,436161		
TC0200000850.mm.1	3,02	Galnt13	JUC0200003523.mm.1	-5,26	0,000841	0,311886		
TC0200000850.mm.1	3,02	Galnt13	PSR0200006925.mm.1	-5,43	0,000729	0,306408	Alternative 5' Donc	0,05
TC0200000850.mm.1	3,02	Galnt13	JUC0200003521.mm.1	-5,71	0,004908	0,372662		
TC0200000850.mm.1	3,02	Galnt13	PSR0200006938.mm.1	-5,88	0,002679	0,349612	Alternative 5' Donc	0,34
TC0200000850.mm.1	3,02	Galnt13	PSR0200006920.mm.1	-5,93	0,003272	0,354243	Alternative 3' Acce	0,15
TC0300002781.mm.1	1,13	Amy2a5; Amy2a2; Amy2a	JUC0300011388.mm.1	4,88	0,000191	0,28803		
TC0300002781.mm.1	1,13	Amy2a5; Amy2a2; Amy2a	PSR0300021852.mm.1	3,17	0,047788	0,559217	Cassette Exon	0,19
TC0300002781.mm.1	1,13	Amy2a5; Amy2a2; Amy2a	JUC0300011387.mm.1	2,45	0,006879	0,394271		
TC0300002781.mm.1	1,13	Amy2a5; Amy2a2; Amy2a	PSR0300021854.mm.1	2,14	0,021124	0,480611	Cassette Exon	0,17
TC0200003533.mm.1	1,32	Cacnb4	JUC0200015312.mm.1	4,88	0,012453	0,43528		
TC0200003533.mm.1	1,32	Cacnb4	PSR0200030223.mm.1	-2,19	0,023732	0,491573	Alternative 5' Donc	0,18
TC0100003129.mm.1	-1,39	Nav1	JUC0100014442.mm.1	4,87	0,032942	0,52122		
TC0100003129.mm.1	-1,39	Nav1	JUC0100014428.mm.1	3,19	0,027942	0,505612		
TC0100003129.mm.1	-1,39	Nav1	JUC0100014427.mm.1	2,97	0,018884	0,47116		
TC0100003129.mm.1	-1,39	Nav1	JUC0100014408.mm.1	2,45	0,012191	0,433076		
TC0700001423.mm.1	1,12	Fchsd2	JUC0700006299.mm.1	4,87	0,031896	0,518021		
TC1200000852.mm.1	4,36	Esrrb	JUC1200003524.mm.1	4,86	0,009989	0,418456		
TC1200000852.mm.1	4,36	Esrrb	PSR1200006245.mm.1	3,95	0,025526	0,497712		
TC1200000852.mm.1	4,36	Esrrb	PSR1200006249.mm.1	2,71	0,002478	0,349135	Mutually Exclusive	0,4
TC1200000852.mm.1	4,36	Esrrb	JUC1200003527.mm.1	2,22	0,015084	0,452418		
TC1200000852.mm.1	4,36	Esrrb	PSR1200006251.mm.1	-2,66	0,004642	0,370181	Cassette Exon	0,15
TC1200000852.mm.1	4,36	Esrrb	PSR1200006253.mm.1	-3,44	0,0036	0,356223	Cassette Exon	0,16
TC1200000852.mm.1	4,36	Esrrb	JUC1200003521.mm.1	-3,76	0,018125	0,467234		
TC1200000852.mm.1	4,36	Esrrb	JUC1200003528.mm.1	-4,03	0,008129	0,405267		
TC1200000852.mm.1	4,36	Esrrb	PSR1200006256.mm.1	-4,3	0,00219	0,344463	Cassette Exon	0,25
TC1200000852.mm.1	4,36	Esrrb	PSR1200006244.mm.1	-4,96	0,002525	0,349501	Alternative 3' Acce	0,48
TC1200000852.mm.1	4,36	Esrrb	JUC1200003530.mm.1	-5,23	0,001346	0,325349		
TC1200000852.mm.1	4,36	Esrrb	PSR1200006250.mm.1	-5,3	0,01031	0,420505	Mutually Exclusive	0,4
TC1200000852.mm.1	4,36	Esrrb	JUC1200003525.mm.1	-5,39	0,003225	0,354243		
TC1200000852.mm.1	4,36	Esrrb	PSR1200006243.mm.1	-5,94	0,001341	0,325349	Cassette Exon	0,4
TC1200000852.mm.1	4,36	Esrrb	JUC1200003529.mm.1	-8,53	0,000752	0,308185		
TC1200000852.mm.1	4,36	Esrrb	PSR1200006247.mm.1	-9,46	0,004771	0,371044	Alternative 3' Acce	0,34

TC1700000596.mm.1	1,93	BC033916; Gm7029	PSR1700005500.mm.1	4,85	0,001856	0,337543	Cassette Exon	0,33
TC1700000596.mm.1	1,93	BC033916; Gm7029	JUC1700003016.mm.1	2,09	0,034244	0,525168		
TC1700000596.mm.1	1,93	BC033916; Gm7029	JUC1700003019.mm.1	2,09	0,034244	0,525168		
TC1700000596.mm.1	1,93	BC033916; Gm7029	PSR1700005489.mm.1	-2,08	0,034025	0,524517	Cassette Exon	0,26
TC1700000596.mm.1	1,93	BC033916; Gm7029	PSR1700005495.mm.1	-2,52	0,019443	0,474116	Cassette Exon	0,22
TC1700000596.mm.1	1,93	BC033916; Gm7029	JUC1700003021.mm.1	-2,6	0,016712	0,460199		
TC1700000596.mm.1	1,93	BC033916; Gm7029	JUC1700003024.mm.1	-3,44	0,025496	0,497664		
TC1700000596.mm.1	1,93	BC033916; Gm7029	PSR1700005487.mm.1	-3,47	0,012973	0,439245	Cassette Exon	0,24
TC1700000596.mm.1	1,93	BC033916; Gm7029	PSR1700005497.mm.1	-3,47	0,012973	0,439245	Cassette Exon	0,15
TC1700000596.mm.1	1,93	BC033916; Gm7029	JUC1700003025.mm.1	-5,34	0,011728	0,429969		
TC0400002768.mm.1	-1,05	Mup1	JUC0400011752.mm.1	4,85	0,045543	0,554382		
TC0X00003185.mm.1	-1,18	Ribc1	JUC0X00010111.mm.1	4,85	0,004509	0,368648		
TC1000002408.mm.1	4,62	Pcbp3	JUC1000009564.mm.1	4,84	0,008568	0,40821		
TC1000002408.mm.1	4,62	Pcbp3	PSR1000017450.mm.1	2,55	0,00238	0,348564	Cassette Exon	0,3
TC1000002408.mm.1	4,62	Pcbp3	JUC1000009559.mm.1	2,06	0,014226	0,447947		
TC1000002408.mm.1	4,62	Pcbp3	PSR1000017437.mm.1	-2,02	0,015312	0,453561	Cassette Exon	0,13
TC1000002408.mm.1	4,62	Pcbp3	JUC1000009557.mm.1	-2,18	0,011491	0,42815		
TC1000002408.mm.1	4,62	Pcbp3	PSR1000017452.mm.1	-2,32	0,044598	0,552057	Cassette Exon	0,12
TC1000002408.mm.1	4,62	Pcbp3	JUC1000009563.mm.1	-2,66	0,011019	0,425624		
TC1000002408.mm.1	4,62	Pcbp3	JUC1000009568.mm.1	-2,71	0,005384	0,378801		
TC1000002408.mm.1	4,62	Pcbp3	JUC1000009571.mm.1	-2,8	0,049687	0,563531		
TC1000002408.mm.1	4,62	Pcbp3	PSR1000017448.mm.1	-2,86	0,001483	0,329081	Cassette Exon	0,45
TC1000002408.mm.1	4,62	Pcbp3	JUC1000009581.mm.1	-3,03	0,030345	0,512923		
TC1000002408.mm.1	4,62	Pcbp3	JUC1000009575.mm.1	-3,04	0,037415	0,533831		
TC1000002408.mm.1	4,62	Pcbp3	PSR1000017433.mm.1	-3,36	0,008326	0,406313	Cassette Exon	0,36
TC1000002408.mm.1	4,62	Pcbp3	PSR1000017453.mm.1	-3,41	0,021171	0,480978	Cassette Exon	0,15
TC1000002408.mm.1	4,62	Pcbp3	PSR1000017449.mm.1	-3,54	0,012749	0,436821	Cassette Exon	0,53
TC1000002408.mm.1	4,62	Pcbp3	PSR1000017459.mm.1	-4,74	0,008456	0,406611	Cassette Exon	0,28
TC1000002408.mm.1	4,62	Pcbp3	JUC1000009572.mm.1	-5,93	0,001251	0,322251		
TC1200002045.mm.1	3,02	Dpf3	PSR1200014049.mm.1	4,83	0,001397	0,325997	Alternative 5' Donc	0,6
TC1200002045.mm.1	3,02	Dpf3	JUC1200007729.mm.1	3,76	0,004818	0,371408		
TC1200002045.mm.1	3,02	Dpf3	PSR1200014042.mm.1	2,78	0,002308	0,346468	Alternative 5' Donc	0,28
TC1200002045.mm.1	3,02	Dpf3	JUC1200007730.mm.1	-2,11	0,025397	0,497403		
TC1200002045.mm.1	3,02	Dpf3	JUC1200007725.mm.1	-3,14	0,032059	0,518469		
TC1200002045.mm.1	3,02	Dpf3	JUC1200007739.mm.1	-3,2	0,033447	0,522793		
TC1200002045.mm.1	3,02	Dpf3	PSR1200014048.mm.1	-3,25	0,003148	0,353892	Cassette Exon	0,25
TC1200002045.mm.1	3,02	Dpf3	PSR1200014061.mm.1	-3,75	0,003784	0,357586	Cassette Exon	0,23
TC1200002045.mm.1	3,02	Dpf3	PSR1200014059.mm.1	-4,49	0,002911	0,352289	Cassette Exon	0,41
TC1200002045.mm.1	3,02	Dpf3	JUC1200007735.mm.1	-7,9	0,000596	0,304044		
TC1100000033.mm.1	-1,33	Sec14l3	JUC1100000186.mm.1	4,83	0,021419	0,482126		
TC0100002606.mm.1	4,1	Ptprn	JUC0100012029.mm.1	4,82	0,005751	0,383101		
TC0100002606.mm.1	4,1	Ptprn	JUC0100012012.mm.1	3,38	0,005115	0,376246		
TC0100002606.mm.1	4,1	Ptprn	PSR0100021227.mm.1	2,92	0,004277	0,363899	Cassette Exon	0,06
TC0100002606.mm.1	4,1	Ptprn	PSR0100021217.mm.1	2,67	0,02705	0,502979	Cassette Exon	0,09
TC0100002606.mm.1	4,1	Ptprn	JUC0100012013.mm.1	2,63	0,022124	0,485558		
TC0100002606.mm.1	4,1	Ptprn	JUC0100012016.mm.1	2,56	0,000436	0,297771		
TC0100002606.mm.1	4,1	Ptprn	JUC0100012026.mm.1	2,29	0,008322	0,406313		
TC0100002606.mm.1	4,1	Ptprn	JUC0100012019.mm.1	2,22	0,018345	0,468287		

TC0100002606.mm.1	4,1	Ptprn	PSR0100021216.mm.1	2,12	0,023568	0,490947		
TC0100002606.mm.1	4,1	Ptprn	PSR0100021210.mm.1	-2,01	0,01383	0,445221	Cassette Exon	0,36
TC0100002606.mm.1	4,1	Ptprn	PSR0100021213.mm.1	-2,41	0,000072	0,251428	Cassette Exon	0,1
TC0100002606.mm.1	4,1	Ptprn	PSR0100021212.mm.1	-2,65	0,006125	0,387351	Cassette Exon	0,06
TC0100002606.mm.1	4,1	Ptprn	JUC0100012023.mm.1	-2,91	0,003838	0,358714		
TC0100002606.mm.1	4,1	Ptprn	PSR0100021207.mm.1	-3	0,001466	0,329023	Cassette Exon	0,27
TC0100002606.mm.1	4,1	Ptprn	JUC0100012014.mm.1	-3,61	0,01756	0,464258		
TC0100002606.mm.1	4,1	Ptprn	JUC0100012017.mm.1	-4,17	0,001623	0,332831		
TC0100002606.mm.1	4,1	Ptprn	JUC0100012018.mm.1	-4,27	0,014102	0,447108		
TC0100002606.mm.1	4,1	Ptprn	JUC0100012022.mm.1	-4,51	0,001864	0,337974		
TC0100002606.mm.1	4,1	Ptprn	JUC0100012033.mm.1	-4,92	0,000264	0,28803		
TC0100002606.mm.1	4,1	Ptprn	PSR0100021218.mm.1	-5,09	0,000334	0,28927	Alternative 3' Acce	0,31
TC0100002606.mm.1	4,1	Ptprn	PSR0100021228.mm.1	-6,86	0,000656	0,304044	Cassette Exon	0,41
TC1000001679.mm.1	1,71	Syne1	JUC1000006622.mm.1	4,82	0,045397	0,554085		
TC1000001679.mm.1	1,71	Syne1	JUC1000006580.mm.1	2,72	0,006539	0,391916		
TC1000001679.mm.1	1,71	Syne1	PSR1000012193.mm.1	-2,31	0,010403	0,421477	Cassette Exon	0,15
TC1000001679.mm.1	1,71	Syne1	PSR1000012215.mm.1	-2,4	0,010377	0,421341	Cassette Exon	0,2
TC1000001679.mm.1	1,71	Syne1	JUC1000006621.mm.1	-2,53	0,011649	0,42985		
TC1300000726.mm.1	1,07	Spata31d1b	JUC1300002515.mm.1	4,82	0,040973	0,543536		
TC0100003264.mm.1	1,33	Rgl1	JUC0100015106.mm.1	4,82	0,037233	0,533373		
TC1800000322.mm.1	1,15	Psd2	JUC1800001403.mm.1	4,81	0,029508	0,510135		
TC1800000322.mm.1	1,15	Psd2	JUC1800001393.mm.1	2,09	0,028948	0,508766		
TC0400002977.mm.1	1,06	Haus6	JUC0400012584.mm.1	4,8	0,014536	0,449545		
TC1200001010.mm.1	5,39	Slc24a4	JUC1200003953.mm.1	4,78	0,000081	0,25272		
TC1200001010.mm.1	5,39	Slc24a4	PSR1200007105.mm.1	3,65	0,036343	0,530566	Cassette Exon	0,09
TC1200001010.mm.1	5,39	Slc24a4	JUC1200003966.mm.1	2,98	0,024341	0,49362		
TC1200001010.mm.1	5,39	Slc24a4	PSR1200007097.mm.1	2,45	0,000875	0,311909	Cassette Exon	0,3
TC1200001010.mm.1	5,39	Slc24a4	JUC1200003955.mm.1	2,32	0,023843	0,491997		
TC1200001010.mm.1	5,39	Slc24a4	PSR1200007115.mm.1	2,18	0,001717	0,335996	Alternative 5' Donc	0,1
TC1200001010.mm.1	5,39	Slc24a4	PSR1200007112.mm.1	-2,08	0,000493	0,299348	Alternative 3' Acce	0,1
TC1200001010.mm.1	5,39	Slc24a4	PSR1200007110.mm.1	-2,26	0,001793	0,336311	Cassette Exon	0,29
TC1200001010.mm.1	5,39	Slc24a4	PSR1200007113.mm.1	-2,29	0,016017	0,457051	Intron Retention	0,19
TC1200001010.mm.1	5,39	Slc24a4	PSR1200007100.mm.1	-2,54	0,03271	0,520439	Cassette Exon	0,21
TC1200001010.mm.1	5,39	Slc24a4	PSR1200007109.mm.1	-2,58	0,001844	0,337414	Cassette Exon	0,22
TC1200001010.mm.1	5,39	Slc24a4	JUC1200003964.mm.1	-2,58	0,0033	0,354243		
TC1200001010.mm.1	5,39	Slc24a4	PSR1200007094.mm.1	-2,97	0,000997	0,316361	Alternative 3' Acce	0,39
TC1200001010.mm.1	5,39	Slc24a4	JUC1200003957.mm.1	-3,22	0,006131	0,387388		
TC1200001010.mm.1	5,39	Slc24a4	JUC1200003965.mm.1	-3,24	0,010201	0,419711		
TC1200001010.mm.1	5,39	Slc24a4	JUC1200003959.mm.1	-3,43	0,000589	0,304044		
TC1200001010.mm.1	5,39	Slc24a4	PSR1200007096.mm.1	-3,72	0,010417	0,42148	Cassette Exon	0,22
TC1200001010.mm.1	5,39	Slc24a4	JUC1200003956.mm.1	-3,86	0,003234	0,354243		
TC1200001010.mm.1	5,39	Slc24a4	JUC1200003967.mm.1	-4,35	0,019402	0,473802		
TC1200001010.mm.1	5,39	Slc24a4	JUC1200003961.mm.1	-4,58	0,015436	0,454229		
TC1200001010.mm.1	5,39	Slc24a4	PSR1200007093.mm.1	-7,07	0,007721	0,402552	Alternative 3' Acce	0,39
TC1600000771.mm.1	2,23	Gabrr3	JUC1600003545.mm.1	4,78	0,010465	0,421696		
TC1600000771.mm.1	2,23	Gabrr3	PSR1600006675.mm.1	-2,48	0,00832	0,406313	Cassette Exon	0,27
TC1600000771.mm.1	2,23	Gabrr3	PSR1600006674.mm.1	-2,6	0,016659	0,460023	Cassette Exon	0,27
TC1600000771.mm.1	2,23	Gabrr3	JUC1600003542.mm.1	-3,72	0,02122	0,481286		

TC1600000771.mm.1	2,23	Gabrr3	JUC1600003548.mm.1	-3,85	0,013029	0,439367		
TC0100000551.mm.1	3,97	Map2; A730034C02	JUC0100002615.mm.1	4,77	0,029346	0,509981		
TC0100000551.mm.1	3,97	Map2; A730034C02	PSR0100004455.mm.1	4,37	0,017194	0,462491		
TC0100000551.mm.1	3,97	Map2; A730034C02	JUC0100002609.mm.1	-2,44	0,007323	0,398968		
TC0100000551.mm.1	3,97	Map2; A730034C02	PSR0100004477.mm.1	-2,47	0,007363	0,39938	Cassette Exon	0,12
TC0100000551.mm.1	3,97	Map2; A730034C02	PSR0100004450.mm.1	-2,49	0,001198	0,321568	Cassette Exon	0,24
TC0100000551.mm.1	3,97	Map2; A730034C02	JUC0100002597.mm.1	-2,49	0,012326	0,434392		
TC0100000551.mm.1	3,97	Map2; A730034C02	PSR0100004453.mm.1	-2,54	0,00054	0,304044	Alternative 3' Acce	0,33
TC0100000551.mm.1	3,97	Map2; A730034C02	PSR0100004443.mm.1	-2,79	0,032532	0,519949	Alternative 3' Acce	0,03
TC0100000551.mm.1	3,97	Map2; A730034C02	PSR0100004457.mm.1	-2,8	0,036357	0,530624	Intron Retention	0,5
TC0100000551.mm.1	3,97	Map2; A730034C02	PSR0100004480.mm.1	-2,94	0,020753	0,479248	Alternative 5' Donc	0,21
TC0100000551.mm.1	3,97	Map2; A730034C02	JUC0100002619.mm.1	-2,97	0,005219	0,377078		
TC0100000551.mm.1	3,97	Map2; A730034C02	PSR0100004499.mm.1	-3,07	0,011271	0,426793	Cassette Exon	0,35
TC0100000551.mm.1	3,97	Map2; A730034C02	PSR0100004447.mm.1	-3,34	0,002182	0,344453	Cassette Exon	0,32
TC0100000551.mm.1	3,97	Map2; A730034C02	PSR0100004456.mm.1	-3,47	0,023881	0,492304	Intron Retention	0,5
TC0100000551.mm.1	3,97	Map2; A730034C02	PSR0100004439.mm.1	-3,62	0,005744	0,382993	Alternative 3' Acce	0,13
TC0100000551.mm.1	3,97	Map2; A730034C02	JUC0100002628.mm.1	-3,94	0,043257	0,549133		
TC0100000551.mm.1	3,97	Map2; A730034C02	JUC0100002613.mm.1	-4,03	0,015236	0,452847		
TC0100000551.mm.1	3,97	Map2; A730034C02	PSR0100004448.mm.1	-4,81	0,026947	0,502747	Cassette Exon	0,17
TC0100000551.mm.1	3,97	Map2; A730034C02	PSR0100004445.mm.1	-5,19	0,003091	0,353892	Alternative 3' Acce	0,22
TC0100000551.mm.1	3,97	Map2; A730034C02	PSR0100004451.mm.1	-5,2	0,005958	0,385385	Alternative 3' Acce	0,33
TC0100000551.mm.1	3,97	Map2; A730034C02	JUC0100002623.mm.1	-6,97	0,015806	0,455923		
TC0800001770.mm.1	-1,03	Csmd1	JUC0800007788.mm.1	4,77	0,010257	0,420147		
TC0800001770.mm.1	-1,03	Csmd1	JUC0800007756.mm.1	3,34	0,028889	0,50858		
TC0800001770.mm.1	-1,03	Csmd1	JUC0800007742.mm.1	2,52	0,039391	0,539461		
TC0800001770.mm.1	-1,03	Csmd1	PSR0800014211.mm.1	2,23	0,001242	0,322251	Cassette Exon	0,18
TC0800001770.mm.1	-1,03	Csmd1	PSR0800014265.mm.1	2,19	0,024555	0,494116	Cassette Exon	0,14
TC0800001770.mm.1	-1,03	Csmd1	PSR0800014244.mm.1	2,11	0,021387	0,482022	Cassette Exon	0,12
TC0800001770.mm.1	-1,03	Csmd1	JUC0800007799.mm.1	2,1	0,003124	0,353892		
TC1200000700.mm.1	1,2	Gphn	JUC1200002831.mm.1	4,77	0,0059	0,383835		
TC1200000700.mm.1	1,2	Gphn	PSR1200004987.mm.1	2,58	0,026127	0,499897	Cassette Exon	0,17
TC0200003560.mm.1	-2,37	Nr4a2	JUC0200015384.mm.1	4,77	0,040745	0,543028		
TC0200003560.mm.1	-2,37	Nr4a2	PSR0200030356.mm.1	2,5	0,042818	0,548097		
TC0200003560.mm.1	-2,37	Nr4a2	PSR0200030352.mm.1	2,14	0,024769	0,495014	Cassette Exon	0,08
TC1100001687.mm.1	2,26	Rundc3a	JUC1100008231.mm.1	4,76	0,002435	0,349135		
TC1100001687.mm.1	2,26	Rundc3a	JUC1100008232.mm.1	4,26	0,010396	0,421426		
TC1100001687.mm.1	2,26	Rundc3a	PSR1100015874.mm.1	3,29	0,00207	0,342141	Cassette Exon	0,38
TC1100001687.mm.1	2,26	Rundc3a	PSR1100015865.mm.1	2,09	0,000527	0,304044		
TC1100001687.mm.1	2,26	Rundc3a	PSR1100015889.mm.1	-2,35	0,001796	0,336311	Cassette Exon	0,14
TC1100001687.mm.1	2,26	Rundc3a	PSR1100015873.mm.1	-2,51	0,006786	0,393554	Intron Retention	0,38
TC1100001687.mm.1	2,26	Rundc3a	JUC1100008227.mm.1	-2,59	0,005266	0,378003		
TC1100001687.mm.1	2,26	Rundc3a	PSR1100015886.mm.1	-2,68	0,005785	0,383104	Alternative 3' Acce	0,2
TC1100001687.mm.1	2,26	Rundc3a	PSR1100015879.mm.1	-2,93	0,011473	0,428022	Intron Retention	0,4
TC1100001687.mm.1	2,26	Rundc3a	JUC1100008239.mm.1	-3,06	0,028048	0,505874		
TC1100001687.mm.1	2,26	Rundc3a	PSR1100015887.mm.1	-3,21	0,00098	0,314914	Alternative 3' Acce	0,2
TC1100001687.mm.1	2,26	Rundc3a	PSR1100015866.mm.1	-3,35	0,003389	0,3547	Alternative 3' Acce	0,43
TC1100001687.mm.1	2,26	Rundc3a	PSR1100015892.mm.1	-3,4	0,011023	0,425635	Alternative 5' Donc	0,3
TC1100001687.mm.1	2,26	Rundc3a	PSR1100015875.mm.1	-3,53	0,007508	0,401082	Alternative 3' Acce	0,51

TC1100001687.mm.1	2,26	Rundc3a	PSR1100015871.mm.1	-4,12	0,00105	0,316361	Alternative 3' Acce	0,51
TC1100001687.mm.1	2,26	Rundc3a	JUC1100008236.mm.1	-4,46	0,004228	0,363295		
TC0400004097.mm.1	3,92	Kcnab2; Gm16334	PSR0400034188.mm.1	4,76	0,000007	0,179072	Cassette Exon	0,36
TC0400004097.mm.1	3,92	Kcnab2; Gm16334	PSR0400034172.mm.1	4,4	0,000049	0,24627	Cassette Exon	0,33
TC0400004097.mm.1	3,92	Kcnab2; Gm16334	PSR0400034170.mm.1	4,23	0,00014	0,272178	Cassette Exon	0,25
TC0400004097.mm.1	3,92	Kcnab2; Gm16334	JUC0400017814.mm.1	3	0,001151	0,317344		
TC0400004097.mm.1	3,92	Kcnab2; Gm16334	PSR0400034182.mm.1	-2,08	0,038102	0,535656	Cassette Exon	0,11
TC0400004097.mm.1	3,92	Kcnab2; Gm16334	JUC0400017808.mm.1	-2,18	0,013617	0,44395		
TC0400004097.mm.1	3,92	Kcnab2; Gm16334	PSR0400034179.mm.1	-2,34	0,010824	0,424109		
TC0400004097.mm.1	3,92	Kcnab2; Gm16334	JUC0400017836.mm.1	-2,69	0,025778	0,498465		
TC0400004097.mm.1	3,92	Kcnab2; Gm16334	PSR0400034193.mm.1	-3,21	0,011982	0,432286	Cassette Exon	0,23
TC0400004097.mm.1	3,92	Kcnab2; Gm16334	PSR0400034155.mm.1	-3,26	0,012111	0,432704	Alternative 3' Acce	0,36
TC0400004097.mm.1	3,92	Kcnab2; Gm16334	PSR0400034178.mm.1	-3,32	0,015863	0,456449	Alternative 3' Acce	0,16
TC0400004097.mm.1	3,92	Kcnab2; Gm16334	PSR0400034173.mm.1	-4,63	0,006911	0,394898	Alternative 3' Acce	0,41
TC0400004097.mm.1	3,92	Kcnab2; Gm16334	JUC0400017835.mm.1	-4,67	0,01829	0,468049		
TC0400004097.mm.1	3,92	Kcnab2; Gm16334	PSR0400034160.mm.1	-4,95	0,000901	0,313363	Cassette Exon	0,26
TC0400004097.mm.1	3,92	Kcnab2; Gm16334	PSR0400034180.mm.1	-5,14	0,004624	0,369794	Alternative 5' Donc	0,4
TC0400004097.mm.1	3,92	Kcnab2; Gm16334	PSR0400034201.mm.1	-5,21	0,014008	0,446353	Cassette Exon	0,28
TC0400004097.mm.1	3,92	Kcnab2; Gm16334	PSR0400034198.mm.1	-5,32	0,013412	0,442624	Cassette Exon	0,28
TC0400004097.mm.1	3,92	Kcnab2; Gm16334	PSR0400034191.mm.1	-5,49	0,01305	0,439551	Cassette Exon	0,21
TC0400004097.mm.1	3,92	Kcnab2; Gm16334	JUC0400017828.mm.1	-5,52	0,008211	0,405909		
TC0400004097.mm.1	3,92	Kcnab2; Gm16334	PSR0400034184.mm.1	-5,61	0,006774	0,393372	Cassette Exon	0,16
TC0400004097.mm.1	3,92	Kcnab2; Gm16334	JUC0400017833.mm.1	-5,7	0,004751	0,371039		
TC0400004097.mm.1	3,92	Kcnab2; Gm16334	PSR0400034194.mm.1	-6,2	0,010858	0,424161	Cassette Exon	0,35
TC0400004097.mm.1	3,92	Kcnab2; Gm16334	JUC0400017829.mm.1	-6,51	0,027113	0,503022		
TC0400004097.mm.1	3,92	Kcnab2; Gm16334	PSR0400034195.mm.1	-7,19	0,010557	0,422341	Cassette Exon	0,28
TC0400004097.mm.1	3,92	Kcnab2; Gm16334	PSR0400034196.mm.1	-7,35	0,005711	0,382644	Cassette Exon	0,28
TC0400004097.mm.1	3,92	Kcnab2; Gm16334	PSR0400034190.mm.1	-9,11	0,007375	0,39943	Cassette Exon	0,21
TC0400004097.mm.1	3,92	Kcnab2; Gm16334	JUC0400017820.mm.1	-9,18	0,002882	0,352207		
TC0400004097.mm.1	3,92	Kcnab2; Gm16334	PSR0400034186.mm.1	-9,54	0,004119	0,3623	Cassette Exon	0,21
TC0400004097.mm.1	3,92	Kcnab2; Gm16334	JUC0400017834.mm.1	-9,68	0,001829	0,337273		
TC0400004097.mm.1	3,92	Kcnab2; Gm16334	JUC0400017830.mm.1	-11,45	0,007473	0,400381		
TC0300003150.mm.1	1,26	St6galnac5	PSR0300024725.mm.1	4,76	0,006325	0,389424	Cassette Exon	0,28
TC0200002987.mm.1	-1,11	Trdmt1	JUC0200012527.mm.1	4,76	0,048404	0,560669		
TC0200004636.mm.1	-2,47	Slc4a11	PSR0200039683.mm.1	4,75	0,005393	0,378916	Intron Retention	0,73
TC0200004636.mm.1	-2,47	Slc4a11	PSR0200039662.mm.1	3,54	0,029241	0,509699	Cassette Exon	0,31
TC0200004636.mm.1	-2,47	Slc4a11	PSR0200039666.mm.1	3,17	0,007828	0,403089	Intron Retention	0,46
TC0200004636.mm.1	-2,47	Slc4a11	JUC0200020545.mm.1	2,99	0,003352	0,354243		
TC0200004636.mm.1	-2,47	Slc4a11	PSR0200039663.mm.1	2,93	0,039703	0,540417	Cassette Exon	0,26
TC0200004636.mm.1	-2,47	Slc4a11	JUC0200020542.mm.1	2,59	0,046228	0,556056		
TC0200004636.mm.1	-2,47	Slc4a11	PSR0200039670.mm.1	2,41	0,003399	0,354874	Alternative 3' Acce	0,21
TC0200004636.mm.1	-2,47	Slc4a11	PSR0200039688.mm.1	2,32	0,042881	0,548178	Intron Retention	0,32
TC0200004636.mm.1	-2,47	Slc4a11	PSR0200039679.mm.1	2,28	0,031707	0,517443	Alternative 5' Donc	0,2
TC0200004636.mm.1	-2,47	Slc4a11	PSR0200039677.mm.1	2,25	0,018091	0,46716	Intron Retention	0,3
TC0X00001931.mm.1	4,57	Syn1	JUC0X00006192.mm.1	4,75	0,003812	0,35811		
TC0X00001931.mm.1	4,57	Syn1	JUC0X00006191.mm.1	3,02	0,027247	0,503642		
TC0X00001931.mm.1	4,57	Syn1	JUC0X00006196.mm.1	2,98	0,003028	0,353502		
TC0X00001931.mm.1	4,57	Syn1	PSR0X00012249.mm.1	2,9	0,018901	0,47127	Cassette Exon	0,4

TC0X00001931.mm.1	4,57	Syn1	PSR0X00012253.mm.1	2,86	0,003766	0,357586	Cassette Exon	0,16
TC0X00001931.mm.1	4,57	Syn1	PSR0X00012245.mm.1	-2,36	0,011219	0,426683	Cassette Exon	0,28
TC0X00001931.mm.1	4,57	Syn1	PSR0X00012256.mm.1	-2,63	0,004618	0,369794	Cassette Exon	0,15
TC0X00001931.mm.1	4,57	Syn1	JUC0X00006186.mm.1	-2,74	0,017488	0,463964		
TC0X00001931.mm.1	4,57	Syn1	PSR0X00012243.mm.1	-3,24	0,003205	0,354243	Cassette Exon	0,42
TC0X00001931.mm.1	4,57	Syn1	JUC0X00006195.mm.1	-3,28	0,004144	0,3623		
TC0X00001931.mm.1	4,57	Syn1	JUC0X00006190.mm.1	-3,45	0,001914	0,339584		
TC0X00001931.mm.1	4,57	Syn1	PSR0X00012246.mm.1	-3,77	0,004727	0,370888	Cassette Exon	0,39
TC0X00001931.mm.1	4,57	Syn1	JUC0X00006189.mm.1	-4,67	0,003009	0,353447		
TC0X00001931.mm.1	4,57	Syn1	PSR0X00012244.mm.1	-5,51	0,003936	0,359818	Intron Retention	0,3
TC0X00001931.mm.1	4,57	Syn1	JUC0X00006187.mm.1	-6,82	0,000431	0,297771		
TC1300001997.mm.1	2,27	Shc3	JUC1300006420.mm.1	4,75	0,000469	0,298999		
TC1300001997.mm.1	2,27	Shc3	PSR1300012439.mm.1	4,57	0,000069	0,250992	Cassette Exon	0,32
TC1300001997.mm.1	2,27	Shc3	PSR1300012440.mm.1	3,61	0,00816	0,40551	Cassette Exon	0,37
TC1300001997.mm.1	2,27	Shc3	PSR1300012443.mm.1	3,32	0,003589	0,356223	Cassette Exon	0,23
TC1300001997.mm.1	2,27	Shc3	JUC1300006414.mm.1	-2,22	0,042775	0,548097		
TC1300001997.mm.1	2,27	Shc3	PSR1300012434.mm.1	-2,41	0,008187	0,40568	Cassette Exon	0,19
TC1600000711.mm.1	-1,38	Gm19723	JUC1600003285.mm.1	4,75	0,004148	0,3623		
TC0X00002797.mm.1	1,21	Abcb7	JUC0X00008913.mm.1	4,75	0,006033	0,386586		
TC1200001625.mm.1	2,86	Hdac9	JUC1200006294.mm.1	4,74	0,001998	0,341091		
TC1200001625.mm.1	2,86	Hdac9	PSR1200011355.mm.1	2,5	0,007719	0,402552	Cassette Exon	0,17
TC1200001625.mm.1	2,86	Hdac9	JUC1200006277.mm.1	-2,06	0,022057	0,485327		
TC1200001625.mm.1	2,86	Hdac9	PSR1200011352.mm.1	-2,22	0,014923	0,451919	Cassette Exon	0,21
TC1200001625.mm.1	2,86	Hdac9	PSR1200011330.mm.1	-2,27	0,036594	0,531454	Cassette Exon	0,2
TC1200001625.mm.1	2,86	Hdac9	PSR1200011329.mm.1	-2,35	0,04475	0,552338	Cassette Exon	0,13
TC1200001625.mm.1	2,86	Hdac9	PSR1200011361.mm.1	-2,35	0,01973	0,474922	Cassette Exon	0,13
TC1200001625.mm.1	2,86	Hdac9	PSR1200011337.mm.1	-2,42	0,013995	0,446353	Cassette Exon	0,27
TC1200001625.mm.1	2,86	Hdac9	JUC1200006269.mm.1	-2,42	0,033505	0,523018		
TC1200001625.mm.1	2,86	Hdac9	PSR1200011325.mm.1	-2,79	0,018083	0,46716	Cassette Exon	0,3
TC1200001625.mm.1	2,86	Hdac9	JUC1200006266.mm.1	-2,8	0,001974	0,340775		
TC1200001625.mm.1	2,86	Hdac9	PSR1200011354.mm.1	-3,12	0,017296	0,463191	Alternative 3' Acce	0,33
TC1200001625.mm.1	2,86	Hdac9	PSR1200011338.mm.1	-3,14	0,0069	0,394768	Cassette Exon	0,33
TC1200001625.mm.1	2,86	Hdac9	PSR1200011359.mm.1	-3,26	0,010612	0,422786	Cassette Exon	0,28
TC1200001625.mm.1	2,86	Hdac9	PSR1200011339.mm.1	-3,38	0,004927	0,373093	Cassette Exon	0,36
TC1200001625.mm.1	2,86	Hdac9	PSR1200011340.mm.1	-3,38	0,004076	0,362082	Cassette Exon	0,36
TC1200001625.mm.1	2,86	Hdac9	PSR1200011333.mm.1	-3,55	0,007228	0,39826	Cassette Exon	0,38
TC1200001625.mm.1	2,86	Hdac9	PSR1200011332.mm.1	-3,67	0,013746	0,444758	Cassette Exon	0,33
TC1200001625.mm.1	2,86	Hdac9	PSR1200011328.mm.1	-3,75	0,008554	0,408183	Cassette Exon	0,39
TC1200001625.mm.1	2,86	Hdac9	JUC1200006282.mm.1	-4,27	0,002494	0,349135		
TC1200001625.mm.1	2,86	Hdac9	PSR1200011360.mm.1	-4,37	0,016522	0,459611	Cassette Exon	0,32
TC1200001625.mm.1	2,86	Hdac9	PSR1200011331.mm.1	-4,73	0,019245	0,473063	Cassette Exon	0,36
TC1200001625.mm.1	2,86	Hdac9	JUC1200006273.mm.1	-4,99	0,047538	0,558701		
TC1200001625.mm.1	2,86	Hdac9	JUC1200006271.mm.1	-5,21	0,025304	0,497227		
TC1200001625.mm.1	2,86	Hdac9	PSR1200011323.mm.1	-5,27	0,008613	0,408434	Alternative 3' Acce	0,4
TC1200001625.mm.1	2,86	Hdac9	PSR1200011362.mm.1	-6,55	0,010951	0,425009	Cassette Exon	0,41
TC1200001625.mm.1	2,86	Hdac9	PSR1200011336.mm.1	-6,84	0,01029	0,420394	Cassette Exon	0,35
TC1200001625.mm.1	2,86	Hdac9	JUC1200006264.mm.1	-6,96	0,001777	0,336311		
TC1200001625.mm.1	2,86	Hdac9	JUC1200006291.mm.1	-7,08	0,007939	0,403819		

TC1200001625.mm.1	2,86	Hdac9	JUC1200006275.mm.1	-7,27	0,003521	0,355749		
TC1200001625.mm.1	2,86	Hdac9	PSR1200011334.mm.1	-7,34	0,001945	0,340349	Cassette Exon	0,38
TC1200001625.mm.1	2,86	Hdac9	JUC1200006288.mm.1	-7,84	0,009431	0,416082		
TC1200001625.mm.1	2,86	Hdac9	JUC1200006262.mm.1	-8,52	0,003498	0,355638		
TC0700003342.mm.1	6,51		PSR0700028636.mm.1	4,74	0,001603	0,332773	Cassette Exon	0,17
TC0700003342.mm.1	6,51		JUC0700015155.mm.1	-3,42	0,034066	0,524583		
TC0700003342.mm.1	6,51		PSR0700028637.mm.1	-4,74	0,001603	0,332773	Cassette Exon	0,39
TC1400001455.mm.1	1,05	Fezf2	JUC1400005870.mm.1	4,74	0,015021	0,452111		
TC1400001455.mm.1	1,05	Fezf2	JUC1400005872.mm.1	4,05	0,02581	0,498589		
TC1400001455.mm.1	1,05	Fezf2	PSR1400010933.mm.1	2,43	0,036503	0,531304	Cassette Exon	0,27
TC0200001033.mm.1	-1,28	Dync1i2	JUC0200004265.mm.1	4,73	0,023975	0,492725		
TC0200001033.mm.1	-1,28	Dync1i2	PSR0200008319.mm.1	4,24	0,01038	0,421341	Cassette Exon	0,46
TC0200001033.mm.1	-1,28	Dync1i2	PSR0200008306.mm.1	2,04	0,006527	0,391502	Cassette Exon	0,1
TC1700001439.mm.1	1,84	Pacrg	JUC1700006970.mm.1	4,73	0,011435	0,427663		
TC1700001439.mm.1	1,84	Pacrg	PSR1700012817.mm.1	2,09	0,016709	0,460199	Mutually Exclusive	0,27
TC1700001439.mm.1	1,84	Pacrg	PSR1700012814.mm.1	-2,06	0,004805	0,371408	Cassette Exon	0,1
TC1700001439.mm.1	1,84	Pacrg	PSR1700012813.mm.1	-2,23	0,008173	0,405603	Cassette Exon	0,1
TC1700001439.mm.1	1,84	Pacrg	PSR1700012818.mm.1	-2,56	0,002382	0,348564	Mutually Exclusive	0,27
TC0100002071.mm.1	-1,32	Fam135a	JUC0100009574.mm.1	4,73	0,001099	0,317328		
TC0100002071.mm.1	-1,32	Fam135a	PSR0100016852.mm.1	2,55	0,049199	0,562457	Alternative 5' Donc	0,15
TC0700001983.mm.1	1,02	Ppp2r2d	JUC0700009198.mm.1	4,73	0,000356	0,290838		
TC1300002747.mm.1	2,31	Nim1k	JUC1300009982.mm.1	4,72	0,006295	0,389009		
TC1300002747.mm.1	2,31	Nim1k	JUC1300009981.mm.1	2,14	0,01222	0,433326		
TC1300002747.mm.1	2,31	Nim1k	PSR1300018587.mm.1	-2,17	0,0342	0,525083	Cassette Exon	0,01
TC1300002747.mm.1	2,31	Nim1k	PSR1300018586.mm.1	-2,3	0,008098	0,405071	Alternative 5' Donc	0,17
TC1300002747.mm.1	2,31	Nim1k	JUC1300009983.mm.1	-2,35	0,010666	0,423012		
TC0X00001526.mm.1	-1,12	Huwe1; Mir3113	JUC0X00004804.mm.1	4,72	0,001885	0,338712		
TC0X00001526.mm.1	-1,12	Huwe1; Mir3113	JUC0X00004850.mm.1	2,56	0,009158	0,413447		
TC0X00001526.mm.1	-1,12	Huwe1; Mir3113	JUC0X00004794.mm.1	2,39	0,006347	0,389463		
TC0X00000808.mm.1	2,64	Dmd	JUC0X00002717.mm.1	4,71	0,002773	0,350871		
TC0X00000808.mm.1	2,64	Dmd	PSR0X00005402.mm.1	3,95	0,011979	0,432253	Cassette Exon	0,41
TC0X00000808.mm.1	2,64	Dmd	PSR0X00005405.mm.1	3,75	0,00205	0,341391	Cassette Exon	0,42
TC0X00000808.mm.1	2,64	Dmd	JUC0X00002705.mm.1	3,37	0,048315	0,56048		
TC0X00000808.mm.1	2,64	Dmd	JUC0X00002712.mm.1	3,07	0,000205	0,28803		
TC0X00000808.mm.1	2,64	Dmd	PSR0X00005424.mm.1	2,58	0,006786	0,393554	Cassette Exon	0,09
TC0X00000808.mm.1	2,64	Dmd	PSR0X00005423.mm.1	2,53	0,000167	0,272566	Cassette Exon	0,06
TC0X00000808.mm.1	2,64	Dmd	JUC0X00002754.mm.1	2,51	0,017762	0,465425		
TC0X00000808.mm.1	2,64	Dmd	PSR0X00005406.mm.1	2,41	0,010127	0,419203	Cassette Exon	0,23
TC0X00000808.mm.1	2,64	Dmd	JUC0X00002715.mm.1	2,35	0,015924	0,456718		
TC0X00000808.mm.1	2,64	Dmd	PSR0X00005408.mm.1	2,31	0,004466	0,367639	Cassette Exon	0,21
TC0X00000808.mm.1	2,64	Dmd	JUC0X00002706.mm.1	2,28	0,005591	0,381035		
TC0X00000808.mm.1	2,64	Dmd	JUC0X00002708.mm.1	2,22	0,012935	0,438537		
TC0X00000808.mm.1	2,64	Dmd	PSR0X00005420.mm.1	2,18	0,001474	0,329052	Cassette Exon	0,24
TC0X00000808.mm.1	2,64	Dmd	PSR0X00005409.mm.1	2,17	0,042634	0,548097	Cassette Exon	0,23
TC0X00000808.mm.1	2,64	Dmd	PSR0X00005433.mm.1	2,08	0,002553	0,349501	Alternative 3' Acce	0,19
TC0X00000808.mm.1	2,64	Dmd	PSR0X00005407.mm.1	2,01	0,007454	0,400326	Cassette Exon	0,21
TC0X00000808.mm.1	2,64	Dmd	JUC0X00002707.mm.1	2,01	0,022241	0,485896		
TC0X00000808.mm.1	2,64	Dmd	JUC0X00002703.mm.1	-2,01	0,043804	0,550456		

TC0X00000808.mm.1	2,64	Dmd	PSR0X00005456.mm.1	-2,03	0,016275	0,45836	Cassette Exon	0,14
TC0X00000808.mm.1	2,64	Dmd	PSR0X00005367.mm.1	-2,04	0,003803	0,358103	Cassette Exon	0,09
TC0X00000808.mm.1	2,64	Dmd	PSR0X00005454.mm.1	-2,13	0,011371	0,427487	Cassette Exon	0,2
TC0X00000808.mm.1	2,64	Dmd	PSR0X00005460.mm.1	-2,26	0,049177	0,562376	Cassette Exon	0,17
TC0X00000808.mm.1	2,64	Dmd	JUC0X00002734.mm.1	-2,32	0,010747	0,423772		
TC0X00000808.mm.1	2,64	Dmd	PSR0X00005463.mm.1	-2,37	0,033745	0,523558	Cassette Exon	0,11
TC0X00000808.mm.1	2,64	Dmd	PSR0X00005422.mm.1	-2,38	0,037309	0,533573	Cassette Exon	0,22
TC0X00000808.mm.1	2,64	Dmd	JUC0X00002745.mm.1	-2,4	0,015973	0,456885		
TC0X00000808.mm.1	2,64	Dmd	PSR0X00005478.mm.1	-2,51	0,006527	0,391502	Alternative 5' Donc	0,1
TC0X00000808.mm.1	2,64	Dmd	PSR0X00005455.mm.1	-2,53	0,01629	0,458454	Cassette Exon	0,23
TC0X00000808.mm.1	2,64	Dmd	PSR0X00005471.mm.1	-2,54	0,011826	0,430458	Cassette Exon	0,11
TC0X00000808.mm.1	2,64	Dmd	PSR0X00005370.mm.1	-2,66	0,002912	0,352289		
TC0X00000808.mm.1	2,64	Dmd	PSR0X00005450.mm.1	-2,72	0,012927	0,438472	Cassette Exon	0,31
TC0X00000808.mm.1	2,64	Dmd	PSR0X00005452.mm.1	-2,73	0,035061	0,527574	Alternative 5' Donc	0,19
TC0X00000808.mm.1	2,64	Dmd	JUC0X00002759.mm.1	-2,74	0,008793	0,409822		
TC0X00000808.mm.1	2,64	Dmd	JUC0X00002746.mm.1	-2,78	0,049557	0,563325		
TC0X00000808.mm.1	2,64	Dmd	JUC0X00002739.mm.1	-2,83	0,034328	0,525546		
TC0X00000808.mm.1	2,64	Dmd	PSR0X00005372.mm.1	-2,89	0,047012	0,557749	Cassette Exon	0,32
TC0X00000808.mm.1	2,64	Dmd	PSR0X00005473.mm.1	-3,14	0,022642	0,487549	Cassette Exon	0,11
TC0X00000808.mm.1	2,64	Dmd	JUC0X00002732.mm.1	-3,21	0,042654	0,548097		
TC0X00000808.mm.1	2,64	Dmd	PSR0X00005371.mm.1	-3,22	0,00447	0,367767		
TC0X00000808.mm.1	2,64	Dmd	PSR0X00005458.mm.1	-3,4	0,001046	0,316361	Alternative 5' Donc	0,22
TC0X00000808.mm.1	2,64	Dmd	PSR0X00005459.mm.1	-3,7	0,016649	0,459985	Cassette Exon	0,27
TC0X00000808.mm.1	2,64	Dmd	PSR0X00005448.mm.1	-3,73	0,014936	0,451968	Cassette Exon	0,31
TC0X00000808.mm.1	2,64	Dmd	JUC0X00002675.mm.1	-4,62	0,005582	0,381035		
TC0X00000808.mm.1	2,64	Dmd	JUC0X00002758.mm.1	-4,76	0,015443	0,454271		
TC0X00000808.mm.1	2,64	Dmd	PSR0X00005369.mm.1	-4,83	0,013434	0,442693	Cassette Exon	0,41
TC0X00000808.mm.1	2,64	Dmd	JUC0X00002747.mm.1	-5,07	0,017728	0,465216		
TC0X00000808.mm.1	2,64	Dmd	PSR0X00005436.mm.1	-5,28	0,001902	0,338712	Cassette Exon	0,3
TC1400001125.mm.1	1,15	Tdrd3	PSR1400008984.mm.1	4,71	0,044866	0,552613	Cassette Exon	0,24
TC0800000274.mm.1	-1,08	Tpte	JUC0800001086.mm.1	4,71	0,030784	0,514144		
TC0800000274.mm.1	-1,08	Tpte	JUC0800001112.mm.1	-3,17	0,023564	0,490947		
TC0200003603.mm.1	4,57	Pla2r1	JUC0200015608.mm.1	4,7	0,001967	0,340561		
TC0200003603.mm.1	4,57	Pla2r1	JUC0200015600.mm.1	3,6	0,031576	0,517177		
TC0200003603.mm.1	4,57	Pla2r1	JUC0200015609.mm.1	2,46	0,012313	0,434392		
TC0200003603.mm.1	4,57	Pla2r1	JUC0200015606.mm.1	2,35	0,000269	0,28803		
TC0200003603.mm.1	4,57	Pla2r1	PSR0200030756.mm.1	2,27	0,038769	0,537422	Cassette Exon	0,32
TC0200003603.mm.1	4,57	Pla2r1	PSR0200030737.mm.1	-2,06	0,001027	0,316361	Cassette Exon	0,22
TC0200003603.mm.1	4,57	Pla2r1	PSR0200030741.mm.1	-2,17	0,021362	0,481914	Intron Retention	0,23
TC0200003603.mm.1	4,57	Pla2r1	PSR0200030744.mm.1	-2,57	0,000038	0,244636	Alternative 5' Donc	0,17
TC0200003603.mm.1	4,57	Pla2r1	JUC0200015607.mm.1	-2,7	0,021435	0,482126		
TC0200003603.mm.1	4,57	Pla2r1	PSR0200030743.mm.1	-2,77	0,001354	0,325427	Intron Retention	0,22
TC0200003603.mm.1	4,57	Pla2r1	PSR0200030739.mm.1	-3,01	0,000992	0,316265	Alternative 3' Acce	0,28
TC0200003603.mm.1	4,57	Pla2r1	PSR0200030721.mm.1	-3,08	0,001084	0,317191	Cassette Exon	0,36
TC0200003603.mm.1	4,57	Pla2r1	JUC0200015596.mm.1	-3,16	0,005218	0,377078		
TC0200003603.mm.1	4,57	Pla2r1	PSR0200030738.mm.1	-3,36	0,016677	0,460085	Cassette Exon	0,22
TC0200003603.mm.1	4,57	Pla2r1	PSR0200030733.mm.1	-3,71	0,009162	0,413447	Intron Retention	0,53
TC0200003603.mm.1	4,57	Pla2r1	JUC0200015587.mm.1	-3,89	0,014951	0,451968		

TC0200003603.mm.1	4,57	Pla2r1	PSR0200030720.mm.1	-4,26	0,001928	0,340171	Intron Retention	0,41
TC0200003603.mm.1	4,57	Pla2r1	PSR0200030762.mm.1	-4,28	0,001154	0,317344	Alternative 3' Acce	0,36
TC0200003603.mm.1	4,57	Pla2r1	PSR0200030735.mm.1	-7	0,006904	0,394866	Cassette Exon	0,22
TC1300002453.mm.1	3,07	Pde8b	PSR1300016381.mm.1	4,7	0,017702	0,465159	Cassette Exon	0,31
TC1300002453.mm.1	3,07	Pde8b	JUC1300008647.mm.1	4,11	0,002171	0,344453		
TC1300002453.mm.1	3,07	Pde8b	JUC1300008648.mm.1	2,16	0,001757	0,335996		
TC1300002453.mm.1	3,07	Pde8b	PSR1300016380.mm.1	2,1	0,048017	0,559595	Cassette Exon	0,27
TC1300002453.mm.1	3,07	Pde8b	PSR1300016379.mm.1	2,04	0,000007	0,179072	Cassette Exon	0,28
TC1300002453.mm.1	3,07	Pde8b	JUC1300008651.mm.1	-2,04	0,046268	0,556184		
TC1300002453.mm.1	3,07	Pde8b	PSR1300016348.mm.1	-2,2	0,016881	0,460797	Cassette Exon	0,18
TC1300002453.mm.1	3,07	Pde8b	PSR1300016362.mm.1	-2,24	0,028929	0,50862	Cassette Exon	0,19
TC1300002453.mm.1	3,07	Pde8b	PSR1300016382.mm.1	-2,35	0,006346	0,389463	Cassette Exon	0,1
TC1300002453.mm.1	3,07	Pde8b	PSR1300016361.mm.1	-2,36	0,024003	0,492783	Cassette Exon	0,19
TC1300002453.mm.1	3,07	Pde8b	JUC1300008658.mm.1	-2,37	0,005126	0,376377		
TC1300002453.mm.1	3,07	Pde8b	PSR1300016394.mm.1	-2,41	0,010702	0,423331	Alternative 3' Acce	0,21
TC1300002453.mm.1	3,07	Pde8b	JUC1300008638.mm.1	-2,47	0,000766	0,308403		
TC1300002453.mm.1	3,07	Pde8b	PSR1300016387.mm.1	-2,96	0,003806	0,358103	Cassette Exon	0,16
TC1300002453.mm.1	3,07	Pde8b	PSR1300016360.mm.1	-3	0,020176	0,477086	Cassette Exon	0,19
TC1300002453.mm.1	3,07	Pde8b	PSR1300016392.mm.1	-3,02	0,001791	0,336311	Cassette Exon	0,16
TC1300002453.mm.1	3,07	Pde8b	PSR1300016390.mm.1	-3,19	0,030893	0,514574	Cassette Exon	0,2
TC1300002453.mm.1	3,07	Pde8b	PSR1300016389.mm.1	-3,35	0,015882	0,456526	Cassette Exon	0,2
TC1300002453.mm.1	3,07	Pde8b	JUC1300008665.mm.1	-3,38	0,042595	0,548047		
TC1300002453.mm.1	3,07	Pde8b	PSR1300016393.mm.1	-3,51	0,002905	0,352262	Alternative 3' Acce	0,21
TC1300002453.mm.1	3,07	Pde8b	PSR1300016388.mm.1	-3,62	0,00676	0,393343	Cassette Exon	0,2
TC1300002453.mm.1	3,07	Pde8b	JUC1300008655.mm.1	-3,73	0,035195	0,528237		
TC1300002453.mm.1	3,07	Pde8b	PSR1300016356.mm.1	-3,99	0,014555	0,449658	Alternative 3' Acce	0,45
TC1300002453.mm.1	3,07	Pde8b	JUC1300008667.mm.1	-3,99	0,021731	0,484184		
TC1300002453.mm.1	3,07	Pde8b	PSR1300016368.mm.1	-4,6	0,00869	0,408815	Cassette Exon	0,33
TC1300002453.mm.1	3,07	Pde8b	JUC1300008663.mm.1	-4,67	0,009915	0,418378		
TC1300002453.mm.1	3,07	Pde8b	JUC1300008636.mm.1	-4,84	0,00036	0,290838		
TC1300002453.mm.1	3,07	Pde8b	JUC1300008666.mm.1	-5,52	0,019528	0,474538		
TC1300002453.mm.1	3,07	Pde8b	JUC1300008660.mm.1	-5,86	0,009101	0,412874		
TC1300002453.mm.1	3,07	Pde8b	JUC1300008634.mm.1	-9,66	0,009893	0,418237		
TC0400004194.mm.1	-1,38	Ubr4	JUC0400007582.mm.1	4,7	0,005932	0,384945		
TC0400004194.mm.1	-1,38	Ubr4	JUC0400007479.mm.1	2,69	0,027903	0,505612		
TC0400004194.mm.1	-1,38	Ubr4	PSR0400014429.mm.1	2,61	0,002975	0,352501	Alternative 3' Acce	0,23
TC0400004194.mm.1	-1,38	Ubr4	JUC0400007572.mm.1	2,58	0,010349	0,421002		
TC0400004194.mm.1	-1,38	Ubr4	JUC0400007515.mm.1	2,44	0,018287	0,468049		
TC0400004194.mm.1	-1,38	Ubr4	JUC0400007574.mm.1	2,37	0,04904	0,561971		
TC0400004194.mm.1	-1,38	Ubr4	PSR0400014412.mm.1	2,26	0,041978	0,546028	Cassette Exon	0,15
TC0400004194.mm.1	-1,38	Ubr4	PSR0400014404.mm.1	2,21	0,004525	0,368648	Cassette Exon	0,13
TC0400004194.mm.1	-1,38	Ubr4	PSR0400014423.mm.1	2,08	0,040238	0,541714	Cassette Exon	0,19
TC0400004194.mm.1	-1,38	Ubr4	PSR0400014381.mm.1	2,08	0,022337	0,486348	Cassette Exon	0,12
TC0400004194.mm.1	-1,38	Ubr4	JUC0400007576.mm.1	2,04	0,005684	0,382387		
TC0200000395.mm.1	1,39	Nsmf	JUC0200001004.mm.1	4,7	0,009157	0,413447		
TC0200000395.mm.1	1,39	Nsmf	PSR0200002144.mm.1	2,24	0,015576	0,454968	Cassette Exon	0,11
TC0200000395.mm.1	1,39	Nsmf	JUC0200000996.mm.1	-2,41	0,044042	0,550824		
TC1800000990.mm.1	1,07	Tmem241	JUC1800004060.mm.1	4,69	0,00917	0,413447		

TC1800000990.mm.1	1,07	Tmem241	JUC1800004067.mm.1	-2,49	0,015396	0,453964	
TC0100003596.mm.1	-1,15		JUC0100016791.mm.1	4,69	0,005535	0,380557	
TC1700001113.mm.1	1,17	Dlgap1	JUC1700005679.mm.1	4,68	0,005543	0,380603	
TC1700001113.mm.1	1,17	Dlgap1	PSR1700010483.mm.1	3,58	0,014457	0,44909 Cassette Exon	0,35
TC1700001113.mm.1	1,17	Dlgap1	JUC1700005681.mm.1	3,58	0,038247	0,535883	
TC1700001113.mm.1	1,17	Dlgap1	PSR1700010480.mm.1	3,34	0,026962	0,502831 Cassette Exon	0,33
TC1700001113.mm.1	1,17	Dlgap1	PSR1700010477.mm.1	3,27	0,006773	0,393372 Cassette Exon	0,41
TC0100003294.mm.1	1,14	Cacna1e	JUC0100015330.mm.1	4,68	0,007766	0,402774	
TC0100003294.mm.1	1,14	Cacna1e	PSR0100026825.mm.1	2,35	0,000466	0,298999 Cassette Exon	0,15
TC1000000036.mm.1	1,2	Vip	JUC1000000126.mm.1	4,68	0,005941	0,385111	
TC0400000485.mm.1	2,9	Frmpd1	JUC0400001676.mm.1	4,67	0,004791	0,37131	
TC0400000485.mm.1	2,9	Frmpd1	PSR0400003408.mm.1	2,87	0,013964	0,446239 Cassette Exon	0,31
TC0400000485.mm.1	2,9	Frmpd1	JUC0400001672.mm.1	2,31	0,014414	0,448786	
TC0400000485.mm.1	2,9	Frmpd1	PSR0400003421.mm.1	2,14	0,025925	0,49907 Cassette Exon	0,18
TC0400000485.mm.1	2,9	Frmpd1	JUC0400001674.mm.1	2,07	0,040238	0,541714	
TC0400000485.mm.1	2,9	Frmpd1	PSR0400003414.mm.1	2,04	0,012147	0,432868 Cassette Exon	0,11
TC0400000485.mm.1	2,9	Frmpd1	PSR0400003417.mm.1	-2,11	0,002025	0,341377 Cassette Exon	0,08
TC0400000485.mm.1	2,9	Frmpd1	PSR0400003406.mm.1	-2,48	0,010237	0,420128 Cassette Exon	0,14
TC0100001806.mm.1	2,46	Esrrg	JUC0100008541.mm.1	4,67	0,002949	0,352501	
TC0100001806.mm.1	2,46	Esrrg	PSR0100015005.mm.1	-2,69	0,00799	0,403853 Cassette Exon	0,29
TC0100001806.mm.1	2,46	Esrrg	PSR0100015003.mm.1	-2,9	0,040522	0,54238 Cassette Exon	0,18
TC0100001806.mm.1	2,46	Esrrg	JUC0100008532.mm.1	-5,74	0,013888	0,445828	
TC0800000741.mm.1	1,04	Sugp2; LOC100503831	JUC0800002731.mm.1	4,67	0,000798	0,311765	
TC0800000741.mm.1	1,04	Sugp2; LOC100503831	PSR0800005144.mm.1	2,26	0,014088	0,446971 Alternative 5' Donc	0,14
TC0800000741.mm.1	1,04	Sugp2; LOC100503831	PSR0800005147.mm.1	2,24	0,011377	0,427487 Alternative 3' Acce	0,18
TC0X00000059.mm.1	6,68	Cacna1f	JUC0X00000095.mm.1	4,64	0,002844	0,352207	
TC0X00000059.mm.1	6,68	Cacna1f	JUC0X00000096.mm.1	3,33	0,008814	0,410077	
TC0X00000059.mm.1	6,68	Cacna1f	JUC0X00000098.mm.1	2,94	0,009551	0,416698	
TC0X00000059.mm.1	6,68	Cacna1f	PSR0X00000227.mm.1	2,3	0,005525	0,380518 Cassette Exon	0,25
TC0X00000059.mm.1	6,68	Cacna1f	JUC0X00000092.mm.1	2,21	0,041841	0,545586	
TC0X00000059.mm.1	6,68	Cacna1f	PSR0X00000220.mm.1	-2,09	0,022673	0,487625 Cassette Exon	0,11
TC0X00000059.mm.1	6,68	Cacna1f	PSR0X00000260.mm.1	-2,2	0,007057	0,396413 Alternative 3' Acce	0,33
TC0X00000059.mm.1	6,68	Cacna1f	PSR0X00000263.mm.1	-2,26	0,024023	0,492873 Alternative 5' Donc	0,2
TC0X00000059.mm.1	6,68	Cacna1f	JUC0X00000117.mm.1	-2,27	0,005247	0,377696	
TC0X00000059.mm.1	6,68	Cacna1f	PSR0X00000226.mm.1	-2,33	0,018204	0,467586 Intron Retention	0,33
TC0X00000059.mm.1	6,68	Cacna1f	JUC0X00000129.mm.1	-2,47	0,046546	0,55676	
TC0X00000059.mm.1	6,68	Cacna1f	JUC0X00000091.mm.1	-2,5	0,002463	0,349135	
TC0X00000059.mm.1	6,68	Cacna1f	PSR0X00000233.mm.1	-2,84	0,001903	0,338712 Cassette Exon	0,3
TC0X00000059.mm.1	6,68	Cacna1f	JUC0X00000116.mm.1	-3,29	0,009275	0,41476	
TC0X00000059.mm.1	6,68	Cacna1f	JUC0X00000114.mm.1	-3,33	0,039732	0,540494	
TC0X00000059.mm.1	6,68	Cacna1f	JUC0X00000102.mm.1	-3,38	0,001124	0,317328	
TC0X00000059.mm.1	6,68	Cacna1f	PSR0X00000270.mm.1	-3,48	0,000118	0,269605 Cassette Exon	0,34
TC0X00000059.mm.1	6,68	Cacna1f	JUC0X00000132.mm.1	-3,53	0,028302	0,506691	
TC0X00000059.mm.1	6,68	Cacna1f	JUC0X00000127.mm.1	-3,56	0,024735	0,494884	
TC0X00000059.mm.1	6,68	Cacna1f	PSR0X00000268.mm.1	-3,64	0,002649	0,349612 Alternative 5' Donc	0,45
TC0X00000059.mm.1	6,68	Cacna1f	PSR0X00000278.mm.1	-3,72	0,000027	0,229631 Intron Retention	0,52
TC0X00000059.mm.1	6,68	Cacna1f	JUC0X00000110.mm.1	-3,75	0,009946	0,41841	
TC0X00000059.mm.1	6,68	Cacna1f	JUC0X00000141.mm.1	-3,78	0,033504	0,523014	

TC0X00000059.mm.1	6,68	Cacna1f	PSR0X00000255.mm.1	-3,94	0,000802	0,311765	Alternative 3' Acce	0,49
TC0X00000059.mm.1	6,68	Cacna1f	PSR0X00000241.mm.1	-4,42	0,002676	0,349612	Cassette Exon	0,55
TC0X00000059.mm.1	6,68	Cacna1f	PSR0X00000240.mm.1	-4,45	0,004178	0,362543	Alternative 5' Donc	0,33
TC0X00000059.mm.1	6,68	Cacna1f	PSR0X00000264.mm.1	-4,65	0,003638	0,356619	Alternative 3' Acce	0,44
TC0X00000059.mm.1	6,68	Cacna1f	PSR0X00000242.mm.1	-5,11	0,000092	0,255472	Cassette Exon	0,07
TC0X00000059.mm.1	6,68	Cacna1f	JUC0X00000131.mm.1	-5,17	0,008278	0,405976		
TC0X00000059.mm.1	6,68	Cacna1f	JUC0X00000136.mm.1	-5,19	0,004265	0,363868		
TC0X00000059.mm.1	6,68	Cacna1f	PSR0X00000286.mm.1	-5,43	0,002301	0,346364	Alternative 5' Donc	0,38
TC0X00000059.mm.1	6,68	Cacna1f	JUC0X00000134.mm.1	-5,47	0,009476	0,416553		
TC0X00000059.mm.1	6,68	Cacna1f	PSR0X00000230.mm.1	-5,78	0,019458	0,474165	Alternative 5' Donc	0,48
TC0X00000059.mm.1	6,68	Cacna1f	JUC0X00000140.mm.1	-5,83	0,006564	0,39213		
TC0X00000059.mm.1	6,68	Cacna1f	PSR0X00000245.mm.1	-7,1	0,002743	0,349639	Intron Retention	0,63
TC0X00000059.mm.1	6,68	Cacna1f	JUC0X00000109.mm.1	-7,68	0,000831	0,311886		
TC0X00000059.mm.1	6,68	Cacna1f	JUC0X00000107.mm.1	-9,52	0,00263	0,349612		
TC0X00000059.mm.1	6,68	Cacna1f	JUC0X00000143.mm.1	-9,71	0,005101	0,375768		
TC0X00000059.mm.1	6,68	Cacna1f	JUC0X00000100.mm.1	-13,2	0,000979	0,314913		
TC0X00000059.mm.1	6,68	Cacna1f	JUC0X00000108.mm.1	-13,86	0,001154	0,317344		
TC0X00000059.mm.1	6,68	Cacna1f	JUC0X00000142.mm.1	-16,33	0,030545	0,51337		
TC1400002086.mm.1	4,54	Nrl	JUC1400008679.mm.1	4,64	0,006537	0,391916		
TC1400002086.mm.1	4,54	Nrl	PSR1400015886.mm.1	2,28	0,031495	0,516965	Cassette Exon	0,26
TC1400002086.mm.1	4,54	Nrl	PSR1400015890.mm.1	-2,2	0,009192	0,413763	Alternative 5' Donc	0,3
TC1400002086.mm.1	4,54	Nrl	JUC1400008682.mm.1	-3,42	0,02945	0,510051		
TC1400002086.mm.1	4,54	Nrl	PSR1400015891.mm.1	-4,77	0,012444	0,43528	Cassette Exon	0,37
TC1400002086.mm.1	4,54	Nrl	PSR1400015883.mm.1	-5,78	0,001311	0,323877	Alternative 3' Acce	0,48
TC1400002086.mm.1	4,54	Nrl	JUC1400008680.mm.1	-7,2	0,021726	0,484124		
TC1200000580.mm.1	1,52	Atl1; Gm3086	JUC1200002154.mm.1	4,64	0,03866	0,537102		
TC1200000580.mm.1	1,52	Atl1; Gm3086	PSR1200003913.mm.1	-2,31	0,023099	0,489699	Cassette Exon	0,15
TC1200000580.mm.1	1,52	Atl1; Gm3086	JUC1200002165.mm.1	-2,56	0,043164	0,548758		
TC1200000580.mm.1	1,52	Atl1; Gm3086	PSR1200003897.mm.1	-3,53	0,04219	0,546939	Alternative 3' Acce	0,41
TC1500001038.mm.1	-1,69	Atf1; Gm1862	JUC1500004646.mm.1	4,63	0,036211	0,530149		
TC1500001038.mm.1	-1,69	Atf1; Gm1862	PSR1500008207.mm.1	2,89	0,007305	0,398847	Alternative 5' Donc	0,16
TC0500002557.mm.1	1,07	Gabrg1	JUC0500012294.mm.1	4,63	0,008833	0,410293		
TC1000000896.mm.1	2,51	Dohh	PSR1000007097.mm.1	4,62	0,042217	0,546983	Alternative 5' Donc	0,47
TC1000000896.mm.1	2,51	Dohh	PSR1000007090.mm.1	-2,04	0,000306	0,288663	Cassette Exon	0,04
TC1000000896.mm.1	2,51	Dohh	PSR1000007087.mm.1	-2,06	0,000808	0,311886	Cassette Exon	0,35
TC1000000896.mm.1	2,51	Dohh	PSR1000007081.mm.1	-2,18	0,002909	0,352289	Alternative 5' Donc	0,16
TC1000000896.mm.1	2,51	Dohh	PSR1000007089.mm.1	-2,37	0,005218	0,377078	Alternative 5' Donc	0,16
TC1000000896.mm.1	2,51	Dohh	PSR1000007080.mm.1	-2,38	0,028547	0,507497	Cassette Exon	0,14
TC1000000896.mm.1	2,51	Dohh	PSR1000007085.mm.1	-2,61	0,003103	0,353892	Cassette Exon	0,35
TC1000000896.mm.1	2,51	Dohh	JUC1000003835.mm.1	-2,64	0,015319	0,453644		
TC1000000896.mm.1	2,51	Dohh	PSR1000007078.mm.1	-2,74	0,000509	0,303579	Cassette Exon	0,14
TC1000000896.mm.1	2,51	Dohh	PSR1000007082.mm.1	-3,11	0,000478	0,298999	Alternative 5' Donc	0,16
TC1000000896.mm.1	2,51	Dohh	PSR1000007077.mm.1	-3,41	0,004629	0,369804	Alternative 5' Donc	0,31
TC1000000896.mm.1	2,51	Dohh	PSR1000007086.mm.1	-3,68	0,000036	0,238339	Cassette Exon	0,35
TC1000000896.mm.1	2,51	Dohh	JUC1000003834.mm.1	-3,69	0,005796	0,383104		
TC1000000896.mm.1	2,51	Dohh	JUC1000003836.mm.1	-4,08	0,002066	0,341993		
TC0800001429.mm.1	1,3	Klhl36	JUC0800006383.mm.1	4,62	0,042086	0,546486		
TC0800001429.mm.1	1,3	Klhl36	PSR0800011643.mm.1	2,4	0,006473	0,390467	Cassette Exon	0,32

TC0800001429.mm.1	1,3	Klhl36	JUC0800006384.mm.1	2,18	0,004878	0,372195		
TC0800001429.mm.1	1,3	Klhl36	PSR0800011644.mm.1	2,16	0,026201	0,50003	Cassette Exon	0,14
TC0800000637.mm.1	1,3	Gm15882	PSR0800004265.mm.1	4,61	0,00626	0,388622	Cassette Exon	0,28
TC0800000637.mm.1	1,3	Gm15882	PSR0800004264.mm.1	-4,61	0,00626	0,388622	Cassette Exon	0,28
TC0500001773.mm.1	-1,07	Cyp3a57	JUC0500008946.mm.1	4,61	0,002018	0,34123		
TC0300002926.mm.1	-1,04	Enpep	JUC0300012081.mm.1	4,61	0,001763	0,336201		
TC0400003803.mm.1	-1,6	Cdc42	JUC0400016310.mm.1	4,61	0,011457	0,427947		
TC0100000696.mm.1	-1,32	1700016L21Rik	JUC0100003399.mm.1	4,58	0,041245	0,544166		
TC0900000019.mm.1	-1,11	Gucy1a2	PSR0900000183.mm.1	4,58	0,008509	0,407344		
TC0900000019.mm.1	-1,11	Gucy1a2	JUC0900000096.mm.1	2,88	0,012023	0,432286		
TC0600001463.mm.1	3,44	Ano2	JUC0600006374.mm.1	4,56	0,001156	0,317344		
TC0600001463.mm.1	3,44	Ano2	JUC0600006388.mm.1	3,51	0,013009	0,439291		
TC0600001463.mm.1	3,44	Ano2	JUC0600006371.mm.1	2,96	0,006857	0,39401		
TC0600001463.mm.1	3,44	Ano2	JUC0600006370.mm.1	2,7	0,004758	0,371039		
TC0600001463.mm.1	3,44	Ano2	PSR0600012088.mm.1	2,39	0,002152	0,343789	Cassette Exon	0,17
TC0600001463.mm.1	3,44	Ano2	JUC0600006378.mm.1	2,27	0,006664	0,393187		
TC0600001463.mm.1	3,44	Ano2	PSR0600012106.mm.1	-2,18	0,032115	0,518585	Cassette Exon	0,1
TC0600001463.mm.1	3,44	Ano2	JUC0600006386.mm.1	-2,42	0,035662	0,528966		
TC0600001463.mm.1	3,44	Ano2	PSR0600012097.mm.1	-2,6	0,000066	0,250119	Cassette Exon	0,06
TC0600001463.mm.1	3,44	Ano2	JUC0600006396.mm.1	-2,89	0,016286	0,458454		
TC0600001463.mm.1	3,44	Ano2	PSR0600012093.mm.1	-3,11	0,030869	0,514456	Cassette Exon	0,13
TC0600001463.mm.1	3,44	Ano2	PSR0600012101.mm.1	-3,23	0,007706	0,402552	Cassette Exon	0,1
TC0600001463.mm.1	3,44	Ano2	PSR0600012072.mm.1	-3,58	0,005047	0,374728	Cassette Exon	0,24
TC0600001463.mm.1	3,44	Ano2	PSR0600012076.mm.1	-3,62	0,007142	0,397526	Cassette Exon	0,44
TC0600001463.mm.1	3,44	Ano2	PSR0600012096.mm.1	-3,87	0,006733	0,393343	Alternative 5' Donc	0,5
TC0600001463.mm.1	3,44	Ano2	JUC0600006395.mm.1	-5,03	0,007355	0,399339		
TC0600001463.mm.1	3,44	Ano2	JUC0600006397.mm.1	-6,07	0,004025	0,361567		
TC0600001463.mm.1	3,44	Ano2	JUC0600006383.mm.1	-6,62	0,000702	0,30566		
TC0900001481.mm.1	-2,73	Ltf	JUC0900006940.mm.1	4,56	0,038126	0,535709		
TC0700000047.mm.1	-1,13	Isoc2a	JUC0700000284.mm.1	4,56	0,00413	0,3623		
TC0X00001277.mm.1	1,12	Drp2	JUC0X00003918.mm.1	4,56	0,018161	0,467432		
TC1200002311.mm.1	1,36	Atg2b	JUC1200008930.mm.1	4,55	0,023178	0,489982		
TC1200002311.mm.1	1,36	Atg2b	JUC1200008937.mm.1	2,57	0,008346	0,406435		
TC1200002311.mm.1	1,36	Atg2b	JUC1200008901.mm.1	-2,6	0,036275	0,530445		
TC1800000908.mm.1	1,11	Rttn; Mir6359; mmu-mir-6	JUC1800003630.mm.1	4,55	0,014086	0,446959		
TC1800000908.mm.1	1,11	Rttn; Mir6359; mmu-mir-6	JUC1800003635.mm.1	2,15	0,041808	0,545586		
TC1000002259.mm.1	1,13	Vps26a	JUC1000008832.mm.1	4,55	0,017381	0,463704		
TC1000002259.mm.1	1,13	Vps26a	JUC1000008839.mm.1	2,82	0,041083	0,543902		
TC1000000813.mm.1	1,48	Hcn2	JUC1000003200.mm.1	4,55	0,005918	0,384299		
TC0500001587.mm.1	1,87	Srrm3	PSR0500014329.mm.1	4,54	0,016712	0,460199	Cassette Exon	0,49
TC0500001587.mm.1	1,87	Srrm3	JUC0500007814.mm.1	4,16	0,009856	0,417898		
TC0500001587.mm.1	1,87	Srrm3	JUC0500007811.mm.1	3,3	0,004354	0,365487		
TC0500001587.mm.1	1,87	Srrm3	JUC0500007813.mm.1	2,71	0,033155	0,521956		
TC0500001587.mm.1	1,87	Srrm3	PSR0500014338.mm.1	-3,16	0,002927	0,352289	Cassette Exon	0,23
TC1900001045.mm.1	1,32	Rtn3; A830039H05Rik	JUC1900005347.mm.1	4,54	0,002314	0,346468		
TC1900001045.mm.1	1,32	Rtn3; A830039H05Rik	JUC1900005345.mm.1	2,96	0,00905	0,412827		
TC1900001045.mm.1	1,32	Rtn3; A830039H05Rik	JUC1900005343.mm.1	2,35	0,046737	0,556856		
TC0500000926.mm.1	-1,22	Fras1	JUC0500004352.mm.1	4,54	0,037699	0,534511		

TC0600002166.mm.1	1,14	Fam131b	JUC0600008972.mm.1	4,54	0,046165	0,555838		
TC0300001999.mm.1	1,43	Nbea	JUC0300008023.mm.1	4,54	0,022285	0,486062		
TC0300001999.mm.1	1,43	Nbea	JUC0300008027.mm.1	2,94	0,00347	0,355628		
TC0300001999.mm.1	1,43	Nbea	JUC0300008001.mm.1	2,55	0,006992	0,395722		
TC0300001999.mm.1	1,43	Nbea	JUC0300008000.mm.1	2,31	0,007562	0,401384		
TC0300001999.mm.1	1,43	Nbea	JUC0300008022.mm.1	2,28	0,040827	0,543283		
TC0300001999.mm.1	1,43	Nbea	JUC0300008015.mm.1	-2,28	0,046785	0,55703		
TC0X00000957.mm.1	9,1	Arr3	JUC0X00003025.mm.1	4,53	0,000947	0,313363		
TC0X00000957.mm.1	9,1	Arr3	JUC0X00003030.mm.1	2,07	0,026919	0,502543		
TC0X00000957.mm.1	9,1	Arr3	JUC0X00003036.mm.1	-2,55	0,032009	0,518367		
TC0X00000957.mm.1	9,1	Arr3	PSR0X00006195.mm.1	-2,72	0,042823	0,548097	Cassette Exon	0,29
TC0X00000957.mm.1	9,1	Arr3	PSR0X00006179.mm.1	-3,02	0,019956	0,476103	Intron Retention	0,48
TC0X00000957.mm.1	9,1	Arr3	JUC0X00003035.mm.1	-3,32	0,002445	0,349135		
TC0X00000957.mm.1	9,1	Arr3	PSR0X00006170.mm.1	-3,83	0,002537	0,349501	Cassette Exon	0,36
TC0X00000957.mm.1	9,1	Arr3	PSR0X00006186.mm.1	-4,02	0,01971	0,474887	Alternative 3' Acce	0,49
TC0X00000957.mm.1	9,1	Arr3	PSR0X00006173.mm.1	-6,56	0,013755	0,444758	Alternative 5' Donc	0,64
TC0X00000957.mm.1	9,1	Arr3	JUC0X00003037.mm.1	-6,81	0,030394	0,512923		
TC0X00000957.mm.1	9,1	Arr3	JUC0X00003028.mm.1	-8,68	0,000026	0,229534		
TC0X00000957.mm.1	9,1	Arr3	PSR0X00006169.mm.1	-9,44	0,02338	0,490202	Cassette Exon	0,41
TC0X00000957.mm.1	9,1	Arr3	JUC0X00003039.mm.1	-48,08	0,010875	0,424174		
TC0100001060.mm.1	1,26	Clasp1	JUC0100005054.mm.1	4,53	0,003144	0,353892		
TC0100001060.mm.1	1,26	Clasp1	JUC0100005059.mm.1	3,34	0,001652	0,334162		
TC0100001060.mm.1	1,26	Clasp1	JUC0100005083.mm.1	-2,34	0,024406	0,4937		
TC1100002426.mm.1	1,15	Eml6	JUC1100011778.mm.1	4,52	0,004228	0,363295		
TC1100002426.mm.1	1,15	Eml6	JUC1100011791.mm.1	2,92	0,002112	0,342919		
TC1100002426.mm.1	1,15	Eml6	PSR1100022702.mm.1	2,71	0,033968	0,524252	Cassette Exon	0,2
TC1100002426.mm.1	1,15	Eml6	JUC1100011808.mm.1	2,68	0,019017	0,471745		
TC1100002426.mm.1	1,15	Eml6	JUC1100011796.mm.1	2,65	0,021781	0,48438		
TC1100002426.mm.1	1,15	Eml6	JUC1100011785.mm.1	2,51	0,001038	0,316361		
TC1100002426.mm.1	1,15	Eml6	JUC1100011792.mm.1	2,51	0,040181	0,541655		
TC1100002426.mm.1	1,15	Eml6	PSR1100022720.mm.1	2,42	0,004018	0,361353	Cassette Exon	0,22
TC1100002426.mm.1	1,15	Eml6	PSR1100022717.mm.1	2,25	0,012426	0,43528	Cassette Exon	0,11
TC1100002426.mm.1	1,15	Eml6	PSR1100022698.mm.1	2,17	0,003038	0,353813	Cassette Exon	0,21
TC1100002426.mm.1	1,15	Eml6	PSR1100022718.mm.1	2,15	0,000883	0,312378	Cassette Exon	0,11
TC1100002426.mm.1	1,15	Eml6	PSR1100022692.mm.1	-4,23	0,023993	0,492783	Alternative 3' Acce	0,46
TC1600001607.mm.1	-1,01	Poglut1	JUC1600006848.mm.1	4,52	0,010248	0,420147		
TC0400002119.mm.1	-1,68	Gm11787	JUC0400009366.mm.1	4,52	0,018857	0,470956		
TC0800002632.mm.1	1,07	Siah1a	PSR0800020223.mm.1	4,51	0,014185	0,447608	Alternative 3' Acce	0,43
TC1000000160.mm.1	1,74	Ahi1	JUC1000000607.mm.1	4,51	0,007893	0,403819		
TC1000000160.mm.1	1,74	Ahi1	PSR1000001149.mm.1	2,67	0,004532	0,368648	Cassette Exon	0,32
TC1000000160.mm.1	1,74	Ahi1	JUC1000000600.mm.1	2,24	0,026728	0,502096		
TC1000000160.mm.1	1,74	Ahi1	JUC1000000609.mm.1	2,15	0,003123	0,353892		
TC1000000160.mm.1	1,74	Ahi1	PSR1000001150.mm.1	2,05	0,008259	0,405976	Cassette Exon	0,18
TC0400000162.mm.1	2,01	Cngb3	JUC0400000491.mm.1	4,51	0,00731	0,398847		
TC0400000162.mm.1	2,01	Cngb3	PSR0400001005.mm.1	-2,18	0,020327	0,477245	Cassette Exon	0,17
TC0400000162.mm.1	2,01	Cngb3	PSR0400001006.mm.1	-2,26	0,038436	0,536405	Cassette Exon	0,12
TC0300002371.mm.1	-1,42	Npr1	JUC0300009659.mm.1	4,51	0,01716	0,46232		
TC0300002371.mm.1	-1,42	Npr1	JUC0300009667.mm.1	2,58	0,007737	0,402774		

TC0300002371.mm.1	-1,42	Npr1	JUC0300009676.mm.1	2,26	0,005901	0,383835		
TC0300002371.mm.1	-1,42	Npr1	PSR0300018461.mm.1	2,02	0,037356	0,533577	Cassette Exon	0,13
TC1700002155.mm.1	2,01	Tmem63b	JUC1700010755.mm.1	4,5	0,002506	0,349237		
TC1700002155.mm.1	2,01	Tmem63b	PSR1700020249.mm.1	-2,07	0,037461	0,533948	Cassette Exon	0,15
TC1500001993.mm.1	1,79	Phf21b	JUC1500008974.mm.1	4,49	0,024848	0,495511		
TC1500001993.mm.1	1,79	Phf21b	PSR1500015816.mm.1	2,2	0,010872	0,424174	Cassette Exon	0,11
TC1500001993.mm.1	1,79	Phf21b	PSR1500015810.mm.1	-2,13	0,000297	0,288663	Alternative 5' Donc	0,19
TC1500001993.mm.1	1,79	Phf21b	PSR1500015806.mm.1	-2,2	0,03684	0,532256	Alternative 5' Donc	0,22
TC1500001993.mm.1	1,79	Phf21b	PSR1500015819.mm.1	-2,2	0,032844	0,520984		
TC1500001993.mm.1	1,79	Phf21b	JUC1500008980.mm.1	-2,84	0,014345	0,448202		
TC0800003161.mm.1	-2,9	Acta1	PSR0800024257.mm.1	4,48	0,02008	0,476581	Alternative 5' Donc	0,48
TC0700003804.mm.1	-1,11	Slco2b1	JUC0700016938.mm.1	4,48	0,002295	0,346364		
TC1000000265.mm.1	-1,56	Echdc1	JUC1000000996.mm.1	4,48	0,012014	0,432286		
TC0700002946.mm.1	1	Iglon5	PSR0700026091.mm.1	4,47	0,019749	0,474932	Cassette Exon	0,26
TC1600001519.mm.1	1,44	Iqcg	JUC1600006487.mm.1	4,46	0,002057	0,341838		
TC0900001113.mm.1	1,21	Tpbg	JUC0900004731.mm.1	4,46	0,039747	0,540496		
TC0500003532.mm.1	2,77	Mmd2	JUC0500017612.mm.1	4,45	0,00377	0,357586		
TC0500003532.mm.1	2,77	Mmd2	PSR0500032366.mm.1	2,81	0,02027	0,477245	Cassette Exon	0,19
TC0500003532.mm.1	2,77	Mmd2	PSR0500032358.mm.1	-3,57	0,030428	0,513134	Alternative 3' Acce	0,41
TC1700002492.mm.1	1,04	Smchd1	JUC1700012242.mm.1	4,45	0,029056	0,508954		
TC1700002492.mm.1	1,04	Smchd1	JUC1700012234.mm.1	2,46	0,012084	0,43255		
TC0400004094.mm.1	4,64	Rnf207	JUC0400017790.mm.1	4,44	0,001814	0,336883		
TC0400004094.mm.1	4,64	Rnf207	PSR0400034104.mm.1	3,71	0,001591	0,331726	Cassette Exon	0,15
TC0400004094.mm.1	4,64	Rnf207	PSR0400034127.mm.1	3,26	0,000287	0,28803	Cassette Exon	0,07
TC0400004094.mm.1	4,64	Rnf207	PSR0400034101.mm.1	2,51	0,025919	0,499027		
TC0400004094.mm.1	4,64	Rnf207	PSR0400034102.mm.1	2,12	0,008436	0,406611	Alternative 3' Acce	0,31
TC0400004094.mm.1	4,64	Rnf207	PSR0400034130.mm.1	-2,14	0,003543	0,3562	Alternative 5' Donc	0,21
TC0400004094.mm.1	4,64	Rnf207	PSR0400034138.mm.1	-2,42	0,000448	0,297771	Cassette Exon	0,27
TC0400004094.mm.1	4,64	Rnf207	PSR0400034126.mm.1	-2,44	0,003285	0,354243	Intron Retention	0,36
TC0400004094.mm.1	4,64	Rnf207	PSR0400034144.mm.1	-2,75	0,013935	0,446202	Alternative 5' Donc	0,33
TC0400004094.mm.1	4,64	Rnf207	PSR0400034136.mm.1	-2,89	0,000587	0,304044	Intron Retention	0,08
TC0400004094.mm.1	4,64	Rnf207	PSR0400034134.mm.1	-3,05	0,010537	0,422281	Alternative 5' Donc	0,36
TC0400004094.mm.1	4,64	Rnf207	PSR0400034128.mm.1	-3,37	0,019452	0,474131	Intron Retention	0,59
TC0400004094.mm.1	4,64	Rnf207	JUC0400017797.mm.1	-3,38	0,010118	0,419126		
TC0400004094.mm.1	4,64	Rnf207	PSR0400034141.mm.1	-3,4	0,003178	0,354243	Alternative 5' Donc	0,33
TC0400004094.mm.1	4,64	Rnf207	PSR0400034122.mm.1	-3,47	0,003526	0,355858	Cassette Exon	0,19
TC0400004094.mm.1	4,64	Rnf207	PSR0400034135.mm.1	-3,85	0,002323	0,3467	Cassette Exon	0,53
TC0400004094.mm.1	4,64	Rnf207	JUC0400017798.mm.1	-3,86	0,002452	0,349135		
TC0400004094.mm.1	4,64	Rnf207	PSR0400034107.mm.1	-3,87	0,006045	0,386586		
TC0400004094.mm.1	4,64	Rnf207	JUC0400017783.mm.1	-4,01	0,005579	0,381035		
TC0400004094.mm.1	4,64	Rnf207	PSR0400034137.mm.1	-4,02	0,016054	0,457283	Intron Retention	0,34
TC0400004094.mm.1	4,64	Rnf207	PSR0400034109.mm.1	-4,06	0,008572	0,40821		
TC0400004094.mm.1	4,64	Rnf207	JUC0400017794.mm.1	-4,82	0,001764	0,336311		
TC0400004094.mm.1	4,64	Rnf207	JUC0400017799.mm.1	-4,88	0,000993	0,316361		
TC0400004094.mm.1	4,64	Rnf207	PSR0400034108.mm.1	-4,9	0,005467	0,379939	Cassette Exon	0,32
TC0400004094.mm.1	4,64	Rnf207	PSR0400034100.mm.1	-5,08	0,003336	0,354243	Alternative 5' Donc	0,4
TC0400004094.mm.1	4,64	Rnf207	JUC0400017803.mm.1	-5,17	0,012671	0,43631		
TC0400004094.mm.1	4,64	Rnf207	JUC0400017795.mm.1	-5,62	0,016375	0,458693		

TC0400004094.mm.1	4,64	Rnf207	JUC0400017802.mm.1	-6,57	0,017584	0,464264		
TC0400004094.mm.1	4,64	Rnf207	JUC0400017785.mm.1	-6,64	0,003379	0,354303		
TC0400004094.mm.1	4,64	Rnf207	JUC0400017804.mm.1	-9,59	0,003246	0,354243		
TC0500001351.mm.1	-1,08	Oas1h	JUC0500006468.mm.1	4,44	0,027304	0,503732		
TC0700002042.mm.1	-1,1	Lrrc56	JUC0700009552.mm.1	4,44	0,008565	0,40821		
TC0200005472.mm.1	-1,25	Eng	JUC0200002778.mm.1	4,43	0,000077	0,25272		
TC0200005472.mm.1	-1,25	Eng	PSR0200005416.mm.1	2,56	0,011423	0,427656	Alternative 5' Donc	0,22
TC0200005472.mm.1	-1,25	Eng	JUC0200002765.mm.1	-2,1	0,004113	0,3623		
TC1800000047.mm.1	1,13	Rab18	JUC1800000170.mm.1	4,43	0,034337	0,525546		
TC1800000620.mm.1	1,2	Camk2a	JUC1800002474.mm.1	4,42	0,027447	0,504045		
TC1800000620.mm.1	1,2	Camk2a	PSR1800004454.mm.1	2,21	0,006727	0,393343	Cassette Exon	0,19
TC1800000620.mm.1	1,2	Camk2a	JUC1800002479.mm.1	2,19	0,007981	0,403853		
TC1800000620.mm.1	1,2	Camk2a	PSR1800004459.mm.1	-2,03	0,029671	0,510913	Alternative 3' Acce	0,12
TC1000001947.mm.1	1,22	Ncoa7	JUC1000007645.mm.1	4,42	0,000122	0,270905		
TC0900000452.mm.1	1,66	Scn3b	JUC0900001528.mm.1	4,41	0,001586	0,331726		
TC0900000452.mm.1	1,66	Scn3b	PSR0900002967.mm.1	3,41	0,000075	0,251428		
TC0900000452.mm.1	1,66	Scn3b	PSR0900002965.mm.1	-2,01	0,001674	0,335182	Alternative 5' Donc	0,14
TC0900000452.mm.1	1,66	Scn3b	PSR0900002956.mm.1	-2,14	0,016524	0,459611	Alternative 3' Acce	0,2
TC0900000452.mm.1	1,66	Scn3b	PSR0900002954.mm.1	-2,36	0,009861	0,417945	Alternative 3' Acce	0,2
TC0900000452.mm.1	1,66	Scn3b	PSR0900002957.mm.1	-2,51	0,014267	0,447979	Intron Retention	0,23
TC0900000452.mm.1	1,66	Scn3b	JUC0900001533.mm.1	-3,06	0,020074	0,476576		
TC1700000745.mm.1	-1,23	Trim26	JUC1700004084.mm.1	4,41	0,048242	0,560272		
TC1700000745.mm.1	-1,23	Trim26	JUC1700004097.mm.1	2,2	0,008253	0,405976		
TC0X00002808.mm.1	1,49	Gm14824	PSR0X00017796.mm.1	4,4	0,002182	0,344453	Alternative 5' Donc	0,41
TC0X00002808.mm.1	1,49	Gm14824	PSR0X00017795.mm.1	3,17	0,014258	0,447947	Alternative 5' Donc	0,41
TC0X00002808.mm.1	1,49	Gm14824	PSR0X00017765.mm.1	2,87	0,021876	0,484675	Cassette Exon	0,17
TC0X00002808.mm.1	1,49	Gm14824	PSR0X00017800.mm.1	2,68	0,002762	0,35022	Cassette Exon	0,16
TC0X00002808.mm.1	1,49	Gm14824	PSR0X00017793.mm.1	2,14	0,018633	0,469964	Cassette Exon	0,09
TC0X00002808.mm.1	1,49	Gm14824	JUC0X00008968.mm.1	-3,26	0,000786	0,309905		
TC1200001911.mm.1	3,49	4930447C04Rik; Six6os1	PSR1200013119.mm.1	4,4	0,01447	0,449178	Alternative 3' Acce	0,25
TC1200001911.mm.1	3,49	4930447C04Rik; Six6os1	PSR1200013112.mm.1	3,22	0,048753	0,561437	Cassette Exon	0,25
TC1200001911.mm.1	3,49	4930447C04Rik; Six6os1	JUC1200007279.mm.1	3,06	0,012042	0,432309		
TC1200001911.mm.1	3,49	4930447C04Rik; Six6os1	PSR1200013141.mm.1	-2,02	0,034538	0,525975	Cassette Exon	0,17
TC1200001911.mm.1	3,49	4930447C04Rik; Six6os1	PSR1200013153.mm.1	-2,22	0,009475	0,416549	Cassette Exon	0,2
TC1200001911.mm.1	3,49	4930447C04Rik; Six6os1	PSR1200013154.mm.1	-2,43	0,010306	0,420505	Cassette Exon	0,2
TC1200001911.mm.1	3,49	4930447C04Rik; Six6os1	PSR1200013130.mm.1	-2,61	0,019203	0,472951	Alternative 5' Donc	0,36
TC1200001911.mm.1	3,49	4930447C04Rik; Six6os1	PSR1200013151.mm.1	-2,62	0,01531	0,453551	Cassette Exon	0,17
TC1200001911.mm.1	3,49	4930447C04Rik; Six6os1	JUC1200007290.mm.1	-2,7	0,027728	0,504987		
TC1200001911.mm.1	3,49	4930447C04Rik; Six6os1	JUC1200007291.mm.1	-2,87	0,024076	0,49304		
TC1200001911.mm.1	3,49	4930447C04Rik; Six6os1	JUC1200007304.mm.1	-3,01	0,01418	0,447583		
TC1200001911.mm.1	3,49	4930447C04Rik; Six6os1	PSR1200013156.mm.1	-3,02	0,002185	0,34446	Alternative 5' Donc	0,2
TC1200001911.mm.1	3,49	4930447C04Rik; Six6os1	JUC1200007296.mm.1	-3,15	0,016438	0,459014		
TC1200001911.mm.1	3,49	4930447C04Rik; Six6os1	JUC1200007280.mm.1	-3,57	0,020294	0,477245		
TC1200001911.mm.1	3,49	4930447C04Rik; Six6os1	JUC1200007294.mm.1	-3,75	0,005659	0,381922		
TC1200001911.mm.1	3,49	4930447C04Rik; Six6os1	JUC1200007292.mm.1	-3,91	0,005566	0,380926		
TC1200001911.mm.1	3,49	4930447C04Rik; Six6os1	PSR1200013144.mm.1	-4,18	0,004167	0,3623	Cassette Exon	0,31
TC1200001911.mm.1	3,49	4930447C04Rik; Six6os1	PSR1200013146.mm.1	-4,28	0,042473	0,547627	Alternative 3' Acce	0,39
TC1200001911.mm.1	3,49	4930447C04Rik; Six6os1	JUC1200007283.mm.1	-4,65	0,012925	0,438459		

TC1200001911.mm.1	3,49	4930447C04Rik; Six6os1	PSR1200013150.mm.1	-5,19	0,000908	0,313363	Cassette Exon	0,32
TC1200001911.mm.1	3,49	4930447C04Rik; Six6os1	JUC1200007285.mm.1	-7,19	0,001479	0,329081		
TC1200001911.mm.1	3,49	4930447C04Rik; Six6os1	JUC1200007305.mm.1	-10,03	0,004786	0,371288		
TC0400000497.mm.1	-1,04	E230008N13Rik	JUC0400001758.mm.1	4,4	0,018912	0,47128		
TC0400000497.mm.1	-1,04	E230008N13Rik	JUC0400001766.mm.1	3,57	0,011487	0,428148		
TC0400000497.mm.1	-1,04	E230008N13Rik	PSR0400003573.mm.1	3,4	0,011689	0,429969	Cassette Exon	0,24
TC0400000497.mm.1	-1,04	E230008N13Rik	JUC0400001760.mm.1	3,29	0,038132	0,535709		
TC0400000497.mm.1	-1,04	E230008N13Rik	PSR0400003570.mm.1	2,91	0,0169	0,460797	Cassette Exon	0,29
TC0400000497.mm.1	-1,04	E230008N13Rik	PSR0400003574.mm.1	2,29	0,028607	0,507714	Cassette Exon	0,13
TC0400000497.mm.1	-1,04	E230008N13Rik	PSR0400003571.mm.1	2,21	0,028483	0,50733	Cassette Exon	0,22
TC0400000497.mm.1	-1,04	E230008N13Rik	PSR0400003577.mm.1	2,11	0,014192	0,447608	Cassette Exon	0,11
TC1600000387.mm.1	1,29	Opa1	JUC1600001934.mm.1	4,4	0,019699	0,474887		
TC1600000387.mm.1	1,29	Opa1	PSR1600003668.mm.1	-2,06	0,017586	0,464264	Alternative 3' Acce	0,12
TC1600000387.mm.1	1,29	Opa1	JUC1600001920.mm.1	-2,12	0,013288	0,441719		
TC0200004970.mm.1	1,04	Edem2	JUC0200021802.mm.1	4,4	0,004391	0,366156		
TC1700001844.mm.1	-1,28	Rasal3	JUC1700009225.mm.1	4,4	0,009501	0,416578		
TC0200002381.mm.1	1,02	Ergic3	JUC0200009679.mm.1	4,39	0,030545	0,51337		
TC0200002381.mm.1	1,02	Ergic3	JUC0200009678.mm.1	4,19	0,045952	0,555359		
TC0200002381.mm.1	1,02	Ergic3	JUC0200009683.mm.1	2,67	0,030904	0,514574		
TC1100001149.mm.1	-1,49	Rab34	JUC1100005533.mm.1	4,39	0,001273	0,322251		
TC1100001149.mm.1	-1,49	Rab34	JUC1100005534.mm.1	2,47	0,035967	0,529457		
TC0X00001306.mm.1	1,11	Gprasp1; Armcx5; Mir764	PSR0X00008194.mm.1	4,38	0,005701	0,382524	Cassette Exon	0,35
TC0X00001306.mm.1	1,11	Gprasp1; Armcx5; Mir764	PSR0X00008193.mm.1	4,1	0,023344	0,490143	Cassette Exon	0,3
TC0X00001306.mm.1	1,11	Gprasp1; Armcx5; Mir764	PSR0X00008198.mm.1	2,79	0,017863	0,466232	Cassette Exon	0,11
TC0X00001306.mm.1	1,11	Gprasp1; Armcx5; Mir764	PSR0X00008196.mm.1	2,61	0,020208	0,477145	Cassette Exon	0,26
TC0X00001306.mm.1	1,11	Gprasp1; Armcx5; Mir764	PSR0X00008195.mm.1	2,42	0,012263	0,433955	Cassette Exon	0,27
TC0X00001306.mm.1	1,11	Gprasp1; Armcx5; Mir764	PSR0X00008192.mm.1	2,4	0,038737	0,537362	Cassette Exon	0,25
TC0X00001306.mm.1	1,11	Gprasp1; Armcx5; Mir764	JUC0X00004025.mm.1	2,08	0,017429	0,463827		
TC0X00001306.mm.1	1,11	Gprasp1; Armcx5; Mir764	PSR0X00008157.mm.1	2,01	0,020713	0,479166	Alternative 3' Acce	0,14
TC1000000255.mm.1	-3,86		PSR1000001786.mm.1	4,38	0,001283	0,322295	Alternative 3' Acce	0,16
TC1900000333.mm.1	-1,93	E030003E18Rik	JUC1900001710.mm.1	4,38	0,007692	0,402552		
TC1900000333.mm.1	-1,93	E030003E18Rik	PSR1900003332.mm.1	2,54	0,018659	0,470148	Cassette Exon	0,04
TC0200005072.mm.1	-1,08	Ptprt	JUC0200022351.mm.1	4,38	0,011824	0,430456		
TC0200005072.mm.1	-1,08	Ptprt	JUC0200022357.mm.1	2,5	0,049563	0,563325		
TC0300003187.mm.1	-1,05	4930570G19Rik	PSR0300024995.mm.1	4,37	0,001882	0,338712	Alternative 3' Acce	0,52
TC0300003187.mm.1	-1,05	4930570G19Rik	PSR0300024996.mm.1	3,44	0,006714	0,393343	Alternative 3' Acce	0,52
TC0300003187.mm.1	-1,05	4930570G19Rik	PSR0300024999.mm.1	2,7	0,039653	0,540212	Alternative 3' Acce	0,27
TC0300003187.mm.1	-1,05	4930570G19Rik	PSR0300024993.mm.1	2,43	0,002546	0,349501	Alternative 3' Acce	0,31
TC0300003187.mm.1	-1,05	4930570G19Rik	JUC0300013109.mm.1	-2,52	0,022877	0,488524		
TC0300003187.mm.1	-1,05	4930570G19Rik	JUC0300013105.mm.1	-2,76	0,019127	0,472431		
TC0300003187.mm.1	-1,05	4930570G19Rik	JUC0300013108.mm.1	-3	0,01006	0,418902		
TC1500002033.mm.1	4,07	Mlc1	PSR1500016127.mm.1	4,37	0,019991	0,476227	Cassette Exon	0,28
TC1500002033.mm.1	4,07	Mlc1	PSR1500016126.mm.1	3,79	0,046402	0,556478	Cassette Exon	0,18
TC1500002033.mm.1	4,07	Mlc1	JUC1500009157.mm.1	3,01	0,017401	0,463753		
TC1500002033.mm.1	4,07	Mlc1	PSR1500016130.mm.1	2,3	0,017732	0,465216	Cassette Exon	0,26
TC1500002033.mm.1	4,07	Mlc1	JUC1500009158.mm.1	2,07	0,040651	0,542648		
TC1500002033.mm.1	4,07	Mlc1	PSR1500016128.mm.1	2,06	0,00798	0,403853	Cassette Exon	0,03
TC1500002033.mm.1	4,07	Mlc1	PSR1500016135.mm.1	-2,37	0,033571	0,523171	Cassette Exon	0,2

TC1500002033.mm.1	4,07	Mlc1	JUC1500009153.mm.1	-2,64	0,021146	0,480814		
TC1500002033.mm.1	4,07	Mlc1	JUC1500009156.mm.1	-2,74	0,000742	0,307808		
TC1500002033.mm.1	4,07	Mlc1	PSR1500016133.mm.1	-2,91	0,03776	0,534682	Cassette Exon	0,26
TC0200000445.mm.1	1,61	Gpsm1	JUC0200001324.mm.1	4,37	0,042826	0,548097		
TC0200000445.mm.1	1,61	Gpsm1	JUC0200001320.mm.1	3,38	0,012147	0,432868		
TC0200000445.mm.1	1,61	Gpsm1	PSR0200002808.mm.1	-2,02	0,005763	0,383104	Cassette Exon	0,1
TC0200000445.mm.1	1,61	Gpsm1	JUC0200001330.mm.1	-2,22	0,007415	0,399901		
TC0200000445.mm.1	1,61	Gpsm1	PSR0200002798.mm.1	-2,23	0,001252	0,322251	Alternative 3' Acce	0,17
TC0100000524.mm.1	1,74	Adam23	JUC0100002471.mm.1	4,37	0,003251	0,354243		
TC0100000524.mm.1	1,74	Adam23	PSR0100004248.mm.1	-2,04	0,023231	0,490032	Cassette Exon	0,09
TC0100000524.mm.1	1,74	Adam23	JUC0100002472.mm.1	-2,38	0,009566	0,416927		
TC0700002762.mm.1	-1,05	Gapdhs	JUC0700013089.mm.1	4,37	0,009676	0,417605		
TC0700002762.mm.1	-1,05	Gapdhs	JUC0700013090.mm.1	2,75	0,015347	0,453763		
TC1100003664.mm.1	-1,37	Cacnb1	JUC1100017660.mm.1	4,37	0,005489	0,379939		
TC0300002648.mm.1	1,33	Lrig2	JUC0300010743.mm.1	4,37	0,003703	0,357266		
TC0300002648.mm.1	1,33	Lrig2	JUC0300010740.mm.1	2,97	0,02793	0,505612		
TC1400000141.mm.1	1,13	Nkiras1	JUC1400000715.mm.1	4,36	0,01619	0,45809		
TC1700002793.mm.1	1,07	Frs3	JUC1700004736.mm.1	4,36	0,012359	0,434689		
TC0500002744.mm.1	-1,09	Cdkl2	JUC0500013172.mm.1	4,36	0,008775	0,40982		
TC1000002161.mm.1	-1,42		JUC1000008232.mm.1	4,36	0,008835	0,410293		
TC0900000573.mm.1	1,53	Cadm1	JUC0900002103.mm.1	4,35	0,001255	0,322251		
TC0900000573.mm.1	1,53	Cadm1	JUC0900002097.mm.1	2,75	0,028708	0,508054		
TC0900000573.mm.1	1,53	Cadm1	PSR0900004055.mm.1	2,19	0,004356	0,365487	Cassette Exon	0,23
TC0900000573.mm.1	1,53	Cadm1	PSR0900004054.mm.1	-2,3	0,004543	0,368648	Alternative 3' Acce	0,21
TC1400001149.mm.1	-1,67		JUC1400004844.mm.1	4,35	0,028779	0,508148		
TC1600000288.mm.1	1,01	Eif4g1	JUC1600001465.mm.1	4,35	0,026815	0,502373		
TC0700003364.mm.1	1,19	B230209E15Rik	PSR0700028828.mm.1	4,34	0,026043	0,49952	Alternative 3' Acce	0,48
TC0700003364.mm.1	1,19	B230209E15Rik	PSR0700028838.mm.1	-2,03	0,033789	0,523781	Intron Retention	0,21
TC0700003364.mm.1	1,19	B230209E15Rik	JUC0700015254.mm.1	-2,39	0,022323	0,486244		
TC1300002605.mm.1	-1,09	Fam159b	PSR1300017746.mm.1	4,34	0,019105	0,472235	Cassette Exon	0,41
TC1300002605.mm.1	-1,09	Fam159b	JUC1300009490.mm.1	4,22	0,001445	0,328204		
TC1500001785.mm.1	-1,37	Cyhr1	PSR1500013835.mm.1	4,34	0,017036	0,461729	Alternative 5' Donc	0,38
TC1500001785.mm.1	-1,37	Cyhr1	JUC1500007908.mm.1	2,61	0,029402	0,509995		
TC0200005115.mm.1	-1,26	Gm14302	PSR0200043623.mm.1	4,34	0,020492	0,478019	Cassette Exon	0,28
TC1400001741.mm.1	-1,24	Ccser2	JUC1400007409.mm.1	4,34	0,019167	0,472737		
TC1400001741.mm.1	-1,24	Ccser2	JUC1400007412.mm.1	-2,19	0,047171	0,558015		
TC1100001256.mm.1	1,19	Ccl4	PSR1100011820.mm.1	4,33	0,004766	0,371039	Alternative 5' Donc	0,48
TC0800001964.mm.1	1,55	Unc5d	JUC0800008514.mm.1	4,33	0,019659	0,474887		
TC0800001964.mm.1	1,55	Unc5d	JUC0800008505.mm.1	3,23	0,047225	0,55815		
TC0800001964.mm.1	1,55	Unc5d	JUC0800008508.mm.1	-2,43	0,000092	0,255472		
TC0500001428.mm.1	-1,22	Kntc1	JUC0500006936.mm.1	4,33	0,004644	0,370181		
TC0400003219.mm.1	-1,27	Dio1	JUC0400013350.mm.1	4,33	0,002281	0,34605		
TC0500000186.mm.1	-1,1	6030443J06Rik	PSR0500001362.mm.1	4,32	0,003497	0,355638	Cassette Exon	0,27
TC1700001974.mm.1	-1,1	Vars2	JUC1700010012.mm.1	4,32	0,013717	0,444758		
TC1700001974.mm.1	-1,1	Vars2	JUC1700010023.mm.1	2,74	0,036541	0,531355		
TC1700001974.mm.1	-1,1	Vars2	PSR1700018742.mm.1	2,06	0,026509	0,501346	Cassette Exon	0,11
TC1000000514.mm.1	-1,25	Lims1	JUC1000001883.mm.1	4,32	0,044819	0,552553		
TC0900002783.mm.1	3,94	Snap91	JUC0900012915.mm.1	4,31	0,004242	0,363295		

TC0900002783.mm.1	3,94	Snap91	JUC0900012896.mm.1	3,09	0,023798	0,491893		
TC0900002783.mm.1	3,94	Snap91	PSR0900023071.mm.1	3,06	0,00648	0,390542	Cassette Exon	0,24
TC0900002783.mm.1	3,94	Snap91	PSR0900023072.mm.1	2,73	0,003108	0,353892	Cassette Exon	0,33
TC0900002783.mm.1	3,94	Snap91	PSR0900023073.mm.1	2,7	0,018765	0,470767	Cassette Exon	0,31
TC0900002783.mm.1	3,94	Snap91	JUC0900012899.mm.1	2,58	0,031314	0,516204		
TC0900002783.mm.1	3,94	Snap91	PSR0900023070.mm.1	2,55	0,021609	0,483425	Cassette Exon	0,2
TC0900002783.mm.1	3,94	Snap91	JUC0900012911.mm.1	2,47	0,041439	0,544646		
TC0900002783.mm.1	3,94	Snap91	PSR0900023074.mm.1	2,34	0,004167	0,3623	Cassette Exon	0,14
TC0900002783.mm.1	3,94	Snap91	PSR0900023066.mm.1	-2,4	0,009015	0,412516	Alternative 3' Acce	0,45
TC0900002783.mm.1	3,94	Snap91	JUC0900012892.mm.1	-2,73	0,005795	0,383104		
TC0900002783.mm.1	3,94	Snap91	PSR0900023051.mm.1	-2,9	0,007753	0,402774	Cassette Exon	0,01
TC0900002783.mm.1	3,94	Snap91	PSR0900023054.mm.1	-3,01	0,002578	0,349501	Cassette Exon	0,15
TC0900002783.mm.1	3,94	Snap91	PSR0900023040.mm.1	-3,11	0,000063	0,250119	Cassette Exon	0,2
TC0900002783.mm.1	3,94	Snap91	PSR0900023056.mm.1	-3,34	0,001809	0,336544	Alternative 5' Donc	0,41
TC0900002783.mm.1	3,94	Snap91	PSR0900023067.mm.1	-3,73	0,006072	0,386691	Alternative 3' Acce	0,45
TC0900002783.mm.1	3,94	Snap91	JUC0900012901.mm.1	-4,06	0,000725	0,306408		
TC0900002783.mm.1	3,94	Snap91	PSR0900023077.mm.1	-4,22	0,006707	0,393343	Cassette Exon	0,19
TC0900002783.mm.1	3,94	Snap91	PSR0900023082.mm.1	-4,44	0,002495	0,349135	Cassette Exon	0,41
TC0900002783.mm.1	3,94	Snap91	JUC0900012924.mm.1	-4,86	0,041446	0,544646		
TC0900002783.mm.1	3,94	Snap91	PSR0900023068.mm.1	-5,25	0,002974	0,352501	Alternative 3' Acce	0,45
TC0900002783.mm.1	3,94	Snap91	JUC0900012918.mm.1	-6,42	0,031622	0,517405		
TC0900002783.mm.1	3,94	Snap91	JUC0900012929.mm.1	-6,6	0,003867	0,358861		
TC0900002783.mm.1	3,94	Snap91	PSR0900023076.mm.1	-6,68	0,001805	0,336311	Cassette Exon	0,19
TC0900002783.mm.1	3,94	Snap91	JUC0900012928.mm.1	-6,78	0,013501	0,443238		
TC0900002783.mm.1	3,94	Snap91	PSR0900023080.mm.1	-7,32	0,014247	0,447947	Cassette Exon	0,19
TC0900002783.mm.1	3,94	Snap91	JUC0900012921.mm.1	-8,05	0,016031	0,45719		
TC0900002783.mm.1	3,94	Snap91	JUC0900012927.mm.1	-8,53	0,022867	0,488492		
TC0900002783.mm.1	3,94	Snap91	JUC0900012932.mm.1	-9,41	0,002017	0,34123		
TC0900002783.mm.1	3,94	Snap91	JUC0900012934.mm.1	-14,72	0,000343	0,289576		
TC0900002783.mm.1	3,94	Snap91	JUC0900012933.mm.1	-19,34	0,012857	0,437778		
TC0900002783.mm.1	3,94	Snap91	JUC0900012935.mm.1	-23,35	0,006876	0,394271		
TC1500000133.mm.1	1,93		PSR1500001136.mm.1	4,31	0,00126	0,322251	Cassette Exon	0,34
TC1500000133.mm.1	1,93		JUC1500000670.mm.1	2,42	0,013039	0,439418		
TC0100002572.mm.1	-1,37	6030407O03Rik	JUC0100011724.mm.1	4,31	0,003212	0,354243		
TC1900001361.mm.1	1,22	Prkg1	JUC1900006602.mm.1	4,31	0,035926	0,529399		
TC0400001064.mm.1	1,14	Mier1	JUC0400003728.mm.1	4,31	0,027016	0,502979		
TC0400001064.mm.1	1,14	Mier1	PSR0400007281.mm.1	2,03	0,02861	0,507714		
TC0400001064.mm.1	1,14	Mier1	JUC0400003731.mm.1	-3,16	0,03638	0,530811		
TC1600001810.mm.1	1,36	Epha6	JUC1600007547.mm.1	4,3	0,001804	0,336311		
TC1600001810.mm.1	1,36	Epha6	PSR1600014553.mm.1	2,45	0,046507	0,556739	Cassette Exon	0,28
TC1600001810.mm.1	1,36	Epha6	PSR1600014556.mm.1	-2,03	0,003697	0,357266		
TC1600001810.mm.1	1,36	Epha6	JUC1600007562.mm.1	-2,6	0,008625	0,408463		
TC1600001810.mm.1	1,36	Epha6	JUC1600007563.mm.1	-2,97	0,014556	0,449658		
TC1600001810.mm.1	1,36	Epha6	JUC1600007550.mm.1	-3,52	0,009094	0,412827		
TC0600001462.mm.1	-1,24	Vwf	JUC0600006338.mm.1	4,3	0,009673	0,417579		
TC0X00000677.mm.1	-1,3	Xlr4b	JUC0X00002174.mm.1	4,3	0,008293	0,406087		
TC1600002145.mm.1	1,78	Dscam	PSR1600016559.mm.1	4,29	0,007772	0,402826	Cassette Exon	0,32
TC1600002145.mm.1	1,78	Dscam	JUC1600008552.mm.1	3,77	0,000303	0,288663		

TC1600002145.mm.1	1,78	Dscam	JUC1600008570.mm.1	3,62	0,000682	0,304417		
TC1600002145.mm.1	1,78	Dscam	JUC1600008568.mm.1	2,38	0,010421	0,42148		
TC1600002145.mm.1	1,78	Dscam	JUC1600008559.mm.1	2,36	0,009706	0,417765		
TC1600002145.mm.1	1,78	Dscam	PSR1600016560.mm.1	2,32	0,013153	0,440265	Cassette Exon	0,13
TC1600002145.mm.1	1,78	Dscam	JUC1600008576.mm.1	2,15	0,043368	0,549472		
TC1600002145.mm.1	1,78	Dscam	JUC1600008574.mm.1	-2,01	0,000601	0,304044		
TC1600002145.mm.1	1,78	Dscam	PSR1600016586.mm.1	-2,26	0,01683	0,460665	Cassette Exon	0,06
TC1600002145.mm.1	1,78	Dscam	JUC1600008572.mm.1	-2,44	0,003118	0,353892		
TC1600002145.mm.1	1,78	Dscam	JUC1600008573.mm.1	-3,76	0,039422	0,539611		
TC0400004164.mm.1	-1,36	Gm10560; Gm16008	PSR0400034986.mm.1	4,29	0,043705	0,550256		
TC0400004164.mm.1	-1,36	Gm10560; Gm16008	PSR0400034973.mm.1	3,8	0,002349	0,34761	Alternative 3' Acce	0,16
TC0400004164.mm.1	-1,36	Gm10560; Gm16008	PSR0400034989.mm.1	2,3	0,030405	0,513027	Alternative 5' Donc	0,08
TC0400004164.mm.1	-1,36	Gm10560; Gm16008	PSR0400034972.mm.1	2,19	0,01958	0,474672	Alternative 3' Acce	0,16
TC0400004164.mm.1	-1,36	Gm10560; Gm16008	PSR0400034983.mm.1	2,17	0,031315	0,516204		
TC1300001263.mm.1	1,78	Ankrd55	PSR1300007956.mm.1	4,28	0,000928	0,313363	Cassette Exon	0,37
TC1300001263.mm.1	1,78	Ankrd55	JUC1300004131.mm.1	3,04	0,014409	0,448751		
TC1300001263.mm.1	1,78	Ankrd55	JUC1300004132.mm.1	2,43	0,049064	0,56202		
TC1300001263.mm.1	1,78	Ankrd55	PSR1300007962.mm.1	-2,79	0,004217	0,363236	Cassette Exon	0,17
TC1300001263.mm.1	1,78	Ankrd55	PSR1300007953.mm.1	-3,22	0,003791	0,357817	Cassette Exon	0,21
TC0900001487.mm.1	2,36	Trank1	JUC0900006977.mm.1	4,28	0,000416	0,297771		
TC0900001487.mm.1	2,36	Trank1	PSR0900012476.mm.1	2,97	0,006045	0,386586	Cassette Exon	0,33
TC0900001487.mm.1	2,36	Trank1	PSR0900012481.mm.1	2,74	0,012805	0,437316	Mutually Exclusive	0,25
TC0900001487.mm.1	2,36	Trank1	PSR0900012497.mm.1	-2,33	0,032425	0,519384	Cassette Exon	0,25
TC0900001487.mm.1	2,36	Trank1	PSR0900012482.mm.1	-2,59	0,04537	0,55404	Mutually Exclusive	0,25
TC0900001487.mm.1	2,36	Trank1	JUC0900006996.mm.1	-3,9	0,007996	0,403853		
TC0800001900.mm.1	1,67	Adam32	JUC0800008254.mm.1	4,28	0,007302	0,398847		
TC0800001900.mm.1	1,67	Adam32	PSR0800015103.mm.1	-2,1	0,031676	0,517405	Cassette Exon	0,19
TC0800001900.mm.1	1,67	Adam32	PSR0800015107.mm.1	-2,45	0,006912	0,394898	Alternative 5' Donc	0,3
TC0800001900.mm.1	1,67	Adam32	JUC0800008241.mm.1	-2,53	0,012274	0,434165		
TC0800001900.mm.1	1,67	Adam32	JUC0800008258.mm.1	-2,62	0,015185	0,452699		
TC0800001900.mm.1	1,67	Adam32	PSR0800015119.mm.1	-2,95	0,039969	0,540973	Alternative 5' Donc	0,23
TC0800001900.mm.1	1,67	Adam32	JUC0800008247.mm.1	-3,42	0,032213	0,518863		
TC0800001900.mm.1	1,67	Adam32	JUC0800008246.mm.1	-6,37	0,015135	0,452699		
TC1200001077.mm.1	1,52	Vrk1	JUC1200004327.mm.1	4,28	0,024481	0,493992		
TC1300002224.mm.1	-1,24		JUC1300007495.mm.1	4,28	0,010779	0,423955		
TC0400000138.mm.1	5,18	Calb1	PSR0400000895.mm.1	4,27	0,000231	0,28803	Cassette Exon	0,41
TC0400000138.mm.1	5,18	Calb1	JUC0400000433.mm.1	3,68	0,009519	0,416602		
TC0400000138.mm.1	5,18	Calb1	PSR0400000886.mm.1	2,04	0,023542	0,490947	Cassette Exon	0,08
TC0400000138.mm.1	5,18	Calb1	PSR0400000899.mm.1	-2,46	0,007541	0,401345	Alternative 5' Donc	0,26
TC0400000138.mm.1	5,18	Calb1	PSR0400000900.mm.1	-3,11	0,003555	0,356223	Alternative 5' Donc	0,26
TC0400000138.mm.1	5,18	Calb1	PSR0400000884.mm.1	-3,75	0,000011	0,179072	Cassette Exon	0,25
TC0400000138.mm.1	5,18	Calb1	PSR0400000883.mm.1	-3,79	0,000222	0,28803	Cassette Exon	0,26
TC0400000138.mm.1	5,18	Calb1	PSR0400000901.mm.1	-5,2	0,001123	0,317328	Alternative 5' Donc	0,26
TC0400000138.mm.1	5,18	Calb1	PSR0400000890.mm.1	-5,21	0,006419	0,389893	Alternative 5' Donc	0,47
TC0400000138.mm.1	5,18	Calb1	PSR0400000888.mm.1	-7,41	0,001782	0,336311	Intron Retention	0,74
TC0400000138.mm.1	5,18	Calb1	PSR0400000891.mm.1	-7,58	0,002289	0,34605	Alternative 5' Donc	0,47
TC0400000138.mm.1	5,18	Calb1	PSR0400000882.mm.1	-8,02	0,001673	0,335182	Cassette Exon	0,26
TC1500001825.mm.1	3,06	Cacng2	JUC1500008126.mm.1	4,27	0,033835	0,524006		

TC1500001825.mm.1	3,06	Cacng2	JUC1500008128.mm.1	3,26	0,006082	0,386691		
TC1500001825.mm.1	3,06	Cacng2	PSR1500014250.mm.1	2,86	0,005741	0,382993	Cassette Exon	0,27
TC1500001825.mm.1	3,06	Cacng2	PSR1500014245.mm.1	-2,21	0,027088	0,503022		
TC1500001825.mm.1	3,06	Cacng2	PSR1500014239.mm.1	-3,72	0,009411	0,415853	Alternative 3' Acce	0,41
TC1400000779.mm.1	1,75	Lrrc16b	JUC1400003249.mm.1	4,27	0,008679	0,408815		
TC1400000779.mm.1	1,75	Lrrc16b	PSR1400006097.mm.1	2,99	0,003334	0,354243	Cassette Exon	0,23
TC1400000779.mm.1	1,75	Lrrc16b	JUC1400003243.mm.1	2,52	0,033763	0,523686		
TC1400000779.mm.1	1,75	Lrrc16b	JUC1400003238.mm.1	2,3	0,006624	0,392458		
TC1400000779.mm.1	1,75	Lrrc16b	PSR1400006130.mm.1	-2,02	0,018445	0,468671	Cassette Exon	0,17
TC1400000779.mm.1	1,75	Lrrc16b	JUC1400003274.mm.1	-2,07	0,039449	0,539705		
TC1400000779.mm.1	1,75	Lrrc16b	JUC1400003241.mm.1	-2,49	0,007585	0,401712		
TC1400000779.mm.1	1,75	Lrrc16b	PSR1400006129.mm.1	-2,6	0,002659	0,349612	Cassette Exon	0,21
TC1400000779.mm.1	1,75	Lrrc16b	JUC1400003270.mm.1	-2,72	0,017259	0,462804		
TC1400000779.mm.1	1,75	Lrrc16b	JUC1400003266.mm.1	-3,49	0,002008	0,341091		
TC1800000613.mm.1	-1,79	Dctn4	JUC1800002432.mm.1	4,27	0,005899	0,383835		
TC1800001272.mm.1	-1,11	Jakmip2	JUC1800005281.mm.1	4,27	0,001051	0,316361		
TC1500000814.mm.1	-1,08		JUC1500003604.mm.1	4,27	0,022006	0,485226		
TC0200001030.mm.1	1,07	Dcaf17	JUC0200004246.mm.1	4,27	0,047947	0,559549		
TC0200001030.mm.1	1,07	Dcaf17	JUC0200004251.mm.1	2,18	0,040667	0,542693		
TC0200001030.mm.1	1,07	Dcaf17	JUC0200004237.mm.1	-2,08	0,02361	0,491012		
TC0700001118.mm.1	-1,12	Acan	JUC0700005032.mm.1	4,26	0,02038	0,477622		
TC0X00000929.mm.1	-1,96	Ar	PSR0X00005981.mm.1	4,25	0,04018	0,541655	Alternative 5' Donc	0,17
TC0X00000929.mm.1	-1,96	Ar	PSR0X00005973.mm.1	3,17	0,024368	0,49362	Cassette Exon	0,28
TC0X00000929.mm.1	-1,96	Ar	PSR0X00005976.mm.1	2,48	0,015501	0,454456	Cassette Exon	0,14
TC0X00000929.mm.1	-1,96	Ar	PSR0X00005988.mm.1	2,46	0,000792	0,310881	Alternative 5' Donc	0,21
TC0X00000929.mm.1	-1,96	Ar	PSR0X00005972.mm.1	2,38	0,006172	0,387873	Cassette Exon	0,19
TC0X00000929.mm.1	-1,96	Ar	PSR0X00005975.mm.1	2,34	0,009865	0,417945	Cassette Exon	0,14
TC0900001261.mm.1	-1,31	Stag1	JUC0900005429.mm.1	4,25	0,020327	0,477245		
TC0900001261.mm.1	-1,31	Stag1	PSR0900009893.mm.1	2,83	0,005808	0,383104	Cassette Exon	0,27
TC0900001261.mm.1	-1,31	Stag1	JUC0900005419.mm.1	2,67	0,024	0,492783		
TC0900001261.mm.1	-1,31	Stag1	JUC0900005434.mm.1	2,45	0,040336	0,541859		
TC0900001261.mm.1	-1,31	Stag1	JUC0900005432.mm.1	2,42	0,030978	0,51471		
TC0900001261.mm.1	-1,31	Stag1	JUC0900005433.mm.1	2,36	0,006287	0,388916		
TC0900001261.mm.1	-1,31	Stag1	JUC0900005430.mm.1	2,11	0,012378	0,434939		
TC1200000624.mm.1	-1,14	Pcnxl4	JUC1200002435.mm.1	4,25	0,01075	0,423812		
TC1200000624.mm.1	-1,14	Pcnxl4	PSR1200004344.mm.1	3,19	0,021875	0,484675	Cassette Exon	0,25
TC1500001054.mm.1	1,6	Slc4a8	JUC1500004716.mm.1	4,24	0,017739	0,465238		
TC1500001054.mm.1	1,6	Slc4a8	JUC1500004711.mm.1	2,5	0,01426	0,447947		
TC1500001054.mm.1	1,6	Slc4a8	PSR1500008359.mm.1	-2,08	0,002742	0,349612	Alternative 3' Acce	0,18
TC1500001054.mm.1	1,6	Slc4a8	JUC1500004730.mm.1	-2,16	0,012417	0,435195		
TC1500001054.mm.1	1,6	Slc4a8	PSR1500008331.mm.1	-2,99	0,006394	0,389654	Intron Retention	0,33
TC1500001054.mm.1	1,6	Slc4a8	JUC1500004727.mm.1	-3,37	0,010103	0,418998		
TC0700001988.mm.1	1,46	Dpysl4	JUC0700009233.mm.1	4,24	0,032759	0,520598		
TC0700001988.mm.1	1,46	Dpysl4	PSR0700017395.mm.1	2,04	0,039493	0,539762	Cassette Exon	0,11
TC0700001988.mm.1	1,46	Dpysl4	PSR0700017403.mm.1	-2,55	0,003133	0,353892	Alternative 5' Donc	0,29
TC0700001988.mm.1	1,46	Dpysl4	JUC0700009229.mm.1	-3,14	0,001982	0,341091		
TC1500002061.mm.1	1,6	Syt10	JUC1500009464.mm.1	4,24	0,026435	0,501198		
TC1500002061.mm.1	1,6	Syt10	JUC1500009463.mm.1	2,28	0,018161	0,467432		

TC0100002696.mm.1	3,44	Dner	JUC0100012398.mm.1	4,23	0,004863	0,371692		
TC0100002696.mm.1	3,44	Dner	JUC0100012400.mm.1	2,09	0,017322	0,463351		
TC0100002696.mm.1	3,44	Dner	JUC0100012403.mm.1	-2,93	0,028411	0,507154		
TC0100002696.mm.1	3,44	Dner	PSR0100021861.mm.1	-3,53	0,014149	0,447466	Cassette Exon	0,22
TC0100002696.mm.1	3,44	Dner	PSR0100021856.mm.1	-4,95	0,001237	0,322251	Alternative 3' Acce	0,48
TC1700002384.mm.1	-1,11	Fbxl17	JUC1700011915.mm.1	4,23	0,018064	0,46716		
TC1100003049.mm.1	2,29	Atp1b2	PSR1100027867.mm.1	4,22	0,005602	0,381115	Cassette Exon	0,59
TC1100003049.mm.1	2,29	Atp1b2	JUC1100014630.mm.1	3,4	0,012237	0,433659		
TC1100003049.mm.1	2,29	Atp1b2	JUC1100014628.mm.1	3,39	0,012006	0,432286		
TC1100003049.mm.1	2,29	Atp1b2	JUC1100014623.mm.1	-3,47	0,002678	0,349612		
TC0200003027.mm.1	1,34	Skida1	PSR0200024978.mm.1	4,22	0,033145	0,521912		
TC0200003027.mm.1	1,34	Skida1	PSR0200024975.mm.1	4,01	0,031555	0,517144	Alternative 5' Donc	0,37
TC0200003027.mm.1	1,34	Skida1	PSR0200024973.mm.1	3,77	0,016622	0,459979	Alternative 5' Donc	0,37
TC0200003027.mm.1	1,34	Skida1	PSR0200024976.mm.1	3,31	0,022374	0,486433		
TC0200003027.mm.1	1,34	Skida1	PSR0200024971.mm.1	3,29	0,017157	0,46232	Alternative 3' Acce	0,19
TC0500003688.mm.1	1,06	N4bp2l2	PSR0500033693.mm.1	4,22	0,003049	0,353892	Cassette Exon	0,29
TC0900000052.mm.1	-1,2	Mmp20	JUC0900000236.mm.1	4,22	0,007852	0,403345		
TC1100000245.mm.1	-1,05	Pus10	JUC1100001133.mm.1	4,22	0,036466	0,531117		
TC1100000245.mm.1	-1,05	Pus10	JUC1100001132.mm.1	2,68	0,030894	0,514574		
TC1900000163.mm.1	4,68	LOC102308570; 1810009A	PSR1900002184.mm.1	4,21	0,016744	0,460378	Cassette Exon	0,25
TC1900000163.mm.1	4,68	LOC102308570; 1810009A	PSR1900002182.mm.1	2,47	0,00591	0,384002	Cassette Exon	0,15
TC1900000163.mm.1	4,68	LOC102308570; 1810009A	PSR1900002190.mm.1	-2,08	0,008529	0,40772	Alternative 3' Acce	0,35
TC1900000163.mm.1	4,68	LOC102308570; 1810009A	JUC1900001098.mm.1	-2,18	0,043163	0,548754		
TC1900000163.mm.1	4,68	LOC102308570; 1810009A	JUC1900001101.mm.1	-4,57	0,010732	0,423745		
TC1900000163.mm.1	4,68	LOC102308570; 1810009A	JUC1900001102.mm.1	-5,51	0,012993	0,439254		
TC1900000163.mm.1	4,68	LOC102308570; 1810009A	PSR1900002189.mm.1	-5,91	0,01572	0,455416	Cassette Exon	0,45
TC1000000734.mm.1	-4,15	Gm16222	PSR1000005247.mm.1	4,2	0,00335	0,354243	Cassette Exon	0,29
TC0900002766.mm.1	1,63	Lca5	JUC0900012802.mm.1	4,19	0,021521	0,482856		
TC0900002766.mm.1	1,63	Lca5	JUC0900012798.mm.1	3,23	0,013447	0,442693		
TC0900002766.mm.1	1,63	Lca5	PSR0900022885.mm.1	3,16	0,005697	0,382524	Cassette Exon	0,33
TC0900002766.mm.1	1,63	Lca5	PSR0900022888.mm.1	2,6	0,029279	0,509874	Cassette Exon	0,34
TC0900002766.mm.1	1,63	Lca5	PSR0900022887.mm.1	2,49	0,001269	0,322251	Cassette Exon	0,34
TC0900002766.mm.1	1,63	Lca5	JUC0900012803.mm.1	-2,25	0,047222	0,558148		
TC1500001056.mm.1	1,81	Scn8a	JUC1500004735.mm.1	4,19	0,000959	0,313363		
TC1500001056.mm.1	1,81	Scn8a	JUC1500004744.mm.1	3,57	0,001382	0,32572		
TC1500001056.mm.1	1,81	Scn8a	JUC1500004736.mm.1	2,68	0,00985	0,417839		
TC1500001056.mm.1	1,81	Scn8a	JUC1500004753.mm.1	-2,07	0,039842	0,540922		
TC1500001056.mm.1	1,81	Scn8a	PSR1500008375.mm.1	-2,16	0,007284	0,398687	Cassette Exon	0,31
TC1500001056.mm.1	1,81	Scn8a	JUC1500004751.mm.1	-2,3	0,027556	0,504437		
TC1500001056.mm.1	1,81	Scn8a	PSR1500008371.mm.1	-2,39	0,006946	0,395542	Cassette Exon	0,08
TC1500001056.mm.1	1,81	Scn8a	JUC1500004754.mm.1	-2,42	0,01255	0,435693		
TC1500001056.mm.1	1,81	Scn8a	JUC1500004768.mm.1	-2,49	0,01882	0,470836		
TC1500001056.mm.1	1,81	Scn8a	PSR1500008390.mm.1	-2,57	0,014433	0,448934		
TC1500001056.mm.1	1,81	Scn8a	JUC1500004761.mm.1	-2,69	0,013598	0,443703		
TC1500001056.mm.1	1,81	Scn8a	JUC1500004749.mm.1	-2,78	0,007762	0,402774		
TC1500001056.mm.1	1,81	Scn8a	JUC1500004731.mm.1	-2,9	0,041087	0,543912		
TC1500001056.mm.1	1,81	Scn8a	JUC1500004767.mm.1	-3	0,015073	0,45241		
TC0600000027.mm.1	1,72	Dync1i1	JUC0600000184.mm.1	4,19	0,00242	0,34896		

TC0600000027.mm.1	1,72	Dync1i1	PSR0600000315.mm.1	2,65	0,010725	0,423745	Cassette Exon	0,22
TC0600000027.mm.1	1,72	Dync1i1	PSR0600000310.mm.1	2,24	0,002409	0,348653	Cassette Exon	0,08
TC0600000027.mm.1	1,72	Dync1i1	PSR0600000293.mm.1	-2,16	0,008179	0,40568	Alternative 3' Acce	0,13
TC0600000027.mm.1	1,72	Dync1i1	JUC0600000182.mm.1	-2,36	0,017988	0,466929		
TC0100003382.mm.1	5,39	Dnm3	JUC0100015848.mm.1	4,18	0,000713	0,306015		
TC0100003382.mm.1	5,39	Dnm3	JUC0100015847.mm.1	3,71	0,033792	0,523795		
TC0100003382.mm.1	5,39	Dnm3	JUC0100015849.mm.1	2,58	0,001623	0,332831		
TC0100003382.mm.1	5,39	Dnm3	PSR0100027727.mm.1	2,32	0,008904	0,410728	Cassette Exon	0,13
TC0100003382.mm.1	5,39	Dnm3	PSR0100027711.mm.1	-2,12	0,015133	0,452685	Cassette Exon	0,31
TC0100003382.mm.1	5,39	Dnm3	PSR0100027709.mm.1	-2,24	0,013908	0,445962	Cassette Exon	0,15
TC0100003382.mm.1	5,39	Dnm3	PSR0100027697.mm.1	-2,54	0,023249	0,490032		
TC0100003382.mm.1	5,39	Dnm3	PSR0100027710.mm.1	-2,75	0,04804	0,559636	Cassette Exon	0,31
TC0100003382.mm.1	5,39	Dnm3	JUC0100015865.mm.1	-2,93	0,043138	0,548664		
TC0100003382.mm.1	5,39	Dnm3	JUC0100015845.mm.1	-3,11	0,0157	0,455416		
TC0100003382.mm.1	5,39	Dnm3	JUC0100015869.mm.1	-3,62	0,025862	0,498741		
TC0100003382.mm.1	5,39	Dnm3	JUC0100015860.mm.1	-3,69	0,007806	0,403089		
TC0100003382.mm.1	5,39	Dnm3	PSR0100027725.mm.1	-4,08	0,015349	0,453763	Alternative 5' Donc	0,4
TC0100003382.mm.1	5,39	Dnm3	PSR0100027744.mm.1	-4,6	0,031309	0,516204	Alternative 5' Donc	0,48
TC0100003382.mm.1	5,39	Dnm3	PSR0100027696.mm.1	-5,23	0,013193	0,440783		
TC0100003382.mm.1	5,39	Dnm3	PSR0100027721.mm.1	-5,35	0,004882	0,372195	Cassette Exon	0,28
TC0100003382.mm.1	5,39	Dnm3	PSR0100027699.mm.1	-5,36	0,028519	0,50743	Alternative 5' Donc	0,47
TC0100003382.mm.1	5,39	Dnm3	PSR0100027733.mm.1	-5,56	0,001296	0,322705	Intron Retention	0,75
TC0100003382.mm.1	5,39	Dnm3	JUC0100015863.mm.1	-6,5	0,00448	0,367858		
TC0100003382.mm.1	5,39	Dnm3	PSR0100027707.mm.1	-6,67	0,00127	0,322251	Cassette Exon	0,41
TC0100003382.mm.1	5,39	Dnm3	PSR0100027742.mm.1	-7,19	0,007022	0,395932	Cassette Exon	0,21
TC0100003382.mm.1	5,39	Dnm3	JUC0100015861.mm.1	-7,19	0,034569	0,525975		
TC0100003382.mm.1	5,39	Dnm3	PSR0100027716.mm.1	-7,94	0,005666	0,381922	Cassette Exon	0,38
TC0100003382.mm.1	5,39	Dnm3	JUC0100015868.mm.1	-8,25	0,022308	0,486127		
TC0100003382.mm.1	5,39	Dnm3	PSR0100027708.mm.1	-8,95	0,007481	0,400439	Cassette Exon	0,41
TC0100003382.mm.1	5,39	Dnm3	PSR0100027738.mm.1	-13,36	0,008201	0,405909	Cassette Exon	0,44
TC0X00000557.mm.1	3,05	C230004F18Rik	PSR0X00003703.mm.1	4,18	0,007046	0,396259	Intron Retention	0,38
TC0X00000557.mm.1	3,05	C230004F18Rik	PSR0X00003712.mm.1	-2,44	0,036583	0,531424	Cassette Exon	0,19
TC0X00000557.mm.1	3,05	C230004F18Rik	JUC0X00001905.mm.1	-5,2	0,038013	0,535454		
TC1600001744.mm.1	1,35	Cep97	JUC1600007356.mm.1	4,18	0,027767	0,505135		
TC1600001744.mm.1	1,35	Cep97	PSR1600014119.mm.1	-2,14	0,047992	0,559574	Alternative 3' Acce	0,32
TC1600001744.mm.1	1,35	Cep97	JUC1600007354.mm.1	-2,35	0,04084	0,543283		
TC0200005377.mm.1	1,02	C330013J21Rik	PSR0200045726.mm.1	4,18	0,018454	0,468671	Cassette Exon	0,28
TC0200005377.mm.1	1,02	C330013J21Rik	JUC0200023534.mm.1	2,89	0,029286	0,509874		
TC0300001173.mm.1	-1,22	Col11a1	JUC0300004991.mm.1	4,18	0,024299	0,49362		
TC0300001173.mm.1	-1,22	Col11a1	PSR0300009581.mm.1	2,34	0,008436	0,406611	Cassette Exon	0,12
TC1400002230.mm.1	-1,31	Gm17116	JUC1400009451.mm.1	4,18	0,030286	0,512627		
TC0300000747.mm.1	1,07	Pklr	JUC0300002901.mm.1	4,17	0,002572	0,349501		
TC0300000747.mm.1	1,07	Pklr	PSR0300005490.mm.1	2,02	0,000366	0,291725	Cassette Exon	0,14
TC0300000747.mm.1	1,07	Pklr	PSR0300005477.mm.1	-2,32	0,038492	0,536557	Cassette Exon	0,11
TC0700004075.mm.1	1,13	Trim66	JUC0700017655.mm.1	4,17	0,009673	0,417579		
TC0700004075.mm.1	1,13	Trim66	JUC0700017663.mm.1	-2,15	0,006873	0,394271		
TC0400003473.mm.1	-1,16	Cap1	JUC0400014661.mm.1	4,17	0,024431	0,493932		
TC0400002665.mm.1	1,22	Abca1	JUC0400011178.mm.1	4,17	0,003641	0,356757		

TC0400002665.mm.1	1,22	Abca1	JUC0400011204.mm.1	2,37	0,04565	0,554766		
TC0600003474.mm.1	-1,21	Fam60a	PSR0600027461.mm.1	4,16	0,011411	0,427582	Alternative 3' Acce	0,47
TC0300000716.mm.1	1,47	Mef2d	JUC0300002620.mm.1	4,16	0,014585	0,449876		
TC0300000716.mm.1	1,47	Mef2d	JUC0300002621.mm.1	-2,02	0,037545	0,534001		
TC0300000716.mm.1	1,47	Mef2d	JUC0300002613.mm.1	-2,34	0,017494	0,463964		
TC0100001955.mm.1	1,2	Arfgef1	JUC0100009091.mm.1	4,16	0,004408	0,366753		
TC1600000436.mm.1	1,01	Rnf168	JUC1600002079.mm.1	4,16	0,014313	0,448078		
TC1300002314.mm.1	1,05	Ahrr	JUC1300007977.mm.1	4,15	0,000041	0,245341		
TC1300002314.mm.1	1,05	Ahrr	PSR1300015204.mm.1	3,28	0,039205	0,538692	Cassette Exon	0,36
TC1300002314.mm.1	1,05	Ahrr	PSR1300015201.mm.1	2,49	0,01177	0,430069	Alternative 3' Acce	0,21
TC1300002314.mm.1	1,05	Ahrr	PSR1300015206.mm.1	2,17	0,014812	0,451034	Cassette Exon	0,26
TC1100000327.mm.1	1,22	Nsg2	JUC1100001472.mm.1	4,15	0,002494	0,349135		
TC1100000327.mm.1	1,22	Nsg2	JUC1100001471.mm.1	2,71	0,000469	0,298999		
TC1100000327.mm.1	1,22	Nsg2	JUC1100001474.mm.1	2,29	0,00505	0,374728		
TC1100000327.mm.1	1,22	Nsg2	PSR1100002771.mm.1	2,15	0,007173	0,397855	Cassette Exon	0,18
TC0900001052.mm.1	-1,29	Ddx43	JUC0900004492.mm.1	4,15	0,005938	0,385008		
TC0900001052.mm.1	-1,29	Ddx43	PSR0900008289.mm.1	-2,18	0,014718	0,45037		
TC0900001052.mm.1	-1,29	Ddx43	JUC0900004496.mm.1	-2,77	0,008531	0,407721		
TC0700000934.mm.1	1,4	A230056P14Rik; Gm1588	JUC0700004290.mm.1	4,15	0,028372	0,506881		
TC0400002013.mm.1	-1,22	Tnfrsf25	JUC0400008639.mm.1	4,15	0,005332	0,378059		
TC0900000674.mm.1	1,78	AI118078	PSR0900004852.mm.1	4,14	0,000123	0,270905	Cassette Exon	0,27
TC0900000674.mm.1	1,78	AI118078	PSR0900004846.mm.1	-3,56	0,005677	0,382164	Cassette Exon	0,24
TC0400002800.mm.1	1,63	Whrn	JUC0400012012.mm.1	4,14	0,000324	0,288663		
TC0400002800.mm.1	1,63	Whrn	JUC0400012006.mm.1	3,14	0,001591	0,331726		
TC0400002800.mm.1	1,63	Whrn	JUC0400012011.mm.1	2,23	0,009791	0,417839		
TC0400002800.mm.1	1,63	Whrn	JUC0400012014.mm.1	2,15	0,005707	0,382524		
TC0400002800.mm.1	1,63	Whrn	PSR0400023044.mm.1	2,05	0,022456	0,487003	Cassette Exon	0,16
TC0400002800.mm.1	1,63	Whrn	JUC0400012016.mm.1	-2,01	0,029772	0,511132		
TC0400002800.mm.1	1,63	Whrn	JUC0400012013.mm.1	-2,27	0,036671	0,531675		
TC0400002800.mm.1	1,63	Whrn	PSR0400023040.mm.1	-2,28	0,008607	0,40841	Cassette Exon	0,1
TC0400002800.mm.1	1,63	Whrn	JUC0400012017.mm.1	-2,28	0,047319	0,558291		
TC1100003841.mm.1	1,33	Gpatch8	JUC1100018745.mm.1	4,14	0,000891	0,313363		
TC1700000428.mm.1	1,4	Syngap1	JUC1700001994.mm.1	4,14	0,021417	0,482126		
TC1700000428.mm.1	1,4	Syngap1	JUC1700001996.mm.1	-2,19	0,001783	0,336311		
TC1700000428.mm.1	1,4	Syngap1	JUC1700001998.mm.1	-2,76	0,044469	0,551693		
TC0700004108.mm.1	-1,14	Eif4g2; Gm23262	PSR0700034062.mm.1	4,14	0,019858	0,475698		
TC1500000791.mm.1	-1,01	Prr5	JUC1500003502.mm.1	4,13	0,004435	0,366981		
TC1500000791.mm.1	-1,01	Prr5	PSR1500006231.mm.1	2,05	0,006728	0,393343	Cassette Exon	0,12
TC0100003874.mm.1	-1,29	Ankrd23	JUC0100009918.mm.1	4,13	0,043654	0,550225		
TC0800000592.mm.1	1,62	Gpm6a	JUC0800002216.mm.1	4,13	0,025259	0,496939		
TC1200002422.mm.1	2,52	Rd3l	JUC1200009273.mm.1	4,12	0,047659	0,558821		
TC1200002422.mm.1	2,52	Rd3l	JUC1200009274.mm.1	-2,99	0,02596	0,499144		
TC1200002422.mm.1	2,52	Rd3l	PSR1200016831.mm.1	-5,25	0,002097	0,342919	Intron Retention	0,75
TC0400002854.mm.1	1,2	Tle1	JUC0400012189.mm.1	4,12	0,005002	0,373851		
TC0400002854.mm.1	1,2	Tle1	PSR0400023375.mm.1	2,04	0,017932	0,466466	Cassette Exon	0,09
TC0400002854.mm.1	1,2	Tle1	PSR0400023327.mm.1	-2,04	0,027397	0,503961	Alternative 3' Acce	0,17
TC1200002195.mm.1	2,08	Eml5	JUC1200008360.mm.1	4,12	0,011499	0,428152		
TC1200002195.mm.1	2,08	Eml5	JUC1200008378.mm.1	2,56	0,000092	0,255472		

TC1200002195.mm.1	2,08	Eml5	PSR1200015160.mm.1	-2,05	0,033038	0,521501	Cassette Exon	0,11
TC1200002195.mm.1	2,08	Eml5	JUC1200008367.mm.1	-2,5	0,005198	0,377015		
TC1200002195.mm.1	2,08	Eml5	PSR1200015177.mm.1	-2,6	0,004562	0,369273	Alternative 3' Acce	0,14
TC1200002195.mm.1	2,08	Eml5	JUC1200008388.mm.1	-2,87	0,0122	0,433268		
TC1200002195.mm.1	2,08	Eml5	JUC1200008355.mm.1	-3,23	0,01328	0,441719		
TC0700004667.mm.1	1,4	Ctbp2	PSR0700036874.mm.1	4,12	0,046377	0,556329	Alternative 5' Donc	0,13
TC1700000691.mm.1	-1,37	Pou5f1	JUC1700003870.mm.1	4,11	0,02794	0,505612		
TC0600003039.mm.1	1,16	Ccdc77	JUC0600012243.mm.1	4,11	0,015253	0,452847		
TC1000001374.mm.1	2,53	Mdm1	PSR1000010259.mm.1	4,1	0,000535	0,304044	Cassette Exon	0,39
TC1000001374.mm.1	2,53	Mdm1	PSR1000010252.mm.1	3,3	0,000628	0,304044	Cassette Exon	0,24
TC1000001374.mm.1	2,53	Mdm1	JUC1000005547.mm.1	2,96	0,008251	0,405976		
TC1000001374.mm.1	2,53	Mdm1	PSR1000010248.mm.1	2,89	0,001849	0,337414	Cassette Exon	0,23
TC1000001374.mm.1	2,53	Mdm1	PSR1000010250.mm.1	2,7	0,007164	0,397855		
TC1000001374.mm.1	2,53	Mdm1	PSR1000010257.mm.1	2,33	0,008906	0,410728	Cassette Exon	0,22
TC1000001374.mm.1	2,53	Mdm1	JUC1000005546.mm.1	2,3	0,004247	0,363417		
TC1000001374.mm.1	2,53	Mdm1	PSR1000010255.mm.1	2,25	0,005821	0,383169	Cassette Exon	0,13
TC1000001374.mm.1	2,53	Mdm1	JUC1000005541.mm.1	2,24	0,006692	0,393293		
TC1000001374.mm.1	2,53	Mdm1	PSR1000010246.mm.1	-2,04	0,039201	0,538692	Cassette Exon	0,13
TC1000001374.mm.1	2,53	Mdm1	JUC1000005550.mm.1	-2,43	0,009917	0,418378		
TC1000001374.mm.1	2,53	Mdm1	PSR1000010247.mm.1	-2,67	0,006056	0,386642	Cassette Exon	0,16
TC1000001374.mm.1	2,53	Mdm1	JUC1000005556.mm.1	-2,86	0,009312	0,414883		
TC1000001374.mm.1	2,53	Mdm1	JUC1000005551.mm.1	-6,18	0,00173	0,335996		
TC1800000962.mm.1	-1,28	Ccny	PSR1800007137.mm.1	4,1	0,031315	0,516204	Alternative 5' Donc	0,32
TC1800000962.mm.1	-1,28	Ccny	PSR1800007135.mm.1	2,02	0,04961	0,563407	Alternative 5' Donc	0,31
TC0400000435.mm.1	-1,13	Rusc2	JUC0400001428.mm.1	4,1	0,042232	0,547034		
TC0100003835.mm.1	-1,48	Traf3ip3	PSR0100031358.mm.1	4,09	0,038338	0,53622	Cassette Exon	0,28
TC0100003835.mm.1	-1,48	Traf3ip3	PSR0100031362.mm.1	3,56	0,018852	0,470954	Alternative 5' Donc	0,19
TC0600002084.mm.1	1,87	Dgki	JUC0600008562.mm.1	4,09	0,016164	0,457969		
TC0600002084.mm.1	1,87	Dgki	JUC0600008560.mm.1	3,37	0,001531	0,330287		
TC0600002084.mm.1	1,87	Dgki	PSR0600016364.mm.1	-2,02	0,041831	0,545586	Cassette Exon	0,08
TC0600002084.mm.1	1,87	Dgki	JUC0600008566.mm.1	-2,14	0,034115	0,524882		
TC0600002084.mm.1	1,87	Dgki	PSR0600016381.mm.1	-2,21	0,024815	0,495292	Cassette Exon	0,19
TC0600002084.mm.1	1,87	Dgki	JUC0600008586.mm.1	-2,27	0,037199	0,533261		
TC0700000720.mm.1	1,8	Vstm2b	JUC0700002931.mm.1	4,08	0,00414	0,3623		
TC0700000720.mm.1	1,8	Vstm2b	PSR0700005888.mm.1	2,56	0,013909	0,445962	Cassette Exon	0,18
TC1400002650.mm.1	-1,55		JUC1400010916.mm.1	4,08	0,000286	0,28803		
TC1300000978.mm.1	1,77	C130071C03Rik; Mir9-2	PSR1300006309.mm.1	4,07	0,002867	0,352207	Alternative 5' Donc	0,48
TC1300000978.mm.1	1,77	C130071C03Rik; Mir9-2	JUC1300003324.mm.1	-2,84	0,036317	0,530547		
TC0200002075.mm.1	-1,1	Ankef1; BC034902	JUC0200008317.mm.1	4,07	0,009069	0,412827		
TC0200002075.mm.1	-1,1	Ankef1; BC034902	PSR0200016520.mm.1	3,39	0,004621	0,369794	Cassette Exon	0,31
TC0200002075.mm.1	-1,1	Ankef1; BC034902	JUC0200008324.mm.1	2,95	0,006513	0,391376		
TC0200002075.mm.1	-1,1	Ankef1; BC034902	PSR0200016523.mm.1	2,11	0,001131	0,317328	Cassette Exon	0,1
TC0500001270.mm.1	-1,11	Cit	JUC0500006051.mm.1	4,07	0,001098	0,317328		
TC0400003042.mm.1	1,11		JUC0400012711.mm.1	4,07	0,046574	0,556761		
TC0300000334.mm.1	2,4	Pcdh10	JUC0300001268.mm.1	4,06	0,016668	0,460057		
TC0300000334.mm.1	2,4	Pcdh10	JUC0300001270.mm.1	2,62	0,004488	0,367995		
TC0300000334.mm.1	2,4	Pcdh10	JUC0300001269.mm.1	2,23	0,000508	0,303579		
TC0300000334.mm.1	2,4	Pcdh10	PSR0300002462.mm.1	-2,01	0,009961	0,418429	Alternative 5' Donc	0,15

TC0300000334.mm.1	2,4	Pcdh10	PSR0300002453.mm.1	-3,53	0,003734	0,357586	Alternative 5' Donc	0,54
TC0300000334.mm.1	2,4	Pcdh10	JUC0300001271.mm.1	-4,65	0,003737	0,357586		
TC0700000907.mm.1	1,58	Slc6a5	JUC0700004102.mm.1	4,06	0,012459	0,435282		
TC0700000907.mm.1	1,58	Slc6a5	PSR0700008183.mm.1	3,51	0,015205	0,452738	Cassette Exon	0,24
TC0700000907.mm.1	1,58	Slc6a5	JUC0700004099.mm.1	3,27	0,041084	0,543902		
TC0500003117.mm.1	1,94	Srrm4	JUC0500015115.mm.1	4,05	0,012178	0,433072		
TC0500003117.mm.1	1,94	Srrm4	PSR0500027756.mm.1	3,15	0,021595	0,4833	Cassette Exon	0,33
TC0500003117.mm.1	1,94	Srrm4	JUC0500015119.mm.1	3,03	0,016439	0,459014		
TC0500003117.mm.1	1,94	Srrm4	PSR0500027758.mm.1	3,01	0,000657	0,304044	Alternative 3' Acce	0,25
TC0500003117.mm.1	1,94	Srrm4	PSR0500027770.mm.1	2,09	0,031379	0,516345	Cassette Exon	0,09
TC0500003117.mm.1	1,94	Srrm4	PSR0500027762.mm.1	-2,25	0,020117	0,47686	Cassette Exon	0,1
TC0500003117.mm.1	1,94	Srrm4	PSR0500027754.mm.1	-2,44	0,048024	0,55961	Alternative 5' Donc	0,15
TC0700004537.mm.1	1,3	Cend1	PSR0700038147.mm.1	4,05	0,028091	0,50608	Alternative 5' Donc	0,27
TC0200002099.mm.1	1,32	Macrod2	JUC0200008421.mm.1	4,05	0,024855	0,495511		
TC0200002099.mm.1	1,32	Macrod2	PSR0200016721.mm.1	2,7	0,003073	0,353892	Cassette Exon	0,15
TC0200002099.mm.1	1,32	Macrod2	PSR0200016720.mm.1	2,61	0,002964	0,352501	Cassette Exon	0,15
TC0200002099.mm.1	1,32	Macrod2	PSR0200016713.mm.1	2,18	0,016391	0,458754	Cassette Exon	0,09
TC0200002099.mm.1	1,32	Macrod2	JUC0200008446.mm.1	-3,36	0,005952	0,385276		
TC1800000791.mm.1	-1,18	Myo5b	JUC1800003164.mm.1	4,05	0,011933	0,431703		
TC1800000791.mm.1	-1,18	Myo5b	JUC1800003151.mm.1	3,04	0,022271	0,485954		
TC0100001744.mm.1	-1,31	1700047M11Rik	JUC0100008258.mm.1	4,05	0,013055	0,439579		
TC1500002226.mm.1	-1,16		JUC1500010411.mm.1	4,05	0,004719	0,370888		
TC0500001757.mm.1	1,04	Trrap	JUC0500008855.mm.1	4,05	0,007749	0,402774		
TC0500001757.mm.1	1,04	Trrap	JUC0500008858.mm.1	3,02	0,048155	0,560004		
TC0500001757.mm.1	1,04	Trrap	JUC0500008798.mm.1	-2,56	0,018346	0,468287		
TC1100001786.mm.1	-1	Cep95	JUC1100008798.mm.1	4,05	0,009215	0,414093		
TC0X00000963.mm.1	1,15	Dlg3	PSR0X00006302.mm.1	4,04	0,008659	0,408463	Cassette Exon	0,36
TC0X00000963.mm.1	1,15	Dlg3	JUC0X00003096.mm.1	3,18	0,009792	0,417839		
TC0X00000963.mm.1	1,15	Dlg3	JUC0X00003093.mm.1	2,05	0,042017	0,546122		
TC1800000124.mm.1	-1,08	Taf4b	PSR1800000908.mm.1	4,04	0,00088	0,312015	Cassette Exon	0,29
TC1800000124.mm.1	-1,08	Taf4b	JUC1800000532.mm.1	2,25	0,0252	0,49668		
TC1300000174.mm.1	-1,49	Hist1h3h; Hist1h3g; Hist1	PSR1300001169.mm.1	4,04	0,04289	0,548183	Cassette Exon	0,28
TC0X00002199.mm.1	-1,08	Hs6st2	JUC0X00007193.mm.1	4,04	0,001744	0,335996		
TC0X00002199.mm.1	-1,08	Hs6st2	JUC0X00007197.mm.1	2,04	0,046851	0,557261		
TC0X00002199.mm.1	-1,08	Hs6st2	PSR0X00014051.mm.1	2,02	0,012785	0,437236	Alternative 5' Donc	0,17
TC0600001335.mm.1	9,58	Cacna2d4	PSR0600010576.mm.1	4,03	0,031733	0,517489	Cassette Exon	0,32
TC0600001335.mm.1	9,58	Cacna2d4	JUC0600005498.mm.1	4,01	0,017231	0,462633		
TC0600001335.mm.1	9,58	Cacna2d4	JUC0600005496.mm.1	3,07	0,010897	0,424424		
TC0600001335.mm.1	9,58	Cacna2d4	JUC0600005503.mm.1	2,42	0,001026	0,316361		
TC0600001335.mm.1	9,58	Cacna2d4	PSR0600010593.mm.1	2,34	0,012166	0,432998	Cassette Exon	0,14
TC0600001335.mm.1	9,58	Cacna2d4	JUC0600005523.mm.1	2,31	0,004613	0,369794		
TC0600001335.mm.1	9,58	Cacna2d4	PSR0600010581.mm.1	2,1	0,003157	0,353892	Cassette Exon	0,19
TC0600001335.mm.1	9,58	Cacna2d4	PSR0600010583.mm.1	2,04	0,003272	0,354243	Cassette Exon	0,28
TC0600001335.mm.1	9,58	Cacna2d4	PSR0600010597.mm.1	-2,22	0,001428	0,327447	Cassette Exon	0,32
TC0600001335.mm.1	9,58	Cacna2d4	PSR0600010604.mm.1	-2,3	0,020966	0,479982	Cassette Exon	0,27
TC0600001335.mm.1	9,58	Cacna2d4	PSR0600010598.mm.1	-2,41	0,010192	0,419711	Cassette Exon	0,34
TC0600001335.mm.1	9,58	Cacna2d4	PSR0600010608.mm.1	-2,51	0,003479	0,355638	Cassette Exon	0,29
TC0600001335.mm.1	9,58	Cacna2d4	JUC0600005510.mm.1	-2,68	0,035541	0,528764		

TC0600001335.mm.1	9,58	Cacna2d4	PSR0600010600.mm.1	-3,21	0,001665	0,335182	Cassette Exon	0,34
TC0600001335.mm.1	9,58	Cacna2d4	JUC0600005519.mm.1	-3,27	0,001563	0,331357		
TC0600001335.mm.1	9,58	Cacna2d4	JUC0600005518.mm.1	-3,32	0,029583	0,510483		
TC0600001335.mm.1	9,58	Cacna2d4	JUC0600005515.mm.1	-3,44	0,013721	0,444758		
TC0600001335.mm.1	9,58	Cacna2d4	PSR0600010596.mm.1	-3,69	0,000585	0,304044	Cassette Exon	0,37
TC0600001335.mm.1	9,58	Cacna2d4	JUC0600005520.mm.1	-3,89	0,006374	0,389488		
TC0600001335.mm.1	9,58	Cacna2d4	PSR0600010601.mm.1	-3,93	0,0155	0,454456	Cassette Exon	0,28
TC0600001335.mm.1	9,58	Cacna2d4	JUC0600005509.mm.1	-4,25	0,023815	0,491938		
TC0600001335.mm.1	9,58	Cacna2d4	JUC0600005517.mm.1	-4,98	0,027799	0,505407		
TC0600001335.mm.1	9,58	Cacna2d4	PSR0600010610.mm.1	-5,57	0,001331	0,325349	Cassette Exon	0,33
TC0600001335.mm.1	9,58	Cacna2d4	PSR0600010599.mm.1	-6,84	0,003539	0,3562	Cassette Exon	0,55
TC0600001335.mm.1	9,58	Cacna2d4	PSR0600010571.mm.1	-7,05	0,001352	0,325427	Alternative 3' Acce	0,37
TC0600001335.mm.1	9,58	Cacna2d4	JUC0600005512.mm.1	-7,92	0,004216	0,363236		
TC0600001335.mm.1	9,58	Cacna2d4	JUC0600005511.mm.1	-8,97	0,000585	0,304044		
TC0600001335.mm.1	9,58	Cacna2d4	JUC0600005502.mm.1	-11,29	0,014773	0,450801		
TC0600001335.mm.1	9,58	Cacna2d4	JUC0600005522.mm.1	-11,89	0,000037	0,238339		
TC0600001335.mm.1	9,58	Cacna2d4	PSR0600010578.mm.1	-12,36	0,000833	0,311886	Cassette Exon	0,27
TC1700002316.mm.1	1,25	Sema6b	JUC1700011539.mm.1	4,03	0,005027	0,374176		
TC1700002316.mm.1	1,25	Sema6b	JUC1700011537.mm.1	3,17	0,030084	0,511908		
TC1700002316.mm.1	1,25	Sema6b	PSR1700021622.mm.1	-2,15	0,000136	0,270912	Cassette Exon	0,11
TC1700002316.mm.1	1,25	Sema6b	JUC1700011536.mm.1	-2,3	0,003248	0,354243		
TC1400002740.mm.1	1,02	Dock9	PSR1400020397.mm.1	4,02	0,019872	0,475704	Cassette Exon	0,33
TC1200001207.mm.1	-1,01	Ppp2r5c	PSR1200008435.mm.1	4,02	0,034181	0,525025	Cassette Exon	0,32
TC1200001207.mm.1	-1,01	Ppp2r5c	PSR1200008433.mm.1	2,27	0,029503	0,510123	Cassette Exon	0,16
TC0300001041.mm.1	-1,43	Nras	JUC0300004322.mm.1	4	0,014019	0,446496		
TC0300001041.mm.1	-1,43	Nras	JUC0300004330.mm.1	2,42	0,011104	0,426		
TC1400000104.mm.1	2,26	Synpr	JUC1400000516.mm.1	3,99	0,000999	0,316361		
TC1400000104.mm.1	2,26	Synpr	PSR1400000853.mm.1	-2,27	0,001981	0,341091	Cassette Exon	0,07
TC1400000104.mm.1	2,26	Synpr	PSR1400000857.mm.1	-2,56	0,018609	0,469665	Alternative 5' Donc	0,21
TC1400000104.mm.1	2,26	Synpr	PSR1400000852.mm.1	-2,63	0,048549	0,561008	Cassette Exon	0,09
TC1400000104.mm.1	2,26	Synpr	PSR1400000847.mm.1	-2,65	0,040591	0,542474	Cassette Exon	0,19
TC1400000104.mm.1	2,26	Synpr	JUC1400000521.mm.1	-2,69	0,008044	0,40434		
TC1400000104.mm.1	2,26	Synpr	JUC1400000515.mm.1	-2,82	0,010389	0,421341		
TC1400000104.mm.1	2,26	Synpr	PSR1400000861.mm.1	-4,72	0,000291	0,288663	Alternative 5' Donc	0,42
TC1400000104.mm.1	2,26	Synpr	PSR1400000850.mm.1	-5,56	0,009534	0,416602	Alternative 3' Acce	0,22
TC0600001176.mm.1	1,7	Cntn6	JUC0600004652.mm.1	3,99	0,002466	0,349135		
TC0600001176.mm.1	1,7	Cntn6	PSR0600009073.mm.1	2,74	0,003026	0,353502	Cassette Exon	0,14
TC0600001176.mm.1	1,7	Cntn6	PSR0600009061.mm.1	2,44	0,022517	0,487114	Cassette Exon	0,11
TC0600001176.mm.1	1,7	Cntn6	JUC0600004650.mm.1	-2,02	0,025363	0,497403		
TC0600001176.mm.1	1,7	Cntn6	JUC0600004642.mm.1	-2,5	0,042926	0,548271		
TC0600001176.mm.1	1,7	Cntn6	PSR0600009053.mm.1	-2,65	0,000356	0,290838	Alternative 3' Acce	0,24
TC0600001176.mm.1	1,7	Cntn6	JUC0600004660.mm.1	-2,78	0,009671	0,417554		
TC0500003191.mm.1	-1,13	Mapkapk5; Adam1a	JUC0500015462.mm.1	3,99	0,002691	0,349612		
TC0400000567.mm.1	-1,14	Smc2	JUC0400002056.mm.1	3,99	0,001281	0,322251		
TC0400000567.mm.1	-1,14	Smc2	JUC0400002076.mm.1	3,16	0,020457	0,478018		
TC1100000109.mm.1	2,79	Adcy1	JUC1100000587.mm.1	3,98	0,004327	0,364665		
TC1100000109.mm.1	2,79	Adcy1	JUC1100000589.mm.1	3,33	0,002029	0,341377		
TC1100000109.mm.1	2,79	Adcy1	JUC1100000588.mm.1	2,86	0,030161	0,512193		

TC1100000109.mm.1	2,79	Adcy1	JUC1100000584.mm.1	-2,17	0,014242	0,447947		
TC1100000109.mm.1	2,79	Adcy1	PSR1100001134.mm.1	-2,28	0,00228	0,345991	Cassette Exon	0,13
TC1100000109.mm.1	2,79	Adcy1	PSR1100001126.mm.1	-2,28	0,001379	0,32572	Cassette Exon	0,02
TC1100000109.mm.1	2,79	Adcy1	PSR1100001112.mm.1	-2,49	0,000261	0,28803	Cassette Exon	0,2
TC1100000109.mm.1	2,79	Adcy1	JUC1100000603.mm.1	-2,63	0,01507	0,45236		
TC1100000109.mm.1	2,79	Adcy1	PSR1100001111.mm.1	-2,7	0,003336	0,354243	Cassette Exon	0,2
TC1100000109.mm.1	2,79	Adcy1	JUC1100000600.mm.1	-2,81	0,013382	0,442371		
TC1100000109.mm.1	2,79	Adcy1	JUC1100000596.mm.1	-2,93	0,003907	0,359564		
TC1100000109.mm.1	2,79	Adcy1	PSR1100001132.mm.1	-3,08	0,040284	0,541823	Cassette Exon	0,28
TC1100000109.mm.1	2,79	Adcy1	JUC1100000595.mm.1	-3,56	0,005024	0,374162		
TC1100000109.mm.1	2,79	Adcy1	JUC1100000597.mm.1	-3,62	0,000374	0,291789		
TC1100000109.mm.1	2,79	Adcy1	JUC1100000604.mm.1	-3,8	0,007765	0,402774		
TC1100000109.mm.1	2,79	Adcy1	PSR1100001127.mm.1	-4,01	0,014263	0,447947	Alternative 3' Acce	0,46
TC0800000899.mm.1	1,34	Inpp4b	PSR0800006675.mm.1	3,98	0,01185	0,430711	Cassette Exon	0,44
TC0800000899.mm.1	1,34	Inpp4b	PSR0800006671.mm.1	3,59	0,001842	0,337414	Cassette Exon	0,29
TC0800000899.mm.1	1,34	Inpp4b	JUC0800003531.mm.1	3,5	0,009054	0,412827		
TC0800000899.mm.1	1,34	Inpp4b	JUC0800003537.mm.1	3,45	0,000784	0,309887		
TC0800000899.mm.1	1,34	Inpp4b	JUC0800003534.mm.1	2,11	0,014905	0,451803		
TC0800000899.mm.1	1,34	Inpp4b	PSR0800006674.mm.1	2,1	0,012685	0,436333	Cassette Exon	0,15
TC0800000899.mm.1	1,34	Inpp4b	PSR0800006704.mm.1	-2,26	0,007221	0,39826	Cassette Exon	0,13
TC0800000899.mm.1	1,34	Inpp4b	JUC0800003559.mm.1	-2,59	0,016326	0,458616		
TC1000001549.mm.1	1,98	Agap2	JUC1000005885.mm.1	3,98	0,002392	0,348564		
TC1000001549.mm.1	1,98	Agap2	PSR1000010981.mm.1	3,93	0,000108	0,267977	Cassette Exon	0,07
TC1000001549.mm.1	1,98	Agap2	JUC1000005889.mm.1	3,24	0,002082	0,342315		
TC1000001549.mm.1	1,98	Agap2	JUC1000005883.mm.1	3,21	0,000161	0,272566		
TC1000001549.mm.1	1,98	Agap2	PSR1000010990.mm.1	2,16	0,012207	0,433286	Cassette Exon	0,14
TC1000001549.mm.1	1,98	Agap2	PSR1000010989.mm.1	2,13	0,0154	0,453964	Cassette Exon	0,24
TC1000001549.mm.1	1,98	Agap2	PSR1000010998.mm.1	-2,02	0,008433	0,406611	Cassette Exon	0,12
TC1000001549.mm.1	1,98	Agap2	PSR1000010992.mm.1	-2,05	0,005428	0,379581	Cassette Exon	0,12
TC1000001549.mm.1	1,98	Agap2	PSR1000010999.mm.1	-2,47	0,006293	0,388979	Cassette Exon	0,12
TC1000001549.mm.1	1,98	Agap2	PSR1000010979.mm.1	-2,59	0,006595	0,39213	Cassette Exon	0,22
TC1000001549.mm.1	1,98	Agap2	JUC1000005882.mm.1	-2,91	0,000215	0,28803		
TC1000001549.mm.1	1,98	Agap2	PSR1000011000.mm.1	-2,96	0,001435	0,327493	Cassette Exon	0,18
TC1000001549.mm.1	1,98	Agap2	JUC1000005881.mm.1	-3,59	0,017455	0,463886		
TC0500002178.mm.1	-1,25	Ppm1g	JUC0500010877.mm.1	3,98	0,002478	0,349135		
TC0800002838.mm.1	1,15	Cenpt	JUC0800011945.mm.1	3,98	0,002017	0,34123		
TC0800002838.mm.1	1,15	Cenpt	JUC0800011951.mm.1	2,83	0,008909	0,410728		
TC0400003043.mm.1	1,93	Elavl2; Mir6402; mmu-mi	PSR0400024439.mm.1	3,97	0,003929	0,359818	Cassette Exon	0,3
TC0400003043.mm.1	1,93	Elavl2; Mir6402; mmu-mi	PSR0400024443.mm.1	2,89	0,000216	0,28803	Alternative 3' Acce	0,32
TC0400003043.mm.1	1,93	Elavl2; Mir6402; mmu-mi	PSR0400024436.mm.1	-2,03	0,006229	0,388088	Alternative 5' Donc	0,1
TC0400003043.mm.1	1,93	Elavl2; Mir6402; mmu-mi	PSR0400024472.mm.1	-2,58	0,022195	0,485794	Alternative 5' Donc	0,12
TC0400003043.mm.1	1,93	Elavl2; Mir6402; mmu-mi	JUC0400012727.mm.1	-2,58	0,038272	0,536002		
TC0400003043.mm.1	1,93	Elavl2; Mir6402; mmu-mi	PSR0400024464.mm.1	-2,64	0,021244	0,481413	Alternative 3' Acce	0,2
TC0400003043.mm.1	1,93	Elavl2; Mir6402; mmu-mi	PSR0400024475.mm.1	-3,01	0,031676	0,517405	Cassette Exon	0,25
TC0400003043.mm.1	1,93	Elavl2; Mir6402; mmu-mi	JUC0400012730.mm.1	-3,89	0,023038	0,489281		
TC0400003043.mm.1	1,93	Elavl2; Mir6402; mmu-mi	JUC0400012725.mm.1	-4,06	0,00362	0,356223		
TC0400003043.mm.1	1,93	Elavl2; Mir6402; mmu-mi	PSR0400024453.mm.1	-5,1	0,00515	0,37646	Cassette Exon	0,23
TC0200000591.mm.1	1,05	Sh2d3c	JUC0200002788.mm.1	3,97	0,045289	0,553726		

TC0200001049.mm.1	-1,46	Rapgef4	JUC0200004413.mm.1	3,97	0,000344	0,289576		
TC0600002644.mm.1	-1,64	Anxa4	JUC0600010640.mm.1	3,97	0,001575	0,331411		
TC0600002644.mm.1	-1,64	Anxa4	JUC0600010644.mm.1	3,93	0,007334	0,399002		
TC0400004208.mm.1	-1,16	Mup7; Gm2083; Mup1	JUC0400011673.mm.1	3,97	0,037262	0,533479		
TC1400001711.mm.1	1,2	Mapk8	JUC1400007287.mm.1	3,95	0,013186	0,440686		
TC1400001711.mm.1	1,2	Mapk8	PSR1400013336.mm.1	3,35	0,002741	0,349612	Cassette Exon	0,41
TC1400001711.mm.1	1,2	Mapk8	PSR1400013314.mm.1	2,52	0,029141	0,509333		
TC1400001711.mm.1	1,2	Mapk8	JUC1400007286.mm.1	2,51	0,02416	0,493245		
TC0200001681.mm.1	-1,02	Fmn1	PSR0200012485.mm.1	3,95	0,019033	0,471848	Cassette Exon	0,31
TC0200001681.mm.1	-1,02	Fmn1	JUC0200006214.mm.1	2,47	0,049735	0,563552		
TC0200002706.mm.1	-1,02	Tubb1	JUC0200011244.mm.1	3,95	0,001901	0,338712		
TC0900000124.mm.1	-1,51	Smco4	JUC0900000450.mm.1	3,95	0,016016	0,457051		
TC0500000947.mm.1	7,94	Prdm8	JUC0500004472.mm.1	3,94	0,007676	0,402414		
TC0500000947.mm.1	7,94	Prdm8	PSR0500008149.mm.1	2,19	0,008653	0,408463	Cassette Exon	0,23
TC0500000947.mm.1	7,94	Prdm8	PSR0500008146.mm.1	-2,09	0,006752	0,393343	Alternative 3' Acce	0,17
TC0500000947.mm.1	7,94	Prdm8	PSR0500008148.mm.1	-3,69	0,010347	0,421002	Alternative 5' Donc	0,42
TC1100002788.mm.1	4,41	Glra1	JUC1100013151.mm.1	3,94	0,043303	0,549297		
TC1100002788.mm.1	4,41	Glra1	PSR1100025313.mm.1	3,42	0,000927	0,313363	Cassette Exon	0,22
TC1100002788.mm.1	4,41	Glra1	PSR1100025315.mm.1	2,19	0,003186	0,354243		
TC1100002788.mm.1	4,41	Glra1	PSR1100025303.mm.1	-2,35	0,027643	0,504658	Cassette Exon	0,28
TC1100002788.mm.1	4,41	Glra1	PSR1100025320.mm.1	-2,46	0,003093	0,353892	Alternative 5' Donc	0,32
TC1100002788.mm.1	4,41	Glra1	PSR1100025306.mm.1	-2,71	0,005012	0,373867	Cassette Exon	0,28
TC1100002788.mm.1	4,41	Glra1	PSR1100025321.mm.1	-3,34	0,007181	0,397855	Alternative 5' Donc	0,14
TC1100002788.mm.1	4,41	Glra1	PSR1100025309.mm.1	-3,35	0,000081	0,25272	Cassette Exon	0,24
TC1100002788.mm.1	4,41	Glra1	PSR1100025305.mm.1	-4,84	0,002528	0,349501	Cassette Exon	0,28
TC1100002788.mm.1	4,41	Glra1	PSR1100025319.mm.1	-4,95	0,001778	0,336311	Alternative 5' Donc	0,32
TC1100002788.mm.1	4,41	Glra1	JUC1100013154.mm.1	-5,98	0,000291	0,288663		
TC1100002788.mm.1	4,41	Glra1	PSR1100025318.mm.1	-6,2	0,00071	0,306015	Alternative 5' Donc	0,4
TC1100002788.mm.1	4,41	Glra1	JUC1100013155.mm.1	-7,98	0,004539	0,368648		
TC1100002788.mm.1	4,41	Glra1	JUC1100013152.mm.1	-8,02	0,011069	0,425887		
TC1100002788.mm.1	4,41	Glra1	JUC1100013153.mm.1	-16,49	0,000581	0,304044		
TC0100003010.mm.1	2,3	Tmem163	JUC0100013672.mm.1	3,94	0,00064	0,304044		
TC0100003010.mm.1	2,3	Tmem163	JUC0100013671.mm.1	2,71	0,024327	0,49362		
TC0100003010.mm.1	2,3	Tmem163	PSR0100024130.mm.1	-2,02	0,011706	0,429969	Alternative 3' Acce	0,29
TC0100003010.mm.1	2,3	Tmem163	PSR0100024142.mm.1	-2,5	0,009888	0,418122	Cassette Exon	0,18
TC0100003010.mm.1	2,3	Tmem163	PSR0100024129.mm.1	-2,59	0,002668	0,349612	Cassette Exon	0,27
TC0100003010.mm.1	2,3	Tmem163	PSR0100024143.mm.1	-3,33	0,017719	0,465209	Cassette Exon	0,18
TC0100003010.mm.1	2,3	Tmem163	JUC0100013677.mm.1	-3,75	0,01574	0,455416		
TC0100003010.mm.1	2,3	Tmem163	JUC0100013669.mm.1	-3,81	0,032012	0,518371		
TC1900000386.mm.1	2,13	Apba1	JUC1900001839.mm.1	3,94	0,002894	0,352207		
TC1900000386.mm.1	2,13	Apba1	PSR1900003596.mm.1	-2,05	0,028932	0,508622	Cassette Exon	0,16
TC1900000386.mm.1	2,13	Apba1	PSR1900003600.mm.1	-2,33	0,01576	0,455437	Cassette Exon	0,12
TC1900000386.mm.1	2,13	Apba1	JUC1900001836.mm.1	-2,39	0,006633	0,392776		
TC1900000386.mm.1	2,13	Apba1	PSR1900003594.mm.1	-2,46	0,004862	0,371692	Cassette Exon	0,2
TC1700001975.mm.1	1,25	Gtf2h4	JUC1700010039.mm.1	3,94	0,035559	0,528789		
TC1700001975.mm.1	1,25	Gtf2h4	PSR1700018774.mm.1	-2,19	0,040227	0,541714	Alternative 3' Acce	0,11
TC0700004084.mm.1	1,09	Scube2	JUC0700017716.mm.1	3,94	0,007883	0,403723		
TC0700004084.mm.1	1,09	Scube2	JUC0700017724.mm.1	-2,02	0,000857	0,311909		

TC0700004084.mm.1	1,09	Scube2	JUC0700017720.mm.1	-2,11	0,005385	0,378801		
TC0700001669.mm.1	1,57	Pde3b	JUC0700007173.mm.1	3,94	0,040132	0,541573		
TC0200004920.mm.1	2,7	Dusp15	PSR0200041715.mm.1	3,93	0,005676	0,382164	Cassette Exon	0,39
TC0200004920.mm.1	2,7	Dusp15	JUC0200021535.mm.1	3,2	0,030949	0,514624		
TC0200004920.mm.1	2,7	Dusp15	PSR0200041718.mm.1	-2,19	0,039542	0,539764	Cassette Exon	0,11
TC0200004920.mm.1	2,7	Dusp15	PSR0200041708.mm.1	-3,63	0,04046	0,542132	Intron Retention	0,6
TC0200004920.mm.1	2,7	Dusp15	PSR0200041710.mm.1	-3,77	0,010121	0,41919	Alternative 5' Donc	0,42
TC0200003884.mm.1	1,23	Rtn4rl2	PSR0200033606.mm.1	3,93	0,018305	0,468049	Alternative 3' Acce	0,45
TC0400002407.mm.1	1,67	Lingo2	JUC0400010177.mm.1	3,93	0,000548	0,304044		
TC0400002407.mm.1	1,67	Lingo2	JUC0400010179.mm.1	3,59	0,007379	0,39943		
TC0400002407.mm.1	1,67	Lingo2	PSR0400019537.mm.1	3,3	0,036308	0,530487	Cassette Exon	0,23
TC0400002407.mm.1	1,67	Lingo2	JUC0400010170.mm.1	2,62	0,049834	0,563773		
TC0400002407.mm.1	1,67	Lingo2	PSR0400019533.mm.1	2,2	0,003042	0,353892	Cassette Exon	0,13
TC0400002407.mm.1	1,67	Lingo2	PSR0400019547.mm.1	-2,64	0,0391	0,538405	Alternative 5' Donc	0,24
TC0200003316.mm.1	1,38	Lrsam1	JUC0200014281.mm.1	3,93	0,002867	0,352207		
TC0200003316.mm.1	1,38	Lrsam1	JUC0200014277.mm.1	-2,19	0,00155	0,330631		
TC0200003316.mm.1	1,38	Lrsam1	PSR0200028296.mm.1	-2,23	0,006076	0,386691	Cassette Exon	0,12
TC1400001627.mm.1	1,01	Appl1	JUC1400006631.mm.1	3,93	0,031737	0,517489		
TC1400001627.mm.1	1,01	Appl1	JUC1400006621.mm.1	2,47	0,036997	0,532502		
TC0100002410.mm.1	1,03		JUC0100011186.mm.1	3,93	0,04372	0,550256		
TC0800001731.mm.1	1,04		JUC0800007630.mm.1	3,93	0,047312	0,558289		
TC1500000859.mm.1	1,03	Panx2	JUC1500003786.mm.1	3,92	0,039529	0,539764		
TC1500000859.mm.1	1,03	Panx2	PSR1500006723.mm.1	2,97	0,038951	0,537876	Cassette Exon	0,31
TC0600002965.mm.1	-1,58	Plxnd1	JUC0600011848.mm.1	3,92	0,010568	0,422432		
TC0600002965.mm.1	-1,58	Plxnd1	JUC0600011829.mm.1	3,24	0,00075	0,308185		
TC0600002965.mm.1	-1,58	Plxnd1	JUC0600011824.mm.1	2,52	0,00572	0,382767		
TC0600002965.mm.1	-1,58	Plxnd1	PSR0600022808.mm.1	2,33	0,005853	0,383741	Alternative 3' Acce	0,21
TC0600002965.mm.1	-1,58	Plxnd1	PSR0600022815.mm.1	2,28	0,04315	0,548704	Cassette Exon	0,12
TC0100002832.mm.1	-1,15	Pask	JUC0100013041.mm.1	3,92	0,016046	0,457283		
TC1800001180.mm.1	-1,41	Ecscr	JUC1800004856.mm.1	3,92	0,010089	0,418998		
TC1800001180.mm.1	-1,41	Ecscr	JUC1800004854.mm.1	3,39	0,042296	0,547224		
TC1800001180.mm.1	-1,41	Ecscr	PSR1800008707.mm.1	2,53	0,00926	0,414559		
TC1500001585.mm.1	1,43	Mtss1	JUC1500006739.mm.1	3,92	0,020601	0,478531		
TC0800001594.mm.1	-1,4	Nrp1; Mir1903	JUC0800007106.mm.1	3,92	0,037868	0,534872		
TC0300001364.mm.1	-1,21	Cenpe	JUC0300005776.mm.1	3,92	0,002676	0,349612		
TC1100003650.mm.1	-1,02	Gm11613; Gm24158	JUC1100017563.mm.1	3,92	0,048991	0,561849		
TC1800000385.mm.1	1,92	Rell2	PSR1800002920.mm.1	3,91	0,004545	0,368716	Cassette Exon	0,29
TC1800000385.mm.1	1,92	Rell2	JUC1800001621.mm.1	3,24	0,002037	0,341377		
TC1800000385.mm.1	1,92	Rell2	PSR1800002912.mm.1	2,36	0,003164	0,353901	Cassette Exon	0,23
TC1800000385.mm.1	1,92	Rell2	PSR1800002910.mm.1	-2,05	0,001565	0,331411	Alternative 3' Acce	0,12
TC1800000385.mm.1	1,92	Rell2	PSR1800002904.mm.1	-2,22	0,046778	0,557017	Cassette Exon	0,23
TC1800000385.mm.1	1,92	Rell2	JUC1800001623.mm.1	-2,33	0,011103	0,426		
TC1800000385.mm.1	1,92	Rell2	PSR1800002913.mm.1	-2,87	0,014963	0,452048	Intron Retention	0,37
TC1800000385.mm.1	1,92	Rell2	PSR1800002895.mm.1	-2,94	0,001286	0,322384	Alternative 3' Acce	0,27
TC1800000385.mm.1	1,92	Rell2	JUC1800001624.mm.1	-3,62	0,025245	0,496939		
TC1800000385.mm.1	1,92	Rell2	JUC1800001618.mm.1	-3,67	0,012352	0,434601		
TC1700000612.mm.1	-1,06	Col11a2	JUC1700003188.mm.1	3,91	0,024751	0,494905		
TC1000002646.mm.1	-1,87	Mybpc1	JUC1000010981.mm.1	3,91	0,036926	0,532327		

TC1000002646.mm.1	-1,87	Mybpc1	JUC1000010968.mm.1	-2,14	0,028663	0,507969		
TC1100001524.mm.1	1,13	Prr15l	PSR1100014019.mm.1	3,89	0,021967	0,485125	Alternative 3' Acce	0,46
TC0800002393.mm.1	3,55	Tmem59l	PSR0800018139.mm.1	3,89	0,002048	0,341391	Cassette Exon	0,29
TC0800002393.mm.1	3,55	Tmem59l	JUC0800009847.mm.1	3,01	0,009684	0,417605		
TC0800002393.mm.1	3,55	Tmem59l	JUC0800009851.mm.1	-2,1	0,039917	0,540953		
TC0800002393.mm.1	3,55	Tmem59l	PSR0800018142.mm.1	-2,67	0,017286	0,463143	Cassette Exon	0,2
TC1600000218.mm.1	-1,02	Aifm3	JUC1600000928.mm.1	3,89	0,047907	0,55942		
TC1600000218.mm.1	-1,02	Aifm3	JUC1600000925.mm.1	2,24	0,017579	0,464264		
TC0400001139.mm.1	4,19	Slc1a7	JUC0400004153.mm.1	3,88	0,000137	0,270987		
TC0400001139.mm.1	4,19	Slc1a7	JUC0400004154.mm.1	2,8	0,002147	0,343679		
TC0400001139.mm.1	4,19	Slc1a7	PSR0400008024.mm.1	2,01	0,007958	0,403853	Cassette Exon	0,11
TC0400001139.mm.1	4,19	Slc1a7	PSR0400008029.mm.1	-5,45	0,000755	0,308387	Alternative 3' Acce	0,46
TC0900001695.mm.1	2,87	Gria4; Gm23811	JUC0900008049.mm.1	3,88	0,01444	0,449008		
TC0900001695.mm.1	2,87	Gria4; Gm23811	PSR0900014327.mm.1	3,82	0,014957	0,451983	Cassette Exon	0,35
TC0900001695.mm.1	2,87	Gria4; Gm23811	JUC0900008051.mm.1	2,92	0,00497	0,373851		
TC0900001695.mm.1	2,87	Gria4; Gm23811	PSR0900014326.mm.1	2,14	0,000517	0,303623	Cassette Exon	0,26
TC0900001695.mm.1	2,87	Gria4; Gm23811	PSR0900014333.mm.1	-2,02	0,003629	0,3566	Cassette Exon	0,13
TC0900001695.mm.1	2,87	Gria4; Gm23811	PSR0900014313.mm.1	-2,08	0,005699	0,382524	Cassette Exon	0,17
TC0900001695.mm.1	2,87	Gria4; Gm23811	PSR0900014314.mm.1	-2,38	0,012394	0,434939	Cassette Exon	0,21
TC0900001695.mm.1	2,87	Gria4; Gm23811	JUC0900008055.mm.1	-2,52	0,041132	0,543971		
TC0900001695.mm.1	2,87	Gria4; Gm23811	PSR0900014316.mm.1	-2,61	0,003492	0,355638		
TC0900001695.mm.1	2,87	Gria4; Gm23811	PSR0900014334.mm.1	-2,91	0,006616	0,392269	Alternative 5' Donc	0,14
TC0900001695.mm.1	2,87	Gria4; Gm23811	JUC0900008045.mm.1	-9,38	0,035498	0,528724		
TC1400001906.mm.1	1,47	Ddhd1; Mir5131	JUC1400007923.mm.1	3,88	0,006516	0,391382		
TC1400001906.mm.1	1,47	Ddhd1; Mir5131	JUC1400007926.mm.1	2,46	0,034278	0,525322		
TC1400001906.mm.1	1,47	Ddhd1; Mir5131	PSR1400014431.mm.1	2,41	0,00055	0,304044	Cassette Exon	0,25
TC1400001906.mm.1	1,47	Ddhd1; Mir5131	PSR1400014429.mm.1	-2,12	0,004478	0,367858	Alternative 5' Donc	0,2
TC1400001906.mm.1	1,47	Ddhd1; Mir5131	JUC1400007911.mm.1	-2,26	0,010372	0,421273		
TC1400001906.mm.1	1,47	Ddhd1; Mir5131	PSR1400014444.mm.1	-3,06	0,009313	0,414883	Alternative 5' Donc	0,28
TC0300000647.mm.1	-3,09	Sfrp2	JUC0300002242.mm.1	3,88	0,020314	0,477245		
TC0300000647.mm.1	-3,09	Sfrp2	PSR0300004292.mm.1	2,79	0,011356	0,427374	Alternative 3' Acce	0,27
TC0500000642.mm.1	1,03	Limch1	JUC0500003115.mm.1	3,88	0,001143	0,317344		
TC0500000642.mm.1	1,03	Limch1	PSR0500005662.mm.1	2,07	0,033917	0,52424	Cassette Exon	0,09
TC0500000642.mm.1	1,03	Limch1	PSR0500005645.mm.1	2,04	0,010972	0,42518	Cassette Exon	0,27
TC0200002482.mm.1	1,69	Tox2	JUC0200010298.mm.1	3,88	0,039591	0,539898		
TC0200002482.mm.1	1,69	Tox2	JUC0200010301.mm.1	2,47	0,018604	0,469657		
TC0100002950.mm.1	1,19	Epb4.1l5	JUC0100013440.mm.1	3,88	0,006855	0,393968		
TC0100002950.mm.1	1,19	Epb4.1l5	JUC0100013435.mm.1	2,44	0,018722	0,470679		
TC0600001342.mm.1	-1,4	Ninj2	PSR0600010667.mm.1	3,87	0,000526	0,304044	Alternative 3' Acce	0,46
TC0600001852.mm.1	1,74	Ica1	PSR0600014623.mm.1	3,87	0,00154	0,330388	Alternative 3' Acce	0,39
TC0600001852.mm.1	1,74	Ica1	JUC0600007648.mm.1	2,18	0,020663	0,478862		
TC0600001852.mm.1	1,74	Ica1	PSR0600014619.mm.1	-2,01	0,025565	0,497808	Cassette Exon	0,11
TC0600001852.mm.1	1,74	Ica1	PSR0600014603.mm.1	-2,13	0,029872	0,511209	Cassette Exon	0,1
TC0600001852.mm.1	1,74	Ica1	JUC0600007658.mm.1	-2,86	0,040181	0,541655		
TC0600001852.mm.1	1,74	Ica1	JUC0600007664.mm.1	-3,46	0,001721	0,335996		
TC0600001852.mm.1	1,74	Ica1	JUC0600007657.mm.1	-3,62	0,021872	0,484657		
TC0600001852.mm.1	1,74	Ica1	JUC0600007660.mm.1	-6,25	0,001151	0,317344		
TC0900001739.mm.1	1,17	Cntn5	PSR0900014679.mm.1	3,87	0,00386	0,358861	Cassette Exon	0,27

TC0900001739.mm.1	1,17	Cntn5	PSR0900014680.mm.1	3,01	0,027676	0,504734	Cassette Exon	0,23
TC0900001739.mm.1	1,17	Cntn5	PSR0900014682.mm.1	2,89	0,046789	0,557037	Cassette Exon	0,18
TC0900001739.mm.1	1,17	Cntn5	PSR0900014677.mm.1	2,76	0,004284	0,363973	Cassette Exon	0,29
TC0900001739.mm.1	1,17	Cntn5	PSR0900014676.mm.1	2,5	0,003675	0,357266	Cassette Exon	0,21
TC0900001739.mm.1	1,17	Cntn5	JUC0900008265.mm.1	-2,1	0,045037	0,553049		
TC0900001739.mm.1	1,17	Cntn5	JUC0900008268.mm.1	-2,77	0,019611	0,474765		
TC0900001739.mm.1	1,17	Cntn5	JUC0900008272.mm.1	-3,97	0,00285	0,352207		
TC1800001402.mm.1	-2,13	Synpo	JUC1800005660.mm.1	3,87	0,049548	0,563325		
TC0100001734.mm.1	1,13	Cnih3	JUC0100008150.mm.1	3,87	0,010182	0,419711		
TC0500002687.mm.1	-1,16	Tmprss11e	JUC0500012919.mm.1	3,87	0,034237	0,525168		
TC0900000567.mm.1	-1,25		JUC0900002076.mm.1	3,87	0,010741	0,423772		
TC0600000839.mm.1	-1,18	Polr1a	JUC0600003229.mm.1	3,87	0,017506	0,463964		
TC0700001521.mm.1	-1,18	Gm15133	JUC0700006651.mm.1	3,87	0,01404	0,44654		
TC0900001879.mm.1	-1,16	Yipf2	JUC0900008896.mm.1	3,86	0,001211	0,322251		
TC0900001879.mm.1	-1,16	Yipf2	JUC0900008895.mm.1	3,16	0,023991	0,492783		
TC0900001879.mm.1	-1,16	Yipf2	PSR0900015780.mm.1	2,19	0,013847	0,445385	Cassette Exon	0,2
TC0400003650.mm.1	1,42	Ptpru	JUC0400015552.mm.1	3,86	0,014715	0,45037		
TC0400003650.mm.1	1,42	Ptpru	JUC0400015556.mm.1	2,61	0,015734	0,455416		
TC0400003650.mm.1	1,42	Ptpru	JUC0400015547.mm.1	2,14	0,004726	0,370888		
TC0400003650.mm.1	1,42	Ptpru	JUC0400015523.mm.1	2,06	0,003099	0,353892		
TC0400003650.mm.1	1,42	Ptpru	PSR0400029818.mm.1	-2,13	0,012746	0,436784	Cassette Exon	0,11
TC0400003650.mm.1	1,42	Ptpru	PSR0400029839.mm.1	-2,16	0,048531	0,560991	Alternative 5' Donc	0,14
TC0400003650.mm.1	1,42	Ptpru	PSR0400029810.mm.1	-2,28	0,018117	0,467161	Alternative 3' Acce	0,16
TC0400003650.mm.1	1,42	Ptpru	JUC0400015555.mm.1	-2,73	0,022587	0,487325		
TC0400003650.mm.1	1,42	Ptpru	JUC0400015544.mm.1	-3,15	0,034177	0,524992		
TC0800000814.mm.1	-1,49	Tpm4	JUC0800003161.mm.1	3,86	0,007814	0,403089		
TC0900003172.mm.1	1	Cnot10	JUC0900015110.mm.1	3,86	0,003882	0,359196		
TC0900003172.mm.1	1	Cnot10	JUC0900015106.mm.1	2,13	0,020446	0,478018		
TC0900003172.mm.1	1	Cnot10	JUC0900015107.mm.1	2,08	0,039271	0,538976		
TC0400003881.mm.1	-1,08	Clcnkb	JUC0400016730.mm.1	3,86	0,00476	0,371039		
TC1000001124.mm.1	-1,12	Cdk17	JUC1000004549.mm.1	3,86	0,002156	0,344024		
TC1000001124.mm.1	-1,12	Cdk17	JUC1000004541.mm.1	2,15	0,020068	0,476558		
TC0700000866.mm.1	-1,65		PSR0700007659.mm.1	3,85	0,022887	0,488524	Cassette Exon	0,28
TC1100000908.mm.1	2,55	Kcnab3	PSR1100007699.mm.1	3,85	0,000119	0,269605	Cassette Exon	0,26
TC1100000908.mm.1	2,55	Kcnab3	JUC1100004053.mm.1	-2,42	0,0022	0,345178		
TC1100000908.mm.1	2,55	Kcnab3	PSR1100007685.mm.1	-2,67	0,019731	0,474922	Cassette Exon	0,15
TC1100000908.mm.1	2,55	Kcnab3	PSR1100007692.mm.1	-2,75	0,024805	0,495221	Alternative 3' Acce	0,25
TC1100000908.mm.1	2,55	Kcnab3	PSR1100007686.mm.1	-2,79	0,02604	0,49952	Cassette Exon	0,16
TC0600000044.mm.1	2,22	Tac1	JUC0600000236.mm.1	3,85	0,008887	0,410717		
TC0600000044.mm.1	2,22	Tac1	PSR0600000414.mm.1	2,21	0,007018	0,395834	Cassette Exon	0,24
TC0600000044.mm.1	2,22	Tac1	PSR0600000404.mm.1	-2,01	0,033856	0,524049	Cassette Exon	0,1
TC0600000044.mm.1	2,22	Tac1	PSR0600000422.mm.1	-2,08	0,039972	0,540973	Cassette Exon	0,12
TC0600000044.mm.1	2,22	Tac1	PSR0600000407.mm.1	-2,14	0,039453	0,539705	Cassette Exon	0,11
TC0600000044.mm.1	2,22	Tac1	PSR0600000409.mm.1	-2,43	0,022365	0,48639	Cassette Exon	0,06
TC0600000044.mm.1	2,22	Tac1	PSR0600000423.mm.1	-2,53	0,022636	0,487464	Cassette Exon	0,12
TC0600000044.mm.1	2,22	Tac1	JUC0600000242.mm.1	-4,26	0,013964	0,446239		
TC1600001978.mm.1	-1,15	LOC102635020	JUC1600007905.mm.1	3,85	0,00062	0,304044		
TC1400001078.mm.1	2,07	Enox1	JUC1400004654.mm.1	3,84	0,008187	0,40568		

TC1400001078.mm.1	2,07	Enox1	JUC1400004644.mm.1	3,63	0,021175	0,480978		
TC1400001078.mm.1	2,07	Enox1	PSR1400008690.mm.1	3,34	0,02978	0,511132	Cassette Exon	0,39
TC1400001078.mm.1	2,07	Enox1	JUC1400004652.mm.1	3,13	0,008987	0,411965		
TC1400001078.mm.1	2,07	Enox1	PSR1400008691.mm.1	2,71	0,003254	0,354243	Cassette Exon	0,29
TC1400001078.mm.1	2,07	Enox1	PSR1400008673.mm.1	-2,14	0,043053	0,54841	Cassette Exon	0,08
TC1400001078.mm.1	2,07	Enox1	PSR1400008666.mm.1	-2,26	0,033037	0,521501	Cassette Exon	0,12
TC1400001078.mm.1	2,07	Enox1	PSR1400008665.mm.1	-2,39	0,003007	0,353447	Cassette Exon	0,13
TC1400001078.mm.1	2,07	Enox1	JUC1400004637.mm.1	-2,59	0,049797	0,563698		
TC1400001078.mm.1	2,07	Enox1	JUC1400004656.mm.1	-2,72	0,000221	0,28803		
TC1400001078.mm.1	2,07	Enox1	JUC1400004655.mm.1	-2,77	0,002689	0,349612		
TC0100001688.mm.1	1,21	Efcab2	JUC0100007919.mm.1	3,84	0,002473	0,349135		
TC1900000859.mm.1	-1,77	E330013P04Rik	JUC1900004072.mm.1	3,84	0,02626	0,500386		
TC1900000859.mm.1	-1,77	E330013P04Rik	PSR1900007412.mm.1	2,04	0,046521	0,556739		
TC1100001272.mm.1	1,1	Ddx52	JUC1100006245.mm.1	3,84	0,002697	0,349612		
TC1100000937.mm.1	5,82	Dlg4	JUC1100004261.mm.1	3,83	0,019576	0,474672		
TC1100000937.mm.1	5,82	Dlg4	JUC1100004257.mm.1	2,7	0,002003	0,341091		
TC1100000937.mm.1	5,82	Dlg4	JUC1100004262.mm.1	2,65	0,012774	0,437233		
TC1100000937.mm.1	5,82	Dlg4	PSR1100008160.mm.1	2,18	0,022551	0,487281	Cassette Exon	0,22
TC1100000937.mm.1	5,82	Dlg4	JUC1100004267.mm.1	-2,38	0,043115	0,54862		
TC1100000937.mm.1	5,82	Dlg4	JUC1100004269.mm.1	-2,76	0,008418	0,406611		
TC1100000937.mm.1	5,82	Dlg4	PSR1100008136.mm.1	-2,95	0,010912	0,424587		
TC1100000937.mm.1	5,82	Dlg4	PSR1100008133.mm.1	-2,98	0,02974	0,511108	Cassette Exon	0,2
TC1100000937.mm.1	5,82	Dlg4	PSR1100008126.mm.1	-3,23	0,026285	0,50053	Cassette Exon	0,29
TC1100000937.mm.1	5,82	Dlg4	PSR1100008134.mm.1	-4,22	0,001435	0,327493		
TC1100000937.mm.1	5,82	Dlg4	JUC1100004270.mm.1	-4,3	0,029473	0,510123		
TC1100000937.mm.1	5,82	Dlg4	JUC1100004247.mm.1	-4,32	0,011793	0,430163		
TC1100000937.mm.1	5,82	Dlg4	JUC1100004272.mm.1	-4,35	0,007489	0,400686		
TC1100000937.mm.1	5,82	Dlg4	JUC1100004265.mm.1	-4,54	0,001696	0,335395		
TC1100000937.mm.1	5,82	Dlg4	PSR1100008142.mm.1	-4,7	0,030607	0,51348	Cassette Exon	0,34
TC1100000937.mm.1	5,82	Dlg4	PSR1100008158.mm.1	-4,87	0,002098	0,342919	Cassette Exon	0,31
TC1100000937.mm.1	5,82	Dlg4	PSR1100008167.mm.1	-4,99	0,002968	0,352501	Alternative 3' Acce	0,46
TC1100000937.mm.1	5,82	Dlg4	PSR1100008135.mm.1	-5,97	0,002984	0,352501	Cassette Exon	0,41
TC1100000937.mm.1	5,82	Dlg4	PSR1100008130.mm.1	-6,09	0,006179	0,387873	Cassette Exon	0,3
TC1100000937.mm.1	5,82	Dlg4	PSR1100008138.mm.1	-6,51	0,003718	0,357586	Cassette Exon	0,41
TC1100000937.mm.1	5,82	Dlg4	PSR1100008123.mm.1	-6,99	0,000899	0,313363	Cassette Exon	0,41
TC1100000937.mm.1	5,82	Dlg4	PSR1100008129.mm.1	-8,45	0,003053	0,353892	Cassette Exon	0,3
TC1100000937.mm.1	5,82	Dlg4	PSR1100008146.mm.1	-8,68	0,003683	0,357266	Intron Retention	0,73
TC1100000937.mm.1	5,82	Dlg4	JUC1100004273.mm.1	-9,36	0,004832	0,371555		
TC1100000937.mm.1	5,82	Dlg4	PSR1100008132.mm.1	-10,65	0,000896	0,313363	Cassette Exon	0,38
TC1100000937.mm.1	5,82	Dlg4	JUC1100004274.mm.1	-12,28	0,005362	0,378627		
TC0700000800.mm.1	1,73	Shank1	JUC0700003262.mm.1	3,83	0,037221	0,533311		
TC0700000800.mm.1	1,73	Shank1	PSR0700006533.mm.1	-2,14	0,026526	0,501411	Cassette Exon	0,12
TC0700000800.mm.1	1,73	Shank1	JUC0700003259.mm.1	-2,32	0,045843	0,555021		
TC0700000800.mm.1	1,73	Shank1	PSR0700006542.mm.1	-2,34	0,041789	0,545586	Cassette Exon	0,17
TC0700000800.mm.1	1,73	Shank1	PSR0700006530.mm.1	-2,45	0,011653	0,429904	Cassette Exon	0,13
TC0700000800.mm.1	1,73	Shank1	PSR0700006548.mm.1	-2,84	0,027374	0,503961	Cassette Exon	0,13
TC0700000800.mm.1	1,73	Shank1	PSR0700006565.mm.1	-3,42	0,0043	0,364027	Cassette Exon	0,24
TC1300002498.mm.1	1,01	Arhgef28	JUC1300008866.mm.1	3,83	0,042545	0,547918		

TC1300002498.mm.1	1,01	Arhgef28	JUC1300008883.mm.1	3,13	0,024531	0,493992		
TC1300002498.mm.1	1,01	Arhgef28	JUC1300008898.mm.1	3,06	0,005976	0,386059		
TC1300002498.mm.1	1,01	Arhgef28	JUC1300008892.mm.1	2,73	0,011208	0,426683		
TC1100001176.mm.1	1,49	Nf1	JUC1100005753.mm.1	3,83	0,007885	0,403754		
TC1100001176.mm.1	1,49	Nf1	JUC1100005775.mm.1	3,76	0,008064	0,40448		
TC1100003455.mm.1	1,13	Cltc	JUC1100016614.mm.1	3,83	0,043583	0,550052		
TC1100003455.mm.1	1,13	Cltc	JUC1100016613.mm.1	3,8	0,02483	0,495379		
TC1200000887.mm.1	-1,27	Gm2042	PSR1200006477.mm.1	3,82	0,002898	0,352207	Alternative 3' Acce	0,45
TC0500000021.mm.1	1,17	Akap9	PSR0500000269.mm.1	3,82	0,03374	0,523558	Alternative 5' Donc	0,41
TC0500000021.mm.1	1,17	Akap9	JUC0500000118.mm.1	2,33	0,018653	0,470078		
TC0500000021.mm.1	1,17	Akap9	JUC0500000113.mm.1	-2,11	0,028069	0,505989		
TC0500000021.mm.1	1,17	Akap9	PSR0500000220.mm.1	-3,06	0,024336	0,49362	Alternative 3' Acce	0,17
TC0500001860.mm.1	1,15	Fry	JUC0500009352.mm.1	3,82	0,035789	0,529153		
TC0500001860.mm.1	1,15	Fry	JUC0500009309.mm.1	2,07	0,012938	0,438537		
TC0500001860.mm.1	1,15	Fry	PSR0500017182.mm.1	-2,15	0,008247	0,405976	Alternative 5' Donc	0,1
TC0900000465.mm.1	-1,98	3110039I08Rik	JUC0900001580.mm.1	3,82	0,012154	0,432923		
TC0900000465.mm.1	-1,98	3110039I08Rik	PSR0900003096.mm.1	2,01	0,001788	0,336311	Cassette Exon	0,09
TC1400002764.mm.1	1,72	Fgf14	JUC1400011420.mm.1	3,82	0,001281	0,322251		
TC1400002764.mm.1	1,72	Fgf14	JUC1400011425.mm.1	-3,62	0,008679	0,408815		
TC1400002764.mm.1	1,72	Fgf14	JUC1400011423.mm.1	-4,94	0,007312	0,398848		
TC0700000864.mm.1	1,71	Lmtk3	PSR0700007627.mm.1	3,81	0,00341	0,355242	Cassette Exon	0,32
TC0700000864.mm.1	1,71	Lmtk3	JUC0700003763.mm.1	2,37	0,001522	0,330287		
TC0700000864.mm.1	1,71	Lmtk3	JUC0700003765.mm.1	2,02	0,009461	0,416514		
TC0700000864.mm.1	1,71	Lmtk3	PSR0700007648.mm.1	-2,18	0,025943	0,499144	Cassette Exon	0,12
TC0700000864.mm.1	1,71	Lmtk3	PSR0700007613.mm.1	-2,23	0,004166	0,3623	Alternative 3' Acce	0,1
TC0700000864.mm.1	1,71	Lmtk3	JUC0700003780.mm.1	-4,23	0,001524	0,330287		
TC0400003870.mm.1	1,47	Crocc	JUC0400016658.mm.1	3,81	0,015608	0,455078		
TC0400003870.mm.1	1,47	Crocc	JUC0400016655.mm.1	2,61	0,015884	0,456526		
TC0400003870.mm.1	1,47	Crocc	PSR0400031980.mm.1	2,45	0,005802	0,383104	Cassette Exon	0,14
TC0400003870.mm.1	1,47	Crocc	PSR0400031955.mm.1	-2,01	0,021408	0,482126	Intron Retention	0,25
TC0400003870.mm.1	1,47	Crocc	JUC0400016648.mm.1	-2,1	0,009248	0,414515		
TC0400003870.mm.1	1,47	Crocc	PSR0400031966.mm.1	-2,39	0,00103	0,316361	Cassette Exon	0,15
TC0400003870.mm.1	1,47	Crocc	JUC0400016638.mm.1	-2,77	0,00384	0,358714		
TC0400003870.mm.1	1,47	Crocc	JUC0400016654.mm.1	-2,97	0,021205	0,48113		
TC0900002919.mm.1	-1,02	4930422M22Rik	JUC0900013423.mm.1	3,81	0,007081	0,396891		
TC1200000899.mm.1	4,63	Nrxn3	JUC1200003684.mm.1	3,8	0,000882	0,312293		
TC1200000899.mm.1	4,63	Nrxn3	PSR1200006571.mm.1	3,79	0,005309	0,378059	Cassette Exon	0,38
TC1200000899.mm.1	4,63	Nrxn3	JUC1200003681.mm.1	3,67	0,018397	0,468484		
TC1200000899.mm.1	4,63	Nrxn3	JUC1200003670.mm.1	3,6	0,002831	0,352207		
TC1200000899.mm.1	4,63	Nrxn3	JUC1200003687.mm.1	3,5	0,004513	0,368648		
TC1200000899.mm.1	4,63	Nrxn3	JUC1200003675.mm.1	3,46	0,009747	0,417839		
TC1200000899.mm.1	4,63	Nrxn3	PSR1200006572.mm.1	3,37	0,000448	0,297771	Mutually Exclusive	0,54
TC1200000899.mm.1	4,63	Nrxn3	JUC1200003686.mm.1	3,33	0,002399	0,348564		
TC1200000899.mm.1	4,63	Nrxn3	JUC1200003685.mm.1	2,73	0,041007	0,543644		
TC1200000899.mm.1	4,63	Nrxn3	PSR1200006591.mm.1	2,51	0,00946	0,416514		
TC1200000899.mm.1	4,63	Nrxn3	PSR1200006559.mm.1	2,05	0,011621	0,429746	Alternative 3' Acce	0,13
TC1200000899.mm.1	4,63	Nrxn3	PSR1200006557.mm.1	-2,01	0,020753	0,479248	Cassette Exon	0,25
TC1200000899.mm.1	4,63	Nrxn3	PSR1200006545.mm.1	-2,36	0,014167	0,447466		

TC1200000899.mm.1	4,63	Nrxn3	PSR1200006584.mm.1	-2,79	0,003936	0,359818	Cassette Exon	0,3
TC1200000899.mm.1	4,63	Nrxn3	PSR1200006581.mm.1	-2,89	0,001342	0,325349	Cassette Exon	0,27
TC1200000899.mm.1	4,63	Nrxn3	JUC1200003704.mm.1	-3,01	0,021157	0,480912		
TC1200000899.mm.1	4,63	Nrxn3	PSR1200006574.mm.1	-3,99	0,000384	0,293869	Mutually Exclusive	0,54
TC1200000899.mm.1	4,63	Nrxn3	PSR1200006542.mm.1	-4,5	0,000855	0,311909	Cassette Exon	0,45
TC1200000899.mm.1	4,63	Nrxn3	PSR1200006552.mm.1	-4,89	0,000318	0,288663	Alternative 5' Donc	0,54
TC1200000899.mm.1	4,63	Nrxn3	JUC1200003699.mm.1	-5,08	0,010037	0,41878		
TC1200000899.mm.1	4,63	Nrxn3	PSR1200006553.mm.1	-5,56	0,000503	0,303022	Cassette Exon	0,41
TC1200000899.mm.1	4,63	Nrxn3	JUC1200003689.mm.1	-6,1	0,001927	0,34017		
TC1200000899.mm.1	4,63	Nrxn3	PSR1200006563.mm.1	-6,43	0,015102	0,452453	Alternative 5' Donc	0,35
TC1200000899.mm.1	4,63	Nrxn3	PSR1200006556.mm.1	-7,83	0,003769	0,357586	Cassette Exon	0,55
TC1200000899.mm.1	4,63	Nrxn3	PSR1200006589.mm.1	-8,96	0,0002	0,28803		
TC1200000899.mm.1	4,63	Nrxn3	PSR1200006555.mm.1	-9,06	0,001227	0,322251	Cassette Exon	0,41
TC1200000899.mm.1	4,63	Nrxn3	JUC1200003701.mm.1	-10,22	0,008956	0,411476		
TC1200000899.mm.1	4,63	Nrxn3	JUC1200003697.mm.1	-10,32	0,000757	0,308387		
TC1200000899.mm.1	4,63	Nrxn3	JUC1200003706.mm.1	-10,43	0,001562	0,331357		
TC1200000899.mm.1	4,63	Nrxn3	PSR1200006540.mm.1	-11,21	0,000251	0,28803	Alternative 3' Acce	0,48
TC1200000899.mm.1	4,63	Nrxn3	JUC1200003702.mm.1	-11,35	0,013416	0,442624		
TC1200000899.mm.1	4,63	Nrxn3	JUC1200003676.mm.1	-11,37	0,007815	0,403089		
TC1200000899.mm.1	4,63	Nrxn3	JUC1200003698.mm.1	-14,2	0,001293	0,32263		
TC1200000899.mm.1	4,63	Nrxn3	JUC1200003703.mm.1	-14,51	0,001783	0,336311		
TC0300002025.mm.1	-1,35	Wwtr1	PSR0300015346.mm.1	3,8	0,002169	0,344453	Alternative 5' Donc	0,45
TC1700000092.mm.1	2,27	Pde10a	JUC1700000428.mm.1	3,8	0,036473	0,531124		
TC1700000092.mm.1	2,27	Pde10a	JUC1700000444.mm.1	3,55	0,006853	0,393893		
TC1700000092.mm.1	2,27	Pde10a	PSR1700000794.mm.1	2,41	0,012113	0,432704	Cassette Exon	0,08
TC1700000092.mm.1	2,27	Pde10a	PSR1700000781.mm.1	-2,36	0,021351	0,481836	Cassette Exon	0,05
TC1700000092.mm.1	2,27	Pde10a	PSR1700000772.mm.1	-2,5	0,001888	0,338712	Cassette Exon	0,21
TC1700000092.mm.1	2,27	Pde10a	JUC1700000435.mm.1	-2,63	0,015435	0,454229		
TC1700000092.mm.1	2,27	Pde10a	JUC1700000426.mm.1	-2,69	0,012468	0,435315		
TC1700000092.mm.1	2,27	Pde10a	JUC1700000443.mm.1	-2,99	0,001681	0,335182		
TC1700000092.mm.1	2,27	Pde10a	PSR1700000796.mm.1	-3,12	0,000133	0,270905	Cassette Exon	0,31
TC1700000092.mm.1	2,27	Pde10a	JUC1700000419.mm.1	-3,17	0,039817	0,540852		
TC1700000092.mm.1	2,27	Pde10a	JUC1700000452.mm.1	-3,43	0,033697	0,523549		
TC1700000092.mm.1	2,27	Pde10a	JUC1700000447.mm.1	-4,01	0,003779	0,357586		
TC1700000092.mm.1	2,27	Pde10a	JUC1700000423.mm.1	-4,57	0,002583	0,349501		
TC0200002792.mm.1	2,11	Slco4a1	JUC0200011578.mm.1	3,8	0,026211	0,500076		
TC0200002792.mm.1	2,11	Slco4a1	JUC0200011564.mm.1	-2,02	0,04021	0,541714		
TC0200002792.mm.1	2,11	Slco4a1	PSR0200022817.mm.1	-2,1	0,00936	0,415092	Cassette Exon	0,09
TC0200002792.mm.1	2,11	Slco4a1	JUC0200011568.mm.1	-2,3	0,015459	0,454312		
TC0200002792.mm.1	2,11	Slco4a1	PSR0200022828.mm.1	-2,35	0,003344	0,354243	Alternative 5' Donc	0,15
TC0200002792.mm.1	2,11	Slco4a1	JUC0200011576.mm.1	-2,4	0,027106	0,503022		
TC0200002792.mm.1	2,11	Slco4a1	PSR0200022820.mm.1	-2,43	0,003368	0,354278	Alternative 3' Acce	0,23
TC0200002792.mm.1	2,11	Slco4a1	JUC0200011577.mm.1	-3,36	0,002612	0,349501		
TC1000001561.mm.1	-1,06	R3hdm2	JUC1000006009.mm.1	3,8	0,000297	0,288663		
TC1000001561.mm.1	-1,06	R3hdm2	PSR1000011139.mm.1	2,5	0,009389	0,415556	Cassette Exon	0,1
TC1000001561.mm.1	-1,06	R3hdm2	JUC1000006011.mm.1	2,43	0,032632	0,520338		
TC1000001561.mm.1	-1,06	R3hdm2	JUC1000006015.mm.1	2,24	0,013936	0,446202		
TC1000001561.mm.1	-1,06	R3hdm2	JUC1000006002.mm.1	2,2	0,002955	0,352501		

TC0X00000951.mm.1	-1,69	Igbp1	JUC0X00003003.mm.1	3,8	0,023325	0,490065		
TC0300000202.mm.1	7,37	Samd7	JUC0300000529.mm.1	3,79	0,003214	0,354243		
TC0300000202.mm.1	7,37	Samd7	JUC0300000533.mm.1	2,92	0,017987	0,466929		
TC0300000202.mm.1	7,37	Samd7	PSR0300001088.mm.1	2,06	0,007022	0,395932	Cassette Exon	0,22
TC0300000202.mm.1	7,37	Samd7	JUC0300000534.mm.1	2,01	0,033023	0,521478		
TC0300000202.mm.1	7,37	Samd7	PSR0300001086.mm.1	-7,44	0,0135	0,443238	Cassette Exon	0,35
TC0300000202.mm.1	7,37	Samd7	JUC0300000530.mm.1	-12,36	0,001551	0,330671		
TC0300001795.mm.1	1,73	Slc7a14	PSR0300013907.mm.1	3,79	0,001934	0,340349	Cassette Exon	0,22
TC0300001795.mm.1	1,73	Slc7a14	JUC0300007329.mm.1	-2,36	0,018701	0,470452		
TC0300001795.mm.1	1,73	Slc7a14	PSR0300013899.mm.1	-2,64	0,017738	0,465238	Alternative 3' Acce	0,26
TC0300001795.mm.1	1,73	Slc7a14	PSR0300013910.mm.1	-2,98	0,021864	0,484635	Cassette Exon	0,18
TC0400000309.mm.1	1,05	Rars2	PSR0400001971.mm.1	3,79	0,000677	0,304338	Cassette Exon	0,26
TC1800000897.mm.1	2,29	Neto1	PSR1800006488.mm.1	3,79	0,002046	0,341377	Cassette Exon	0,15
TC1800000897.mm.1	2,29	Neto1	PSR1800006490.mm.1	2,29	0,001734	0,335996	Alternative 5' Donc	0,12
TC1800000897.mm.1	2,29	Neto1	PSR1800006478.mm.1	-2,24	0,017655	0,464608	Alternative 3' Acce	0,14
TC1800000897.mm.1	2,29	Neto1	JUC1800003584.mm.1	-3,21	0,019379	0,473624		
TC1200002586.mm.1	4	Ighv14-4	PSR1200017871.mm.1	3,78	0,017894	0,466466	Cassette Exon	0,2
TC1200002586.mm.1	4	Ighv14-4	PSR1200018887.mm.1	-3,78	0,017894	0,466466	Cassette Exon	0,31
TC0500002933.mm.1	1,3	Evi5	PSR0500025940.mm.1	3,78	0,023568	0,490947	Cassette Exon	0,17
TC0500002933.mm.1	1,3	Evi5	JUC0500014137.mm.1	2,96	0,021342	0,481754		
TC0500002933.mm.1	1,3	Evi5	JUC0500014145.mm.1	2,91	0,045081	0,55315		
TC1100003819.mm.1	1,24	Mpp3	PSR1100035399.mm.1	3,78	0,008232	0,405976		
TC1100003819.mm.1	1,24	Mpp3	JUC1100018499.mm.1	-2,45	0,003442	0,3554		
TC1900000550.mm.1	-1,18		JUC1900002619.mm.1	3,78	0,00165	0,334069		
TC1900000100.mm.1	1,09	Gm550	JUC1900000575.mm.1	3,78	0,032633	0,520338		
TC0500003099.mm.1	1,16		PSR0500027598.mm.1	3,77	0,001653	0,334268	Alternative 5' Donc	0,26
TC0500003099.mm.1	1,16		PSR0500027597.mm.1	2,18	0,029509	0,510139		
TC0300002320.mm.1	-1,21	Gm20652	JUC0300009264.mm.1	3,77	0,038872	0,537834		
TC0400001627.mm.1	1,35	Ubxn11	JUC0400006704.mm.1	3,77	0,04026	0,541822		
TC0400001627.mm.1	1,35	Ubxn11	JUC0400006716.mm.1	-3,23	0,017068	0,46205		
TC0400001627.mm.1	1,35	Ubxn11	JUC0400006698.mm.1	-3,27	0,013322	0,441836		
TC0400002189.mm.1	-1,12	Asph	JUC0400009546.mm.1	3,77	0,035373	0,528536		
TC0400002189.mm.1	-1,12	Asph	JUC0400009522.mm.1	3,19	0,02295	0,488667		
TC0400002189.mm.1	-1,12	Asph	JUC0400009540.mm.1	2,23	0,005307	0,378059		
TC0400002189.mm.1	-1,12	Asph	JUC0400009544.mm.1	-3,12	0,006257	0,388622		
TC0700004538.mm.1	3,87	Slc25a22	PSR0700038172.mm.1	3,76	0,005842	0,383458		
TC0700004538.mm.1	3,87	Slc25a22	PSR0700038202.mm.1	-2,02	0,027315	0,503732		
TC0700004538.mm.1	3,87	Slc25a22	JUC0700020135.mm.1	-2,18	0,032594	0,520272		
TC0700004538.mm.1	3,87	Slc25a22	JUC0700020132.mm.1	-2,23	0,043945	0,550715		
TC0700004538.mm.1	3,87	Slc25a22	PSR0700038161.mm.1	-2,55	0,047656	0,558801	Cassette Exon	0,21
TC0700004538.mm.1	3,87	Slc25a22	JUC0700020136.mm.1	-2,59	0,035494	0,528724		
TC0700004538.mm.1	3,87	Slc25a22	PSR0700038201.mm.1	-2,64	0,011963	0,431914		
TC0700004538.mm.1	3,87	Slc25a22	JUC0700020129.mm.1	-2,66	0,034763	0,52661		
TC0700004538.mm.1	3,87	Slc25a22	PSR0700038186.mm.1	-2,7	0,013024	0,439354	Intron Retention	0,45
TC0700004538.mm.1	3,87	Slc25a22	PSR0700038196.mm.1	-2,71	0,003471	0,355628	Intron Retention	0,39
TC0700004538.mm.1	3,87	Slc25a22	PSR0700038203.mm.1	-2,79	0,004823	0,371408		
TC0700004538.mm.1	3,87	Slc25a22	PSR0700038163.mm.1	-2,8	0,016355	0,458693	Alternative 3' Acce	0,16
TC0700004538.mm.1	3,87	Slc25a22	PSR0700038174.mm.1	-3,02	0,01938	0,473624	Intron Retention	0,48

TC0700004538.mm.1	3,87	Slc25a22	PSR0700038177.mm.1	-3,1	0,00742	0,399901	Intron Retention	0,17
TC0700004538.mm.1	3,87	Slc25a22	PSR0700038198.mm.1	-3,12	0,012858	0,437778	Intron Retention	0,28
TC0700004538.mm.1	3,87	Slc25a22	PSR0700038169.mm.1	-3,14	0,003476	0,355628	Intron Retention	0,53
TC0700004538.mm.1	3,87	Slc25a22	JUC0700020134.mm.1	-3,17	0,008667	0,408649		
TC0700004538.mm.1	3,87	Slc25a22	PSR0700038212.mm.1	-3,23	0,0159	0,456586	Alternative 5' Donc	0,22
TC0700004538.mm.1	3,87	Slc25a22	PSR0700038181.mm.1	-3,25	0,042847	0,548097		
TC0700004538.mm.1	3,87	Slc25a22	PSR0700038217.mm.1	-3,28	0,031608	0,517338	Alternative 5' Donc	0,05
TC0700004538.mm.1	3,87	Slc25a22	PSR0700038188.mm.1	-3,51	0,015462	0,454312	Intron Retention	0,41
TC0700004538.mm.1	3,87	Slc25a22	PSR0700038166.mm.1	-3,67	0,016665	0,460023	Intron Retention	0,65
TC0700004538.mm.1	3,87	Slc25a22	PSR0700038192.mm.1	-4,12	0,009826	0,417839	Intron Retention	0,75
TC0700004538.mm.1	3,87	Slc25a22	PSR0700038185.mm.1	-4,63	0,039268	0,538976	Intron Retention	0,45
TC0700004538.mm.1	3,87	Slc25a22	JUC0700020127.mm.1	-6,26	0,002184	0,34446		
TC1900001144.mm.1	6,31	Stx3	JUC1900005868.mm.1	3,76	0,005188	0,376876		
TC1900001144.mm.1	6,31	Stx3	JUC1900005869.mm.1	3,72	0,017058	0,461937		
TC1900001144.mm.1	6,31	Stx3	PSR1900010742.mm.1	-2,69	0,000638	0,304044	Cassette Exon	0,34
TC1900001144.mm.1	6,31	Stx3	JUC1900005877.mm.1	-4,3	0,003661	0,357266		
TC1900001144.mm.1	6,31	Stx3	PSR1900010743.mm.1	-5,36	0,002008	0,341091	Cassette Exon	0,51
TC1900001144.mm.1	6,31	Stx3	PSR1900010734.mm.1	-5,39	0,001474	0,329052	Cassette Exon	0,6
TC1900001144.mm.1	6,31	Stx3	PSR1900010746.mm.1	-5,96	0,000952	0,313363	Cassette Exon	0,34
TC1900001144.mm.1	6,31	Stx3	PSR1900010748.mm.1	-6,24	0,007865	0,403545	Cassette Exon	0,34
TC1900001144.mm.1	6,31	Stx3	PSR1900010737.mm.1	-6,7	0,001745	0,335996	Cassette Exon	0,73
TC1900001144.mm.1	6,31	Stx3	JUC1900005879.mm.1	-10,72	0,003125	0,353892		
TC1900001144.mm.1	6,31	Stx3	JUC1900005881.mm.1	-11,46	0,008551	0,408183		
TC1900001144.mm.1	6,31	Stx3	PSR1900010735.mm.1	-13,32	0,001546	0,330631	Cassette Exon	0,73
TC1900001144.mm.1	6,31	Stx3	JUC1900005882.mm.1	-13,69	0,003289	0,354243		
TC1900001144.mm.1	6,31	Stx3	JUC1900005880.mm.1	-23	0,002534	0,349501		
TC0200002341.mm.1	1,26	Cbfa2t2	PSR0200018592.mm.1	3,76	0,022891	0,488524	Alternative 3' Acce	0,43
TC0200001940.mm.1	1,18	Zc3h6; Gm14027	PSR0200015289.mm.1	3,76	0,04842	0,560736	Alternative 3' Acce	0,42
TC1100004192.mm.1	1,39	Aatk	JUC1100020933.mm.1	3,76	0,016357	0,458693		
TC1100004192.mm.1	1,39	Aatk	JUC1100020929.mm.1	3,71	0,033871	0,524121		
TC1100004192.mm.1	1,39	Aatk	JUC1100020927.mm.1	2,97	0,031913	0,518021		
TC1100004192.mm.1	1,39	Aatk	PSR1100039828.mm.1	2,79	0,011653	0,429904	Alternative 3' Acce	0,37
TC1100004192.mm.1	1,39	Aatk	PSR1100039845.mm.1	-2,16	0,003845	0,358723	Cassette Exon	0,11
TC1100004192.mm.1	1,39	Aatk	JUC1100020926.mm.1	-2,26	0,006307	0,389234		
TC1100004192.mm.1	1,39	Aatk	PSR1100039841.mm.1	-2,46	0,001047	0,316361	Cassette Exon	0,35
TC1100004192.mm.1	1,39	Aatk	JUC1100020937.mm.1	-2,82	0,008378	0,4065		
TC0X00003246.mm.1	1,04	Phex	JUC0X00010242.mm.1	3,76	0,018579	0,469618		
TC0X00003246.mm.1	1,04	Phex	JUC0X00010238.mm.1	-2,08	0,010641	0,422898		
TC0X00003246.mm.1	1,04	Phex	PSR0X00020328.mm.1	-4,81	0,020479	0,478018	Alternative 5' Donc	0,36
TC0200001013.mm.1	1,19	Ssb	PSR0200008003.mm.1	3,76	0,019377	0,473624	Alternative 5' Donc	0,35
TC1200001704.mm.1	2,66	Prkd1	JUC1200006498.mm.1	3,76	0,043779	0,550398		
TC1200001704.mm.1	2,66	Prkd1	JUC1200006492.mm.1	2,58	0,016461	0,459205		
TC1200001704.mm.1	2,66	Prkd1	PSR1200011738.mm.1	2,26	0,000372	0,291725	Cassette Exon	0,18
TC1200001704.mm.1	2,66	Prkd1	PSR1200011744.mm.1	-2,14	0,016015	0,457051	Cassette Exon	0,14
TC1200001704.mm.1	2,66	Prkd1	PSR1200011751.mm.1	-2,24	0,005806	0,383104	Cassette Exon	0,18
TC1200001704.mm.1	2,66	Prkd1	JUC1200006509.mm.1	-2,52	0,012094	0,43255		
TC1200001704.mm.1	2,66	Prkd1	JUC1200006499.mm.1	-2,72	0,048375	0,56056		
TC1700000971.mm.1	1,02	Kcnh8	JUC1700004930.mm.1	3,76	0,047854	0,559373		

TC0500001452.mm.1	-1,14	Dnah10	JUC0500007174.mm.1	3,76	0,032985	0,521353		
TC0500001452.mm.1	-1,14	Dnah10	JUC0500007235.mm.1	3,21	0,018435	0,468646		
TC0500000060.mm.1	1,26	9330182L06Rik	JUC0500000331.mm.1	3,76	0,008179	0,40568		
TC0500000060.mm.1	1,26	9330182L06Rik	JUC0500000328.mm.1	2,32	0,012894	0,438165		
TC0900002254.mm.1	2,89	Ncam1	JUC0900010589.mm.1	3,75	0,006687	0,393201		
TC0900002254.mm.1	2,89	Ncam1	JUC0900010585.mm.1	3,73	0,000149	0,272178		
TC0900002254.mm.1	2,89	Ncam1	PSR0900018801.mm.1	3,1	0,003612	0,356223	Cassette Exon	0,22
TC0900002254.mm.1	2,89	Ncam1	JUC0900010591.mm.1	2,47	0,00918	0,413594		
TC0900002254.mm.1	2,89	Ncam1	JUC0900010590.mm.1	2,33	0,001494	0,329143		
TC0900002254.mm.1	2,89	Ncam1	PSR0900018821.mm.1	-2,01	0,034181	0,525025	Alternative 5' Donc	0,18
TC0900002254.mm.1	2,89	Ncam1	PSR0900018788.mm.1	-2,55	0,034611	0,52602	Cassette Exon	0,35
TC0900002254.mm.1	2,89	Ncam1	JUC0900010587.mm.1	-2,59	0,024885	0,49558		
TC0900002254.mm.1	2,89	Ncam1	PSR0900018822.mm.1	-2,68	0,043567	0,550033	Alternative 5' Donc	0,18
TC0900002254.mm.1	2,89	Ncam1	PSR0900018796.mm.1	-2,8	0,008552	0,408183		
TC0900002254.mm.1	2,89	Ncam1	PSR0900018806.mm.1	-2,86	0,031837	0,517856	Alternative 3' Acce	0,26
TC0900002254.mm.1	2,89	Ncam1	PSR0900018799.mm.1	-2,87	0,03958	0,539856	Cassette Exon	0,44
TC0900002254.mm.1	2,89	Ncam1	PSR0900018798.mm.1	-3,38	0,036163	0,529905	Cassette Exon	0,36
TC0900002254.mm.1	2,89	Ncam1	PSR0900018797.mm.1	-3,38	0,017755	0,465425	Alternative 5' Donc	0,09
TC0900002254.mm.1	2,89	Ncam1	PSR0900018786.mm.1	-3,71	0,007616	0,401872	Cassette Exon	0,13
TC0900002254.mm.1	2,89	Ncam1	PSR0900018808.mm.1	-4,85	0,034839	0,526823	Alternative 5' Donc	0,48
TC0900002254.mm.1	2,89	Ncam1	JUC0900010586.mm.1	-5,12	0,006972	0,395722		
TC0900002254.mm.1	2,89	Ncam1	PSR0900018795.mm.1	-7,52	0,00505	0,374728	Alternative 3' Acce	0,19
TC0900002254.mm.1	2,89	Ncam1	JUC0900010608.mm.1	-8,85	0,017324	0,463383		
TC1100002025.mm.1	2,67	Fscn2	JUC1100010158.mm.1	3,75	0,007536	0,401261		
TC1100002025.mm.1	2,67	Fscn2	PSR1100019443.mm.1	3,16	0,006186	0,387873	Alternative 3' Acce	0,43
TC1100002025.mm.1	2,67	Fscn2	PSR1100019445.mm.1	-2,23	0,0127	0,43642	Cassette Exon	0,25
TC1100002025.mm.1	2,67	Fscn2	PSR1100019442.mm.1	-2,27	0,005791	0,383104	Alternative 3' Acce	0,33
TC1100002025.mm.1	2,67	Fscn2	PSR1100019446.mm.1	-2,56	0,005657	0,381879	Intron Retention	0,13
TC1100002025.mm.1	2,67	Fscn2	PSR1100019440.mm.1	-2,96	0,006918	0,394973	Alternative 3' Acce	0,31
TC1100002025.mm.1	2,67	Fscn2	JUC1100010160.mm.1	-4	0,036424	0,530958		
TC1600001011.mm.1	1,44	Itsn1	PSR1600007947.mm.1	3,75	0,022656	0,487609	Cassette Exon	0,35
TC1600001011.mm.1	1,44	Itsn1	JUC1600004124.mm.1	3,56	0,002898	0,352207		
TC1600001011.mm.1	1,44	Itsn1	JUC1600004120.mm.1	2,95	0,033382	0,522707		
TC1600001011.mm.1	1,44	Itsn1	PSR1600007950.mm.1	2,71	0,024674	0,49472	Cassette Exon	0,23
TC1600001011.mm.1	1,44	Itsn1	PSR1600007954.mm.1	2,56	0,024815	0,495292	Cassette Exon	0,28
TC1600001011.mm.1	1,44	Itsn1	PSR1600007946.mm.1	2,51	0,000349	0,290082	Cassette Exon	0,19
TC1600001011.mm.1	1,44	Itsn1	JUC1600004116.mm.1	2,16	0,013671	0,444535		
TC1600001011.mm.1	1,44	Itsn1	JUC1600004118.mm.1	2,14	0,032979	0,521324		
TC1600001011.mm.1	1,44	Itsn1	JUC1600004117.mm.1	2,02	0,024301	0,49362		
TC1600001011.mm.1	1,44	Itsn1	PSR1600007948.mm.1	2,01	0,005416	0,379167	Cassette Exon	0,21
TC1600001011.mm.1	1,44	Itsn1	JUC1600004088.mm.1	-2,02	0,020354	0,47734		
TC1600001011.mm.1	1,44	Itsn1	PSR1600007924.mm.1	-2,14	0,000682	0,304417	Cassette Exon	0,12
TC1600001011.mm.1	1,44	Itsn1	JUC1600004091.mm.1	-2,17	0,043563	0,550033		
TC1600001011.mm.1	1,44	Itsn1	JUC1600004111.mm.1	-2,21	0,020534	0,478295		
TC1600001011.mm.1	1,44	Itsn1	JUC1600004134.mm.1	-2,54	0,027877	0,505612		
TC0X00002116.mm.1	1,5	Tenm1	JUC0X00006891.mm.1	3,75	0,017248	0,462729		
TC0X00002116.mm.1	1,5	Tenm1	PSR0X00013485.mm.1	2,26	0,01503	0,452111	Cassette Exon	0,18
TC0X00002116.mm.1	1,5	Tenm1	JUC0X00006879.mm.1	2,23	0,025511	0,497664		

TC0X00002116.mm.1	1,5	Tenm1	PSR0X00013492.mm.1	-2,1	0,017014	0,461709	Cassette Exon	0,05
TC0X00002116.mm.1	1,5	Tenm1	JUC0X00006863.mm.1	-2,29	0,020536	0,478295		
TC1000001334.mm.1	1,61	Ptprr	JUC1000005392.mm.1	3,75	0,004546	0,368794		
TC1000001334.mm.1	1,61	Ptprr	PSR1000009960.mm.1	2,41	0,009095	0,412827	Cassette Exon	0,08
TC1000001334.mm.1	1,61	Ptprr	PSR1000009951.mm.1	-2,14	0,015207	0,452773	Cassette Exon	0,15
TC1000001334.mm.1	1,61	Ptprr	JUC1000005399.mm.1	-2,24	0,037683	0,534394		
TC1000001334.mm.1	1,61	Ptprr	PSR1000009947.mm.1	-2,38	0,027035	0,502979	Alternative 3' Acce	0,15
TC1000001334.mm.1	1,61	Ptprr	PSR1000009956.mm.1	-2,41	0,017798	0,465641	Cassette Exon	0,03
TC1000001334.mm.1	1,61	Ptprr	PSR1000009962.mm.1	-2,46	0,029542	0,510303	Cassette Exon	0,17
TC1000001334.mm.1	1,61	Ptprr	JUC1000005401.mm.1	-2,61	0,036137	0,529805		
TC1400001579.mm.1	1,18	Polr3a	JUC1400006465.mm.1	3,75	0,046644	0,55681		
TC1400001579.mm.1	1,18	Polr3a	JUC1400006489.mm.1	2,65	0,003832	0,358553		
TC1400001579.mm.1	1,18	Polr3a	JUC1400006471.mm.1	-2,1	0,024725	0,494838		
TC1300001112.mm.1	1,15	Ankra2	JUC1300003710.mm.1	3,75	0,026716	0,502096		
TC0300002765.mm.1	3,02	Ntng1	PSR0300021775.mm.1	3,74	0,001088	0,317224	Mutually Exclusive	0,46
TC0300002765.mm.1	3,02	Ntng1	JUC0300011341.mm.1	3,55	0,009469	0,416539		
TC0300002765.mm.1	3,02	Ntng1	JUC0300011345.mm.1	-2,02	0,034242	0,525168		
TC0300002765.mm.1	3,02	Ntng1	PSR0300021795.mm.1	-2,04	0,038438	0,536405	Cassette Exon	0,1
TC0300002765.mm.1	3,02	Ntng1	PSR0300021766.mm.1	-2,31	0,001476	0,329052	Alternative 3' Acce	0,16
TC0300002765.mm.1	3,02	Ntng1	PSR0300021771.mm.1	-2,43	0,000149	0,272178	Alternative 5' Donc	0,18
TC0300002765.mm.1	3,02	Ntng1	PSR0300021774.mm.1	-2,68	0,004375	0,365775	Mutually Exclusive	0,46
TC0300002765.mm.1	3,02	Ntng1	PSR0300021773.mm.1	-2,85	0,019383	0,473624	Mutually Exclusive	0,46
TC0300002765.mm.1	3,02	Ntng1	JUC0300011342.mm.1	-3,34	0,002387	0,348564		
TC0300002765.mm.1	3,02	Ntng1	JUC0300011362.mm.1	-3,64	0,049417	0,563031		
TC0300002765.mm.1	3,02	Ntng1	JUC0300011355.mm.1	-3,86	0,00445	0,367227		
TC0300002765.mm.1	3,02	Ntng1	JUC0300011354.mm.1	-4,06	0,029934	0,511499		
TC0300002765.mm.1	3,02	Ntng1	JUC0300011353.mm.1	-5,01	0,02793	0,505612		
TC0300002765.mm.1	3,02	Ntng1	PSR0300021783.mm.1	-6,93	0,007334	0,399002	Cassette Exon	0,24
TC0300002765.mm.1	3,02	Ntng1	PSR0300021780.mm.1	-18,6	0,03396	0,524252	Cassette Exon	0,21
TC0100001133.mm.1	-1,23		PSR0100009205.mm.1	3,74	0,032186	0,518833	Alternative 5' Donc	0,44
TC0700000300.mm.1	1,55	Ercc2; Mir343	JUC0700001144.mm.1	3,74	0,008752	0,409704		
TC0700000300.mm.1	1,55	Ercc2; Mir343	PSR0700002382.mm.1	-2,17	0,016696	0,460085	Intron Retention	0,29
TC0700000300.mm.1	1,55	Ercc2; Mir343	PSR0700002378.mm.1	-2,35	0,015945	0,456831		
TC0700000300.mm.1	1,55	Ercc2; Mir343	JUC0700001154.mm.1	-4,49	0,045786	0,554863		
TC0100002948.mm.1	1,66	RP23-386K20.2	PSR0100023670.mm.1	3,74	0,019578	0,474672	Cassette Exon	0,24
TC0100002948.mm.1	1,66	RP23-386K20.2	PSR0100023677.mm.1	-2,35	0,030355	0,512923	Intron Retention	0,22
TC0100002948.mm.1	1,66	RP23-386K20.2	JUC0100013427.mm.1	-2,39	0,026984	0,502838		
TC1500000777.mm.1	-1,12	Mpped1	JUC1500003421.mm.1	3,74	0,020947	0,47998		
TC0800000916.mm.1	1,55	Tbc1d9	JUC0800003585.mm.1	3,74	0,02382	0,491955		
TC0800000916.mm.1	1,55	Tbc1d9	JUC0800003597.mm.1	2,3	0,036412	0,530904		
TC0800000916.mm.1	1,55	Tbc1d9	JUC0800003584.mm.1	-3,59	0,011862	0,430929		
TC0500000221.mm.1	-1,1	Nub1	JUC0500000995.mm.1	3,74	0,000335	0,28927		
TC0500002558.mm.1	2,39	Gabra2	PSR0500022647.mm.1	3,73	0,034564	0,525975	Alternative 5' Donc	0,44
TC0500002558.mm.1	2,39	Gabra2	PSR0500022646.mm.1	-2,51	0,011569	0,429011		
TC0800000488.mm.1	-1,03	Pcm1	JUC0800001815.mm.1	3,73	0,019797	0,475212		
TC0600000563.mm.1	1,15	AI854703	JUC0600002195.mm.1	3,73	0,020594	0,478531		
TC1100001629.mm.1	-1,08	Nkiras2	JUC1100007826.mm.1	3,73	0,019355	0,473512		
TC1100001629.mm.1	-1,08	Nkiras2	JUC1100007825.mm.1	2,15	0,003784	0,357586		

TC0100001048.mm.1	1,4	Cntnap5a	JUC0100005002.mm.1	3,72	0,01397	0,446239		
TC0100001048.mm.1	1,4	Cntnap5a	JUC0100005009.mm.1	3,31	0,01976	0,474996		
TC0100001048.mm.1	1,4	Cntnap5a	PSR0100008681.mm.1	2,93	0,00471	0,370888	Cassette Exon	0,29
TC0100001048.mm.1	1,4	Cntnap5a	JUC0100005000.mm.1	2,66	0,004359	0,365487		
TC0100001048.mm.1	1,4	Cntnap5a	PSR0100008685.mm.1	2,05	0,011146	0,426226	Cassette Exon	0,13
TC0100001048.mm.1	1,4	Cntnap5a	JUC0100005011.mm.1	-2,81	0,047962	0,559552		
TC0100001048.mm.1	1,4	Cntnap5a	JUC0100005008.mm.1	-3,19	0,014366	0,448293		
TC0100002827.mm.1	2,8	Kif1a	JUC0100013013.mm.1	3,72	0,029924	0,511499		
TC0100002827.mm.1	2,8	Kif1a	JUC0100012967.mm.1	-2,23	0,016692	0,460085		
TC0100002827.mm.1	2,8	Kif1a	PSR0100022847.mm.1	-2,58	0,011011	0,425601	Cassette Exon	0,2
TC0100002827.mm.1	2,8	Kif1a	JUC0100013004.mm.1	-2,79	0,000106	0,265347		
TC0100002827.mm.1	2,8	Kif1a	JUC0100012980.mm.1	-4,15	0,011589	0,429241		
TC1400001288.mm.1	-3,73	Cldn10	JUC1400005157.mm.1	3,72	0,005129	0,376377		
TC1400001288.mm.1	-3,73	Cldn10	JUC1400005161.mm.1	3,63	0,019245	0,473063		
TC1400001288.mm.1	-3,73	Cldn10	JUC1400005163.mm.1	3,11	0,017158	0,46232		
TC1400001288.mm.1	-3,73	Cldn10	PSR1400009700.mm.1	2,65	0,041923	0,545814	Cassette Exon	0,13
TC1400001288.mm.1	-3,73	Cldn10	PSR1400009702.mm.1	2,63	0,007773	0,402826	Alternative 3' Acce	0,2
TC1400001288.mm.1	-3,73	Cldn10	PSR1400009701.mm.1	2,2	0,035714	0,529027	Cassette Exon	0,13
TC0200000518.mm.1	1,64	Sptan1	JUC0200001999.mm.1	3,72	0,008242	0,405976		
TC0200000518.mm.1	1,64	Sptan1	PSR0200004005.mm.1	-2,4	0,031481	0,516965	Alternative 5' Donc	0,11
TC0900000313.mm.1	1,52	Kirrel3; RP24-225I21.1	JUC0900001270.mm.1	3,72	0,015834	0,456139		
TC0900000313.mm.1	1,52	Kirrel3; RP24-225I21.1	JUC0900001281.mm.1	2,93	0,009919	0,418378		
TC0900000313.mm.1	1,52	Kirrel3; RP24-225I21.1	JUC0900001271.mm.1	-2,59	0,047072	0,557783		
TC0300001647.mm.1	1,04	Snx16	JUC0300006941.mm.1	3,72	0,00746	0,400347		
TC0Y00000006.mm.1	6,73	Eif2s3y	JUC0Y00000065.mm.1	3,72	0,011119	0,426		
TC0200000953.mm.1	2,63	Scn2a1	JUC0200003870.mm.1	3,71	0,037923	0,535147		
TC0200000953.mm.1	2,63	Scn2a1	JUC0200003868.mm.1	2,53	0,020349	0,47734		
TC0200000953.mm.1	2,63	Scn2a1	PSR0200007548.mm.1	-3,83	0,025487	0,497664	Alternative 3' Acce	0,45
TC0900000066.mm.1	2	Trpc6	JUC0900000279.mm.1	3,71	0,022716	0,487767		
TC0900000066.mm.1	2	Trpc6	PSR0900000500.mm.1	-2,04	0,003859	0,358861	Cassette Exon	0,14
TC0900000066.mm.1	2	Trpc6	PSR0900000496.mm.1	-2,88	0,010388	0,421341	Alternative 3' Acce	0,12
TC1400002873.mm.1	-1,69		JUC1400010406.mm.1	3,71	0,01509	0,452418		
TC0200001945.mm.1	-1,36	Ttl	JUC0200007710.mm.1	3,71	0,008144	0,405416		
TC0500001031.mm.1	-1,6	Zfp33b	JUC0500004799.mm.1	3,71	0,043055	0,54841		
TC0500001031.mm.1	-1,6	Zfp33b	JUC0500004810.mm.1	2,98	0,048987	0,561849		
TC0500001031.mm.1	-1,6	Zfp33b	JUC0500004793.mm.1	2,92	0,03917	0,538679		
TC0500001031.mm.1	-1,6	Zfp33b	JUC0500004794.mm.1	2,92	0,03917	0,538679		
TC0500001031.mm.1	-1,6	Zfp33b	JUC0500004797.mm.1	2,92	0,03917	0,538679		
TC0500001031.mm.1	-1,6	Zfp33b	JUC0500004804.mm.1	2,45	0,030245	0,512493		
TC0800003184.mm.1	1,05	Sipa1l2	JUC0800013368.mm.1	3,71	0,042273	0,547092		
TC0200004776.mm.1	-1,07	Ralgapa2	PSR0200040923.mm.1	3,7	0,021122	0,480611	Cassette Exon	0,3
TC0200004776.mm.1	-1,07	Ralgapa2	JUC0200021192.mm.1	3,55	0,017513	0,464009		
TC0200004776.mm.1	-1,07	Ralgapa2	PSR0200040924.mm.1	2,84	0,033551	0,523131	Cassette Exon	0,17
TC0200004776.mm.1	-1,07	Ralgapa2	PSR0200040918.mm.1	2,69	0,045653	0,554766	Cassette Exon	0,29
TC0200004776.mm.1	-1,07	Ralgapa2	PSR0200040911.mm.1	2,07	0,041245	0,544166	Cassette Exon	0,13
TC1400000411.mm.1	-1,32	Mat1a	JUC1400002119.mm.1	3,7	0,000331	0,28927		
TC1200000427.mm.1	1,38	Arhgap5	JUC1200001694.mm.1	3,7	0,002557	0,349501		
TC0100002806.mm.1	-1,02	Hdac4	JUC0100012888.mm.1	3,7	0,02299	0,488935		

TC0500001079.mm.1	1,37	A830010M20Rik; 170002ɛ	PSR0500009101.mm.1	3,69	0,00136	0,32572	Intron Retention	0,48
TC0500001079.mm.1	1,37	A830010M20Rik; 170002ɛ	PSR0500009102.mm.1	3,04	0,001529	0,330287	Intron Retention	0,48
TC0500001079.mm.1	1,37	A830010M20Rik; 170002ɛ	JUC0500004960.mm.1	2,64	0,036753	0,531894		
TC0500001079.mm.1	1,37	A830010M20Rik; 170002ɛ	PSR0500009098.mm.1	2,19	0,029287	0,509874	Alternative 3' Acce	0,16
TC0500001079.mm.1	1,37	A830010M20Rik; 170002ɛ	JUC0500004972.mm.1	-2,8	0,040248	0,541731		
TC0700002710.mm.1	-2,79	Eif3k	PSR0700023795.mm.1	3,69	0,02085	0,479521	Alternative 5' Donc	0,42
TC1100003034.mm.1	2,54	Gucy2e	PSR1100027555.mm.1	3,69	0,034174	0,524992	Cassette Exon	0,33
TC1100003034.mm.1	2,54	Gucy2e	JUC1100014416.mm.1	3,3	0,002228	0,345773		
TC1100003034.mm.1	2,54	Gucy2e	JUC1100014402.mm.1	3,14	0,028211	0,506368		
TC1100003034.mm.1	2,54	Gucy2e	JUC1100014408.mm.1	2,92	0,000676	0,304338		
TC1100003034.mm.1	2,54	Gucy2e	JUC1100014406.mm.1	2,6	0,002258	0,34578		
TC1100003034.mm.1	2,54	Gucy2e	PSR1100027551.mm.1	2,59	0,015538	0,454617	Cassette Exon	0,24
TC1100003034.mm.1	2,54	Gucy2e	JUC1100014409.mm.1	2,45	0,017226	0,462633		
TC1100003034.mm.1	2,54	Gucy2e	JUC1100014403.mm.1	2,14	0,04157	0,544941		
TC1100003034.mm.1	2,54	Gucy2e	JUC1100014415.mm.1	2,11	0,047311	0,558289		
TC1100003034.mm.1	2,54	Gucy2e	PSR1100027570.mm.1	2,03	0,036941	0,532353	Cassette Exon	0,08
TC1100003034.mm.1	2,54	Gucy2e	JUC1100014413.mm.1	-2,19	0,019018	0,471745		
TC1100003034.mm.1	2,54	Gucy2e	JUC1100014418.mm.1	-2,22	0,039806	0,540795		
TC1100003034.mm.1	2,54	Gucy2e	PSR1100027562.mm.1	-2,56	0,036876	0,532257	Alternative 3' Acce	0,24
TC1100003034.mm.1	2,54	Gucy2e	PSR1100027552.mm.1	-2,59	0,004946	0,373293	Intron Retention	0,34
TC0X00003260.mm.1	2,26	Cnksr2	JUC0X00010294.mm.1	3,69	0,017922	0,466466		
TC0X00003260.mm.1	2,26	Cnksr2	JUC0X00010290.mm.1	-2,02	0,036539	0,531355		
TC0X00003260.mm.1	2,26	Cnksr2	JUC0X00010299.mm.1	-2,18	0,020539	0,478295		
TC0X00003260.mm.1	2,26	Cnksr2	PSR0X00020427.mm.1	-2,34	0,009058	0,412827	Cassette Exon	0,18
TC0X00003260.mm.1	2,26	Cnksr2	PSR0X00020405.mm.1	-2,44	0,035092	0,527591	Cassette Exon	0,08
TC0X00003260.mm.1	2,26	Cnksr2	PSR0X00020428.mm.1	-2,98	0,030954	0,514654	Cassette Exon	0,18
TC0X00003260.mm.1	2,26	Cnksr2	JUC0X00010291.mm.1	-2,98	0,004979	0,373851		
TC0X00003260.mm.1	2,26	Cnksr2	JUC0X00010295.mm.1	-3,23	0,024335	0,49362		
TC1700001096.mm.1	-1,05	Lama1	JUC1700005609.mm.1	3,69	0,010104	0,418998		
TC1700001096.mm.1	-1,05	Lama1	PSR1700010320.mm.1	-2,12	0,007115	0,397032	Alternative 5' Donc	0,18
TC1100003814.mm.1	-1,1	Etv4	JUC1100018475.mm.1	3,69	0,035323	0,528381		
TC1100003814.mm.1	-1,1	Etv4	JUC1100018471.mm.1	2,36	0,009472	0,416539		
TC1800001431.mm.1	-1	Ablim3	JUC1800005850.mm.1	3,69	0,025976	0,499207		
TC1800001431.mm.1	-1	Ablim3	JUC1800005844.mm.1	3	0,024603	0,494497		
TC1100001702.mm.1	1,43	Fam187a; Ccdc103	JUC1100008313.mm.1	3,69	0,00313	0,353892		
TC0500000267.mm.1	1,14	Rnf32	PSR0500002049.mm.1	3,68	0,047676	0,558878	Alternative 5' Donc	0,43
TC0500000267.mm.1	1,14	Rnf32	PSR0500002034.mm.1	2,6	0,047635	0,558743		
TC0500000267.mm.1	1,14	Rnf32	PSR0500002033.mm.1	2,45	0,002403	0,348653		
TC1500001982.mm.1	1,85	Sult4a1	PSR1500015745.mm.1	3,68	0,000128	0,270905	Cassette Exon	0,24
TC1500001982.mm.1	1,85	Sult4a1	JUC1500008936.mm.1	3,14	0,000225	0,28803		
TC1500001982.mm.1	1,85	Sult4a1	JUC1500008937.mm.1	2,46	0,015617	0,45509		
TC1500001982.mm.1	1,85	Sult4a1	PSR1500015742.mm.1	2,16	0,000229	0,28803	Cassette Exon	0,08
TC1500001982.mm.1	1,85	Sult4a1	PSR1500015747.mm.1	-2,22	0,014274	0,448054		
TC1500001982.mm.1	1,85	Sult4a1	JUC1500008940.mm.1	-2,74	0,016553	0,459612		
TC0300001803.mm.1	1,18	Mrpl47	JUC0300007357.mm.1	3,68	0,014734	0,450562		
TC0600002802.mm.1	1,56	Frmd4b	PSR0600021775.mm.1	3,67	0,000468	0,298999	Cassette Exon	0,38
TC0600002802.mm.1	1,56	Frmd4b	JUC0600011324.mm.1	3,42	0,020546	0,478319		
TC0600002802.mm.1	1,56	Frmd4b	JUC0600011326.mm.1	2,91	0,004768	0,371039		

TC0600002802.mm.1	1,56	Frmd4b	PSR0600021763.mm.1	2,87	0,00132	0,324348	Cassette Exon	0,16
TC0600002802.mm.1	1,56	Frmd4b	PSR0600021776.mm.1	2,73	0,003833	0,358553	Cassette Exon	0,34
TC0600002802.mm.1	1,56	Frmd4b	PSR0600021765.mm.1	2,55	0,000869	0,311909	Cassette Exon	0,2
TC0600002802.mm.1	1,56	Frmd4b	JUC0600011323.mm.1	2,54	0,010109	0,418998		
TC0600002802.mm.1	1,56	Frmd4b	PSR0600021768.mm.1	2,43	0,003952	0,359922	Cassette Exon	0,21
TC0600002802.mm.1	1,56	Frmd4b	JUC0600011322.mm.1	2,4	0,019904	0,475879		
TC0600002802.mm.1	1,56	Frmd4b	PSR0600021771.mm.1	2,39	0,001178	0,319796	Mutually Exclusive	0,21
TC0600002802.mm.1	1,56	Frmd4b	PSR0600021781.mm.1	2,29	0,012102	0,43255	Cassette Exon	0,28
TC0600002802.mm.1	1,56	Frmd4b	JUC0600011319.mm.1	2,1	0,003545	0,356223		
TC0600002802.mm.1	1,56	Frmd4b	JUC0600011321.mm.1	2,03	0,02901	0,508842		
TC0600002802.mm.1	1,56	Frmd4b	PSR0600021773.mm.1	-2,2	0,029312	0,509874	Mutually Exclusive	0,21
TC0600002802.mm.1	1,56	Frmd4b	PSR0600021783.mm.1	-2,25	0,046096	0,555713	Cassette Exon	0,18
TC0600002802.mm.1	1,56	Frmd4b	JUC0600011310.mm.1	-2,56	0,006836	0,393893		
TC0600002802.mm.1	1,56	Frmd4b	PSR0600021769.mm.1	-2,6	0,016253	0,458312	Alternative 5' Donc	0,26
TC0600002802.mm.1	1,56	Frmd4b	PSR0600021757.mm.1	-3,04	0,026244	0,500211	Alternative 5' Donc	0,33
TC0600002802.mm.1	1,56	Frmd4b	PSR0600021756.mm.1	-3,52	0,031709	0,517443	Alternative 5' Donc	0,33
TC0600002802.mm.1	1,56	Frmd4b	JUC0600011314.mm.1	-3,63	0,003915	0,359719		
TC0600002802.mm.1	1,56	Frmd4b	JUC0600011329.mm.1	-4,51	0,000257	0,28803		
TC0600002802.mm.1	1,56	Frmd4b	PSR0600021758.mm.1	-4,91	0,049282	0,562691	Alternative 5' Donc	0,33
TC0200003447.mm.1	1,09	Lrp1b	JUC0200014898.mm.1	3,67	0,00046	0,298999		
TC0200003447.mm.1	1,09	Lrp1b	JUC0200014896.mm.1	3,13	0,001499	0,329714		
TC0200003447.mm.1	1,09	Lrp1b	PSR0200029618.mm.1	3,03	0,005875	0,383835	Cassette Exon	0,26
TC0200003447.mm.1	1,09	Lrp1b	PSR0200029622.mm.1	2,96	0,013929	0,446193	Cassette Exon	0,3
TC0200003447.mm.1	1,09	Lrp1b	PSR0200029590.mm.1	2,74	0,00359	0,356223	Cassette Exon	0,16
TC0200003447.mm.1	1,09	Lrp1b	JUC0200014975.mm.1	2,58	0,036078	0,529704		
TC0200003447.mm.1	1,09	Lrp1b	JUC0200014967.mm.1	2,55	0,004524	0,368648		
TC0200003447.mm.1	1,09	Lrp1b	JUC0200014972.mm.1	2,37	0,036486	0,531183		
TC0200003447.mm.1	1,09	Lrp1b	PSR0200029595.mm.1	2,14	0,017583	0,464264	Cassette Exon	0,12
TC0200003447.mm.1	1,09	Lrp1b	JUC0200014976.mm.1	2,07	0,046369	0,556313		
TC0200003447.mm.1	1,09	Lrp1b	JUC0200014928.mm.1	-3,08	0,031145	0,515638		
TC1300002287.mm.1	-1,49	Adamts16	JUC1300007840.mm.1	3,67	0,047401	0,558302		
TC0100000493.mm.1	-1,1	9530026F06Rik	JUC0100002363.mm.1	3,67	0,017759	0,465425		
TC1600000948.mm.1	1		JUC1600003884.mm.1	3,67	0,010237	0,420128		
TC0500002877.mm.1	-1,43	1700016H13Rik	JUC0500013818.mm.1	3,67	0,035309	0,528381		
TC0500002877.mm.1	-1,43	1700016H13Rik	JUC0500013819.mm.1	2,62	0,001927	0,34017		
TC0600000396.mm.1	-1,18	8030453O22Rik	JUC0600001536.mm.1	3,67	0,034818	0,526823		
TC1800000261.mm.1	2,31	Bin1	JUC1800001119.mm.1	3,66	0,001669	0,335182		
TC1800000261.mm.1	2,31	Bin1	PSR1800001875.mm.1	3,6	0,000394	0,29748	Cassette Exon	0,6
TC1800000261.mm.1	2,31	Bin1	JUC1800001118.mm.1	2,37	0,027463	0,504045		
TC1800000261.mm.1	2,31	Bin1	PSR1800001885.mm.1	-2,02	0,012648	0,436303	Cassette Exon	0,18
TC1800000261.mm.1	2,31	Bin1	JUC1800001115.mm.1	-2,02	0,049549	0,563325		
TC1800000261.mm.1	2,31	Bin1	PSR1800001884.mm.1	-2,61	0,016851	0,460797	Cassette Exon	0,33
TC1800000261.mm.1	2,31	Bin1	JUC1800001109.mm.1	-3,57	0,029312	0,509874		
TC1800000261.mm.1	2,31	Bin1	JUC1800001124.mm.1	-4,71	0,000701	0,30566		
TC0200005155.mm.1	1,66	Zmynd8	JUC0200022743.mm.1	3,66	0,027006	0,502955		
TC0200005155.mm.1	1,66	Zmynd8	JUC0200022744.mm.1	3,15	0,007673	0,402377		
TC0200005155.mm.1	1,66	Zmynd8	JUC0200022763.mm.1	-2,04	0,000656	0,304044		
TC0200005155.mm.1	1,66	Zmynd8	JUC0200022762.mm.1	-2,09	0,035012	0,527415		

TC0200005155.mm.1	1,66	Zmynd8	PSR0200044097.mm.1	-2,13	0,027239	0,503642	Cassette Exon	0,09
TC0200005155.mm.1	1,66	Zmynd8	PSR0200044098.mm.1	-2,16	0,002432	0,349135	Cassette Exon	0,09
TC0200005155.mm.1	1,66	Zmynd8	JUC0200022747.mm.1	-2,17	0,001549	0,330631		
TC0200005155.mm.1	1,66	Zmynd8	PSR0200044106.mm.1	-2,2	0,046348	0,556272	Alternative 3' Acce	0,19
TC0200005155.mm.1	1,66	Zmynd8	PSR0200044115.mm.1	-2,35	0,015755	0,455437	Alternative 5' Donc	0,11
TC0200005155.mm.1	1,66	Zmynd8	PSR0200044104.mm.1	-2,38	0,031783	0,517596	Cassette Exon	0,17
TC0200005155.mm.1	1,66	Zmynd8	PSR0200044099.mm.1	-2,51	0,014776	0,450801	Alternative 5' Donc	0,06
TC0200005155.mm.1	1,66	Zmynd8	JUC0200022741.mm.1	-2,58	0,00053	0,304044		
TC0200005155.mm.1	1,66	Zmynd8	PSR0200044117.mm.1	-4	0,000126	0,270905	Cassette Exon	0,26
TC0600001111.mm.1	1,19		PSR0600008734.mm.1	3,66	0,010457	0,421652	Cassette Exon	0,25
TC0400002289.mm.1	1,52	Prdm13	PSR0400018934.mm.1	3,66	0,001429	0,327447	Cassette Exon	0,22
TC1900001753.mm.1	1,13	Sfxn4	JUC1900008615.mm.1	3,66	0,022431	0,486856		
TC1900001753.mm.1	1,13	Sfxn4	JUC1900008607.mm.1	3,11	0,029355	0,509995		
TC0400002705.mm.1	1,31	Ikbkap	JUC0400011306.mm.1	3,66	0,006189	0,387873		
TC0600001268.mm.1	11,84	Slc6a1	JUC0600005169.mm.1	3,65	0,007637	0,402113		
TC0600001268.mm.1	11,84	Slc6a1	JUC0600005162.mm.1	2,17	0,011428	0,427656		
TC0600001268.mm.1	11,84	Slc6a1	PSR0600009990.mm.1	-2,15	0,000069	0,250992	Cassette Exon	0,25
TC0600001268.mm.1	11,84	Slc6a1	PSR0600009980.mm.1	-2,18	0,028616	0,507749	Cassette Exon	0,21
TC0600001268.mm.1	11,84	Slc6a1	PSR0600009974.mm.1	-2,2	0,010137	0,419217	Cassette Exon	0,17
TC0600001268.mm.1	11,84	Slc6a1	JUC0600005160.mm.1	-2,21	0,015014	0,452101		
TC0600001268.mm.1	11,84	Slc6a1	JUC0600005164.mm.1	-2,28	0,049305	0,562723		
TC0600001268.mm.1	11,84	Slc6a1	PSR0600009975.mm.1	-2,44	0,002095	0,342919	Alternative 3' Acce	0,41
TC0600001268.mm.1	11,84	Slc6a1	PSR0600009979.mm.1	-2,47	0,000772	0,308801	Cassette Exon	0,1
TC0600001268.mm.1	11,84	Slc6a1	PSR0600009983.mm.1	-2,54	0,006814	0,39387	Cassette Exon	0,27
TC0600001268.mm.1	11,84	Slc6a1	JUC0600005161.mm.1	-2,74	0,006879	0,394271		
TC0600001268.mm.1	11,84	Slc6a1	PSR0600009988.mm.1	-7,05	0,003039	0,353826	Alternative 5' Donc	0,49
TC0600001268.mm.1	11,84	Slc6a1	JUC0600005166.mm.1	-7,71	0,000242	0,28803		
TC0600001268.mm.1	11,84	Slc6a1	PSR0600009981.mm.1	-7,78	0,004637	0,37015	Alternative 5' Donc	0,29
TC0600001268.mm.1	11,84	Slc6a1	JUC0600005168.mm.1	-9,03	0,002713	0,349612		
TC0600001268.mm.1	11,84	Slc6a1	JUC0600005173.mm.1	-12,71	0,000695	0,305213		
TC0600001268.mm.1	11,84	Slc6a1	PSR0600009972.mm.1	-15,01	0,001741	0,335996	Cassette Exon	0,28
TC0600001268.mm.1	11,84	Slc6a1	PSR0600009976.mm.1	-38,44	0,002704	0,349612	Intron Retention	0,57
TC1100001728.mm.1	-1,34	Myl4	JUC1100008460.mm.1	3,65	0,011933	0,431703		
TC1100001728.mm.1	-1,34	Myl4	PSR1100016339.mm.1	-3,33	0,040455	0,542112	Alternative 3' Acce	0,37
TC0600002331.mm.1	-1,41	Pde1c	JUC0600009530.mm.1	3,65	0,000967	0,313456		
TC0600002331.mm.1	-1,41	Pde1c	PSR0600018234.mm.1	2,7	0,030367	0,512923	Cassette Exon	0,28
TC0100003565.mm.1	-1,08	Atp1a4	JUC0100016636.mm.1	3,65	0,033416	0,522793		
TC0100003565.mm.1	-1,08	Atp1a4	PSR0100029283.mm.1	2,55	0,010387	0,421341	Cassette Exon	0,18
TC0100003565.mm.1	-1,08	Atp1a4	JUC0100016647.mm.1	-2,55	0,025553	0,497808		
TC0200001976.mm.1	1,1	Ebf4	JUC0200007879.mm.1	3,65	0,018295	0,468049		
TC0800002528.mm.1	-1,11	Smarca5	JUC0800010471.mm.1	3,65	0,036089	0,529716		
TC0X00002741.mm.1	1,1	Hdac8	JUC0X00008741.mm.1	3,65	0,031897	0,518021		
TC1100001154.mm.1	6,64	BC030499	JUC1100005602.mm.1	3,64	0,00137	0,32572		
TC1100001154.mm.1	6,64	BC030499	PSR1100010674.mm.1	2,44	0,000005	0,179072	Cassette Exon	0,22
TC1100001154.mm.1	6,64	BC030499	PSR1100010687.mm.1	-2,02	0,03167	0,517405	Alternative 5' Donc	0,08
TC1100001154.mm.1	6,64	BC030499	PSR1100010690.mm.1	-2,15	0,000217	0,28803	Alternative 5' Donc	0,18
TC1100001154.mm.1	6,64	BC030499	PSR1100010671.mm.1	-2,2	0,002148	0,343679	Cassette Exon	0,24
TC1100001154.mm.1	6,64	BC030499	PSR1100010673.mm.1	-2,34	0,005594	0,381078	Intron Retention	0,34

TC1100001154.mm.1	6,64	BC030499	PSR1100010667.mm.1	-2,39	0,001514	0,330268	Cassette Exon	0,22
TC1100001154.mm.1	6,64	BC030499	PSR1100010666.mm.1	-2,46	0,001139	0,317344	Cassette Exon	0,22
TC1100001154.mm.1	6,64	BC030499	JUC1100005599.mm.1	-2,46	0,003188	0,354243		
TC1100001154.mm.1	6,64	BC030499	JUC1100005608.mm.1	-2,7	0,003566	0,356223		
TC1100001154.mm.1	6,64	BC030499	PSR1100010677.mm.1	-2,92	0,00337	0,354278	Intron Retention	0,48
TC1100001154.mm.1	6,64	BC030499	PSR1100010679.mm.1	-2,93	0,00139	0,325997	Alternative 5' Donc	0,39
TC1100001154.mm.1	6,64	BC030499	PSR1100010693.mm.1	-3,8	0,000513	0,303579	Alternative 5' Donc	0,18
TC1100001154.mm.1	6,64	BC030499	PSR1100010694.mm.1	-4,86	0,006331	0,389463	Cassette Exon	0,41
TC1100001154.mm.1	6,64	BC030499	JUC1100005606.mm.1	-6,05	0,003774	0,357586		
TC1100001154.mm.1	6,64	BC030499	PSR1100010681.mm.1	-6,06	0,006041	0,386586	Alternative 5' Donc	0,39
TC1100001154.mm.1	6,64	BC030499	JUC1100005611.mm.1	-6,07	0,018058	0,46716		
TC1100001154.mm.1	6,64	BC030499	JUC1100005601.mm.1	-6,37	0,002621	0,349612		
TC1100001154.mm.1	6,64	BC030499	JUC1100005610.mm.1	-7,37	0,000974	0,314364		
TC1100001154.mm.1	6,64	BC030499	PSR1100010663.mm.1	-8,76	0,001151	0,317344	Cassette Exon	0,35
TC1100001154.mm.1	6,64	BC030499	JUC1100005605.mm.1	-9,17	0,001363	0,32572		
TC1100001154.mm.1	6,64	BC030499	PSR1100010660.mm.1	-9,86	0,003463	0,355628	Cassette Exon	0,35
TC1100001154.mm.1	6,64	BC030499	PSR1100010692.mm.1	-14,91	0,003376	0,354303	Alternative 5' Donc	0,18
TC0700004340.mm.1	2,33	Stx1b	JUC0700019222.mm.1	3,64	0,032691	0,520439		
TC0700004340.mm.1	2,33	Stx1b	JUC0700019225.mm.1	2,41	0,026954	0,502779		
TC0700004340.mm.1	2,33	Stx1b	PSR0700036448.mm.1	2,31	0,005374	0,378731	Alternative 3' Acce	0,21
TC0700004340.mm.1	2,33	Stx1b	PSR0700036453.mm.1	2,27	0,013019	0,439354	Cassette Exon	0,16
TC0700004340.mm.1	2,33	Stx1b	JUC0700019223.mm.1	-2,17	0,00059	0,304044		
TC0700004340.mm.1	2,33	Stx1b	PSR0700036456.mm.1	-2,23	0,005482	0,379939	Cassette Exon	0,17
TC0700004340.mm.1	2,33	Stx1b	PSR0700036452.mm.1	-2,49	0,013319	0,441836	Intron Retention	0,27
TC0700004340.mm.1	2,33	Stx1b	PSR0700036463.mm.1	-2,5	0,002618	0,349612	Alternative 5' Donc	0,14
TC1100000234.mm.1	-1,09	Cct4	JUC1100000980.mm.1	3,64	0,006127	0,387351		
TC1100000234.mm.1	-1,09	Cct4	JUC1100000973.mm.1	2,62	0,011294	0,427117		
TC1100000234.mm.1	-1,09	Cct4	JUC1100000971.mm.1	2,07	0,019966	0,476202		
TC1100000234.mm.1	-1,09	Cct4	PSR1100001902.mm.1	2,01	0,001067	0,316361	Alternative 5' Donc	0,14
TC0200002566.mm.1	-1,49		JUC0200010708.mm.1	3,64	0,030057	0,511847		
TC0600001082.mm.1	1,12	Hdac11	JUC0600004289.mm.1	3,64	0,027223	0,503582		
TC0600001082.mm.1	1,12	Hdac11	JUC0600004288.mm.1	-2,15	0,01761	0,464312		
TC0600001082.mm.1	1,12	Hdac11	JUC0600004294.mm.1	-3,95	0,022809	0,488243		
TC0X00000298.mm.1	-1,31		JUC0X00000975.mm.1	3,64	0,029324	0,509941		
TC0400001472.mm.1	1,1	AU040320	JUC0400005840.mm.1	3,64	0,006635	0,392813		
TC0400001472.mm.1	1,1	AU040320	JUC0400005846.mm.1	-4,89	0,014776	0,450801		
TC1000001171.mm.1	-1,11	Eea1	JUC1000004789.mm.1	3,64	0,016179	0,457969		
TC0X00002709.mm.1	-1,09	Tex11	JUC0X00008615.mm.1	3,64	0,02535	0,497375		
TC1000002448.mm.1	3,28	Agpat3	PSR1000017856.mm.1	3,63	0,006805	0,393762	Cassette Exon	0,47
TC1000002448.mm.1	3,28	Agpat3	JUC1000009822.mm.1	3,2	0,007177	0,397855		
TC1000002448.mm.1	3,28	Agpat3	JUC1000009820.mm.1	-2,11	0,014875	0,451677		
TC1000002448.mm.1	3,28	Agpat3	PSR1000017853.mm.1	-2,24	0,003759	0,357586	Cassette Exon	0,03
TC1000002448.mm.1	3,28	Agpat3	PSR1000017857.mm.1	-2,49	0,008337	0,406435	Cassette Exon	0,1
TC1000002448.mm.1	3,28	Agpat3	JUC1000009816.mm.1	-2,76	0,021114	0,480564		
TC1000002448.mm.1	3,28	Agpat3	PSR1000017862.mm.1	-2,79	0,020664	0,478862	Cassette Exon	0,3
TC1000002448.mm.1	3,28	Agpat3	PSR1000017868.mm.1	-2,83	0,013004	0,439291	Cassette Exon	0,3
TC1000002448.mm.1	3,28	Agpat3	JUC1000009814.mm.1	-2,88	0,044318	0,551368		
TC1000002448.mm.1	3,28	Agpat3	PSR1000017866.mm.1	-3,37	0,009207	0,414013		

TC1000002448.mm.1	3,28	Agpat3	JUC1000009817.mm.1	-3,48	0,004911	0,372662		
TC1000002448.mm.1	3,28	Agpat3	PSR1000017858.mm.1	-4,38	0,011281	0,426885	Cassette Exon	0,36
TC1000002448.mm.1	3,28	Agpat3	JUC1000009823.mm.1	-4,5	0,014152	0,447466		
TC1000002448.mm.1	3,28	Agpat3	JUC1000009819.mm.1	-4,81	0,000832	0,311886		
TC1000002448.mm.1	3,28	Agpat3	PSR1000017865.mm.1	-5,05	0,035369	0,528536		
TC1100003222.mm.1	-2,06	Serpinf1	JUC1100015524.mm.1	3,63	0,000728	0,306408		
TC1100003222.mm.1	-2,06	Serpinf1	PSR1100029650.mm.1	2,45	0,004202	0,363236	Alternative 3' Acce	0,23
TC1100003222.mm.1	-2,06	Serpinf1	PSR1100029659.mm.1	2,36	0,017903	0,466466	Alternative 3' Acce	0,22
TC1100003222.mm.1	-2,06	Serpinf1	PSR1100029664.mm.1	2,3	0,009421	0,415976	Cassette Exon	0,19
TC1100003222.mm.1	-2,06	Serpinf1	PSR1100029666.mm.1	2,17	0,00045	0,297771	Cassette Exon	0,12
TC0500003687.mm.1	2,12		PSR0500033690.mm.1	3,63	0,007991	0,403853		
TC0500003687.mm.1	2,12		JUC0500018289.mm.1	3,51	0,005024	0,374162		
TC0500003687.mm.1	2,12		PSR0500033692.mm.1	2,08	0,018393	0,468484	Cassette Exon	0,21
TC0500003205.mm.1	-1,09	Tctn1	JUC0500015591.mm.1	3,63	0,013976	0,446242		
TC0500003205.mm.1	-1,09	Tctn1	PSR0500028630.mm.1	2,07	0,028465	0,507299	Alternative 3' Acce	0,17
TC0200004822.mm.1	-1,49		JUC0200021277.mm.1	3,63	0,027532	0,504339		
TC1900000654.mm.1	-1,16	Pax2	JUC1900003079.mm.1	3,63	0,003825	0,358333		
TC0200003026.mm.1	1,73	A930004D18Rik	PSR0200024960.mm.1	3,62	0,028031	0,505851	Alternative 3' Acce	0,3
TC0200003026.mm.1	1,73	A930004D18Rik	PSR0200024957.mm.1	-2,23	0,007819	0,403089	Cassette Exon	0,16
TC0200003026.mm.1	1,73	A930004D18Rik	JUC0200012634.mm.1	-3,05	0,015313	0,453561		
TC1700001289.mm.1	1,42	Prkce	JUC1700006452.mm.1	3,62	0,023915	0,492316		
TC1700001289.mm.1	1,42	Prkce	PSR1700011792.mm.1	2,17	0,041819	0,545586	Cassette Exon	0,08
TC1700001289.mm.1	1,42	Prkce	PSR1700011803.mm.1	2,06	0,012966	0,439129	Cassette Exon	0,09
TC1700001289.mm.1	1,42	Prkce	JUC1700006457.mm.1	-2,07	0,016916	0,460832		
TC0900001563.mm.1	-1,39	Itga9	JUC0900007302.mm.1	3,62	0,021727	0,484124		
TC0900001563.mm.1	-1,39	Itga9	JUC0900007303.mm.1	3,21	0,029629	0,510558		
TC0900001563.mm.1	-1,39	Itga9	JUC0900007301.mm.1	2,85	0,024307	0,49362		
TC1400000798.mm.1	-1,23	Cma2	JUC1400003458.mm.1	3,61	0,020039	0,476453		
TC0100001870.mm.1	-1,02	4930570N18Rik	JUC0100008819.mm.1	3,61	0,001682	0,335182		
TC0500000052.mm.1	-1,06	Abcb1b	JUC0500000241.mm.1	3,61	0,003584	0,356223		
TC0500000052.mm.1	-1,06	Abcb1b	JUC0500000267.mm.1	2,42	0,046264	0,556183		
TC1800000655.mm.1	1,54	Wdr7	PSR1800004824.mm.1	3,6	0,006183	0,387873	Cassette Exon	0,49
TC1800000655.mm.1	1,54	Wdr7	JUC1800002691.mm.1	3,34	0,033498	0,522941		
TC1800000655.mm.1	1,54	Wdr7	JUC1800002692.mm.1	3,33	0,007608	0,401767		
TC1800000655.mm.1	1,54	Wdr7	JUC1800002680.mm.1	2,65	0,010316	0,420661		
TC1500001183.mm.1	3,17	Slc1a3	JUC1500005378.mm.1	3,6	0,0319	0,518021		
TC1500001183.mm.1	3,17	Slc1a3	JUC1500005376.mm.1	2,84	0,014271	0,448053		
TC1500001183.mm.1	3,17	Slc1a3	PSR1500009528.mm.1	-2,85	0,032735	0,52047	Alternative 5' Donc	0,3
TC1500001183.mm.1	3,17	Slc1a3	PSR1500009530.mm.1	-3,67	0,044073	0,550881	Cassette Exon	0,42
TC1500001183.mm.1	3,17	Slc1a3	PSR1500009543.mm.1	-5,62	0,016661	0,460023	Cassette Exon	0,33
TC0700000557.mm.1	3,36	Clip3	JUC0700002382.mm.1	3,6	0,003211	0,354243		
TC0700000557.mm.1	3,36	Clip3	JUC0700002383.mm.1	2,47	0,019349	0,47345		
TC0700000557.mm.1	3,36	Clip3	JUC0700002375.mm.1	2,17	0,02892	0,50858		
TC0700000557.mm.1	3,36	Clip3	JUC0700002380.mm.1	2,07	0,01921	0,472971		
TC0700000557.mm.1	3,36	Clip3	PSR0700004769.mm.1	-3,6	0,042418	0,547627	Alternative 5' Donc	0,41
TC1700002817.mm.1	-1,36	C2	JUC1700009782.mm.1	3,6	0,025808	0,498589		
TC1600002139.mm.1	1,4	Hmgn1	PSR1600016485.mm.1	3,6	0,001344	0,325349		
TC1300000113.mm.1	1,23	Inhba	PSR1300000833.mm.1	3,59	0,035931	0,529399	Alternative 5' Donc	0,4

TC0500000726.mm.1	-1,06	C530008M17Rik; A73008!	JUC0500003581.mm.1	3,59	0,002412	0,348816		
TC0500000726.mm.1	-1,06	C530008M17Rik; A73008!	PSR0500006533.mm.1	2,05	0,006056	0,386642	Alternative 5' Donc	0,17
TC0200001760.mm.1	-1,16	Rad51	JUC0200006555.mm.1	3,59	0,018599	0,469657		
TC0900000917.mm.1	-1,05	Ice2; Narg2	JUC0900003817.mm.1	3,59	0,025583	0,497808		
TC1400001882.mm.1	-1,19	Gm5930	JUC1400007811.mm.1	3,58	0,020946	0,47998		
TC1200001490.mm.1	-1,06	Adam17	JUC1200005778.mm.1	3,58	0,047149	0,557948		
TC0800002793.mm.1	-1,31		JUC0800011655.mm.1	3,58	0,020714	0,479166		
TC0400004018.mm.1	1,03	Ptchd2	JUC0400017346.mm.1	3,58	0,007173	0,397855		
TC0400004018.mm.1	1,03	Ptchd2	JUC0400017354.mm.1	-2,29	0,023308	0,49004		
TC0500000589.mm.1	-1,29	Pgm1	PSR0500005218.mm.1	3,57	0,002838	0,352207	Alternative 5' Donc	0,41
TC0300000272.mm.1	1,42	4932438A13Rik	PSR0300001884.mm.1	3,57	0,000833	0,311886	Cassette Exon	0,32
TC0300000272.mm.1	1,42	4932438A13Rik	JUC0300000959.mm.1	2,78	0,027044	0,502979		
TC0300000272.mm.1	1,42	4932438A13Rik	JUC0300000972.mm.1	2,25	0,003508	0,355749		
TC0300000272.mm.1	1,42	4932438A13Rik	JUC0300000962.mm.1	-2,22	0,008525	0,407684		
TC0600000937.mm.1	-1,05	Atp6v1b1	JUC0600003629.mm.1	3,57	0,001107	0,317328		
TC0600000937.mm.1	-1,05	Atp6v1b1	JUC0600003637.mm.1	-2,55	0,019467	0,474209		
TC0100001373.mm.1	1,63	Nmnat2	JUC0100006550.mm.1	3,56	0,021406	0,482126		
TC0100001373.mm.1	1,63	Nmnat2	PSR0100011356.mm.1	2,41	0,00278	0,351017	Cassette Exon	0,25
TC1500002048.mm.1	1,02	Sbf1	JUC1500009356.mm.1	3,56	0,03513	0,527805		
TC1500002048.mm.1	1,02	Sbf1	JUC1500009365.mm.1	3,04	0,006764	0,393343		
TC1500002048.mm.1	1,02	Sbf1	PSR1500016457.mm.1	2,44	0,024719	0,494838	Cassette Exon	0,23
TC1900001710.mm.1	-1,22	Afap1l2	JUC1900008402.mm.1	3,56	0,011303	0,427133		
TC1900001710.mm.1	-1,22	Afap1l2	PSR1900015065.mm.1	-2,03	0,044128	0,551013	Cassette Exon	0,11
TC1400002111.mm.1	-1,48	Mcpt9	JUC1400008873.mm.1	3,56	0,032401	0,519282		
TC1600001580.mm.1	-1,42	Cd86	JUC1600006705.mm.1	3,56	0,00799	0,403853		
TC0700000894.mm.1	-1,48	Zdhhc13	JUC0700003987.mm.1	3,56	0,009617	0,417315		
TC0300002705.mm.1	1,17		JUC0300010960.mm.1	3,56	0,028101	0,506104		
TC1000001891.mm.1	1	Gm15270	JUC1000007388.mm.1	3,56	0,046451	0,556553		
TC1000001891.mm.1	1	Gm15270	JUC1000007389.mm.1	3,56	0,015143	0,452699		
TC1800000956.mm.1	-1,2	Mpp7	PSR1800007075.mm.1	3,55	0,016753	0,460383	Cassette Exon	0,27
TC0400000836.mm.1	1,29	Ccdc171	JUC0400002893.mm.1	3,55	0,004274	0,363868		
TC0400000836.mm.1	1,29	Ccdc171	JUC0400002897.mm.1	2,15	0,019511	0,474538		
TC0400000836.mm.1	1,29	Ccdc171	PSR0400005715.mm.1	2,06	0,006659	0,393187	Cassette Exon	0,18
TC0X00003163.mm.1	1,22		PSR0X00019816.mm.1	3,55	0,001628	0,332831	Cassette Exon	0,18
TC0X00003163.mm.1	1,22		JUC0X00010017.mm.1	-2,14	0,023433	0,490383		
TC0200003801.mm.1	1,22	Pde11a	JUC0200016542.mm.1	3,55	0,037113	0,532942		
TC0900002368.mm.1	-1,06	Ppcdc	JUC0900011193.mm.1	3,55	0,026196	0,50003		
TC0900002541.mm.1	-1,67	Tpm1	PSR0900021342.mm.1	3,54	0,020456	0,478018	Cassette Exon	0,26
TC0900002541.mm.1	-1,67	Tpm1	PSR0900021316.mm.1	3,04	0,005942	0,385164	Alternative 3' Acce	0,27
TC0900002541.mm.1	-1,67	Tpm1	PSR0900021336.mm.1	2,33	0,03219	0,518843	Intron Retention	0,26
TC0900002541.mm.1	-1,67	Tpm1	JUC0900011989.mm.1	2,04	0,039882	0,54093		
TC0700001253.mm.1	1,85	Grm5; C030032F19Rik	JUC0700005615.mm.1	3,54	0,031668	0,517405		
TC0700001253.mm.1	1,85	Grm5; C030032F19Rik	PSR0700010845.mm.1	2,32	0,010173	0,419711	Alternative 3' Acce	0,21
TC0700001253.mm.1	1,85	Grm5; C030032F19Rik	JUC0700005611.mm.1	-2,08	0,014582	0,449876		
TC0700001253.mm.1	1,85	Grm5; C030032F19Rik	PSR0700010858.mm.1	-2,23	0,015689	0,455416	Alternative 5' Donc	0,16
TC0700001253.mm.1	1,85	Grm5; C030032F19Rik	PSR0700010850.mm.1	-2,31	0,004627	0,369794	Cassette Exon	0,05
TC0700001253.mm.1	1,85	Grm5; C030032F19Rik	PSR0700010841.mm.1	-2,89	0,005544	0,380631	Cassette Exon	0,17
TC0700001253.mm.1	1,85	Grm5; C030032F19Rik	PSR0700010842.mm.1	-2,95	0,003135	0,353892	Cassette Exon	0,17

TC1000001826.mm.1	1,69	4930405J17Rik	PSR1000013290.mm.1	3,54	0,00131	0,323877	Cassette Exon	0,2
TC0800000932.mm.1	-1,11	Ddx39	JUC0800003660.mm.1	3,54	0,022591	0,487325		
TC0800000932.mm.1	-1,11	Ddx39	JUC0800003649.mm.1	2,46	0,002414	0,348907		
TC0700002692.mm.1	-1,1	Dll3	JUC0700012474.mm.1	3,54	0,013322	0,441836		
TC0X00000660.mm.1	-1,19	Prrg3	JUC0X00002096.mm.1	3,54	0,032116	0,518585		
TC0900002516.mm.1	1,26	Zfp609	JUC0900011870.mm.1	3,54	0,036137	0,529805		
TC0900002516.mm.1	1,26	Zfp609	JUC0900011872.mm.1	2,29	0,028482	0,50733		
TC0400001486.mm.1	1,25	Dlgap3	JUC0400005898.mm.1	3,54	0,021236	0,481319		
TC0200000319.mm.1	-1,43	Etl4	JUC0200000660.mm.1	3,53	0,003596	0,356223		
TC0200000319.mm.1	-1,43	Etl4	PSR0200001510.mm.1	3,18	0,017401	0,463753	Alternative 5' Donc	0,33
TC0200000319.mm.1	-1,43	Etl4	PSR0200001493.mm.1	2,48	0,012166	0,432998	Cassette Exon	0,25
TC0200000319.mm.1	-1,43	Etl4	PSR0200001505.mm.1	2,28	0,001688	0,335346	Cassette Exon	0,09
TC0200004356.mm.1	1,05	Ryr3	JUC0200018586.mm.1	3,53	0,018624	0,46991		
TC0200004356.mm.1	1,05	Ryr3	JUC0200018538.mm.1	3,47	0,007352	0,399281		
TC0200004356.mm.1	1,05	Ryr3	JUC0200018584.mm.1	2,57	0,023115	0,489756		
TC0200004356.mm.1	1,05	Ryr3	JUC0200018535.mm.1	2,02	0,007435	0,399934		
TC0200004356.mm.1	1,05	Ryr3	JUC0200018638.mm.1	-3,02	0,013783	0,444974		
TC0200004356.mm.1	1,05	Ryr3	JUC0200018575.mm.1	-3,06	0,027974	0,505748		
TC1700000055.mm.1	1,68	Tmem181a	JUC1700000220.mm.1	3,53	0,006036	0,386586		
TC1600001272.mm.1	1		JUC1600005368.mm.1	3,53	0,02367	0,491379		
TC0600002077.mm.1	-1,06	9330158H04Rik	JUC0600008535.mm.1	3,53	0,021295	0,481714		
TC0X00001800.mm.1	1,04	Porcn	JUC0X00005831.mm.1	3,53	0,004798	0,371408		
TC0700004607.mm.1	-1,3	Fadd	PSR0700039044.mm.1	3,52	0,046943	0,557545	Alternative 5' Donc	0,4
TC0900002991.mm.1	1,41	Atp2c1	JUC0900013826.mm.1	3,52	0,042893	0,54819		
TC0900002991.mm.1	1,41	Atp2c1	JUC0900013824.mm.1	3,11	0,026803	0,502337		
TC0900002991.mm.1	1,41	Atp2c1	JUC0900013813.mm.1	-2,52	0,017482	0,463964		
TC0900002991.mm.1	1,41	Atp2c1	PSR0900024677.mm.1	-2,93	0,027598	0,504485	Alternative 5' Donc	0,28
TC1700000888.mm.1	-1,42	Dlk2	PSR1700008461.mm.1	3,52	0,025496	0,497664	Cassette Exon	0,24
TC0900003268.mm.1	-1,56	Lyzl4	JUC0900015516.mm.1	3,52	0,007479	0,400381		
TC0900003268.mm.1	-1,56	Lyzl4	PSR0900027547.mm.1	2,31	0,026243	0,500211	Cassette Exon	0,11
TC0200004433.mm.1	-1,09	Ino80	JUC0200018967.mm.1	3,52	0,008798	0,409822		
TC0700001910.mm.1	1,08	Plekha1	PSR0700016718.mm.1	3,51	0,038541	0,536695	Alternative 5' Donc	0,39
TC0700003550.mm.1	-2,52	Gm26633	JUC0700015811.mm.1	3,51	0,042679	0,548097		
TC0700003550.mm.1	-2,52	Gm26633	PSR0700029979.mm.1	2,74	0,013934	0,446202	Cassette Exon	0,2
TC1000001946.mm.1	-1,93	Hint3	PSR1000013995.mm.1	3,5	0,014051	0,44654	Cassette Exon	0,22
TC1000001946.mm.1	-1,93	Hint3	PSR1000013989.mm.1	2,16	0,020124	0,47686	Cassette Exon	0,15
TC0200004537.mm.1	-1,24	Atp8b4	JUC0200020018.mm.1	3,5	0,026296	0,5006		
TC0200004729.mm.1	-1,2	Esf1	JUC0200020961.mm.1	3,5	0,00136	0,32572		
TC0500001808.mm.1	1,11	Pan3	PSR0500016734.mm.1	3,5	0,026495	0,501252		
TC0500001808.mm.1	1,11	Pan3	JUC0500009080.mm.1	2,38	0,018183	0,467441		
TC0800002634.mm.1	-1,41	N4bp1	JUC0800010996.mm.1	3,5	0,000134	0,270905		
TC0800002634.mm.1	-1,41	N4bp1	JUC0800010998.mm.1	2,08	0,029166	0,509333		
TC0800002634.mm.1	-1,41	N4bp1	JUC0800011004.mm.1	2,08	0,044287	0,551322		
TC0700000412.mm.1	4,97	Tmem145	JUC0700001561.mm.1	3,49	0,00052	0,303623		
TC0700000412.mm.1	4,97	Tmem145	PSR0700003163.mm.1	3,09	0,002702	0,349612	Cassette Exon	0,08
TC0700000412.mm.1	4,97	Tmem145	PSR0700003157.mm.1	2,05	0,006513	0,391376	Cassette Exon	0,12
TC0700000412.mm.1	4,97	Tmem145	PSR0700003173.mm.1	-2,99	0,011192	0,426444	Cassette Exon	0,23
TC0700000412.mm.1	4,97	Tmem145	JUC0700001559.mm.1	-3,36	0,002126	0,342919		

TC0700000412.mm.1	4,97	Tmem145	PSR0700003156.mm.1	-3,98	0,017647	0,464582	Cassette Exon	0,24
TC0700000412.mm.1	4,97	Tmem145	PSR0700003174.mm.1	-4,04	0,008416	0,406611	Cassette Exon	0,28
TC0700000412.mm.1	4,97	Tmem145	PSR0700003160.mm.1	-4,35	0,013748	0,444758	Intron Retention	0,5
TC0700000412.mm.1	4,97	Tmem145	JUC0700001565.mm.1	-5,57	0,045311	0,553742		
TC0700000412.mm.1	4,97	Tmem145	JUC0700001557.mm.1	-5,58	0,005107	0,376064		
TC0700000412.mm.1	4,97	Tmem145	JUC0700001558.mm.1	-7,65	0,003771	0,357586		
TC0700000412.mm.1	4,97	Tmem145	JUC0700001554.mm.1	-20,93	0,016524	0,459611		
TC1100003644.mm.1	2,74	Gpr179	JUC1100017528.mm.1	3,49	0,022906	0,488524		
TC1100003644.mm.1	2,74	Gpr179	JUC1100017530.mm.1	3,14	0,00863	0,408463		
TC1100003644.mm.1	2,74	Gpr179	PSR1100033486.mm.1	2,52	0,001567	0,331411	Cassette Exon	0,2
TC1100003644.mm.1	2,74	Gpr179	PSR1100033483.mm.1	2,05	0,015688	0,455416	Cassette Exon	0,08
TC1100003644.mm.1	2,74	Gpr179	JUC1100017526.mm.1	2,02	0,008932	0,41101		
TC1100003644.mm.1	2,74	Gpr179	JUC1100017532.mm.1	-2,28	0,015912	0,456594		
TC1100003644.mm.1	2,74	Gpr179	JUC1100017529.mm.1	-2,67	0,049003	0,561874		
TC1100003644.mm.1	2,74	Gpr179	PSR1100033484.mm.1	-3	0,002647	0,349612	Cassette Exon	0,18
TC1100003644.mm.1	2,74	Gpr179	PSR1100033492.mm.1	-3,39	0,003592	0,356223	Cassette Exon	0,32
TC1100003644.mm.1	2,74	Gpr179	PSR1100033495.mm.1	-4,27	0,000356	0,290838	Alternative 5' Donc	0,44
TC1100003644.mm.1	2,74	Gpr179	JUC1100017531.mm.1	-5,37	0,007422	0,399901		
TC0800000300.mm.1	1,68	Zmat4	JUC0800001240.mm.1	3,49	0,009116	0,413187		
TC0800000300.mm.1	1,68	Zmat4	PSR0800002280.mm.1	-2,43	0,013353	0,442045	Cassette Exon	0,13
TC0800000300.mm.1	1,68	Zmat4	PSR0800002279.mm.1	-2,63	0,008978	0,411895	Cassette Exon	0,06
TC0800000300.mm.1	1,68	Zmat4	PSR0800002295.mm.1	-3,52	0,012788	0,437236	Cassette Exon	0,23
TC0800000300.mm.1	1,68	Zmat4	JUC0800001243.mm.1	-3,69	0,027297	0,503732		
TC0800000300.mm.1	1,68	Zmat4	PSR0800002275.mm.1	-3,8	0,005115	0,376246	Cassette Exon	0,32
TC0800000300.mm.1	1,68	Zmat4	JUC0800001248.mm.1	-5,92	0,041552	0,544924		
TC0900002106.mm.1	-1,38	Ubash3b	PSR0900017403.mm.1	3,49	0,020345	0,477323	Cassette Exon	0,25
TC0900002106.mm.1	-1,38	Ubash3b	PSR0900017397.mm.1	2,63	0,013619	0,444002	Cassette Exon	0,17
TC0900002106.mm.1	-1,38	Ubash3b	PSR0900017396.mm.1	2,39	0,025729	0,498173	Cassette Exon	0,14
TC0300001476.mm.1	-1,12	Col24a1	PSR0300011870.mm.1	3,49	0,045287	0,553724	Cassette Exon	0,23
TC0200004963.mm.1	1,54	Ggt7	PSR0200042119.mm.1	3,49	0,034258	0,525276	Cassette Exon	0,22
TC0200004963.mm.1	1,54	Ggt7	PSR0200042105.mm.1	-2,16	0,010512	0,422115	Cassette Exon	0,11
TC0300000078.mm.1	1,79	Ralyl	JUC0300000115.mm.1	3,49	0,014789	0,450876		
TC0300000078.mm.1	1,79	Ralyl	PSR0300000292.mm.1	3,08	0,005756	0,383101	Cassette Exon	0,19
TC0300000078.mm.1	1,79	Ralyl	PSR0300000296.mm.1	-2,03	0,003806	0,358103	Cassette Exon	0,06
TC0300000078.mm.1	1,79	Ralyl	JUC0300000113.mm.1	-2,09	0,001376	0,32572		
TC0300000078.mm.1	1,79	Ralyl	PSR0300000290.mm.1	-2,11	0,040322	0,541841	Alternative 5' Donc	0,15
TC0300000078.mm.1	1,79	Ralyl	PSR0300000285.mm.1	-2,12	0,015772	0,455535		
TC0300000078.mm.1	1,79	Ralyl	PSR0300000294.mm.1	-2,36	0,002798	0,352011	Alternative 3' Acce	0,09
TC0300000078.mm.1	1,79	Ralyl	JUC0300000116.mm.1	-3,16	0,008174	0,405614		
TC0300000078.mm.1	1,79	Ralyl	JUC0300000109.mm.1	-3,25	0,006181	0,387873		
TC1100003783.mm.1	-1,08	Kcnh4	JUC1100018253.mm.1	3,49	0,023021	0,489186		
TC1100003783.mm.1	-1,08	Kcnh4	JUC1100018257.mm.1	-2,31	0,000301	0,288663		
TC1400002635.mm.1	-1	Mycbp2	JUC1400010884.mm.1	3,49	0,043354	0,549429		
TC1400002635.mm.1	-1	Mycbp2	JUC1400010844.mm.1	2,54	0,023621	0,491012		
TC1400002635.mm.1	-1	Mycbp2	JUC1400010877.mm.1	2,02	0,00105	0,316361		
TC1100003691.mm.1	-1,23		JUC1100017803.mm.1	3,49	0,031541	0,517122		
TC1000000479.mm.1	-2,15	Pln	JUC1000001755.mm.1	3,49	0,049651	0,563521		
TC1600000745.mm.1	6,86	Impg2	PSR1600006441.mm.1	3,48	0,019513	0,474538	Mutually Exclusive	0,45

TC1600000745.mm.1	6,86	Impg2	PSR1600006444.mm.1	2,69	0,012205	0,433268	Cassette Exon	0,15
TC1600000745.mm.1	6,86	Impg2	JUC1600003404.mm.1	-2,18	0,037199	0,533261		
TC1600000745.mm.1	6,86	Impg2	PSR1600006461.mm.1	-2,5	0,007783	0,402902	Alternative 5' Donc	0,23
TC1600000745.mm.1	6,86	Impg2	PSR1600006440.mm.1	-3,81	0,004359	0,365487	Mutually Exclusive	0,45
TC1600000745.mm.1	6,86	Impg2	JUC1600003406.mm.1	-4,87	0,003185	0,354243		
TC1600000745.mm.1	6,86	Impg2	JUC1600003415.mm.1	-7,99	0,005033	0,374447		
TC1600000745.mm.1	6,86	Impg2	PSR1600006459.mm.1	-8,5	0,000614	0,304044	Cassette Exon	0,44
TC1600000745.mm.1	6,86	Impg2	JUC1600003414.mm.1	-10,83	0,002821	0,352207		
TC1600000745.mm.1	6,86	Impg2	JUC1600003409.mm.1	-13,04	0,000612	0,304044		
TC0300001227.mm.1	-1,94		PSR0300009936.mm.1	3,48	0,048488	0,560864	Cassette Exon	0,23
TC0300001227.mm.1	-1,94		PSR0300009935.mm.1	-3,48	0,048488	0,560864	Cassette Exon	0,23
TC1200001509.mm.1	1,32	2410018L13Rik; 9030624(	PSR1200010561.mm.1	3,48	0,01025	0,420147	Cassette Exon	0,14
TC1200001509.mm.1	1,32	2410018L13Rik; 9030624(	PSR1200010564.mm.1	2,49	0,003929	0,359818	Cassette Exon	0,2
TC1900000420.mm.1	-1,12		JUC1900002033.mm.1	3,48	0,01409	0,446971		
TC0200000302.mm.1	1,05	Bmi1	JUC0200000592.mm.1	3,48	0,003104	0,353892		
TC0700002483.mm.1	1,1	Mark4	JUC0700011645.mm.1	3,48	0,012846	0,437777		
TC0700002483.mm.1	1,1	Mark4	JUC0700011649.mm.1	-2,95	0,030144	0,512049		
TC0700002901.mm.1	1,09	A230077H06Rik	PSR0700025832.mm.1	3,47	0,003532	0,356184	Alternative 3' Acce	0,38
TC1100003855.mm.1	-1,11	Eftud2	PSR1100035963.mm.1	3,47	0,034692	0,526294	Alternative 5' Donc	0,38
TC0800000930.mm.1	-2,18	Gipc1	PSR0800006866.mm.1	3,47	0,033212	0,522175	Cassette Exon	0,32
TC0800000930.mm.1	-2,18	Gipc1	JUC0800003641.mm.1	3,13	0,025716	0,498173		
TC0800000930.mm.1	-2,18	Gipc1	PSR0800006868.mm.1	2,57	0,029703	0,51095	Cassette Exon	0,22
TC1000002238.mm.1	-1,56	Gm20610; Gm20625	JUC1000008597.mm.1	3,47	0,000229	0,28803		
TC1000002238.mm.1	-1,56	Gm20610; Gm20625	PSR1000015741.mm.1	2,18	0,042842	0,548097	Cassette Exon	0,09
TC0300000228.mm.1	-1	Usp13	JUC0300000705.mm.1	3,47	0,010664	0,423012		
TC0200004896.mm.1	1,03	Trib3	JUC0200021501.mm.1	3,47	0,004091	0,36228		
TC0200004896.mm.1	1,03	Trib3	JUC0200021500.mm.1	2,25	0,01385	0,445398		
TC0300002295.mm.1	1,07	Bcan	JUC0300009125.mm.1	3,47	0,025271	0,497004		
TC0900001885.mm.1	-1,31	Dock6	JUC0900008937.mm.1	3,47	0,006441	0,390159		
TC0900001885.mm.1	-1,31	Dock6	JUC0900008969.mm.1	2,26	0,036551	0,531355		
TC0700000023.mm.1	4,94	Ttyh1	JUC0700000139.mm.1	3,46	0,00033	0,28927		
TC0700000023.mm.1	4,94	Ttyh1	JUC0700000137.mm.1	2,98	0,004635	0,370096		
TC0700000023.mm.1	4,94	Ttyh1	PSR0700000307.mm.1	2,65	0,00275	0,35008	Cassette Exon	0,18
TC0700000023.mm.1	4,94	Ttyh1	PSR0700000324.mm.1	2,1	0,001861	0,337823	Cassette Exon	0,34
TC0700000023.mm.1	4,94	Ttyh1	JUC0700000140.mm.1	2,07	0,002653	0,349612		
TC0700000023.mm.1	4,94	Ttyh1	JUC0700000144.mm.1	-2,03	0,046231	0,556083		
TC0700000023.mm.1	4,94	Ttyh1	PSR0700000292.mm.1	-2,61	0,004151	0,3623	Alternative 3' Acce	0,35
TC0700000023.mm.1	4,94	Ttyh1	PSR0700000332.mm.1	-2,92	0,027035	0,502979	Alternative 5' Donc	0,38
TC0700000023.mm.1	4,94	Ttyh1	PSR0700000302.mm.1	-3,05	0,001624	0,332831	Cassette Exon	0,23
TC0700000023.mm.1	4,94	Ttyh1	PSR0700000309.mm.1	-3,1	0,015543	0,454617	Alternative 5' Donc	0,34
TC0700000023.mm.1	4,94	Ttyh1	PSR0700000300.mm.1	-3,15	0,007692	0,402552	Cassette Exon	0,3
TC0700000023.mm.1	4,94	Ttyh1	JUC0700000130.mm.1	-3,23	0,005545	0,380631		
TC0700000023.mm.1	4,94	Ttyh1	PSR0700000306.mm.1	-3,46	0,013098	0,439759	Alternative 3' Acce	0,4
TC0700000023.mm.1	4,94	Ttyh1	PSR0700000295.mm.1	-4,13	0,028755	0,508148	Alternative 3' Acce	0,34
TC0700000023.mm.1	4,94	Ttyh1	PSR0700000322.mm.1	-4,4	0,000614	0,304044	Intron Retention	0,75
TC0700000023.mm.1	4,94	Ttyh1	PSR0700000291.mm.1	-4,7	0,004262	0,363802	Alternative 3' Acce	0,35
TC0700000023.mm.1	4,94	Ttyh1	PSR0700000316.mm.1	-4,74	0,009879	0,417993	Alternative 5' Donc	0,38
TC0700000023.mm.1	4,94	Ttyh1	JUC0700000138.mm.1	-4,8	0,009532	0,416602		

TC0700000023.mm.1	4,94	Ttyh1	JUC0700000146.mm.1	-5,31	0,003249	0,354243		
TC0700000023.mm.1	4,94	Ttyh1	PSR0700000313.mm.1	-5,36	0,036101	0,529729	Cassette Exon	0,32
TC0700000023.mm.1	4,94	Ttyh1	PSR0700000333.mm.1	-5,5	0,009671	0,417554	Alternative 5' Donc	0,38
TC0700000023.mm.1	4,94	Ttyh1	JUC0700000142.mm.1	-6,13	0,002188	0,344463		
TC0700000023.mm.1	4,94	Ttyh1	PSR0700000320.mm.1	-6,97	0,009716	0,417826	Intron Retention	0,75
TC0700000023.mm.1	4,94	Ttyh1	JUC0700000145.mm.1	-8,65	0,003167	0,353901		
TC0100001114.mm.1	1,7	Mgat5	JUC0100005242.mm.1	3,46	0,003215	0,354243		
TC0100001114.mm.1	1,7	Mgat5	JUC0100005237.mm.1	2,43	0,001708	0,335996		
TC0100001114.mm.1	1,7	Mgat5	PSR0100009065.mm.1	-2,02	0,018648	0,470078	Alternative 5' Donc	0,16
TC0100001114.mm.1	1,7	Mgat5	PSR0100009054.mm.1	-3	0,018583	0,469618	Alternative 5' Donc	0,32
TC0400001538.mm.1	1,51	Bai2	PSR0400011986.mm.1	3,46	0,016977	0,461419	Cassette Exon	0,26
TC0400001538.mm.1	1,51	Bai2	JUC0400006236.mm.1	2,88	0,030066	0,511856		
TC0400001538.mm.1	1,51	Bai2	JUC0400006234.mm.1	2,3	0,043642	0,550199		
TC0400001538.mm.1	1,51	Bai2	PSR0400011966.mm.1	2,15	0,028012	0,505851	Cassette Exon	0,1
TC0400001538.mm.1	1,51	Bai2	PSR0400011996.mm.1	2,06	0,0297	0,510949	Cassette Exon	0,18
TC0400001538.mm.1	1,51	Bai2	PSR0400011988.mm.1	-2,09	0,021216	0,481269	Cassette Exon	0,1
TC0400001538.mm.1	1,51	Bai2	PSR0400012005.mm.1	-2,13	0,002997	0,353111	Alternative 5' Donc	0,17
TC0400001538.mm.1	1,51	Bai2	JUC0400006245.mm.1	-2,39	0,033192	0,522148		
TC1400001656.mm.1	1,22	Cacna1d	PSR1400012450.mm.1	3,46	0,035726	0,529027	Cassette Exon	0,25
TC1400001656.mm.1	1,22	Cacna1d	JUC1400006750.mm.1	3,13	0,001628	0,332831		
TC1400001656.mm.1	1,22	Cacna1d	JUC1400006753.mm.1	2,53	0,005591	0,381035		
TC1400001656.mm.1	1,22	Cacna1d	PSR1400012476.mm.1	-2,45	0,00655	0,39213	Cassette Exon	0,14
TC0Y00000209.mm.1	-1,41	Gm21095; Gm21627; Gm2	JUC0Y00000819.mm.1	3,46	0,018787	0,470789		
TC0Y00000209.mm.1	-1,41	Gm21095; Gm21627; Gm2	PSR0Y00001124.mm.1	-2,38	0,026281	0,50053	Cassette Exon	0,12
TC0800000111.mm.1	1,53	Atp11a	JUC0800000533.mm.1	3,46	0,033939	0,524252		
TC0800000111.mm.1	1,53	Atp11a	PSR0800000936.mm.1	-2,02	0,024012	0,492783	Alternative 5' Donc	0,11
TC0300001446.mm.1	-1,85	Gbp3	JUC0300006124.mm.1	3,46	0,006114	0,387268		
TC0300001446.mm.1	-1,85	Gbp3	JUC0300006130.mm.1	2,33	0,007649	0,402219		
TC0300001446.mm.1	-1,85	Gbp3	PSR0300011649.mm.1	2,26	0,015621	0,45509	Cassette Exon	0,09
TC1500000921.mm.1	-1,07	Pphln1	JUC1500004163.mm.1	3,46	0,003145	0,353892		
TC1500000921.mm.1	-1,07	Pphln1	PSR1500007348.mm.1	2,01	0,000857	0,311909	Cassette Exon	0,08
TC0700004296.mm.1	1,39	Aldoa	PSR0700035952.mm.1	3,45	0,004867	0,371692	Alternative 3' Acce	0,38
TC0700004296.mm.1	1,39	Aldoa	PSR0700035974.mm.1	-2,25	0,031585	0,517177	Cassette Exon	0,16
TC1900001509.mm.1	1,38	Slit1	PSR1900013246.mm.1	3,45	0,000813	0,311886	Cassette Exon	0,23
TC1900001509.mm.1	1,38	Slit1	PSR1900013247.mm.1	3,29	0,021648	0,483748	Cassette Exon	0,29
TC1900001509.mm.1	1,38	Slit1	JUC1900007303.mm.1	3,26	0,009221	0,41411		
TC1900001509.mm.1	1,38	Slit1	JUC1900007318.mm.1	2,74	0,038119	0,535709		
TC1900001509.mm.1	1,38	Slit1	JUC1900007308.mm.1	2,55	0,036545	0,531355		
TC0X00001545.mm.1	1,75	3010001F23Rik	PSR0X00009849.mm.1	3,45	0,00501	0,373851	Cassette Exon	0,23
TC0X00001545.mm.1	1,75	3010001F23Rik	JUC0X00004965.mm.1	-2,46	0,045271	0,553703		
TC0100000201.mm.1	1,26	Khdrbs2	JUC0100000620.mm.1	3,45	0,007859	0,40351		
TC0100000201.mm.1	1,26	Khdrbs2	JUC0100000623.mm.1	3,05	0,022293	0,486095		
TC0100000201.mm.1	1,26	Khdrbs2	PSR0100001192.mm.1	2,12	0,037599	0,534053	Cassette Exon	0,22
TC1500000353.mm.1	-1,24	Pkhd1l1	JUC1500001410.mm.1	3,45	0,022458	0,487003		
TC1500001053.mm.1	-1,35		JUC1500004703.mm.1	3,45	0,007873	0,403545		
TC0900001385.mm.1	2,78	Camkv	JUC0900006014.mm.1	3,44	0,000115	0,267977		
TC0900001385.mm.1	2,78	Camkv	JUC0900006016.mm.1	3,39	0,028017	0,505851		
TC0900001385.mm.1	2,78	Camkv	PSR0900010935.mm.1	2,94	0,000236	0,28803	Cassette Exon	0,28

TC0900001385.mm.1	2,78	Camkv	PSR0900010930.mm.1	2,24	0,003051	0,353892	Cassette Exon	0,15
TC0900001385.mm.1	2,78	Camkv	PSR0900010938.mm.1	2,07	0,000981	0,31497	Cassette Exon	0,22
TC0900001385.mm.1	2,78	Camkv	PSR0900010939.mm.1	-2,19	0,008448	0,406611	Cassette Exon	0,1
TC0900001385.mm.1	2,78	Camkv	JUC0900006013.mm.1	-2,55	0,036729	0,531779		
TC0900001385.mm.1	2,78	Camkv	PSR0900010929.mm.1	-2,8	0,01357	0,443703	Cassette Exon	0,17
TC0900001385.mm.1	2,78	Camkv	PSR0900010933.mm.1	-3,24	0,014456	0,44909	Intron Retention	0,54
TC0600003316.mm.1	3,81	Gsg1	JUC0600013564.mm.1	3,44	0,003297	0,354243		
TC0600003316.mm.1	3,81	Gsg1	JUC0600013565.mm.1	3,42	0,030665	0,513807		
TC0600003316.mm.1	3,81	Gsg1	PSR0600025997.mm.1	2,93	0,00386	0,358861	Cassette Exon	0,25
TC0600003316.mm.1	3,81	Gsg1	PSR0600025996.mm.1	2,12	0,016791	0,460623	Cassette Exon	0,32
TC0600003316.mm.1	3,81	Gsg1	JUC0600013569.mm.1	-2,58	0,010626	0,422841		
TC0600003316.mm.1	3,81	Gsg1	PSR0600026002.mm.1	-2,83	0,001395	0,325997	Cassette Exon	0,02
TC0600003316.mm.1	3,81	Gsg1	PSR0600026001.mm.1	-3,09	0,026827	0,502373	Cassette Exon	0,04
TC0600003316.mm.1	3,81	Gsg1	PSR0600026004.mm.1	-4,41	0,007784	0,402902	Cassette Exon	0,28
TC0600003316.mm.1	3,81	Gsg1	PSR0600026005.mm.1	-4,48	0,008906	0,410728	Cassette Exon	0,28
TC0600003316.mm.1	3,81	Gsg1	JUC0600013571.mm.1	-4,67	0,012748	0,436821		
TC0600003316.mm.1	3,81	Gsg1	PSR0600026003.mm.1	-6,09	0,002785	0,35133	Cassette Exon	0,41
TC1100003665.mm.1	2,73	Stac2	JUC1100017678.mm.1	3,44	0,006496	0,390944		
TC1100003665.mm.1	2,73	Stac2	PSR1100033737.mm.1	2,34	0,00541	0,379167	Cassette Exon	0,05
TC1100003665.mm.1	2,73	Stac2	PSR1100033738.mm.1	-2,36	0,031003	0,514829	Cassette Exon	0,2
TC1100003665.mm.1	2,73	Stac2	JUC1100017674.mm.1	-2,57	0,011302	0,427133		
TC1100003665.mm.1	2,73	Stac2	PSR1100033748.mm.1	-2,58	0,033576	0,523185	Alternative 5' Donc	0,32
TC1100003665.mm.1	2,73	Stac2	JUC1100017671.mm.1	-2,72	0,018363	0,468484		
TC1100003665.mm.1	2,73	Stac2	PSR1100033739.mm.1	-2,74	0,015202	0,452738	Cassette Exon	0,3
TC1100003665.mm.1	2,73	Stac2	JUC1100017676.mm.1	-3,15	0,000083	0,252976		
TC1100003665.mm.1	2,73	Stac2	PSR1100033749.mm.1	-3,37	0,019536	0,474538	Alternative 5' Donc	0,32
TC1100003665.mm.1	2,73	Stac2	JUC1100017680.mm.1	-4,16	0,000712	0,306015		
TC0200000492.mm.1	-1,07	Ak8	JUC0200001727.mm.1	3,44	0,00833	0,406336		
TC0X00001005.mm.1	-1,24	Gm9126	JUC0X00003382.mm.1	3,44	0,019993	0,476227		
TC0X00002417.mm.1	-1,08	Xlr4a	JUC0X00007584.mm.1	3,44	0,018273	0,468043		
TC0200002835.mm.1	2	Myt1	JUC0200011905.mm.1	3,43	0,002074	0,342181		
TC0200002835.mm.1	2	Myt1	JUC0200011897.mm.1	3,33	0,009302	0,414883		
TC0200002835.mm.1	2	Myt1	PSR0200023525.mm.1	3,06	0,001123	0,317328	Alternative 5' Donc	0,3
TC0200002835.mm.1	2	Myt1	JUC0200011917.mm.1	2,33	0,005813	0,383104		
TC0200002835.mm.1	2	Myt1	PSR0200023495.mm.1	2,26	0,009948	0,41841	Alternative 3' Acce	0,2
TC0200002835.mm.1	2	Myt1	JUC0200011906.mm.1	2,14	0,035786	0,529153		
TC0200002835.mm.1	2	Myt1	PSR0200023496.mm.1	-2,01	0,006884	0,39438	Cassette Exon	0,09
TC0200002835.mm.1	2	Myt1	PSR0200023519.mm.1	-2,31	0,00265	0,349612	Cassette Exon	0,17
TC0200002835.mm.1	2	Myt1	JUC0200011908.mm.1	-2,32	0,004275	0,363868		
TC0200002835.mm.1	2	Myt1	JUC0200011911.mm.1	-2,37	0,04607	0,555658		
TC0200002835.mm.1	2	Myt1	PSR0200023494.mm.1	-2,39	0,000128	0,270905	Cassette Exon	0,22
TC0200002835.mm.1	2	Myt1	PSR0200023484.mm.1	-2,46	0,038212	0,535805	Cassette Exon	0,14
TC0200002835.mm.1	2	Myt1	JUC0200011920.mm.1	-2,54	0,028312	0,506741		
TC0200002835.mm.1	2	Myt1	PSR0200023490.mm.1	-2,64	0,009564	0,416927		
TC0200002835.mm.1	2	Myt1	PSR0200023487.mm.1	-3,03	0,011142	0,426226	Alternative 3' Acce	0,06
TC0200002835.mm.1	2	Myt1	JUC0200011899.mm.1	-3,07	0,019435	0,473998		
TC0200002835.mm.1	2	Myt1	JUC0200011926.mm.1	-3,13	0,003109	0,353892		
TC0200002835.mm.1	2	Myt1	PSR0200023518.mm.1	-3,17	0,003667	0,357266	Cassette Exon	0,24

TC0200002835.mm.1	2	Myt1	JUC0200011924.mm.1	-3,31	0,033613	0,523309		
TC0200002835.mm.1	2	Myt1	PSR0200023513.mm.1	-3,65	0,007084	0,396891	Alternative 5' Donc	0,52
TC0200002835.mm.1	2	Myt1	JUC0200011909.mm.1	-6,42	0,002126	0,342919		
TC0200004108.mm.1	2,51	Madd	JUC0200017542.mm.1	3,43	0,002922	0,352289		
TC0200004108.mm.1	2,51	Madd	PSR0200034324.mm.1	2,14	0,033066	0,521667	Cassette Exon	0,1
TC0200004108.mm.1	2,51	Madd	PSR0200034359.mm.1	-2,03	0,014928	0,451919	Alternative 5' Donc	0,2
TC0200004108.mm.1	2,51	Madd	PSR0200034316.mm.1	-2,05	0,007202	0,398011	Alternative 5' Donc	0,12
TC0200004108.mm.1	2,51	Madd	PSR0200034394.mm.1	-2,07	0,015922	0,456718	Cassette Exon	0,14
TC0200004108.mm.1	2,51	Madd	JUC0200017573.mm.1	-2,09	0,009849	0,417839		
TC0200004108.mm.1	2,51	Madd	PSR0200034386.mm.1	-2,2	0,008361	0,406435	Cassette Exon	0,14
TC0200004108.mm.1	2,51	Madd	JUC0200017533.mm.1	-2,24	0,015612	0,455082		
TC0200004108.mm.1	2,51	Madd	PSR0200034384.mm.1	-2,25	0,014499	0,449528	Cassette Exon	0,14
TC0200004108.mm.1	2,51	Madd	JUC0200017551.mm.1	-2,28	0,041163	0,544039		
TC0200004108.mm.1	2,51	Madd	PSR0200034377.mm.1	-2,42	0,00934	0,415075	Alternative 3' Acce	0,23
TC0200004108.mm.1	2,51	Madd	PSR0200034326.mm.1	-2,59	0,024493	0,493992	Alternative 3' Acce	0,28
TC0200004108.mm.1	2,51	Madd	JUC0200017561.mm.1	-2,61	0,020835	0,479521		
TC0200004108.mm.1	2,51	Madd	PSR0200034322.mm.1	-2,75	0,015512	0,454456	Alternative 3' Acce	0,32
TC0200004108.mm.1	2,51	Madd	PSR0200034393.mm.1	-2,79	0,01283	0,437626	Cassette Exon	0,14
TC0200004108.mm.1	2,51	Madd	PSR0200034396.mm.1	-2,99	0,041332	0,544314	Alternative 5' Donc	0,15
TC0200004108.mm.1	2,51	Madd	PSR0200034338.mm.1	-3,01	0,003588	0,356223	Intron Retention	0,5
TC0200004108.mm.1	2,51	Madd	PSR0200034323.mm.1	-3,06	0,010633	0,422841	Alternative 3' Acce	0,34
TC0200004108.mm.1	2,51	Madd	JUC0200017550.mm.1	-3,12	0,002263	0,34578		
TC0200004108.mm.1	2,51	Madd	JUC0200017562.mm.1	-3,17	0,010515	0,422183		
TC0200004108.mm.1	2,51	Madd	JUC0200017546.mm.1	-3,22	0,00268	0,349612		
TC0200004108.mm.1	2,51	Madd	JUC0200017539.mm.1	-5,06	0,020306	0,477245		
TC1300000482.mm.1	1,58	Phactr1	JUC1300001504.mm.1	3,43	0,008349	0,406435		
TC1300000482.mm.1	1,58	Phactr1	PSR1300002917.mm.1	-2,27	0,007707	0,402552	Intron Retention	0,28
TC1300000482.mm.1	1,58	Phactr1	PSR1300002888.mm.1	-2,52	0,042748	0,548097	Cassette Exon	0,16
TC1300000482.mm.1	1,58	Phactr1	PSR1300002890.mm.1	-2,77	0,002742	0,349612	Alternative 3' Acce	0,29
TC1300000482.mm.1	1,58	Phactr1	JUC1300001513.mm.1	-5,35	0,015374	0,453849		
TC1000002586.mm.1	-1,31	Appl2	JUC1000010667.mm.1	3,43	0,00101	0,316361		
TC1000002586.mm.1	-1,31	Appl2	PSR1000019535.mm.1	-2,66	0,034943	0,52717	Alternative 3' Acce	0,26
TC0100001447.mm.1	1,04	Tnr	JUC0100006801.mm.1	3,43	0,021912	0,484828		
TC0100001447.mm.1	1,04	Tnr	JUC0100006800.mm.1	2,68	0,006206	0,387873		
TC0100001447.mm.1	1,04	Tnr	JUC0100006807.mm.1	2,12	0,03382	0,523933		
TC0200002277.mm.1	1,17	Csnk2a1	JUC0200008952.mm.1	3,43	0,045925	0,555257		
TC0800001566.mm.1	-1,32	Ntpcr	JUC0800007013.mm.1	3,43	0,043692	0,550248		
TC0700001942.mm.1	1,52	Zranb1	JUC0700008989.mm.1	3,43	0,030385	0,512923		
TC0700002045.mm.1	3,54	Drd4	JUC0700009595.mm.1	3,43	0,023542	0,490947		
TC1000000240.mm.1	-1,19	Arhgap18	JUC1000000904.mm.1	3,43	0,016295	0,458465		
TC0200002077.mm.1	13,12	Snap25	JUC0200008338.mm.1	3,42	0,00106	0,316361		
TC0200002077.mm.1	13,12	Snap25	PSR0200016535.mm.1	2,77	0,007268	0,398281	Cassette Exon	0,17
TC0200002077.mm.1	13,12	Snap25	PSR0200016537.mm.1	2,03	0,023211	0,490032	Cassette Exon	0,12
TC0200002077.mm.1	13,12	Snap25	PSR0200016538.mm.1	-4,28	0,047922	0,559453	Alternative 5' Donc	0,51
TC0300002785.mm.1	-1,16	Amy2a1	PSR0300021920.mm.1	3,42	0,035642	0,528956	Cassette Exon	0,22
TC1100003128.mm.1	-1,48	Nlrp1c-ps; Nlrp1c	JUC1100015187.mm.1	3,42	0,044534	0,551894		
TC1100003128.mm.1	-1,48	Nlrp1c-ps; Nlrp1c	PSR1100028999.mm.1	2,19	0,000533	0,304044	Cassette Exon	0,22
TC1100003932.mm.1	-1,28	Pecam1	JUC1100019272.mm.1	3,42	0,008423	0,406611		

TC1100003932.mm.1	-1,28	Pecam1	PSR1100036859.mm.1	2,3	0,020725	0,479166	Cassette Exon	0,14
TC1100003932.mm.1	-1,28	Pecam1	PSR1100036879.mm.1	2,14	0,005559	0,380773	Cassette Exon	0,12
TC1100003932.mm.1	-1,28	Pecam1	JUC1100019269.mm.1	2,05	0,035458	0,528724		
TC1600000150.mm.1	-1,42	Mkl2	JUC1600000562.mm.1	3,42	0,038774	0,537463		
TC1600000150.mm.1	-1,42	Mkl2	JUC1600000543.mm.1	2,29	0,002495	0,349135		
TC0600000006.mm.1	5,46	Gngt1	JUC0600000041.mm.1	3,41	0,000279	0,28803		
TC0600000006.mm.1	5,46	Gngt1	JUC0600000042.mm.1	3,3	0,002009	0,341091		
TC0600000006.mm.1	5,46	Gngt1	PSR0600000074.mm.1	-2,12	0,026824	0,502373	Alternative 5' Donc	0,18
TC0600000006.mm.1	5,46	Gngt1	PSR0600000068.mm.1	-5,66	0,005673	0,382036	Alternative 3' Acce	0,48
TC0500002931.mm.1	-1,83	4930428O21Rik	PSR0500025899.mm.1	3,41	0,010071	0,418974	Alternative 5' Donc	0,36
TC0800001509.mm.1	1,22	Cpne7; Mir7080; mmu-mi	JUC0800006715.mm.1	3,41	0,004904	0,372548		
TC0800001509.mm.1	1,22	Cpne7; Mir7080; mmu-mi	JUC0800006719.mm.1	3,09	0,026994	0,502864		
TC0800001509.mm.1	1,22	Cpne7; Mir7080; mmu-mi	PSR0800012330.mm.1	2,09	0,024225	0,493294	Cassette Exon	0,1
TC0800001509.mm.1	1,22	Cpne7; Mir7080; mmu-mi	PSR0800012329.mm.1	-2,28	0,000473	0,298999	Intron Retention	0,32
TC0800001509.mm.1	1,22	Cpne7; Mir7080; mmu-mi	JUC0800006716.mm.1	-2,92	0,013271	0,441614		
TC1100003538.mm.1	-2,76	Tom1l1	PSR1100032314.mm.1	3,41	0,006138	0,387543	Cassette Exon	0,31
TC1100003538.mm.1	-2,76	Tom1l1	JUC1100016883.mm.1	3,2	0,009386	0,415457		
TC1100003538.mm.1	-2,76	Tom1l1	PSR1100032310.mm.1	3,02	0,019403	0,473802	Cassette Exon	0,22
TC1100003538.mm.1	-2,76	Tom1l1	JUC1100016895.mm.1	2,85	0,017963	0,466771		
TC1100003538.mm.1	-2,76	Tom1l1	PSR1100032308.mm.1	2,78	0,004488	0,367995	Cassette Exon	0,19
TC1100003538.mm.1	-2,76	Tom1l1	PSR1100032318.mm.1	2,72	0,009483	0,416578	Cassette Exon	0,16
TC1100003538.mm.1	-2,76	Tom1l1	JUC1100016892.mm.1	2,64	0,00058	0,304044		
TC1100003538.mm.1	-2,76	Tom1l1	PSR1100032303.mm.1	2,39	0,007269	0,398281	Alternative 5' Donc	0,11
TC1100003538.mm.1	-2,76	Tom1l1	PSR1100032287.mm.1	2,27	0,022224	0,485896	Cassette Exon	0,13
TC1100003538.mm.1	-2,76	Tom1l1	JUC1100016898.mm.1	2,27	0,0243	0,49362		
TC0700004444.mm.1	1,87	Fam196a	PSR0700037341.mm.1	3,41	0,004814	0,371408	Cassette Exon	0,22
TC0700004444.mm.1	1,87	Fam196a	JUC0700019673.mm.1	-3,85	0,011578	0,429122		
TC1600000529.mm.1	1,31	Hcls1	PSR1600004994.mm.1	3,41	0,011003	0,425491	Cassette Exon	0,22
TC1600000529.mm.1	1,31	Hcls1	JUC1600002637.mm.1	-4,97	0,023008	0,48911		
TC0400003832.mm.1	-1,56	Pla2g5	PSR0400031604.mm.1	3,41	0,048137	0,559938	Cassette Exon	0,21
TC0600003321.mm.1	1,51	Grin2b	JUC0600013580.mm.1	3,41	0,017611	0,464312		
TC0600003321.mm.1	1,51	Grin2b	JUC0600013577.mm.1	3,01	0,039227	0,53882		
TC0600003321.mm.1	1,51	Grin2b	PSR0600026047.mm.1	2,17	0,020942	0,479959	Cassette Exon	0,14
TC0600003321.mm.1	1,51	Grin2b	PSR0600026050.mm.1	-3,2	0,005448	0,379921	Cassette Exon	0,21
TC0900003041.mm.1	2,23	Gnat1	JUC0900014265.mm.1	3,41	0,016912	0,460797		
TC0900003041.mm.1	2,23	Gnat1	JUC0900014261.mm.1	-2,38	0,00239	0,348564		
TC0900003041.mm.1	2,23	Gnat1	PSR0900025426.mm.1	-2,51	0,001128	0,317328	Cassette Exon	0,16
TC1200002150.mm.1	-1,47	Cep128	JUC1200008225.mm.1	3,41	0,018667	0,470205		
TC1200002150.mm.1	-1,47	Cep128	PSR1200014946.mm.1	2,56	0,018173	0,467441	Cassette Exon	0,13
TC1200002150.mm.1	-1,47	Cep128	PSR1200014912.mm.1	2,19	0,047526	0,558686	Cassette Exon	0,09
TC1200002150.mm.1	-1,47	Cep128	PSR1200014928.mm.1	2,07	0,016001	0,457051	Cassette Exon	0,1
TC1200002150.mm.1	-1,47	Cep128	JUC1200008205.mm.1	2,04	0,040416	0,541998		
TC0400000080.mm.1	-1,85	Gm11827	JUC0400000241.mm.1	3,41	0,020952	0,479982		
TC0400000080.mm.1	-1,85	Gm11827	JUC0400000239.mm.1	2,97	0,000149	0,272178		
TC0400000080.mm.1	-1,85	Gm11827	PSR0400000500.mm.1	2,24	0,000628	0,304044	Cassette Exon	0,12
TC0400000080.mm.1	-1,85	Gm11827	PSR0400000501.mm.1	2,17	0,00206	0,341885	Cassette Exon	0,12
TC0800000550.mm.1	1,26	Rwdd4a	JUC0800002102.mm.1	3,41	0,004818	0,371408		
TC0900001458.mm.1	1,14	Elp6	JUC0900006757.mm.1	3,41	0,016272	0,45836		

TC0700003001.mm.1	-1,13	Trpm4	JUC0700014483.mm.1	3,41	0,021443	0,482173		
TC0400001090.mm.1	-1,38	1700024P16Rik	JUC0400003823.mm.1	3,41	0,010446	0,421652		
TC1700000754.mm.1	2,03	Gabbr1	JUC1700004140.mm.1	3,4	0,023297	0,49004		
TC1700000754.mm.1	2,03	Gabbr1	JUC1700004150.mm.1	3,22	0,011784	0,430144		
TC1700000754.mm.1	2,03	Gabbr1	JUC1700004137.mm.1	2,29	0,007708	0,402552		
TC1700000754.mm.1	2,03	Gabbr1	JUC1700004143.mm.1	2,18	0,028455	0,507299		
TC1700000754.mm.1	2,03	Gabbr1	PSR1700007622.mm.1	-2,11	0,035756	0,529087		
TC1700000754.mm.1	2,03	Gabbr1	PSR1700007645.mm.1	-2,21	0,041724	0,545379	Alternative 3' Acce	0,2
TC1700000754.mm.1	2,03	Gabbr1	PSR1700007620.mm.1	-2,35	0,048461	0,560828	Cassette Exon	0,21
TC1700000754.mm.1	2,03	Gabbr1	PSR1700007632.mm.1	-2,5	0,031592	0,517221	Alternative 3' Acce	0,38
TC1700000754.mm.1	2,03	Gabbr1	PSR1700007623.mm.1	-2,65	0,026466	0,501252	Cassette Exon	0,18
TC1700000754.mm.1	2,03	Gabbr1	PSR1700007619.mm.1	-2,65	0,015661	0,45537	Cassette Exon	0,14
TC1700000754.mm.1	2,03	Gabbr1	PSR1700007633.mm.1	-2,9	0,019838	0,475536	Alternative 3' Acce	0,41
TC1700000754.mm.1	2,03	Gabbr1	JUC1700004136.mm.1	-2,97	0,008287	0,406032		
TC1700000754.mm.1	2,03	Gabbr1	PSR1700007654.mm.1	-3,25	0,017046	0,461805	Alternative 3' Acce	0,35
TC1700000754.mm.1	2,03	Gabbr1	PSR1700007624.mm.1	-3,96	0,022458	0,487003	Alternative 5' Donc	0,27
TC1700000754.mm.1	2,03	Gabbr1	PSR1700007628.mm.1	-4,06	0,012378	0,434939	Alternative 5' Donc	0,48
TC1700000754.mm.1	2,03	Gabbr1	JUC1700004139.mm.1	-4,88	0,021094	0,480377		
TC0X00003339.mm.1	2,76	Glra2	PSR0X00020818.mm.1	3,4	0,002513	0,349347	Cassette Exon	0,31
TC0X00003339.mm.1	2,76	Glra2	JUC0X00010503.mm.1	2,34	0,038403	0,536405		
TC0X00003339.mm.1	2,76	Glra2	PSR0X00020824.mm.1	-2,02	0,039527	0,539764	Alternative 5' Donc	0,15
TC0X00003339.mm.1	2,76	Glra2	PSR0X00020816.mm.1	-2,19	0,001299	0,322929	Cassette Exon	0,07
TC0400001514.mm.1	-1,24	Fndc5	JUC0400006088.mm.1	3,4	0,006864	0,394116		
TC0400001514.mm.1	-1,24	Fndc5	PSR0400011660.mm.1	2,72	0,000624	0,304044	Cassette Exon	0,27
TC0400001514.mm.1	-1,24	Fndc5	JUC0400006085.mm.1	2,52	0,03043	0,513134		
TC0400001514.mm.1	-1,24	Fndc5	PSR0400011658.mm.1	2,29	0,047875	0,559408	Cassette Exon	0,16
TC0200003192.mm.1	-1,11	Notch1	JUC0200013543.mm.1	3,4	0,00965	0,417359		
TC0200003192.mm.1	-1,11	Notch1	JUC0200013523.mm.1	2,16	0,001463	0,328846		
TC1200002101.mm.1	-1,64	0610007P14Rik	JUC1200008029.mm.1	3,4	0,008446	0,406611		
TC0200004983.mm.1	-1,11	Rbm39	JUC0200021986.mm.1	3,4	0,049678	0,563531		
TC1200002406.mm.1	4,02	Gm266	PSR1200016702.mm.1	3,39	0,020302	0,477245	Alternative 3' Acce	0,38
TC1200002406.mm.1	4,02	Gm266	PSR1200016704.mm.1	-3,39	0,020302	0,477245	Cassette Exon	0,22
TC0900001297.mm.1	-1,73	Slco2a1	JUC0900005541.mm.1	3,39	0,011852	0,430752		
TC0900001297.mm.1	-1,73	Slco2a1	PSR0900010077.mm.1	2,26	0,03462	0,52602	Cassette Exon	0,12
TC0900001297.mm.1	-1,73	Slco2a1	PSR0900010103.mm.1	2,02	0,001921	0,34017	Cassette Exon	0,1
TC1800001054.mm.1	1,17	Garem	JUC1800004345.mm.1	3,39	0,044579	0,551978		
TC0700000018.mm.1	-1,35	Tsen34	JUC0700000114.mm.1	3,39	0,048868	0,561704		
TC0500002979.mm.1	1,25	Zfp932; Gm17655	JUC0500014364.mm.1	3,39	0,024227	0,493294		
TC0500002979.mm.1	1,25	Zfp932; Gm17655	JUC0500014362.mm.1	-2,51	0,033256	0,52224		
TC0X00001343.mm.1	2,09	Zcchc18; Gm15038	PSR0X00008471.mm.1	3,38	0,000357	0,290838	Alternative 5' Donc	0,34
TC0X00001343.mm.1	2,09	Zcchc18; Gm15038	PSR0X00008476.mm.1	-2,04	0,046379	0,556334	Alternative 5' Donc	0,17
TC0X00001343.mm.1	2,09	Zcchc18; Gm15038	PSR0X00008466.mm.1	-2,93	0,041283	0,544166	Alternative 3' Acce	0,18
TC1700002422.mm.1	-1,36	Ndufv2	PSR1700022485.mm.1	3,38	0,002433	0,349135	Cassette Exon	0,28
TC0200002078.mm.1	-1,15	Slx4ip	JUC0200008342.mm.1	3,38	0,024566	0,494179		
TC0200002078.mm.1	-1,15	Slx4ip	JUC0200008350.mm.1	2,04	0,013252	0,441416		
TC1000003132.mm.1	-1,32	Spryd4	JUC1000012946.mm.1	3,38	0,030221	0,512452		
TC0400004050.mm.1	1,65	Slc25a33	JUC0400017582.mm.1	3,38	0,008735	0,409597		
TC0900002698.mm.1	-1,88	Mlip	JUC0900012490.mm.1	3,38	0,022942	0,488667		

TC0900002698.mm.1	-1,88	Mlip	JUC0900012487.mm.1	2,18	0,034363	0,525595		
TC1200000792.mm.1	-1,34	Psen1	PSR1200005610.mm.1	3,37	0,018681	0,470289	Alternative 3' Acce	0,35
TC0600001802.mm.1	-1,01	Sgce	PSR0600014235.mm.1	3,37	0,023135	0,489756	Cassette Exon	0,34
TC0400003337.mm.1	1,89	Mast2	PSR0400026655.mm.1	3,37	0,010452	0,421652	Alternative 3' Acce	0,33
TC0400003337.mm.1	1,89	Mast2	PSR0400026667.mm.1	3,19	0,004477	0,367858	Cassette Exon	0,29
TC0400003337.mm.1	1,89	Mast2	PSR0400026638.mm.1	-2,16	0,0103	0,420394	Alternative 5' Dono	0,18
TC1400002366.mm.1	1,07	LOC675947	JUC1400010201.mm.1	3,37	0,018324	0,468127		
TC1400002366.mm.1	1,07	LOC675947	JUC1400010203.mm.1	3,18	0,009941	0,41841		
TC1400002366.mm.1	1,07	LOC675947	JUC1400010195.mm.1	2,95	0,023204	0,490032		
TC1400002366.mm.1	1,07	LOC675947	PSR1400018492.mm.1	2,19	0,026713	0,502096	Cassette Exon	0,17
TC1400002366.mm.1	1,07	LOC675947	PSR1400018498.mm.1	2,05	0,011195	0,426491	Cassette Exon	0,2
TC1400002366.mm.1	1,07	LOC675947	JUC1400010219.mm.1	-2,15	0,046234	0,556097		
TC1400002366.mm.1	1,07	LOC675947	JUC1400010198.mm.1	-2,2	0,003215	0,354243		
TC1800000641.mm.1	1,09	Htr4	JUC1800002625.mm.1	3,37	0,007173	0,397855		
TC0100001202.mm.1	1,02	Ppp1r15b	JUC0100005665.mm.1	3,37	0,0316	0,517317		
TC1500002238.mm.1	-1,01	Slc11a2	JUC1500010441.mm.1	3,37	0,025011	0,496061		
TC0800001463.mm.1	1,07	1700030M09Rik	JUC0800006521.mm.1	3,37	0,015152	0,452699		
TC0700000169.mm.1	-1,44	Sult2a2	JUC0700000650.mm.1	3,37	0,035252	0,52837		
TC0600000182.mm.1	-1,16	Cped1	JUC0600000577.mm.1	3,37	0,037434	0,533831		
TC0600000182.mm.1	-1,16	Cped1	JUC0600000564.mm.1	3,21	0,042356	0,547419		
TC0X00000388.mm.1	1,4	Gria3; Gm22413	JUC0X00001295.mm.1	3,37	0,011436	0,427663		
TC0X00000388.mm.1	1,4	Gria3; Gm22413	JUC0X00001296.mm.1	-2,17	0,019233	0,472971		
TC0800000112.mm.1	1,85	Mcf2l	JUC0800000549.mm.1	3,36	0,002125	0,342919		
TC0800000112.mm.1	1,85	Mcf2l	JUC0800000558.mm.1	2,15	0,022462	0,487037		
TC0800000112.mm.1	1,85	Mcf2l	JUC0800000542.mm.1	2,15	0,02735	0,503766		
TC0800000112.mm.1	1,85	Mcf2l	PSR0800001006.mm.1	2,14	0,001004	0,316361	Cassette Exon	0,15
TC0800000112.mm.1	1,85	Mcf2l	PSR0800001031.mm.1	-2,01	0,034223	0,525151		
TC0800000112.mm.1	1,85	Mcf2l	JUC0800000551.mm.1	-2,03	0,046176	0,555838		
TC0800000112.mm.1	1,85	Mcf2l	PSR0800000967.mm.1	-2,04	0,039375	0,539409	Alternative 3' Acce	0,11
TC0800000112.mm.1	1,85	Mcf2l	JUC0800000566.mm.1	-2,07	0,019191	0,472846		
TC0800000112.mm.1	1,85	Mcf2l	PSR0800000960.mm.1	-2,09	0,022933	0,488642		
TC0800000112.mm.1	1,85	Mcf2l	JUC0800000536.mm.1	-2,1	0,005234	0,377425		
TC0800000112.mm.1	1,85	Mcf2l	PSR0800001027.mm.1	-2,11	0,036866	0,532257	Intron Retention	0,08
TC0800000112.mm.1	1,85	Mcf2l	JUC0800000563.mm.1	-2,11	0,00752	0,401141		
TC0800000112.mm.1	1,85	Mcf2l	JUC0800000572.mm.1	-2,11	0,02713	0,503041		
TC0800000112.mm.1	1,85	Mcf2l	PSR0800000974.mm.1	-2,13	0,031139	0,515568	Alternative 3' Acce	0,08
TC0800000112.mm.1	1,85	Mcf2l	PSR0800000963.mm.1	-2,16	0,005665	0,381922	Alternative 3' Acce	0,16
TC0800000112.mm.1	1,85	Mcf2l	PSR0800000982.mm.1	-2,26	0,019063	0,471993	Alternative 5' Dono	0,09
TC0800000112.mm.1	1,85	Mcf2l	JUC0800000540.mm.1	-2,34	0,030971	0,514659		
TC0800000112.mm.1	1,85	Mcf2l	PSR0800000955.mm.1	-2,38	0,00269	0,349612	Cassette Exon	0,13
TC0800000112.mm.1	1,85	Mcf2l	JUC0800000547.mm.1	-2,75	0,013811	0,445064		
TC0800000112.mm.1	1,85	Mcf2l	JUC0800000539.mm.1	-2,75	0,03356	0,523131		
TC0800000112.mm.1	1,85	Mcf2l	PSR0800000970.mm.1	-2,8	0,008748	0,409704	Alternative 3' Acce	0,13
TC0800000112.mm.1	1,85	Mcf2l	PSR0800000957.mm.1	-2,82	0,0315	0,516965	Cassette Exon	0,27
TC0800000112.mm.1	1,85	Mcf2l	JUC0800000543.mm.1	-3,31	0,016888	0,460797		
TC0800000112.mm.1	1,85	Mcf2l	JUC0800000573.mm.1	-3,31	0,028526	0,507436		
TC0800000112.mm.1	1,85	Mcf2l	JUC0800000538.mm.1	-5,22	0,002332	0,347203		
TC0100000044.mm.1	-1,4	Sgk3	PSR0100000348.mm.1	3,36	0,008298	0,406122	Cassette Exon	0,22

TC0600003176.mm.1	1,92	Prmt8	PSR0600024799.mm.1	3,36	0,000089	0,255472	Cassette Exon	0,2
TC0600003176.mm.1	1,92	Prmt8	PSR0600024795.mm.1	2,1	0,009331	0,414973	Cassette Exon	0,09
TC1000002903.mm.1	1,7	Lgr5	JUC1000012045.mm.1	3,36	0,048778	0,561474		
TC1000002903.mm.1	1,7	Lgr5	JUC1000012052.mm.1	3,28	0,01058	0,422493		
TC1000002903.mm.1	1,7	Lgr5	JUC1000012053.mm.1	2,21	0,016894	0,460797		
TC1000002903.mm.1	1,7	Lgr5	PSR1000021922.mm.1	-2,29	0,016102	0,457676	Intron Retention	0,15
TC1000002903.mm.1	1,7	Lgr5	PSR1000021929.mm.1	-2,52	0,000816	0,311886	Alternative 3' Acce	0,13
TC1200000149.mm.1	1	Gm9222	JUC1200000744.mm.1	3,36	0,006905	0,394866		
TC1200000149.mm.1	1	Gm9222	JUC1200000748.mm.1	2,62	0,001655	0,334378		
TC1200000149.mm.1	1	Gm9222	JUC1200000743.mm.1	2,12	0,037906	0,535116		
TC1200000149.mm.1	1	Gm9222	JUC1200000751.mm.1	-2,5	0,047765	0,559125		
TC1000000752.mm.1	1,01	Slc19a1	JUC1000002978.mm.1	3,36	0,041429	0,544646		
TC1200000247.mm.1	1,58	Myt1l	PSR1200001881.mm.1	3,35	0,001198	0,321568	Cassette Exon	0,22
TC1200000247.mm.1	1,58	Myt1l	JUC1200001075.mm.1	2,7	0,004077	0,36209		
TC1200000247.mm.1	1,58	Myt1l	PSR1200001874.mm.1	2,47	0,002595	0,349501	Cassette Exon	0,18
TC1200000247.mm.1	1,58	Myt1l	JUC1200001068.mm.1	2,36	0,033872	0,524121		
TC1200000247.mm.1	1,58	Myt1l	PSR1200001893.mm.1	2,16	0,009159	0,413447	Cassette Exon	0,15
TC1200000247.mm.1	1,58	Myt1l	PSR1200001905.mm.1	2,07	0,016839	0,460729	Cassette Exon	0,14
TC1200000247.mm.1	1,58	Myt1l	PSR1200001870.mm.1	-2,04	0,035757	0,529087	Alternative 5' Donc	0,08
TC1200000247.mm.1	1,58	Myt1l	PSR1200001911.mm.1	-2,05	0,038218	0,535805		
TC1200000247.mm.1	1,58	Myt1l	PSR1200001871.mm.1	-2,52	0,040761	0,543092	Alternative 5' Donc	0,08
TC1200000247.mm.1	1,58	Myt1l	PSR1200001882.mm.1	-2,58	0,012317	0,434392	Alternative 5' Donc	0,39
TC1200000247.mm.1	1,58	Myt1l	PSR1200001894.mm.1	-2,6	0,036948	0,532361	Cassette Exon	0,22
TC1200000247.mm.1	1,58	Myt1l	PSR1200001883.mm.1	-3,22	0,004314	0,364152	Alternative 3' Acce	0,49
TC1200000247.mm.1	1,58	Myt1l	JUC1200001085.mm.1	-3,43	0,006755	0,393343		
TC1200000247.mm.1	1,58	Myt1l	JUC1200001095.mm.1	-4,08	0,017109	0,46232		
TC0900000096.mm.1	-1,06	Fam76b	PSR0900000681.mm.1	3,35	0,01765	0,464582	Alternative 5' Donc	0,39
TC0800000127.mm.1	11,67	Grk1	JUC0800000673.mm.1	3,35	0,007106	0,396897		
TC0800000127.mm.1	11,67	Grk1	PSR0800001188.mm.1	-2,23	0,013822	0,445154	Cassette Exon	0,25
TC0800000127.mm.1	11,67	Grk1	JUC0800000677.mm.1	-2,84	0,006382	0,389554		
TC0800000127.mm.1	11,67	Grk1	JUC0800000676.mm.1	-10,45	0,002051	0,341397		
TC1700000085.mm.1	-1,8	Mpc1	JUC1700000379.mm.1	3,35	0,023246	0,490032		
TC1700000085.mm.1	-1,8	Mpc1	PSR1700000670.mm.1	2,97	0,048486	0,560864	Cassette Exon	0,1
TC1700000085.mm.1	-1,8	Mpc1	PSR1700000675.mm.1	2,86	0,030464	0,513244	Cassette Exon	0,09
TC1700000085.mm.1	-1,8	Mpc1	JUC1700000377.mm.1	2,18	0,048431	0,560744		
TC0200001786.mm.1	-1,03	Mapkbp1	JUC0200006753.mm.1	3,35	0,009568	0,416927		
TC0200001786.mm.1	-1,03	Mapkbp1	JUC0200006747.mm.1	2,66	0,020813	0,479514		
TC0700004363.mm.1	-2,13	Rgs10	JUC0700019318.mm.1	3,35	0,003393	0,354856		
TC0700004122.mm.1	-1,36	2310014F06Rik	JUC0700017949.mm.1	3,35	0,04886	0,561704		
TC0400001137.mm.1	-1,17	0610037L13Rik	JUC0400004137.mm.1	3,35	0,038934	0,537874		
TC0400003957.mm.1	-1,26	Tnfrsf1b	JUC0400017108.mm.1	3,35	0,038431	0,536405		
TC0900002427.mm.1	11,4	Nr2e3; Gm15507	PSR0900020342.mm.1	3,34	0,000012	0,179072		
TC0900002427.mm.1	11,4	Nr2e3; Gm15507	JUC0900011470.mm.1	3,31	0,000003	0,179072		
TC0900002427.mm.1	11,4	Nr2e3; Gm15507	JUC0900011467.mm.1	2,51	0,000314	0,288663		
TC0900002427.mm.1	11,4	Nr2e3; Gm15507	JUC0900011465.mm.1	-2,22	0,016764	0,460504		
TC0900002427.mm.1	11,4	Nr2e3; Gm15507	PSR0900020332.mm.1	-3,25	0,043571	0,550033	Alternative 3' Acce	0,28
TC0900002427.mm.1	11,4	Nr2e3; Gm15507	PSR0900020341.mm.1	-4,51	0,001133	0,317344		
TC0900002427.mm.1	11,4	Nr2e3; Gm15507	JUC0900011471.mm.1	-6,84	0,000135	0,270905		

TC0900002427.mm.1	11,4	Nr2e3; Gm15507	PSR0900020334.mm.1	-8,1	0,012976	0,439245	Intron Retention	0,73
TC0900002427.mm.1	11,4	Nr2e3; Gm15507	JUC0900011472.mm.1	-8,13	0,001525	0,330287		
TC0900002427.mm.1	11,4	Nr2e3; Gm15507	PSR0900020328.mm.1	-8,18	0,008893	0,410717	Alternative 3' Acce	0,45
TC0900002427.mm.1	11,4	Nr2e3; Gm15507	JUC0900011473.mm.1	-8,31	0,000371	0,291725		
TC0900002427.mm.1	11,4	Nr2e3; Gm15507	PSR0900020349.mm.1	-8,7	0,005442	0,379743	Intron Retention	0,67
TC0900002427.mm.1	11,4	Nr2e3; Gm15507	PSR0900020363.mm.1	-10,37	0,002385	0,348564	Cassette Exon	0,21
TC0900002427.mm.1	11,4	Nr2e3; Gm15507	PSR0900020346.mm.1	-11	0,006102	0,387166	Intron Retention	0,68
TC0900002427.mm.1	11,4	Nr2e3; Gm15507	PSR0900020344.mm.1	-11,33	0,000438	0,297771	Intron Retention	0,75
TC0900002427.mm.1	11,4	Nr2e3; Gm15507	PSR0900020356.mm.1	-12,37	0,002686	0,349612	Cassette Exon	0,21
TC0900002427.mm.1	11,4	Nr2e3; Gm15507	PSR0900020348.mm.1	-14,24	0,001251	0,322251	Intron Retention	0,67
TC0900002427.mm.1	11,4	Nr2e3; Gm15507	PSR0900020353.mm.1	-14,46	0,00225	0,34578	Alternative 5' Donc	0,44
TC0900002427.mm.1	11,4	Nr2e3; Gm15507	JUC0900011477.mm.1	-19,76	0,030913	0,514584		
TC0900002427.mm.1	11,4	Nr2e3; Gm15507	PSR0900020355.mm.1	-21,62	0,000217	0,28803	Cassette Exon	0,21
TC0900002427.mm.1	11,4	Nr2e3; Gm15507	PSR0900020360.mm.1	-24,08	0,000618	0,304044	Cassette Exon	0,55
TC0900002427.mm.1	11,4	Nr2e3; Gm15507	JUC0900011476.mm.1	-24,09	0,001693	0,335395		
TC0900002427.mm.1	11,4	Nr2e3; Gm15507	PSR0900020364.mm.1	-24,5	0,000539	0,304044	Cassette Exon	0,41
TC0900002427.mm.1	11,4	Nr2e3; Gm15507	JUC0900011475.mm.1	-36,87	0,000643	0,304044		
TC0900002427.mm.1	11,4	Nr2e3; Gm15507	PSR0900020338.mm.1	-38,34	0,0003	0,288663	Intron Retention	0,75
TC0900002427.mm.1	11,4	Nr2e3; Gm15507	JUC0900011478.mm.1	-54,12	0,000491	0,298999		
TC0900002427.mm.1	11,4	Nr2e3; Gm15507	PSR0900020365.mm.1	-61,46	0,000666	0,304088	Cassette Exon	0,28
TC1100003387.mm.1	-3,07		JUC1100016224.mm.1	3,34	0,025578	0,497808		
TC1100003387.mm.1	-3,07		PSR1100031034.mm.1	3,19	0,048335	0,560493	Cassette Exon	0,31
TC1300002436.mm.1	-1,1	Bhmt	JUC1300008596.mm.1	3,34	0,000103	0,262646		
TC0100002554.mm.1	-1,08	Pecr	JUC0100011656.mm.1	3,34	0,034316	0,525546		
TC1600001112.mm.1	-1,12	Slx4	JUC1600004595.mm.1	3,34	0,019872	0,475704		
TC0600001522.mm.1	-1,49	Clec2g	JUC0600006530.mm.1	3,34	0,016176	0,457969		
TC1100001066.mm.1	1,01	Smyd4	JUC1100005047.mm.1	3,34	0,002496	0,349135		
TC0X00003376.mm.1	1,15	Hccs	JUC0X00010655.mm.1	3,34	0,00616	0,387873		
TC1500000049.mm.1	3,22		PSR1500000434.mm.1	3,33	0,025499	0,497664	Cassette Exon	0,23
TC1500000049.mm.1	3,22		JUC1500000265.mm.1	-3,46	0,019136	0,472431		
TC1700000946.mm.1	2,58	Lrfn2	PSR1700009122.mm.1	3,33	0,042403	0,547627	Cassette Exon	0,2
TC0700004579.mm.1	-1,07	Trpm5	JUC0700020367.mm.1	3,33	0,013635	0,444143		
TC0700004579.mm.1	-1,07	Trpm5	JUC0700020349.mm.1	2,09	0,022216	0,485896		
TC0900000221.mm.1	1,31	9530077C05Rik	JUC0900000887.mm.1	3,33	0,001191	0,321162		
TC0400001893.mm.1	-2,04		JUC0400008174.mm.1	3,33	0,000074	0,251428		
TC0X00002423.mm.1	-1,26	Xlr4c	JUC0X00007631.mm.1	3,33	0,019511	0,474538		
TC1000002702.mm.1	1,17	Vezt; Mir331	PSR1000020790.mm.1	3,32	0,025187	0,496653	Cassette Exon	0,29
TC1000002702.mm.1	1,17	Vezt; Mir331	JUC1000011432.mm.1	-2,03	0,009311	0,414883		
TC1300001012.mm.1	-1,57	Tmem167	PSR1300006471.mm.1	3,32	0,016286	0,458454	Alternative 5' Donc	0,27
TC1300001012.mm.1	-1,57	Tmem167	JUC1300003393.mm.1	-2,25	0,014989	0,452048		
TC0200001620.mm.1	-1,05	Kif18a	JUC0200006021.mm.1	3,32	0,040368	0,541959		
TC0200001620.mm.1	-1,05	Kif18a	PSR0200012093.mm.1	-2,32	0,042855	0,548131	Alternative 5' Donc	0,24
TC0200001620.mm.1	-1,05	Kif18a	JUC0200006020.mm.1	-2,61	0,02163	0,483552		
TC0300001231.mm.1	-1,36		JUC0300005166.mm.1	3,32	0,01251	0,435429		
TC1800000754.mm.1	-1,29		JUC1800003022.mm.1	3,32	0,043585	0,550052		
TC1800000754.mm.1	-1,29		JUC1800003024.mm.1	2,71	0,008397	0,406601		
TC1500000125.mm.1	1,41	Cdh10	JUC1500000656.mm.1	3,32	0,003342	0,354243		
TC1500000125.mm.1	1,41	Cdh10	JUC1500000662.mm.1	2,15	0,023043	0,489301		

TC0800000516.mm.1	-1,05	Sorbs2; LOC100861925; G	JUC0800001934.mm.1	3,32	0,03917	0,538679		
TC0600001890.mm.1	-1,37	Tfec	JUC0600007769.mm.1	3,32	0,028016	0,505851		
TC0500003008.mm.1	-1,43	E130006D01Rik	JUC0500014579.mm.1	3,32	0,003203	0,354243		
TC1100003124.mm.1	-1,22	Nlrp1a	JUC1100015155.mm.1	3,32	0,025891	0,498956		
TC0300002153.mm.1	1,84	Slitrk3	JUC0300008492.mm.1	3,32	0,025776	0,498465		
TC0900002338.mm.1	1,18	Scaper	JUC0900011085.mm.1	3,32	0,003733	0,357586		
TC0400002578.mm.1	-1,74	Slc25a51; Gm25692	JUC0400010904.mm.1	3,32	0,000769	0,308403		
TC0400002578.mm.1	-1,74	Slc25a51; Gm25692	JUC0400010902.mm.1	3,08	0,000823	0,311886		
TC0400002578.mm.1	-1,74	Slc25a51; Gm25692	PSR0400021025.mm.1	2,67	0,003503	0,355749		
TC0300002242.mm.1	1,77	Trim2	JUC0300008876.mm.1	3,31	0,007155	0,397804		
TC0300002242.mm.1	1,77	Trim2	PSR0300016879.mm.1	2,44	0,006953	0,395699	Alternative 3' Acce	0,19
TC0300002242.mm.1	1,77	Trim2	PSR0300016907.mm.1	-2,01	0,029543	0,510303	Cassette Exon	0,03
TC0300002242.mm.1	1,77	Trim2	PSR0300016857.mm.1	-2,07	0,0117	0,429969	Cassette Exon	0,11
TC0300002242.mm.1	1,77	Trim2	PSR0300016906.mm.1	-2,13	0,039854	0,54093	Alternative 3' Acce	0,02
TC0300002242.mm.1	1,77	Trim2	PSR0300016910.mm.1	-2,17	0,023805	0,491893	Alternative 5' Donc	0,15
TC0300002242.mm.1	1,77	Trim2	JUC0300008883.mm.1	-2,35	0,030883	0,514551		
TC0300002242.mm.1	1,77	Trim2	JUC0300008878.mm.1	-2,87	0,004291	0,363973		
TC0300002242.mm.1	1,77	Trim2	PSR0300016903.mm.1	-3,42	0,024366	0,49362	Alternative 5' Donc	0,29
TC0200003373.mm.1	1,47	Ttll11	JUC0200014650.mm.1	3,31	0,02754	0,504368		
TC0200003373.mm.1	1,47	Ttll11	PSR0200029011.mm.1	3	0,002439	0,349135	Cassette Exon	0,28
TC0200003373.mm.1	1,47	Ttll11	JUC0200014656.mm.1	-2,67	0,013328	0,441836		
TC0200003373.mm.1	1,47	Ttll11	JUC0200014654.mm.1	-2,82	0,000025	0,225289		
TC0600001958.mm.1	1,97	Grm8; Mir592	PSR0600015338.mm.1	3,31	0,020373	0,477557	Cassette Exon	0,26
TC0600001958.mm.1	1,97	Grm8; Mir592	JUC0600008032.mm.1	2,61	0,002873	0,352207		
TC0600001958.mm.1	1,97	Grm8; Mir592	PSR0600015341.mm.1	2,18	0,007811	0,403089	Cassette Exon	0,12
TC0600001958.mm.1	1,97	Grm8; Mir592	PSR0600015335.mm.1	2,11	0,026628	0,50179	Cassette Exon	0,11
TC0600001958.mm.1	1,97	Grm8; Mir592	PSR0600015337.mm.1	2,11	0,006026	0,386586	Cassette Exon	0,11
TC0600001958.mm.1	1,97	Grm8; Mir592	PSR0600015339.mm.1	2,1	0,015697	0,455416	Cassette Exon	0,22
TC0600001958.mm.1	1,97	Grm8; Mir592	PSR0600015351.mm.1	-2,34	0,014876	0,451677	Alternative 5' Donc	0,21
TC0600001958.mm.1	1,97	Grm8; Mir592	PSR0600015345.mm.1	-3,19	0,018256	0,467907	Cassette Exon	0,11
TC0600001958.mm.1	1,97	Grm8; Mir592	PSR0600015356.mm.1	-3,9	0,010631	0,422841	Cassette Exon	0,27
TC0500000466.mm.1	1,37	Bst1	JUC0500002519.mm.1	3,31	0,024906	0,495688		
TC0500000466.mm.1	1,37	Bst1	JUC0500002524.mm.1	2,82	0,000824	0,311886		
TC0500000466.mm.1	1,37	Bst1	PSR0500004509.mm.1	2,64	0,011762	0,430069	Cassette Exon	0,17
TC0500000466.mm.1	1,37	Bst1	JUC0500002525.mm.1	-2,08	0,008423	0,406611		
TC0200002735.mm.1	1,23	2210418O10Rik; Gm1725	JUC0200011321.mm.1	3,31	0,02613	0,499897		
TC0300001247.mm.1	-1,09	Bcar3	JUC0300005313.mm.1	3,31	0,002154	0,343877		
TC0100000658.mm.1	-1,05	Speg	JUC0100003216.mm.1	3,31	0,008864	0,410539		
TC0100000658.mm.1	-1,05	Speg	JUC0100003223.mm.1	2,14	0,004059	0,361877		
TC0200001387.mm.1	-2,29		PSR0200010911.mm.1	3,31	0,004852	0,37168		
TC0100000638.mm.1	-1,1	Bcs1l	JUC0100003032.mm.1	3,31	0,023229	0,490032		
TC0200001794.mm.1	-1,16	Snap23	JUC0200006872.mm.1	3,31	0,027408	0,503961		
TC0700002938.mm.1	-1,02	Vmn2r63	JUC0700013906.mm.1	3,31	0,018925	0,47133		
TC1100000374.mm.1	-1,29	Slit3	JUC1100001660.mm.1	3,31	0,015839	0,456215		
TC1100000374.mm.1	-1,29	Slit3	JUC1100001642.mm.1	2,62	0,003083	0,353892		
TC1100002180.mm.1	1,05	Nacad	JUC1100011078.mm.1	3,31	0,026564	0,501526		
TC1100002180.mm.1	1,05	Nacad	JUC1100011076.mm.1	-2,73	0,001812	0,336654		
TC0Y00000293.mm.1	-1,37	Gm21488	JUC0Y00001252.mm.1	3,31	0,018794	0,470789		

TC0Y00000292.mm.1	-1,37	Gm20890	JUC0Y00001243.mm.1	3,31	0,018794	0,470789		
TC0600003074.mm.1	2,78	Slc2a3	JUC0600012413.mm.1	3,3	0,031845	0,517896		
TC0600003074.mm.1	2,78	Slc2a3	PSR0600023811.mm.1	-2,37	0,00942	0,41597	Alternative 3' Acce	0,23
TC0600003074.mm.1	2,78	Slc2a3	PSR0600023837.mm.1	-2,48	0,029139	0,509333	Cassette Exon	0,17
TC0600003074.mm.1	2,78	Slc2a3	PSR0600023836.mm.1	-2,64	0,008418	0,406611	Cassette Exon	0,32
TC0600003074.mm.1	2,78	Slc2a3	PSR0600023818.mm.1	-2,87	0,035766	0,529126	Alternative 5' Donc	0,25
TC0600003074.mm.1	2,78	Slc2a3	PSR0600023812.mm.1	-3,53	0,013656	0,444386	Alternative 3' Acce	0,32
TC0600003074.mm.1	2,78	Slc2a3	PSR0600023834.mm.1	-3,55	0,021851	0,484607	Cassette Exon	0,03
TC0600003074.mm.1	2,78	Slc2a3	PSR0600023833.mm.1	-4,17	0,022084	0,485424	Alternative 5' Donc	0,47
TC0600003074.mm.1	2,78	Slc2a3	JUC0600012420.mm.1	-4,33	0,026739	0,502096		
TC0600003074.mm.1	2,78	Slc2a3	JUC0600012418.mm.1	-4,48	0,035696	0,529027		
TC0600003074.mm.1	2,78	Slc2a3	JUC0600012421.mm.1	-4,55	0,03099	0,514765		
TC0600001953.mm.1	3,36	Gpr37	PSR0600015281.mm.1	3,3	0,00443	0,366832	Cassette Exon	0,22
TC0600001953.mm.1	3,36	Gpr37	PSR0600015283.mm.1	-3,42	0,000612	0,304044	Alternative 5' Donc	0,43
TC0600001953.mm.1	3,36	Gpr37	PSR0600015284.mm.1	-4,73	0,002217	0,345773	Alternative 5' Donc	0,43
TC0400001391.mm.1	1,58	Hpcal4	PSR0400010490.mm.1	3,3	0,003701	0,357266	Alternative 5' Donc	0,29
TC0400001391.mm.1	1,58	Hpcal4	PSR0400010489.mm.1	2,32	0,006288	0,388916	Alternative 5' Donc	0,29
TC0400001391.mm.1	1,58	Hpcal4	PSR0400010491.mm.1	2,3	0,032415	0,519327	Alternative 5' Donc	0,26
TC0400001391.mm.1	1,58	Hpcal4	PSR0400010481.mm.1	-2,4	0,006034	0,386586	Alternative 3' Acce	0,17
TC1000001466.mm.1	1,99	Grip1	JUC1000005608.mm.1	3,3	0,002905	0,352262		
TC1000001466.mm.1	1,99	Grip1	JUC1000005607.mm.1	3,01	0,003029	0,353543		
TC1000001466.mm.1	1,99	Grip1	PSR1000010451.mm.1	2,13	0,000657	0,304044	Cassette Exon	0,21
TC1000001466.mm.1	1,99	Grip1	PSR1000010434.mm.1	-2,17	0,016626	0,459985	Cassette Exon	0,07
TC1000001466.mm.1	1,99	Grip1	PSR1000010439.mm.1	-2,35	0,024917	0,4958	Alternative 5' Donc	0,11
TC1000001466.mm.1	1,99	Grip1	JUC1000005609.mm.1	-2,63	0,049548	0,563325		
TC1000001466.mm.1	1,99	Grip1	PSR1000010411.mm.1	-2,67	0,003694	0,357266	Cassette Exon	0,23
TC1000001466.mm.1	1,99	Grip1	JUC1000005597.mm.1	-2,69	0,00257	0,349501		
TC1000001466.mm.1	1,99	Grip1	PSR1000010418.mm.1	-2,96	0,004531	0,368648	Cassette Exon	0,07
TC1000001466.mm.1	1,99	Grip1	JUC1000005588.mm.1	-4,54	0,017173	0,46232		
TC1000001466.mm.1	1,99	Grip1	PSR1000010420.mm.1	-4,73	0,001056	0,316361	Cassette Exon	0,27
TC1400001536.mm.1	1	Ttc18	JUC1400006143.mm.1	3,3	0,013837	0,445311		
TC1200002224.mm.1	1,26	Ttc7b	JUC1200008444.mm.1	3,3	0,00868	0,408815		
TC1200002224.mm.1	1,26	Ttc7b	JUC1200008446.mm.1	2,1	0,037411	0,533831		
TC1900000944.mm.1	1,09	Npas4	JUC1900004588.mm.1	3,3	0,012395	0,434939		
TC0100003318.mm.1	-1,23	Axdnd1	JUC0100015495.mm.1	3,3	0,000668	0,304088		
TC0100003318.mm.1	-1,23	Axdnd1	JUC0100015521.mm.1	-3,75	0,024725	0,494838		
TC0500000215.mm.1	1,11	Slc4a2	JUC0500000927.mm.1	3,3	0,049543	0,563325		
TC0900001463.mm.1	-1,24	Setd2	JUC0900006822.mm.1	3,3	0,013475	0,442984		
TC0900001463.mm.1	-1,24	Setd2	JUC0900006830.mm.1	3,24	0,041006	0,543644		
TC0900001463.mm.1	-1,24	Setd2	JUC0900006846.mm.1	-2,94	0,028361	0,506881		
TC0500001492.mm.1	-1,1		JUC0500007359.mm.1	3,3	0,019015	0,471745		
TC0200000347.mm.1	1,95	Gad2	PSR0200001683.mm.1	3,29	0,000156	0,272178	Cassette Exon	0,22
TC0200000347.mm.1	1,95	Gad2	PSR0200001684.mm.1	3,11	0,000144	0,272178	Cassette Exon	0,2
TC0200000347.mm.1	1,95	Gad2	PSR0200001682.mm.1	2,28	0,011098	0,426	Cassette Exon	0,16
TC0200000347.mm.1	1,95	Gad2	PSR0200001685.mm.1	2,11	0,018149	0,467399	Alternative 3' Acce	0,19
TC0200000347.mm.1	1,95	Gad2	PSR0200001677.mm.1	2,09	0,035585	0,528808	Cassette Exon	0,07
TC0200000347.mm.1	1,95	Gad2	JUC0200000748.mm.1	-2,3	0,010282	0,42034		
TC0200000347.mm.1	1,95	Gad2	PSR0200001673.mm.1	-2,31	0,002579	0,349501	Alternative 5' Donc	0,2

TC0200000347.mm.1	1,95	Gad2	PSR0200001663.mm.1	-2,41	0,013411	0,442624	Cassette Exon	0,15
TC0200000347.mm.1	1,95	Gad2	PSR0200001665.mm.1	-3,42	0,001418	0,327189	Cassette Exon	0,26
TC0200000347.mm.1	1,95	Gad2	JUC0200000750.mm.1	-3,91	0,010111	0,418998		
TC0200000347.mm.1	1,95	Gad2	PSR0200001666.mm.1	-4,84	0,005608	0,381115	Alternative 3' Acce	0,35
TC0600001103.mm.1	-1,06	9530026P05Rik; LOC1005(	JUC0600004439.mm.1	3,29	0,012217	0,433321		
TC0600001103.mm.1	-1,06	9530026P05Rik; LOC1005(	PSR0600008705.mm.1	-3,18	0,005806	0,383104	Alternative 5' Donc	0,19
TC0500002752.mm.1	3,86	Ppef2	JUC0500013205.mm.1	3,29	0,001912	0,339584		
TC0500002752.mm.1	3,86	Ppef2	JUC0500013198.mm.1	2,89	0,042306	0,547224		
TC0500002752.mm.1	3,86	Ppef2	JUC0500013195.mm.1	2,05	0,011377	0,427487		
TC0500002752.mm.1	3,86	Ppef2	PSR0500024228.mm.1	-2,24	0,031379	0,516345	Alternative 3' Acce	0,15
TC0500002752.mm.1	3,86	Ppef2	PSR0500024235.mm.1	-2,42	0,021253	0,481431	Cassette Exon	0,16
TC0500002752.mm.1	3,86	Ppef2	PSR0500024244.mm.1	-2,42	0,001638	0,33312	Cassette Exon	0,15
TC0500002752.mm.1	3,86	Ppef2	JUC0500013197.mm.1	-4,25	0,000608	0,304044		
TC0400000271.mm.1	1,16	Mdn1	JUC0400000854.mm.1	3,29	0,033704	0,523549		
TC0400000271.mm.1	1,16	Mdn1	PSR0400001632.mm.1	2,61	0,019998	0,476227	Cassette Exon	0,15
TC0400000271.mm.1	1,16	Mdn1	JUC0400000795.mm.1	2,05	0,01733	0,463416		
TC0300003156.mm.1	-1,97		JUC0300012983.mm.1	3,29	0,004077	0,36209		
TC0300003156.mm.1	-1,97		PSR0300024745.mm.1	2,19	0,030131	0,512017	Cassette Exon	0,11
TC0300003156.mm.1	-1,97		JUC0300012982.mm.1	2,02	0,001131	0,317328		
TC1500002040.mm.1	-1,12	Plxnb2	JUC1500009286.mm.1	3,29	0,019585	0,474672		
TC1500002040.mm.1	-1,12	Plxnb2	PSR1500016284.mm.1	-2,4	0,00813	0,405267	Alternative 3' Acce	0,1
TC1400000808.mm.1	-1,46	Parp4	JUC1400003552.mm.1	3,29	0,025849	0,498657		
TC0200005354.mm.1	1,27	Gm14296; 2210418O10Ril	JUC0200023470.mm.1	3,29	0,027306	0,503732		
TC1300001178.mm.1	-1,44	Srek1ip1	JUC1300003923.mm.1	3,29	0,013062	0,439579		
TC0500000317.mm.1	1,01	Nrbp1	JUC0500001522.mm.1	3,29	0,002546	0,349501		
TC0600001373.mm.1	-1,14	Mug1	JUC0600005760.mm.1	3,29	0,005151	0,37646		
TC1000001465.mm.1	-1,35	1700025F24Rik	JUC1000005586.mm.1	3,29	0,009089	0,412827		
TC0400001539.mm.1	-1,18	Col16a1	JUC0400006313.mm.1	3,29	0,003356	0,354243		
TC0400001539.mm.1	-1,18	Col16a1	JUC0400006261.mm.1	2,15	0,000555	0,304044		
TC0Y00000193.mm.1	-1,24	Gm20870; Gm20736	JUC0Y00000698.mm.1	3,29	0,010097	0,418998		
TC0Y00000213.mm.1	-1,45	Gm20906; Gm20736	JUC0Y00000862.mm.1	3,29	0,008872	0,410554		
TC0200002072.mm.1	8,1	Lamp5	PSR0200016482.mm.1	3,28	0,003143	0,353892	Cassette Exon	0,29
TC0200002072.mm.1	8,1	Lamp5	JUC0200008307.mm.1	2,68	0,013006	0,439291		
TC0200002072.mm.1	8,1	Lamp5	JUC0200008305.mm.1	-4,04	0,030903	0,514574		
TC0200002072.mm.1	8,1	Lamp5	PSR0200016472.mm.1	-4,83	0,005903	0,383835	Cassette Exon	0,41
TC0200002072.mm.1	8,1	Lamp5	PSR0200016481.mm.1	-4,98	0,000718	0,306015	Alternative 3' Acce	0,32
TC0200002072.mm.1	8,1	Lamp5	PSR0200016478.mm.1	-5,87	0,003448	0,3554	Intron Retention	0,74
TC0200002072.mm.1	8,1	Lamp5	PSR0200016479.mm.1	-6,03	0,003227	0,354243	Intron Retention	0,74
TC0200002072.mm.1	8,1	Lamp5	PSR0200016474.mm.1	-6,47	0,000803	0,311765	Alternative 3' Acce	0,57
TC0200002072.mm.1	8,1	Lamp5	PSR0200016485.mm.1	-6,48	0,00274	0,349612	Alternative 5' Donc	0,44
TC0200002072.mm.1	8,1	Lamp5	JUC0200008304.mm.1	-8,74	0,000265	0,28803		
TC0200002072.mm.1	8,1	Lamp5	PSR0200016477.mm.1	-12,6	0,002261	0,34578	Intron Retention	0,74
TC1400002641.mm.1	-2,7	Ednrb	PSR1400019790.mm.1	3,28	0,011017	0,425624	Alternative 5' Donc	0,26
TC0800000049.mm.1	-1,57	Gm17215	PSR0800000511.mm.1	3,28	0,04008	0,541392	Cassette Exon	0,24
TC1000002406.mm.1	1,13	Col6a1	JUC1000009551.mm.1	3,28	0,007927	0,403819		
TC1000002406.mm.1	1,13	Col6a1	PSR1000017406.mm.1	-2,31	0,000238	0,28803	Cassette Exon	0,12
TC1000002406.mm.1	1,13	Col6a1	PSR1000017420.mm.1	-2,42	0,002478	0,349135	Alternative 5' Donc	0,23
TC1000002406.mm.1	1,13	Col6a1	JUC1000009547.mm.1	-2,46	0,013178	0,44063		

TC0400002233.mm.1	-1,08	Lrrc69	JUC0400009761.mm.1	3,28	0,010745	0,423772		
TC0400002233.mm.1	-1,08	Lrrc69	PSR0400018665.mm.1	2,45	0,01276	0,436997	Cassette Exon	0,22
TC0200000741.mm.1	1,3	Arhgap15	PSR0200006410.mm.1	3,28	0,019916	0,475879	Cassette Exon	0,21
TC0200002558.mm.1	-1,2	Ncoa3	JUC0200010688.mm.1	3,28	0,011139	0,426226		
TC0200004548.mm.1	-1,14	Itpripl1	JUC0200020175.mm.1	3,28	0,023063	0,489502		
TC0100003191.mm.1	-1,36	Cfhr2; Gm16332	JUC0100014729.mm.1	3,28	0,033996	0,524346		
TC1500002035.mm.1	-3,26	1810021B22Rik	JUC1500009171.mm.1	3,28	0,021847	0,484607		
TC1500002035.mm.1	-3,26	1810021B22Rik	JUC1500009165.mm.1	2,79	0,011451	0,427811		
TC0600000308.mm.1	1,14	Exoc4	JUC0600001158.mm.1	3,28	0,031862	0,517902		
TC0300002779.mm.1	-1,15	Amy2-ps1	JUC0300011386.mm.1	3,28	0,015202	0,452738		
TC0300002674.mm.1	-1,04	Rap1a	JUC0300010838.mm.1	3,28	0,003762	0,357586		
TC1000000696.mm.1	3,9	Pcdh15	JUC1000002651.mm.1	3,27	0,033589	0,523246		
TC1000000696.mm.1	3,9	Pcdh15	JUC1000002634.mm.1	2,18	0,037345	0,533573		
TC1000000696.mm.1	3,9	Pcdh15	PSR1000004883.mm.1	-2,09	0,003928	0,359818		
TC1000000696.mm.1	3,9	Pcdh15	PSR1000004890.mm.1	-2,18	0,020761	0,479263		
TC1000000696.mm.1	3,9	Pcdh15	PSR1000004898.mm.1	-2,31	0,047024	0,557783	Cassette Exon	0,27
TC1000000696.mm.1	3,9	Pcdh15	PSR1000004855.mm.1	-2,55	0,036525	0,531352	Cassette Exon	0,35
TC1000000696.mm.1	3,9	Pcdh15	PSR1000004904.mm.1	-2,55	0,001222	0,322251	Cassette Exon	0,11
TC1000000696.mm.1	3,9	Pcdh15	PSR1000004922.mm.1	-2,57	0,031504	0,516965	Cassette Exon	0,18
TC1000000696.mm.1	3,9	Pcdh15	JUC1000002633.mm.1	-2,64	0,000342	0,289576		
TC1000000696.mm.1	3,9	Pcdh15	PSR1000004913.mm.1	-2,65	0,002653	0,349612	Alternative 3' Acce	0,26
TC1000000696.mm.1	3,9	Pcdh15	JUC1000002653.mm.1	-2,66	0,043356	0,549429		
TC1000000696.mm.1	3,9	Pcdh15	JUC1000002668.mm.1	-2,7	0,009698	0,417707		
TC1000000696.mm.1	3,9	Pcdh15	JUC1000002677.mm.1	-2,9	0,042327	0,547323		
TC1000000696.mm.1	3,9	Pcdh15	JUC1000002690.mm.1	-2,9	0,027566	0,50444		
TC1000000696.mm.1	3,9	Pcdh15	PSR1000004910.mm.1	-2,91	0,006206	0,387873	Cassette Exon	0,05
TC1000000696.mm.1	3,9	Pcdh15	JUC1000002687.mm.1	-3	0,00218	0,344453		
TC1000000696.mm.1	3,9	Pcdh15	JUC1000002682.mm.1	-3,05	0,011377	0,427487		
TC1000000696.mm.1	3,9	Pcdh15	PSR1000004903.mm.1	-3,13	0,030237	0,512468	Cassette Exon	0,17
TC1000000696.mm.1	3,9	Pcdh15	PSR1000004906.mm.1	-3,2	0,021457	0,482386	Alternative 5' Donc	0,22
TC1000000696.mm.1	3,9	Pcdh15	PSR1000004881.mm.1	-3,54	0,007105	0,396897	Cassette Exon	0,03
TC1000000696.mm.1	3,9	Pcdh15	JUC1000002683.mm.1	-3,56	0,03295	0,52122		
TC1000000696.mm.1	3,9	Pcdh15	PSR1000004889.mm.1	-3,73	0,015168	0,452699	Alternative 5' Donc	0,22
TC1000000696.mm.1	3,9	Pcdh15	JUC1000002638.mm.1	-3,73	0,00651	0,391307		
TC1000000696.mm.1	3,9	Pcdh15	JUC1000002674.mm.1	-3,78	0,042194	0,546939		
TC1000000696.mm.1	3,9	Pcdh15	JUC1000002688.mm.1	-4,09	0,022054	0,485327		
TC1000000696.mm.1	3,9	Pcdh15	PSR1000004923.mm.1	-4,13	0,007298	0,398847	Cassette Exon	0,28
TC1000000696.mm.1	3,9	Pcdh15	PSR1000004859.mm.1	-4,18	0,007913	0,403819	Alternative 3' Acce	0,48
TC1000000696.mm.1	3,9	Pcdh15	PSR1000004853.mm.1	-4,22	0,01092	0,424705	Cassette Exon	0,41
TC1000000696.mm.1	3,9	Pcdh15	JUC1000002658.mm.1	-4,96	0,020574	0,478487		
TC1000000696.mm.1	3,9	Pcdh15	PSR1000004852.mm.1	-5,16	0,036627	0,531552	Cassette Exon	0,41
TC1000000696.mm.1	3,9	Pcdh15	PSR1000004854.mm.1	-5,18	0,017389	0,463716	Cassette Exon	0,41
TC1000000696.mm.1	3,9	Pcdh15	JUC1000002639.mm.1	-5,63	0,002303	0,346364		
TC1000000696.mm.1	3,9	Pcdh15	JUC1000002652.mm.1	-5,65	0,002958	0,352501		
TC1000000696.mm.1	3,9	Pcdh15	JUC1000002637.mm.1	-6,06	0,024983	0,496025		
TC1000000696.mm.1	3,9	Pcdh15	JUC1000002679.mm.1	-6,1	0,023019	0,489186		
TC1000000696.mm.1	3,9	Pcdh15	JUC1000002669.mm.1	-6,18	0,008895	0,410717		
TC1000000696.mm.1	3,9	Pcdh15	PSR1000004856.mm.1	-6,25	0,007347	0,399281	Cassette Exon	0,41

TC1000000696.mm.1	3,9	Pcdh15	JUC1000002660.mm.1	-6,36	0,027881	0,505612		
TC1000000696.mm.1	3,9	Pcdh15	JUC1000002622.mm.1	-6,74	0,010273	0,420305		
TC1000000696.mm.1	3,9	Pcdh15	JUC1000002620.mm.1	-7,05	0,004451	0,367239		
TC1000000696.mm.1	3,9	Pcdh15	JUC1000002678.mm.1	-7,07	0,003072	0,353892		
TC1000000696.mm.1	3,9	Pcdh15	JUC1000002689.mm.1	-7,52	0,044352	0,551441		
TC1000000696.mm.1	3,9	Pcdh15	PSR1000004857.mm.1	-9,04	0,003208	0,354243	Cassette Exon	0,28
TC1000000696.mm.1	3,9	Pcdh15	JUC1000002686.mm.1	-11,93	0,006523	0,391502		
TC1000000696.mm.1	3,9	Pcdh15	JUC1000002670.mm.1	-17,7	0,001148	0,317344		
TC1000000696.mm.1	3,9	Pcdh15	JUC1000002666.mm.1	-18,9	0,000816	0,311886		
TC0500002528.mm.1	1,08	Atp8a1	JUC0500012248.mm.1	3,27	0,043047	0,54841		
TC0500002528.mm.1	1,08	Atp8a1	PSR0500022509.mm.1	2,11	0,032793	0,520798	Cassette Exon	0,21
TC0500002528.mm.1	1,08	Atp8a1	PSR0500022513.mm.1	-2,41	0,002846	0,352207	Alternative 3' Acce	0,24
TC0100003860.mm.1	-1,77	Lypla1	PSR0100000024.mm.1	3,27	0,005773	0,383104	Cassette Exon	0,06
TC0100003860.mm.1	-1,77	Lypla1	JUC0100000004.mm.1	2,96	0,013554	0,443605		
TC0100003860.mm.1	-1,77	Lypla1	JUC0100000012.mm.1	2,29	0,021417	0,482126		
TC0100003860.mm.1	-1,77	Lypla1	PSR0100000032.mm.1	2,12	0,009813	0,417839	Intron Retention	0,22
TC0100003860.mm.1	-1,77	Lypla1	PSR0100000045.mm.1	2,07	0,00074	0,307808	Cassette Exon	0,16
TC0100003860.mm.1	-1,77	Lypla1	JUC0100000008.mm.1	2,05	0,018337	0,468212		
TC0700003512.mm.1	1,17	St8sia2	JUC0700015711.mm.1	3,27	0,019254	0,473074		
TC0700003512.mm.1	1,17	St8sia2	PSR0700029771.mm.1	3,23	0,035162	0,528029	Cassette Exon	0,21
TC1600000242.mm.1	-1,41	Txnrd2	JUC1600001147.mm.1	3,27	0,00562	0,381231		
TC1600000242.mm.1	-1,41	Txnrd2	PSR1600002209.mm.1	2,16	0,042182	0,54689	Cassette Exon	0,11
TC1600000242.mm.1	-1,41	Txnrd2	PSR1600002227.mm.1	2,14	0,04823	0,560257	Cassette Exon	0,21
TC1400000501.mm.1	-1,83	Cdkn3	JUC1400002394.mm.1	3,27	0,022063	0,485335		
TC0200004425.mm.1	-1,07	Ppp1r14d	JUC0200018929.mm.1	3,27	0,000292	0,288663		
TC1500000641.mm.1	-1,23	Csf2rb; mmu-mir-7676-1; r	JUC1500002655.mm.1	3,27	0,028043	0,50586		
TC1700000659.mm.1	-1,15	Vwa7	JUC1700003669.mm.1	3,27	0,015302	0,45339		
TC1700000659.mm.1	-1,15	Vwa7	JUC1700003670.mm.1	2,38	0,002277	0,345991		
TC0900000189.mm.1	1,03	Pde4a	JUC0900000614.mm.1	3,27	0,000667	0,304088		
TC0800002021.mm.1	-1,15	Dctn6	PSR0800015827.mm.1	3,26	0,027848	0,505597	Alternative 3' Acce	0,33
TC0800002021.mm.1	-1,15	Dctn6	JUC0800008646.mm.1	2,43	0,009094	0,412827		
TC1200002523.mm.1	-2,22	Fntb	PSR1200004913.mm.1	3,26	0,026377	0,501011	Cassette Exon	0,26
TC1200002523.mm.1	-2,22	Fntb	PSR1200004899.mm.1	3,24	0,014928	0,451919	Cassette Exon	0,27
TC1200002523.mm.1	-2,22	Fntb	PSR1200004900.mm.1	2,51	0,012954	0,438936	Cassette Exon	0,07
TC1200002523.mm.1	-2,22	Fntb	PSR1200004905.mm.1	2,35	0,016724	0,460261	Cassette Exon	0,08
TC1200002523.mm.1	-2,22	Fntb	PSR1200004917.mm.1	2,2	0,025527	0,497712	Cassette Exon	0,09
TC1200002523.mm.1	-2,22	Fntb	PSR1200004912.mm.1	2,09	0,043507	0,549909	Cassette Exon	0,16
TC1200002523.mm.1	-2,22	Fntb	PSR1200004901.mm.1	2,01	0,03314	0,521898	Cassette Exon	0,05
TC1700000159.mm.1	-1,19	Mllt4	JUC1700000665.mm.1	3,26	0,034399	0,525736		
TC1700000159.mm.1	-1,19	Mllt4	JUC1700000672.mm.1	2,8	0,038602	0,536862		
TC1700000159.mm.1	-1,19	Mllt4	JUC1700000679.mm.1	2,71	0,002922	0,352289		
TC1700000159.mm.1	-1,19	Mllt4	PSR1700001240.mm.1	2,01	0,026145	0,500002	Cassette Exon	0,24
TC1000002249.mm.1	-1,11	Col13a1	PSR1000015940.mm.1	3,26	0,03279	0,520798	Cassette Exon	0,21
TC1000002249.mm.1	-1,11	Col13a1	PSR1000015946.mm.1	-2,67	0,028969	0,508766	Cassette Exon	0,11
TC1000001701.mm.1	-1,5	Ginm1; 6530403G13Rik	JUC1000006746.mm.1	3,26	0,046901	0,557454		
TC1000001701.mm.1	-1,5	Ginm1; 6530403G13Rik	PSR1000012449.mm.1	2,8	0,002121	0,342919	Alternative 3' Acce	0,07
TC0100001644.mm.1	-1,1	Spta1	JUC0100007719.mm.1	3,26	0,026706	0,502096		
TC0100001644.mm.1	-1,1	Spta1	JUC0100007720.mm.1	-2,43	0,012278	0,434165		

TC0200001798.mm.1	-1,23	Stard9	JUC0200006901.mm.1	3,26	0,030402	0,513014		
TC1600001400.mm.1	-1,06	Bcl6	JUC1600006056.mm.1	3,26	0,048934	0,5618		
TC0700000351.mm.1	1,11	Nlrp5	JUC0700001277.mm.1	3,26	0,035246	0,52837		
TC0700000351.mm.1	1,11	Nlrp5	JUC0700001276.mm.1	3,07	0,03343	0,522793		
TC0700000351.mm.1	1,11	Nlrp5	JUC0700001274.mm.1	2,15	0,0263	0,500634		
TC0700003559.mm.1	1,16	Polg	JUC0700015856.mm.1	3,26	0,007669	0,402377		
TC0700003559.mm.1	1,16	Polg	JUC0700015846.mm.1	2,5	0,025082	0,496264		
TC0700003559.mm.1	1,16	Polg	JUC0700015863.mm.1	-2,23	0,03126	0,516001		
TC0700003559.mm.1	1,16	Polg	JUC0700015865.mm.1	-2,33	0,000444	0,297771		
TC1100001204.mm.1	-1,05	Zfp207	JUC1100005948.mm.1	3,26	0,012444	0,43528		
TC1100001204.mm.1	-1,05	Zfp207	JUC1100005944.mm.1	2,91	0,01863	0,469941		
TC0700001795.mm.1	2,43	D430042O09Rik	PSR0700015292.mm.1	3,25	0,000212	0,28803	Mutually Exclusive	0,27
TC0700001795.mm.1	2,43	D430042O09Rik	JUC0700008041.mm.1	-2,38	0,038368	0,536303		
TC0700001795.mm.1	2,43	D430042O09Rik	PSR0700015290.mm.1	-2,41	0,045505	0,554359	Mutually Exclusive	0,27
TC0700001795.mm.1	2,43	D430042O09Rik	JUC0700008013.mm.1	-2,54	0,019723	0,474887		
TC0700001795.mm.1	2,43	D430042O09Rik	PSR0700015305.mm.1	-2,59	0,013937	0,446202	Alternative 5' Donc	0,16
TC0700001795.mm.1	2,43	D430042O09Rik	JUC0700008016.mm.1	-2,6	0,014161	0,447466		
TC0700001795.mm.1	2,43	D430042O09Rik	JUC0700008038.mm.1	-2,61	0,043188	0,548921		
TC0700001795.mm.1	2,43	D430042O09Rik	PSR0700015273.mm.1	-2,62	0,014416	0,448786	Alternative 5' Donc	0,14
TC0700001795.mm.1	2,43	D430042O09Rik	PSR0700015284.mm.1	-2,68	0,020221	0,477145	Cassette Exon	0,36
TC0700001795.mm.1	2,43	D430042O09Rik	PSR0700015287.mm.1	-2,76	0,011726	0,429969	Cassette Exon	0,32
TC0700001795.mm.1	2,43	D430042O09Rik	PSR0700015274.mm.1	-2,94	0,044754	0,552348	Alternative 5' Donc	0,14
TC0700001795.mm.1	2,43	D430042O09Rik	PSR0700015280.mm.1	-2,97	0,002838	0,352207	Cassette Exon	0,22
TC0700001795.mm.1	2,43	D430042O09Rik	JUC0700008017.mm.1	-2,98	0,012933	0,438537		
TC0700001795.mm.1	2,43	D430042O09Rik	PSR0700015285.mm.1	-3,07	0,018251	0,467881	Cassette Exon	0,33
TC0700001795.mm.1	2,43	D430042O09Rik	PSR0700015283.mm.1	-3,12	0,019039	0,471934	Cassette Exon	0,17
TC0700001795.mm.1	2,43	D430042O09Rik	JUC0700008019.mm.1	-3,25	0,038504	0,536557		
TC0700001795.mm.1	2,43	D430042O09Rik	PSR0700015282.mm.1	-3,36	0,007469	0,400381	Cassette Exon	0,29
TC0700001795.mm.1	2,43	D430042O09Rik	PSR0700015278.mm.1	-3,43	0,004896	0,372257	Cassette Exon	0,31
TC0700001795.mm.1	2,43	D430042O09Rik	JUC0700008040.mm.1	-3,66	0,039372	0,539409		
TC0700001795.mm.1	2,43	D430042O09Rik	PSR0700015286.mm.1	-3,98	0,010927	0,424768	Cassette Exon	0,35
TC0700001795.mm.1	2,43	D430042O09Rik	JUC0700008018.mm.1	-3,99	0,014601	0,449914		
TC0700001795.mm.1	2,43	D430042O09Rik	JUC0700008036.mm.1	-4,08	0,016661	0,460023		
TC0700001795.mm.1	2,43	D430042O09Rik	JUC0700008014.mm.1	-4,3	0,037513	0,533968		
TC0700001795.mm.1	2,43	D430042O09Rik	PSR0700015288.mm.1	-4,44	0,006014	0,386586	Cassette Exon	0,5
TC0700001795.mm.1	2,43	D430042O09Rik	PSR0700015277.mm.1	-4,71	0,004718	0,370888	Cassette Exon	0,46
TC0700001795.mm.1	2,43	D430042O09Rik	PSR0700015289.mm.1	-5,09	0,033311	0,522389	Cassette Exon	0,51
TC0700001795.mm.1	2,43	D430042O09Rik	JUC0700008009.mm.1	-5,93	0,010603	0,422777		
TC1100004164.mm.1	1,9	Rbfox3	JUC1100020824.mm.1	3,25	0,034882	0,526858		
TC1100004164.mm.1	1,9	Rbfox3	PSR1100039623.mm.1	-2,01	0,028404	0,507085	Cassette Exon	0,16
TC1100004164.mm.1	1,9	Rbfox3	PSR1100039615.mm.1	-2,2	0,008817	0,410077	Cassette Exon	0,12
TC1100004164.mm.1	1,9	Rbfox3	JUC1100020825.mm.1	-2,23	0,004475	0,367858		
TC1100004164.mm.1	1,9	Rbfox3	JUC1100020840.mm.1	-2,58	0,008757	0,409704		
TC1100004164.mm.1	1,9	Rbfox3	PSR1100039630.mm.1	-2,73	0,016428	0,459007	Cassette Exon	0,18
TC1100004164.mm.1	1,9	Rbfox3	PSR1100039627.mm.1	-2,76	0,016795	0,460623	Cassette Exon	0,16
TC1100004164.mm.1	1,9	Rbfox3	JUC1100020816.mm.1	-2,92	0,018797	0,4708		
TC1700001215.mm.1	-1,02	Arhgef33	JUC1700006220.mm.1	3,25	0,011898	0,431468		
TC1700001215.mm.1	-1,02	Arhgef33	PSR1700011386.mm.1	2,16	0,003768	0,357586	Cassette Exon	0,12

TC1300000765.mm.1	1,29		JUC1300002658.mm.1	3,25	0,027283	0,503732		
TC1000001275.mm.1	-1,37		JUC1000005173.mm.1	3,25	0,010437	0,421595		
TC1000000413.mm.1	-1,21	Atg5	JUC1000001532.mm.1	3,25	0,048448	0,560744		
TC0Y00000024.mm.1	-1,35	Gm20824	JUC0Y00000174.mm.1	3,25	0,01742	0,463792		
TC0300002207.mm.1	8,59	Gria2; Gm25749	JUC0300008717.mm.1	3,24	0,000251	0,28803		
TC0300002207.mm.1	8,59	Gria2; Gm25749	PSR0300016586.mm.1	-2,42	0,003276	0,354243	Cassette Exon	0,28
TC0300002207.mm.1	8,59	Gria2; Gm25749	PSR0300016581.mm.1	-2,52	0,002219	0,345773	Cassette Exon	0,21
TC0300002207.mm.1	8,59	Gria2; Gm25749	JUC0300008712.mm.1	-2,53	0,004199	0,363134		
TC0300002207.mm.1	8,59	Gria2; Gm25749	JUC0300008706.mm.1	-2,86	0,000906	0,313363		
TC0300002207.mm.1	8,59	Gria2; Gm25749	PSR0300016582.mm.1	-3,06	0,003268	0,354243	Cassette Exon	0,28
TC0300002207.mm.1	8,59	Gria2; Gm25749	JUC0300008715.mm.1	-3,28	0,016905	0,460797		
TC0300002207.mm.1	8,59	Gria2; Gm25749	JUC0300008711.mm.1	-3,45	0,008576	0,40821		
TC0300002207.mm.1	8,59	Gria2; Gm25749	PSR0300016584.mm.1	-3,84	0,000012	0,179072	Intron Retention	0,44
TC0300002207.mm.1	8,59	Gria2; Gm25749	JUC0300008713.mm.1	-3,92	0,00001	0,179072		
TC0300002207.mm.1	8,59	Gria2; Gm25749	PSR0300016592.mm.1	-4,06	0,003416	0,355257	Alternative 3' Acce	0,45
TC0300002207.mm.1	8,59	Gria2; Gm25749	PSR0300016590.mm.1	-4,31	0,001096	0,317328	Alternative 3' Acce	0,45
TC0300002207.mm.1	8,59	Gria2; Gm25749	JUC0300008724.mm.1	-4,58	0,006984	0,395722		
TC0300002207.mm.1	8,59	Gria2; Gm25749	PSR0300016576.mm.1	-4,63	0,000841	0,311886		
TC0300002207.mm.1	8,59	Gria2; Gm25749	JUC0300008720.mm.1	-5,25	0,004029	0,361636		
TC0300002207.mm.1	8,59	Gria2; Gm25749	JUC0300008710.mm.1	-5,75	0,000009	0,179072		
TC0300002207.mm.1	8,59	Gria2; Gm25749	PSR0300016580.mm.1	-6,34	0,001787	0,336311	Cassette Exon	0,52
TC0300002207.mm.1	8,59	Gria2; Gm25749	PSR0300016594.mm.1	-6,48	0,000306	0,288663	Intron Retention	0,68
TC0300002207.mm.1	8,59	Gria2; Gm25749	PSR0300016577.mm.1	-7,16	0,001153	0,317344	Cassette Exon	0,28
TC0300002207.mm.1	8,59	Gria2; Gm25749	PSR0300016605.mm.1	-7,29	0,001996	0,341091	Alternative 5' Donc	0,53
TC0300002207.mm.1	8,59	Gria2; Gm25749	PSR0300016585.mm.1	-7,29	0,000092	0,255472	Intron Retention	0,44
TC0300002207.mm.1	8,59	Gria2; Gm25749	PSR0300016589.mm.1	-8,77	0,003425	0,355303	Alternative 3' Acce	0,45
TC0300002207.mm.1	8,59	Gria2; Gm25749	PSR0300016607.mm.1	-8,81	0,000847	0,311909	Alternative 5' Donc	0,43
TC0300002207.mm.1	8,59	Gria2; Gm25749	PSR0300016608.mm.1	-11,12	0,000508	0,303579	Alternative 5' Donc	0,43
TC0300002207.mm.1	8,59	Gria2; Gm25749	JUC0300008723.mm.1	-46,02	0,00016	0,272566		
TC0300002207.mm.1	8,59	Gria2; Gm25749	JUC0300008721.mm.1	-49,94	0,000311	0,288663		
TC1200001018.mm.1	3,38	Chga	JUC1200003997.mm.1	3,24	0,008646	0,408463		
TC1200001018.mm.1	3,38	Chga	PSR1200007173.mm.1	-2,57	0,044012	0,550779	Cassette Exon	0,27
TC1200001018.mm.1	3,38	Chga	PSR1200007166.mm.1	-5,24	0,006727	0,393343	Alternative 3' Acce	0,48
TC1200001018.mm.1	3,38	Chga	JUC1200004000.mm.1	-5,64	0,000259	0,28803		
TC1200001018.mm.1	3,38	Chga	JUC1200004002.mm.1	-7,59	0,005475	0,379939		
TC0900002366.mm.1	-1,44	Commd4	PSR0900019814.mm.1	3,24	0,027234	0,503642	Alternative 3' Acce	0,31
TC0900000840.mm.1	2,8	Ubap1l	JUC0900003461.mm.1	3,24	0,020778	0,479405		
TC0900000840.mm.1	2,8	Ubap1l	PSR0900006412.mm.1	-2,28	0,02863	0,50782	Cassette Exon	0,18
TC0900000840.mm.1	2,8	Ubap1l	JUC0900003465.mm.1	-2,37	0,016797	0,460623		
TC0300001206.mm.1	3,11	4833424O15Rik	JUC0300005112.mm.1	3,24	0,000056	0,24627		
TC0300001206.mm.1	3,11	4833424O15Rik	JUC0300005115.mm.1	2,94	0,010005	0,418559		
TC0300001206.mm.1	3,11	4833424O15Rik	PSR0300009822.mm.1	-2,43	0,033867	0,524083	Cassette Exon	0,16
TC0300001206.mm.1	3,11	4833424O15Rik	JUC0300005114.mm.1	-3,03	0,006566	0,39213		
TC0300001206.mm.1	3,11	4833424O15Rik	PSR0300009820.mm.1	-3,49	0,009232	0,414336	Alternative 3' Acce	0,17
TC1200001231.mm.1	-1,15	Traf3	JUC1200004741.mm.1	3,24	0,00107	0,31648		
TC0100001733.mm.1	-1,1		JUC0100008149.mm.1	3,24	0,01454	0,449545		
TC0700004141.mm.1	-1,43	Cyp2r1	JUC0700018042.mm.1	3,24	0,004419	0,366832		
TC0700001660.mm.1	1,46	Arntl	JUC0700007129.mm.1	3,24	0,032919	0,521149		

TC0700001660.mm.1	1,46	Arntl	JUC0700007128.mm.1	2,69	0,00602	0,386586		
TC0900003264.mm.1	1,35	Ulk4	JUC0900015481.mm.1	3,24	0,001532	0,330287		
TC0900003264.mm.1	1,35	Ulk4	JUC0900015491.mm.1	2,05	0,045274	0,553703		
TC0900003264.mm.1	1,35	Ulk4	JUC0900015457.mm.1	-2,16	0,024607	0,494523		
TC0600001974.mm.1	3,19	Impdh1	JUC0600008113.mm.1	3,23	0,000689	0,304792		
TC0600001974.mm.1	3,19	Impdh1	JUC0600008096.mm.1	3,19	0,00037	0,291725		
TC0600001974.mm.1	3,19	Impdh1	PSR0600015512.mm.1	2,58	0,006129	0,387368	Cassette Exon	0,17
TC0600001974.mm.1	3,19	Impdh1	JUC0600008104.mm.1	2,34	0,032951	0,52122		
TC0600001974.mm.1	3,19	Impdh1	PSR0600015495.mm.1	2,33	0,007466	0,400381	Cassette Exon	0,32
TC0600001974.mm.1	3,19	Impdh1	JUC0600008109.mm.1	2,3	0,002341	0,347317		
TC0600001974.mm.1	3,19	Impdh1	PSR0600015493.mm.1	-2,01	0,024777	0,495014	Cassette Exon	0,09
TC0600001974.mm.1	3,19	Impdh1	PSR0600015507.mm.1	-2,12	0,000056	0,24627		
TC0600001974.mm.1	3,19	Impdh1	JUC0600008114.mm.1	-3,02	0,010155	0,419579		
TC0600001974.mm.1	3,19	Impdh1	JUC0600008097.mm.1	-3,85	0,000961	0,313363		
TC0600001974.mm.1	3,19	Impdh1	PSR0600015517.mm.1	-4,03	0,00315	0,353892	Alternative 5' Donc	0,61
TC0600001974.mm.1	3,19	Impdh1	PSR0600015509.mm.1	-4,56	0,000684	0,304511	Alternative 3' Acce	0,54
TC0600001974.mm.1	3,19	Impdh1	JUC0600008111.mm.1	-4,59	0,005011	0,373851		
TC0600001974.mm.1	3,19	Impdh1	JUC0600008115.mm.1	-5,88	0,00146	0,328595		
TC0500002989.mm.1	-1,3	Fbrsl1	JUC0500014439.mm.1	3,23	0,038359	0,536265		
TC0500002989.mm.1	-1,3	Fbrsl1	JUC0500014438.mm.1	2,63	0,025255	0,496939		
TC0500002989.mm.1	-1,3	Fbrsl1	PSR0500026462.mm.1	2,39	0,037618	0,534161	Cassette Exon	0,3
TC0500002989.mm.1	-1,3	Fbrsl1	PSR0500026463.mm.1	2,25	0,046323	0,556186	Cassette Exon	0,25
TC1400000829.mm.1	2,65	Fgf9	JUC1400003659.mm.1	3,23	0,003427	0,355303		
TC1400000829.mm.1	2,65	Fgf9	PSR1400006801.mm.1	2,01	0,02137	0,481977	Alternative 5' Donc	0,16
TC1400000829.mm.1	2,65	Fgf9	PSR1400006798.mm.1	-3,16	0,046426	0,556547	Cassette Exon	0,16
TC1200001233.mm.1	1,03	Amn	JUC1200004754.mm.1	3,23	0,028442	0,507234		
TC0700003027.mm.1	-1,56	Syngr4	JUC0700014676.mm.1	3,23	0,033316	0,522414		
TC0700003027.mm.1	-1,56	Syngr4	JUC0700014679.mm.1	2,42	0,03061	0,513498		
TC0600000240.mm.1	-1,01	Tspan33	JUC0600000845.mm.1	3,23	0,039458	0,539705		
TC1000002543.mm.1	1,63	Celf5	JUC1000010478.mm.1	3,23	0,027554	0,504437		
TC1000002543.mm.1	1,63	Celf5	JUC1000010489.mm.1	-2,71	0,018605	0,469657		
TC1000002543.mm.1	1,63	Celf5	JUC1000010481.mm.1	-3,77	0,000286	0,28803		
TC1700002724.mm.1	3,74	Nrxn1	JUC1700013277.mm.1	3,22	0,021507	0,482741		
TC1700002724.mm.1	3,74	Nrxn1	PSR1700024602.mm.1	2,75	0,001276	0,322251	Alternative 3' Acce	0,15
TC1700002724.mm.1	3,74	Nrxn1	JUC1700013258.mm.1	2,64	0,008569	0,40821		
TC1700002724.mm.1	3,74	Nrxn1	PSR1700024582.mm.1	-2,07	0,048671	0,561341	Cassette Exon	0,36
TC1700002724.mm.1	3,74	Nrxn1	PSR1700024567.mm.1	-2,24	0,040997	0,5436	Cassette Exon	0,32
TC1700002724.mm.1	3,74	Nrxn1	PSR1700024572.mm.1	-2,24	0,032556	0,520128	Cassette Exon	0,32
TC1700002724.mm.1	3,74	Nrxn1	PSR1700024580.mm.1	-2,3	0,037386	0,533689	Cassette Exon	0,32
TC1700002724.mm.1	3,74	Nrxn1	PSR1700024566.mm.1	-2,32	0,00818	0,40568	Cassette Exon	0,32
TC1700002724.mm.1	3,74	Nrxn1	PSR1700024619.mm.1	-2,44	0,013144	0,440196	Alternative 3' Acce	0,15
TC1700002724.mm.1	3,74	Nrxn1	JUC1700013275.mm.1	-2,58	0,005211	0,377078		
TC1700002724.mm.1	3,74	Nrxn1	PSR1700024606.mm.1	-2,66	0,026684	0,502057	Alternative 3' Acce	0,25
TC1700002724.mm.1	3,74	Nrxn1	PSR1700024579.mm.1	-2,68	0,011252	0,426718	Cassette Exon	0,32
TC1700002724.mm.1	3,74	Nrxn1	PSR1700024630.mm.1	-2,72	0,008274	0,405976	Alternative 3' Acce	0,35
TC1700002724.mm.1	3,74	Nrxn1	JUC1700013282.mm.1	-2,84	0,00143	0,327447		
TC1700002724.mm.1	3,74	Nrxn1	JUC1700013271.mm.1	-2,89	0,00246	0,349135		
TC1700002724.mm.1	3,74	Nrxn1	PSR1700024586.mm.1	-2,95	0,006111	0,387239	Cassette Exon	0,31

TC1700002724.mm.1	3,74	Nrxn1	PSR1700024593.mm.1	-3,08	0,007582	0,40171	Cassette Exon	0,33
TC1700002724.mm.1	3,74	Nrxn1	PSR1700024631.mm.1	-3,14	0,003095	0,353892	Alternative 3' Acce	0,35
TC1700002724.mm.1	3,74	Nrxn1	PSR1700024608.mm.1	-3,19	0,001751	0,335996	Alternative 5' Donc	0,35
TC1700002724.mm.1	3,74	Nrxn1	PSR1700024573.mm.1	-3,43	0,002695	0,349612	Cassette Exon	0,32
TC1700002724.mm.1	3,74	Nrxn1	JUC1700013254.mm.1	-3,53	0,005139	0,376442		
TC1700002724.mm.1	3,74	Nrxn1	PSR1700024585.mm.1	-3,77	0,00387	0,358872	Cassette Exon	0,17
TC1700002724.mm.1	3,74	Nrxn1	PSR1700024594.mm.1	-3,79	0,010136	0,419217	Cassette Exon	0,5
TC1700002724.mm.1	3,74	Nrxn1	JUC1700013288.mm.1	-3,82	0,001469	0,329052		
TC1700002724.mm.1	3,74	Nrxn1	JUC1700013280.mm.1	-3,86	0,012434	0,43528		
TC1700002724.mm.1	3,74	Nrxn1	PSR1700024569.mm.1	-4,03	0,005311	0,378059	Cassette Exon	0,32
TC1700002724.mm.1	3,74	Nrxn1	PSR1700024568.mm.1	-4,54	0,024716	0,494838	Cassette Exon	0,32
TC1700002724.mm.1	3,74	Nrxn1	PSR1700024587.mm.1	-4,61	0,013262	0,441524	Cassette Exon	0,35
TC1700002724.mm.1	3,74	Nrxn1	JUC1700013259.mm.1	-4,76	0,002845	0,352207		
TC1700002724.mm.1	3,74	Nrxn1	PSR1700024632.mm.1	-4,94	0,004524	0,368648	Alternative 3' Acce	0,35
TC1700002724.mm.1	3,74	Nrxn1	PSR1700024565.mm.1	-5,08	0,007616	0,401872	Cassette Exon	0,32
TC1700002724.mm.1	3,74	Nrxn1	PSR1700024596.mm.1	-5,11	0,002818	0,352207	Cassette Exon	0,39
TC1700002724.mm.1	3,74	Nrxn1	PSR1700024589.mm.1	-5,24	0,005609	0,381115	Alternative 3' Acce	0,33
TC1700002724.mm.1	3,74	Nrxn1	JUC1700013285.mm.1	-5,8	0,005896	0,383835		
TC1700002724.mm.1	3,74	Nrxn1	JUC1700013253.mm.1	-7	0,003992	0,360753		
TC0300003122.mm.1	1,51	Lphn2	PSR0300024526.mm.1	3,22	0,001033	0,316361	Cassette Exon	0,01
TC0300003122.mm.1	1,51	Lphn2	JUC0300012871.mm.1	-2,32	0,001267	0,322251		
TC0300003122.mm.1	1,51	Lphn2	JUC0300012869.mm.1	-2,42	0,011574	0,429058		
TC0300003122.mm.1	1,51	Lphn2	PSR0300024524.mm.1	-2,86	0,003084	0,353892	Cassette Exon	0,31
TC0300003122.mm.1	1,51	Lphn2	PSR0300024529.mm.1	-3,22	0,001033	0,316361	Cassette Exon	0,34
TC0300003122.mm.1	1,51	Lphn2	JUC0300012870.mm.1	-4,08	0,00943	0,416081		
TC0300003122.mm.1	1,51	Lphn2	JUC0300012868.mm.1	-7,61	0,002787	0,351436		
TC0X00003278.mm.1	-1,26	Cdkl5	JUC0X00010350.mm.1	3,22	0,047653	0,558801		
TC0X00003278.mm.1	-1,26	Cdkl5	JUC0X00010358.mm.1	2,79	0,01809	0,46716		
TC0X00003278.mm.1	-1,26	Cdkl5	PSR0X00020508.mm.1	2,69	0,006867	0,394135	Cassette Exon	0,3
TC0X00003278.mm.1	-1,26	Cdkl5	PSR0X00020495.mm.1	2,69	0,007422	0,399901	Cassette Exon	0,14
TC0X00003278.mm.1	-1,26	Cdkl5	PSR0X00020514.mm.1	2,65	0,008884	0,410642	Cassette Exon	0,24
TC0X00003278.mm.1	-1,26	Cdkl5	PSR0X00020511.mm.1	2,64	0,008288	0,406032	Cassette Exon	0,17
TC0X00003278.mm.1	-1,26	Cdkl5	JUC0X00010354.mm.1	2,51	0,013937	0,446202		
TC0X00003278.mm.1	-1,26	Cdkl5	JUC0X00010355.mm.1	2,42	0,026759	0,502096		
TC0X00003278.mm.1	-1,26	Cdkl5	PSR0X00020512.mm.1	2,39	0,004336	0,364898	Cassette Exon	0,21
TC0X00003278.mm.1	-1,26	Cdkl5	PSR0X00020510.mm.1	2,27	0,011516	0,42831	Cassette Exon	0,16
TC0X00003278.mm.1	-1,26	Cdkl5	JUC0X00010357.mm.1	2,18	0,030887	0,514574		
TC0X00003278.mm.1	-1,26	Cdkl5	PSR0X00020515.mm.1	2,17	0,013912	0,445966	Cassette Exon	0,19
TC0X00003278.mm.1	-1,26	Cdkl5	PSR0X00020513.mm.1	2,16	0,005534	0,380557	Cassette Exon	0,24
TC0X00003278.mm.1	-1,26	Cdkl5	PSR0X00020505.mm.1	2,1	0,003716	0,357586	Cassette Exon	0,12
TC0X00003278.mm.1	-1,26	Cdkl5	JUC0X00010351.mm.1	2,08	0,009624	0,417315		
TC1400000096.mm.1	-1,09	Ptprg	JUC1400000490.mm.1	3,22	0,012486	0,435429		
TC1400000096.mm.1	-1,09	Ptprg	JUC1400000479.mm.1	2,44	0,014313	0,448078		
TC1400000096.mm.1	-1,09	Ptprg	PSR1400000769.mm.1	2,24	0,037866	0,534863	Alternative 3' Acce	0,2
TC1200000765.mm.1	1,09	Pcnx	JUC1200003079.mm.1	3,22	0,021978	0,485125		
TC1200000765.mm.1	1,09	Pcnx	PSR1200005378.mm.1	2,15	0,026505	0,50132	Cassette Exon	0,11
TC0200005110.mm.1	-1,34	Svs3b	JUC0200022457.mm.1	3,22	0,015873	0,456526		
TC0100002381.mm.1	-1,73	Clk1	JUC0100010968.mm.1	3,22	0,009967	0,418446		

TC0600002923.mm.1	1,15	Prrt3	JUC0600011647.mm.1	3,22	0,028061	0,505951		
TC0700002708.mm.1	-1,28	Actn4	JUC0700012632.mm.1	3,22	0,023118	0,489756		
TC0400000240.mm.1	-1,03		JUC0400000705.mm.1	3,22	0,008372	0,406495		
TC1200001142.mm.1	2,42	Rian; AF357355; AF35735	PSR1200008236.mm.1	3,21	0,020618	0,478652	Cassette Exon	0,27
TC1200001142.mm.1	2,42	Rian; AF357355; AF35735	PSR1200008178.mm.1	3,11	0,000314	0,288663	Alternative 3' Acce	0,07
TC1200001142.mm.1	2,42	Rian; AF357355; AF35735	PSR1200008216.mm.1	2,76	0,006166	0,387873		
TC1200001142.mm.1	2,42	Rian; AF357355; AF35735	JUC1200004504.mm.1	2,71	0,039742	0,540494		
TC1200001142.mm.1	2,42	Rian; AF357355; AF35735	PSR1200008179.mm.1	2,67	0,00509	0,375607	Cassette Exon	0,12
TC1200001142.mm.1	2,42	Rian; AF357355; AF35735	PSR1200008170.mm.1	2,57	0,042838	0,548097	Cassette Exon	0,14
TC1200001142.mm.1	2,42	Rian; AF357355; AF35735	JUC1200004528.mm.1	2,37	0,018553	0,469453		
TC1200001142.mm.1	2,42	Rian; AF357355; AF35735	JUC1200004527.mm.1	2,32	0,005855	0,383747		
TC1200001142.mm.1	2,42	Rian; AF357355; AF35735	PSR1200008237.mm.1	2,28	0,017446	0,463886	Cassette Exon	0,18
TC1200001142.mm.1	2,42	Rian; AF357355; AF35735	PSR1200008217.mm.1	2,28	0,007868	0,403545	Cassette Exon	0,1
TC1200001142.mm.1	2,42	Rian; AF357355; AF35735	PSR1200008165.mm.1	-2,02	0,036519	0,531352	Alternative 3' Acce	0,17
TC1200001142.mm.1	2,42	Rian; AF357355; AF35735	PSR1200008227.mm.1	-2,16	0,042771	0,548097	Intron Retention	0,17
TC1200001142.mm.1	2,42	Rian; AF357355; AF35735	PSR1200008230.mm.1	-2,24	0,00036	0,290838		
TC1200001142.mm.1	2,42	Rian; AF357355; AF35735	PSR1200008244.mm.1	-2,29	0,018088	0,46716	Alternative 3' Acce	0,21
TC1200001142.mm.1	2,42	Rian; AF357355; AF35735	JUC1200004505.mm.1	-2,37	0,006041	0,386586		
TC1200001142.mm.1	2,42	Rian; AF357355; AF35735	PSR1200008176.mm.1	-2,71	0,00357	0,356223	Cassette Exon	0,16
TC1200001142.mm.1	2,42	Rian; AF357355; AF35735	JUC1200004535.mm.1	-2,82	0,023544	0,490947		
TC1200001142.mm.1	2,42	Rian; AF357355; AF35735	JUC1200004542.mm.1	-2,91	0,018006	0,466939		
TC1200001142.mm.1	2,42	Rian; AF357355; AF35735	JUC1200004509.mm.1	-3,02	0,015591	0,454982		
TC1200001142.mm.1	2,42	Rian; AF357355; AF35735	PSR1200008218.mm.1	-3,16	0,00064	0,304044	Intron Retention	0,37
TC1200001142.mm.1	2,42	Rian; AF357355; AF35735	PSR1200008200.mm.1	-3,28	0,02224	0,485896		
TC1200001142.mm.1	2,42	Rian; AF357355; AF35735	PSR1200008145.mm.1	-3,29	0,011497	0,428152	Alternative 3' Acce	0,05
TC1200001142.mm.1	2,42	Rian; AF357355; AF35735	PSR1200008152.mm.1	-3,56	0,000052	0,24627	Cassette Exon	0,18
TC1200001142.mm.1	2,42	Rian; AF357355; AF35735	JUC1200004513.mm.1	-4,24	0,013597	0,443703		
TC1800000266.mm.1	1,31	Camk4	PSR1800001950.mm.1	3,21	0,00002	0,211693	Cassette Exon	0,2
TC1800000266.mm.1	1,31	Camk4	JUC1800001160.mm.1	2,44	0,008496	0,407077		
TC0Y00000212.mm.1	-1,35	Gm21477; Gm21627; Gm2	JUC0Y00000851.mm.1	3,21	0,012333	0,434392		
TC0Y00000212.mm.1	-1,35	Gm21477; Gm21627; Gm2	PSR0Y00001166.mm.1	-2,46	0,023841	0,491997	Cassette Exon	0,13
TC0Y00000319.mm.1	-1,35	Gm20896; Gm21627; Gm2	JUC0Y00001484.mm.1	3,21	0,012333	0,434392		
TC0Y00000319.mm.1	-1,35	Gm20896; Gm21627; Gm2	PSR0Y00001948.mm.1	-2,46	0,023841	0,491997	Cassette Exon	0,13
TC0100000302.mm.1	-1,17	Slc9a2	JUC0100001288.mm.1	3,21	0,002603	0,349501		
TC0100000302.mm.1	-1,17	Slc9a2	PSR0100002274.mm.1	2,19	0,022761	0,487938	Cassette Exon	0,11
TC0200003103.mm.1	-1,03	Mastl	JUC0200012809.mm.1	3,21	0,047334	0,558291		
TC0100001031.mm.1	-1,59	Serpinb8	JUC0100004945.mm.1	3,21	0,036674	0,531675		
TC0100000307.mm.1	-1,03		JUC0100001307.mm.1	3,21	0,036386	0,530857		
TC0700000301.mm.1	-2,27	Ckm	JUC0700001175.mm.1	3,21	0,03166	0,517405		
TC1100002007.mm.1	1,04	Rptor	JUC1100010048.mm.1	3,21	0,0217	0,484104		
TC1100002007.mm.1	1,04	Rptor	JUC1100010081.mm.1	-3	0,021028	0,480239		
TC0600003060.mm.1	4,29	Iqsec3	JUC0600012338.mm.1	3,2	0,000689	0,304792		
TC0600003060.mm.1	4,29	Iqsec3	PSR0600023661.mm.1	2,37	0,002175	0,344453	Cassette Exon	0,11
TC0600003060.mm.1	4,29	Iqsec3	PSR0600023657.mm.1	2,18	0,003716	0,357586	Cassette Exon	0,15
TC0600003060.mm.1	4,29	Iqsec3	PSR0600023653.mm.1	-2,23	0,01429	0,448073	Cassette Exon	0,12
TC0600003060.mm.1	4,29	Iqsec3	JUC0600012334.mm.1	-3,32	0,000041	0,24627		
TC0600003060.mm.1	4,29	Iqsec3	PSR0600023664.mm.1	-3,82	0,005891	0,383835	Alternative 3' Acce	0,34
TC0600003060.mm.1	4,29	Iqsec3	PSR0600023655.mm.1	-4,56	0,012897	0,438165	Cassette Exon	0,48

TC0600003060.mm.1	4,29	Iqsec3	PSR0600023667.mm.1	-4,59	0,0021	0,342919	Cassette Exon	0,35
TC0600003060.mm.1	4,29	Iqsec3	PSR0600023669.mm.1	-4,7	0,003627	0,356534	Cassette Exon	0,25
TC0600003060.mm.1	4,29	Iqsec3	PSR0600023670.mm.1	-5,31	0,00555	0,380756	Cassette Exon	0,25
TC0600003060.mm.1	4,29	Iqsec3	PSR0600023671.mm.1	-6,15	0,002128	0,342919	Cassette Exon	0,25
TC0600003060.mm.1	4,29	Iqsec3	JUC0600012330.mm.1	-6,5	0,006431	0,389972		
TC1000001797.mm.1	-1,62	Gm20655	JUC1000007118.mm.1	3,2	0,000509	0,303579		
TC1000001797.mm.1	-1,62	Gm20655	PSR1000013058.mm.1	3,13	0,021051	0,480262	Cassette Exon	0,21
TC1000001797.mm.1	-1,62	Gm20655	PSR1000013066.mm.1	3,05	0,016418	0,458966	Cassette Exon	0,29
TC1600001335.mm.1	2,65	A930003A15Rik	PSR1600011001.mm.1	3,2	0,007814	0,403089	Cassette Exon	0,29
TC1600001335.mm.1	2,65	A930003A15Rik	PSR1600011007.mm.1	-3,19	0,001365	0,32572	Alternative 5' Donc	0,25
TC0800002066.mm.1	-1,02	Dlc1	JUC0800008757.mm.1	3,2	0,025716	0,498173		
TC0800002066.mm.1	-1,02	Dlc1	JUC0800008758.mm.1	2,2	0,000055	0,24627		
TC0800002066.mm.1	-1,02	Dlc1	JUC0800008756.mm.1	2,12	0,023992	0,492783		
TC0800002066.mm.1	-1,02	Dlc1	PSR0800016042.mm.1	2,02	0,014147	0,447466	Cassette Exon	0,24
TC0X00001829.mm.1	-1,66	Cybb	JUC0X00005926.mm.1	3,2	0,022707	0,487767		
TC0X00001829.mm.1	-1,66	Cybb	JUC0X00005919.mm.1	2,56	0,024018	0,492809		
TC0X00001829.mm.1	-1,66	Cybb	PSR0X00011736.mm.1	2,54	0,020521	0,478264	Cassette Exon	0,24
TC0X00001829.mm.1	-1,66	Cybb	PSR0X00011713.mm.1	2,27	0,030956	0,514654	Alternative 5' Donc	0,06
TC0X00001829.mm.1	-1,66	Cybb	PSR0X00011727.mm.1	-2,06	0,014185	0,447608	Cassette Exon	0,08
TC0600003337.mm.1	-2,36	Arhgdib	PSR0600026200.mm.1	3,2	0,009073	0,412827	Cassette Exon	0,2
TC0600003337.mm.1	-2,36	Arhgdib	PSR0600026201.mm.1	2,63	0,011341	0,427345	Cassette Exon	0,17
TC0600003337.mm.1	-2,36	Arhgdib	PSR0600026207.mm.1	2,4	0,039724	0,540494	Cassette Exon	0,18
TC0100003743.mm.1	1,66	Mark1	JUC0100017412.mm.1	3,2	0,01239	0,434939		
TC0100003743.mm.1	1,66	Mark1	JUC0100017418.mm.1	2,02	0,01892	0,471305		
TC0100003743.mm.1	1,66	Mark1	PSR0100030667.mm.1	-2,04	0,042729	0,548097	Cassette Exon	0,09
TC1700000625.mm.1	-1,55	H2-Ob	JUC1700003307.mm.1	3,2	0,014481	0,449332		
TC0800001349.mm.1	-1,29	Cntnap4	JUC0800006059.mm.1	3,2	0,003636	0,3566		
TC0800001349.mm.1	-1,29	Cntnap4	JUC0800006066.mm.1	2,35	0,021586	0,483245		
TC0800001349.mm.1	-1,29	Cntnap4	JUC0800006069.mm.1	2,07	0,022892	0,488524		
TC0500001719.mm.1	1,22	Slc29a4	JUC0500008580.mm.1	3,2	0,036861	0,532257		
TC0500001719.mm.1	1,22	Slc29a4	JUC0500008578.mm.1	2,41	0,007235	0,39826		
TC0200002427.mm.1	5,04	Snhg11; Gm25187	PSR0200019714.mm.1	3,19	0,004129	0,3623	Alternative 5' Donc	0,16
TC0200002427.mm.1	5,04	Snhg11; Gm25187	PSR0200019715.mm.1	2,83	0,008953	0,411469	Alternative 5' Donc	0,16
TC0200002427.mm.1	5,04	Snhg11; Gm25187	PSR0200019716.mm.1	2,38	0,033948	0,524252	Alternative 5' Donc	0,16
TC0200002427.mm.1	5,04	Snhg11; Gm25187	PSR0200019699.mm.1	2,04	0,002978	0,352501	Cassette Exon	0,16
TC0200002427.mm.1	5,04	Snhg11; Gm25187	PSR0200019708.mm.1	-2,84	0,01873	0,470733	Alternative 5' Donc	0,27
TC0200002427.mm.1	5,04	Snhg11; Gm25187	PSR0200019705.mm.1	-3,16	0,027672	0,504723	Alternative 5' Donc	0,27
TC0200002427.mm.1	5,04	Snhg11; Gm25187	PSR0200019707.mm.1	-3,16	0,007938	0,403819	Alternative 5' Donc	0,27
TC0200002427.mm.1	5,04	Snhg11; Gm25187	PSR0200019700.mm.1	-3,4	0,001264	0,322251	Intron Retention	0,6
TC0200002427.mm.1	5,04	Snhg11; Gm25187	PSR0200019703.mm.1	-3,42	0,000209	0,28803	Intron Retention	0,54
TC0200002427.mm.1	5,04	Snhg11; Gm25187	PSR0200019693.mm.1	-3,42	0,018608	0,469665	Alternative 3' Acce	0,38
TC1000002316.mm.1	7,35	Zfp365	PSR1000016542.mm.1	3,19	0,00809	0,405014	Cassette Exon	0,07
TC1000002316.mm.1	7,35	Zfp365	PSR1000016541.mm.1	2,27	0,035873	0,529376	Cassette Exon	0,12
TC1000002316.mm.1	7,35	Zfp365	PSR1000016547.mm.1	-3,98	0,030356	0,512923	Cassette Exon	0,28
TC1000002316.mm.1	7,35	Zfp365	JUC1000009049.mm.1	-5,55	0,003716	0,357586		
TC1000002316.mm.1	7,35	Zfp365	PSR1000016543.mm.1	-5,79	0,001035	0,316361	Alternative 3' Acce	0,34
TC1000002316.mm.1	7,35	Zfp365	PSR1000016545.mm.1	-6,59	0,008845	0,410308	Cassette Exon	0,38
TC1000002316.mm.1	7,35	Zfp365	PSR1000016551.mm.1	-7,58	0,006041	0,386586	Cassette Exon	0,28

TC1000002316.mm.1	7,35	Zfp365	PSR1000016548.mm.1	-8,31	0,003059	0,353892	Cassette Exon	0,28
TC0800002560.mm.1	1,09	Gm26721	PSR0800019510.mm.1	3,19	0,022296	0,486095	Alternative 5' Donc	0,35
TC1800001744.mm.1	1,01	Arhgap26	JUC1800001704.mm.1	3,19	0,000157	0,272178		
TC1800001744.mm.1	1,01	Arhgap26	PSR1800003052.mm.1	2,81	0,007966	0,403853	Cassette Exon	0,28
TC1800001744.mm.1	1,01	Arhgap26	JUC1800001687.mm.1	2,45	0,012485	0,435429		
TC1800001744.mm.1	1,01	Arhgap26	JUC1800001696.mm.1	2,44	0,00544	0,379743		
TC1800001744.mm.1	1,01	Arhgap26	PSR1800003055.mm.1	2,21	0,003509	0,355749	Cassette Exon	0,11
TC1800001744.mm.1	1,01	Arhgap26	PSR1800003058.mm.1	2,17	0,00758	0,40171	Cassette Exon	0,12
TC1800001744.mm.1	1,01	Arhgap26	JUC1800001686.mm.1	2,1	0,019856	0,475698		
TC1100002993.mm.1	1,71	Dnah9	PSR1100027161.mm.1	3,19	0,001261	0,322251	Cassette Exon	0,26
TC1100002993.mm.1	1,71	Dnah9	PSR1100027135.mm.1	2,72	0,000931	0,313363	Cassette Exon	0,18
TC1100002993.mm.1	1,71	Dnah9	PSR1100027158.mm.1	2,44	0,027405	0,503961	Cassette Exon	0,17
TC1100002993.mm.1	1,71	Dnah9	PSR1100027153.mm.1	-2,14	0,025363	0,497403	Cassette Exon	0,13
TC1100002993.mm.1	1,71	Dnah9	JUC1100014131.mm.1	-2,14	0,001137	0,317344		
TC1100002993.mm.1	1,71	Dnah9	JUC1100014192.mm.1	-2,33	0,012141	0,432868		
TC1100002993.mm.1	1,71	Dnah9	PSR1100027151.mm.1	-2,41	0,00204	0,341377	Cassette Exon	0,19
TC1100002993.mm.1	1,71	Dnah9	JUC1100014127.mm.1	-2,46	0,022833	0,488349		
TC1100002993.mm.1	1,71	Dnah9	PSR1100027096.mm.1	-2,5	0,003233	0,354243	Cassette Exon	0,2
TC1100002993.mm.1	1,71	Dnah9	PSR1100027152.mm.1	-2,54	0,021792	0,484434	Cassette Exon	0,17
TC1100002993.mm.1	1,71	Dnah9	PSR1100027095.mm.1	-2,65	0,002926	0,352289	Cassette Exon	0,23
TC0200003245.mm.1	2,22	Ntng2; 6530402F18Rik	PSR0200027399.mm.1	3,19	0,012184	0,433072	Alternative 5' Donc	0,23
TC0200003245.mm.1	2,22	Ntng2; 6530402F18Rik	PSR0200027398.mm.1	2,59	0,046299	0,556186	Alternative 5' Donc	0,23
TC0200003245.mm.1	2,22	Ntng2; 6530402F18Rik	PSR0200027391.mm.1	2,41	0,000723	0,306174	Alternative 3' Acce	0,13
TC0200003245.mm.1	2,22	Ntng2; 6530402F18Rik	PSR0200027403.mm.1	2,27	0,002893	0,352207	Alternative 5' Donc	0,2
TC0200003245.mm.1	2,22	Ntng2; 6530402F18Rik	PSR0200027393.mm.1	2,23	0,016973	0,461419	Alternative 3' Acce	0,13
TC0200003245.mm.1	2,22	Ntng2; 6530402F18Rik	PSR0200027400.mm.1	2,02	0,006961	0,395722	Alternative 5' Donc	0,2
TC0200003245.mm.1	2,22	Ntng2; 6530402F18Rik	PSR0200027378.mm.1	-2,15	0,033649	0,523479	Alternative 3' Acce	0,12
TC0200003245.mm.1	2,22	Ntng2; 6530402F18Rik	PSR0200027389.mm.1	-2,75	0,013397	0,442454	Cassette Exon	0,25
TC0200003245.mm.1	2,22	Ntng2; 6530402F18Rik	PSR0200027375.mm.1	-2,86	0,037758	0,534682	Alternative 3' Acce	0,1
TC0200003245.mm.1	2,22	Ntng2; 6530402F18Rik	JUC0200013852.mm.1	-2,92	0,011629	0,429746		
TC0200003245.mm.1	2,22	Ntng2; 6530402F18Rik	JUC0200013847.mm.1	-3,16	0,012919	0,438344		
TC0200003245.mm.1	2,22	Ntng2; 6530402F18Rik	JUC0200013855.mm.1	-3,57	0,005337	0,378059		
TC1500000139.mm.1	3,73	Cdh12	JUC1500000681.mm.1	3,19	0,000406	0,297771		
TC1500000139.mm.1	3,73	Cdh12	PSR1500001154.mm.1	2,23	0,007692	0,402552	Cassette Exon	0,14
TC1500000139.mm.1	3,73	Cdh12	JUC1500000682.mm.1	2,06	0,024112	0,49322		
TC1500000139.mm.1	3,73	Cdh12	PSR1500001145.mm.1	-2,03	0,006891	0,394558	Cassette Exon	0,17
TC1500000139.mm.1	3,73	Cdh12	PSR1500001155.mm.1	-2,21	0,009804	0,417839	Cassette Exon	0,13
TC1500000139.mm.1	3,73	Cdh12	JUC1500000674.mm.1	-2,56	0,001218	0,322251		
TC1500000139.mm.1	3,73	Cdh12	JUC1500000675.mm.1	-6,59	0,000491	0,298999		
TC0700000896.mm.1	-1,16	Nav2; Gm2788; LOC54596	JUC0700004051.mm.1	3,19	0,021103	0,480467		
TC0700000896.mm.1	-1,16	Nav2; Gm2788; LOC54596	PSR0700008015.mm.1	-2,38	0,009973	0,418446	Alternative 5' Donc	0,16
TC0200002359.mm.1	1,26	Itch	JUC0200009498.mm.1	3,19	0,001238	0,322251		
TC0200002359.mm.1	1,26	Itch	JUC0200009515.mm.1	-2,06	0,006162	0,387873		
TC0200002359.mm.1	1,26	Itch	PSR0200018773.mm.1	-2,13	0,002489	0,349135	Alternative 3' Acce	0,14
TC0200002359.mm.1	1,26	Itch	JUC0200009517.mm.1	-2,37	0,027359	0,503858		
TC0200000872.mm.1	-1,07	Galnt5	JUC0200003573.mm.1	3,19	0,045054	0,553098		
TC1000001704.mm.1	-1,09	Tab2	JUC1000006759.mm.1	3,19	0,022877	0,488524		
TC0700003361.mm.1	1,65	A230006K03Rik	PSR0700028769.mm.1	3,18	0,014494	0,449491	Cassette Exon	0,29

TC0700003361.mm.1	1,65	A230006K03Rik	PSR0700028804.mm.1	2,07	0,011404	0,427549	Mutually Exclusive	0,2
TC0700003361.mm.1	1,65	A230006K03Rik	PSR0700028796.mm.1	2,07	0,011404	0,427549	Mutually Exclusive	0,18
TC0700003361.mm.1	1,65	A230006K03Rik	PSR0700028795.mm.1	2,04	0,043973	0,550752	Cassette Exon	0,11
TC0700003361.mm.1	1,65	A230006K03Rik	PSR0700028797.mm.1	-2,07	0,011404	0,427549	Mutually Exclusive	0,18
TC0700003361.mm.1	1,65	A230006K03Rik	JUC0700015234.mm.1	-2,08	0,000154	0,272178		
TC0700003361.mm.1	1,65	A230006K03Rik	JUC0700015209.mm.1	-2,13	0,009736	0,417839		
TC0700003361.mm.1	1,65	A230006K03Rik	JUC0700015231.mm.1	-2,24	0,004888	0,372195		
TC0700003361.mm.1	1,65	A230006K03Rik	JUC0700015219.mm.1	-2,27	0,00944	0,416119		
TC0700003361.mm.1	1,65	A230006K03Rik	PSR0700028793.mm.1	-2,33	0,004471	0,367776	Alternative 3' Acce	0,15
TC0700003361.mm.1	1,65	A230006K03Rik	PSR0700028771.mm.1	-2,4	0,009847	0,417839	Cassette Exon	0,13
TC0700003361.mm.1	1,65	A230006K03Rik	JUC0700015205.mm.1	-2,5	0,029329	0,509981		
TC0700003361.mm.1	1,65	A230006K03Rik	JUC0700015233.mm.1	-2,87	0,009333	0,414973		
TC0700003361.mm.1	1,65	A230006K03Rik	JUC0700015226.mm.1	-3,03	0,003933	0,359818		
TC0700003361.mm.1	1,65	A230006K03Rik	PSR0700028805.mm.1	-3,05	0,00029	0,288663	Mutually Exclusive	0,2
TC0700003361.mm.1	1,65	A230006K03Rik	JUC0700015235.mm.1	-3,29	0,005669	0,381983		
TC0700003361.mm.1	1,65	A230006K03Rik	JUC0700015215.mm.1	-4,11	0,047852	0,559373		
TC1200000641.mm.1	-1,04	Slc38a6	JUC1200002466.mm.1	3,18	0,00964	0,417354		
TC1200000641.mm.1	-1,04	Slc38a6	JUC1200002480.mm.1	2,72	0,01037	0,421259		
TC1200000641.mm.1	-1,04	Slc38a6	JUC1200002467.mm.1	2,49	0,018984	0,471445		
TC1200000641.mm.1	-1,04	Slc38a6	JUC1200002477.mm.1	2,22	0,033728	0,523549		
TC1200000641.mm.1	-1,04	Slc38a6	PSR1200004410.mm.1	2,03	0,028317	0,506741	Cassette Exon	0,27
TC1200000641.mm.1	-1,04	Slc38a6	JUC1200002469.mm.1	-3,48	0,01459	0,449914		
TC0400002460.mm.1	1,33	Ubap2; Gm23090	PSR0400019865.mm.1	3,18	0,02899	0,508842	Cassette Exon	0,23
TC1900001128.mm.1	-1,33	Ms4a1	JUC1900005800.mm.1	3,18	0,001046	0,316361		
TC1900001590.mm.1	1,4	Kcnip2	JUC1900007803.mm.1	3,18	0,034518	0,525929		
TC0100000697.mm.1	-1,03	Nyap2	JUC0100003403.mm.1	3,18	0,001716	0,335996		
TC0700000908.mm.1	1,09	Nell1	JUC0700004109.mm.1	3,18	0,018409	0,468484		
TC0600002209.mm.1	-1,3		JUC0600009121.mm.1	3,18	0,044951	0,552878		
TC0700002168.mm.1	-1,16	Tmem150b	JUC0700010534.mm.1	3,18	0,005982	0,386153		
TC0X00001390.mm.1	-1,4	Atg4a	JUC0X00004312.mm.1	3,18	0,021101	0,480439		
TC0X00001390.mm.1	-1,4	Atg4a	JUC0X00004323.mm.1	2,14	0,003343	0,354243		
TC0400002362.mm.1	-1,41	Bach2os; BC024582	JUC0400010010.mm.1	3,18	0,002887	0,352207		
TC0300001493.mm.1	-1,59	Gng5; Gm15776	PSR0300012103.mm.1	3,17	0,026859	0,502454	Intron Retention	0,54
TC0100000222.mm.1	-1,26	Imp4	PSR0100001425.mm.1	3,17	0,036871	0,532257	Alternative 5' Donc	0,35
TC1400002648.mm.1	1,18	Pou4f1; RP24-312G4.2	PSR1400019812.mm.1	3,17	0,025705	0,498173	Alternative 3' Acce	0,19
TC1400002648.mm.1	1,18	Pou4f1; RP24-312G4.2	JUC1400010907.mm.1	-2,44	0,008352	0,406435		
TC0300002497.mm.1	1,22	Bola1	JUC0300010154.mm.1	3,17	0,031754	0,517489		
TC0300002497.mm.1	1,22	Bola1	PSR0300019506.mm.1	-2,23	0,014452	0,44909	Cassette Exon	0,14
TC0100002193.mm.1	-1,46	Tmem131	JUC0100010027.mm.1	3,17	0,014748	0,450662		
TC0900003338.mm.1	1,43	Dync2h1	JUC0900008095.mm.1	3,17	0,017566	0,464258		
TC0900003338.mm.1	1,43	Dync2h1	JUC0900008161.mm.1	-3,16	0,022917	0,488524		
TC0900003338.mm.1	1,43	Dync2h1	JUC0900008135.mm.1	-3,57	0,000716	0,306015		
TC0900003338.mm.1	1,43	Dync2h1	JUC0900008134.mm.1	-3,67	0,041435	0,544646		
TC0900000544.mm.1	1,25	Bace1	JUC0900001974.mm.1	3,17	0,036185	0,529982		
TC0700002888.mm.1	-1,07	Gm6124	JUC0700013773.mm.1	3,17	0,046743	0,556856		
TC1200001109.mm.1	1,99	Eml1	JUC1200004404.mm.1	3,16	0,043956	0,550745		
TC1200001109.mm.1	1,99	Eml1	PSR1200007876.mm.1	-2,32	0,011954	0,431862	Alternative 3' Acce	0,13
TC1200001109.mm.1	1,99	Eml1	PSR1200007897.mm.1	-2,43	0,037754	0,534682	Alternative 5' Donc	0,24

TC1200001109.mm.1	1,99	Eml1	PSR1200007868.mm.1	-2,67	0,005689	0,382387	Alternative 3' Acce	0,12
TC1200001109.mm.1	1,99	Eml1	PSR1200007874.mm.1	-2,8	0,01329	0,441719	Alternative 3' Acce	0,17
TC1200001109.mm.1	1,99	Eml1	JUC1200004425.mm.1	-3,19	0,01328	0,441719		
TC1200001109.mm.1	1,99	Eml1	JUC1200004406.mm.1	-4,41	0,018408	0,468484		
TC0500000652.mm.1	1,08	C330024D21Rik	PSR0500005785.mm.1	3,16	0,041283	0,544166	Mutually Exclusive	0,21
TC0500000652.mm.1	1,08	C330024D21Rik	PSR0500005784.mm.1	-2,15	0,012574	0,435847	Mutually Exclusive	0,21
TC0700002502.mm.1	-2,9	Cblc	JUC0700011812.mm.1	3,16	0,037372	0,533674		
TC0700002502.mm.1	-2,9	Cblc	PSR0700022126.mm.1	2,48	0,007904	0,403819	Cassette Exon	0,14
TC0700002502.mm.1	-2,9	Cblc	PSR0700022148.mm.1	2,48	0,049892	0,563773	Cassette Exon	0,14
TC1400001475.mm.1	-1,18		JUC1400005974.mm.1	3,16	0,03085	0,514341		
TC1700001940.mm.1	-1,12	Msh5	JUC1700009837.mm.1	3,16	0,003725	0,357586		
TC0100000678.mm.1	1,96	Sgpp2	JUC0100003351.mm.1	3,16	0,037514	0,533968		
TC0200001985.mm.1	1,15	Ptpra	JUC0200007924.mm.1	3,16	0,014463	0,44909		
TC0600001481.mm.1	1,32	Parp11	JUC0600006449.mm.1	3,16	0,028944	0,50876		
TC1100000316.mm.1	-1,06	Asb3	JUC1100001442.mm.1	3,16	0,034751	0,526574		
TC0Y00000322.mm.1	-1,22	Gm20897	JUC0Y00001510.mm.1	3,16	0,016586	0,459685		
TC0Y00000151.mm.1	-1,36	Gm21258; LOC101056210	JUC0Y00000455.mm.1	3,16	0,018737	0,470733		
TC0Y00000176.mm.1	-1,36	Gm20736; LOC101056210	JUC0Y00000661.mm.1	3,16	0,018737	0,470733		
TC0900002636.mm.1	-1,04	BC065403; RP24-282D16.3	PSR0900021890.mm.1	3,15	0,011719	0,429969	Alternative 3' Acce	0,27
TC0500003730.mm.1	-2,04	Gbp9	PSR0500025580.mm.1	3,15	0,009339	0,415075	Cassette Exon	0,25
TC0700003129.mm.1	3,4		JUC0700015075.mm.1	3,15	0,010972	0,42518		
TC0700003129.mm.1	3,4		PSR0700028296.mm.1	-3,1	0,003863	0,358861	Cassette Exon	0,16
TC0200004528.mm.1	-1	Cep152	JUC0200019938.mm.1	3,15	0,027126	0,503022		
TC0200004528.mm.1	-1	Cep152	JUC0200019935.mm.1	-2,51	0,027891	0,505612		
TC0800003067.mm.1	-1,38		JUC0800012948.mm.1	3,15	0,044398	0,551445		
TC0400000190.mm.1	-1,02	Fbxl4	JUC0400000620.mm.1	3,15	0,012103	0,43255		
TC0300000609.mm.1	3,75	Fstl5	PSR0300004024.mm.1	3,14	0,003665	0,357266	Cassette Exon	0,16
TC0300000609.mm.1	3,75	Fstl5	JUC0300002101.mm.1	2,86	0,01438	0,448389		
TC0300000609.mm.1	3,75	Fstl5	JUC0300002098.mm.1	2,78	0,018602	0,469657		
TC0300000609.mm.1	3,75	Fstl5	JUC0300002111.mm.1	2,77	0,014781	0,45081		
TC0300000609.mm.1	3,75	Fstl5	PSR0300004020.mm.1	-2,23	0,004542	0,368648	Cassette Exon	0,18
TC0300000609.mm.1	3,75	Fstl5	PSR0300004027.mm.1	-2,34	0,000606	0,304044	Cassette Exon	0,12
TC0300000609.mm.1	3,75	Fstl5	PSR0300004035.mm.1	-2,54	0,024151	0,493245		
TC0300000609.mm.1	3,75	Fstl5	PSR0300004019.mm.1	-2,93	0,010216	0,419955	Cassette Exon	0,18
TC0300000609.mm.1	3,75	Fstl5	PSR0300004017.mm.1	-3,54	0,006194	0,387873	Cassette Exon	0,18
TC0300000609.mm.1	3,75	Fstl5	PSR0300004030.mm.1	-4,02	0,000674	0,304338	Alternative 3' Acce	0,45
TC0300000609.mm.1	3,75	Fstl5	JUC0300002114.mm.1	-4,04	0,031129	0,51548		
TC0300000609.mm.1	3,75	Fstl5	PSR0300004032.mm.1	-4,07	0,004909	0,372662	Alternative 5' Donc	0,47
TC0300000609.mm.1	3,75	Fstl5	JUC0300002113.mm.1	-4,11	0,025882	0,498873		
TC0300000609.mm.1	3,75	Fstl5	PSR0300004029.mm.1	-5,05	0,003738	0,357586	Cassette Exon	0,29
TC0300000609.mm.1	3,75	Fstl5	PSR0300004021.mm.1	-8,75	0,000172	0,27703	Cassette Exon	0,44
TC1100001425.mm.1	1,77	Spag9	JUC1100006965.mm.1	3,14	0,036088	0,529716		
TC1100001425.mm.1	1,77	Spag9	PSR1100013262.mm.1	-2,08	0,020489	0,478019	Intron Retention	0,27
TC1100001425.mm.1	1,77	Spag9	JUC1100006932.mm.1	-2,08	0,044107	0,550916		
TC1100001425.mm.1	1,77	Spag9	JUC1100006933.mm.1	-2,14	0,017834	0,466084		
TC1100001425.mm.1	1,77	Spag9	PSR1100013278.mm.1	-2,46	0,036021	0,529546	Alternative 3' Acce	0,23
TC1100001425.mm.1	1,77	Spag9	PSR1100013290.mm.1	-3,05	0,007377	0,39943	Alternative 5' Donc	0,34
TC0500003483.mm.1	3	Prkar1b; Gm15672	JUC0500017309.mm.1	3,14	0,019958	0,476104		

TC0500003483.mm.1	3	Prkar1b; Gm15672	JUC0500017312.mm.1	2,23	0,00607	0,386691		
TC0500003483.mm.1	3	Prkar1b; Gm15672	JUC0500017308.mm.1	2,18	0,001102	0,317328		
TC0500003483.mm.1	3	Prkar1b; Gm15672	PSR0500031884.mm.1	-2,4	0,01512	0,452652		
TC0500003483.mm.1	3	Prkar1b; Gm15672	PSR0500031875.mm.1	-2,5	0,006096	0,386954	Alternative 3' Acce	0,24
TC0500003483.mm.1	3	Prkar1b; Gm15672	PSR0500031894.mm.1	-2,5	0,028743	0,508141	Cassette Exon	0,12
TC0500003483.mm.1	3	Prkar1b; Gm15672	PSR0500031889.mm.1	-2,54	0,011513	0,42831	Cassette Exon	0,07
TC0500003483.mm.1	3	Prkar1b; Gm15672	PSR0500031878.mm.1	-3,05	0,007818	0,403089	Cassette Exon	0,29
TC0500003483.mm.1	3	Prkar1b; Gm15672	PSR0500031881.mm.1	-3,22	0,017837	0,466096	Cassette Exon	0,11
TC0500003483.mm.1	3	Prkar1b; Gm15672	JUC0500017319.mm.1	-3,32	0,02988	0,51129		
TC0500003483.mm.1	3	Prkar1b; Gm15672	PSR0500031893.mm.1	-3,6	0,008917	0,410822	Cassette Exon	0,3
TC0500003483.mm.1	3	Prkar1b; Gm15672	PSR0500031862.mm.1	-3,69	0,016765	0,460504	Cassette Exon	0,33
TC0500003483.mm.1	3	Prkar1b; Gm15672	JUC0500017324.mm.1	-4,11	0,008046	0,40434		
TC0500003483.mm.1	3	Prkar1b; Gm15672	PSR0500031895.mm.1	-4,13	0,038206	0,535805	Cassette Exon	0,32
TC0500003483.mm.1	3	Prkar1b; Gm15672	JUC0500017317.mm.1	-4,15	0,012187	0,433072		
TC0500003483.mm.1	3	Prkar1b; Gm15672	JUC0500017322.mm.1	-4,25	0,009411	0,415853		
TC0500003483.mm.1	3	Prkar1b; Gm15672	JUC0500017327.mm.1	-4,26	0,016482	0,459355		
TC0500003483.mm.1	3	Prkar1b; Gm15672	PSR0500031890.mm.1	-4,28	0,015761	0,455437	Cassette Exon	0,21
TC0500003483.mm.1	3	Prkar1b; Gm15672	PSR0500031877.mm.1	-4,33	0,018401	0,468484	Cassette Exon	0,29
TC0500003483.mm.1	3	Prkar1b; Gm15672	PSR0500031892.mm.1	-4,34	0,008859	0,410501	Cassette Exon	0,21
TC0500003483.mm.1	3	Prkar1b; Gm15672	PSR0500031891.mm.1	-4,63	0,008622	0,408442	Cassette Exon	0,21
TC0500003483.mm.1	3	Prkar1b; Gm15672	JUC0500017323.mm.1	-4,85	0,001358	0,325659		
TC0500003483.mm.1	3	Prkar1b; Gm15672	PSR0500031887.mm.1	-5,23	0,013648	0,444323	Cassette Exon	0,1
TC0400002093.mm.1	1,22	Aurkaip1	PSR0400017465.mm.1	3,14	0,000236	0,28803	Intron Retention	0,29
TC0700002037.mm.1	1,73	B4galnt4	PSR0700017931.mm.1	3,14	0,022043	0,48529	Cassette Exon	0,25
TC0700002037.mm.1	1,73	B4galnt4	JUC0700009509.mm.1	-2,31	0,000069	0,250992		
TC1400001752.mm.1	1,37	Nrg3	PSR1400013641.mm.1	3,14	0,000661	0,304044	Cassette Exon	0,22
TC1400001752.mm.1	1,37	Nrg3	JUC1400007457.mm.1	-2,37	0,003118	0,353892		
TC1400001752.mm.1	1,37	Nrg3	PSR1400013636.mm.1	-2,7	0,049559	0,563325	Cassette Exon	0,16
TC1400001752.mm.1	1,37	Nrg3	JUC1400007463.mm.1	-2,99	0,006675	0,393187		
TC1000003006.mm.1	-1,09		JUC1000012307.mm.1	3,14	0,025195	0,49668		
TC1000003006.mm.1	-1,09		PSR1000022458.mm.1	2,28	0,021924	0,484893	Cassette Exon	0,14
TC1900000689.mm.1	-1,4	Nfkb2	PSR1900006145.mm.1	3,14	0,048718	0,561355	Alternative 3' Acce	0,02
TC1400002336.mm.1	1,01	Slc39a14	JUC1400010013.mm.1	3,14	0,004672	0,370181		
TC1400002336.mm.1	1,01	Slc39a14	JUC1400010018.mm.1	3,14	0,004672	0,370181		
TC1400002336.mm.1	1,01	Slc39a14	PSR1400018216.mm.1	2,2	0,005271	0,378059		
TC1100003920.mm.1	-1,22	Cd79b	JUC1100019186.mm.1	3,14	0,017825	0,465913		
TC1100003920.mm.1	-1,22	Cd79b	JUC1100019187.mm.1	-3,13	0,019441	0,474109		
TC0600000533.mm.1	-1,13		JUC0600001976.mm.1	3,14	0,016293	0,458465		
TC1000000626.mm.1	1,1	Jmjd1c	JUC1000002347.mm.1	3,14	0,024503	0,493992		
TC1000000626.mm.1	1,1	Jmjd1c	JUC1000002359.mm.1	-2,12	0,009386	0,415457		
TC0100002143.mm.1	2,24	Bend6	JUC0100009763.mm.1	3,13	0,005418	0,379167		
TC0100002143.mm.1	2,24	Bend6	PSR0100017227.mm.1	2,39	0,008524	0,407684	Cassette Exon	0,29
TC0100002143.mm.1	2,24	Bend6	PSR0100017225.mm.1	2,01	0,006552	0,39213	Cassette Exon	0,2
TC0100002143.mm.1	2,24	Bend6	PSR0100017233.mm.1	-2,7	0,004466	0,367639	Alternative 5' Donc	0,2
TC0100002143.mm.1	2,24	Bend6	PSR0100017229.mm.1	-2,83	0,005254	0,377869	Cassette Exon	0,18
TC0100002143.mm.1	2,24	Bend6	PSR0100017235.mm.1	-3,15	0,003256	0,354243	Cassette Exon	0,24
TC0100002143.mm.1	2,24	Bend6	JUC0100009767.mm.1	-3,21	0,008783	0,409822		
TC0100002143.mm.1	2,24	Bend6	PSR0100017226.mm.1	-3,41	0,022308	0,486127	Alternative 3' Acce	0,45

TC0100002143.mm.1	2,24	Bend6	PSR0100017234.mm.1	-4,4	0,001221	0,322251	Cassette Exon	0,32
TC0400002077.mm.1	2,63	Gnb1	JUC0400009069.mm.1	3,13	0,039127	0,538487		
TC0400002077.mm.1	2,63	Gnb1	PSR0400017238.mm.1	2,74	0,019193	0,472846	Alternative 5' Donc	0,28
TC0400002077.mm.1	2,63	Gnb1	PSR0400017228.mm.1	-2,19	0,013042	0,439418	Cassette Exon	0,08
TC0400002077.mm.1	2,63	Gnb1	PSR0400017214.mm.1	-2,38	0,011733	0,429969	Cassette Exon	0,13
TC0400002077.mm.1	2,63	Gnb1	PSR0400017227.mm.1	-2,41	0,007895	0,403819	Alternative 5' Donc	0,2
TC1200001192.mm.1	1,58	Mirg; Mir134; Mir377; M	PSR1200008345.mm.1	3,13	0,030638	0,513746	Cassette Exon	0,2
TC1200001192.mm.1	1,58	Mirg; Mir134; Mir377; M	PSR1200008399.mm.1	2,06	0,008276	0,405976		
TC1200001192.mm.1	1,58	Mirg; Mir134; Mir377; M	PSR1200008328.mm.1	-2,13	0,000949	0,313363	Cassette Exon	0,13
TC1200001192.mm.1	1,58	Mirg; Mir134; Mir377; M	PSR1200008375.mm.1	-3	0,001821	0,336896	Cassette Exon	0,12
TC0600003064.mm.1	-1,51	Phc1	PSR0600023725.mm.1	3,13	0,017683	0,464956	Cassette Exon	0,18
TC1600001758.mm.1	-1,05	Tbc1d23	JUC1600007446.mm.1	3,13	0,030269	0,512602		
TC1600001758.mm.1	-1,05	Tbc1d23	PSR1600014284.mm.1	2,57	0,03401	0,524445	Alternative 5' Donc	0,14
TC0300000714.mm.1	-1,41	Iqgap3	JUC0300002566.mm.1	3,13	0,027081	0,502997		
TC0300000714.mm.1	-1,41	Iqgap3	JUC0300002567.mm.1	-2,13	0,002315	0,346468		
TC1300000912.mm.1	-1,62	Ell2	JUC1300003130.mm.1	3,13	0,001959	0,340561		
TC1700002656.mm.1	2,12	Lrpprc	JUC1700013001.mm.1	3,13	0,000163	0,272566		
TC1700002656.mm.1	2,12	Lrpprc	JUC1700013009.mm.1	-2,22	0,023861	0,492097		
TC1700002656.mm.1	2,12	Lrpprc	JUC1700013019.mm.1	-3,41	0,003566	0,356223		
TC1700002656.mm.1	2,12	Lrpprc	JUC1700013020.mm.1	-5,54	0,006215	0,387912		
TC0500001737.mm.1	-1,22	Cyth3	JUC0500008677.mm.1	3,13	0,014692	0,450253		
TC0700004301.mm.1	-1,1	Ino80e	JUC0700019020.mm.1	3,13	0,012113	0,432704		
TC0900001650.mm.1	-1,07	Zfp105	JUC0900007782.mm.1	3,13	0,041282	0,544166		
TC1700001160.mm.1	1,03	Birc6	JUC1700006013.mm.1	3,12	0,025058	0,496166		
TC1700001160.mm.1	1,03	Birc6	JUC1700005955.mm.1	2,17	0,039441	0,539677		
TC1700001160.mm.1	1,03	Birc6	PSR1700010930.mm.1	-5,3	0,044245	0,551191	Alternative 5' Donc	0,48
TC0100000115.mm.1	1,4	Tfap2d	JUC0100000450.mm.1	3,12	0,033722	0,523549		
TC0100000115.mm.1	1,4	Tfap2d	PSR0100000834.mm.1	2,02	0,044261	0,551268	Alternative 5' Donc	0,26
TC0100000115.mm.1	1,4	Tfap2d	PSR0100000824.mm.1	-2,88	0,002061	0,341987	Alternative 3' Acce	0,3
TC1100004277.mm.1	-3,18	Ramp2	PSR1100015354.mm.1	3,12	0,012213	0,433321	Alternative 3' Acce	0,27
TC0900003324.mm.1	-1,89	Bcl9l	PSR0900003320.mm.1	3,12	0,039185	0,538692	Alternative 3' Acce	0,21
TC0X00000916.mm.1	-2,21	Msn	PSR0X00005879.mm.1	3,12	0,042395	0,547599	Alternative 5' Donc	0,21
TC0X00000916.mm.1	-2,21	Msn	JUC0X00002899.mm.1	2,2	0,023021	0,489186		
TC0X00000916.mm.1	-2,21	Msn	JUC0X00002888.mm.1	2,02	0,045947	0,555339		
TC0300002334.mm.1	-1,54	Fdps	PSR0300017841.mm.1	3,12	0,028854	0,508485	Cassette Exon	0,2
TC1600001070.mm.1	-1,67	Kcnj15	JUC1600004405.mm.1	3,12	0,00276	0,35022		
TC1600001070.mm.1	-1,67	Kcnj15	PSR1600008453.mm.1	2,08	0,015966	0,456885	Cassette Exon	0,13
TC1200001692.mm.1	1,16	Nova1	JUC1200006479.mm.1	3,12	0,014306	0,448073		
TC1600001277.mm.1	-1,41	2610318N02Rik	JUC1600005396.mm.1	3,12	0,005241	0,377642		
TC0600002567.mm.1	-1,03	Tet3	JUC0600010353.mm.1	3,12	0,037663	0,53431		
TC0600003005.mm.1	-1,12	Cacna1c	JUC0600012083.mm.1	3,12	0,017148	0,46232		
TC1100003095.mm.1	1,28	Pelp1	JUC1100014973.mm.1	3,12	0,019743	0,474932		
TC0X00003348.mm.1	1,05	Tceanc	JUC0X00010540.mm.1	3,12	0,00433	0,36486		
TC0700000881.mm.1	2	Ldha	PSR0700007923.mm.1	3,11	0,045405	0,554085	Alternative 3' Acce	0,34
TC0700000881.mm.1	2	Ldha	PSR0700007921.mm.1	2,31	0,047031	0,557783	Cassette Exon	0,13
TC0700000881.mm.1	2	Ldha	JUC0700003952.mm.1	2,1	0,005825	0,383169		
TC0700000881.mm.1	2	Ldha	PSR0700007912.mm.1	-4,96	0,025727	0,498173	Alternative 3' Acce	0,4
TC0300001094.mm.1	-1,32	Fam212b	PSR0300008841.mm.1	3,11	0,014109	0,447137	Alternative 5' Donc	0,24

TC0900001652.mm.1	-1,55	Kif15	PSR0900013859.mm.1	3,11	0,025898	0,498978	Cassette Exon	0,23
TC0900001652.mm.1	-1,55	Kif15	PSR0900013853.mm.1	2,87	0,014304	0,448073		
TC0900001652.mm.1	-1,55	Kif15	PSR0900013886.mm.1	2,29	0,021718	0,484104	Cassette Exon	0,1
TC0900001652.mm.1	-1,55	Kif15	JUC0900007820.mm.1	-12,74	0,000186	0,284485		
TC0100000817.mm.1	-1,16	Neu2	PSR0100006929.mm.1	3,11	0,002715	0,349612	Cassette Exon	0,2
TC1100002066.mm.1	-1,51	Tbcd	JUC1100010420.mm.1	3,11	0,01516	0,452699		
TC1100002066.mm.1	-1,51	Tbcd	JUC1100010415.mm.1	2,99	0,019809	0,475245		
TC1100002066.mm.1	-1,51	Tbcd	JUC1100010407.mm.1	2,27	0,015229	0,452839		
TC1100002066.mm.1	-1,51	Tbcd	PSR1100019968.mm.1	2,06	0,037595	0,534053	Cassette Exon	0,12
TC1100002066.mm.1	-1,51	Tbcd	PSR1100019964.mm.1	2,05	0,027564	0,50444	Cassette Exon	0,15
TC1100002066.mm.1	-1,51	Tbcd	PSR1100019966.mm.1	2,05	0,034421	0,525828	Cassette Exon	0,13
TC1100002066.mm.1	-1,51	Tbcd	PSR1100019970.mm.1	2,03	0,009987	0,418456	Cassette Exon	0,12
TC1000000717.mm.1	-2,52	Ggt1	JUC1000002806.mm.1	3,11	0,02778	0,505265		
TC1000000717.mm.1	-2,52	Ggt1	PSR1000005096.mm.1	2,68	0,018156	0,467427	Cassette Exon	0,12
TC1000000717.mm.1	-2,52	Ggt1	JUC1000002797.mm.1	2,55	0,027894	0,505612		
TC1000000717.mm.1	-2,52	Ggt1	JUC1000002784.mm.1	2,13	0,017631	0,464508		
TC1500002284.mm.1	1,09	Spryd3	JUC1500010771.mm.1	3,11	0,046717	0,556856		
TC1500002284.mm.1	1,09	Spryd3	JUC1500010775.mm.1	2,04	0,005372	0,378731		
TC0700000723.mm.1	-1,39	Gm4884; Gm2128	JUC0700002940.mm.1	3,11	0,017467	0,463886		
TC0500003047.mm.1	1,33	Sart3	JUC0500014800.mm.1	3,11	0,023942	0,49258		
TC0900002997.mm.1	-1,25	Col6a6	JUC0900013871.mm.1	3,11	0,020272	0,477245		
TC1000000286.mm.1	-2,67	Trdn	JUC1000001052.mm.1	3,11	0,037445	0,533849		
TC1000000286.mm.1	-2,67	Trdn	JUC1000001021.mm.1	-7,23	0,001905	0,338712		
TC0400001663.mm.1	-1,31	Myom3	JUC0400006864.mm.1	3,11	0,003025	0,353502		
TC0400001663.mm.1	-1,31	Myom3	JUC0400006871.mm.1	2,19	0,00508	0,375342		
TC0400001663.mm.1	-1,31	Myom3	JUC0400006888.mm.1	-2,32	0,001028	0,316361		
TC0900001940.mm.1	2,6	Glb1l3	JUC0900009212.mm.1	3,1	0,004727	0,370888		
TC0900001940.mm.1	2,6	Glb1l3	JUC0900009222.mm.1	2,7	0,005308	0,378059		
TC0900001940.mm.1	2,6	Glb1l3	PSR0900016274.mm.1	-2,15	0,032598	0,520272	Alternative 3' Acce	0,15
TC0900001940.mm.1	2,6	Glb1l3	PSR0900016295.mm.1	-2,3	0,024533	0,493992	Cassette Exon	0,26
TC0900001940.mm.1	2,6	Glb1l3	JUC0900009226.mm.1	-2,87	0,002464	0,349135		
TC0900001940.mm.1	2,6	Glb1l3	JUC0900009216.mm.1	-3,01	0,01573	0,455416		
TC0900001940.mm.1	2,6	Glb1l3	JUC0900009230.mm.1	-4,23	0,018083	0,46716		
TC0900001940.mm.1	2,6	Glb1l3	PSR0900016294.mm.1	-4,46	0,011364	0,427452	Cassette Exon	0,41
TC0800000291.mm.1	1,42	Ank1	JUC0800001176.mm.1	3,1	0,003903	0,35953		
TC0800000291.mm.1	1,42	Ank1	PSR0800002195.mm.1	2,93	0,001361	0,32572	Cassette Exon	0,19
TC0800000291.mm.1	1,42	Ank1	JUC0800001179.mm.1	2,72	0,004404	0,366624		
TC0800000291.mm.1	1,42	Ank1	JUC0800001177.mm.1	2,68	0,028262	0,506565		
TC0800000291.mm.1	1,42	Ank1	PSR0800002185.mm.1	2,66	0,000851	0,311909	Cassette Exon	0,31
TC0800000291.mm.1	1,42	Ank1	PSR0800002225.mm.1	2,61	0,0114	0,427549	Cassette Exon	0,05
TC0800000291.mm.1	1,42	Ank1	PSR0800002218.mm.1	2,44	0,003828	0,358463	Cassette Exon	0,12
TC0800000291.mm.1	1,42	Ank1	JUC0800001175.mm.1	2,32	0,006835	0,393893		
TC0800000291.mm.1	1,42	Ank1	PSR0800002187.mm.1	2,07	0,004072	0,361956	Cassette Exon	0,17
TC0800000291.mm.1	1,42	Ank1	PSR0800002252.mm.1	-2,01	0,048037	0,559636	Alternative 5' Donc	0,17
TC0800000291.mm.1	1,42	Ank1	JUC0800001208.mm.1	-2,03	0,032451	0,519588		
TC0800000291.mm.1	1,42	Ank1	PSR0800002240.mm.1	-2,05	0,031316	0,516204	Cassette Exon	0,13
TC0800000291.mm.1	1,42	Ank1	PSR0800002210.mm.1	-2,11	0,01851	0,469047	Cassette Exon	0,06
TC0800000291.mm.1	1,42	Ank1	PSR0800002236.mm.1	-2,31	0,012269	0,434074	Cassette Exon	0,08

TC0800000291.mm.1	1,42	Ank1	PSR0800002239.mm.1	-2,33	0,028172	0,506368	Cassette Exon	0,31
TC0800000291.mm.1	1,42	Ank1	PSR0800002249.mm.1	-2,33	0,022385	0,486477	Alternative 5' Dono	0,2
TC0800000291.mm.1	1,42	Ank1	JUC0800001219.mm.1	-2,65	0,049939	0,563958		
TC0800000291.mm.1	1,42	Ank1	PSR0800002246.mm.1	-2,86	0,000156	0,272178	Alternative 5' Dono	0,26
TC0800000291.mm.1	1,42	Ank1	JUC0800001216.mm.1	-2,91	0,004464	0,367578		
TC0800000291.mm.1	1,42	Ank1	JUC0800001214.mm.1	-3,2	0,007509	0,401082		
TC0800000291.mm.1	1,42	Ank1	PSR0800002247.mm.1	-3,34	0,028309	0,506741	Alternative 5' Dono	0,26
TC0600001088.mm.1	-1,67	Lsm3	PSR0600008525.mm.1	3,1	0,034446	0,525886	Alternative 5' Dono	0,27
TC1300000284.mm.1	1,36	Gpld1	JUC1300000875.mm.1	3,1	0,001055	0,316361		
TC1300000284.mm.1	1,36	Gpld1	JUC1300000873.mm.1	-2,28	0,016283	0,458425		
TC1300000284.mm.1	1,36	Gpld1	PSR1300001764.mm.1	-2,3	0,025313	0,497278	Alternative 5' Dono	0,19
TC1300000284.mm.1	1,36	Gpld1	PSR1300001742.mm.1	-2,61	0,003351	0,354243	Alternative 3' Acce	0,26
TC0200004645.mm.1	1,46	Gfra4; Mir6973b; mmu-mi	PSR0200039831.mm.1	3,1	0,012267	0,43405	Alternative 3' Acce	0,19
TC1200002442.mm.1	-1,13		JUC1200009335.mm.1	3,1	0,001624	0,332831		
TC1300000464.mm.1	-1,42	Sycp2l	JUC1300001441.mm.1	3,1	0,034604	0,52602		
TC0800002941.mm.1	1,05	Glg1	JUC0800012392.mm.1	3,1	0,013872	0,445672		
TC0800002941.mm.1	1,05	Glg1	JUC0800012370.mm.1	-2,25	0,031469	0,516922		
TC0500001514.mm.1	-1,3	Sumf2	JUC0500007511.mm.1	3,1	0,03075	0,514077		
TC0500001514.mm.1	-1,3	Sumf2	JUC0500007516.mm.1	2,31	0,039093	0,538395		
TC1100000856.mm.1	-2,27	Myh8	JUC1100003697.mm.1	3,1	0,001929	0,340171		
TC0400001712.mm.1	-1,01	Rap1gap	JUC0400007199.mm.1	3,1	0,000844	0,311909		
TC0400001712.mm.1	-1,01	Rap1gap	JUC0400007200.mm.1	2,61	0,026244	0,500211		
TC0400001712.mm.1	-1,01	Rap1gap	JUC0400007214.mm.1	-2,58	0,008236	0,405976		
TC0400003293.mm.1	-1,32	Skint6	PSR0400026224.mm.1	3,09	0,007707	0,402552	Cassette Exon	0,25
TC0400003293.mm.1	-1,32	Skint6	JUC0400013640.mm.1	2,63	0,032277	0,519116		
TC0400003293.mm.1	-1,32	Skint6	PSR0400026232.mm.1	-2,09	0,001757	0,335996	Cassette Exon	0,05
TC0400003293.mm.1	-1,32	Skint6	JUC0400013631.mm.1	-2,17	0,001949	0,34055		
TC0200001664.mm.1	1,72	Katnbl1; Slc12a6; Gm2198	JUC0200006149.mm.1	3,09	0,013491	0,443193		
TC0200001664.mm.1	1,72	Katnbl1; Slc12a6; Gm2198	JUC0200006173.mm.1	-2,09	0,023451	0,490586		
TC0200001664.mm.1	1,72	Katnbl1; Slc12a6; Gm2198	PSR0200012405.mm.1	-2,1	0,006373	0,389488	Cassette Exon	0,13
TC0200001664.mm.1	1,72	Katnbl1; Slc12a6; Gm2198	PSR0200012390.mm.1	-2,46	0,024063	0,493026	Cassette Exon	0,16
TC0200001664.mm.1	1,72	Katnbl1; Slc12a6; Gm2198	PSR0200012409.mm.1	-2,54	0,013178	0,44063	Cassette Exon	0,14
TC0200001664.mm.1	1,72	Katnbl1; Slc12a6; Gm2198	JUC0200006174.mm.1	-3,13	0,002741	0,349612		
TC0200001664.mm.1	1,72	Katnbl1; Slc12a6; Gm2198	JUC0200006171.mm.1	-3,68	0,001664	0,335182		
TC1400002274.mm.1	-1,07	1700001G11Rik	JUC1400009626.mm.1	3,09	0,000639	0,304044		
TC1400002274.mm.1	-1,07	1700001G11Rik	PSR1400017573.mm.1	2,02	0,003959	0,359922	Cassette Exon	0,15
TC0300002778.mm.1	1,38	Amy2b	JUC0300011369.mm.1	3,09	0,015627	0,45509		
TC0300002778.mm.1	1,38	Amy2b	PSR0300021827.mm.1	2,25	0,02531	0,49725	Cassette Exon	0,1
TC0300002778.mm.1	1,38	Amy2b	JUC0300011368.mm.1	2,19	0,02334	0,490131		
TC0100003536.mm.1	1,31	Ppox	JUC0100016496.mm.1	3,09	0,04574	0,554855		
TC0700003793.mm.1	-1,09	Dgat2	JUC0700016905.mm.1	3,09	0,018546	0,469354		
TC0700002270.mm.1	-1,31	Gm18191	JUC0700010905.mm.1	3,09	0,000466	0,298999		
TC1100001020.mm.1	-1,39	Trpv3	JUC1100004892.mm.1	3,09	0,038993	0,537976		
TC1000002067.mm.1	1,05	Nr2e1	JUC1000008032.mm.1	3,09	0,0405	0,542217		
TC0900002570.mm.1	-1,65	4930502A04Rik	JUC0900012076.mm.1	3,09	0,008575	0,40821		
TC0700003823.mm.1	4,14	Plekhb1	JUC0700017035.mm.1	3,08	0,008882	0,410619		
TC0700003823.mm.1	4,14	Plekhb1	PSR0700032203.mm.1	2,73	0,000759	0,308387	Cassette Exon	0,29
TC0700003823.mm.1	4,14	Plekhb1	PSR0700032212.mm.1	-2,19	0,018917	0,471305		

TC0700003823.mm.1	4,14	Plekhb1	JUC0700017038.mm.1	-2,93	0,008841	0,410293		
TC0700003823.mm.1	4,14	Plekhb1	PSR0700032216.mm.1	-3,5	0,04574	0,554855	Alternative 5' Donc	0,28
TC0700003823.mm.1	4,14	Plekhb1	PSR0700032220.mm.1	-3,59	0,014764	0,450801	Alternative 5' Donc	0,41
TC1000001260.mm.1	4,49	Lin7a	PSR1000009502.mm.1	3,08	0,011399	0,427549	Alternative 3' Acce	0,28
TC1000001260.mm.1	4,49	Lin7a	JUC1000005124.mm.1	2,77	0,002878	0,352207		
TC1000001260.mm.1	4,49	Lin7a	PSR1000009497.mm.1	2,06	0,002466	0,349135	Cassette Exon	0,3
TC1000001260.mm.1	4,49	Lin7a	PSR1000009499.mm.1	-2,67	0,037141	0,533034	Cassette Exon	0,35
TC1000001260.mm.1	4,49	Lin7a	JUC1000005128.mm.1	-3,02	0,025686	0,498173		
TC1000001260.mm.1	4,49	Lin7a	PSR1000009496.mm.1	-3,33	0,004095	0,36228	Cassette Exon	0,2
TC1000001260.mm.1	4,49	Lin7a	JUC1000005130.mm.1	-3,82	0,025364	0,497403		
TC1000001260.mm.1	4,49	Lin7a	PSR1000009495.mm.1	-6,08	0,001807	0,336311	Cassette Exon	0,29
TC0700004358.mm.1	-1,95	Cox6a2	PSR0700036594.mm.1	3,08	0,005274	0,378059	Alternative 3' Acce	0,33
TC1500000591.mm.1	1,01	Mroh1; Mir6954; mmu-mi	JUC1500002446.mm.1	3,08	0,031225	0,515879		
TC1500000591.mm.1	1,01	Mroh1; Mir6954; mmu-mi	JUC1500002427.mm.1	-2,02	0,048817	0,561556		
TC1500000591.mm.1	1,01	Mroh1; Mir6954; mmu-mi	PSR1500004233.mm.1	-2,79	0,002049	0,341391	Alternative 5' Donc	0,29
TC0200004948.mm.1	2,55	Gm14216	PSR0200041964.mm.1	3,08	0,014163	0,447466	Cassette Exon	0,19
TC0200004948.mm.1	2,55	Gm14216	JUC0200021660.mm.1	-4,5	0,047349	0,558291		
TC0900001892.mm.1	2,46	Elavl3	PSR0900015972.mm.1	3,08	0,005726	0,382767	Cassette Exon	0,05
TC0900001892.mm.1	2,46	Elavl3	PSR0900015971.mm.1	-2,45	0,026117	0,499897	Alternative 3' Acce	0,14
TC0900001892.mm.1	2,46	Elavl3	PSR0900015978.mm.1	-2,64	0,008001	0,403872	Cassette Exon	0,14
TC0900001892.mm.1	2,46	Elavl3	JUC0900009031.mm.1	-3,11	0,008586	0,40821		
TC0900001892.mm.1	2,46	Elavl3	JUC0900009037.mm.1	-4,24	0,037294	0,533573		
TC0900001892.mm.1	2,46	Elavl3	JUC0900009038.mm.1	-4,27	0,035266	0,52837		
TC1400001441.mm.1	-1,26	4930452B06Rik	JUC1400005841.mm.1	3,08	0,017639	0,464582		
TC1700002240.mm.1	-1,1	Daam2	JUC1700011257.mm.1	3,08	0,023601	0,490995		
TC1700002240.mm.1	-1,1	Daam2	JUC1700011258.mm.1	-2,38	0,013629	0,444136		
TC0100002285.mm.1	-1,02	Slc40a1	JUC0100010450.mm.1	3,08	0,025761	0,498442		
TC0100003778.mm.1	-1,12	Kcnk2	JUC0100017525.mm.1	3,08	0,001893	0,338712		
TC0100003778.mm.1	-1,12	Kcnk2	JUC0100017529.mm.1	2,21	0,020211	0,477145		
TC0500000286.mm.1	-1,32	Hadhb	JUC0500001236.mm.1	3,08	0,001771	0,336311		
TC0500001327.mm.1	1,03	Tbx3	JUC0500006318.mm.1	3,08	0,033301	0,522389		
TC0500001327.mm.1	1,03	Tbx3	JUC0500006315.mm.1	2,09	0,012355	0,434613		
TC1000002944.mm.1	1,38	Nup107	JUC1000012246.mm.1	3,08	0,00792	0,403819		
TC0400000706.mm.1	-1,15	Gm11210	JUC0400002529.mm.1	3,08	0,042809	0,548097		
TC0200002543.mm.1	6,6	Slc12a5; Gm11459	JUC0200010605.mm.1	3,07	0,00002	0,211693		
TC0200002543.mm.1	6,6	Slc12a5; Gm11459	PSR0200020865.mm.1	2,99	0,032687	0,520439	Cassette Exon	0,08
TC0200002543.mm.1	6,6	Slc12a5; Gm11459	JUC0200010597.mm.1	2,89	0,005454	0,379939		
TC0200002543.mm.1	6,6	Slc12a5; Gm11459	JUC0200010604.mm.1	2,43	0,004206	0,363236		
TC0200002543.mm.1	6,6	Slc12a5; Gm11459	PSR0200020877.mm.1	2,41	0,038494	0,536557	Cassette Exon	0,01
TC0200002543.mm.1	6,6	Slc12a5; Gm11459	PSR0200020866.mm.1	2,39	0,016728	0,460261		
TC0200002543.mm.1	6,6	Slc12a5; Gm11459	JUC0200010595.mm.1	2,29	0,012704	0,436428		
TC0200002543.mm.1	6,6	Slc12a5; Gm11459	PSR0200020871.mm.1	2,12	0,010872	0,424174	Cassette Exon	0,17
TC0200002543.mm.1	6,6	Slc12a5; Gm11459	PSR0200020861.mm.1	-2,05	0,000153	0,272178	Cassette Exon	0,37
TC0200002543.mm.1	6,6	Slc12a5; Gm11459	PSR0200020862.mm.1	-2,33	0,000959	0,313363	Cassette Exon	0,33
TC0200002543.mm.1	6,6	Slc12a5; Gm11459	PSR0200020884.mm.1	-3	0,006028	0,386586	Cassette Exon	0,46
TC0200002543.mm.1	6,6	Slc12a5; Gm11459	PSR0200020860.mm.1	-3,04	0,00448	0,367858	Cassette Exon	0,32
TC0200002543.mm.1	6,6	Slc12a5; Gm11459	JUC0200010589.mm.1	-3,58	0,005705	0,382524		
TC0200002543.mm.1	6,6	Slc12a5; Gm11459	PSR0200020885.mm.1	-3,61	0,000005	0,179072	Cassette Exon	0,37

TC0200002543.mm.1	6,6	Slc12a5; Gm11459	PSR0200020869.mm.1	-4,14	0,003106	0,353892	Alternative 3' Acce	0,39
TC0200002543.mm.1	6,6	Slc12a5; Gm11459	JUC0200010607.mm.1	-4,37	0,027843	0,505564		
TC0200002543.mm.1	6,6	Slc12a5; Gm11459	PSR0200020855.mm.1	-4,54	0,010372	0,421273	Cassette Exon	0,55
TC0200002543.mm.1	6,6	Slc12a5; Gm11459	JUC0200010587.mm.1	-4,72	0,000348	0,290082		
TC0200002543.mm.1	6,6	Slc12a5; Gm11459	JUC0200010591.mm.1	-4,72	0,000182	0,283791		
TC0200002543.mm.1	6,6	Slc12a5; Gm11459	JUC0200010601.mm.1	-5,13	0,012598	0,435933		
TC0200002543.mm.1	6,6	Slc12a5; Gm11459	JUC0200010608.mm.1	-5,3	0,000996	0,316361		
TC0200002543.mm.1	6,6	Slc12a5; Gm11459	JUC0200010602.mm.1	-7,17	0,000013	0,185427		
TC0200002543.mm.1	6,6	Slc12a5; Gm11459	PSR0200020859.mm.1	-7,68	0,002702	0,349612	Cassette Exon	0,28
TC0200002543.mm.1	6,6	Slc12a5; Gm11459	PSR0200020854.mm.1	-7,9	0,001895	0,338712	Cassette Exon	0,41
TC0200002543.mm.1	6,6	Slc12a5; Gm11459	PSR0200020856.mm.1	-8,73	0,000308	0,288663	Cassette Exon	0,41
TC0200002543.mm.1	6,6	Slc12a5; Gm11459	PSR0200020888.mm.1	-9,36	0,000769	0,308403	Intron Retention	0,35
TC0200002543.mm.1	6,6	Slc12a5; Gm11459	PSR0200020886.mm.1	-10,75	0,000642	0,304044	Alternative 3' Acce	0,31
TC0200002543.mm.1	6,6	Slc12a5; Gm11459	PSR0200020858.mm.1	-12,52	0,0006	0,304044	Cassette Exon	0,28
TC0200002543.mm.1	6,6	Slc12a5; Gm11459	JUC0200010584.mm.1	-15,01	0,000851	0,311909		
TC0200002543.mm.1	6,6	Slc12a5; Gm11459	JUC0200010612.mm.1	-17,28	0,000097	0,261355		
TC0200002543.mm.1	6,6	Slc12a5; Gm11459	JUC0200010583.mm.1	-19,02	0,002079	0,342181		
TC0200002543.mm.1	6,6	Slc12a5; Gm11459	JUC0200010586.mm.1	-20,74	0,002189	0,344463		
TC0600000250.mm.1	3,41	Strip2	JUC0600000902.mm.1	3,07	0,002688	0,349612		
TC0600000250.mm.1	3,41	Strip2	PSR0600001777.mm.1	2,69	0,001338	0,325349	Alternative 3' Acce	0,29
TC0600000250.mm.1	3,41	Strip2	PSR0600001773.mm.1	2,59	0,018614	0,469723	Cassette Exon	0,18
TC0600000250.mm.1	3,41	Strip2	PSR0600001758.mm.1	2,39	0,006023	0,386586	Cassette Exon	0,23
TC0600000250.mm.1	3,41	Strip2	JUC0600000901.mm.1	2,18	0,010732	0,423745		
TC0600000250.mm.1	3,41	Strip2	PSR0600001756.mm.1	2,03	0,004798	0,371408	Cassette Exon	0,14
TC0600000250.mm.1	3,41	Strip2	PSR0600001762.mm.1	-2,15	0,011374	0,427487	Cassette Exon	0,05
TC0600000250.mm.1	3,41	Strip2	JUC0600000912.mm.1	-2,16	0,034468	0,525929		
TC0600000250.mm.1	3,41	Strip2	JUC0600000899.mm.1	-2,78	0,028474	0,507299		
TC0600000250.mm.1	3,41	Strip2	PSR0600001764.mm.1	-2,83	0,00386	0,358861	Intron Retention	0,39
TC0600000250.mm.1	3,41	Strip2	PSR0600001784.mm.1	-3,12	0,002534	0,349501	Alternative 5' Donc	0,34
TC1700001264.mm.1	-1,31	Dync2li1	PSR1700011663.mm.1	3,07	0,048142	0,559956	Cassette Exon	0,18
TC1700001264.mm.1	-1,31	Dync2li1	JUC1700006381.mm.1	3,07	0,007884	0,403723		
TC1700001264.mm.1	-1,31	Dync2li1	JUC1700006377.mm.1	2,98	0,045525	0,554359		
TC0700002145.mm.1	1,69	9430041J12Rik	JUC0700010366.mm.1	3,07	0,018407	0,468484		
TC0700002145.mm.1	1,69	9430041J12Rik	PSR0700019493.mm.1	-2,01	0,041296	0,544228	Alternative 3' Acce	0,14
TC0700002145.mm.1	1,69	9430041J12Rik	PSR0700019508.mm.1	-2,12	0,021228	0,481319	Alternative 5' Donc	0,15
TC0700002145.mm.1	1,69	9430041J12Rik	JUC0700010371.mm.1	-2,53	0,028929	0,50862		
TC0300003222.mm.1	1,72	Ovgp1	JUC0300004616.mm.1	3,07	0,009719	0,417826		
TC0300003222.mm.1	1,72	Ovgp1	JUC0300004618.mm.1	2,52	0,026736	0,502096		
TC0200005092.mm.1	1,06	Hnf4aos; 0610008F07Rik	JUC0200022394.mm.1	3,07	0,01551	0,454456		
TC0100003550.mm.1	-1,67	Slamf7	JUC0100016573.mm.1	3,07	0,014997	0,452052		
TC0100003323.mm.1	1,04	Ralgps2	JUC0100015582.mm.1	3,07	0,041103	0,543933		
TC0600001672.mm.1	-1,36	Slco1b2	JUC0600007001.mm.1	3,07	0,011038	0,425682		
TC0600001672.mm.1	-1,36	Slco1b2	JUC0600006995.mm.1	2,06	0,039458	0,539705		
TC0900002026.mm.1	-1,23	9230110F15Rik; RP23-319	JUC0900009496.mm.1	3,07	0,014861	0,451623		
TC0Y00000149.mm.1	-1,27	Gm20920	JUC0Y00000446.mm.1	3,07	0,013843	0,445333		
TC0Y00000174.mm.1	-1,27	Gm20908	JUC0Y00000652.mm.1	3,07	0,013843	0,445333		
TC0900000232.mm.1	-1,71	Npsr1	PSR0900001777.mm.1	3,06	0,019554	0,474538	Alternative 3' Acce	0,34
TC0700004475.mm.1	-1	Stk32c	JUC0700019780.mm.1	3,06	0,009232	0,414336		

TC0700004475.mm.1	-1	Stk32c	PSR0700037538.mm.1	2,96	0,004814	0,371408	Cassette Exon	0,28
TC1000000261.mm.1	2,08	Soga3	PSR1000001800.mm.1	3,06	0,003295	0,354243	Cassette Exon	0,27
TC1000000261.mm.1	2,08	Soga3	JUC1000000976.mm.1	2,8	0,031163	0,515677		
TC1000000261.mm.1	2,08	Soga3	PSR1000001796.mm.1	-2,66	0,009658	0,417484	Cassette Exon	0,17
TC1000000261.mm.1	2,08	Soga3	PSR1000001797.mm.1	-2,95	0,003997	0,361023	Cassette Exon	0,19
TC1000000261.mm.1	2,08	Soga3	PSR1000001798.mm.1	-2,96	0,006565	0,39213	Cassette Exon	0,19
TC1000000261.mm.1	2,08	Soga3	JUC1000000979.mm.1	-4,3	0,004426	0,366832		
TC1200000823.mm.1	-1,72	Fcf1	JUC1200003354.mm.1	3,06	0,045001	0,552975		
TC1200000823.mm.1	-1,72	Fcf1	PSR1200005937.mm.1	2,39	0,000128	0,270905	Cassette Exon	0,25
TC0200003865.mm.1	-1,86	Calcrl	JUC0200017204.mm.1	3,06	0,027854	0,505597		
TC0200003865.mm.1	-1,86	Calcrl	PSR0200033352.mm.1	2,48	0,029782	0,511132	Alternative 3' Acce	0,23
TC0400001306.mm.1	-1,04	Med8	PSR0400009807.mm.1	3,06	0,014826	0,451164	Alternative 3' Acce	0,17
TC0900002542.mm.1	1,37	Tln2	JUC0900012057.mm.1	3,06	0,010842	0,424161		
TC0900002542.mm.1	1,37	Tln2	JUC0900012001.mm.1	2,89	0,015179	0,452699		
TC0900002542.mm.1	1,37	Tln2	JUC0900012060.mm.1	2,65	0,018533	0,469204		
TC0900002542.mm.1	1,37	Tln2	JUC0900012027.mm.1	2,35	0,016794	0,460623		
TC0900002542.mm.1	1,37	Tln2	PSR0900021433.mm.1	2,33	0,004029	0,361636	Cassette Exon	0,06
TC0900002542.mm.1	1,37	Tln2	JUC0900012030.mm.1	2,25	0,012094	0,43255		
TC0900002542.mm.1	1,37	Tln2	JUC0900012056.mm.1	2,25	0,02382	0,491955		
TC0900002542.mm.1	1,37	Tln2	JUC0900012043.mm.1	2,22	0,01144	0,427685		
TC0900002542.mm.1	1,37	Tln2	JUC0900012050.mm.1	2,13	0,013825	0,445193		
TC0900002542.mm.1	1,37	Tln2	PSR0900021436.mm.1	2,06	0,009591	0,417183	Cassette Exon	0,17
TC0900002542.mm.1	1,37	Tln2	JUC0900012061.mm.1	-2,36	0,008872	0,410549		
TC0900002542.mm.1	1,37	Tln2	JUC0900012016.mm.1	-3,69	0,02906	0,508976		
TC1300002099.mm.1	1,33	Hnrnpk	JUC1300006930.mm.1	3,06	0,016856	0,460797		
TC1300002099.mm.1	1,33	Hnrnpk	PSR1300013378.mm.1	2,18	0,037059	0,532719	Alternative 3' Acce	0,17
TC1900001485.mm.1	-1,09	Sorbs1	JUC1900007153.mm.1	3,06	0,002907	0,352262		
TC1900001485.mm.1	-1,09	Sorbs1	JUC1900007148.mm.1	2,5	0,009109	0,413018		
TC1100002488.mm.1	1,22	Ranbp17	JUC1100012037.mm.1	3,06	0,042795	0,548097		
TC1100000110.mm.1	1,61		PSR1100001141.mm.1	3,05	0,017133	0,46232	Alternative 3' Acce	0,33
TC1100003137.mm.1	1,97	Pitpnm3	PSR1100029057.mm.1	3,05	0,000905	0,313363	Cassette Exon	0,17
TC1100003137.mm.1	1,97	Pitpnm3	JUC1100015209.mm.1	2,76	0,03215	0,518586		
TC1100003137.mm.1	1,97	Pitpnm3	PSR1100029058.mm.1	2,46	0,004364	0,365487	Cassette Exon	0,08
TC1100003137.mm.1	1,97	Pitpnm3	JUC1100015222.mm.1	2,35	0,020029	0,476453		
TC1100003137.mm.1	1,97	Pitpnm3	PSR1100029053.mm.1	2,24	0,002825	0,352207	Cassette Exon	0,13
TC1100003137.mm.1	1,97	Pitpnm3	JUC1100015213.mm.1	2,23	0,002521	0,349501		
TC1100003137.mm.1	1,97	Pitpnm3	PSR1100029062.mm.1	-2,1	0,043467	0,549841	Cassette Exon	0,09
TC1100003137.mm.1	1,97	Pitpnm3	PSR1100029037.mm.1	-2,11	0,011686	0,429961	Alternative 5' Donc	0,18
TC1100003137.mm.1	1,97	Pitpnm3	PSR1100029059.mm.1	-2,36	0,014305	0,448073	Alternative 5' Donc	0,16
TC1100003137.mm.1	1,97	Pitpnm3	JUC1100015221.mm.1	-2,36	0,010761	0,423859		
TC1100003137.mm.1	1,97	Pitpnm3	PSR1100029035.mm.1	-2,39	0,042205	0,546983	Cassette Exon	0,15
TC1100003137.mm.1	1,97	Pitpnm3	PSR1100029039.mm.1	-2,47	0,000862	0,311909	Alternative 3' Acce	0,21
TC1100003137.mm.1	1,97	Pitpnm3	JUC1100015215.mm.1	-2,48	0,030319	0,51274		
TC1300000405.mm.1	-1,21	Cdyl	JUC1300001251.mm.1	3,05	0,006519	0,391382		
TC1300000405.mm.1	-1,21	Cdyl	PSR1300002451.mm.1	2,16	0,01369	0,444581	Alternative 5' Donc	0,16
TC1300000405.mm.1	-1,21	Cdyl	JUC1300001254.mm.1	2,02	0,032626	0,520338		
TC0200000507.mm.1	1,18	Rapgef1	JUC0200001828.mm.1	3,05	0,004621	0,369794		
TC0200000507.mm.1	1,18	Rapgef1	PSR0200003684.mm.1	2,15	0,042006	0,546101	Cassette Exon	0,12

TC0700003854.mm.1	-1,07	Nup98	JUC0700017259.mm.1	3,05	0,00014	0,272178		
TC0700003854.mm.1	-1,07	Nup98	JUC0700017252.mm.1	2,27	0,039626	0,540106		
TC0900002010.mm.1	-1,01	Fam118b	JUC0900009450.mm.1	3,05	0,00558	0,381035		
TC0X00002108.mm.1	1,18	Thoc2	JUC0X00006838.mm.1	3,05	0,000946	0,313363		
TC0400002470.mm.1	-1,25	1110017D15Rik	PSR0400019940.mm.1	3,04	0,020375	0,477572	Alternative 3' Acce	0,33
TC0700001564.mm.1	-1,25	Olfr701	JUC0700006763.mm.1	3,04	0,016978	0,461419		
TC0700001564.mm.1	-1,25	Olfr701	PSR0700013146.mm.1	2,11	0,013462	0,442805	Alternative 3' Acce	0,17
TC0200002826.mm.1	-1,08	Tpd52l2; Gm25184	JUC0200011822.mm.1	3,04	0,02693	0,502599		
TC0200002826.mm.1	-1,08	Tpd52l2; Gm25184	JUC0200011821.mm.1	2,58	0,000286	0,28803		
TC1500000395.mm.1	-1,6	Col14a1	JUC1500001616.mm.1	3,04	0,04843	0,560744		
TC1500000395.mm.1	-1,6	Col14a1	JUC1500001612.mm.1	2,07	0,048014	0,559589		
TC0700000917.mm.1	1,05	Gas2	JUC0700004167.mm.1	3,04	0,028854	0,508485		
TC0700000917.mm.1	1,05	Gas2	JUC0700004175.mm.1	-2,52	0,011901	0,431543		
TC0600000269.mm.1	-1,03	Cpa2	JUC0600000977.mm.1	3,04	0,033795	0,523795		
TC0400000912.mm.1	-1,3	Gm12603	JUC0400003112.mm.1	3,04	0,005138	0,376442		
TC0400003880.mm.1	-1,06	Clcnka	JUC0400016715.mm.1	3,04	0,011147	0,426226		
TC0100003104.mm.1	1,69	Adora1	PSR0100025012.mm.1	3,03	0,043143	0,548664	Alternative 5' Donc	0,32
TC0100003104.mm.1	1,69	Adora1	PSR0100025015.mm.1	-2,39	0,03428	0,525322	Alternative 5' Donc	0,16
TC0700000486.mm.1	-1,12	9530053A07Rik	PSR0700003937.mm.1	3,03	0,018339	0,468225	Alternative 5' Donc	0,32
TC0900001424.mm.1	2,62	Celsr3; Gm23156	JUC0900006376.mm.1	3,03	0,012513	0,435429		
TC0900001424.mm.1	2,62	Celsr3; Gm23156	PSR0900011568.mm.1	2,99	0,020118	0,47686	Mutually Exclusive	0,19
TC0900001424.mm.1	2,62	Celsr3; Gm23156	JUC0900006386.mm.1	2,77	0,031188	0,515739		
TC0900001424.mm.1	2,62	Celsr3; Gm23156	PSR0900011579.mm.1	2,63	0,001592	0,331726	Cassette Exon	0,15
TC0900001424.mm.1	2,62	Celsr3; Gm23156	PSR0900011567.mm.1	2,26	0,002779	0,351017	Cassette Exon	0,05
TC0900001424.mm.1	2,62	Celsr3; Gm23156	PSR0900011543.mm.1	2,18	0,032218	0,518916	Cassette Exon	0,12
TC0900001424.mm.1	2,62	Celsr3; Gm23156	PSR0900011573.mm.1	2,11	0,015174	0,452699	Mutually Exclusive	0,11
TC0900001424.mm.1	2,62	Celsr3; Gm23156	PSR0900011570.mm.1	-2,16	0,001939	0,340349	Mutually Exclusive	0,19
TC0900001424.mm.1	2,62	Celsr3; Gm23156	JUC0900006387.mm.1	-2,19	0,009587	0,417183		
TC0900001424.mm.1	2,62	Celsr3; Gm23156	JUC0900006394.mm.1	-2,26	0,041442	0,544646		
TC0900001424.mm.1	2,62	Celsr3; Gm23156	PSR0900011553.mm.1	-2,38	0,014755	0,450728	Cassette Exon	0,21
TC0900001424.mm.1	2,62	Celsr3; Gm23156	JUC0900006399.mm.1	-2,47	0,007697	0,402552		
TC0900001424.mm.1	2,62	Celsr3; Gm23156	PSR0900011558.mm.1	-2,6	0,001865	0,337974	Cassette Exon	0,28
TC0900001424.mm.1	2,62	Celsr3; Gm23156	JUC0900006390.mm.1	-2,61	0,031981	0,518197		
TC0900001424.mm.1	2,62	Celsr3; Gm23156	JUC0900006378.mm.1	-2,8	0,000538	0,304044		
TC0900001424.mm.1	2,62	Celsr3; Gm23156	JUC0900006372.mm.1	-2,94	0,004165	0,3623		
TC0900001424.mm.1	2,62	Celsr3; Gm23156	JUC0900006388.mm.1	-3,89	0,000251	0,28803		
TC0900001424.mm.1	2,62	Celsr3; Gm23156	JUC0900006383.mm.1	-4,3	0,001368	0,32572		
TC0200005154.mm.1	-2,45		PSR0200044037.mm.1	3,03	0,010835	0,424161	Cassette Exon	0,25
TC0200005154.mm.1	-2,45		JUC0200022720.mm.1	2,4	0,043549	0,550022		
TC0500001531.mm.1	2,17	Caln1	JUC0500007592.mm.1	3,03	0,041331	0,544314		
TC0500001531.mm.1	2,17	Caln1	PSR0500013861.mm.1	-2,03	0,009565	0,416927	Cassette Exon	0,12
TC0500001531.mm.1	2,17	Caln1	PSR0500013852.mm.1	-2,71	0,014863	0,451623	Cassette Exon	0,16
TC0500001531.mm.1	2,17	Caln1	PSR0500013854.mm.1	-3,13	0,017641	0,464582	Cassette Exon	0,23
TC0500001531.mm.1	2,17	Caln1	PSR0500013856.mm.1	-3,62	0,002781	0,351017	Cassette Exon	0,19
TC0300002325.mm.1	-1,07	1500004A13Rik; RP23-19	PSR0300017715.mm.1	3,03	0,02623	0,50019	Alternative 5' Donc	0,11
TC1700001927.mm.1	-1,15	Cyp21a1	JUC1700009708.mm.1	3,03	0,040142	0,541609		
TC0900000153.mm.1	1,35	Zfp317	JUC0900000505.mm.1	3,03	0,037635	0,534189		
TC0600002781.mm.1	-1,34	1700010K10Rik	JUC0600011211.mm.1	3,03	0,004009	0,36119		

TC0700003740.mm.1	1,42	Pcf11	JUC0700016659.mm.1	3,03	0,006656	0,393187		
TC0700003740.mm.1	1,42	Pcf11	JUC0700016660.mm.1	-2,43	0,004454	0,367239		
TC1200000866.mm.1	4,19	Tmem63c	PSR1200006336.mm.1	3,02	0,002078	0,342181	Cassette Exon	0,19
TC1200000866.mm.1	4,19	Tmem63c	PSR1200006335.mm.1	2,08	0,016199	0,458183	Cassette Exon	0,09
TC1200000866.mm.1	4,19	Tmem63c	JUC1200003586.mm.1	-2,15	0,029125	0,509285		
TC1200000866.mm.1	4,19	Tmem63c	PSR1200006345.mm.1	-2,32	0,002856	0,352207	Cassette Exon	0,13
TC1200000866.mm.1	4,19	Tmem63c	JUC1200003585.mm.1	-3,14	0,004815	0,371408		
TC1200000866.mm.1	4,19	Tmem63c	PSR1200006328.mm.1	-3,28	0,003786	0,357586	Cassette Exon	0,37
TC1200000866.mm.1	4,19	Tmem63c	JUC1200003577.mm.1	-3,4	0,009947	0,41841		
TC1200000866.mm.1	4,19	Tmem63c	PSR1200006327.mm.1	-3,46	0,002496	0,349135	Cassette Exon	0,02
TC1200000866.mm.1	4,19	Tmem63c	PSR1200006329.mm.1	-3,48	0,001524	0,330287	Cassette Exon	0,33
TC1200000866.mm.1	4,19	Tmem63c	PSR1200006354.mm.1	-3,75	0,010142	0,419347	Cassette Exon	0,25
TC1200000866.mm.1	4,19	Tmem63c	PSR1200006352.mm.1	-4,12	0,011585	0,429241	Cassette Exon	0,29
TC1200000866.mm.1	4,19	Tmem63c	JUC1200003565.mm.1	-4,21	0,024406	0,4937		
TC1200000866.mm.1	4,19	Tmem63c	PSR1200006353.mm.1	-4,36	0,015254	0,452847	Cassette Exon	0,28
TC1200000866.mm.1	4,19	Tmem63c	JUC1200003567.mm.1	-4,65	0,001698	0,335395		
TC1200000866.mm.1	4,19	Tmem63c	PSR1200006325.mm.1	-4,68	0,004675	0,370194	Cassette Exon	0,07
TC1200000866.mm.1	4,19	Tmem63c	PSR1200006322.mm.1	-5,09	0,011459	0,427962	Cassette Exon	0,41
TC1200000866.mm.1	4,19	Tmem63c	PSR1200006323.mm.1	-5,52	0,001572	0,331411	Alternative 3' Acce	0,16
TC1200000866.mm.1	4,19	Tmem63c	JUC1200003583.mm.1	-5,63	0,00021	0,28803		
TC0X00002815.mm.1	2,54	5330434G04Rik	PSR0X00017813.mm.1	3,02	0,012453	0,43528	Alternative 3' Acce	0,32
TC0X00002815.mm.1	2,54	5330434G04Rik	PSR0X00017824.mm.1	2,35	0,020148	0,477043	Cassette Exon	0,12
TC0X00002815.mm.1	2,54	5330434G04Rik	PSR0X00017821.mm.1	-2,55	0,038612	0,536911	Cassette Exon	0,19
TC0X00002815.mm.1	2,54	5330434G04Rik	PSR0X00017822.mm.1	-2,92	0,006831	0,393893	Alternative 3' Acce	0,26
TC0X00002815.mm.1	2,54	5330434G04Rik	PSR0X00017827.mm.1	-2,99	0,007722	0,402552	Alternative 3' Acce	0,19
TC0X00002815.mm.1	2,54	5330434G04Rik	PSR0X00017828.mm.1	-3,44	0,00023	0,28803	Intron Retention	0,33
TC0X00002815.mm.1	2,54	5330434G04Rik	PSR0X00017826.mm.1	-3,5	0,016431	0,459007	Alternative 3' Acce	0,19
TC0X00002815.mm.1	2,54	5330434G04Rik	JUC0X00008981.mm.1	-5,98	0,01159	0,429241		
TC0X00000405.mm.1	2,02	Sh2d1a	PSR0X00002733.mm.1	3,02	0,016232	0,458312	Cassette Exon	0,24
TC0X00000405.mm.1	2,02	Sh2d1a	PSR0X00002728.mm.1	-2,37	0,036904	0,532283	Alternative 3' Acce	0,3
TC0X00000405.mm.1	2,02	Sh2d1a	PSR0X00002726.mm.1	-3,44	0,005551	0,380756	Alternative 3' Acce	0,3
TC0X00000405.mm.1	2,02	Sh2d1a	PSR0X00002725.mm.1	-5,09	0,012429	0,43528	Alternative 3' Acce	0,3
TC0X00000405.mm.1	2,02	Sh2d1a	PSR0X00002727.mm.1	-5,19	0,029854	0,511132	Alternative 3' Acce	0,3
TC0X00001619.mm.1	2,67	Map7d2	JUC0X00005108.mm.1	3,02	0,018496	0,468939		
TC0X00001619.mm.1	2,67	Map7d2	JUC0X00005112.mm.1	2,41	0,011962	0,431914		
TC0X00001619.mm.1	2,67	Map7d2	JUC0X00005114.mm.1	-2,16	0,024977	0,496		
TC0X00001619.mm.1	2,67	Map7d2	PSR0X00010170.mm.1	-2,41	0,007635	0,402113	Cassette Exon	0,13
TC0X00001619.mm.1	2,67	Map7d2	PSR0X00010189.mm.1	-2,63	0,02934	0,509981	Cassette Exon	0,15
TC0X00001619.mm.1	2,67	Map7d2	PSR0X00010176.mm.1	-2,78	0,003673	0,357266	Alternative 3' Acce	0,23
TC0X00001619.mm.1	2,67	Map7d2	PSR0X00010178.mm.1	-2,79	0,012327	0,434392		
TC0X00001619.mm.1	2,67	Map7d2	PSR0X00010196.mm.1	-2,86	0,025399	0,497403	Alternative 5' Donc	0,2
TC0X00001619.mm.1	2,67	Map7d2	JUC0X00005120.mm.1	-2,88	0,010033	0,41878		
TC0X00001619.mm.1	2,67	Map7d2	JUC0X00005115.mm.1	-3,48	0,011602	0,429473		
TC0700000596.mm.1	-2,2	Scgb1b12	PSR0700005285.mm.1	3,02	0,018984	0,471445	Cassette Exon	0,22
TC0300000778.mm.1	-1,16	Tpm3	PSR0300006009.mm.1	3,02	0,027837	0,50552	Cassette Exon	0,19
TC0X00002739.mm.1	-2,22	Cited1	PSR0X00017287.mm.1	3,02	0,03734	0,533573	Cassette Exon	0,19
TC0X00002739.mm.1	-2,22	Cited1	PSR0X00017283.mm.1	2,51	0,04191	0,545811	Cassette Exon	0,17
TC0X00002739.mm.1	-2,22	Cited1	PSR0X00017279.mm.1	2,35	0,049025	0,561902		

TC0200005442.mm.1	2,2	Stmn3	JUC0200023917.mm.1	3,02	0,020769	0,479303		
TC0200005442.mm.1	2,2	Stmn3	PSR0200046406.mm.1	2,16	0,014338	0,448173	Cassette Exon	0,11
TC0200005442.mm.1	2,2	Stmn3	PSR0200046413.mm.1	-2,51	0,013583	0,443703	Cassette Exon	0,05
TC0200005442.mm.1	2,2	Stmn3	PSR0200046415.mm.1	-3,03	0,020127	0,47686	Alternative 5' Donc	0,08
TC1800001462.mm.1	1,02	Nars	JUC1800006007.mm.1	3,02	0,037861	0,534818		
TC1500001690.mm.1	1,28	Ptk2	JUC1500007258.mm.1	3,02	0,030198	0,512394		
TC1500001690.mm.1	1,28	Ptk2	JUC1500007224.mm.1	2,3	0,036848	0,532256		
TC1500001690.mm.1	1,28	Ptk2	JUC1500007259.mm.1	-2,64	0,033443	0,522793		
TC0900001633.mm.1	-1,19	Fam198a	JUC0900007696.mm.1	3,02	0,000811	0,311886		
TC0700001131.mm.1	-1,61	Ticrr	JUC0700005115.mm.1	3,02	0,014752	0,450714		
TC0600002745.mm.1	-1,17	Prickle2	JUC0600011087.mm.1	3,02	0,000057	0,24627		
TC0600001258.mm.1	-1,42	Irak2	JUC0600005114.mm.1	3,02	0,045305	0,553742		
TC0500000057.mm.1	1,4	Tmem243	JUC0500000300.mm.1	3,02	0,011437	0,427663		
TC0X00002307.mm.1	1,06	Atp11c	JUC0X00007442.mm.1	3,02	0,004919	0,37289		
TC0X00002307.mm.1	1,06	Atp11c	JUC0X00007462.mm.1	2,2	0,04282	0,548097		
TC1100001802.mm.1	-1,49	Helz	PSR1100016991.mm.1	3,01	0,024928	0,49582	Cassette Exon	0,25
TC1100001802.mm.1	-1,49	Helz	PSR1100017000.mm.1	2,52	0,043766	0,55037	Cassette Exon	0,15
TC1200001931.mm.1	1,5	Kcnh5	PSR1200013260.mm.1	3,01	0,003118	0,353892	Cassette Exon	0,18
TC1200001931.mm.1	1,5	Kcnh5	PSR1200013266.mm.1	2,88	0,008726	0,409438	Cassette Exon	0,23
TC1200001931.mm.1	1,5	Kcnh5	JUC1200007358.mm.1	2,43	0,012672	0,43631		
TC1200001931.mm.1	1,5	Kcnh5	PSR1200013258.mm.1	2,32	0,046482	0,556634	Cassette Exon	0,12
TC1200001931.mm.1	1,5	Kcnh5	PSR1200013265.mm.1	2,16	0,031856	0,517902	Cassette Exon	0,13
TC0X00002803.mm.1	-1,56	Zdhhc15	JUC0X00008928.mm.1	3,01	0,002481	0,349135		
TC0X00002803.mm.1	-1,56	Zdhhc15	PSR0X00017673.mm.1	2,28	0,005582	0,381035	Cassette Exon	0,21
TC0700003171.mm.1	6,25	Gm22130	PSR0700028355.mm.1	3,01	0,001078	0,317191	Cassette Exon	0,19
TC1200001725.mm.1	1,04	Heatr5a	JUC1200006608.mm.1	3,01	0,018299	0,468049		
TC1200001725.mm.1	1,04	Heatr5a	JUC1200006597.mm.1	2,81	0,01499	0,452048		
TC1200001725.mm.1	1,04	Heatr5a	JUC1200006596.mm.1	2,41	0,000878	0,311909		
TC1200001725.mm.1	1,04	Heatr5a	PSR1200011889.mm.1	2,27	0,013208	0,440872	Cassette Exon	0,14
TC1200001725.mm.1	1,04	Heatr5a	JUC1200006599.mm.1	2,23	0,031853	0,517902		
TC1200001725.mm.1	1,04	Heatr5a	PSR1200011890.mm.1	2,17	0,035668	0,528966	Cassette Exon	0,12
TC1200001725.mm.1	1,04	Heatr5a	PSR1200011872.mm.1	2,1	0,024718	0,494838	Cassette Exon	0,16
TC1200001725.mm.1	1,04	Heatr5a	PSR1200011882.mm.1	2,06	0,007367	0,39938	Cassette Exon	0,19
TC1700001500.mm.1	1,43	Pdcd2	JUC1700007333.mm.1	3,01	0,041166	0,544039		
TC1700001500.mm.1	1,43	Pdcd2	PSR1700013436.mm.1	-2,16	0,031266	0,516032	Alternative 3' Acce	0,18
TC1700000665.mm.1	-1,95	Ly6g6e	PSR1700006712.mm.1	3,01	0,02525	0,496939	Alternative 3' Acce	0,15
TC1700000665.mm.1	-1,95	Ly6g6e	PSR1700006717.mm.1	2,1	0,03104	0,515011	Cassette Exon	0,14
TC1800000456.mm.1	-1,06		JUC1800001880.mm.1	3,01	0,030655	0,513785		
TC0100000985.mm.1	1,17	Cntnap5b	PSR0100008268.mm.1	3,01	0,004283	0,363973		
TC0100000985.mm.1	1,17	Cntnap5b	JUC0100004748.mm.1	2,48	0,019202	0,472922		
TC0100000985.mm.1	1,17	Cntnap5b	JUC0100004753.mm.1	-2,2	0,0259	0,498978		
TC0100000985.mm.1	1,17	Cntnap5b	JUC0100004766.mm.1	-2,61	0,049575	0,563357		
TC1700000412.mm.1	1,09	Neurl1b	JUC1700001897.mm.1	3,01	0,009847	0,417839		
TC0600003503.mm.1	-1,29	Herc6	JUC0600002711.mm.1	3,01	0,00472	0,370888		
TC0600003503.mm.1	-1,29	Herc6	JUC0600002703.mm.1	2,23	0,008577	0,40821		
TC0600002733.mm.1	1,14	Grip2	JUC0600011036.mm.1	3,01	0,000322	0,288663		
TC0600003604.mm.1	-1,09	Atn1	JUC0600012584.mm.1	3,01	0,037915	0,535116		
TC1000001708.mm.1	-1,02	Ust	JUC1000006773.mm.1	3,01	0,040572	0,542455		

TC1000000718.mm.1	-1,44	Ggt5	JUC1000002822.mm.1	3,01	0,029633	0,510569		
TC0500003556.mm.1	-1,29	Zdhhc4	PSR0500032603.mm.1	3	0,011688	0,429969	Alternative 3' Acce	0,28
TC0800002959.mm.1	1,22	Tmem231	JUC0800012501.mm.1	3	0,048584	0,561078		
TC0700001431.mm.1	-1,17	Arap1	JUC0700006355.mm.1	3	0,026668	0,501915		
TC0700001431.mm.1	-1,17	Arap1	JUC0700006361.mm.1	-2,34	0,016209	0,458312		
TC0700002491.mm.1	-1,15	Relb	JUC0700011729.mm.1	3	0,025249	0,496939		
TC1100000930.mm.1	-1,03	Ybx2	JUC1100004200.mm.1	3	0,010033	0,41878		
TC1100001744.mm.1	1,23	Tlk2	JUC1100008565.mm.1	3	0,016818	0,460623		
TC0400003208.mm.1	1	Acot11	JUC0400013284.mm.1	3	0,039194	0,538692		
TC1800000105.mm.1	2,36	Ttc39c	JUC1800000467.mm.1	2,99	0,009262	0,414559		
TC1800000105.mm.1	2,36	Ttc39c	JUC1800000465.mm.1	2,78	0,010591	0,42255		
TC1800000105.mm.1	2,36	Ttc39c	PSR1800000797.mm.1	-2,25	0,004943	0,373242	Alternative 5' Donc	0,19
TC1800000105.mm.1	2,36	Ttc39c	PSR1800000793.mm.1	-2,81	0,012714	0,43654	Alternative 3' Acce	0,25
TC1800000105.mm.1	2,36	Ttc39c	PSR1800000798.mm.1	-2,81	0,000565	0,304044	Cassette Exon	0,09
TC1800000105.mm.1	2,36	Ttc39c	PSR1800000792.mm.1	-3,56	0,002421	0,34896	Alternative 3' Acce	0,25
TC1800000105.mm.1	2,36	Ttc39c	PSR1800000802.mm.1	-3,93	0,000445	0,297771	Alternative 5' Donc	0,52
TC0700000038.mm.1	2,37	Brsk1	JUC0700000242.mm.1	2,99	0,024249	0,493315		
TC0700000038.mm.1	2,37	Brsk1	JUC0700000234.mm.1	2,87	0,002775	0,350935		
TC0700000038.mm.1	2,37	Brsk1	PSR0700000478.mm.1	-2,11	0,023057	0,489443	Cassette Exon	0,12
TC0700000038.mm.1	2,37	Brsk1	PSR0700000494.mm.1	-2,25	0,000865	0,311909	Cassette Exon	0,08
TC0700000038.mm.1	2,37	Brsk1	PSR0700000501.mm.1	-2,3	0,002552	0,349501	Cassette Exon	0,12
TC0700000038.mm.1	2,37	Brsk1	PSR0700000500.mm.1	-2,87	0,002213	0,34564	Alternative 5' Donc	0,3
TC0700000038.mm.1	2,37	Brsk1	PSR0700000490.mm.1	-3	0,017982	0,466929	Alternative 5' Donc	0,33
TC0700000038.mm.1	2,37	Brsk1	PSR0700000479.mm.1	-3,02	0,00263	0,349612	Alternative 3' Acce	0,18
TC0400003559.mm.1	1,03	Zmym1	PSR0400029016.mm.1	2,99	0,03619	0,530002	Alternative 5' Donc	0,32
TC0400003559.mm.1	1,03	Zmym1	JUC0400015151.mm.1	-5,31	0,012802	0,437296		
TC0600000669.mm.1	-1,4	Gm3279	PSR0600005191.mm.1	2,99	0,005579	0,381035	Cassette Exon	0,18
TC0400002847.mm.1	-1,05	Cdk5rap2; Gm11226; Ywh	JUC0400012153.mm.1	2,99	0,005456	0,379939		
TC0400002847.mm.1	-1,05	Cdk5rap2; Gm11226; Ywh	JUC0400012142.mm.1	2,43	0,006593	0,39213		
TC0400002847.mm.1	-1,05	Cdk5rap2; Gm11226; Ywh	JUC0400012125.mm.1	2,22	0,017284	0,463143		
TC0400002847.mm.1	-1,05	Cdk5rap2; Gm11226; Ywh	PSR0400023260.mm.1	2,03	0,00004	0,245341	Cassette Exon	0,15
TC0Y00000148.mm.1	-1,36	Gm21117; Gm21258	JUC0Y00000436.mm.1	2,99	0,008259	0,405976		
TC0Y00000148.mm.1	-1,36	Gm21117; Gm21258	PSR0Y00000651.mm.1	-2,13	0,027012	0,502979	Cassette Exon	0,1
TC0Y00000148.mm.1	-1,36	Gm21117; Gm21258	PSR0Y00000660.mm.1	-2,31	0,043488	0,549901	Cassette Exon	0,11
TC1200000651.mm.1	-1,5		JUC1200002510.mm.1	2,99	0,038641	0,537096		
TC0300001193.mm.1	-1,57	Lrrc39	JUC0300005064.mm.1	2,99	0,030968	0,514654		
TC0200003528.mm.1	-1,38	Nmi	JUC0200015113.mm.1	2,99	0,002467	0,349135		
TC1700001774.mm.1	-1,02	Stk38; Gm23887	JUC1700008817.mm.1	2,99	0,025786	0,498505		
TC0100001016.mm.1	-1,1	Zcchc2	JUC0100004849.mm.1	2,99	0,039247	0,53893		
TC1600001663.mm.1	1,46	Tagln3	JUC1600007098.mm.1	2,99	0,00288	0,352207		
TC0700000410.mm.1	-1,46	Cic	JUC0700001521.mm.1	2,99	0,016361	0,458693		
TC1100003965.mm.1	2,03	Cacng5	JUC1100019439.mm.1	2,98	0,012095	0,43255		
TC1100003965.mm.1	2,03	Cacng5	PSR1100037211.mm.1	-2,09	0,00255	0,349501	Cassette Exon	0,02
TC1100003965.mm.1	2,03	Cacng5	JUC1100019441.mm.1	-2,41	0,027252	0,503642		
TC1100003965.mm.1	2,03	Cacng5	JUC1100019438.mm.1	-3,01	0,004205	0,363236		
TC1100003965.mm.1	2,03	Cacng5	JUC1100019442.mm.1	-3,12	0,000255	0,28803		
TC1100003965.mm.1	2,03	Cacng5	PSR1100037202.mm.1	-3,65	0,046116	0,555792	Alternative 3' Acce	0,42
TC0200004556.mm.1	3,72	Kcnip3	PSR0200039036.mm.1	2,98	0,020007	0,476351	Cassette Exon	0,19

TC0200004556.mm.1	3,72	Kcnip3	PSR0200039040.mm.1	-2,16	0,03528	0,52837	Cassette Exon	0,08
TC0200004556.mm.1	3,72	Kcnip3	PSR0200039029.mm.1	-2,28	0,00488	0,372195	Alternative 3' Acce	0,2
TC0200004556.mm.1	3,72	Kcnip3	PSR0200039038.mm.1	-3,33	0,011151	0,42626	Cassette Exon	0,32
TC0200004556.mm.1	3,72	Kcnip3	JUC0200020209.mm.1	-3,55	0,0124	0,434948		
TC1400001914.mm.1	-2,58	Bmp4	PSR1400014472.mm.1	2,98	0,044144	0,551024	Alternative 5' Donc	0,3
TC1200000173.mm.1	-1,08		JUC1200000774.mm.1	2,98	0,007893	0,403819		
TC1200000173.mm.1	-1,08		PSR1200001366.mm.1	2,67	0,001517	0,330268	Cassette Exon	0,27
TC1200000173.mm.1	-1,08		JUC1200000775.mm.1	2,65	0,009925	0,418378		
TC0500000670.mm.1	-2,52	Atp10d	PSR0500005910.mm.1	2,98	0,006337	0,389463	Cassette Exon	0,26
TC0500000670.mm.1	-2,52	Atp10d	JUC0500003250.mm.1	2,77	0,006158	0,387873		
TC0500000670.mm.1	-2,52	Atp10d	PSR0500005909.mm.1	2,58	0,002224	0,345773	Cassette Exon	0,24
TC0500000670.mm.1	-2,52	Atp10d	JUC0500003271.mm.1	2,41	0,031947	0,518113		
TC0700003031.mm.1	1,19	Abcc8	PSR0700027601.mm.1	2,98	0,001439	0,32773	Cassette Exon	0,22
TC0X00000618.mm.1	-1	Aff2	PSR0X00003915.mm.1	2,98	0,025541	0,497789	Cassette Exon	0,19
TC1600000344.mm.1	-1,93	LOC102633188	PSR1600003423.mm.1	2,98	0,019795	0,475212	Cassette Exon	0,18
TC0100001434.mm.1	-1,16	Sec16b	JUC0100006731.mm.1	2,98	0,004934	0,373093		
TC1400002753.mm.1	-1,24	2610035F20Rik	JUC1400011331.mm.1	2,98	0,012666	0,43631		
TC1500000604.mm.1	1,29	Ppp1r16a	JUC1500002528.mm.1	2,98	0,020668	0,478862		
TC0900001670.mm.1	-1,36	Ccr9; Gm17200	JUC0900007905.mm.1	2,98	0,023254	0,490032		
TC0900001462.mm.1	1,01	Kif9	JUC0900006798.mm.1	2,98	0,015287	0,453287		
TC0500001130.mm.1	1,09	Plcxd1	JUC0500005233.mm.1	2,98	0,0011	0,317328		
TC0500001130.mm.1	1,09	Plcxd1	JUC0500005237.mm.1	2,03	0,000953	0,313363		
TC0700003448.mm.1	-1,23	Lrrc28	JUC0700015535.mm.1	2,98	0,034565	0,525975		
TC0400000185.mm.1	-1,02	Coq3	JUC0400000598.mm.1	2,98	0,029151	0,509333		
TC1000001743.mm.1	-1,59	Adgb	JUC1000006878.mm.1	2,98	0,003866	0,358861		
TC1000001743.mm.1	-1,59	Adgb	JUC1000006863.mm.1	2,74	0,004578	0,369794		
TC1000001743.mm.1	-1,59	Adgb	JUC1000006872.mm.1	2,64	0,043716	0,550256		
TC1000001743.mm.1	-1,59	Adgb	JUC1000006851.mm.1	2,21	0,005783	0,383104		
TC1000001743.mm.1	-1,59	Adgb	JUC1000006853.mm.1	-2,05	0,007327	0,399002		
TC1000001743.mm.1	-1,59	Adgb	JUC1000006870.mm.1	-2,41	0,00044	0,297771		
TC1000001336.mm.1	-2	Ptprb	JUC1000005415.mm.1	2,98	0,005131	0,376377		
TC0300001490.mm.1	-1,24	Lpar3	JUC0300006360.mm.1	2,98	0,001703	0,335511		
TC0900002236.mm.1	-1,08	Rbm7	PSR0900018661.mm.1	2,97	0,017032	0,461729	Intron Retention	0,36
TC0900002236.mm.1	-1,08	Rbm7	JUC0900010510.mm.1	2,05	0,032562	0,520128		
TC1400001253.mm.1	1,07	Gpc6	PSR1400009608.mm.1	2,97	0,006067	0,386691	Cassette Exon	0,28
TC1400001253.mm.1	1,07	Gpc6	JUC1400005120.mm.1	-2,29	0,046697	0,556856		
TC0300002743.mm.1	1,27	5330417C22Rik	JUC0300011247.mm.1	2,97	0,005358	0,378582		
TC0300002743.mm.1	1,27	5330417C22Rik	PSR0300021586.mm.1	-2,32	0,026085	0,499688	Cassette Exon	0,1
TC0300002743.mm.1	1,27	5330417C22Rik	PSR0300021592.mm.1	-2,34	0,02107	0,480303	Alternative 5' Donc	0,23
TC0200004312.mm.1	1,55	Ano3	JUC0200018441.mm.1	2,97	0,048719	0,561355		
TC0200004312.mm.1	1,55	Ano3	JUC0200018436.mm.1	2,47	0,005215	0,377078		
TC0200004312.mm.1	1,55	Ano3	PSR0200036126.mm.1	-2,16	0,020656	0,478862	Cassette Exon	0,13
TC0200004312.mm.1	1,55	Ano3	JUC0200018444.mm.1	-2,21	0,008464	0,406624		
TC0200004312.mm.1	1,55	Ano3	PSR0200036123.mm.1	-2,25	0,007584	0,40171	Cassette Exon	0,14
TC0200004312.mm.1	1,55	Ano3	PSR0200036125.mm.1	-2,27	0,026676	0,501977	Cassette Exon	0,13
TC0200004312.mm.1	1,55	Ano3	JUC0200018447.mm.1	-3,95	0,001075	0,316759		
TC0700003770.mm.1	-1,03	Myo7a	JUC0700016772.mm.1	2,97	0,017382	0,463704		
TC0700003770.mm.1	-1,03	Myo7a	PSR0700031724.mm.1	-2,25	0,013244	0,441395	Alternative 3' Acce	0,14

TC1900000206.mm.1	-1,09	Vwce	JUC1900001348.mm.1	2,97	0,038974	0,53792		
TC1500002109.mm.1	-1,04	Adamts20	JUC1500009644.mm.1	2,97	0,049735	0,563552		
TC1500002109.mm.1	-1,04	Adamts20	PSR1500016948.mm.1	-2,12	0,015379	0,453849		
TC0200000427.mm.1	-1,1	Lcn8	JUC0200001216.mm.1	2,97	0,028775	0,508148		
TC0500001911.mm.1	1,16	A330021E22Rik	JUC0500009575.mm.1	2,97	0,003114	0,353892		
TC0500001911.mm.1	1,16	A330021E22Rik	JUC0500009584.mm.1	-3,25	0,000536	0,304044		
TC0300001645.mm.1	-1,24	Zfand1	JUC0300006930.mm.1	2,97	0,033043	0,521532		
TC0300001645.mm.1	-1,24	Zfand1	JUC0300006934.mm.1	2,42	0,02676	0,502096		
TC0X00000426.mm.1	-1,47	Xpnpep2	JUC0X00001415.mm.1	2,97	0,000437	0,297771		
TC0900002870.mm.1	1,33	Pls1	JUC0900013225.mm.1	2,97	0,001848	0,337414		
TC1900000417.mm.1	7,08	Kcnv2	JUC1900002027.mm.1	2,96	0,02127	0,481596		
TC1900000417.mm.1	7,08	Kcnv2	PSR1900003899.mm.1	-3,58	0,025504	0,497664	Alternative 3' Acce	0,41
TC0200000477.mm.1	-1,05	Rxra	PSR0200003231.mm.1	2,96	0,024788	0,495082	Cassette Exon	0,18
TC1000001228.mm.1	1,77	Mgat4c; 4930402I19Rik	JUC1000005012.mm.1	2,96	0,007665	0,402377		
TC1000001228.mm.1	1,77	Mgat4c; 4930402I19Rik	PSR1000009278.mm.1	-2,05	0,013123	0,439932	Alternative 5' Donc	0,15
TC1000001228.mm.1	1,77	Mgat4c; 4930402I19Rik	PSR1000009287.mm.1	-2,05	0,033278	0,522358	Cassette Exon	0,07
TC1000001228.mm.1	1,77	Mgat4c; 4930402I19Rik	JUC1000005018.mm.1	-2,22	0,03988	0,54093		
TC1000001228.mm.1	1,77	Mgat4c; 4930402I19Rik	PSR1000009279.mm.1	-2,23	0,047687	0,558901	Alternative 5' Donc	0,15
TC1000001228.mm.1	1,77	Mgat4c; 4930402I19Rik	PSR1000009283.mm.1	-2,32	0,022872	0,488524	Cassette Exon	0,12
TC1000001228.mm.1	1,77	Mgat4c; 4930402I19Rik	PSR1000009273.mm.1	-2,45	0,037869	0,534872	Alternative 3' Acce	0,16
TC1000001228.mm.1	1,77	Mgat4c; 4930402I19Rik	PSR1000009272.mm.1	-3,05	0,015384	0,453849	Cassette Exon	0,16
TC1000001228.mm.1	1,77	Mgat4c; 4930402I19Rik	JUC1000005020.mm.1	-3,26	0,008146	0,405457		
TC0400001758.mm.1	1,48	Gm13029	PSR0400014527.mm.1	2,96	0,004847	0,37168	Cassette Exon	0,05
TC0400001758.mm.1	1,48	Gm13029	JUC0400007638.mm.1	-8,53	0,024388	0,493641		
TC1400002027.mm.1	1,51	Chd8	JUC1400008333.mm.1	2,96	0,036396	0,530877		
TC0300000639.mm.1	-1,39	Rbm46os; 4930564K09Rik	JUC0300002198.mm.1	2,96	0,044666	0,55216		
TC0300000639.mm.1	-1,39	Rbm46os; 4930564K09Rik	JUC0300002205.mm.1	2,43	0,018873	0,4711		
TC0300000631.mm.1	-1,27	Pdgfc	JUC0300002154.mm.1	2,96	0,002697	0,349612		
TC0200003837.mm.1	-1,08	Nckap1	JUC0200017175.mm.1	2,96	0,033686	0,523549		
TC0700004487.mm.1	1,15	Adam8	JUC0700019866.mm.1	2,96	0,009996	0,418456		
TC1100000565.mm.1	1,09	Clk4; Gm25082	JUC1100002256.mm.1	2,96	0,032149	0,518586		
TC1000001542.mm.1	-1,47	Ctdsp2; Mir26a-2; Mir546	PSR1000010862.mm.1	2,96	0,003006	0,353447		
TC1100002988.mm.1	-1,28	Map2k4	JUC1100014121.mm.1	2,96	0,013558	0,443655		
TC0900001012.mm.1	8,28	Gnb5	JUC0900004334.mm.1	2,95	0,006572	0,39213		
TC0900001012.mm.1	8,28	Gnb5	JUC0900004335.mm.1	2,26	0,004538	0,368648		
TC0900001012.mm.1	8,28	Gnb5	JUC0900004336.mm.1	2,06	0,02356	0,490947		
TC0900001012.mm.1	8,28	Gnb5	PSR0900008004.mm.1	-2,03	0,028154	0,506347	Alternative 5' Donc	0,13
TC0900001012.mm.1	8,28	Gnb5	PSR0900007992.mm.1	-2,83	0,004737	0,370933	Cassette Exon	0,39
TC0900001012.mm.1	8,28	Gnb5	JUC0900004345.mm.1	-6,98	0,014949	0,451968		
TC0900001012.mm.1	8,28	Gnb5	PSR0900007989.mm.1	-7,2	0,005624	0,381314	Alternative 3' Acce	0,44
TC0900001012.mm.1	8,28	Gnb5	PSR0900007985.mm.1	-9,15	0,000205	0,28803	Cassette Exon	0,28
TC0200000929.mm.1	3,58	Slc4a10	JUC0200003794.mm.1	2,95	0,010945	0,425002		
TC0200000929.mm.1	3,58	Slc4a10	JUC0200003798.mm.1	2,44	0,009865	0,417945		
TC0200000929.mm.1	3,58	Slc4a10	PSR0200007464.mm.1	2,37	0,019228	0,472971	Cassette Exon	0,14
TC0200000929.mm.1	3,58	Slc4a10	JUC0200003803.mm.1	2,07	0,008591	0,408237		
TC0200000929.mm.1	3,58	Slc4a10	PSR0200007450.mm.1	-2,02	0,013949	0,446234	Cassette Exon	0,19
TC0200000929.mm.1	3,58	Slc4a10	PSR0200007459.mm.1	-2,24	0,012778	0,437233	Cassette Exon	0,15
TC0200000929.mm.1	3,58	Slc4a10	PSR0200007455.mm.1	-2,37	0,011061	0,425887	Cassette Exon	0,35

TC0200000929.mm.1	3,58	Slc4a10	PSR0200007443.mm.1	-2,53	0,004362	0,365487	Cassette Exon	0,22
TC0200000929.mm.1	3,58	Slc4a10	JUC0200003801.mm.1	-2,76	0,034229	0,525168		
TC0200000929.mm.1	3,58	Slc4a10	PSR0200007461.mm.1	-2,83	0,046262	0,556176	Alternative 3' Acce	0,18
TC0200000929.mm.1	3,58	Slc4a10	PSR0200007444.mm.1	-2,89	0,007258	0,39826	Cassette Exon	0,22
TC0200000929.mm.1	3,58	Slc4a10	JUC0200003808.mm.1	-3,04	0,029908	0,511464		
TC0200000929.mm.1	3,58	Slc4a10	PSR0200007446.mm.1	-3,16	0,021974	0,485125	Cassette Exon	0,38
TC0200000929.mm.1	3,58	Slc4a10	JUC0200003813.mm.1	-3,4	0,040877	0,543423		
TC0200000929.mm.1	3,58	Slc4a10	PSR0200007441.mm.1	-4,2	0,008041	0,40434	Alternative 3' Acce	0,23
TC0200000929.mm.1	3,58	Slc4a10	PSR0200007440.mm.1	-4,22	0,007801	0,403089	Alternative 3' Acce	0,23
TC0200000929.mm.1	3,58	Slc4a10	JUC0200003814.mm.1	-4,59	0,011684	0,429961		
TC0200000929.mm.1	3,58	Slc4a10	JUC0200003800.mm.1	-5,01	0,019484	0,474383		
TC0200000929.mm.1	3,58	Slc4a10	JUC0200003809.mm.1	-5,33	0,037681	0,534394		
TC0200000929.mm.1	3,58	Slc4a10	JUC0200003806.mm.1	-6,46	0,022186	0,485756		
TC0400001683.mm.1	1,25	Hnrnpr; 9130020K20Rik	PSR0400013548.mm.1	2,95	0,037839	0,534803	Alternative 3' Acce	0,31
TC0200004594.mm.1	6,05	AI847159	JUC0200020394.mm.1	2,95	0,048872	0,561713		
TC0200004594.mm.1	6,05	AI847159	PSR0200039359.mm.1	2,05	0,008658	0,408463		
TC0200004594.mm.1	6,05	AI847159	PSR0200039365.mm.1	-4,82	0,035989	0,529491	Cassette Exon	0,28
TC0200004594.mm.1	6,05	AI847159	JUC0200020395.mm.1	-5,3	0,016313	0,45857		
TC0200004594.mm.1	6,05	AI847159	JUC0200020397.mm.1	-6,73	0,005608	0,381115		
TC0200004594.mm.1	6,05	AI847159	PSR0200039353.mm.1	-6,82	0,003357	0,354243	Cassette Exon	0,28
TC0200004594.mm.1	6,05	AI847159	PSR0200039366.mm.1	-8,58	0,01564	0,455245	Cassette Exon	0,28
TC0500001451.mm.1	-1,43	Atp6v0a2	JUC0500007149.mm.1	2,95	0,040817	0,543269		
TC0500001451.mm.1	-1,43	Atp6v0a2	JUC0500007159.mm.1	2,85	0,025447	0,497549		
TC0500001451.mm.1	-1,43	Atp6v0a2	JUC0500007154.mm.1	2,73	0,035864	0,529376		
TC0500001451.mm.1	-1,43	Atp6v0a2	PSR0500013072.mm.1	2,3	0,016803	0,460623	Cassette Exon	0,22
TC0500001451.mm.1	-1,43	Atp6v0a2	PSR0500013083.mm.1	2,1	0,048212	0,560209	Cassette Exon	0,18
TC1100003736.mm.1	-2,16	Krtap4-8	PSR1100034339.mm.1	2,95	0,001786	0,336311	Cassette Exon	0,18
TC1100002921.mm.1	-1,25	Ulk2	JUC1100013889.mm.1	2,95	0,032877	0,521147		
TC1100002921.mm.1	-1,25	Ulk2	PSR1100026616.mm.1	2,07	0,005662	0,381922	Cassette Exon	0,13
TC1100002921.mm.1	-1,25	Ulk2	JUC1100013883.mm.1	2,07	0,009197	0,413763		
TC0100000053.mm.1	-1,02		JUC0100000258.mm.1	2,95	0,02498	0,496025		
TC1500002147.mm.1	-1,38	Slc38a4	JUC1500009810.mm.1	2,95	0,010326	0,420743		
TC1600001259.mm.1	1,17	Dnm1l	JUC1600005297.mm.1	2,95	0,00635	0,389463		
TC0500002846.mm.1	1,15	Fam175a	JUC0500013659.mm.1	2,95	0,024503	0,493992		
TC0700000147.mm.1	1,44	Zfp110	JUC0700000532.mm.1	2,95	0,015786	0,455641		
TC0600003020.mm.1	-1,15	Erc1	JUC0600012153.mm.1	2,95	0,008267	0,405976		
TC0700002472.mm.1	-1,18	Gipr	JUC0700011593.mm.1	2,95	0,000593	0,304044		
TC0300001314.mm.1	-1,02	Pla2g12a	JUC0300005544.mm.1	2,95	0,035956	0,529431		
TC0300001508.mm.1	-1,51		JUC0300006441.mm.1	2,95	0,005588	0,381035		
TC0Y00000283.mm.1	-1,28	Gm20736	JUC0Y00001178.mm.1	2,95	0,016073	0,457489		
TC1700002802.mm.1	2,59	Rhot2	PSR1700015405.mm.1	2,94	0,015744	0,455416	Cassette Exon	0,19
TC1700002802.mm.1	2,59	Rhot2	PSR1700015406.mm.1	2,42	0,024337	0,49362	Cassette Exon	0,14
TC1700002802.mm.1	2,59	Rhot2	JUC1700008354.mm.1	-2,65	0,007557	0,401384		
TC1700002802.mm.1	2,59	Rhot2	PSR1700015370.mm.1	-2,99	0,037783	0,534693	Intron Retention	0,33
TC1700002802.mm.1	2,59	Rhot2	PSR1700015382.mm.1	-3,68	0,007872	0,403545	Alternative 3' Acce	0,42
TC1700002802.mm.1	2,59	Rhot2	JUC1700008373.mm.1	-4,34	0,00596	0,385397		
TC1100001157.mm.1	-1,08	Pigs	PSR1100010772.mm.1	2,94	0,019695	0,474887	Alternative 3' Acce	0,34
TC1100001157.mm.1	-1,08	Pigs	PSR1100010777.mm.1	2,16	0,005397	0,378916	Intron Retention	0,25

TC1100001157.mm.1	-1,08	Pigs	PSR1100010768.mm.1	2,13	0,003558	0,356223	Intron Retention	0,28
TC0400001695.mm.1	3,63	Lactbl1	JUC0400007040.mm.1	2,94	0,021825	0,484607		
TC0400001695.mm.1	3,63	Lactbl1	PSR0400013625.mm.1	2,22	0,008435	0,406611	Cassette Exon	0,21
TC0400001695.mm.1	3,63	Lactbl1	JUC0400007038.mm.1	2,02	0,003625	0,356444		
TC0400001695.mm.1	3,63	Lactbl1	PSR0400013628.mm.1	-2,08	0,027001	0,502911	Cassette Exon	0,12
TC0400001695.mm.1	3,63	Lactbl1	PSR0400013627.mm.1	-2,92	0,000909	0,313363	Cassette Exon	0,31
TC0400001695.mm.1	3,63	Lactbl1	JUC0400007037.mm.1	-3,05	0,000109	0,267977		
TC0400001695.mm.1	3,63	Lactbl1	JUC0400007041.mm.1	-3,49	0,002405	0,348653		
TC0500002676.mm.1	-1,67	Uba6	PSR0500023584.mm.1	2,94	0,048098	0,559875	Alternative 5' Donc	0,28
TC0500001221.mm.1	-1,24	Acacb	JUC0500005692.mm.1	2,94	0,030891	0,514574		
TC0500001221.mm.1	-1,24	Acacb	PSR0500010371.mm.1	-2,11	0,047066	0,557783	Cassette Exon	0,12
TC0200003654.mm.1	-1,01	Grb14	JUC0200015800.mm.1	2,94	0,014994	0,452052		
TC1900001619.mm.1	1,1	Pcgf6	JUC1900007957.mm.1	2,94	0,048251	0,560279		
TC0200002270.mm.1	-1,03	Angpt4	JUC0200008922.mm.1	2,94	0,008267	0,405976		
TC1600001556.mm.1	-1,38	Parp14	JUC1600006655.mm.1	2,94	0,02419	0,493294		
TC0800001861.mm.1	-1	Nek5	JUC0800007994.mm.1	2,94	0,039879	0,54093		
TC0900000351.mm.1	1,39	Fez1	JUC0900001406.mm.1	2,94	0,049735	0,563552		
TC1100000935.mm.1	1,16	Phf23	JUC1100004224.mm.1	2,94	0,033915	0,52424		
TC1100000935.mm.1	1,16	Phf23	JUC1100004228.mm.1	-5,13	0,049539	0,563325		
TC1000001597.mm.1	1,3	Pan2	JUC1000006298.mm.1	2,94	0,00168	0,335182		
TC1100001579.mm.1	-1,14	Csf3	JUC1100007631.mm.1	2,94	0,005893	0,383835		
TC0900002095.mm.1	2,94	Gramd1b	JUC0900009721.mm.1	2,93	0,011385	0,427549		
TC0900002095.mm.1	2,94	Gramd1b	PSR0900017325.mm.1	-2,13	0,030672	0,51384	Alternative 5' Donc	0,18
TC0900002095.mm.1	2,94	Gramd1b	PSR0900017322.mm.1	-2,23	0,041353	0,54446	Cassette Exon	0,01
TC0900002095.mm.1	2,94	Gramd1b	JUC0900009737.mm.1	-3,24	0,006016	0,386586		
TC0900002095.mm.1	2,94	Gramd1b	JUC0900009733.mm.1	-3,31	0,009641	0,417354		
TC0900002095.mm.1	2,94	Gramd1b	PSR0900017327.mm.1	-4,19	0,009782	0,417839	Cassette Exon	0,3
TC0900002095.mm.1	2,94	Gramd1b	PSR0900017329.mm.1	-4,46	0,033697	0,523549	Cassette Exon	0,44
TC0900002095.mm.1	2,94	Gramd1b	JUC0900009743.mm.1	-4,89	0,004697	0,370559		
TC0900002095.mm.1	2,94	Gramd1b	JUC0900009744.mm.1	-5,46	0,001868	0,337974		
TC0900002095.mm.1	2,94	Gramd1b	JUC0900009735.mm.1	-6,77	0,000125	0,270905		
TC0500001898.mm.1	2,68	Cdk14	JUC0500009511.mm.1	2,93	0,002281	0,34605		
TC0500001898.mm.1	2,68	Cdk14	PSR0500017489.mm.1	2,4	0,000153	0,272178	Alternative 3' Acce	0,17
TC0500001898.mm.1	2,68	Cdk14	PSR0500017502.mm.1	-2,54	0,041484	0,544745	Cassette Exon	0,18
TC0500001898.mm.1	2,68	Cdk14	PSR0500017473.mm.1	-2,66	0,013082	0,4396	Alternative 3' Acce	0,25
TC0500001898.mm.1	2,68	Cdk14	JUC0500009518.mm.1	-2,91	0,048087	0,55985		
TC0500001898.mm.1	2,68	Cdk14	PSR0500017499.mm.1	-2,92	0,012037	0,432286	Cassette Exon	0,2
TC0500001898.mm.1	2,68	Cdk14	JUC0500009512.mm.1	-4,58	0,012043	0,432309		
TC0900000180.mm.1	2,95	Rdh8	JUC0900000547.mm.1	2,93	0,0029	0,352207		
TC0900000180.mm.1	2,95	Rdh8	PSR0900001124.mm.1	2,72	0,003749	0,357586	Cassette Exon	0,19
TC0900000180.mm.1	2,95	Rdh8	JUC0900000550.mm.1	2,24	0,006432	0,389972		
TC0900000180.mm.1	2,95	Rdh8	PSR0900001123.mm.1	2,12	0,011127	0,426023	Cassette Exon	0,23
TC0200004368.mm.1	3,39	Gjd2	PSR0200036492.mm.1	2,93	0,008113	0,405267	Cassette Exon	0,15
TC0200004368.mm.1	3,39	Gjd2	PSR0200036493.mm.1	-2,93	0,008113	0,405267	Cassette Exon	0,21
TC0Y00000203.mm.1	-1,26	Gm20916; Gm21627; Gm2	JUC0Y00000771.mm.1	2,93	0,027936	0,505612		
TC0Y00000203.mm.1	-1,26	Gm20916; Gm21627; Gm2	PSR0Y00001065.mm.1	-3,14	0,027498	0,504313	Cassette Exon	0,19
TC1200002438.mm.1	-1,41	Ahnak2	PSR1200016938.mm.1	2,93	0,0458	0,554863	Cassette Exon	0,18
TC1200002438.mm.1	-1,41	Ahnak2	PSR1200016924.mm.1	2,22	0,001986	0,341091	Cassette Exon	0,11

TC1200002438.mm.1	-1,41	Ahnak2	PSR1200016928.mm.1	2,09	0,018121	0,467204		
TC0800003233.mm.1	1,21	Trmt1	PSR0800007200.mm.1	2,93	0,003635	0,3566	Intron Retention	0,16
TC0800003233.mm.1	1,21	Trmt1	JUC0800003836.mm.1	2,36	0,02195	0,485095		
TC1000002645.mm.1	-1,47	Chpt1	JUC1000010951.mm.1	2,93	0,007261	0,39826		
TC1000002645.mm.1	-1,47	Chpt1	JUC1000010949.mm.1	2,36	0,026064	0,499627		
TC1000002645.mm.1	-1,47	Chpt1	PSR1000020010.mm.1	2,35	0,042273	0,547092	Cassette Exon	0,1
TC0200002834.mm.1	1,03	Oprl1	JUC0200011882.mm.1	2,93	0,003918	0,359788		
TC0300000411.mm.1	1,09	Supt20	JUC0300001461.mm.1	2,93	0,00089	0,313363		
TC0300000411.mm.1	1,09	Supt20	JUC0300001492.mm.1	-2,7	0,029273	0,509873		
TC1100003831.mm.1	1,15	Ubtf	JUC1100018631.mm.1	2,93	0,012975	0,439245		
TC0500001597.mm.1	1,32	Rasa4; Mir7035; mmu-mir	JUC0500007870.mm.1	2,93	0,037721	0,534622		
TC0500001597.mm.1	1,32	Rasa4; Mir7035; mmu-mir	PSR0500014445.mm.1	2,04	0,006402	0,38966		
TC0500001597.mm.1	1,32	Rasa4; Mir7035; mmu-mir	JUC0500007892.mm.1	-2,66	0,018158	0,467432		
TC0700000990.mm.1	1,05	Vimp	JUC0700004669.mm.1	2,93	0,046227	0,556056		
TC0400001180.mm.1	1,16	Agbl4	JUC0400004428.mm.1	2,93	0,01346	0,442805		
TC0400001116.mm.1	-1,02	Ssbp3	JUC0400003974.mm.1	2,93	0,024485	0,493992		
TC0300001427.mm.1	1,02	Stpg2	JUC0300006051.mm.1	2,93	0,007531	0,40125		
TC1100002255.mm.1	1,02	Etaa1	JUC1100011391.mm.1	2,93	0,016768	0,460504		
TC0400001453.mm.1	1,1	Stk40	JUC0400005770.mm.1	2,93	0,027931	0,505612		
TC1800001088.mm.1	3,54	Celf4	PSR1800008017.mm.1	2,92	0,001061	0,316361		
TC1800001088.mm.1	3,54	Celf4	JUC1800004464.mm.1	2,73	0,005485	0,379939		
TC1800001088.mm.1	3,54	Celf4	PSR1800008006.mm.1	2,32	0,018427	0,468598	Cassette Exon	0,11
TC1800001088.mm.1	3,54	Celf4	JUC1800004454.mm.1	2,24	0,000649	0,304044		
TC1800001088.mm.1	3,54	Celf4	JUC1800004468.mm.1	-3,68	0,044656	0,552129		
TC1800001088.mm.1	3,54	Celf4	PSR1800008022.mm.1	-3,69	0,021552	0,483034	Cassette Exon	0,17
TC1800001088.mm.1	3,54	Celf4	JUC1800004456.mm.1	-3,79	0,010009	0,418602		
TC1800001088.mm.1	3,54	Celf4	JUC1800004470.mm.1	-3,84	0,0181	0,467161		
TC1800001088.mm.1	3,54	Celf4	PSR1800008012.mm.1	-4,06	0,008867	0,410539	Cassette Exon	0,55
TC1800001088.mm.1	3,54	Celf4	JUC1800004457.mm.1	-4,5	0,001304	0,323504		
TC1800001088.mm.1	3,54	Celf4	JUC1800004462.mm.1	-4,67	0,009635	0,417354		
TC1800001088.mm.1	3,54	Celf4	JUC1800004460.mm.1	-5,06	0,034962	0,52724		
TC1800001088.mm.1	3,54	Celf4	JUC1800004466.mm.1	-5,29	0,00767	0,402377		
TC1800001088.mm.1	3,54	Celf4	PSR1800008020.mm.1	-7,11	0,00295	0,352501	Alternative 3' Acce	0,41
TC1800001088.mm.1	3,54	Celf4	JUC1800004467.mm.1	-8,19	0,003474	0,355628		
TC1800001088.mm.1	3,54	Celf4	JUC1800004465.mm.1	-9,46	0,01232	0,434392		
TC0200003300.mm.1	6,61	Dnm1	JUC0200014145.mm.1	2,92	0,003895	0,359372		
TC0200003300.mm.1	6,61	Dnm1	PSR0200028056.mm.1	2,43	0,00953	0,416602	Cassette Exon	0,16
TC0200003300.mm.1	6,61	Dnm1	JUC0200014135.mm.1	2,3	0,016501	0,459428		
TC0200003300.mm.1	6,61	Dnm1	JUC0200014146.mm.1	2,01	0,002395	0,348564		
TC0200003300.mm.1	6,61	Dnm1	PSR0200028049.mm.1	-2,12	0,002843	0,352207	Cassette Exon	0,26
TC0200003300.mm.1	6,61	Dnm1	PSR0200028023.mm.1	-2,18	0,034065	0,524583	Alternative 5' Donc	0,18
TC0200003300.mm.1	6,61	Dnm1	PSR0200028034.mm.1	-2,45	0,004564	0,369273	Cassette Exon	0,27
TC0200003300.mm.1	6,61	Dnm1	JUC0200014147.mm.1	-2,64	0,016468	0,459288		
TC0200003300.mm.1	6,61	Dnm1	PSR0200028021.mm.1	-2,65	0,031726	0,517489	Alternative 5' Donc	0,25
TC0200003300.mm.1	6,61	Dnm1	PSR0200028024.mm.1	-2,73	0,006341	0,389463	Alternative 5' Donc	0,18
TC0200003300.mm.1	6,61	Dnm1	PSR0200028047.mm.1	-2,87	0,013305	0,441836	Cassette Exon	0,17
TC0200003300.mm.1	6,61	Dnm1	PSR0200028025.mm.1	-3,21	0,002583	0,349501	Cassette Exon	0,2
TC0200003300.mm.1	6,61	Dnm1	JUC0200014133.mm.1	-3,63	0,009507	0,416602		

TC0200003300.mm.1	6,61	Dnm1	PSR0200028060.mm.1	-4,62	0,020509	0,47825	Cassette Exon	0,41
TC0200003300.mm.1	6,61	Dnm1	PSR0200028036.mm.1	-5,28	0,000454	0,298307	Alternative 5' Donc	0,47
TC0200003300.mm.1	6,61	Dnm1	JUC0200014154.mm.1	-6	0,001844	0,337414		
TC0200003300.mm.1	6,61	Dnm1	PSR0200028022.mm.1	-6,48	0,002075	0,342181	Cassette Exon	0,28
TC0200003300.mm.1	6,61	Dnm1	PSR0200028027.mm.1	-6,89	0,019861	0,475704	Cassette Exon	0,28
TC0200003300.mm.1	6,61	Dnm1	PSR0200028028.mm.1	-7,04	0,003095	0,353892		
TC0200003300.mm.1	6,61	Dnm1	JUC0200014150.mm.1	-7,62	0,009622	0,417315		
TC0200003300.mm.1	6,61	Dnm1	PSR0200028059.mm.1	-7,78	0,002633	0,349612	Cassette Exon	0,21
TC0200003300.mm.1	6,61	Dnm1	PSR0200028068.mm.1	-8,1	0,005119	0,37627	Cassette Exon	0,41
TC0200003300.mm.1	6,61	Dnm1	JUC0200014155.mm.1	-8,22	0,003413	0,355249		
TC0200003300.mm.1	6,61	Dnm1	PSR0200028041.mm.1	-12,3	0,000806	0,311765	Cassette Exon	0,4
TC0200003300.mm.1	6,61	Dnm1	JUC0200014160.mm.1	-14,31	0,005264	0,377955		
TC0200003300.mm.1	6,61	Dnm1	JUC0200014158.mm.1	-21,43	0,000015	0,18968		
TC1400001703.mm.1	1,45	Chat	JUC1400007196.mm.1	2,92	0,025832	0,498598		
TC1400001703.mm.1	1,45	Chat	PSR1400013218.mm.1	-3,67	0,010464	0,421696	Alternative 5' Donc	0,41
TC0X00002450.mm.1	-2,15	Lage3	PSR0X00015576.mm.1	2,92	0,008307	0,406196	Alternative 3' Acce	0,23
TC0200000651.mm.1	-1,52	Ptgs1	PSR0200005995.mm.1	2,92	0,002346	0,34761	Cassette Exon	0,22
TC0200000651.mm.1	-1,52	Ptgs1	JUC0200003065.mm.1	2,06	0,01425	0,447947		
TC0600000293.mm.1	-2,28	Gm13844	PSR0600002161.mm.1	2,92	0,012245	0,433659	Cassette Exon	0,22
TC0300000990.mm.1	-1,99	Tbx15	PSR0300008005.mm.1	2,92	0,002282	0,34605	Alternative 3' Acce	0,21
TC1700001834.mm.1	-1,38	Notch3	PSR1700016886.mm.1	2,92	0,048509	0,560989	Cassette Exon	0,21
TC0400000036.mm.1	1,05	Ubxn2b	PSR0400000244.mm.1	2,92	0,030655	0,513785	Cassette Exon	0,19
TC0300002567.mm.1	-1,59	5730437C11Rik	PSR0300020062.mm.1	2,92	0,043597	0,550133	Cassette Exon	0,18
TC0800000936.mm.1	1,32	Lphn1	JUC0800003669.mm.1	2,92	0,001656	0,334378		
TC0800000936.mm.1	1,32	Lphn1	PSR0800006948.mm.1	-2,12	0,040889	0,543423	Alternative 5' Donc	0,18
TC0800000936.mm.1	1,32	Lphn1	JUC0800003692.mm.1	-2,26	0,002205	0,345178		
TC0700003474.mm.1	-2,02	LOC624549	JUC0700015583.mm.1	2,92	0,038279	0,536002		
TC0700003474.mm.1	-2,02	LOC624549	PSR0700029519.mm.1	2,49	0,022307	0,486127	Alternative 5' Donc	0,13
TC0200002496.mm.1	-1,41	Wisp2	JUC0200010364.mm.1	2,92	0,001314	0,324276		
TC0200004457.mm.1	-1,08	Pla2g4f	JUC0200019206.mm.1	2,92	0,006706	0,393343		
TC0200004457.mm.1	-1,08	Pla2g4f	JUC0200019210.mm.1	-2,27	0,017549	0,464165		
TC0500000387.mm.1	-1,39	Rgs12	JUC0500002129.mm.1	2,92	0,015967	0,456885		
TC0900001410.mm.1	1,11	Qars	JUC0900006265.mm.1	2,92	0,036585	0,531424		
TC0700004189.mm.1	-1,06	Eri2	JUC0700018354.mm.1	2,92	0,008368	0,406435		
TC1900000546.mm.1	3,26	Pde6c	JUC1900002583.mm.1	2,91	0,00595	0,385276		
TC1900000546.mm.1	3,26	Pde6c	JUC1900002593.mm.1	2,53	0,004863	0,371692		
TC1900000546.mm.1	3,26	Pde6c	PSR1900004884.mm.1	2,22	0,002007	0,341091	Cassette Exon	0,14
TC1900000546.mm.1	3,26	Pde6c	PSR1900004892.mm.1	-2,98	0,003964	0,359943	Cassette Exon	0,31
TC1900000546.mm.1	3,26	Pde6c	PSR1900004872.mm.1	-3,15	0,001256	0,322251	Cassette Exon	0,11
TC1900000546.mm.1	3,26	Pde6c	JUC1900002603.mm.1	-5,05	0,004449	0,367227		
TC1600001528.mm.1	-1,16	Kalrn	PSR1600012530.mm.1	2,91	0,029789	0,511132	Cassette Exon	0,21
TC1600001528.mm.1	-1,16	Kalrn	PSR1600012532.mm.1	2,25	0,035349	0,528451	Cassette Exon	0,12
TC1600001528.mm.1	-1,16	Kalrn	JUC1600006544.mm.1	2,18	0,002611	0,349501		
TC1600001528.mm.1	-1,16	Kalrn	JUC1600006586.mm.1	-2,45	0,040716	0,542933		
TC0100000338.mm.1	-1,44	Gulp1	JUC0100001431.mm.1	2,91	0,016117	0,457756		
TC0100000338.mm.1	-1,44	Gulp1	JUC0100001427.mm.1	2,51	0,014676	0,450139		
TC0100000338.mm.1	-1,44	Gulp1	PSR0100002529.mm.1	2,3	0,001095	0,317328	Alternative 3' Acce	0,15
TC1100003778.mm.1	-1,2	Zfp385c	JUC1100018188.mm.1	2,91	0,041899	0,545772		

TC1100003778.mm.1	-1,2	Zfp385c	JUC1100018190.mm.1	2,59	0,005005	0,373851		
TC1100003778.mm.1	-1,2	Zfp385c	JUC1100018191.mm.1	-2,03	0,029629	0,510558		
TC1200002158.mm.1	1,11	Sel1l	JUC1200008269.mm.1	2,91	0,029296	0,509874		
TC1200002158.mm.1	1,11	Sel1l	JUC1200008282.mm.1	-3,3	0,036694	0,531756		
TC1500001776.mm.1	-1,17	Bop1	JUC1500007768.mm.1	2,91	0,022031	0,485268		
TC1500001776.mm.1	-1,17	Bop1	JUC1500007769.mm.1	2,27	0,000771	0,308743		
TC0500001594.mm.1	-1,23	Dtx2	JUC0500007835.mm.1	2,91	0,043695	0,550256		
TC0600000226.mm.1	1,14	Hilpda	JUC0600000714.mm.1	2,91	0,036327	0,530547		
TC0400003073.mm.1	-1,06	Plaa	JUC0400012772.mm.1	2,91	0,013747	0,444758		
TC0400003124.mm.1	-1,13	Dock7	JUC0400012973.mm.1	2,91	0,024545	0,494083		
TC0500003092.mm.1	2,6	Cabp1	PSR0500027504.mm.1	2,9	0,002606	0,349501	Cassette Exon	0,04
TC0500003092.mm.1	2,6	Cabp1	JUC0500015009.mm.1	2,73	0,037337	0,533573		
TC0500003092.mm.1	2,6	Cabp1	JUC0500015013.mm.1	2,29	0,030764	0,514109		
TC0500003092.mm.1	2,6	Cabp1	JUC0500015007.mm.1	2,26	0,002754	0,350151		
TC0500003092.mm.1	2,6	Cabp1	PSR0500027498.mm.1	-2,1	0,006303	0,389124	Cassette Exon	0,03
TC0500003092.mm.1	2,6	Cabp1	JUC0500015016.mm.1	-2,12	0,029574	0,510456		
TC0500003092.mm.1	2,6	Cabp1	PSR0500027508.mm.1	-2,18	0,009452	0,416438	Cassette Exon	0,22
TC0500003092.mm.1	2,6	Cabp1	PSR0500027509.mm.1	-2,19	0,018292	0,468049	Cassette Exon	0,22
TC0500003092.mm.1	2,6	Cabp1	PSR0500027505.mm.1	-2,27	0,000593	0,304044	Cassette Exon	0,22
TC0500003092.mm.1	2,6	Cabp1	PSR0500027513.mm.1	-2,55	0,002279	0,345991	Alternative 5' Donc	0,25
TC0500003092.mm.1	2,6	Cabp1	PSR0500027515.mm.1	-2,84	0,006576	0,39213	Cassette Exon	0,11
TC0500003092.mm.1	2,6	Cabp1	JUC0500015010.mm.1	-5,62	0,047273	0,558224		
TC1600000911.mm.1	-1,14	1700066C05Rik	PSR1600007255.mm.1	2,9	0,005322	0,378059	Cassette Exon	0,18
TC0500003446.mm.1	-1,45	Zan	JUC0500017024.mm.1	2,9	0,005472	0,379939		
TC0500003446.mm.1	-1,45	Zan	PSR0500031301.mm.1	2,47	0,034097	0,524826	Cassette Exon	0,16
TC0500003446.mm.1	-1,45	Zan	JUC0500017045.mm.1	2,05	0,004853	0,37168		
TC0500003446.mm.1	-1,45	Zan	PSR0500031323.mm.1	2,03	0,036293	0,530447	Cassette Exon	0,09
TC1100004236.mm.1	1,12	Csnk1d	JUC1100021310.mm.1	2,9	0,005264	0,377955		
TC1500002327.mm.1	-1,2	Prlr	JUC1500000442.mm.1	2,9	0,045454	0,554199		
TC0600003120.mm.1	1,31	Usp5	JUC0600012628.mm.1	2,9	0,002893	0,352207		
TC0700001896.mm.1	1,11	Ppapdc1a	JUC0700008698.mm.1	2,9	0,025971	0,499207		
TC1600001642.mm.1	1,13	Sidt1	JUC1600006991.mm.1	2,89	0,003737	0,357586		
TC1600001642.mm.1	1,13	Sidt1	PSR1600013405.mm.1	-3,61	0,024125	0,493245	Alternative 5' Donc	0,41
TC0100000659.mm.1	-1,54	Gmppa	PSR0100005635.mm.1	2,89	0,025348	0,497375	Alternative 5' Donc	0,3
TC0700000980.mm.1	1,2	Apba2	PSR0700008978.mm.1	2,89	0,007438	0,399934	Alternative 5' Donc	0,3
TC0700000980.mm.1	1,2	Apba2	PSR0700008967.mm.1	2,3	0,032335	0,519155	Cassette Exon	0,15
TC0700000980.mm.1	1,2	Apba2	PSR0700008961.mm.1	2,08	0,004994	0,373851	Cassette Exon	0,11
TC0700000980.mm.1	1,2	Apba2	JUC0700004583.mm.1	2,05	0,033995	0,524346		
TC0400000460.mm.1	-1,08	Clta	PSR0400003240.mm.1	2,89	0,01535	0,453763	Cassette Exon	0,26
TC1000003113.mm.1	-1,63	Gli1	JUC1000012798.mm.1	2,89	0,02109	0,480371		
TC1000003113.mm.1	-1,63	Gli1	PSR1000023280.mm.1	2,16	0,035447	0,52871	Cassette Exon	0,2
TC0X00000069.mm.1	1,13	Gripap1	PSR0X00000475.mm.1	2,89	0,022512	0,487114	Cassette Exon	0,18
TC1500000594.mm.1	2,3		PSR1500004267.mm.1	2,89	0,00922	0,41411	Cassette Exon	0,17
TC1500000594.mm.1	2,3		PSR1500004265.mm.1	-2,6	0,00112	0,317328	Cassette Exon	0,15
TC0Y00000217.mm.1	-1,23	Gm21294; Gm20736	JUC0Y00000891.mm.1	2,89	0,023266	0,49004		
TC0Y00000217.mm.1	-1,23	Gm21294; Gm20736	PSR0Y00001213.mm.1	-2,32	0,03657	0,531355	Cassette Exon	0,13
TC1300000072.mm.1	1,32	Ero1lb	JUC1300000311.mm.1	2,89	0,043063	0,54841		
TC0800001620.mm.1	-1,4	Rprl3	PSR0800013329.mm.1	2,89	0,036532	0,531352		

TC1500001177.mm.1	-1,2		JUC1500005321.mm.1	2,89	0,012671	0,43631		
TC1500000034.mm.1	-1,01	C9	JUC1500000174.mm.1	2,89	0,013299	0,441759		
TC0800000981.mm.1	1,16	Dhps	JUC0800003986.mm.1	2,89	0,012676	0,43631		
TC1000003156.mm.1	1,02	Ikzf4	JUC1000013083.mm.1	2,89	0,011655	0,429904		
TC1000003156.mm.1	1,02	Ikzf4	JUC1000013095.mm.1	-3,5	0,028333	0,506831		
TC1000000301.mm.1	-1,95	Frk	JUC1000001090.mm.1	2,89	0,001872	0,338238		
TC0X00003018.mm.1	1,15	Bex2	JUC0X00009580.mm.1	2,89	0,000608	0,304044		
TC1100001162.mm.1	6,14	Vtn	JUC1100005678.mm.1	2,88	0,014236	0,447947		
TC1100001162.mm.1	6,14	Vtn	JUC1100005676.mm.1	2,4	0,023564	0,490947		
TC1100001162.mm.1	6,14	Vtn	PSR1100010837.mm.1	-2,41	0,037194	0,533261	Alternative 3' Acce	0,22
TC1100001162.mm.1	6,14	Vtn	PSR1100010833.mm.1	-3,41	0,002596	0,349501	Intron Retention	0,6
TC1100001162.mm.1	6,14	Vtn	PSR1100010826.mm.1	-3,49	0,000634	0,304044	Alternative 3' Acce	0,44
TC1100001162.mm.1	6,14	Vtn	PSR1100010827.mm.1	-5,54	0,000063	0,250119	Alternative 3' Acce	0,44
TC1100001162.mm.1	6,14	Vtn	PSR1100010830.mm.1	-7,18	0,00002	0,211693	Intron Retention	0,73
TC1400000947.mm.1	-1,17	Adra1a	PSR1400007628.mm.1	2,88	0,023446	0,490562	Alternative 3' Acce	0,21
TC0X00001514.mm.1	1,55	Wnk3	JUC0X00004716.mm.1	2,88	0,048123	0,559938		
TC0X00001514.mm.1	1,55	Wnk3	JUC0X00004720.mm.1	-2,56	0,032054	0,518469		
TC0X00001514.mm.1	1,55	Wnk3	PSR0X00009460.mm.1	-3,15	0,003933	0,359818	Alternative 5' Donc	0,17
TC1700001942.mm.1	-1,43	G6b; AU023871	JUC1700009874.mm.1	2,88	0,009318	0,414883		
TC1500000666.mm.1	-1,3	Micall1	JUC1500002841.mm.1	2,88	0,008205	0,405909		
TC1500000666.mm.1	-1,3	Micall1	JUC1500002848.mm.1	2,22	0,021077	0,480371		
TC0800001899.mm.1	-1,03	Adam5	JUC0800008216.mm.1	2,88	0,004092	0,36228		
TC0600002592.mm.1	-1,11	Cyp26b1	JUC0600010443.mm.1	2,88	0,010352	0,421002		
TC0Y00000205.mm.1	-1,28	Gm20911	JUC0Y00000788.mm.1	2,88	0,029037	0,508904		
TC0600001180.mm.1	3,62	Cntn4	PSR0600009130.mm.1	2,87	0,024509	0,493992	Cassette Exon	0,19
TC0600001180.mm.1	3,62	Cntn4	JUC0600004680.mm.1	2,62	0,002395	0,348564		
TC0600001180.mm.1	3,62	Cntn4	JUC0600004683.mm.1	-2,04	0,000472	0,298999		
TC0600001180.mm.1	3,62	Cntn4	PSR0600009133.mm.1	-2,08	0,003457	0,355628	Cassette Exon	0,29
TC0600001180.mm.1	3,62	Cntn4	JUC0600004672.mm.1	-2,09	0,01876	0,470733		
TC0600001180.mm.1	3,62	Cntn4	PSR0600009100.mm.1	-2,1	0,002043	0,341377	Alternative 3' Acce	0,35
TC0600001180.mm.1	3,62	Cntn4	PSR0600009095.mm.1	-2,23	0,025521	0,497708	Cassette Exon	0,12
TC0600001180.mm.1	3,62	Cntn4	JUC0600004699.mm.1	-2,87	0,000188	0,285336		
TC0600001180.mm.1	3,62	Cntn4	PSR0600009103.mm.1	-3,04	0,037023	0,532502	Cassette Exon	0,19
TC0600001180.mm.1	3,62	Cntn4	JUC0600004673.mm.1	-3,17	0,032521	0,519901		
TC0600001180.mm.1	3,62	Cntn4	JUC0600004668.mm.1	-3,28	0,001754	0,335996		
TC0600001180.mm.1	3,62	Cntn4	PSR0600009134.mm.1	-3,3	0,009189	0,413763	Cassette Exon	0,29
TC0600001180.mm.1	3,62	Cntn4	PSR0600009093.mm.1	-3,35	0,016823	0,460623	Cassette Exon	0,2
TC0600001180.mm.1	3,62	Cntn4	PSR0600009097.mm.1	-3,6	0,020039	0,476453	Cassette Exon	0,38
TC0600001180.mm.1	3,62	Cntn4	PSR0600009089.mm.1	-3,67	0,006068	0,386691	Cassette Exon	0,36
TC0600001180.mm.1	3,62	Cntn4	PSR0600009096.mm.1	-3,75	0,038242	0,535883	Cassette Exon	0,26
TC0600001180.mm.1	3,62	Cntn4	PSR0600009128.mm.1	-3,84	0,001574	0,331411	Cassette Exon	0,32
TC0600001180.mm.1	3,62	Cntn4	PSR0600009124.mm.1	-4,02	0,000101	0,262646	Alternative 5' Donc	0,46
TC0600001180.mm.1	3,62	Cntn4	PSR0600009135.mm.1	-4,04	0,005013	0,373867	Alternative 3' Acce	0,48
TC0600001180.mm.1	3,62	Cntn4	PSR0600009091.mm.1	-4,37	0,026827	0,502373	Cassette Exon	0,36
TC0600001180.mm.1	3,62	Cntn4	PSR0600009115.mm.1	-4,56	0,019972	0,476227	Alternative 5' Donc	0,55
TC0600001180.mm.1	3,62	Cntn4	PSR0600009126.mm.1	-4,91	0,007889	0,403787	Cassette Exon	0,28
TC0600001180.mm.1	3,62	Cntn4	JUC0600004676.mm.1	-5,03	0,001065	0,316361		
TC0600001180.mm.1	3,62	Cntn4	JUC0600004691.mm.1	-5,42	0,023439	0,490451		

TC0600001180.mm.1	3,62	Cntn4	PSR0600009106.mm.1	-5,53	0,00756	0,401384	Alternative 5' Donc	0,34
TC0600001180.mm.1	3,62	Cntn4	PSR0600009099.mm.1	-5,68	0,004066	0,361956	Alternative 3' Acce	0,35
TC0600001180.mm.1	3,62	Cntn4	PSR0600009098.mm.1	-5,85	0,009764	0,417839	Alternative 3' Acce	0,35
TC0600001180.mm.1	3,62	Cntn4	PSR0600009118.mm.1	-6,61	0,004029	0,361636	Alternative 5' Donc	0,49
TC0600001180.mm.1	3,62	Cntn4	JUC0600004666.mm.1	-7,24	0,000194	0,28803		
TC0600001180.mm.1	3,62	Cntn4	PSR0600009125.mm.1	-7,55	0,001319	0,324348	Cassette Exon	0,3
TC0600001180.mm.1	3,62	Cntn4	JUC0600004670.mm.1	-8,2	0,00994	0,41841		
TC0800000353.mm.1	-1,94	Eif4ebp1	JUC0800001473.mm.1	2,87	0,00266	0,349612		
TC0800000353.mm.1	-1,94	Eif4ebp1	PSR0800002713.mm.1	2,53	0,007958	0,403853	Cassette Exon	0,23
TC0800000353.mm.1	-1,94	Eif4ebp1	PSR0800002712.mm.1	2,48	0,005081	0,375342	Cassette Exon	0,23
TC1600001334.mm.1	1,55	Lamp3	PSR1600010994.mm.1	2,87	0,045959	0,555364	Cassette Exon	0,21
TC0600002607.mm.1	1,4	Sfxn5	PSR0600020237.mm.1	2,87	0,003017	0,353447	Cassette Exon	0,17
TC0600002607.mm.1	1,4	Sfxn5	PSR0600020235.mm.1	2,37	0,011929	0,431666	Cassette Exon	0,13
TC0600002607.mm.1	1,4	Sfxn5	PSR0600020244.mm.1	2,03	0,018012	0,466939		
TC0600002607.mm.1	1,4	Sfxn5	JUC0600010498.mm.1	-3,6	0,011301	0,427126		
TC1300001512.mm.1	1,07	Pou6f2	PSR1300009581.mm.1	2,87	0,004661	0,370181	Alternative 3' Acce	0,17
TC0Y00000210.mm.1	-1,1	Gm21409	JUC0Y00000830.mm.1	2,87	0,020428	0,478018		
TC0Y00000210.mm.1	-1,1	Gm21409	PSR0Y00001140.mm.1	-2,49	0,033587	0,523246	Cassette Exon	0,14
TC0400001451.mm.1	1,45	Oscp1	JUC0400005749.mm.1	2,87	0,038889	0,537855		
TC0400001451.mm.1	1,45	Oscp1	PSR0400011037.mm.1	-2,25	0,043911	0,550662	Cassette Exon	0,12
TC1800000076.mm.1	1,1	Greb1l	JUC1800000259.mm.1	2,87	0,027039	0,502979		
TC1800000076.mm.1	1,1	Greb1l	JUC1800000278.mm.1	-2,18	0,005575	0,381035		
TC0500000602.mm.1	-1,47	Fam114a1	JUC0500002954.mm.1	2,87	0,019521	0,474538		
TC1100002841.mm.1	-1,18	Obscn	JUC1100013308.mm.1	2,87	0,043996	0,550779		
TC1100002841.mm.1	-1,18	Obscn	JUC1100013343.mm.1	-2,84	0,020573	0,478487		
TC1100001723.mm.1	1,2	Crhr1	JUC1100008413.mm.1	2,87	0,038655	0,537102		
TC0X00002994.mm.1	-1,33	Btk	JUC0X00009427.mm.1	2,87	0,000235	0,28803		
TC0X00002994.mm.1	-1,33	Btk	JUC0X00009430.mm.1	2,23	0,014531	0,449545		
TC0300000354.mm.1	-1,5	Ccrn4l	PSR0300002538.mm.1	2,86	0,013445	0,442693	Alternative 5' Donc	0,28
TC1000001901.mm.1	1,48	Akap7	JUC1000007479.mm.1	2,86	0,006299	0,389034		
TC1000001901.mm.1	1,48	Akap7	PSR1000013724.mm.1	-2,07	0,043832	0,550456	Cassette Exon	0,1
TC1000001901.mm.1	1,48	Akap7	PSR1000013709.mm.1	-2,1	0,008634	0,408463	Alternative 5' Donc	0,18
TC1000001901.mm.1	1,48	Akap7	JUC1000007473.mm.1	-2,1	0,012973	0,439245		
TC0800000782.mm.1	1,08	Myo9b	JUC0800002970.mm.1	2,86	0,000055	0,24627		
TC0800000782.mm.1	1,08	Myo9b	PSR0800005553.mm.1	-2,07	0,000347	0,290082	Cassette Exon	0,13
TC1300000566.mm.1	-1,76	Aspn	JUC1300001802.mm.1	2,86	0,0306	0,513461		
TC0100002707.mm.1	-1,31	C130026I21Rik; A530032[	JUC0100012467.mm.1	2,86	0,009822	0,417839		
TC0100002707.mm.1	-1,31	C130026I21Rik; A530032[	JUC0100012469.mm.1	-2,07	0,035604	0,528912		
TC0500003161.mm.1	-2,02	1700021F13Rik	JUC0500015248.mm.1	2,86	0,009316	0,414883		
TC0300002683.mm.1	-1,23	Chi3l7; Chil5	JUC0300010867.mm.1	2,86	0,036547	0,531355		
TC0400003285.mm.1	-1,29	Slc5a9	JUC0400013594.mm.1	2,86	0,010463	0,421696		
TC0900002604.mm.1	1,04	Aqp9	JUC0900012183.mm.1	2,86	0,040378	0,541959		
TC0600000103.mm.1	-1,49	Foxp2	PSR0600000683.mm.1	2,85	0,014534	0,449545	Alternative 3' Acce	0,29
TC0600000103.mm.1	-1,49	Foxp2	JUC0600000366.mm.1	2,03	0,04469	0,552181		
TC0400001193.mm.1	-1,37	Skint7	PSR0400008672.mm.1	2,85	0,01132	0,427133	Cassette Exon	0,18
TC0600000387.mm.1	-1,57	4930502C15Rik	JUC0600001496.mm.1	2,85	0,017255	0,46278		
TC0600000387.mm.1	-1,57	4930502C15Rik	PSR0600002966.mm.1	2,03	0,041471	0,54472	Cassette Exon	0,09
TC0200002485.mm.1	1,7	Gdap1l1	JUC0200010315.mm.1	2,85	0,003528	0,355858		

TC0200002485.mm.1	1,7	Gdap1l1	JUC0200010311.mm.1	2,41	0,007558	0,401384		
TC0200002485.mm.1	1,7	Gdap1l1	JUC0200010309.mm.1	-2,02	0,002764	0,350231		
TC0200002485.mm.1	1,7	Gdap1l1	JUC0200010314.mm.1	-2,55	0,006827	0,393893		
TC0200003649.mm.1	1,11	Gm13575	JUC0200015784.mm.1	2,85	0,010724	0,423745		
TC1700002618.mm.1	-1,33		JUC1700012841.mm.1	2,85	0,022427	0,48681		
TC1700002368.mm.1	-1,74	Efna5	JUC1700011909.mm.1	2,85	0,023366	0,490143		
TC1700001036.mm.1	-1,05	Cntnap5c	JUC1700005360.mm.1	2,85	0,031575	0,517177		
TC0900001344.mm.1	-1,2	Poc1a	JUC0900005764.mm.1	2,85	0,003786	0,357586		
TC0500001006.mm.1	-1,17	Ptpn13	JUC0500004663.mm.1	2,85	0,031501	0,516965		
TC0300002876.mm.1	1,1	Mettl14	JUC0300011873.mm.1	2,85	0,000449	0,297771		
TC0300002876.mm.1	1,1	Mettl14	JUC0300011874.mm.1	2,16	0,033549	0,523131		
TC1000002288.mm.1	1,42	Lrrtm3	JUC1000008993.mm.1	2,85	0,015228	0,452839		
TC1000002288.mm.1	1,42	Lrrtm3	PSR1000016407.mm.1	2,26	0,015463	0,454312		
TC0900003204.mm.1	-1,19	Rbms3	PSR0900027014.mm.1	2,85	0,047888	0,559408		
TC0400001535.mm.1	1,02	E330017L17Rik	JUC0400006188.mm.1	2,85	0,008806	0,409934		
TC0300000928.mm.1	3,19	Sv2a	JUC0300003857.mm.1	2,84	0,007411	0,399901		
TC0300000928.mm.1	3,19	Sv2a	JUC0300003848.mm.1	-2,41	0,043196	0,548962		
TC0300000928.mm.1	3,19	Sv2a	PSR0300007427.mm.1	-3,16	0,01379	0,444974	Alternative 3' Acce	0,25
TC0300000928.mm.1	3,19	Sv2a	PSR0300007426.mm.1	-3,18	0,003797	0,357968	Cassette Exon	0,33
TC0300000928.mm.1	3,19	Sv2a	PSR0300007424.mm.1	-3,67	0,013097	0,439759	Cassette Exon	0,31
TC1300001395.mm.1	3,72	Pfkp	PSR1300008736.mm.1	2,84	0,000921	0,313363	Alternative 3' Acce	0,12
TC1300001395.mm.1	3,72	Pfkp	PSR1300008750.mm.1	-2,02	0,006298	0,389033	Cassette Exon	0,06
TC1300001395.mm.1	3,72	Pfkp	JUC1300004541.mm.1	-2,06	0,035051	0,527561		
TC1300001395.mm.1	3,72	Pfkp	PSR1300008754.mm.1	-2,11	0,00173	0,335996	Cassette Exon	0,14
TC1300001395.mm.1	3,72	Pfkp	PSR1300008721.mm.1	-2,16	0,01574	0,455416	Cassette Exon	0,2
TC1300001395.mm.1	3,72	Pfkp	JUC1300004529.mm.1	-2,16	0,040417	0,541998		
TC1300001395.mm.1	3,72	Pfkp	JUC1300004550.mm.1	-2,36	0,011808	0,430357		
TC1300001395.mm.1	3,72	Pfkp	PSR1300008755.mm.1	-2,48	0,00354	0,3562	Alternative 3' Acce	0,17
TC1300001395.mm.1	3,72	Pfkp	JUC1300004531.mm.1	-2,55	0,002498	0,349142		
TC1300001395.mm.1	3,72	Pfkp	PSR1300008719.mm.1	-2,75	0,01462	0,449914	Cassette Exon	0,2
TC1300001395.mm.1	3,72	Pfkp	PSR1300008773.mm.1	-3,06	0,004479	0,367858	Cassette Exon	0,19
TC1300001395.mm.1	3,72	Pfkp	PSR1300008777.mm.1	-3,2	0,011945	0,431753	Alternative 5' Donc	0,33
TC1300001395.mm.1	3,72	Pfkp	PSR1300008766.mm.1	-3,24	0,000892	0,313363	Cassette Exon	0,24
TC1300001395.mm.1	3,72	Pfkp	PSR1300008727.mm.1	-3,31	0,00412	0,3623	Alternative 3' Acce	0,33
TC1300001395.mm.1	3,72	Pfkp	PSR1300008764.mm.1	-3,38	0,003778	0,357586	Alternative 3' Acce	0,29
TC1300001395.mm.1	3,72	Pfkp	PSR1300008718.mm.1	-3,6	0,017569	0,464258	Cassette Exon	0,2
TC1300001395.mm.1	3,72	Pfkp	PSR1300008726.mm.1	-3,71	0,016716	0,460202	Cassette Exon	0,28
TC1300001395.mm.1	3,72	Pfkp	JUC1300004551.mm.1	-4,7	0,015362	0,453813		
TC1300001395.mm.1	3,72	Pfkp	JUC1300004555.mm.1	-4,81	0,018598	0,469657		
TC1300001395.mm.1	3,72	Pfkp	JUC1300004552.mm.1	-4,94	0,00308	0,353892		
TC1700001600.mm.1	-1,16	1520401A03Rik	PSR1700013984.mm.1	2,84	0,009526	0,416602	Cassette Exon	0,18
TC1700001600.mm.1	-1,16	1520401A03Rik	JUC1700007614.mm.1	-2,45	0,001897	0,338712		
TC1700001600.mm.1	-1,16	1520401A03Rik	JUC1700007622.mm.1	-2,45	0,001897	0,338712		
TC0800002144.mm.1	1,26	Klkb1; Cyp4v3	PSR0800016472.mm.1	2,84	0,012432	0,43528	Cassette Exon	0,12
TC1300002146.mm.1	-1,35	Ctsq	JUC1300007144.mm.1	2,84	0,045858	0,555043		
TC0800002404.mm.1	-1,17	Pik3r2	JUC0800009932.mm.1	2,84	0,025455	0,497549		
TC0800002404.mm.1	-1,17	Pik3r2	JUC0800009930.mm.1	-2,24	0,010671	0,423038		
TC0400004074.mm.1	1,23	Per3	JUC0400017716.mm.1	2,83	0,045232	0,553651		

TC0400004074.mm.1	1,23	Per3	PSR0400033947.mm.1	2,31	0,006955	0,395722	Cassette Exon	0,26
TC1600001884.mm.1	1,56	Robo2	JUC1600007678.mm.1	2,83	0,000533	0,304044		
TC1600001884.mm.1	1,56	Robo2	PSR1600014841.mm.1	2,65	0,002042	0,341377	Cassette Exon	0,16
TC1600001884.mm.1	1,56	Robo2	JUC1600007682.mm.1	2,13	0,01306	0,439579		
TC1600001884.mm.1	1,56	Robo2	JUC1600007687.mm.1	-2,5	0,039433	0,539618		
TC1600000292.mm.1	-1,11	Chrd	JUC1600001517.mm.1	2,83	0,024706	0,494838		
TC0800001023.mm.1	-1,22	Cnep1r1	JUC0800004158.mm.1	2,83	0,010341	0,421002		
TC0700002749.mm.1	-1,15	Wdr62	JUC0700013020.mm.1	2,83	0,004662	0,370181		
TC0700002749.mm.1	-1,15	Wdr62	JUC0700013031.mm.1	-2,08	0,032409	0,519325		
TC0400000576.mm.1	-1,58	Nipsnap3b	JUC0400002094.mm.1	2,83	0,021974	0,485125		
TC0400000576.mm.1	-1,58	Nipsnap3b	JUC0400002091.mm.1	2,51	0,020311	0,477245		
TC0Y00000321.mm.1	-1,13	Gm20736	JUC0Y00001501.mm.1	2,83	0,011772	0,430069		
TC0400002035.mm.1	1,16	Cep104	JUC0400008795.mm.1	2,83	0,024941	0,495859		
TC0Y00000215.mm.1	-1,13	Gm20736	JUC0Y00000875.mm.1	2,83	0,011772	0,430069		
TC0X00003040.mm.1	1,27	Glra4	JUC0X00009640.mm.1	2,83	0,033829	0,523962		
TC0X00003040.mm.1	1,27	Glra4	JUC0X00009641.mm.1	-2,18	0,012248	0,43367		
TC0500002840.mm.1	-1,98	Plac8	PSR0500025033.mm.1	2,82	0,0058	0,383104	Alternative 3' Acce	0,27
TC1100003562.mm.1	1,36	Cacna1g	PSR1100032513.mm.1	2,82	0,007071	0,396677	Cassette Exon	0,26
TC1100003562.mm.1	1,36	Cacna1g	PSR1100032536.mm.1	-2,23	0,048372	0,56056	Cassette Exon	0,12
TC0300001092.mm.1	1,5	Kcnd3	JUC0300004578.mm.1	2,82	0,021462	0,482404		
TC0300001092.mm.1	1,5	Kcnd3	PSR0300008820.mm.1	2,15	0,000983	0,31497	Cassette Exon	0,13
TC0300001092.mm.1	1,5	Kcnd3	PSR0300008825.mm.1	2,14	0,044902	0,55271	Cassette Exon	0,21
TC0300001092.mm.1	1,5	Kcnd3	JUC0300004577.mm.1	-4,11	0,044652	0,552129		
TC1000000906.mm.1	1,43	Ankrd24	JUC1000003903.mm.1	2,82	0,015575	0,454968		
TC1000000906.mm.1	1,43	Ankrd24	JUC1000003922.mm.1	-2,06	0,014578	0,449876		
TC1000000906.mm.1	1,43	Ankrd24	PSR1000007242.mm.1	-2,08	0,001092	0,317225	Alternative 3' Acce	0,07
TC1000000906.mm.1	1,43	Ankrd24	PSR1000007230.mm.1	-2,15	0,016406	0,458907	Alternative 3' Acce	0,17
TC1000000906.mm.1	1,43	Ankrd24	JUC1000003909.mm.1	-2,41	0,023122	0,489756		
TC1000000906.mm.1	1,43	Ankrd24	PSR1000007261.mm.1	-2,58	0,036876	0,532257	Alternative 5' Donc	0,16
TC1200001235.mm.1	-1,14	A230065H16Rik	JUC1200004765.mm.1	2,82	0,035095	0,527591		
TC1300001244.mm.1	-1,16	LOC102635797	JUC1300004086.mm.1	2,82	0,031841	0,517877		
TC0200003869.mm.1	-1,32	Ctnnd1	JUC0200017274.mm.1	2,82	0,040917	0,543423		
TC0800001599.mm.1	-1,11	Itgb1	JUC0800007128.mm.1	2,82	0,007265	0,39826		
TC0900001008.mm.1	-1,45	Arpp19	JUC0900004230.mm.1	2,82	0,044559	0,551921		
TC0600001955.mm.1	-1,19	Pot1a	JUC0600008009.mm.1	2,82	0,028246	0,506552		
TC0600001955.mm.1	-1,19	Pot1a	JUC0600008011.mm.1	2,69	0,045317	0,553774		
TC0600003440.mm.1	1,05	Tm7sf3	JUC0600014269.mm.1	2,82	0,013458	0,442805		
TC0600003003.mm.1	1,08	Ankrd26	JUC0600012004.mm.1	2,82	0,01582	0,45594		
TC0600003003.mm.1	1,08	Ankrd26	JUC0600012016.mm.1	-2,16	0,006671	0,393187		
TC0400000179.mm.1	-1,14	Usp45	JUC0400000558.mm.1	2,82	0,014828	0,451168		
TC0400001105.mm.1	-1,05	Usp24	JUC0400003883.mm.1	2,82	0,042526	0,547845		
TC0300002245.mm.1	-1,33	Fhdc1	JUC0300008900.mm.1	2,82	0,001634	0,332913		
TC0400003383.mm.1	-1,03	St3gal3	JUC0400014186.mm.1	2,82	0,001165	0,318159		
TC0400003383.mm.1	-1,03	St3gal3	JUC0400014191.mm.1	-2,77	0,000849	0,311909		
TC0400004104.mm.1	1,5	Ajap1	JUC0400017842.mm.1	2,82	0,030492	0,513341		
TC0400004104.mm.1	1,5	Ajap1	JUC0400017846.mm.1	-2,53	0,004589	0,369794		
TC0400004104.mm.1	1,5	Ajap1	JUC0400017843.mm.1	-2,82	0,009063	0,412827		
TC1000000473.mm.1	1,79	Slc35f1	JUC1000001747.mm.1	2,82	0,0055	0,379939		

TC1000000473.mm.1	1,79	Slc35f1	JUC1000001745.mm.1	2,8	0,022342	0,486348		
TC1100002072.mm.1	-1,86	Gm12586	JUC1100010463.mm.1	2,81	0,040299	0,541823		
TC1100002072.mm.1	-1,86	Gm12586	PSR1100020047.mm.1	2,59	0,010509	0,422115	Cassette Exon	0,23
TC1100003669.mm.1	-1,01	Med1	PSR1100033819.mm.1	2,81	0,011935	0,431703	Cassette Exon	0,2
TC1400002003.mm.1	-1,59	4930597G03Rik; Gm3719	PSR1400015051.mm.1	2,81	0,015584	0,454974	Cassette Exon	0,18
TC0200004454.mm.1	-1,45	Ehd4	JUC0200019138.mm.1	2,81	0,015564	0,454812		
TC0200004454.mm.1	-1,45	Ehd4	PSR0200037310.mm.1	-2,35	0,013339	0,441924	Cassette Exon	0,16
TC1200001761.mm.1	1,09	Gm2436	PSR1200012134.mm.1	2,81	0,04656	0,556761	Cassette Exon	0,16
TC1200001762.mm.1	1,09	Gm2446	PSR1200012143.mm.1	2,81	0,04656	0,556761	Cassette Exon	0,16
TC0200002272.mm.1	-2,33	Slc52a3	PSR0200017773.mm.1	2,81	0,034956	0,52724	Alternative 3' Acce	0,15
TC0300000407.mm.1	1,38	Trpc4	JUC0300001428.mm.1	2,81	0,004832	0,371555		
TC0300000407.mm.1	1,38	Trpc4	JUC0300001423.mm.1	-2,07	0,028346	0,506843		
TC0100002543.mm.1	-1,73	Abca12	JUC0100011534.mm.1	2,81	0,01078	0,423955		
TC0100002543.mm.1	-1,73	Abca12	JUC0100011535.mm.1	2,25	0,025595	0,497814		
TC0100000375.mm.1	-1,35	Stat1	JUC0100001709.mm.1	2,81	0,024165	0,493245		
TC1700000183.mm.1	1,19	Tbp	JUC1700000817.mm.1	2,81	0,004945	0,373282		
TC1700000183.mm.1	1,19	Tbp	JUC1700000806.mm.1	-2,13	0,014424	0,448786		
TC1600000491.mm.1	1,24	Adcy5	JUC1600002447.mm.1	2,81	0,003073	0,353892		
TC0900000720.mm.1	1,68	Sema7a	JUC0900002793.mm.1	2,81	0,012701	0,43642		
TC0900000187.mm.1	-1,23	1700084C06Rik	JUC0900000598.mm.1	2,81	0,004071	0,361956		
TC1100002729.mm.1	1,36	Uqcrq	JUC1100012873.mm.1	2,81	0,012556	0,435721		
TC1100003382.mm.1	-1,29	Slfn8	JUC1100016216.mm.1	2,81	0,018774	0,470789		
TC1100001792.mm.1	-1,8		JUC1100008820.mm.1	2,81	0,017538	0,464105		
TC0X00000414.mm.1	-1,33	Prr32; 1110059M19Rik	JUC0X00001376.mm.1	2,81	0,033561	0,523131		
TC0900002283.mm.1	-1,44	Layn	JUC0900010743.mm.1	2,81	0,026622	0,50179		
TC0900002283.mm.1	-1,44	Layn	JUC0900010742.mm.1	2,46	0,001676	0,335182		
TC1000000541.mm.1	1,09	Ascc1	JUC1000002008.mm.1	2,81	0,019397	0,473783		
TC0100003175.mm.1	1,55	Lhx9	PSR0100025751.mm.1	2,8	0,046682	0,556849	Alternative 3' Acce	0,29
TC1300001666.mm.1	1,05	Gmnn	PSR1300010376.mm.1	2,8	0,04971	0,563531	Alternative 5' Donc	0,29
TC0400002105.mm.1	1,07	9430015G10Rik	PSR0400017762.mm.1	2,8	0,000061	0,250119	Alternative 3' Acce	0,28
TC0100002561.mm.1	-1,56		PSR0100020649.mm.1	2,8	0,033306	0,522389	Alternative 5' Donc	0,27
TC0100002561.mm.1	-1,56		JUC0100011700.mm.1	2,57	0,005426	0,379494		
TC0100002561.mm.1	-1,56		PSR0100020645.mm.1	2,27	0,001344	0,325349	Cassette Exon	0,19
TC1900001592.mm.1	-1,39	9130011E15Rik	PSR1900014161.mm.1	2,8	0,032512	0,519846	Cassette Exon	0,17
TC1900001592.mm.1	-1,39	9130011E15Rik	PSR1900014158.mm.1	2,74	0,013588	0,443703	Cassette Exon	0,23
TC1900001592.mm.1	-1,39	9130011E15Rik	JUC1900007821.mm.1	2,34	0,024231	0,493294		
TC1900001592.mm.1	-1,39	9130011E15Rik	JUC1900007840.mm.1	2,23	0,020447	0,478018		
TC0300001318.mm.1	-1,02	Col25a1; 4930502M04Rik	PSR0300010687.mm.1	2,8	0,003286	0,354243	Cassette Exon	0,21
TC0300001318.mm.1	-1,02	Col25a1; 4930502M04Rik	PSR0300010667.mm.1	2,71	0,008351	0,406435	Cassette Exon	0,14
TC0300001318.mm.1	-1,02	Col25a1; 4930502M04Rik	JUC0300005579.mm.1	2,61	0,03088	0,51451		
TC0500000247.mm.1	-2,2	Gm7361	PSR0500001870.mm.1	2,8	0,033552	0,523131	Cassette Exon	0,18
TC0500000247.mm.1	-2,2	Gm7361	JUC0500001062.mm.1	2,24	0,01039	0,421341		
TC0600002564.mm.1	-1,17	Gm15624; LOC102635553	PSR0600019899.mm.1	2,8	0,008068	0,404508	Cassette Exon	0,17
TC0200005505.mm.1	1,76	4930402H24Rik; Gm1405	JUC0200020576.mm.1	2,8	0,011994	0,432286		
TC0200005505.mm.1	1,76	4930402H24Rik; Gm1405	PSR0200039760.mm.1	-2,21	0,029569	0,510456	Alternative 3' Acce	0,14
TC1400001886.mm.1	-1,25	Gm5624	JUC1400007821.mm.1	2,8	0,009606	0,417256		
TC0300000635.mm.1	-1,06	Ctso	JUC0300002157.mm.1	2,8	0,000961	0,313363		
TC1300000101.mm.1	-1,03	Psma2	JUC1300000461.mm.1	2,8	0,029354	0,509995		

TC0100002801.mm.1	-1,14	Per2	JUC0100012865.mm.1	2,8	0,024186	0,493294		
TC1700001606.mm.1	-1,36	Tceb2	JUC1700007672.mm.1	2,8	0,00296	0,352501		
TC1900001649.mm.1	1,45	Sorcs1	JUC1900008137.mm.1	2,8	0,002727	0,349612		
TC1900001649.mm.1	1,45	Sorcs1	JUC1900008132.mm.1	2,19	0,033234	0,522175		
TC1800001449.mm.1	-1,28	Piezo2	JUC1800005911.mm.1	2,8	0,031992	0,518312		
TC1800001449.mm.1	-1,28	Piezo2	JUC1800005942.mm.1	2,63	0,005062	0,375196		
TC1800001384.mm.1	-1,23	Fbn2	JUC1800005580.mm.1	2,8	0,016115	0,457756		
TC1800001384.mm.1	-1,23	Fbn2	JUC1800005613.mm.1	-3,13	0,002555	0,349501		
TC1500000558.mm.1	1,04	Zfp41	JUC1500002248.mm.1	2,8	0,028821	0,508362		
TC1600001742.mm.1	-1,21	Nfkbiz	JUC1600007343.mm.1	2,8	0,006437	0,390047		
TC1600001047.mm.1	-1,26	Dopey2	JUC1600004228.mm.1	2,8	0,007375	0,39943		
TC0800000525.mm.1	1,34	Ufsp2	JUC0800001977.mm.1	2,8	0,020661	0,478862		
TC0500000701.mm.1	-1,06	Fip1l1	JUC0500003415.mm.1	2,8	0,034581	0,52602		
TC0500000701.mm.1	-1,06	Fip1l1	JUC0500003407.mm.1	2,37	0,016744	0,460378		
TC0800002777.mm.1	-1,19		JUC0800011630.mm.1	2,8	0,015446	0,454271		
TC0800002869.mm.1	-1,16	Pdf; Cog8	JUC0800012090.mm.1	2,8	0,048272	0,560283		
TC0700002790.mm.1	-1,09	Hpn	JUC0700013377.mm.1	2,8	0,003318	0,354243		
TC0600000556.mm.1	3,05	Atp6v0e2	JUC0600002175.mm.1	2,8	0,010702	0,423331		
TC0400004174.mm.1	1,13	Klhl17; Mir7658; mmu-mi	JUC0400018289.mm.1	2,8	0,007737	0,402774		
TC0Y00000564.mm.1	-1,11	Gm20835	JUC0Y00001470.mm.1	2,8	0,020161	0,477086		
TC1400000344.mm.1	1,56	Ogdhl	JUC1400001796.mm.1	2,79	0,036701	0,531756		
TC1400000344.mm.1	1,56	Ogdhl	PSR1400003038.mm.1	2,77	0,029339	0,509981	Cassette Exon	0,15
TC1400000344.mm.1	1,56	Ogdhl	JUC1400001805.mm.1	-2,51	0,007816	0,403089		
TC1400000344.mm.1	1,56	Ogdhl	PSR1400003034.mm.1	-3,35	0,014357	0,448293	Alternative 3' Acce	0,4
TC0400000280.mm.1	9,09	Gabrr1	PSR0400001830.mm.1	2,79	0,001783	0,336311	Mutually Exclusive	0,3
TC0400000280.mm.1	9,09	Gabrr1	PSR0400001836.mm.1	-2,21	0,013254	0,441424	Cassette Exon	0,37
TC0400000280.mm.1	9,09	Gabrr1	PSR0400001828.mm.1	-2,4	0,000761	0,308387	Cassette Exon	0,29
TC0400000280.mm.1	9,09	Gabrr1	PSR0400001831.mm.1	-2,55	0,002889	0,352207	Mutually Exclusive	0,18
TC0400000280.mm.1	9,09	Gabrr1	PSR0400001837.mm.1	-2,84	0,025786	0,498505	Cassette Exon	0,31
TC0400000280.mm.1	9,09	Gabrr1	PSR0400001829.mm.1	-3	0,011001	0,425491	Mutually Exclusive	0,3
TC0400000280.mm.1	9,09	Gabrr1	JUC0400000911.mm.1	-3,31	0,001619	0,332831		
TC0400000280.mm.1	9,09	Gabrr1	PSR0400001827.mm.1	-3,61	0,016497	0,459419	Cassette Exon	0,29
TC0400000280.mm.1	9,09	Gabrr1	JUC0400000918.mm.1	-3,66	0,002247	0,34578		
TC0400000280.mm.1	9,09	Gabrr1	JUC0400000919.mm.1	-12,17	0,001271	0,322251		
TC1700000407.mm.1	2,15	Rgs11	JUC1700001865.mm.1	2,79	0,002118	0,342919		
TC1700000407.mm.1	2,15	Rgs11	PSR1700003449.mm.1	2,43	0,001756	0,335996	Cassette Exon	0,26
TC1700000407.mm.1	2,15	Rgs11	JUC1700001864.mm.1	2,09	0,020129	0,476862		
TC1700000407.mm.1	2,15	Rgs11	JUC1700001879.mm.1	-2,04	0,011273	0,426793		
TC1700000407.mm.1	2,15	Rgs11	PSR1700003438.mm.1	-2,08	0,006598	0,39213	Intron Retention	0,27
TC1700000407.mm.1	2,15	Rgs11	PSR1700003460.mm.1	-2,12	0,010668	0,423012	Cassette Exon	0,1
TC1700000407.mm.1	2,15	Rgs11	JUC1700001878.mm.1	-2,26	0,042474	0,547627		
TC1700000407.mm.1	2,15	Rgs11	JUC1700001863.mm.1	-2,34	0,029475	0,510123		
TC1700000407.mm.1	2,15	Rgs11	PSR1700003459.mm.1	-2,44	0,003778	0,357586	Alternative 3' Acce	0,05
TC1700000407.mm.1	2,15	Rgs11	PSR1700003436.mm.1	-2,75	0,004855	0,37168	Alternative 3' Acce	0,19
TC1700000407.mm.1	2,15	Rgs11	JUC1700001876.mm.1	-2,88	0,0087	0,409002		
TC1600001661.mm.1	-1,08	Tmprss7	JUC1600007082.mm.1	2,79	0,010415	0,42148		
TC1600001661.mm.1	-1,08	Tmprss7	PSR1600013583.mm.1	2,01	0,046293	0,556186	Cassette Exon	0,09
TC1200001597.mm.1	-1,19	Bcap29	JUC1200006129.mm.1	2,79	0,033325	0,522415		

TC1200002108.mm.1	1,02	Angel1	JUC1200008052.mm.1	2,79	0,010692	0,423294		
TC0200004455.mm.1	-1,13	Pla2g4e; Gm13997	JUC0200019151.mm.1	2,79	0,013784	0,444974		
TC0200004455.mm.1	-1,13	Pla2g4e; Gm13997	JUC0200019147.mm.1	2,3	0,041204	0,544126		
TC1800001672.mm.1	1,09	4930594M17Rik	JUC1800006680.mm.1	2,79	0,029213	0,509515		
TC1900000817.mm.1	-1,21		JUC1900003927.mm.1	2,79	0,015695	0,455416		
TC0200000036.mm.1	1,26	Sephs1	JUC0200000129.mm.1	2,79	0,019295	0,473074		
TC0500001261.mm.1	1,07	Pla2g1b	JUC0500005941.mm.1	2,79	0,04793	0,559506		
TC0600002912.mm.1	1,35	Lhfpl4	JUC0600011595.mm.1	2,79	0,031704	0,517443		
TC0600000362.mm.1	-1,17	Trim24	JUC0600001407.mm.1	2,79	0,032264	0,519058		
TC1000001767.mm.1	-1,48	Ltv1	JUC1000007001.mm.1	2,79	0,002477	0,349135		
TC0300001485.mm.1	-1,4	2410004B18Rik	JUC0300006324.mm.1	2,79	0,021301	0,481714		
TC1000000372.mm.1	1,09	Sesn1	JUC1000001419.mm.1	2,79	0,032912	0,521149		
TC0700000832.mm.1	9,65	Slc17a7	JUC0700003520.mm.1	2,78	0,001116	0,317328		
TC0700000832.mm.1	9,65	Slc17a7	JUC0700003523.mm.1	-2,28	0,005812	0,383104		
TC0700000832.mm.1	9,65	Slc17a7	JUC0700003526.mm.1	-3,02	0,035248	0,52837		
TC0700000832.mm.1	9,65	Slc17a7	PSR0700007123.mm.1	-4,5	0,022156	0,48561	Intron Retention	0,38
TC0700000832.mm.1	9,65	Slc17a7	PSR0700007133.mm.1	-5,68	0,025354	0,497395	Alternative 5' Donc	0,45
TC0700000832.mm.1	9,65	Slc17a7	PSR0700007127.mm.1	-5,84	0,015425	0,454071	Intron Retention	0,71
TC0700000832.mm.1	9,65	Slc17a7	JUC0700003517.mm.1	-6,39	0,025767	0,498444		
TC0700000832.mm.1	9,65	Slc17a7	PSR0700007125.mm.1	-6,85	0,005993	0,386404	Intron Retention	0,75
TC0700000832.mm.1	9,65	Slc17a7	PSR0700007131.mm.1	-6,93	0,007079	0,396891	Intron Retention	0,7
TC0700000832.mm.1	9,65	Slc17a7	PSR0700007119.mm.1	-7,7	0,022974	0,48884	Cassette Exon	0,27
TC0700000832.mm.1	9,65	Slc17a7	PSR0700007129.mm.1	-12,16	0,006277	0,388804	Intron Retention	0,69
TC0800001362.mm.1	1,3	Wwox	JUC0800006120.mm.1	2,78	0,007702	0,402552		
TC0800001362.mm.1	1,3	Wwox	JUC0800006132.mm.1	-2,36	0,030722	0,514036		
TC0800001362.mm.1	1,3	Wwox	PSR0800011226.mm.1	-2,84	0,007577	0,401703	Alternative 5' Donc	0,23
TC1100004115.mm.1	2,1	Foxj1; Rnf157	PSR1100038958.mm.1	2,78	0,006588	0,39213	Cassette Exon	0,16
TC1100004115.mm.1	2,1	Foxj1; Rnf157	PSR1100038949.mm.1	2,25	0,032621	0,520338	Cassette Exon	0,13
TC1100004115.mm.1	2,1	Foxj1; Rnf157	JUC1100020439.mm.1	-2,08	0,041835	0,545586		
TC1100004115.mm.1	2,1	Foxj1; Rnf157	PSR1100038912.mm.1	-2,32	0,040454	0,542112	Cassette Exon	0,12
TC1100004115.mm.1	2,1	Foxj1; Rnf157	PSR1100038913.mm.1	-2,67	0,027935	0,505612	Intron Retention	0,13
TC1100004115.mm.1	2,1	Foxj1; Rnf157	JUC1100020419.mm.1	-2,92	0,010529	0,42227		
TC1100004115.mm.1	2,1	Foxj1; Rnf157	PSR1100038961.mm.1	-2,98	0,01748	0,463933	Alternative 5' Donc	0,19
TC0Y00000341.mm.1	-1,45	LOC101055632; 1700040F	PSR0Y00002054.mm.1	2,78	0,014861	0,451623	Cassette Exon	0,18
TC0Y00000341.mm.1	-1,45	LOC101055632; 1700040F	JUC0Y00001576.mm.1	2,46	0,006216	0,387912		
TC0400002097.mm.1	-2,06	Cpsf3l	PSR0400017550.mm.1	2,78	0,038985	0,537923	Cassette Exon	0,17
TC1600001024.mm.1	1,07	Kcne2	PSR1600008024.mm.1	2,78	0,002703	0,349612	Cassette Exon	0,14
TC1600001024.mm.1	1,07	Kcne2	JUC1600004160.mm.1	2,34	0,010048	0,418902		
TC1200000348.mm.1	-1,11	Etv1; Gm5454	JUC1200001433.mm.1	2,78	0,001782	0,336311		
TC1200000348.mm.1	-1,11	Etv1; Gm5454	JUC1200001437.mm.1	2,23	0,000379	0,293183		
TC0200001365.mm.1	-1,19	Rapsn	JUC0200005252.mm.1	2,78	0,003861	0,358861		
TC0200002201.mm.1	-1,37	Cst8	JUC0200008770.mm.1	2,78	0,02504	0,496116		
TC0500000690.mm.1	-1,12	Spata18	JUC0500003369.mm.1	2,78	0,018893	0,471219		
TC0500000690.mm.1	-1,12	Spata18	JUC0500003370.mm.1	2,68	0,001404	0,325997		
TC0700003414.mm.1	1,58	Fam189a1	JUC0700015382.mm.1	2,78	0,027146	0,503137		
TC1100000596.mm.1	-1,41	Fstl4	JUC1100002432.mm.1	2,78	0,01263	0,436139		
TC1000001584.mm.1	1,44	Atp5b	JUC1000006161.mm.1	2,78	0,04339	0,54959		
TC0300002358.mm.1	1,13	Atp8b2	JUC0300009536.mm.1	2,78	0,004918	0,372872		

TC0300002358.mm.1	1,13	Atp8b2	JUC0300009540.mm.1	2,58	0,010569	0,422432		
TC0900003015.mm.1	1,52	Grm2	JUC0900014052.mm.1	2,78	0,000186	0,284485		
TC0400001544.mm.1	-1,29	Snrnp40	JUC0400006345.mm.1	2,78	0,02705	0,502979		
TC0400001544.mm.1	-1,29	Snrnp40	JUC0400006341.mm.1	2,38	0,027893	0,505612		
TC0X00002706.mm.1	-2,06	Pdzd11	JUC0X00008604.mm.1	2,78	0,012318	0,434392		
TC0500002232.mm.1	1,97	Zfyve28	JUC0500011129.mm.1	2,77	0,009134	0,413353		
TC0500002232.mm.1	1,97	Zfyve28	PSR0500020402.mm.1	2,63	0,014337	0,448173	Mutually Exclusive	0,18
TC0500002232.mm.1	1,97	Zfyve28	PSR0500020395.mm.1	2,63	0,000305	0,288663	Cassette Exon	0,12
TC0500002232.mm.1	1,97	Zfyve28	PSR0500020393.mm.1	2,44	0,006591	0,39213	Alternative 3' Acce	0,2
TC0500002232.mm.1	1,97	Zfyve28	JUC0500011130.mm.1	2,17	0,001703	0,335511		
TC0500002232.mm.1	1,97	Zfyve28	PSR0500020401.mm.1	-2,18	0,037311	0,533573	Mutually Exclusive	0,18
TC0500002232.mm.1	1,97	Zfyve28	PSR0500020408.mm.1	-2,46	0,010019	0,418618	Cassette Exon	0,28
TC0500002232.mm.1	1,97	Zfyve28	JUC0500011128.mm.1	-2,9	0,024951	0,495859		
TC0500002232.mm.1	1,97	Zfyve28	JUC0500011134.mm.1	-2,97	0,0064	0,389654		
TC0500002232.mm.1	1,97	Zfyve28	JUC0500011141.mm.1	-3,21	0,019445	0,474131		
TC0600001288.mm.1	1,29	Ift122	PSR0600010165.mm.1	2,77	0,015371	0,453849	Alternative 3' Acce	0,28
TC0600001288.mm.1	1,29	Ift122	JUC0600005281.mm.1	2,15	0,043097	0,548553		
TC0600001288.mm.1	1,29	Ift122	PSR0600010164.mm.1	-2,02	0,033251	0,52224	Alternative 3' Acce	0,16
TC0600001288.mm.1	1,29	Ift122	PSR0600010195.mm.1	-2,17	0,026254	0,500323	Alternative 3' Acce	0,2
TC1900001599.mm.1	3,28	Psd	PSR1900014227.mm.1	2,77	0,000551	0,304044	Mutually Exclusive	0,22
TC1900001599.mm.1	3,28	Psd	PSR1900014233.mm.1	2,65	0,008462	0,406624	Cassette Exon	0,21
TC1900001599.mm.1	3,28	Psd	PSR1900014240.mm.1	2,14	0,020324	0,477245		
TC1900001599.mm.1	3,28	Psd	PSR1900014222.mm.1	-2,42	0,024678	0,49472	Cassette Exon	0,13
TC1900001599.mm.1	3,28	Psd	JUC1900007879.mm.1	-2,82	0,00797	0,403853		
TC1900001599.mm.1	3,28	Psd	PSR1900014228.mm.1	-2,96	0,005723	0,382767	Mutually Exclusive	0,22
TC1900001599.mm.1	3,28	Psd	JUC1900007881.mm.1	-3,26	0,032133	0,518586		
TC1900001599.mm.1	3,28	Psd	JUC1900007878.mm.1	-3,31	0,01625	0,458312		
TC1900001599.mm.1	3,28	Psd	PSR1900014229.mm.1	-3,44	0,006344	0,389463	Cassette Exon	0,23
TC1900001599.mm.1	3,28	Psd	PSR1900014243.mm.1	-4,01	0,004421	0,366832	Cassette Exon	0,28
TC0300003237.mm.1	-1,52	Mtx1	PSR0300017903.mm.1	2,77	0,011092	0,425947	Alternative 5' Donc	0,27
TC0300003237.mm.1	-1,52	Mtx1	PSR0300017904.mm.1	2,45	0,028057	0,505951		
TC0300003237.mm.1	-1,52	Mtx1	PSR0300017886.mm.1	2,17	0,03594	0,529399	Alternative 3' Acce	0,15
TC0400003478.mm.1	-1,35	Ppie	PSR0400028188.mm.1	2,77	0,00185	0,337414	Alternative 3' Acce	0,24
TC0200001741.mm.1	1,46	Bub1b	JUC0200006427.mm.1	2,77	0,009561	0,416927		
TC0200001741.mm.1	1,46	Bub1b	PSR0200012894.mm.1	2,02	0,031403	0,516484	Alternative 5' Donc	0,16
TC0200001741.mm.1	1,46	Bub1b	PSR0200012880.mm.1	-2,68	0,009187	0,413756	Cassette Exon	0,18
TC0200002026.mm.1	-1,05	Prnd; Prnp; Prn; RP23-401	PSR0200016115.mm.1	2,77	0,003422	0,355303	Cassette Exon	0,17
TC0200002026.mm.1	-1,05	Prnd; Prnp; Prn; RP23-401	PSR0200016121.mm.1	2,15	0,045973	0,555395	Cassette Exon	0,11
TC0200002026.mm.1	-1,05	Prnd; Prnp; Prn; RP23-401	PSR0200016123.mm.1	2,12	0,013747	0,444758	Cassette Exon	0,11
TC0400000743.mm.1	-1,76	Tlr4	PSR0400005325.mm.1	2,77	0,018306	0,468049	Cassette Exon	0,17
TC0400002530.mm.1	-1,64	Tln1	JUC0400010738.mm.1	2,77	0,031494	0,516965		
TC0400002530.mm.1	-1,64	Tln1	PSR0400020601.mm.1	2,52	0,024362	0,49362	Cassette Exon	0,17
TC0400002530.mm.1	-1,64	Tln1	JUC0400010719.mm.1	2,14	0,008667	0,408649		
TC0200005360.mm.1	1,02	Gm14410; Gm14411; Gm1	PSR0200045648.mm.1	2,77	0,028455	0,507299	Cassette Exon	0,16
TC0X00003430.mm.1	-1,32	Arhgap4	JUC0X00007787.mm.1	2,77	0,00172	0,335996		
TC0X00003430.mm.1	-1,32	Arhgap4	PSR0X00015214.mm.1	2,1	0,008724	0,409361	Cassette Exon	0,14
TC0X00003430.mm.1	-1,32	Arhgap4	PSR0X00015201.mm.1	2,03	0,032011	0,518367	Alternative 5' Donc	0,14
TC0900003311.mm.1	1,6	Fyco1; Gm17021	JUC0900015710.mm.1	2,77	0,024954	0,495885		

TC0900003311.mm.1	1,6	Fyco1; Gm17021	PSR0900027966.mm.1	2,23	0,036432	0,530967	Cassette Exon	0,12
TC1900000118.mm.1	-1,12	Rasgrp2	JUC1900000810.mm.1	2,77	0,025662	0,498118		
TC0800001236.mm.1	1,13	Dus2; Dus2l	JUC0800005380.mm.1	2,77	0,003714	0,357586		
TC0900001011.mm.1	-1,11	Myo5c	JUC0900004310.mm.1	2,77	0,022707	0,487767		
TC0900001011.mm.1	-1,11	Myo5c	JUC0900004325.mm.1	2,12	0,024654	0,49468		
TC1100000654.mm.1	3,72	Gria1	JUC1100002742.mm.1	2,76	0,002445	0,349135		
TC1100000654.mm.1	3,72	Gria1	PSR1100005290.mm.1	2,25	0,032857	0,521076	Cassette Exon	0,25
TC1100000654.mm.1	3,72	Gria1	JUC1100002741.mm.1	-2,2	0,046725	0,556856		
TC1100000654.mm.1	3,72	Gria1	JUC1100002738.mm.1	-2,87	0,024078	0,49304		
TC1100000654.mm.1	3,72	Gria1	PSR1100005276.mm.1	-3,02	0,031789	0,517596	Cassette Exon	0,2
TC1100000654.mm.1	3,72	Gria1	PSR1100005279.mm.1	-4,96	0,041755	0,545454	Cassette Exon	0,3
TC0200003365.mm.1	-1,27	Ggta1	PSR0200028965.mm.1	2,76	0,015738	0,455416	Cassette Exon	0,16
TC0X00003069.mm.1	-1,54	Nup62cl	PSR0X00019282.mm.1	2,76	0,027908	0,505612	Cassette Exon	0,16
TC0X00003069.mm.1	-1,54	Nup62cl	JUC0X00009767.mm.1	2,5	0,020637	0,478837		
TC1300000708.mm.1	-1,22	1700066J03Rik	JUC1300002436.mm.1	2,76	0,007719	0,402552		
TC0800003009.mm.1	-1,17	Pkd1l2	JUC0800012680.mm.1	2,76	0,004574	0,369789		
TC0500000093.mm.1	1,55	Sema3e	JUC0500000469.mm.1	2,76	0,000362	0,290942		
TC0X00002068.mm.1	-1,37	Rhox2g	JUC0X00006699.mm.1	2,76	0,002724	0,349612		
TC1100002557.mm.1	6,03	Gabra1	JUC1100012317.mm.1	2,75	0,001281	0,322251		
TC1100002557.mm.1	6,03	Gabra1	JUC1100012316.mm.1	2,54	0,002081	0,34222		
TC1100002557.mm.1	6,03	Gabra1	PSR1100023663.mm.1	2,43	0,001841	0,337414	Cassette Exon	0,12
TC1100002557.mm.1	6,03	Gabra1	JUC1100012313.mm.1	-2,12	0,010841	0,424161		
TC1100002557.mm.1	6,03	Gabra1	PSR1100023668.mm.1	-2,15	0,001131	0,317328	Cassette Exon	0,04
TC1100002557.mm.1	6,03	Gabra1	PSR1100023654.mm.1	-2,85	0,027667	0,504707	Alternative 3' Acce	0,25
TC1100002557.mm.1	6,03	Gabra1	JUC1100012318.mm.1	-3	0,039963	0,540973		
TC1100002557.mm.1	6,03	Gabra1	PSR1100023671.mm.1	-3,52	0,001593	0,331726	Cassette Exon	0,37
TC1100002557.mm.1	6,03	Gabra1	JUC1100012321.mm.1	-4,08	0,0321	0,518557		
TC1100002557.mm.1	6,03	Gabra1	JUC1100012323.mm.1	-4,68	0,031832	0,517847		
TC1100002557.mm.1	6,03	Gabra1	PSR1100023669.mm.1	-5,05	0,015366	0,453849	Cassette Exon	0,21
TC1100002557.mm.1	6,03	Gabra1	PSR1100023674.mm.1	-5,33	0,010584	0,42255	Alternative 5' Donc	0,38
TC1100002557.mm.1	6,03	Gabra1	PSR1100023677.mm.1	-5,48	0,008256	0,405976	Alternative 5' Donc	0,38
TC1100002557.mm.1	6,03	Gabra1	JUC1100012324.mm.1	-6,34	0,035688	0,529027		
TC1100002557.mm.1	6,03	Gabra1	PSR1100023676.mm.1	-11,05	0,005718	0,382767	Alternative 5' Donc	0,38
TC1100002557.mm.1	6,03	Gabra1	PSR1100023666.mm.1	-15,37	0,005325	0,378059	Cassette Exon	0,27
TC0200004102.mm.1	-1,18	Ndufs3	PSR0200034231.mm.1	2,75	0,003761	0,357586	Alternative 3' Acce	0,28
TC1000002482.mm.1	1,21	BC005764	PSR1000018180.mm.1	2,75	0,020137	0,476995	Cassette Exon	0,14
TC1000002482.mm.1	1,21	BC005764	JUC1000009977.mm.1	-2,6	0,045149	0,553323		
TC1000002482.mm.1	1,21	BC005764	PSR1000018162.mm.1	-2,93	0,045118	0,553234	Cassette Exon	0,25
TC0X00002593.mm.1	2,09	Mir1906-2; Gm27000	PSR0X00016306.mm.1	2,75	0,00138	0,32572	Alternative 3' Acce	0,17
TC0X00002593.mm.1	2,09	Mir1906-2; Gm27000	PSR0X00016308.mm.1	2,41	0,024792	0,495094	Alternative 5' Donc	0,23
TC0400003076.mm.1	-1,71	Mysm1	JUC0400012813.mm.1	2,75	0,036708	0,531762		
TC0400003076.mm.1	-1,71	Mysm1	PSR0400024629.mm.1	2,32	0,011625	0,429746	Alternative 3' Acce	0,19
TC1100003559.mm.1	1,18	Luc7l3	PSR1100032448.mm.1	2,75	0,00587	0,383835	Cassette Exon	0,14
TC1100003559.mm.1	1,18	Luc7l3	PSR1100032442.mm.1	-2,13	0,035181	0,528125	Alternative 5' Donc	0,18
TC1100003559.mm.1	1,18	Luc7l3	JUC1100016952.mm.1	-3,74	0,019571	0,474672		
TC1100003559.mm.1	1,18	Luc7l3	JUC1100016949.mm.1	-4,01	0,020463	0,478018		
TC1100001572.mm.1	-2,24		PSR1100014579.mm.1	2,75	0,031656	0,517405	Cassette Exon	0,16
TC1100001572.mm.1	-2,24		PSR1100014580.mm.1	2,25	0,023549	0,490947	Cassette Exon	0,14

TC1200001512.mm.1	1,66	Gm9292	PSR1200010578.mm.1	2,75	0,017386	0,463704	Cassette Exon	0,09
TC1500001104.mm.1	-1,3		JUC1500005046.mm.1	2,75	0,001992	0,341091		
TC0600000917.mm.1	-1,1	Dqx1	JUC0600003477.mm.1	2,75	0,031761	0,517489		
TC0700001212.mm.1	-1,3	2610206C17Rik	JUC0700005526.mm.1	2,75	0,003556	0,356223		
TC0600002472.mm.1	1,31	Kdm3a	JUC0600009914.mm.1	2,75	0,037664	0,53431		
TC0600003373.mm.1	1,03	Recql	JUC0600013877.mm.1	2,75	0,005727	0,382767		
TC0600001644.mm.1	-1,14		JUC0600006863.mm.1	2,75	0,029121	0,509241		
TC0700001356.mm.1	1,04	Rsf1	JUC0700005939.mm.1	2,75	0,034044	0,524583		
TC0700002062.mm.1	-1,4	Muc5ac	JUC0700009772.mm.1	2,75	0,020246	0,477245		
TC0700002062.mm.1	-1,4	Muc5ac	JUC0700009792.mm.1	2,31	0,01928	0,473074		
TC0700002062.mm.1	-1,4	Muc5ac	JUC0700009764.mm.1	-2,09	0,027668	0,504707		
TC1100002462.mm.1	-1,45		JUC1100011908.mm.1	2,75	0,006286	0,388916		
TC0400001437.mm.1	1,35	Meaf6	JUC0400005682.mm.1	2,75	0,023418	0,490343		
TC1500001060.mm.1	3,45	Ankrd33	JUC1500004770.mm.1	2,74	0,001536	0,330388		
TC1500001060.mm.1	3,45	Ankrd33	PSR1500008414.mm.1	-3,24	0,01097	0,42518	Cassette Exon	0,34
TC1500001060.mm.1	3,45	Ankrd33	JUC1500004772.mm.1	-4,07	0,005952	0,385276		
TC1200002263.mm.1	-1,82	Asb2	PSR1200015725.mm.1	2,74	0,015099	0,452453	Alternative 5' Donc	0,29
TC1300001775.mm.1	-1,11	Serpinb6e	JUC1300005643.mm.1	2,74	0,008734	0,409583		
TC1300001775.mm.1	-1,11	Serpinb6e	PSR1300011050.mm.1	-3,76	0,019141	0,472472	Cassette Exon	0,29
TC1900000628.mm.1	2,34	Cnnm1	JUC1900002970.mm.1	2,74	0,015811	0,455923		
TC1900000628.mm.1	2,34	Cnnm1	PSR1900005580.mm.1	-2,14	0,036711	0,531762	Cassette Exon	0,06
TC1900000628.mm.1	2,34	Cnnm1	PSR1900005581.mm.1	-2,35	0,000631	0,304044	Cassette Exon	0,1
TC1900000628.mm.1	2,34	Cnnm1	PSR1900005578.mm.1	-2,54	0,011428	0,427656	Cassette Exon	0,29
TC1900000628.mm.1	2,34	Cnnm1	JUC1900002976.mm.1	-3,51	0,003197	0,354243		
TC0100000765.mm.1	-1,39	Sp140; Gm2427	JUC0100003626.mm.1	2,74	0,003791	0,357817		
TC0100000765.mm.1	-1,39	Sp140; Gm2427	PSR0100006332.mm.1	2,12	0,004511	0,368648	Cassette Exon	0,19
TC0100000765.mm.1	-1,39	Sp140; Gm2427	JUC0100003639.mm.1	2,02	0,048479	0,560864		
TC0400001721.mm.1	1,14	Sh2d5; Kif17	PSR0400014046.mm.1	2,74	0,017463	0,463886	Cassette Exon	0,18
TC0400001721.mm.1	1,14	Sh2d5; Kif17	PSR0400014045.mm.1	2,31	0,002656	0,349612	Cassette Exon	0,18
TC1400000340.mm.1	1,06	Ncoa4	JUC1400001755.mm.1	2,74	0,038939	0,537874		
TC1300001928.mm.1	1,12	Dek	JUC1300006174.mm.1	2,74	0,005441	0,379743		
TC0900000643.mm.1	1,06	Npat	JUC0900002404.mm.1	2,74	0,007756	0,402774		
TC0600003433.mm.1	-1,44	Itpr2	JUC0600014219.mm.1	2,74	0,04559	0,554548		
TC0600003433.mm.1	-1,44	Itpr2	JUC0600014191.mm.1	2,47	0,038843	0,537777		
TC0600003433.mm.1	-1,44	Itpr2	JUC0600014236.mm.1	2,39	0,045122	0,553247		
TC0600003433.mm.1	-1,44	Itpr2	JUC0600014225.mm.1	2,26	0,024135	0,493245		
TC0700001348.mm.1	-1,54	Alg8	JUC0700005894.mm.1	2,74	0,031756	0,517489		
TC0700001737.mm.1	1,08	Polr3e	JUC0700007709.mm.1	2,74	0,037695	0,534488		
TC1100002515.mm.1	-1,11	Tenm2	JUC1100012237.mm.1	2,74	0,032136	0,518586		
TC0100002974.mm.1	-1,15	Ddx18	PSR0100023977.mm.1	2,73	0,004155	0,3623	Alternative 5' Donc	0,26
TC1600001142.mm.1	-1,72		PSR1600009111.mm.1	2,73	0,02727	0,503711	Alternative 5' Donc	0,25
TC1600001142.mm.1	-1,72		PSR1600009109.mm.1	2,3	0,010794	0,424049	Alternative 3' Acce	0,18
TC0700003140.mm.1	4,28		PSR0700028313.mm.1	2,73	0,032413	0,519325	Cassette Exon	0,21
TC0700003140.mm.1	4,28		PSR0700028314.mm.1	-2,73	0,032413	0,519325	Cassette Exon	0,11
TC0300001014.mm.1	-1,82	Igsf3	PSR0300008176.mm.1	2,73	0,001192	0,321265	Alternative 5' Donc	0,19
TC0300001014.mm.1	-1,82	Igsf3	PSR0300008173.mm.1	2,7	0,007802	0,403089	Cassette Exon	0,19
TC0300001014.mm.1	-1,82	Igsf3	PSR0300008172.mm.1	2,42	0,004187	0,36279	Cassette Exon	0,18
TC0300001014.mm.1	-1,82	Igsf3	JUC0300004240.mm.1	2,36	0,003632	0,3566		

TC0300001014.mm.1	-1,82	Igsf3	PSR0300008183.mm.1	2,26	0,001634	0,332913	Cassette Exon	0,16
TC0300001014.mm.1	-1,82	Igsf3	PSR0300008187.mm.1	2,18	0,043629	0,550161	Alternative 3' Acce	0,1
TC0300001014.mm.1	-1,82	Igsf3	PSR0300008178.mm.1	2,04	0,002713	0,349612	Cassette Exon	0,12
TC0100000766.mm.1	-1,5	Sp100; n-R5s215	JUC0100003663.mm.1	2,73	0,026031	0,49949		
TC0100000766.mm.1	-1,5	Sp100; n-R5s215	PSR0100006365.mm.1	2,54	0,020175	0,477086	Cassette Exon	0,13
TC0100000766.mm.1	-1,5	Sp100; n-R5s215	PSR0100006404.mm.1	2,01	0,039636	0,540125	Alternative 5' Donc	0,11
TC1000001745.mm.1	1,36	Grm1	JUC1000006886.mm.1	2,73	0,005823	0,383169		
TC1000001745.mm.1	1,36	Grm1	PSR1000012695.mm.1	-2,01	0,008135	0,405276	Cassette Exon	0,09
TC1000001745.mm.1	1,36	Grm1	JUC1000006894.mm.1	-2,37	0,028997	0,508842		
TC1200001953.mm.1	1,43	Sptb	JUC1200007424.mm.1	2,73	0,029622	0,510558		
TC1200001953.mm.1	1,43	Sptb	JUC1200007430.mm.1	-2,6	0,014976	0,452048		
TC1800000063.mm.1	-1,36	Colec12	JUC1800000214.mm.1	2,73	0,015905	0,456586		
TC0100000546.mm.1	-1,21	Pth2r	JUC0100002590.mm.1	2,73	0,023265	0,49004		
TC1600001629.mm.1	-1,51	4932412D23Rik	JUC1600006890.mm.1	2,73	0,015016	0,452101		
TC0800002856.mm.1	1,26	Smpd3	JUC0800012059.mm.1	2,73	0,014714	0,45037		
TC0800002856.mm.1	1,26	Smpd3	PSR0800022050.mm.1	2,31	0,031552	0,517136		
TC0800002856.mm.1	1,26	Smpd3	PSR0800022052.mm.1	-2,14	0,022511	0,487114		
TC0800002856.mm.1	1,26	Smpd3	JUC0800012061.mm.1	-2,42	0,007474	0,400381		
TC0800002856.mm.1	1,26	Smpd3	JUC0800012063.mm.1	-4,75	0,004034	0,361636		
TC0500003466.mm.1	-1,42	Cyp3a13	JUC0500017198.mm.1	2,73	0,023293	0,49004		
TC0700004193.mm.1	-1,03	Zp2	JUC0700018431.mm.1	2,73	0,025697	0,498173		
TC0700004193.mm.1	-1,03	Zp2	JUC0700018435.mm.1	2,58	0,006837	0,393893		
TC0700001651.mm.1	-1,59	Tead1	JUC0700007090.mm.1	2,73	0,011525	0,428401		
TC0700001651.mm.1	-1,59	Tead1	JUC0700007077.mm.1	2,19	0,009846	0,417839		
TC1100002985.mm.1	-1,23	Gm12295	JUC1100014098.mm.1	2,73	0,018386	0,468484		
TC0100002640.mm.1	11,26	Scg2	PSR0100021473.mm.1	2,72	0,005846	0,383654	Cassette Exon	0,14
TC0100002640.mm.1	11,26	Scg2	PSR0100021472.mm.1	-3,57	0,003791	0,357817	Intron Retention	0,64
TC0100002640.mm.1	11,26	Scg2	PSR0100021475.mm.1	-8,66	0,000779	0,309605	Alternative 5' Donc	0,48
TC0100002640.mm.1	11,26	Scg2	PSR0100021470.mm.1	-29,55	0,002279	0,345991	Alternative 3' Acce	0,48
TC0500000300.mm.1	2,77	Mapre3	JUC0500001315.mm.1	2,72	0,001139	0,317344		
TC0500000300.mm.1	2,77	Mapre3	PSR0500002376.mm.1	-2,15	0,047586	0,558743	Alternative 3' Acce	0,12
TC0500000300.mm.1	2,77	Mapre3	PSR0500002381.mm.1	-2,29	0,023533	0,490947	Cassette Exon	0,37
TC0500000300.mm.1	2,77	Mapre3	PSR0500002393.mm.1	-2,75	0,011068	0,425887	Alternative 5' Donc	0,25
TC0500000300.mm.1	2,77	Mapre3	JUC0500001322.mm.1	-4,59	0,028085	0,506061		
TC0200002042.mm.1	7,67	Chgb	JUC0200008170.mm.1	2,72	0,016151	0,457969		
TC0200002042.mm.1	7,67	Chgb	JUC0200008168.mm.1	-3,13	0,004532	0,368648		
TC0200002042.mm.1	7,67	Chgb	PSR0200016248.mm.1	-10,01	0,001011	0,316361	Cassette Exon	0,28
TC0200002042.mm.1	7,67	Chgb	PSR0200016247.mm.1	-12,27	0,003753	0,357586	Cassette Exon	0,28
TC0100003092.mm.1	1,23	Snrpe	PSR0100024850.mm.1	2,72	0,01645	0,459106	Alternative 3' Acce	0,25
TC1700002214.mm.1	-1,55	Mdfi	PSR1700020933.mm.1	2,72	0,013926	0,446193	Alternative 5' Donc	0,22
TC1700002214.mm.1	-1,55	Mdfi	PSR1700020922.mm.1	2,51	0,01658	0,459685	Alternative 5' Donc	0,23
TC1700002214.mm.1	-1,55	Mdfi	JUC1700011147.mm.1	2,32	0,032873	0,521141		
TC0600003212.mm.1	-1,62	Clec2j	PSR0600025112.mm.1	2,72	0,035585	0,528808	Cassette Exon	0,16
TC1600000760.mm.1	1,23	Dcbld2	JUC1600003504.mm.1	2,72	0,019915	0,475879		
TC1600000760.mm.1	1,23	Dcbld2	PSR1600006600.mm.1	-2,19	0,012318	0,434392	Cassette Exon	0,12
TC0100002335.mm.1	1,3	Dnah7a	JUC0100010644.mm.1	2,72	0,042424	0,547627		
TC0100002335.mm.1	1,3	Dnah7a	PSR0100018877.mm.1	2,59	0,008582	0,40821	Cassette Exon	0,07
TC0100002335.mm.1	1,3	Dnah7a	JUC0100010682.mm.1	-2,44	0,014618	0,449914		

TC1100003776.mm.1	-1,09	Dnajc7	JUC1100018178.mm.1	2,72	0,002476	0,349135		
TC1700002253.mm.1	-1	Tbc1d5; Gm25177	JUC1700011327.mm.1	2,72	0,02658	0,501657		
TC1700001794.mm.1	1,17	Mdga1	JUC1700008914.mm.1	2,72	0,022647	0,487573		
TC1700001794.mm.1	1,17	Mdga1	JUC1700008927.mm.1	-2,01	0,014423	0,448786		
TC1700001794.mm.1	1,17	Mdga1	JUC1700008912.mm.1	-2,58	0,029597	0,510558		
TC0500002277.mm.1	-1,13		JUC0500011429.mm.1	2,72	0,039813	0,540831		
TC0800000581.mm.1	-1,73	1190028D05Rik	JUC0800002171.mm.1	2,72	0,029524	0,510193		
TC0500000385.mm.1	1,09	Htt	JUC0500002064.mm.1	2,72	0,005246	0,377696		
TC0500003219.mm.1	1,27	Camkk2	PSR0500028769.mm.1	2,72	0,014648	0,450098		
TC0500003219.mm.1	1,27	Camkk2	JUC0500015679.mm.1	-2,32	0,035778	0,529143		
TC0700001951.mm.1	-1,07	Dock1	JUC0700009053.mm.1	2,72	0,009946	0,41841		
TC0400000064.mm.1	1	Clvs1	JUC0400000206.mm.1	2,72	0,013806	0,445064		
TC1000002650.mm.1	-1,08	Utp20	JUC1000011053.mm.1	2,72	0,004466	0,367639		
TC0X00000999.mm.1	-1,46	Dmrtc1b	JUC0X00003335.mm.1	2,72	0,016899	0,460797		
TC0900002906.mm.1	-1,44		JUC0900013358.mm.1	2,72	0,005901	0,383835		
TC0600000885.mm.1	3,07	Lrrtm4	PSR0600006705.mm.1	2,71	0,034599	0,52602	Cassette Exon	0,02
TC0600000885.mm.1	3,07	Lrrtm4	PSR0600006709.mm.1	-3,03	0,04227	0,547092	Cassette Exon	0,38
TC0600000885.mm.1	3,07	Lrrtm4	PSR0600006715.mm.1	-3,05	0,001899	0,338712	Alternative 5' Donc	0,31
TC0600000885.mm.1	3,07	Lrrtm4	PSR0600006701.mm.1	-3,89	0,014457	0,44909	Alternative 3' Acce	0,47
TC0600000885.mm.1	3,07	Lrrtm4	JUC0600003382.mm.1	-3,99	0,00659	0,39213		
TC0600000885.mm.1	3,07	Lrrtm4	PSR0600006707.mm.1	-4,6	0,001043	0,316361	Cassette Exon	0,38
TC0600000885.mm.1	3,07	Lrrtm4	PSR0600006703.mm.1	-5,16	0,00037	0,291725	Alternative 3' Acce	0,48
TC0600000885.mm.1	3,07	Lrrtm4	PSR0600006716.mm.1	-5,47	0,000867	0,311909	Cassette Exon	0,41
TC0600000885.mm.1	3,07	Lrrtm4	PSR0600006706.mm.1	-5,49	0,012782	0,437236	Cassette Exon	0,38
TC0600000885.mm.1	3,07	Lrrtm4	PSR0600006702.mm.1	-5,76	0,003614	0,356223	Alternative 3' Acce	0,48
TC0600000885.mm.1	3,07	Lrrtm4	PSR0600006717.mm.1	-5,88	0,017762	0,465425	Cassette Exon	0,28
TC0600000885.mm.1	3,07	Lrrtm4	JUC0600003385.mm.1	-5,94	0,002279	0,345991		
TC0600000885.mm.1	3,07	Lrrtm4	JUC0600003381.mm.1	-5,99	0,003953	0,359922		
TC1500000183.mm.1	2,24	Ctnnd2	JUC1500000883.mm.1	2,71	0,033269	0,522286		
TC1500000183.mm.1	2,24	Ctnnd2	PSR1500001459.mm.1	-2,06	0,049922	0,563902	Cassette Exon	0,13
TC1500000183.mm.1	2,24	Ctnnd2	JUC1500000894.mm.1	-2,09	0,006387	0,389608		
TC1500000183.mm.1	2,24	Ctnnd2	PSR1500001466.mm.1	-2,43	0,039095	0,538395	Cassette Exon	0,21
TC1500000183.mm.1	2,24	Ctnnd2	JUC1500000886.mm.1	-4,62	0,010096	0,418998		
TC0700002773.mm.1	-1	Haus5; Rbm42; Gm21982	PSR0700024760.mm.1	2,71	0,036914	0,5323	Cassette Exon	0,2
TC0700002773.mm.1	-1	Haus5; Rbm42; Gm21982	JUC0700013232.mm.1	2,21	0,039364	0,539381		
TC0700002773.mm.1	-1	Haus5; Rbm42; Gm21982	PSR0700024735.mm.1	2,01	0,03735	0,533573	Cassette Exon	0,1
TC1300002532.mm.1	-1,31	Naip6; Naip7	PSR1300017120.mm.1	2,71	0,020782	0,479424	Cassette Exon	0,19
TC0700000612.mm.1	-1,9	Scgb1b20	PSR0700005318.mm.1	2,71	0,009661	0,417484	Cassette Exon	0,18
TC0600001629.mm.1	-1,1	BC049715	PSR0600012958.mm.1	2,71	0,002829	0,352207	Cassette Exon	0,16
TC1400001445.mm.1	-1,42	Fhit	JUC1400005862.mm.1	2,71	0,012876	0,438024		
TC1400001445.mm.1	-1,42	Fhit	JUC1400005861.mm.1	2,27	0,010308	0,420505		
TC1400001445.mm.1	-1,42	Fhit	JUC1400005850.mm.1	2,02	0,023204	0,490032		
TC1400002833.mm.1	-1,01	Rnf31	JUC1400003359.mm.1	2,71	0,000875	0,311909		
TC1400002833.mm.1	-1,01	Rnf31	JUC1400003371.mm.1	-2,02	0,009885	0,418122		
TC1400002833.mm.1	-1,01	Rnf31	JUC1400003364.mm.1	-2,96	0,000566	0,304044		
TC0200002410.mm.1	-1,05	Src	JUC0200009887.mm.1	2,71	0,000705	0,306015		
TC0500000368.mm.1	-1,14	4930557J02Rik	JUC0500001943.mm.1	2,71	0,03593	0,529399		
TC1000002611.mm.1	1,26	Prdm4	JUC1000010745.mm.1	2,71	0,016067	0,457431		

TC1000002611.mm.1	1,26	Prdm4	JUC1000010738.mm.1	-2,05	0,034483	0,525929		
TC0200001866.mm.1	-1,52	Sema6d	PSR0200014535.mm.1	2,7	0,03219	0,518843	Alternative 3' Acce	0,24
TC0200001866.mm.1	-1,52	Sema6d	JUC0200007278.mm.1	2,16	0,015776	0,455583		
TC0200001866.mm.1	-1,52	Sema6d	JUC0200007290.mm.1	2,1	0,027404	0,503961		
TC0700000932.mm.1	-1,1	Tubgcp5	PSR0700008422.mm.1	2,7	0,007665	0,402377	Cassette Exon	0,14
TC0400002023.mm.1	1,69	Chd5	PSR0400016641.mm.1	2,7	0,013487	0,443178	Cassette Exon	0,1
TC0400002023.mm.1	1,69	Chd5	PSR0400016623.mm.1	2,61	0,009094	0,412827	Cassette Exon	0,13
TC0400002023.mm.1	1,69	Chd5	JUC0400008714.mm.1	2,34	0,023052	0,489379		
TC0400002023.mm.1	1,69	Chd5	JUC0400008728.mm.1	-2,31	0,006821	0,39387		
TC0400002023.mm.1	1,69	Chd5	JUC0400008734.mm.1	-4,05	0,041197	0,544126		
TC0300000485.mm.1	1,18	Mbnl1	JUC0300001774.mm.1	2,7	0,015992	0,456985		
TC0200000235.mm.1	-1,43		JUC0200000385.mm.1	2,7	0,000268	0,28803		
TC0600000672.mm.1	1,81	Ppp1r17	JUC0600002636.mm.1	2,7	0,044382	0,551441		
TC0600000672.mm.1	1,81	Ppp1r17	JUC0600002637.mm.1	-2,66	0,021151	0,480825		
TC0X00001922.mm.1	2,96	Slc9a7	JUC0X00006173.mm.1	2,69	0,024673	0,494711		
TC0X00001922.mm.1	2,96	Slc9a7	PSR0X00012223.mm.1	-2,81	0,034651	0,526169	Cassette Exon	0,14
TC0X00001922.mm.1	2,96	Slc9a7	PSR0X00012210.mm.1	-3,8	0,003658	0,357266	Cassette Exon	0,06
TC0X00001922.mm.1	2,96	Slc9a7	PSR0X00012208.mm.1	-3,94	0,012021	0,432286	Cassette Exon	0,29
TC0X00001922.mm.1	2,96	Slc9a7	PSR0X00012205.mm.1	-4,12	0,003235	0,354243	Alternative 3' Acce	0,36
TC0X00001922.mm.1	2,96	Slc9a7	JUC0X00006170.mm.1	-4,47	0,010567	0,422432		
TC0X00001922.mm.1	2,96	Slc9a7	JUC0X00006182.mm.1	-8,25	0,015727	0,455416		
TC0400001431.mm.1	-1,08	Epha10	PSR0400010793.mm.1	2,69	0,014531	0,449545	Alternative 5' Donc	0,23
TC0700003719.mm.1	-1,08	l7Rn6	JUC0700016580.mm.1	2,69	0,000826	0,311886		
TC0700003719.mm.1	-1,08	l7Rn6	PSR0700031362.mm.1	2,19	0,002243	0,34578	Cassette Exon	0,09
TC1900000297.mm.1	-1,86	C130060C02Rik	PSR1900003086.mm.1	2,69	0,047145	0,557948	Alternative 3' Acce	0,07
TC1200001241.mm.1	1,33	Mark3	JUC1200004824.mm.1	2,69	0,039707	0,540443		
TC0200004487.mm.1	-1,03	Catsper2	JUC0200019603.mm.1	2,69	0,012597	0,435933		
TC1700001447.mm.1	1,2	Map3k4	JUC1700007009.mm.1	2,69	0,033348	0,522553		
TC1800001637.mm.1	-1,73	Slc14a1	JUC1800006556.mm.1	2,69	0,003143	0,353892		
TC1400002872.mm.1	1,15		JUC1400011435.mm.1	2,69	0,005465	0,379939		
TC0200000033.mm.1	1,32	Bend7	JUC0200000118.mm.1	2,69	0,031109	0,515319		
TC0800001400.mm.1	-1,06	Cmip	JUC0800006209.mm.1	2,69	0,001178	0,319796		
TC0600001980.mm.1	9,61	Opn1sw	JUC0600008123.mm.1	2,68	0,018403	0,468484		
TC0600001980.mm.1	9,61	Opn1sw	PSR0600015535.mm.1	-2,52	0,024441	0,49395	Cassette Exon	0,18
TC0600001980.mm.1	9,61	Opn1sw	PSR0600015536.mm.1	-2,57	0,015963	0,456885	Cassette Exon	0,18
TC0600001980.mm.1	9,61	Opn1sw	JUC0600008124.mm.1	-4,96	0,015848	0,456345		
TC0600001980.mm.1	9,61	Opn1sw	JUC0600008125.mm.1	-7,94	0,010606	0,422777		
TC0600001980.mm.1	9,61	Opn1sw	PSR0600015546.mm.1	-8,84	0,022228	0,485896	Alternative 5' Donc	0,48
TC0600001980.mm.1	9,61	Opn1sw	PSR0600015548.mm.1	-9,34	0,014886	0,451777	Alternative 5' Donc	0,48
TC0600001980.mm.1	9,61	Opn1sw	PSR0600015549.mm.1	-9,45	0,020887	0,479589	Cassette Exon	0,41
TC0600001980.mm.1	9,61	Opn1sw	JUC0600008126.mm.1	-12,99	0,014202	0,447698		
TC0400002514.mm.1	-1,01	Fancg	PSR0400020329.mm.1	2,68	0,013838	0,445311	Intron Retention	0,32
TC0400002514.mm.1	-1,01	Fancg	JUC0400010571.mm.1	-2,84	0,012508	0,435429		
TC1600001343.mm.1	1,36	Abcc5	PSR1600011136.mm.1	2,68	0,010137	0,419217	Alternative 5' Donc	0,26
TC1600001343.mm.1	1,36	Abcc5	JUC1600005796.mm.1	2,09	0,039123	0,538475		
TC0200003529.mm.1	-2,25	Neb	JUC0200015129.mm.1	2,68	0,014941	0,451968		
TC0200003529.mm.1	-2,25	Neb	PSR0200030029.mm.1	2,51	0,01202	0,432286	Alternative 3' Acce	0,25
TC0200003529.mm.1	-2,25	Neb	PSR0200030039.mm.1	2,3	0,002365	0,34829	Alternative 3' Acce	0,21

TC0200003529.mm.1	-2,25	Neb	PSR0200030178.mm.1	2,27	0,033323	0,522414	Cassette Exon	0,14
TC0200003529.mm.1	-2,25	Neb	JUC0200015195.mm.1	2,26	0,044618	0,552129		
TC0200005022.mm.1	-1,2	Gm14286	PSR0200043002.mm.1	2,68	0,010147	0,419448	Alternative 5' Donc	0,25
TC0200005022.mm.1	-1,2	Gm14286	JUC0200022183.mm.1	2,67	0,003013	0,353447		
TC0800000661.mm.1	2,58	Spock3	PSR0800004508.mm.1	2,68	0,041739	0,545426	Cassette Exon	0,16
TC0800000661.mm.1	2,58	Spock3	PSR0800004516.mm.1	2,28	0,025793	0,498519	Cassette Exon	0,11
TC0800000661.mm.1	2,58	Spock3	JUC0800002416.mm.1	-2,38	0,003411	0,355242		
TC0800000661.mm.1	2,58	Spock3	PSR0800004498.mm.1	-2,5	0,009624	0,417315	Cassette Exon	0,12
TC0800000661.mm.1	2,58	Spock3	PSR0800004495.mm.1	-2,96	0,030334	0,512885	Cassette Exon	0,19
TC0800000661.mm.1	2,58	Spock3	PSR0800004496.mm.1	-3,04	0,014284	0,448073	Cassette Exon	0,25
TC0800000661.mm.1	2,58	Spock3	PSR0800004511.mm.1	-3,2	0,015487	0,454456	Cassette Exon	0,22
TC0800000661.mm.1	2,58	Spock3	PSR0800004505.mm.1	-3,21	0,001096	0,317328	Cassette Exon	0,13
TC0800000661.mm.1	2,58	Spock3	PSR0800004499.mm.1	-4,39	0,031918	0,518021	Alternative 3' Acce	0,16
TC0800000661.mm.1	2,58	Spock3	JUC0800002417.mm.1	-4,95	0,020734	0,479166		
TC0400000772.mm.1	-2,02	Gm11240	PSR0400005449.mm.1	2,68	0,013152	0,440265	Cassette Exon	0,16
TC0400000772.mm.1	-2,02	Gm11240	PSR0400005452.mm.1	2,43	0,02998	0,51153	Alternative 5' Donc	0,2
TC0400000772.mm.1	-2,02	Gm11240	PSR0400005437.mm.1	2,17	0,002211	0,345614	Alternative 3' Acce	0,14
TC0200002788.mm.1	-1	Gata5os; Gm14318	JUC0200011560.mm.1	2,68	0,004043	0,36172		
TC1500000565.mm.1	-1,1	Gsdmd	JUC1500002285.mm.1	2,68	0,010872	0,424174		
TC1700000813.mm.1	-1,35	Esp6; Esp5; Esp6-esp5	JUC1700004237.mm.1	2,68	0,019638	0,474876		
TC1600000454.mm.1	1,17	Lrch3	JUC1600002235.mm.1	2,68	0,029307	0,509874		
TC0500002002.mm.1	1,42	4921504A21Rik	JUC0500009891.mm.1	2,68	0,005198	0,377015		
TC0700003757.mm.1	-1,03	Gm15413	JUC0700016691.mm.1	2,68	0,029014	0,508842		
TC1800000627.mm.1	10,34	Pde6a	JUC1800002544.mm.1	2,67	0,000475	0,298999		
TC1800000627.mm.1	10,34	Pde6a	PSR1800004574.mm.1	-2,18	0,008654	0,408463	Alternative 5' Donc	0,13
TC1800000627.mm.1	10,34	Pde6a	PSR1800004544.mm.1	-12,08	0,001566	0,331411	Alternative 3' Acce	0,48
TC1800000627.mm.1	10,34	Pde6a	PSR1800004563.mm.1	-12,1	0,000668	0,304088	Cassette Exon	0,36
TC1800000627.mm.1	10,34	Pde6a	JUC1800002563.mm.1	-13,67	0,009962	0,418429		
TC1600001849.mm.1	3,49	Cadm2	PSR1600014725.mm.1	2,67	0,019406	0,473802		
TC1600001849.mm.1	3,49	Cadm2	PSR1600014728.mm.1	2,28	0,000028	0,229631	Cassette Exon	0,12
TC1600001849.mm.1	3,49	Cadm2	JUC1600007634.mm.1	2,02	0,044615	0,552129		
TC1600001849.mm.1	3,49	Cadm2	PSR1600014735.mm.1	-2,05	0,009643	0,417354	Cassette Exon	0,13
TC1600001849.mm.1	3,49	Cadm2	PSR1600014747.mm.1	-2,58	0,030834	0,514305	Cassette Exon	0,19
TC1600001849.mm.1	3,49	Cadm2	PSR1600014749.mm.1	-2,79	0,012819	0,437476	Cassette Exon	0,19
TC1600001849.mm.1	3,49	Cadm2	PSR1600014727.mm.1	-3,17	0,001846	0,337414		
TC1600001849.mm.1	3,49	Cadm2	PSR1600014746.mm.1	-3,59	0,005369	0,378662	Alternative 5' Donc	0,46
TC1600001849.mm.1	3,49	Cadm2	PSR1600014750.mm.1	-3,74	0,005196	0,377015	Cassette Exon	0,19
TC1600001849.mm.1	3,49	Cadm2	PSR1600014744.mm.1	-5,43	0,019978	0,476227	Cassette Exon	0,27
TC1700000496.mm.1	-2,1	Cdkn1a	PSR1700004363.mm.1	2,67	0,039857	0,54093	Alternative 3' Acce	0,27
TC1200002522.mm.1	-1,37	Churc1	PSR1200004888.mm.1	2,67	0,029696	0,510949	Alternative 5' Donc	0,26
TC1200002522.mm.1	-1,37	Churc1	PSR1200004890.mm.1	2,09	0,037394	0,533743	Alternative 5' Donc	0,26
TC0900002375.mm.1	5,5	Cplx3	JUC0900011213.mm.1	2,67	0,013575	0,443703		
TC0900002375.mm.1	5,5	Cplx3	PSR0900019890.mm.1	-2,47	0,013119	0,439856	Alternative 3' Acce	0,24
TC1700002520.mm.1	1,58	Galnt14	PSR1700023089.mm.1	2,67	0,009845	0,417839	Alternative 3' Acce	0,23
TC1700002520.mm.1	1,58	Galnt14	PSR1700023105.mm.1	-2,07	0,011392	0,427549	Alternative 3' Acce	0,2
TC0600000337.mm.1	1,03	Agbl3	JUC0600001270.mm.1	2,67	0,00317	0,354089		
TC0600000337.mm.1	1,03	Agbl3	PSR0600002523.mm.1	-2,5	0,031893	0,51802	Alternative 3' Acce	0,22
TC1400002216.mm.1	-1,27	Neil2	JUC1400009380.mm.1	2,67	0,001598	0,332515		

TC0200002602.mm.1	-1,28	Ptpn1	JUC0200010896.mm.1	2,67	0,027666	0,504707		
TC1100003934.mm.1	1,83	Polg2	JUC1100019279.mm.1	2,67	0,00677	0,393372		
TC0200004199.mm.1	2,73	Gm13810	JUC0200018014.mm.1	2,67	0,004724	0,370888		
TC1200002152.mm.1	1,25	Gtf2a1	JUC1200008242.mm.1	2,67	0,031158	0,515677		
TC1400002738.mm.1	-1,56	Slc15a1	JUC1400011226.mm.1	2,67	0,00342	0,355274		
TC0900000369.mm.1	-1,21	Vsig2	JUC0900001471.mm.1	2,67	0,030388	0,512923		
TC0500001222.mm.1	1,03	Myo1h; Gm13789	JUC0500005729.mm.1	2,67	0,00641	0,389851		
TC0500001222.mm.1	1,03	Myo1h; Gm13789	JUC0500005705.mm.1	-3,14	0,04447	0,551693		
TC0700001875.mm.1	-1,35	Itgam	JUC0700008508.mm.1	2,67	0,00138	0,32572		
TC0700001875.mm.1	-1,35	Itgam	JUC0700008504.mm.1	2,1	0,029703	0,51095		
TC0300001444.mm.1	-1,06	Gbp2b; Gbp5	JUC0300006088.mm.1	2,67	0,028073	0,506002		
TC0300001497.mm.1	1,61	Ttll7	JUC0300006424.mm.1	2,67	0,013518	0,443431		
TC0300001497.mm.1	1,61	Ttll7	JUC0300006417.mm.1	-2,32	0,012589	0,435933		
TC1100003339.mm.1	1,54	Crlf3	JUC1100016049.mm.1	2,67	0,012959	0,438967		
TC1100003339.mm.1	1,54	Crlf3	JUC1100016056.mm.1	-2,47	0,016724	0,460261		
TC0X00001275.mm.1	1,01	Tmem35	JUC0X00003883.mm.1	2,67	0,009679	0,417605		
TC0900002320.mm.1	2,09	Dmxl2	JUC0900010960.mm.1	2,66	0,02063	0,478805		
TC0900002320.mm.1	2,09	Dmxl2	JUC0900010982.mm.1	-2,08	0,046883	0,557414		
TC0900002320.mm.1	2,09	Dmxl2	PSR0900019456.mm.1	-2,27	0,035247	0,52837	Alternative 5' Donc	0,1
TC0900002320.mm.1	2,09	Dmxl2	PSR0900019450.mm.1	-2,5	0,021605	0,483422	Cassette Exon	0,15
TC0900002320.mm.1	2,09	Dmxl2	PSR0900019407.mm.1	-2,84	0,007811	0,403089	Cassette Exon	0,44
TC0900002320.mm.1	2,09	Dmxl2	JUC0900010945.mm.1	-3,99	0,001631	0,332897		
TC0900002320.mm.1	2,09	Dmxl2	JUC0900010946.mm.1	-4,14	0,007244	0,39826		
TC0900002320.mm.1	2,09	Dmxl2	PSR0900019445.mm.1	-4,85	0,005582	0,381035	Alternative 3' Acce	0,49
TC0400001557.mm.1	-2,22	Laptm5	PSR0400012261.mm.1	2,66	0,046297	0,556186	Alternative 5' Donc	0,06
TC0400001557.mm.1	-2,22	Laptm5	PSR0400012246.mm.1	2,36	0,031962	0,518173	Alternative 5' Donc	0,21
TC0400001557.mm.1	-2,22	Laptm5	PSR0400012260.mm.1	2,3	0,047181	0,558037		
TC0700004081.mm.1	1,67	Nrip3	PSR0700033719.mm.1	2,66	0,000063	0,250119	Cassette Exon	0,18
TC0700004081.mm.1	1,67	Nrip3	JUC0700017711.mm.1	-3,02	0,00293	0,352289		
TC0500000815.mm.1	-1,72	Mob1b	PSR0500007189.mm.1	2,66	0,037189	0,533252	Cassette Exon	0,17
TC1800001507.mm.1	1,92	Spire1	JUC1800006175.mm.1	2,66	0,006163	0,387873		
TC1800001507.mm.1	1,92	Spire1	PSR1800011060.mm.1	-2,1	0,003943	0,359922	Cassette Exon	0,11
TC1800001507.mm.1	1,92	Spire1	PSR1800011077.mm.1	-2,25	0,002495	0,349135	Cassette Exon	0,12
TC1800001507.mm.1	1,92	Spire1	PSR1800011061.mm.1	-2,33	0,003341	0,354243	Cassette Exon	0,12
TC0800001317.mm.1	1,04	Hydin	JUC0800005830.mm.1	2,66	0,012942	0,438596		
TC0800001317.mm.1	1,04	Hydin	PSR0800010701.mm.1	-2,14	0,04802	0,559603	Cassette Exon	0,1
TC0800001317.mm.1	1,04	Hydin	JUC0800005862.mm.1	-2,16	0,024513	0,493992		
TC1400000309.mm.1	1,02	Rft1	JUC1400001485.mm.1	2,66	0,036545	0,531355		
TC1200000523.mm.1	1,15	Fam179b	JUC1200002012.mm.1	2,66	0,034055	0,524583		
TC1200000013.mm.1	-1,35	Dtnb	JUC1200000087.mm.1	2,66	0,000184	0,284485		
TC0100002367.mm.1	1,55	BC055402	JUC0100010909.mm.1	2,66	0,047891	0,559408		
TC1500001069.mm.1	1,14	6030408B16Rik	JUC1500004826.mm.1	2,66	0,027325	0,503766		
TC0900001468.mm.1	-1,13	Myl3	JUC0900006857.mm.1	2,66	0,002071	0,342181		
TC0600001252.mm.1	-1,48	Il17rc	JUC0600005045.mm.1	2,66	0,035865	0,529376		
TC0500003297.mm.1	1,06	Stx2	JUC0500016268.mm.1	2,66	0,04292	0,548271		
TC1100000125.mm.1	1,11	Vwc2	JUC1100000707.mm.1	2,66	0,010353	0,421002		
TC0X00002867.mm.1	1,29	Hdx	JUC0X00009244.mm.1	2,66	0,033221	0,522175		
TC0100003878.mm.1	6,84	Mpp4	JUC0100011088.mm.1	2,65	0,001368	0,32572		

TC0100003878.mm.1	6,84	Mpp4	JUC0100011098.mm.1	2,63	0,000196	0,28803		
TC0100003878.mm.1	6,84	Mpp4	JUC0100011100.mm.1	2,57	0,047516	0,558686		
TC0100003878.mm.1	6,84	Mpp4	JUC0100011086.mm.1	2,5	0,009495	0,416578		
TC0100003878.mm.1	6,84	Mpp4	JUC0100011093.mm.1	2,4	0,014501	0,449528		
TC0100003878.mm.1	6,84	Mpp4	JUC0100011106.mm.1	2,22	0,033908	0,52424		
TC0100003878.mm.1	6,84	Mpp4	PSR0100019580.mm.1	-2,16	0,018605	0,469657	Cassette Exon	0,28
TC0100003878.mm.1	6,84	Mpp4	PSR0100019561.mm.1	-2,42	0,026756	0,502096	Alternative 5' Donc	0,3
TC0100003878.mm.1	6,84	Mpp4	JUC0100011105.mm.1	-2,63	0,009157	0,413447		
TC0100003878.mm.1	6,84	Mpp4	PSR0100019587.mm.1	-3,26	0,001727	0,335996	Cassette Exon	0,48
TC0100003878.mm.1	6,84	Mpp4	PSR0100019557.mm.1	-4,19	0,000036	0,238339	Alternative 3' Acce	0,39
TC0100003878.mm.1	6,84	Mpp4	JUC0100011107.mm.1	-4,44	0,000359	0,290838		
TC0100003878.mm.1	6,84	Mpp4	JUC0100011103.mm.1	-4,66	0,000053	0,24627		
TC0100003878.mm.1	6,84	Mpp4	PSR0100019584.mm.1	-4,82	0,010998	0,425491	Alternative 5' Donc	0,41
TC0100003878.mm.1	6,84	Mpp4	PSR0100019570.mm.1	-5	0,001122	0,317328	Alternative 3' Acce	0,48
TC0100003878.mm.1	6,84	Mpp4	JUC0100011097.mm.1	-5,78	0,003089	0,353892		
TC0100003878.mm.1	6,84	Mpp4	JUC0100011108.mm.1	-6,07	0,001519	0,330268		
TC0100003878.mm.1	6,84	Mpp4	PSR0100019590.mm.1	-7,03	0,005036	0,374502	Cassette Exon	0,41
TC0100003878.mm.1	6,84	Mpp4	JUC0100011111.mm.1	-7,37	0,001707	0,335996		
TC0100003878.mm.1	6,84	Mpp4	JUC0100011099.mm.1	-18,62	0,003551	0,356223		
TC0100003878.mm.1	6,84	Mpp4	JUC0100011115.mm.1	-21,16	0,001583	0,331726		
TC0100003878.mm.1	6,84	Mpp4	JUC0100011113.mm.1	-21,71	0,001271	0,322251		
TC0500002719.mm.1	2,9	Adamts3	JUC0500013060.mm.1	2,65	0,000812	0,311886		
TC0500002719.mm.1	2,9	Adamts3	PSR0500023990.mm.1	2,57	0,002856	0,352207	Alternative 5' Donc	0,23
TC0500002719.mm.1	2,9	Adamts3	JUC0500013054.mm.1	-2,09	0,025282	0,497095		
TC0500002719.mm.1	2,9	Adamts3	PSR0500023969.mm.1	-2,52	0,007696	0,402552	Cassette Exon	0,29
TC0500002719.mm.1	2,9	Adamts3	PSR0500023968.mm.1	-2,62	0,004847	0,37168	Cassette Exon	0,18
TC0500002719.mm.1	2,9	Adamts3	JUC0500013062.mm.1	-3,17	0,048399	0,560651		
TC0500002719.mm.1	2,9	Adamts3	JUC0500013048.mm.1	-3,86	0,032198	0,518843		
TC0500002719.mm.1	2,9	Adamts3	JUC0500013047.mm.1	-4,41	0,003585	0,356223		
TC0500002719.mm.1	2,9	Adamts3	JUC0500013058.mm.1	-4,75	0,000114	0,267977		
TC0500002719.mm.1	2,9	Adamts3	JUC0500013051.mm.1	-5	0,040159	0,541622		
TC0500002719.mm.1	2,9	Adamts3	PSR0500023963.mm.1	-5,64	0,001994	0,341091	Alternative 3' Acce	0,47
TC0900002274.mm.1	2,27	Dixdc1	PSR0900018967.mm.1	2,65	0,020126	0,47686	Cassette Exon	0,18
TC0900002274.mm.1	2,27	Dixdc1	JUC0900010687.mm.1	2,54	0,012754	0,436914		
TC0900002274.mm.1	2,27	Dixdc1	PSR0900018971.mm.1	2,43	0,02697	0,502831	Alternative 3' Acce	0,18
TC0900002274.mm.1	2,27	Dixdc1	JUC0900010673.mm.1	-2,25	0,009101	0,412874		
TC0900002274.mm.1	2,27	Dixdc1	PSR0900018963.mm.1	-2,28	0,049272	0,562691	Alternative 5' Donc	0,12
TC0900002274.mm.1	2,27	Dixdc1	PSR0900018976.mm.1	-2,52	0,009597	0,417256	Alternative 5' Donc	0,23
TC0900002274.mm.1	2,27	Dixdc1	JUC0900010690.mm.1	-2,71	0,014133	0,447373		
TC0900002274.mm.1	2,27	Dixdc1	PSR0900018961.mm.1	-3,07	0,011761	0,430069	Cassette Exon	0,14
TC0900002274.mm.1	2,27	Dixdc1	PSR0900018973.mm.1	-3,37	0,008992	0,41198	Cassette Exon	0,22
TC0900002274.mm.1	2,27	Dixdc1	PSR0900018979.mm.1	-4,39	0,007573	0,401638	Alternative 5' Donc	0,41
TC0900002274.mm.1	2,27	Dixdc1	JUC0900010693.mm.1	-4,57	0,021439	0,482126		
TC1900000312.mm.1	1,38	Prune2	JUC1900001624.mm.1	2,65	0,008141	0,405394		
TC1900000312.mm.1	1,38	Prune2	PSR1900003178.mm.1	-2,06	0,023113	0,489756	Alternative 5' Donc	0,23
TC1900000312.mm.1	1,38	Prune2	PSR1900003161.mm.1	-2,06	0,001265	0,322251	Cassette Exon	0,15
TC1900000312.mm.1	1,38	Prune2	JUC1900001626.mm.1	-2,21	0,021076	0,480371		
TC1900000312.mm.1	1,38	Prune2	PSR1900003153.mm.1	-2,3	0,000842	0,311886	Cassette Exon	0,17

TC1900000312.mm.1	1,38	Prune2	PSR1900003152.mm.1	-2,53	0,001924	0,34017	Cassette Exon	0,16
TC1900000312.mm.1	1,38	Prune2	PSR1900003157.mm.1	-2,93	0,016162	0,457969	Cassette Exon	0,22
TC0100001541.mm.1	2,02	Fam78b	PSR0100012696.mm.1	2,65	0,006186	0,387873	Cassette Exon	0,15
TC0100001541.mm.1	2,02	Fam78b	PSR0100012698.mm.1	-2,37	0,006349	0,389463	Alternative 5' Donc	0,22
TC1000001224.mm.1	2,37	Cep290	JUC1000004946.mm.1	2,65	0,008587	0,40821		
TC1000001224.mm.1	2,37	Cep290	JUC1000004981.mm.1	-2,04	0,000601	0,304044		
TC1000001224.mm.1	2,37	Cep290	JUC1000004943.mm.1	-2,12	0,011396	0,427549		
TC1000001224.mm.1	2,37	Cep290	PSR1000009223.mm.1	-2,87	0,016669	0,460057	Cassette Exon	0,17
TC0900001639.mm.1	-1,08	9530059O14Rik	PSR0900013769.mm.1	2,65	0,022506	0,487114	Cassette Exon	0,16
TC0700001402.mm.1	2,46	Pgm2l1	JUC0700006163.mm.1	2,65	0,046184	0,555876		
TC0700001402.mm.1	2,46	Pgm2l1	JUC0700006172.mm.1	2,46	0,010726	0,423745		
TC0700001402.mm.1	2,46	Pgm2l1	PSR0700011889.mm.1	2,32	0,006416	0,389881	Cassette Exon	0,12
TC0700001402.mm.1	2,46	Pgm2l1	PSR0700011904.mm.1	2,15	0,036598	0,531454		
TC0700001402.mm.1	2,46	Pgm2l1	PSR0700011902.mm.1	-2,35	0,002824	0,352207	Alternative 3' Acce	0,04
TC0700001402.mm.1	2,46	Pgm2l1	PSR0700011884.mm.1	-2,48	0,009554	0,41681	Alternative 3' Acce	0,13
TC0700001402.mm.1	2,46	Pgm2l1	JUC0700006165.mm.1	-2,69	0,003937	0,359818		
TC0700001402.mm.1	2,46	Pgm2l1	JUC0700006171.mm.1	-2,82	0,040855	0,543295		
TC0700001402.mm.1	2,46	Pgm2l1	JUC0700006174.mm.1	-4,35	0,006196	0,387873		
TC1400001188.mm.1	-1,17	Scel	JUC1400004998.mm.1	2,65	0,038159	0,535786		
TC0200002945.mm.1	-1,34	Itih2	JUC0200012270.mm.1	2,65	0,02685	0,502454		
TC1100004194.mm.1	1	Cep131; Gm25948; Azi1	JUC1100020960.mm.1	2,65	0,033288	0,522367		
TC1100004194.mm.1	1	Cep131; Gm25948; Azi1	JUC1100020943.mm.1	-2,01	0,021186	0,481033		
TC0300003218.mm.1	-1,15	Itga10	JUC0300003937.mm.1	2,65	0,00286	0,352207		
TC0300003218.mm.1	-1,15	Itga10	JUC0300003925.mm.1	-2,27	0,033806	0,52387		
TC0200004506.mm.1	-1,17	Duox2	JUC0200019767.mm.1	2,65	0,029485	0,510123		
TC0200004506.mm.1	-1,17	Duox2	JUC0200019747.mm.1	-3,35	0,020286	0,477245		
TC0200004506.mm.1	-1,17	Duox2	JUC0200019765.mm.1	-3,53	0,002247	0,34578		
TC0200004815.mm.1	-1,19	9230104L09Rik	JUC0200021267.mm.1	2,65	0,037599	0,534053		
TC0200002067.mm.1	1,29	Plcb4	JUC0200008253.mm.1	2,65	0,047968	0,559552		
TC0200002067.mm.1	1,29	Plcb4	JUC0200008255.mm.1	2,06	0,000253	0,28803		
TC0600001193.mm.1	1,07	Itpr1	JUC0600004721.mm.1	2,65	0,029289	0,509874		
TC1100000464.mm.1	-1,11	Adam19	JUC1100001874.mm.1	2,65	0,036197	0,530037		
TC0300002047.mm.1	1,55	Clrn1	JUC0300008115.mm.1	2,65	0,046948	0,557563		
TC0300001670.mm.1	-3,11		JUC0300006977.mm.1	2,65	0,021171	0,480978		
TC1100001660.mm.1	1,17	Nbr1	JUC1100008084.mm.1	2,65	0,019863	0,475704		
TC1100001660.mm.1	1,17	Nbr1	JUC1100008069.mm.1	-2,06	0,044782	0,552442		
TC1100001660.mm.1	1,17	Nbr1	JUC1100008089.mm.1	-2,31	0,00919	0,413763		
TC0900003260.mm.1	-1,08	Gm19385	JUC0900015447.mm.1	2,65	0,029397	0,509995		
TC0900001921.mm.1	1,02	Dpy19l1	JUC0900009136.mm.1	2,65	0,022996	0,488935		
TC1500000303.mm.1	5,35	Rims2	JUC1500001273.mm.1	2,64	0,013115	0,439759		
TC1500000303.mm.1	5,35	Rims2	PSR1500002190.mm.1	2,43	0,012894	0,438165	Cassette Exon	0,23
TC1500000303.mm.1	5,35	Rims2	JUC1500001267.mm.1	2,14	0,003697	0,357266		
TC1500000303.mm.1	5,35	Rims2	JUC1500001268.mm.1	2,04	0,00963	0,417315		
TC1500000303.mm.1	5,35	Rims2	PSR1500002185.mm.1	2,02	0,022188	0,485756	Cassette Exon	0,04
TC1500000303.mm.1	5,35	Rims2	PSR1500002191.mm.1	2,01	0,006984	0,395722	Cassette Exon	0,14
TC1500000303.mm.1	5,35	Rims2	PSR1500002182.mm.1	-2,09	0,023942	0,49258	Cassette Exon	0,37
TC1500000303.mm.1	5,35	Rims2	JUC1500001265.mm.1	-2,31	0,006684	0,393187		
TC1500000303.mm.1	5,35	Rims2	JUC1500001285.mm.1	-2,39	0,015128	0,452674		

TC1500000303.mm.1	5,35	Rims2	PSR1500002214.mm.1	-2,58	0,010692	0,423294	Alternative 5' Donc	0,25
TC1500000303.mm.1	5,35	Rims2	PSR1500002179.mm.1	-2,98	0,003555	0,356223	Cassette Exon	0,26
TC1500000303.mm.1	5,35	Rims2	PSR1500002209.mm.1	-3,09	0,007429	0,399925	Cassette Exon	0,46
TC1500000303.mm.1	5,35	Rims2	JUC1500001261.mm.1	-3,26	0,001118	0,317328		
TC1500000303.mm.1	5,35	Rims2	PSR1500002183.mm.1	-3,42	0,000542	0,304044	Cassette Exon	0,41
TC1500000303.mm.1	5,35	Rims2	JUC1500001275.mm.1	-3,79	0,004561	0,369273		
TC1500000303.mm.1	5,35	Rims2	JUC1500001276.mm.1	-3,84	0,009989	0,418456		
TC1500000303.mm.1	5,35	Rims2	JUC1500001282.mm.1	-3,87	0,017775	0,465534		
TC1500000303.mm.1	5,35	Rims2	PSR1500002204.mm.1	-3,91	0,003954	0,359922	Cassette Exon	0,24
TC1500000303.mm.1	5,35	Rims2	PSR1500002181.mm.1	-4,28	0,002868	0,352207	Cassette Exon	0,26
TC1500000303.mm.1	5,35	Rims2	PSR1500002208.mm.1	-4,6	0,004102	0,36228		
TC1500000303.mm.1	5,35	Rims2	JUC1500001264.mm.1	-4,67	0,001884	0,338712		
TC1500000303.mm.1	5,35	Rims2	JUC1500001281.mm.1	-4,88	0,004432	0,366894		
TC1500000303.mm.1	5,35	Rims2	JUC1500001287.mm.1	-5,3	0,00389	0,359225		
TC1500000303.mm.1	5,35	Rims2	PSR1500002180.mm.1	-5,47	0,000113	0,267977	Cassette Exon	0,26
TC1500000303.mm.1	5,35	Rims2	JUC1500001290.mm.1	-5,53	0,005165	0,376735		
TC1500000303.mm.1	5,35	Rims2	PSR1500002206.mm.1	-5,86	0,006866	0,394116		
TC1500000303.mm.1	5,35	Rims2	JUC1500001295.mm.1	-6,03	0,008243	0,405976		
TC1500000303.mm.1	5,35	Rims2	JUC1500001262.mm.1	-6,32	0,00044	0,297771		
TC1500000303.mm.1	5,35	Rims2	PSR1500002207.mm.1	-6,34	0,003377	0,354303		
TC1500000303.mm.1	5,35	Rims2	PSR1500002210.mm.1	-6,37	0,000742	0,307808	Cassette Exon	0,44
TC1500000303.mm.1	5,35	Rims2	JUC1500001280.mm.1	-6,61	0,015705	0,455416		
TC1500000303.mm.1	5,35	Rims2	PSR1500002184.mm.1	-7,31	0,00309	0,353892	Cassette Exon	0,27
TC1500000303.mm.1	5,35	Rims2	JUC1500001297.mm.1	-9	0,002845	0,352207		
TC1500000303.mm.1	5,35	Rims2	JUC1500001291.mm.1	-9,21	0,000044	0,24627		
TC1500000303.mm.1	5,35	Rims2	PSR1500002178.mm.1	-11,36	0,002365	0,34829	Cassette Exon	0,31
TC0100003795.mm.1	1,45	Rps6kc1	JUC0100017609.mm.1	2,64	0,034015	0,524484		
TC0100003795.mm.1	1,45	Rps6kc1	PSR0100031028.mm.1	-2,06	0,03764	0,534204	Cassette Exon	0,07
TC0100003795.mm.1	1,45	Rps6kc1	JUC0100017592.mm.1	-2,11	0,002847	0,352207		
TC0100003795.mm.1	1,45	Rps6kc1	JUC0100017606.mm.1	-2,23	0,047353	0,558291		
TC0100003795.mm.1	1,45	Rps6kc1	PSR0100030995.mm.1	-2,52	0,004169	0,3623	Cassette Exon	0,28
TC0100003795.mm.1	1,45	Rps6kc1	JUC0100017593.mm.1	-3,61	0,001281	0,322251		
TC0100003795.mm.1	1,45	Rps6kc1	JUC0100017591.mm.1	-4,95	0,000416	0,297771		
TC0200003811.mm.1	1,42	Sestd1	JUC0200016992.mm.1	2,64	0,004087	0,36228		
TC0200003811.mm.1	1,42	Sestd1	PSR0200032979.mm.1	-2,02	0,007591	0,401712	Intron Retention	0,22
TC0200003811.mm.1	1,42	Sestd1	JUC0200016995.mm.1	-2,03	0,020169	0,477086		
TC1500000781.mm.1	-1,3		PSR1500006126.mm.1	2,64	0,042093	0,546488	Cassette Exon	0,2
TC1500000781.mm.1	-1,3		JUC1500003435.mm.1	2,21	0,006266	0,388719		
TC0700001185.mm.1	1,06	Sh3gl3	PSR0700010448.mm.1	2,64	0,011119	0,426	Cassette Exon	0,15
TC0700001185.mm.1	1,06	Sh3gl3	PSR0700010456.mm.1	-2,02	0,042097	0,546488	Alternative 5' Donc	0,16
TC0700001185.mm.1	1,06	Sh3gl3	JUC0700005374.mm.1	-2,44	0,040298	0,541823		
TC0700002591.mm.1	-1,78	Zfp111	PSR0700022374.mm.1	2,64	0,014139	0,447453	Cassette Exon	0,16
TC0700002591.mm.1	-1,78	Zfp111	PSR0700022366.mm.1	2,57	0,006572	0,39213	Alternative 3' Acce	0,15
TC0700002591.mm.1	-1,78	Zfp111	PSR0700022367.mm.1	2,32	0,000752	0,308185	Alternative 3' Acce	0,15
TC0700002591.mm.1	-1,78	Zfp111	PSR0700022373.mm.1	2,14	0,012597	0,435933	Cassette Exon	0,16
TC1800000684.mm.1	-2,04		PSR1800005062.mm.1	2,64	0,009123	0,413316	Cassette Exon	0,15
TC0200002438.mm.1	1,33	Actr5; Mir3474	PSR0200019818.mm.1	2,64	0,028326	0,506809	Cassette Exon	0,13
TC0200002438.mm.1	1,33	Actr5; Mir3474	JUC0200010050.mm.1	-2,71	0,04468	0,55216		

TC0X00001407.mm.1	-1,24	Tmem164	JUC0X00004413.mm.1	2,64	0,004762	0,371039		
TC0X00001407.mm.1	-1,24	Tmem164	PSR0X00008927.mm.1	2,13	0,0062	0,387873	Cassette Exon	0,11
TC1300002303.mm.1	-1,19	Slc6a19	JUC1300007912.mm.1	2,64	0,00899	0,41198		
TC0100000639.mm.1	1,56	Stk36	JUC0100003059.mm.1	2,64	0,011876	0,431125		
TC0100000639.mm.1	1,56	Stk36	JUC0100003042.mm.1	-2,35	0,00443	0,366832		
TC0100000639.mm.1	1,56	Stk36	JUC0100003065.mm.1	-2,83	0,023712	0,491428		
TC0100003273.mm.1	-1,08	Lamc1	JUC0100015194.mm.1	2,64	0,00011	0,267977		
TC0200001007.mm.1	2,26	Bbs5	JUC0200004035.mm.1	2,64	0,008839	0,410293		
TC0200001007.mm.1	2,26	Bbs5	JUC0200004034.mm.1	2,13	0,00399	0,360753		
TC0200001007.mm.1	2,26	Bbs5	JUC0200004037.mm.1	-2,83	0,025298	0,497227		
TC0200001007.mm.1	2,26	Bbs5	JUC0200004043.mm.1	-2,91	0,040415	0,541998		
TC0400000458.mm.1	-1,28	Glipr2	JUC0400001569.mm.1	2,64	0,01936	0,473603		
TC0400001238.mm.1	-1,02	Mob3c	JUC0400004759.mm.1	2,64	0,044284	0,551322		
TC1000001605.mm.1	1,45	Rnf41	JUC1000006373.mm.1	2,64	0,044798	0,552475		
TC0X00001700.mm.1	-1,43	Arhgap6	JUC0X00005510.mm.1	2,64	0,034842	0,526823		
TC0X00001700.mm.1	-1,43	Arhgap6	JUC0X00005517.mm.1	2,25	0,003154	0,353892		
TC0400001974.mm.1	1,24	Eno1; Eno1b	PSR0400016168.mm.1	2,63	0,042763	0,548097	Alternative 3' Acce	0,26
TC0400001974.mm.1	1,24	Eno1; Eno1b	JUC0400008496.mm.1	-6,06	0,037546	0,534001		
TC0700001922.mm.1	1,03	Acadsb	PSR0700016878.mm.1	2,63	0,043329	0,549369	Alternative 5' Donc	0,26
TC1900001360.mm.1	-1,33	Dkk1	PSR1900012072.mm.1	2,63	0,030734	0,514036	Alternative 5' Donc	0,25
TC1300000500.mm.1	1,3		PSR1300003034.mm.1	2,63	0,023166	0,489896	Intron Retention	0,22
TC1900000099.mm.1	-1,14	Cdca5	PSR1900001143.mm.1	2,63	0,047117	0,557905	Cassette Exon	0,17
TC1200001337.mm.1	1,31	Tmem196	JUC1200005310.mm.1	2,63	0,002245	0,34578		
TC1200001337.mm.1	1,31	Tmem196	PSR1200009593.mm.1	2,09	0,012701	0,43642	Cassette Exon	0,12
TC0500002233.mm.1	1,43	Gm15513	PSR0500020421.mm.1	2,63	0,01124	0,426718	Cassette Exon	0,11
TC0900001022.mm.1	1,72	Hmgcll1	PSR0900008085.mm.1	2,63	0,039421	0,539611	Cassette Exon	0,11
TC1100003604.mm.1	-1,52	Igf2bp1; Mir3063	JUC1100017303.mm.1	2,63	0,008381	0,4065		
TC1100003604.mm.1	-1,52	Igf2bp1; Mir3063	JUC1100017310.mm.1	2,51	0,021432	0,482126		
TC1100003604.mm.1	-1,52	Igf2bp1; Mir3063	PSR1100033051.mm.1	2,01	0,005799	0,383104	Cassette Exon	0,1
TC1400001710.mm.1	-1,01	Gm15512	JUC1400007281.mm.1	2,63	0,024833	0,495408		
TC0100000892.mm.1	-1,37	Scly	JUC0100004336.mm.1	2,63	0,025011	0,496061		
TC0100000892.mm.1	-1,37	Scly	JUC0100004318.mm.1	2,3	0,03205	0,518459		
TC0800001414.mm.1	-1,2	Cdh13	JUC0800006289.mm.1	2,63	0,036763	0,531925		
TC0800001202.mm.1	-1,32	Ces4a	JUC0800005072.mm.1	2,63	0,028771	0,508148		
TC0800002949.mm.1	1,49	Wdr59	JUC0800012446.mm.1	2,63	0,048721	0,561355		
TC0800002949.mm.1	1,49	Wdr59	JUC0800012433.mm.1	-3,36	0,041536	0,544809		
TC0500003514.mm.1	1,16	Iqce	JUC0500017532.mm.1	2,63	0,017111	0,46232		
TC1000002248.mm.1	1,05	H2afy2	JUC1000008680.mm.1	2,63	0,042245	0,547034		
TC0400003324.mm.1	-1,66		JUC0400013806.mm.1	2,63	0,022181	0,485756		
TC0400001854.mm.1	-1,25	Gm13177	JUC0400007992.mm.1	2,63	0,020229	0,477145		
TC1000000656.mm.1	-1,09	Rhobtb1	JUC1000002440.mm.1	2,63	0,008047	0,40434		
TC1000000656.mm.1	-1,09	Rhobtb1	JUC1000002447.mm.1	2,46	0,005487	0,379939		
TC0X00002995.mm.1	1,21	Gla	JUC0X00009450.mm.1	2,63	0,032432	0,519471		
TC0600000231.mm.1	1,69	Ccdc136; Gm26220	JUC0600000751.mm.1	2,62	0,010598	0,422764		
TC0600000231.mm.1	1,69	Ccdc136; Gm26220	JUC0600000758.mm.1	2,53	0,032892	0,521149		
TC0600000231.mm.1	1,69	Ccdc136; Gm26220	PSR0600001522.mm.1	-4,25	0,008437	0,406611	Alternative 5' Donc	0,48
TC0100000588.mm.1	1,89	Vwc2l	PSR0100004738.mm.1	2,62	0,042798	0,548097	Alternative 5' Donc	0,39
TC0100000588.mm.1	1,89	Vwc2l	PSR0100004734.mm.1	2,5	0,004068	0,361956	Alternative 5' Donc	0,12

TC0100000588.mm.1	1,89	Vwc2l	PSR0100004740.mm.1	-2,58	0,018971	0,471398	Cassette Exon	0,12
TC0100000588.mm.1	1,89	Vwc2l	JUC0100002789.mm.1	-2,8	0,014417	0,448786		
TC0100000588.mm.1	1,89	Vwc2l	JUC0100002785.mm.1	-4,98	0,005336	0,378059		
TC1900001078.mm.1	1,18		PSR1900010093.mm.1	2,62	0,039044	0,538264	Alternative 3' Acce	0,26
TC0100003320.mm.1	-1,14	Tor3a	PSR0100027189.mm.1	2,62	0,043787	0,550404	Alternative 5' Donc	0,25
TC0400001766.mm.1	-1,77	Rcc2; Gm25951	PSR0400014556.mm.1	2,62	0,028117	0,506228	Cassette Exon	0,22
TC0400001766.mm.1	-1,77	Rcc2; Gm25951	JUC0400007654.mm.1	2,48	0,005006	0,373851		
TC0400001766.mm.1	-1,77	Rcc2; Gm25951	PSR0400014560.mm.1	2,18	0,006518	0,391382	Cassette Exon	0,17
TC0300002543.mm.1	-1,27	Pde4dip; Mir7225; mmu-n	PSR0300019885.mm.1	2,62	0,046171	0,555838	Cassette Exon	0,17
TC0300002543.mm.1	-1,27	Pde4dip; Mir7225; mmu-n	PSR0300019865.mm.1	2,22	0,009364	0,41516	Alternative 3' Acce	0,09
TC0300002543.mm.1	-1,27	Pde4dip; Mir7225; mmu-n	JUC0300010312.mm.1	-2,08	0,013765	0,444925		
TC0300002543.mm.1	-1,27	Pde4dip; Mir7225; mmu-n	PSR0300019835.mm.1	-2,68	0,008632	0,408463	Alternative 3' Acce	0,21
TC0X00003017.mm.1	-1,55		JUC0X00009578.mm.1	2,62	0,020829	0,47952		
TC0X00003017.mm.1	-1,55		PSR0X00018906.mm.1	2,3	0,016687	0,460085	Cassette Exon	0,19
TC0X00000437.mm.1	1,22	Slc25a14	JUC0X00001475.mm.1	2,62	0,018835	0,470905		
TC0X00000437.mm.1	1,22	Slc25a14	JUC0X00001488.mm.1	2,28	0,009565	0,416927		
TC0X00000437.mm.1	1,22	Slc25a14	PSR0X00002937.mm.1	-2,54	0,007632	0,402047	Cassette Exon	0,18
TC0300002828.mm.1	2,09	Ptbp2	JUC0300011652.mm.1	2,62	0,00131	0,323877		
TC0300002828.mm.1	2,09	Ptbp2	JUC0300011655.mm.1	2,44	0,030064	0,511856		
TC0300002828.mm.1	2,09	Ptbp2	PSR0300022368.mm.1	-2,02	0,046448	0,556553	Cassette Exon	0,09
TC0300002828.mm.1	2,09	Ptbp2	PSR0300022372.mm.1	-2,23	0,006221	0,387912	Alternative 5' Donc	0,17
TC1400002805.mm.1	1,77	Trav6n-6	PSR1400005133.mm.1	2,62	0,000158	0,272178		
TC1400002805.mm.1	1,77	Trav6n-6	PSR1400020710.mm.1	-2,62	0,000158	0,272178	Cassette Exon	0,17
TC1200000670.mm.1	-1,92		PSR1200004603.mm.1	2,62	0,007419	0,399901	Cassette Exon	0,15
TC0200001498.mm.1	-1,32		JUC0200005666.mm.1	2,62	0,01181	0,430377		
TC0800001078.mm.1	1,41	Lpcat2	JUC0800004425.mm.1	2,62	0,036769	0,531966		
TC0800000659.mm.1	-1,4	Ddx60	JUC0800002404.mm.1	2,62	0,048929	0,5618		
TC0800000659.mm.1	-1,4	Ddx60	JUC0800002386.mm.1	2,29	0,007538	0,401261		
TC0700001773.mm.1	-1,15	Lcmt1	JUC0700007950.mm.1	2,62	0,001743	0,335996		
TC0700002061.mm.1	1,19	Muc2; MUC2	JUC0700009722.mm.1	2,62	0,038044	0,535548		
TC0700002061.mm.1	1,19	Muc2; MUC2	JUC0700009759.mm.1	-3,57	0,026416	0,501198		
TC0400003780.mm.1	-1,04	Gm13009	JUC0400016202.mm.1	2,62	0,007183	0,397855		
TC0700003552.mm.1	2,06	Mfge8	JUC0700015822.mm.1	2,61	0,002058	0,341838		
TC0700003552.mm.1	2,06	Mfge8	JUC0700015821.mm.1	2,49	0,046796	0,557041		
TC0700003552.mm.1	2,06	Mfge8	PSR0700029997.mm.1	-2,01	0,019768	0,475035	Alternative 5' Donc	0,27
TC1000000851.mm.1	1,4	Apc2	PSR1000006490.mm.1	2,61	0,044936	0,552848	Cassette Exon	0,15
TC1000000851.mm.1	1,4	Apc2	PSR1000006489.mm.1	2,34	0,003577	0,356223	Cassette Exon	0,16
TC1000000851.mm.1	1,4	Apc2	PSR1000006474.mm.1	2,29	0,026496	0,501252	Cassette Exon	0,12
TC1000000851.mm.1	1,4	Apc2	PSR1000006473.mm.1	-2,03	0,01341	0,442624	Intron Retention	0,25
TC1000000851.mm.1	1,4	Apc2	JUC1000003496.mm.1	-2,25	0,045466	0,554267		
TC1100000956.mm.1	-1,34	Pld2	PSR1100008436.mm.1	2,61	0,048709	0,561355	Alternative 3' Acce	0,24
TC1100004158.mm.1	-1,8	BC100451	PSR1100039535.mm.1	2,61	0,003506	0,355749	Alternative 5' Donc	0,24
TC0400001327.mm.1	-1,88	Ccdc23	PSR0400010036.mm.1	2,61	0,037181	0,533188	Alternative 3' Acce	0,23
TC0500000381.mm.1	-1,02	Add1; Mir7036b; mmu-mi	PSR0500003682.mm.1	2,61	0,017138	0,46232	Cassette Exon	0,18
TC0500000381.mm.1	-1,02	Add1; Mir7036b; mmu-mi	JUC0500002039.mm.1	-2,02	0,030639	0,513746		
TC0400000705.mm.1	1,05	Rgs3	JUC0400002519.mm.1	2,61	0,024131	0,493245		
TC0400000705.mm.1	1,05	Rgs3	PSR0400005018.mm.1	2,34	0,012001	0,432286	Cassette Exon	0,16
TC0400000705.mm.1	1,05	Rgs3	JUC0400002498.mm.1	2,02	0,038064	0,535633		

TC0X00001625.mm.1	-1,15	Sh3kbp1	JUC0X00005146.mm.1	2,61	0,043539	0,549995		
TC0X00001625.mm.1	-1,15	Sh3kbp1	PSR0X00010244.mm.1	2,09	0,04894	0,5618	Cassette Exon	0,16
TC0X00001625.mm.1	-1,15	Sh3kbp1	JUC0X00005170.mm.1	2,06	0,003562	0,356223		
TC0100002073.mm.1	-1,14	Col19a1	PSR0100016873.mm.1	2,61	0,005341	0,378188	Alternative 5' Donc	0,14
TC1500002310.mm.1	2,19	Smug1	PSR1500019150.mm.1	2,61	0,00093	0,313363	Alternative 5' Donc	0,14
TC1500002310.mm.1	2,19	Smug1	JUC1500010929.mm.1	-4,67	0,02332	0,490065		
TC1500002310.mm.1	2,19	Smug1	JUC1500010936.mm.1	-4,8	0,030787	0,514144		
TC0400001933.mm.1	-1,07	Exosc10	JUC0400008315.mm.1	2,61	0,011403	0,427549		
TC0400001933.mm.1	-1,07	Exosc10	PSR0400015839.mm.1	2,1	0,00444	0,367041	Cassette Exon	0,1
TC1700001348.mm.1	1,47	Pisd-ps2	JUC1700006688.mm.1	2,61	0,038005	0,535454		
TC1700001348.mm.1	1,47	Pisd-ps2	PSR1700012254.mm.1	2,44	0,02811	0,506167		
TC1700001348.mm.1	1,47	Pisd-ps2	JUC1700006681.mm.1	-2,19	0,023306	0,49004		
TC1700001348.mm.1	1,47	Pisd-ps2	PSR1700012248.mm.1	-2,45	0,015932	0,456765	Cassette Exon	0,09
TC1200001506.mm.1	-1,09	Gm10476	JUC1200005817.mm.1	2,61	0,006199	0,387873		
TC1900000028.mm.1	-1,76	Tmem134	JUC1900000207.mm.1	2,61	0,049926	0,56393		
TC1900000028.mm.1	-1,76	Tmem134	JUC1900000201.mm.1	2,09	0,043874	0,550542		
TC1600000421.mm.1	-1,28	Mfi2	JUC1600002030.mm.1	2,61	0,031315	0,516204		
TC0500002102.mm.1	1,07	Kmt2c	JUC0500010440.mm.1	2,61	0,033218	0,522175		
TC0500000790.mm.1	1,07	Ythdc1	JUC0500003750.mm.1	2,61	0,026467	0,501252		
TC0600001515.mm.1	-1,02	Clec2h	JUC0600006508.mm.1	2,61	0,025862	0,498741		
TC1100003075.mm.1	-1,18	2810408A11Rik	JUC1100014815.mm.1	2,61	0,007972	0,403853		
TC0300002343.mm.1	-1,53	Slc50a1	JUC0300009393.mm.1	2,61	0,022021	0,485226		
TC1000000726.mm.1	-1,1	Derl3	JUC1000002833.mm.1	2,61	0,01769	0,465018		
TC0900002869.mm.1	7,78	Trpc1	JUC0900013205.mm.1	2,6	0,011545	0,42877		
TC0900002869.mm.1	7,78	Trpc1	JUC0900013204.mm.1	2,58	0,009383	0,415457		
TC0900002869.mm.1	7,78	Trpc1	JUC0900013208.mm.1	-2,59	0,031668	0,517405		
TC0900002869.mm.1	7,78	Trpc1	PSR0900023620.mm.1	-4,07	0,004706	0,370888	Alternative 3' Acce	0,54
TC0900002869.mm.1	7,78	Trpc1	PSR0900023624.mm.1	-4,13	0,004673	0,370181	Alternative 5' Donc	0,47
TC0900002869.mm.1	7,78	Trpc1	PSR0900023622.mm.1	-4,63	0,002335	0,347265	Intron Retention	0,74
TC0900002869.mm.1	7,78	Trpc1	JUC0900013202.mm.1	-5,67	0,002142	0,343679		
TC0900002869.mm.1	7,78	Trpc1	PSR0900023631.mm.1	-9,11	0,004237	0,363295	Cassette Exon	0,27
TC0200004813.mm.1	12,43	Napb	JUC0200021262.mm.1	2,6	0,016312	0,45857		
TC0200004813.mm.1	12,43	Napb	PSR0200041071.mm.1	-2,52	0,015232	0,452839	Alternative 3' Acce	0,14
TC0200004813.mm.1	12,43	Napb	JUC0200021253.mm.1	-4,13	0,031386	0,516345		
TC0200004813.mm.1	12,43	Napb	JUC0200021260.mm.1	-5,16	0,01437	0,448293		
TC0200004813.mm.1	12,43	Napb	PSR0200041081.mm.1	-5,48	0,001211	0,322251	Alternative 3' Acce	0,51
TC0200004813.mm.1	12,43	Napb	PSR0200041087.mm.1	-8,73	0,001885	0,338712	Cassette Exon	0,41
TC0200004813.mm.1	12,43	Napb	PSR0200041084.mm.1	-10,02	0,000974	0,314364	Intron Retention	0,68
TC0200004813.mm.1	12,43	Napb	PSR0200041091.mm.1	-13,74	0,002699	0,349612	Cassette Exon	0,41
TC1800000924.mm.1	1,11	Crem	PSR1800006769.mm.1	2,6	0,013295	0,441721	Alternative 5' Donc	0,26
TC1800000924.mm.1	1,11	Crem	JUC1800003744.mm.1	2,33	0,021508	0,482741		
TC1800000924.mm.1	1,11	Crem	PSR1800006730.mm.1	-3,48	0,01324	0,441329	Alternative 5' Donc	0,37
TC1400002290.mm.1	-1,2	Cdca2	PSR1400017699.mm.1	2,6	0,002046	0,341377	Alternative 5' Donc	0,26
TC0200003707.mm.1	-1,72	Gm16292	PSR0200031752.mm.1	2,6	0,027411	0,503961	Alternative 3' Acce	0,25
TC1600000526.mm.1	1,51	Iqcb1	JUC1600002592.mm.1	2,6	0,007263	0,39826		
TC1600000526.mm.1	1,51	Iqcb1	JUC1600002597.mm.1	-2,77	0,009839	0,417839		
TC1600000526.mm.1	1,51	Iqcb1	PSR1600004924.mm.1	-2,93	0,041243	0,544166	Alternative 5' Donc	0,25
TC0100002724.mm.1	-1,46		PSR0100022037.mm.1	2,6	0,01167	0,429939	Alternative 3' Acce	0,24

TC0700004629.mm.1	-1,27	Setd1a	PSR0700016021.mm.1	2,6	0,017904	0,466466	Alternative 5' Donc	0,23
TC1500000897.mm.1	-1,42	Lrrk2	JUC1500003983.mm.1	2,6	0,018502	0,468972		
TC1500000897.mm.1	-1,42	Lrrk2	PSR1500007121.mm.1	2,56	0,037066	0,532747	Cassette Exon	0,16
TC1500000897.mm.1	-1,42	Lrrk2	PSR1500007101.mm.1	2,51	0,02588	0,498873	Cassette Exon	0,21
TC1500000897.mm.1	-1,42	Lrrk2	JUC1500004016.mm.1	2,26	0,019586	0,474672		
TC1500000897.mm.1	-1,42	Lrrk2	PSR1500007090.mm.1	2,05	0,027144	0,503137	Cassette Exon	0,09
TC1400000988.mm.1	1,26	Pebp4	JUC1400004271.mm.1	2,6	0,036698	0,531756		
TC1400000988.mm.1	1,26	Pebp4	PSR1400007952.mm.1	-2,13	0,018409	0,468484	Cassette Exon	0,18
TC1400000988.mm.1	1,26	Pebp4	PSR1400007950.mm.1	-2,25	0,024516	0,493992	Cassette Exon	0,04
TC1400000988.mm.1	1,26	Pebp4	JUC1400004273.mm.1	-2,76	0,022699	0,487767		
TC1400000988.mm.1	1,26	Pebp4	JUC1400004269.mm.1	-6,19	0,012981	0,439245		
TC1100001095.mm.1	-1,6	Fam57a; 4932415L08RIK	PSR1100009969.mm.1	2,6	0,000998	0,316361	Cassette Exon	0,15
TC1400001934.mm.1	-1,48		JUC1400008010.mm.1	2,6	0,020263	0,477245		
TC0500001853.mm.1	-1,63	B3glct; B3galtl	JUC0500009254.mm.1	2,6	0,018003	0,466939		
TC0500001853.mm.1	-1,63	B3glct; B3galtl	JUC0500009256.mm.1	2,27	0,018348	0,468287		
TC0900000842.mm.1	-1,28	Rasl12	JUC0900003470.mm.1	2,6	0,016566	0,459612		
TC0600001239.mm.1	-1,21	Setd5	JUC0600004890.mm.1	2,6	0,045557	0,554404		
TC0600001239.mm.1	-1,21	Setd5	JUC0600004882.mm.1	2,47	0,041528	0,544763		
TC1000001570.mm.1	-1,02	Myo1a	JUC1000006073.mm.1	2,6	0,031324	0,516204		
TC1000001570.mm.1	-1,02	Myo1a	JUC1000006084.mm.1	-2,22	0,026375	0,501		
TC0700000424.mm.1	-2,18	Tgfb1	PSR0700003315.mm.1	2,59	0,012126	0,432868	Intron Retention	0,33
TC0900000085.mm.1	-2,17		PSR0900000612.mm.1	2,59	0,040317	0,541838	Alternative 5' Donc	0,25
TC1800001544.mm.1	1,65	Dcc	PSR1800011321.mm.1	2,59	0,00127	0,322251	Cassette Exon	0,13
TC1800001544.mm.1	1,65	Dcc	JUC1800006315.mm.1	2,54	0,003202	0,354243		
TC1800001544.mm.1	1,65	Dcc	JUC1800006319.mm.1	2,43	0,025798	0,498529		
TC1800001544.mm.1	1,65	Dcc	PSR1800011340.mm.1	2,32	0,01349	0,44319	Cassette Exon	0,1
TC1800001544.mm.1	1,65	Dcc	JUC1800006329.mm.1	-2,01	0,043049	0,54841		
TC1800001544.mm.1	1,65	Dcc	PSR1800011343.mm.1	-2,03	0,036077	0,529704	Alternative 3' Acce	0,19
TC1800001544.mm.1	1,65	Dcc	PSR1800011325.mm.1	-2,08	0,015746	0,455416	Cassette Exon	0,17
TC1800001544.mm.1	1,65	Dcc	JUC1800006327.mm.1	-2,12	0,00185	0,337414		
TC1800001544.mm.1	1,65	Dcc	PSR1800011324.mm.1	-2,25	0,029216	0,509515	Cassette Exon	0,22
TC1800001544.mm.1	1,65	Dcc	JUC1800006332.mm.1	-2,67	0,036766	0,531925		
TC1800001544.mm.1	1,65	Dcc	PSR1800011315.mm.1	-2,71	0,025714	0,498173	Cassette Exon	0,23
TC1800001544.mm.1	1,65	Dcc	PSR1800011339.mm.1	-2,71	0,005462	0,379939	Alternative 3' Acce	0,22
TC1800001544.mm.1	1,65	Dcc	JUC1800006325.mm.1	-2,96	0,018146	0,467399		
TC1800001544.mm.1	1,65	Dcc	JUC1800006314.mm.1	-4,42	0,012139	0,432868		
TC0100002793.mm.1	-1,09	Rab17	PSR0100022617.mm.1	2,59	0,040043	0,541323	Alternative 5' Donc	0,22
TC1300000496.mm.1	-1,22	Gm20751	PSR1300003004.mm.1	2,59	0,045283	0,553724	Cassette Exon	0,15
TC1300000105.mm.1	-2,07	Gli3	PSR1300000784.mm.1	2,59	0,022883	0,488524	Alternative 3' Acce	0,13
TC1300000105.mm.1	-2,07	Gli3	JUC1300000482.mm.1	2,51	0,007459	0,400326		
TC1300000105.mm.1	-2,07	Gli3	PSR1300000783.mm.1	2,27	0,01855	0,469405	Cassette Exon	0,12
TC0100000824.mm.1	1,37	Atg16l1	PSR0100007018.mm.1	2,59	0,000811	0,311886	Alternative 3' Acce	0,1
TC0100000824.mm.1	1,37	Atg16l1	PSR0100007019.mm.1	2,09	0,008879	0,410619	Cassette Exon	0,1
TC0700000811.mm.1	1,37	Kcnc3	PSR0700006735.mm.1	2,59	0,034139	0,524882	Cassette Exon	0,05
TC0700000811.mm.1	1,37	Kcnc3	JUC0700003346.mm.1	-2,07	0,031325	0,516208		
TC0700000811.mm.1	1,37	Kcnc3	JUC0700003343.mm.1	-3,12	0,024629	0,494656		
TC1400002357.mm.1	-1,56		JUC1400010183.mm.1	2,59	0,023251	0,490032		
TC0200004481.mm.1	1,12	Zscan29	JUC0200019472.mm.1	2,59	0,007669	0,402377		

TC1300000569.mm.1	-2,12	Ogn	JUC1300001811.mm.1	2,59	0,02024	0,477187		
TC1300000569.mm.1	-2,12	Ogn	JUC1300001807.mm.1	2,03	0,021319	0,481723		
TC0100000946.mm.1	-1,1	Ing5	JUC0100004627.mm.1	2,59	0,04348	0,549896		
TC1800001748.mm.1	1,39	Apbb3	JUC1800004917.mm.1	2,59	0,034052	0,524583		
TC1900000523.mm.1	1,56	Btaf1	JUC1900002458.mm.1	2,59	0,016595	0,459691		
TC1900000523.mm.1	1,56	Btaf1	JUC1900002485.mm.1	-2,19	0,042904	0,548218		
TC1700000385.mm.1	1,26	Narfl; Mir6966; mmu-mir-	JUC1700001758.mm.1	2,59	0,002443	0,349135		
TC0700000017.mm.1	-2,07	Gm15929	JUC0700000102.mm.1	2,59	0,008902	0,410728		
TC0700000017.mm.1	-2,07	Gm15929	JUC0700000101.mm.1	2,47	0,000104	0,263684		
TC0700003603.mm.1	-1,17		JUC0700016179.mm.1	2,59	0,012956	0,438944		
TC1100001987.mm.1	-1,76		JUC1100009815.mm.1	2,59	0,017382	0,463704		
TC0400004000.mm.1	-1	Zfp933	JUC0400017194.mm.1	2,59	0,001285	0,322384		
TC0X00002470.mm.1	-1,09	Mpp1	JUC0X00008036.mm.1	2,59	0,020995	0,480143		
TC0400002987.mm.1	2,39	Slc24a2	PSR0400024189.mm.1	2,58	0,021204	0,48113	Alternative 3' Acce	0,15
TC0400002987.mm.1	2,39	Slc24a2	JUC0400012622.mm.1	-2,08	0,039935	0,540973		
TC0400002987.mm.1	2,39	Slc24a2	JUC0400012624.mm.1	-2,18	0,025209	0,49669		
TC0400002987.mm.1	2,39	Slc24a2	PSR0400024206.mm.1	-2,63	0,019219	0,472971	Alternative 3' Acce	0,29
TC0400002987.mm.1	2,39	Slc24a2	PSR0400024211.mm.1	-2,64	0,037782	0,534693	Alternative 5' Donc	0,24
TC0400002987.mm.1	2,39	Slc24a2	PSR0400024209.mm.1	-2,88	0,01085	0,424161	Alternative 3' Acce	0,29
TC0400002987.mm.1	2,39	Slc24a2	PSR0400024194.mm.1	-3,08	0,014387	0,44839	Cassette Exon	0,2
TC0400002987.mm.1	2,39	Slc24a2	PSR0400024218.mm.1	-3,15	0,012096	0,43255	Cassette Exon	0,22
TC0400002987.mm.1	2,39	Slc24a2	JUC0400012633.mm.1	-3,24	0,036087	0,529712		
TC0400002987.mm.1	2,39	Slc24a2	JUC0400012636.mm.1	-3,77	0,014719	0,45037		
TC0400002987.mm.1	2,39	Slc24a2	PSR0400024205.mm.1	-4,2	0,012806	0,437316	Alternative 3' Acce	0,29
TC0400002987.mm.1	2,39	Slc24a2	JUC0400012632.mm.1	-4,3	0,015664	0,455416		
TC0700000102.mm.1	-2,03	Mir5620; Gm23572	PSR0700000973.mm.1	2,58	0,005887	0,383835	Alternative 3' Acce	0,25
TC0900000494.mm.1	3,43		PSR0900003232.mm.1	2,58	0,003883	0,359196	Cassette Exon	0,21
TC0900000494.mm.1	3,43		JUC0900001646.mm.1	2,48	0,035893	0,529385		
TC0900000494.mm.1	3,43		PSR0900003231.mm.1	-2,58	0,003883	0,359196	Cassette Exon	0,08
TC1500000603.mm.1	1,65	Kifc2	JUC1500002515.mm.1	2,58	0,012836	0,437683		
TC1500000603.mm.1	1,65	Kifc2	JUC1500002509.mm.1	-2,11	0,01168	0,429939		
TC1500000603.mm.1	1,65	Kifc2	PSR1500004352.mm.1	-2,14	0,027501	0,504313	Cassette Exon	0,19
TC1900000913.mm.1	-1,09	Ssh3	PSR1900007953.mm.1	2,58	0,03812	0,535709	Cassette Exon	0,19
TC0200004482.mm.1	1,26	Trp53bp1	JUC0200019497.mm.1	2,58	0,008038	0,40434		
TC0200004482.mm.1	1,26	Trp53bp1	JUC0200019489.mm.1	2,34	0,00719	0,397927		
TC0200004482.mm.1	1,26	Trp53bp1	PSR0200037909.mm.1	2,13	0,001042	0,316361	Cassette Exon	0,17
TC0200004482.mm.1	1,26	Trp53bp1	PSR0200037908.mm.1	2,11	0,000664	0,304044	Cassette Exon	0,17
TC0500000214.mm.1	1,75	Asic3	PSR0500001590.mm.1	2,58	0,00726	0,39826	Cassette Exon	0,16
TC0500000214.mm.1	1,75	Asic3	PSR0500001591.mm.1	2,46	0,020504	0,478221	Cassette Exon	0,15
TC0500000214.mm.1	1,75	Asic3	JUC0500000921.mm.1	2,03	0,04338	0,549544		
TC0600002904.mm.1	1,44	Srgap3	JUC0600011587.mm.1	2,58	0,040149	0,541609		
TC0600002904.mm.1	1,44	Srgap3	PSR0600022303.mm.1	-2,01	0,025522	0,497712	Alternative 3' Acce	0,16
TC0600002904.mm.1	1,44	Srgap3	JUC0600011589.mm.1	-2,11	0,029569	0,510456		
TC0600002904.mm.1	1,44	Srgap3	JUC0600011577.mm.1	-2,29	0,046342	0,556272		
TC1600001548.mm.1	1,48	Pdia5	PSR1600012709.mm.1	2,58	0,014043	0,44654	Cassette Exon	0,15
TC1600001548.mm.1	1,48	Pdia5	PSR1600012708.mm.1	2,27	0,046761	0,556925	Cassette Exon	0,16
TC1600001548.mm.1	1,48	Pdia5	PSR1600012707.mm.1	2,2	0,046943	0,557545	Cassette Exon	0,13
TC0100003308.mm.1	1,84	Lhx4; Pdzrn4	PSR0100026935.mm.1	2,58	0,003992	0,360753	Cassette Exon	0,15

TC0100003308.mm.1	1,84	Lhx4; Pdzrn4	PSR0100026943.mm.1	-2,26	0,009961	0,418429	Cassette Exon	0,12
TC0200004236.mm.1	-3,99	Gm10799	PSR0200035651.mm.1	2,58	0,049981	0,564078	Cassette Exon	0,15
TC0200004236.mm.1	-3,99	Gm10799	PSR0200035652.mm.1	-2,58	0,049981	0,564078	Cassette Exon	0,15
TC1900000409.mm.1	-1,26	Smarca2	PSR1900003764.mm.1	2,58	0,006094	0,386848	Cassette Exon	0,12
TC0600000319.mm.1	1,52	Lrguk	PSR0600002328.mm.1	2,58	0,005724	0,382767	Cassette Exon	0,1
TC1400002871.mm.1	1,07	Gm4285	JUC1400011434.mm.1	2,58	0,014546	0,449562		
TC0200005090.mm.1	-1,47	Oser1	JUC0200022388.mm.1	2,58	0,01434	0,448173		
TC1800000682.mm.1	-1,12	Sec11c	JUC1800002819.mm.1	2,58	0,014972	0,452048		
TC1800001541.mm.1	1,3	Poli	JUC1800006294.mm.1	2,58	0,043388	0,549583		
TC1800001541.mm.1	1,3	Poli	JUC1800006300.mm.1	-4,07	0,001913	0,339584		
TC1500001933.mm.1	1,14	Polr3h	JUC1500008609.mm.1	2,58	0,005334	0,378059		
TC0500002139.mm.1	1,4	Lmbr1	JUC0500010603.mm.1	2,58	0,00929	0,414799		
TC0500000877.mm.1	-1,72	Stbd1	JUC0500004179.mm.1	2,58	0,017536	0,464105		
TC0600002543.mm.1	-1,65	Mrpl19	JUC0600010225.mm.1	2,58	0,039228	0,53882		
TC0600003122.mm.1	1,08	Leprel2	JUC0600012662.mm.1	2,58	0,017215	0,462603		
TC0300002870.mm.1	-1,78	Myoz2	JUC0300011856.mm.1	2,58	0,000387	0,295505		
TC0300002636.mm.1	-1,1	Magi3	JUC0300010712.mm.1	2,58	0,020195	0,477145		
TC1000000555.mm.1	-1,77	Gm26947	JUC1000002079.mm.1	2,58	0,024856	0,495511		
TC1000000048.mm.1	-1,07	Lats1	JUC1000000199.mm.1	2,58	0,003014	0,353447		
TC1000001010.mm.1	1,23	A230046K03Rik	JUC1000004053.mm.1	2,58	0,00194	0,340349		
TC1000001010.mm.1	1,23	A230046K03Rik	JUC1000004031.mm.1	-2,02	0,034004	0,524385		
TC0100003832.mm.1	3,42	Syt14	PSR0100031328.mm.1	2,57	0,007538	0,401261	Cassette Exon	0,34
TC0100003832.mm.1	3,42	Syt14	JUC0100017774.mm.1	-2,67	0,016837	0,4607		
TC0100003832.mm.1	3,42	Syt14	JUC0100017780.mm.1	-2,83	0,045106	0,553206		
TC0100003832.mm.1	3,42	Syt14	PSR0100031331.mm.1	-3,14	0,002134	0,343402	Cassette Exon	0,24
TC0100003832.mm.1	3,42	Syt14	JUC0100017781.mm.1	-3,89	0,010629	0,422841		
TC0100003832.mm.1	3,42	Syt14	JUC0100017773.mm.1	-8,93	0,000429	0,297771		
TC0600002325.mm.1	-1,39	Crhr2	PSR0600018188.mm.1	2,57	0,009434	0,416082	Alternative 5' Donc	0,25
TC0600002325.mm.1	-1,39	Crhr2	PSR0600018181.mm.1	2,2	0,034208	0,525106	Alternative 5' Donc	0,21
TC0600002325.mm.1	-1,39	Crhr2	PSR0600018165.mm.1	-2,18	0,013206	0,440851	Cassette Exon	0,12
TC0900000826.mm.1	1,01	Dpp8	PSR0900006268.mm.1	2,57	0,023919	0,492324	Alternative 5' Donc	0,25
TC0400002095.mm.1	-1,46	Mxra8	PSR0400017489.mm.1	2,57	0,023526	0,490947	Cassette Exon	0,23
TC0400002095.mm.1	-1,46	Mxra8	JUC0400009180.mm.1	2,57	0,028349	0,506843		
TC0100002326.mm.1	-2,89		PSR0100018726.mm.1	2,57	0,034685	0,526294	Alternative 3' Acce	0,22
TC1500000489.mm.1	1,22	Wisp1	PSR1500003504.mm.1	2,57	0,021514	0,482767	Alternative 5' Donc	0,21
TC1500000489.mm.1	1,22	Wisp1	PSR1500003501.mm.1	2,05	0,005156	0,376575	Cassette Exon	0,11
TC1500000489.mm.1	1,22	Wisp1	PSR1500003495.mm.1	-2,06	0,012295	0,434277	Cassette Exon	0,11
TC0300001909.mm.1	-1,39	Pgrmc2	PSR0300014646.mm.1	2,57	0,009196	0,413763	Cassette Exon	0,15
TC0300001909.mm.1	-1,39	Pgrmc2	PSR0300014643.mm.1	2,12	0,034298	0,525445	Cassette Exon	0,19
TC0700003938.mm.1	-1,89	Trim12a	PSR0700032899.mm.1	2,57	0,001236	0,322251	Cassette Exon	0,19
TC1100002334.mm.1	1,11	Ehbp1	PSR1100022149.mm.1	2,57	0,026602	0,501778	Cassette Exon	0,19
TC1100002334.mm.1	1,11	Ehbp1	JUC1100011530.mm.1	2,31	0,003121	0,353892		
TC0300000733.mm.1	-1,32	2810403A07Rik	PSR0300005289.mm.1	2,57	0,046733	0,556856	Alternative 5' Donc	0,17
TC0900000596.mm.1	4,55		PSR0900004224.mm.1	2,57	0,002815	0,352207	Cassette Exon	0,14
TC0900000596.mm.1	4,55		PSR0900004223.mm.1	-2,57	0,002815	0,352207	Cassette Exon	0,15
TC0200002056.mm.1	1,32	Plcb1	PSR0200016337.mm.1	2,57	0,018207	0,467586	Cassette Exon	0,13
TC0200002056.mm.1	1,32	Plcb1	JUC0200008233.mm.1	2,31	0,005902	0,383835		
TC0200002056.mm.1	1,32	Plcb1	JUC0200008225.mm.1	-2,31	0,012054	0,432426		

TC1400001303.mm.1	-1,5	Mbnl2	JUC1400005206.mm.1	2,57	0,000102	0,262646		
TC1200001715.mm.1	1,12	Strn3	JUC1200006531.mm.1	2,57	0,010153	0,419557		
TC1700001989.mm.1	1,23	Abcf1	JUC1700010143.mm.1	2,57	0,037289	0,533573		
TC0100002864.mm.1	1,01	Ppip5k2	JUC0100013192.mm.1	2,57	0,004682	0,370323		
TC1700001798.mm.1	1,17	Btbd9	JUC1700008936.mm.1	2,57	0,029544	0,510303		
TC1500001807.mm.1	-2,31	Apol7a	JUC1500008013.mm.1	2,57	0,009192	0,413763		
TC0500000357.mm.1	-1,07	Uvssa	JUC0500001840.mm.1	2,57	0,017409	0,463768		
TC0500000357.mm.1	-1,07	Uvssa	JUC0500001855.mm.1	2,14	0,018264	0,46797		
TC0500003379.mm.1	-1,22	Eln	JUC0500016609.mm.1	2,57	0,037586	0,534053		
TC0500003379.mm.1	-1,22	Eln	JUC0500016631.mm.1	-2,1	0,04755	0,558707		
TC0500003379.mm.1	-1,22	Eln	JUC0500016634.mm.1	-2,3	0,011161	0,426295		
TC0500003379.mm.1	-1,22	Eln	JUC0500016607.mm.1	-2,35	0,033644	0,523479		
TC0700001689.mm.1	1,98	Xylt1	JUC0700007251.mm.1	2,57	0,01996	0,476104		
TC0700001689.mm.1	1,98	Xylt1	JUC0700007249.mm.1	-2,05	0,039029	0,538193		
TC0700001689.mm.1	1,98	Xylt1	JUC0700007257.mm.1	-2,4	0,003012	0,353447		
TC0700001689.mm.1	1,98	Xylt1	JUC0700007252.mm.1	-2,78	0,00333	0,354243		
TC0400000183.mm.1	1,47	Pnisr; Sfrs18	JUC0400000581.mm.1	2,57	0,011163	0,426295		
TC0400000183.mm.1	1,47	Pnisr; Sfrs18	JUC0400000593.mm.1	-2,77	0,009315	0,414883		
TC0400000183.mm.1	1,47	Pnisr; Sfrs18	JUC0400000596.mm.1	-3,45	0,001061	0,316361		
TC1100002032.mm.1	-1,17	Gcgr	JUC1100010223.mm.1	2,57	0,020775	0,479352		
TC0900003246.mm.1	-1,26	Scn11a	JUC0900015396.mm.1	2,57	0,040296	0,541823		
TC0400003701.mm.1	-1,52	Gm13257	JUC0400015794.mm.1	2,57	0,006456	0,390284		
TC0900002772.mm.1	1,11	Ibtk	JUC0900012833.mm.1	2,57	0,024501	0,493992		
TC0900002772.mm.1	1,11	Ibtk	JUC0900012841.mm.1	2,22	0,015605	0,455078		
TC0600001458.mm.1	-2,01	Scnn1a	PSR0600011919.mm.1	2,56	0,011873	0,431125	Alternative 3' Acce	0,19
TC0600001458.mm.1	-2,01	Scnn1a	PSR0600011916.mm.1	2,48	0,02832	0,506741	Alternative 3' Acce	0,11
TC1800000201.mm.1	1,99	Fhod3	PSR1800001503.mm.1	2,56	0,021666	0,483789	Cassette Exon	0,18
TC1800000201.mm.1	1,99	Fhod3	JUC1800000867.mm.1	2,47	0,01769	0,465018		
TC1800000201.mm.1	1,99	Fhod3	JUC1800000874.mm.1	2,37	0,034101	0,524833		
TC1800000201.mm.1	1,99	Fhod3	PSR1800001497.mm.1	-2,03	0,005543	0,380603	Cassette Exon	0,19
TC1800000201.mm.1	1,99	Fhod3	PSR1800001500.mm.1	-2,03	0,049673	0,563531		
TC1800000201.mm.1	1,99	Fhod3	PSR1800001495.mm.1	-2,14	0,000741	0,307808	Cassette Exon	0,12
TC1800000201.mm.1	1,99	Fhod3	JUC1800000872.mm.1	-2,2	0,046566	0,556761		
TC1800000201.mm.1	1,99	Fhod3	PSR1800001498.mm.1	-2,27	0,003393	0,354841	Cassette Exon	0,19
TC1800000201.mm.1	1,99	Fhod3	PSR1800001501.mm.1	-2,43	0,027525	0,504313	Cassette Exon	0,06
TC1300002549.mm.1	-2,73	4922502H24Rik	PSR1300017389.mm.1	2,56	0,020731	0,479166	Cassette Exon	0,16
TC0900000254.mm.1	-1	Thyn1	PSR0900001890.mm.1	2,56	0,036595	0,531454	Cassette Exon	0,15
TC0X00003432.mm.1	1,72	Mecp2	PSR0X00015436.mm.1	2,56	0,004389	0,366156	Cassette Exon	0,15
TC0X00003432.mm.1	1,72	Mecp2	PSR0X00015450.mm.1	-2,01	0,042589	0,548047	Alternative 5' Donc	0,04
TC0X00003432.mm.1	1,72	Mecp2	JUC0X00007871.mm.1	-3,05	0,010737	0,423772		
TC1400001674.mm.1	-1,08	Dnah1	PSR1400012903.mm.1	2,56	0,031854	0,517902	Cassette Exon	0,14
TC1400001674.mm.1	-1,08	Dnah1	JUC1400007062.mm.1	2,01	0,03787	0,534873		
TC1400001674.mm.1	-1,08	Dnah1	JUC1400007005.mm.1	-3,52	0,043992	0,550776		
TC0800002683.mm.1	1,05	Rpgrip1l	JUC0800011164.mm.1	2,56	0,008927	0,410972		
TC0800002683.mm.1	1,05	Rpgrip1l	JUC0800011143.mm.1	2,49	0,01414	0,447464		
TC0800002683.mm.1	1,05	Rpgrip1l	PSR0800020544.mm.1	-2,27	0,040149	0,541609	Cassette Exon	0,12
TC0800002683.mm.1	1,05	Rpgrip1l	JUC0800011166.mm.1	-2,63	0,026065	0,499627		
TC0200003303.mm.1	-1,08	Lcn2	PSR0200028090.mm.1	2,56	0,008912	0,410728	Alternative 5' Donc	0,05

TC0300001070.mm.1	1,15	Phtf1	JUC0300004490.mm.1	2,56	0,040091	0,541412		
TC0300001070.mm.1	1,15	Phtf1	JUC0300004496.mm.1	2,25	0,01513	0,452674		
TC1200002579.mm.1	-1,77	Ighv11-1	JUC1200009972.mm.1	2,56	0,000589	0,304044		
TC0200004737.mm.1	-1	Kif16b	JUC0200021022.mm.1	2,56	0,03176	0,517489		
TC0200001158.mm.1	1,17	Itga4	JUC0200004749.mm.1	2,56	0,015724	0,455416		
TC0700000543.mm.1	-1,33		JUC0700002333.mm.1	2,56	0,03032	0,51274		
TC0600002741.mm.1	-1,2	Mrps25	JUC0600011062.mm.1	2,56	0,041338	0,544348		
TC1000002846.mm.1	-1,27	Nav3	JUC1000011861.mm.1	2,56	0,010299	0,420394		
TC1000002846.mm.1	-1,27	Nav3	JUC1000011865.mm.1	2,15	0,026529	0,501411		
TC1000002846.mm.1	-1,27	Nav3	JUC1000011895.mm.1	-3,22	0,000743	0,307808		
TC1100001969.mm.1	-1,48	Tmc8	JUC1100009744.mm.1	2,56	0,046166	0,555838		
TC0400001449.mm.1	-1,5	Csf3r	JUC0400005731.mm.1	2,56	0,001882	0,338712		
TC1700000676.mm.1	2,31	Atp6v1g2	PSR1700006894.mm.1	2,55	0,028985	0,508806		
TC1700000676.mm.1	2,31	Atp6v1g2	PSR1700006876.mm.1	-2,28	0,032953	0,52122	Cassette Exon	0,17
TC1700000676.mm.1	2,31	Atp6v1g2	PSR1700006884.mm.1	-2,55	0,015146	0,452699	Cassette Exon	0,16
TC1700000676.mm.1	2,31	Atp6v1g2	PSR1700006881.mm.1	-3,14	0,035666	0,528966	Cassette Exon	0,36
TC1700000676.mm.1	2,31	Atp6v1g2	PSR1700006880.mm.1	-3,16	0,002757	0,350151	Intron Retention	0,19
TC1700000676.mm.1	2,31	Atp6v1g2	PSR1700006886.mm.1	-3,29	0,000525	0,304044	Alternative 3' Acce	0,22
TC1700000676.mm.1	2,31	Atp6v1g2	PSR1700006877.mm.1	-3,31	0,012283	0,434168	Cassette Exon	0,21
TC1700000676.mm.1	2,31	Atp6v1g2	JUC1700003782.mm.1	-4,46	0,002139	0,343541		
TC0300002071.mm.1	1,58	Plch1	JUC0300008200.mm.1	2,55	0,024834	0,495411		
TC0300002071.mm.1	1,58	Plch1	PSR0300015655.mm.1	-2,07	0,006191	0,387873	Alternative 5' Donc	0,09
TC0300002071.mm.1	1,58	Plch1	PSR0300015661.mm.1	-2,25	0,024284	0,493611	Alternative 5' Donc	0,15
TC0300002071.mm.1	1,58	Plch1	PSR0300015623.mm.1	-2,43	0,017676	0,464879	Alternative 5' Donc	0,17
TC0300002071.mm.1	1,58	Plch1	JUC0300008191.mm.1	-2,45	0,00452	0,368648		
TC0300002071.mm.1	1,58	Plch1	PSR0300015657.mm.1	-3,05	0,046302	0,556186	Alternative 3' Acce	0,28
TC0300002071.mm.1	1,58	Plch1	JUC0300008217.mm.1	-3,36	0,003845	0,358723		
TC1200002021.mm.1	-1,75		PSR1200013935.mm.1	2,55	0,001788	0,336311	Alternative 3' Acce	0,24
TC0200000914.mm.1	1,61	Gm13583	PSR0200007325.mm.1	2,55	0,036888	0,532257	Alternative 5' Donc	0,2
TC0400004139.mm.1	1,8	Prkcz	JUC0400018069.mm.1	2,55	0,015998	0,45702		
TC0400004139.mm.1	1,8	Prkcz	PSR0400034663.mm.1	-2,02	0,022023	0,485237	Alternative 5' Donc	0,2
TC0400004139.mm.1	1,8	Prkcz	PSR0400034681.mm.1	-2,04	0,009061	0,412827	Cassette Exon	0,12
TC0400004139.mm.1	1,8	Prkcz	PSR0400034684.mm.1	-2,1	0,001558	0,331094	Cassette Exon	0,12
TC0400004139.mm.1	1,8	Prkcz	PSR0400034678.mm.1	-2,42	0,028934	0,508625	Intron Retention	0,2
TC0400004139.mm.1	1,8	Prkcz	PSR0400034666.mm.1	-2,77	0,002962	0,352501	Cassette Exon	0,15
TC0100000610.mm.1	-2,52	Igfbp2	PSR0100004913.mm.1	2,55	0,00715	0,39769	Cassette Exon	0,16
TC0100000610.mm.1	-2,52	Igfbp2	PSR0100004914.mm.1	2,09	0,026076	0,499646	Alternative 3' Acce	0,15
TC0900001845.mm.1	1,28	Olfm2	PSR0900015399.mm.1	2,55	0,005758	0,383101	Cassette Exon	0,16
TC0900001656.mm.1	-1,78	Exosc7	PSR0900013932.mm.1	2,55	0,040503	0,542217	Cassette Exon	0,12
TC0900001656.mm.1	-1,78	Exosc7	PSR0900013939.mm.1	2,3	0,040364	0,541959	Cassette Exon	0,15
TC0900001656.mm.1	-1,78	Exosc7	PSR0900013929.mm.1	2,25	0,045752	0,554863	Cassette Exon	0,13
TC0600001848.mm.1	1,51	A430035B10Rik; LOC1008	PSR0600014589.mm.1	2,55	0,040757	0,543082	Cassette Exon	0,14
TC0700000558.mm.1	1,42	Alkbh6; Gm24757	PSR0700004793.mm.1	2,55	0,017963	0,466771	Cassette Exon	0,13
TC1400002867.mm.1	-1,08	Xpo4	JUC1400009018.mm.1	2,55	0,005804	0,383104		
TC1100003921.mm.1	-1,13	Scn4a	JUC1100019211.mm.1	2,55	0,002219	0,345773		
TC0300000979.mm.1	-1,06	Hmgcs2	JUC0300004092.mm.1	2,55	0,010812	0,424055		
TC1300001938.mm.1	-1,04	A330048O09Rik	JUC1300006206.mm.1	2,55	0,008159	0,40551		
TC0100002242.mm.1	-1,16	Rfx8	JUC0100010238.mm.1	2,55	0,029416	0,509995		

TC0100002242.mm.1	-1,16 Rfx8	JUC0100010242.mm.1	-2,01	0,047513	0,558686		
TC1700001428.mm.1	-2,18 Qk	JUC1700006957.mm.1	2,55	0,02069	0,478999		
TC1900001213.mm.1	-1,23 Vps13a	JUC1900006027.mm.1	2,55	0,011885	0,431306		
TC1900001516.mm.1	1,19 Mms19	JUC1900007400.mm.1	2,55	0,021799	0,484444		
TC0100000771.mm.1	-1,34 Cab39	JUC0100003688.mm.1	2,55	0,000591	0,304044		
TC0200001369.mm.1	-1,12 Mybpc3	JUC0200005290.mm.1	2,55	0,00165	0,333988		
TC0200001380.mm.1	1,01 Arhgap1	JUC0200005481.mm.1	2,55	0,023307	0,49004		
TC1600000680.mm.1	1,02 Gm15591	JUC1600003126.mm.1	2,55	0,018429	0,468623		
TC0700002982.mm.1	1 Scaf1	JUC0700014306.mm.1	2,55	0,018016	0,466939		
TC0500003658.mm.1	-1,33 Gm15997	JUC0500018235.mm.1	2,55	0,031475	0,516922		
TC0700001991.mm.1	1,05 Inpp5a	JUC0700009266.mm.1	2,55	0,003257	0,354243		
TC0700001991.mm.1	1,05 Inpp5a	JUC0700009280.mm.1	-2,45	0,005171	0,376797		
TC1000003160.mm.1	1,01 Cdk2	JUC1000013109.mm.1	2,55	0,007447	0,40013		
TC0900002389.mm.1	-1,42 Pml	JUC0900011328.mm.1	2,55	0,016372	0,458693		
TC0400000849.mm.1	1,57 Cntln	PSR0400005773.mm.1	2,54	0,012075	0,43255	Cassette Exon	0,24
TC0400000849.mm.1	1,57 Cntln	JUC0400002943.mm.1	2,06	0,031682	0,517423		
TC0700003075.mm.1	-1,73 E2f8	PSR0700028050.mm.1	2,54	0,048129	0,559938	Cassette Exon	0,15
TC1000001687.mm.1	-1,03 Mtrf1l	JUC1000006672.mm.1	2,54	0,029543	0,510303		
TC1000001687.mm.1	-1,03 Mtrf1l	PSR1000012300.mm.1	2,26	0,022189	0,485756	Alternative 5' Donc	0,15
TC1000002373.mm.1	-1,14	PSR1000016907.mm.1	2,54	0,009987	0,418456	Cassette Exon	0,14
TC0200004201.mm.1	-1,31 Rag1; B230118H07Rik	JUC0200018015.mm.1	2,54	0,004794	0,371408		
TC0200004201.mm.1	-1,31 Rag1; B230118H07Rik	PSR0200035265.mm.1	2,32	0,006458	0,390284	Cassette Exon	0,1
TC0200004201.mm.1	-1,31 Rag1; B230118H07Rik	PSR0200035264.mm.1	2,11	0,004784	0,371208	Cassette Exon	0,13
TC0200004201.mm.1	-1,31 Rag1; B230118H07Rik	PSR0200035239.mm.1	2,1	0,01554	0,454617	Alternative 3' Acce	0,11
TC1100001208.mm.1	1,29 Cdk5r1	PSR1100011374.mm.1	2,54	0,00404	0,361702	Alternative 5' Donc	0,07
TC0900000776.mm.1	-1,51 Tle3	PSR0900005794.mm.1	2,54	0,040745	0,543028	Cassette Exon	0,06
TC0200004646.mm.1	-1 Adam33	JUC0200020666.mm.1	2,54	0,002693	0,349612		
TC1700002553.mm.1	-1,28 Strn	JUC1700012523.mm.1	2,54	0,019265	0,473074		
TC0100003202.mm.1	-1,04 Cdc73	JUC0100014797.mm.1	2,54	0,033648	0,523479		
TC1700001987.mm.1	-1,36 Mrps18b	JUC1700010123.mm.1	2,54	0,012028	0,432286		
TC1900000990.mm.1	1,2 Dpf2	JUC1900004955.mm.1	2,54	0,025566	0,497808		
TC1900000990.mm.1	1,2 Dpf2	JUC1900004946.mm.1	-2,1	0,007871	0,403545		
TC0100001787.mm.1	1,16 Eprs	JUC0100008451.mm.1	2,54	0,018322	0,468127		
TC0100001787.mm.1	1,16 Eprs	JUC0100008466.mm.1	-2,05	0,001956	0,340561		
TC1500001789.mm.1	-1,06 Recql4	JUC1500007920.mm.1	2,54	0,040991	0,543571		
TC1500001789.mm.1	-1,06 Recql4	JUC1500007918.mm.1	2,04	0,017509	0,463964		
TC1500001790.mm.1	1,18 C030006K11Rik; Lrrc24	JUC1500007941.mm.1	2,54	0,011444	0,427741		
TC1500001790.mm.1	1,18 C030006K11Rik; Lrrc24	JUC1500007940.mm.1	2,38	0,017794	0,465621		
TC1500001790.mm.1	1,18 C030006K11Rik; Lrrc24	JUC1500007939.mm.1	2,17	0,049597	0,563406		
TC0500001229.mm.1	1,16	JUC0500005780.mm.1	2,54	0,006162	0,387873		
TC0600001813.mm.1	-1,47 Slc25a13	JUC0600007522.mm.1	2,54	0,018165	0,467441		
TC0600001931.mm.1	1,17 Cadps2	JUC0600007916.mm.1	2,54	0,012327	0,434392		
TC0600001931.mm.1	1,17 Cadps2	JUC0600007921.mm.1	-2,06	0,01467	0,450098		
TC0600001931.mm.1	1,17 Cadps2	JUC0600007915.mm.1	-2,28	0,046599	0,55677		
TC0600001931.mm.1	1,17 Cadps2	JUC0600007952.mm.1	-3,05	0,028418	0,507154		
TC0600001931.mm.1	1,17 Cadps2	JUC0600007956.mm.1	-3,2	0,001971	0,340622		
TC1100001520.mm.1	-2,09 Copz2	JUC1100007274.mm.1	2,54	0,009719	0,417826		
TC0500001103.mm.1	14,39 Pde6b	JUC0500005113.mm.1	2,53	0,020081	0,476581		

TC0500001103.mm.1	14,39	Pde6b	JUC0500005122.mm.1	2,38	0,005276	0,378059		
TC0500001103.mm.1	14,39	Pde6b	PSR0500009393.mm.1	-2,01	0,01206	0,432426	Intron Retention	0,23
TC0500001103.mm.1	14,39	Pde6b	PSR0500009382.mm.1	-2,02	0,000884	0,312542	Cassette Exon	0,09
TC0500001103.mm.1	14,39	Pde6b	PSR0500009388.mm.1	-2,36	0,030054	0,511847	Intron Retention	0,31
TC0500001103.mm.1	14,39	Pde6b	PSR0500009407.mm.1	-2,92	0,016926	0,460872	Cassette Exon	0,22
TC0500001103.mm.1	14,39	Pde6b	PSR0500009406.mm.1	-3,23	0,005603	0,381115	Cassette Exon	0,22
TC0500001103.mm.1	14,39	Pde6b	PSR0500009405.mm.1	-3,32	0,023747	0,491646	Cassette Exon	0,22
TC0500001103.mm.1	14,39	Pde6b	PSR0500009399.mm.1	-3,33	0,021671	0,483837	Intron Retention	0,47
TC0500001103.mm.1	14,39	Pde6b	JUC0500005121.mm.1	-3,37	0,040417	0,541998		
TC0500001103.mm.1	14,39	Pde6b	PSR0500009397.mm.1	-3,97	0,008821	0,410155	Intron Retention	0,74
TC0500001103.mm.1	14,39	Pde6b	PSR0500009409.mm.1	-5,98	0,003306	0,354243	Cassette Exon	0,22
TC0500001103.mm.1	14,39	Pde6b	JUC0500005120.mm.1	-10,54	0,001867	0,337974		
TC0500001103.mm.1	14,39	Pde6b	PSR0500009376.mm.1	-17,99	0,001697	0,335395	Alternative 3' Acce	0,47
TC0500001103.mm.1	14,39	Pde6b	JUC0500005109.mm.1	-22,9	0,000074	0,251428		
TC0500001103.mm.1	14,39	Pde6b	JUC0500005123.mm.1	-23,48	0,003092	0,353892		
TC1400000913.mm.1	5,43	Rp1l1	PSR1400007301.mm.1	2,53	0,001228	0,322251	Cassette Exon	0,11
TC1400000913.mm.1	5,43	Rp1l1	JUC1400003920.mm.1	-5,09	0,018036	0,467135		
TC1400000913.mm.1	5,43	Rp1l1	PSR1400007300.mm.1	-5,54	0,001822	0,336896	Alternative 3' Acce	0,48
TC1100001407.mm.1	4,56	Car10	PSR1100013135.mm.1	2,53	0,008804	0,409934	Cassette Exon	0,01
TC1100001407.mm.1	4,56	Car10	JUC1100006887.mm.1	2,21	0,025207	0,49668		
TC1100001407.mm.1	4,56	Car10	PSR1100013133.mm.1	2,04	0,004053	0,361816	Cassette Exon	0,16
TC1100001407.mm.1	4,56	Car10	PSR1100013138.mm.1	-2,11	0,000101	0,262646	Cassette Exon	0,17
TC1100001407.mm.1	4,56	Car10	JUC1100006896.mm.1	-2,25	0,019644	0,474876		
TC1100001407.mm.1	4,56	Car10	PSR1100013142.mm.1	-2,28	0,008316	0,406313	Cassette Exon	0,1
TC1100001407.mm.1	4,56	Car10	PSR1100013145.mm.1	-2,3	0,000219	0,28803	Cassette Exon	0,18
TC1100001407.mm.1	4,56	Car10	PSR1100013148.mm.1	-2,36	0,023678	0,491379	Cassette Exon	0,18
TC1100001407.mm.1	4,56	Car10	JUC1100006895.mm.1	-2,51	0,029164	0,509333		
TC1100001407.mm.1	4,56	Car10	PSR1100013127.mm.1	-2,55	0,01895	0,471398	Cassette Exon	0,2
TC1100001407.mm.1	4,56	Car10	PSR1100013143.mm.1	-2,55	0,000261	0,28803	Cassette Exon	0,15
TC1100001407.mm.1	4,56	Car10	PSR1100013144.mm.1	-2,64	0,00884	0,410293	Cassette Exon	0,28
TC1100001407.mm.1	4,56	Car10	PSR1100013134.mm.1	-3	0,023133	0,489756	Alternative 5' Donc	0,33
TC1100001407.mm.1	4,56	Car10	PSR1100013137.mm.1	-3,06	0,00452	0,368648	Cassette Exon	0,26
TC1100001407.mm.1	4,56	Car10	PSR1100013140.mm.1	-3,81	0,008655	0,408463	Alternative 5' Donc	0,34
TC1100001407.mm.1	4,56	Car10	JUC1100006890.mm.1	-4,89	0,009786	0,417839		
TC1100001407.mm.1	4,56	Car10	JUC1100006889.mm.1	-5,77	0,003457	0,355628		
TC1100001407.mm.1	4,56	Car10	PSR1100013124.mm.1	-6,53	0,001258	0,322251	Cassette Exon	0,33
TC1100003193.mm.1	1,3	Rap1gap2	JUC1100015383.mm.1	2,53	0,048185	0,560088		
TC1100003193.mm.1	1,3	Rap1gap2	PSR1100029435.mm.1	-2,26	0,038066	0,535633	Cassette Exon	0,1
TC1100003193.mm.1	1,3	Rap1gap2	PSR1100029429.mm.1	-2,71	0,039844	0,540922	Alternative 5' Donc	0,32
TC1600000772.mm.1	-1,68	Mina	PSR1600006697.mm.1	2,53	0,011147	0,426226	Alternative 5' Donc	0,26
TC1600000772.mm.1	-1,68	Mina	JUC1600003561.mm.1	-12,06	0,009927	0,418378		
TC1700000418.mm.1	-2,02	Atp6v0e	PSR1700003566.mm.1	2,53	0,017824	0,465913	Alternative 5' Donc	0,26
TC0300001797.mm.1	-1,53	Zmat3	PSR0300013928.mm.1	2,53	0,009018	0,412541	Alternative 3' Acce	0,24
TC0400002549.mm.1	-1,38	Gne	PSR0400020812.mm.1	2,53	0,044106	0,550916	Alternative 3' Acce	0,24
TC1400002032.mm.1	1,69	Mettl3	PSR1400015325.mm.1	2,53	0,048613	0,561169	Alternative 5' Donc	0,24
TC1200000988.mm.1	-1,12	Tdp1	PSR1200006884.mm.1	2,53	0,044443	0,551619	Alternative 3' Acce	0,21
TC0400000018.mm.1	-1,03	Chchd7	PSR0400000154.mm.1	2,53	0,014595	0,449914	Cassette Exon	0,12
TC0400000018.mm.1	-1,03	Chchd7	PSR0400000137.mm.1	2,2	0,047858	0,559373	Alternative 5' Donc	0,18

TC0500001914.mm.1	-1,35	Steap1	PSR0500017668.mm.1	2,53	0,046459	0,556553	Cassette Exon	0,14
TC1000002154.mm.1	-1,03	Ros1	PSR1000015109.mm.1	2,53	0,0269	0,502518	Cassette Exon	0,13
TC1400001036.mm.1	-1,14	Sucla2	JUC1400004454.mm.1	2,53	0,037622	0,534172		
TC1200001420.mm.1	-1,42	Gen1	JUC1200005527.mm.1	2,53	0,029638	0,510607		
TC0300000763.mm.1	-1,16	Shc1	JUC0300003047.mm.1	2,53	0,010035	0,41878		
TC1500001044.mm.1	-1,13	Higd1c; Mettl7a2Higd1c;	JUC1500004676.mm.1	2,53	0,024546	0,494088		
TC0800001797.mm.1	-1,34	Angpt2	JUC0800007829.mm.1	2,53	0,02123	0,481319		
TC0800000091.mm.1	1,14	Ing1	JUC0800000436.mm.1	2,53	0,009874	0,417993		
TC0500000607.mm.1	1,36	Wdr19	JUC0500003009.mm.1	2,53	0,014487	0,449376		
TC0500000607.mm.1	1,36	Wdr19	JUC0500003005.mm.1	2,06	0,036313	0,530532		
TC1000002464.mm.1	1,07	2610008E11Rik	JUC1000009872.mm.1	2,53	0,049705	0,563531		
TC1100003035.mm.1	-1,09	Cntrob	JUC1100014424.mm.1	2,53	0,008595	0,408294		
TC1100003035.mm.1	-1,09	Cntrob	JUC1100014427.mm.1	2,03	0,005645	0,381706		
TC1100001580.mm.1	1,09	Thra	JUC1100007649.mm.1	2,53	0,023954	0,4926		
TC1100001580.mm.1	1,09	Thra	JUC1100007638.mm.1	2,28	0,013026	0,439354		
TC1100002225.mm.1	-1,55	Ddc	JUC1100011279.mm.1	2,53	0,034154	0,524919		
TC0400003440.mm.1	1,01	Nfyc	JUC0400014576.mm.1	2,53	0,018016	0,466939		
TC0500000299.mm.1	1,84	Dpysl5	PSR0500002359.mm.1	2,52	0,000055	0,24627	Alternative 3' Acce	0,13
TC0500000299.mm.1	1,84	Dpysl5	PSR0500002354.mm.1	-2,27	0,028676	0,508017	Cassette Exon	0,12
TC0500000299.mm.1	1,84	Dpysl5	JUC0500001301.mm.1	-2,3	0,001466	0,329023		
TC0500000299.mm.1	1,84	Dpysl5	PSR0500002357.mm.1	-2,4	0,004121	0,3623	Cassette Exon	0,04
TC0500000299.mm.1	1,84	Dpysl5	PSR0500002356.mm.1	-2,61	0,000222	0,28803	Cassette Exon	0,21
TC0500000299.mm.1	1,84	Dpysl5	PSR0500002355.mm.1	-2,64	0,021691	0,484029	Alternative 5' Donc	0,06
TC0500000299.mm.1	1,84	Dpysl5	PSR0500002368.mm.1	-3,51	0,016237	0,458312	Alternative 3' Acce	0,35
TC0700004243.mm.1	-2,24	Gm27040; Gm26974	PSR0700035341.mm.1	2,52	0,019163	0,472719	Cassette Exon	0,14
TC0700004243.mm.1	-2,24	Gm27040; Gm26974	PSR0700035321.mm.1	2,47	0,007932	0,403819		
TC0700004243.mm.1	-2,24	Gm27040; Gm26974	PSR0700035322.mm.1	2,32	0,008772	0,40982		
TC0700004243.mm.1	-2,24	Gm27040; Gm26974	PSR0700035335.mm.1	2,22	0,009087	0,412827	Intron Retention	0,23
TC0700004243.mm.1	-2,24	Gm27040; Gm26974	JUC0700018640.mm.1	2,03	0,039778	0,540568		
TC0100003645.mm.1	1,81	Hnrnpu; Gm16586; AI503	JUC0100016960.mm.1	2,52	0,038299	0,536106		
TC0100003645.mm.1	1,81	Hnrnpu; Gm16586; AI503	PSR0100029858.mm.1	2,09	0,023034	0,489272	Cassette Exon	0,19
TC0100003645.mm.1	1,81	Hnrnpu; Gm16586; AI503	PSR0100029862.mm.1	2,07	0,024994	0,49605	Cassette Exon	0,17
TC0500003296.mm.1	1,24	Rimbp2	JUC0500016250.mm.1	2,52	0,029927	0,511499		
TC0500003296.mm.1	1,24	Rimbp2	PSR0500029806.mm.1	2,37	0,012779	0,437233	Cassette Exon	0,17
TC0500003296.mm.1	1,24	Rimbp2	PSR0500029805.mm.1	2,3	0,021436	0,482126	Cassette Exon	0,17
TC0500003296.mm.1	1,24	Rimbp2	JUC0500016237.mm.1	2,16	0,017035	0,461729		
TC0500003296.mm.1	1,24	Rimbp2	JUC0500016251.mm.1	-2,24	0,009484	0,416578		
TC0500002586.mm.1	1,25	Fryl	PSR0500022961.mm.1	2,52	0,048215	0,560209	Cassette Exon	0,15
TC0500002586.mm.1	1,25	Fryl	JUC0500012487.mm.1	-2,99	0,01975	0,474932		
TC1800001620.mm.1	1,52	Katnal2	PSR1800011659.mm.1	2,52	0,040035	0,54131	Cassette Exon	0,14
TC1800001620.mm.1	1,52	Katnal2	JUC1800006484.mm.1	-2,18	0,014867	0,451641		
TC1600001722.mm.1	-1,91	Alcam	PSR1600014013.mm.1	2,52	0,031017	0,514914	Alternative 5' Donc	0,13
TC0200003188.mm.1	1,11	Snapc4	JUC0200013454.mm.1	2,52	0,047381	0,558302		
TC0200003188.mm.1	1,11	Snapc4	JUC0200013447.mm.1	-2,81	0,018479	0,468729		
TC1200000856.mm.1	1,21	Vash1	JUC1200003535.mm.1	2,52	0,001053	0,316361		
TC1200000856.mm.1	1,21	Vash1	JUC1200003538.mm.1	-2,01	0,019086	0,472208		
TC1200001885.mm.1	-1,13	Pygl	JUC1200007180.mm.1	2,52	0,013538	0,44358		
TC1200001885.mm.1	-1,13	Pygl	JUC1200007171.mm.1	2,22	0,046668	0,556812		

TC0200005218.mm.1	-1,91	Nfatc2	JUC0200023025.mm.1	2,52	0,042242	0,547034		
TC1900001766.mm.1	-1,02	Zfpl1	JUC1900005053.mm.1	2,52	0,045797	0,554863		
TC1900001053.mm.1	-1,13	Slc22a19	JUC1900005361.mm.1	2,52	0,011278	0,426842		
TC0900000625.mm.1	-1,13	4933407I05Rik	JUC0900002352.mm.1	2,52	0,015439	0,454266		
TC0900000625.mm.1	-1,13	4933407I05Rik	JUC0900002347.mm.1	2,04	0,027938	0,505612		
TC0500000836.mm.1	-1,22	Afm	JUC0500004029.mm.1	2,52	0,015771	0,455535		
TC0700000382.mm.1	-1,17	Smg9	JUC0700001339.mm.1	2,52	0,017461	0,463886		
TC0600000768.mm.1	-1,11	Prdm5	JUC0600002898.mm.1	2,52	0,01912	0,472394		
TC0600000768.mm.1	-1,11	Prdm5	JUC0600002896.mm.1	2,36	0,009192	0,413763		
TC0700002090.mm.1	-1,35	Kcnq1	JUC0700010034.mm.1	2,52	0,031919	0,518021		
TC0700002090.mm.1	-1,35	Kcnq1	JUC0700010023.mm.1	-2,33	0,046203	0,555959		
TC1100002835.mm.1	-1,03	Zfp39	JUC1100013289.mm.1	2,52	0,040993	0,543577		
TC0X00000053.mm.1	-1,28	Dgkk	JUC0X00000045.mm.1	2,52	0,036405	0,530878		
TC0X00000053.mm.1	-1,28	Dgkk	JUC0X00000057.mm.1	2,22	0,027581	0,504482		
TC0X00001612.mm.1	-1,07	Rps6ka3	JUC0X00005073.mm.1	2,52	0,031605	0,517338		
TC0X00001612.mm.1	-1,07	Rps6ka3	JUC0X00005077.mm.1	-2,41	0,043424	0,549656		
TC0400003885.mm.1	-1,27	Fblim1	JUC0400016773.mm.1	2,52	0,002489	0,349135		
TC0800000765.mm.1	3,46	Rab3a	JUC0800002883.mm.1	2,51	0,003266	0,354243		
TC0800000765.mm.1	3,46	Rab3a	PSR0800005464.mm.1	-2,88	0,049645	0,563498	Alternative 5' Donc	0,29
TC0800000765.mm.1	3,46	Rab3a	PSR0800005452.mm.1	-2,92	0,028344	0,506843	Alternative 3' Acce	0,3
TC0800000765.mm.1	3,46	Rab3a	JUC0800002882.mm.1	-2,95	0,023944	0,492582		
TC0800000765.mm.1	3,46	Rab3a	PSR0800005451.mm.1	-3,18	0,009701	0,417746	Alternative 3' Acce	0,3
TC0800000765.mm.1	3,46	Rab3a	PSR0800005461.mm.1	-3,33	0,009167	0,413447	Intron Retention	0,66
TC0800000765.mm.1	3,46	Rab3a	PSR0800005456.mm.1	-3,43	0,013378	0,442312	Intron Retention	0,59
TC0800000765.mm.1	3,46	Rab3a	PSR0800005462.mm.1	-4,05	0,00778	0,402902	Intron Retention	0,66
TC0800000765.mm.1	3,46	Rab3a	PSR0800005459.mm.1	-4,62	0,024198	0,493294	Intron Retention	0,75
TC0800000765.mm.1	3,46	Rab3a	PSR0800005453.mm.1	-6,97	0,005828	0,383235	Alternative 3' Acce	0,3
TC0800000765.mm.1	3,46	Rab3a	JUC0800002887.mm.1	-12,82	0,000586	0,304044		
TC1300001859.mm.1	2,73	Elovl2	JUC1300005957.mm.1	2,51	0,007039	0,396189		
TC1300001859.mm.1	2,73	Elovl2	JUC1300005961.mm.1	-2,66	0,037635	0,534189		
TC1300001859.mm.1	2,73	Elovl2	PSR1300011611.mm.1	-3,23	0,026536	0,501411	Cassette Exon	0,28
TC1900001323.mm.1	-2,71	Glis3	PSR1900011785.mm.1	2,51	0,011873	0,431125	Alternative 5' Donc	0,22
TC1900001323.mm.1	-2,71	Glis3	PSR1900011791.mm.1	2,28	0,015398	0,453964	Cassette Exon	0,06
TC1900001323.mm.1	-2,71	Glis3	JUC1900006460.mm.1	2,27	0,039035	0,53823		
TC1900001323.mm.1	-2,71	Glis3	PSR1900011772.mm.1	2,24	0,017492	0,463964	Alternative 3' Acce	0,16
TC1300000267.mm.1	-1,24	Slc17a1	PSR1300001553.mm.1	2,51	0,004492	0,368077	Cassette Exon	0,21
TC1300000267.mm.1	-1,24	Slc17a1	JUC1300000761.mm.1	2,47	0,010337	0,420958		
TC0100003328.mm.1	1,94	Rasal2	JUC0100015608.mm.1	2,51	0,036044	0,52965		
TC0100003328.mm.1	1,94	Rasal2	PSR0100027304.mm.1	-2,09	0,046704	0,556856	Alternative 3' Acce	0,17
TC0700002105.mm.1	1,25	Shank2	PSR0700019018.mm.1	2,51	0,039134	0,538491	Cassette Exon	0,14
TC0700002105.mm.1	1,25	Shank2	JUC0700010104.mm.1	2,34	0,040415	0,541998		
TC0700002105.mm.1	1,25	Shank2	PSR0700019004.mm.1	2,12	0,018116	0,467161	Cassette Exon	0,16
TC1700002178.mm.1	1,23	Cul9	PSR1700020496.mm.1	2,51	0,003219	0,354243	Cassette Exon	0,13
TC1700002178.mm.1	1,23	Cul9	JUC1700010898.mm.1	2,31	0,010526	0,422224		
TC1600001584.mm.1	-2,16	Eaf2	JUC1600006736.mm.1	2,51	0,01395	0,446239		
TC1600001584.mm.1	-2,16	Eaf2	PSR1600012957.mm.1	2,29	0,030727	0,514036	Cassette Exon	0,09
TC0200003807.mm.1	-1,8	Ttn	JUC0200016902.mm.1	2,51	0,038305	0,536106		
TC0200003807.mm.1	-1,8	Ttn	JUC0200016920.mm.1	-2,77	0,001114	0,317328		

TC1300001098.mm.1	1,79	Fam169a	JUC1300003651.mm.1	2,51	0,028532	0,507444		
TC0500002010.mm.1	1,27	Ptpn12	JUC0500009936.mm.1	2,51	0,028024	0,505851		
TC0500000310.mm.1	1,33	Atraid	JUC0500001454.mm.1	2,51	0,037747	0,53468		
TC0700002792.mm.1	1,27	Gramd1a	JUC0700013408.mm.1	2,51	0,001204	0,322097		
TC0700002792.mm.1	1,27	Gramd1a	JUC0700013414.mm.1	-3,39	0,026161	0,50003		
TC0700004647.mm.1	1,28	AW146154	JUC0700013824.mm.1	2,51	0,030419	0,513079		
TC0700004191.mm.1	-1,13	Dnah3	JUC0700018402.mm.1	2,51	0,006795	0,393554		
TC0400001328.mm.1	-1,14	Gm12898	JUC0400005248.mm.1	2,51	0,008159	0,40551		
TC1100002810.mm.1	-1,26	Gm12248	JUC1100013214.mm.1	2,51	0,018796	0,470789		
TC1100001933.mm.1	-1,38	Gm11739	JUC1100009573.mm.1	2,51	0,03952	0,539762		
TC1100002863.mm.1	-1,05	Cops3	PSR1100025993.mm.1	2,5	0,047557	0,558707	Alternative 5' Donc	0,25
TC1100002863.mm.1	-1,05	Cops3	PSR1100025997.mm.1	-2,53	0,038702	0,537225	Alternative 5' Donc	0,25
TC0200002757.mm.1	1,3	Phactr3	PSR0200022513.mm.1	2,5	0,029945	0,511499	Alternative 3' Acce	0,24
TC1700001755.mm.1	-1,96	Tead3	PSR1700016150.mm.1	2,5	0,004749	0,371039	Alternative 3' Acce	0,19
TC1700001755.mm.1	-1,96	Tead3	PSR1700016152.mm.1	2,23	0,036434	0,530977	Alternative 3' Acce	0,19
TC0600003601.mm.1	-1,62	Foxp1	PSR0600021891.mm.1	2,5	0,044193	0,551181	Cassette Exon	0,18
TC0600003601.mm.1	-1,62	Foxp1	JUC0600011352.mm.1	2,04	0,033904	0,52424		
TC0200001757.mm.1	-1,26	Casc5	PSR0200013117.mm.1	2,5	0,000405	0,297771	Alternative 3' Acce	0,14
TC0200001757.mm.1	-1,26	Casc5	JUC0200006541.mm.1	-3,38	0,019029	0,471827		
TC0600002044.mm.1	1,15	Slc35b4	PSR0600016062.mm.1	2,5	0,016565	0,459612	Cassette Exon	0,13
TC1400000985.mm.1	1,46	4930480K23Rik	JUC1400004256.mm.1	2,5	0,033638	0,523462		
TC0100002600.mm.1	-1,04	Cnppd1	JUC0100011948.mm.1	2,5	0,02068	0,478929		
TC0100002600.mm.1	-1,04	Cnppd1	JUC0100011950.mm.1	-2,27	0,000829	0,311886		
TC0100002844.mm.1	-1,24	St8sia4	JUC0100013107.mm.1	2,5	0,011711	0,429969		
TC1900000977.mm.1	-1,1	Sipa1	JUC1900004812.mm.1	2,5	0,025628	0,497926		
TC0100000334.mm.1	1,27	Ercc5	JUC0100001399.mm.1	2,5	0,026735	0,502096		
TC0100000334.mm.1	1,27	Ercc5	JUC0100001396.mm.1	2,41	0,017728	0,465216		
TC1500000278.mm.1	-1,48	Grhl2	JUC1500001200.mm.1	2,5	0,008306	0,406181		
TC1500000088.mm.1	-1,59	Slc45a2	JUC1500000504.mm.1	2,5	0,032503	0,519746		
TC0200000082.mm.1	1,03	Kin	JUC0200000251.mm.1	2,5	0,020033	0,476453		
TC0200000486.mm.1	1,07	Ralgds	JUC0200001668.mm.1	2,5	0,004658	0,370181		
TC0700004491.mm.1	-1,37		JUC0700019898.mm.1	2,5	0,005457	0,379939		
TC0500000298.mm.1	-1,18	Cenpa	JUC0500001294.mm.1	2,5	0,025053	0,496166		
TC0700000588.mm.1	-1,48	Gm10640	JUC0700002627.mm.1	2,5	0,030893	0,514574		
TC0300001802.mm.1	-1,36	Gnb4	JUC0300007350.mm.1	2,5	0,006091	0,386809		
TC0300001802.mm.1	-1,36	Gnb4	JUC0300007347.mm.1	-2,06	0,007302	0,398847		
TC0X00001658.mm.1	-1,05	Ap1s2	JUC0X00005376.mm.1	2,5	0,019513	0,474538		
TC0900001939.mm.1	3,67	Glb1l2	JUC0900009200.mm.1	2,49	0,041749	0,545426		
TC0900001939.mm.1	3,67	Glb1l2	JUC0900009204.mm.1	-2,16	0,019058	0,471978		
TC0900001939.mm.1	3,67	Glb1l2	PSR0900016246.mm.1	-2,33	0,006334	0,389463	Alternative 3' Acce	0,09
TC0900001939.mm.1	3,67	Glb1l2	PSR0900016240.mm.1	-2,35	0,030742	0,514065	Intron Retention	0,26
TC0900001939.mm.1	3,67	Glb1l2	JUC0900009198.mm.1	-2,43	0,014488	0,449377		
TC0900001939.mm.1	3,67	Glb1l2	PSR0900016260.mm.1	-2,68	0,025344	0,497329	Cassette Exon	0,2
TC0900001939.mm.1	3,67	Glb1l2	PSR0900016267.mm.1	-3,01	0,00953	0,416602	Cassette Exon	0,36
TC0900001939.mm.1	3,67	Glb1l2	PSR0900016269.mm.1	-3,28	0,008021	0,404268	Cassette Exon	0,21
TC0900001939.mm.1	3,67	Glb1l2	JUC0900009207.mm.1	-3,38	0,009593	0,417183		
TC0900001939.mm.1	3,67	Glb1l2	PSR0900016264.mm.1	-3,42	0,010728	0,423745	Cassette Exon	0,28
TC0900001939.mm.1	3,67	Glb1l2	JUC0900009201.mm.1	-3,58	0,004977	0,373851		

TC0900001939.mm.1	3,67	Glb1l2	JUC0900009188.mm.1	-4,39	0,000777	0,309336		
TC0900001939.mm.1	3,67	Glb1l2	JUC0900009209.mm.1	-9,17	0,00115	0,317344		
TC0500003582.mm.1	3,72	Tmem130	JUC0500017855.mm.1	2,49	0,002662	0,349612		
TC0500003582.mm.1	3,72	Tmem130	PSR0500032835.mm.1	-2,1	0,012874	0,438024	Cassette Exon	0,2
TC0500003582.mm.1	3,72	Tmem130	JUC0500017853.mm.1	-2,88	0,042937	0,548271		
TC0500003582.mm.1	3,72	Tmem130	PSR0500032845.mm.1	-3,2	0,009481	0,416578	Alternative 5' Donc	0,22
TC0500003582.mm.1	3,72	Tmem130	JUC0500017851.mm.1	-4,44	0,008988	0,411965		
TC1000001138.mm.1	1,17	Ntn4	PSR1000008635.mm.1	2,49	0,045035	0,553049	Alternative 5' Donc	0,22
TC0500000360.mm.1	1,27	Tacc3	JUC0500001862.mm.1	2,49	0,028274	0,506588		
TC0500000360.mm.1	1,27	Tacc3	PSR0500003353.mm.1	2,38	0,024304	0,49362	Alternative 3' Acce	0,21
TC1200002030.mm.1	-1,13		PSR1200013996.mm.1	2,49	0,01222	0,433326	Alternative 5' Donc	0,21
TC1800001312.mm.1	1,65	Sema6a	JUC1800005405.mm.1	2,49	0,019997	0,476227		
TC1800001312.mm.1	1,65	Sema6a	JUC1800005411.mm.1	-2,28	0,004538	0,368648		
TC1800001312.mm.1	1,65	Sema6a	PSR1800009740.mm.1	-2,37	0,034617	0,52602	Cassette Exon	0,19
TC1800001312.mm.1	1,65	Sema6a	PSR1800009741.mm.1	-2,57	0,009711	0,417826	Cassette Exon	0,21
TC1000002393.mm.1	-2,16	Gm867	PSR1000017154.mm.1	2,49	0,009616	0,417315	Cassette Exon	0,19
TC1000002393.mm.1	-2,16	Gm867	JUC1000009356.mm.1	2,16	0,017098	0,46232		
TC1900001549.mm.1	-1,65	Cpn1	PSR1900013672.mm.1	2,49	0,010948	0,425002	Alternative 5' Donc	0,17
TC0Y00000221.mm.1	-1,29	Gm21857; Mid1	PSR0Y00001252.mm.1	2,49	0,012607	0,435947	Cassette Exon	0,16
TC0Y00000224.mm.1	-1,29	Mid1	PSR0Y00001284.mm.1	2,49	0,012607	0,435947	Cassette Exon	0,16
TC1700000564.mm.1	-1,28	Cyp4f16	PSR1700005108.mm.1	2,49	0,049253	0,562576	Cassette Exon	0,16
TC1300002024.mm.1	1,24	Drd1a	PSR1300012641.mm.1	2,49	0,01105	0,42586	Alternative 5' Donc	0,12
TC1300002024.mm.1	1,24	Drd1a	PSR1300012639.mm.1	-2,01	0,001772	0,336311	Alternative 3' Acce	0,15
TC1700001511.mm.1	-1,69		PSR1700013491.mm.1	2,49	0,022281	0,486046	Cassette Exon	0,15
TC0300001161.mm.1	1,03	Vav3	JUC0300004924.mm.1	2,49	0,049882	0,563773		
TC0300001161.mm.1	1,03	Vav3	PSR0300009521.mm.1	2,44	0,019161	0,472719	Cassette Exon	0,14
TC0700001096.mm.1	-2,2		JUC0700004957.mm.1	2,49	0,037173	0,53313		
TC0700001096.mm.1	-2,2		PSR0700009680.mm.1	2,13	0,000648	0,304044	Cassette Exon	0,1
TC0200003106.mm.1	1,11	Spopl	JUC0200012824.mm.1	2,49	0,023856	0,492097		
TC0300000910.mm.1	-1,35	Mcl1	JUC0300003769.mm.1	2,49	0,000148	0,272178		
TC0200003592.mm.1	1,35	Baz2b; LOC100505179	JUC0200015499.mm.1	2,49	0,048548	0,561008		
TC0200003592.mm.1	1,35	Baz2b; LOC100505179	JUC0200015523.mm.1	2,49	0,004683	0,370323		
TC0200003592.mm.1	1,35	Baz2b; LOC100505179	JUC0200015497.mm.1	2,11	0,018483	0,46874		
TC0200003592.mm.1	1,35	Baz2b; LOC100505179	JUC0200015536.mm.1	-2,84	0,003475	0,355628		
TC0100003119.mm.1	-1,18	Lgr6	JUC0100014284.mm.1	2,49	0,019387	0,473657		
TC1700001084.mm.1	-1,08	Ddx11	JUC1700005515.mm.1	2,49	0,005783	0,383104		
TC1900001169.mm.1	1,02	Zfp91; Cntf; Zfp91Cntf	JUC1900005915.mm.1	2,49	0,012431	0,43528		
TC0100000464.mm.1	-1,17	Fam117b	JUC0100002201.mm.1	2,49	0,04761	0,558743		
TC1800001723.mm.1	-1,26		JUC1800006825.mm.1	2,49	0,026589	0,501731		
TC0100001125.mm.1	1,16	Rab3gap1	JUC0100005296.mm.1	2,49	0,033964	0,524252		
TC0100001125.mm.1	1,16	Rab3gap1	JUC0100005288.mm.1	2,07	0,039312	0,539263		
TC1900000491.mm.1	1,02	Lipn	JUC1900002276.mm.1	2,49	0,048178	0,560073		
TC1500002159.mm.1	1,05	Vdr	JUC1500009944.mm.1	2,49	0,016439	0,459014		
TC1500000878.mm.1	1,07	Shank3	JUC1500003918.mm.1	2,49	0,00304	0,353826		
TC1500000878.mm.1	1,07	Shank3	JUC1500003903.mm.1	-2,75	0,01955	0,474538		
TC0700001332.mm.1	1,36	Tenm4	JUC0700005855.mm.1	2,49	0,03235	0,519174		
TC0700002672.mm.1	1,02	Map3k10	JUC0700012406.mm.1	2,49	0,021256	0,481453		
TC0700002049.mm.1	-1,38	Taldo1	JUC0700009633.mm.1	2,49	0,004298	0,364027		

TC0900002196.mm.1	-1,24	Tmprss4	JUC0900010318.mm.1	2,49	0,007577	0,401703		
TC0900002127.mm.1	-1,18	Pou2f3	JUC0900009983.mm.1	2,49	0,004459	0,36753		
TC1000000175.mm.1	-1,28	H60b; Raet1a; Raet1b; Ra	JUC1000000714.mm.1	2,49	0,040637	0,542602		
TC0Y00000367.mm.1	-1,67	Gm20923; LOC101056194	JUC0Y00001630.mm.1	2,49	0,005893	0,383835		
TC0Y00000426.mm.1	-1,67	Gm21171; LOC101056194	JUC0Y00001740.mm.1	2,49	0,005893	0,383835		
TC0Y00000437.mm.1	-1,67	Gm20874; LOC101056194	JUC0Y00001750.mm.1	2,49	0,005893	0,383835		
TC0Y00000438.mm.1	-1,67	Gm20937; LOC101056194	JUC0Y00001756.mm.1	2,49	0,005893	0,383835		
TC0Y00000452.mm.1	-1,67	Gm20843; LOC101056194	JUC0Y00001777.mm.1	2,49	0,005893	0,383835		
TC0Y00000145.mm.1	-1,67	Gm21209; LOC101056194	JUC0Y00000414.mm.1	2,49	0,005893	0,383835		
TC0Y00000170.mm.1	-1,67	Gm21518; LOC101056194	JUC0Y00000623.mm.1	2,49	0,005893	0,383835		
TC0Y00000190.mm.1	-1,67	Gm20888; LOC101056194	JUC0Y00000669.mm.1	2,49	0,005893	0,383835		
TC0Y00000202.mm.1	-1,67	Gm21650; LOC101056194	JUC0Y00000764.mm.1	2,49	0,005893	0,383835		
TC0Y00000201.mm.1	-1,67	Gm21638; LOC101056194	JUC0Y00000758.mm.1	2,49	0,005893	0,383835		
TC0400001594.mm.1	-1,1	Eya3	PSR0400012506.mm.1	2,48	0,007947	0,403819	Cassette Exon	0,24
TC0200005333.mm.1	1,28	Gm14443	JUC0200023398.mm.1	2,48	0,029738	0,511096		
TC0200005333.mm.1	1,28	Gm14443	PSR0200045440.mm.1	2,15	0,004147	0,3623	Cassette Exon	0,19
TC1700001395.mm.1	-1,38	Tagap1	PSR1700012581.mm.1	2,48	0,015417	0,454055	Cassette Exon	0,17
TC0500000991.mm.1	-1,6	9430085M18Rik	PSR0500008374.mm.1	2,48	0,021181	0,480978	Cassette Exon	0,16
TC0800001959.mm.1	4,98	Chrna6	JUC0800008499.mm.1	2,48	0,016376	0,458693		
TC0800001959.mm.1	4,98	Chrna6	PSR0800015561.mm.1	2,32	0,014506	0,449545	Cassette Exon	0,12
TC0800001959.mm.1	4,98	Chrna6	JUC0800008498.mm.1	-2,2	0,023611	0,491012		
TC0800001959.mm.1	4,98	Chrna6	PSR0800015560.mm.1	-2,28	0,00269	0,349612	Cassette Exon	0,05
TC0200002797.mm.1	-1	Col9a3	JUC0200011604.mm.1	2,48	0,023195	0,490032		
TC0200004958.mm.1	-1,18	Pigu	JUC0200021692.mm.1	2,48	0,021832	0,484607		
TC1700000858.mm.1	-1,11	B230354K17Rik	JUC1700004399.mm.1	2,48	0,036801	0,532069		
TC1500001534.mm.1	-1,47	Dscc1	JUC1500006551.mm.1	2,48	0,043112	0,548605		
TC1700000349.mm.1	-1,38	Rps2; Snora64; Gm8842; (	JUC1700001477.mm.1	2,48	0,02337	0,490143		
TC1700000349.mm.1	-1,38	Rps2; Snora64; Gm8842; (	PSR1700002692.mm.1	2,24	0,006599	0,39213		
TC1600001233.mm.1	-1,3	Pla2g10	JUC1600005127.mm.1	2,48	0,000617	0,304044		
TC0900000876.mm.1	-1,25	Rps27l	PSR0900006793.mm.1	2,48	0,04176	0,545489		
TC0800002628.mm.1	-1,05	Abcc12	JUC0800010965.mm.1	2,48	0,002269	0,34579		
TC0800002628.mm.1	-1,05	Abcc12	JUC0800010967.mm.1	-2,73	0,00569	0,382387		
TC0700002924.mm.1	-1,01	Zfp141	JUC0700013870.mm.1	2,48	0,012146	0,432868		
TC0700002924.mm.1	-1,01	Zfp141	JUC0700013874.mm.1	-2,88	0,031002	0,514828		
TC0400003747.mm.1	1,18	Tmem57	JUC0400016052.mm.1	2,48	0,049691	0,563531		
TC0400003670.mm.1	-2,38	Phactr4	PSR0400030116.mm.1	2,48	0,006583	0,39213		
TC1700001247.mm.1	-1,43	Mta3	PSR1700011553.mm.1	2,47	0,039405	0,539557	Cassette Exon	0,17
TC0400003395.mm.1	-1,9	Tmem125	PSR0400027543.mm.1	2,47	0,014157	0,447466	Cassette Exon	0,15
TC0400003395.mm.1	-1,9	Tmem125	PSR0400027544.mm.1	2,28	0,027138	0,503096	Cassette Exon	0,13
TC0700000402.mm.1	-1,55	Cd79a	PSR0700002998.mm.1	2,47	0,003276	0,354243	Cassette Exon	0,15
TC0X00000316.mm.1	-2,39	Il13ra1	PSR0X00002103.mm.1	2,47	0,016182	0,457969	Alternative 3' Acce	0,12
TC1100001235.mm.1	-1,29	Unc45b	JUC1100006063.mm.1	2,47	0,007239	0,39826		
TC1100001235.mm.1	-1,29	Unc45b	PSR1100011581.mm.1	-2,15	0,036468	0,531117	Cassette Exon	0,11
TC0100003113.mm.1	1,34	Ppp1r12b	JUC0100014264.mm.1	2,47	0,021398	0,482036		
TC1500000787.mm.1	-1	Parvg	PSR1500006202.mm.1	2,47	0,012096	0,43255		
TC1500000787.mm.1	-1	Parvg	JUC1500003477.mm.1	-2,56	0,028509	0,507386		
TC1700000431.mm.1	-1,08	Itpr3; Mir7677; mmu-mir-	JUC1700002038.mm.1	2,47	0,022569	0,487322		
TC0800001110.mm.1	-1,16	Nlrc5; Mir7072; mmu-mir-	JUC0800004587.mm.1	2,47	0,020659	0,478862		

TC0800001110.mm.1	-1,16	Nlrc5; Mir7072; mmu-mir-	PSR0800008610.mm.1	-2,04	0,009467	0,416539		
TC0800001586.mm.1	-1,31	Pard3	JUC0800007098.mm.1	2,47	0,00953	0,416602		
TC0900001200.mm.1	-1,04	Pcolce2	JUC0900005115.mm.1	2,47	0,041023	0,543661		
TC0800003163.mm.1	1,11	Abcb10	JUC0800013264.mm.1	2,47	0,006863	0,394112		
TC0600001418.mm.1	1,08	Cd163	JUC0600006017.mm.1	2,47	0,037596	0,534053		
TC0300001445.mm.1	-1,47	Gbp7	JUC0300006113.mm.1	2,47	0,012989	0,439245		
TC1100001331.mm.1	1,58	Trim37	JUC1100006523.mm.1	2,47	0,009307	0,414883		
TC1100001331.mm.1	1,58	Trim37	JUC1100006518.mm.1	2,24	0,010732	0,423745		
TC0900003064.mm.1	1,61	Wdr6	JUC0900014478.mm.1	2,47	0,037841	0,534803		
TCX_GL456233_random00	2,69	Spry3	PSRX_GL456233_random	2,46	0,011016	0,425624	Cassette Exon	0,23
TCX_GL456233_random00	2,69	Spry3	PSRX_GL456233_random	2,41	0,010946	0,425002	Alternative 5' Donc	0,32
TC1700000662.mm.1	-1,91	Clic1	PSR1700006675.mm.1	2,46	0,006977	0,395722	Alternative 5' Donc	0,23
TC0100002470.mm.1	-1,56	Fzd5	PSR0100020049.mm.1	2,46	0,000879	0,311909	Alternative 3' Acce	0,21
TC1700001453.mm.1	-1,35	Gm7162	PSR1700012965.mm.1	2,46	0,005824	0,383169	Alternative 5' Donc	0,18
TC0400002021.mm.1	1,64	Icmt	JUC0400008676.mm.1	2,46	0,002417	0,348907		
TC0400002021.mm.1	1,64	Icmt	JUC0400008677.mm.1	2,46	0,016178	0,457969		
TC0400002021.mm.1	1,64	Icmt	JUC0400008673.mm.1	2,41	0,008122	0,405267		
TC0400002021.mm.1	1,64	Icmt	PSR0400016558.mm.1	2,25	0,021559	0,483034	Cassette Exon	0,07
TC0400002021.mm.1	1,64	Icmt	PSR0400016559.mm.1	2,1	0,004568	0,369396	Cassette Exon	0,17
TC0200000694.mm.1	1,02	Crb2	PSR0200006134.mm.1	2,46	0,002367	0,34829	Cassette Exon	0,16
TC0200000694.mm.1	1,02	Crb2	PSR0200006130.mm.1	2,03	0,020218	0,477145	Cassette Exon	0,14
TC1800000281.mm.1	-1,5		JUC1800001178.mm.1	2,46	0,040313	0,541838		
TC1800000281.mm.1	-1,5		PSR1800002003.mm.1	2,11	0,041492	0,544746	Alternative 5' Donc	0,16
TC1800000281.mm.1	-1,5		JUC1800001175.mm.1	2,04	0,036923	0,53232		
TC0X00001274.mm.1	-1,02	Arl13a	PSR0X00007895.mm.1	2,46	0,005726	0,382767	Cassette Exon	0,14
TC0X00001274.mm.1	-1,02	Arl13a	PSR0X00007888.mm.1	-2,46	0,011151	0,42626	Cassette Exon	0,13
TC1500001480.mm.1	1,43	Csmd3	PSR1500011314.mm.1	2,46	0,007344	0,399264	Cassette Exon	0,14
TC1500001480.mm.1	1,43	Csmd3	JUC1500006372.mm.1	-2,12	0,038606	0,536875		
TC1500001480.mm.1	1,43	Csmd3	JUC1500006387.mm.1	-4,78	0,003425	0,355303		
TC0600000788.mm.1	-1,34	Serbp1	PSR0600005891.mm.1	2,46	0,03669	0,531756	Alternative 5' Donc	0,08
TC1400000946.mm.1	2,34	Stmn4	PSR1400007617.mm.1	2,46	0,031422	0,51654	Cassette Exon	0,04
TC1400000946.mm.1	2,34	Stmn4	JUC1400004104.mm.1	2,02	0,038429	0,536405		
TC1400000946.mm.1	2,34	Stmn4	JUC1400004113.mm.1	-3,09	0,039739	0,540494		
TC1400001920.mm.1	-1,47	Cnih1; Cnih	JUC1400007958.mm.1	2,46	0,000877	0,311909		
TC1300000718.mm.1	1,56	Ntrk2	JUC1300002473.mm.1	2,46	0,040033	0,541307		
TC1500001662.mm.1	-1,38		JUC1500007082.mm.1	2,46	0,003385	0,354476		
TC0200001597.mm.1	1,03	Mpped2	JUC0200006003.mm.1	2,46	0,005579	0,381035		
TC1500000862.mm.1	-1,23	Selo	JUC1500003808.mm.1	2,46	0,033567	0,523133		
TC0200001121.mm.1	-1,04	Agps	JUC0200004608.mm.1	2,46	0,007132	0,397304		
TC0200001121.mm.1	-1,04	Agps	JUC0200004591.mm.1	2,04	0,024037	0,493014		
TC0700001269.mm.1	-1,24	Me3	JUC0700005637.mm.1	2,46	0,036614	0,531494		
TC0600001787.mm.1	-1,88	Samd9l	JUC0600007386.mm.1	2,46	0,028371	0,506881		
TC1100002567.mm.1	-1,42	Pttg1	JUC1100012350.mm.1	2,46	0,031315	0,516204		
TC0X00001798.mm.1	-1,01	Tbc1d25	JUC0X00005816.mm.1	2,46	0,010016	0,418611		
TC0400001688.mm.1	-1,26	Luzp1	JUC0400007031.mm.1	2,46	0,017201	0,462491		
TC0800000286.mm.1	-1,44	Plat	PSR0800002129.mm.1	2,45	0,034335	0,525546	Intron Retention	0,36
TC1200001938.mm.1	-1,26	Wdr89; Rplp2-ps1	PSR1200013305.mm.1	2,45	0,016144	0,457937	Alternative 5' Donc	0,23
TC1000000543.mm.1	2,02	Spock2	JUC1000002025.mm.1	2,45	0,011037	0,425682		

TC1000000543.mm.1	2,02	Spock2	JUC1000002016.mm.1	2,44	0,024178	0,493294		
TC1000000543.mm.1	2,02	Spock2	PSR1000003670.mm.1	-2,04	0,002118	0,342919	Alternative 5' Donc	0,22
TC1000000543.mm.1	2,02	Spock2	PSR1000003658.mm.1	-2,04	0,017809	0,46577	Alternative 3' Acce	0,12
TC1000000543.mm.1	2,02	Spock2	PSR1000003659.mm.1	-2,07	0,022573	0,487325	Alternative 3' Acce	0,13
TC1000000543.mm.1	2,02	Spock2	JUC1000002020.mm.1	-2,09	0,036186	0,529982		
TC1000000543.mm.1	2,02	Spock2	JUC1000002024.mm.1	-2,29	0,015784	0,455613		
TC1000000543.mm.1	2,02	Spock2	JUC1000002027.mm.1	-5,23	0,014245	0,447947		
TC0500001392.mm.1	-1,43	Arpc3	PSR0500012322.mm.1	2,45	0,04269	0,548097	Alternative 5' Donc	0,21
TC1100001707.mm.1	1,23	Hexim2	PSR1100016145.mm.1	2,45	0,009239	0,414373	Alternative 5' Donc	0,21
TC0700001169.mm.1	1,97	Pde8a	PSR0700010293.mm.1	2,45	0,049714	0,563531	Cassette Exon	0,1
TC0700001169.mm.1	1,97	Pde8a	JUC0700005322.mm.1	-2,14	0,048153	0,560004		
TC0700001169.mm.1	1,97	Pde8a	PSR0700010280.mm.1	-2,21	0,024462	0,493992	Alternative 5' Donc	0,18
TC0700001169.mm.1	1,97	Pde8a	PSR0700010304.mm.1	-2,23	0,029419	0,509995	Cassette Exon	0,17
TC0700001169.mm.1	1,97	Pde8a	PSR0700010291.mm.1	-2,63	0,008038	0,40434	Cassette Exon	0,16
TC0700001169.mm.1	1,97	Pde8a	JUC0700005321.mm.1	-4,2	0,009641	0,417354		
TC0600003221.mm.1	-2,32		PSR0600025177.mm.1	2,45	0,024785	0,495079	Alternative 3' Acce	0,15
TC1700001360.mm.1	-1,71	4930470H14Rik	PSR1700012328.mm.1	2,45	0,037641	0,534212	Cassette Exon	0,14
TC0X00001318.mm.1	-1,71	5730412P04Rik	PSR0X00008298.mm.1	2,45	0,014036	0,44654	Cassette Exon	0,13
TC0X00001318.mm.1	-1,71	5730412P04Rik	PSR0X00008301.mm.1	2,32	0,00016	0,272566	Cassette Exon	0,12
TC1800000809.mm.1	-1,56	Zbtb7c	PSR1800005863.mm.1	2,45	0,020516	0,478264	Cassette Exon	0,12
TC1400001988.mm.1	-1,19	Tep1	JUC1400008140.mm.1	2,45	0,01527	0,452983		
TC1400000327.mm.1	1,12	Capn7	JUC1400001697.mm.1	2,45	0,003212	0,354243		
TC0300000742.mm.1	1,05	Ash1l	JUC0300002871.mm.1	2,45	0,008203	0,405909		
TC1700002225.mm.1	-1,21	Nfya	JUC1700011196.mm.1	2,45	0,026493	0,501252		
TC0100001268.mm.1	-1,46	Ddx59	JUC0100006090.mm.1	2,45	0,000216	0,28803		
TC1500001819.mm.1	-1,43	Myh9; Gm22107	JUC1500008090.mm.1	2,45	0,048933	0,5618		
TC0800001437.mm.1	1,17	Gse1	JUC0800006469.mm.1	2,45	0,049123	0,562253		
TC1100001908.mm.1	1,18	Llgl2	JUC1100009361.mm.1	2,45	0,023833	0,491992		
TC1100001908.mm.1	1,18	Llgl2	JUC1100009378.mm.1	-2,26	0,001545	0,330631		
TC0900001979.mm.1	1,37	Aplp2	JUC0900009359.mm.1	2,45	0,048665	0,561341		
TC0900001979.mm.1	1,37	Aplp2	JUC0900009357.mm.1	2,36	0,032963	0,52127		
TC0900001979.mm.1	1,37	Aplp2	JUC0900009352.mm.1	-2,55	0,044882	0,552704		
TC1300001653.mm.1	4,94	Scgn	JUC1300005233.mm.1	2,44	0,033609	0,523309		
TC1300001653.mm.1	4,94	Scgn	PSR1300010240.mm.1	-2,47	0,012498	0,435429	Cassette Exon	0,05
TC1300001653.mm.1	4,94	Scgn	PSR1300010234.mm.1	-8,48	0,001791	0,336311	Cassette Exon	0,34
TC1300001653.mm.1	4,94	Scgn	JUC1300005225.mm.1	-9,74	0,000578	0,304044		
TC1100002060.mm.1	1,75	Hexdc	PSR1100019879.mm.1	2,44	0,042664	0,548097	Alternative 5' Donc	0,12
TC1100002060.mm.1	1,75	Hexdc	PSR1100019857.mm.1	-2,21	0,015656	0,455329		
TC1100002060.mm.1	1,75	Hexdc	PSR1100019880.mm.1	-2,4	0,009447	0,416265	Intron Retention	0,28
TC1400002186.mm.1	2,25	Ebpl	PSR1400016933.mm.1	2,44	0,004484	0,367949	Cassette Exon	0,25
TC1400002186.mm.1	2,25	Ebpl	PSR1400016935.mm.1	-2,23	0,004384	0,366156	Alternative 5' Donc	0,19
TC1400002186.mm.1	2,25	Ebpl	JUC1400009266.mm.1	-2,62	0,025182	0,496638		
TC0700002135.mm.1	1,23	Mboat7	JUC0700010232.mm.1	2,44	0,037338	0,533573		
TC0700002135.mm.1	1,23	Mboat7	PSR0700019279.mm.1	-2,03	0,034641	0,526115	Intron Retention	0,24
TC1000000136.mm.1	2,53	Gm20139	PSR1000000950.mm.1	2,44	0,019494	0,474486	Alternative 5' Donc	0,23
TC1000000136.mm.1	2,53	Gm20139	JUC1000000481.mm.1	-3,29	0,01625	0,458312		
TC1000000136.mm.1	2,53	Gm20139	JUC1000000480.mm.1	-3,46	0,004178	0,362543		
TC1000000136.mm.1	2,53	Gm20139	JUC1000000479.mm.1	-3,82	0,000287	0,28803		

TC1000000136.mm.1	2,53	Gm20139	JUC1000000478.mm.1	-3,84	0,000929	0,313363		
TC0800002265.mm.1	2,13	Galntl6	JUC0800009362.mm.1	2,44	0,012977	0,439245		
TC0800002265.mm.1	2,13	Galntl6	PSR0800017232.mm.1	-2,15	0,013831	0,445222	Cassette Exon	0,2
TC0800002265.mm.1	2,13	Galntl6	PSR0800017219.mm.1	-2,5	0,008959	0,411476	Cassette Exon	0,15
TC0800002265.mm.1	2,13	Galntl6	PSR0800017213.mm.1	-2,96	0,00205	0,341391	Cassette Exon	0,04
TC0800002265.mm.1	2,13	Galntl6	JUC0800009354.mm.1	-3,16	0,024807	0,495222		
TC0800002265.mm.1	2,13	Galntl6	JUC0800009372.mm.1	-3,83	0,019622	0,47483		
TC0800002265.mm.1	2,13	Galntl6	JUC0800009369.mm.1	-3,87	0,009171	0,413447		
TC0500000806.mm.1	-1,26	Smr3a	PSR0500007080.mm.1	2,44	0,011682	0,429939	Cassette Exon	0,15
TC0900003050.mm.1	1,53	Rnf123	JUC0900014362.mm.1	2,44	0,013279	0,441719		
TC0900003050.mm.1	1,53	Rnf123	PSR0900025679.mm.1	-2,01	0,009237	0,414373	Cassette Exon	0,06
TC0900003050.mm.1	1,53	Rnf123	PSR0900025599.mm.1	-2,08	0,0249	0,495661	Alternative 3' Acce	0,15
TC0900003050.mm.1	1,53	Rnf123	JUC0900014357.mm.1	-2,09	0,020286	0,477245		
TC1400000846.mm.1	2,04	Amer2	PSR1400006919.mm.1	2,44	0,010035	0,41878	Alternative 5' Donc	0,15
TC1400000846.mm.1	2,04	Amer2	JUC1400003728.mm.1	-2,19	0,02212	0,485558		
TC1500002294.mm.1	1,89	Map3k12	JUC1500010852.mm.1	2,44	0,014609	0,449914		
TC1500002294.mm.1	1,89	Map3k12	PSR1500018979.mm.1	-2,11	0,019714	0,474887	Alternative 5' Donc	0,14
TC1500002294.mm.1	1,89	Map3k12	PSR1500018986.mm.1	-2,73	0,014252	0,447947	Alternative 5' Donc	0,15
TC1500002294.mm.1	1,89	Map3k12	JUC1500010863.mm.1	-2,99	0,019808	0,475245		
TC0800002398.mm.1	1,64	Ssbp4	JUC0800009892.mm.1	2,44	0,024645	0,49468		
TC0800002398.mm.1	1,64	Ssbp4	PSR0800018231.mm.1	2,08	0,010229	0,419955	Cassette Exon	0,14
TC0800002398.mm.1	1,64	Ssbp4	PSR0800018225.mm.1	2,03	0,000364	0,290942	Cassette Exon	0,12
TC0800002398.mm.1	1,64	Ssbp4	JUC0800009879.mm.1	-2,45	0,035332	0,528404		
TC1000002185.mm.1	1,74	Tbc1d32	JUC1000008344.mm.1	2,44	0,041951	0,54592		
TC1000002185.mm.1	1,74	Tbc1d32	PSR1000015336.mm.1	2,19	0,019607	0,474765	Cassette Exon	0,1
TC1000002185.mm.1	1,74	Tbc1d32	PSR1000015312.mm.1	-2,11	0,040247	0,541731	Cassette Exon	0,14
TC1300002095.mm.1	1	Ubqln1	PSR1300013302.mm.1	2,44	0,034047	0,524583	Cassette Exon	0,14
TC1100002641.mm.1	-1,78	Irgm1	PSR1100024154.mm.1	2,44	0,012784	0,437236	Alternative 3' Acce	0,13
TC1100002641.mm.1	-1,78	Irgm1	PSR1100024152.mm.1	2,03	0,029679	0,510918	Cassette Exon	0,11
TC0200004959.mm.1	-1,42	Ncoa6	JUC0200021706.mm.1	2,44	0,018808	0,470836		
TC0100000661.mm.1	1,13	Tmem198	JUC0100003273.mm.1	2,44	0,004023	0,361485		
TC1500001966.mm.1	1,34	Poldip3	JUC1500008770.mm.1	2,44	0,030057	0,511847		
TC0500001716.mm.1	-1,05	Ap5z1	JUC0500008542.mm.1	2,44	0,001149	0,317344		
TC0600000958.mm.1	-1,86	Emx1	JUC0600003768.mm.1	2,44	0,028297	0,506675		
TC0600003598.mm.1	-1,4	Cml3	JUC0600010535.mm.1	2,44	0,046011	0,555624		
TC0600003598.mm.1	-1,4	Cml3	JUC0600010534.mm.1	-2,84	0,018177	0,467441		
TC1100003437.mm.1	-1,01	Ints2	JUC1100016489.mm.1	2,44	0,049296	0,562691		
TC0300001521.mm.1	-1,47	Eltd1	JUC0300006473.mm.1	2,44	0,015083	0,452418		
TC0300001521.mm.1	-1,47	Eltd1	JUC0300006476.mm.1	2,35	0,048885	0,561732		
TC0400001347.mm.1	-1,14	Scmh1	JUC0400005356.mm.1	2,44	0,022634	0,487464		
TC1700001105.mm.1	2,3	Epb4.1l3	PSR1700010435.mm.1	2,43	0,039068	0,538368	Cassette Exon	0,24
TC1700001105.mm.1	2,3	Epb4.1l3	JUC1700005654.mm.1	2,19	0,012062	0,432426		
TC1700001105.mm.1	2,3	Epb4.1l3	JUC1700005661.mm.1	2,12	0,030098	0,511942		
TC1700001105.mm.1	2,3	Epb4.1l3	PSR1700010432.mm.1	-2,34	0,022781	0,488128	Cassette Exon	0,31
TC1700001105.mm.1	2,3	Epb4.1l3	JUC1700005668.mm.1	-2,75	0,016697	0,460085		
TC1700001105.mm.1	2,3	Epb4.1l3	PSR1700010428.mm.1	-3,15	0,003114	0,353892	Alternative 5' Donc	0,27
TC1700001105.mm.1	2,3	Epb4.1l3	JUC1700005652.mm.1	-3,59	0,018786	0,470789		
TC1700001105.mm.1	2,3	Epb4.1l3	JUC1700005663.mm.1	-3,59	0,025322	0,497278		

TC0200001551.mm.1	-1,7	Cd59b	JUC0200005824.mm.1	2,43	0,019297	0,473074		
TC0200001551.mm.1	-1,7	Cd59b	PSR0200011655.mm.1	2,32	0,001412	0,326543	Alternative 5' Donc	0,16
TC0200001551.mm.1	-1,7	Cd59b	PSR0200011649.mm.1	2,22	0,042808	0,548097	Cassette Exon	0,15
TC0200001551.mm.1	-1,7	Cd59b	JUC0200005817.mm.1	2,07	0,025391	0,497403		
TC1300001636.mm.1	-1,08	Hist1h2be; Hist1h1e; Hist	PSR1300010149.mm.1	2,43	0,002886	0,352207	Alternative 3' Acce	0,04
TC1300001636.mm.1	-1,08	Hist1h2be; Hist1h1e; Hist	PSR1300010145.mm.1	2,26	0,009905	0,418332	Alternative 3' Acce	0,03
TC1300001636.mm.1	-1,08	Hist1h2be; Hist1h1e; Hist	PSR1300010148.mm.1	2,1	0,011378	0,427502	Alternative 3' Acce	0,04
TC1300001636.mm.1	-1,08	Hist1h2be; Hist1h1e; Hist	PSR1300010147.mm.1	2,08	0,0499	0,563773	Alternative 3' Acce	0,04
TC0300000144.mm.1	-1,09	Tbl1xr1	JUC0300000312.mm.1	2,43	0,020824	0,47952		
TC1200002220.mm.1	1,27	Nrde2	JUC1200008431.mm.1	2,43	0,006112	0,387239		
TC1200002220.mm.1	1,27	Nrde2	JUC1200008421.mm.1	-2,04	0,024149	0,493245		
TC1700002186.mm.1	1,03	Ppp2r5d	JUC1700010995.mm.1	2,43	0,03891	0,537874		
TC0100002856.mm.1	-1,39	Slco4c1	JUC0100013126.mm.1	2,43	0,011992	0,432286		
TC1900000708.mm.1	-1,19	2010012O05Rik	JUC1900003388.mm.1	2,43	0,020434	0,478018		
TC1500000119.mm.1	1,3	Cdh9	JUC1500000651.mm.1	2,43	0,023835	0,491992		
TC1600000850.mm.1	-1,14	Rbm11	JUC1600003702.mm.1	2,43	0,020419	0,477971		
TC1600001473.mm.1	-1,56	Acap2	JUC1600006356.mm.1	2,43	0,041334	0,544327		
TC1600001473.mm.1	-1,56	Acap2	JUC1600006345.mm.1	2,24	0,036975	0,532502		
TC0800001300.mm.1	1,14	Zfp821	JUC0800005708.mm.1	2,43	0,026671	0,501946		
TC0800002798.mm.1	-1,48	Cmtm2a; Cmtm1	JUC0800011681.mm.1	2,43	0,013639	0,444258		
TC0500001644.mm.1	1,4	Ap4m1	JUC0500008204.mm.1	2,43	0,014458	0,44909		
TC0900002239.mm.1	1	Nnmt	JUC0900010517.mm.1	2,43	0,017219	0,462614		
TC1000000848.mm.1	1,05	Dazap1	JUC1000003483.mm.1	2,43	0,024377	0,49362		
TC1000000848.mm.1	1,05	Dazap1	JUC1000003490.mm.1	-2,04	0,017265	0,462888		
TC0400002743.mm.1	1,07	AI314180	JUC0400011527.mm.1	2,43	0,022625	0,487464		
TC0X00003189.mm.1	1,09	Kantr; 2900056M20Rik	PSR0X00020038.mm.1	2,43	0,027382	0,503961		
TC0X00003189.mm.1	1,09	Kantr; 2900056M20Rik	JUC0X00010118.mm.1	-2,79	0,019245	0,473063		
TC1900000027.mm.1	3,17	Pitpnm1	JUC1900000177.mm.1	2,42	0,038949	0,537874		
TC1900000027.mm.1	3,17	Pitpnm1	PSR1900000338.mm.1	-2,13	0,021512	0,482748	Cassette Exon	0,04
TC1900000027.mm.1	3,17	Pitpnm1	JUC1900000193.mm.1	-2,33	0,000016	0,192353		
TC1900000027.mm.1	3,17	Pitpnm1	PSR1900000384.mm.1	-2,4	0,000864	0,311909	Intron Retention	0,54
TC1900000027.mm.1	3,17	Pitpnm1	PSR1900000369.mm.1	-2,68	0,017758	0,465425	Intron Retention	0,34
TC1900000027.mm.1	3,17	Pitpnm1	PSR1900000347.mm.1	-3,38	0,004642	0,370181	Alternative 5' Donc	0,37
TC1900000027.mm.1	3,17	Pitpnm1	PSR1900000335.mm.1	-3,54	0,006649	0,393053	Cassette Exon	0,25
TC1900000027.mm.1	3,17	Pitpnm1	JUC1900000198.mm.1	-3,56	0,006908	0,394872		
TC1900000027.mm.1	3,17	Pitpnm1	PSR1900000360.mm.1	-3,68	0,01772	0,465209	Alternative 3' Acce	0,31
TC1900000027.mm.1	3,17	Pitpnm1	JUC1900000186.mm.1	-3,74	0,016761	0,460504		
TC1900000027.mm.1	3,17	Pitpnm1	PSR1900000337.mm.1	-3,76	0,001623	0,332831	Cassette Exon	0,39
TC1900000027.mm.1	3,17	Pitpnm1	PSR1900000336.mm.1	-3,82	0,009208	0,414013	Cassette Exon	0,25
TC1900000027.mm.1	3,17	Pitpnm1	PSR1900000383.mm.1	-3,9	0,013318	0,441836	Intron Retention	0,55
TC1900000027.mm.1	3,17	Pitpnm1	JUC1900000199.mm.1	-4,11	0,004935	0,373093		
TC1900000027.mm.1	3,17	Pitpnm1	PSR1900000379.mm.1	-4,54	0,001621	0,332831	Intron Retention	0,72
TC1900000027.mm.1	3,17	Pitpnm1	PSR1900000340.mm.1	-4,55	0,005469	0,379939	Alternative 3' Acce	0,34
TC1900000027.mm.1	3,17	Pitpnm1	PSR1900000366.mm.1	-4,77	0,010589	0,42255	Alternative 3' Acce	0,38
TC1900000027.mm.1	3,17	Pitpnm1	JUC1900000173.mm.1	-6,27	0,003066	0,353892		
TC1900000027.mm.1	3,17	Pitpnm1	JUC1900000196.mm.1	-6,73	0,025226	0,496835		
TC0400003171.mm.1	3,85	Wdr78	JUC0400013116.mm.1	2,42	0,010625	0,422841		
TC0400003171.mm.1	3,85	Wdr78	PSR0400025192.mm.1	2,02	0,005561	0,380778	Cassette Exon	0,12

TC0400003171.mm.1	3,85	Wdr78	PSR0400025194.mm.1	-2,09	0,013794	0,444974	Alternative 5' Donc	0,18
TC0400003171.mm.1	3,85	Wdr78	PSR0400025193.mm.1	-2,15	0,015047	0,452154	Alternative 5' Donc	0,18
TC0400003171.mm.1	3,85	Wdr78	JUC0400013120.mm.1	-2,19	0,012254	0,433778		
TC0400003171.mm.1	3,85	Wdr78	JUC0400013127.mm.1	-2,44	0,018471	0,468729		
TC0400003171.mm.1	3,85	Wdr78	JUC0400013121.mm.1	-2,45	0,034139	0,524882		
TC0400003171.mm.1	3,85	Wdr78	PSR0400025202.mm.1	-2,52	0,002589	0,349501	Cassette Exon	0,34
TC0400003171.mm.1	3,85	Wdr78	PSR0400025203.mm.1	-3,55	0,002312	0,346468	Cassette Exon	0,33
TC0400003171.mm.1	3,85	Wdr78	JUC0400013128.mm.1	-4,47	0,009219	0,41411		
TC0400003171.mm.1	3,85	Wdr78	PSR0400025183.mm.1	-4,67	0,015476	0,45435	Cassette Exon	0,32
TC0400003171.mm.1	3,85	Wdr78	PSR0400025206.mm.1	-4,81	0,006361	0,389469	Alternative 5' Donc	0,37
TC0400003171.mm.1	3,85	Wdr78	JUC0400013129.mm.1	-7,13	0,032594	0,520272		
TC1200001949.mm.1	-1,46	4930426I24Rik	PSR1200013380.mm.1	2,42	0,0197	0,474887	Alternative 5' Donc	0,18
TC0400002601.mm.1	-1,18	Trim14	PSR0400021210.mm.1	2,42	0,045718	0,554833	Cassette Exon	0,14
TC0400002601.mm.1	-1,18	Trim14	JUC0400010987.mm.1	2,05	0,025117	0,496358		
TC0400004225.mm.1	1,38	Epha8	JUC0400016257.mm.1	2,42	0,03807	0,535633		
TC0400004225.mm.1	1,38	Epha8	PSR0400031287.mm.1	2,25	0,003726	0,357586	Cassette Exon	0,08
TC0400004225.mm.1	1,38	Epha8	PSR0400031284.mm.1	2,22	0,012321	0,434392	Cassette Exon	0,14
TC0400004225.mm.1	1,38	Epha8	JUC0400016256.mm.1	-2,39	0,000773	0,308853		
TC0400004225.mm.1	1,38	Epha8	JUC0400016258.mm.1	-2,81	0,014418	0,448786		
TC0800002958.mm.1	-1,85	Chst5	PSR0800022848.mm.1	2,42	0,014098	0,44706	Cassette Exon	0,13
TC0X00000694.mm.1	1,01	Srpk3	PSR0X00004534.mm.1	2,42	0,015182	0,452699	Cassette Exon	0,12
TC0900002898.mm.1	1,22	Clstn2	JUC0900013349.mm.1	2,42	0,000949	0,313363		
TC0900002898.mm.1	1,22	Clstn2	PSR0900023911.mm.1	-2,17	0,008807	0,409934	Cassette Exon	0,11
TC1600002055.mm.1	-1,22	Mis18a	JUC1600008084.mm.1	2,42	0,024003	0,492783		
TC0700004257.mm.1	-1,81		JUC0700018661.mm.1	2,42	0,033367	0,522601		
TC0800000336.mm.1	1,22	Rnf170	JUC0800001398.mm.1	2,42	0,045771	0,554863		
TC0900001155.mm.1	-1,7	Ctsh	JUC0900004958.mm.1	2,42	0,01792	0,466466		
TC0500003028.mm.1	-1,04	Sgsm1	JUC0500014746.mm.1	2,42	0,021188	0,481036		
TC0400002799.mm.1	1,2	Akna	JUC0400011984.mm.1	2,42	0,019051	0,471978		
TC0400004011.mm.1	-1,24	Gm13201	JUC0400017300.mm.1	2,42	0,039674	0,540322		
TC0X00002165.mm.1	1,01	Aifm1	JUC0X00006983.mm.1	2,42	0,020794	0,479488		
TC1100002932.mm.1	-1,6	Zswim7	PSR1100026680.mm.1	2,41	0,028032	0,505851	Cassette Exon	0,13
TC1100002932.mm.1	-1,6	Zswim7	JUC1100013912.mm.1	2,3	0,025592	0,497808		
TC1100002932.mm.1	-1,6	Zswim7	PSR1100026678.mm.1	2,02	0,001671	0,335182	Cassette Exon	0,24
TC0400002174.mm.1	2,24	Car8	JUC0400009492.mm.1	2,41	0,049739	0,563564		
TC0400002174.mm.1	2,24	Car8	PSR0400018182.mm.1	-3,24	0,000927	0,313363	Cassette Exon	0,21
TC0100000353.mm.1	1,49	Dnah7b; Dnah7c	JUC0100001522.mm.1	2,41	0,00947	0,416539		
TC0100000353.mm.1	1,49	Dnah7b; Dnah7c	PSR0100002674.mm.1	2,17	0,015151	0,452699	Cassette Exon	0,2
TC0100000353.mm.1	1,49	Dnah7b; Dnah7c	PSR0100002682.mm.1	2,03	0,032614	0,520338	Cassette Exon	0,1
TC0300000129.mm.1	-1,55	Dnajc5b	PSR0300000553.mm.1	2,41	0,011056	0,425887	Alternative 3' Acce	0,19
TC1100002374.mm.1	-1,71	Rel	PSR1100022380.mm.1	2,41	0,01939	0,47371	Alternative 3' Acce	0,19
TC1100002374.mm.1	-1,71	Rel	PSR1100022392.mm.1	2,2	0,032945	0,52122	Alternative 5' Donc	0,17
TC1700001642.mm.1	1,18	Ndufb10	PSR1700014712.mm.1	2,41	0,031629	0,517405	Alternative 5' Donc	0,19
TC0300000521.mm.1	1,65	Kcnab1	JUC0300001869.mm.1	2,41	0,032492	0,519646		
TC0300000521.mm.1	1,65	Kcnab1	JUC0300001872.mm.1	-2,09	0,00096	0,313363		
TC0300000521.mm.1	1,65	Kcnab1	PSR0300003565.mm.1	-2,33	0,042269	0,547092	Cassette Exon	0,16
TC0300000521.mm.1	1,65	Kcnab1	PSR0300003559.mm.1	-2,52	0,021499	0,482708	Cassette Exon	0,17
TC1600001590.mm.1	1,25	Stxbp5l	PSR1600013007.mm.1	2,41	0,041139	0,544	Cassette Exon	0,14

TC1600001590.mm.1	1,25	Stxbp5l	PSR1600012999.mm.1	-2,08	0,023109	0,489756	Alternative 3' Acce	0,16
TC0100000509.mm.1	-1,51	Nrp2	PSR0100004095.mm.1	2,41	0,013734	0,444758	Alternative 3' Acce	0,15
TC0900000493.mm.1	2,34	Gm3898	PSR0900003227.mm.1	2,41	0,009835	0,417839	Cassette Exon	0,15
TC0900000493.mm.1	2,34	Gm3898	PSR0900003225.mm.1	-2,05	0,037498	0,533968	Cassette Exon	0,1
TC0500001607.mm.1	-1,09	Myl10	PSR0500014536.mm.1	2,41	0,001017	0,316361	Cassette Exon	0,14
TC0500002819.mm.1	1,59	A930011G23Rik	PSR0500024766.mm.1	2,41	0,023807	0,491893	Cassette Exon	0,14
TC0500002819.mm.1	1,59	A930011G23Rik	JUC0500013473.mm.1	-2,52	0,012085	0,43255		
TC1100002895.mm.1	1,01	Top3a	PSR1100026250.mm.1	2,41	0,03982	0,540873	Cassette Exon	0,14
TC1300000705.mm.1	-2,24		PSR1300004653.mm.1	2,41	0,028484	0,50733	Cassette Exon	0,13
TC0600001403.mm.1	-1,36	Clec4n	PSR0600011323.mm.1	2,41	0,002123	0,342919	Cassette Exon	0,12
TC0600002861.mm.1	-2,07	Pdzrn3	JUC0600011426.mm.1	2,41	0,021556	0,483034		
TC0600002861.mm.1	-2,07	Pdzrn3	PSR0600022025.mm.1	2,1	0,024688	0,494744	Cassette Exon	0,12
TC1400002476.mm.1	1,11	Dgkh	JUC1400010494.mm.1	2,41	0,010335	0,420915		
TC1400002476.mm.1	1,11	Dgkh	JUC1400010517.mm.1	-2,22	0,046055	0,555632		
TC1400002476.mm.1	1,11	Dgkh	PSR1400019063.mm.1	-2,88	0,039595	0,539903	Cassette Exon	0,12
TC0300000467.mm.1	1,21	Med12l	JUC0300001699.mm.1	2,41	0,01403	0,44654		
TC0300000467.mm.1	1,21	Med12l	JUC0300001682.mm.1	2,08	0,036886	0,532257		
TC1500001912.mm.1	-1,81	Mkl1	JUC1500008531.mm.1	2,41	0,027031	0,502979		
TC1700000269.mm.1	-1,43	Zfp677	JUC1700001079.mm.1	2,41	0,033972	0,524252		
TC0100003399.mm.1	-1,14	Fmo4	JUC0100015935.mm.1	2,41	0,002372	0,34829		
TC0800001340.mm.1	-1,68		JUC0800006034.mm.1	2,41	0,011295	0,427117		
TC0500000178.mm.1	1,28		JUC0500000758.mm.1	2,41	0,035294	0,52837		
TC0500001153.mm.1	1,03	Ttc28	JUC0500005431.mm.1	2,41	0,00618	0,387873		
TC0700002033.mm.1	-1,38	Athl1	JUC0700009469.mm.1	2,41	0,017037	0,461729		
TC0700002033.mm.1	-1,38	Athl1	JUC0700009484.mm.1	2,29	0,022056	0,485327		
TC0400003116.mm.1	1,23	Tm2d1	JUC0400012948.mm.1	2,41	0,020043	0,476453		
TC0400003116.mm.1	1,23	Tm2d1	JUC0400012956.mm.1	-8,36	0,038645	0,537098		
TC0900002029.mm.1	-1,42	Gm5916; RP23-319C8.2	JUC0900009506.mm.1	2,41	0,005726	0,382767		
TC1000001190.mm.1	-1,82	Lum	JUC1000004825.mm.1	2,41	0,01852	0,469138		
TC1100004272.mm.1	1,76	sept-04	PSR1100012532.mm.1	2,4	0,003974	0,360082	Alternative 5' Donc	0,11
TC1100004272.mm.1	1,76	sept-04	JUC1100006579.mm.1	2,38	0,047536	0,558701		
TC1100004272.mm.1	1,76	sept-04	PSR1100012528.mm.1	2,2	0,015201	0,452738	Cassette Exon	0,18
TC0100000814.mm.1	1,07	Gigyf2	JUC0100003907.mm.1	2,4	0,048588	0,561078		
TC0100000814.mm.1	1,07	Gigyf2	PSR0100006850.mm.1	2,02	0,027028	0,502979	Cassette Exon	0,14
TC0100000814.mm.1	1,07	Gigyf2	PSR0100006858.mm.1	-2,08	0,00852	0,407614		
TC0100000814.mm.1	1,07	Gigyf2	PSR0100006859.mm.1	-2,68	0,009789	0,417839	Alternative 5' Donc	0,1
TC0100001617.mm.1	-1,97	Tagln2	PSR0100013485.mm.1	2,4	0,014977	0,452048	Cassette Exon	0,13
TC1800001577.mm.1	1	Smad4	PSR1800011413.mm.1	2,4	0,006971	0,395722	Cassette Exon	0,11
TC0500000669.mm.1	-1,65		PSR0500005905.mm.1	2,4	0,010916	0,424696	Cassette Exon	0,06
TC0200002477.mm.1	-1,15	Ift52	JUC0200010279.mm.1	2,4	0,01107	0,425887		
TC1200001251.mm.1	-1,28		JUC1200004888.mm.1	2,4	0,036426	0,530958		
TC1300002437.mm.1	-1,18	Bhmt2	JUC1300008600.mm.1	2,4	0,000223	0,28803		
TC1700002573.mm.1	1,23	Atl2	JUC1700012655.mm.1	2,4	0,022966	0,488789		
TC0100002602.mm.1	-1,19	Atg9a	JUC0100011971.mm.1	2,4	0,010588	0,42255		
TC0100002602.mm.1	-1,19	Atg9a	JUC0100011982.mm.1	2,23	0,029811	0,511132		
TC1700001738.mm.1	1,25	Grm4	JUC1700008626.mm.1	2,4	0,002997	0,353111		
TC1700001738.mm.1	1,25	Grm4	JUC1700008629.mm.1	-3,67	0,031127	0,515475		
TC0200001448.mm.1	-1,67	Trp53i11	JUC0200005614.mm.1	2,4	0,040124	0,541532		

TC0200002421.mm.1	1,21	Rprd1b	JUC0200009932.mm.1	2,4	0,033108	0,521786		
TC0900001474.mm.1	-1,39	Prss50	JUC0900006890.mm.1	2,4	0,002532	0,349501		
TC0500001548.mm.1	1,22	Gtf2ird2	JUC0500007622.mm.1	2,4	0,029091	0,509063		
TC0600002250.mm.1	-1,55	Osbpl3	JUC0600009273.mm.1	2,4	0,011681	0,429939		
TC0600003328.mm.1	-1,47		JUC0600013600.mm.1	2,4	0,010821	0,424068		
TC0700002850.mm.1	-1,52	C230052I12Rik	JUC0700013623.mm.1	2,4	0,026005	0,499257		
TC0700003581.mm.1	1,22	Idh2	JUC0700015955.mm.1	2,4	0,023326	0,490065		
TC0700003581.mm.1	1,22	Idh2	JUC0700015949.mm.1	-2,12	0,01995	0,476079		
TC0700001363.mm.1	-1,34	Gdpd4	JUC0700005985.mm.1	2,4	0,025387	0,497403		
TC1100001887.mm.1	-1,67	Tmem104	JUC1100009205.mm.1	2,4	0,049894	0,563773		
TC0900002745.mm.1	13,62	Impg1	JUC0900012705.mm.1	2,39	0,038062	0,535633		
TC0900002745.mm.1	13,62	Impg1	JUC0900012709.mm.1	-2,35	0,003292	0,354243		
TC0900002745.mm.1	13,62	Impg1	JUC0900012708.mm.1	-2,99	0,003019	0,353447		
TC0900002745.mm.1	13,62	Impg1	PSR0900022713.mm.1	-4,95	0,023297	0,49004	Cassette Exon	0,41
TC0900002745.mm.1	13,62	Impg1	JUC0900012706.mm.1	-6,68	0,001197	0,321568		
TC0900002745.mm.1	13,62	Impg1	JUC0900012720.mm.1	-7,18	0,004228	0,363295		
TC0900002745.mm.1	13,62	Impg1	PSR0900022730.mm.1	-11,49	0,004157	0,3623	Alternative 5' Donc	0,47
TC0500003399.mm.1	-1,5	Ccl24	JUC0500016732.mm.1	2,39	0,025293	0,497226		
TC0500003399.mm.1	-1,5	Ccl24	PSR0500030728.mm.1	2,34	0,003968	0,360063	Intron Retention	0,29
TC0300000923.mm.1	1,11	Otud7b	PSR0300007371.mm.1	2,39	0,017922	0,466466	Alternative 5' Donc	0,22
TC0300000923.mm.1	1,11	Otud7b	JUC0300003812.mm.1	-4,15	0,004275	0,363868		
TC0800000366.mm.1	1,3	Dusp26	PSR0800002796.mm.1	2,39	0,011339	0,427345	Alternative 5' Donc	0,22
TC1100000926.mm.1	-1,57	Plscr3	PSR1100007899.mm.1	2,39	0,038858	0,537834	Alternative 5' Donc	0,22
TC1100000926.mm.1	-1,57	Plscr3	PSR1100007884.mm.1	2,02	0,026951	0,502779	Cassette Exon	0,11
TC1100003923.mm.1	-1,38	Icam2	PSR1100036768.mm.1	2,39	0,041287	0,544179	Alternative 5' Donc	0,22
TC1800001060.mm.1	-1,42	Klhl14	PSR1800007814.mm.1	2,39	0,01119	0,426444	Alternative 5' Donc	0,22
TC1000000267.mm.1	-2,16		PSR1000001839.mm.1	2,39	0,045405	0,554085	Alternative 3' Acce	0,22
TC0X00002427.mm.1	-2,2	Trex2	PSR0X00015003.mm.1	2,39	0,020896	0,479701	Cassette Exon	0,13
TC1400002332.mm.1	-1,16	9930012K11Rik	JUC1400009953.mm.1	2,39	0,006281	0,388863		
TC1300001737.mm.1	-1,02	Exoc2	JUC1300005507.mm.1	2,39	0,033724	0,523549		
TC1300001737.mm.1	-1,02	Exoc2	JUC1300005517.mm.1	2,15	0,012252	0,433767		
TC1300000746.mm.1	1,05	Zfp808; BC052688; Gm199	JUC1300002600.mm.1	2,39	0,020997	0,480143		
TC1800000396.mm.1	1,18	Rnf14	JUC1800001650.mm.1	2,39	0,006542	0,391994		
TC1700001877.mm.1	-1,79	Angptl4	JUC1700009376.mm.1	2,39	0,048796	0,561497		
TC0100000535.mm.1	1,18	Creb1	JUC0100002522.mm.1	2,39	0,00752	0,401141		
TC0200001452.mm.1	-1,37	Gm10804	JUC0200005623.mm.1	2,39	0,021499	0,482708		
TC0500000513.mm.1	-1,56		JUC0500002672.mm.1	2,39	0,022619	0,487464		
TC0600001733.mm.1	-1,28	Fgfr1op2	JUC0600007185.mm.1	2,39	0,003493	0,355638		
TC0600001733.mm.1	-1,28	Fgfr1op2	JUC0600007180.mm.1	2,3	0,025014	0,496061		
TC0600001354.mm.1	-1,02	Slc25a18	JUC0600005627.mm.1	2,39	0,003535	0,3562		
TC0700003439.mm.1	1,13	Asb7	JUC0700015495.mm.1	2,39	0,013446	0,442693		
TC0400000438.mm.1	-1,51	Car9	JUC0400001453.mm.1	2,39	0,007243	0,39826		
TC1000002892.mm.1	-1,08	Tph2	JUC1000011995.mm.1	2,39	0,00863	0,408463		
TC1100002680.mm.1	1,86	Zfp879	JUC1100012684.mm.1	2,39	0,040529	0,542404		
TC1100001646.mm.1	-1,18	Wnk4	JUC1100007999.mm.1	2,39	0,024165	0,493245		
TC1000000145.mm.1	-1,53	Il20ra	JUC1000000514.mm.1	2,39	0,046178	0,555838		
TC1400001524.mm.1	2,19	Gng2	JUC1400006097.mm.1	2,38	0,017934	0,466474		
TC1400001524.mm.1	2,19	Gng2	PSR1400011349.mm.1	-2,01	0,048711	0,561355		

TC1400001524.mm.1	2,19	Gng2	PSR1400011347.mm.1	-2,24	0,040253	0,541756	Cassette Exon	0,07
TC1400001524.mm.1	2,19	Gng2	PSR1400011351.mm.1	-2,96	0,003563	0,356223	Alternative 5' Donc	0,28
TC1400001524.mm.1	2,19	Gng2	PSR1400011352.mm.1	-3,18	0,017846	0,466209	Alternative 5' Donc	0,28
TC1400001524.mm.1	2,19	Gng2	PSR1400011355.mm.1	-3,49	0,01691	0,460797	Alternative 5' Donc	0,28
TC1400001524.mm.1	2,19	Gng2	JUC1400006105.mm.1	-3,54	0,003666	0,357266		
TC1400001524.mm.1	2,19	Gng2	PSR1400011353.mm.1	-4,35	0,002925	0,352289	Alternative 5' Donc	0,28
TC0900002462.mm.1	-2,07		PSR0900020599.mm.1	2,38	0,011701	0,429969	Alternative 3' Acce	0,22
TC0900003332.mm.1	-1	Mthfs	PSR0900008987.mm.1	2,38	0,033004	0,521478	Alternative 5' Donc	0,21
TC0900003332.mm.1	-1	Mthfs	JUC0900004891.mm.1	-2,08	0,039018	0,538105		
TC1900000572.mm.1	-1,62	Entpd1	JUC1900002777.mm.1	2,38	0,005394	0,378916		
TC1900000572.mm.1	-1,62	Entpd1	PSR1900005214.mm.1	2,22	0,037307	0,533573	Alternative 5' Donc	0,18
TC1900000402.mm.1	1,25	Kank1	PSR1900003714.mm.1	2,38	0,031963	0,518173	Cassette Exon	0,17
TC1600000221.mm.1	-1,34	Smpd4	PSR1600001911.mm.1	2,38	0,048814	0,561539	Cassette Exon	0,15
TC0X00003043.mm.1	-1,4	Slc25a53	PSR0X00019073.mm.1	2,38	0,001854	0,337479	Cassette Exon	0,13
TC1000002330.mm.1	-3,18	1700040L02Rik	PSR1000016615.mm.1	2,38	0,010875	0,424174	Cassette Exon	0,13
TC0200003122.mm.1	-1,57	Arrdc1	JUC0200012984.mm.1	2,38	0,01808	0,46716		
TC1400000999.mm.1	1,68	Lgi3	JUC1400004304.mm.1	2,38	0,038526	0,536634		
TC0200002653.mm.1	-1,23		JUC0200010999.mm.1	2,38	0,045208	0,553564		
TC1400002057.mm.1	1,19	Prmt5	JUC1400008432.mm.1	2,38	0,028348	0,506843		
TC1400002864.mm.1	-1,27	Tm9sf1	JUC1400008749.mm.1	2,38	0,031729	0,517489		
TC0100002305.mm.1	-1,57	Nabp1	JUC0100010484.mm.1	2,38	0,003009	0,353447		
TC0200002315.mm.1	1,09	Asxl1	JUC0200009156.mm.1	2,38	0,011364	0,427452		
TC0200002371.mm.1	-1,11	Gm14256; 0610038P03Rik	JUC0200009565.mm.1	2,38	0,006297	0,389033		
TC1600000977.mm.1	-1,68		JUC1600003958.mm.1	2,38	0,035588	0,528831		
TC0700001122.mm.1	-1,45	Fanci	JUC0700005067.mm.1	2,38	0,001618	0,332831		
TC0700001122.mm.1	-1,45	Fanci	JUC0700005083.mm.1	-7,52	0,002726	0,349612		
TC0500003585.mm.1	-1,14	Smurf1	JUC0500017876.mm.1	2,38	0,000584	0,304044		
TC0400000051.mm.1	-1,39		JUC0400000145.mm.1	2,38	0,041513	0,544746		
TC1000002000.mm.1	-1,18	Nt5dc1	JUC1000007752.mm.1	2,38	0,029882	0,51129		
TC0800003169.mm.1	1,92	Pgbd5	JUC0800013287.mm.1	2,37	0,029981	0,51153		
TC0800003169.mm.1	1,92	Pgbd5	JUC0800013286.mm.1	2,29	0,02336	0,490143		
TC0800003169.mm.1	1,92	Pgbd5	PSR0800024342.mm.1	-2,07	0,016152	0,457969	Cassette Exon	0,1
TC0800003169.mm.1	1,92	Pgbd5	PSR0800024330.mm.1	-2,17	0,019628	0,474852	Cassette Exon	0,08
TC0800003169.mm.1	1,92	Pgbd5	PSR0800024334.mm.1	-2,26	0,011459	0,427962	Cassette Exon	0,05
TC0800003169.mm.1	1,92	Pgbd5	PSR0800024333.mm.1	-2,96	0,004108	0,36228	Cassette Exon	0,19
TC0800003169.mm.1	1,92	Pgbd5	PSR0800024332.mm.1	-3,29	0,000975	0,314364	Alternative 5' Donc	0,34
TC0800003169.mm.1	1,92	Pgbd5	JUC0800013290.mm.1	-3,36	0,002637	0,349612		
TC0100001438.mm.1	1,92	Astn1	JUC0100006753.mm.1	2,37	0,002349	0,34761		
TC0100001438.mm.1	1,92	Astn1	PSR0100011777.mm.1	-2,02	0,000926	0,313363	Cassette Exon	0,13
TC0100001438.mm.1	1,92	Astn1	JUC0100006774.mm.1	-2,04	0,006686	0,393201		
TC0100001438.mm.1	1,92	Astn1	PSR0100011766.mm.1	-2,26	0,004673	0,370181	Cassette Exon	0,25
TC0100001438.mm.1	1,92	Astn1	PSR0100011779.mm.1	-2,37	0,010421	0,42148	Cassette Exon	0,15
TC0100001438.mm.1	1,92	Astn1	JUC0100006761.mm.1	-2,38	0,032653	0,520431		
TC0100001438.mm.1	1,92	Astn1	PSR0100011761.mm.1	-2,43	0,006366	0,389469	Alternative 3' Acce	0,2
TC0100001438.mm.1	1,92	Astn1	PSR0100011760.mm.1	-2,44	0,01554	0,454617	Alternative 3' Acce	0,2
TC0100001438.mm.1	1,92	Astn1	JUC0100006772.mm.1	-2,68	0,005309	0,378059		
TC0100001438.mm.1	1,92	Astn1	PSR0100011786.mm.1	-3,67	0,023183	0,490016	Cassette Exon	0,3
TC0100001438.mm.1	1,92	Astn1	JUC0100006754.mm.1	-4,5	0,001254	0,322251		

TC1900001767.mm.1	-1,07	Slc22a29	PSR1900009907.mm.1	2,37	0,028265	0,506588	Cassette Exon	0,16
TC1900001767.mm.1	-1,07	Slc22a29	PSR1900009910.mm.1	-2,18	0,03009	0,511932	Intron Retention	0,29
TC0400001944.mm.1	1,07	A930028C08Rik; Ube4bos	PSR0400015988.mm.1	2,37	0,021831	0,484607	Cassette Exon	0,22
TC1700001933.mm.1	-1,46	1110038B12Rik; Snord52;	PSR1700018307.mm.1	2,37	0,029784	0,511132	Alternative 3' Acce	0,22
TC1600000620.mm.1	-1,2	BC002163	PSR1600005559.mm.1	2,37	0,031296	0,516173	Alternative 3' Acce	0,22
TC0800003208.mm.1	-1,03		PSR0800024662.mm.1	2,37	0,000539	0,304044	Alternative 3' Acce	0,2
TC0500000876.mm.1	-1,07	Fam47e	PSR0500007623.mm.1	2,37	0,019668	0,474887	Alternative 5' Donc	0,17
TC0600002381.mm.1	-1,37	Fam13a	PSR0600018448.mm.1	2,37	0,004462	0,36753	Cassette Exon	0,11
TC0600002381.mm.1	-1,37	Fam13a	PSR0600018452.mm.1	2,17	0,031533	0,51704	Alternative 5' Donc	0,14
TC0600002381.mm.1	-1,37	Fam13a	JUC0600009612.mm.1	2,12	0,001365	0,32572		
TC1100001809.mm.1	-1,05	Gm11657	PSR1100017050.mm.1	2,37	0,041726	0,545379	Cassette Exon	0,14
TC0300001488.mm.1	-1,93	Mcoln3	PSR0300012013.mm.1	2,37	0,020797	0,479489	Cassette Exon	0,12
TC0400001329.mm.1	-1,12	Lepre1	PSR0400010063.mm.1	2,37	0,013973	0,446239	Cassette Exon	0,12
TC0900001644.mm.1	-1,73	Topaz1	PSR0900013781.mm.1	2,37	0,040436	0,542058	Cassette Exon	0,07
TC1400002206.mm.1	-1,18	Gucy1b2	JUC1400009337.mm.1	2,37	0,009038	0,412748		
TC1200000978.mm.1	1,53	Ttc8	JUC1200003799.mm.1	2,37	0,045603	0,55464		
TC1200000978.mm.1	1,53	Ttc8	JUC1200003804.mm.1	-2,31	0,020452	0,478018		
TC0300001038.mm.1	1,45	Sike1	JUC0300004295.mm.1	2,37	0,029915	0,511499		
TC1200000411.mm.1	-1,4		JUC1200001603.mm.1	2,37	0,013396	0,442454		
TC0200004362.mm.1	-1,11	4930533B01Rik	JUC0200018654.mm.1	2,37	0,004015	0,361291		
TC1200002127.mm.1	1,08	Snw1	JUC1200008167.mm.1	2,37	0,027936	0,505612		
TC1700002273.mm.1	-1,1		JUC1700011366.mm.1	2,37	0,020935	0,479947		
TC1700000535.mm.1	1,11	Pde9a	JUC1700002704.mm.1	2,37	0,047269	0,558223		
TC0500002731.mm.1	-1,26	Rassf6	JUC0500013125.mm.1	2,37	0,026461	0,501252		
TC0600001095.mm.1	1	Fgd5	JUC0600004396.mm.1	2,37	0,039703	0,540417		
TC0600001095.mm.1	1	Fgd5	JUC0600004387.mm.1	2,25	0,021352	0,481836		
TC0600002342.mm.1	-1,17	Nt5c3	JUC0600009562.mm.1	2,37	0,000834	0,311886		
TC0700002373.mm.1	-1,07	Sult2a4	JUC0700011213.mm.1	2,37	0,026723	0,502096		
TC1100000211.mm.1	1,02	Vps54	JUC1100000902.mm.1	2,37	0,005268	0,378003		
TC1000000051.mm.1	-1,67	B430219N15Rik	JUC1000000220.mm.1	2,37	0,033059	0,521637		
TC0200001524.mm.1	3,32	Slc1a2	PSR0200011453.mm.1	2,36	0,001982	0,341091	Cassette Exon	0,03
TC0200001524.mm.1	3,32	Slc1a2	PSR0200011465.mm.1	2,22	0,001333	0,325349		
TC0200001524.mm.1	3,32	Slc1a2	JUC0200005718.mm.1	2,03	0,034661	0,526274		
TC0200001524.mm.1	3,32	Slc1a2	PSR0200011447.mm.1	-2,29	0,010577	0,422452	Cassette Exon	0,38
TC0200001524.mm.1	3,32	Slc1a2	PSR0200011437.mm.1	-2,88	0,009071	0,412827	Cassette Exon	0,44
TC0200001524.mm.1	3,32	Slc1a2	JUC0200005716.mm.1	-3,8	0,002554	0,349501		
TC0200001524.mm.1	3,32	Slc1a2	PSR0200011448.mm.1	-3,84	0,005554	0,380773	Cassette Exon	0,37
TC0200001524.mm.1	3,32	Slc1a2	PSR0200011436.mm.1	-3,92	0,000765	0,308403	Cassette Exon	0,44
TC0200001524.mm.1	3,32	Slc1a2	PSR0200011441.mm.1	-4,27	0,012453	0,43528	Cassette Exon	0,44
TC0200001524.mm.1	3,32	Slc1a2	JUC0200005727.mm.1	-5,08	0,008477	0,406791		
TC0200001524.mm.1	3,32	Slc1a2	PSR0200011431.mm.1	-5,3	0,005632	0,381532	Cassette Exon	0,28
TC0200001524.mm.1	3,32	Slc1a2	PSR0200011443.mm.1	-6,26	0,000553	0,304044	Cassette Exon	0,44
TC0200001524.mm.1	3,32	Slc1a2	JUC0200005739.mm.1	-6,6	0,020146	0,477043		
TC0200001524.mm.1	3,32	Slc1a2	JUC0200005720.mm.1	-6,8	0,002195	0,344814		
TC0200001524.mm.1	3,32	Slc1a2	PSR0200011439.mm.1	-6,96	0,001118	0,317328	Cassette Exon	0,44
TC0200001524.mm.1	3,32	Slc1a2	PSR0200011438.mm.1	-7,59	0,00182	0,336896	Cassette Exon	0,44
TC0200001524.mm.1	3,32	Slc1a2	JUC0200005736.mm.1	-8,66	0,014229	0,447947		
TC0200001524.mm.1	3,32	Slc1a2	JUC0200005735.mm.1	-8,67	0,011492	0,42815		

TC0200001524.mm.1	3,32	Slc1a2	JUC0200005730.mm.1	-9,84	0,003512	0,355749		
TC0200001524.mm.1	3,32	Slc1a2	JUC0200005722.mm.1	-13,04	0,000015	0,18968		
TC0200001524.mm.1	3,32	Slc1a2	JUC0200005734.mm.1	-15,82	0,001291	0,322541		
TC0100003739.mm.1	-1,45	Marc2	PSR0100030639.mm.1	2,36	0,045404	0,554085	Alternative 3' Acce	0,22
TC0100003739.mm.1	-1,45	Marc2	PSR0100030635.mm.1	2,16	0,011727	0,429969	Cassette Exon	0,12
TC0400000346.mm.1	1,7	Tmem215	PSR0400002109.mm.1	2,36	0,00003	0,229631	Alternative 5' Donc	0,22
TC1500001760.mm.1	-1,17	Scrib	PSR1500013351.mm.1	2,36	0,030801	0,514144	Alternative 5' Donc	0,2
TC1500001760.mm.1	-1,17	Scrib	JUC1500007591.mm.1	2,2	0,025341	0,497329		
TC0400001569.mm.1	1,1	Mecr	PSR0400012330.mm.1	2,36	0,009534	0,416602	Alternative 3' Acce	0,18
TC1300000460.mm.1	-1,37	Gcnt2	PSR1300002716.mm.1	2,36	0,026734	0,502096	Cassette Exon	0,14
TC1200001520.mm.1	-1,01	Gm20474	JUC1200005859.mm.1	2,36	0,044396	0,551441		
TC1200001520.mm.1	-1,01	Gm20474	PSR1200010611.mm.1	2,1	0,015151	0,452699	Cassette Exon	0,13
TC1200001520.mm.1	-1,01	Gm20474	JUC1200005856.mm.1	2,06	0,015882	0,456526		
TC0600000025.mm.1	-1,36	Asb4	PSR0600000267.mm.1	2,36	0,03094	0,514624	Cassette Exon	0,12
TC0600000025.mm.1	-1,36	Asb4	JUC0600000164.mm.1	-2,53	0,017051	0,461826		
TC0900001785.mm.1	-1,62	4931406C07Rik	PSR0900014986.mm.1	2,36	0,048753	0,561437	Cassette Exon	0,12
TC1200001686.mm.1	-1,06	Stxbp6	JUC1200006462.mm.1	2,36	0,044698	0,552206		
TC0200003699.mm.1	1,12	Lrp2	JUC0200016154.mm.1	2,36	0,035477	0,528724		
TC0200003699.mm.1	1,12	Lrp2	JUC0200016177.mm.1	2,03	0,009036	0,412748		
TC0100003889.mm.1	-1,55	Mnda	JUC0100016782.mm.1	2,36	0,049318	0,56273		
TC1700000110.mm.1	1,19	Park2	JUC1700000487.mm.1	2,36	0,005536	0,380557		
TC0100003507.mm.1	1,28	Uap1	JUC0100016350.mm.1	2,36	0,049734	0,563552		
TC1600001624.mm.1	1,04	Gap43	JUC1600006883.mm.1	2,36	0,023529	0,490947		
TC0800001103.mm.1	-1,16	Slc12a3	JUC0800004525.mm.1	2,36	0,014498	0,449528		
TC0900001299.mm.1	2,84	Rab6b	JUC0900005545.mm.1	2,36	0,033273	0,522325		
TC0900001299.mm.1	2,84	Rab6b	JUC0900005550.mm.1	-2,21	0,008266	0,405976		
TC0500001470.mm.1	-1,25		JUC0500007303.mm.1	2,36	0,017769	0,465467		
TC0700000016.mm.1	-1,45	Cnot3; Mir3572	JUC0700000079.mm.1	2,36	0,04045	0,542094		
TC0700001174.mm.1	-1,07	Whamm	JUC0700005341.mm.1	2,36	0,045482	0,554313		
TC0600002975.mm.1	-1,11	Alox5	JUC0600011886.mm.1	2,36	0,047323	0,558291		
TC0700002001.mm.1	-1,54	Prap1	JUC0700009343.mm.1	2,36	0,041874	0,545696		
TC0700002102.mm.1	-1,27	Dhcr7	JUC0700010064.mm.1	2,36	0,013578	0,443703		
TC0300002800.mm.1	-1,31	Slc30a7	JUC0300011486.mm.1	2,36	0,031091	0,515208		
TC0300002800.mm.1	-1,31	Slc30a7	JUC0300011487.mm.1	2,07	0,039849	0,540922		
TC0400000873.mm.1	1,23	Acer2	JUC0400003039.mm.1	2,36	0,002645	0,349612		
TC0400002762.mm.1	-1,16	Inip	JUC0400011657.mm.1	2,36	0,015876	0,456526		
TC0100003241.mm.1	-1,23	Hmcn1	PSR0100026324.mm.1	2,35	0,020801	0,479493	Cassette Exon	0,02
TC0100003241.mm.1	-1,23	Hmcn1	JUC0100014959.mm.1	2,3	0,034491	0,525929		
TC0100003241.mm.1	-1,23	Hmcn1	JUC0100014945.mm.1	2,14	0,023743	0,491602		
TC0100003241.mm.1	-1,23	Hmcn1	PSR0100026311.mm.1	-2,23	0,002235	0,34578	Cassette Exon	0,13
TC0100003241.mm.1	-1,23	Hmcn1	PSR0100026306.mm.1	-3,05	0,040124	0,541532	Cassette Exon	0,29
TC0100003241.mm.1	-1,23	Hmcn1	JUC0100014995.mm.1	-3,09	0,030577	0,513415		
TC1400000772.mm.1	2,64	Thtpa; Zfhx2os; Gm20687;	PSR1400006023.mm.1	2,35	0,000521	0,303623	Alternative 3' Acce	0,22
TC1400000772.mm.1	2,64	Thtpa; Zfhx2os; Gm20687;	PSR1400006026.mm.1	-2,14	0,019091	0,47221	Alternative 5' Donc	0,02
TC1400000772.mm.1	2,64	Thtpa; Zfhx2os; Gm20687;	JUC1400003202.mm.1	-2,16	0,048222	0,560225		
TC1400000772.mm.1	2,64	Thtpa; Zfhx2os; Gm20687;	PSR1400006018.mm.1	-2,2	0,001733	0,335996	Cassette Exon	0,19
TC1400000772.mm.1	2,64	Thtpa; Zfhx2os; Gm20687;	PSR1400006019.mm.1	-2,27	0,000414	0,297771	Cassette Exon	0,19
TC1400000772.mm.1	2,64	Thtpa; Zfhx2os; Gm20687;	PSR1400006024.mm.1	-2,35	0,000521	0,303623		

TC1400000772.mm.1	2,64	Thtpa; Zfhx2os; Gm20687;	PSR1400006012.mm.1	-2,38	0,027591	0,504485	Cassette Exon	0,13
TC1400000772.mm.1	2,64	Thtpa; Zfhx2os; Gm20687;	PSR1400006036.mm.1	-2,51	0,002969	0,352501	Alternative 3' Acce	0,21
TC1400000772.mm.1	2,64	Thtpa; Zfhx2os; Gm20687;	PSR1400006039.mm.1	-2,61	0,000221	0,28803	Alternative 3' Acce	0,21
TC1400000772.mm.1	2,64	Thtpa; Zfhx2os; Gm20687;	PSR1400006028.mm.1	-2,67	0,000153	0,272178	Cassette Exon	0,23
TC1400000772.mm.1	2,64	Thtpa; Zfhx2os; Gm20687;	PSR1400006037.mm.1	-2,69	0,003154	0,353892	Alternative 3' Acce	0,21
TC1400000772.mm.1	2,64	Thtpa; Zfhx2os; Gm20687;	PSR1400006027.mm.1	-2,72	0,010947	0,425002	Alternative 5' Donc	0,08
TC1400000772.mm.1	2,64	Thtpa; Zfhx2os; Gm20687;	PSR1400006038.mm.1	-3,07	0,002326	0,346856	Cassette Exon	0,25
TC1400000772.mm.1	2,64	Thtpa; Zfhx2os; Gm20687;	PSR1400006022.mm.1	-3,4	0,005886	0,383835	Cassette Exon	0,18
TC1400000772.mm.1	2,64	Thtpa; Zfhx2os; Gm20687;	PSR1400006015.mm.1	-3,61	0,00162	0,332831	Cassette Exon	0,29
TC1400000772.mm.1	2,64	Thtpa; Zfhx2os; Gm20687;	JUC1400003217.mm.1	-4,14	0,026544	0,501435		
TC0100001585.mm.1	1,17	Dedd	PSR0100013030.mm.1	2,35	0,002475	0,349135	Cassette Exon	0,17
TC0800001076.mm.1	1,05	Irx6	PSR0800008263.mm.1	2,35	0,020108	0,47686	Cassette Exon	0,17
TC0900001431.mm.1	-1,69	Shisa5	PSR0900011828.mm.1	2,35	0,038654	0,537102	Alternative 3' Acce	0,17
TC0900001431.mm.1	-1,69	Shisa5	PSR0900011834.mm.1	2,08	0,001266	0,322251	Alternative 5' Donc	0,16
TC0900001431.mm.1	-1,69	Shisa5	PSR0900011810.mm.1	2,05	0,030358	0,512923	Alternative 3' Acce	0,17
TC0200003880.mm.1	-1,79	Serping1	PSR0200033581.mm.1	2,35	0,034743	0,526574	Alternative 5' Donc	0,16
TC0700001620.mm.1	1,39	Ipo7; Snora23	PSR0700013430.mm.1	2,35	0,022379	0,486433	Cassette Exon	0,15
TC0X00000366.mm.1	-1,05	Gm7590	PSR0X00002492.mm.1	2,35	0,040359	0,541949	Cassette Exon	0,15
TC0600002425.mm.1	-1,31	Il23r	PSR0600018626.mm.1	2,35	0,025395	0,497403	Cassette Exon	0,14
TC1900000733.mm.1	-1,07	Sorcs3	PSR1900006547.mm.1	2,35	0,002538	0,349501	Cassette Exon	0,13
TC0100002308.mm.1	1,56	Myo1b	JUC0100010511.mm.1	2,35	0,005176	0,376851		
TC0100002308.mm.1	1,56	Myo1b	PSR0100018556.mm.1	2,14	0,029372	0,509995	Cassette Exon	0,12
TC0100002308.mm.1	1,56	Myo1b	JUC0100010501.mm.1	-2,12	0,007034	0,39605		
TC0500001225.mm.1	-1,23	Mvk	PSR0500010514.mm.1	2,35	0,045387	0,554078	Cassette Exon	0,08
TC0500001225.mm.1	-1,23	Mvk	JUC0500005770.mm.1	2,15	0,022532	0,487133		
TC0200002530.mm.1	1,55	Dnttip1	JUC0200010494.mm.1	2,35	0,003411	0,355242		
TC0200002530.mm.1	1,55	Dnttip1	JUC0200010503.mm.1	-2,69	0,008894	0,410717		
TC1200002299.mm.1	1,43	Syne3	JUC1200008873.mm.1	2,35	0,046054	0,555632		
TC1200002299.mm.1	1,43	Syne3	JUC1200008876.mm.1	2,24	0,027353	0,503805		
TC1700002502.mm.1	5,05	BC027072	PSR1700022986.mm.1	2,35	0,024531	0,493992		
TC1700002502.mm.1	5,05	BC027072	JUC1700012300.mm.1	-6,37	0,001717	0,335996		
TC0100003253.mm.1	-1,03	Swt1	JUC0100015048.mm.1	2,35	0,01419	0,447608		
TC0100001703.mm.1	1,22	Cdc42bpa	JUC0100008006.mm.1	2,35	0,037808	0,534767		
TC0100001703.mm.1	1,22	Cdc42bpa	JUC0100007993.mm.1	2,18	0,025158	0,496555		
TC1600000088.mm.1	1,22	Mettl22	JUC1600000291.mm.1	2,35	0,00566	0,381922		
TC0200002329.mm.1	-1,21	Bpifb3	JUC0200009273.mm.1	2,35	0,012766	0,43712		
TC1700002765.mm.1	-1,32	T2	JUC1700000397.mm.1	2,35	0,007505	0,401045		
TC1600000472.mm.1	-1,25	Slc12a8	JUC1600002331.mm.1	2,35	0,004777	0,371044		
TC0200000461.mm.1	-1,14	Adamts13	JUC0200001457.mm.1	2,35	0,033784	0,523756		
TC0500002182.mm.1	1,42	Ift172	JUC0500010912.mm.1	2,35	0,021517	0,48281		
TC0500002895.mm.1	-1,37	Gbp10; Gbp6	JUC0500013947.mm.1	2,35	0,026534	0,501411		
TC0700001398.mm.1	-1,1	Xrra1	JUC0700006133.mm.1	2,35	0,033098	0,521753		
TC0700002372.mm.1	-1,17	Sult2a1	JUC0700011210.mm.1	2,35	0,030074	0,511908		
TC0300002972.mm.1	-1,85	Npnt	JUC0300012245.mm.1	2,35	0,041716	0,545356		
TC0300002972.mm.1	-1,85	Npnt	JUC0300012237.mm.1	-2,06	0,023954	0,4926		
TC0300003052.mm.1	-1,14	Gm15540	JUC0300012550.mm.1	2,35	0,028498	0,507345		
TC0300001984.mm.1	-1,08	Ufm1	JUC0300007915.mm.1	2,35	0,018787	0,470789		
TC0X00001367.mm.1	1,15	Mum1l1	JUC0X00004214.mm.1	2,35	0,044541	0,551915		

TC1400001540.mm.1	1,43	Ppp3cb	JUC1400006206.mm.1	2,34	0,002266	0,34578		
TC1400001540.mm.1	1,43	Ppp3cb	PSR1400011509.mm.1	-3,41	0,005975	0,386028	Alternative 5' Donc	0,39
TC0400004055.mm.1	-1,16	Spsb1	JUC0400017588.mm.1	2,34	0,025167	0,496555		
TC0400004055.mm.1	-1,16	Spsb1	PSR0400033723.mm.1	-2,42	0,010589	0,42255	Alternative 5' Donc	0,29
TC0600002742.mm.1	1,43	Zfyve20	JUC0600011067.mm.1	2,34	0,007662	0,402377		
TC0600002742.mm.1	1,43	Zfyve20	PSR0600021340.mm.1	-2,81	0,002179	0,344453	Alternative 5' Donc	0,29
TC0700003426.mm.1	2,33	Gm7546	PSR0700029208.mm.1	2,34	0,012737	0,436709	Cassette Exon	0,12
TC0700003426.mm.1	2,33	Gm7546	PSR0700029212.mm.1	-2,63	0,017547	0,464139	Alternative 5' Donc	0,26
TC0200003526.mm.1	-1,59	Rbm43	JUC0200015107.mm.1	2,34	0,012996	0,439291		
TC0200003526.mm.1	-1,59	Rbm43	PSR0200029950.mm.1	2,14	0,025567	0,497808	Alternative 3' Acce	0,17
TC0500000238.mm.1	1,86	Actr3b	PSR0500001837.mm.1	2,34	0,002635	0,349612	Cassette Exon	0,15
TC0500000238.mm.1	1,86	Actr3b	PSR0500001845.mm.1	-2,1	0,003087	0,353892	Cassette Exon	0,1
TC0500000238.mm.1	1,86	Actr3b	JUC0500001045.mm.1	-2,79	0,009326	0,414973		
TC0900001020.mm.1	1,4	Bmp5	JUC0900004380.mm.1	2,34	0,020766	0,479303		
TC0900001020.mm.1	1,4	Bmp5	PSR0900008063.mm.1	-2,5	0,036153	0,529844	Cassette Exon	0,14
TC0200001583.mm.1	1,13	Pax6	JUC0200005939.mm.1	2,34	0,017373	0,463612		
TC0200001583.mm.1	1,13	Pax6	JUC0200005909.mm.1	2,14	0,037802	0,534741		
TC0200001583.mm.1	1,13	Pax6	PSR0200011894.mm.1	-2,01	0,046295	0,556186	Alternative 5' Donc	0,13
TC1500000031.mm.1	-2,03	Dab2; LOC101055883	PSR1500000249.mm.1	2,34	0,042672	0,548097	Alternative 3' Acce	0,13
TC1300001546.mm.1	-1,53	Zfp389	PSR1300009783.mm.1	2,34	0,041613	0,545095	Cassette Exon	0,12
TC1300000175.mm.1	-4,17	Hist1h2bm	PSR1300001172.mm.1	2,34	0,015568	0,454866	Cassette Exon	0,12
TC0600000344.mm.1	1,08	Nup205	JUC0600001302.mm.1	2,34	0,017212	0,462545		
TC0600000344.mm.1	1,08	Nup205	PSR0600002644.mm.1	-2,01	0,020026	0,476453	Alternative 5' Donc	0,11
TC1200000263.mm.1	-1,23	Lamb1	JUC1200001172.mm.1	2,34	0,047178	0,558033		
TC1500001868.mm.1	1,11	Pla2g6	JUC1500008311.mm.1	2,34	0,00564	0,381609		
TC0200002146.mm.1	1,16	Pet117; Csrp2bp	JUC0200008503.mm.1	2,34	0,018418	0,468484		
TC0100003892.mm.1	-1,17	Gm1305	JUC0100017016.mm.1	2,34	0,010539	0,422281		
TC0100003892.mm.1	-1,17	Gm1305	JUC0100017023.mm.1	2,21	0,043134	0,548664		
TC1700000629.mm.1	-1,08	Btnl2	JUC1700003339.mm.1	2,34	0,020084	0,4766		
TC0100003443.mm.1	1,14	Dcaf6	JUC0100016137.mm.1	2,34	0,007946	0,403819		
TC1100001730.mm.1	-1,07	Itgb3	JUC1100008481.mm.1	2,34	0,015026	0,452111		
TC0700000989.mm.1	1,12	Snrpa1	JUC0700004657.mm.1	2,34	0,017413	0,46377		
TC0700000936.mm.1	1,34	Herc2	JUC0700004353.mm.1	2,34	0,021711	0,484104		
TC0700000936.mm.1	1,34	Herc2	JUC0700004361.mm.1	-2,39	0,007158	0,397855		
TC0700000936.mm.1	1,34	Herc2	JUC0700004297.mm.1	-2,45	0,012555	0,435721		
TC0600002782.mm.1	-1,33	AY512915	JUC0600011218.mm.1	2,34	0,003429	0,355303		
TC0300001289.mm.1	1,23	Zgrf1	JUC0300005477.mm.1	2,34	0,007696	0,402552		
TC0900002688.mm.1	-1,53	Fam83b	JUC0900012465.mm.1	2,34	0,029311	0,509874		
TC0900002735.mm.1	-1,68	Col12a1	JUC0900012622.mm.1	2,34	0,012916	0,438335		
TC0900002735.mm.1	-1,68	Col12a1	JUC0900012656.mm.1	-2,23	0,044008	0,550779		
TC1000001079.mm.1	-1,17	Gnptab	JUC1000004385.mm.1	2,34	0,048762	0,561437		
TC1000001079.mm.1	-1,17	Gnptab	JUC1000004366.mm.1	2,03	0,019278	0,473074		
TC0Y00000291.mm.1	1,1	Gm20858	JUC0Y00001233.mm.1	2,34	0,020478	0,478018		
TC0X00002447.mm.1	-1,04	Flna	JUC0X00007932.mm.1	2,34	0,031041	0,515011		
TC0Y00000285.mm.1	1,1	Gm20857; Gm20858	JUC0Y00001197.mm.1	2,34	0,020478	0,478018		
TC0Y00000288.mm.1	1,1	Gm20858	JUC0Y00001215.mm.1	2,34	0,020478	0,478018		
TC0Y00000290.mm.1	1,1	Gm20858	JUC0Y00001224.mm.1	2,34	0,020478	0,478018		
TC0600003135.mm.1	1,07	Gapdh; Gm20899	PSR0600024459.mm.1	2,33	0,008271	0,405976	Alternative 3' Acce	0,18

TC0600003135.mm.1	1,07	Gapdh; Gm20899	PSR0600024446.mm.1	2,3	0,002015	0,34123	Intron Retention	0,33
TC0600003135.mm.1	1,07	Gapdh; Gm20899	PSR0600024458.mm.1	2,14	0,016546	0,459612	Alternative 5' Dono	0,18
TC0600003135.mm.1	1,07	Gapdh; Gm20899	JUC0600012734.mm.1	2,06	0,006256	0,388622		
TC0600003135.mm.1	1,07	Gapdh; Gm20899	PSR0600024465.mm.1	2,01	0,012283	0,434168	Cassette Exon	0,1
TC0400003956.mm.1	1,3	Vps13d	JUC0400017081.mm.1	2,33	0,028957	0,508766		
TC0400003956.mm.1	1,3	Vps13d	PSR0400032781.mm.1	-2,17	0,001345	0,325349	Cassette Exon	0,04
TC0400003956.mm.1	1,3	Vps13d	PSR0400032764.mm.1	-2,61	0,002035	0,341377	Alternative 3' Acce	0,24
TC0400003956.mm.1	1,3	Vps13d	JUC0400017103.mm.1	-2,69	0,000767	0,308403		
TC0100003541.mm.1	-1,32	Nit1	PSR0100029053.mm.1	2,33	0,008268	0,405976	Cassette Exon	0,12
TC0100003541.mm.1	-1,32	Nit1	PSR0100029068.mm.1	2,02	0,04928	0,562691	Intron Retention	0,23
TC0100002380.mm.1	-1,61		PSR0100019324.mm.1	2,33	0,000163	0,272566	Alternative 3' Acce	0,21
TC0200001264.mm.1	-1,1	Olfr1033	PSR0200009985.mm.1	2,33	0,032992	0,521365	Alternative 5' Dono	0,21
TC1300002143.mm.1	-1,23	Ctla2a	PSR1300013751.mm.1	2,33	0,008489	0,406944	Alternative 3' Acce	0,21
TC1400000882.mm.1	-1,53	Fam124a	PSR1400007106.mm.1	2,33	0,040289	0,541823	Alternative 3' Acce	0,17
TC1000001282.mm.1	-1,21	E2f7	PSR1000009576.mm.1	2,33	0,012081	0,43255	Alternative 3' Acce	0,15
TC1600000244.mm.1	-1,13	Gnb1l; Gm16314	PSR1600002243.mm.1	2,33	0,037713	0,534585	Cassette Exon	0,14
TC1600000244.mm.1	-1,13	Gnb1l; Gm16314	JUC1600001174.mm.1	2,32	0,004763	0,371039		
TC0400002709.mm.1	-1,55	Tmem245	PSR0400021915.mm.1	2,33	0,043846	0,55048	Cassette Exon	0,13
TC0400002709.mm.1	-1,55	Tmem245	PSR0400021887.mm.1	2,32	0,005294	0,378059	Alternative 5' Dono	0,04
TC0400002709.mm.1	-1,55	Tmem245	JUC0400011352.mm.1	2,22	0,046111	0,555792		
TC0400001152.mm.1	-1,09	Cc2d1b	PSR0400008197.mm.1	2,33	0,039962	0,540973	Cassette Exon	0,12
TC0900002799.mm.1	1,16	Snhg5; Gm25122; Gm244	PSR0900023256.mm.1	2,33	0,038905	0,537874	Cassette Exon	0,12
TC0400002063.mm.1	1,62	Pank4	JUC0400008947.mm.1	2,33	0,042028	0,546158		
TC0400002063.mm.1	1,62	Pank4	JUC0400008944.mm.1	2,28	0,043021	0,54841		
TC0400002063.mm.1	1,62	Pank4	JUC0400008943.mm.1	-2,2	0,036623	0,531548		
TC0400002063.mm.1	1,62	Pank4	PSR0400017009.mm.1	-2,28	0,023556	0,490947	Intron Retention	0,11
TC0400002063.mm.1	1,62	Pank4	JUC0400008955.mm.1	-2,49	0,022996	0,488935		
TC0700002038.mm.1	-1,8	Pkp3	PSR0700017953.mm.1	2,33	0,044698	0,552206	Alternative 3' Acce	0,1
TC0700002038.mm.1	-1,8	Pkp3	JUC0700009528.mm.1	2,12	0,040377	0,541959		
TC0900000742.mm.1	2,09	Hcn4	JUC0900002891.mm.1	2,33	0,033949	0,524252		
TC0900000742.mm.1	2,09	Hcn4	PSR0900005471.mm.1	-2,09	0,038145	0,535762	Cassette Exon	0,1
TC1200001450.mm.1	1,55	Trib2	JUC1200005589.mm.1	2,33	0,01119	0,426444		
TC1200001450.mm.1	1,55	Trib2	PSR1200010141.mm.1	2,06	0,016348	0,458641	Cassette Exon	0,1
TC1200001450.mm.1	1,55	Trib2	PSR1200010140.mm.1	-2,02	0,046105	0,555758		
TC0300002312.mm.1	-1,57	Sema4a; Mir7011; mmu-n	JUC0300009230.mm.1	2,33	0,025452	0,497549		
TC0300002312.mm.1	-1,57	Sema4a; Mir7011; mmu-n	PSR0300017581.mm.1	2,12	0,047308	0,558289	Cassette Exon	0,09
TC0700002475.mm.1	1,8	Ppm1n	PSR0700021787.mm.1	2,33	0,002995	0,353092	Cassette Exon	0,04
TC0700002475.mm.1	1,8	Ppm1n	JUC0700011613.mm.1	-2,78	0,017452	0,463886		
TC0200002500.mm.1	-1,15	Pabpc1l	JUC0200010377.mm.1	2,33	0,028317	0,506741		
TC1100003830.mm.1	-1,04	Atxn7l3	JUC1100018601.mm.1	2,33	0,042244	0,547034		
TC1300002201.mm.1	1,02	Aaed1	JUC1300007401.mm.1	2,33	0,024934	0,495842		
TC1300002201.mm.1	1,02	Aaed1	JUC1300007402.mm.1	-3,43	0,044228	0,55119		
TC1800000727.mm.1	1,28	Rnmt	JUC1800002956.mm.1	2,33	0,007549	0,401384		
TC1800000727.mm.1	1,28	Rnmt	PSR1800005305.mm.1	-2,15	0,001976	0,340886		
TC1800000727.mm.1	1,28	Rnmt	JUC1800002964.mm.1	-9,78	0,008598	0,408294		
TC1500001568.mm.1	-1,63	Atad2	JUC1500006671.mm.1	2,33	0,014969	0,452048		
TC0900000867.mm.1	-1,06	Herc1	JUC0900003582.mm.1	2,33	0,048415	0,560736		
TC0800002558.mm.1	-1,39	Cd97; Mir1668; mmu-mir-	JUC0800010617.mm.1	2,33	0,004413	0,366761		

TC0700001439.mm.1	-1,21	Phox2a	JUC0700006430.mm.1	2,33	0,009696	0,417696		
TC0600000180.mm.1	-1,05	Ing3	JUC0600000552.mm.1	2,33	0,047028	0,557783		
TC1100000694.mm.1	1,04	Trim58	JUC1100002893.mm.1	2,33	0,014366	0,448293		
TC0400000993.mm.1	-1,14	Angptl3	JUC0400003425.mm.1	2,33	0,01722	0,462614		
TC1100003083.mm.1	1,32	Acadvl	JUC1100014888.mm.1	2,33	0,028076	0,506011		
TC1100003083.mm.1	1,32	Acadvl	JUC1100014870.mm.1	-2,13	0,016336	0,458616		
TC0100001860.mm.1	2,41	Rd3	JUC0100008770.mm.1	2,32	0,044608	0,552129		
TC0100001860.mm.1	2,41	Rd3	PSR0100015405.mm.1	-2,38	0,044029	0,550824	Alternative 3' Acce	0,22
TC0100001860.mm.1	2,41	Rd3	JUC0100008768.mm.1	-4,28	0,042728	0,548097		
TC0100001860.mm.1	2,41	Rd3	PSR0100015408.mm.1	-4,51	0,027601	0,504485	Cassette Exon	0,39
TC0200004602.mm.1	-1,96	Il1b	PSR0200039424.mm.1	2,32	0,009602	0,417256	Cassette Exon	0,21
TC0600002127.mm.1	1,01	Braf	PSR0600016831.mm.1	2,32	0,01091	0,424541	Cassette Exon	0,21
TC1100003821.mm.1	1,42	Mpp2	PSR1100035432.mm.1	2,32	0,039332	0,539324	Alternative 3' Acce	0,21
TC0100001696.mm.1	-1,54	A730054J21Rik	PSR0100014009.mm.1	2,32	0,002599	0,349501	Alternative 3' Acce	0,2
TC1000002616.mm.1	-1,22	Syn3	PSR1000019725.mm.1	2,32	0,032903	0,521149	Alternative 3' Acce	0,1
TC1000002616.mm.1	-1,22	Syn3	JUC1000010782.mm.1	2,2	0,03725	0,53345		
TC1000002616.mm.1	-1,22	Syn3	JUC1000010788.mm.1	2,06	0,013445	0,442693		
TC0400002386.mm.1	1,09	Orc3	PSR0400019347.mm.1	2,32	0,043772	0,550398	Cassette Exon	0,07
TC0400002386.mm.1	1,09	Orc3	JUC0400010086.mm.1	-2,02	0,035535	0,528764		
TC0400002386.mm.1	1,09	Orc3	JUC0400010069.mm.1	-2,14	0,002973	0,352501		
TC0200002879.mm.1	1,34	Cdc123	PSR0200023898.mm.1	2,32	0,04856	0,561008	Cassette Exon	0,06
TC1400002182.mm.1	-1,11	Sgcg	PSR1400016898.mm.1	2,32	0,003752	0,357586		
TC1400002182.mm.1	-1,11	Sgcg	JUC1400009249.mm.1	-2,21	0,042262	0,547092		
TC1300002086.mm.1	-1,05	Spock1	JUC1300006832.mm.1	2,32	0,03688	0,532257		
TC0100003120.mm.1	-1,11	Ptprv	JUC0100014334.mm.1	2,32	0,047075	0,557792		
TC0100003120.mm.1	-1,11	Ptprv	JUC0100014323.mm.1	-2,06	0,040837	0,543283		
TC1700001693.mm.1	1,42	Itfg3	JUC1700008548.mm.1	2,32	0,014824	0,45116		
TC1900000951.mm.1	1,44	Cnih2	JUC1900004608.mm.1	2,32	0,008018	0,404221		
TC1500001167.mm.1	-1,29	Osmr	JUC1500005268.mm.1	2,32	0,021847	0,484607		
TC1500000255.mm.1	-1,3		JUC1500001129.mm.1	2,32	0,044683	0,552176		
TC1500000485.mm.1	-1,08	Phf20l1	JUC1500002003.mm.1	2,32	0,046744	0,556856		
TC0800000882.mm.1	1,3	Otud4	JUC0800003499.mm.1	2,32	0,049623	0,563422		
TC0900001504.mm.1	1,8	Clasp2	JUC0900007049.mm.1	2,32	0,039936	0,540973		
TC0900001504.mm.1	1,8	Clasp2	JUC0900007063.mm.1	2,18	0,0128	0,437279		
TC0900001504.mm.1	1,8	Clasp2	JUC0900007067.mm.1	-2,25	0,000409	0,297771		
TC0900001504.mm.1	1,8	Clasp2	JUC0900007037.mm.1	-2,32	0,031052	0,515022		
TC0600000711.mm.1	-1,14	Abcg2; 4930430M16Rik	JUC0600002741.mm.1	2,32	0,002877	0,352207		
TC0600002551.mm.1	-1,04		JUC0600010262.mm.1	2,32	0,005183	0,376851		
TC0500003696.mm.1	-1,53		JUC0500018316.mm.1	2,32	0,002926	0,352289		
TC0700002053.mm.1	-1,4	Pnpla2	JUC0700009644.mm.1	2,32	0,024196	0,493294		
TC1100001684.mm.1	-1,29	BC030867	JUC1100008210.mm.1	2,32	0,018176	0,467441		
TC0900002269.mm.1	-1,13	Bco2	JUC0900010629.mm.1	2,32	0,006416	0,389881		
TC1100002553.mm.1	3,96	Gabrg2	JUC1100012309.mm.1	2,31	0,012588	0,435924		
TC1100002553.mm.1	3,96	Gabrg2	PSR1100023639.mm.1	2,08	0,001599	0,332546	Cassette Exon	0,12
TC1100002553.mm.1	3,96	Gabrg2	PSR1100023641.mm.1	2,06	0,007365	0,39938	Cassette Exon	0,15
TC1100002553.mm.1	3,96	Gabrg2	PSR1100023624.mm.1	-2,07	0,009607	0,417256	Alternative 3' Acce	0,08
TC1100002553.mm.1	3,96	Gabrg2	PSR1100023643.mm.1	-2,6	0,02632	0,500739	Alternative 5' Donc	0,19
TC1100002553.mm.1	3,96	Gabrg2	PSR1100023632.mm.1	-3,25	0,015604	0,455078	Alternative 3' Acce	0,36

TC1100002553.mm.1	3,96	Gabrg2	PSR1100023634.mm.1	-3,43	0,008625	0,408463	Alternative 5' Donc	0,39
TC1100002553.mm.1	3,96	Gabrg2	PSR1100023645.mm.1	-3,62	0,013962	0,446239	Alternative 5' Donc	0,42
TC1100002553.mm.1	3,96	Gabrg2	PSR1100023635.mm.1	-3,96	0,002486	0,349135	Alternative 3' Acce	0,47
TC1100002553.mm.1	3,96	Gabrg2	PSR1100023629.mm.1	-4,46	0,004668	0,370181		
TC1100002553.mm.1	3,96	Gabrg2	PSR1100023647.mm.1	-11,84	0,001961	0,340561	Alternative 5' Donc	0,37
TC1100002553.mm.1	3,96	Gabrg2	JUC1100012311.mm.1	-16,07	0,003389	0,3547		
TC1100002553.mm.1	3,96	Gabrg2	JUC1100012302.mm.1	-16,41	0,000072	0,251428		
TC0200004493.mm.1	5,99	Frmd5	JUC0200019659.mm.1	2,31	0,021552	0,483034		
TC0200004493.mm.1	5,99	Frmd5	JUC0200019656.mm.1	2,17	0,006634	0,392813		
TC0200004493.mm.1	5,99	Frmd5	PSR0200038140.mm.1	-2,36	0,000949	0,313363	Cassette Exon	0,13
TC0200004493.mm.1	5,99	Frmd5	PSR0200038145.mm.1	-2,55	0,000166	0,272566	Cassette Exon	0,06
TC0200004493.mm.1	5,99	Frmd5	PSR0200038139.mm.1	-2,91	0,000399	0,297747	Cassette Exon	0,13
TC0200004493.mm.1	5,99	Frmd5	PSR0200038163.mm.1	-3,59	0,00622	0,387912		
TC0200004493.mm.1	5,99	Frmd5	PSR0200038148.mm.1	-3,62	0,011218	0,426683	Alternative 3' Acce	0,42
TC0200004493.mm.1	5,99	Frmd5	PSR0200038127.mm.1	-3,79	0,005133	0,376377	Alternative 3' Acce	0,45
TC0200004493.mm.1	5,99	Frmd5	JUC0200019663.mm.1	-4,1	0,00041	0,297771		
TC0200004493.mm.1	5,99	Frmd5	PSR0200038164.mm.1	-4,59	0,006319	0,38927	Alternative 5' Donc	0,07
TC0200004493.mm.1	5,99	Frmd5	PSR0200038137.mm.1	-4,7	0,002335	0,347265	Cassette Exon	0,13
TC0200004493.mm.1	5,99	Frmd5	JUC0200019668.mm.1	-4,72	0,0075	0,400933		
TC0200004493.mm.1	5,99	Frmd5	JUC0200019670.mm.1	-5,01	0,003067	0,353892		
TC0200004493.mm.1	5,99	Frmd5	PSR0200038167.mm.1	-5,25	0,047407	0,558302	Alternative 5' Donc	0,07
TC0200004493.mm.1	5,99	Frmd5	PSR0200038155.mm.1	-6,17	0,001382	0,32572	Cassette Exon	0,28
TC0200004493.mm.1	5,99	Frmd5	PSR0200038160.mm.1	-6,43	0,003126	0,353892	Cassette Exon	0,44
TC0200004493.mm.1	5,99	Frmd5	PSR0200038147.mm.1	-7,28	0,003618	0,356223	Cassette Exon	0,29
TC0200004493.mm.1	5,99	Frmd5	JUC0200019654.mm.1	-7,53	0,000314	0,288663		
TC0200004493.mm.1	5,99	Frmd5	JUC0200019666.mm.1	-9	0,005403	0,378967		
TC0200004493.mm.1	5,99	Frmd5	JUC0200019652.mm.1	-9,06	0,008901	0,410728		
TC1300001955.mm.1	3,17	Susd3	JUC1300006317.mm.1	2,31	0,002263	0,34578		
TC1300001955.mm.1	3,17	Susd3	PSR1300012244.mm.1	-2,15	0,037861	0,534818	Cassette Exon	0,05
TC1300001955.mm.1	3,17	Susd3	PSR1300012245.mm.1	-2,58	0,009785	0,417839	Cassette Exon	0,27
TC1300001955.mm.1	3,17	Susd3	PSR1300012254.mm.1	-3,42	0,01518	0,452699	Cassette Exon	0,17
TC0400003599.mm.1	-1,29	Hdac1	PSR0400029350.mm.1	2,31	0,004564	0,369273	Cassette Exon	0,11
TC1200000126.mm.1	-1,31		PSR1200001102.mm.1	2,31	0,01679	0,460623	Cassette Exon	0,1
TC0800001215.mm.1	1,4	Slc9a5	JUC0800005183.mm.1	2,31	0,007933	0,403819		
TC0800001215.mm.1	1,4	Slc9a5	JUC0800005176.mm.1	2,13	0,03153	0,517029		
TC0800001215.mm.1	1,4	Slc9a5	PSR0800009596.mm.1	-2,05	0,001457	0,328449	Cassette Exon	0,06
TC1400002120.mm.1	-2,05	Gzmb	JUC1400008917.mm.1	2,31	0,000307	0,288663		
TC0200002821.mm.1	1,18	Rtel1	JUC0200011739.mm.1	2,31	0,02226	0,485896		
TC0200002821.mm.1	1,18	Rtel1	JUC0200011753.mm.1	2,16	0,024367	0,49362		
TC0200002821.mm.1	1,18	Rtel1	JUC0200011773.mm.1	-2,06	0,002204	0,345178		
TC0200002821.mm.1	1,18	Rtel1	JUC0200011772.mm.1	-2,19	0,012595	0,435933		
TC0200002821.mm.1	1,18	Rtel1	JUC0200011769.mm.1	-2,5	0,002878	0,352207		
TC1200000359.mm.1	-1,29	Dock4	JUC1200001479.mm.1	2,31	0,014149	0,447466		
TC1200000359.mm.1	-1,29	Dock4	JUC1200001476.mm.1	-2,29	0,031	0,514828		
TC0200003656.mm.1	-1,37	Cobll1	JUC0200015804.mm.1	2,31	0,012053	0,432426		
TC1700001156.mm.1	1,35	Spast	JUC1700005914.mm.1	2,31	0,028486	0,50733		
TC0800000875.mm.1	-1,45		PSR0800006550.mm.1	2,31	0,033608	0,523309		
TC1700000176.mm.1	1,4	Ermard; Gm3435	JUC1700000772.mm.1	2,31	0,047644	0,558744		

TC1700000176.mm.1	1,4	Ermard; Gm3435	JUC1700000788.mm.1	-2,42	0,000362	0,290942		
TC1600001340.mm.1	-1,15	Parl	JUC1600005749.mm.1	2,31	0,002496	0,349135		
TC1600001352.mm.1	1,01	Clcn2	JUC1600005855.mm.1	2,31	0,025745	0,498353		
TC0500002157.mm.1	-1,05	Otof	JUC0500010695.mm.1	2,31	0,014943	0,451968		
TC0500002252.mm.1	-1,25	Htra3	JUC0500011240.mm.1	2,31	0,020869	0,479521		
TC0500002252.mm.1	-1,25	Htra3	JUC0500011244.mm.1	-2,05	0,007472	0,400381		
TC0900000696.mm.1	-1,02	Man2c1	JUC0900002696.mm.1	2,31	0,007704	0,402552		
TC0900000696.mm.1	-1,02	Man2c1	JUC0900002688.mm.1	-2,67	0,022798	0,488229		
TC0800003093.mm.1	-1,31	Chmp1a	JUC0800013134.mm.1	2,31	0,016363	0,458693		
TC0600000813.mm.1	-1,17	Eif2ak3	JUC0600003111.mm.1	2,31	0,000168	0,272566		
TC0700001352.mm.1	-1,18	Kctd14	JUC0700005912.mm.1	2,31	0,043627	0,550161		
TC1100001054.mm.1	1,1	Smg6	JUC1100005027.mm.1	2,31	0,023559	0,490947		
TC0300002752.mm.1	-1,05	Stxbp3a	JUC0300011292.mm.1	2,31	0,031504	0,516965		
TC1000002257.mm.1	1,39	Hkdc1	JUC1000008805.mm.1	2,31	0,025114	0,496358		
TC0300002450.mm.1	-1,58	Tuft1	JUC0300009801.mm.1	2,31	0,007235	0,39826		
TC0Y00000286.mm.1	1,11	Gm20858	JUC0Y00001206.mm.1	2,31	0,020525	0,478276		
TC0200004539.mm.1	3,07	Hdc	JUC0200020056.mm.1	2,3	0,019856	0,475698		
TC0200004539.mm.1	3,07	Hdc	JUC0200020047.mm.1	2,01	0,030048	0,511812		
TC0200004539.mm.1	3,07	Hdc	PSR0200038740.mm.1	-2,56	0,020985	0,480104	Alternative 5' Donc	0,16
TC0200004539.mm.1	3,07	Hdc	JUC0200020051.mm.1	-2,58	0,003136	0,353892		
TC0200004539.mm.1	3,07	Hdc	PSR0200038726.mm.1	-2,78	0,015515	0,454456	Alternative 3' Acce	0,28
TC0200004539.mm.1	3,07	Hdc	PSR0200038725.mm.1	-3,16	0,015925	0,456718	Alternative 5' Donc	0,31
TC0200004539.mm.1	3,07	Hdc	JUC0200020055.mm.1	-3,2	0,003181	0,354243		
TC0200004539.mm.1	3,07	Hdc	PSR0200038736.mm.1	-3,23	0,017121	0,46232	Alternative 5' Donc	0,26
TC0200004539.mm.1	3,07	Hdc	PSR0200038744.mm.1	-4,16	0,003159	0,353892	Cassette Exon	0,37
TC0200004539.mm.1	3,07	Hdc	PSR0200038721.mm.1	-5,08	0,005663	0,381922	Alternative 3' Acce	0,48
TC1800000418.mm.1	1,77	Kctd16	JUC1800001728.mm.1	2,3	0,013317	0,441836		
TC1800000418.mm.1	1,77	Kctd16	PSR1800003108.mm.1	-2,61	0,013365	0,442156	Cassette Exon	0,26
TC1800000418.mm.1	1,77	Kctd16	JUC1800001731.mm.1	-2,79	0,018371	0,468484		
TC1400002171.mm.1	1,04	Nupl1; Mir719	PSR1400016832.mm.1	2,3	0,029169	0,509333	Alternative 3' Acce	0,21
TC1600001770.mm.1	-1,56	St3gal6	JUC1600007475.mm.1	2,3	0,048851	0,561704		
TC1600001770.mm.1	-1,56	St3gal6	PSR1600014395.mm.1	2,13	0,029255	0,509776	Alternative 5' Donc	0,15
TC1600001770.mm.1	-1,56	St3gal6	PSR1600014402.mm.1	2,13	0,022639	0,487515	Intron Retention	0,15
TC1200002527.mm.1	-1,64	Rpl36al; Rpl36a	JUC1200006971.mm.1	2,3	0,015333	0,453763		
TC1200002527.mm.1	-1,64	Rpl36al; Rpl36a	PSR1200012614.mm.1	2,03	0,031276	0,516064	Intron Retention	0,13
TC0400001777.mm.1	-1,09	Spata21	PSR0400014702.mm.1	2,3	0,019479	0,474295	Cassette Exon	0,12
TC0400000207.mm.1	-1,25	Mms22l	PSR0400001343.mm.1	2,3	0,006698	0,393343	Cassette Exon	0,11
TC1200000989.mm.1	-1,81	Kcnk13	PSR1200006897.mm.1	2,3	0,000717	0,306015	Alternative 5' Donc	0,11
TC0700002446.mm.1	-1,06		PSR0700021558.mm.1	2,3	0,021566	0,483109	Cassette Exon	0,08
TC0500003024.mm.1	1,33	Adrbk2	JUC0500014710.mm.1	2,3	0,016672	0,460085		
TC0500003024.mm.1	1,33	Adrbk2	PSR0500026886.mm.1	2,18	0,002136	0,343402	Cassette Exon	0,07
TC0500003024.mm.1	1,33	Adrbk2	JUC0500014696.mm.1	-6,61	0,001793	0,336311		
TC0200003121.mm.1	-1,47		JUC0200012969.mm.1	2,3	0,000463	0,298999		
TC1900001524.mm.1	1,03	Crtac1	JUC1900007434.mm.1	2,3	0,023587	0,490995		
TC1900001524.mm.1	1,03	Crtac1	JUC1900007436.mm.1	2,06	0,028815	0,508347		
TC1600001667.mm.1	-1,47	Phldb2	JUC1600007136.mm.1	2,3	0,014341	0,448173		
TC0700004664.mm.1	-1,24	Prss53	JUC0700019244.mm.1	2,3	0,0109	0,42445		
TC0700000938.mm.1	1,09	Oca2	JUC0700004412.mm.1	2,3	0,034685	0,526294		

TC0700000938.mm.1	1,09	Oca2	JUC0700004396.mm.1	2,13	0,002919	0,352289		
TC0600003439.mm.1	1,25	Asun	JUC0600014258.mm.1	2,3	0,032721	0,520439		
TC0300003084.mm.1	1,1	Clca3	JUC0300012714.mm.1	2,3	0,022367	0,486407		
TC1100002844.mm.1	1,85	Guk1	JUC1100013446.mm.1	2,3	0,01425	0,447947		
TC1100002844.mm.1	1,85	Guk1	JUC1100013451.mm.1	-2,21	0,047997	0,559574		
TC0500000013.mm.1	1,01	1700109H08Rik	JUC0500000035.mm.1	2,3	0,035153	0,527974		
TC1000001257.mm.1	1,94	Ppfia2	JUC1000005091.mm.1	2,29	0,031015	0,514914		
TC1000001257.mm.1	1,94	Ppfia2	PSR1000009489.mm.1	-2,06	0,013597	0,443703	Alternative 5' Donc	0,16
TC1000001257.mm.1	1,94	Ppfia2	PSR1000009453.mm.1	-2,43	0,02676	0,502096	Cassette Exon	0,14
TC1000001257.mm.1	1,94	Ppfia2	JUC1000005120.mm.1	-2,55	0,013518	0,443431		
TC1000001257.mm.1	1,94	Ppfia2	PSR1000009458.mm.1	-2,58	0,002734	0,349612	Cassette Exon	0,14
TC1000001257.mm.1	1,94	Ppfia2	JUC1000005122.mm.1	-2,91	0,045334	0,553867		
TC1000001257.mm.1	1,94	Ppfia2	JUC1000005110.mm.1	-3,42	0,027585	0,504482		
TC1000001257.mm.1	1,94	Ppfia2	PSR1000009480.mm.1	-3,49	0,0098	0,417839	Cassette Exon	0,36
TC1000001257.mm.1	1,94	Ppfia2	JUC1000005104.mm.1	-4,72	0,022629	0,487464		
TC0200001221.mm.1	1,52	Ypel4	PSR0200009729.mm.1	2,29	0,022149	0,48561	Cassette Exon	0,16
TC0200001221.mm.1	1,52	Ypel4	PSR0200009735.mm.1	-2,14	0,003569	0,356223	Alternative 5' Donc	0,18
TC0200001221.mm.1	1,52	Ypel4	PSR0200009720.mm.1	-5,6	0,000006	0,179072	Alternative 3' Acce	0,34
TC0600002493.mm.1	1,1	Sh2d6	PSR0600019332.mm.1	2,29	0,000787	0,309905	Cassette Exon	0,11
TC0600002493.mm.1	1,1	Sh2d6	PSR0600019331.mm.1	-3,92	0,034758	0,52661	Cassette Exon	0,29
TC0900002767.mm.1	8,56	Elovl4	JUC0900012805.mm.1	2,29	0,011597	0,429361		
TC0900002767.mm.1	8,56	Elovl4	PSR0900022903.mm.1	-2,25	0,000752	0,308185	Cassette Exon	0,2
TC0900002767.mm.1	8,56	Elovl4	JUC0900012806.mm.1	-4,51	0,003618	0,356223		
TC0900002767.mm.1	8,56	Elovl4	PSR0900022907.mm.1	-14,41	0,007967	0,403853	Alternative 5' Donc	0,28
TC0400003702.mm.1	3,31	Trnp1	PSR0400030353.mm.1	2,29	0,004007	0,36119	Cassette Exon	0,13
TC0400003702.mm.1	3,31	Trnp1	PSR0400030350.mm.1	2,08	0,008882	0,410619	Cassette Exon	0,08
TC0400003702.mm.1	3,31	Trnp1	PSR0400030352.mm.1	2,05	0,002315	0,346468	Cassette Exon	0,08
TC0400003702.mm.1	3,31	Trnp1	PSR0400030354.mm.1	-2,61	0,049835	0,563773	Alternative 5' Donc	0,26
TC0100003568.mm.1	2,68	Kcnj9	PSR0100029333.mm.1	2,29	0,004909	0,372662	Alternative 5' Donc	0,21
TC0100003568.mm.1	2,68	Kcnj9	PSR0100029330.mm.1	-2,25	0,009411	0,415853	Cassette Exon	0,2
TC0100003568.mm.1	2,68	Kcnj9	PSR0100029335.mm.1	-2,42	0,034673	0,526294	Cassette Exon	0,13
TC0100003568.mm.1	2,68	Kcnj9	JUC0100016679.mm.1	-2,79	0,029085	0,509063		
TC0100003872.mm.1	-1,55	Irf6	JUC0100008805.mm.1	2,29	0,001619	0,332831		
TC0100003872.mm.1	-1,55	Irf6	PSR0100015486.mm.1	2,07	0,045446	0,554176	Alternative 3' Acce	0,17
TC0100002586.mm.1	-1,15	Zfp142	PSR0100020883.mm.1	2,29	0,026028	0,499483	Cassette Exon	0,14
TC0400004088.mm.1	1,21	Zbtb48	JUC0400017738.mm.1	2,29	0,010273	0,420305		
TC0400004088.mm.1	1,21	Zbtb48	JUC0400017741.mm.1	2,26	0,020881	0,479589		
TC0400004088.mm.1	1,21	Zbtb48	PSR0400034003.mm.1	-2,43	0,008023	0,404268	Alternative 5' Donc	0,14
TC0100000742.mm.1	-1,33	Gm6264	PSR0100006232.mm.1	2,29	0,038466	0,536428	Cassette Exon	0,12
TC0X00000212.mm.1	1,3	Uba1	PSR0X00001351.mm.1	2,29	0,043949	0,550739	Cassette Exon	0,12
TC1500002059.mm.1	1,49	Rabl2	PSR1500016627.mm.1	2,29	0,002651	0,349612	Cassette Exon	0,12
TC1600001220.mm.1	-1,27		PSR1600009796.mm.1	2,29	0,04395	0,550739	Cassette Exon	0,11
TC1600001220.mm.1	-1,27		PSR1600009784.mm.1	2,2	0,0354	0,528631	Cassette Exon	0,12
TC1600001220.mm.1	-1,27		PSR1600009789.mm.1	2,14	0,013138	0,440196	Cassette Exon	0,12
TC0300000700.mm.1	-1,04	Arhgef11	JUC0300002450.mm.1	2,29	0,035296	0,52837		
TC0300000700.mm.1	-1,04	Arhgef11	JUC0300002451.mm.1	2,24	0,009316	0,414883		
TC0200004568.mm.1	-1,1	Bub1	JUC0200020296.mm.1	2,29	0,033916	0,52424		
TC0200004568.mm.1	-1,1	Bub1	JUC0200020288.mm.1	-2,41	0,014521	0,449545		

TC0200001382.mm.1	1,2	Ambra1	JUC0200005500.mm.1	2,29	0,03302	0,521478		
TC0500002910.mm.1	1,86	Barhl2	JUC0500013982.mm.1	2,29	0,018018	0,466939		
TC0800002428.mm.1	1,46	Ano8	JUC0800010084.mm.1	2,29	0,01356	0,443675		
TC0800002428.mm.1	1,46	Ano8	JUC0800010074.mm.1	-2,23	0,003127	0,353892		
TC0900001428.mm.1	1,12	Col7a1; Mir711	JUC0900006490.mm.1	2,29	0,013677	0,444535		
TC0900001428.mm.1	1,12	Col7a1; Mir711	JUC0900006465.mm.1	-2,29	0,034605	0,52602		
TC0900001428.mm.1	1,12	Col7a1; Mir711	JUC0900006556.mm.1	-2,29	0,027706	0,504872		
TC0900001428.mm.1	1,12	Col7a1; Mir711	JUC0900006444.mm.1	-2,6	0,031323	0,516204		
TC0600001076.mm.1	-1,03	Aldh1l1	JUC0600004253.mm.1	2,29	0,004429	0,366832		
TC0700000285.mm.1	-1,08	Dmpk	JUC0700001035.mm.1	2,29	0,038297	0,536106		
TC0700002711.mm.1	-1,66	Ryr1	JUC0700012751.mm.1	2,29	0,040536	0,542414		
TC0700002711.mm.1	-1,66	Ryr1	JUC0700012745.mm.1	2,2	0,043799	0,550421		
TC0700002711.mm.1	-1,66	Ryr1	JUC0700012702.mm.1	-2,16	0,014245	0,447947		
TC0700002711.mm.1	-1,66	Ryr1	JUC0700012697.mm.1	-2,26	0,039749	0,540505		
TC0700002711.mm.1	-1,66	Ryr1	JUC0700012704.mm.1	-2,53	0,030817	0,514173		
TC0400003648.mm.1	-1,3	Gm16080	JUC0400015519.mm.1	2,29	0,040392	0,541998		
TC0400003836.mm.1	-1,25	Otud3	JUC0400016442.mm.1	2,29	0,042971	0,54838		
TC1000000676.mm.1	-2,3		PSR1000004714.mm.1	2,29	0,047105	0,557863		
TC1300002722.mm.1	5,82	Isl1	PSR1300018433.mm.1	2,28	0,011885	0,431306	Cassette Exon	0,13
TC1300002722.mm.1	5,82	Isl1	PSR1300018427.mm.1	-2,14	0,001962	0,340561	Cassette Exon	0,21
TC1300002722.mm.1	5,82	Isl1	PSR1300018430.mm.1	-2,51	0,000771	0,308743	Cassette Exon	0,39
TC1300002722.mm.1	5,82	Isl1	PSR1300018422.mm.1	-3,33	0,009255	0,414559	Alternative 3' Acce	0,37
TC1300002722.mm.1	5,82	Isl1	JUC1300009898.mm.1	-3,46	0,031096	0,51525		
TC1300002722.mm.1	5,82	Isl1	PSR1300018431.mm.1	-3,58	0,008046	0,40434	Cassette Exon	0,4
TC1300002722.mm.1	5,82	Isl1	JUC1300009900.mm.1	-3,67	0,031644	0,517405		
TC1300002722.mm.1	5,82	Isl1	PSR1300018432.mm.1	-4,42	0,013176	0,440629	Alternative 3' Acce	0,48
TC1300002722.mm.1	5,82	Isl1	PSR1300018436.mm.1	-7,38	0,002024	0,341377	Alternative 5' Donc	0,39
TC1300002722.mm.1	5,82	Isl1	JUC1300009904.mm.1	-14,77	0,005666	0,381922		
TC0X00003096.mm.1	3,28	Gucy2f	JUC0X00009853.mm.1	2,28	0,00499	0,373851		
TC0X00003096.mm.1	3,28	Gucy2f	JUC0X00009842.mm.1	-2,35	0,01489	0,45179		
TC0X00003096.mm.1	3,28	Gucy2f	JUC0X00009849.mm.1	-2,4	0,011739	0,429995		
TC0X00003096.mm.1	3,28	Gucy2f	JUC0X00009844.mm.1	-2,69	0,002202	0,345178		
TC0X00003096.mm.1	3,28	Gucy2f	JUC0X00009841.mm.1	-4,25	0,000251	0,28803		
TC0X00003096.mm.1	3,28	Gucy2f	PSR0X00019442.mm.1	-5,36	0,001305	0,323504	Cassette Exon	0,47
TC1100002158.mm.1	2,57	Camk2b	PSR1100021004.mm.1	2,28	0,00154	0,330388	Cassette Exon	0,06
TC1100002158.mm.1	2,57	Camk2b	PSR1100021021.mm.1	-2,17	0,027415	0,503961	Cassette Exon	0,2
TC1100002158.mm.1	2,57	Camk2b	PSR1100021040.mm.1	-2,24	0,032824	0,520896	Alternative 5' Donc	0,24
TC1100002158.mm.1	2,57	Camk2b	PSR1100021028.mm.1	-2,27	0,011607	0,429583	Cassette Exon	0,27
TC1100002158.mm.1	2,57	Camk2b	JUC1100010960.mm.1	-2,32	0,014985	0,452048		
TC1100002158.mm.1	2,57	Camk2b	PSR1100021016.mm.1	-2,46	0,002428	0,349	Cassette Exon	0,16
TC1100002158.mm.1	2,57	Camk2b	JUC1100010974.mm.1	-2,55	0,039744	0,540494		
TC1100002158.mm.1	2,57	Camk2b	PSR1100021007.mm.1	-2,56	0,023916	0,492316	Cassette Exon	0,31
TC1100002158.mm.1	2,57	Camk2b	PSR1100021015.mm.1	-2,56	0,042528	0,547845	Cassette Exon	0,2
TC1100002158.mm.1	2,57	Camk2b	PSR1100021044.mm.1	-2,58	0,043989	0,550776	Cassette Exon	0,17
TC1100002158.mm.1	2,57	Camk2b	JUC1100010989.mm.1	-2,62	0,029415	0,509995		
TC1100002158.mm.1	2,57	Camk2b	JUC1100010991.mm.1	-2,63	0,032296	0,519116		
TC1100002158.mm.1	2,57	Camk2b	PSR1100021005.mm.1	-2,76	0,009836	0,417839	Intron Retention	0,44
TC1100002158.mm.1	2,57	Camk2b	PSR1100021042.mm.1	-2,77	0,045111	0,553206	Alternative 5' Donc	0,24

TC1100002158.mm.1	2,57	Camk2b	JUC1100010976.mm.1	-2,79	0,04292	0,548271		
TC1100002158.mm.1	2,57	Camk2b	JUC1100010986.mm.1	-2,79	0,04292	0,548271		
TC1100002158.mm.1	2,57	Camk2b	PSR1100021008.mm.1	-3,49	0,020541	0,478308	Cassette Exon	0,31
TC1100002158.mm.1	2,57	Camk2b	JUC1100010981.mm.1	-3,66	0,037191	0,533252		
TC1100002158.mm.1	2,57	Camk2b	PSR1100021009.mm.1	-4,05	0,011464	0,428022	Cassette Exon	0,31
TC1100002158.mm.1	2,57	Camk2b	JUC1100010964.mm.1	-4,1	0,002039	0,341377		
TC1100002158.mm.1	2,57	Camk2b	JUC1100010982.mm.1	-4,92	0,011315	0,427133		
TC0700003332.mm.1	2,97		PSR0700028623.mm.1	2,28	0,004624	0,369794	Cassette Exon	0,02
TC0700003332.mm.1	2,97		PSR0700028624.mm.1	-2,28	0,004624	0,369794	Cassette Exon	0,21
TC0700003332.mm.1	2,97		JUC0700015154.mm.1	-3,13	0,005099	0,375732		
TC1000003007.mm.1	1,03	Cand1	JUC1000012310.mm.1	2,28	0,044372	0,551441		
TC1000003007.mm.1	1,03	Cand1	PSR1000022471.mm.1	-2,31	0,010432	0,421581	Alternative 5' Donc	0,2
TC1300002773.mm.1	-1,28	Zfp953; ZFP953	PSR1300018651.mm.1	2,28	0,00346	0,355628	Cassette Exon	0,14
TC1900001137.mm.1	-1,37	Ms4a6d	PSR1900010674.mm.1	2,28	0,016316	0,45857	Cassette Exon	0,12
TC1900001137.mm.1	-1,37	Ms4a6d	PSR1900010675.mm.1	2,06	0,010806	0,424055	Cassette Exon	0,13
TC0100001677.mm.1	1,56	Gm7068	PSR0100013874.mm.1	2,28	0,006349	0,389463	Cassette Exon	0,12
TC0400003446.mm.1	-1,48	Zfp69	PSR0400028000.mm.1	2,28	0,008207	0,405909	Cassette Exon	0,12
TC0400003446.mm.1	-1,48	Zfp69	PSR0400027998.mm.1	2,2	0,005359	0,378582	Cassette Exon	0,12
TC0900002486.mm.1	-1,29	Smad6	PSR0900020819.mm.1	2,28	0,002347	0,34761	Cassette Exon	0,12
TC0900002486.mm.1	-1,29	Smad6	JUC0900011733.mm.1	-2,01	0,030363	0,512923		
TC0500003444.mm.1	1,18		PSR0500031237.mm.1	2,28	0,030284	0,512627	Cassette Exon	0,1
TC0X00003003.mm.1	-1,31	Nxf2	JUC0X00009493.mm.1	2,28	0,033686	0,523549		
TC0X00003003.mm.1	-1,31	Nxf2	PSR0X00018751.mm.1	-2,02	0,008649	0,408463	Cassette Exon	0,09
TC1200002172.mm.1	-1,09		JUC1200008295.mm.1	2,28	0,006244	0,388361		
TC1700002692.mm.1	1,23	Calm2; Calm3; Calm1	JUC1700013150.mm.1	2,28	0,023485	0,490681		
TC0100002583.mm.1	-1,62	Tmbim1	JUC0100011802.mm.1	2,28	0,015037	0,452111		
TC0100002212.mm.1	-1,32	Lyg1	JUC0100010111.mm.1	2,28	0,004762	0,371039		
TC1700000901.mm.1	1,19	Pex6	JUC1700004630.mm.1	2,28	0,006884	0,39438		
TC1800001666.mm.1	-1,11	Atp9b	JUC1800006666.mm.1	2,28	0,015681	0,455416		
TC0200002264.mm.1	-1,31	Sdcbp2	JUC0200008905.mm.1	2,28	0,024038	0,493014		
TC1600000960.mm.1	-1,37	4930590A17Rik	JUC1600003949.mm.1	2,28	0,041096	0,543933		
TC0500002628.mm.1	1,07	Clock	JUC0500012651.mm.1	2,28	0,044841	0,552566		
TC1100001150.mm.1	-1,42	Proca1	JUC1100005548.mm.1	2,28	0,000229	0,28803		
TC1000002491.mm.1	1,3	Dos	JUC1000010085.mm.1	2,28	0,001159	0,317849		
TC1000002491.mm.1	1,3	Dos	JUC1000010081.mm.1	-2,23	0,026914	0,502518		
TC0300001816.mm.1	1,1	Ccdc39	JUC0300007412.mm.1	2,28	0,016601	0,459739		
TC0400002633.mm.1	-1,27	Acnat2	JUC0400011113.mm.1	2,28	0,043108	0,548605		
TC0700002759.mm.1	8,54	Aplp1	PSR0700024471.mm.1	2,27	0,02097	0,480007	Cassette Exon	0,08
TC0700002759.mm.1	8,54	Aplp1	PSR0700024475.mm.1	-6,62	0,039236	0,538862	Cassette Exon	0,37
TC1400001745.mm.1	5,79	Cdhr1	JUC1400007434.mm.1	2,27	0,007393	0,399627		
TC1400001745.mm.1	5,79	Cdhr1	JUC1400007431.mm.1	2,15	0,004511	0,368648		
TC1400001745.mm.1	5,79	Cdhr1	JUC1400007432.mm.1	-2,65	0,000374	0,291853		
TC1400001745.mm.1	5,79	Cdhr1	PSR1400013595.mm.1	-7,08	0,005948	0,385276	Alternative 5' Donc	0,34
TC0600000784.mm.1	2,49	A430010J10Rik	PSR0600005860.mm.1	2,27	0,003498	0,355638	Cassette Exon	0,17
TC0600000784.mm.1	2,49	A430010J10Rik	JUC0600002932.mm.1	2,17	0,033907	0,52424		
TC0600000784.mm.1	2,49	A430010J10Rik	PSR0600005858.mm.1	-2,15	0,011916	0,431651	Cassette Exon	0,11
TC0600000784.mm.1	2,49	A430010J10Rik	PSR0600005867.mm.1	-2,19	0,035941	0,529399	Cassette Exon	0,11
TC0600000784.mm.1	2,49	A430010J10Rik	PSR0600005863.mm.1	-2,48	0,001597	0,332399	Alternative 5' Donc	0,22

TC0600000784.mm.1	2,49	A430010J10Rik	PSR0600005862.mm.1	-2,6	0,000964	0,313363	Alternative 5' Donc	0,22
TC0600000784.mm.1	2,49	A430010J10Rik	PSR0600005859.mm.1	-2,64	0,000849	0,311909	Alternative 3' Acce	0,26
TC0600000784.mm.1	2,49	A430010J10Rik	PSR0600005864.mm.1	-2,82	0,008132	0,405267	Alternative 5' Donc	0,22
TC0600000784.mm.1	2,49	A430010J10Rik	PSR0600005865.mm.1	-3,1	0,03151	0,516965	Cassette Exon	0,24
TC1200002006.mm.1	2,96	Ccdc177	PSR1200013801.mm.1	2,27	0,015882	0,456526	Alternative 3' Acce	0,17
TC1200002006.mm.1	2,96	Ccdc177	PSR1200013805.mm.1	-2,58	0,008451	0,406611	Cassette Exon	0,22
TC1200002006.mm.1	2,96	Ccdc177	PSR1200013806.mm.1	-3,38	0,000011	0,179072	Cassette Exon	0,22
TC0200003670.mm.1	-1,42	Galnt3	PSR0200031278.mm.1	2,27	0,00123	0,322251	Alternative 5' Donc	0,2
TC0900002452.mm.1	-1,45	Paqr5	PSR0900020555.mm.1	2,27	0,047437	0,558338	Alternative 5' Donc	0,19
TC1700000657.mm.1	-1,42	Lsm2	PSR1700006566.mm.1	2,27	0,026006	0,499257	Alternative 3' Acce	0,19
TC1700000657.mm.1	-1,42	Lsm2	JUC1700003630.mm.1	2,16	0,002593	0,349501		
TC1400000899.mm.1	-1,03	Fam167a	PSR1400007219.mm.1	2,27	0,017394	0,463736	Alternative 3' Acce	0,18
TC1400001487.mm.1	-1,6	Rarb	PSR1400011138.mm.1	2,27	0,009977	0,418456	Alternative 3' Acce	0,17
TC1600001593.mm.1	-1,35	Ndufb4; Gm3873; Gm324	PSR1600013054.mm.1	2,27	0,024192	0,493294	Cassette Exon	0,16
TC1100002975.mm.1	1,35	Arhgap44	PSR1100026979.mm.1	2,27	0,002163	0,344238	Cassette Exon	0,15
TC1100002975.mm.1	1,35	Arhgap44	JUC1100014062.mm.1	2,23	0,007096	0,396897		
TC0400004224.mm.1	-1,04	Ephb2	PSR0400031266.mm.1	2,27	0,03361	0,523309	Cassette Exon	0,14
TC0700001410.mm.1	-1,68	Coa4	PSR0700012052.mm.1	2,27	0,044414	0,551538	Cassette Exon	0,14
TC0700001838.mm.1	1,11	Zfp553	PSR0700015737.mm.1	2,27	0,040219	0,541714	Cassette Exon	0,13
TC0900003095.mm.1	-1,37	Fbxw27	PSR0900026142.mm.1	2,27	0,004212	0,363236	Cassette Exon	0,13
TC0900003095.mm.1	-1,37	Fbxw27	JUC0900014676.mm.1	2,05	0,029499	0,510123		
TC0500003138.mm.1	1,69	Rnft2	JUC0500015195.mm.1	2,27	0,022445	0,487003		
TC0500003138.mm.1	1,69	Rnft2	JUC0500015194.mm.1	2,21	0,007958	0,403853		
TC0500003138.mm.1	1,69	Rnft2	PSR0500027925.mm.1	-2,27	0,012495	0,435429	Cassette Exon	0,12
TC1800001510.mm.1	1,21	Cep76	PSR1800011095.mm.1	2,27	0,007531	0,40125	Cassette Exon	0,06
TC1400000320.mm.1	1,23	Pbrm1	JUC1400001603.mm.1	2,27	0,03222	0,518925		
TC1400000320.mm.1	1,23	Pbrm1	JUC1400001621.mm.1	2,11	0,001835	0,337414		
TC1400000320.mm.1	1,23	Pbrm1	JUC1400001620.mm.1	-4,38	0,015671	0,455416		
TC1100003808.mm.1	1,05	Brca1	JUC1100018459.mm.1	2,27	0,019924	0,475902		
TC0300000828.mm.1	-1,55	Sprr2h	PSR0300006409.mm.1	2,27	0,022988	0,488935		
TC1300002048.mm.1	1,28	Uimc1	JUC1300006638.mm.1	2,27	0,021085	0,480371		
TC1300002702.mm.1	-1,05	A430090L17Rik	JUC1300009819.mm.1	2,27	0,01947	0,474209		
TC1300000730.mm.1	-1,07	A530065N20Rik	JUC1300002527.mm.1	2,27	0,012456	0,43528		
TC1700001791.mm.1	1,03	Ccdc167	JUC1700008896.mm.1	2,27	0,047288	0,558242		
TC1500001676.mm.1	-1,03	Col22a1	JUC1500007137.mm.1	2,27	0,007932	0,403819		
TC1500001676.mm.1	-1,03	Col22a1	JUC1500007153.mm.1	2,12	0,028537	0,507479		
TC1500001676.mm.1	-1,03	Col22a1	JUC1500007139.mm.1	-2,46	0,002457	0,349135		
TC1600000042.mm.1	1,45	4930451G09Rik	JUC1600000182.mm.1	2,27	0,006962	0,395722		
TC0700004571.mm.1	-1,38	Igf2	JUC0700020291.mm.1	2,27	0,032463	0,519612		
TC0700000819.mm.1	-1,18	Akt1s1; Mir707	PSR0700006843.mm.1	2,27	0,025015	0,496061		
TC0700000819.mm.1	-1,18	Akt1s1; Mir707	JUC0700003399.mm.1	2,04	0,028206	0,506368		
TC0700000879.mm.1	-1,11	Gtf2h1	JUC0700003937.mm.1	2,27	0,003284	0,354243		
TC0700003570.mm.1	-1,85	Anpep	JUC0700015930.mm.1	2,27	0,028205	0,506368		
TC1100001779.mm.1	-1,16		JUC1100008749.mm.1	2,27	0,011478	0,428022		
TC0400002837.mm.1	1,7	Brinp1; Dbc1	JUC0400012103.mm.1	2,27	0,011613	0,429617		
TC0900002374.mm.1	1,26	Mpi	JUC0900011207.mm.1	2,27	0,034846	0,526823		
TC0900002374.mm.1	1,26	Mpi	JUC0900011211.mm.1	-2,87	0,004582	0,369794		
TC1800000184.mm.1	2,38	Mapre2	JUC1800000783.mm.1	2,26	0,000434	0,297771		

TC1800000184.mm.1	2,38	Mapre2	PSR1800001322.mm.1	-2,86	0,047482	0,558611	Cassette Exon	0,38
TC1800000184.mm.1	2,38	Mapre2	JUC1800000774.mm.1	-2,9	0,038221	0,535805		
TC1800000184.mm.1	2,38	Mapre2	JUC1800000781.mm.1	-4,21	0,020589	0,478531		
TC1800000184.mm.1	2,38	Mapre2	JUC1800000782.mm.1	-5,75	0,00579	0,383104		
TC0100000363.mm.1	-1,17		PSR0100002815.mm.1	2,26	0,00518	0,376851	Alternative 3' Acce	0,2
TC1000003106.mm.1	13,68	Kif5a	PSR1000023184.mm.1	2,26	0,036703	0,531756	Cassette Exon	0,2
TC1000003106.mm.1	13,68	Kif5a	PSR1000023165.mm.1	-2,06	0,0482	0,56017		
TC1100000302.mm.1	1,49	Rtn4	PSR1100002556.mm.1	2,26	0,048359	0,56056	Alternative 3' Acce	0,2
TC0700003036.mm.1	-1,09	Tph1	PSR0700027708.mm.1	2,26	0,001553	0,330671	Alternative 5' Donc	0,18
TC1800001682.mm.1	1,21	Zfp236	JUC1800006724.mm.1	2,26	0,018624	0,46991		
TC1800001682.mm.1	1,21	Zfp236	PSR1800012048.mm.1	-2,11	0,03667	0,531675	Alternative 5' Donc	0,18
TC0X00003041.mm.1	1,53	Rab9b	PSR0X00019050.mm.1	2,26	0,003161	0,353901	Cassette Exon	0,16
TC0600003342.mm.1	-1,1	Eps8	PSR0600026271.mm.1	2,26	0,022722	0,487767	Cassette Exon	0,15
TC0500003329.mm.1	-1,21	Auts2	PSR0500030147.mm.1	2,26	0,045477	0,554304	Cassette Exon	0,14
TC0500003329.mm.1	-1,21	Auts2	PSR0500030133.mm.1	-2,28	0,000147	0,272178		
TC0500003418.mm.1	-1,26	Cux1	PSR0500030960.mm.1	2,26	0,038937	0,537874	Cassette Exon	0,14
TC0200002588.mm.1	-1,25	Rnf114	PSR0200021338.mm.1	2,26	0,001305	0,323504	Cassette Exon	0,12
TC0800003242.mm.1	-1,41	Trim61; Trim60	PSR0800017514.mm.1	2,26	0,007362	0,39938	Cassette Exon	0,12
TC0X00002046.mm.1	-1,34	Gm14569	PSR0X00012970.mm.1	2,26	0,035871	0,529376	Cassette Exon	0,12
TC1100003858.mm.1	1,71	C1ql1	PSR1100036021.mm.1	2,26	0,027102	0,503022	Cassette Exon	0,12
TC0500000591.mm.1	-1,32	Tbc1d1	JUC0500002919.mm.1	2,26	0,033772	0,523738		
TC0500000591.mm.1	-1,32	Tbc1d1	PSR0500005221.mm.1	2,14	0,005794	0,383104	Cassette Exon	0,11
TC0X00000730.mm.1	1,17	Xlr3e-ps	PSR0X00005046.mm.1	2,26	0,006809	0,393777	Cassette Exon	0,11
TC0900002811.mm.1	-1,35	Gm2396	JUC0900013057.mm.1	2,26	0,011189	0,426444		
TC0900002811.mm.1	-1,35	Gm2396	PSR0900023319.mm.1	2,01	0,009952	0,41841	Cassette Exon	0,1
TC0300000030.mm.1	-1,3		JUC0300000047.mm.1	2,26	0,007014	0,395741		
TC1100003935.mm.1	2,6	Ddx5; Mir3064; Gm25994	JUC1100019302.mm.1	2,26	0,0053	0,378059		
TC0200004297.mm.1	-1	Mettl15	JUC0200018396.mm.1	2,26	0,011183	0,426414		
TC1300002151.mm.1	-1,37	Cts7	JUC1300007179.mm.1	2,26	0,025262	0,496939		
TC0200004775.mm.1	1,14	Crnkl1	JUC0200021163.mm.1	2,26	0,015204	0,452738		
TC1700002665.mm.1	-1,27	Srbd1	JUC1700013089.mm.1	2,26	0,023471	0,490635		
TC1700002665.mm.1	-1,27	Srbd1	JUC1700013093.mm.1	2,14	0,040728	0,542962		
TC1700001383.mm.1	-1,29	Tulp4; LOC101055731; Syt	JUC1700006774.mm.1	2,26	0,038303	0,536106		
TC1700000924.mm.1	-1,02	Tfeb	JUC1700004755.mm.1	2,26	0,012492	0,435429		
TC1900001697.mm.1	-1,06		JUC1900008298.mm.1	2,26	0,038945	0,537874		
TC0100001866.mm.1	-1,62	1700065J18Rik	JUC0100008797.mm.1	2,26	0,045413	0,554105		
TC0200001782.mm.1	1,12	Mga	JUC0200006709.mm.1	2,26	0,048148	0,559995		
TC1500000821.mm.1	1,14	Ppara	JUC1500003621.mm.1	2,26	0,042993	0,548409		
TC1600001080.mm.1	-2,03	Sh3bgr	JUC1600004443.mm.1	2,26	0,049317	0,56273		
TC1600001080.mm.1	-2,03	Sh3bgr	JUC1600004447.mm.1	2,25	0,043569	0,550033		
TC0700004555.mm.1	1,03		JUC0700020251.mm.1	2,26	0,027613	0,504579		
TC0900000923.mm.1	-1,38	Gtf2a2	JUC0900003874.mm.1	2,26	0,004153	0,3623		
TC0600000735.mm.1	1,31	Grid2	JUC0600002833.mm.1	2,26	0,028918	0,50858		
TC0600003469.mm.1	1,44	Caprin2	JUC0600014407.mm.1	2,26	0,026262	0,500408		
TC1100000343.mm.1	-1,05	Stk10	JUC1100001535.mm.1	2,26	0,008001	0,403872		
TC1100000435.mm.1	-1,38	Gm12153	JUC1100001786.mm.1	2,26	0,014075	0,446841		
TC0400000501.mm.1	1,15	Ncbp1	JUC0400001815.mm.1	2,26	0,048394	0,56064		
TC1100002967.mm.1	-1,07	Cox10	JUC1100014041.mm.1	2,26	0,011244	0,426718		

TC1100002165.mm.1	-1,14	Npc1l1	JUC1100011016.mm.1	2,26	0,037576	0,534053		
TC0X00001601.mm.1	-1,59	Smpx	JUC0X00005065.mm.1	2,26	0,037516	0,533968		
TC0X00001690.mm.1	-1,66	Gm15228	JUC0X00005505.mm.1	2,26	0,037857	0,534808		
TC0X00001336.mm.1	1,14	BC065397; Gm15009	JUC0X00004120.mm.1	2,26	0,014364	0,448293		
TC0900002915.mm.1	-1,24	Pik3cb	JUC0900013414.mm.1	2,26	0,000294	0,288663		
TC0900002915.mm.1	-1,24	Pik3cb	JUC0900013395.mm.1	2,16	0,046079	0,555658		
TC0900002588.mm.1	-1,31	Fam81a	JUC0900012111.mm.1	2,26	0,018921	0,471305		
TC1000000147.mm.1	-1,05	Map3k5	JUC1000000535.mm.1	2,26	0,005135	0,376442		
TC0400001407.mm.1	-1,33	LOC101056089	JUC0400005524.mm.1	2,26	0,032166	0,518706		
TC0400002721.mm.1	-1,26	1700042G15Rik	JUC0400011433.mm.1	2,26	0,009988	0,418456		
TC1100000264.mm.1	-1,39	Fancl	PSR1100002254.mm.1	2,25	0,017325	0,463398	Cassette Exon	0,1
TC1100000264.mm.1	-1,39	Fancl	PSR1100002261.mm.1	2,01	0,024165	0,493245	Intron Retention	0,25
TC0200002311.mm.1	-1,52		PSR0200018158.mm.1	2,25	0,018275	0,468049	Alternative 5' Donc	0,2
TC0100001985.mm.1	-1,71	Eya1	PSR0100016240.mm.1	2,25	0,003199	0,354243	Cassette Exon	0,18
TC0700000014.mm.1	-1,68	Ndufa3	PSR0700000122.mm.1	2,25	0,03262	0,520338	Intron Retention	0,18
TC1200000524.mm.1	1,51	Prpf39	PSR1200003648.mm.1	2,25	0,02919	0,509374	Cassette Exon	0,18
TC0800001507.mm.1	1,48	Spg7	PSR0800012263.mm.1	2,25	0,01449	0,449409	Cassette Exon	0,13
TC0800001507.mm.1	1,48	Spg7	PSR0800012290.mm.1	-2,13	0,03113	0,51548	Alternative 5' Donc	0,17
TC0800001507.mm.1	1,48	Spg7	JUC0800006701.mm.1	-2,17	0,007496	0,400809		
TC0200004283.mm.1	-1,22	Arl14ep	PSR0200035995.mm.1	2,25	0,045675	0,554833	Cassette Exon	0,16
TC1100001661.mm.1	-1,8	Tmem106a	JUC1100008115.mm.1	2,25	0,016388	0,458738		
TC1100001661.mm.1	-1,8	Tmem106a	PSR1100015638.mm.1	2,13	0,006385	0,389571	Alternative 5' Donc	0,16
TC1800000071.mm.1	-1,03	Thoc1	JUC1800000218.mm.1	2,25	0,027081	0,502997		
TC1800000071.mm.1	-1,03	Thoc1	PSR1800000424.mm.1	2,13	0,034848	0,526823	Cassette Exon	0,16
TC1000000326.mm.1	1,53	Fyn	JUC1000001196.mm.1	2,25	0,002398	0,348564		
TC1000000326.mm.1	1,53	Fyn	PSR1000002167.mm.1	-2,21	0,031836	0,517856	Cassette Exon	0,14
TC1000000326.mm.1	1,53	Fyn	PSR1000002196.mm.1	-2,31	0,021045	0,480262	Alternative 5' Donc	0,15
TC1000000326.mm.1	1,53	Fyn	JUC1000001188.mm.1	-2,43	0,049926	0,56393		
TC0600000386.mm.1	1,43	Clec2l	PSR0600002959.mm.1	2,25	0,037116	0,532942	Cassette Exon	0,12
TC1700001315.mm.1	-1,13	Msh6	PSR1700012009.mm.1	2,25	0,035972	0,529465	Cassette Exon	0,12
TC0100001493.mm.1	-1,07	Scyl3	PSR0100012252.mm.1	2,25	0,000442	0,297771	Cassette Exon	0,11
TC0400003366.mm.1	-1,24	Kif2c	PSR0400026963.mm.1	2,25	0,031618	0,517391	Cassette Exon	0,11
TC1500000668.mm.1	-1,19	Gm10863	PSR1500005006.mm.1	2,25	0,035981	0,529491	Cassette Exon	0,11
TC1600000654.mm.1	1,53	Spice1	JUC1600002936.mm.1	2,25	0,031511	0,516965		
TC1600000654.mm.1	1,53	Spice1	PSR1600005693.mm.1	2,17	0,028452	0,507299	Cassette Exon	0,11
TC1600000654.mm.1	1,53	Spice1	PSR1600005704.mm.1	-2,09	0,010734	0,423745	Cassette Exon	0,11
TC1600000654.mm.1	1,53	Spice1	JUC1600002938.mm.1	-2,26	0,003643	0,356894		
TC1400000465.mm.1	-1,23	Gm8232; Gm21991	JUC1400002282.mm.1	2,25	0,01899	0,471485		
TC0300000323.mm.1	-1,14	D3Ertd751e	PSR0300002418.mm.1	2,25	0,030366	0,512923		
TC1200002581.mm.1	-1,34	Igh-V11; Ighv11-2	JUC1200010369.mm.1	2,25	0,001399	0,325997		
TC0200004555.mm.1	-1,44	Fahd2a	JUC0200020198.mm.1	2,25	0,026522	0,501411		
TC0200004555.mm.1	-1,44	Fahd2a	JUC0200020197.mm.1	2,05	0,016957	0,461296		
TC1700001166.mm.1	-1,01	Ltbp1	JUC1700006063.mm.1	2,25	0,02159	0,48325		
TC1700001166.mm.1	-1,01	Ltbp1	JUC1700006059.mm.1	-3,44	0,000644	0,304044		
TC0100001862.mm.1	-1,3	Gm10516	JUC0100008781.mm.1	2,25	0,017398	0,46374		
TC0500001888.mm.1	-1,01	Ankib1	JUC0500009471.mm.1	2,25	0,02496	0,495932		
TC0600000410.mm.1	-1	Ssbp1	JUC0600001597.mm.1	2,25	0,016788	0,460623		
TC0300003000.mm.1	-1,39	1700030L20Rik	JUC0300012373.mm.1	2,25	0,048076	0,559803		

TC1100001698.mm.1	1,31	Adam11	JUC1100008300.mm.1	2,25	0,012156	0,432923		
TC1100001698.mm.1	1,31	Adam11	JUC1100008290.mm.1	-2,45	0,000543	0,304044		
TC1100001247.mm.1	-1,3	Ap2b1	JUC1100006118.mm.1	2,25	0,026195	0,50003		
TC0400003327.mm.1	1,26	Faah	JUC0400013826.mm.1	2,25	0,046308	0,556186		
TC0400003327.mm.1	1,26	Faah	PSR0400026487.mm.1	-2,01	0,041914	0,545811		
TC0400003327.mm.1	1,26	Faah	JUC0400013815.mm.1	-2,02	0,019155	0,472667		
TC0400002757.mm.1	-1,67	Ptbp3	JUC0400011640.mm.1	2,25	0,013332	0,44185		
TC1400000365.mm.1	5,2	Rbp3	JUC1400001938.mm.1	2,24	0,028728	0,508141		
TC1400000365.mm.1	5,2	Rbp3	PSR1400003246.mm.1	-5,41	0,022816	0,488253	Alternative 3' Acce	0,48
TC0600001508.mm.1	-1,64	Foxm1; 4933413G19Rik	JUC0600006483.mm.1	2,24	0,00455	0,36886		
TC0600001508.mm.1	-1,64	Foxm1; 4933413G19Rik	PSR0600012294.mm.1	2,21	0,005295	0,378059	Alternative 5' Donc	0,2
TC0600001508.mm.1	-1,64	Foxm1; 4933413G19Rik	PSR0600012285.mm.1	2,02	0,001608	0,332831	Alternative 3' Acce	0,16
TC0400000213.mm.1	-1,37	Gpr63; Gm11906	PSR0400001401.mm.1	2,24	0,047874	0,559408	Alternative 5' Donc	0,19
TC1300001934.mm.1	-1,58	A330033J07Rik	PSR1300012041.mm.1	2,24	0,000308	0,288663	Alternative 3' Acce	0,19
TC0700001280.mm.1	-1,5	Sytl2	JUC0700005705.mm.1	2,24	0,025513	0,497664		
TC0700001280.mm.1	-1,5	Sytl2	PSR0700011055.mm.1	2,13	0,048064	0,55976	Alternative 3' Acce	0,18
TC1900000170.mm.1	-1,15	Eef1g; LOC101055956	PSR1900002344.mm.1	2,24	0,038744	0,537366	Alternative 5' Donc	0,18
TC1300001075.mm.1	-1,08	Zbed3	PSR1300006855.mm.1	2,24	0,024421	0,493848	Alternative 3' Acce	0,16
TC0400003587.mm.1	-1,86	C77080	PSR0400029231.mm.1	2,24	0,038043	0,535548	Cassette Exon	0,15
TC1600001704.mm.1	-1,33	Gm4827	JUC1600007246.mm.1	2,24	0,047029	0,557783		
TC1600001704.mm.1	-1,33	Gm4827	PSR1600013897.mm.1	2,07	0,009702	0,417746	Cassette Exon	0,15
TC1700000382.mm.1	2,75	Gng13	PSR1700003184.mm.1	2,24	0,003698	0,357266	Cassette Exon	0,09
TC1700000382.mm.1	2,75	Gng13	PSR1700003182.mm.1	-2,34	0,029862	0,511132	Cassette Exon	0,14
TC0400001070.mm.1	-1,25	Oma1	PSR0400007333.mm.1	2,24	0,020578	0,478502	Cassette Exon	0,13
TC0800002039.mm.1	1,09	Tnks	PSR0800015919.mm.1	2,24	0,019146	0,472526	Cassette Exon	0,11
TC0X00001100.mm.1	-1,41		PSR0X00007252.mm.1	2,24	0,038103	0,535659	Cassette Exon	0,11
TC1700001719.mm.1	-1,29	9630028I04Rik	PSR1700015910.mm.1	2,24	0,000209	0,28803	Cassette Exon	0,11
TC1800001243.mm.1	1,05	Nr3c1	PSR1800009242.mm.1	2,24	0,012471	0,435343	Cassette Exon	0,11
TC1800001243.mm.1	1,05	Nr3c1	JUC1800005137.mm.1	2,21	0,001281	0,322251		
TC0200003423.mm.1	-1,13	Nr5a1	JUC0200014806.mm.1	2,24	0,01493	0,451919		
TC1300001087.mm.1	1,56	Poc5	JUC1300003606.mm.1	2,24	0,049293	0,562691		
TC0100001804.mm.1	1,05	Gpatch2; Gm21710	JUC0100008519.mm.1	2,24	0,04772	0,55896		
TC1600001375.mm.1	-1,03	Igf2bp2	JUC1600005920.mm.1	2,24	0,04401	0,550779		
TC1600001163.mm.1	-1,65		JUC1600004866.mm.1	2,24	0,035345	0,528451		
TC0800000653.mm.1	-1,17	Sh3rf1	JUC0800002361.mm.1	2,24	0,049747	0,563579		
TC0800002409.mm.1	-1,24	Kcnn1	JUC0800009976.mm.1	2,24	0,008186	0,40568		
TC0800002934.mm.1	1,14	Ddx19a	JUC0800012323.mm.1	2,24	0,024052	0,493026		
TC0700000528.mm.1	1,23	Dpf1	JUC0700002267.mm.1	2,24	0,046077	0,555658		
TC0700000528.mm.1	1,23	Dpf1	JUC0700002276.mm.1	2,21	0,016609	0,459827		
TC0700000528.mm.1	1,23	Dpf1	JUC0700002281.mm.1	-2,24	0,027652	0,50467		
TC0700000528.mm.1	1,23	Dpf1	JUC0700002272.mm.1	-2,76	0,007243	0,39826		
TC0600002763.mm.1	1,31	Magi1	JUC0600011174.mm.1	2,24	0,02608	0,499688		
TC1100002575.mm.1	-1,07	Adra1b	JUC1100012376.mm.1	2,24	0,039383	0,539438		
TC0400002605.mm.1	-1,06	Tbc1d2	JUC0400011017.mm.1	2,24	0,001549	0,330631		
TC0400002580.mm.1	-1,28	Shb	JUC0400010913.mm.1	2,24	0,044066	0,55088		
TC0400002069.mm.1	-1,44	2610002J02Rik	JUC0400008991.mm.1	2,24	0,011181	0,426414		
TC1100003999.mm.1	2,88	Abca8a	JUC1100019608.mm.1	2,23	0,000085	0,255472		
TC1100003999.mm.1	2,88	Abca8a	JUC1100019616.mm.1	-2,06	0,001144	0,317344		

TC1100003999.mm.1	2,88	Abca8a	JUC1100019593.mm.1	-2,12	0,000454	0,298307		
TC1100003999.mm.1	2,88	Abca8a	PSR1100037483.mm.1	-2,24	0,034404	0,525784	Cassette Exon	0,15
TC1100003999.mm.1	2,88	Abca8a	JUC1100019604.mm.1	-2,32	0,025788	0,498505		
TC1100003999.mm.1	2,88	Abca8a	JUC1100019618.mm.1	-2,36	0,036891	0,532257		
TC1100003999.mm.1	2,88	Abca8a	PSR1100037515.mm.1	-2,4	0,020293	0,477245	Cassette Exon	0,18
TC1100003999.mm.1	2,88	Abca8a	PSR1100037496.mm.1	-2,46	0,017592	0,464302	Intron Retention	0,37
TC1100003999.mm.1	2,88	Abca8a	PSR1100037474.mm.1	-2,72	0,000787	0,309905	Alternative 3' Acce	0,22
TC1100003999.mm.1	2,88	Abca8a	PSR1100037520.mm.1	-2,73	0,000562	0,304044		
TC1100003999.mm.1	2,88	Abca8a	JUC1100019588.mm.1	-2,84	0,017317	0,463282		
TC1100003999.mm.1	2,88	Abca8a	PSR1100037519.mm.1	-2,91	0,004166	0,3623		
TC1100003999.mm.1	2,88	Abca8a	PSR1100037476.mm.1	-3,45	0,006354	0,389469	Alternative 5' Donc	0,37
TC1100003999.mm.1	2,88	Abca8a	JUC1100019620.mm.1	-3,72	0,012133	0,432868		
TC1100003999.mm.1	2,88	Abca8a	PSR1100037479.mm.1	-4,18	0,000719	0,306015	Alternative 3' Acce	0,49
TC1100003999.mm.1	2,88	Abca8a	JUC1100019589.mm.1	-4,83	0,017515	0,464019		
TC1100003999.mm.1	2,88	Abca8a	PSR1100037491.mm.1	-5,52	0,001658	0,334725	Alternative 5' Donc	0,48
TC1100003999.mm.1	2,88	Abca8a	JUC1100019624.mm.1	-6,61	0,017754	0,465425		
TC1400000604.mm.1	4,58	Rpgrip1	JUC1400002760.mm.1	2,23	0,005182	0,376851		
TC1400000604.mm.1	4,58	Rpgrip1	JUC1400002777.mm.1	2,18	0,005014	0,37387		
TC1400000604.mm.1	4,58	Rpgrip1	PSR1400004833.mm.1	-2,09	0,011423	0,427656	Alternative 3' Acce	0,17
TC1400000604.mm.1	4,58	Rpgrip1	PSR1400004865.mm.1	-2,16	0,031197	0,515739	Alternative 5' Donc	0,13
TC1400000604.mm.1	4,58	Rpgrip1	PSR1400004852.mm.1	-2,36	0,004772	0,371044	Alternative 3' Acce	0,17
TC1400000604.mm.1	4,58	Rpgrip1	PSR1400004818.mm.1	-2,56	0,014742	0,450615	Cassette Exon	0,23
TC1400000604.mm.1	4,58	Rpgrip1	JUC1400002762.mm.1	-2,71	0,002742	0,349612		
TC1400000604.mm.1	4,58	Rpgrip1	PSR1400004820.mm.1	-2,85	0,026655	0,501823	Cassette Exon	0,4
TC1400000604.mm.1	4,58	Rpgrip1	PSR1400004860.mm.1	-2,88	0,003775	0,357586	Alternative 5' Donc	0,42
TC1400000604.mm.1	4,58	Rpgrip1	JUC1400002797.mm.1	-3,18	0,014367	0,448293		
TC1400000604.mm.1	4,58	Rpgrip1	PSR1400004819.mm.1	-3,26	0,006477	0,390467	Cassette Exon	0,42
TC1400000604.mm.1	4,58	Rpgrip1	JUC1400002771.mm.1	-3,28	0,001565	0,331411		
TC1400000604.mm.1	4,58	Rpgrip1	PSR1400004831.mm.1	-3,38	0,001791	0,336311	Alternative 5' Donc	0,38
TC1400000604.mm.1	4,58	Rpgrip1	JUC1400002800.mm.1	-3,71	0,01432	0,448086		
TC1400000604.mm.1	4,58	Rpgrip1	JUC1400002799.mm.1	-3,83	0,004981	0,373851		
TC1400000604.mm.1	4,58	Rpgrip1	JUC1400002773.mm.1	-4,09	0,013502	0,443238		
TC1400000604.mm.1	4,58	Rpgrip1	PSR1400004855.mm.1	-5,71	0,030435	0,513139	Alternative 5' Donc	0,48
TC1400000604.mm.1	4,58	Rpgrip1	PSR1400004822.mm.1	-5,76	0,02046	0,478018	Alternative 5' Donc	0,46
TC1400000604.mm.1	4,58	Rpgrip1	PSR1400004809.mm.1	-7,11	0,003068	0,353892	Alternative 3' Acce	0,34
TC1400000604.mm.1	4,58	Rpgrip1	PSR1400004857.mm.1	-7,55	0,002927	0,352289	Alternative 5' Donc	0,48
TC1400000604.mm.1	4,58	Rpgrip1	JUC1400002792.mm.1	-8,71	0,023521	0,490947		
TC1400000604.mm.1	4,58	Rpgrip1	JUC1400002789.mm.1	-10,13	0,000746	0,30798		
TC1400000604.mm.1	4,58	Rpgrip1	JUC1400002790.mm.1	-10,63	0,007981	0,403853		
TC1400000604.mm.1	4,58	Rpgrip1	PSR1400004856.mm.1	-11,58	0,004098	0,36228	Alternative 5' Donc	0,48
TC1400000604.mm.1	4,58	Rpgrip1	JUC1400002787.mm.1	-13,1	0,004759	0,371039		
TC1400000604.mm.1	4,58	Rpgrip1	JUC1400002798.mm.1	-16,78	0,000448	0,297771		
TC1400002297.mm.1	4,43	Nefm	JUC1400009761.mm.1	2,23	0,037886	0,534993		
TC1400002297.mm.1	4,43	Nefm	JUC1400009760.mm.1	-2,58	0,002606	0,349501		
TC1400002297.mm.1	4,43	Nefm	PSR1400017779.mm.1	-2,78	0,045698	0,554833	Alternative 5' Donc	0,3
TC1400002297.mm.1	4,43	Nefm	PSR1400017772.mm.1	-3,09	0,003099	0,353892	Cassette Exon	0,33
TC1400002297.mm.1	4,43	Nefm	PSR1400017773.mm.1	-4,72	0,007931	0,403819	Cassette Exon	0,41
TC1400002297.mm.1	4,43	Nefm	PSR1400017774.mm.1	-4,8	0,007669	0,402377	Alternative 3' Acce	0,48

TC1400002297.mm.1	4,43	Nefm	PSR1400017780.mm.1	-5,13	0,003109	0,353892	Alternative 5' Donc	0,3
TC1400002297.mm.1	4,43	Nefm	JUC1400009758.mm.1	-8,51	0,003269	0,354243		
TC0100000216.mm.1	1,5	Dst; bpag1-e	JUC0100000734.mm.1	2,23	0,0189	0,47127		
TC0100000216.mm.1	1,5	Dst; bpag1-e	JUC0100000649.mm.1	2,17	0,022618	0,487464		
TC0100000216.mm.1	1,5	Dst; bpag1-e	JUC0100000675.mm.1	-2,04	0,04332	0,549306		
TC0100000216.mm.1	1,5	Dst; bpag1-e	PSR0100001299.mm.1	-2,05	0,033935	0,524252	Intron Retention	0,16
TC0100000216.mm.1	1,5	Dst; bpag1-e	PSR0100001307.mm.1	-2,1	0,017598	0,464312	Alternative 5' Donc	0,16
TC0100000216.mm.1	1,5	Dst; bpag1-e	JUC0100000686.mm.1	-2,17	0,028351	0,506843		
TC0100000216.mm.1	1,5	Dst; bpag1-e	PSR0100001330.mm.1	-2,31	0,017032	0,461729	Cassette Exon	0,26
TC0100000216.mm.1	1,5	Dst; bpag1-e	JUC0100000690.mm.1	-2,37	0,024523	0,493992		
TC0100000216.mm.1	1,5	Dst; bpag1-e	PSR0100001331.mm.1	-2,72	0,00632	0,38927	Cassette Exon	0,26
TC0100000216.mm.1	1,5	Dst; bpag1-e	JUC0100000747.mm.1	-2,78	0,020294	0,477245		
TC0100000216.mm.1	1,5	Dst; bpag1-e	PSR0100001305.mm.1	-2,96	0,005486	0,379939	Intron Retention	0,38
TC0100000216.mm.1	1,5	Dst; bpag1-e	JUC0100000688.mm.1	-3,97	0,03216	0,518685		
TC0600000398.mm.1	1,28	Tmem178b	PSR0600003102.mm.1	2,23	0,004665	0,370181	Cassette Exon	0,1
TC0600000398.mm.1	1,28	Tmem178b	PSR0600003114.mm.1	-2,69	0,044792	0,552442	Alternative 5' Donc	0,25
TC0800000831.mm.1	1,24	Nwd1	JUC0800003230.mm.1	2,23	0,026645	0,50179		
TC0800000831.mm.1	1,24	Nwd1	PSR0800006159.mm.1	-2,48	0,035324	0,528381	Alternative 5' Donc	0,24
TC0500003733.mm.1	1,58	Zfp644; LOC100504779	JUC0500013992.mm.1	2,23	0,017342	0,46353		
TC0500003733.mm.1	1,58	Zfp644; LOC100504779	JUC0500014006.mm.1	-2,05	0,048781	0,56148		
TC0500003733.mm.1	1,58	Zfp644; LOC100504779	JUC0500014002.mm.1	-2,25	0,02928	0,509874		
TC0500003733.mm.1	1,58	Zfp644; LOC100504779	PSR0500025701.mm.1	-2,41	0,018105	0,467161	Alternative 3' Acce	0,21
TC0500000044.mm.1	-1,22	Sri	PSR0500000378.mm.1	2,23	0,029682	0,510918	Alternative 5' Donc	0,2
TC1900000233.mm.1	1,29		PSR1900002827.mm.1	2,23	0,03935	0,539364	Alternative 5' Donc	0,2
TC0700003941.mm.1	-1,59	Trim30b	PSR0700032951.mm.1	2,23	0,040957	0,543459	Alternative 3' Acce	0,19
TC0200005066.mm.1	1,02	Chd6	JUC0200022323.mm.1	2,23	0,043299	0,549297		
TC0200005066.mm.1	1,02	Chd6	PSR0200043295.mm.1	-2,03	0,020822	0,47952	Alternative 3' Acce	0,16
TC0700004612.mm.1	1,02	Tpcn2	JUC0700020625.mm.1	2,23	0,018774	0,470789		
TC0700004612.mm.1	1,02	Tpcn2	JUC0700020633.mm.1	2,11	0,040989	0,543565		
TC0700004612.mm.1	1,02	Tpcn2	PSR0700039126.mm.1	-2,3	0,013989	0,446308	Cassette Exon	0,16
TC0X00002630.mm.1	-2,02		PSR0X00016405.mm.1	2,23	0,037522	0,533968	Alternative 3' Acce	0,15
TC0900002105.mm.1	-1,11	Crtam	PSR0900017387.mm.1	2,23	0,006431	0,389972	Cassette Exon	0,14
TC1100002912.mm.1	1,08	Rnf112	PSR1100026430.mm.1	2,23	0,000629	0,304044	Cassette Exon	0,14
TC1100002912.mm.1	1,08	Rnf112	PSR1100026432.mm.1	2,06	0,006999	0,395737	Cassette Exon	0,12
TC1600001380.mm.1	1,4	Dgkg	PSR1600011496.mm.1	2,23	0,014167	0,447466	Cassette Exon	0,13
TC1600001380.mm.1	1,4	Dgkg	JUC1600005964.mm.1	2,15	0,009469	0,416539		
TC0900002912.mm.1	-1,86	E330023G01Rik	PSR0900023970.mm.1	2,23	0,000087	0,255472	Cassette Exon	0,12
TC1700001827.mm.1	-1,43	Sik1	PSR1700016814.mm.1	2,23	0,049876	0,563773	Cassette Exon	0,12
TC0X00000512.mm.1	-1,23	3830403N18Rik	PSR0X00003341.mm.1	2,23	0,000337	0,289576	Cassette Exon	0,11
TC0X00000512.mm.1	-1,23	3830403N18Rik	JUC0X00001710.mm.1	-2,27	0,037521	0,533968		
TC1100000361.mm.1	-1,06	Kcnmb1	PSR1100002995.mm.1	2,23	0,011154	0,426295	Cassette Exon	0,11
TC0300002822.mm.1	-1,93	Snx7	PSR0300022340.mm.1	2,23	0,007936	0,403819	Cassette Exon	0,1
TC1100001644.mm.1	1,49	Cntnap1	JUC1100007943.mm.1	2,23	0,006833	0,393893		
TC1100001644.mm.1	1,49	Cntnap1	JUC1100007952.mm.1	2,2	0,001086	0,317191		
TC1100001644.mm.1	1,49	Cntnap1	PSR1100015315.mm.1	-2,03	0,014014	0,446443	Cassette Exon	0,1
TC0600002248.mm.1	-1	Dfna5	PSR0600017627.mm.1	2,23	0,038438	0,536405	Cassette Exon	0,07
TC0900002017.mm.1	1,72	Ddx25	PSR0900016777.mm.1	2,23	0,012214	0,433321	Cassette Exon	0,07
TC0500003610.mm.1	1,29	Gpr12	PSR0500033148.mm.1	2,23	0,015513	0,454456	Cassette Exon	0,06

TC0800000779.mm.1	1,22	Gm3643	PSR0800005527.mm.1	2,23	0,001081	0,317191	Cassette Exon	0,04
TC0800000779.mm.1	1,22	Gm3643	JUC0800002932.mm.1	-2,65	0,011717	0,429969		
TC1400001898.mm.1	1,12	Ero1l	JUC1400007882.mm.1	2,23	0,023361	0,490143		
TC1300000924.mm.1	-1	Ttc37	JUC1300003167.mm.1	2,23	0,001531	0,330287		
TC1800001746.mm.1	1,39	Hdhd2	JUC1800003293.mm.1	2,23	0,019434	0,473998		
TC1800000448.mm.1	-1,55	Spink5	JUC1800001818.mm.1	2,23	0,002694	0,349612		
TC1800000448.mm.1	-1,55	Spink5	JUC1800001841.mm.1	2,12	0,02762	0,504604		
TC1700002183.mm.1	1,39	Klc4	JUC1700010957.mm.1	2,23	0,004046	0,361816		
TC1500002071.mm.1	1,05	Kif21a	JUC1500009516.mm.1	2,23	0,032027	0,51843		
TC1600000190.mm.1	1,08	Prkdc	JUC1600000767.mm.1	2,23	0,020037	0,476453		
TC1600000190.mm.1	1,08	Prkdc	JUC1600000753.mm.1	-2,08	0,001756	0,335996		
TC1600000190.mm.1	1,08	Prkdc	JUC1600000752.mm.1	-3,21	0,006612	0,392269		
TC0200000365.mm.1	-1,08	4931423N10Rik	JUC0200000835.mm.1	2,23	0,006423	0,389915		
TC0500000920.mm.1	1,1	Gm5559	JUC0500004330.mm.1	2,23	0,040078	0,541392		
TC0700004219.mm.1	1,16	Cog7	JUC0700018509.mm.1	2,23	0,020312	0,477245		
TC0600000866.mm.1	-1,4	Suclg1	JUC0600003338.mm.1	2,23	0,002692	0,349612		
TC0600001970.mm.1	1,09	Rbm28	JUC0600008086.mm.1	2,23	0,018308	0,468049		
TC0700003005.mm.1	-1,01	Ruvbl2	JUC0700014544.mm.1	2,23	0,000748	0,308185		
TC0400000425.mm.1	1,41	N28178	JUC0400001292.mm.1	2,23	0,021238	0,481319		
TC0400000425.mm.1	1,41	N28178	JUC0400001298.mm.1	-2,03	0,00949	0,416578		
TC0400001177.mm.1	-1,12	Dmrta2	JUC0400004421.mm.1	2,23	0,006385	0,389571		
TC0300001447.mm.1	-1,11	Gbp2	JUC0300006136.mm.1	2,23	0,03611	0,529748		
TC0400002043.mm.1	-1,09	Megf6	JUC0400008863.mm.1	2,23	0,006583	0,39213		
TC0700004650.mm.1	17,19	Ipw; Snord116	PSR0700028491.mm.1	2,22	0,028422	0,507179	Cassette Exon	0,11
TC0700004650.mm.1	17,19	Ipw; Snord116	PSR0700028515.mm.1	-2,19	0,024483	0,493992	Cassette Exon	0,09
TC0700004650.mm.1	17,19	Ipw; Snord116	PSR0700028499.mm.1	-3,02	0,039207	0,538692	Cassette Exon	0,12
TC0700004650.mm.1	17,19	Ipw; Snord116	JUC0700015106.mm.1	-3,87	0,002917	0,352289		
TC0700004650.mm.1	17,19	Ipw; Snord116	PSR0700028518.mm.1	-4,44	0,004878	0,372195	Cassette Exon	0,55
TC0700004650.mm.1	17,19	Ipw; Snord116	JUC0700015097.mm.1	-5,17	0,00403	0,361636		
TC0700004650.mm.1	17,19	Ipw; Snord116	PSR0700028522.mm.1	-5,23	0,003695	0,357266	Cassette Exon	0,41
TC0700004650.mm.1	17,19	Ipw; Snord116	JUC0700015102.mm.1	-5,44	0,003342	0,354243		
TC0700004650.mm.1	17,19	Ipw; Snord116	JUC0700015103.mm.1	-5,44	0,003342	0,354243		
TC0700004650.mm.1	17,19	Ipw; Snord116	JUC0700015108.mm.1	-5,44	0,003342	0,354243		
TC0700004650.mm.1	17,19	Ipw; Snord116	PSR0700028520.mm.1	-5,93	0,008429	0,406611	Cassette Exon	0,41
TC0700004650.mm.1	17,19	Ipw; Snord116	PSR0700028475.mm.1	-6,52	0,001518	0,330268	Cassette Exon	0,3
TC0700004650.mm.1	17,19	Ipw; Snord116	PSR0700028479.mm.1	-6,57	0,001591	0,331726	Cassette Exon	0,43
TC0700004650.mm.1	17,19	Ipw; Snord116	PSR0700028487.mm.1	-7,38	0,000785	0,309887	Alternative 3' Acce	0,5
TC0700004650.mm.1	17,19	Ipw; Snord116	JUC0700015151.mm.1	-8,51	0,004708	0,370888		
TC0700004650.mm.1	17,19	Ipw; Snord116	PSR0700028517.mm.1	-9,18	0,008061	0,40448	Cassette Exon	0,41
TC0700004650.mm.1	17,19	Ipw; Snord116	PSR0700028478.mm.1	-9,72	0,000074	0,251428	Cassette Exon	0,3
TC0700004650.mm.1	17,19	Ipw; Snord116	JUC0700015146.mm.1	-10,54	0,003439	0,3554		
TC0700004650.mm.1	17,19	Ipw; Snord116	PSR0700028509.mm.1	-11,08	0,00296	0,352501	Cassette Exon	0,29
TC0700004650.mm.1	17,19	Ipw; Snord116	PSR0700028512.mm.1	-11,82	0,000596	0,304044	Alternative 5' Donc	0,49
TC0700004650.mm.1	17,19	Ipw; Snord116	PSR0700028477.mm.1	-12,1	0,007126	0,397139	Cassette Exon	0,3
TC0700004650.mm.1	17,19	Ipw; Snord116	PSR0700028507.mm.1	-13,17	0,00378	0,357586	Alternative 5' Donc	0,48
TC0700004650.mm.1	17,19	Ipw; Snord116	PSR0700028471.mm.1	-13,95	0,002728	0,349612	Cassette Exon	0,28
TC0700004650.mm.1	17,19	Ipw; Snord116	PSR0700028472.mm.1	-14,7	0,001226	0,322251	Cassette Exon	0,28
TC0700004650.mm.1	17,19	Ipw; Snord116	PSR0700028508.mm.1	-15,49	0,000606	0,304044	Cassette Exon	0,42

TC0700004650.mm.1	17,19	Ipw; Snord116	PSR0700028474.mm.1	-17,63	0,000046	0,24627	Cassette Exon	0,3
TC0700004650.mm.1	17,19	Ipw; Snord116	PSR0700028494.mm.1	-18,88	0,001904	0,338712	Cassette Exon	0,28
TC0700004650.mm.1	17,19	Ipw; Snord116	PSR0700028488.mm.1	-19,09	0,002248	0,34578	Alternative 3' Acce	0,5
TC0700004650.mm.1	17,19	Ipw; Snord116	JUC0700015142.mm.1	-19,47	0,004533	0,368648		
TC0700004650.mm.1	17,19	Ipw; Snord116	JUC0700015114.mm.1	-19,51	0,000623	0,304044		
TC0700004650.mm.1	17,19	Ipw; Snord116	PSR0700028469.mm.1	-20,65	0,000117	0,269605	Cassette Exon	0,28
TC0700004650.mm.1	17,19	Ipw; Snord116	JUC0700015126.mm.1	-24,94	0,000409	0,297771		
TC0700004650.mm.1	17,19	Ipw; Snord116	JUC0700015144.mm.1	-28,29	0,001019	0,316361		
TC0700004650.mm.1	17,19	Ipw; Snord116	JUC0700015135.mm.1	-32,58	0,003963	0,359922		
TC0700004650.mm.1	17,19	Ipw; Snord116	PSR0700028521.mm.1	-39,44	0,001922	0,34017	Cassette Exon	0,41
TC0700004650.mm.1	17,19	Ipw; Snord116	JUC0700015141.mm.1	-40,11	0,000715	0,306015		
TC0200002829.mm.1	3,21	Uckl1os; Gm16119	PSR0200023372.mm.1	2,22	0,033703	0,523549	Alternative 5' Donc	0,13
TC0200002829.mm.1	3,21	Uckl1os; Gm16119	PSR0200023364.mm.1	-2,6	0,01946	0,474165	Alternative 3' Acce	0,09
TC0200002829.mm.1	3,21	Uckl1os; Gm16119	PSR0200023359.mm.1	-2,79	0,03179	0,517596	Alternative 3' Acce	0,17
TC0200002829.mm.1	3,21	Uckl1os; Gm16119	PSR0200023379.mm.1	-3,25	0,043169	0,548781	Alternative 5' Donc	0,32
TC0600001243.mm.1	1,24	Mtmr14	PSR0600009523.mm.1	2,22	0,023482	0,490673	Alternative 3' Acce	0,15
TC0600001243.mm.1	1,24	Mtmr14	PSR0600009519.mm.1	-2,19	0,01147	0,428022	Cassette Exon	0,11
TC0600001243.mm.1	1,24	Mtmr14	PSR0600009543.mm.1	-2,24	0,02737	0,503961	Alternative 5' Donc	0,19
TC0600001243.mm.1	1,24	Mtmr14	JUC0600004914.mm.1	-2,42	0,008083	0,404868		
TC1100002850.mm.1	1,35	Prss38	PSR1100025837.mm.1	2,22	0,033814	0,523913	Alternative 3' Acce	0,18
TC1900000848.mm.1	-1,32		PSR1900007352.mm.1	2,22	0,011853	0,430752	Alternative 3' Acce	0,17
TC1200001972.mm.1	-1,06	Plek2	JUC1200007497.mm.1	2,22	0,043069	0,548423		
TC1200001972.mm.1	-1,06	Plek2	PSR1200013515.mm.1	-2,09	0,024721	0,494838	Cassette Exon	0,16
TC1200001972.mm.1	-1,06	Plek2	JUC1200007490.mm.1	-2,19	0,013512	0,443333		
TC1300000187.mm.1	-1,4	Pom121l2	PSR1300001242.mm.1	2,22	0,00298	0,352501	Cassette Exon	0,14
TC0500000125.mm.1	-1,08	Hgf	PSR0500001008.mm.1	2,22	0,018574	0,469618	Alternative 5' Donc	0,13
TC0500000125.mm.1	-1,08	Hgf	PSR0500001006.mm.1	-2,24	0,016446	0,459051	Cassette Exon	0,1
TC0900001576.mm.1	-1,09	Acaa1a	PSR0900013144.mm.1	2,22	0,017385	0,463704	Intron Retention	0,13
TC0800003202.mm.1	1,16	Rbm34	PSR0800024633.mm.1	2,22	0,009905	0,418332	Cassette Exon	0,1
TC0400003071.mm.1	-1,11	Caap1	PSR0400024561.mm.1	2,22	0,015069	0,45236	Cassette Exon	0,09
TC0600001839.mm.1	1,97	Asns	JUC0600007575.mm.1	2,22	0,008933	0,41101		
TC0600001839.mm.1	1,97	Asns	PSR0600014487.mm.1	2,07	0,007674	0,402377	Cassette Exon	0,09
TC0600001839.mm.1	1,97	Asns	JUC0600007567.mm.1	-2,18	0,036719	0,531762		
TC1100003005.mm.1	-1,16	Glp2r	PSR1100027245.mm.1	2,22	0,014796	0,450927	Cassette Exon	0,08
TC0600002870.mm.1	-1,09	Cntn3	JUC0600011456.mm.1	2,22	0,018405	0,468484		
TC0600002870.mm.1	-1,09	Cntn3	PSR0600022044.mm.1	2,08	0,048036	0,559634	Cassette Exon	0,06
TC0300000889.mm.1	-1,9	Tmod4	JUC0300003600.mm.1	2,22	0,024225	0,493294		
TC1300001927.mm.1	1,01	Tpmt	JUC1300006163.mm.1	2,22	0,020442	0,478018		
TC1300001927.mm.1	1,01	Tpmt	JUC1300006165.mm.1	2,17	0,003879	0,359196		
TC0100003270.mm.1	-1,06		JUC0100015122.mm.1	2,22	0,003501	0,355638		
TC1900000549.mm.1	-1,32	Slc35g1	JUC1900002618.mm.1	2,22	0,038331	0,53622		
TC0200001056.mm.1	-1,95	Cdca7	JUC0200004445.mm.1	2,22	0,031728	0,517489		
TC0200001056.mm.1	-1,95	Cdca7	JUC0200004446.mm.1	2,15	0,032776	0,520748		
TC0200001002.mm.1	-1,12	Nostrin	JUC0200004014.mm.1	2,22	0,038558	0,536762		
TC1100000957.mm.1	-1,81	Mink1	JUC1100004425.mm.1	2,22	0,041288	0,544179		
TC1000002068.mm.1	-1,43	Gm15200	JUC1000008036.mm.1	2,22	0,015477	0,45435		
TC1100003147.mm.1	-1,18	Spns3	JUC1100015309.mm.1	2,22	0,006476	0,390467		
TC0X00000659.mm.1	-1,19	Gm1141	JUC0X00002091.mm.1	2,22	0,004938	0,373093		

TC0400002962.mm.1	-1,28	Bnc2	JUC0400012555.mm.1	2,22	0,017627	0,464508		
TC0400002962.mm.1	-1,28	Bnc2	JUC0400012566.mm.1	-2,66	0,042116	0,546549		
TC0X00001781.mm.1	-1,23	Magix	JUC0X00005689.mm.1	2,22	0,032452	0,519591		
TC0X00001781.mm.1	-1,23	Magix	JUC0X00005694.mm.1	2,02	0,001991	0,341091		
TC1000000574.mm.1	1,28	Aifm2	JUC1000002152.mm.1	2,22	0,008415	0,406611		
TC1800000658.mm.1	4,14	St8sia3	PSR1800004850.mm.1	2,21	0,001428	0,327447	Alternative 5' Donc	0,14
TC1800000658.mm.1	4,14	St8sia3	PSR1800004842.mm.1	-3,36	0,022367	0,486408	Cassette Exon	0,38
TC1800000658.mm.1	4,14	St8sia3	PSR1800004840.mm.1	-4,27	0,01629	0,458454	Cassette Exon	0,28
TC1800000658.mm.1	4,14	St8sia3	PSR1800004843.mm.1	-4,63	0,003281	0,354243	Cassette Exon	0,38
TC1800000658.mm.1	4,14	St8sia3	PSR1800004846.mm.1	-5,09	0,003122	0,353892	Alternative 3' Acce	0,48
TC1800000658.mm.1	4,14	St8sia3	JUC1800002709.mm.1	-5,68	0,020968	0,479989		
TC1800000658.mm.1	4,14	St8sia3	JUC1800002705.mm.1	-7,64	0,024449	0,493957		
TC1300000998.mm.1	4,05	Edil3	PSR1300006420.mm.1	2,21	0,030071	0,5119	Cassette Exon	0,11
TC1300000998.mm.1	4,05	Edil3	PSR1300006412.mm.1	-2,95	0,002239	0,34578		
TC1300000998.mm.1	4,05	Edil3	PSR1300006415.mm.1	-2,98	0,048035	0,559634	Alternative 5' Donc	0,3
TC1300000998.mm.1	4,05	Edil3	PSR1300006419.mm.1	-3,15	0,027745	0,505023	Alternative 5' Donc	0,32
TC1300000998.mm.1	4,05	Edil3	PSR1300006417.mm.1	-3,2	0,008371	0,406476	Alternative 5' Donc	0,37
TC1300000998.mm.1	4,05	Edil3	PSR1300006407.mm.1	-3,38	0,014611	0,449914	Cassette Exon	0,25
TC1300000998.mm.1	4,05	Edil3	PSR1300006428.mm.1	-3,41	0,037636	0,534189	Alternative 5' Donc	0,3
TC1300000998.mm.1	4,05	Edil3	PSR1300006425.mm.1	-3,46	0,031969	0,518183	Cassette Exon	0,2
TC1300000998.mm.1	4,05	Edil3	PSR1300006411.mm.1	-3,58	0,003665	0,357266		
TC1300000998.mm.1	4,05	Edil3	JUC1300003372.mm.1	-4,37	0,006284	0,388916		
TC1300000998.mm.1	4,05	Edil3	PSR1300006408.mm.1	-4,45	0,042884	0,548178	Alternative 3' Acce	0,34
TC1300000998.mm.1	4,05	Edil3	PSR1300006401.mm.1	-6,89	0,006898	0,394731	Cassette Exon	0,25
TC0200002012.mm.1	-1,03	Mavs	PSR0200015990.mm.1	2,21	0,044305	0,551368	Alternative 3' Acce	0,19
TC0400001359.mm.1	1,38	Rims3	PSR0400010316.mm.1	2,21	0,046357	0,556272	Alternative 5' Donc	0,19
TC0500003589.mm.1	-1,01	Pdap1	PSR0500032925.mm.1	2,21	0,021187	0,481033	Alternative 5' Donc	0,18
TC1200000200.mm.1	-1,44	Grhl1	PSR1200001566.mm.1	2,21	0,035869	0,529376	Alternative 3' Acce	0,17
TC0200004114.mm.1	1,91	1110051M20Rik	PSR0200034470.mm.1	2,21	0,000154	0,272178	Cassette Exon	0,12
TC0200004114.mm.1	1,91	1110051M20Rik	PSR0200034476.mm.1	-2,38	0,035925	0,529399	Cassette Exon	0,16
TC0200004114.mm.1	1,91	1110051M20Rik	JUC0200017616.mm.1	-3,21	0,010486	0,421937		
TC1100003450.mm.1	-1,35	Vmp1; Mir21a; Mir21	PSR1100031706.mm.1	2,21	0,046401	0,556478	Cassette Exon	0,16
TC1100003450.mm.1	-1,35	Vmp1; Mir21a; Mir21	JUC1100016576.mm.1	2,16	0,011408	0,427567		
TC1100003450.mm.1	-1,35	Vmp1; Mir21a; Mir21	PSR1100031699.mm.1	2,02	0,002012	0,341091	Cassette Exon	0,14
TC1100003450.mm.1	-1,35	Vmp1; Mir21a; Mir21	PSR1100031709.mm.1	2,02	0,004099	0,36228	Cassette Exon	0,11
TC0500002859.mm.1	1,18	Wdfy3	JUC0500013720.mm.1	2,21	0,012511	0,435429		
TC0500002859.mm.1	1,18	Wdfy3	PSR0500025242.mm.1	-2,15	0,013448	0,442693	Cassette Exon	0,15
TC0X00000359.mm.1	-1,74	Rhox10	PSR0X00002466.mm.1	2,21	0,001313	0,324276	Cassette Exon	0,15
TC0400004186.mm.1	-1,45	Nfia	PSR0400006497.mm.1	2,21	0,00102	0,316361	Cassette Exon	0,13
TC0400003545.mm.1	1,35	Ago4; Eif2c4	PSR0400028890.mm.1	2,21	0,04977	0,563646	Cassette Exon	0,12
TC0400004066.mm.1	-1,07	Slc45a1	PSR0400033793.mm.1	2,21	0,016738	0,460345	Cassette Exon	0,12
TC0400001441.mm.1	-2,05		PSR0400010937.mm.1	2,21	0,01031	0,420505	Cassette Exon	0,11
TC0600002047.mm.1	-1,66	Gm13858	PSR0600016110.mm.1	2,21	0,040804	0,5432	Cassette Exon	0,11
TC0900000641.mm.1	-1,48	4930550C14Rik	PSR0900004568.mm.1	2,21	0,011672	0,429939	Cassette Exon	0,11
TC1100002826.mm.1	-1,5	Olfr328	PSR1100025554.mm.1	2,21	0,019986	0,476227	Cassette Exon	0,11
TC1200000160.mm.1	1,11		PSR1200001326.mm.1	2,21	0,009244	0,414452	Cassette Exon	0,11
TC1400002215.mm.1	-1,32	Fdft1	PSR1400017146.mm.1	2,21	0,00757	0,401494	Cassette Exon	0,11
TC0800003200.mm.1	1,61	Tomm20	PSR0800024613.mm.1	2,21	0,004778	0,371044	Alternative 3' Acce	0,08

TC1400000226.mm.1	-1,37	E330034G19Rik	JUC1400001016.mm.1	2,21	0,033895	0,524202		
TC1400002790.mm.1	-1,06	2610042L04Rik	JUC1400000143.mm.1	2,21	0,002627	0,349612		
TC1200000729.mm.1	-1,97	Gm26669	JUC1200002943.mm.1	2,21	0,007704	0,402552		
TC0300000876.mm.1	-1,07		JUC0300003510.mm.1	2,21	0,012114	0,432704		
TC0200004443.mm.1	1,64	Ndufaf1	JUC0200019000.mm.1	2,21	0,017701	0,465159		
TC1500000019.mm.1	-1,69	Rpl37; Gm13826	JUC1500000106.mm.1	2,21	0,040073	0,541386		
TC1600000135.mm.1	1,23	2610020C07Rik	JUC1600000476.mm.1	2,21	0,00808	0,404795		
TC1600000918.mm.1	1,81	Ncam2	JUC1600003819.mm.1	2,21	0,003924	0,359818		
TC0500000896.mm.1	1,16	Ccng2	PSR0500007739.mm.1	2,21	0,007098	0,396897		
TC0500000896.mm.1	1,16	Ccng2	JUC0500004221.mm.1	2,18	0,039924	0,540966		
TC0900000363.mm.1	1,25	Hepacam	JUC0900001426.mm.1	2,21	0,009093	0,412827		
TC0500003381.mm.1	-1,12	Wbscr28	JUC0500016640.mm.1	2,21	0,038998	0,537976		
TC0300001960.mm.1	-1,52	Maml3	JUC0300007844.mm.1	2,21	0,035646	0,528956		
TC0300001960.mm.1	-1,52	Maml3	PSR0300014963.mm.1	2,11	0,026895	0,502518		
TC1100003481.mm.1	-1,72		JUC1100016694.mm.1	2,21	0,038789	0,537528		
TC1100001382.mm.1	-1,13	4930556N13Rik	JUC1100006799.mm.1	2,21	0,012021	0,432286		
TC1100001945.mm.1	1,12	Mgat5b	JUC1100009655.mm.1	2,21	0,046356	0,556272		
TC0900002836.mm.1	-1,33	Zic1	JUC0900013132.mm.1	2,21	0,018585	0,469618		
TC0900002156.mm.1	4,1	C2cd2l	PSR0900017914.mm.1	2,2	0,032376	0,519221	Cassette Exon	0,1
TC0900002156.mm.1	4,1	C2cd2l	PSR0900017901.mm.1	-2,15	0,000231	0,28803	Alternative 3' Acce	0,19
TC0900002156.mm.1	4,1	C2cd2l	JUC0900010087.mm.1	-2,84	0,005233	0,377425		
TC0900002156.mm.1	4,1	C2cd2l	JUC0900010086.mm.1	-3,29	0,009114	0,413086		
TC0900002156.mm.1	4,1	C2cd2l	PSR0900017899.mm.1	-3,74	0,009302	0,414883	Alternative 3' Acce	0,39
TC0900002156.mm.1	4,1	C2cd2l	PSR0900017916.mm.1	-4,03	0,003467	0,355628	Alternative 5' Donc	0,39
TC1900001072.mm.1	3,12	Gng3	JUC1900005482.mm.1	2,2	0,017529	0,464105		
TC1900001072.mm.1	3,12	Gng3	PSR1900010057.mm.1	-2,79	0,031641	0,517405	Alternative 3' Acce	0,27
TC1900001231.mm.1	6,55	Rorb	PSR1900011178.mm.1	2,2	0,004686	0,370338	Alternative 5' Donc	0,11
TC1900001231.mm.1	6,55	Rorb	PSR1900011196.mm.1	-2,2	0,002339	0,347265	Cassette Exon	0,25
TC1900001231.mm.1	6,55	Rorb	JUC1900006124.mm.1	-2,73	0,021052	0,480262		
TC1900001231.mm.1	6,55	Rorb	JUC1900006126.mm.1	-5,52	0,001246	0,322251		
TC0400002367.mm.1	1,35	Ankrd6	PSR0400019236.mm.1	2,2	0,037751	0,534682	Alternative 3' Acce	0,23
TC0400002367.mm.1	1,35	Ankrd6	JUC0400010029.mm.1	-2,95	0,004601	0,369794		
TC0900002212.mm.1	-1,59	Tagln	PSR0900018453.mm.1	2,2	0,016588	0,459685	Alternative 5' Donc	0,19
TC1000001917.mm.1	1,01	Gm8709	PSR1000013805.mm.1	2,2	0,005144	0,376442	Alternative 5' Donc	0,18
TC1200002416.mm.1	1,34	Ppp1r13b	PSR1200016802.mm.1	2,2	0,00823	0,405976	Cassette Exon	0,14
TC0100001063.mm.1	-1,59	Tfcp2l1; Gm23497	PSR0100008789.mm.1	2,2	0,044918	0,552764	Cassette Exon	0,13
TC0X00002999.mm.1	-1,09	Armcx6	PSR0X00018714.mm.1	2,2	0,003962	0,359922	Cassette Exon	0,13
TC1600000205.mm.1	-1,01	Ypel1	PSR1600001658.mm.1	2,2	0,016547	0,459612	Cassette Exon	0,13
TC1100001108.mm.1	1,22	Tmigd1	PSR1100010045.mm.1	2,2	0,01932	0,473294	Cassette Exon	0,11
TC1200002432.mm.1	1,04	Tmem179	PSR1200016871.mm.1	2,2	0,000967	0,313456	Cassette Exon	0,11
TC1400001174.mm.1	-2,04	Uchl3	PSR1400009166.mm.1	2,2	0,000445	0,297771	Cassette Exon	0,11
TC0600000413.mm.1	-1,94	Tas2r108	PSR0600003203.mm.1	2,2	0,026537	0,501411	Cassette Exon	0,11
TC1200001108.mm.1	1,56	Cyp46a1	JUC1200004390.mm.1	2,2	0,014021	0,446501		
TC1200001108.mm.1	1,56	Cyp46a1	JUC1200004395.mm.1	-2,76	0,00327	0,354243		
TC1900000940.mm.1	1,51	Bbs1	JUC1900004554.mm.1	2,2	0,045834	0,555013		
TC0100000268.mm.1	-1,44	Mrpl30; Gm6238	JUC0100001063.mm.1	2,2	0,027127	0,503022		
TC1900001722.mm.1	-1,2	Gfra1	JUC1900008497.mm.1	2,2	0,018274	0,468043		
TC1500001878.mm.1	-1,06	Dmc1	JUC1500008392.mm.1	2,2	0,037424	0,533831		

TC1500001920.mm.1	-1,47	St13	JUC1500008556.mm.1	2,2	0,046284	0,556186		
TC1500001920.mm.1	-1,47	St13	JUC1500008562.mm.1	2,1	0,046009	0,555624		
TC1500000793.mm.1	-1,01	Arhgap8	JUC1500003518.mm.1	2,2	0,033032	0,521499		
TC0200000814.mm.1	-1,37		JUC0200003410.mm.1	2,2	0,016504	0,459434		
TC1100003693.mm.1	-1,16	Top2a; Gm23451	JUC1100017837.mm.1	2,2	0,027807	0,505442		
TC0800002710.mm.1	-1,04		JUC0800011308.mm.1	2,2	0,023175	0,489953		
TC0600002139.mm.1	-1,32	Prss37	JUC0600008853.mm.1	2,2	0,005097	0,375732		
TC0600002139.mm.1	-1,32	Prss37	JUC0600008858.mm.1	-2,56	0,0455	0,554359		
TC1100001419.mm.1	1,04	Mbtd1	JUC1100006905.mm.1	2,2	0,012693	0,43642		
TC1100001419.mm.1	1,04	Mbtd1	JUC1100006920.mm.1	-2,79	0,028194	0,506368		
TC0400004112.mm.1	-1,08	Smim1	PSR0400034307.mm.1	2,2	0,019086	0,472208		
TC0900002982.mm.1	-1,4	Ackr4	JUC0900013696.mm.1	2,2	0,021785	0,48438		
TC0400003730.mm.1	1,06	Catsper4	JUC0400015945.mm.1	2,2	0,011074	0,42589		
TC0400003730.mm.1	1,06	Catsper4	JUC0400015942.mm.1	-3,2	0,034966	0,52724		
TC1000000446.mm.1	-1,17	Ascc3	JUC1000001626.mm.1	2,2	0,017395	0,463736		
TC1000000446.mm.1	-1,17	Ascc3	JUC1000001610.mm.1	2,04	0,002316	0,346468		
TC0400000278.mm.1	7,32	Gabrr2	JUC0400000906.mm.1	2,19	0,012189	0,433076		
TC0400000278.mm.1	7,32	Gabrr2	PSR0400001809.mm.1	-2,6	0,02039	0,477777	Cassette Exon	0,21
TC0400000278.mm.1	7,32	Gabrr2	JUC0400000902.mm.1	-3,11	0,000852	0,311909		
TC0400000278.mm.1	7,32	Gabrr2	PSR0400001814.mm.1	-5,01	0,01019	0,419711	Cassette Exon	0,44
TC0400000278.mm.1	7,32	Gabrr2	JUC0400000909.mm.1	-6,03	0,028471	0,507299		
TC0400000278.mm.1	7,32	Gabrr2	JUC0400000910.mm.1	-15,68	0,009171	0,413447		
TC1700001620.mm.1	1,96	Tbc1d24; Ntn3	PSR1700014274.mm.1	2,19	0,006029	0,386586	Cassette Exon	0,02
TC1700001620.mm.1	1,96	Tbc1d24; Ntn3	PSR1700014280.mm.1	-2,09	0,00253	0,349501	Cassette Exon	0,17
TC1700001620.mm.1	1,96	Tbc1d24; Ntn3	JUC1700007766.mm.1	-2,09	0,046267	0,556184		
TC1700001620.mm.1	1,96	Tbc1d24; Ntn3	JUC1700007763.mm.1	-2,12	0,040072	0,541386		
TC1700001620.mm.1	1,96	Tbc1d24; Ntn3	PSR1700014290.mm.1	-2,24	0,025324	0,497278	Alternative 5' Donc	0,08
TC1700001620.mm.1	1,96	Tbc1d24; Ntn3	JUC1700007776.mm.1	-2,32	0,038998	0,537976		
TC1700001620.mm.1	1,96	Tbc1d24; Ntn3	JUC1700007777.mm.1	-2,6	0,026353	0,500865		
TC1700001620.mm.1	1,96	Tbc1d24; Ntn3	PSR1700014286.mm.1	-2,67	0,02208	0,48541	Cassette Exon	0,21
TC1700001620.mm.1	1,96	Tbc1d24; Ntn3	PSR1700014288.mm.1	-2,69	0,010608	0,422786	Alternative 3' Acce	0,22
TC1700001620.mm.1	1,96	Tbc1d24; Ntn3	PSR1700014287.mm.1	-2,76	0,024122	0,493227	Cassette Exon	0,29
TC1700001620.mm.1	1,96	Tbc1d24; Ntn3	JUC1700007764.mm.1	-2,93	0,008004	0,403872		
TC1700001620.mm.1	1,96	Tbc1d24; Ntn3	JUC1700007773.mm.1	-2,99	0,028066	0,505968		
TC1700001620.mm.1	1,96	Tbc1d24; Ntn3	PSR1700014277.mm.1	-3,02	0,034874	0,526858	Cassette Exon	0,24
TC1700001620.mm.1	1,96	Tbc1d24; Ntn3	PSR1700014285.mm.1	-4,31	0,003773	0,357586	Cassette Exon	0,28
TC0X00001508.mm.1	1,07	Fgd1	PSR0X00009422.mm.1	2,19	0,003606	0,356223	Alternative 5' Donc	0,22
TC0X00001508.mm.1	1,07	Fgd1	PSR0X00009421.mm.1	-2,31	0,030501	0,51337	Cassette Exon	0,15
TC1000003207.mm.1	4,91	Neurod4	PSR1000023994.mm.1	2,19	0,003564	0,356223	Cassette Exon	0,03
TC1000003207.mm.1	4,91	Neurod4	PSR1000023995.mm.1	-2,19	0,003564	0,356223	Cassette Exon	0,19
TC1500000733.mm.1	1,85	Csdc2	PSR1500005733.mm.1	2,19	0,009994	0,418456	Alternative 5' Donc	0,19
TC1500000733.mm.1	1,85	Csdc2	PSR1500005734.mm.1	-2,02	0,025752	0,498405	Alternative 3' Acce	0,16
TC0100002210.mm.1	-1,54	Mitd1	PSR0100017809.mm.1	2,19	0,001886	0,338712	Alternative 3' Acce	0,18
TC0400004189.mm.1	-1	Ccdc163	PSR0400009408.mm.1	2,19	0,006574	0,39213	Alternative 3' Acce	0,18
TC1100000637.mm.1	-1,04	Hint1	PSR1100005143.mm.1	2,19	0,010536	0,422281	Alternative 3' Acce	0,18
TC0300002657.mm.1	1,76	Fam19a3	JUC0300010758.mm.1	2,19	0,041429	0,544646		
TC0300002657.mm.1	1,76	Fam19a3	PSR0300020694.mm.1	-2,6	0,039734	0,540494	Cassette Exon	0,15
TC0400000164.mm.1	-1,01	Rmdn1; Gm12353	PSR0400001035.mm.1	2,19	0,012532	0,43545	Cassette Exon	0,15

TC0800001928.mm.1	1,24	Hook3	PSR0800015454.mm.1	2,19	0,01659	0,459691	Cassette Exon	0,14
TC1600001050.mm.1	-1,71	Chaf1b	PSR1600008184.mm.1	2,19	0,02985	0,511132	Alternative 5' Donc	0,14
TC0200000798.mm.1	1,2	Epc2	PSR0200006613.mm.1	2,19	0,012815	0,437459	Cassette Exon	0,13
TC0700002856.mm.1	-1,46	Pdcd5; Gm3837	PSR0700025539.mm.1	2,19	0,04033	0,541841	Alternative 5' Donc	0,13
TC0500000337.mm.1	-1,37	Plb1	PSR0500003083.mm.1	2,19	0,028031	0,505851	Cassette Exon	0,12
TC1700000009.mm.1	-1,04	Tiam2	PSR1700000087.mm.1	2,19	0,047985	0,559574	Cassette Exon	0,12
TC1700000009.mm.1	-1,04	Tiam2	JUC1700000032.mm.1	-2,02	0,04228	0,547133		
TC0800003160.mm.1	1,5	Ccsap	PSR0800024242.mm.1	2,19	0,017902	0,466466	Cassette Exon	0,11
TC0800003160.mm.1	1,5	Ccsap	JUC0800013225.mm.1	-3,64	0,015211	0,452773		
TC1100001015.mm.1	-1,09	Itgae	PSR1100009204.mm.1	2,19	0,017915	0,466466	Cassette Exon	0,11
TC1700002755.mm.1	-1,27	Mettl4	PSR1700024694.mm.1	2,19	0,02301	0,48912	Cassette Exon	0,05
TC1400002843.mm.1	-1,35	LOC100861615; Gm3411	JUC1400005477.mm.1	2,19	0,033404	0,522793		
TC1400000112.mm.1	-1,03	Il3ra	JUC1400000542.mm.1	2,19	0,011087	0,425935		
TC1200000622.mm.1	-1,18	Lrrc9	JUC1200002401.mm.1	2,19	0,029611	0,510558		
TC1300002269.mm.1	-1,08	Mtrr	JUC1300007729.mm.1	2,19	0,029442	0,510013		
TC1300000887.mm.1	-1,05	Slc12a7	JUC1300003023.mm.1	2,19	0,011903	0,431543		
TC1300000908.mm.1	1,22	Pcsk1	JUC1300003123.mm.1	2,19	0,0406	0,542474		
TC1300000908.mm.1	1,22	Pcsk1	JUC1300003120.mm.1	2,04	0,014834	0,451201		
TC1700002467.mm.1	1,01		JUC1700012163.mm.1	2,19	0,028992	0,508842		
TC1700002467.mm.1	1,01		JUC1700012162.mm.1	-2,49	0,02133	0,481727		
TC1700002298.mm.1	1,06	Pot1b	JUC1700011450.mm.1	2,19	0,036578	0,531424		
TC1900000165.mm.1	1,56	Ganab	JUC1900001113.mm.1	2,19	0,026473	0,501252		
TC1900000165.mm.1	1,56	Ganab	JUC1900001112.mm.1	-2,17	0,002107	0,342919		
TC1500001211.mm.1	-1,05		JUC1500005515.mm.1	2,19	0,002819	0,352207		
TC0200001377.mm.1	1,09	Lrp4	JUC0200005405.mm.1	2,19	0,004824	0,371476		
TC0800001144.mm.1	-1,08	Setd6	JUC0800004844.mm.1	2,19	0,039118	0,538475		
TC0500002251.mm.1	1,03	4931431C16Rik	JUC0500011237.mm.1	2,19	0,015944	0,456831		
TC0500003549.mm.1	-1,12	Rbak	JUC0500017690.mm.1	2,19	0,005905	0,383835		
TC0600000236.mm.1	-1,15	Irf5	JUC0600000831.mm.1	2,19	0,023304	0,49004		
TC1000003018.mm.1	-1,14	Helb	JUC1000012355.mm.1	2,19	0,006287	0,388916		
TC1000003018.mm.1	-1,14	Helb	JUC1000012353.mm.1	2,04	0,019297	0,473074		
TC0300001313.mm.1	-1,24	Cfi	JUC0300005541.mm.1	2,19	0,043899	0,550649		
TC1100001875.mm.1	1,74	Ttyh2	JUC1100009117.mm.1	2,19	0,02995	0,511499		
TC1100001875.mm.1	1,74	Ttyh2	PSR1100017532.mm.1	-2,07	0,004604	0,369794		
TC0500000138.mm.1	1,23	Magi2	JUC0500000647.mm.1	2,19	0,022234	0,485896		
TC0500000138.mm.1	1,23	Magi2	JUC0500000664.mm.1	-2,06	0,017487	0,463964		
TC0400002797.mm.1	-1,02	Kif12	JUC0400011972.mm.1	2,19	0,029755	0,511132		
TC0400002797.mm.1	-1,02	Kif12	JUC0400011970.mm.1	-2,4	0,015831	0,456083		
TC0Y00000173.mm.1	-1,42	Gm20885	JUC0Y00000645.mm.1	2,19	0,006603	0,39213		
TC0X00002660.mm.1	2,28	Arhgef9	JUC0X00008444.mm.1	2,18	0,000398	0,29748		
TC0X00002660.mm.1	2,28	Arhgef9	JUC0X00008454.mm.1	2,11	0,034607	0,52602		
TC0X00002660.mm.1	2,28	Arhgef9	PSR0X00016640.mm.1	-2,04	0,009525	0,416602	Intron Retention	0,3
TC0X00002660.mm.1	2,28	Arhgef9	PSR0X00016644.mm.1	-2,05	0,016606	0,459802	Intron Retention	0,3
TC0X00002660.mm.1	2,28	Arhgef9	PSR0X00016662.mm.1	-2,05	0,04764	0,558743	Alternative 5' Donc	0,05
TC0X00002660.mm.1	2,28	Arhgef9	PSR0X00016670.mm.1	-2,13	0,040572	0,542455	Cassette Exon	0,14
TC0X00002660.mm.1	2,28	Arhgef9	PSR0X00016639.mm.1	-2,37	0,004409	0,366753	Intron Retention	0,3
TC0X00002660.mm.1	2,28	Arhgef9	PSR0X00016665.mm.1	-2,37	0,012534	0,43545	Alternative 5' Donc	0,11
TC0X00002660.mm.1	2,28	Arhgef9	JUC0X00008468.mm.1	-2,37	0,009777	0,417839		

TC0X00002660.mm.1	2,28	Arhgef9	JUC0X00008460.mm.1	-2,57	0,000783	0,309887		
TC0X00002660.mm.1	2,28	Arhgef9	PSR0X00016645.mm.1	-2,68	0,038759	0,537385	Intron Retention	0,3
TC0X00002660.mm.1	2,28	Arhgef9	PSR0X00016669.mm.1	-2,85	0,015102	0,452453	Cassette Exon	0,24
TC0X00002660.mm.1	2,28	Arhgef9	JUC0X00008456.mm.1	-2,88	0,031821	0,517751		
TC0X00002660.mm.1	2,28	Arhgef9	PSR0X00016666.mm.1	-3,13	0,016857	0,460797	Cassette Exon	0,18
TC0X00002660.mm.1	2,28	Arhgef9	PSR0X00016643.mm.1	-3,31	0,0264	0,50115	Intron Retention	0,3
TC0X00002660.mm.1	2,28	Arhgef9	JUC0X00008463.mm.1	-3,5	0,001559	0,331143		
TC0300001242.mm.1	2,93	Abca4	JUC0300005268.mm.1	2,18	0,013171	0,440567		
TC0300001242.mm.1	2,93	Abca4	PSR0300010061.mm.1	2,08	0,012851	0,437778	Cassette Exon	0,11
TC0300001242.mm.1	2,93	Abca4	PSR0300010069.mm.1	-2,25	0,015188	0,452699	Cassette Exon	0,28
TC0300001242.mm.1	2,93	Abca4	PSR0300010063.mm.1	-2,38	0,006244	0,388361	Cassette Exon	0,28
TC0300001242.mm.1	2,93	Abca4	PSR0300010062.mm.1	-2,47	0,03707	0,532747	Cassette Exon	0,29
TC0300001242.mm.1	2,93	Abca4	PSR0300010120.mm.1	-2,65	0,019469	0,474209	Alternative 5' Donc	0,12
TC0300001242.mm.1	2,93	Abca4	JUC0300005285.mm.1	-2,69	0,001627	0,332831		
TC0300001242.mm.1	2,93	Abca4	JUC0300005258.mm.1	-2,98	0,006807	0,393762		
TC0300001242.mm.1	2,93	Abca4	JUC0300005284.mm.1	-3,14	0,001523	0,330287		
TC0300001242.mm.1	2,93	Abca4	JUC0300005278.mm.1	-4,31	0,007506	0,401057		
TC0300001242.mm.1	2,93	Abca4	JUC0300005281.mm.1	-5,12	0,005286	0,378059		
TC0300001242.mm.1	2,93	Abca4	JUC0300005282.mm.1	-6,43	0,000438	0,297771		
TC0300001242.mm.1	2,93	Abca4	JUC0300005286.mm.1	-6,88	0,00731	0,398847		
TC0300001242.mm.1	2,93	Abca4	JUC0300005280.mm.1	-8,12	0,000938	0,313363		
TC0400003721.mm.1	1,42	Hmgn2; Gm7931; Gm9525	PSR0400030528.mm.1	2,18	0,026129	0,499897	Intron Retention	0,29
TC0400003721.mm.1	1,42	Hmgn2; Gm7931; Gm9525	JUC0400015882.mm.1	-2,54	0,024898	0,495661		
TC0800002580.mm.1	1,4	Ccdc130	PSR0800019689.mm.1	2,18	0,042836	0,548097	Alternative 3' Acce	0,19
TC0800002580.mm.1	1,4	Ccdc130	PSR0800019685.mm.1	-2,53	0,020251	0,477245	Alternative 5' Donc	0,25
TC0200004221.mm.1	1,05	Pdhx	PSR0200035432.mm.1	2,18	0,034217	0,525138	Alternative 5' Donc	0,19
TC0200004221.mm.1	1,05	Pdhx	JUC0200018108.mm.1	2,18	0,033468	0,522837		
TC0100003314.mm.1	-1,28		PSR0100027065.mm.1	2,18	0,014939	0,451968	Alternative 5' Donc	0,19
TC1700001010.mm.1	-1,17	Safb	PSR1700009683.mm.1	2,18	0,002112	0,342919	Intron Retention	0,18
TC1700001010.mm.1	-1,17	Safb	PSR1700009685.mm.1	2,18	0,024288	0,49362	Cassette Exon	0,11
TC0X00000063.mm.1	-1,1	Gpkow	PSR0X00000337.mm.1	2,18	0,022056	0,485327	Alternative 5' Donc	0,17
TC0200003002.mm.1	1,31	Gm13269	PSR0200024805.mm.1	2,18	0,003886	0,359225	Mutually Exclusive	0,15
TC0200003002.mm.1	1,31	Gm13269	PSR0200024804.mm.1	-2,11	0,011337	0,427345	Mutually Exclusive	0,15
TC1100000258.mm.1	-1,45	4933430M04Rik; Gm1206	PSR1100002216.mm.1	2,18	0,012733	0,436679	Cassette Exon	0,15
TC1000002806.mm.1	1,55	Mettl25	PSR1000021308.mm.1	2,18	0,014819	0,451071	Cassette Exon	0,13
TC1900000356.mm.1	-1,06	Trpm3	JUC1900001809.mm.1	2,18	0,035576	0,528808		
TC1900000356.mm.1	-1,06	Trpm3	PSR1900003486.mm.1	2,09	0,035699	0,529027	Cassette Exon	0,13
TC0300002934.mm.1	1,76	Lrit3	PSR0300023206.mm.1	2,18	0,030117	0,511997	Cassette Exon	0,12
TC0X00002189.mm.1	-1,36	Rap2c	PSR0X00013993.mm.1	2,18	0,00511	0,376191		
TC0X00002189.mm.1	-1,36	Rap2c	PSR0X00013996.mm.1	2,14	0,005043	0,374724	Cassette Exon	0,12
TC0400000698.mm.1	-1,61	Slc31a1	PSR0400004899.mm.1	2,18	0,049382	0,562882	Intron Retention	0,11
TC0400003689.mm.1	-1,79		PSR0400030247.mm.1	2,18	0,022726	0,48778	Cassette Exon	0,11
TC1700001013.mm.1	-1,16	Catsperd	PSR1700009733.mm.1	2,18	0,009769	0,417839	Cassette Exon	0,11
TC0500002254.mm.1	1,47	Sorcs2	PSR0500020717.mm.1	2,18	0,014654	0,450098	Cassette Exon	0,1
TC0500002254.mm.1	1,47	Sorcs2	JUC0500011277.mm.1	-2,21	0,024414	0,493825		
TC0900000922.mm.1	1,02	Bnip2	PSR0900007114.mm.1	2,18	0,021847	0,484607	Alternative 5' Donc	0,1
TC1200000496.mm.1	1,39	Gm17529	PSR1200003445.mm.1	2,18	0,021263	0,481565	Cassette Exon	0,1
TC1400002346.mm.1	1,13	Dmtn	JUC1400010099.mm.1	2,18	0,038743	0,537362		

TC1200000725.mm.1	-1,19		JUC1200002941.mm.1	2,18	0,039238	0,538875		
TC1200000528.mm.1	1,09	Fancm	JUC1200002058.mm.1	2,18	0,011375	0,427487		
TC0200003867.mm.1	-1,27	Gm13710	JUC0200017234.mm.1	2,18	0,038442	0,536405		
TC1300000938.mm.1	-1,08	Fam172a	JUC1300003248.mm.1	2,18	0,041258	0,544166		
TC1300000724.mm.1	-1,63	Naa35	JUC1300002485.mm.1	2,18	0,048182	0,560082		
TC1700002324.mm.1	-1,04	Ptprs	JUC1700011613.mm.1	2,18	0,012818	0,437471		
TC1700001384.mm.1	1,82	Tmem181b-ps; Tmem181a	JUC1700006794.mm.1	2,18	0,009795	0,417839		
TC1700000949.mm.1	-1,12	Kif6	JUC1700004887.mm.1	2,18	0,024264	0,4935		
TC1700001075.mm.1	1,03	Ppp4r1	JUC1700005463.mm.1	2,18	0,031255	0,516001		
TC0100001680.mm.1	1,6	Desi2	JUC0100007906.mm.1	2,18	0,047646	0,558744		
TC1900000582.mm.1	-1,04	Zfp518a; Mir8092; mmu-n	JUC1900002833.mm.1	2,18	0,025152	0,496542		
TC0200005480.mm.1	-1,21	Capn3	JUC0200006828.mm.1	2,18	0,009166	0,413447		
TC1700000692.mm.1	-1,01	Cchcr1	JUC1700003892.mm.1	2,18	0,023889	0,492304		
TC1700000663.mm.1	-1,15	Ddah2	JUC1700003692.mm.1	2,18	0,001387	0,325997		
TC1700000663.mm.1	-1,15	Ddah2	JUC1700003691.mm.1	2,05	0,015644	0,455298		
TC1600000666.mm.1	-1,33	Cd200r3	JUC1600003046.mm.1	2,18	0,038528	0,536634		
TC1600000666.mm.1	-1,33	Cd200r3	JUC1600003052.mm.1	-4,01	0,020197	0,477145		
TC1600001390.mm.1	-1,96	Rfc4	JUC1600006028.mm.1	2,18	0,004811	0,371408		
TC0700004553.mm.1	2,11	Dusp8	JUC0700020242.mm.1	2,18	0,007341	0,399144		
TC0800000874.mm.1	-1,34	Zfp827	JUC0800003471.mm.1	2,18	0,000714	0,306015		
TC0900001153.mm.1	1,06	Rasgrf1	JUC0900004922.mm.1	2,18	0,025839	0,498615		
TC0900000835.mm.1	-1,05	Clpx	JUC0900003448.mm.1	2,18	0,01392	0,446135		
TC1100001133.mm.1	-1,09	Myo18a	JUC1100005425.mm.1	2,18	0,030673	0,51384		
TC1000002823.mm.1	-1,14	Otogl	JUC1000011791.mm.1	2,18	0,020295	0,477245		
TC1000002823.mm.1	-1,14	Otogl	JUC1000011822.mm.1	-2,74	0,042348	0,547408		
TC1000002233.mm.1	-1,27	Cdh23	JUC1000008543.mm.1	2,18	0,038617	0,536942		
TC1100003461.mm.1	-1,48		JUC1100016643.mm.1	2,18	0,021869	0,484645		
TC0300002659.mm.1	-1,25	Mov10	JUC0300010785.mm.1	2,18	0,034268	0,5253		
TC1100001448.mm.1	-1,57	Col1a1	JUC1100007040.mm.1	2,18	0,030462	0,513244		
TC1100001451.mm.1	1,14	Ppp1r9b	JUC1100007066.mm.1	2,18	0,036489	0,531207		
TC0900003299.mm.1	1,05	Zdhhc3	JUC0900015640.mm.1	2,18	0,039743	0,540494		
TC0900003299.mm.1	1,05	Zdhhc3	JUC0900015646.mm.1	2,11	0,031977	0,518183		
TC0X00002078.mm.1	-1,23	4930525M21Rik	JUC0X00006732.mm.1	2,18	0,043532	0,549995		
TC1100003882.mm.1	1,81	Nsf	JUC1100018986.mm.1	2,17	0,040993	0,543577		
TC1100003882.mm.1	1,81	Nsf	PSR1100036328.mm.1	-2,18	0,029715	0,511008	Alternative 3' Acce	0,22
TC1100003882.mm.1	1,81	Nsf	PSR1100036341.mm.1	-2,57	0,013374	0,442285	Cassette Exon	0,16
TC1100003882.mm.1	1,81	Nsf	JUC1100019006.mm.1	-2,76	0,049909	0,563808		
TC1100003882.mm.1	1,81	Nsf	PSR1100036310.mm.1	-2,82	0,020814	0,479514	Alternative 5' Donc	0,29
TC1100003882.mm.1	1,81	Nsf	PSR1100036311.mm.1	-2,9	0,026502	0,501298	Alternative 3' Acce	0,3
TC1100003882.mm.1	1,81	Nsf	JUC1100019005.mm.1	-2,95	0,01317	0,440567		
TC1100003882.mm.1	1,81	Nsf	PSR1100036344.mm.1	-3,4	0,040978	0,543537		
TC1100003882.mm.1	1,81	Nsf	PSR1100036343.mm.1	-3,41	0,013948	0,446234	Cassette Exon	0,3
TC1100003882.mm.1	1,81	Nsf	PSR1100036333.mm.1	-4,14	0,014641	0,450098	Intron Retention	0,75
TC1100003882.mm.1	1,81	Nsf	JUC1100019007.mm.1	-6,46	0,007558	0,401384		
TC0200001806.mm.1	1,82	Adal	JUC0200006961.mm.1	2,17	0,017598	0,464312		
TC0200001806.mm.1	1,82	Adal	JUC0200006970.mm.1	-2,05	0,046075	0,555658		
TC0200001806.mm.1	1,82	Adal	PSR0200013919.mm.1	-2,41	0,022218	0,485896	Alternative 3' Acce	0,15
TC0200001806.mm.1	1,82	Adal	PSR0200013939.mm.1	-3,16	0,006961	0,395722	Cassette Exon	0,35

TC0200001806.mm.1	1,82	Adal	JUC0200006971.mm.1	-4,37	0,011704	0,429969		
TC1300001043.mm.1	1,32	Homer1	JUC1300003480.mm.1	2,17	0,028599	0,507685		
TC1300001043.mm.1	1,32	Homer1	PSR1300006673.mm.1	-2,62	0,049215	0,562465	Alternative 5' Donc	0,26
TC1300001043.mm.1	1,32	Homer1	JUC1300003491.mm.1	-2,62	0,007944	0,403819		
TC1300001043.mm.1	1,32	Homer1	JUC1300003483.mm.1	-4,18	0,02937	0,509995		
TC1000000834.mm.1	-1,54	Cnn2	PSR1000006122.mm.1	2,17	0,04354	0,549995	Alternative 5' Donc	0,19
TC0900001037.mm.1	-2,34	Klhl31	PSR0900008122.mm.1	2,17	0,01365	0,444359	Alternative 3' Acce	0,18
TC1900001758.mm.1	1,19	5830428H23Rik	JUC1900008632.mm.1	2,17	0,011275	0,426793		
TC1900001758.mm.1	1,19	5830428H23Rik	PSR1900015537.mm.1	-2,06	0,007255	0,39826	Alternative 3' Acce	0,16
TC1900001758.mm.1	1,19	5830428H23Rik	PSR1900015515.mm.1	-2,46	0,009175	0,413547	Cassette Exon	0,15
TC1900000082.mm.1	-1,39	Ltbp3	PSR1900000972.mm.1	2,17	0,00398	0,360363	Cassette Exon	0,13
TC0300002973.mm.1	1,05	Gstcd	PSR0300023480.mm.1	2,17	0,003451	0,3554	Cassette Exon	0,12
TC0300002973.mm.1	1,05	Gstcd	JUC0300012260.mm.1	2,08	0,028603	0,507714		
TC0300002973.mm.1	1,05	Gstcd	PSR0300023464.mm.1	-2,06	0,042308	0,547224	Cassette Exon	0,12
TC0100003476.mm.1	1,25	Gm16701	PSR0100028481.mm.1	2,17	0,016366	0,458693	Cassette Exon	0,11
TC0300001260.mm.1	2,16	Tram1l1	PSR0300010292.mm.1	2,17	0,008486	0,406895	Cassette Exon	0,11
TC0700000002.mm.1	-2,12	Speer9-ps1	PSR0700000008.mm.1	2,17	0,023948	0,4926	Cassette Exon	0,09
TC1900001636.mm.1	-1,29	Col17a1	PSR1900014489.mm.1	2,17	0,013492	0,443193	Cassette Exon	0,09
TC1900001636.mm.1	-1,29	Col17a1	JUC1900008013.mm.1	-2,17	0,014301	0,448073		
TC1400002309.mm.1	-1,7		JUC1400009841.mm.1	2,17	0,0292	0,509482		
TC1100004233.mm.1	-1,12	Fasn	JUC1100021271.mm.1	2,17	0,049217	0,562473		
TC1300002250.mm.1	-1,31	Zfp874b	JUC1300007653.mm.1	2,17	0,012909	0,438243		
TC1700002331.mm.1	1,72	Safb2	JUC1700011648.mm.1	2,17	0,018307	0,468049		
TC1700002331.mm.1	1,72	Safb2	JUC1700011635.mm.1	-2,27	0,039596	0,539903		
TC1800000240.mm.1	-1,34	Sap130	JUC1800001005.mm.1	2,17	0,047906	0,55942		
TC1800000564.mm.1	-1,34	Gramd3	JUC1800002234.mm.1	2,17	0,029731	0,511096		
TC1700002071.mm.1	-1,81	H2-M2	JUC1700010417.mm.1	2,17	0,007468	0,400381		
TC1900000683.mm.1	1,03	Nolc1	JUC1900003238.mm.1	2,17	0,030993	0,514779		
TC1500000736.mm.1	-1,08	Xrcc6	JUC1500003242.mm.1	2,17	0,049551	0,563325		
TC0800002235.mm.1	-1,17	Neil3	JUC0800009227.mm.1	2,17	0,034507	0,525929		
TC0700004519.mm.1	-1,28	Sigirr	JUC0700019975.mm.1	2,17	0,016194	0,458125		
TC0700004328.mm.1	1,05	Zfp689	JUC0700019176.mm.1	2,17	0,018215	0,4676		
TC0800000948.mm.1	1,1	4930432K21Rik	JUC0800003761.mm.1	2,17	0,025986	0,499207		
TC0900001604.mm.1	-1,09	Entpd3	JUC0900007559.mm.1	2,17	0,000005	0,179072		
TC0800002831.mm.1	1,05	Acd	JUC0800011904.mm.1	2,17	0,034423	0,525828		
TC0600000758.mm.1	1,2	Smarcad1	JUC0600002848.mm.1	2,17	0,004765	0,371039		
TC0600000758.mm.1	1,2	Smarcad1	JUC0600002843.mm.1	-6,89	0,029462	0,510123		
TC0600001712.mm.1	1,18	Lyrm5	JUC0600007145.mm.1	2,17	0,021053	0,480262		
TC0600001639.mm.1	-1,73	Mgst1	PSR0600013062.mm.1	2,17	0,04081	0,543231		
TC1100000851.mm.1	-2,12	Myh3	JUC1100003504.mm.1	2,17	0,031622	0,517405		
TC0300002199.mm.1	-1,24	Etfdh	JUC0300008665.mm.1	2,17	0,049448	0,563157		
TC0X00000641.mm.1	-1,13		JUC0X00002002.mm.1	2,17	0,005677	0,382164		
TC0400002786.mm.1	-1,11	Fkbp15	JUC0400011888.mm.1	2,17	0,01358	0,443703		
TC0400002786.mm.1	-1,11	Fkbp15	JUC0400011866.mm.1	2,02	0,020734	0,479166		
TC0400002007.mm.1	-1	Dnajc11	JUC0400008578.mm.1	2,17	0,046732	0,556856		
TC1000000907.mm.1	1,79	Gm10778	JUC1000003928.mm.1	2,17	0,031406	0,516484		
TC1000001089.mm.1	1,64	Uhrf1bp1l	JUC1000004430.mm.1	2,17	0,038586	0,536862		
TC1000000647.mm.1	-1,07	Rtkn2	JUC1000002402.mm.1	2,17	0,005596	0,381115		

TC0400002434.mm.1	-1,2	Ddx58	JUC0400010214.mm.1	2,17	0,04115	0,544		
TC0300001130.mm.1	5,57	Gnat2	JUC0300004740.mm.1	2,16	0,013091	0,439759		
TC0300001130.mm.1	5,57	Gnat2	PSR0300009186.mm.1	2,14	0,01116	0,426295	Cassette Exon	0,15
TC0300001130.mm.1	5,57	Gnat2	PSR0300009176.mm.1	-2,22	0,003057	0,353892	Cassette Exon	0,22
TC0300001130.mm.1	5,57	Gnat2	PSR0300009181.mm.1	-2,39	0,031589	0,51718	Alternative 5' Donc	0,18
TC0300001130.mm.1	5,57	Gnat2	PSR0300009177.mm.1	-3,49	0,013325	0,441836	Cassette Exon	0,16
TC0300001130.mm.1	5,57	Gnat2	JUC0300004745.mm.1	-3,95	0,006846	0,393893		
TC0300001130.mm.1	5,57	Gnat2	PSR0300009174.mm.1	-4,14	0,002388	0,348564	Alternative 3' Acce	0,31
TC0300001130.mm.1	5,57	Gnat2	JUC0300004739.mm.1	-4,5	0,012088	0,43255		
TC0300001130.mm.1	5,57	Gnat2	PSR0300009185.mm.1	-4,6	0,000574	0,304044	Cassette Exon	0,36
TC0300001130.mm.1	5,57	Gnat2	PSR0300009180.mm.1	-4,91	0,000996	0,316361	Alternative 5' Donc	0,48
TC0300001130.mm.1	5,57	Gnat2	JUC0300004737.mm.1	-6,84	0,001069	0,31648		
TC0300001130.mm.1	5,57	Gnat2	JUC0300004746.mm.1	-7,01	0,007314	0,398848		
TC0300001130.mm.1	5,57	Gnat2	PSR0300009168.mm.1	-7,55	0,002758	0,350151	Alternative 3' Acce	0,39
TC0300001271.mm.1	2,51	Camk2d	PSR0300010359.mm.1	2,16	0,008227	0,405976		
TC0300001271.mm.1	2,51	Camk2d	PSR0300010344.mm.1	-2,07	0,018969	0,471398	Alternative 3' Acce	0,18
TC0300001271.mm.1	2,51	Camk2d	PSR0300010348.mm.1	-2,15	0,009365	0,41517	Alternative 3' Acce	0,18
TC0300001271.mm.1	2,51	Camk2d	JUC0300005433.mm.1	-2,33	0,038416	0,536405		
TC0300001271.mm.1	2,51	Camk2d	PSR0300010382.mm.1	-2,55	0,015119	0,452652	Cassette Exon	0,09
TC0300001271.mm.1	2,51	Camk2d	PSR0300010339.mm.1	-2,62	0,04189	0,545772	Alternative 3' Acce	0,18
TC0300001271.mm.1	2,51	Camk2d	PSR0300010363.mm.1	-2,8	0,042012	0,546109	Alternative 3' Acce	0,24
TC0300001271.mm.1	2,51	Camk2d	PSR0300010364.mm.1	-2,82	0,011995	0,432286	Alternative 3' Acce	0,24
TC0300001271.mm.1	2,51	Camk2d	JUC0300005432.mm.1	-2,91	0,005561	0,380776		
TC0300001271.mm.1	2,51	Camk2d	PSR0300010397.mm.1	-2,93	0,012016	0,432286	Alternative 5' Donc	0,26
TC0300001271.mm.1	2,51	Camk2d	PSR0300010388.mm.1	-3,55	0,003193	0,354243	Alternative 5' Donc	0,38
TC0300001271.mm.1	2,51	Camk2d	PSR0300010368.mm.1	-3,73	0,015161	0,452699	Alternative 3' Acce	0,43
TC0300001271.mm.1	2,51	Camk2d	JUC0300005439.mm.1	-3,81	0,012043	0,432309		
TC0300001271.mm.1	2,51	Camk2d	JUC0300005429.mm.1	-3,94	0,002205	0,345178		
TC0300001271.mm.1	2,51	Camk2d	PSR0300010367.mm.1	-4,33	0,028384	0,50695	Intron Retention	0,36
TC0300001271.mm.1	2,51	Camk2d	JUC0300005440.mm.1	-4,37	0,004726	0,370888		
TC0300001271.mm.1	2,51	Camk2d	JUC0300005426.mm.1	-4,55	0,002096	0,342919		
TC1600001993.mm.1	4,12	Grik1	JUC1600007998.mm.1	2,16	0,032143	0,518586		
TC1600001993.mm.1	4,12	Grik1	PSR1600015471.mm.1	-2,08	0,020979	0,480052	Alternative 3' Acce	0,11
TC1600001993.mm.1	4,12	Grik1	PSR1600015469.mm.1	-2,11	0,012859	0,437778		
TC1600001993.mm.1	4,12	Grik1	JUC1600008003.mm.1	-2,56	0,001452	0,328414		
TC1600001993.mm.1	4,12	Grik1	PSR1600015475.mm.1	-2,67	0,016245	0,458312	Alternative 3' Acce	0,25
TC1600001993.mm.1	4,12	Grik1	PSR1600015465.mm.1	-2,94	0,022045	0,485297	Cassette Exon	0,26
TC1600001993.mm.1	4,12	Grik1	PSR1600015466.mm.1	-3,49	0,015031	0,452111	Cassette Exon	0,28
TC1600001993.mm.1	4,12	Grik1	PSR1600015478.mm.1	-3,63	0,009392	0,415597	Alternative 3' Acce	0,4
TC1600001993.mm.1	4,12	Grik1	JUC1600008002.mm.1	-8,55	0,002409	0,348653		
TC1800001150.mm.1	1,25	Nme5	PSR1800008398.mm.1	2,16	0,012346	0,43457	Cassette Exon	0,09
TC1800001150.mm.1	1,25	Nme5	PSR1800008394.mm.1	-3,49	0,03437	0,525628	Alternative 3' Acce	0,4
TC1500001171.mm.1	3,01	Egflam	PSR1500009396.mm.1	2,16	0,006992	0,395722	Cassette Exon	0,13
TC1500001171.mm.1	3,01	Egflam	JUC1500005289.mm.1	2,04	0,012119	0,432781		
TC1500001171.mm.1	3,01	Egflam	PSR1500009371.mm.1	-2,07	0,008715	0,409266	Cassette Exon	0,19
TC1500001171.mm.1	3,01	Egflam	JUC1500005280.mm.1	-2,18	0,031202	0,515739		
TC1500001171.mm.1	3,01	Egflam	PSR1500009401.mm.1	-2,35	0,003727	0,357586	Alternative 5' Donc	0,29
TC1500001171.mm.1	3,01	Egflam	PSR1500009378.mm.1	-2,54	0,003228	0,354243	Alternative 3' Acce	0,25

TC1500001171.mm.1	3,01	Egflam	PSR1500009365.mm.1	-2,56	0,008115	0,405267	Alternative 3' Acce	0,25
TC1500001171.mm.1	3,01	Egflam	PSR1500009403.mm.1	-2,73	0,031789	0,517596	Alternative 5' Donc	0,29
TC1500001171.mm.1	3,01	Egflam	JUC1500005274.mm.1	-2,87	0,000649	0,304044		
TC1500001171.mm.1	3,01	Egflam	PSR1500009390.mm.1	-3,08	0,018802	0,470831	Cassette Exon	0,28
TC1500001171.mm.1	3,01	Egflam	PSR1500009375.mm.1	-3,27	0,006965	0,395722	Alternative 5' Donc	0,37
TC1500001171.mm.1	3,01	Egflam	PSR1500009404.mm.1	-3,4	0,022338	0,486348	Alternative 5' Donc	0,29
TC1500001171.mm.1	3,01	Egflam	JUC1500005285.mm.1	-3,51	0,00179	0,336311		
TC1000002420.mm.1	2,01	Adarb1	JUC1000009645.mm.1	2,16	0,004633	0,36999		
TC1000002420.mm.1	2,01	Adarb1	PSR1000017575.mm.1	-3,13	0,023229	0,490032	Cassette Exon	0,29
TC1000002420.mm.1	2,01	Adarb1	PSR1000017577.mm.1	-3,16	0,015263	0,452932	Cassette Exon	0,29
TC1000002420.mm.1	2,01	Adarb1	JUC1000009656.mm.1	-5,04	0,023051	0,48937		
TC1800000665.mm.1	1,46	Nedd4l	JUC1800002750.mm.1	2,16	0,034555	0,525975		
TC1800000665.mm.1	1,46	Nedd4l	JUC1800002744.mm.1	-2,04	0,020471	0,478018		
TC1800000665.mm.1	1,46	Nedd4l	PSR1800004894.mm.1	-2,09	0,021399	0,482036	Cassette Exon	0,28
TC1800000665.mm.1	1,46	Nedd4l	JUC1800002727.mm.1	-2,12	0,044499	0,551741		
TC1800000665.mm.1	1,46	Nedd4l	PSR1800004879.mm.1	-2,73	0,040031	0,541304	Cassette Exon	0,15
TC1800000665.mm.1	1,46	Nedd4l	JUC1800002725.mm.1	-3,9	0,009995	0,418456		
TC1400002497.mm.1	-1,34	Gm10845	PSR1400019195.mm.1	2,16	0,00586	0,383791	Alternative 3' Acce	0,19
TC0900000593.mm.1	4,13	Drd2	PSR0900004207.mm.1	2,16	0,006487	0,390655	Mutually Exclusive	0,18
TC0900000593.mm.1	4,13	Drd2	PSR0900004208.mm.1	-2,27	0,001462	0,328794	Mutually Exclusive	0,18
TC0900000593.mm.1	4,13	Drd2	JUC0900002200.mm.1	-3,46	0,000134	0,270905		
TC1900000547.mm.1	-1,63	Lgi1	PSR1900004896.mm.1	2,16	0,033577	0,52319	Alternative 3' Acce	0,17
TC0600002142.mm.1	-1,12	Clec5a	PSR0600016914.mm.1	2,16	0,018964	0,471398	Alternative 5' Donc	0,16
TC0700002758.mm.1	-1,54	Hcst	PSR0700024448.mm.1	2,16	0,038027	0,535494	Alternative 3' Acce	0,16
TC1500000356.mm.1	1,33	A930017M01Rik; 231006	PSR1500002538.mm.1	2,16	0,038082	0,535633	Alternative 5' Donc	0,15
TC1800000457.mm.1	1,23	Dcp2	PSR1800003356.mm.1	2,16	0,024619	0,494589	Alternative 5' Donc	0,15
TC1700001750.mm.1	1,44	Tcp11	PSR1700016113.mm.1	2,16	0,004136	0,3623	Cassette Exon	0,11
TC1700001750.mm.1	1,44	Tcp11	PSR1700016105.mm.1	-2,52	0,023028	0,489236	Cassette Exon	0,13
TC0400000149.mm.1	-1,3	Mmp16	PSR0400000953.mm.1	2,16	0,036044	0,52965	Cassette Exon	0,12
TC1100003093.mm.1	-2,76	Alox15	PSR1100028571.mm.1	2,16	0,027355	0,50383	Cassette Exon	0,12
TC1200000161.mm.1	-1,13	Gm5953	PSR1200001332.mm.1	2,16	0,004892	0,372195	Cassette Exon	0,12
TC0100003254.mm.1	-1,15	Rnf2	JUC0100015069.mm.1	2,16	0,021708	0,484104		
TC0100003254.mm.1	-1,15	Rnf2	PSR0100026433.mm.1	2,03	0,008343	0,406435	Cassette Exon	0,11
TC0200002293.mm.1	-1,32	Rem1	PSR0200017920.mm.1	2,16	0,003978	0,360326	Cassette Exon	0,11
TC1600000381.mm.1	-2,18		PSR1600003633.mm.1	2,16	0,003148	0,353892	Cassette Exon	0,11
TC1600000381.mm.1	-2,18		PSR1600003634.mm.1	-2,16	0,003148	0,353892	Cassette Exon	0,11
TC1000000847.mm.1	1,16	Ndufs7	PSR1000006412.mm.1	2,16	0,037727	0,534622	Alternative 5' Donc	0,1
TC1400002840.mm.1	-1,04	Gm8206; Gm3642	PSR1400010251.mm.1	2,16	0,016511	0,459491	Cassette Exon	0,1
TC1600000322.mm.1	-1,02	Dnajb11	PSR1600003171.mm.1	2,16	0,001733	0,335996	Cassette Exon	0,1
TC1400002427.mm.1	1,18	Cog3	JUC1400010378.mm.1	2,16	0,031077	0,515159		
TC1200000751.mm.1	1,12	Smoc1	JUC1200003025.mm.1	2,16	0,028836	0,508414		
TC0200005287.mm.1	-1,13	Ctcfl	JUC0200023230.mm.1	2,16	0,001413	0,326575		
TC0200005287.mm.1	-1,13	Ctcfl	JUC0200023229.mm.1	-2,67	0,005201	0,377042		
TC1100003863.mm.1	1,15	Plcd3	JUC1100018857.mm.1	2,16	0,007921	0,403819		
TC1300001139.mm.1	-1,13	Taf9; Ak6; Gm12372	JUC1300003773.mm.1	2,16	0,013443	0,442693		
TC1300001447.mm.1	-2,07	Actn2	JUC1300004835.mm.1	2,16	0,029091	0,509063		
TC1200002388.mm.1	1,07	1700001K19Rik	JUC1200009118.mm.1	2,16	0,030255	0,512521		
TC1200002388.mm.1	1,07	1700001K19Rik	JUC1200009116.mm.1	-3,21	0,007959	0,403853		

TC1300000507.mm.1	-1,1		JUC1300001585.mm.1	2,16	0,010869	0,424174		
TC1300000571.mm.1	-1,03	Nol8	JUC1300001823.mm.1	2,16	0,002811	0,352207		
TC1800000593.mm.1	-1,04	Adamts19	JUC1800002365.mm.1	2,16	0,007263	0,39826		
TC1800000593.mm.1	-1,04	Adamts19	JUC1800002369.mm.1	-2,35	0,034512	0,525929		
TC1700001792.mm.1	-1,34		JUC1700008898.mm.1	2,16	0,042273	0,547092		
TC0100000792.mm.1	1,2	Cops7b	JUC0100003791.mm.1	2,16	0,043144	0,548664		
TC0100000792.mm.1	1,2	Cops7b	JUC0100003794.mm.1	-2,19	0,047828	0,559283		
TC1900000760.mm.1	1,25	Rbm20	JUC1900003651.mm.1	2,16	0,00377	0,357586		
TC1400002701.mm.1	-1,14	4930505G20Rik	JUC1400011042.mm.1	2,16	0,031963	0,518173		
TC0200000897.mm.1	-1,32	Tanc1	JUC0200003680.mm.1	2,16	0,012443	0,43528		
TC0200000897.mm.1	-1,32	Tanc1	JUC0200003679.mm.1	2,02	0,037832	0,534803		
TC0900000994.mm.1	-1,17	RP23-339G10.1	JUC0900004175.mm.1	2,16	0,033353	0,522553		
TC0600003382.mm.1	-1,15	Gm766	JUC0600013972.mm.1	2,16	0,011573	0,429058		
TC0600002918.mm.1	-1,26	Tada3	JUC0600011625.mm.1	2,16	0,036848	0,532256		
TC0700001877.mm.1	-1,12	Itgad	JUC0700008571.mm.1	2,16	0,02655	0,501435		
TC1100001021.mm.1	-1,13	Spata22; Olfr20	JUC1100004912.mm.1	2,16	0,002819	0,352207		
TC1100000922.mm.1	-1,04	2010012P19Rik; Tnfsf13os	JUC1100004116.mm.1	2,16	0,036993	0,532502		
TC1000002735.mm.1	1,26		PSR1000020990.mm.1	2,16	0,031297	0,516173		
TC1000002735.mm.1	1,26		JUC1000011536.mm.1	-2,96	0,037347	0,533573		
TC0300002475.mm.1	-1,01	Setdb1	JUC0300009982.mm.1	2,16	0,031651	0,517405		
TC1100001378.mm.1	-1,38	C030037D09Rik	JUC1100006791.mm.1	2,16	0,001648	0,333988		
TC0400003271.mm.1	-1,37	Cdkn2c	JUC0400013567.mm.1	2,16	0,020033	0,476453		
TC0900002917.mm.1	-1,08	Gm6432	JUC0900013420.mm.1	2,16	0,019924	0,475902		
TC0900002920.mm.1	-1,24	Esyt3	JUC0900013443.mm.1	2,16	0,010012	0,418602		
TC1000000126.mm.1	1,08	Reps1	JUC1000000424.mm.1	2,16	0,026256	0,500359		
TC1000000126.mm.1	1,08	Reps1	JUC1000000446.mm.1	-2,11	0,018012	0,466939		
TC0100002081.mm.1	5,48	Bai3	JUC0100009654.mm.1	2,15	0,010486	0,421937		
TC0100002081.mm.1	5,48	Bai3	PSR0100016984.mm.1	-2,05	0,011392	0,427549	Cassette Exon	0,24
TC0100002081.mm.1	5,48	Bai3	PSR0100016961.mm.1	-2,23	0,002494	0,349135	Cassette Exon	0,2
TC0100002081.mm.1	5,48	Bai3	JUC0100009650.mm.1	-2,44	0,030028	0,511744		
TC0100002081.mm.1	5,48	Bai3	PSR0100016985.mm.1	-2,45	0,015613	0,455082	Cassette Exon	0,27
TC0100002081.mm.1	5,48	Bai3	PSR0100016963.mm.1	-2,64	0,001478	0,329081	Cassette Exon	0,34
TC0100002081.mm.1	5,48	Bai3	JUC0100009665.mm.1	-2,7	0,033691	0,523549		
TC0100002081.mm.1	5,48	Bai3	PSR0100016982.mm.1	-2,79	0,003915	0,359719	Cassette Exon	0,26
TC0100002081.mm.1	5,48	Bai3	JUC0100009649.mm.1	-2,85	0,002929	0,352289		
TC0100002081.mm.1	5,48	Bai3	PSR0100016990.mm.1	-2,94	0,000316	0,288663	Cassette Exon	0,15
TC0100002081.mm.1	5,48	Bai3	PSR0100016996.mm.1	-2,98	0,003033	0,353543	Cassette Exon	0,37
TC0100002081.mm.1	5,48	Bai3	PSR0100016980.mm.1	-3,2	0,000154	0,272178	Cassette Exon	0,25
TC0100002081.mm.1	5,48	Bai3	PSR0100016988.mm.1	-3,32	0,005396	0,378916	Cassette Exon	0,21
TC0100002081.mm.1	5,48	Bai3	PSR0100016972.mm.1	-3,65	0,005475	0,379939	Alternative 5' Donc	0,48
TC0100002081.mm.1	5,48	Bai3	PSR0100016989.mm.1	-3,72	0,000239	0,28803	Cassette Exon	0,22
TC0100002081.mm.1	5,48	Bai3	JUC0100009651.mm.1	-3,82	0,002471	0,349135		
TC0100002081.mm.1	5,48	Bai3	PSR0100016995.mm.1	-4,19	0,000444	0,297771	Cassette Exon	0,41
TC0100002081.mm.1	5,48	Bai3	PSR0100016987.mm.1	-4,25	0,006852	0,393893	Cassette Exon	0,31
TC0100002081.mm.1	5,48	Bai3	PSR0100016983.mm.1	-4,35	0,002529	0,349501	Cassette Exon	0,36
TC0100002081.mm.1	5,48	Bai3	PSR0100016968.mm.1	-4,54	0,002478	0,349135	Alternative 3' Acce	0,48
TC0100002081.mm.1	5,48	Bai3	JUC0100009642.mm.1	-4,99	0,010892	0,424403		
TC0100002081.mm.1	5,48	Bai3	PSR0100016991.mm.1	-5,93	0,000352	0,290838	Cassette Exon	0,28

TC0100002081.mm.1	5,48	Bai3	PSR0100016973.mm.1	-6,41	0,001482	0,329081	Alternative 5' Donc	0,48
TC0100002081.mm.1	5,48	Bai3	PSR0100016953.mm.1	-6,71	0,001791	0,336311	Cassette Exon	0,41
TC0100002081.mm.1	5,48	Bai3	PSR0100016967.mm.1	-7,23	0,001516	0,330268	Cassette Exon	0,24
TC0100002081.mm.1	5,48	Bai3	PSR0100016976.mm.1	-7,55	0,003378	0,354303	Alternative 5' Donc	0,48
TC0100002081.mm.1	5,48	Bai3	PSR0100016975.mm.1	-7,76	0,001428	0,327447	Alternative 5' Donc	0,48
TC0100002081.mm.1	5,48	Bai3	PSR0100016956.mm.1	-9,64	0,000335	0,28927	Alternative 3' Acce	0,46
TC0100002081.mm.1	5,48	Bai3	JUC0100009667.mm.1	-10,83	0,001282	0,322289		
TC0100002081.mm.1	5,48	Bai3	JUC0100009676.mm.1	-13,91	0,001732	0,335996		
TC0800000619.mm.1	3,45	Glra3	PSR0800004161.mm.1	2,15	0,010606	0,422777	Cassette Exon	0,05
TC0800000619.mm.1	3,45	Glra3	PSR0800004173.mm.1	-2,07	0,012613	0,435987	Cassette Exon	0,17
TC0800000619.mm.1	3,45	Glra3	JUC0800002224.mm.1	-2,33	0,017918	0,466466		
TC0800000619.mm.1	3,45	Glra3	PSR0800004159.mm.1	-2,61	0,011446	0,427766	Alternative 3' Acce	0,37
TC0800000619.mm.1	3,45	Glra3	JUC0800002233.mm.1	-2,96	0,026349	0,50085		
TC0800000619.mm.1	3,45	Glra3	PSR0800004158.mm.1	-3,37	0,021379	0,482	Alternative 3' Acce	0,37
TC0800000619.mm.1	3,45	Glra3	JUC0800002225.mm.1	-5,61	0,004931	0,373093		
TC0800000619.mm.1	3,45	Glra3	PSR0800004160.mm.1	-6,86	0,000596	0,304044	Alternative 3' Acce	0,48
TC0X00000700.mm.1	16,51	Opn1mw	PSR0X00004617.mm.1	2,15	0,000347	0,290082	Cassette Exon	0,24
TC0X00000700.mm.1	16,51	Opn1mw	JUC0X00002338.mm.1	-2,6	0,049692	0,563531		
TC0X00000700.mm.1	16,51	Opn1mw	PSR0X00004611.mm.1	-3,06	0,005616	0,381191	Cassette Exon	0,2
TC0X00000700.mm.1	16,51	Opn1mw	JUC0X00002341.mm.1	-3,52	0,015996	0,45699		
TC0X00000700.mm.1	16,51	Opn1mw	PSR0X00004620.mm.1	-3,54	0,000861	0,311909	Alternative 5' Donc	0,33
TC0X00000700.mm.1	16,51	Opn1mw	JUC0X00002342.mm.1	-13,99	0,017403	0,463753		
TC0X00000700.mm.1	16,51	Opn1mw	PSR0X00004614.mm.1	-17,73	0,005034	0,374447	Cassette Exon	0,4
TC0600001664.mm.1	1,82	Pde3a	JUC0600006974.mm.1	2,15	0,007714	0,402552		
TC0600001664.mm.1	1,82	Pde3a	PSR0600013270.mm.1	-2,78	0,014434	0,448934	Alternative 3' Acce	0,25
TC0600001664.mm.1	1,82	Pde3a	JUC0600006976.mm.1	-3	0,032439	0,519525		
TC0200000301.mm.1	1,04	Commd3	PSR0200001328.mm.1	2,15	0,046712	0,556856	Alternative 3' Acce	0,19
TC0700000559.mm.1	1,06	Syne4	PSR0700004823.mm.1	2,15	0,007733	0,402774	Alternative 3' Acce	0,18
TC0700000559.mm.1	1,06	Syne4	PSR0700004846.mm.1	-2,9	0,002016	0,34123		
TC0700002996.mm.1	1,17	Ccdc155	PSR0700027002.mm.1	2,15	0,004309	0,364142	Alternative 3' Acce	0,18
TC1500001742.mm.1	1,36	Top1mt	PSR1500013126.mm.1	2,15	0,001118	0,317328	Alternative 3' Acce	0,18
TC1700002313.mm.1	-1,37	Plin4	PSR1700021594.mm.1	2,15	0,019551	0,474538	Alternative 5' Donc	0,18
TC0100003664.mm.1	-1,27	Psen2	PSR0100030130.mm.1	2,15	0,008173	0,405603	Cassette Exon	0,17
TC0600000004.mm.1	1,17	Ccdc132	PSR0600000026.mm.1	2,15	0,048605	0,561139	Cassette Exon	0,1
TC0600000004.mm.1	1,17	Ccdc132	PSR0600000054.mm.1	-2,25	0,047967	0,559552	Cassette Exon	0,17
TC1000000819.mm.1	-1,42	Misp	PSR1000005940.mm.1	2,15	0,022962	0,488776	Alternative 5' Donc	0,16
TC1000000819.mm.1	-1,42	Misp	JUC1000003230.mm.1	2,09	0,011651	0,429885		
TC1000000819.mm.1	-1,42	Misp	JUC1000003229.mm.1	2,08	0,001853	0,337479		
TC1000000819.mm.1	-1,42	Misp	PSR1000005941.mm.1	2,05	0,001799	0,336311	Cassette Exon	0,17
TC1100004123.mm.1	1,4	Cygb	PSR1100039077.mm.1	2,15	0,00219	0,344463	Cassette Exon	0,16
TC1500001353.mm.1	-1,22		PSR1500010435.mm.1	2,15	0,026789	0,502211	Intron Retention	0,15
TC0200005482.mm.1	-1,39	Hypk	PSR0200046672.mm.1	2,15	0,023228	0,490032	Alternative 3' Acce	0,14
TC0500002177.mm.1	-1,03	Zfp513	PSR0500019921.mm.1	2,15	0,026279	0,50053	Alternative 5' Donc	0,13
TC0X00002211.mm.1	-1,8	Gpc3	PSR0X00014114.mm.1	2,15	0,020848	0,479521	Alternative 5' Donc	0,13
TC1700000796.mm.1	-1,13	Olfr133	PSR1700007773.mm.1	2,15	0,00377	0,357586	Cassette Exon	0,12
TC0400003439.mm.1	-1,34	Kcnq4	PSR0400027928.mm.1	2,15	0,006636	0,392813	Cassette Exon	0,11
TC0500002805.mm.1	1,46	Antxr2	PSR0500024664.mm.1	2,15	0,043278	0,549286	Cassette Exon	0,11
TC1700000050.mm.1	-1,18	Tulp4; Gm2808	PSR1700000348.mm.1	2,15	0,047561	0,558707	Alternative 3' Acce	0,11

TC1700000050.mm.1	-1,18	Tulp4; Gm2808	JUC1700000195.mm.1	-2,8	0,007845	0,403342		
TC0100001522.mm.1	-1,21	Adcy10	PSR0100012535.mm.1	2,15	0,035872	0,529376	Cassette Exon	0,1
TC1500000901.mm.1	1,72	Cntn1	JUC1500004126.mm.1	2,15	0,021847	0,484607		
TC1500000901.mm.1	1,72	Cntn1	PSR1500007252.mm.1	-2,36	0,016188	0,45809	Cassette Exon	0,06
TC1500000901.mm.1	1,72	Cntn1	JUC1500004108.mm.1	-2,86	0,029163	0,509333		
TC0200003133.mm.1	1,22	Nelfb	JUC0200013018.mm.1	2,15	0,003316	0,354243		
TC1400002091.mm.1	-1,07	Psme2; Psme2b	JUC1400008697.mm.1	2,15	0,014075	0,446841		
TC1400002091.mm.1	-1,07	Psme2; Psme2b	JUC1400008700.mm.1	2,07	0,038159	0,535786		
TC1200000806.mm.1	1,05	Ptgr2	JUC1200003259.mm.1	2,15	0,010771	0,423955		
TC1300001033.mm.1	-1,31	Dhfr	JUC1300003442.mm.1	2,15	0,006932	0,395269		
TC0100002608.mm.1	-1,01	Dnpep	JUC0100012052.mm.1	2,15	0,027243	0,503642		
TC1700000896.mm.1	-1,14	Dnph1	JUC1700004577.mm.1	2,15	0,029801	0,511132		
TC1900001772.mm.1	-1,16	4430402I18Rik	JUC1900006484.mm.1	2,15	0,028122	0,506228		
TC0100000343.mm.1	-1,11	Col3a1	JUC0100001434.mm.1	2,15	0,032383	0,519221		
TC0100000343.mm.1	-1,11	Col3a1	JUC0100001432.mm.1	-2,53	0,007765	0,402774		
TC1800001521.mm.1	-1,28	Fam210a; Mir7219; mmu-	JUC1800006220.mm.1	2,15	0,023581	0,490995		
TC0100001947.mm.1	-1,04	Snhg6	JUC0100009053.mm.1	2,15	0,024108	0,49322		
TC0200000825.mm.1	-1,27	4930573O16Rik	JUC0200003462.mm.1	2,15	0,017316	0,463282		
TC1600000468.mm.1	1,02	Zfp148	JUC1600002304.mm.1	2,15	0,001528	0,330287		
TC1600001256.mm.1	-1,36	Spidr	JUC1600005285.mm.1	2,15	0,04148	0,544745		
TC0500003716.mm.1	1,07	Pms2	JUC0500008714.mm.1	2,15	0,007119	0,397044		
TC0800002463.mm.1	-1,51	Cherp	JUC0800010280.mm.1	2,15	0,016414	0,458966		
TC0700000218.mm.1	-1,19	Zfp541	JUC0700000736.mm.1	2,15	0,019411	0,473825		
TC0700001822.mm.1	1,26	Hirip3	PSR0700015598.mm.1	2,15	0,001711	0,335996		
TC0700001822.mm.1	1,26	Hirip3	JUC0700008176.mm.1	-2,9	0,049876	0,563773		
TC0700002277.mm.1	-1,79	Vmn2r39	JUC0700010935.mm.1	2,15	0,020594	0,478531		
TC0400000348.mm.1	-1,42		JUC0400001047.mm.1	2,15	0,013194	0,440783		
TC1000001244.mm.1	-1,11	Slc6a15	JUC1000005045.mm.1	2,15	0,046942	0,557545		
TC1000002519.mm.1	-1,05	Lmnb2	JUC1000010324.mm.1	2,15	0,014502	0,449545		
TC1100003276.mm.1	1,06	Pipox	JUC1100015792.mm.1	2,15	0,029402	0,509995		
TC0300002165.mm.1	-1,43	Zbbx	JUC0300008530.mm.1	2,15	0,022674	0,487625		
TC0X00000981.mm.1	1,04	Ogt	JUC0X00003275.mm.1	2,15	0,012099	0,43255		
TC0500000092.mm.1	-1,17	Gm6650	JUC0500000453.mm.1	2,15	0,02545	0,497549		
TC0X00002333.mm.1	1,08	Slitrk4	JUC0X00007480.mm.1	2,15	0,018395	0,468484		
TC0400002638.mm.1	-1,22	Aldob	JUC0400011129.mm.1	2,15	0,009627	0,417315		
TC0100001262.mm.1	2,74	Kif21b	PSR0100010488.mm.1	2,14	0,003882	0,359196		
TC0100001262.mm.1	2,74	Kif21b	PSR0100010504.mm.1	-2,42	0,014156	0,447466	Cassette Exon	0,15
TC0100001262.mm.1	2,74	Kif21b	JUC0100006045.mm.1	-2,52	0,002167	0,344341		
TC0100001262.mm.1	2,74	Kif21b	JUC0100006056.mm.1	-2,52	0,005441	0,379743		
TC0100001262.mm.1	2,74	Kif21b	PSR0100010467.mm.1	-2,54	0,016941	0,460989	Cassette Exon	0,15
TC0100001262.mm.1	2,74	Kif21b	PSR0100010496.mm.1	-2,61	0,012456	0,43528	Cassette Exon	0,28
TC0100001262.mm.1	2,74	Kif21b	PSR0100010497.mm.1	-2,62	0,003811	0,358103	Cassette Exon	0,29
TC0100001262.mm.1	2,74	Kif21b	PSR0100010465.mm.1	-2,7	0,004827	0,371481	Cassette Exon	0,15
TC0100001262.mm.1	2,74	Kif21b	JUC0100006057.mm.1	-2,74	0,005131	0,376377		
TC0100001262.mm.1	2,74	Kif21b	PSR0100010509.mm.1	-2,98	0,006557	0,39213	Alternative 5' Donc	0,34
TC0100001262.mm.1	2,74	Kif21b	PSR0100010477.mm.1	-2,99	0,002463	0,349135	Alternative 5' Donc	0,33
TC0100001262.mm.1	2,74	Kif21b	PSR0100010498.mm.1	-3,02	0,0068	0,393614	Alternative 3' Acce	0,09
TC0100001262.mm.1	2,74	Kif21b	PSR0100010499.mm.1	-3,07	0,002896	0,352207	Alternative 5' Donc	0,06

TC0100001262.mm.1	2,74	Kif21b	JUC0100006051.mm.1	-3,57	0,020162	0,477086		
TC0100001262.mm.1	2,74	Kif21b	JUC0100006064.mm.1	-4,23	0,017204	0,462491		
TC0100001262.mm.1	2,74	Kif21b	PSR0100010472.mm.1	-4,38	0,0034	0,354874	Intron Retention	0,63
TC0100001262.mm.1	2,74	Kif21b	PSR0100010474.mm.1	-4,53	0,003126	0,353892	Intron Retention	0,69
TC0100003587.mm.1	5,46	Cadm3	JUC0100016714.mm.1	2,14	0,002456	0,349135		
TC0100003587.mm.1	5,46	Cadm3	PSR0100029413.mm.1	-2,06	0,014076	0,446841	Cassette Exon	0,23
TC0100003587.mm.1	5,46	Cadm3	PSR0100029423.mm.1	-2,47	0,011246	0,426718	Alternative 5' Donc	0,24
TC0100003587.mm.1	5,46	Cadm3	PSR0100029427.mm.1	-3,3	0,007317	0,39887	Alternative 3' Acce	0,37
TC0100003587.mm.1	5,46	Cadm3	JUC0100016722.mm.1	-4,27	0,001135	0,317344		
TC0100003587.mm.1	5,46	Cadm3	PSR0100029412.mm.1	-5,12	0,000813	0,311886	Alternative 3' Acce	0,31
TC0100003587.mm.1	5,46	Cadm3	JUC0100016725.mm.1	-5,37	0,012149	0,432871		
TC0100003587.mm.1	5,46	Cadm3	JUC0100016717.mm.1	-5,38	0,000722	0,306115		
TC0100003587.mm.1	5,46	Cadm3	JUC0100016716.mm.1	-6,06	0,002118	0,342919		
TC0100003587.mm.1	5,46	Cadm3	PSR0100029417.mm.1	-7,21	0,00185	0,337414	Alternative 3' Acce	0,48
TC0100003587.mm.1	5,46	Cadm3	PSR0100029432.mm.1	-7,67	0,001021	0,316361	Cassette Exon	0,51
TC0100003587.mm.1	5,46	Cadm3	PSR0100029433.mm.1	-7,91	0,003118	0,353892	Cassette Exon	0,37
TC0100003587.mm.1	5,46	Cadm3	PSR0100029426.mm.1	-9,72	0,000832	0,311886	Alternative 5' Donc	0,41
TC0100003587.mm.1	5,46	Cadm3	JUC0100016724.mm.1	-14,35	0,003048	0,353892		
TC0800001143.mm.1	5,21	Ndrg4	PSR0800009013.mm.1	2,14	0,01479	0,450876	Cassette Exon	0,22
TC0800001143.mm.1	5,21	Ndrg4	PSR0800008995.mm.1	-2,15	0,000294	0,288663	Cassette Exon	0,1
TC0800001143.mm.1	5,21	Ndrg4	PSR0800008990.mm.1	-2,31	0,0409	0,543423		
TC0800001143.mm.1	5,21	Ndrg4	PSR0800008997.mm.1	-2,57	0,018638	0,470078	Cassette Exon	0,15
TC0800001143.mm.1	5,21	Ndrg4	PSR0800009014.mm.1	-2,68	0,025019	0,496077	Cassette Exon	0,29
TC0800001143.mm.1	5,21	Ndrg4	PSR0800008994.mm.1	-2,88	0,001283	0,322295	Cassette Exon	0,17
TC0800001143.mm.1	5,21	Ndrg4	JUC0800004825.mm.1	-3,06	0,000988	0,315787		
TC0800001143.mm.1	5,21	Ndrg4	JUC0800004828.mm.1	-3,25	0,003152	0,353892		
TC0800001143.mm.1	5,21	Ndrg4	PSR0800008988.mm.1	-3,27	0,020865	0,479521	Cassette Exon	0,35
TC0800001143.mm.1	5,21	Ndrg4	PSR0800009011.mm.1	-3,76	0,008242	0,405976	Alternative 5' Donc	0,36
TC0800001143.mm.1	5,21	Ndrg4	PSR0800008991.mm.1	-4,14	0,04716	0,557948	Alternative 3' Acce	0,47
TC0800001143.mm.1	5,21	Ndrg4	PSR0800009012.mm.1	-4,31	0,007782	0,402902	Alternative 3' Acce	0,48
TC0800001143.mm.1	5,21	Ndrg4	JUC0800004826.mm.1	-4,65	0,00825	0,405976		
TC0800001143.mm.1	5,21	Ndrg4	JUC0800004836.mm.1	-4,73	0,011179	0,426414		
TC0800001143.mm.1	5,21	Ndrg4	JUC0800004833.mm.1	-5,08	0,00003	0,229631		
TC0800001143.mm.1	5,21	Ndrg4	PSR0800009018.mm.1	-5,19	0,001964	0,340561	Alternative 5' Donc	0,38
TC0800001143.mm.1	5,21	Ndrg4	PSR0800008984.mm.1	-5,25	0,006636	0,392813	Cassette Exon	0,41
TC0800001143.mm.1	5,21	Ndrg4	PSR0800009010.mm.1	-5,33	0,000406	0,297771	Alternative 5' Donc	0,38
TC0800001143.mm.1	5,21	Ndrg4	JUC0800004838.mm.1	-5,54	0,013802	0,445012		
TC0800001143.mm.1	5,21	Ndrg4	JUC0800004816.mm.1	-6,06	0,0058	0,383104		
TC0800001143.mm.1	5,21	Ndrg4	JUC0800004834.mm.1	-6,42	0,004237	0,363295		
TC0800001143.mm.1	5,21	Ndrg4	JUC0800004837.mm.1	-6,56	0,000824	0,311886		
TC0800001143.mm.1	5,21	Ndrg4	PSR0800008986.mm.1	-6,97	0,003559	0,356223	Cassette Exon	0,41
TC0800001143.mm.1	5,21	Ndrg4	PSR0800008985.mm.1	-7,14	0,002389	0,348564	Cassette Exon	0,41
TC0800001143.mm.1	5,21	Ndrg4	PSR0800008983.mm.1	-8,31	0,002916	0,352289	Cassette Exon	0,41
TC1300002116.mm.1	3,66	Agtpbp1; A230056J06Rik	JUC1300006995.mm.1	2,14	0,049751	0,563595		
TC1300002116.mm.1	3,66	Agtpbp1; A230056J06Rik	PSR1300013507.mm.1	-2,34	0,010318	0,420696	Alternative 3' Acce	0,13
TC1300002116.mm.1	3,66	Agtpbp1; A230056J06Rik	PSR1300013577.mm.1	-2,6	0,047703	0,55896	Cassette Exon	0,15
TC1300002116.mm.1	3,66	Agtpbp1; A230056J06Rik	PSR1300013524.mm.1	-2,74	0,028444	0,507234	Alternative 3' Acce	0,33
TC1300002116.mm.1	3,66	Agtpbp1; A230056J06Rik	PSR1300013529.mm.1	-2,83	0,010674	0,423096	Cassette Exon	0,24

TC1300002116.mm.1	3,66	Agtpbp1; A230056J06Rik	PSR1300013525.mm.1	-2,87	0,018628	0,469941	Alternative 3' Acce	0,33
TC1300002116.mm.1	3,66	Agtpbp1; A230056J06Rik	PSR1300013506.mm.1	-2,87	0,022464	0,487039	Alternative 3' Acce	0,13
TC1300002116.mm.1	3,66	Agtpbp1; A230056J06Rik	JUC1300007005.mm.1	-3,33	0,004233	0,363295		
TC1300002116.mm.1	3,66	Agtpbp1; A230056J06Rik	PSR1300013568.mm.1	-3,4	0,010803	0,424055	Cassette Exon	0,33
TC1300002116.mm.1	3,66	Agtpbp1; A230056J06Rik	PSR1300013513.mm.1	-3,66	0,020088	0,4766	Cassette Exon	0,06
TC1300002116.mm.1	3,66	Agtpbp1; A230056J06Rik	JUC1300007015.mm.1	-3,78	0,028109	0,506167		
TC1300002116.mm.1	3,66	Agtpbp1; A230056J06Rik	PSR1300013556.mm.1	-3,79	0,017811	0,46577	Cassette Exon	0,28
TC1300002116.mm.1	3,66	Agtpbp1; A230056J06Rik	PSR1300013567.mm.1	-3,79	0,03321	0,522175	Cassette Exon	0,24
TC1300002116.mm.1	3,66	Agtpbp1; A230056J06Rik	PSR1300013523.mm.1	-4,06	0,005868	0,383835	Alternative 3' Acce	0,33
TC1300002116.mm.1	3,66	Agtpbp1; A230056J06Rik	PSR1300013563.mm.1	-4,15	0,003866	0,358861		
TC1300002116.mm.1	3,66	Agtpbp1; A230056J06Rik	PSR1300013562.mm.1	-4,28	0,023576	0,490947	Cassette Exon	0,19
TC1300002116.mm.1	3,66	Agtpbp1; A230056J06Rik	PSR1300013566.mm.1	-4,51	0,018483	0,46874		
TC1300002116.mm.1	3,66	Agtpbp1; A230056J06Rik	PSR1300013536.mm.1	-4,58	0,013806	0,445064	Cassette Exon	0,42
TC1300002116.mm.1	3,66	Agtpbp1; A230056J06Rik	PSR1300013564.mm.1	-5,09	0,01358	0,443703		
TC1300002116.mm.1	3,66	Agtpbp1; A230056J06Rik	PSR1300013551.mm.1	-5,16	0,005059	0,375079	Alternative 3' Acce	0,46
TC1300002116.mm.1	3,66	Agtpbp1; A230056J06Rik	JUC1300006979.mm.1	-6,05	0,014164	0,447466		
TC1300002116.mm.1	3,66	Agtpbp1; A230056J06Rik	JUC1300007007.mm.1	-7,04	0,049059	0,562009		
TC1300002116.mm.1	3,66	Agtpbp1; A230056J06Rik	JUC1300007009.mm.1	-7,14	0,019219	0,472971		
TC1300002116.mm.1	3,66	Agtpbp1; A230056J06Rik	JUC1300007006.mm.1	-7,48	0,00253	0,349501		
TC1300002116.mm.1	3,66	Agtpbp1; A230056J06Rik	JUC1300007016.mm.1	-13,06	0,008106	0,405169		
TC0700000465.mm.1	1,2	Akt2	JUC0700001883.mm.1	2,14	0,003629	0,3566		
TC0700000465.mm.1	1,2	Akt2	PSR0700003750.mm.1	-2,57	0,046758	0,556906	Intron Retention	0,38
TC0100002748.mm.1	1,38	Pde6d	JUC0100012582.mm.1	2,14	0,007061	0,396462		
TC0100002748.mm.1	1,38	Pde6d	PSR0100022196.mm.1	-3,33	0,009809	0,417839	Alternative 3' Acce	0,36
TC0100003142.mm.1	-1,24	5730559C18Rik	PSR0100025566.mm.1	2,14	0,013034	0,439367	Cassette Exon	0,2
TC0200001102.mm.1	-1,07	Mtx2	PSR0200008925.mm.1	2,14	0,028723	0,508141	Alternative 3' Acce	0,18
TC0900000532.mm.1	-1,5	Mpzl3	PSR0900003654.mm.1	2,14	0,025763	0,498442	Alternative 3' Acce	0,18
TC0400003011.mm.1	-1,13	Ifna16; Gm13280	PSR0400024333.mm.1	2,14	0,036442	0,530977	Alternative 5' Donc	0,18
TC0100002810.mm.1	-1,03	Ndufa10	PSR0100022760.mm.1	2,14	0,023219	0,490032	Alternative 3' Acce	0,17
TC0500002615.mm.1	-1,56	Chic2	PSR0500023172.mm.1	2,14	0,020644	0,478859	Cassette Exon	0,16
TC0800001501.mm.1	-1,06	Cdh15	PSR0800012236.mm.1	2,14	0,000663	0,304044	Alternative 5' Donc	0,16
TC0500001795.mm.1	-1,13	Rpl21; Gm15682; Gm1004	PSR0500016615.mm.1	2,14	0,045186	0,55351	Alternative 3' Acce	0,15
TC0500003529.mm.1	1,17	Radil	PSR0500032338.mm.1	2,14	0,01694	0,460989	Cassette Exon	0,14
TC0300001953.mm.1	1,25	Elf2	JUC0300007814.mm.1	2,14	0,015723	0,455416		
TC0300001953.mm.1	1,25	Elf2	PSR0300014870.mm.1	-2,18	0,016268	0,458348	Cassette Exon	0,13
TC0X00000436.mm.1	-1,13	Rab33a	PSR0X00002916.mm.1	2,14	0,002538	0,349501	Cassette Exon	0,12
TC0200005402.mm.1	1,07	Lama5	PSR0200045961.mm.1	2,14	0,030186	0,512365	Cassette Exon	0,11
TC0700004591.mm.1	-1,48	Tnfrsf26	PSR0700038814.mm.1	2,14	0,001719	0,335996	Cassette Exon	0,11
TC0700004591.mm.1	-1,48	Tnfrsf26	JUC0700020432.mm.1	2,01	0,008368	0,406435		
TC1000001810.mm.1	-1,14	Gm4922	PSR1000013186.mm.1	2,14	0,008138	0,405362	Cassette Exon	0,11
TC1900000034.mm.1	-1,66		PSR1900000483.mm.1	2,14	0,025853	0,498657	Cassette Exon	0,11
TC0100003176.mm.1	-1,58	2310009B15Rik	PSR0100025769.mm.1	2,14	0,012283	0,434168	Alternative 3' Acce	0,08
TC1100001012.mm.1	1,34	Camkk1	PSR1100009129.mm.1	2,14	0,006613	0,392269	Cassette Exon	0,08
TC0200003147.mm.1	-1,1	Uap1l1	PSR0200025976.mm.1	2,14	0,040842	0,543283		
TC0200005146.mm.1	1,02	Elmo2	JUC0200022678.mm.1	2,14	0,002501	0,349189		
TC0200005146.mm.1	1,02	Elmo2	JUC0200022686.mm.1	-2,1	0,031393	0,516433		
TC1100003928.mm.1	-1,22	Tex2	JUC1100019258.mm.1	2,14	0,015042	0,45215		
TC1300001729.mm.1	-1,06	E2f3	JUC1300005484.mm.1	2,14	0,006275	0,388804		

TC1300002301.mm.1	-1,28	D630045M09Rik	JUC1300007889.mm.1	2,14	0,036075	0,529704		
TC1900001025.mm.1	-1,41	Plcb3	JUC1900005223.mm.1	2,14	0,004391	0,366156		
TC1800001160.mm.1	-1,43	Gm26538	JUC1800004769.mm.1	2,14	0,000467	0,298999		
TC0200001411.mm.1	-1,1	Pex16	JUC0200005561.mm.1	2,14	0,009669	0,417554		
TC0200001411.mm.1	-1,1	Pex16	JUC0200005559.mm.1	2,08	0,011677	0,429939		
TC0200000495.mm.1	1,02	Ddx31	JUC0200001750.mm.1	2,14	0,026966	0,502831		
TC0800000590.mm.1	-1,31	Spata4	JUC0800002206.mm.1	2,14	0,027486	0,504189		
TC0700000420.mm.1	1,49	Atp5sl	JUC0700001634.mm.1	2,14	0,046455	0,556553		
TC0700000420.mm.1	1,49	Atp5sl	JUC0700001638.mm.1	-2,47	0,018117	0,467161		
TC0700000420.mm.1	1,49	Atp5sl	JUC0700001636.mm.1	-2,91	0,044739	0,552331		
TC0700000239.mm.1	-1,32	Prkd2	JUC0700000876.mm.1	2,14	0,013576	0,443703		
TC0600002578.mm.1	-1,58	Clec4f	JUC0600010397.mm.1	2,14	0,044326	0,551401		
TC0600001748.mm.1	-1,31	Ccdc91	JUC0600007300.mm.1	2,14	0,011734	0,429969		
TC0300003249.mm.1	-1,16	Hiat1	JUC0300011558.mm.1	2,14	0,017989	0,466929		
TC1100000900.mm.1	1,05	Per1	JUC1100003995.mm.1	2,14	0,045233	0,553651		
TC0400001246.mm.1	1,25	Pomgnt1; 2510003B16Rik	JUC0400004824.mm.1	2,14	0,012582	0,435924		
TC1100001820.mm.1	-1,03	Axin2	JUC1100008942.mm.1	2,14	0,009348	0,415092		
TC0400004216.mm.1	-1,57	Podn	JUC0400013388.mm.1	2,14	0,028204	0,506368		
TC1000000888.mm.1	1,05	Zfr2	JUC1000003741.mm.1	2,14	0,047527	0,558686		
TC0400004175.mm.1	26,52	Samd11	JUC0400018306.mm.1	2,13	0,02766	0,504707		
TC0400004175.mm.1	26,52	Samd11	PSR0400035138.mm.1	-2,04	0,039269	0,538976	Cassette Exon	0,2
TC0400004175.mm.1	26,52	Samd11	JUC0400018303.mm.1	-3,1	0,002599	0,349501		
TC0400004175.mm.1	26,52	Samd11	PSR0400035152.mm.1	-3,91	0,01811	0,467161	Alternative 5' Donc	0,46
TC0400004175.mm.1	26,52	Samd11	PSR0400035142.mm.1	-5,99	0,008036	0,40434	Intron Retention	0,62
TC0400004175.mm.1	26,52	Samd11	PSR0400035137.mm.1	-6,04	0,000196	0,28803	Alternative 3' Acce	0,31
TC0400004175.mm.1	26,52	Samd11	PSR0400035156.mm.1	-13,72	0,003764	0,357586	Alternative 5' Donc	0,42
TC0100003791.mm.1	10,39	Prox1	PSR0100030972.mm.1	2,13	0,029833	0,511132		
TC0100003791.mm.1	10,39	Prox1	PSR0100030977.mm.1	-2,22	0,003329	0,354243	Alternative 3' Acce	0,23
TC0100003791.mm.1	10,39	Prox1	PSR0100030975.mm.1	-4,39	0,006343	0,389463	Alternative 3' Acce	0,23
TC0100003791.mm.1	10,39	Prox1	PSR0100030967.mm.1	-4,64	0,038286	0,536059	Alternative 3' Acce	0,42
TC0100003791.mm.1	10,39	Prox1	PSR0100030986.mm.1	-5,52	0,011668	0,429939	Cassette Exon	0,28
TC0100003791.mm.1	10,39	Prox1	PSR0100030985.mm.1	-5,68	0,006128	0,387351	Cassette Exon	0,28
TC0100003791.mm.1	10,39	Prox1	PSR0100030966.mm.1	-6,72	0,021573	0,483165	Alternative 3' Acce	0,42
TC0100003791.mm.1	10,39	Prox1	JUC0100017585.mm.1	-9,06	0,016566	0,459612		
TC0100003791.mm.1	10,39	Prox1	JUC0100017580.mm.1	-10,15	0,032362	0,519174		
TC0100003791.mm.1	10,39	Prox1	PSR0100030984.mm.1	-10,21	0,006377	0,389488	Cassette Exon	0,41
TC0X00000613.mm.1	1,38	Fmr1	JUC0X00001955.mm.1	2,13	0,016317	0,45857		
TC0X00000613.mm.1	1,38	Fmr1	PSR0X00003863.mm.1	-2,97	0,010107	0,418998	Alternative 3' Acce	0,3
TC0X00002453.mm.1	-1,09	G6pdx	PSR0X00015677.mm.1	2,13	0,000287	0,28803	Alternative 5' Donc	0,18
TC0200003931.mm.1	-1,69	Olfr1056	PSR0200033754.mm.1	2,13	0,021491	0,482644	Alternative 3' Acce	0,17
TC1200002210.mm.1	-1,59		PSR1200015261.mm.1	2,13	0,019315	0,473288	Alternative 3' Acce	0,17
TC1600001575.mm.1	-2,5	Stfa3	PSR1600012853.mm.1	2,13	0,012886	0,438165	Cassette Exon	0,15
TC0100002576.mm.1	-1,54	Tns1	PSR0100020744.mm.1	2,13	0,005771	0,383104	Cassette Exon	0,14
TC0100002576.mm.1	-1,54	Tns1	PSR0100020739.mm.1	2,08	0,024661	0,494682	Cassette Exon	0,11
TC0500000623.mm.1	1,52	N4bp2	JUC0500003047.mm.1	2,13	0,040377	0,541959		
TC0500000623.mm.1	1,52	N4bp2	PSR0500005523.mm.1	-2,11	0,04327	0,54924	Alternative 5' Donc	0,13
TC0500000623.mm.1	1,52	N4bp2	JUC0500003063.mm.1	-2,5	0,028683	0,508019		
TC0100001779.mm.1	1,31	Rab3gap2	PSR0100014787.mm.1	2,13	0,017751	0,465425	Alternative 5' Donc	0,12

TC0600002565.mm.1	-1,15	Mthfd2	PSR0600019915.mm.1	2,13	0,016278	0,458372	Cassette Exon	0,11
TC0600002565.mm.1	-1,15	Mthfd2	JUC0600010337.mm.1	-2,44	0,026575	0,501647		
TC0800001212.mm.1	-1,64	E2f4	PSR0800009534.mm.1	2,13	0,014951	0,451968	Cassette Exon	0,11
TC1900000635.mm.1	1,06	Cutc	PSR1900005637.mm.1	2,13	0,005304	0,378059	Cassette Exon	0,11
TC0300001081.mm.1	-1,46	Rhoc	PSR0300008738.mm.1	2,13	0,024522	0,493992	Cassette Exon	0,1
TC0700003760.mm.1	-1,55	Gm15415	PSR0700031613.mm.1	2,13	0,043287	0,549297	Cassette Exon	0,1
TC0X00002187.mm.1	-1,03	Frmd7	PSR0X00013985.mm.1	2,13	0,004627	0,369794	Cassette Exon	0,1
TC1000003020.mm.1	-1,47		PSR1000022580.mm.1	2,13	0,000493	0,299348	Cassette Exon	0,1
TC1100002449.mm.1	1,01	Erlec1	PSR1100022850.mm.1	2,13	0,021318	0,481723	Cassette Exon	0,1
TC1200001459.mm.1	-1,49		PSR1200010179.mm.1	2,13	0,00164	0,333296	Cassette Exon	0,1
TC0500001139.mm.1	-1,49	Pole	PSR0500009748.mm.1	2,13	0,035669	0,528966	Cassette Exon	0,08
TC0900001771.mm.1	-1,55	Amotl1	PSR0900014839.mm.1	2,13	0,000876	0,311909	Cassette Exon	0,05
TC1400000317.mm.1	-1,12	Itih4	JUC1400001541.mm.1	2,13	0,038498	0,536557		
TC0200002745.mm.1	1,01	Gm14402	JUC0200011369.mm.1	2,13	0,043815	0,550456		
TC0200004253.mm.1	-1,34	Eif3m	JUC0200018310.mm.1	2,13	0,036716	0,531762		
TC0200004253.mm.1	-1,34	Eif3m	JUC0200018314.mm.1	2,06	0,008832	0,410293		
TC1800000503.mm.1	-1,11	Dmxl1	JUC1800002046.mm.1	2,13	0,017449	0,463886		
TC0100003096.mm.1	-1,06	Atp2b4; Mir6903; mmu-m	JUC0100014175.mm.1	2,13	0,037452	0,533908		
TC0100000257.mm.1	1,17	Vwa3b	JUC0100000957.mm.1	2,13	0,006377	0,389488		
TC0100000257.mm.1	1,17	Vwa3b	JUC0100000980.mm.1	-2,01	0,008618	0,408434		
TC0100003657.mm.1	-1,35	Tfb2m	JUC0100016998.mm.1	2,13	0,029692	0,510949		
TC1700000584.mm.1	-1,02	Adamts10	JUC1700002902.mm.1	2,13	0,010836	0,424161		
TC1700000584.mm.1	-1,02	Adamts10	JUC1700002906.mm.1	-2,24	0,032969	0,521306		
TC0800000148.mm.1	-1,05	Arhgef10	JUC0800000789.mm.1	2,13	0,005335	0,378059		
TC0500000263.mm.1	1,03	Rbm33	JUC0500001118.mm.1	2,13	0,033787	0,523767		
TC0500001632.mm.1	1,1	Sap25; Lrch4; Gm20605	PSR0500014868.mm.1	2,13	0,04766	0,558824		
TC0500001632.mm.1	1,1	Sap25; Lrch4; Gm20605	JUC0500008115.mm.1	-4,91	0,02062	0,478665		
TC0700000387.mm.1	-1,01	Xrcc1	JUC0700001383.mm.1	2,13	0,00126	0,322251		
TC0700000870.mm.1	1,23	Nomo1	JUC0700003834.mm.1	2,13	0,002462	0,349135		
TC0700000870.mm.1	1,23	Nomo1	JUC0700003826.mm.1	2,03	0,022431	0,486856		
TC0600002799.mm.1	1,14	Uba3	JUC0600011290.mm.1	2,13	0,012207	0,433286		
TC0300003080.mm.1	-1,69	Clca4	JUC0300012665.mm.1	2,13	0,033017	0,521478		
TC1100002897.mm.1	-1,34	Shmt1	JUC1100013696.mm.1	2,13	0,014547	0,449564		
TC1100002637.mm.1	1,1	Trim41	JUC1100012545.mm.1	2,13	0,006389	0,389608		
TC0400004133.mm.1	2,22	Plch2	JUC0400018018.mm.1	2,12	0,001451	0,328414		
TC0400004133.mm.1	2,22	Plch2	PSR0400034573.mm.1	-2,05	0,010221	0,419955	Alternative 5' Donc	0,22
TC0400004133.mm.1	2,22	Plch2	PSR0400034592.mm.1	-2,16	0,016958	0,4613	Cassette Exon	0,05
TC0400004133.mm.1	2,22	Plch2	PSR0400034559.mm.1	-2,17	0,013542	0,44358	Cassette Exon	0,14
TC0400004133.mm.1	2,22	Plch2	JUC0400018014.mm.1	-2,23	0,037046	0,532687		
TC0400004133.mm.1	2,22	Plch2	JUC0400018028.mm.1	-2,24	0,007046	0,396259		
TC0400004133.mm.1	2,22	Plch2	PSR0400034593.mm.1	-2,37	0,025265	0,496957	Cassette Exon	0,2
TC0400004133.mm.1	2,22	Plch2	PSR0400034550.mm.1	-2,41	0,019609	0,474765	Intron Retention	0,3
TC0400004133.mm.1	2,22	Plch2	PSR0400034568.mm.1	-2,52	0,016811	0,460623	Alternative 3' Acce	0,25
TC0400004133.mm.1	2,22	Plch2	PSR0400034594.mm.1	-2,65	0,005333	0,378059	Cassette Exon	0,17
TC0400004133.mm.1	2,22	Plch2	PSR0400034590.mm.1	-2,78	0,001628	0,332831	Cassette Exon	0,13
TC0400004133.mm.1	2,22	Plch2	JUC0400018016.mm.1	-3,05	0,026045	0,49952		
TC1500001881.mm.1	1,69	Fam227a	JUC1500008405.mm.1	2,12	0,023483	0,490673		
TC1500001881.mm.1	1,69	Fam227a	JUC1500008400.mm.1	-2,12	0,015961	0,456885		

TC1500001881.mm.1	1,69	Fam227a	JUC1500008398.mm.1	-2,16	0,019983	0,476227		
TC1500001881.mm.1	1,69	Fam227a	PSR1500014744.mm.1	-2,21	0,009238	0,414373	Cassette Exon	0,28
TC1500001881.mm.1	1,69	Fam227a	JUC1500008415.mm.1	-2,27	0,023112	0,489756		
TC1500001881.mm.1	1,69	Fam227a	PSR1500014754.mm.1	-2,36	0,003728	0,357586	Cassette Exon	0,14
TC1500001881.mm.1	1,69	Fam227a	PSR1500014764.mm.1	-2,5	0,000402	0,297771	Cassette Exon	0,14
TC1500001881.mm.1	1,69	Fam227a	JUC1500008411.mm.1	-3,68	0,020199	0,477145		
TC1500001881.mm.1	1,69	Fam227a	JUC1500008395.mm.1	-4,59	0,000734	0,307808		
TC0300001300.mm.1	-2,02	Pitx2	PSR0300010533.mm.1	2,12	0,008857	0,410455	Intron Retention	0,27
TC0300001300.mm.1	-2,02	Pitx2	PSR0300010524.mm.1	2,07	0,04758	0,558743	Cassette Exon	0,11
TC0300001300.mm.1	-2,02	Pitx2	PSR0300010518.mm.1	2,04	0,046121	0,555804	Alternative 3' Acce	0,14
TC0200002772.mm.1	1,47	Cdh4; Gm10711	JUC0200011459.mm.1	2,12	0,008168	0,405603		
TC0200002772.mm.1	1,47	Cdh4; Gm10711	PSR0200022634.mm.1	-2,02	0,016682	0,460085	Cassette Exon	0,12
TC0200002772.mm.1	1,47	Cdh4; Gm10711	PSR0200022612.mm.1	-2,2	0,003282	0,354243	Cassette Exon	0,25
TC0200002772.mm.1	1,47	Cdh4; Gm10711	JUC0200011473.mm.1	-4,06	0,01307	0,4396		
TC0200002784.mm.1	-1,3	Adrm1	PSR0200022766.mm.1	2,12	0,013503	0,443238	Intron Retention	0,23
TC1500000599.mm.1	1,43	Adck5	JUC1500002493.mm.1	2,12	0,012624	0,436032		
TC1500000599.mm.1	1,43	Adck5	PSR1500004324.mm.1	-2,26	0,006869	0,394155	Alternative 3' Acce	0,2
TC0500000201.mm.1	1,12	Klhl7	PSR0500001480.mm.1	2,12	0,008762	0,409779	Cassette Exon	0,19
TC0500000201.mm.1	1,12	Klhl7	JUC0500000856.mm.1	2,08	0,018958	0,471398		
TC0700002366.mm.1	-1,1	6330408A02Rik	PSR0700021008.mm.1	2,12	0,009338	0,415075	Alternative 3' Acce	0,18
TC0900001759.mm.1	-1,39	4930568E12Rik	PSR0900014714.mm.1	2,12	0,028552	0,507497	Alternative 3' Acce	0,18
TC0700002979.mm.1	1,39	Cpt1c	PSR0700026691.mm.1	2,12	0,000843	0,311909	Cassette Exon	0,13
TC0700002979.mm.1	1,39	Cpt1c	PSR0700026689.mm.1	2,11	0,000491	0,298999	Cassette Exon	0,16
TC1100001359.mm.1	2,27	Srsf1	PSR1100012785.mm.1	2,12	0,024772	0,495014	Alternative 3' Acce	0,06
TC1100001359.mm.1	2,27	Srsf1	PSR1100012799.mm.1	-2,04	0,029725	0,511057	Intron Retention	0,15
TC0300001704.mm.1	-2,57	Cpa3	PSR0300013433.mm.1	2,12	0,039425	0,539611	Cassette Exon	0,14
TC1500000782.mm.1	1,62	Pnpla3	PSR1500006133.mm.1	2,12	0,026131	0,499897	Cassette Exon	0,14
TC0100002544.mm.1	-1,27	Fn1	PSR0100020538.mm.1	2,12	0,043127	0,548648	Alternative 3' Acce	0,13
TC0300001047.mm.1	-1,17	Bcas2	PSR0300008448.mm.1	2,12	0,046241	0,556133	Cassette Exon	0,13
TC1500000587.mm.1	-1,08	Cyc1	PSR1500004080.mm.1	2,12	0,025349	0,497375	Intron Retention	0,13
TC0500001466.mm.1	-1,19	Tmem132b	PSR0500013295.mm.1	2,12	0,036293	0,530447	Cassette Exon	0,12
TC1600000765.mm.1	-1,1	Cldn25	PSR1600006646.mm.1	2,12	0,036101	0,529729	Alternative 3' Acce	0,12
TC1600000765.mm.1	-1,1	Cldn25	JUC1600003530.mm.1	2,05	0,024818	0,495304		
TC1000001216.mm.1	-1,36	Kitl	PSR1000009133.mm.1	2,12	0,022062	0,485335	Cassette Exon	0,11
TC1400000517.mm.1	1,5	Ktn1	PSR1400004193.mm.1	2,12	0,008129	0,405267	Alternative 3' Acce	0,11
TC1900001415.mm.1	-1,36	Acta2	PSR1900012407.mm.1	2,12	0,017894	0,466466	Cassette Exon	0,1
TC0500003513.mm.1	2,24	Ttyh3	PSR0500032199.mm.1	2,12	0,02867	0,508006	Cassette Exon	0,09
TC1000003019.mm.1	-1,27	Irak3	JUC1000012373.mm.1	2,12	0,04658	0,556761		
TC1000003019.mm.1	-1,27	Irak3	PSR1000022558.mm.1	-2,01	0,030118	0,511997	Alternative 3' Acce	0,08
TC1800000980.mm.1	1,16	Abhd3	PSR1800007277.mm.1	2,12	0,013246	0,441416	Cassette Exon	0,07
TC1300002169.mm.1	-1,08	6720489N17Rik; Zfp935	JUC1300007233.mm.1	2,12	0,018315	0,468088		
TC1500001762.mm.1	1,28	Puf60	JUC1500007602.mm.1	2,12	0,036717	0,531762		
TC1900000061.mm.1	-1,31	Catsper1	JUC1900000401.mm.1	2,12	0,030946	0,514624		
TC0100003272.mm.1	-1,15	Lamc2	JUC0100015167.mm.1	2,12	0,025985	0,499207		
TC1600001262.mm.1	1,19	Fgd4	JUC1600005333.mm.1	2,12	0,009761	0,417839		
TC0800002515.mm.1	-1,26	1700011L22Rik	JUC0800010401.mm.1	2,12	0,003432	0,355303		
TC0900003323.mm.1	-1,07	C1qtnf5; Mfrp	JUC0900001689.mm.1	2,12	0,020612	0,478576		
TC1100003680.mm.1	-1,22	Ikzf3	JUC1100017746.mm.1	2,12	0,019811	0,475266		

TC1700002348.mm.1	5,77	Tubb4a	JUC1700011808.mm.1	2,11	0,028131	0,506229		
TC1700002348.mm.1	5,77	Tubb4a	PSR1700022044.mm.1	-5,63	0,042234	0,547034	Alternative 5' Donc	0,46
TC0800002238.mm.1	10,85	Wdr17	PSR0800017036.mm.1	2,11	0,019506	0,47452	Alternative 3' Acce	0,18
TC0800002238.mm.1	10,85	Wdr17	PSR0800017032.mm.1	2,08	0,006379	0,389488	Cassette Exon	0,11
TC0800002238.mm.1	10,85	Wdr17	PSR0800017054.mm.1	-2,01	0,004048	0,361816	Cassette Exon	0,33
TC0800002238.mm.1	10,85	Wdr17	JUC0800009273.mm.1	-2,01	0,0398	0,540753		
TC0800002238.mm.1	10,85	Wdr17	JUC0800009254.mm.1	-2,07	0,044565	0,551921		
TC0800002238.mm.1	10,85	Wdr17	PSR0800017022.mm.1	-2,49	0,010508	0,422115	Alternative 5' Donc	0,16
TC0800002238.mm.1	10,85	Wdr17	PSR0800017058.mm.1	-3,15	0,008587	0,40821	Cassette Exon	0,13
TC0800002238.mm.1	10,85	Wdr17	PSR0800017063.mm.1	-3,56	0,016027	0,457132	Alternative 5' Donc	0,1
TC0800002238.mm.1	10,85	Wdr17	PSR0800017005.mm.1	-4,21	0,030256	0,512521	Alternative 3' Acce	0,44
TC0800002238.mm.1	10,85	Wdr17	JUC0800009252.mm.1	-4,33	0,008956	0,411476		
TC0800002238.mm.1	10,85	Wdr17	PSR0800017056.mm.1	-4,35	0,001335	0,325349	Cassette Exon	0,07
TC0800002238.mm.1	10,85	Wdr17	PSR0800017025.mm.1	-4,6	0,003769	0,357586	Cassette Exon	0,29
TC0800002238.mm.1	10,85	Wdr17	JUC0800009275.mm.1	-4,74	0,00684	0,393893		
TC0800002238.mm.1	10,85	Wdr17	JUC0800009247.mm.1	-4,92	0,00479	0,37131		
TC0800002238.mm.1	10,85	Wdr17	PSR0800017045.mm.1	-5,55	0,026381	0,50105	Alternative 3' Acce	0,42
TC0800002238.mm.1	10,85	Wdr17	JUC0800009284.mm.1	-6,62	0,021793	0,484434		
TC0800002238.mm.1	10,85	Wdr17	PSR0800017066.mm.1	-7,1	0,00334	0,354243	Cassette Exon	0,41
TC0800002238.mm.1	10,85	Wdr17	JUC0800009279.mm.1	-8,12	0,003008	0,353447		
TC0800002238.mm.1	10,85	Wdr17	JUC0800009291.mm.1	-8,34	0,008152	0,40551		
TC0800002238.mm.1	10,85	Wdr17	PSR0800017024.mm.1	-8,37	0,005689	0,382387	Cassette Exon	0,28
TC0800002238.mm.1	10,85	Wdr17	PSR0800017055.mm.1	-8,54	0,001277	0,322251	Cassette Exon	0,07
TC0800002238.mm.1	10,85	Wdr17	PSR0800017069.mm.1	-8,64	0,004562	0,369273	Cassette Exon	0,41
TC0800002238.mm.1	10,85	Wdr17	PSR0800017067.mm.1	-10,25	0,01349	0,44319	Cassette Exon	0,41
TC0800002238.mm.1	10,85	Wdr17	PSR0800017064.mm.1	-10,82	0,001925	0,34017	Cassette Exon	0,41
TC0800002238.mm.1	10,85	Wdr17	JUC0800009283.mm.1	-11,12	0,004705	0,370888		
TC0800002238.mm.1	10,85	Wdr17	JUC0800009272.mm.1	-12,64	0,000585	0,304044		
TC0800002238.mm.1	10,85	Wdr17	PSR0800017065.mm.1	-13,01	0,003738	0,357586	Cassette Exon	0,41
TC0800002238.mm.1	10,85	Wdr17	JUC0800009289.mm.1	-14,38	0,002895	0,352207		
TC0800002238.mm.1	10,85	Wdr17	PSR0800017062.mm.1	-15,09	0,001114	0,317328	Alternative 5' Donc	0,14
TC0800002238.mm.1	10,85	Wdr17	JUC0800009285.mm.1	-16,06	0,008563	0,40821		
TC0800002238.mm.1	10,85	Wdr17	JUC0800009287.mm.1	-16,23	0,003988	0,360652		
TC0800002238.mm.1	10,85	Wdr17	JUC0800009286.mm.1	-19,34	0,000278	0,28803		
TC0800002238.mm.1	10,85	Wdr17	JUC0800009277.mm.1	-35,86	0,000241	0,28803		
TC1400002335.mm.1	3	Ppp3cc	PSR1400018181.mm.1	2,11	0,041682	0,545356	Cassette Exon	0,23
TC1400002335.mm.1	3	Ppp3cc	PSR1400018180.mm.1	-2,28	0,02691	0,502518	Alternative 5' Donc	0,22
TC1400002335.mm.1	3	Ppp3cc	PSR1400018178.mm.1	-2,44	0,002846	0,352207	Intron Retention	0,29
TC1400002335.mm.1	3	Ppp3cc	JUC1400010008.mm.1	-3,33	0,016996	0,461553		
TC1400002335.mm.1	3	Ppp3cc	JUC1400010005.mm.1	-3,75	0,035663	0,528966		
TC0800000744.mm.1	-1,16	Cope	PSR0800005223.mm.1	2,11	0,030134	0,512017	Intron Retention	0,23
TC0200002232.mm.1	-1,76	Gins1	PSR0200017585.mm.1	2,11	0,008502	0,407296	Alternative 3' Acce	0,18
TC0200002778.mm.1	-1,29	Lsm14b	PSR0200022662.mm.1	2,11	0,015675	0,455416	Cassette Exon	0,18
TC0200002778.mm.1	-1,29	Lsm14b	PSR0200022661.mm.1	2,09	0,032777	0,520748	Cassette Exon	0,12
TC0300000668.mm.1	1,02	Pet112	PSR0300004403.mm.1	2,11	0,003241	0,354243	Cassette Exon	0,17
TC0900000497.mm.1	1,7	Usp2	PSR0900003270.mm.1	2,11	0,030277	0,512602	Alternative 3' Acce	0,16
TC0200001396.mm.1	-1,03	Phf21a	PSR0200010953.mm.1	2,11	0,026302	0,500653	Cassette Exon	0,15
TC1600002132.mm.1	-2,15		PSR1600016372.mm.1	2,11	0,001403	0,325997	Cassette Exon	0,14

TC0100002631.mm.1	-1,07	Farsb	PSR0100021416.mm.1	2,11	0,031088	0,515208	Cassette Exon	0,13
TC1900000508.mm.1	-1,13	Kif20b	PSR1900004487.mm.1	2,11	0,000918	0,313363	Cassette Exon	0,1
TC1900000508.mm.1	-1,13	Kif20b	JUC1900002361.mm.1	2,11	0,025408	0,497403		
TC1900000508.mm.1	-1,13	Kif20b	PSR1900004489.mm.1	2,05	0,04292	0,548271	Cassette Exon	0,13
TC1900000508.mm.1	-1,13	Kif20b	JUC1900002346.mm.1	-2,65	0,006493	0,390878		
TC1900000508.mm.1	-1,13	Kif20b	JUC1900002348.mm.1	-2,76	0,000659	0,304044		
TC0200005500.mm.1	-1,11	Alkbh3	PSR0200035074.mm.1	2,11	0,013315	0,441836	Cassette Exon	0,11
TC0200005500.mm.1	-1,11	Alkbh3	PSR0200035072.mm.1	2,08	0,038715	0,537292	Cassette Exon	0,09
TC0400001925.mm.1	-1,26	Mad2l2	PSR0400015693.mm.1	2,11	0,004413	0,366761	Intron Retention	0,11
TC1200000282.mm.1	-1,74	Sypl	PSR1200002191.mm.1	2,11	0,043323	0,549316	Cassette Exon	0,11
TC0900000050.mm.1	1,04	Mmp8	PSR0900000408.mm.1	2,11	0,0429	0,548193	Cassette Exon	0,1
TC1700002527.mm.1	-1,01	Srd5a2	PSR1700023166.mm.1	2,11	0,031709	0,517443	Cassette Exon	0,1
TC1100003318.mm.1	1,27	Wsb1	PSR1100030561.mm.1	2,11	0,008351	0,406435	Alternative 5' Donc	0,08
TC1100004000.mm.1	-1,09	Abca9	JUC1100019629.mm.1	2,11	0,034474	0,525929		
TC1100004000.mm.1	-1,09	Abca9	JUC1100019658.mm.1	-2,82	0,032478	0,519641		
TC1100004000.mm.1	-1,09	Abca9	JUC1100019660.mm.1	-3,51	0,00685	0,393893		
TC0300001063.mm.1	-1,19		JUC0300004453.mm.1	2,11	0,026225	0,500162		
TC1300002725.mm.1	1,26	Parp8	JUC1300009928.mm.1	2,11	0,03248	0,519641		
TC1800000771.mm.1	-1,14	Mex3c	JUC1800003072.mm.1	2,11	0,024968	0,495945		
TC1900000521.mm.1	1,25	Tnks2	JUC1900002432.mm.1	2,11	0,022975	0,48884		
TC0200001591.mm.1	-1,11	Dcdc5; BC048594	JUC0200005972.mm.1	2,11	0,015034	0,452111		
TC0200002301.mm.1	-1,05	Tpx2	JUC0200009036.mm.1	2,11	0,022219	0,485896		
TC0500002168.mm.1	-1,09	Cgref1	JUC0500010746.mm.1	2,11	0,021269	0,48159		
TC0800001089.mm.1	-1,83	Ogfod1	JUC0800004486.mm.1	2,11	0,022626	0,487464		
TC0800001130.mm.1	-1	Katnb1	JUC0800004750.mm.1	2,11	0,019778	0,475077		
TC0800002184.mm.1	-1,56	Wwc2	JUC0800009172.mm.1	2,11	0,041814	0,545586		
TC0700004599.mm.1	1,02	Nadsyn1	JUC0700020493.mm.1	2,11	0,001534	0,330388		
TC0800002452.mm.1	-1,13	Cib3	JUC0800010224.mm.1	2,11	0,020531	0,478295		
TC0600002098.mm.1	-1,16	Zc3hav1l	JUC0600008673.mm.1	2,11	0,004306	0,364136		
TC0600003296.mm.1	-1,72		JUC0600013485.mm.1	2,11	0,033858	0,524049		
TC0700001736.mm.1	-1,45	Eef2k	JUC0700007683.mm.1	2,11	0,02302	0,489186		
TC0700001736.mm.1	-1,45	Eef2k	JUC0700007691.mm.1	-2,17	0,014854	0,451513		
TC0700001878.mm.1	-1,03	Armc5	JUC0700008606.mm.1	2,11	0,02719	0,50335		
TC1000000537.mm.1	1,02	Micu1	JUC1000001973.mm.1	2,11	0,033453	0,522793		
TC0X00003083.mm.1	-1,19	Col4a6	JUC0X00009833.mm.1	2,11	0,041831	0,545586		
TC0100000558.mm.1	2,96	Unc80	PSR0100004524.mm.1	2,1	0,013996	0,446353	Cassette Exon	0,16
TC0100000558.mm.1	2,96	Unc80	JUC0100002664.mm.1	-2,02	0,033166	0,52199		
TC0100000558.mm.1	2,96	Unc80	PSR0100004591.mm.1	-2,03	0,018747	0,470733	Cassette Exon	0,17
TC0100000558.mm.1	2,96	Unc80	JUC0100002663.mm.1	-2,03	0,04423	0,55119		
TC0100000558.mm.1	2,96	Unc80	PSR0100004535.mm.1	-2,04	0,03232	0,519155	Cassette Exon	0,12
TC0100000558.mm.1	2,96	Unc80	PSR0100004558.mm.1	-2,13	0,038528	0,536634	Cassette Exon	0,16
TC0100000558.mm.1	2,96	Unc80	JUC0100002645.mm.1	-2,14	0,021467	0,48244		
TC0100000558.mm.1	2,96	Unc80	JUC0100002695.mm.1	-2,27	0,007477	0,400381		
TC0100000558.mm.1	2,96	Unc80	PSR0100004586.mm.1	-2,3	0,023098	0,489699	Cassette Exon	0,14
TC0100000558.mm.1	2,96	Unc80	PSR0100004585.mm.1	-2,36	0,009602	0,417256	Cassette Exon	0,14
TC0100000558.mm.1	2,96	Unc80	JUC0100002655.mm.1	-2,42	0,017272	0,462993		
TC0100000558.mm.1	2,96	Unc80	JUC0100002667.mm.1	-2,43	0,029782	0,511132		
TC0100000558.mm.1	2,96	Unc80	PSR0100004582.mm.1	-2,44	0,035537	0,528764	Cassette Exon	0,14

TC0100000558.mm.1	2,96	Unc80	PSR0100004520.mm.1	-2,45	0,030551	0,51337	Alternative 3' Acce	0,12
TC0100000558.mm.1	2,96	Unc80	JUC0100002661.mm.1	-2,45	0,00223	0,345773		
TC0100000558.mm.1	2,96	Unc80	PSR0100004575.mm.1	-2,49	0,04352	0,54998	Cassette Exon	0,13
TC0100000558.mm.1	2,96	Unc80	PSR0100004574.mm.1	-2,74	0,021774	0,48438	Cassette Exon	0,16
TC0100000558.mm.1	2,96	Unc80	JUC0100002696.mm.1	-3,02	0,034236	0,525168		
TC0100000558.mm.1	2,96	Unc80	PSR0100004588.mm.1	-3,04	0,010104	0,418998	Cassette Exon	0,2
TC0100000558.mm.1	2,96	Unc80	PSR0100004573.mm.1	-3,21	0,01341	0,442624	Cassette Exon	0,23
TC0100000558.mm.1	2,96	Unc80	PSR0100004529.mm.1	-3,22	0,008839	0,410293	Alternative 5' Donc	0,28
TC0300000680.mm.1	1,27	Lrba	JUC0300002335.mm.1	2,1	0,03146	0,516861		
TC0300000680.mm.1	1,27	Lrba	PSR0300004522.mm.1	-2,08	0,045572	0,554511	Intron Retention	0,25
TC0500000363.mm.1	1,42	Whsc1	PSR0500003439.mm.1	2,1	0,042541	0,547908	Alternative 3' Acce	0,17
TC0500000363.mm.1	1,42	Whsc1	JUC0500001928.mm.1	-2,75	0,043183	0,548893		
TC0500000363.mm.1	1,42	Whsc1	JUC0500001919.mm.1	-3,6	0,011676	0,429939		
TC1100000780.mm.1	-2,93	Mfap4	PSR1100006246.mm.1	2,1	0,000801	0,311765	Cassette Exon	0,15
TC0600000764.mm.1	-1,43	Tnip3	PSR0600005714.mm.1	2,1	0,015692	0,455416	Alternative 3' Acce	0,14
TC0800002188.mm.1	1,5	Tenm3	JUC0800009208.mm.1	2,1	0,031257	0,516001		
TC0800002188.mm.1	1,5	Tenm3	PSR0800016901.mm.1	-2,12	0,016815	0,460623	Alternative 5' Donc	0,14
TC1100001988.mm.1	-1,86	C1qtnf1	PSR1100018888.mm.1	2,1	0,01597	0,456885	Cassette Exon	0,14
TC1400000267.mm.1	-1,4	Asb14	PSR1400002192.mm.1	2,1	0,024595	0,494461	Alternative 5' Donc	0,14
TC1500002352.mm.1	-1,24	Olfr288	PSR1500017593.mm.1	2,1	0,030012	0,511709	Cassette Exon	0,14
TC1500001683.mm.1	1,36	Kcnk9	PSR1500012669.mm.1	2,1	0,036531	0,531352	Alternative 3' Acce	0,13
TC1400000156.mm.1	-1,84		PSR1400001193.mm.1	2,1	0,005072	0,375237	Cassette Exon	0,11
TC0100001943.mm.1	-1,03	Mybl1	PSR0100015936.mm.1	2,1	0,032665	0,520431	Cassette Exon	0,1
TC0300000172.mm.1	1,49	Tnik	PSR0300000887.mm.1	2,1	0,003576	0,356223	Cassette Exon	0,1
TC0700000820.mm.1	1,53	Pnkp	PSR0700006881.mm.1	2,1	0,000759	0,308387	Cassette Exon	0,1
TC0700000820.mm.1	1,53	Pnkp	JUC0700003415.mm.1	-2,13	0,000113	0,267977		
TC1900001547.mm.1	-1	Dnmbp	PSR1900013651.mm.1	2,1	0,01637	0,458693	Cassette Exon	0,1
TC1100001560.mm.1	-1,21	Cdk12; Mir5119	PSR1100014361.mm.1	2,1	0,034171	0,524988	Alternative 3' Acce	0,09
TC1100001011.mm.1	1,07	P2rx1	PSR1100009110.mm.1	2,1	0,009609	0,417256	Cassette Exon	0,06
TC1900001165.mm.1	-1,3	A330040F15Rik	PSR1900010801.mm.1	2,1	0,00523	0,377401	Cassette Exon	0,04
TC0700002738.mm.1	-1,16	Zfp74	PSR0700024245.mm.1	2,1	0,008036	0,40434	Alternative 3' Acce	0,03
TC1200001461.mm.1	-1,04	Greb1	JUC1200005648.mm.1	2,1	0,043015	0,54841		
TC1100003869.mm.1	-1,5	Spata32	JUC1100018884.mm.1	2,1	0,025115	0,496358		
TC0300000880.mm.1	-1,1	Selenbp1	JUC0300003537.mm.1	2,1	0,005837	0,383458		
TC1900000102.mm.1	-1,5	Batf2	PSR1900001182.mm.1	2,1	0,041935	0,545823		
TC1900000443.mm.1	1,12	C030046E11Rik	JUC1900002128.mm.1	2,1	0,00701	0,395737		
TC1500001207.mm.1	-1,44	Rai14	JUC1500005492.mm.1	2,1	0,026917	0,502535		
TC1700000375.mm.1	-1,22	Tpsg1	JUC1700001695.mm.1	2,1	0,016624	0,459985		
TC0100003371.mm.1	-1,07	Dars2	JUC0100015770.mm.1	2,1	0,026423	0,501198		
TC0200001115.mm.1	1,39	Hnrnpa3; Gm24574; Gm6	PSR0200008995.mm.1	2,1	0,049883	0,563773		
TC1600000305.mm.1	1,36	Map3k13	JUC1600001651.mm.1	2,1	0,024701	0,494838		
TC1600001398.mm.1	1,05	Sst	JUC1600006054.mm.1	2,1	0,048318	0,56048		
TC1600001130.mm.1	-1,81	Srl	JUC1600004677.mm.1	2,1	0,01055	0,422341		
TC0500001689.mm.1	1,08	Nudt1	JUC0500008419.mm.1	2,1	0,043455	0,549792		
TC0800002390.mm.1	1,16	Upf1	JUC0800009812.mm.1	2,1	0,032064	0,518469		
TC0800002390.mm.1	1,16	Upf1	JUC0800009821.mm.1	-2,14	0,00112	0,317328		
TC0700004282.mm.1	1	Eif3c	JUC0700018912.mm.1	2,1	0,041674	0,545356		
TC0500002850.mm.1	-1,14		JUC0500013672.mm.1	2,1	0,024805	0,495221		

TC0800002757.mm.1	-1,16	Slc38a7	JUC0800011589.mm.1	2,1	0,042331	0,547323		
TC0600002216.mm.1	1,08	Zfp777	JUC0600009138.mm.1	2,1	0,041172	0,544073		
TC0700002426.mm.1	1,31	Sae1	JUC0700011395.mm.1	2,1	0,037866	0,534863		
TC1100003755.mm.1	-1,23	Gm12347	JUC1100018008.mm.1	2,1	0,033407	0,522793		
TC0400001253.mm.1	-1,05	Ipp	JUC0400004859.mm.1	2,1	0,041125	0,543963		
TC1000001621.mm.1	-1,17	Mmp19	JUC1000006442.mm.1	2,1	0,013012	0,439291		
TC0400000613.mm.1	-1,18	Rad23b	JUC0400002192.mm.1	2,1	0,018522	0,469138		
TC1100002754.mm.1	-1,39	Slc22a4	JUC1100012961.mm.1	2,1	0,04109	0,543933		
TC0300002101.mm.1	-1,43		JUC0300008318.mm.1	2,1	0,045056	0,553098		
TC1100001210.mm.1	-1,15	Spaca3	JUC1100005988.mm.1	2,1	0,031926	0,518047		
TC0900003094.mm.1	-1,19	Fbxw26	JUC0900014667.mm.1	2,1	0,012036	0,432286		
TC0900003115.mm.1	-1,84	Nbeal2; Mir8107; mmu-m	PSR0900026370.mm.1	2,1	0,034662	0,526274		
TC0900003115.mm.1	-1,84	Nbeal2; Mir8107; mmu-m	JUC0900014823.mm.1	2,05	0,049499	0,563325		
TC0900003115.mm.1	-1,84	Nbeal2; Mir8107; mmu-m	JUC0900014811.mm.1	2,02	0,005876	0,383835		
TC1000000854.mm.1	-1,14	Gm15123; Gm15124	JUC1000003537.mm.1	2,1	0,013487	0,443178		
TC0400002713.mm.1	1,12	Epb4.1l4b	JUC0400011389.mm.1	2,1	0,042819	0,548097		
TC0300001641.mm.1	5,37	Fabp12	JUC0300006907.mm.1	2,09	0,004593	0,369794		
TC0300001641.mm.1	5,37	Fabp12	JUC0300006906.mm.1	-3,32	0,000744	0,307808		
TC0300001641.mm.1	5,37	Fabp12	JUC0300006913.mm.1	-3,42	0,047578	0,558743		
TC0300001641.mm.1	5,37	Fabp12	PSR0300013055.mm.1	-4,73	0,00415	0,3623	Cassette Exon	0,41
TC0300001641.mm.1	5,37	Fabp12	JUC0300006911.mm.1	-5,16	0,019294	0,473074		
TC0300001641.mm.1	5,37	Fabp12	PSR0300013054.mm.1	-5,58	0,00092	0,313363	Cassette Exon	0,38
TC0300001641.mm.1	5,37	Fabp12	PSR0300013051.mm.1	-8,38	0,002834	0,352207		
TC0300001641.mm.1	5,37	Fabp12	JUC0300006912.mm.1	-9,26	0,002099	0,342919		
TC0300001641.mm.1	5,37	Fabp12	PSR0300013052.mm.1	-9,27	0,005658	0,381922		
TC0300001641.mm.1	5,37	Fabp12	PSR0300013053.mm.1	-11,47	0,011812	0,430381		
TC0300001641.mm.1	5,37	Fabp12	JUC0300006909.mm.1	-12,9	0,002544	0,349501		
TC0300001641.mm.1	5,37	Fabp12	JUC0300006910.mm.1	-15,49	0,001797	0,336311		
TC1600002060.mm.1	2,68	Synj1	JUC1600008157.mm.1	2,09	0,043576	0,550052		
TC1600002060.mm.1	2,68	Synj1	JUC1600008177.mm.1	-2,46	0,012035	0,432286		
TC1600002060.mm.1	2,68	Synj1	JUC1600008144.mm.1	-2,47	0,011511	0,42831		
TC1600002060.mm.1	2,68	Synj1	PSR1600015840.mm.1	-2,55	0,001141	0,317344	Alternative 3' Acce	0,16
TC1600002060.mm.1	2,68	Synj1	PSR1600015842.mm.1	-2,73	0,012025	0,432286	Cassette Exon	0,12
TC1600002060.mm.1	2,68	Synj1	PSR1600015845.mm.1	-2,78	0,005635	0,381532	Alternative 5' Donc	0,01
TC1600002060.mm.1	2,68	Synj1	JUC1600008139.mm.1	-2,82	0,037665	0,53431		
TC1600002060.mm.1	2,68	Synj1	PSR1600015848.mm.1	-2,86	0,003338	0,354243		
TC1600002060.mm.1	2,68	Synj1	PSR1600015819.mm.1	-2,95	0,046474	0,556596	Alternative 3' Acce	0,32
TC1600002060.mm.1	2,68	Synj1	PSR1600015807.mm.1	-3,26	0,001258	0,322251	Cassette Exon	0,19
TC1200001148.mm.1	1,61	Gm24899; Gm26945	PSR1200008258.mm.1	2,09	0,026111	0,499897	Alternative 5' Donc	0,17
TC0200004816.mm.1	-1,08	Cst3	PSR0200041109.mm.1	2,09	0,046116	0,555792	Alternative 3' Acce	0,15
TC1800000610.mm.1	-2	Iigp1	PSR1800004320.mm.1	2,09	0,021009	0,480143	Cassette Exon	0,15
TC0X00002865.mm.1	1,02	Rps6ka6	PSR0X00018231.mm.1	2,09	0,045606	0,55464	Cassette Exon	0,14
TC0200005518.mm.1	-1,14	Znf512b	PSR0200046564.mm.1	2,09	0,028487	0,50733	Alternative 3' Acce	0,13
TC0400002609.mm.1	-1,2	Anks6	PSR0400021327.mm.1	2,09	0,04335	0,549429	Cassette Exon	0,11
TC0700003614.mm.1	-1,71	Homer2	PSR0700030796.mm.1	2,09	0,009941	0,41841	Cassette Exon	0,11
TC1100003774.mm.1	-1,03	Klhl11	PSR1100034681.mm.1	2,09	0,028419	0,507154	Cassette Exon	0,11
TC1800001640.mm.1	-1,17	Setbp1	PSR1800011818.mm.1	2,09	0,013726	0,444758	Cassette Exon	0,11
TC0400003413.mm.1	-1,14	Gm12867	PSR0400027621.mm.1	2,09	0,010555	0,422341	Cassette Exon	0,1

TC1300002617.mm.1	-1,39	Ipo11; LOC100504500; Lrr	PSR1300017814.mm.1	2,09	0,03874	0,537362	Cassette Exon	0,1
TC1400000019.mm.1	-1,07	Gm3127; Gm3685	PSR1400000333.mm.1	2,09	0,032238	0,51899	Cassette Exon	0,1
TC0200002696.mm.1	1,06	Stx16	JUC0200011177.mm.1	2,09	0,002406	0,348653		
TC1300000327.mm.1	1,17	Gm11368	JUC1300001004.mm.1	2,09	0,036418	0,530922		
TC0100002449.mm.1	-1,17	Gm11608	JUC0100011295.mm.1	2,09	0,031844	0,517896		
TC1700001482.mm.1	-1,14	Dact2	JUC1700007212.mm.1	2,09	0,049883	0,563773		
TC0100000878.mm.1	1,05	Cops8	JUC0100004225.mm.1	2,09	0,020643	0,478859		
TC1900000980.mm.1	-1,06	Ehbp1l1	JUC1900004861.mm.1	2,09	0,00209	0,342919		
TC1900001551.mm.1	-1,37	Erlin1	JUC1900007596.mm.1	2,09	0,011039	0,425682		
TC0200001749.mm.1	-1,12	Ivd	JUC0200006495.mm.1	2,09	0,002714	0,349612		
TC1500001300.mm.1	1,23	Fam105a	JUC1500005700.mm.1	2,09	0,018369	0,468484		
TC1700000402.mm.1	1,69	Tmem8	JUC1700001838.mm.1	2,09	0,033337	0,522474		
TC1700000402.mm.1	1,69	Tmem8	JUC1700001835.mm.1	-2,08	0,010839	0,424161		
TC1700000472.mm.1	1,08	Zfp523	JUC1700002207.mm.1	2,09	0,005751	0,383101		
TC1600002181.mm.1	1,01	Dgcr2; Gm20518	JUC1600005551.mm.1	2,09	0,013523	0,443433		
TC0800001112.mm.1	-1,52	Cpne2	JUC0800004618.mm.1	2,09	0,042036	0,546173		
TC0700004522.mm.1	-1,61	Rnh1	JUC0700020018.mm.1	2,09	0,039024	0,53816		
TC0500002799.mm.1	1,07	Paqr3	JUC0500013395.mm.1	2,09	0,035966	0,529457		
TC0800000411.mm.1	-1,08		JUC0800001623.mm.1	2,09	0,021591	0,483253		
TC0800002525.mm.1	-1,65	Hhip	JUC0800010445.mm.1	2,09	0,003124	0,353892		
TC0500003496.mm.1	-1,11	Micall2	JUC0500017381.mm.1	2,09	0,000147	0,272178		
TC0700001343.mm.1	1,09	Nars2	JUC0700005862.mm.1	2,09	0,031412	0,516527		
TC0700002636.mm.1	-1,34	Ceacam2	JUC0700012108.mm.1	2,09	0,026774	0,502153		
TC0300003182.mm.1	1,02	Fpgt	JUC0300013097.mm.1	2,09	0,036517	0,531352		
TC1100003349.mm.1	-1,12		JUC1100016105.mm.1	2,09	0,016143	0,457937		
TC1100001893.mm.1	-1	Kctd2	JUC1100009255.mm.1	2,09	0,00707	0,396677		
TC0900003011.mm.1	-1,25	Parp3	JUC0900014035.mm.1	2,09	0,030951	0,514626		
TC0800001232.mm.1	1,42	Nrn1l; Edc4	JUC0800005324.mm.1	2,08	0,021602	0,483387		
TC0800001232.mm.1	1,42	Nrn1l; Edc4	PSR0800009861.mm.1	-2,04	0,024339	0,49362	Cassette Exon	0,16
TC0800001232.mm.1	1,42	Nrn1l; Edc4	JUC0800005353.mm.1	-2,04	0,002369	0,34829		
TC0800001232.mm.1	1,42	Nrn1l; Edc4	JUC0800005350.mm.1	-2,07	0,002942	0,352501		
TC0800001232.mm.1	1,42	Nrn1l; Edc4	PSR0800009867.mm.1	-2,25	0,015693	0,455416	Cassette Exon	0,1
TC0800001232.mm.1	1,42	Nrn1l; Edc4	PSR0800009863.mm.1	-2,31	0,001732	0,335996	Cassette Exon	0,17
TC0800001232.mm.1	1,42	Nrn1l; Edc4	PSR0800009834.mm.1	-3,25	0,045888	0,555077	Intron Retention	0,52
TC0100000526.mm.1	-1,07	Fastkd2	PSR0100004267.mm.1	2,08	0,047006	0,557708	Alternative 5' Donc	0,18
TC0X00000075.mm.1	-1,24	Slc35a2	PSR0X00000582.mm.1	2,08	0,02485	0,495511	Alternative 5' Donc	0,18
TC0100003338.mm.1	1,54	Brinp2	JUC0100015625.mm.1	2,08	0,04097	0,543536		
TC0100003338.mm.1	1,54	Brinp2	PSR0100027332.mm.1	2,05	0,01415	0,447466	Cassette Exon	0,15
TC0400000464.mm.1	-1,13	Melk	PSR0400003265.mm.1	2,08	0,037441	0,533839	Cassette Exon	0,13
TC0800001258.mm.1	-1,12	Cirh1a	PSR0800010164.mm.1	2,08	0,02106	0,480272	Cassette Exon	0,13
TC1100000961.mm.1	-1,06	Rnf167	PSR1100008563.mm.1	2,08	0,027348	0,503766		
TC1100000961.mm.1	-1,06	Rnf167	PSR1100008594.mm.1	-2,12	0,015674	0,455416	Alternative 5' Donc	0,13
TC1100001577.mm.1	-1,41	Gsdma	PSR1100014632.mm.1	2,08	0,022383	0,486468	Cassette Exon	0,13
TC1100003109.mm.1	-1,16	Spag7	PSR1100028713.mm.1	2,08	0,027641	0,504658	Alternative 5' Donc	0,13
TC0900002204.mm.1	1,58	Cep164	JUC0900010372.mm.1	2,08	0,001943	0,340349		
TC0900002204.mm.1	1,58	Cep164	PSR0900018371.mm.1	-2,13	0,04907	0,562033	Alternative 5' Donc	0,11
TC1400001896.mm.1	1,3	Txndc16	PSR1400014319.mm.1	2,08	0,030006	0,511659	Cassette Exon	0,11
TC1400001896.mm.1	1,3	Txndc16	JUC1400007843.mm.1	-2,58	0,003091	0,353892		

TC0100003856.mm.1	-1,37	Cr2	PSR0100031572.mm.1	2,08	0,009437	0,416101	Cassette Exon	0,1
TC0400001266.mm.1	-1,21	Mutyh	PSR0400009467.mm.1	2,08	0,049388	0,562923	Cassette Exon	0,1
TC1400001900.mm.1	1,03	Gnpnat1	PSR1400014363.mm.1	2,08	0,029758	0,511132	Cassette Exon	0,09
TC0300002688.mm.1	-1,11	Cept1	PSR0300020969.mm.1	2,08	0,000684	0,304511	Cassette Exon	0,05
TC1800000103.mm.1	-1,19	Lama3	JUC1800000391.mm.1	2,08	0,032176	0,518779		
TC1800000103.mm.1	-1,19	Lama3	JUC1800000410.mm.1	-2,19	0,019369	0,473624		
TC0100003155.mm.1	-1,29	Nr5a2	JUC0100014586.mm.1	2,08	0,018758	0,470733		
TC0100002764.mm.1	1,01	Ngef	JUC0100012642.mm.1	2,08	0,00678	0,393554		
TC1900001026.mm.1	1,15	Fkbp2	JUC1900005232.mm.1	2,08	0,000895	0,313363		
TC0100000387.mm.1	-1,41	Osgepl1	PSR0100003012.mm.1	2,08	0,028336	0,506831		
TC1500000180.mm.1	-1,15	Dnah5	JUC1500000809.mm.1	2,08	0,011802	0,430351		
TC1500000180.mm.1	-1,15	Dnah5	JUC1500000796.mm.1	2,03	0,011834	0,43053		
TC1500000932.mm.1	-1,65	Irak4	JUC1500004200.mm.1	2,08	0,014552	0,44963		
TC0100003893.mm.1	1,28	Ahctf1	JUC0100017048.mm.1	2,08	0,004198	0,363134		
TC0100003893.mm.1	1,28	Ahctf1	JUC0100017060.mm.1	-2,12	0,023272	0,49004		
TC1600000938.mm.1	1,78	Jam2	JUC1600003844.mm.1	2,08	0,024108	0,49322		
TC1600000938.mm.1	1,78	Jam2	JUC1600003842.mm.1	-2,18	0,001471	0,329052		
TC0800001477.mm.1	1,07	Banp	JUC0800006551.mm.1	2,08	0,040697	0,542909		
TC0900000631.mm.1	-1,56		JUC0900002373.mm.1	2,08	0,003634	0,3566		
TC0800002935.mm.1	1,1	Ddx19b	JUC0800012345.mm.1	2,08	0,015905	0,456586		
TC0500001146.mm.1	-1,02	Galnt9	JUC0500005389.mm.1	2,08	0,002328	0,346856		
TC0700000509.mm.1	1,04	Sars2	JUC0700002067.mm.1	2,08	0,021899	0,484824		
TC0500003247.mm.1	1,07	Abcb9	JUC0500015912.mm.1	2,08	0,011261	0,426787		
TC0700003983.mm.1	1,01	Trim3	JUC0700017477.mm.1	2,08	0,041506	0,544746		
TC0700003403.mm.1	-1,08	Chrna7	JUC0700015330.mm.1	2,08	0,008517	0,407576		
TC0700001769.mm.1	1,44	Tnrc6a	JUC0700007916.mm.1	2,08	0,030894	0,514574		
TC0700002183.mm.1	-1,54	Gm15510; LOC102640192	JUC0700010606.mm.1	2,08	0,035673	0,528974		
TC0700002646.mm.1	-1,13	Cyp2s1	JUC0700012185.mm.1	2,08	0,02556	0,497808		
TC0600000201.mm.1	-1,51	Asb15	JUC0600000624.mm.1	2,08	0,007089	0,396897		
TC1000001307.mm.1	-1,18	Caps2	JUC1000005293.mm.1	2,08	0,040154	0,541609		
TC0400000869.mm.1	-1,25	Dennd4c	JUC0400003028.mm.1	2,08	0,039849	0,540922		
TC1100002436.mm.1	-1,38	4930505A04Rik	JUC1100011854.mm.1	2,08	0,023012	0,489143		
TC1100002473.mm.1	-1,06	Nprl3	JUC1100011977.mm.1	2,08	0,016158	0,457969		
TC0400001791.mm.1	-1,03	Zbtb17	JUC0400007841.mm.1	2,08	0,047276	0,558232		
TC1000003218.mm.1	-1,11	Atp5d	JUC1000003419.mm.1	2,08	0,003944	0,359922		
TC1100000001.mm.1	1,44	Pisd-ps1	JUC1100000001.mm.1	2,07	0,03511	0,527632		
TC1100000001.mm.1	1,44	Pisd-ps1	PSR1100000050.mm.1	-2,26	0,011552	0,428841	Cassette Exon	0,24
TC1100000001.mm.1	1,44	Pisd-ps1	JUC1100000010.mm.1	-3,3	0,017459	0,463886		
TC0200001854.mm.1	-2,83	AA467197; Mir147	PSR0200014433.mm.1	2,07	0,003423	0,355303	Intron Retention	0,2
TC0X00000396.mm.1	2,2	Xiap	JUC0X00001306.mm.1	2,07	0,046144	0,555804		
TC0X00000396.mm.1	2,2	Xiap	JUC0X00001303.mm.1	-2,13	0,036868	0,532257		
TC0X00000396.mm.1	2,2	Xiap	JUC0X00001302.mm.1	-2,39	0,010255	0,420147		
TC0X00000396.mm.1	2,2	Xiap	JUC0X00001315.mm.1	-2,96	0,038653	0,537102		
TC0X00000396.mm.1	2,2	Xiap	PSR0X00002631.mm.1	-2,99	0,043358	0,549429	Alternative 3' Acce	0,17
TC0X00000396.mm.1	2,2	Xiap	PSR0X00002626.mm.1	-3,18	0,00838	0,4065	Alternative 3' Acce	0,19
TC0X00000396.mm.1	2,2	Xiap	JUC0X00001313.mm.1	-3,29	0,008304	0,406122		
TC0600003302.mm.1	-1,06	Lrp6	PSR0600025911.mm.1	2,07	0,041104	0,543933	Alternative 3' Acce	0,18
TC1600001136.mm.1	1,02	Cdip1	PSR1600009077.mm.1	2,07	0,012242	0,433659	Intron Retention	0,18

TC0600001171.mm.1	-1,75	Chl1	PSR0600009007.mm.1	2,07	0,009887	0,418122	Alternative 3' Acce	0,17
TC1400000296.mm.1	-1,21		PSR1400002387.mm.1	2,07	0,018043	0,467148	Alternative 3' Acce	0,17
TC1500001214.mm.1	-1,13	Tars	PSR1500009773.mm.1	2,07	0,029653	0,510799	Alternative 3' Acce	0,17
TC0X00001014.mm.1	-1,44		PSR0X00006798.mm.1	2,07	0,033718	0,523549	Alternative 5' Donc	0,16
TC0100000260.mm.1	-1,07	Unc50	PSR0100001855.mm.1	2,07	0,000362	0,290942	Alternative 5' Donc	0,14
TC0X00001667.mm.1	-1,23	Figf	PSR0X00010664.mm.1	2,07	0,008457	0,406611	Cassette Exon	0,14
TC1700002642.mm.1	-2,94		PSR1700023968.mm.1	2,07	0,001141	0,317344	Cassette Exon	0,14
TC0100003725.mm.1	1,02	Mia3	PSR0100030562.mm.1	2,07	0,001519	0,330268	Cassette Exon	0,13
TC1500002221.mm.1	-1,11	Cers5	PSR1500018215.mm.1	2,07	0,049698	0,563531	Cassette Exon	0,13
TC0100001227.mm.1	1,69	Cyb5r1	JUC0100005787.mm.1	2,07	0,029511	0,510139		
TC0100001227.mm.1	1,69	Cyb5r1	PSR0100010069.mm.1	-2,12	0,032946	0,52122	Cassette Exon	0,12
TC0200000222.mm.1	-1,23	Il15ra	PSR0200000856.mm.1	2,07	0,020951	0,479982	Alternative 5' Donc	0,12
TC1200000098.mm.1	-1,36	Fam49a	PSR1200000959.mm.1	2,07	0,000707	0,306015	Cassette Exon	0,11
TC0100003233.mm.1	-1,27	Pla2g4a	PSR0100026157.mm.1	2,07	0,013079	0,4396	Cassette Exon	0,1
TC0200001931.mm.1	-1,46		PSR0200015165.mm.1	2,07	0,019643	0,474876	Cassette Exon	0,1
TC0600000923.mm.1	-1,29	Rtkn	PSR0600006958.mm.1	2,07	0,025335	0,497306	Cassette Exon	0,1
TC1200000719.mm.1	-1,05	Rad51b	PSR1200005136.mm.1	2,07	0,044077	0,550897	Cassette Exon	0,1
TC1300000176.mm.1	-1,53	Hist1h4j; Hist1h4m; Hist1	PSR1300001178.mm.1	2,07	0,032193	0,518843	Cassette Exon	0,1
TC1500000420.mm.1	-1,26	Trmt12	PSR1500002988.mm.1	2,07	0,000137	0,270987	Cassette Exon	0,1
TC0100002997.mm.1	-1,32	Lypd1	PSR0100024073.mm.1	2,07	0,024713	0,494838	Cassette Exon	0,07
TC1400001958.mm.1	-1,12	Exoc5	JUC1400008070.mm.1	2,07	0,019996	0,476227		
TC0200002801.mm.1	-1,49	Gid8	JUC0200011630.mm.1	2,07	0,03484	0,526823		
TC1300001242.mm.1	-1,46	Plk2	JUC1300004076.mm.1	2,07	0,001407	0,326083		
TC1300002211.mm.1	-1,16	Nlrp4f	JUC1300007460.mm.1	2,07	0,017288	0,463143		
TC1300002270.mm.1	1,19	Adcy2	JUC1300007742.mm.1	2,07	0,017434	0,463827		
TC1300002270.mm.1	1,19	Adcy2	JUC1300007740.mm.1	-2,11	0,01065	0,422925		
TC1800000130.mm.1	-1,12		JUC1800000544.mm.1	2,07	0,014062	0,446724		
TC1700001747.mm.1	-1,25	Taf11	JUC1700008681.mm.1	2,07	0,026551	0,501435		
TC0100000431.mm.1	-1,19	Aox2	JUC0100002037.mm.1	2,07	0,027405	0,503961		
TC1800001072.mm.1	1,06	Zscan30	JUC1800004405.mm.1	2,07	0,004397	0,366253		
TC1600000298.mm.1	-1,57	Ephb3	JUC1600001555.mm.1	2,07	0,019788	0,475149		
TC0200000417.mm.1	1,17	Abca2	JUC0200001122.mm.1	2,07	0,04675	0,556858		
TC0200000417.mm.1	1,17	Abca2	JUC0200001123.mm.1	-2,25	0,031698	0,517443		
TC0500001695.mm.1	1,24	Lfng	JUC0500008451.mm.1	2,07	0,01742	0,463792		
TC0500002272.mm.1	-1	Evc	JUC0500011402.mm.1	2,07	0,002286	0,34605		
TC0500000688.mm.1	-1,1	Cwh43	JUC0500003328.mm.1	2,07	0,005321	0,378059		
TC0500000212.mm.1	-1,03	Nos3	JUC0500000880.mm.1	2,07	0,040093	0,541422		
TC0500000212.mm.1	-1,03	Nos3	JUC0500000875.mm.1	-2,7	0,032874	0,521141		
TC0700001052.mm.1	-1,14		JUC0700004858.mm.1	2,07	0,01729	0,463143		
TC1100002224.mm.1	-1,24	Fignl1	JUC1100011263.mm.1	2,07	0,025424	0,497527		
TC0400003343.mm.1	-1,26	Nasp	JUC0400013933.mm.1	2,07	0,015007	0,452083		
TC0X00000058.mm.1	-1,08	Foxp3; Ppp1r3fos; 493052	JUC0X00000084.mm.1	2,07	0,017117	0,46232		
TC0400003542.mm.1	-1,03	ago-01	JUC0400015061.mm.1	2,07	0,028908	0,50858		
TC0900002883.mm.1	-1,04	Rasa2	JUC0900013276.mm.1	2,07	0,031871	0,51793		
TC0400002527.mm.1	-1,14	Arhgef39	JUC0400010657.mm.1	2,07	0,004922	0,373082		
TC0100001712.mm.1	1,04	Lin9	PSR0100014210.mm.1	2,06	0,004489	0,367995	Cassette Exon	0,1
TC0100001712.mm.1	1,04	Lin9	JUC0100008066.mm.1	-2,15	0,010503	0,42211		
TC0100001712.mm.1	1,04	Lin9	PSR0100014219.mm.1	-2,94	0,02635	0,50085	Cassette Exon	0,28

TC0100001712.mm.1	1,04	Lin9	JUC0100008064.mm.1	-3,66	0,005242	0,377642		
TC0900001304.mm.1	-1,15	5830418P13Rik	JUC0900005586.mm.1	2,06	0,039771	0,540537		
TC0900001304.mm.1	-1,15	5830418P13Rik	PSR0900010164.mm.1	-2,27	0,02053	0,478295	Cassette Exon	0,2
TC0X00002671.mm.1	-1	Las1l	PSR0X00016733.mm.1	2,06	0,048437	0,560744	Intron Retention	0,2
TC1000001110.mm.1	1,12	Ikbip	PSR1000008445.mm.1	2,06	0,024043	0,493026	Alternative 5' Donc	0,15
TC0600000232.mm.1	-1,5	Flnc	JUC0600000799.mm.1	2,06	0,019427	0,473969		
TC0600000232.mm.1	-1,5	Flnc	PSR0600001579.mm.1	-2,25	0,020006	0,476344	Cassette Exon	0,14
TC0X00002434.mm.1	-1,11	Idh3g	PSR0X00015089.mm.1	2,06	0,041268	0,544166	Intron Retention	0,14
TC0X00002434.mm.1	-1,11	Idh3g	PSR0X00015101.mm.1	2,06	0,036083	0,529712	Intron Retention	0,14
TC1100002003.mm.1	-1,75	Rnf213	JUC1100010005.mm.1	2,06	0,010258	0,420147		
TC1100002003.mm.1	-1,75	Rnf213	PSR1100019174.mm.1	-2,37	0,043019	0,54841	Cassette Exon	0,14
TC1500000848.mm.1	-1,38	B230214G05Rik	PSR1500006641.mm.1	2,06	0,038855	0,537829	Cassette Exon	0,13
TC0700002057.mm.1	1,98	Ap2a2	JUC0700009712.mm.1	2,06	0,030514	0,51337		
TC0700002057.mm.1	1,98	Ap2a2	PSR0700018334.mm.1	-2,02	0,018284	0,468049	Cassette Exon	0,12
TC0700002057.mm.1	1,98	Ap2a2	PSR0700018333.mm.1	-2,08	0,02248	0,48709	Cassette Exon	0,12
TC1300000596.mm.1	3,41	Nxnl2	PSR1300003763.mm.1	2,06	0,011317	0,427133	Cassette Exon	0,11
TC1300000596.mm.1	3,41	Nxnl2	PSR1300003762.mm.1	-2,06	0,011317	0,427133	Cassette Exon	0,08
TC1600001194.mm.1	1,72	Tvp23a	JUC1600004947.mm.1	2,06	0,015017	0,452101		
TC1600001194.mm.1	1,72	Tvp23a	PSR1600009508.mm.1	-2,06	0,01432	0,448086	Alternative 5' Donc	0,03
TC1600001194.mm.1	1,72	Tvp23a	PSR1600009509.mm.1	-2,08	0,016885	0,460797	Intron Retention	0,11
TC1600001194.mm.1	1,72	Tvp23a	PSR1600009496.mm.1	-2,11	0,011318	0,427133	Cassette Exon	0,03
TC1600001194.mm.1	1,72	Tvp23a	JUC1600004946.mm.1	-2,12	0,009936	0,41841		
TC1600001194.mm.1	1,72	Tvp23a	PSR1600009507.mm.1	-2,27	0,017384	0,463704	Alternative 5' Donc	0,03
TC1600001194.mm.1	1,72	Tvp23a	JUC1600004954.mm.1	-2,34	0,015848	0,456345		
TC1600001194.mm.1	1,72	Tvp23a	JUC1600004956.mm.1	-3,16	0,035319	0,528381		
TC1600001194.mm.1	1,72	Tvp23a	JUC1600004951.mm.1	-3,51	0,022786	0,488142		
TC1600001194.mm.1	1,72	Tvp23a	JUC1600004957.mm.1	-5,08	0,022733	0,487829		
TC0700000808.mm.1	-1,22	Fam71e1	PSR0700006711.mm.1	2,06	0,010962	0,425117	Cassette Exon	0,1
TC0700000983.mm.1	1,31	BC046251	PSR0700008996.mm.1	2,06	0,015106	0,452457	Cassette Exon	0,1
TC0700000983.mm.1	1,31	BC046251	JUC0700004593.mm.1	-2,49	0,028905	0,50858		
TC0900003241.mm.1	-1,25	Myd88	PSR0900027283.mm.1	2,06	0,012468	0,435315	Alternative 5' Donc	0,1
TC1200002025.mm.1	1,16	Med6	PSR1200013964.mm.1	2,06	0,000878	0,311909	Cassette Exon	0,1
TC1300000637.mm.1	-1	Eif4e1b	PSR1300004119.mm.1	2,06	0,035222	0,528366	Cassette Exon	0,1
TC1500001403.mm.1	1,25	Ubr5	PSR1500010915.mm.1	2,06	0,029018	0,508842	Cassette Exon	0,1
TC1900000900.mm.1	-1,83		PSR1900007806.mm.1	2,06	0,037475	0,533968	Cassette Exon	0,07
TC1400001471.mm.1	1,12	Psmd6	JUC1400005964.mm.1	2,06	0,011122	0,426		
TC1400000813.mm.1	1,5	Zmym2	JUC1400003604.mm.1	2,06	0,01142	0,427656		
TC0300000264.mm.1	-1,11	Acad9	JUC0300000882.mm.1	2,06	0,00476	0,371039		
TC0200004419.mm.1	-1,1	A430105I19Rik	JUC0200018890.mm.1	2,06	0,03524	0,52837		
TC1300002184.mm.1	-1,08	Fancc	JUC1300007283.mm.1	2,06	0,037342	0,533573		
TC1300002249.mm.1	-1,2	Zfp874a	JUC1300007648.mm.1	2,06	0,048329	0,560488		
TC1300002249.mm.1	-1,2	Zfp874a	JUC1300007649.mm.1	-4,06	0,00548	0,379939		
TC1300000045.mm.1	-1,37	Idi1	JUC1300000159.mm.1	2,06	0,046779	0,557017		
TC0100000846.mm.1	-1,24	Trpm8	JUC0100004131.mm.1	2,06	0,034999	0,527405		
TC0100000846.mm.1	-1,24	Trpm8	JUC0100004135.mm.1	-2,07	0,001401	0,325997		
TC1900001498.mm.1	-1,54	Blnk	JUC1900007214.mm.1	2,06	0,02911	0,509179		
TC0100000999.mm.1	1,08	Cdh20	JUC0100004788.mm.1	2,06	0,02969	0,510949		
TC0100000351.mm.1	1	Wdr75	JUC0100001489.mm.1	2,06	0,039123	0,538475		

TC0100000497.mm.1	-1,24	Pard3b	JUC0100002386.mm.1	2,06	0,001048	0,316361		
TC1900000543.mm.1	-1,36	Cep55	JUC1900002570.mm.1	2,06	0,013062	0,439579		
TC0200001355.mm.1	-1,2	Agbl2	JUC0200005187.mm.1	2,06	0,010751	0,423812		
TC1500002067.mm.1	-1,65	Cpne8	JUC1500009486.mm.1	2,06	0,001697	0,335395		
TC1500001089.mm.1	1,36	Igfbp6	JUC1500004917.mm.1	2,06	0,037276	0,533572		
TC0200001133.mm.1	-1,32	Dfnb59	JUC0200004662.mm.1	2,06	0,031745	0,517489		
TC1600000746.mm.1	-1,69	Abi3bp	JUC1600003445.mm.1	2,06	0,022282	0,486046		
TC0800001748.mm.1	-1,06	Tdrp	JUC0800007714.mm.1	2,06	0,039724	0,540494		
TC0900000892.mm.1	1,57	Rora	PSR0900006979.mm.1	2,06	0,023142	0,489776		
TC0900000970.mm.1	-1,23	Rfx7	PSR0900007479.mm.1	2,06	0,018727	0,470733		
TC0900000117.mm.1	-1,4	Taf1d; Mir7650; Gm2579	PSR0900000849.mm.1	2,06	0,006532	0,391754		
TC0900000117.mm.1	-1,4	Taf1d; Mir7650; Gm2579	PSR0900000843.mm.1	-55,38	0,00999	0,418456		
TC0700000872.mm.1	-1,07	Otog	JUC0700003901.mm.1	2,06	0,016842	0,460749		
TC0600003396.mm.1	-1,35	1700060C16Rik	JUC0600014035.mm.1	2,06	0,008609	0,408415		
TC0500003741.mm.1	1,07	Gpc2	JUC0500017267.mm.1	2,06	0,000045	0,24627		
TC0300002932.mm.1	-1,03	Egf	JUC0300012107.mm.1	2,06	0,018571	0,469618		
TC0300002056.mm.1	-1,43	Igsf10	JUC0300008139.mm.1	2,06	0,044204	0,551186		
TC1100002092.mm.1	-1,32	Smtn	JUC1100010616.mm.1	2,06	0,044823	0,552553		
TC0400003549.mm.1	1,31	Ncdn	JUC0400015101.mm.1	2,06	0,017476	0,463933		
TC0X00001178.mm.1	-1,02	Pcdh11x	PSR0X00007526.mm.1	2,06	0,012396	0,434939		
TC0X00001178.mm.1	-1,02	Pcdh11x	JUC0X00003691.mm.1	-3,59	0,017226	0,462633		
TC0900002369.mm.1	3,82	Scamp5	JUC0900011197.mm.1	2,06	0,015862	0,456449		
TC0900002369.mm.1	3,82	Scamp5	JUC0900011195.mm.1	-2,01	0,005527	0,380518		
TC0900002369.mm.1	3,82	Scamp5	JUC0900011200.mm.1	-2,36	0,027626	0,504621		
TC0900002369.mm.1	3,82	Scamp5	PSR0900019852.mm.1	-3,31	0,022239	0,485896		
TC1000000794.mm.1	-1,05	Slc1a6	JUC1000003140.mm.1	2,06	0,03883	0,537696		
TC0500002623.mm.1	2,13	Kdr	JUC0500012623.mm.1	2,05	0,038179	0,535805		
TC0500002623.mm.1	2,13	Kdr	JUC0500012621.mm.1	-2,19	0,007078	0,396891		
TC0500002623.mm.1	2,13	Kdr	PSR0500023224.mm.1	-4,48	0,010988	0,425397	Alternative 5' Donc	0,47
TC1100000417.mm.1	2	Gabrb2	PSR1100003267.mm.1	2,05	0,024172	0,49329	Alternative 5' Donc	0,08
TC1100000417.mm.1	2	Gabrb2	JUC1100001713.mm.1	-2,96	0,028597	0,507685		
TC1100000417.mm.1	2	Gabrb2	PSR1100003255.mm.1	-3,2	0,00749	0,400686	Alternative 5' Donc	0,29
TC0300002122.mm.1	1,52	Ift80	PSR0300016009.mm.1	2,05	0,039778	0,540568	Cassette Exon	0,09
TC0300002122.mm.1	1,52	Ift80	PSR0300016020.mm.1	-2,56	0,0087	0,409002	Cassette Exon	0,16
TC0300002122.mm.1	1,52	Ift80	PSR0300016035.mm.1	-3,94	0,036655	0,531675	Intron Retention	0,27
TC0400002789.mm.1	1,99	Wdr31	PSR0400022882.mm.1	2,05	0,007304	0,398847	Cassette Exon	0,1
TC0400002789.mm.1	1,99	Wdr31	PSR0400022887.mm.1	-2,14	0,006982	0,395722	Alternative 5' Donc	0,14
TC0400002789.mm.1	1,99	Wdr31	PSR0400022891.mm.1	-2,15	0,039573	0,539809	Alternative 3' Acce	0,17
TC0400002789.mm.1	1,99	Wdr31	PSR0400022895.mm.1	-2,36	0,015179	0,452699	Alternative 3' Acce	0,27
TC0400002789.mm.1	1,99	Wdr31	PSR0400022897.mm.1	-2,49	0,010815	0,424055	Alternative 3' Acce	0,27
TC0400002789.mm.1	1,99	Wdr31	PSR0400022894.mm.1	-2,61	0,010356	0,421055	Alternative 5' Donc	0,23
TC0400002789.mm.1	1,99	Wdr31	JUC0400011915.mm.1	-2,72	0,011256	0,426718		
TC0400002789.mm.1	1,99	Wdr31	JUC0400011914.mm.1	-3,04	0,013803	0,445022		
TC0400002789.mm.1	1,99	Wdr31	JUC0400011912.mm.1	-3,58	0,037498	0,533968		
TC1800001000.mm.1	1,21	Osbpl1a	PSR1800007445.mm.1	2,05	0,04889	0,561732	Intron Retention	0,26
TC1000000853.mm.1	2,3	Plk5	PSR1000006517.mm.1	2,05	0,000318	0,288663	Cassette Exon	0,08
TC1000000853.mm.1	2,3	Plk5	JUC1000003522.mm.1	-2,14	0,016678	0,460085		
TC1000000853.mm.1	2,3	Plk5	PSR1000006522.mm.1	-2,15	0,004593	0,369794	Intron Retention	0,25

TC1600000461.mm.1	1,44	Lmln	PSR1600004368.mm.1	2,05	0,009191	0,413763	Cassette Exon	0,13
TC1600000461.mm.1	1,44	Lmln	PSR1600004380.mm.1	-2,53	0,006345	0,389463	Alternative 5' Donc	0,21
TC1100001439.mm.1	-1,39	Chad	PSR1100013357.mm.1	2,05	0,010276	0,420305	Alternative 3' Acce	0,17
TC1100001439.mm.1	-1,39	Chad	JUC1100006994.mm.1	-2,25	0,021174	0,480978		
TC1100001439.mm.1	-1,39	Chad	JUC1100006992.mm.1	-3,01	0,049058	0,562009		
TC1600001395.mm.1	-1,32	Masp1	PSR1600011620.mm.1	2,05	0,010921	0,424705	Alternative 3' Acce	0,17
TC1600001395.mm.1	-1,32	Masp1	PSR1600011617.mm.1	-2,26	0,008273	0,405976	Cassette Exon	0,12
TC0200004791.mm.1	1	Nkx2-2	PSR0200040975.mm.1	2,05	0,011455	0,42789	Alternative 5' Donc	0,16
TC0300000095.mm.1	-3,19	Car3	PSR0300000415.mm.1	2,05	0,013367	0,442169	Alternative 3' Acce	0,16
TC0500001865.mm.1	-1,04	Brca2	PSR0500017196.mm.1	2,05	0,008791	0,409822	Alternative 3' Acce	0,16
TC0X00001639.mm.1	-1,21	Rai2	PSR0X00010475.mm.1	2,05	0,026935	0,50263	Alternative 3' Acce	0,15
TC1900001278.mm.1	-1,59	Pip5k1b	PSR1900011552.mm.1	2,05	0,028872	0,508574	Alternative 5' Donc	0,15
TC0700000883.mm.1	-1,68	Gm9999	PSR0700007956.mm.1	2,05	0,000043	0,24627	Alternative 3' Acce	0,13
TC0700001581.mm.1	-1,35	Ppfibp2	PSR0700013235.mm.1	2,05	0,007686	0,402552	Cassette Exon	0,13
TC0700004617.mm.1	-1	Adck4	PSR0700003578.mm.1	2,05	0,036391	0,530857	Cassette Exon	0,12
TC0900000285.mm.1	1,03	Arhgap32	PSR0900002194.mm.1	2,05	0,021091	0,480371	Cassette Exon	0,11
TC0100000039.mm.1	-1,73	Adhfe1	PSR0100000294.mm.1	2,05	0,001015	0,316361	Alternative 3' Acce	0,1
TC0200002045.mm.1	-1,11	Gm14102	PSR0200016301.mm.1	2,05	0,013902	0,445922	Cassette Exon	0,09
TC0200002045.mm.1	-1,11	Gm14102	PSR0200016300.mm.1	-2,05	0,013902	0,445922	Cassette Exon	0,1
TC0400001222.mm.1	-1,68	Gm12836	PSR0400008921.mm.1	2,05	0,037117	0,532942	Cassette Exon	0,1
TC0400004069.mm.1	-1,41	1700045H11Rik	PSR0400033812.mm.1	2,05	0,021712	0,484104	Cassette Exon	0,1
TC0400000644.mm.1	-1,2	Musk	PSR0400004578.mm.1	2,05	0,018151	0,467399	Cassette Exon	0,09
TC0400000644.mm.1	-1,2	Musk	JUC0400002253.mm.1	-2,07	0,049429	0,563094		
TC0400000644.mm.1	-1,2	Musk	JUC0400002257.mm.1	-2,27	0,034244	0,525168		
TC0700004483.mm.1	-1,02	9330101J02Rik	PSR0700037603.mm.1	2,05	0,017344	0,46353	Cassette Exon	0,09
TC0800000011.mm.1	1,13	Pnpla6	JUC0800000088.mm.1	2,05	0,033875	0,524132		
TC0800000011.mm.1	1,13	Pnpla6	PSR0800000157.mm.1	-2,01	0,027342	0,503766	Cassette Exon	0,09
TC0X00002794.mm.1	-1,04	C77370	PSR0X00017628.mm.1	2,05	0,007743	0,402774	Cassette Exon	0,09
TC1900001414.mm.1	-1,4	Ankrd22	PSR1900012389.mm.1	2,05	0,031517	0,516965	Cassette Exon	0,07
TC1900001414.mm.1	-1,4	Ankrd22	PSR1900012390.mm.1	2,05	0,031517	0,516965	Cassette Exon	0,07
TC1400000782.mm.1	1,18	Dcaf11	PSR1400006203.mm.1	2,05	0,013497	0,443238		
TC0200003232.mm.1	-1,09	Cel	JUC0200013773.mm.1	2,05	0,011932	0,431703		
TC0200003232.mm.1	-1,09	Cel	JUC0200013772.mm.1	-2,67	0,005501	0,379939		
TC0200005184.mm.1	-1,18	Ptgis	JUC0200022900.mm.1	2,05	0,001237	0,322251		
TC0200004459.mm.1	-1,34	Tmem87a	JUC0200019255.mm.1	2,05	0,021902	0,484824		
TC0200003689.mm.1	1,17	Stk39	JUC0200016047.mm.1	2,05	0,009066	0,412827		
TC0100002411.mm.1	-1,04	Wdr12	JUC0100011193.mm.1	2,05	0,01973	0,474922		
TC1700002267.mm.1	-1,24	Satb1	PSR1700021273.mm.1	2,05	0,015313	0,453561		
TC0100002203.mm.1	1,04	Coa5	JUC0100010037.mm.1	2,05	0,001829	0,337273		
TC0100000845.mm.1	-1,19	Mroh2a	JUC0100004081.mm.1	2,05	0,002988	0,352663		
TC1900000718.mm.1	1,2	Pdcd11	JUC1900003453.mm.1	2,05	0,034499	0,525929		
TC1900000756.mm.1	-1,3	Dusp5	JUC1900003612.mm.1	2,05	0,041262	0,544166		
TC0800000650.mm.1	1,25	Nek1	JUC0800002325.mm.1	2,05	0,016662	0,460023		
TC0800000650.mm.1	1,25	Nek1	JUC0800002326.mm.1	-2,15	0,041316	0,54428		
TC0800000650.mm.1	1,25	Nek1	JUC0800002333.mm.1	-2,18	0,041392	0,544587		
TC0900001483.mm.1	-1,12	Lrrfip2	JUC0900006972.mm.1	2,05	0,006746	0,393343		
TC0500000459.mm.1	-1,48	Cpeb2	JUC0500002464.mm.1	2,05	0,036068	0,529704		
TC0500000459.mm.1	-1,48	Cpeb2	JUC0500002465.mm.1	-3,07	0,037915	0,535116		

TC0600003393.mm.1	1,11		JUC0600014030.mm.1	2,05	0,019657	0,474887		
TC0700001676.mm.1	-1,49		JUC0700007214.mm.1	2,05	0,021334	0,481727		
TC0600000190.mm.1	-1,15	Wnt16	JUC0600000589.mm.1	2,05	0,019322	0,473294		
TC0400001215.mm.1	-1,18	Stil	JUC0400004580.mm.1	2,05	0,042225	0,546983		
TC1000002583.mm.1	1,02	Aldh1l2	JUC1000010634.mm.1	2,05	0,034236	0,525168		
TC1100002799.mm.1	1,29	Fam114a2	JUC1100013182.mm.1	2,05	0,033847	0,524033		
TC1100003438.mm.1	-1,19	Med13	JUC1100016536.mm.1	2,05	0,015464	0,454312		
TC1100001748.mm.1	-1,4	Mrc2	JUC1100008595.mm.1	2,05	0,004334	0,364898		
TC0100003840.mm.1	1,52	Camk1g	JUC0100017834.mm.1	2,04	0,012011	0,432286		
TC0100003840.mm.1	1,52	Camk1g	PSR0100031446.mm.1	-2,05	0,00343	0,355303	Alternative 3' Acce	0,22
TC0100003840.mm.1	1,52	Camk1g	JUC0100017831.mm.1	-2,16	0,013248	0,441416		
TC0100003840.mm.1	1,52	Camk1g	PSR0100031436.mm.1	-2,24	0,028023	0,505851	Cassette Exon	0,04
TC0100003840.mm.1	1,52	Camk1g	PSR0100031451.mm.1	-2,8	0,004628	0,369794	Cassette Exon	0,35
TC0100003840.mm.1	1,52	Camk1g	JUC0100017842.mm.1	-3,69	0,01042	0,42148		
TC1600000353.mm.1	-1,83	Lpp	PSR1600003466.mm.1	2,04	0,044906	0,55271	Alternative 3' Acce	0,19
TC0300001699.mm.1	-1,02	Crh	PSR0300013353.mm.1	2,04	0,003841	0,358714	Alternative 3' Acce	0,17
TC0500001785.mm.1	2,18		PSR0500016528.mm.1	2,04	0,003609	0,356223	Alternative 5' Donc	0,17
TC0500001785.mm.1	2,18		JUC0500008998.mm.1	-3,29	0,014686	0,450253		
TC0800002839.mm.1	-1,53		PSR0800021864.mm.1	2,04	0,009429	0,416077	Alternative 3' Acce	0,16
TC1900001709.mm.1	-1,16	Ccdc186; A630007B06Rik	PSR1900015055.mm.1	2,04	0,030608	0,513486	Alternative 5' Donc	0,15
TC1300000611.mm.1	-1,68		PSR1300003857.mm.1	2,04	0,006715	0,393343	Alternative 3' Acce	0,14
TC0400001646.mm.1	1,73	Rsrp1; D4Wsu53e	PSR0400013126.mm.1	2,04	0,043475	0,549882	Intron Retention	0,13
TC0400001646.mm.1	1,73	Rsrp1; D4Wsu53e	JUC0400006801.mm.1	-2,07	0,026469	0,501252		
TC0700000628.mm.1	-1,51	Scgb1b27	PSR0700005366.mm.1	2,04	0,00819	0,40571	Cassette Exon	0,11
TC1900001098.mm.1	1,21	Dagla	PSR1900010280.mm.1	2,04	0,027417	0,503961	Cassette Exon	0,09
TC1900001098.mm.1	1,21	Dagla	PSR1900010271.mm.1	-2,19	0,034518	0,525929	Cassette Exon	0,11
TC0300002159.mm.1	-1,41	Gm20356	PSR0300016229.mm.1	2,04	0,018048	0,467148	Cassette Exon	0,1
TC0600000348.mm.1	-1,35		PSR0600002666.mm.1	2,04	0,016435	0,459007	Cassette Exon	0,1
TC1700000844.mm.1	1,18		PSR1700008103.mm.1	2,04	0,017507	0,463964	Cassette Exon	0,1
TC1100002944.mm.1	-1,86		PSR1100026834.mm.1	2,04	0,049684	0,563531	Cassette Exon	0,09
TC0100001602.mm.1	-1,35	Cd84	PSR0100013240.mm.1	2,04	0,024643	0,49468	Alternative 5' Donc	0,07
TC0800002160.mm.1	-1,09	Primpol; Ccdc111	PSR0800016653.mm.1	2,04	0,009557	0,416829	Alternative 3' Acce	0,06
TC0900001137.mm.1	1,3	Gm10634	JUC0900004864.mm.1	2,04	0,021428	0,482126		
TC0900001137.mm.1	1,3	Gm10634	PSR0900008924.mm.1	2,01	0,012562	0,435777	Cassette Exon	0,06
TC1200001030.mm.1	-1,83	Ifi27; Ifi27l1	JUC1200004093.mm.1	2,04	0,00061	0,304044		
TC1300001140.mm.1	-1,31	Ccdc125	JUC1300003785.mm.1	2,04	0,00531	0,378059		
TC0100002235.mm.1	-1,23		JUC0100010198.mm.1	2,04	0,013033	0,439367		
TC1700001188.mm.1	1,05	Gpatch11	JUC1700006150.mm.1	2,04	0,022852	0,488353		
TC0100002772.mm.1	-1,06	A730008H23Rik; Hjurp; 6	JUC0100012695.mm.1	2,04	0,004852	0,37168		
TC0100000077.mm.1	-1,1		JUC0100000360.mm.1	2,04	0,003498	0,355638		
TC0200002419.mm.1	1,07	Vstm2l	JUC0200009925.mm.1	2,04	0,019599	0,474724		
TC1500001050.mm.1	-1,15		JUC1500004694.mm.1	2,04	0,016545	0,459612		
TC1500000686.mm.1	-1,09		JUC1500002916.mm.1	2,04	0,008732	0,409583		
TC1500000742.mm.1	-1,1	Srebf2	JUC1500003299.mm.1	2,04	0,004341	0,365077		
TC0200002125.mm.1	-1,2	4930511F01Rik; Kif16bos	JUC0200008456.mm.1	2,04	0,024485	0,493992		
TC1700000036.mm.1	1,35	Zdhhc14	JUC1700000113.mm.1	2,04	0,019817	0,475294		
TC1600001751.mm.1	-1,04	Gpr128	JUC1600007397.mm.1	2,04	0,021334	0,481727		
TC1600001751.mm.1	-1,04	Gpr128	JUC1600007395.mm.1	-2,38	0,011275	0,426793		

TC0900000535.mm.1	-2,51	Scn4b	JUC0900001891.mm.1	2,04	0,045404	0,554085		
TC0700001580.mm.1	-1,54	Olfml1	JUC0700006793.mm.1	2,04	0,028267	0,506588		
TC1100002549.mm.1	-1,41	Hmmr	JUC1100012290.mm.1	2,04	0,016123	0,457772		
TC1100002770.mm.1	-1,02	Tnip1	JUC1100013022.mm.1	2,04	0,031944	0,518107		
TC1100002001.mm.1	1,03	Slc26a11	JUC1100009934.mm.1	2,04	0,048882	0,561732		
TC1100003829.mm.1	1,86	Hdac5; Mir8101; mmu-mi	PSR1100035562.mm.1	2,03	0,049825	0,563773	Cassette Exon	0,19
TC1100003829.mm.1	1,86	Hdac5; Mir8101; mmu-mi	JUC1100018597.mm.1	-2,28	0,022288	0,486084		
TC1100003829.mm.1	1,86	Hdac5; Mir8101; mmu-mi	JUC1100018584.mm.1	-2,44	0,024212	0,493294		
TC1100003829.mm.1	1,86	Hdac5; Mir8101; mmu-mi	PSR1100035558.mm.1	-2,47	0,027409	0,503961	Intron Retention	0,35
TC1100003829.mm.1	1,86	Hdac5; Mir8101; mmu-mi	PSR1100035571.mm.1	-3,43	0,043453	0,549792	Cassette Exon	0,21
TC1100003829.mm.1	1,86	Hdac5; Mir8101; mmu-mi	JUC1100018580.mm.1	-4,19	0,007978	0,403853		
TC1700001852.mm.1	1,89	Zfp799	JUC1700009253.mm.1	2,03	0,026729	0,502096		
TC1700001852.mm.1	1,89	Zfp799	PSR1700017139.mm.1	-3,31	0,003118	0,353892	Alternative 5' Donc	0,32
TC1400001725.mm.1	5,81	Sncg	JUC1400007337.mm.1	2,03	0,033675	0,523549		
TC1400001725.mm.1	5,81	Sncg	PSR1400013426.mm.1	-2,39	0,002394	0,348564	Cassette Exon	0,16
TC1400001725.mm.1	5,81	Sncg	PSR1400013423.mm.1	-2,66	0,032149	0,518586	Cassette Exon	0,18
TC1400001725.mm.1	5,81	Sncg	PSR1400013428.mm.1	-5	0,003398	0,354856	Alternative 5' Donc	0,26
TC0300000359.mm.1	-1,01	Naa15	PSR0300002561.mm.1	2,03	0,044415	0,551538	Intron Retention	0,24
TC0400001307.mm.1	-1,64	Elovl1	PSR0400009836.mm.1	2,03	0,030527	0,51337	Intron Retention	0,24
TC1000001126.mm.1	-1,93		PSR1000008517.mm.1	2,03	0,026485	0,501252	Intron Retention	0,23
TC0400003174.mm.1	-1,39	4921539E11Rik	PSR0400025237.mm.1	2,03	0,041368	0,544518	Alternative 3' Acce	0,17
TC0900002990.mm.1	1,12	Nek11	PSR0900024657.mm.1	2,03	0,000063	0,250119	Alternative 5' Donc	0,15
TC0200004010.mm.1	-2,51	Olfr1167	PSR0200033930.mm.1	2,03	0,031318	0,516204	Alternative 3' Acce	0,15
TC1000001914.mm.1	-1,48		PSR1000013798.mm.1	2,03	0,024326	0,49362	Alternative 5' Donc	0,13
TC1700002120.mm.1	-1,23	Cd2ap	PSR1700019843.mm.1	2,03	0,006134	0,387442	Cassette Exon	0,12
TC0100003313.mm.1	-1,35	Tor1aip1	PSR0100027059.mm.1	2,03	0,003528	0,355858	Cassette Exon	0,11
TC0400003760.mm.1	-1,37	Srrm1	PSR0400030967.mm.1	2,03	0,004822	0,371408	Intron Retention	0,11
TC1100001196.mm.1	-1,15	Adap2	PSR1100011159.mm.1	2,03	0,007964	0,403853	Cassette Exon	0,1
TC1200001516.mm.1	1,12	Gm9285	PSR1200010596.mm.1	2,03	0,013364	0,442141	Cassette Exon	0,1
TC1700002669.mm.1	-1,14		PSR1700024290.mm.1	2,03	0,012353	0,434604	Cassette Exon	0,1
TC0100002266.mm.1	-1,05	Uxs1	PSR0100018213.mm.1	2,03	0,000482	0,298999	Alternative 3' Acce	0,09
TC0600002812.mm.1	2,14		PSR0600021800.mm.1	2,03	0,001021	0,316361	Cassette Exon	0,09
TC0600002812.mm.1	2,14		PSR0600021801.mm.1	-2,03	0,001021	0,316361	Cassette Exon	0,09
TC1500000939.mm.1	-1,18	Ano6	PSR1500007455.mm.1	2,03	0,036505	0,531328	Cassette Exon	0,09
TC0100003090.mm.1	-1,43	Sox13	PSR0100024822.mm.1	2,03	0,048531	0,560991	Cassette Exon	0,08
TC0400001425.mm.1	1,39	Inpp5b; Mir698	PSR0400010708.mm.1	2,03	0,039882	0,54093	Cassette Exon	0,08
TC0500003734.mm.1	1,31	1500011B03Rik	PSR0500033836.mm.1	2,03	0,02109	0,480371	Cassette Exon	0,07
TC1100000950.mm.1	-1,01	Arrb2; Mir7115; mmu-mir	PSR1100008323.mm.1	2,03	0,005082	0,375364	Cassette Exon	0,07
TC0400002572.mm.1	-1,34	Tomm5; Gm8069	PSR0400020986.mm.1	2,03	0,025183	0,496638	Cassette Exon	0,05
TC0400002572.mm.1	-1,34	Tomm5; Gm8069	JUC0400010888.mm.1	-2,17	0,048051	0,559717		
TC0200003156.mm.1	-1,02	C8g	JUC0200013182.mm.1	2,03	0,031494	0,516965		
TC1400000930.mm.1	-1,28	Ints9	JUC1400003980.mm.1	2,03	0,035532	0,528764		
TC0200003269.mm.1	1	Sh3glb2	JUC0200013957.mm.1	2,03	0,024495	0,493992		
TC0200002979.mm.1	-1,04		JUC0200012428.mm.1	2,03	0,003209	0,354243		
TC1100003890.mm.1	1,06	Kansl1	JUC1100019022.mm.1	2,03	0,016899	0,460797		
TC1100003890.mm.1	1,06	Kansl1	JUC1100019029.mm.1	-4,26	0,002057	0,341838		
TC0200004430.mm.1	1,02	Gm14207	JUC0200018939.mm.1	2,03	0,006432	0,389972		
TC0200004122.mm.1	-1,15	F2	JUC0200017639.mm.1	2,03	0,034464	0,525929		

TC1700002556.mm.1	1,31	Heatr5b	JUC1700012546.mm.1	2,03	0,041525	0,544763		
TC1700001386.mm.1	1,27	Tmem181c-ps; Tmem181a	JUC1700006831.mm.1	2,03	0,041271	0,544166		
TC1700002135.mm.1	-1,17	Enpp4	JUC1700010609.mm.1	2,03	0,009955	0,41841		
TC0100000315.mm.1	1,05	Mrps9	JUC0100001330.mm.1	2,03	0,0356	0,528887		
TC0200001368.mm.1	-1,56	Spi1; LOC100862024; Sfpi1	JUC0200005277.mm.1	2,03	0,013543	0,44358		
TC0500002579.mm.1	-1,13	Txk	JUC0500012409.mm.1	2,03	0,047352	0,558291		
TC0500002579.mm.1	-1,13	Txk	JUC0500012407.mm.1	-2,23	0,012076	0,43255		
TC0700004316.mm.1	1,13	Tbc1d10b	JUC0700019131.mm.1	2,03	0,019226	0,472971		
TC0500000687.mm.1	-1,24		JUC0500003326.mm.1	2,03	0,006559	0,39213		
TC0500000417.mm.1	1,58	Ppp2r2c	JUC0500002293.mm.1	2,03	0,00393	0,359818		
TC0700000315.mm.1	-1,18	Ceacam20	JUC0700001215.mm.1	2,03	0,015563	0,454812		
TC0700000658.mm.1	-1,69	Rhpn2	JUC0700002742.mm.1	2,03	0,036662	0,531675		
TC0700001198.mm.1	1,04	Stard5	JUC0700005493.mm.1	2,03	0,040586	0,542474		
TC0600001394.mm.1	2,56	Necap1	JUC0600005921.mm.1	2,03	0,037517	0,533968		
TC0600001394.mm.1	2,56	Necap1	JUC0600005927.mm.1	-3,02	0,018939	0,471376		
TC0300003085.mm.1	-1,4	Clca5	JUC0300012728.mm.1	2,03	0,030077	0,511908		
TC1100001293.mm.1	1,07	Bcas3	JUC1100006412.mm.1	2,03	0,004678	0,370201		
TC0300003234.mm.1	-1,15	Pear1	JUC0300009043.mm.1	2,03	0,036792	0,532063		
TC1100002181.mm.1	-1,02	Tbrg4; Snora5c	JUC1100011095.mm.1	2,03	0,035072	0,527576		
TC0400004119.mm.1	-1,21	Prdm16	JUC0400017954.mm.1	2,03	0,018666	0,470205		
TC0400004119.mm.1	-1,21	Prdm16	JUC0400017947.mm.1	-2,1	0,011026	0,425657		
TC0400003847.mm.1	-1,03	Pqlc2	JUC0400016479.mm.1	2,03	0,013652	0,444374		
TC1000000025.mm.1	-1,08	Rmnd1; Gm16153	JUC1000000073.mm.1	2,03	0,010071	0,418974		
TC0X00002556.mm.1	-1,63	Gyk	JUC0X00008255.mm.1	2,03	0,029369	0,509995		
TC0400002523.mm.1	-1,51	Cd72	JUC0400010643.mm.1	2,03	0,029062	0,508976		
TC1000002828.mm.1	7,21	Syt1	JUC1000011854.mm.1	2,02	0,018069	0,46716		
TC1000002828.mm.1	7,21	Syt1	PSR1000021546.mm.1	-2,52	0,006711	0,393343	Alternative 5' Donc	0,23
TC1000002828.mm.1	7,21	Syt1	JUC1000011856.mm.1	-2,55	0,006976	0,395722		
TC1000002828.mm.1	7,21	Syt1	PSR1000021534.mm.1	-2,93	0,004879	0,372195	Alternative 5' Donc	0,22
TC1000002828.mm.1	7,21	Syt1	PSR1000021543.mm.1	-3,12	0,010413	0,42148	Cassette Exon	0,2
TC1000002828.mm.1	7,21	Syt1	PSR1000021537.mm.1	-3,4	0,000486	0,298999	Cassette Exon	0,22
TC1000002828.mm.1	7,21	Syt1	JUC1000011859.mm.1	-3,4	0,003083	0,353892		
TC1000002828.mm.1	7,21	Syt1	PSR1000021540.mm.1	-3,59	0,003011	0,353447	Alternative 3' Acce	0,48
TC1000002828.mm.1	7,21	Syt1	PSR1000021539.mm.1	-18,01	0,005702	0,382524	Alternative 3' Acce	0,48
TC0700002695.mm.1	-1,13	Plekhg2	PSR0700023594.mm.1	2,02	0,007623	0,401947	Intron Retention	0,2
TC1300002370.mm.1	2,02	Gm26803	PSR1300015780.mm.1	2,02	0,00143	0,327447		
TC1300002370.mm.1	2,02	Gm26803	PSR1300015781.mm.1	-2,02	0,00143	0,327447	Cassette Exon	0,2
TC1300002370.mm.1	2,02	Gm26803	JUC1300008324.mm.1	-3,38	0,000957	0,313363		
TC0200002478.mm.1	-1,11	Mybl2	PSR0200020224.mm.1	2,02	0,026517	0,501411	Alternative 3' Acce	0,16
TC0400000580.mm.1	1,06	Slc44a1	PSR0400004226.mm.1	2,02	0,009848	0,417839	Alternative 5' Donc	0,16
TC0900002451.mm.1	-1,05	Kif23	PSR0900020542.mm.1	2,02	0,006388	0,389608	Alternative 5' Donc	0,16
TC1300002197.mm.1	-1,26	Zfp367	PSR1300014168.mm.1	2,02	0,038888	0,537855	Alternative 3' Acce	0,16
TC0600000078.mm.1	-1,2	Phf14	PSR0600000600.mm.1	2,02	0,004856	0,37168	Alternative 5' Donc	0,14
TC0900001171.mm.1	-1,6	Plscr2	PSR0900009221.mm.1	2,02	0,029019	0,508842	Alternative 5' Donc	0,14
TC0100001678.mm.1	1,79	Gm16432; Gm7068	PSR0100013905.mm.1	2,02	0,002612	0,349501	Cassette Exon	0,12
TC0100001678.mm.1	1,79	Gm16432; Gm7068	PSR0100013896.mm.1	-2,06	0,00974	0,417839	Cassette Exon	0,1
TC1900000787.mm.1	-1,83	Tcf7l2	PSR1900006905.mm.1	2,02	0,045149	0,553323	Cassette Exon	0,12
TC0100001260.mm.1	-1,68	Cacna1s	PSR0100010437.mm.1	2,02	0,045699	0,554833	Cassette Exon	0,1

TC0100001260.mm.1	-1,68	Cacna1s	JUC0100006033.mm.1	2,02	0,014902	0,451803		
TC0100001260.mm.1	-1,68	Cacna1s	JUC0100006002.mm.1	-2,25	0,026034	0,49949		
TC0500001399.mm.1	-1,22	P2rx7; mmu-mir-8115	PSR0500012387.mm.1	2,02	0,04212	0,546571	Cassette Exon	0,1
TC1100003672.mm.1	1,61	Neurod2	PSR1100033834.mm.1	2,02	0,013654	0,444384	Cassette Exon	0,09
TC1100003672.mm.1	1,61	Neurod2	PSR1100033835.mm.1	-2,05	0,012625	0,436032	Cassette Exon	0,1
TC0400000455.mm.1	1,02	Reck	PSR0400003194.mm.1	2,02	0,007783	0,402902	Cassette Exon	0,09
TC0700000447.mm.1	-1,41	Cyp2f2	PSR0700003515.mm.1	2,02	0,008786	0,409822	Cassette Exon	0,09
TC1000000502.mm.1	1,36	Hsf2	PSR1000003266.mm.1	2,02	0,002129	0,342919	Cassette Exon	0,09
TC1600000487.mm.1	-1,53	Mylk	PSR1600004616.mm.1	2,02	0,042548	0,547918	Cassette Exon	0,09
TC1600000487.mm.1	-1,53	Mylk	JUC1600002428.mm.1	2,01	0,022914	0,488524		
TC0100002371.mm.1	1,35	Tyw5	PSR0100019261.mm.1	2,02	0,029469	0,510123	Intron Retention	0,08
TC0400002446.mm.1	-1,05	Gm6297	PSR0400019702.mm.1	2,02	0,02418	0,493294	Alternative 3' Acce	0,08
TC1400002320.mm.1	-1,04	RP23-103I12.3	PSR1400017985.mm.1	2,02	0,027251	0,503642	Cassette Exon	0,08
TC1400000514.mm.1	-1,26	Fbxo34	JUC1400002445.mm.1	2,02	0,019479	0,474295		
TC0200003512.mm.1	-1,29	Rnd3	JUC0200015085.mm.1	2,02	0,020379	0,477603		
TC1100003817.mm.1	-1,23	Dusp3	JUC1100018497.mm.1	2,02	0,00761	0,401767		
TC1200000018.mm.1	1,55	Dnajc27	JUC1200000143.mm.1	2,02	0,019302	0,473104		
TC0200004936.mm.1	-1,43		JUC0200021603.mm.1	2,02	0,002702	0,349612		
TC1700002644.mm.1	-1,11	Gm19696	JUC1700012910.mm.1	2,02	0,004493	0,36808		
TC1700001671.mm.1	1,02	Msln	JUC1700008301.mm.1	2,02	0,030731	0,514036		
TC1700001671.mm.1	1,02	Msln	JUC1700008307.mm.1	-2,05	0,030919	0,514584		
TC1900001617.mm.1	-1,17	Nt5c2	JUC1900007944.mm.1	2,02	0,018325	0,468127		
TC0100001294.mm.1	-1,04	Dennd1b	JUC0100006207.mm.1	2,02	0,035028	0,527498		
TC1500000300.mm.1	-1,37	Cthrc1	JUC1500001247.mm.1	2,02	0,046252	0,556168		
TC1600000376.mm.1	1,14	Il1rap	JUC1600001862.mm.1	2,02	0,028275	0,506588		
TC0200000512.mm.1	-1,25	Urm1	JUC0200001887.mm.1	2,02	0,036562	0,531355		
TC0800001080.mm.1	-1,31	Slc6a2	JUC0800004436.mm.1	2,02	0,007774	0,402841		
TC0800001125.mm.1	-1,21	Gpr114	JUC0800004692.mm.1	2,02	0,041035	0,543746		
TC0900000500.mm.1	-1,15	Mcam	JUC0900001705.mm.1	2,02	0,021864	0,484635		
TC0700003029.mm.1	-1,12	Abcc6	JUC0700014710.mm.1	2,02	0,023238	0,490032		
TC0500003563.mm.1	1,21	Usp42	JUC0500017760.mm.1	2,02	0,012802	0,437296		
TC0700001879.mm.1	-1,54	Tgfb1i1	JUC0700008621.mm.1	2,02	0,01625	0,458312		
TC0400004187.mm.1	-1,13	Cyp4a10; Cyp4a31	JUC0400004688.mm.1	2,02	0,041596	0,545		
TC1000002501.mm.1	-1,25	Tcf3	JUC1000010164.mm.1	2,02	0,049044	0,561978		
TC0Y00000005.mm.1	3,59	Kdm5d	PSR0Y00000069.mm.1	2,02	0,016658	0,460022		
TC0400002205.mm.1	1,08	Ints8	JUC0400009611.mm.1	2,02	0,026729	0,502096		
TC0200003313.mm.1	5,68	Stxbp1	PSR0200028278.mm.1	2,01	0,002469	0,349135	Cassette Exon	0,17
TC0200003313.mm.1	5,68	Stxbp1	PSR0200028285.mm.1	-3,32	0,034217	0,525138	Cassette Exon	0,25
TC0200003313.mm.1	5,68	Stxbp1	JUC0200014258.mm.1	-3,48	0,004119	0,3623		
TC0200003313.mm.1	5,68	Stxbp1	PSR0200028263.mm.1	-3,64	0,02041	0,477916	Alternative 3' Acce	0,41
TC0200003313.mm.1	5,68	Stxbp1	PSR0200028266.mm.1	-3,68	0,00599	0,386319	Cassette Exon	0,27
TC0200003313.mm.1	5,68	Stxbp1	PSR0200028286.mm.1	-6,2	0,009424	0,415987	Cassette Exon	0,25
TC0200003313.mm.1	5,68	Stxbp1	PSR0200028279.mm.1	-7,4	0,003592	0,356223	Cassette Exon	0,41
TC1000000079.mm.1	1,25	Shprh	JUC1000000298.mm.1	2,01	0,035808	0,529247		
TC1000000079.mm.1	1,25	Shprh	PSR1000000539.mm.1	-2,08	0,033829	0,523962	Alternative 5' Donc	0,18
TC1000000079.mm.1	1,25	Shprh	PSR1000000580.mm.1	-2,36	0,004131	0,3623	Intron Retention	0,3
TC0X00000912.mm.1	1,22	Zc3h12b	PSR0X00005852.mm.1	2,01	0,026573	0,501636	Intron Retention	0,25
TC0X00000912.mm.1	1,22	Zc3h12b	JUC0X00002883.mm.1	-2,7	0,011108	0,426		

TC1100000294.mm.1	1,59	Ccdc88a	PSR1100002430.mm.1	2,01	0,008792	0,409822	Cassette Exon	0,1
TC1100000294.mm.1	1,59	Ccdc88a	JUC1100001319.mm.1	-2,07	0,029962	0,511499		
TC1100000294.mm.1	1,59	Ccdc88a	PSR1100002457.mm.1	-2,12	0,01003	0,41878	Alternative 5' Donc	0,14
TC1100000294.mm.1	1,59	Ccdc88a	PSR1100002480.mm.1	-2,8	0,029953	0,511499	Alternative 5' Donc	0,22
TC0700001644.mm.1	-1,2	Mical2	PSR0700013660.mm.1	2,01	0,017041	0,461771	Cassette Exon	0,12
TC0700001644.mm.1	-1,2	Mical2	JUC0700007039.mm.1	-2,15	0,048536	0,560994		
TC0700001644.mm.1	-1,2	Mical2	PSR0700013671.mm.1	-3,05	0,014436	0,448973	Intron Retention	0,18
TC0X00000742.mm.1	-1,08	Brcc3	PSR0X00005159.mm.1	2,01	0,005774	0,383104	Alternative 5' Donc	0,18
TC1100002369.mm.1	1,75	0610010F05Rik	JUC1100011601.mm.1	2,01	0,025188	0,496656		
TC1100002369.mm.1	1,75	0610010F05Rik	PSR1100022314.mm.1	-2,31	0,01746	0,463886	Alternative 3' Acce	0,17
TC1600001048.mm.1	1,22	Morc3	PSR1600008167.mm.1	2,01	0,044306	0,551368	Alternative 5' Donc	0,16
TC0800002857.mm.1	1,82	A930006D01Rik	PSR0800022061.mm.1	2,01	0,011915	0,431651	Alternative 3' Acce	0,16
TC0800002857.mm.1	1,82	A930006D01Rik	PSR0800022062.mm.1	-2,01	0,011915	0,431651	Cassette Exon	0,09
TC0300002451.mm.1	-1,15	BC021767	PSR0300018832.mm.1	2,01	0,041858	0,545617	Alternative 5' Donc	0,15
TC1800000890.mm.1	-1,71	Cyb5a; Cyb5	PSR1800006447.mm.1	2,01	0,001176	0,319796	Alternative 5' Donc	0,15
TC0200003360.mm.1	1,24	Rab14	JUC0200014600.mm.1	2,01	0,023859	0,492097		
TC0200003360.mm.1	1,24	Rab14	PSR0200028910.mm.1	-2,02	0,023604	0,490995	Alternative 3' Acce	0,14
TC0100003186.mm.1	-1,59	Cfhr1	PSR0100025837.mm.1	2,01	0,00045	0,297771	Alternative 5' Donc	0,13
TC1000002629.mm.1	-1,04	Stab2	JUC1000010868.mm.1	2,01	0,048881	0,561732		
TC1000002629.mm.1	-1,04	Stab2	PSR1000019884.mm.1	-2,38	0,037726	0,534622	Cassette Exon	0,13
TC0300001449.mm.1	-1,58	Gtf2b	PSR0300011718.mm.1	2,01	0,003998	0,361023	Cassette Exon	0,12
TC0600003534.mm.1	1,75	Vamp1	PSR0600011872.mm.1	2,01	0,008861	0,410539	Cassette Exon	0,11
TC0700003149.mm.1	4,54		PSR0700028323.mm.1	2,01	0,006209	0,387912	Cassette Exon	0,07
TC0700003149.mm.1	4,54		PSR0700028324.mm.1	-2,01	0,006209	0,387912	Cassette Exon	0,11
TC0700004550.mm.1	-1,32	Mob2	PSR0700038394.mm.1	2,01	0,028815	0,508347	Alternative 3' Acce	0,11
TC0100003881.mm.1	1,5	D630023F18Rik	PSR0100020159.mm.1	2,01	0,008376	0,4065	Cassette Exon	0,09
TC0700002826.mm.1	-1,19	Uba2	PSR0700025246.mm.1	2,01	0,019118	0,472394	Cassette Exon	0,09
TC0700004224.mm.1	-1,02	Ndufab1	PSR0700035180.mm.1	2,01	0,044384	0,551441	Cassette Exon	0,09
TC0X00001419.mm.1	3,3	A730046J19Rik	PSR0X00008991.mm.1	2,01	0,00548	0,379939	Cassette Exon	0,09
TC0700000368.mm.1	1,16	Zfp180	PSR0700002652.mm.1	2,01	0,017595	0,464312	Cassette Exon	0,08
TC0X00002382.mm.1	1,03	1700111N16Rik	PSR0X00014729.mm.1	2,01	0,003311	0,354243	Cassette Exon	0,08
TC0400003237.mm.1	-1,04	Zyg11b	PSR0400025778.mm.1	2,01	0,002316	0,346468	Alternative 3' Acce	0,07
TC0700000806.mm.1	-1,55	Josd2; Mir7052; mmu-mir	PSR0700006656.mm.1	2,01	0,044648	0,552129	Cassette Exon	0,07
TC0600000941.mm.1	-1,35	Nagk	PSR0600007213.mm.1	2,01	0,026017	0,499373	Alternative 3' Acce	0,06
TC1400001902.mm.1	-1,03	Fermt2	JUC1400007906.mm.1	2,01	0,040921	0,543423		
TC0200005145.mm.1	1,11	Slc35c2	JUC0200022647.mm.1	2,01	0,004979	0,373851		
TC0200004942.mm.1	-1,36	E2f1	JUC0200021647.mm.1	2,01	0,029482	0,510123		
TC0100002236.mm.1	1,52	Tbc1d8	JUC0100010216.mm.1	2,01	0,004982	0,373851		
TC0100002236.mm.1	1,52	Tbc1d8	JUC0100010201.mm.1	-3,69	0,047828	0,559283		
TC0100000332.mm.1	-1,05	Tpp2	JUC0100001360.mm.1	2,01	0,004386	0,366156		
TC1900000439.mm.1	-1,08	Jak2	JUC1900002087.mm.1	2,01	0,013275	0,441681		
TC0200002401.mm.1	-1,16	Tgif2	JUC0200009829.mm.1	2,01	0,039511	0,539762		
TC1700000835.mm.1	1,34	Pla2g7	JUC1700004331.mm.1	2,01	0,044991	0,552961		
TC0800001630.mm.1	-1,17	BC068157	PSR0800013434.mm.1	2,01	0,000092	0,255472		
TC0800000132.mm.1	-1,05	Upf3a	JUC0800000717.mm.1	2,01	0,004159	0,3623		
TC0900000783.mm.1	-1,14		JUC0900003131.mm.1	2,01	0,013328	0,441836		
TC0900000126.mm.1	1,07	Slc36a4	JUC0900000463.mm.1	2,01	0,009838	0,417839		
TC0600000852.mm.1	-1,39	Capg	JUC0600003288.mm.1	2,01	0,026228	0,500189		

TC0600000388.mm.1	-1,35	Tbxas1	JUC0600001506.mm.1	2,01	0,024684	0,49472		
TC0700002024.mm.1	-1,72	Urah	JUC0700009411.mm.1	2,01	0,022919	0,488542		
TC1100002207.mm.1	1,03	Tns3	JUC1100011138.mm.1	2,01	0,014744	0,450631		
TC0X00003413.mm.1	-1,25	Gm14459	JUC0X00005868.mm.1	2,01	0,048803	0,561497		
TC0Y00000016.mm.1	-1,03	Gm21064	JUC0Y00000156.mm.1	2,01	0,020858	0,479521		
TC0Y00000016.mm.1	-1,03	Gm21064	JUC0Y00000138.mm.1	-2,15	0,034599	0,52602		
TC0Y00000016.mm.1	-1,03	Gm21064	JUC0Y00000160.mm.1	-2,27	0,000797	0,311589		
TC0100003620.mm.1	2,11	Cep170	JUC0100016915.mm.1	-2,01	0,024009	0,492783		
TC0100003620.mm.1	2,11	Cep170	JUC0100016900.mm.1	-2,07	0,025096	0,496269		
TC0100003620.mm.1	2,11	Cep170	PSR0100029759.mm.1	-2,5	0,01359	0,443703	Cassette Exon	0,18
TC0100003620.mm.1	2,11	Cep170	JUC0100016910.mm.1	-2,54	0,026906	0,502518		
TC0100003620.mm.1	2,11	Cep170	PSR0100029750.mm.1	-2,59	0,028168	0,506368	Cassette Exon	0,19
TC0100003620.mm.1	2,11	Cep170	PSR0100029734.mm.1	-3,78	0,002984	0,352501	Intron Retention	0,67
TC0100003620.mm.1	2,11	Cep170	JUC0100016912.mm.1	-5,38	0,0068	0,393614		
TC0900001942.mm.1	1,23	Acad8	JUC0900009261.mm.1	-2,01	0,019221	0,472971		
TC0900001942.mm.1	1,23	Acad8	PSR0900016347.mm.1	-2,42	0,034221	0,525151	Alternative 5' Donc	0,24
TC0900001942.mm.1	1,23	Acad8	PSR0900016339.mm.1	-3,11	0,030081	0,511908	Intron Retention	0,52
TC0900001942.mm.1	1,23	Acad8	JUC0900009266.mm.1	-3,51	0,004601	0,369794		
TC1200000718.mm.1	11,07	Rdh12	PSR1200005115.mm.1	-2,01	0,016442	0,459025	Cassette Exon	0,23
TC1200000718.mm.1	11,07	Rdh12	JUC1200002925.mm.1	-2,56	0,024198	0,493294		
TC1200000718.mm.1	11,07	Rdh12	PSR1200005117.mm.1	-2,87	0,019717	0,474887	Intron Retention	0,44
TC1200000718.mm.1	11,07	Rdh12	JUC1200002927.mm.1	-3,26	0,00474	0,370933		
TC1200000718.mm.1	11,07	Rdh12	PSR1200005113.mm.1	-3,51	0,015673	0,455416	Cassette Exon	0,29
TC1200000718.mm.1	11,07	Rdh12	PSR1200005114.mm.1	-7,82	0,002274	0,345991	Alternative 3' Acce	0,26
TC1200000718.mm.1	11,07	Rdh12	PSR1200005118.mm.1	-13,49	0,001572	0,331411	Intron Retention	0,44
TC0300000419.mm.1	2,12	Dclk1	PSR0300003024.mm.1	-2,01	0,007217	0,39826	Cassette Exon	0,31
TC0300000419.mm.1	2,12	Dclk1	JUC0300001556.mm.1	-2,07	0,003229	0,354243		
TC0300000419.mm.1	2,12	Dclk1	PSR0300003000.mm.1	-2,09	0,01877	0,470771	Cassette Exon	0,23
TC0300000419.mm.1	2,12	Dclk1	JUC0300001566.mm.1	-2,14	0,024193	0,493294		
TC0300000419.mm.1	2,12	Dclk1	PSR0300003011.mm.1	-2,2	0,025512	0,497664	Alternative 5' Donc	0,12
TC0300000419.mm.1	2,12	Dclk1	PSR0300003003.mm.1	-2,33	0,007293	0,398847	Cassette Exon	0,1
TC0300000419.mm.1	2,12	Dclk1	PSR0300003017.mm.1	-2,41	0,001013	0,316361	Cassette Exon	0,18
TC0300000419.mm.1	2,12	Dclk1	PSR0300003002.mm.1	-2,47	0,004035	0,361636	Cassette Exon	0,29
TC0300000419.mm.1	2,12	Dclk1	PSR0300003026.mm.1	-2,5	0,001819	0,336896	Alternative 5' Donc	0,15
TC0300000419.mm.1	2,12	Dclk1	PSR0300003023.mm.1	-2,57	0,027146	0,503137	Cassette Exon	0,34
TC0300000419.mm.1	2,12	Dclk1	PSR0300003006.mm.1	-2,59	0,018104	0,467161	Alternative 3' Acce	0,25
TC0300000419.mm.1	2,12	Dclk1	PSR0300003004.mm.1	-2,61	0,018423	0,468579	Cassette Exon	0,15
TC0300000419.mm.1	2,12	Dclk1	JUC0300001576.mm.1	-2,74	0,034315	0,525546		
TC0300000419.mm.1	2,12	Dclk1	JUC0300001557.mm.1	-2,84	0,01077	0,423955		
TC0300000419.mm.1	2,12	Dclk1	PSR0300003007.mm.1	-2,85	0,01816	0,467432	Alternative 3' Acce	0,25
TC0300000419.mm.1	2,12	Dclk1	JUC0300001572.mm.1	-2,92	0,043315	0,549297		
TC0300000419.mm.1	2,12	Dclk1	PSR0300003005.mm.1	-2,94	0,003194	0,354243	Alternative 3' Acce	0,25
TC0300000419.mm.1	2,12	Dclk1	PSR0300003001.mm.1	-2,96	0,020693	0,479027	Cassette Exon	0,4
TC0300000419.mm.1	2,12	Dclk1	JUC0300001578.mm.1	-3,97	0,002553	0,349501		
TC0300000419.mm.1	2,12	Dclk1	JUC0300001558.mm.1	-4,42	0,021834	0,484607		
TC0300000419.mm.1	2,12	Dclk1	PSR0300002998.mm.1	-4,99	0,024559	0,494116	Cassette Exon	0,28
TC0500002073.mm.1	2,74	Kcnh2	PSR0500018833.mm.1	-2,01	0,003959	0,359922		
TC0500002073.mm.1	2,74	Kcnh2	PSR0500018837.mm.1	-2,05	0,004558	0,369262	Cassette Exon	0,1

TC0500002073.mm.1	2,74	Kcnh2	PSR0500018817.mm.1	-2,36	0,011241	0,426718	Cassette Exon	0,12
TC0500002073.mm.1	2,74	Kcnh2	PSR0500018821.mm.1	-2,55	0,014874	0,451677	Cassette Exon	0,24
TC0500002073.mm.1	2,74	Kcnh2	JUC0500010262.mm.1	-2,59	0,003034	0,353543		
TC0500002073.mm.1	2,74	Kcnh2	PSR0500018820.mm.1	-2,82	0,004102	0,36228	Cassette Exon	0,25
TC0500002073.mm.1	2,74	Kcnh2	JUC0500010255.mm.1	-2,91	0,003752	0,357586		
TC0500002073.mm.1	2,74	Kcnh2	PSR0500018828.mm.1	-3,18	0,00154	0,330388	Alternative 3' Acce	0,35
TC0500002073.mm.1	2,74	Kcnh2	PSR0500018827.mm.1	-3,5	0,000983	0,31497	Alternative 5' Donc	0,4
TC0500002073.mm.1	2,74	Kcnh2	PSR0500018838.mm.1	-3,77	0,000381	0,293183	Cassette Exon	0,28
TC0500003104.mm.1	2,24	Ccdc64	PSR0500027643.mm.1	-2,01	0,000032	0,237924	Cassette Exon	0,15
TC0500003104.mm.1	2,24	Ccdc64	PSR0500027656.mm.1	-2,06	0,008826	0,410288	Alternative 5' Donc	0,11
TC0500003104.mm.1	2,24	Ccdc64	PSR0500027651.mm.1	-2,07	0,016089	0,457532	Cassette Exon	0,19
TC0500003104.mm.1	2,24	Ccdc64	PSR0500027634.mm.1	-2,19	0,006383	0,389554	Cassette Exon	0,16
TC0500003104.mm.1	2,24	Ccdc64	PSR0500027636.mm.1	-2,28	0,031323	0,516204	Cassette Exon	0,22
TC0500003104.mm.1	2,24	Ccdc64	PSR0500027653.mm.1	-2,28	0,018866	0,471054	Alternative 3' Acce	0,2
TC0500003104.mm.1	2,24	Ccdc64	JUC0500015060.mm.1	-2,84	0,008301	0,406122		
TC0500003104.mm.1	2,24	Ccdc64	JUC0500015074.mm.1	-2,93	0,029915	0,511499		
TC0500003104.mm.1	2,24	Ccdc64	JUC0500015067.mm.1	-3,34	0,016502	0,459434		
TC0500003104.mm.1	2,24	Ccdc64	PSR0500027648.mm.1	-3,53	0,036007	0,529546	Alternative 5' Donc	0,4
TC0300000494.mm.1	3,63	Arhgef26	PSR0300003429.mm.1	-2,01	0,034522	0,52593	Cassette Exon	0,24
TC0300000494.mm.1	3,63	Arhgef26	JUC0300001791.mm.1	-2,23	0,015455	0,45429		
TC0300000494.mm.1	3,63	Arhgef26	PSR0300003422.mm.1	-2,41	0,021491	0,482644	Cassette Exon	0,18
TC0300000494.mm.1	3,63	Arhgef26	PSR0300003434.mm.1	-2,55	0,00338	0,354359	Cassette Exon	0,29
TC0300000494.mm.1	3,63	Arhgef26	PSR0300003420.mm.1	-2,62	0,018646	0,470078	Cassette Exon	0,13
TC0300000494.mm.1	3,63	Arhgef26	PSR0300003415.mm.1	-3,31	0,003564	0,356223	Alternative 3' Acce	0,39
TC0300000494.mm.1	3,63	Arhgef26	JUC0300001797.mm.1	-3,36	0,022968	0,488818		
TC0300000494.mm.1	3,63	Arhgef26	PSR0300003414.mm.1	-3,52	0,008738	0,409622	Alternative 3' Acce	0,39
TC0300000494.mm.1	3,63	Arhgef26	JUC0300001799.mm.1	-3,57	0,016488	0,459391		
TC0300000494.mm.1	3,63	Arhgef26	PSR0300003423.mm.1	-3,8	0,000559	0,304044	Cassette Exon	0,31
TC0300000494.mm.1	3,63	Arhgef26	PSR0300003413.mm.1	-5,01	0,001079	0,317191	Alternative 3' Acce	0,39
TC0300000494.mm.1	3,63	Arhgef26	JUC0300001794.mm.1	-6,52	0,015035	0,452111		
TC1200001110.mm.1	1,75	Evl	PSR1200007914.mm.1	-2,01	0,013035	0,439367	Cassette Exon	0,09
TC1200001110.mm.1	1,75	Evl	PSR1200007916.mm.1	-2,42	0,007182	0,397855	Cassette Exon	0,19
TC1200001110.mm.1	1,75	Evl	PSR1200007929.mm.1	-2,68	0,012469	0,435315	Cassette Exon	0,35
TC1200001110.mm.1	1,75	Evl	PSR1200007927.mm.1	-2,68	0,007012	0,395737	Cassette Exon	0,18
TC1200001110.mm.1	1,75	Evl	JUC1200004439.mm.1	-2,82	0,002001	0,341091		
TC1200001110.mm.1	1,75	Evl	JUC1200004438.mm.1	-3,56	0,000183	0,284485		
TC0500002830.mm.1	2,96	Tmem150c	PSR0500024897.mm.1	-2,01	0,004621	0,369794	Alternative 3' Acce	0,13
TC0500002830.mm.1	2,96	Tmem150c	PSR0500024908.mm.1	-2,12	0,030846	0,514331	Cassette Exon	0,19
TC0500002830.mm.1	2,96	Tmem150c	PSR0500024906.mm.1	-2,84	0,010807	0,424055	Cassette Exon	0,17
TC0500002830.mm.1	2,96	Tmem150c	PSR0500024907.mm.1	-2,94	0,009258	0,414559	Cassette Exon	0,18
TC0500002830.mm.1	2,96	Tmem150c	PSR0500024904.mm.1	-2,97	0,007244	0,39826	Cassette Exon	0,18
TC0500002830.mm.1	2,96	Tmem150c	PSR0500024909.mm.1	-4,35	0,005914	0,384138	Alternative 5' Donc	0,3
TC0400000396.mm.1	3,08	Gm13306; Ccl27b; Ccl27a;	JUC0400001233.mm.1	-2,01	0,044044	0,550824		
TC0400000396.mm.1	3,08	Gm13306; Ccl27b; Ccl27a;	PSR0400002555.mm.1	-2,46	0,042776	0,548097	Cassette Exon	0,23
TC0400000396.mm.1	3,08	Gm13306; Ccl27b; Ccl27a;	PSR0400002541.mm.1	-2,66	0,010391	0,421344	Alternative 3' Acce	0,25
TC0400000396.mm.1	3,08	Gm13306; Ccl27b; Ccl27a;	PSR0400002548.mm.1	-2,78	0,003201	0,354243	Cassette Exon	0,22
TC0400000396.mm.1	3,08	Gm13306; Ccl27b; Ccl27a;	PSR0400002538.mm.1	-3,27	0,021772	0,48438	Alternative 3' Acce	0,29
TC0400000396.mm.1	3,08	Gm13306; Ccl27b; Ccl27a;	JUC0400001236.mm.1	-3,32	0,028832	0,508414		

TC0400000396.mm.1	3,08	Gm13306; Ccl27b; Ccl27a;	JUC0400001239.mm.1	-3,71	0,004514	0,368648		
TC0400000396.mm.1	3,08	Gm13306; Ccl27b; Ccl27a;	JUC0400001237.mm.1	-3,86	0,002924	0,352289		
TC1700002420.mm.1	2,88	Ankrd12	PSR1700022447.mm.1	-2,01	0,039887	0,54093	Cassette Exon	0,12
TC1700002420.mm.1	2,88	Ankrd12	PSR1700022451.mm.1	-2,2	0,010743	0,423772	Cassette Exon	0,13
TC1700002420.mm.1	2,88	Ankrd12	PSR1700022438.mm.1	-2,41	0,013089	0,439727	Cassette Exon	0,07
TC1700002420.mm.1	2,88	Ankrd12	PSR1700022462.mm.1	-2,48	0,024635	0,49468	Cassette Exon	0,28
TC1700002420.mm.1	2,88	Ankrd12	PSR1700022463.mm.1	-2,5	0,026294	0,5006	Cassette Exon	0,28
TC1700002420.mm.1	2,88	Ankrd12	JUC1700012017.mm.1	-2,62	0,023952	0,4926		
TC1700002420.mm.1	2,88	Ankrd12	PSR1700022453.mm.1	-2,63	0,024206	0,493294	Alternative 3' Acce	0,21
TC1700002420.mm.1	2,88	Ankrd12	PSR1700022444.mm.1	-2,69	0,006153	0,387873	Cassette Exon	0,09
TC1700002420.mm.1	2,88	Ankrd12	PSR1700022465.mm.1	-2,76	0,04116	0,544039	Cassette Exon	0,17
TC1700002420.mm.1	2,88	Ankrd12	JUC1700012029.mm.1	-2,81	0,002866	0,352207		
TC1700002420.mm.1	2,88	Ankrd12	PSR1700022457.mm.1	-2,85	0,008715	0,409266	Alternative 3' Acce	0,17
TC1700002420.mm.1	2,88	Ankrd12	JUC1700012031.mm.1	-5,35	0,014793	0,450911		
TC0100000055.mm.1	2,65	Prex2	PSR0100000545.mm.1	-2,01	0,024296	0,49362	Alternative 5' Donc	0,11
TC0100000055.mm.1	2,65	Prex2	JUC0100000292.mm.1	-2,05	0,012731	0,436677		
TC0100000055.mm.1	2,65	Prex2	JUC0100000293.mm.1	-2,2	0,041439	0,544646		
TC0100000055.mm.1	2,65	Prex2	PSR0100000539.mm.1	-2,3	0,029061	0,508976	Cassette Exon	0,14
TC0100000055.mm.1	2,65	Prex2	PSR0100000546.mm.1	-2,33	0,01576	0,455437	Alternative 5' Donc	0,11
TC0100000055.mm.1	2,65	Prex2	PSR0100000501.mm.1	-2,36	0,033601	0,523309	Cassette Exon	0,22
TC0100000055.mm.1	2,65	Prex2	PSR0100000519.mm.1	-2,4	0,016738	0,460345	Alternative 5' Donc	0,16
TC0100000055.mm.1	2,65	Prex2	PSR0100000516.mm.1	-2,62	0,044915	0,552742	Alternative 3' Acce	0,22
TC0100000055.mm.1	2,65	Prex2	PSR0100000499.mm.1	-2,76	0,020819	0,47952	Cassette Exon	0,17
TC0100000055.mm.1	2,65	Prex2	PSR0100000523.mm.1	-3,37	0,008538	0,407916	Alternative 5' Donc	0,23
TC1600000213.mm.1	1,72	Tmem191c	PSR1600001745.mm.1	-2,01	0,027462	0,504045	Intron Retention	0,23
TC1600000213.mm.1	1,72	Tmem191c	JUC1600000902.mm.1	-2,3	0,006028	0,386586		
TC1600000213.mm.1	1,72	Tmem191c	PSR1600001751.mm.1	-2,41	0,036294	0,530447	Alternative 5' Donc	0,18
TC1600001134.mm.1	1,46	Coro7	PSR1600008992.mm.1	-2,01	0,02492	0,4958	Cassette Exon	0,22
TC1600001134.mm.1	1,46	Coro7	JUC1600004707.mm.1	-2,25	0,000068	0,250992		
TC1600001134.mm.1	1,46	Coro7	JUC1600004723.mm.1	-2,83	0,027446	0,504041		
TC1600001134.mm.1	1,46	Coro7	JUC1600004691.mm.1	-3,16	0,019609	0,474765		
TC1300000882.mm.1	1,81	Lpcat1	JUC1300002953.mm.1	-2,01	0,035804	0,529215		
TC1300000882.mm.1	1,81	Lpcat1	PSR1300005637.mm.1	-2,29	0,007935	0,403819	Alternative 5' Donc	0,21
TC1300000882.mm.1	1,81	Lpcat1	JUC1300002954.mm.1	-3,36	0,00742	0,399901		
TC0100002209.mm.1	1,45	Tsga10	PSR0100017781.mm.1	-2,01	0,04961	0,563407	Alternative 3' Acce	0,15
TC0100002209.mm.1	1,45	Tsga10	PSR0100017783.mm.1	-2,16	0,044115	0,550916	Intron Retention	0,2
TC1400000119.mm.1	1,92	Slc4a7	JUC1400000577.mm.1	-2,01	0,037648	0,534212		
TC1400000119.mm.1	1,92	Slc4a7	PSR1400000938.mm.1	-2,02	0,033865	0,524071	Cassette Exon	0,2
TC1400000119.mm.1	1,92	Slc4a7	PSR1400000951.mm.1	-2,08	0,013739	0,444758	Alternative 5' Donc	0,13
TC1400000119.mm.1	1,92	Slc4a7	JUC1400000564.mm.1	-2,26	0,0086	0,408294		
TC1400000119.mm.1	1,92	Slc4a7	JUC1400000569.mm.1	-2,66	0,018698	0,470429		
TC1400000119.mm.1	1,92	Slc4a7	JUC1400000570.mm.1	-2,84	0,00359	0,356223		
TC1400000119.mm.1	1,92	Slc4a7	JUC1400000592.mm.1	-3,53	0,004056	0,361816		
TC1400000119.mm.1	1,92	Slc4a7	JUC1400000587.mm.1	-3,77	0,0117	0,429969		
TC1400000119.mm.1	1,92	Slc4a7	JUC1400000593.mm.1	-3,84	0,012794	0,437245		
TC1400000119.mm.1	1,92	Slc4a7	JUC1400000596.mm.1	-5,08	0,005078	0,375342		
TC1400001184.mm.1	1,78		PSR1400009299.mm.1	-2,01	0,022498	0,487114	Cassette Exon	0,09
TC1400001184.mm.1	1,78		PSR1400009275.mm.1	-2,08	0,046939	0,557545	Cassette Exon	0,17

TC1400001184.mm.1	1,78		JUC1400004966.mm.1	-2,12	0,033514	0,523077		
TC1400001184.mm.1	1,78		PSR1400009273.mm.1	-2,24	0,009276	0,414791	Cassette Exon	0,12
TC1400001184.mm.1	1,78		JUC1400004969.mm.1	-2,5	0,031769	0,517539		
TC1400001184.mm.1	1,78		PSR1400009296.mm.1	-2,52	0,009705	0,417765	Alternative 5' Donc	0,19
TC1400001184.mm.1	1,78		PSR1400009277.mm.1	-2,53	0,00143	0,327447	Cassette Exon	0,17
TC1400001184.mm.1	1,78		PSR1400009295.mm.1	-2,55	0,029581	0,510459	Alternative 5' Donc	0,19
TC1400001184.mm.1	1,78		JUC1400004974.mm.1	-3,73	0,007916	0,403819		
TC0600003417.mm.1	1,35	Kras	PSR0600026935.mm.1	-2,01	0,025689	0,498173	Cassette Exon	0,18
TC0600003417.mm.1	1,35	Kras	JUC0600014130.mm.1	-2,32	0,010841	0,424161		
TC1200000016.mm.1	1,41	Dnmt3a	JUC1200000119.mm.1	-2,01	0,016684	0,460085		
TC1200000016.mm.1	1,41	Dnmt3a	PSR1200000206.mm.1	-2,21	0,010483	0,421891	Cassette Exon	0,12
TC1200000016.mm.1	1,41	Dnmt3a	JUC1200000128.mm.1	-2,23	0,035262	0,52837		
TC1200000016.mm.1	1,41	Dnmt3a	PSR1200000208.mm.1	-3,07	0,031353	0,516297	Alternative 5' Donc	0,18
TC1800001529.mm.1	-1,09		PSR1800011211.mm.1	-2,01	0,002195	0,344814	Alternative 5' Donc	0,17
TC0200004802.mm.1	-1,15	Foxa2	PSR0200041040.mm.1	-2,01	0,010004	0,418553	Alternative 5' Donc	0,16
TC0700002828.mm.1	1,48	Gpi1	PSR0700025274.mm.1	-2,01	0,002119	0,342919	Alternative 3' Acce	0,11
TC0700002828.mm.1	1,48	Gpi1	PSR0700025301.mm.1	-2,8	0,000897	0,313363	Cassette Exon	0,16
TC1500001720.mm.1	1,05	Ly6k	PSR1500012981.mm.1	-2,01	0,00833	0,406336	Alternative 5' Donc	0,16
TC1500001720.mm.1	1,05	Ly6k	JUC1500007374.mm.1	-2,29	0,011775	0,430069		
TC1700000362.mm.1	1,09	Clcn7	PSR1700002968.mm.1	-2,01	0,006172	0,387873	Alternative 3' Acce	0,16
TC1700000362.mm.1	1,09	Clcn7	JUC1700001599.mm.1	-2,62	0,04358	0,550052		
TC1400001074.mm.1	-1,09	1700108F19Rik	PSR1400008643.mm.1	-2,01	0,017206	0,462491	Alternative 5' Donc	0,15
TC1600000479.mm.1	1,03	Muc13	PSR1600004527.mm.1	-2,01	0,001574	0,331411	Alternative 3' Acce	0,15
TC1900000617.mm.1	1,44	R3hcc1l	PSR1900005541.mm.1	-2,01	0,049576	0,563357	Alternative 5' Donc	0,15
TC0100003302.mm.1	1,55	BC034090	PSR0100026877.mm.1	-2,01	0,015341	0,453763	Alternative 3' Acce	0,13
TC0100003302.mm.1	1,55	BC034090	JUC0100015359.mm.1	-2,58	0,002493	0,349135		
TC0200002352.mm.1	1,24	Raly; a	PSR0200018695.mm.1	-2,01	0,025588	0,497808	Alternative 3' Acce	0,05
TC0200002352.mm.1	1,24	Raly; a	JUC0200009484.mm.1	-2,17	0,007463	0,400381		
TC0200002352.mm.1	1,24	Raly; a	PSR0200018747.mm.1	-2,4	0,015632	0,45509	Cassette Exon	0,13
TC0200002352.mm.1	1,24	Raly; a	PSR0200018737.mm.1	-2,64	0,025627	0,497926	Alternative 3' Acce	0,05
TC1000000142.mm.1	1,07	Ifngr1	PSR1000000990.mm.1	-2,01	0,004242	0,363295	Alternative 5' Donc	0,13
TC1000000142.mm.1	1,07	Ifngr1	JUC1000000498.mm.1	-3,11	0,033037	0,521501		
TC1800001469.mm.1	-1,06	Alpk2	PSR1800010854.mm.1	-2,01	0,043121	0,548644	Cassette Exon	0,12
TC1900000953.mm.1	1,67	Klc2	PSR1900008436.mm.1	-2,01	0,029916	0,511499	Alternative 5' Donc	0,12
TC1900000953.mm.1	1,67	Klc2	JUC1900004626.mm.1	-2,73	0,041508	0,544746		
TC1600000099.mm.1	1,38	1810013L24Rik	PSR1600000693.mm.1	-2,01	0,029181	0,509333	Alternative 5' Donc	0,12
TC0500000347.mm.1	1,32	Depdc5	JUC0500001783.mm.1	-2,01	0,022518	0,487114		
TC0500000347.mm.1	1,32	Depdc5	PSR0500003208.mm.1	-2,12	0,007209	0,398121	Alternative 5' Donc	0,11
TC0600002353.mm.1	1,59	Pyurf; Gm26712; PYURF; F	JUC0600009582.mm.1	-2,01	0,031577	0,517177		
TC0600002353.mm.1	1,59	Pyurf; Gm26712; PYURF; F	PSR0600018352.mm.1	-2,09	0,007916	0,403819	Alternative 3' Acce	0,11
TC0600002353.mm.1	1,59	Pyurf; Gm26712; PYURF; F	JUC0600009581.mm.1	-5,73	0,00467	0,370181		
TC0X00001520.mm.1	1,43	Phf8	PSR0X00009526.mm.1	-2,01	0,004312	0,364152	Cassette Exon	0,11
TC1300002717.mm.1	-1	Itga1; Pelo	PSR1300018375.mm.1	-2,01	0,040395	0,541998	Cassette Exon	0,11
TC1300002717.mm.1	-1	Itga1; Pelo	JUC1300009878.mm.1	-2,09	0,005263	0,377955		
TC1300002717.mm.1	-1	Itga1; Pelo	PSR1300018381.mm.1	-2,68	0,029697	0,510949	Cassette Exon	0,1
TC0400003722.mm.1	1,18	Dhdds	PSR0400030564.mm.1	-2,01	0,014602	0,449914	Alternative 5' Donc	0,1
TC0400003722.mm.1	1,18	Dhdds	PSR0400030553.mm.1	-2,09	0,007387	0,399541	Alternative 3' Acce	0,05
TC0700003641.mm.1	1,17	Il16	PSR0700030942.mm.1	-2,01	0,001336	0,325349	Cassette Exon	0,1

TC1700001839.mm.1	1,93	Akap8l	PSR1700016968.mm.1	-2,01	0,019622	0,47483	Cassette Exon	0,1
TC0100001451.mm.1	-1,1	4930523C07Rik	PSR0100011887.mm.1	-2,01	0,043686	0,550236	Cassette Exon	0,09
TC0400000377.mm.1	1,03	Dnaic1	PSR0400002325.mm.1	-2,01	0,043409	0,549603	Cassette Exon	0,09
TC1200000741.mm.1	1,24	Slc39a9	PSR1200005239.mm.1	-2,01	0,004723	0,370888	Cassette Exon	0,09
TC1300000089.mm.1	1,16	Lyst	PSR1300000644.mm.1	-2,01	0,034699	0,526311	Cassette Exon	0,09
TC1500001946.mm.1	-1,04	Cyp2d10	PSR1500015341.mm.1	-2,01	0,049347	0,562803	Cassette Exon	0,09
TC0100001397.mm.1	-1,02	Stx6	PSR0100011534.mm.1	-2,01	0,03247	0,519631	Cassette Exon	0,08
TC1700000279.mm.1	1,02	Zfp229	PSR1700002097.mm.1	-2,01	0,005548	0,380679	Cassette Exon	0,07
TC0600002936.mm.1	1,66	Atp2b2	JUC0600011694.mm.1	-2,01	0,014196	0,447628		
TC0600002936.mm.1	1,66	Atp2b2	PSR0600022579.mm.1	-2,16	0,011534	0,428564	Cassette Exon	0,05
TC0600002936.mm.1	1,66	Atp2b2	JUC0600011712.mm.1	-2,16	0,007337	0,399094		
TC0600002936.mm.1	1,66	Atp2b2	JUC0600011688.mm.1	-4,28	0,032953	0,52122		
TC1600002039.mm.1	1,02	Tiam1	PSR1600015629.mm.1	-2,01	0,027263	0,503682	Cassette Exon	0,02
TC1400000269.mm.1	-1,05	Il17rd	JUC1400001307.mm.1	-2,01	0,026591	0,501731		
TC0300000287.mm.1	1,28	4930594O21Rik	PSR0300002076.mm.1	-2,01	0,002641	0,349612		
TC0300000287.mm.1	1,28	4930594O21Rik	JUC0300001072.mm.1	-2,17	0,035653	0,528956		
TC0300000287.mm.1	1,28	4930594O21Rik	JUC0300001069.mm.1	-2,41	0,031173	0,515739		
TC0100002319.mm.1	-1,18	Nab1	JUC0100010568.mm.1	-2,01	0,039203	0,538692		
TC1900000994.mm.1	-1,08	Slc22a20	JUC1900004979.mm.1	-2,01	0,023603	0,490995		
TC1900000994.mm.1	-1,08	Slc22a20	JUC1900004982.mm.1	-2,29	0,009748	0,417839		
TC0100000427.mm.1	1,01	Aox1	JUC0100001928.mm.1	-2,01	0,040614	0,542549		
TC0100000427.mm.1	1,01	Aox1	JUC0100001917.mm.1	-2,84	0,045396	0,554085		
TC1900000040.mm.1	-1,22	Pcx	JUC1900000278.mm.1	-2,01	0,022284	0,486046		
TC1500002192.mm.1	-1,05	Kmt2d	JUC1500010209.mm.1	-2,01	0,013496	0,443238		
TC1500000417.mm.1	1,13	D15Ertd621e	JUC1500001710.mm.1	-2,01	0,049147	0,562349		
TC1500000037.mm.1	-1,7	Fyb; Gm7666	JUC1500000187.mm.1	-2,01	0,002181	0,344453		
TC0200000456.mm.1	-1,35	Gm2240	JUC0200001403.mm.1	-2,01	0,010611	0,422786		
TC0800003235.mm.1	1,17	Znrf1	JUC0800006021.mm.1	-2,01	0,035554	0,528764		
TC0800003235.mm.1	1,17	Znrf1	JUC0800006022.mm.1	-2,29	0,033354	0,522553		
TC0800000059.mm.1	1,18		JUC0800000282.mm.1	-2,01	0,038313	0,536167		
TC0700004332.mm.1	1,13	Gm166	JUC0700019189.mm.1	-2,01	0,000528	0,304044		
TC0900001409.mm.1	1,48	Usp19	JUC0900006263.mm.1	-2,01	0,017306	0,463271		
TC0600000724.mm.1	-1,1	Mmrn1	JUC0600002800.mm.1	-2,01	0,032715	0,520439		
TC0600001942.mm.1	1,03	Iqub	JUC0600007975.mm.1	-2,01	0,002098	0,342919		
TC0700003002.mm.1	1,35	Ppfia3	JUC0700014496.mm.1	-2,01	0,031747	0,517489		
TC0700002763.mm.1	1,11	Arhgap33	JUC0700013117.mm.1	-2,01	0,017479	0,463933		
TC0700003991.mm.1	1,03	Dchs1	JUC0700017530.mm.1	-2,01	0,033744	0,523558		
TC0700003566.mm.1	-1,02	Kif7	JUC0700015897.mm.1	-2,01	0,007301	0,398847		
TC0700003599.mm.1	1,16	Wdr73	JUC0700016151.mm.1	-2,01	0,026822	0,502373		
TC0700003599.mm.1	1,16	Wdr73	JUC0700016160.mm.1	-2,44	0,041292	0,544227		
TC0700001372.mm.1	-1,01	Prkrir	JUC0700006031.mm.1	-2,01	0,040145	0,541609		
TC1100002492.mm.1	-1,02	Gabrp	JUC1100012071.mm.1	-2,01	0,022491	0,487114		
TC1100003600.mm.1	-1,17	Abi3	JUC1100017278.mm.1	-2,01	0,016407	0,458907		
TC1100003342.mm.1	1,3	5730455P16Rik	JUC1100016072.mm.1	-2,01	0,024366	0,49362		
TC1100001476.mm.1	1,28	4833417C18Rik	JUC1100007172.mm.1	-2,01	0,002711	0,349612		
TC1100001227.mm.1	1,07	Gm11426	JUC1100006031.mm.1	-2,01	0,018135	0,467325		
TC0400001999.mm.1	1,31		PSR0400016322.mm.1	-2,01	0,018608	0,469665		
TC1000000753.mm.1	-1	Pofut2	JUC1000002989.mm.1	-2,01	0,020386	0,477737		

TC0400002642.mm.1	1,13 Grin3a	JUC0400011148.mm.1	-2,01	0,044147	0,551024		
TC0400002642.mm.1	1,13 Grin3a	JUC0400011146.mm.1	-3,5	0,004681	0,370323		
TC0X00003369.mm.1	1,86	JUC0X00010617.mm.1	-2,01	0,021202	0,48113		
TC1400002168.mm.1	6,09 Atp8a2	PSR1400016785.mm.1	-2,02	0,034135	0,524882	Cassette Exon	0,13
TC1400002168.mm.1	6,09 Atp8a2	JUC1400009199.mm.1	-2,03	0,015887	0,456526		
TC1400002168.mm.1	6,09 Atp8a2	PSR1400016789.mm.1	-2,07	0,027551	0,504437	Cassette Exon	0,08
TC1400002168.mm.1	6,09 Atp8a2	PSR1400016784.mm.1	-2,08	0,020948	0,479982	Cassette Exon	0,15
TC1400002168.mm.1	6,09 Atp8a2	PSR1400016776.mm.1	-2,2	0,024201	0,493294	Cassette Exon	0,23
TC1400002168.mm.1	6,09 Atp8a2	PSR1400016805.mm.1	-2,21	0,01122	0,426684	Alternative 3' Acce	0,2
TC1400002168.mm.1	6,09 Atp8a2	PSR1400016807.mm.1	-2,21	0,040481	0,542144	Cassette Exon	0,13
TC1400002168.mm.1	6,09 Atp8a2	PSR1400016783.mm.1	-2,33	0,002696	0,349612	Cassette Exon	0,22
TC1400002168.mm.1	6,09 Atp8a2	PSR1400016796.mm.1	-2,4	0,001953	0,340561	Cassette Exon	0,16
TC1400002168.mm.1	6,09 Atp8a2	PSR1400016802.mm.1	-2,67	0,017867	0,466232	Cassette Exon	0,3
TC1400002168.mm.1	6,09 Atp8a2	PSR1400016795.mm.1	-2,83	0,009005	0,412255	Cassette Exon	0,15
TC1400002168.mm.1	6,09 Atp8a2	JUC1400009173.mm.1	-2,88	0,00956	0,416915		
TC1400002168.mm.1	6,09 Atp8a2	PSR1400016782.mm.1	-2,93	0,002557	0,349501	Cassette Exon	0,4
TC1400002168.mm.1	6,09 Atp8a2	JUC1400009189.mm.1	-3,32	0,016377	0,458693		
TC1400002168.mm.1	6,09 Atp8a2	PSR1400016780.mm.1	-3,36	0,020159	0,477086	Cassette Exon	0,49
TC1400002168.mm.1	6,09 Atp8a2	JUC1400009198.mm.1	-3,38	0,042983	0,548405		
TC1400002168.mm.1	6,09 Atp8a2	JUC1400009200.mm.1	-3,43	0,017064	0,462011		
TC1400002168.mm.1	6,09 Atp8a2	PSR1400016827.mm.1	-4,22	0,009926	0,418378	Cassette Exon	0,41
TC1400002168.mm.1	6,09 Atp8a2	PSR1400016811.mm.1	-4,42	0,006998	0,395737	Cassette Exon	0,25
TC1400002168.mm.1	6,09 Atp8a2	PSR1400016826.mm.1	-4,42	0,006794	0,393554	Cassette Exon	0,07
TC1400002168.mm.1	6,09 Atp8a2	PSR1400016790.mm.1	-4,56	0,020341	0,477323	Cassette Exon	0,41
TC1400002168.mm.1	6,09 Atp8a2	PSR1400016799.mm.1	-4,87	0,00003	0,229631	Cassette Exon	0,52
TC1400002168.mm.1	6,09 Atp8a2	JUC1400009190.mm.1	-4,91	0,000016	0,19183		
TC1400002168.mm.1	6,09 Atp8a2	PSR1400016822.mm.1	-5,14	0,002713	0,349612	Cassette Exon	0,07
TC1400002168.mm.1	6,09 Atp8a2	JUC1400009193.mm.1	-5,31	0,013658	0,444386		
TC1400002168.mm.1	6,09 Atp8a2	PSR1400016778.mm.1	-5,81	0,000558	0,304044	Cassette Exon	0,32
TC1400002168.mm.1	6,09 Atp8a2	PSR1400016821.mm.1	-5,82	0,002739	0,349612	Cassette Exon	0,21
TC1400002168.mm.1	6,09 Atp8a2	PSR1400016823.mm.1	-6,32	0,010179	0,419711	Alternative 3' Acce	0,32
TC1400002168.mm.1	6,09 Atp8a2	JUC1400009172.mm.1	-6,6	0,026136	0,499937		
TC1400002168.mm.1	6,09 Atp8a2	PSR1400016779.mm.1	-6,8	0,00066	0,304044	Cassette Exon	0,45
TC1400002168.mm.1	6,09 Atp8a2	PSR1400016820.mm.1	-6,85	0,001855	0,337479	Alternative 5' Dono	0,26
TC1400002168.mm.1	6,09 Atp8a2	JUC1400009171.mm.1	-7,15	0,004933	0,373093		
TC1400002168.mm.1	6,09 Atp8a2	JUC1400009209.mm.1	-7,96	0,003963	0,359922		
TC1400002168.mm.1	6,09 Atp8a2	PSR1400016819.mm.1	-8,05	0,002263	0,34578	Alternative 5' Dono	0,26
TC1400002168.mm.1	6,09 Atp8a2	PSR1400016825.mm.1	-10,68	0,018416	0,468484	Cassette Exon	0,07
TC1400002168.mm.1	6,09 Atp8a2	JUC1400009181.mm.1	-10,71	0,000688	0,304792		
TC1400002168.mm.1	6,09 Atp8a2	JUC1400009207.mm.1	-12,97	0,003157	0,353892		
TC1400002168.mm.1	6,09 Atp8a2	JUC1400009206.mm.1	-13,04	0,022943	0,488667		
TC1400002168.mm.1	6,09 Atp8a2	JUC1400009205.mm.1	-17,29	0,0021	0,342919		
TC1100000235.mm.1	6,65 Fam161a	PSR1100001920.mm.1	-2,02	0,023492	0,490754	Cassette Exon	0,07
TC1100000235.mm.1	6,65 Fam161a	PSR1100001912.mm.1	-2,31	0,040035	0,54131	Cassette Exon	0,25
TC1100000235.mm.1	6,65 Fam161a	PSR1100001926.mm.1	-2,33	0,003071	0,353892	Cassette Exon	0,26
TC1100000235.mm.1	6,65 Fam161a	PSR1100001913.mm.1	-3,17	0,018781	0,470789	Cassette Exon	0,29
TC1100000235.mm.1	6,65 Fam161a	PSR1100001910.mm.1	-3,88	0,007648	0,402193	Cassette Exon	0,07
TC1100000235.mm.1	6,65 Fam161a	PSR1100001918.mm.1	-4,85	0,025689	0,498173	Alternative 3' Acce	0,48

TC1100000235.mm.1	6,65	Fam161a	JUC1100000989.mm.1	-5,61	0,010077	0,418974		
TC1100000235.mm.1	6,65	Fam161a	JUC1100000984.mm.1	-5,74	0,009732	0,417839		
TC1100000235.mm.1	6,65	Fam161a	PSR1100001911.mm.1	-7,53	0,0006	0,304044	Cassette Exon	0,21
TC1100000235.mm.1	6,65	Fam161a	PSR1100001909.mm.1	-8,67	0,001548	0,330631	Cassette Exon	0,07
TC1100000235.mm.1	6,65	Fam161a	JUC1100000985.mm.1	-9,05	0,000203	0,28803		
TC1100000235.mm.1	6,65	Fam161a	JUC1100000981.mm.1	-10,25	0,011779	0,430069		
TC1100000235.mm.1	6,65	Fam161a	JUC1100000987.mm.1	-12,85	0,002514	0,349406		
TC1100000235.mm.1	6,65	Fam161a	PSR1100001908.mm.1	-19,89	0,000432	0,297771	Cassette Exon	0,41
TC0400000585.mm.1	3,73	Fsd1l	JUC0400002130.mm.1	-2,02	0,036324	0,530547		
TC0400000585.mm.1	3,73	Fsd1l	JUC0400002122.mm.1	-2,16	0,032238	0,51899		
TC0400000585.mm.1	3,73	Fsd1l	PSR0400004278.mm.1	-2,28	0,011374	0,427487	Cassette Exon	0,16
TC0400000585.mm.1	3,73	Fsd1l	PSR0400004275.mm.1	-2,86	0,014783	0,45081	Cassette Exon	0,25
TC0400000585.mm.1	3,73	Fsd1l	PSR0400004265.mm.1	-2,93	0,004986	0,373851	Alternative 5' Donc	0,25
TC0400000585.mm.1	3,73	Fsd1l	PSR0400004257.mm.1	-2,94	0,00675	0,393343	Cassette Exon	0,18
TC0400000585.mm.1	3,73	Fsd1l	PSR0400004260.mm.1	-3,05	0,000206	0,28803	Cassette Exon	0,18
TC0400000585.mm.1	3,73	Fsd1l	JUC0400002134.mm.1	-4,32	0,017735	0,465216		
TC0400000585.mm.1	3,73	Fsd1l	JUC0400002132.mm.1	-4,97	0,002265	0,34578		
TC0400000585.mm.1	3,73	Fsd1l	PSR0400004261.mm.1	-4,98	0,006068	0,386691	Cassette Exon	0,29
TC0400000585.mm.1	3,73	Fsd1l	PSR0400004277.mm.1	-5,96	0,001446	0,328226	Cassette Exon	0,41
TC0400000585.mm.1	3,73	Fsd1l	JUC0400002131.mm.1	-6,01	0,002378	0,348561		
TC0900002435.mm.1	2,59	Lrrc49	PSR0900020452.mm.1	-2,02	0,018224	0,46765	Alternative 5' Donc	0,15
TC0900002435.mm.1	2,59	Lrrc49	JUC0900011522.mm.1	-2,12	0,041688	0,545356		
TC0900002435.mm.1	2,59	Lrrc49	PSR0900020437.mm.1	-2,35	0,028707	0,508054	Alternative 5' Donc	0,2
TC0900002435.mm.1	2,59	Lrrc49	PSR0900020448.mm.1	-2,64	0,009996	0,418456	Cassette Exon	0,19
TC0900002435.mm.1	2,59	Lrrc49	PSR0900020422.mm.1	-2,82	0,003695	0,357266	Alternative 3' Acce	0,27
TC0900002435.mm.1	2,59	Lrrc49	PSR0900020430.mm.1	-3,2	0,028655	0,507969	Alternative 3' Acce	0,35
TC0900002435.mm.1	2,59	Lrrc49	PSR0900020449.mm.1	-3,31	0,021078	0,480371	Cassette Exon	0,19
TC0900002435.mm.1	2,59	Lrrc49	JUC0900011517.mm.1	-4,04	0,005311	0,378059		
TC0900002435.mm.1	2,59	Lrrc49	PSR0900020418.mm.1	-4,74	0,002213	0,34564	Alternative 3' Acce	0,38
TC0200002894.mm.1	1,99	Celf2	PSR0200024067.mm.1	-2,02	0,028031	0,505851	Intron Retention	0,09
TC0200002894.mm.1	1,99	Celf2	PSR0200024104.mm.1	-2,06	0,016105	0,457698	Cassette Exon	0,24
TC0200002894.mm.1	1,99	Celf2	PSR0200024122.mm.1	-2,11	0,024118	0,49322	Cassette Exon	0,1
TC0200002894.mm.1	1,99	Celf2	PSR0200024110.mm.1	-2,13	0,006853	0,393893	Cassette Exon	0,19
TC0200002894.mm.1	1,99	Celf2	PSR0200024072.mm.1	-2,16	0,003731	0,357586		
TC0200002894.mm.1	1,99	Celf2	PSR0200024058.mm.1	-2,24	0,042931	0,548271	Cassette Exon	0,24
TC0200002894.mm.1	1,99	Celf2	JUC0200012176.mm.1	-2,32	0,00921	0,41403		
TC0200002894.mm.1	1,99	Celf2	PSR0200024088.mm.1	-2,37	0,027068	0,502979	Alternative 5' Donc	0,13
TC0200002894.mm.1	1,99	Celf2	PSR0200024111.mm.1	-2,41	0,009862	0,417945	Cassette Exon	0,19
TC0200002894.mm.1	1,99	Celf2	PSR0200024041.mm.1	-2,49	0,003347	0,354243	Alternative 3' Acce	0,12
TC0200002894.mm.1	1,99	Celf2	PSR0200024109.mm.1	-2,54	0,023977	0,492725	Cassette Exon	0,2
TC0200002894.mm.1	1,99	Celf2	PSR0200024098.mm.1	-2,56	0,013995	0,446353	Alternative 3' Acce	0,2
TC0200002894.mm.1	1,99	Celf2	JUC0200012172.mm.1	-2,6	0,04489	0,55271		
TC0200002894.mm.1	1,99	Celf2	PSR0200024114.mm.1	-2,67	0,001696	0,335395	Cassette Exon	0,23
TC0200002894.mm.1	1,99	Celf2	PSR0200024091.mm.1	-2,72	0,049851	0,563773	Cassette Exon	0,2
TC0200002894.mm.1	1,99	Celf2	PSR0200024116.mm.1	-2,81	0,006996	0,395737	Cassette Exon	0,23
TC0200002894.mm.1	1,99	Celf2	PSR0200024120.mm.1	-2,88	0,015967	0,456885	Cassette Exon	0,19
TC0200002894.mm.1	1,99	Celf2	JUC0200012184.mm.1	-2,88	0,020562	0,478336		
TC0200002894.mm.1	1,99	Celf2	JUC0200012190.mm.1	-3,01	0,034688	0,526294		

TC0200002894.mm.1	1,99	Celf2	PSR0200024118.mm.1	-3,02	0,013297	0,44174	Cassette Exon	0,17
TC0200002894.mm.1	1,99	Celf2	PSR0200024095.mm.1	-3,08	0,010757	0,423859	Alternative 3' Acce	0,2
TC0200002894.mm.1	1,99	Celf2	JUC0200012185.mm.1	-3,1	0,000835	0,311886		
TC0200002894.mm.1	1,99	Celf2	JUC0200012181.mm.1	-3,16	0,002875	0,352207		
TC0200002894.mm.1	1,99	Celf2	PSR0200024063.mm.1	-3,21	0,002375	0,34829	Cassette Exon	0,2
TC0200002894.mm.1	1,99	Celf2	PSR0200024100.mm.1	-3,25	0,035632	0,528956	Alternative 5' Donc	0,35
TC0200002894.mm.1	1,99	Celf2	PSR0200024119.mm.1	-3,28	0,000255	0,28803	Cassette Exon	0,21
TC0200002894.mm.1	1,99	Celf2	PSR0200024089.mm.1	-3,29	0,010473	0,421857	Alternative 5' Donc	0,13
TC0200002894.mm.1	1,99	Celf2	JUC0200012186.mm.1	-4,65	0,00129	0,322541		
TC1100000863.mm.1	4,51	Gm12302	PSR1100007148.mm.1	-2,02	0,000751	0,308185	Cassette Exon	0,27
TC1100000863.mm.1	4,51	Gm12302	JUC1100003776.mm.1	-2,62	0,01063	0,422841		
TC1100000863.mm.1	4,51	Gm12302	PSR1100007154.mm.1	-2,84	0,03403	0,524517	Alternative 5' Donc	0,23
TC1100000863.mm.1	4,51	Gm12302	PSR1100007152.mm.1	-3,17	0,009079	0,412827	Alternative 3' Acce	0,28
TC1100000863.mm.1	4,51	Gm12302	PSR1100007146.mm.1	-4,77	0,004776	0,371044	Cassette Exon	0,35
TC1100000863.mm.1	4,51	Gm12302	PSR1100007147.mm.1	-4,95	0,007405	0,399876	Cassette Exon	0,3
TC1100000863.mm.1	4,51	Gm12302	JUC1100003780.mm.1	-4,98	0,004195	0,36298		
TC1100000880.mm.1	2,15	Myh10	JUC1100003894.mm.1	-2,02	0,03291	0,521149		
TC1100000880.mm.1	2,15	Myh10	PSR1100007313.mm.1	-2,08	0,028012	0,505851	Alternative 3' Acce	0,14
TC1100000880.mm.1	2,15	Myh10	JUC1100003891.mm.1	-2,08	0,006912	0,394898		
TC1100000880.mm.1	2,15	Myh10	JUC1100003893.mm.1	-2,08	0,012574	0,435847		
TC1100000880.mm.1	2,15	Myh10	JUC1100003902.mm.1	-2,12	0,015891	0,456526		
TC1100000880.mm.1	2,15	Myh10	PSR1100007321.mm.1	-2,16	0,031961	0,518173	Cassette Exon	0,17
TC1100000880.mm.1	2,15	Myh10	PSR1100007342.mm.1	-2,19	0,01326	0,441506	Cassette Exon	0,09
TC1100000880.mm.1	2,15	Myh10	PSR1100007362.mm.1	-2,2	0,015409	0,453996	Cassette Exon	0,21
TC1100000880.mm.1	2,15	Myh10	JUC1100003885.mm.1	-2,2	0,026219	0,500082		
TC1100000880.mm.1	2,15	Myh10	JUC1100003900.mm.1	-2,26	0,009678	0,417605		
TC1100000880.mm.1	2,15	Myh10	PSR1100007315.mm.1	-2,27	0,013326	0,441836	Alternative 3' Acce	0,15
TC1100000880.mm.1	2,15	Myh10	PSR1100007368.mm.1	-2,27	0,006313	0,389263	Cassette Exon	0,13
TC1100000880.mm.1	2,15	Myh10	PSR1100007319.mm.1	-2,6	0,026559	0,501513	Cassette Exon	0,12
TC1100000880.mm.1	2,15	Myh10	PSR1100007366.mm.1	-2,67	0,046979	0,557653	Cassette Exon	0,16
TC1100000880.mm.1	2,15	Myh10	JUC1100003903.mm.1	-2,83	0,043863	0,550501		
TC1100000880.mm.1	2,15	Myh10	JUC1100003889.mm.1	-2,87	0,036872	0,532257		
TC1100000880.mm.1	2,15	Myh10	JUC1100003901.mm.1	-2,91	0,000602	0,304044		
TC1100000880.mm.1	2,15	Myh10	PSR1100007339.mm.1	-3,04	0,019052	0,471978	Cassette Exon	0,2
TC1100000880.mm.1	2,15	Myh10	PSR1100007322.mm.1	-3,51	0,01206	0,432426	Cassette Exon	0,17
TC1100000880.mm.1	2,15	Myh10	PSR1100007365.mm.1	-4,16	0,01369	0,444581	Cassette Exon	0,33
TC0400002918.mm.1	1,36	Mpdz	PSR0400023685.mm.1	-2,02	0,015815	0,45594	Alternative 5' Donc	0,14
TC0400002918.mm.1	1,36	Mpdz	PSR0400023703.mm.1	-2,04	0,010964	0,425117	Cassette Exon	0,3
TC0400002918.mm.1	1,36	Mpdz	JUC0400012388.mm.1	-2,16	0,02939	0,509995		
TC0400002918.mm.1	1,36	Mpdz	JUC0400012359.mm.1	-2,17	0,019999	0,476227		
TC0400002918.mm.1	1,36	Mpdz	JUC0400012392.mm.1	-3,73	0,005722	0,382767		
TC0400002918.mm.1	1,36	Mpdz	JUC0400012387.mm.1	-4,17	0,002465	0,349135		
TC1300002357.mm.1	2,27	Gpr98	PSR1300015619.mm.1	-2,02	0,013282	0,441719	Cassette Exon	0,04
TC1300002357.mm.1	2,27	Gpr98	PSR1300015683.mm.1	-2,18	0,032333	0,519155	Alternative 5' Donc	0,12
TC1300002357.mm.1	2,27	Gpr98	PSR1300015716.mm.1	-2,32	0,040828	0,543283	Alternative 5' Donc	0,19
TC1300002357.mm.1	2,27	Gpr98	JUC1300008281.mm.1	-2,37	0,041424	0,544646		
TC1300002357.mm.1	2,27	Gpr98	PSR1300015631.mm.1	-2,72	0,013943	0,446234	Cassette Exon	0,14
TC1300002357.mm.1	2,27	Gpr98	JUC1300008293.mm.1	-3,12	0,035572	0,528808		

TC1300002357.mm.1	2,27	Gpr98	JUC1300008233.mm.1	-3,28	0,013058	0,439579		
TC1300002357.mm.1	2,27	Gpr98	PSR1300015634.mm.1	-4,37	0,014905	0,451803	Cassette Exon	0,3
TC0400003530.mm.1	1,44	Thrap3	JUC0400014971.mm.1	-2,02	0,009319	0,414883		
TC0400003530.mm.1	1,44	Thrap3	PSR0400028681.mm.1	-2,23	0,023666	0,491379	Intron Retention	0,28
TC0800002290.mm.1	1,56	Clcn3	PSR0800017287.mm.1	-2,02	0,021297	0,481714	Intron Retention	0,26
TC0800002290.mm.1	1,56	Clcn3	PSR0800017278.mm.1	-2,35	0,037018	0,532502	Cassette Exon	0,12
TC0200003401.mm.1	2,07	Strbp	PSR0200029250.mm.1	-2,02	0,026177	0,50003	Cassette Exon	0,11
TC0200003401.mm.1	2,07	Strbp	JUC0200014756.mm.1	-2,03	0,043056	0,54841		
TC0200003401.mm.1	2,07	Strbp	PSR0200029245.mm.1	-2,1	0,038415	0,536405	Cassette Exon	0,14
TC0200003401.mm.1	2,07	Strbp	PSR0200029225.mm.1	-2,26	0,008675	0,408783	Cassette Exon	0,12
TC0200003401.mm.1	2,07	Strbp	PSR0200029249.mm.1	-2,29	0,01999	0,476227	Alternative 5' Donc	0,09
TC0200003401.mm.1	2,07	Strbp	PSR0200029247.mm.1	-2,42	0,00982	0,417839	Cassette Exon	0,19
TC0200003401.mm.1	2,07	Strbp	JUC0200014759.mm.1	-2,61	0,012006	0,432286		
TC0200003401.mm.1	2,07	Strbp	PSR0200029222.mm.1	-2,66	0,021702	0,484104	Cassette Exon	0,18
TC0200003401.mm.1	2,07	Strbp	PSR0200029210.mm.1	-3,56	0,003134	0,353892	Cassette Exon	0,24
TC0500000724.mm.1	1,36	Cep135	PSR0500006491.mm.1	-2,02	0,016637	0,459985	Intron Retention	0,24
TC0500000724.mm.1	1,36	Cep135	JUC0500003563.mm.1	-3,06	0,027155	0,503174		
TC1000001688.mm.1	1,48	Rgs17	PSR1000012313.mm.1	-2,02	0,003538	0,3562	Cassette Exon	0,14
TC1000001688.mm.1	1,48	Rgs17	PSR1000012305.mm.1	-2,17	0,006594	0,39213	Alternative 3' Acce	0,19
TC1000001688.mm.1	1,48	Rgs17	PSR1000012311.mm.1	-3,84	0,011522	0,428397	Cassette Exon	0,24
TC1800001017.mm.1	2,91	Aqp4	PSR1800007582.mm.1	-2,02	0,0324	0,519282	Cassette Exon	0,17
TC1800001017.mm.1	2,91	Aqp4	PSR1800007580.mm.1	-2,49	0,017376	0,463612	Cassette Exon	0,24
TC1800001017.mm.1	2,91	Aqp4	PSR1800007576.mm.1	-2,95	0,043939	0,550715	Cassette Exon	0,11
TC1800001017.mm.1	2,91	Aqp4	PSR1800007575.mm.1	-2,96	0,037426	0,533831	Cassette Exon	0,17
TC1800001017.mm.1	2,91	Aqp4	PSR1800007578.mm.1	-3,01	0,018702	0,470452	Cassette Exon	0,11
TC1800001017.mm.1	2,91	Aqp4	PSR1800007577.mm.1	-3,6	0,026673	0,501957	Cassette Exon	0,11
TC1800001017.mm.1	2,91	Aqp4	PSR1800007581.mm.1	-4,92	0,014677	0,450148	Cassette Exon	0,24
TC0100003865.mm.1	1,4	Armc9	PSR0100006581.mm.1	-2,02	0,006015	0,386586	Alternative 5' Donc	0,13
TC0100003865.mm.1	1,4	Armc9	JUC0100003776.mm.1	-2,43	0,03572	0,529027		
TC0100003865.mm.1	1,4	Armc9	JUC0100003768.mm.1	-2,46	0,013173	0,440567		
TC0100003865.mm.1	1,4	Armc9	PSR0100006566.mm.1	-3,08	0,005719	0,382767	Cassette Exon	0,22
TC0300001599.mm.1	1,92	2700069I18Rik	PSR0300012829.mm.1	-2,02	0,024724	0,494838	Cassette Exon	0,16
TC0300001599.mm.1	1,92	2700069I18Rik	PSR0300012832.mm.1	-2,14	0,006746	0,393343	Cassette Exon	0,11
TC0300001599.mm.1	1,92	2700069I18Rik	PSR0300012831.mm.1	-2,95	0,005534	0,380557	Cassette Exon	0,19
TC0300001599.mm.1	1,92	2700069I18Rik	PSR0300012827.mm.1	-3,37	0,007333	0,399002	Alternative 3' Acce	0,22
TC0400002769.mm.1	1,04	Mup19; Mup13; LOC1000	PSR0400022640.mm.1	-2,02	0,006876	0,394271	Cassette Exon	0,22
TC0900001632.mm.1	2,38	1700048O20Rik	PSR0900013685.mm.1	-2,02	0,049377	0,562864	Cassette Exon	0,14
TC0900001632.mm.1	2,38	1700048O20Rik	JUC0900007694.mm.1	-2,36	0,016528	0,459611		
TC0900001632.mm.1	2,38	1700048O20Rik	PSR0900013684.mm.1	-3	0,018011	0,466939	Cassette Exon	0,22
TC0700001406.mm.1	1,35	C2cd3	JUC0700006227.mm.1	-2,02	0,008899	0,410728		
TC0700001406.mm.1	1,35	C2cd3	JUC0700006222.mm.1	-2,23	0,001978	0,340886		
TC0700001406.mm.1	1,35	C2cd3	PSR0700011964.mm.1	-2,75	0,004412	0,366761	Cassette Exon	0,21
TC0700003100.mm.1	1,07	Nipa2	JUC0700015033.mm.1	-2,02	0,007458	0,400326		
TC0700003100.mm.1	1,07	Nipa2	PSR0700028151.mm.1	-2,87	0,036118	0,529768	Cassette Exon	0,2
TC0400001482.mm.1	1,3	Gm12942; Zmym6	PSR0400011351.mm.1	-2,02	0,025716	0,498173	Cassette Exon	0,13
TC0400001482.mm.1	1,3	Gm12942; Zmym6	PSR0400011353.mm.1	-2,06	0,04305	0,54841	Cassette Exon	0,19
TC0400001482.mm.1	1,3	Gm12942; Zmym6	JUC0400005894.mm.1	-2,22	0,036878	0,532257		
TC0400001482.mm.1	1,3	Gm12942; Zmym6	JUC0400005882.mm.1	-2,76	0,02194	0,485023		

TC0600003387.mm.1	1,54	C2cd5	PSR0600026725.mm.1	-2,02	0,002836	0,352207	Alternative 3' Acce	0,18
TC1000002277.mm.1	-1,49	Mypn	PSR1000016346.mm.1	-2,02	0,023862	0,492103	Cassette Exon	0,18
TC1000002277.mm.1	-1,49	Mypn	JUC1000008962.mm.1	-2,13	0,044357	0,551441		
TC1200001599.mm.1	2,07	Gpr22	PSR1200011112.mm.1	-2,02	0,009885	0,418122	Intron Retention	0,17
TC1200001599.mm.1	2,07	Gpr22	JUC1200006137.mm.1	-3,02	0,012219	0,433326		
TC1200001599.mm.1	2,07	Gpr22	JUC1200006138.mm.1	-4,44	0,01043	0,421581		
TC0300000302.mm.1	1,05		PSR0300002156.mm.1	-2,02	0,001224	0,322251		
TC0300000302.mm.1	1,05		PSR0300002155.mm.1	-2,41	0,019579	0,474672	Cassette Exon	0,15
TC0700002736.mm.1	1,53	Zfp27	PSR0700024220.mm.1	-2,02	0,014244	0,447947	Alternative 3' Acce	0,15
TC0900001866.mm.1	1,56	Kri1	JUC0900008843.mm.1	-2,02	0,032526	0,519918		
TC0900001866.mm.1	1,56	Kri1	PSR0900015706.mm.1	-2,16	0,030095	0,511942	Alternative 3' Acce	0,12
TC0900001866.mm.1	1,56	Kri1	PSR0900015687.mm.1	-2,28	0,022058	0,485327	Alternative 3' Acce	0,15
TC0900001866.mm.1	1,56	Kri1	JUC0900008859.mm.1	-2,37	0,009778	0,417839		
TC0900001866.mm.1	1,56	Kri1	JUC0900008842.mm.1	-2,87	0,003493	0,355638		
TC0800003022.mm.1	1,22	Mbtps1	JUC0800012721.mm.1	-2,02	0,03231	0,519155		
TC0800003022.mm.1	1,22	Mbtps1	PSR0800023229.mm.1	-2,03	0,007575	0,401691	Cassette Exon	0,14
TC0100002831.mm.1	1,63	Mterfd2	PSR0100022911.mm.1	-2,02	0,041874	0,545696	Alternative 3' Acce	0,13
TC0100002831.mm.1	1,63	Mterfd2	JUC0100013025.mm.1	-2,62	0,036622	0,531548		
TC0800000509.mm.1	-1,28	Fat1	PSR0800003508.mm.1	-2,02	0,021741	0,484249	Cassette Exon	0,13
TC0800001193.mm.1	-1,1	Ces2b	PSR0800009262.mm.1	-2,02	0,011577	0,429122	Alternative 3' Acce	0,13
TC0800001193.mm.1	-1,1	Ces2b	JUC0800004959.mm.1	-4,13	0,039065	0,538366		
TC0X00001797.mm.1	1,09	Rbm3; Gm15453	JUC0X00005810.mm.1	-2,02	0,047911	0,559424		
TC0X00001797.mm.1	1,09	Rbm3; Gm15453	PSR0X00011483.mm.1	-2,1	0,041646	0,545276	Alternative 3' Acce	0,13
TC1200002256.mm.1	-1,05	Btbd7	PSR1200015676.mm.1	-2,02	0,014579	0,449876	Cassette Exon	0,13
TC1200002256.mm.1	-1,05	Btbd7	JUC1200008649.mm.1	-2,57	0,048525	0,560991		
TC1300000781.mm.1	1,02	Cdk20	PSR1300005179.mm.1	-2,02	0,023602	0,490995	Cassette Exon	0,13
TC1300000781.mm.1	1,02	Cdk20	JUC1300002709.mm.1	-2,05	0,018433	0,468646		
TC0200005054.mm.1	-1,01	Gm14221	PSR0200043192.mm.1	-2,02	0,046654	0,556812	Cassette Exon	0,12
TC0X00002272.mm.1	1,62	Rbmx	PSR0X00014437.mm.1	-2,02	0,008024	0,404268	Alternative 3' Acce	0,12
TC0X00002272.mm.1	1,62	Rbmx	JUC0X00007383.mm.1	-2,51	0,034452	0,525886		
TC0100001950.mm.1	3,87	Ppp1r42	PSR0100015991.mm.1	-2,02	0,030318	0,51274	Cassette Exon	0,11
TC0100001950.mm.1	3,87	Ppp1r42	PSR0100015990.mm.1	-2,16	0,013248	0,441416	Cassette Exon	0,11
TC0100001950.mm.1	3,87	Ppp1r42	JUC0100009067.mm.1	-4,09	0,027537	0,504345		
TC0200003017.mm.1	-1,12	Nebl	PSR0200024879.mm.1	-2,02	0,046577	0,556761	Cassette Exon	0,11
TC1100004294.mm.1	-1,9	Glod4; Gemin4; Gm6330	PSR1100029837.mm.1	-2,02	0,007593	0,401712	Alternative 3' Acce	0,11
TC0700000498.mm.1	1,15	Paf1	PSR0700004022.mm.1	-2,02	0,045366	0,554024	Cassette Exon	0,1
TC0900003336.mm.1	1,07	Cdhr4	PSR0900028046.mm.1	-2,02	0,00756	0,401384	Cassette Exon	0,09
TC0900003336.mm.1	1,07	Cdhr4	PSR0900011079.mm.1	-2,14	0,035791	0,529153	Alternative 5' Donc	0,05
TC0900003336.mm.1	1,07	Cdhr4	PSR0900011089.mm.1	-2,21	0,033922	0,52424	Alternative 5' Donc	0,1
TC0900003336.mm.1	1,07	Cdhr4	JUC0900006083.mm.1	-3,58	0,016291	0,458454		
TC1300000135.mm.1	2,24	Amph	PSR1300000911.mm.1	-2,02	0,000836	0,311886	Cassette Exon	0,1
TC1300000135.mm.1	2,24	Amph	JUC1300000552.mm.1	-2,07	0,013005	0,439291		
TC1300000135.mm.1	2,24	Amph	JUC1300000534.mm.1	-2,1	0,023324	0,490065		
TC1300000135.mm.1	2,24	Amph	JUC1300000553.mm.1	-2,62	0,008895	0,410717		
TC1300000135.mm.1	2,24	Amph	JUC1300000546.mm.1	-2,84	0,034913	0,526996		
TC1400001469.mm.1	-1,27	Gm281	JUC1400005933.mm.1	-2,02	0,032833	0,520898		
TC1400001469.mm.1	-1,27	Gm281	PSR1400011024.mm.1	-2,25	0,001179	0,319796	Cassette Exon	0,1
TC1700000713.mm.1	-1,07	Gm8801	PSR1700007359.mm.1	-2,02	0,017644	0,464582	Cassette Exon	0,1

TC0200003210.mm.1	1,02	Tmem8c	PSR0200027029.mm.1	-2,02	0,028376	0,506902	Cassette Exon	0,09
TC0300000749.mm.1	-1,23	Fam189b	PSR0300005587.mm.1	-2,02	0,029507	0,510135	Cassette Exon	0,09
TC0700001046.mm.1	-1,08		PSR0700009459.mm.1	-2,02	0,02758	0,504482	Cassette Exon	0,09
TC1000000735.mm.1	-1,14	Slc5a4a	PSR1000005255.mm.1	-2,02	0,049816	0,563753	Cassette Exon	0,09
TC0700000564.mm.1	-1,02	Prodh2	PSR0700004965.mm.1	-2,02	0,012896	0,438165	Cassette Exon	0,08
TC1300002661.mm.1	1,32	Gpbp1	PSR1300018001.mm.1	-2,02	0,022828	0,488339	Cassette Exon	0,08
TC1400001420.mm.1	-1,03	Gm6676; Gm16434	PSR1400010671.mm.1	-2,02	0,029054	0,508954	Cassette Exon	0,08
TC1400001421.mm.1	-1,03	Gm16434; Gm6676	PSR1400010679.mm.1	-2,02	0,029054	0,508954	Cassette Exon	0,08
TC0600000158.mm.1	1,68	Kcnd2	PSR0600001088.mm.1	-2,02	0,007599	0,401712	Cassette Exon	0,07
TC1200001886.mm.1	1,49	Trim9	JUC1200007198.mm.1	-2,02	0,001673	0,335182		
TC1200001549.mm.1	1,38	Rnf144a	JUC1200005916.mm.1	-2,02	0,002907	0,352262		
TC0200004251.mm.1	-1,26	Prrg4	JUC0200018298.mm.1	-2,02	0,008283	0,406032		
TC1300000065.mm.1	1,12	Gm26861	JUC1300000244.mm.1	-2,02	0,028595	0,507685		
TC1200002428.mm.1	-1,11	A730018C14Rik	JUC1200009284.mm.1	-2,02	0,027329	0,503766		
TC1200002428.mm.1	-1,11	A730018C14Rik	JUC1200009286.mm.1	-2,14	0,029289	0,509874		
TC1200002339.mm.1	-1,15	Bcl11b	JUC1200008998.mm.1	-2,02	0,041769	0,545539		
TC1300000694.mm.1	-1,09	Smad5	JUC1300002409.mm.1	-2,02	0,011219	0,426683		
TC1700002018.mm.1	1,12	Trim39	JUC1700010305.mm.1	-2,02	0,040227	0,541714		
TC0200001764.mm.1	1,04	Zfyve19	JUC0200006577.mm.1	-2,02	0,007931	0,403819		
TC0200001764.mm.1	1,04	Zfyve19	JUC0200006576.mm.1	-2,32	0,028182	0,506368		
TC0200001764.mm.1	1,04	Zfyve19	JUC0200006573.mm.1	-2,49	0,005735	0,382993		
TC0200002337.mm.1	-1,71	Bpifb1	JUC0200009360.mm.1	-2,02	0,040887	0,543423		
TC1700000476.mm.1	1,29	Fance	JUC1700002258.mm.1	-2,02	0,035502	0,528731		
TC1700000476.mm.1	1,29	Fance	JUC1700002255.mm.1	-2,06	0,009801	0,417839		
TC1700000476.mm.1	1,29	Fance	JUC1700002253.mm.1	-2,21	0,025611	0,497893		
TC1700000476.mm.1	1,29	Fance	JUC1700002242.mm.1	-2,4	0,000406	0,297771		
TC0800001019.mm.1	-1,27	Gm2694	JUC0800004140.mm.1	-2,02	0,042333	0,547323		
TC0500002845.mm.1	1,47	Helq	JUC0500013640.mm.1	-2,02	0,029928	0,511499		
TC0500002818.mm.1	-1,29	Rasgef1b	JUC0500013462.mm.1	-2,02	0,040252	0,541756		
TC0800000207.mm.1	2,04	Gm20946	JUC0800000993.mm.1	-2,02	0,005006	0,373851		
TC0900001635.mm.1	1,06	Snrk	JUC0900007717.mm.1	-2,02	0,044387	0,551441		
TC0500001066.mm.1	1,71	Cdc7	JUC0500004902.mm.1	-2,02	0,044373	0,551441		
TC0500001066.mm.1	1,71	Cdc7	JUC0500004908.mm.1	-2,2	0,041683	0,545356		
TC0500001066.mm.1	1,71	Cdc7	JUC0500004912.mm.1	-2,2	0,027706	0,504872		
TC0600000651.mm.1	-1,33	Wipf3	JUC0600002498.mm.1	-2,02	0,022941	0,488667		
TC0600002161.mm.1	-1,02	Kel	JUC0600008966.mm.1	-2,02	0,038249	0,535883		
TC0600003463.mm.1	-1,21		JUC0600014357.mm.1	-2,02	0,006713	0,393343		
TC0700002737.mm.1	-1,03		JUC0700012940.mm.1	-2,02	0,001118	0,317328		
TC1000002992.mm.1	1,26	Tmevpg1	JUC1000012282.mm.1	-2,02	0,046348	0,556272		
TC1000002992.mm.1	1,26	Tmevpg1	PSR1000022404.mm.1	-2,04	0,021974	0,485125		
TC1000002002.mm.1	1,06		JUC1000007756.mm.1	-2,02	0,03784	0,534803		
TC0300002680.mm.1	1,19	I830077J02Rik	JUC0300010844.mm.1	-2,02	0,008436	0,406611		
TC1100002114.mm.1	1,19	Mtmr3	JUC1100010746.mm.1	-2,02	0,003677	0,357266		
TC0X00000122.mm.1	1,21	Sytl5	JUC0X00000387.mm.1	-2,02	0,045024	0,553022		
TC0900002823.mm.1	1,4	Tbc1d2b	JUC0900013120.mm.1	-2,02	0,013392	0,442449		
TC0X00003079.mm.1	-1,16	Tex13	JUC0X00009783.mm.1	-2,02	0,000214	0,28803		
TC0X00003307.mm.1	1,11	Gm15201	JUC0X00010410.mm.1	-2,02	0,03832	0,536178		
TC0600001244.mm.1	2,28	Cpne9	PSR0600009570.mm.1	-2,03	0,013752	0,444758	Alternative 5' Donc	0,22

TC0600001244.mm.1	2,28	Cpne9	JUC0600004936.mm.1	-2,18	0,03052	0,51337		
TC0600001244.mm.1	2,28	Cpne9	PSR0600009574.mm.1	-2,23	0,008958	0,411476	Alternative 5' Donc	0,22
TC0600001244.mm.1	2,28	Cpne9	PSR0600009572.mm.1	-2,24	0,022774	0,488081	Alternative 5' Donc	0,22
TC0600001244.mm.1	2,28	Cpne9	PSR0600009582.mm.1	-2,26	0,002227	0,345773	Intron Retention	0,26
TC0600001244.mm.1	2,28	Cpne9	JUC0600004930.mm.1	-2,55	0,010847	0,424161		
TC0600001244.mm.1	2,28	Cpne9	PSR0600009595.mm.1	-2,86	0,007126	0,397139	Alternative 5' Donc	0,26
TC0600001244.mm.1	2,28	Cpne9	PSR0600009555.mm.1	-2,87	0,011212	0,426683	Alternative 3' Acce	0,32
TC0600001244.mm.1	2,28	Cpne9	PSR0600009556.mm.1	-2,89	0,020532	0,478295	Alternative 3' Acce	0,32
TC0600001244.mm.1	2,28	Cpne9	PSR0600009549.mm.1	-2,94	0,013952	0,446239	Alternative 3' Acce	0,01
TC0600001244.mm.1	2,28	Cpne9	JUC0600004937.mm.1	-2,97	0,037004	0,532502		
TC0600001244.mm.1	2,28	Cpne9	PSR0600009559.mm.1	-3,04	0,032922	0,521149	Intron Retention	0,45
TC0600001244.mm.1	2,28	Cpne9	PSR0600009564.mm.1	-3,09	0,008492	0,406966	Intron Retention	0,5
TC0600001244.mm.1	2,28	Cpne9	JUC0600004923.mm.1	-3,11	0,039047	0,538264		
TC0600001244.mm.1	2,28	Cpne9	PSR0600009557.mm.1	-3,38	0,006557	0,39213	Alternative 3' Acce	0,32
TC0600001244.mm.1	2,28	Cpne9	PSR0600009575.mm.1	-3,76	0,000704	0,305967	Alternative 3' Acce	0,29
TC0600001244.mm.1	2,28	Cpne9	PSR0600009584.mm.1	-3,95	0,000682	0,304417	Intron Retention	0,74
TC0600001244.mm.1	2,28	Cpne9	PSR0600009565.mm.1	-4,69	0,005535	0,380557	Intron Retention	0,5
TC0600001244.mm.1	2,28	Cpne9	JUC0600004946.mm.1	-5,95	0,004092	0,36228		
TC0400001035.mm.1	2,18	Dnajc6	PSR0400007032.mm.1	-2,03	0,038481	0,53651	Alternative 3' Acce	0,13
TC0400001035.mm.1	2,18	Dnajc6	PSR0400007014.mm.1	-2,11	0,008903	0,410728	Cassette Exon	0,11
TC0400001035.mm.1	2,18	Dnajc6	JUC0400003607.mm.1	-2,15	0,023223	0,490032		
TC0400001035.mm.1	2,18	Dnajc6	JUC0400003600.mm.1	-2,35	0,006026	0,386586		
TC0400001035.mm.1	2,18	Dnajc6	PSR0400007017.mm.1	-2,97	0,006422	0,389915	Intron Retention	0,49
TC0300001857.mm.1	3,28	Bbs7	JUC0300007585.mm.1	-2,03	0,030739	0,514065		
TC0300001857.mm.1	3,28	Bbs7	PSR0300014388.mm.1	-2,47	0,035392	0,528596	Alternative 3' Acce	0,24
TC0300001857.mm.1	3,28	Bbs7	PSR0300014398.mm.1	-3,98	0,012715	0,43654	Alternative 5' Donc	0,47
TC0300001857.mm.1	3,28	Bbs7	JUC0300007587.mm.1	-4,09	0,016367	0,458693		
TC0300001857.mm.1	3,28	Bbs7	JUC0300007588.mm.1	-8,88	0,019164	0,472719		
TC0X00001413.mm.1	3,33	Pak3	PSR0X00008967.mm.1	-2,03	0,048805	0,561503	Cassette Exon	0,09
TC0X00001413.mm.1	3,33	Pak3	JUC0X00004423.mm.1	-2,05	0,030129	0,512017		
TC0X00001413.mm.1	3,33	Pak3	JUC0X00004439.mm.1	-2,11	0,013701	0,444746		
TC0X00001413.mm.1	3,33	Pak3	PSR0X00008968.mm.1	-2,26	0,00021	0,28803	Cassette Exon	0,09
TC0X00001413.mm.1	3,33	Pak3	PSR0X00008939.mm.1	-2,6	0,004645	0,370181	Cassette Exon	0,17
TC0X00001413.mm.1	3,33	Pak3	JUC0X00004441.mm.1	-2,81	0,039247	0,53893		
TC0X00001413.mm.1	3,33	Pak3	PSR0X00008972.mm.1	-2,91	0,009622	0,417315	Cassette Exon	0,17
TC0X00001413.mm.1	3,33	Pak3	PSR0X00008960.mm.1	-3	0,003448	0,3554	Alternative 5' Donc	0,27
TC0X00001413.mm.1	3,33	Pak3	PSR0X00008938.mm.1	-3,07	0,008846	0,410308	Cassette Exon	0,17
TC0X00001413.mm.1	3,33	Pak3	PSR0X00008956.mm.1	-3,18	0,006731	0,393343	Cassette Exon	0,05
TC0X00001413.mm.1	3,33	Pak3	JUC0X00004443.mm.1	-3,25	0,010841	0,424161		
TC0X00001413.mm.1	3,33	Pak3	PSR0X00008952.mm.1	-3,39	0,037011	0,532502	Cassette Exon	0,19
TC0X00001413.mm.1	3,33	Pak3	PSR0X00008947.mm.1	-3,48	0,001225	0,322251	Cassette Exon	0,15
TC0X00001413.mm.1	3,33	Pak3	JUC0X00004437.mm.1	-3,68	0,036097	0,529729		
TC0X00001413.mm.1	3,33	Pak3	PSR0X00008954.mm.1	-4,05	0,012306	0,434378	Cassette Exon	0,22
TC0X00001413.mm.1	3,33	Pak3	PSR0X00008946.mm.1	-4,06	0,004267	0,363868	Alternative 3' Acce	0,01
TC0X00001413.mm.1	3,33	Pak3	PSR0X00008953.mm.1	-4,41	0,002636	0,349612	Alternative 5' Donc	0,1
TC0X00001413.mm.1	3,33	Pak3	PSR0X00008949.mm.1	-4,49	0,005896	0,383835	Cassette Exon	0,18
TC0X00001413.mm.1	3,33	Pak3	PSR0X00008955.mm.1	-4,72	0,000316	0,288663	Cassette Exon	0,32
TC0X00001413.mm.1	3,33	Pak3	JUC0X00004434.mm.1	-4,75	0,011596	0,429347		

TC0X00001413.mm.1	3,33	Pak3	PSR0X00008948.mm.1	-5,21	0,00395	0,359922	Intron Retention	0,07
TC0X00001413.mm.1	3,33	Pak3	PSR0X00008942.mm.1	-5,32	0,005819	0,383169	Cassette Exon	0,39
TC0X00001413.mm.1	3,33	Pak3	PSR0X00008940.mm.1	-5,46	0,019158	0,472719	Cassette Exon	0,26
TC0X00001413.mm.1	3,33	Pak3	PSR0X00008950.mm.1	-5,65	0,001017	0,316361	Intron Retention	0,08
TC0X00001413.mm.1	3,33	Pak3	JUC0X00004436.mm.1	-6,03	0,010698	0,423322		
TC0X00001413.mm.1	3,33	Pak3	JUC0X00004432.mm.1	-6,29	0,003231	0,354243		
TC0X00001413.mm.1	3,33	Pak3	JUC0X00004448.mm.1	-7,05	0,003872	0,358883		
TC0X00001413.mm.1	3,33	Pak3	PSR0X00008962.mm.1	-7,57	0,004813	0,371408	Cassette Exon	0,46
TC0X00001413.mm.1	3,33	Pak3	PSR0X00008943.mm.1	-13,65	0,002962	0,352501	Cassette Exon	0,21
TC0200005183.mm.1	8,37	Kcnb1; Gm14290	PSR0200044364.mm.1	-2,03	0,013946	0,446234	Intron Retention	0,25
TC0200005183.mm.1	8,37	Kcnb1; Gm14290	PSR0200044358.mm.1	-2,27	0,027382	0,503961	Alternative 3' Acce	0,16
TC0200005183.mm.1	8,37	Kcnb1; Gm14290	PSR0200044360.mm.1	-2,79	0,037418	0,533831	Intron Retention	0,44
TC0200005183.mm.1	8,37	Kcnb1; Gm14290	PSR0200044374.mm.1	-7,09	0,00463	0,369836		
TC0200005183.mm.1	8,37	Kcnb1; Gm14290	JUC0200022899.mm.1	-10,39	0,000798	0,31174		
TC0200005183.mm.1	8,37	Kcnb1; Gm14290	JUC0200022897.mm.1	-16,76	0,000929	0,313363		
TC0200005183.mm.1	8,37	Kcnb1; Gm14290	PSR0200044380.mm.1	-18,68	0,005136	0,376442	Cassette Exon	0,28
TC1000001949.mm.1	3	Hey2	PSR1000014039.mm.1	-2,03	0,029804	0,511132	Cassette Exon	0,14
TC1000001949.mm.1	3	Hey2	PSR1000014043.mm.1	-2,72	0,016337	0,458616	Intron Retention	0,24
TC1000001949.mm.1	3	Hey2	PSR1000014048.mm.1	-3,57	0,037152	0,533065	Cassette Exon	0,25
TC1000001949.mm.1	3	Hey2	PSR1000014045.mm.1	-3,81	0,022786	0,488142	Alternative 5' Donc	0,31
TC1000001949.mm.1	3	Hey2	PSR1000014046.mm.1	-3,82	0,039772	0,540537	Cassette Exon	0,27
TC1000001949.mm.1	3	Hey2	PSR1000014041.mm.1	-4,02	0,011624	0,429746	Alternative 3' Acce	0,35
TC1100000442.mm.1	1,91	Ebf1	PSR1100003446.mm.1	-2,03	0,045886	0,555077	Alternative 5' Donc	0,1
TC1100000442.mm.1	1,91	Ebf1	PSR1100003447.mm.1	-2,12	0,006759	0,393343	Alternative 3' Acce	0,17
TC1100000442.mm.1	1,91	Ebf1	PSR1100003451.mm.1	-2,39	0,037855	0,534804	Alternative 5' Donc	0,21
TC1100000442.mm.1	1,91	Ebf1	PSR1100003464.mm.1	-2,62	0,0356	0,528887	Cassette Exon	0,14
TC1100000442.mm.1	1,91	Ebf1	JUC1100001826.mm.1	-3,04	0,039717	0,540494		
TC1100000442.mm.1	1,91	Ebf1	PSR1100003474.mm.1	-3,12	0,023262	0,49004	Alternative 5' Donc	0,31
TC1200002012.mm.1	1,88	Slc8a3	PSR1200013845.mm.1	-2,03	0,014341	0,448173	Cassette Exon	0,09
TC1200002012.mm.1	1,88	Slc8a3	PSR1200013850.mm.1	-2,26	0,038876	0,537855	Cassette Exon	0,27
TC1200002012.mm.1	1,88	Slc8a3	PSR1200013847.mm.1	-2,31	0,035845	0,529376	Alternative 3' Acce	0,21
TC1200002012.mm.1	1,88	Slc8a3	PSR1200013853.mm.1	-2,39	0,011384	0,427549	Cassette Exon	0,12
TC1200002012.mm.1	1,88	Slc8a3	JUC1200007652.mm.1	-2,97	0,000332	0,28927		
TC0X00002513.mm.1	1,02	Prrg1	PSR0X00016043.mm.1	-2,03	0,002959	0,352501	Alternative 3' Acce	0,03
TC0X00002513.mm.1	1,02	Prrg1	PSR0X00016060.mm.1	-2,5	0,006292	0,388979	Cassette Exon	0,1
TC0X00002513.mm.1	1,02	Prrg1	PSR0X00016049.mm.1	-2,54	0,024865	0,495577	Alternative 5' Donc	0,25
TC0900001555.mm.1	1,6	Azi2	PSR0900012880.mm.1	-2,03	0,030754	0,514087	Alternative 5' Donc	0,14
TC0900001555.mm.1	1,6	Azi2	PSR0900012885.mm.1	-2,29	0,00138	0,32572	Intron Retention	0,23
TC0900001555.mm.1	1,6	Azi2	PSR0900012896.mm.1	-2,68	0,048115	0,559875	Alternative 5' Donc	0,23
TC0900001555.mm.1	1,6	Azi2	JUC0900007229.mm.1	-3,51	0,02738	0,503961		
TC0900002944.mm.1	-1,35	Ppp2r3a	PSR0900024258.mm.1	-2,03	0,020984	0,480104	Cassette Exon	0,22
TC0900002944.mm.1	-1,35	Ppp2r3a	JUC0900013543.mm.1	-4,5	0,00729	0,398842		
TC0600003208.mm.1	1,58	BC048546	PSR0600025074.mm.1	-2,03	0,026056	0,499575	Cassette Exon	0,11
TC0600003208.mm.1	1,58	BC048546	JUC0600013055.mm.1	-2,16	0,018586	0,469618		
TC0600003208.mm.1	1,58	BC048546	PSR0600025091.mm.1	-2,2	0,036117	0,529765	Intron Retention	0,21
TC0600003208.mm.1	1,58	BC048546	PSR0600025083.mm.1	-2,2	0,004421	0,366832	Cassette Exon	0,13
TC0600003208.mm.1	1,58	BC048546	PSR0600025088.mm.1	-2,44	0,048527	0,560991	Cassette Exon	0,15
TC0600003208.mm.1	1,58	BC048546	JUC0600013067.mm.1	-3,6	0,011249	0,426718		

TC0700003345.mm.1	2,63	D7Ertd715e	PSR0700028673.mm.1	-2,03	0,012568	0,435803	Intron Retention	0,2
TC0700003345.mm.1	2,63	D7Ertd715e	PSR0700028667.mm.1	-2,15	0,031172	0,515739	Alternative 3' Acce	0,15
TC0700003345.mm.1	2,63	D7Ertd715e	PSR0700028657.mm.1	-2,81	0,022889	0,488524		
TC0700003345.mm.1	2,63	D7Ertd715e	PSR0700028659.mm.1	-5,03	0,004475	0,367858		
TC0200001015.mm.1	1,73	Ubr3	JUC0200004134.mm.1	-2,03	0,006174	0,387873		
TC0200001015.mm.1	1,73	Ubr3	PSR0200008055.mm.1	-2,51	0,015835	0,456151	Alternative 5' Donc	0,19
TC0100000424.mm.1	-1,17	Spats2l	PSR0100003262.mm.1	-2,03	0,017417	0,46377	Cassette Exon	0,18
TC0100000424.mm.1	-1,17	Spats2l	JUC0100001891.mm.1	-2,43	0,003097	0,353892		
TC0200001011.mm.1	1,6	Phospho2	PSR0200007978.mm.1	-2,03	0,021305	0,481714	Alternative 3' Acce	0,17
TC0200001011.mm.1	1,6	Phospho2	JUC0200004074.mm.1	-2,38	0,047517	0,558686		
TC0200001011.mm.1	1,6	Phospho2	PSR0200007988.mm.1	-2,4	0,035524	0,528764	Cassette Exon	0,13
TC1400000968.mm.1	1,29	Stc1	PSR1400007761.mm.1	-2,03	0,014692	0,450253	Alternative 5' Donc	0,17
TC0200002691.mm.1	1,25	Rab22a	PSR0200021960.mm.1	-2,03	0,001636	0,332976	Alternative 3' Acce	0,16
TC0300003176.mm.1	1,53	Tyw3	PSR0300024910.mm.1	-2,03	0,00185	0,337414	Alternative 3' Acce	0,16
TC0X00001859.mm.1	-1,7	Med14	PSR0X00011943.mm.1	-2,03	0,004729	0,370888	Alternative 3' Acce	0,16
TC1100000500.mm.1	1,23	Mgat1	JUC1100002014.mm.1	-2,03	0,026653	0,501823		
TC1100000500.mm.1	1,23	Mgat1	PSR1100003869.mm.1	-2,51	0,035753	0,529077	Cassette Exon	0,16
TC1500002100.mm.1	1,33	Prickle1	PSR1500016888.mm.1	-2,03	0,014122	0,447137	Alternative 5' Donc	0,16
TC1600000480.mm.1	1,44	Itgb5	PSR1600004567.mm.1	-2,03	0,023687	0,491379	Alternative 5' Donc	0,16
TC1600000480.mm.1	1,44	Itgb5	JUC1600002382.mm.1	-3,35	0,041368	0,544518		
TC0200002717.mm.1	1,29		PSR0200022215.mm.1	-2,03	0,003406	0,355152	Alternative 5' Donc	0,16
TC0400001362.mm.1	-1,02	Col9a2	JUC0400005404.mm.1	-2,03	0,02285	0,488353		
TC0400001362.mm.1	-1,02	Col9a2	PSR0400010351.mm.1	-2,09	0,021599	0,48335	Alternative 3' Acce	0,15
TC0700003608.mm.1	1,02	Cpeb1	PSR0700030710.mm.1	-2,03	0,046049	0,555632	Alternative 5' Donc	0,15
TC0800002382.mm.1	1,13	Ncan	PSR0800017977.mm.1	-2,03	0,008	0,403872	Alternative 5' Donc	0,15
TC0800002382.mm.1	1,13	Ncan	JUC0800009748.mm.1	-2,03	0,007809	0,403089		
TC0800002382.mm.1	1,13	Ncan	JUC0800009745.mm.1	-2,09	0,002769	0,350512		
TC0900000598.mm.1	1,03	Plet1	PSR0900004233.mm.1	-2,03	0,010576	0,422452	Alternative 5' Donc	0,15
TC0500002993.mm.1	1,05	Noc4l	PSR0500026481.mm.1	-2,03	0,00522	0,377078	Cassette Exon	0,14
TC0500002993.mm.1	1,05	Noc4l	PSR0500026480.mm.1	-2,06	0,040311	0,541838	Cassette Exon	0,14
TC0700000495.mm.1	1,23	Rps16; LOC100862433; Rp	PSR0700004005.mm.1	-2,03	0,034309	0,525528	Alternative 5' Donc	0,13
TC1100003218.mm.1	1,44	Ovca2; Dph1	PSR1100029589.mm.1	-2,03	0,004906	0,372627	Alternative 5' Donc	0,08
TC1100003218.mm.1	1,44	Ovca2; Dph1	PSR1100029609.mm.1	-2,31	0,000665	0,304044	Cassette Exon	0,13
TC1900000645.mm.1	1,71	Scd3	PSR1900005712.mm.1	-2,03	0,025782	0,498468	Cassette Exon	0,13
TC0700004068.mm.1	2,02	Ric3	PSR0700033582.mm.1	-2,03	0,03424	0,525168	Alternative 3' Acce	0,12
TC0700004068.mm.1	2,02	Ric3	JUC0700017619.mm.1	-3,34	0,022895	0,488524		
TC0X00000088.mm.1	1,28	Slc38a5	PSR0X00000662.mm.1	-2,03	0,01537	0,453849	Cassette Exon	0,12
TC1100000499.mm.1	1,06	Zfp62	PSR1100003812.mm.1	-2,03	0,015466	0,454312	Alternative 3' Acce	0,12
TC1100000499.mm.1	1,06	Zfp62	JUC1100002004.mm.1	-2,92	0,000576	0,304044		
TC1100000499.mm.1	1,06	Zfp62	JUC1100002001.mm.1	-4,92	0,034502	0,525929		
TC0100002392.mm.1	1,64	Trak2	PSR0100019498.mm.1	-2,03	0,016461	0,459205	Cassette Exon	0,1
TC0400004030.mm.1	1,3	Pex14	PSR0400033352.mm.1	-2,03	0,031731	0,517489	Cassette Exon	0,1
TC0400004030.mm.1	1,3	Pex14	JUC0400017399.mm.1	-2,31	0,019751	0,474932		
TC0800002728.mm.1	1,19	9330175E14Rik	PSR0800020896.mm.1	-2,03	0,041445	0,544646	Cassette Exon	0,1
TC0800002728.mm.1	1,19	9330175E14Rik	JUC0800011383.mm.1	-4,16	0,045029	0,553037		
TC1400000914.mm.1	1,01	Prss51	PSR1400007312.mm.1	-2,03	0,004716	0,370888	Cassette Exon	0,1
TC1700000278.mm.1	1,34	Zfp760	PSR1700002068.mm.1	-2,03	0,002176	0,344453	Cassette Exon	0,1
TC1700000278.mm.1	1,34	Zfp760	JUC1700001120.mm.1	-2,42	0,046934	0,557521		

TC1800000887.mm.1	1,34		PSR1800006434.mm.1	-2,03	0,035749	0,529051	Cassette Exon	0,1
TC0300001472.mm.1	-1,14	Gm16233	PSR0300011813.mm.1	-2,03	0,027114	0,503022	Cassette Exon	0,08
TC0700003035.mm.1	1,14	Sergef	JUC0700014805.mm.1	-2,03	0,038132	0,535709		
TC0700003035.mm.1	1,14	Sergef	PSR0700027671.mm.1	-2,08	0,022454	0,487003	Cassette Exon	0,08
TC1000000581.mm.1	-1,08	Hk1os; 2210417K05Rik	PSR1000003941.mm.1	-2,03	0,027135	0,503096	Cassette Exon	0,08
TC1900001313.mm.1	-1,16	D19Bwg1357e	PSR1900011696.mm.1	-2,03	0,00522	0,377078	Cassette Exon	0,08
TC0600000609.mm.1	1,7	Snx10	PSR0600004661.mm.1	-2,03	0,011415	0,427626	Alternative 5' Donc	0,05
TC0600001751.mm.1	1,26	Far2	PSR0600013967.mm.1	-2,03	0,028718	0,508133	Alternative 5' Donc	0,04
TC0600001751.mm.1	1,26	Far2	JUC0600007321.mm.1	-2,83	0,012433	0,43528		
TC1400000277.mm.1	1,49	Fam208a	JUC1400001359.mm.1	-2,03	0,041921	0,545814		
TC1400000277.mm.1	1,49	Fam208a	JUC1400001357.mm.1	-3,81	0,000706	0,306015		
TC1400000277.mm.1	1,49	Fam208a	JUC1400001341.mm.1	-7,25	0,015606	0,455078		
TC1400000299.mm.1	1,2	Actr8	JUC1400001427.mm.1	-2,03	0,035382	0,528536		
TC1300002748.mm.1	1,3	Zfp131	JUC1300009986.mm.1	-2,03	0,012914	0,438305		
TC1300002748.mm.1	1,3	Zfp131	JUC1300009992.mm.1	-3,67	0,025444	0,497549		
TC1400002096.mm.1	1,06	Tinf2	JUC1400008767.mm.1	-2,03	0,008865	0,410539		
TC1200000020.mm.1	1,4		JUC1200000153.mm.1	-2,03	0,020188	0,477113		
TC0100002395.mm.1	1,51	Tmem237	JUC0100011075.mm.1	-2,03	0,022034	0,485268		
TC0100002214.mm.1	1,22	Rev1	JUC0100010136.mm.1	-2,03	0,049674	0,563531		
TC0100002214.mm.1	1,22	Rev1	JUC0100010123.mm.1	-2,97	0,008078	0,404784		
TC0200001972.mm.1	1,23	Nop56; Snord57; Gm2445	PSR0200015611.mm.1	-2,03	0,048741	0,56139		
TC0500002075.mm.1	-1,54	Atg9b	JUC0500010284.mm.1	-2,03	0,035478	0,528724		
TC0800002745.mm.1	-1,02	Csnk2a2	JUC0800011510.mm.1	-2,03	0,018012	0,466939		
TC0900000281.mm.1	1,16	Nfrkb	JUC0900001182.mm.1	-2,03	0,016136	0,457846		
TC0700000798.mm.1	1,23	2410002F23Rik; Snord88a	JUC0700003234.mm.1	-2,03	0,006764	0,393343		
TC0600002462.mm.1	-1,42	Smyd1	JUC0600009887.mm.1	-2,03	0,047294	0,558249		
TC0600003388.mm.1	-1,12		JUC0600014017.mm.1	-2,03	0,037576	0,534053		
TC0900001766.mm.1	1,13	Cep57	JUC0900008302.mm.1	-2,03	0,035383	0,528536		
TC1100001115.mm.1	-1,01	Ssh2	JUC1100005324.mm.1	-2,03	0,028934	0,508626		
TC1000002017.mm.1	1,61	Fam229b	JUC1000007776.mm.1	-2,03	0,016894	0,460797		
TC1000002017.mm.1	1,61	Fam229b	JUC1000007774.mm.1	-2,89	0,012487	0,435429		
TC1000002017.mm.1	1,61	Fam229b	JUC1000007778.mm.1	-3,39	0,031369	0,516345		
TC0X00001243.mm.1	-1,04	Gm382	JUC0X00003796.mm.1	-2,03	0,029219	0,509532		
TC0400003200.mm.1	1,05	Pcsk9	JUC0400013206.mm.1	-2,03	0,025464	0,497569		
TC0900002276.mm.1	1,01	2310030G06Rik	JUC0900010699.mm.1	-2,03	0,015468	0,454312		
TC0900002175.mm.1	1,07	Arcn1	JUC0900010202.mm.1	-2,03	0,048955	0,5618		
TC0600003116.mm.1	12,17	Eno2	PSR0600024173.mm.1	-2,04	0,000697	0,305429		
TC0600003116.mm.1	12,17	Eno2	JUC0600012590.mm.1	-2,09	0,036986	0,532502		
TC0600003116.mm.1	12,17	Eno2	PSR0600024179.mm.1	-2,31	0,000665	0,304044	Cassette Exon	0,35
TC0600003116.mm.1	12,17	Eno2	JUC0600012587.mm.1	-2,99	0,000006	0,179072		
TC0600003116.mm.1	12,17	Eno2	PSR0600024188.mm.1	-3,02	0,008356	0,406435	Cassette Exon	0,21
TC0600003116.mm.1	12,17	Eno2	PSR0600024151.mm.1	-3,15	0,005011	0,373851	Alternative 3' Acce	0,34
TC0600003116.mm.1	12,17	Eno2	JUC0600012595.mm.1	-3,25	0,003424	0,355303		
TC0600003116.mm.1	12,17	Eno2	JUC0600012591.mm.1	-3,4	0,007979	0,403853		
TC0600003116.mm.1	12,17	Eno2	JUC0600012600.mm.1	-3,86	0,006989	0,395722		
TC0600003116.mm.1	12,17	Eno2	JUC0600012603.mm.1	-4,16	0,009332	0,414973		
TC0600003116.mm.1	12,17	Eno2	PSR0600024169.mm.1	-5,02	0,003279	0,354243	Alternative 3' Acce	0,54
TC0600003116.mm.1	12,17	Eno2	JUC0600012601.mm.1	-5,51	0,013644	0,444265		

TC0600003116.mm.1	12,17	Eno2	PSR0600024180.mm.1	-5,55	0,000654	0,304044	Alternative 3' Acce	0,48
TC0600003116.mm.1	12,17	Eno2	PSR0600024174.mm.1	-5,82	0,002621	0,349612	Alternative 3' Acce	0,34
TC0600003116.mm.1	12,17	Eno2	PSR0600024187.mm.1	-6,35	0,003649	0,356935	Alternative 3' Acce	0,16
TC0600003116.mm.1	12,17	Eno2	PSR0600024192.mm.1	-6,45	0,004984	0,373851	Alternative 5' Donc	0,16
TC0600003116.mm.1	12,17	Eno2	PSR0600024164.mm.1	-6,8	0,003938	0,359818	Alternative 5' Donc	0,42
TC0600003116.mm.1	12,17	Eno2	JUC0600012598.mm.1	-8,04	0,00215	0,343679		
TC0600003116.mm.1	12,17	Eno2	PSR0600024163.mm.1	-8,38	0,00486	0,371692	Alternative 5' Donc	0,42
TC0600003116.mm.1	12,17	Eno2	PSR0600024175.mm.1	-8,84	0,002176	0,344453	Alternative 3' Acce	0,34
TC0600003116.mm.1	12,17	Eno2	PSR0600024155.mm.1	-8,9	0,000991	0,316265	Intron Retention	0,68
TC0600003116.mm.1	12,17	Eno2	PSR0600024181.mm.1	-9,03	0,003275	0,354243	Alternative 3' Acce	0,48
TC0600003116.mm.1	12,17	Eno2	PSR0600024159.mm.1	-9,1	0,004252	0,363417	Intron Retention	0,75
TC0600003116.mm.1	12,17	Eno2	PSR0600024162.mm.1	-9,77	0,001141	0,317344	Alternative 5' Donc	0,42
TC0600003116.mm.1	12,17	Eno2	PSR0600024185.mm.1	-10,23	0,004752	0,371039	Alternative 5' Donc	0,46
TC0600003116.mm.1	12,17	Eno2	PSR0600024186.mm.1	-10,63	0,003362	0,354278	Alternative 5' Donc	0,46
TC0600003116.mm.1	12,17	Eno2	PSR0600024194.mm.1	-11,39	0,001398	0,325997	Alternative 5' Donc	0,16
TC0600003116.mm.1	12,17	Eno2	JUC0600012599.mm.1	-12,95	0,001015	0,316361		
TC1500000528.mm.1	4,45	Bai1; Gm26117	JUC1500002196.mm.1	-2,04	0,001361	0,32572		
TC1500000528.mm.1	4,45	Bai1; Gm26117	PSR1500003713.mm.1	-2,46	0,009076	0,412827	Cassette Exon	0,14
TC1500000528.mm.1	4,45	Bai1; Gm26117	PSR1500003695.mm.1	-3,1	0,019036	0,471877		
TC1500000528.mm.1	4,45	Bai1; Gm26117	PSR1500003684.mm.1	-3,41	0,005519	0,380501	Cassette Exon	0,49
TC1500000528.mm.1	4,45	Bai1; Gm26117	JUC1500002192.mm.1	-3,46	0,040064	0,541386		
TC1500000528.mm.1	4,45	Bai1; Gm26117	PSR1500003701.mm.1	-3,5	0,002901	0,352207	Cassette Exon	0,37
TC1500000528.mm.1	4,45	Bai1; Gm26117	PSR1500003692.mm.1	-3,52	0,000049	0,24627	Cassette Exon	0,38
TC1500000528.mm.1	4,45	Bai1; Gm26117	PSR1500003705.mm.1	-3,53	0,00058	0,304044	Cassette Exon	0,37
TC1500000528.mm.1	4,45	Bai1; Gm26117	PSR1500003718.mm.1	-3,72	0,012288	0,434238	Cassette Exon	0,39
TC1500000528.mm.1	4,45	Bai1; Gm26117	PSR1500003702.mm.1	-3,72	0,002824	0,352207	Cassette Exon	0,23
TC1500000528.mm.1	4,45	Bai1; Gm26117	JUC1500002177.mm.1	-3,91	0,002841	0,352207		
TC1500000528.mm.1	4,45	Bai1; Gm26117	PSR1500003690.mm.1	-3,93	0,004768	0,371039	Cassette Exon	0,32
TC1500000528.mm.1	4,45	Bai1; Gm26117	PSR1500003697.mm.1	-3,94	0,006466	0,390452		
TC1500000528.mm.1	4,45	Bai1; Gm26117	PSR1500003693.mm.1	-3,96	0,015118	0,452652	Cassette Exon	0,38
TC1500000528.mm.1	4,45	Bai1; Gm26117	PSR1500003710.mm.1	-4,03	0,003714	0,357586	Alternative 3' Acce	0,48
TC1500000528.mm.1	4,45	Bai1; Gm26117	PSR1500003685.mm.1	-4,06	0,008352	0,406435	Cassette Exon	0,55
TC1500000528.mm.1	4,45	Bai1; Gm26117	JUC1500002169.mm.1	-4,17	0,019649	0,474887		
TC1500000528.mm.1	4,45	Bai1; Gm26117	JUC1500002168.mm.1	-4,32	0,014089	0,446971		
TC1500000528.mm.1	4,45	Bai1; Gm26117	PSR1500003717.mm.1	-4,6	0,003311	0,354243	Cassette Exon	0,46
TC1500000528.mm.1	4,45	Bai1; Gm26117	PSR1500003678.mm.1	-4,87	0,00254	0,349501	Cassette Exon	0,28
TC1500000528.mm.1	4,45	Bai1; Gm26117	PSR1500003709.mm.1	-4,93	0,002638	0,349612	Cassette Exon	0,15
TC1500000528.mm.1	4,45	Bai1; Gm26117	JUC1500002200.mm.1	-4,94	0,006233	0,38825		
TC1500000528.mm.1	4,45	Bai1; Gm26117	PSR1500003676.mm.1	-4,99	0,001045	0,316361	Cassette Exon	0,55
TC1500000528.mm.1	4,45	Bai1; Gm26117	PSR1500003677.mm.1	-5,14	0,005026	0,374176	Cassette Exon	0,41
TC1500000528.mm.1	4,45	Bai1; Gm26117	PSR1500003696.mm.1	-5,15	0,014711	0,45037	Alternative 3' Acce	0,01
TC1500000528.mm.1	4,45	Bai1; Gm26117	PSR1500003689.mm.1	-5,26	0,007695	0,402552	Cassette Exon	0,41
TC1500000528.mm.1	4,45	Bai1; Gm26117	PSR1500003698.mm.1	-5,4	0,007719	0,402552	Alternative 5' Donc	0,01
TC1500000528.mm.1	4,45	Bai1; Gm26117	PSR1500003680.mm.1	-5,6	0,001141	0,317344	Cassette Exon	0,41
TC1500000528.mm.1	4,45	Bai1; Gm26117	PSR1500003691.mm.1	-5,71	0,005401	0,378967	Cassette Exon	0,41
TC1500000528.mm.1	4,45	Bai1; Gm26117	PSR1500003675.mm.1	-5,91	0,004082	0,362199	Cassette Exon	0,41
TC1500000528.mm.1	4,45	Bai1; Gm26117	PSR1500003712.mm.1	-5,94	0,030084	0,511908	Cassette Exon	0,28
TC1500000528.mm.1	4,45	Bai1; Gm26117	JUC1500002179.mm.1	-6,07	0,034664	0,52629		

TC1500000528.mm.1	4,45	Bai1; Gm26117	PSR1500003694.mm.1	-6,09	0,001364	0,32572	Cassette Exon	0,46
TC1500000528.mm.1	4,45	Bai1; Gm26117	JUC1500002189.mm.1	-6,09	0,001216	0,322251		
TC1500000528.mm.1	4,45	Bai1; Gm26117	PSR1500003688.mm.1	-6,26	0,006004	0,386556	Cassette Exon	0,55
TC1500000528.mm.1	4,45	Bai1; Gm26117	JUC1500002198.mm.1	-6,82	0,003126	0,353892		
TC1500000528.mm.1	4,45	Bai1; Gm26117	PSR1500003681.mm.1	-6,83	0,005321	0,378059	Cassette Exon	0,55
TC1500000528.mm.1	4,45	Bai1; Gm26117	JUC1500002172.mm.1	-7,16	0,002818	0,352207		
TC1500000528.mm.1	4,45	Bai1; Gm26117	PSR1500003703.mm.1	-7,31	0,003593	0,356223	Cassette Exon	0,31
TC1500000528.mm.1	4,45	Bai1; Gm26117	JUC1500002176.mm.1	-7,58	0,001888	0,338712		
TC1500000528.mm.1	4,45	Bai1; Gm26117	JUC1500002199.mm.1	-7,81	0,002283	0,34605		
TC1500000528.mm.1	4,45	Bai1; Gm26117	PSR1500003687.mm.1	-8,51	0,000928	0,313363	Cassette Exon	0,54
TC1500000528.mm.1	4,45	Bai1; Gm26117	JUC1500002175.mm.1	-8,96	0,020721	0,479166		
TC1500000528.mm.1	4,45	Bai1; Gm26117	JUC1500002178.mm.1	-9	0,015782	0,455613		
TC1500000528.mm.1	4,45	Bai1; Gm26117	PSR1500003686.mm.1	-9,35	0,001092	0,317225	Cassette Exon	0,54
TC1500000528.mm.1	4,45	Bai1; Gm26117	JUC1500002185.mm.1	-9,69	0,003324	0,354243		
TC0X00000126.mm.1	2	Tspan7	PSR0X00000859.mm.1	-2,04	0,027064	0,502979	Alternative 3' Acce	0,06
TC0X00000126.mm.1	2	Tspan7	PSR0X00000870.mm.1	-2,57	0,019953	0,476085	Intron Retention	0,38
TC0500001620.mm.1	2,91	Ache	JUC0500007994.mm.1	-2,04	0,04137	0,544518		
TC0500001620.mm.1	2,91	Ache	PSR0500014663.mm.1	-2,08	0,03608	0,529704	Alternative 3' Acce	0,07
TC0500001620.mm.1	2,91	Ache	JUC0500007993.mm.1	-2,7	0,000674	0,304338		
TC0500001620.mm.1	2,91	Ache	JUC0500007997.mm.1	-3,2	0,043161	0,548752		
TC0500001620.mm.1	2,91	Ache	PSR0500014657.mm.1	-3,21	0,017295	0,46317	Cassette Exon	0,32
TC0500001620.mm.1	2,91	Ache	PSR0500014675.mm.1	-3,23	0,005463	0,379939	Alternative 5' Donc	0,23
TC0500001620.mm.1	2,91	Ache	JUC0500007989.mm.1	-3,64	0,019395	0,47375		
TC0500001620.mm.1	2,91	Ache	JUC0500007996.mm.1	-4,37	0,000341	0,289576		
TC0300000732.mm.1	1,32	Arhgef2	PSR0300005194.mm.1	-2,04	0,047656	0,558801	Alternative 3' Acce	0,06
TC0300000732.mm.1	1,32	Arhgef2	PSR0300005191.mm.1	-2,12	0,010028	0,41878	Cassette Exon	0,11
TC0300000732.mm.1	1,32	Arhgef2	PSR0300005187.mm.1	-2,15	0,016751	0,460378	Cassette Exon	0,15
TC0300000732.mm.1	1,32	Arhgef2	PSR0300005206.mm.1	-2,22	0,025988	0,499207	Alternative 3' Acce	0,18
TC0300000732.mm.1	1,32	Arhgef2	PSR0300005267.mm.1	-2,23	0,007797	0,403089	Intron Retention	0,31
TC0300000732.mm.1	1,32	Arhgef2	JUC0300002772.mm.1	-2,4	0,023236	0,490032		
TC0300000732.mm.1	1,32	Arhgef2	PSR0300005202.mm.1	-2,65	0,015171	0,452699	Cassette Exon	0,13
TC0300000732.mm.1	1,32	Arhgef2	PSR0300005189.mm.1	-2,81	0,019041	0,471937	Alternative 3' Acce	0,18
TC0200002386.mm.1	1,93	Phf20	JUC0200009711.mm.1	-2,04	0,019872	0,475704		
TC0200002386.mm.1	1,93	Phf20	PSR0200019169.mm.1	-2,13	0,000067	0,250119	Cassette Exon	0,04
TC0200002386.mm.1	1,93	Phf20	PSR0200019175.mm.1	-2,31	0,01065	0,422925	Intron Retention	0,29
TC0200002386.mm.1	1,93	Phf20	PSR0200019178.mm.1	-2,48	0,013973	0,446239	Cassette Exon	0,12
TC0200002386.mm.1	1,93	Phf20	PSR0200019196.mm.1	-2,5	0,040388	0,541998	Cassette Exon	0,11
TC0200002386.mm.1	1,93	Phf20	PSR0200019181.mm.1	-2,53	0,003598	0,356223	Alternative 3' Acce	0,19
TC0200002386.mm.1	1,93	Phf20	PSR0200019163.mm.1	-2,61	0,000921	0,313363	Cassette Exon	0,19
TC0200002386.mm.1	1,93	Phf20	JUC0200009724.mm.1	-3	0,001886	0,338712		
TC0200002386.mm.1	1,93	Phf20	JUC0200009727.mm.1	-3,3	0,00326	0,354243		
TC1100001938.mm.1	2,1	Gm11744	PSR1100018415.mm.1	-2,04	0,005338	0,378059	Cassette Exon	0,12
TC1100001938.mm.1	2,1	Gm11744	PSR1100018418.mm.1	-2,2	0,036031	0,529581	Alternative 3' Acce	0,19
TC1100001938.mm.1	2,1	Gm11744	JUC1100009600.mm.1	-2,54	0,008116	0,405267		
TC1100001938.mm.1	2,1	Gm11744	PSR1100018416.mm.1	-2,62	0,002861	0,352207	Cassette Exon	0,22
TC1100001938.mm.1	2,1	Gm11744	PSR1100018425.mm.1	-2,68	0,018214	0,4676	Alternative 5' Donc	0,27
TC1000001192.mm.1	-1,09	Epyc	PSR1000008972.mm.1	-2,04	0,017001	0,461553	Cassette Exon	0,23
TC1000001192.mm.1	-1,09	Epyc	JUC1000004832.mm.1	-5,1	0,041111	0,543948		

TC0100003531.mm.1	2,92	Pcp4l1	PSR0100028875.mm.1	-2,04	0,02049	0,478019	Cassette Exon	0,1
TC0100003531.mm.1	2,92	Pcp4l1	JUC0100016449.mm.1	-2,25	0,044074	0,550884		
TC0100003531.mm.1	2,92	Pcp4l1	PSR0100028876.mm.1	-2,75	0,026943	0,502714	Cassette Exon	0,22
TC0400001957.mm.1	1,83	Clstn1	JUC0400008429.mm.1	-2,04	0,013828	0,445221		
TC0400001957.mm.1	1,83	Clstn1	PSR0400016041.mm.1	-2,76	0,030946	0,514624	Alternative 3' Acce	0,19
TC0500000434.mm.1	1,93	Lyar	PSR0500004317.mm.1	-2,04	0,035996	0,529506	Alternative 5' Donc	0,15
TC0500000434.mm.1	1,93	Lyar	PSR0500004309.mm.1	-2,27	0,014566	0,449757	Alternative 5' Donc	0,19
TC0500000434.mm.1	1,93	Lyar	JUC0500002410.mm.1	-3,4	0,020179	0,477086		
TC1800001740.mm.1	1,74	Pcdhac1; Pcdhac2; Pcdha4	PSR1800002716.mm.1	-2,04	0,011368	0,427487		
TC1800001740.mm.1	1,74	Pcdhac1; Pcdhac2; Pcdha4	PSR1800002729.mm.1	-2,16	0,008356	0,406435	Cassette Exon	0,08
TC1800001740.mm.1	1,74	Pcdhac1; Pcdhac2; Pcdha4	PSR1800002709.mm.1	-2,41	0,037915	0,535116	Alternative 3' Acce	0,19
TC1800001740.mm.1	1,74	Pcdhac1; Pcdhac2; Pcdha4	JUC1800001583.mm.1	-2,59	0,008851	0,410388		
TC1800001740.mm.1	1,74	Pcdhac1; Pcdhac2; Pcdha4	JUC1800001584.mm.1	-2,73	0,004867	0,371692		
TC1800001740.mm.1	1,74	Pcdhac1; Pcdhac2; Pcdha4	JUC1800001577.mm.1	-2,84	0,020302	0,477245		
TC1800001740.mm.1	1,74	Pcdhac1; Pcdhac2; Pcdha4	JUC1800001582.mm.1	-3,47	0,031803	0,517645		
TC1800001740.mm.1	1,74	Pcdhac1; Pcdhac2; Pcdha4	JUC1800001571.mm.1	-3,88	0,032317	0,519155		
TC1800001740.mm.1	1,74	Pcdhac1; Pcdhac2; Pcdha4	JUC1800001570.mm.1	-3,95	0,025544	0,497801		
TC1800001740.mm.1	1,74	Pcdhac1; Pcdhac2; Pcdha4	JUC1800001579.mm.1	-5,07	0,030061	0,511847		
TC0200002415.mm.1	1,39	Ctnnbl1	JUC0200009921.mm.1	-2,04	0,024502	0,493992		
TC0200002415.mm.1	1,39	Ctnnbl1	PSR0200019593.mm.1	-2,29	0,026116	0,499897	Alternative 5' Donc	0,18
TC0200002986.mm.1	-1,27	Cubn	JUC0200012500.mm.1	-2,04	0,030995	0,5148		
TC0200002986.mm.1	-1,27	Cubn	JUC0200012495.mm.1	-2,81	0,02279	0,488201		
TC0200002986.mm.1	-1,27	Cubn	PSR0200024687.mm.1	-2,98	0,0035	0,355638	Cassette Exon	0,17
TC0500003369.mm.1	1,03	Gtf2i	PSR0500030348.mm.1	-2,04	0,010776	0,423955	Alternative 5' Donc	0,17
TC0X00000072.mm.1	1,37	Kcnd1	PSR0X00000513.mm.1	-2,04	0,016345	0,458641	Alternative 3' Acce	0,17
TC0X00000072.mm.1	1,37	Kcnd1	PSR0X00000509.mm.1	-2,55	0,002012	0,341091	Cassette Exon	0,15
TC0100003397.mm.1	1,07	Prrc2c	JUC0100015922.mm.1	-2,04	0,01362	0,444002		
TC0100003397.mm.1	1,07	Prrc2c	PSR0100027822.mm.1	-2,06	0,029015	0,508842	Alternative 5' Donc	0,11
TC0100003397.mm.1	1,07	Prrc2c	PSR0100027810.mm.1	-2,12	0,020674	0,478862	Alternative 3' Acce	0,13
TC0100003397.mm.1	1,07	Prrc2c	PSR0100027831.mm.1	-2,31	0,000015	0,18968	Alternative 5' Donc	0,16
TC0100003397.mm.1	1,07	Prrc2c	JUC0100015909.mm.1	-2,43	0,010882	0,424197		
TC0300002068.mm.1	1,29	Gpr149	PSR0300015604.mm.1	-2,04	0,000792	0,310881	Alternative 5' Donc	0,16
TC0400002475.mm.1	1,75	Cntfr	PSR0400019998.mm.1	-2,04	0,01554	0,454617	Cassette Exon	0,16
TC0400002475.mm.1	1,75	Cntfr	JUC0400010408.mm.1	-2,1	0,012347	0,43457		
TC0400002475.mm.1	1,75	Cntfr	JUC0400010411.mm.1	-2,35	0,005578	0,381035		
TC0400002475.mm.1	1,75	Cntfr	JUC0400010412.mm.1	-2,48	0,007767	0,402774		
TC0900002519.mm.1	1,42	Trip4	JUC0900011898.mm.1	-2,04	0,028029	0,505851		
TC0900002519.mm.1	1,42	Trip4	JUC0900011880.mm.1	-2,22	0,041474	0,544734		
TC0900002519.mm.1	1,42	Trip4	JUC0900011882.mm.1	-2,29	0,039548	0,539764		
TC0900002519.mm.1	1,42	Trip4	PSR0900021097.mm.1	-2,67	0,029083	0,509063	Cassette Exon	0,16
TC0900002519.mm.1	1,42	Trip4	JUC0900011886.mm.1	-2,78	0,03984	0,540922		
TC0Y00000059.mm.1	1,3	Gm21242; Gm21065; Ssty	PSR0Y00000321.mm.1	-2,04	0,040849	0,543284	Alternative 5' Donc	0,16
TC0Y00000434.mm.1	1,3	Ssty2; Gm21242; Gm2106	PSR0Y00002383.mm.1	-2,04	0,040849	0,543284	Alternative 3' Acce	0,16
TC1200001314.mm.1	1,81	Rapgef5	PSR1200009493.mm.1	-2,04	0,001973	0,340754	Cassette Exon	0,11
TC1200001314.mm.1	1,81	Rapgef5	PSR1200009497.mm.1	-2,16	0,040102	0,541437	Cassette Exon	0,12
TC1200001314.mm.1	1,81	Rapgef5	PSR1200009495.mm.1	-2,76	0,042215	0,546983	Cassette Exon	0,16
TC0400000496.mm.1	-1,18	1300002K09Rik	PSR0400003535.mm.1	-2,04	0,016052	0,457283	Alternative 5' Donc	0,15
TC1800001026.mm.1	2,77	Cdh2	PSR1800007622.mm.1	-2,04	0,013669	0,444535	Alternative 3' Acce	0,13

TC1800001026.mm.1	2,77	Cdh2	PSR1800007624.mm.1	-2,29	0,036021	0,529546	Cassette Exon	0,15
TC1800001026.mm.1	2,77	Cdh2	PSR1800007626.mm.1	-2,72	0,017124	0,46232	Cassette Exon	0,15
TC0400001250.mm.1	1,49	Pik3r3	PSR0400009267.mm.1	-2,04	0,009148	0,413447	Alternative 5' Donc	0,14
TC0200003320.mm.1	1,39	Ralgps1	PSR0200028454.mm.1	-2,04	0,031929	0,518048	Alternative 5' Donc	0,13
TC0200003320.mm.1	1,39	Ralgps1	JUC0200014354.mm.1	-2,69	0,03405	0,524583		
TC0800001201.mm.1	1,06	Ces3b	PSR0800009406.mm.1	-2,04	0,028229	0,506368	Cassette Exon	0,13
TC0300000656.mm.1	-1,38	Tmem154	PSR0300004337.mm.1	-2,04	0,015928	0,456754	Cassette Exon	0,12
TC0800001547.mm.1	1,78	Arv1	PSR0800012701.mm.1	-2,04	0,002444	0,349135	Cassette Exon	0,12
TC0800001547.mm.1	1,78	Arv1	PSR0800012700.mm.1	-2,04	0,001938	0,340349	Cassette Exon	0,11
TC0100001546.mm.1	1,05		PSR0100012710.mm.1	-2,04	0,032205	0,51885	Alternative 3' Acce	0,11
TC0500002518.mm.1	1,14	Uchl1os	PSR0500022456.mm.1	-2,04	0,013978	0,446242	Cassette Exon	0,11
TC0800002107.mm.1	1,52	Cnot7	PSR0800016185.mm.1	-2,04	0,020659	0,478862	Alternative 3' Acce	0,11
TC0X00001286.mm.1	1,28	Armcx1	PSR0X00008041.mm.1	-2,04	0,031732	0,517489	Intron Retention	0,11
TC0300000233.mm.1	1,67	Ttc14	PSR0300001432.mm.1	-2,04	0,006669	0,393187	Alternative 3' Acce	0,1
TC0300000233.mm.1	1,67	Ttc14	JUC0300000724.mm.1	-3,03	0,010666	0,423012		
TC0700001698.mm.1	-1,14	Tmc5	PSR0700014138.mm.1	-2,04	0,040291	0,541823	Cassette Exon	0,1
TC0700002873.mm.1	1,14	Uri1	PSR0700025692.mm.1	-2,04	0,007061	0,396462	Cassette Exon	0,1
TC0700003592.mm.1	1,77	Man2a2	PSR0700030431.mm.1	-2,04	0,025883	0,498873	Cassette Exon	0,1
TC0700003592.mm.1	1,77	Man2a2	JUC0700016038.mm.1	-2,32	0,006172	0,387873		
TC0200004980.mm.1	-1,02	Fer1l4	PSR0200042397.mm.1	-2,04	0,017185	0,462373	Cassette Exon	0,09
TC0200004980.mm.1	-1,02	Fer1l4	JUC0200021908.mm.1	-3,02	0,04245	0,547627		
TC0400000911.mm.1	-1,34	LOC102637866	PSR0400006119.mm.1	-2,04	0,018879	0,471134	Cassette Exon	0,09
TC0400003206.mm.1	1,61	Ttc4	PSR0400025419.mm.1	-2,04	0,010992	0,425398	Cassette Exon	0,08
TC0400003206.mm.1	1,61	Ttc4	JUC0400013242.mm.1	-2,35	0,010483	0,421891		
TC1700002804.mm.1	-1,12	Pigq	PSR1700015554.mm.1	-2,04	0,017316	0,463282	Alternative 3' Acce	0,04
TC0200003318.mm.1	-1,12	Garnl3	JUC0200014338.mm.1	-2,04	0,015057	0,452223		
TC0200002728.mm.1	1,72	Gm2020	JUC0200011298.mm.1	-2,04	0,001425	0,327447		
TC1100003992.mm.1	1,16	Wipi1	JUC1100019521.mm.1	-2,04	0,0361	0,529729		
TC1300000280.mm.1	1,43	Tdp2	JUC1300000838.mm.1	-2,04	0,001859	0,337661		
TC1300000904.mm.1	-1,16	Erap1	JUC1300003102.mm.1	-2,04	0,013295	0,441721		
TC0100003879.mm.1	1,41	Als2	JUC0100011127.mm.1	-2,04	0,01248	0,435429		
TC1800000282.mm.1	1,26	Apc	JUC1800001204.mm.1	-2,04	0,042057	0,546262		
TC0100003141.mm.1	-1,07	Mroh3	JUC0100014522.mm.1	-2,04	0,007258	0,39826		
TC1700002822.mm.1	-1,18	H2-T22; H2-T9	JUC1700010205.mm.1	-2,04	0,016878	0,460797		
TC0100001256.mm.1	-1,15	Lad1	JUC0100005946.mm.1	-2,04	0,011199	0,426568		
TC1500000398.mm.1	1,13	Mtbp	JUC1500001619.mm.1	-2,04	0,023146	0,489785		
TC1700000618.mm.1	1,01	H2-DMa	JUC1700003216.mm.1	-2,04	0,038566	0,536789		
TC0200000634.mm.1	-1,37	Cntrl; Cep110	JUC0200002925.mm.1	-2,04	0,024726	0,494838		
TC0800002023.mm.1	1,2	Leprotl1	JUC0800008656.mm.1	-2,04	0,026199	0,50003		
TC0800001740.mm.1	-1,02	Rasa3	JUC0800007685.mm.1	-2,04	0,015058	0,452223		
TC0700004504.mm.1	-1,03	Syce1	JUC0700019940.mm.1	-2,04	0,014165	0,447466		
TC0800000648.mm.1	1,24	2700029M09Rik	JUC0800002304.mm.1	-2,04	0,006593	0,39213		
TC0800000923.mm.1	1,15	Clgn	JUC0800003625.mm.1	-2,04	0,008808	0,409934		
TC0500001132.mm.1	1,19	Chfr	JUC0500005254.mm.1	-2,04	0,04378	0,550398		
TC0500001230.mm.1	1,2	Tchp	JUC0500005791.mm.1	-2,04	0,038719	0,537318		
TC0600000598.mm.1	1,03	5430402O13Rik	JUC0600002334.mm.1	-2,04	0,033752	0,523605		
TC1100000216.mm.1	1,11	Wdpcp	JUC1100000927.mm.1	-2,04	0,027677	0,504734		
TC0400000479.mm.1	1,23	Gm12678	JUC0400001625.mm.1	-2,04	0,00592	0,384341		

TC0400001062.mm.1	1,08	Tctex1d1	JUC0400003718.mm.1	-2,04	0,030081	0,511908		
TC0400001387.mm.1	1,14	Trit1	JUC0400005448.mm.1	-2,04	0,026749	0,502096		
TC0X00000933.mm.1	1,04	Stard8	JUC0X00002967.mm.1	-2,04	0,030351	0,512923		
TC0900002050.mm.1	-1,08	Robo3	JUC0900009654.mm.1	-2,04	0,040665	0,542693		
TC1000000746.mm.1	1,04	Ftcd	JUC1000002954.mm.1	-2,04	0,035196	0,528241		
TC1000000852.mm.1	5,21	Reep6	PSR1000006499.mm.1	-2,05	0,004144	0,3623	Cassette Exon	0,19
TC1000000852.mm.1	5,21	Reep6	JUC1000003515.mm.1	-3,24	0,004091	0,36228		
TC1000000852.mm.1	5,21	Reep6	PSR1000006493.mm.1	-5,98	0,009445	0,416241	Alternative 3' Acce	0,48
TC1000000852.mm.1	5,21	Reep6	PSR1000006495.mm.1	-6,04	0,017223	0,462633	Alternative 3' Acce	0,48
TC1000000852.mm.1	5,21	Reep6	JUC1000003516.mm.1	-7,97	0,003849	0,358836		
TC0900001379.mm.1	7,17		PSR0900010867.mm.1	-2,05	0,044675	0,55216	Alternative 3' Acce	0,21
TC0900001379.mm.1	7,17		PSR0900010879.mm.1	-2,14	0,020674	0,478862	Cassette Exon	0,17
TC0900001379.mm.1	7,17		PSR0900010872.mm.1	-2,17	0,048338	0,560493	Intron Retention	0,27
TC0900001379.mm.1	7,17		PSR0900010882.mm.1	-4,26	0,009778	0,417839	Cassette Exon	0,41
TC0900001379.mm.1	7,17		JUC0900005979.mm.1	-5,16	0,046785	0,55703		
TC0900001379.mm.1	7,17		JUC0900005978.mm.1	-10,15	0,003447	0,3554		
TC0900001379.mm.1	7,17		JUC0900005981.mm.1	-11,38	0,004534	0,368648		
TC0900001379.mm.1	7,17		JUC0900005975.mm.1	-12,14	0,00248	0,349135		
TC0900001379.mm.1	7,17		JUC0900005982.mm.1	-39,28	0,001172	0,319173		
TC0900001379.mm.1	7,17		JUC0900005977.mm.1	-51,61	0,000351	0,290604		
TC0600002562.mm.1	2,15	Wdr54	PSR0600019872.mm.1	-2,05	0,016597	0,459691	Intron Retention	0,26
TC0600002562.mm.1	2,15	Wdr54	PSR0600019886.mm.1	-2,73	0,001955	0,340561	Intron Retention	0,39
TC0700002866.mm.1	2,03	Zfp536	JUC0700013698.mm.1	-2,05	0,020874	0,479521		
TC0700002866.mm.1	2,03	Zfp536	PSR0700025662.mm.1	-2,18	0,001254	0,322251	Cassette Exon	0,1
TC0700002866.mm.1	2,03	Zfp536	JUC0700013703.mm.1	-2,18	0,007005	0,395737		
TC0700002866.mm.1	2,03	Zfp536	PSR0700025665.mm.1	-2,27	0,00068	0,304417	Alternative 5' Donc	0,09
TC0700002866.mm.1	2,03	Zfp536	PSR0700025667.mm.1	-2,37	0,014785	0,45081	Cassette Exon	0,22
TC0700002866.mm.1	2,03	Zfp536	JUC0700013711.mm.1	-2,45	0,004144	0,3623		
TC0700002866.mm.1	2,03	Zfp536	PSR0700025645.mm.1	-2,52	0,023103	0,48975	Alternative 5' Donc	0,14
TC0700002866.mm.1	2,03	Zfp536	JUC0700013709.mm.1	-2,53	0,000852	0,311909		
TC0700002866.mm.1	2,03	Zfp536	JUC0700013708.mm.1	-2,93	0,002727	0,349612		
TC0700002866.mm.1	2,03	Zfp536	JUC0700013701.mm.1	-3,43	0,03565	0,528956		
TC0900001367.mm.1	1,55	Cacna2d2	JUC0900005891.mm.1	-2,05	0,020027	0,476453		
TC0900001367.mm.1	1,55	Cacna2d2	PSR0900010742.mm.1	-2,15	0,020768	0,479303		
TC0900001367.mm.1	1,55	Cacna2d2	JUC0900005862.mm.1	-2,21	0,017216	0,462603		
TC0900001367.mm.1	1,55	Cacna2d2	JUC0900005903.mm.1	-2,21	0,008635	0,408463		
TC0900001367.mm.1	1,55	Cacna2d2	JUC0900005900.mm.1	-2,52	0,004058	0,36184		
TC0900001367.mm.1	1,55	Cacna2d2	JUC0900005901.mm.1	-2,65	0,044672	0,55216		
TC0900001367.mm.1	1,55	Cacna2d2	PSR0900010681.mm.1	-2,79	0,004235	0,363295	Cassette Exon	0,22
TC0900001367.mm.1	1,55	Cacna2d2	JUC0900005906.mm.1	-3,17	0,032057	0,518469		
TC0900001367.mm.1	1,55	Cacna2d2	JUC0900005904.mm.1	-5,64	0,003954	0,359922		
TC0600000648.mm.1	1,73	Chn2	PSR0600004956.mm.1	-2,05	0,011441	0,427685	Alternative 3' Acce	0,14
TC0600000648.mm.1	1,73	Chn2	PSR0600004954.mm.1	-2,49	0,014635	0,450098	Cassette Exon	0,16
TC0600000648.mm.1	1,73	Chn2	PSR0600004955.mm.1	-2,63	0,034523	0,52593	Cassette Exon	0,15
TC0600000648.mm.1	1,73	Chn2	PSR0600004949.mm.1	-3,49	0,017676	0,464879	Cassette Exon	0,19
TC0500002045.mm.1	1,21	5031425E22Rik	JUC0500010148.mm.1	-2,05	0,019652	0,474887		
TC0500002045.mm.1	1,21	5031425E22Rik	PSR0500018613.mm.1	-2,32	0,002067	0,341993	Cassette Exon	0,12
TC0500002045.mm.1	1,21	5031425E22Rik	PSR0500018596.mm.1	-2,91	0,039166	0,538679	Cassette Exon	0,18

TC1000001895.mm.1	-1,53	Enpp3	JUC1000007434.mm.1	-2,05	0,029022	0,508842		
TC1000001895.mm.1	-1,53	Enpp3	PSR1000013653.mm.1	-2,1	0,031219	0,515865	Alternative 3' Acce	0,18
TC1000001895.mm.1	-1,53	Enpp3	JUC1000007424.mm.1	-3,92	0,012593	0,435933		
TC0300000987.mm.1	1,08	Wars2	PSR0300007992.mm.1	-2,05	0,042696	0,548097	Alternative 5' Donc	0,17
TC0300000987.mm.1	1,08	Wars2	PSR0300008000.mm.1	-2,42	0,0056	0,381115	Cassette Exon	0,13
TC0400002098.mm.1	1,2	Acap3	PSR0400017626.mm.1	-2,05	0,033886	0,524166	Alternative 3' Acce	0,17
TC0400002236.mm.1	1,56	Otud6b	PSR0400018672.mm.1	-2,05	0,028412	0,507154	Alternative 3' Acce	0,17
TC0400004211.mm.1	1,11	Mup2; Mup10; Mup19; M	PSR0400022528.mm.1	-2,05	0,010643	0,422925	Cassette Exon	0,17
TC0700000035.mm.1	1,22	Gm15873	JUC0700000227.mm.1	-2,05	0,039634	0,540125		
TC0700000035.mm.1	1,22	Gm15873	PSR0700000469.mm.1	-2,13	0,000487	0,298999	Alternative 3' Acce	0,17
TC1300002238.mm.1	-1,1	4933433G19Rik	PSR1300014503.mm.1	-2,05	0,008169	0,405603	Alternative 3' Acce	0,17
TC0200004130.mm.1	1,06	Dgkz	PSR0200034607.mm.1	-2,05	0,025158	0,496555	Alternative 5' Donc	0,15
TC0200004130.mm.1	1,06	Dgkz	PSR0200034614.mm.1	-2,49	0,007584	0,401712	Cassette Exon	0,14
TC0700003981.mm.1	1,91	Apbb1	JUC0700017455.mm.1	-2,05	0,029679	0,510918		
TC0700003981.mm.1	1,91	Apbb1	PSR0700033135.mm.1	-2,17	0,010986	0,425397	Cassette Exon	0,15
TC0700003981.mm.1	1,91	Apbb1	JUC0700017436.mm.1	-2,7	0,049504	0,563325		
TC0700003981.mm.1	1,91	Apbb1	JUC0700017445.mm.1	-2,7	0,00726	0,39826		
TC0700003981.mm.1	1,91	Apbb1	JUC0700017450.mm.1	-2,77	0,021559	0,483034		
TC0800001133.mm.1	-1,12	Mmp15	PSR0800008939.mm.1	-2,05	0,00205	0,341391	Cassette Exon	0,15
TC1100002124.mm.1	1,1	Gas2l1	PSR1100020651.mm.1	-2,05	0,004492	0,368077	Alternative 3' Acce	0,15
TC1200001508.mm.1	2,57		PSR1200010551.mm.1	-2,05	0,041213	0,544134	Cassette Exon	0,15
TC1200001508.mm.1	2,57		JUC1200005819.mm.1	-2,21	0,012218	0,433321		
TC0900000523.mm.1	1,18	Ift46	PSR0900003609.mm.1	-2,05	0,012185	0,433072	Alternative 5' Donc	0,14
TC1100003780.mm.1	-1,05	Kat2a	PSR1100034819.mm.1	-2,05	0,045455	0,554203	Cassette Exon	0,14
TC1400002252.mm.1	2,03	Fzd3	PSR1400017379.mm.1	-2,05	0,016511	0,459491	Cassette Exon	0,13
TC1400002252.mm.1	2,03	Fzd3	JUC1400009501.mm.1	-3,19	0,001445	0,328204		
TC0600002486.mm.1	1,25	Rnf181	PSR0600019248.mm.1	-2,05	0,015933	0,456765	Intron Retention	0,12
TC0700002347.mm.1	1,49	Zscan18	PSR0700020842.mm.1	-2,05	0,009826	0,417839	Cassette Exon	0,12
TC0700000965.mm.1	1,37	Otud7a	PSR0700008786.mm.1	-2,05	0,018891	0,471219	Cassette Exon	0,1
TC0700000965.mm.1	1,37	Otud7a	JUC0700004479.mm.1	-3,08	0,015318	0,453634		
TC0700000965.mm.1	1,37	Otud7a	JUC0700004481.mm.1	-3,7	0,016106	0,457703		
TC0700002260.mm.1	-1,05	Vmn2r-ps46	PSR0700020432.mm.1	-2,05	0,022352	0,486348	Cassette Exon	0,1
TC1100004040.mm.1	1,06	Sdk2	PSR1100037890.mm.1	-2,05	0,035711	0,529027	Cassette Exon	0,1
TC1100004040.mm.1	1,06	Sdk2	JUC1100019900.mm.1	-2,57	0,017137	0,46232		
TC1300000586.mm.1	1,17	Fbxw17	PSR1300003681.mm.1	-2,05	0,028789	0,508192	Cassette Exon	0,1
TC0100002863.mm.1	2,04	D1Ertd622e	JUC0100013161.mm.1	-2,05	0,027929	0,505612		
TC0100002863.mm.1	2,04	D1Ertd622e	PSR0100023172.mm.1	-2,17	0,005645	0,381706	Cassette Exon	0,09
TC0100002863.mm.1	2,04	D1Ertd622e	JUC0100013162.mm.1	-4,06	0,029984	0,51154		
TC0100001592.mm.1	-1,44	F11r	PSR0100013160.mm.1	-2,05	0,003719	0,357586	Alternative 5' Donc	0,08
TC1400001671.mm.1	-1,04	Stab1	JUC1400006931.mm.1	-2,05	0,012891	0,438165		
TC1400001671.mm.1	-1,04	Stab1	JUC1400006936.mm.1	-2,53	0,003432	0,355303		
TC0300000721.mm.1	1,88	Cct3	JUC0300002664.mm.1	-2,05	0,019394	0,47375		
TC0300000017.mm.1	1,06	Zfhx4	JUC0300000032.mm.1	-2,05	0,006374	0,389488		
TC1100004105.mm.1	1,15	Fbf1	JUC1100020297.mm.1	-2,05	0,020521	0,478264		
TC1200000486.mm.1	-1,22	Prps1l3; Mipol1	JUC1200001874.mm.1	-2,05	0,034679	0,526294		
TC1300002061.mm.1	1	Pdlim7; Mir6945; mmu-mi	JUC1300006709.mm.1	-2,05	0,038443	0,536405		
TC1300002061.mm.1	1	Pdlim7; Mir6945; mmu-mi	JUC1300006710.mm.1	-2,38	0,03766	0,534288		
TC1700001579.mm.1	-1,54	Vmn2r-ps128	JUC1700007517.mm.1	-2,05	0,018237	0,467691		

TC1700002175.mm.1	-1,24	Slc22a7	JUC1700010865.mm.1	-2,05	0,022971	0,488823		
TC1500001504.mm.1	1,25	Eif3h	JUC1500006418.mm.1	-2,05	0,016117	0,457756		
TC1500001504.mm.1	1,25	Eif3h	JUC1500006417.mm.1	-2,26	0,025373	0,497403		
TC1500002096.mm.1	1,39	Yaf2	JUC1500009580.mm.1	-2,05	0,028249	0,506565		
TC0100003282.mm.1	-1,28	Rgsl1	JUC0100015263.mm.1	-2,05	0,031528	0,517029		
TC1700000429.mm.1	-1,19	Zbtb9; Ggnbp1	JUC1700002007.mm.1	-2,05	0,023917	0,492323		
TC1600001155.mm.1	1,05	Nagpa	JUC1600004830.mm.1	-2,05	0,02563	0,49793		
TC0800001194.mm.1	-1,21	Ces2c	JUC0800004970.mm.1	-2,05	0,008901	0,410728		
TC0500001575.mm.1	-1,21	Mlxipl	JUC0500007707.mm.1	-2,05	0,015529	0,454591		
TC0900000561.mm.1	1,01	Apoa5	JUC0900002042.mm.1	-2,05	0,021193	0,481078		
TC0700000534.mm.1	1,06	Zfp30	JUC0700002312.mm.1	-2,05	0,03268	0,520439		
TC0700000534.mm.1	1,06	Zfp30	JUC0700002307.mm.1	-3,36	0,009439	0,416119		
TC0700000154.mm.1	1,3	Zfp446	JUC0700000563.mm.1	-2,05	0,010017	0,418611		
TC0700000154.mm.1	1,3	Zfp446	JUC0700000562.mm.1	-2,69	0,018586	0,469618		
TC0600002631.mm.1	-1,09		JUC0600010575.mm.1	-2,05	0,027982	0,505748		
TC0600001736.mm.1	1,15	Stk38l	JUC0600007210.mm.1	-2,05	0,00899	0,41198		
TC1100001077.mm.1	-1,11	Myo1c	JUC1100005210.mm.1	-2,05	0,020843	0,479521		
TC1100001077.mm.1	-1,11	Myo1c	JUC1100005211.mm.1	-2,17	0,002401	0,348564		
TC1100002677.mm.1	1,08	Gm12200	JUC1100012672.mm.1	-2,05	0,047826	0,559283		
TC1100003087.mm.1	-1,16	Bcl6b	JUC1100014897.mm.1	-2,05	0,047134	0,557915		
TC1100001391.mm.1	1,01		JUC1100006841.mm.1	-2,05	0,011325	0,427133		
TC0400003321.mm.1	-1,15	Cyp4b1	JUC0400013800.mm.1	-2,05	0,027401	0,503961		
TC0900003089.mm.1	-1,26	Fbxw15	JUC0900014632.mm.1	-2,05	0,047544	0,558701		
TC0500000068.mm.1	-1	Gm8871; Gm6465; Gm58	JUC0500000348.mm.1	-2,05	0,017243	0,462729		
TC0500000068.mm.1	-1	Gm8871; Gm6465; Gm58	JUC0500000352.mm.1	-2,05	0,017243	0,462729		
TC0900002956.mm.1	1,38	Ephb1	JUC0900013557.mm.1	-2,05	0,021955	0,485095		
TC0900002956.mm.1	1,38	Ephb1	JUC0900013553.mm.1	-2,19	0,000453	0,298307		
TC0900002956.mm.1	1,38	Ephb1	JUC0900013565.mm.1	-2,34	0,010808	0,424055		
TC0900002956.mm.1	1,38	Ephb1	JUC0900013562.mm.1	-2,39	0,029454	0,51008		
TC0400001651.mm.1	-1,14	Runx3	JUC0400006819.mm.1	-2,05	0,018473	0,468729		
TC0400001651.mm.1	-1,14	Runx3	JUC0400006815.mm.1	-2,18	0,021557	0,483034		
TC0400002309.mm.1	1,71	Klhl32	JUC0400009944.mm.1	-2,05	0,021302	0,481714		
TC0400002309.mm.1	1,71	Klhl32	JUC0400009934.mm.1	-2,35	0,001758	0,335996		
TC0400002309.mm.1	1,71	Klhl32	JUC0400009937.mm.1	-2,44	0,035384	0,528536		
TC0400002309.mm.1	1,71	Klhl32	JUC0400009940.mm.1	-2,52	0,003139	0,353892		
TC0X00003166.mm.1	1,42	Tro	JUC0X00010030.mm.1	-2,05	0,009681	0,417605		
TC0X00003166.mm.1	1,42	Tro	JUC0X00010045.mm.1	-2,06	0,035373	0,528536		
TC0400001570.mm.1	2,05	Srsf4	PSR0400012356.mm.1	-2,06	0,003268	0,354243	Alternative 3' Acce	0,17
TC0400001570.mm.1	2,05	Srsf4	PSR0400012362.mm.1	-2,77	0,027609	0,504556	Intron Retention	0,37
TC0400001570.mm.1	2,05	Srsf4	JUC0400006457.mm.1	-3,71	0,035878	0,52938		
TC0X00002407.mm.1	3,31	Gabra3	PSR0X00014837.mm.1	-2,06	0,010442	0,421595	Cassette Exon	0,18
TC0X00002407.mm.1	3,31	Gabra3	PSR0X00014849.mm.1	-2,12	0,013713	0,444758	Alternative 5' Donc	0,32
TC0X00002407.mm.1	3,31	Gabra3	PSR0X00014835.mm.1	-2,24	0,018017	0,466939	Cassette Exon	0,16
TC0X00002407.mm.1	3,31	Gabra3	JUC0X00007561.mm.1	-2,73	0,006375	0,389488		
TC0X00002407.mm.1	3,31	Gabra3	JUC0X00007568.mm.1	-3,24	0,046326	0,556186		
TC0X00002407.mm.1	3,31	Gabra3	JUC0X00007571.mm.1	-4,01	0,02697	0,502831		
TC0X00002407.mm.1	3,31	Gabra3	PSR0X00014850.mm.1	-5,44	0,008714	0,409266	Alternative 5' Donc	0,32
TC0100001883.mm.1	2,31	A330023F24Rik; Mir29c;	PSR0100015665.mm.1	-2,06	0,01525	0,452847	Cassette Exon	0,25

TC0100001883.mm.1	2,31	A330023F24Rik; Mir29c; I	JUC0100008910.mm.1	-2,08	0,006927	0,395156		
TC0100001883.mm.1	2,31	A330023F24Rik; Mir29c; I	PSR0100015648.mm.1	-2,25	0,027044	0,502979	Cassette Exon	0,14
TC0100001883.mm.1	2,31	A330023F24Rik; Mir29c; I	PSR0100015668.mm.1	-2,3	0,008472	0,406734	Alternative 5' Donc	0,21
TC0100001883.mm.1	2,31	A330023F24Rik; Mir29c; I	JUC0100008909.mm.1	-4,61	0,047763	0,559125		
TC0100001883.mm.1	2,31	A330023F24Rik; Mir29c; I	JUC0100008913.mm.1	-7,69	0,000027	0,229534		
TC0200000465.mm.1	1,04	Adamtsl2	PSR0200003129.mm.1	-2,06	0,049869	0,563773	Intron Retention	0,25
TC0200000465.mm.1	1,04	Adamtsl2	JUC0200001492.mm.1	-2,17	0,027374	0,503961		
TC0200000465.mm.1	1,04	Adamtsl2	JUC0200001487.mm.1	-2,64	0,020849	0,479521		
TC0200000465.mm.1	1,04	Adamtsl2	JUC0200001505.mm.1	-3,24	0,031679	0,51741		
TC0600000067.mm.1	2,54	Nxph1	PSR0600000552.mm.1	-2,06	0,018484	0,46874	Cassette Exon	0,25
TC0600000067.mm.1	2,54	Nxph1	JUC0600000290.mm.1	-2,38	0,038099	0,535633		
TC0600000067.mm.1	2,54	Nxph1	PSR0600000553.mm.1	-2,61	0,031961	0,518173	Cassette Exon	0,21
TC0600000067.mm.1	2,54	Nxph1	PSR0600000548.mm.1	-2,71	0,023657	0,491292	Alternative 3' Acce	0,17
TC0600000067.mm.1	2,54	Nxph1	PSR0600000551.mm.1	-2,95	0,014358	0,448293	Cassette Exon	0,25
TC0600000067.mm.1	2,54	Nxph1	PSR0600000550.mm.1	-3,32	0,034067	0,524583	Cassette Exon	0,25
TC0400002244.mm.1	1,59	Decr1	JUC0400009794.mm.1	-2,06	0,026074	0,499646		
TC0400002244.mm.1	1,59	Decr1	JUC0400009793.mm.1	-2,15	0,022685	0,48769		
TC0400002244.mm.1	1,59	Decr1	PSR0400018716.mm.1	-2,23	0,033464	0,522822	Cassette Exon	0,16
TC0400002244.mm.1	1,59	Decr1	JUC0400009795.mm.1	-2,43	0,019195	0,472847		
TC0400002244.mm.1	1,59	Decr1	PSR0400018719.mm.1	-2,68	0,002468	0,349135	Cassette Exon	0,24
TC0400002244.mm.1	1,59	Decr1	PSR0400018713.mm.1	-3,03	0,000465	0,298999	Cassette Exon	0,2
TC0400002244.mm.1	1,59	Decr1	JUC0400009791.mm.1	-3,36	0,012843	0,437742		
TC1200001344.mm.1	2,06	1110002L01Rik	PSR1200009639.mm.1	-2,06	0,009844	0,417839	Alternative 5' Donc	0,24
TC1200001344.mm.1	2,06	1110002L01Rik	PSR1200009635.mm.1	-2,09	0,02249	0,487114	Cassette Exon	0,13
TC1400002798.mm.1	1,77	Mettl17	PSR1400004645.mm.1	-2,06	0,014819	0,451071	Intron Retention	0,24
TC1400002798.mm.1	1,77	Mettl17	JUC1400002695.mm.1	-2,34	0,03994	0,540973		
TC1400002798.mm.1	1,77	Mettl17	PSR1400004654.mm.1	-2,51	0,042988	0,548408	Alternative 3' Acce	0,24
TC1900000943.mm.1	2,42	Peli3	PSR1900008342.mm.1	-2,06	0,014234	0,447947	Cassette Exon	0,24
TC1900000943.mm.1	2,42	Peli3	PSR1900008350.mm.1	-2,14	0,038471	0,536438	Cassette Exon	0,2
TC1900000943.mm.1	2,42	Peli3	PSR1900008343.mm.1	-2,59	0,007864	0,403545	Alternative 3' Acce	0,2
TC1900000943.mm.1	2,42	Peli3	JUC1900004584.mm.1	-5,31	0,009422	0,415976		
TC1500000099.mm.1	1,45	Zfr; Mir1898	JUC1500000564.mm.1	-2,06	0,041983	0,546028		
TC1500000099.mm.1	1,45	Zfr; Mir1898	PSR1500000916.mm.1	-2,07	0,049962	0,56403	Cassette Exon	0,13
TC1500000099.mm.1	1,45	Zfr; Mir1898	PSR1500000942.mm.1	-2,1	0,03491	0,52699	Alternative 5' Donc	0,1
TC1500000099.mm.1	1,45	Zfr; Mir1898	PSR1500000915.mm.1	-2,16	0,006725	0,393343	Cassette Exon	0,1
TC1500000099.mm.1	1,45	Zfr; Mir1898	PSR1500000906.mm.1	-2,17	0,038023	0,535471	Cassette Exon	0,03
TC1500000099.mm.1	1,45	Zfr; Mir1898	JUC1500000552.mm.1	-2,24	0,006721	0,393343		
TC1500000099.mm.1	1,45	Zfr; Mir1898	PSR1500000912.mm.1	-2,72	0,045064	0,553102	Alternative 3' Acce	0,21
TC0300001359.mm.1	1,61	Cxxc4	JUC0300005732.mm.1	-2,06	0,035941	0,529399		
TC0300001359.mm.1	1,61	Cxxc4	PSR0300010912.mm.1	-2,82	0,018808	0,470836	Cassette Exon	0,19
TC1700002597.mm.1	1,64	Slc8a1	JUC1700012823.mm.1	-2,06	0,028033	0,505851		
TC1700002597.mm.1	1,64	Slc8a1	JUC1700012826.mm.1	-2,07	0,029	0,508842		
TC1700002597.mm.1	1,64	Slc8a1	PSR1700023789.mm.1	-2,25	0,048579	0,56104	Alternative 3' Acce	0,19
TC0900003163.mm.1	1,59	Fbxl2	PSR0900026757.mm.1	-2,06	0,0308	0,514144	Alternative 5' Donc	0,17
TC0900003163.mm.1	1,59	Fbxl2	JUC0900015041.mm.1	-2,3	0,000317	0,288663		
TC0900003163.mm.1	1,59	Fbxl2	JUC0900015047.mm.1	-3,04	0,003816	0,35811		
TC1100000365.mm.1	1,33	Gm12121	PSR1100003046.mm.1	-2,06	0,032437	0,519517	Intron Retention	0,17
TC1100000365.mm.1	1,33	Gm12121	PSR1100003034.mm.1	-2,65	0,016125	0,457772		

TC1900000197.mm.1	1,38	Syt7	JUC1900001287.mm.1	-2,06	0,043098	0,548554		
TC1900000197.mm.1	1,38	Syt7	JUC1900001285.mm.1	-2,08	0,028885	0,50858		
TC1900000197.mm.1	1,38	Syt7	PSR1900002486.mm.1	-2,15	0,031476	0,516924	Alternative 3' Acce	0,17
TC1900000197.mm.1	1,38	Syt7	JUC1900001283.mm.1	-2,2	0,008707	0,409126		
TC1900000197.mm.1	1,38	Syt7	PSR1900002501.mm.1	-2,28	0,001035	0,316361		
TC1900000197.mm.1	1,38	Syt7	JUC1900001281.mm.1	-4,38	0,002655	0,349612		
TC1000000039.mm.1	1,38	Oprm1	JUC1000000134.mm.1	-2,06	0,031546	0,517128		
TC1000000039.mm.1	1,38	Oprm1	PSR1000000297.mm.1	-2,15	0,045556	0,554404	Alternative 5' Donc	0,1
TC1000000039.mm.1	1,38	Oprm1	PSR1000000253.mm.1	-2,19	0,003206	0,354243	Cassette Exon	0,14
TC1000000039.mm.1	1,38	Oprm1	PSR1000000293.mm.1	-2,34	0,017074	0,462124	Alternative 3' Acce	0,16
TC1000000039.mm.1	1,38	Oprm1	JUC1000000176.mm.1	-2,81	0,00369	0,357266		
TC1000000039.mm.1	1,38	Oprm1	JUC1000000139.mm.1	-2,84	0,013384	0,442371		
TC0100001905.mm.1	-1,13	Sox17	PSR0100015743.mm.1	-2,06	0,017202	0,462491	Alternative 5' Donc	0,15
TC0400003292.mm.1	1,6	Skint10	PSR0400026202.mm.1	-2,06	0,029065	0,509008	Cassette Exon	0,15
TC0400003292.mm.1	1,6	Skint10	PSR0400026199.mm.1	-2,21	0,007169	0,397855	Cassette Exon	0,11
TC0400003292.mm.1	1,6	Skint10	JUC0400013624.mm.1	-3,67	0,042899	0,548193		
TC0600001449.mm.1	1,33	Iffo1	PSR0600011826.mm.1	-2,06	0,013407	0,442624	Alternative 3' Acce	0,15
TC0600001449.mm.1	1,33	Iffo1	JUC0600006238.mm.1	-2,25	0,032014	0,518371		
TC0800001728.mm.1	3,12	Grtp1	PSR0800013986.mm.1	-2,06	0,008733	0,409583	Cassette Exon	0,09
TC0800001728.mm.1	3,12	Grtp1	PSR0800013989.mm.1	-2,66	0,002455	0,349135	Cassette Exon	0,15
TC1500001857.mm.1	1,03	Gm17753	PSR1500014487.mm.1	-2,06	0,027922	0,505612	Cassette Exon	0,14
TC0X00002185.mm.1	1,83	Firre; 6720401G13Rik	PSR0X00013887.mm.1	-2,06	0,001131	0,317328	Intron Retention	0,13
TC0X00002185.mm.1	1,83	Firre; 6720401G13Rik	PSR0X00013956.mm.1	-2,11	0,025995	0,499207	Cassette Exon	0,12
TC0X00002185.mm.1	1,83	Firre; 6720401G13Rik	JUC0X00007090.mm.1	-2,23	0,010412	0,42148		
TC0X00002185.mm.1	1,83	Firre; 6720401G13Rik	JUC0X00007145.mm.1	-2,53	0,017757	0,465425		
TC0X00002185.mm.1	1,83	Firre; 6720401G13Rik	JUC0X00007155.mm.1	-3,36	0,034797	0,526823		
TC1000002714.mm.1	1,14	Plxnc1	PSR1000020857.mm.1	-2,06	0,012817	0,437465	Cassette Exon	0,12
TC0200000562.mm.1	1,01	Nup214; Mir7674; mmu-m	PSR0200004919.mm.1	-2,06	0,008247	0,405976	Alternative 5' Donc	0,1
TC0400002230.mm.1	1,12	Gm2560	PSR0400018623.mm.1	-2,06	0,028657	0,507969	Cassette Exon	0,1
TC0500000689.mm.1	1,27	Dcun1d4	PSR0500006077.mm.1	-2,06	0,048909	0,5618	Cassette Exon	0,1
TC0800002425.mm.1	1,85	Abhd8	PSR0800018511.mm.1	-2,06	0,044852	0,5526	Cassette Exon	0,1
TC1300000883.mm.1	-1,21	Slc6a3	PSR1300005668.mm.1	-2,06	0,033498	0,522941	Cassette Exon	0,1
TC1400002283.mm.1	1,2	Ppp2r2a	PSR1400017632.mm.1	-2,06	0,022454	0,487003	Alternative 5' Donc	0,1
TC1800000401.mm.1	1,93	Ndfip1	PSR1800002992.mm.1	-2,06	0,039269	0,538976	Cassette Exon	0,1
TC0500003717.mm.1	1,2	Rsph10b	PSR0500016087.mm.1	-2,06	0,006157	0,387873	Cassette Exon	0,09
TC0500003717.mm.1	1,2	Rsph10b	JUC0500008745.mm.1	-2,06	0,019609	0,474765		
TC1400000993.mm.1	1,01	Gm26908; LOC102636905	PSR1400007983.mm.1	-2,06	0,026531	0,501411	Cassette Exon	0,09
TC1300000266.mm.1	-1,32	Slc17a3	PSR1300001521.mm.1	-2,06	0,001536	0,330388	Cassette Exon	0,08
TC0300000218.mm.1	1,11	Kcnmb2	JUC0300000606.mm.1	-2,06	0,030221	0,512452		
TC0300000538.mm.1	1,22	Rsrc1	JUC0300001928.mm.1	-2,06	0,009384	0,415457		
TC1100003849.mm.1	-1,03	2810433D01Rik	JUC1100018759.mm.1	-2,06	0,019741	0,474932		
TC1200000422.mm.1	1,39	Nubpl	JUC1200001676.mm.1	-2,06	0,025169	0,496555		
TC1300002431.mm.1	-1,28	Papd4	JUC1300008566.mm.1	-2,06	0,0401	0,541437		
TC1300001128.mm.1	1,26	Zfp366	JUC1300003732.mm.1	-2,06	0,015298	0,453376		
TC0200005511.mm.1	1,3	Rbm12; Cpne1; RP23-220D	PSR0200042445.mm.1	-2,06	0,034489	0,525929		
TC0200005511.mm.1	1,3	Rbm12; Cpne1; RP23-220D	JUC0200021948.mm.1	-3,98	0,00438	0,366096		
TC1700001563.mm.1	1,15	Zfp942	JUC1700007457.mm.1	-2,06	0,019967	0,476202		
TC0200001879.mm.1	-1,19	Slc12a1	JUC0200007322.mm.1	-2,06	0,029621	0,510558		

TC0200001829.mm.1	1,21	Ctdspl2	JUC0200007095.mm.1	-2,06	0,004323	0,364614		
TC0200001829.mm.1	1,21	Ctdspl2	JUC0200007099.mm.1	-2,86	0,032067	0,518469		
TC0200002157.mm.1	1,88	Slc24a3	JUC0200008591.mm.1	-2,06	0,019163	0,472719		
TC0800001141.mm.1	-1,05		JUC0800004813.mm.1	-2,06	0,018392	0,468484		
TC0700004565.mm.1	1,56	Ctsd; Ifitm10	JUC0700020261.mm.1	-2,06	0,007249	0,39826		
TC0700004569.mm.1	-1,49	H19; Mir675	JUC0700020285.mm.1	-2,06	0,038799	0,537586		
TC0700000024.mm.1	1,29	Leng8	JUC0700000165.mm.1	-2,06	0,018306	0,468049		
TC0600002553.mm.1	1,08	Sema4f	JUC0600010282.mm.1	-2,06	0,009361	0,415092		
TC0600002553.mm.1	1,08	Sema4f	JUC0600010274.mm.1	-2,48	0,005248	0,377696		
TC0600002553.mm.1	1,08	Sema4f	JUC0600010269.mm.1	-2,77	0,019323	0,473305		
TC0500003378.mm.1	1,17	Limk1	JUC0500016588.mm.1	-2,06	0,047353	0,558291		
TC0500003378.mm.1	1,17	Limk1	JUC0500016595.mm.1	-2,13	0,002886	0,352207		
TC0500003378.mm.1	1,17	Limk1	JUC0500016591.mm.1	-2,35	0,011919	0,431655		
TC0700002978.mm.1	1,31	Ap2a1	JUC0700014250.mm.1	-2,06	0,038927	0,537874		
TC0500003604.mm.1	1,14	Gm6309	JUC0500018014.mm.1	-2,06	0,011728	0,429969		
TC0600000227.mm.1	-1,23	Fam71f2	JUC0600000717.mm.1	-2,06	0,047749	0,559097		
TC1100000776.mm.1	1	Aldh3a1	JUC1100003236.mm.1	-2,06	0,040237	0,541714		
TC0300001949.mm.1	1,01	LOC102639521	JUC0300007800.mm.1	-2,06	0,04643	0,556547		
TC1100003256.mm.1	-1,26		JUC1100015706.mm.1	-2,06	0,009313	0,414883		
TC1100001550.mm.1	1,08	Lasp1	JUC1100007447.mm.1	-2,06	0,018999	0,471564		
TC0300002311.mm.1	1,43	Slc25a44	JUC0300009204.mm.1	-2,06	0,042445	0,547627		
TC1100002015.mm.1	-1,1	Baiap2	JUC1100010109.mm.1	-2,06	0,033582	0,523229		
TC0300002350.mm.1	1,04	Zbtb7b	PSR0300018105.mm.1	-2,06	0,013104	0,439759		
TC0300002350.mm.1	1,04	Zbtb7b	JUC0300009466.mm.1	-2,12	0,048946	0,5618		
TC0X00001555.mm.1	2,2	Rragb	JUC0X00004998.mm.1	-2,06	0,019461	0,474165		
TC0X00001555.mm.1	2,2	Rragb	JUC0X00004988.mm.1	-2,81	0,01125	0,426718		
TC0400001770.mm.1	1,25	Padi2	JUC0400007685.mm.1	-2,06	0,000579	0,304044		
TC1000000560.mm.1	-1,13	Tbata	JUC1000002101.mm.1	-2,06	0,029785	0,511132		
TC0400001935.mm.1	1,58	Masp2	JUC0400008343.mm.1	-2,06	0,015158	0,452699		
TC1000000870.mm.1	1,11	Dot1l	JUC1000003602.mm.1	-2,06	0,006014	0,386586		
TC1100001158.mm.1	4,21	Unc119	JUC1100005661.mm.1	-2,07	0,000952	0,313363		
TC1100001158.mm.1	4,21	Unc119	JUC1100005663.mm.1	-2,92	0,002055	0,341838		
TC1100001158.mm.1	4,21	Unc119	PSR1100010790.mm.1	-3	0,002624	0,349612	Cassette Exon	0,17
TC1100001158.mm.1	4,21	Unc119	PSR1100010804.mm.1	-3,22	0,03399	0,52432	Alternative 5' Donc	0,35
TC1100001158.mm.1	4,21	Unc119	PSR1100010800.mm.1	-4,26	0,003156	0,353892	Alternative 3' Acce	0,52
TC1100001158.mm.1	4,21	Unc119	PSR1100010791.mm.1	-4,26	0,013982	0,446291	Alternative 3' Acce	0,48
TC1100001158.mm.1	4,21	Unc119	PSR1100010797.mm.1	-4,42	0,003727	0,357586	Intron Retention	0,67
TC1100001158.mm.1	4,21	Unc119	PSR1100010789.mm.1	-5,54	0,019592	0,474685	Cassette Exon	0,29
TC1100001158.mm.1	4,21	Unc119	PSR1100010796.mm.1	-10,92	0,001792	0,336311	Intron Retention	0,67
TC0200005504.mm.1	5,66	Serinc4	PSR0200038081.mm.1	-2,07	0,041503	0,544746		
TC0200005504.mm.1	5,66	Serinc4	PSR0200038091.mm.1	-2,65	0,00132	0,324348	Alternative 5' Donc	0,17
TC0200005504.mm.1	5,66	Serinc4	JUC0200019626.mm.1	-2,82	0,046695	0,556856		
TC0200005504.mm.1	5,66	Serinc4	PSR0200038086.mm.1	-3,17	0,001237	0,322251	Cassette Exon	0,34
TC0200005504.mm.1	5,66	Serinc4	PSR0200038102.mm.1	-3,57	0,009854	0,417891	Alternative 5' Donc	0,41
TC0200005504.mm.1	5,66	Serinc4	JUC0200019624.mm.1	-5,36	0,025882	0,498873		
TC1100000296.mm.1	2,03	Mtif2	PSR1100002512.mm.1	-2,07	0,024088	0,493089	Intron Retention	0,21
TC1100000296.mm.1	2,03	Mtif2	JUC1100001329.mm.1	-2,07	0,040126	0,541532		
TC1100000296.mm.1	2,03	Mtif2	PSR1100002500.mm.1	-3,46	0,022001	0,485226	Alternative 5' Donc	0,36

TC1100001198.mm.1	2,63	Rhot1	JUC1100005891.mm.1	-2,07	0,02387	0,492241		
TC1100001198.mm.1	2,63	Rhot1	JUC1100005900.mm.1	-2,51	0,015423	0,454071		
TC1100001198.mm.1	2,63	Rhot1	JUC1100005909.mm.1	-2,52	0,046985	0,557657		
TC1100001198.mm.1	2,63	Rhot1	PSR1100011216.mm.1	-2,82	0,013855	0,445409	Alternative 3' Acce	0,29
TC1100001198.mm.1	2,63	Rhot1	PSR1100011183.mm.1	-3,23	0,014183	0,447608	Alternative 3' Acce	0,25
TC1100001198.mm.1	2,63	Rhot1	PSR1100011212.mm.1	-3,3	0,039384	0,539438	Alternative 3' Acce	0,35
TC1100001198.mm.1	2,63	Rhot1	PSR1100011193.mm.1	-3,8	0,001678	0,335182	Alternative 5' Donc	0,17
TC1100001198.mm.1	2,63	Rhot1	JUC1100005908.mm.1	-5,65	0,00478	0,371122		
TC0400002480.mm.1	2,45	Ccl27a	PSR0400020088.mm.1	-2,07	0,04258	0,548047	Intron Retention	0,29
TC0400002480.mm.1	2,45	Ccl27a	PSR0400020087.mm.1	-2,63	0,005005	0,373851	Intron Retention	0,29
TC0400002480.mm.1	2,45	Ccl27a	PSR0400020071.mm.1	-2,98	0,013358	0,442089	Cassette Exon	0,23
TC0400002480.mm.1	2,45	Ccl27a	JUC0400010446.mm.1	-3,01	0,013632	0,444143		
TC0400002480.mm.1	2,45	Ccl27a	PSR0400020095.mm.1	-3,59	0,009306	0,414883		
TC0400002480.mm.1	2,45	Ccl27a	PSR0400020104.mm.1	-4,38	0,019449	0,474131	Alternative 5' Donc	0,16
TC1900000916.mm.1	1,43	Adrbk1	PSR1900008000.mm.1	-2,07	0,021955	0,485095	Intron Retention	0,27
TC1900000916.mm.1	1,43	Adrbk1	JUC1900004403.mm.1	-2,17	0,013574	0,443703		
TC1000002794.mm.1	1,46	Tmtc2	PSR1000021270.mm.1	-2,07	0,04361	0,550141	Cassette Exon	0,1
TC1000002794.mm.1	1,46	Tmtc2	PSR1000021268.mm.1	-2,26	0,042103	0,546511	Alternative 3' Acce	0,14
TC1000002794.mm.1	1,46	Tmtc2	PSR1000021287.mm.1	-2,69	0,023627	0,491012	Alternative 5' Donc	0,25
TC1000002794.mm.1	1,46	Tmtc2	PSR1000021265.mm.1	-3,07	0,027601	0,504485	Cassette Exon	0,13
TC1100001100.mm.1	1,54	Timm22	PSR1100010006.mm.1	-2,07	0,019103	0,472235	Alternative 5' Donc	0,22
TC1100001100.mm.1	1,54	Timm22	JUC1100005265.mm.1	-2,52	0,015292	0,453368		
TC1100001100.mm.1	1,54	Timm22	PSR1100010013.mm.1	-2,61	0,018869	0,4711	Alternative 5' Donc	0,24
TC0600000016.mm.1	1,37	Ppp1r9a	PSR0600000229.mm.1	-2,07	0,005311	0,378059	Alternative 5' Donc	0,2
TC0600000016.mm.1	1,37	Ppp1r9a	PSR0600000231.mm.1	-2,07	0,022863	0,488467	Alternative 5' Donc	0,2
TC0600000016.mm.1	1,37	Ppp1r9a	PSR0600000230.mm.1	-2,28	0,042633	0,548097	Alternative 5' Donc	0,2
TC0600000016.mm.1	1,37	Ppp1r9a	PSR0600000232.mm.1	-2,54	0,022026	0,485249	Cassette Exon	0,19
TC0600000016.mm.1	1,37	Ppp1r9a	JUC0600000155.mm.1	-2,83	0,004428	0,366832		
TC1600001971.mm.1	1,04	Cyyr1; Gm2541	PSR1600015270.mm.1	-2,07	0,044082	0,550916	Alternative 5' Donc	0,2
TC1600001971.mm.1	1,04	Cyyr1; Gm2541	JUC1600007878.mm.1	-2,92	0,029881	0,51129		
TC0500001267.mm.1	1,19	Gcn1l1; Mir7029; Gm1312	JUC0500005992.mm.1	-2,07	0,017505	0,463964		
TC0500001267.mm.1	1,19	Gcn1l1; Mir7029; Gm1312	PSR0500011007.mm.1	-2,22	0,010699	0,423322	Cassette Exon	0,11
TC0500001267.mm.1	1,19	Gcn1l1; Mir7029; Gm1312	PSR0500011035.mm.1	-2,47	0,014179	0,447583	Cassette Exon	0,19
TC0900002791.mm.1	-1,03	Tbx18	PSR0900023135.mm.1	-2,07	0,004073	0,361986	Alternative 5' Donc	0,19
TC1600001079.mm.1	1,79	Wrb	PSR1600008537.mm.1	-2,07	0,002869	0,352207	Alternative 5' Donc	0,18
TC1600001079.mm.1	1,79	Wrb	PSR1600008532.mm.1	-2,69	0,004436	0,366981	Alternative 5' Donc	0,14
TC1600001079.mm.1	1,79	Wrb	JUC1600004435.mm.1	-4,26	0,039374	0,539409		
TC1600001236.mm.1	1,12	Pdxdc1	PSR1600009915.mm.1	-2,07	0,008927	0,410972	Intron Retention	0,14
TC1600001236.mm.1	1,12	Pdxdc1	PSR1600009930.mm.1	-2,17	0,022129	0,485558	Alternative 3' Acce	0,18
TC1100001018.mm.1	1,06	Shpk	PSR1100009243.mm.1	-2,07	0,008572	0,40821	Alternative 3' Acce	0,17
TC1100001018.mm.1	1,06	Shpk	JUC1100004864.mm.1	-2,31	0,005258	0,377945		
TC1800001228.mm.1	1,19	1700086O06Rik	JUC1800005093.mm.1	-2,07	0,026149	0,50001		
TC1800001228.mm.1	1,19	1700086O06Rik	PSR1800009128.mm.1	-2,27	0,015862	0,456449	Cassette Exon	0,16
TC0100000665.mm.1	1,4	Slc4a3	PSR0100005747.mm.1	-2,07	0,03121	0,51581	Alternative 5' Donc	0,15
TC0700002116.mm.1	1,33	Oraov1	PSR0700019068.mm.1	-2,07	0,03884	0,537749	Alternative 3' Acce	0,15
TC0800003036.mm.1	1,28	A330074K22Rik	PSR0800023389.mm.1	-2,07	0,04935	0,562806	Alternative 5' Donc	0,14
TC1300002481.mm.1	-1,25	Hexb	PSR1300016648.mm.1	-2,07	0,021905	0,484824	Alternative 3' Acce	0,14
TC1600001754.mm.1	2,32	Gm19797	PSR1600014247.mm.1	-2,07	0,003563	0,356223	Cassette Exon	0,14

TC1600001754.mm.1	2,32	Gm19797	PSR1600014243.mm.1	-2,18	0,046428	0,556547	Cassette Exon	0,12
TC1600001754.mm.1	2,32	Gm19797	PSR1600014242.mm.1	-2,23	0,000324	0,288663	Cassette Exon	0,11
TC0100003074.mm.1	2,31	Tmcc2	PSR0100024679.mm.1	-2,07	0,002708	0,349612	Cassette Exon	0,12
TC0100003074.mm.1	2,31	Tmcc2	PSR0100024683.mm.1	-2,1	0,006644	0,392838	Cassette Exon	0,09
TC0100003074.mm.1	2,31	Tmcc2	PSR0100024684.mm.1	-2,23	0,024147	0,493245	Cassette Exon	0,13
TC0100003074.mm.1	2,31	Tmcc2	JUC0100014011.mm.1	-2,55	0,009387	0,415457		
TC1200001299.mm.1	1,69	Vipr2	PSR1200009350.mm.1	-2,07	0,004523	0,368648	Cassette Exon	0,13
TC1200001299.mm.1	1,69	Vipr2	JUC1200005155.mm.1	-2,34	0,014038	0,44654		
TC1200001299.mm.1	1,69	Vipr2	JUC1200005163.mm.1	-3,21	0,015625	0,45509		
TC0700000561.mm.1	1,65	Tyrobp	PSR0700004867.mm.1	-2,07	0,003702	0,357266	Cassette Exon	0,12
TC1600000324.mm.1	1,03	Fetub	PSR1600003215.mm.1	-2,07	0,033999	0,524371	Cassette Exon	0,12
TC1600001471.mm.1	1,43	Xxylt1	PSR1600012140.mm.1	-2,07	0,014256	0,447947	Cassette Exon	0,12
TC0600003333.mm.1	-1,32	Smco3	PSR0600026156.mm.1	-2,07	0,046318	0,556186	Cassette Exon	0,1
TC1100001643.mm.1	1,73	Tubg2	JUC1100007936.mm.1	-2,07	0,00743	0,399925		
TC1100001643.mm.1	1,73	Tubg2	JUC1100007937.mm.1	-2,08	0,040241	0,541721		
TC1100001643.mm.1	1,73	Tubg2	PSR1100015314.mm.1	-2,12	0,007564	0,401384	Cassette Exon	0,1
TC0X00003098.mm.1	-1,2	Acsl4	PSR0X00019491.mm.1	-2,07	0,000581	0,304044	Cassette Exon	0,01
TC1400000768.mm.1	-1,01		JUC1400003188.mm.1	-2,07	0,014013	0,446443		
TC1200001982.mm.1	1,39	Zfyve26	JUC1200007540.mm.1	-2,07	0,026488	0,501252		
TC1300001811.mm.1	-1,16		JUC1300005780.mm.1	-2,07	0,046474	0,556596		
TC0200003643.mm.1	-1,25	Fign	JUC0200015782.mm.1	-2,07	0,032318	0,519155		
TC0200004930.mm.1	1,7	Nol4l; 8430427H17Rik	JUC0200021561.mm.1	-2,07	0,036723	0,531766		
TC0200004930.mm.1	1,7	Nol4l; 8430427H17Rik	JUC0200021572.mm.1	-2,43	0,013388	0,442391		
TC0200004930.mm.1	1,7	Nol4l; 8430427H17Rik	JUC0200021577.mm.1	-2,88	0,02764	0,504658		
TC0200004930.mm.1	1,7	Nol4l; 8430427H17Rik	JUC0200021570.mm.1	-3,71	0,004227	0,363295		
TC0200005028.mm.1	1,06	Tti1	JUC0200022200.mm.1	-2,07	0,033448	0,522793		
TC1800000107.mm.1	1,17	Cabyr	JUC1800000481.mm.1	-2,07	0,01657	0,459612		
TC1700000921.mm.1	1,06	Med20; Usp49; Gm20517	JUC1700004725.mm.1	-2,07	0,040874	0,543423		
TC1700001903.mm.1	-1,28	Gm20496	JUC1700009516.mm.1	-2,07	0,042475	0,547627		
TC1800001215.mm.1	1,26		JUC1800004968.mm.1	-2,07	0,000378	0,293011		
TC0100001129.mm.1	-1,21	R3hdm1	JUC0100005314.mm.1	-2,07	0,044395	0,551441		
TC1500001643.mm.1	-1,03	Oc90	JUC1500006949.mm.1	-2,07	0,009306	0,414883		
TC1600002182.mm.1	1,47	Donson	JUC1600008259.mm.1	-2,07	0,008884	0,410642		
TC0800000012.mm.1	-1,19	C330021F23Rik	JUC0800000102.mm.1	-2,07	0,027704	0,504872		
TC0500002478.mm.1	-1,29		JUC0500012019.mm.1	-2,07	0,034369	0,525628		
TC0900001477.mm.1	2,56	Lrrc2	JUC0900006929.mm.1	-2,07	0,024724	0,494838		
TC0900001477.mm.1	2,56	Lrrc2	JUC0900006928.mm.1	-4,54	0,048911	0,5618		
TC0500001638.mm.1	-1,06	Azgp1	JUC0500008155.mm.1	-2,07	0,032094	0,51852		
TC0600001917.mm.1	-1,14	Tspan12	JUC0600007851.mm.1	-2,07	0,043446	0,549785		
TC0600000553.mm.1	-1,05	Sspo	JUC0600002071.mm.1	-2,07	0,023966	0,49267		
TC0600000553.mm.1	-1,05	Sspo	JUC0600002100.mm.1	-2,75	0,042837	0,548097		
TC1000003050.mm.1	1,1	Xpot; Gm24330	JUC1000012464.mm.1	-2,07	0,040433	0,542058		
TC0400000531.mm.1	1,05	Invs	JUC0400001973.mm.1	-2,07	0,036949	0,532361		
TC1100002908.mm.1	1,02	Slc47a2	JUC1100013735.mm.1	-2,07	0,009298	0,414883		
TC0X00000691.mm.1	-1,04	Abcd1	JUC0X00002264.mm.1	-2,07	0,008149	0,405473		
TC0400004173.mm.1	-1,22	Plekhn1	JUC0400018273.mm.1	-2,07	0,023782	0,491795		
TC0400001856.mm.1	-1,03	Dhrs3	JUC0400007997.mm.1	-2,07	0,013104	0,439759		
TC0900002405.mm.1	3,52	Bbs4	JUC0900011416.mm.1	-2,08	0,01107	0,425887		

TC0900002405.mm.1	3,52	Bbs4	JUC0900011425.mm.1	-2,51	0,009647	0,417354		
TC0900002405.mm.1	3,52	Bbs4	PSR0900020237.mm.1	-3,28	0,001677	0,335182	Cassette Exon	0,26
TC0900002405.mm.1	3,52	Bbs4	PSR0900020233.mm.1	-3,97	0,001476	0,329052	Intron Retention	0,68
TC0900002405.mm.1	3,52	Bbs4	PSR0900020239.mm.1	-4,26	0,005882	0,383835	Cassette Exon	0,26
TC0900002405.mm.1	3,52	Bbs4	PSR0900020231.mm.1	-4,82	0,003753	0,357586	Alternative 3' Acce	0,48
TC0900002405.mm.1	3,52	Bbs4	PSR0900020235.mm.1	-5,87	0,000008	0,179072	Intron Retention	0,67
TC0700002855.mm.1	25,5	Rgs9bp	PSR0700025525.mm.1	-2,08	0,012822	0,437482	Cassette Exon	0,16
TC0700002855.mm.1	25,5	Rgs9bp	PSR0700025527.mm.1	-25	0,003318	0,354243	Alternative 5' Donc	0,32
TC1100001155.mm.1	1,54	Spag5	PSR1100010724.mm.1	-2,08	0,028699	0,508019	Alternative 5' Donc	0,09
TC1100001155.mm.1	1,54	Spag5	PSR1100010708.mm.1	-2,25	0,007256	0,39826	Intron Retention	0,3
TC1100001155.mm.1	1,54	Spag5	JUC1100005612.mm.1	-2,26	0,000266	0,28803		
TC0300000307.mm.1	3,39	Hspa4l	JUC0300001148.mm.1	-2,08	0,014724	0,450372		
TC0300000307.mm.1	3,39	Hspa4l	PSR0300002244.mm.1	-2,43	0,003256	0,354243		
TC0300000307.mm.1	3,39	Hspa4l	PSR0300002202.mm.1	-3,19	0,014995	0,452052	Alternative 3' Acce	0,2
TC0300000307.mm.1	3,39	Hspa4l	PSR0300002209.mm.1	-3,64	0,001431	0,327454	Cassette Exon	0,27
TC0300000307.mm.1	3,39	Hspa4l	JUC0300001142.mm.1	-3,93	0,006622	0,392392		
TC0300000307.mm.1	3,39	Hspa4l	PSR0300002199.mm.1	-4	0,004879	0,372195	Cassette Exon	0,28
TC0700000874.mm.1	3,94	Kcnc1	PSR0700007861.mm.1	-2,08	0,038248	0,535883	Cassette Exon	0,09
TC0700000874.mm.1	3,94	Kcnc1	PSR0700007862.mm.1	-2,33	0,022565	0,487303	Cassette Exon	0,09
TC0700000874.mm.1	3,94	Kcnc1	PSR0700007857.mm.1	-2,35	0,018668	0,470205	Alternative 5' Donc	0,15
TC0700000874.mm.1	3,94	Kcnc1	PSR0700007850.mm.1	-2,62	0,042395	0,547599	Cassette Exon	0,2
TC0700000874.mm.1	3,94	Kcnc1	PSR0700007851.mm.1	-2,66	0,025006	0,496061	Cassette Exon	0,15
TC0700000874.mm.1	3,94	Kcnc1	PSR0700007847.mm.1	-2,94	0,033192	0,522148	Cassette Exon	0,2
TC0700000874.mm.1	3,94	Kcnc1	PSR0700007859.mm.1	-4,3	0,017199	0,462491	Cassette Exon	0,26
TC0700000874.mm.1	3,94	Kcnc1	PSR0700007848.mm.1	-4,37	0,012369	0,434906	Alternative 3' Acce	0,22
TC0200005429.mm.1	2,8	Chrna4	PSR0200046235.mm.1	-2,08	0,044495	0,551741	Cassette Exon	0,21
TC0200005429.mm.1	2,8	Chrna4	PSR0200046223.mm.1	-2,3	0,016584	0,459685	Cassette Exon	0,24
TC0200005429.mm.1	2,8	Chrna4	PSR0200046221.mm.1	-3,09	0,010781	0,423955	Cassette Exon	0,22
TC0200005429.mm.1	2,8	Chrna4	JUC0200023823.mm.1	-3,17	0,024548	0,494088		
TC0200005429.mm.1	2,8	Chrna4	JUC0200023829.mm.1	-3,54	0,008215	0,405934		
TC0300002220.mm.1	2,46	Gucy1a3; Mir7010; mmu-r	PSR0300016694.mm.1	-2,08	0,014464	0,449094	Alternative 3' Acce	0,17
TC0300002220.mm.1	2,46	Gucy1a3; Mir7010; mmu-r	PSR0300016701.mm.1	-2,1	0,010539	0,422281	Cassette Exon	0,11
TC0300002220.mm.1	2,46	Gucy1a3; Mir7010; mmu-r	PSR0300016700.mm.1	-2,27	0,007447	0,40013	Cassette Exon	0,12
TC0300002220.mm.1	2,46	Gucy1a3; Mir7010; mmu-r	JUC0300008776.mm.1	-2,79	0,028216	0,506368		
TC0300002220.mm.1	2,46	Gucy1a3; Mir7010; mmu-r	PSR0300016699.mm.1	-2,94	0,005519	0,380501	Cassette Exon	0,21
TC1600000143.mm.1	1,7	Shisa9	PSR1600001006.mm.1	-2,08	0,000814	0,311886	Cassette Exon	0,21
TC1600000143.mm.1	1,7	Shisa9	JUC1600000511.mm.1	-8,72	0,017541	0,464105		
TC0700001766.mm.1	1,49	Rbbp6	JUC0700007869.mm.1	-2,08	0,042259	0,547092		
TC0700001766.mm.1	1,49	Rbbp6	PSR0700015047.mm.1	-2,14	0,033538	0,523131	Cassette Exon	0,18
TC0700001766.mm.1	1,49	Rbbp6	JUC0700007873.mm.1	-2,75	0,027441	0,504022		
TC0500003248.mm.1	-1,04	Gm16001	PSR0500029268.mm.1	-2,08	0,025931	0,499121	Alternative 3' Acce	0,17
TC0X00001335.mm.1	1,46	Tceal1	PSR0X00008389.mm.1	-2,08	0,032049	0,518459	Alternative 3' Acce	0,17
TC0X00001541.mm.1	1,34	Kdm5c	PSR0X00009779.mm.1	-2,08	0,025248	0,496939	Alternative 3' Acce	0,17
TC1000000905.mm.1	2,2	BC025920	PSR1000007224.mm.1	-2,08	0,027307	0,503732	Intron Retention	0,15
TC1000000905.mm.1	2,2	BC025920	PSR1000007228.mm.1	-2,64	0,023831	0,491992	Cassette Exon	0,15
TC1000000905.mm.1	2,2	BC025920	JUC1000003894.mm.1	-2,75	0,017574	0,464258		
TC1000000905.mm.1	2,2	BC025920	PSR1000007229.mm.1	-2,89	0,004898	0,372268	Cassette Exon	0,17
TC1200000744.mm.1	1,04	4933426M11Rik	PSR1200005277.mm.1	-2,08	0,010502	0,42211	Alternative 5' Donc	0,17

TC0700000764.mm.1	1,1	Zfp658	PSR0700006194.mm.1	-2,08	0,016138	0,45788	Alternative 3' Acce	0,16
TC0X00003000.mm.1	1,64	Armcx2	PSR0X00018737.mm.1	-2,08	0,034156	0,524919	Alternative 3' Acce	0,16
TC0X00003000.mm.1	1,64	Armcx2	PSR0X00018738.mm.1	-2,09	0,042133	0,546588	Alternative 3' Acce	0,16
TC0X00003000.mm.1	1,64	Armcx2	PSR0X00018736.mm.1	-2,35	0,021315	0,481714		
TC1100003492.mm.1	1,6	Gm11508	PSR1100031998.mm.1	-2,08	0,018796	0,470789	Alternative 5' Donc	0,16
TC1000000531.mm.1	1,66	P4ha1	PSR1000003556.mm.1	-2,08	0,024778	0,495014	Alternative 5' Donc	0,13
TC0100003502.mm.1	2,14	Rgs4	PSR0100028600.mm.1	-2,08	0,037613	0,53412	Cassette Exon	0,12
TC0300002721.mm.1	1,36	4933431E20Rik	PSR0300021245.mm.1	-2,08	0,006515	0,391382	Alternative 5' Donc	0,12
TC0300002721.mm.1	1,36	4933431E20Rik	PSR0300021244.mm.1	-2,32	0,038817	0,537595	Alternative 5' Donc	0,12
TC0300002721.mm.1	1,36	4933431E20Rik	JUC0300011049.mm.1	-3,23	0,040104	0,541445		
TC0300002721.mm.1	1,36	4933431E20Rik	JUC0300011045.mm.1	-3,3	0,003958	0,359922		
TC0300002721.mm.1	1,36	4933431E20Rik	JUC0300011047.mm.1	-3,35	0,010173	0,419711		
TC1400000033.mm.1	1,24	Gm3269; Gm3594	PSR1400000444.mm.1	-2,08	0,012497	0,435429	Alternative 5' Donc	0,12
TC0300001491.mm.1	2,03	Ssx2ip	PSR0300012063.mm.1	-2,08	0,024666	0,494682	Cassette Exon	0,11
TC0300001491.mm.1	2,03	Ssx2ip	JUC0300006380.mm.1	-2,58	0,046424	0,556537		
TC0600000967.mm.1	1,58	Alms1-ps1	PSR0600007491.mm.1	-2,08	0,017876	0,466276	Cassette Exon	0,11
TC0600000967.mm.1	1,58	Alms1-ps1	JUC0600003824.mm.1	-3,54	0,01374	0,444758		
TC1500002309.mm.1	1,3	Cbx5	PSR1500019104.mm.1	-2,08	0,006222	0,387912	Alternative 3' Acce	0,11
TC1500002309.mm.1	1,3	Cbx5	PSR1500019124.mm.1	-2,08	0,034127	0,524882	Cassette Exon	0,09
TC0500003554.mm.1	-1,15	E130309D02Rik	PSR0500032566.mm.1	-2,08	0,023556	0,490947	Alternative 3' Acce	0,1
TC0X00002631.mm.1	1,19	Pola1	PSR0X00016417.mm.1	-2,08	0,018211	0,467586	Cassette Exon	0,1
TC0X00002631.mm.1	1,19	Pola1	JUC0X00008366.mm.1	-2,29	0,045606	0,55464		
TC0X00002631.mm.1	1,19	Pola1	JUC0X00008332.mm.1	-2,33	0,002402	0,348564		
TC0X00002631.mm.1	1,19	Pola1	JUC0X00008347.mm.1	-2,96	0,008465	0,406624		
TC1600000703.mm.1	1,55	Gm6931	PSR1600006180.mm.1	-2,08	0,016879	0,460797	Alternative 3' Acce	0,1
TC1400001731.mm.1	-1,02	Opn4	PSR1400013489.mm.1	-2,08	0,00169	0,335395	Cassette Exon	0,09
TC0500000465.mm.1	1,35	Cc2d2a	PSR0500004458.mm.1	-2,08	0,019765	0,474996	Cassette Exon	0,05
TC0500000465.mm.1	1,35	Cc2d2a	JUC0500002515.mm.1	-2,24	0,026275	0,50053		
TC1200000583.mm.1	-1,15	Abhd12b	JUC1200002172.mm.1	-2,08	0,049709	0,563531		
TC1300001882.mm.1	-1,02	Ranbp9	JUC1300006011.mm.1	-2,08	0,048083	0,559824		
TC0100002656.mm.1	-1,21	Dock10	JUC0100012243.mm.1	-2,08	0,006525	0,391502		
TC0200000087.mm.1	-1,16	Sfmbt2	JUC0200000294.mm.1	-2,08	0,018283	0,468049		
TC0500003188.mm.1	1,2	Erp29	JUC0500015458.mm.1	-2,08	0,043895	0,550616		
TC0300003142.mm.1	1,09	Fam73a	JUC0300012942.mm.1	-2,08	0,005169	0,376797		
TC1000002658.mm.1	1,53	Slc17a8	JUC1000011131.mm.1	-2,08	0,004463	0,36753		
TC0400003380.mm.1	1,3	Dph2	JUC0400014143.mm.1	-2,08	0,038877	0,537855		
TC0400002944.mm.1	1,19	Frem1	JUC0400012444.mm.1	-2,08	0,00393	0,359818		
TC0400004041.mm.1	1,04	Rbp7	JUC0400017516.mm.1	-2,08	0,013604	0,443731		
TC0900002656.mm.1	5,78	Unc13c	JUC0900012352.mm.1	-2,08	0,045989	0,555536		
TC1400002759.mm.1	2,29	Nalcn	PSR1400020623.mm.1	-2,09	0,043173	0,548807	Cassette Exon	0,13
TC1400002759.mm.1	2,29	Nalcn	PSR1400020661.mm.1	-2,25	0,028188	0,506368	Cassette Exon	0,19
TC1400002759.mm.1	2,29	Nalcn	PSR1400020630.mm.1	-2,28	0,017102	0,46232	Cassette Exon	0,17
TC1400002759.mm.1	2,29	Nalcn	PSR1400020633.mm.1	-2,43	0,047552	0,558707	Cassette Exon	0,18
TC1400002759.mm.1	2,29	Nalcn	PSR1400020637.mm.1	-2,46	0,016984	0,461441	Cassette Exon	0,21
TC1400002759.mm.1	2,29	Nalcn	PSR1400020612.mm.1	-2,48	0,025728	0,498173	Cassette Exon	0,33
TC1400002759.mm.1	2,29	Nalcn	JUC1400011415.mm.1	-2,67	0,046522	0,556739		
TC1400002759.mm.1	2,29	Nalcn	JUC1400011395.mm.1	-2,71	0,048169	0,56006		
TC1400002759.mm.1	2,29	Nalcn	JUC1400011419.mm.1	-2,75	0,023638	0,491102		

TC1400002759.mm.1	2,29	Nalcn	PSR1400020622.mm.1	-2,78	0,007178	0,397855	Cassette Exon	0,2
TC1400002759.mm.1	2,29	Nalcn	PSR1400020649.mm.1	-2,92	0,004053	0,361816	Cassette Exon	0,2
TC1400002759.mm.1	2,29	Nalcn	PSR1400020624.mm.1	-3,23	0,021842	0,484607	Cassette Exon	0,21
TC1400002759.mm.1	2,29	Nalcn	PSR1400020613.mm.1	-3,4	0,02246	0,487019	Cassette Exon	0,25
TC1400002759.mm.1	2,29	Nalcn	JUC1400011377.mm.1	-3,61	0,046708	0,556856		
TC1400002759.mm.1	2,29	Nalcn	PSR1400020642.mm.1	-3,71	0,013795	0,444974	Alternative 5' Donc	0,42
TC1400002759.mm.1	2,29	Nalcn	PSR1400020640.mm.1	-3,85	0,023562	0,490947	Alternative 3' Acce	0,45
TC1400002759.mm.1	2,29	Nalcn	PSR1400020655.mm.1	-4,12	0,037211	0,533267	Alternative 3' Acce	0,42
TC1400002759.mm.1	2,29	Nalcn	JUC1400011417.mm.1	-4,48	0,012624	0,436032		
TC1400002759.mm.1	2,29	Nalcn	JUC1400011373.mm.1	-5,25	0,032949	0,52122		
TC1000002438.mm.1	2,25	Pfkl	PSR1000017684.mm.1	-2,09	0,00187	0,338121	Alternative 3' Acce	0,06
TC1000002438.mm.1	2,25	Pfkl	PSR1000017717.mm.1	-2,35	0,000158	0,272178	Alternative 5' Donc	0,18
TC1000002438.mm.1	2,25	Pfkl	PSR1000017698.mm.1	-2,63	0,035469	0,528724	Alternative 5' Donc	0,15
TC1000002438.mm.1	2,25	Pfkl	JUC1000009716.mm.1	-3,06	0,009165	0,413447		
TC1000002438.mm.1	2,25	Pfkl	PSR1000017704.mm.1	-3,27	0,001403	0,325997	Alternative 3' Acce	0,34
TC0600002200.mm.1	1,15	Ezh2	JUC0600009084.mm.1	-2,09	0,005446	0,379869		
TC0600002200.mm.1	1,15	Ezh2	PSR0600017337.mm.1	-2,84	0,000181	0,283791	Alternative 5' Donc	0,32
TC0X00003116.mm.1	1,88	Dcx	PSR0X00019599.mm.1	-2,09	0,001589	0,331726	Alternative 5' Donc	0,07
TC0X00003116.mm.1	1,88	Dcx	JUC0X00009921.mm.1	-2,41	0,024229	0,493294		
TC0X00003116.mm.1	1,88	Dcx	PSR0X00019592.mm.1	-2,53	0,041699	0,545356	Cassette Exon	0,01
TC0X00003116.mm.1	1,88	Dcx	PSR0X00019594.mm.1	-2,99	0,001432	0,327493	Cassette Exon	0,32
TC0X00003116.mm.1	1,88	Dcx	JUC0X00009924.mm.1	-3,91	0,005031	0,374382		
TC1300001020.mm.1	2,16	Ssbp2	PSR1300006501.mm.1	-2,09	0,000795	0,310965	Cassette Exon	0,08
TC1300001020.mm.1	2,16	Ssbp2	PSR1300006495.mm.1	-2,2	0,03257	0,520133	Alternative 3' Acce	0,17
TC1300001020.mm.1	2,16	Ssbp2	PSR1300006505.mm.1	-2,46	0,00206	0,341885	Alternative 5' Donc	0,11
TC1300001020.mm.1	2,16	Ssbp2	PSR1300006494.mm.1	-2,5	0,019162	0,472719	Cassette Exon	0,15
TC1300001020.mm.1	2,16	Ssbp2	PSR1300006504.mm.1	-2,78	0,007494	0,400809	Alternative 5' Donc	0,18
TC1300001020.mm.1	2,16	Ssbp2	JUC1300003420.mm.1	-2,92	0,014121	0,447137		
TC1300001020.mm.1	2,16	Ssbp2	PSR1300006514.mm.1	-3,29	0,013576	0,443703	Alternative 3' Acce	0,32
TC0400003349.mm.1	2,44	Mmachc	JUC0400013959.mm.1	-2,09	0,027978	0,505748		
TC0400003349.mm.1	2,44	Mmachc	PSR0400026777.mm.1	-3,65	0,033095	0,521734	Alternative 5' Donc	0,29
TC0500001134.mm.1	1,2	Golga3	JUC0500005290.mm.1	-2,09	0,007642	0,402169		
TC0500001134.mm.1	1,2	Golga3	PSR0500009679.mm.1	-2,16	0,040469	0,542132	Intron Retention	0,28
TC0700001631.mm.1	1,05	Ampd3	PSR0700013513.mm.1	-2,09	0,032116	0,518585	Cassette Exon	0,07
TC0700001631.mm.1	1,05	Ampd3	PSR0700013521.mm.1	-2,72	0,012716	0,43654	Alternative 5' Donc	0,28
TC0700003397.mm.1	1,13	Gm9801	PSR0700029007.mm.1	-2,09	0,005011	0,373851	Intron Retention	0,27
TC0700003397.mm.1	1,13	Gm9801	PSR0700029029.mm.1	-2,82	0,020808	0,479505	Cassette Exon	0,19
TC1000002121.mm.1	2,56	Grik2	PSR1000014993.mm.1	-2,09	0,003602	0,356223	Cassette Exon	0,19
TC1000002121.mm.1	2,56	Grik2	JUC1000008128.mm.1	-2,35	0,001503	0,329896		
TC1000002121.mm.1	2,56	Grik2	PSR1000014989.mm.1	-2,42	0,001453	0,328414	Cassette Exon	0,14
TC1000002121.mm.1	2,56	Grik2	PSR1000014988.mm.1	-2,47	0,026894	0,502518	Cassette Exon	0,12
TC1000002121.mm.1	2,56	Grik2	PSR1000014999.mm.1	-2,69	0,018325	0,468127	Cassette Exon	0,15
TC1000002121.mm.1	2,56	Grik2	JUC1000008133.mm.1	-2,9	0,031637	0,517405		
TC1000002121.mm.1	2,56	Grik2	PSR1000015009.mm.1	-3,8	0,004366	0,365487	Cassette Exon	0,26
TC1000002121.mm.1	2,56	Grik2	PSR1000014985.mm.1	-7,15	0,002791	0,351449	Cassette Exon	0,26
TC0500000015.mm.1	1,39	Pex1	PSR0500000121.mm.1	-2,09	0,03587	0,529376	Cassette Exon	0,09
TC0500000015.mm.1	1,39	Pex1	PSR0500000133.mm.1	-2,15	0,030494	0,513341	Cassette Exon	0,16
TC0500000015.mm.1	1,39	Pex1	PSR0500000132.mm.1	-3,3	0,002869	0,352207	Cassette Exon	0,22

TC1600002136.mm.1	1,44	Brwd1	PSR1600016398.mm.1	-2,09	0,033074	0,521669	Alternative 3' Acce	0,17
TC1600002136.mm.1	1,44	Brwd1	PSR1600016442.mm.1	-2,46	0,015182	0,452699	Alternative 3' Acce	0,22
TC1400002870.mm.1	-1,08	Setdb2; Phf11c; Phf11d	PSR1400016705.mm.1	-2,09	0,00396	0,359922	Cassette Exon	0,21
TC1400002870.mm.1	-1,08	Setdb2; Phf11c; Phf11d	JUC1400009145.mm.1	-2,09	0,024556	0,494116		
TC1400002870.mm.1	-1,08	Setdb2; Phf11c; Phf11d	PSR1400016714.mm.1	-2,1	0,013439	0,442693	Alternative 5' Donc	0,09
TC1400002870.mm.1	-1,08	Setdb2; Phf11c; Phf11d	JUC1400009123.mm.1	-2,88	0,041243	0,544166		
TC0200003540.mm.1	1,02	Stam2; Gm13548	PSR0200030259.mm.1	-2,09	0,032069	0,518469	Alternative 5' Donc	0,18
TC0200000525.mm.1	1,17	Tbc1d13	PSR0200004095.mm.1	-2,09	0,024414	0,493825	Alternative 3' Acce	0,17
TC0200000525.mm.1	1,17	Tbc1d13	JUC0200002062.mm.1	-4,43	0,015941	0,456831		
TC0500002821.mm.1	1,74	Gm16227	PSR0500024774.mm.1	-2,09	0,025914	0,499027	Alternative 3' Acce	0,17
TC1100000170.mm.1	1,04	C1d	PSR1100001543.mm.1	-2,09	0,028061	0,505951	Alternative 5' Donc	0,17
TC1700001390.mm.1	-1,32	Ezr	PSR1700012536.mm.1	-2,09	0,005657	0,381879	Alternative 3' Acce	0,17
TC0200004485.mm.1	1,71	Ppip5k1	PSR0200037973.mm.1	-2,09	0,014357	0,448293	Intron Retention	0,16
TC0200004485.mm.1	1,71	Ppip5k1	JUC0200019556.mm.1	-2,55	0,022079	0,485405		
TC0200004651.mm.1	1,57	Rnf24	PSR0200039986.mm.1	-2,09	0,00614	0,387604	Cassette Exon	0,15
TC0200004651.mm.1	1,57	Rnf24	JUC0200020716.mm.1	-2,76	0,003795	0,357968		
TC0400001302.mm.1	1,27	Gm12841	PSR0400009770.mm.1	-2,09	0,017497	0,463964	Cassette Exon	0,15
TC0400001302.mm.1	1,27	Gm12841	JUC0400005118.mm.1	-2,16	0,006761	0,393343		
TC0X00001271.mm.1	-1,97	Srpx2	PSR0X00007841.mm.1	-2,09	0,017368	0,463612	Alternative 3' Acce	0,15
TC0X00001271.mm.1	-1,97	Srpx2	JUC0X00003850.mm.1	-2,59	0,023721	0,491521		
TC1200002517.mm.1	1,91	Gm5977; Asap2	PSR1200001375.mm.1	-2,09	0,01634	0,458616	Cassette Exon	0,1
TC1200002517.mm.1	1,91	Gm5977; Asap2	PSR1200001379.mm.1	-2,13	0,001571	0,331411	Alternative 3' Acce	0,15
TC1500001278.mm.1	1,29	4930445E18Rik	PSR1500010032.mm.1	-2,09	0,011169	0,426345	Alternative 3' Acce	0,15
TC0100003820.mm.1	1,09	1700034H15Rik	JUC0100017710.mm.1	-2,09	0,018146	0,467399		
TC0100003820.mm.1	1,09	1700034H15Rik	PSR0100031202.mm.1	-2,39	0,014105	0,447123	Alternative 3' Acce	0,14
TC1000002594.mm.1	1,07	Ckap4	PSR1000019580.mm.1	-2,09	0,047405	0,558302	Alternative 3' Acce	0,14
TC0200003815.mm.1	1,96	Zfp385b	JUC0200017016.mm.1	-2,09	0,033947	0,524252		
TC0200003815.mm.1	1,96	Zfp385b	PSR0200033025.mm.1	-2,27	0,044633	0,552129	Cassette Exon	0,1
TC0200003815.mm.1	1,96	Zfp385b	PSR0200033015.mm.1	-2,52	0,011362	0,427452	Cassette Exon	0,13
TC0200003815.mm.1	1,96	Zfp385b	JUC0200017024.mm.1	-3,71	0,003025	0,353502		
TC0200003815.mm.1	1,96	Zfp385b	JUC0200017015.mm.1	-6,26	0,041531	0,544763		
TC1700002308.mm.1	-1,68	Stap2	PSR1700021529.mm.1	-2,09	0,032063	0,518469	Cassette Exon	0,13
TC0200001189.mm.1	1,83	Zfp804a	PSR0200009505.mm.1	-2,09	0,039913	0,54093	Alternative 3' Acce	0,12
TC0200001189.mm.1	1,83	Zfp804a	JUC0200004850.mm.1	-2,46	0,034964	0,52724		
TC0200001189.mm.1	1,83	Zfp804a	JUC0200004852.mm.1	-6,02	0,005572	0,381015		
TC0100000676.mm.1	-1,05		PSR0100005810.mm.1	-2,09	0,020481	0,478018	Cassette Exon	0,1
TC0200002661.mm.1	1,12	Gm14271	PSR0200021659.mm.1	-2,09	0,03638	0,530811	Cassette Exon	0,1
TC0400000662.mm.1	-1,03	Snx30; Gm12543	PSR0400004711.mm.1	-2,09	0,003154	0,353892	Cassette Exon	0,1
TC0400000662.mm.1	-1,03	Snx30; Gm12543	PSR0400004717.mm.1	-2,28	0,019372	0,473624		
TC0400002424.mm.1	1,28	Gm12381	PSR0400019571.mm.1	-2,09	0,033879	0,524139	Cassette Exon	0,1
TC0X00003157.mm.1	-1,35	Gm15104	PSR0X00019775.mm.1	-2,09	0,010577	0,422452	Cassette Exon	0,1
TC1100000268.mm.1	1,47	Gm12070; Gm5523	PSR1100002280.mm.1	-2,09	0,006217	0,387912	Cassette Exon	0,1
TC1500002208.mm.1	1,06	Fam186b	PSR1500018053.mm.1	-2,09	0,00319	0,354243	Cassette Exon	0,1
TC0800002455.mm.1	1,05		PSR0800018782.mm.1	-2,09	0,00647	0,390467	Cassette Exon	0,09
TC1400002179.mm.1	1,2	Gm20017; Gm15919	JUC1400009234.mm.1	-2,09	0,033447	0,522793		
TC1400002179.mm.1	1,2	Gm20017; Gm15919	JUC1400009232.mm.1	-2,63	0,002693	0,349612		
TC0200005346.mm.1	1,28	LOC628147; Gm14295; Gn	JUC0200023443.mm.1	-2,09	0,031717	0,517489		
TC1100004304.mm.1	1,22	Mafg	JUC1100021155.mm.1	-2,09	0,037648	0,534212		

TC0300000956.mm.1	1,36	Pias3	JUC0300003970.mm.1	-2,09	0,044494	0,551741		
TC1300001191.mm.1	1,3	3830408C21Rik	PSR1300007581.mm.1	-2,09	0,013696	0,44468		
TC1300001191.mm.1	1,3	3830408C21Rik	JUC1300003960.mm.1	-3,41	0,041419	0,544646		
TC0100002837.mm.1	1,06	Thap4	JUC0100013091.mm.1	-2,09	0,00408	0,362111		
TC1900001705.mm.1	1,07	Dclre1a	JUC1900008370.mm.1	-2,09	0,022523	0,487133		
TC0200001735.mm.1	-1,08	Eif2ak4	JUC0200006387.mm.1	-2,09	0,016249	0,458312		
TC0200002282.mm.1	-1,11	6820408C15Rik	JUC0200008973.mm.1	-2,09	0,009093	0,412827		
TC1700000522.mm.1	-1,14	Dnah8	JUC1700002587.mm.1	-2,09	0,019534	0,474538		
TC1700000526.mm.1	-1,11	Umodl1	JUC1700002629.mm.1	-2,09	0,012623	0,436032		
TC0200000827.mm.1	-1,25	A430018G15Rik	JUC0200003471.mm.1	-2,09	0,018402	0,468484		
TC0800001572.mm.1	2,21	Kcnk1	JUC0800007041.mm.1	-2,09	0,004622	0,369794		
TC0800001809.mm.1	-1,17	Defa-ps15	JUC0800007861.mm.1	-2,09	0,011045	0,425792		
TC0800003173.mm.1	1,38	Ttc13	JUC0800013326.mm.1	-2,09	0,046079	0,555658		
TC0800003173.mm.1	1,38	Ttc13	JUC0800013313.mm.1	-2,7	0,032578	0,520209		
TC0600001293.mm.1	1,26	Fam21	JUC0600005361.mm.1	-2,09	0,032443	0,519525		
TC0700002717.mm.1	-1,02	Catsperg1	JUC0700012812.mm.1	-2,09	0,045201	0,553548		
TC0700004633.mm.1	-1,01	Gm15448; Pirb; Pira6	JUC0700010290.mm.1	-2,09	0,045253	0,553703		
TC1100000923.mm.1	1,44	Zbtb4	JUC1100004128.mm.1	-2,09	0,012292	0,434277		
TC1100000566.mm.1	1,18	Col23a1	JUC1100002292.mm.1	-2,09	0,017032	0,461729		
TC1000001988.mm.1	-1,14	Zufsp	JUC1000007713.mm.1	-2,09	0,016433	0,459007		
TC0400001239.mm.1	1,05	Mknk1	JUC0400004779.mm.1	-2,09	0,006313	0,389263		
TC0400001239.mm.1	1,05	Mknk1	JUC0400004774.mm.1	-2,24	0,011057	0,425887		
TC1100002065.mm.1	1,34	Fn3k	JUC1100010401.mm.1	-2,09	0,000383	0,293869		
TC0400003536.mm.1	-1,1	Tekt2	JUC0400015022.mm.1	-2,09	0,017718	0,465209		
TC1000003219.mm.1	1,46	BC030307	JUC1000004255.mm.1	-2,09	0,002902	0,352207		
TC1000001166.mm.1	-1,01		JUC1000004744.mm.1	-2,09	0,030586	0,513425		
TC0X00002648.mm.1	1,26	Maged1	JUC0X00008428.mm.1	-2,09	0,010081	0,418998		
TC0300001530.mm.1	2,31	Usp33	PSR0300012351.mm.1	-2,1	0,008989	0,41198	Intron Retention	0,27
TC0300001530.mm.1	2,31	Usp33	PSR0300012346.mm.1	-2,22	0,010905	0,424472	Cassette Exon	0,14
TC0300001530.mm.1	2,31	Usp33	PSR0300012342.mm.1	-2,26	0,022736	0,487845	Cassette Exon	0,18
TC0300001530.mm.1	2,31	Usp33	JUC0300006527.mm.1	-2,52	0,003355	0,354243		
TC0300001530.mm.1	2,31	Usp33	PSR0300012377.mm.1	-2,59	0,011641	0,429841	Alternative 5' Donc	0,24
TC0300001530.mm.1	2,31	Usp33	PSR0300012340.mm.1	-3,97	0,00984	0,417839	Alternative 5' Donc	0,47
TC0500002865.mm.1	7,93	Mapk10	PSR0500025332.mm.1	-2,1	0,002852	0,352207	Cassette Exon	0,1
TC0500002865.mm.1	7,93	Mapk10	JUC0500013777.mm.1	-2,11	0,008591	0,408237		
TC0500002865.mm.1	7,93	Mapk10	PSR0500025345.mm.1	-2,8	0,024868	0,49558	Alternative 3' Acce	0,28
TC0500002865.mm.1	7,93	Mapk10	JUC0500013783.mm.1	-2,81	0,000962	0,313363		
TC0500002865.mm.1	7,93	Mapk10	PSR0500025359.mm.1	-2,93	0,003523	0,355752	Alternative 5' Donc	0,39
TC0500002865.mm.1	7,93	Mapk10	PSR0500025335.mm.1	-2,96	0,011651	0,429885	Cassette Exon	0,15
TC0500002865.mm.1	7,93	Mapk10	PSR0500025320.mm.1	-3,72	0,037796	0,534693	Alternative 3' Acce	0,39
TC0500002865.mm.1	7,93	Mapk10	PSR0500025323.mm.1	-3,98	0,005026	0,374176	Alternative 3' Acce	0,39
TC0500002865.mm.1	7,93	Mapk10	PSR0500025318.mm.1	-4,24	0,027943	0,505612	Alternative 3' Acce	0,39
TC0500002865.mm.1	7,93	Mapk10	PSR0500025338.mm.1	-4,38	0,027532	0,504339	Alternative 3' Acce	0,47
TC0500002865.mm.1	7,93	Mapk10	PSR0500025322.mm.1	-5,02	0,007208	0,398121	Alternative 3' Acce	0,39
TC0500002865.mm.1	7,93	Mapk10	PSR0500025341.mm.1	-5,05	0,001584	0,331726	Cassette Exon	0,39
TC0500002865.mm.1	7,93	Mapk10	PSR0500025321.mm.1	-5,4	0,012109	0,43269	Alternative 3' Acce	0,39
TC0500002865.mm.1	7,93	Mapk10	PSR0500025357.mm.1	-6,76	0,015979	0,456905	Cassette Exon	0,29
TC0500002865.mm.1	7,93	Mapk10	PSR0500025337.mm.1	-7,78	0,009677	0,417605	Alternative 3' Acce	0,47

TC0500002865.mm.1	7,93	Mapk10	JUC0500013791.mm.1	-8,31	0,009715	0,417826		
TC0500002865.mm.1	7,93	Mapk10	PSR0500025343.mm.1	-10,31	0,00815	0,405473	Cassette Exon	0,16
TC0500002865.mm.1	7,93	Mapk10	PSR0500025355.mm.1	-11,37	0,00499	0,373851		
TC0500002865.mm.1	7,93	Mapk10	PSR0500025349.mm.1	-12,24	0,009164	0,413447	Cassette Exon	0,25
TC0500002865.mm.1	7,93	Mapk10	PSR0500025354.mm.1	-13,57	0,010739	0,423772		
TC0500002865.mm.1	7,93	Mapk10	PSR0500025348.mm.1	-14,06	0,003196	0,354243	Cassette Exon	0,38
TC0500002865.mm.1	7,93	Mapk10	PSR0500025330.mm.1	-16,18	0,00278	0,351017	Cassette Exon	0,27
TC0500002865.mm.1	7,93	Mapk10	PSR0500025324.mm.1	-16,82	0,009454	0,416478	Alternative 3' Acce	0,39
TC0500002865.mm.1	7,93	Mapk10	PSR0500025363.mm.1	-18,95	0,005576	0,381035	Cassette Exon	0,41
TC0500002865.mm.1	7,93	Mapk10	JUC0500013797.mm.1	-25,15	0,007529	0,40125		
TC0500002865.mm.1	7,93	Mapk10	PSR0500025319.mm.1	-38,1	0,002869	0,352207	Alternative 3' Acce	0,39
TC0X00001333.mm.1	1,68	Tceal3; LOC331528	PSR0X00008379.mm.1	-2,1	0,00298	0,352501	Cassette Exon	0,18
TC0X00001333.mm.1	1,68	Tceal3; LOC331528	PSR0X00008371.mm.1	-2,12	0,009422	0,415976	Cassette Exon	0,03
TC0X00001333.mm.1	1,68	Tceal3; LOC331528	JUC0X00004106.mm.1	-2,15	0,025629	0,497926		
TC0X00001333.mm.1	1,68	Tceal3; LOC331528	JUC0X00004117.mm.1	-2,38	0,037521	0,533968		
TC0X00001333.mm.1	1,68	Tceal3; LOC331528	JUC0X00004107.mm.1	-2,39	0,001261	0,322251		
TC0X00001333.mm.1	1,68	Tceal3; LOC331528	PSR0X00008380.mm.1	-2,43	0,036023	0,529546	Alternative 3' Acce	0,17
TC0X00001333.mm.1	1,68	Tceal3; LOC331528	PSR0X00008375.mm.1	-2,61	0,026462	0,501252	Cassette Exon	0,07
TC0X00001333.mm.1	1,68	Tceal3; LOC331528	PSR0X00008368.mm.1	-3,86	0,001753	0,335996	Alternative 3' Acce	0,3
TC1300001911.mm.1	2,06	Atxn1	PSR1300011847.mm.1	-2,1	0,035014	0,527423	Alternative 5' Donc	0,3
TC1300001911.mm.1	2,06	Atxn1	PSR1300011848.mm.1	-2,96	0,021451	0,482293	Alternative 5' Donc	0,3
TC0600001907.mm.1	2,47	Cttnbp2	PSR0600014959.mm.1	-2,1	0,047407	0,558302	Alternative 3' Acce	0,21
TC0600001907.mm.1	2,47	Cttnbp2	PSR0600014970.mm.1	-2,15	0,014512	0,449545	Cassette Exon	0,14
TC0600001907.mm.1	2,47	Cttnbp2	PSR0600014966.mm.1	-2,31	0,012933	0,438537	Alternative 3' Acce	0,12
TC0600001907.mm.1	2,47	Cttnbp2	PSR0600014969.mm.1	-2,53	0,015818	0,45594	Alternative 3' Acce	0,06
TC0600001907.mm.1	2,47	Cttnbp2	PSR0600014961.mm.1	-2,72	0,000765	0,308403	Alternative 3' Acce	0,24
TC0600001907.mm.1	2,47	Cttnbp2	PSR0600014932.mm.1	-2,84	0,026195	0,50003	Alternative 3' Acce	0,29
TC0600001907.mm.1	2,47	Cttnbp2	PSR0600014958.mm.1	-2,96	0,034084	0,524705	Cassette Exon	0,25
TC0600001907.mm.1	2,47	Cttnbp2	JUC0600007819.mm.1	-3,19	0,013352	0,442045		
TC0600001907.mm.1	2,47	Cttnbp2	PSR0600014972.mm.1	-3,27	0,00359	0,356223	Alternative 5' Donc	0,18
TC0600001907.mm.1	2,47	Cttnbp2	JUC0600007831.mm.1	-3,56	0,005558	0,380773		
TC0600001907.mm.1	2,47	Cttnbp2	PSR0600014960.mm.1	-3,7	0,003889	0,359225	Alternative 3' Acce	0,24
TC0500002315.mm.1	2,07	Fbxl5	PSR0500021290.mm.1	-2,1	0,039417	0,539608	Alternative 3' Acce	0,13
TC0500002315.mm.1	2,07	Fbxl5	PSR0500021301.mm.1	-2,63	0,011791	0,430163	Alternative 3' Acce	0,26
TC1100003687.mm.1	1,61	Med24; Gm22059	PSR1100033963.mm.1	-2,1	0,040451	0,542094	Alternative 3' Acce	0,25
TC1100003687.mm.1	1,61	Med24; Gm22059	PSR1100033945.mm.1	-2,15	0,027471	0,504083	Alternative 5' Donc	0,15
TC1100003687.mm.1	1,61	Med24; Gm22059	PSR1100033922.mm.1	-2,18	0,015104	0,452457	Alternative 3' Acce	0,12
TC1100003687.mm.1	1,61	Med24; Gm22059	JUC1100017786.mm.1	-2,53	0,028057	0,505951		
TC1100003687.mm.1	1,61	Med24; Gm22059	JUC1100017790.mm.1	-2,87	0,035546	0,528764		
TC1100003687.mm.1	1,61	Med24; Gm22059	JUC1100017791.mm.1	-4,54	0,030095	0,511942		
TC0100003352.mm.1	1,95	Rabgap1l	JUC0100015740.mm.1	-2,1	0,025375	0,497403		
TC0100003352.mm.1	1,95	Rabgap1l	PSR0100027464.mm.1	-2,12	0,002958	0,352501	Alternative 3' Acce	0,22
TC0100003352.mm.1	1,95	Rabgap1l	PSR0100027463.mm.1	-2,64	0,006869	0,394155	Alternative 3' Acce	0,22
TC0100003352.mm.1	1,95	Rabgap1l	PSR0100027455.mm.1	-3,11	0,004731	0,370888	Alternative 3' Acce	0,18
TC1300000270.mm.1	2,91		JUC1300000770.mm.1	-2,1	0,026835	0,502429		
TC1300000270.mm.1	2,91		JUC1300000771.mm.1	-2,12	0,003611	0,356223		
TC1300000270.mm.1	2,91		PSR1300001558.mm.1	-2,34	0,000484	0,298999	Cassette Exon	0,17
TC1300000270.mm.1	2,91		PSR1300001559.mm.1	-3,42	0,000064	0,250119	Cassette Exon	0,2

TC1100003059.mm.1	2,71	Tnfsf12Tnfsf13; Tnfsf13; Tr	PSR1100028041.mm.1	-2,1	0,020064	0,476524	Cassette Exon	0,19
TC1100003059.mm.1	2,71	Tnfsf12Tnfsf13; Tnfsf13; Tr	PSR1100028044.mm.1	-2,15	0,019618	0,4748	Cassette Exon	0,17
TC1100003059.mm.1	2,71	Tnfsf12Tnfsf13; Tnfsf13; Tr	JUC1100014695.mm.1	-2,43	0,030518	0,51337		
TC1100003059.mm.1	2,71	Tnfsf12Tnfsf13; Tnfsf13; Tr	PSR1100028048.mm.1	-2,53	0,017278	0,463088	Alternative 5' Donc	0,11
TC1100003059.mm.1	2,71	Tnfsf12Tnfsf13; Tnfsf13; Tr	PSR1100028043.mm.1	-3	0,036857	0,532257	Cassette Exon	0,19
TC0300002601.mm.1	1,19		PSR0300020286.mm.1	-2,1	0,000079	0,25272	Alternative 3' Acce	0,18
TC0700004547.mm.1	-1,01	Muc6	PSR0700038335.mm.1	-2,1	0,040471	0,542132	Cassette Exon	0,18
TC0300000580.mm.1	1,56	Ppm1l	PSR0300003915.mm.1	-2,1	0,015033	0,452111	Alternative 3' Acce	0,17
TC0400002517.mm.1	-1,13	Fam214b	PSR0400020421.mm.1	-2,1	0,035527	0,528764	Cassette Exon	0,17
TC1100001469.mm.1	1,99	Phospho1; Zfp652	PSR1100013674.mm.1	-2,1	0,035995	0,529506	Alternative 3' Acce	0,17
TC1400000306.mm.1	1,04	Tkt; Mir3076	PSR1400002484.mm.1	-2,1	0,044828	0,552553	Alternative 5' Donc	0,17
TC1700000839.mm.1	1,47	Rcan2	PSR1700008067.mm.1	-2,1	0,013178	0,44063	Cassette Exon	0,17
TC0200005501.mm.1	1,64	Cdan1	PSR0200037590.mm.1	-2,1	0,014735	0,450565	Alternative 3' Acce	0,16
TC0200005501.mm.1	1,64	Cdan1	PSR0200037587.mm.1	-2,11	0,005417	0,379167	Alternative 3' Acce	0,13
TC0200005501.mm.1	1,64	Cdan1	PSR0200037609.mm.1	-2,32	0,025297	0,497227	Cassette Exon	0,14
TC0200005501.mm.1	1,64	Cdan1	JUC0200019320.mm.1	-2,99	0,021074	0,480364		
TC1500001466.mm.1	1,64	Sybu	PSR1500011206.mm.1	-2,1	0,016824	0,460623	Cassette Exon	0,11
TC1500001466.mm.1	1,64	Sybu	PSR1500011195.mm.1	-2,11	0,021096	0,480377	Cassette Exon	0,04
TC1500001466.mm.1	1,64	Sybu	PSR1500011200.mm.1	-2,28	0,0274	0,503961	Alternative 3' Acce	0,02
TC1500001466.mm.1	1,64	Sybu	PSR1500011190.mm.1	-2,67	0,020231	0,477145	Cassette Exon	0,16
TC1500001466.mm.1	1,64	Sybu	JUC1500006297.mm.1	-3,03	0,001691	0,335395		
TC1700001262.mm.1	-1,13	Plekhh2	PSR1700011646.mm.1	-2,1	0,023178	0,489982	Alternative 3' Acce	0,15
TC0400003726.mm.1	1,52	Cep85	PSR0400030625.mm.1	-2,1	0,015469	0,454316	Cassette Exon	0,12
TC0400003726.mm.1	1,52	Cep85	PSR0400030641.mm.1	-2,68	0,002619	0,349612	Cassette Exon	0,14
TC0400003726.mm.1	1,52	Cep85	JUC0400015918.mm.1	-2,76	0,048485	0,560864		
TC0400003726.mm.1	1,52	Cep85	JUC0400015915.mm.1	-3,43	0,027941	0,505612		
TC0600002911.mm.1	1,33	Gt(ROSA)26Sor	PSR0600022320.mm.1	-2,1	0,005613	0,381163	Alternative 3' Acce	0,14
TC1700001472.mm.1	1,36		JUC1700007177.mm.1	-2,1	0,0204	0,477881		
TC1700001472.mm.1	1,36		PSR1700013138.mm.1	-2,53	0,01158	0,429186	Cassette Exon	0,14
TC0400001930.mm.1	1,46	Mtor	PSR0400015754.mm.1	-2,1	0,027158	0,503174	Cassette Exon	0,12
TC0600003456.mm.1	1,9	Gm16583	JUC0600014305.mm.1	-2,1	0,016817	0,460623		
TC0600003456.mm.1	1,9	Gm16583	PSR0600027261.mm.1	-2,34	0,017926	0,466466	Cassette Exon	0,12
TC0900000862.mm.1	1,3	Ppib	PSR0900006616.mm.1	-2,1	0,00377	0,357586	Cassette Exon	0,12
TC0100002278.mm.1	-1,05	Col5a2	PSR0100018357.mm.1	-2,1	0,04747	0,558545	Cassette Exon	0,1
TC0100003412.mm.1	1,09	Gorab	PSR0100028002.mm.1	-2,1	0,015372	0,453849	Cassette Exon	0,1
TC0200005502.mm.1	1,77	Ttbk2	PSR0200037677.mm.1	-2,1	0,005374	0,378731	Cassette Exon	0,1
TC1300002231.mm.1	1,08		PSR1300014443.mm.1	-2,1	0,000322	0,288663	Cassette Exon	0,1
TC0100001855.mm.1	1,76	Lpgat1	PSR0100015350.mm.1	-2,1	0,049765	0,563626	Cassette Exon	0,09
TC0200004878.mm.1	1,83	Snph	PSR0200041438.mm.1	-2,1	0,014384	0,44839	Cassette Exon	0,08
TC0200004878.mm.1	1,83	Snph	JUC0200021433.mm.1	-2,15	0,037434	0,533831		
TC0200004878.mm.1	1,83	Snph	JUC0200021429.mm.1	-2,36	0,014602	0,449914		
TC0200004878.mm.1	1,83	Snph	JUC0200021431.mm.1	-2,65	0,013962	0,446239		
TC0200004878.mm.1	1,83	Snph	JUC0200021432.mm.1	-2,66	0,012093	0,43255		
TC1400001405.mm.1	-1,03	Gm3453	JUC1400005637.mm.1	-2,1	0,034054	0,524583		
TC0200003482.mm.1	-1,04		JUC0200015032.mm.1	-2,1	0,0367	0,531756		
TC1400000662.mm.1	1,11	Trav11n	JUC1400002912.mm.1	-2,1	0,007635	0,402113		
TC1200000654.mm.1	-1,05	Gm5068	JUC1200002532.mm.1	-2,1	0,045761	0,554863		
TC1100004122.mm.1	-1,11	Rhbdf2	JUC1100020492.mm.1	-2,1	0,012325	0,434392		

TC1300001707.mm.1	-1,02	2610307P16Rik	JUC1300005434.mm.1	-2,1	0,038516	0,536564		
TC1300000037.mm.1	1,21	Adarb2	JUC1300000157.mm.1	-2,1	0,00284	0,352207		
TC1300000857.mm.1	-1,1	Med10	JUC1300002900.mm.1	-2,1	0,033153	0,521956		
TC1700002813.mm.1	1,18	Ppt2	JUC1700009599.mm.1	-2,1	0,013728	0,444758		
TC1700001686.mm.1	1	Rab11fip3	JUC1700008502.mm.1	-2,1	0,028911	0,50858		
TC1500001684.mm.1	1,64	Trappc9	JUC1500007195.mm.1	-2,1	0,029018	0,508842		
TC1500000418.mm.1	-1,09	Fer1l6	JUC1500001728.mm.1	-2,1	0,016129	0,457772		
TC0200000245.mm.1	-1,43	Mrc1	JUC0200000449.mm.1	-2,1	0,015506	0,454456		
TC0500002078.mm.1	1,01	Fastk; Gm22333	JUC0500010316.mm.1	-2,1	0,003609	0,356223		
TC0800001426.mm.1	-1,29	Atp2c2	JUC0800006380.mm.1	-2,1	0,043338	0,549411		
TC0800001077.mm.1	-1,03	Mmp2	JUC0800004413.mm.1	-2,1	0,00862	0,408434		
TC0500000682.mm.1	-1,05		JUC0500003307.mm.1	-2,1	0,024478	0,493992		
TC0500001483.mm.1	1,05	Tmem132c	JUC0500007336.mm.1	-2,1	0,043641	0,550199		
TC0500001483.mm.1	1,05	Tmem132c	JUC0500007339.mm.1	-3,63	0,041185	0,544121		
TC0800002694.mm.1	-1,32	Crnde; 4933436C20Rik	JUC0800011184.mm.1	-2,1	0,012215	0,433321		
TC0600001657.mm.1	1,01	Aebp2	JUC0600006951.mm.1	-2,1	0,042814	0,548097		
TC0500003606.mm.1	1,03	B230303O12Rik	JUC0500018019.mm.1	-2,1	0,049445	0,563154		
TC0500003612.mm.1	-1,01		JUC0500018039.mm.1	-2,1	0,027701	0,504872		
TC0400000284.mm.1	1,06	Rngtt	JUC0400000931.mm.1	-2,1	0,024764	0,495014		
TC1000003103.mm.1	1,68	Arhgef25	JUC1000012694.mm.1	-2,1	0,005193	0,376901		
TC0300002256.mm.1	1,31	Gm15535	JUC0300008933.mm.1	-2,1	0,043544	0,549995		
TC0300002357.mm.1	1	Il6ra	JUC0300009514.mm.1	-2,1	0,009516	0,416602		
TC0X00000706.mm.1	1,07	Taz	JUC0X00002386.mm.1	-2,1	0,030313	0,51274		
TC0X00002428.mm.1	-1,09	Haus7	JUC0X00007651.mm.1	-2,1	0,022676	0,487637		
TC0X00001934.mm.1	1,09	Cfp	JUC0X00006204.mm.1	-2,1	0,03986	0,54093		
TC1000002350.mm.1	12,76	Phyhipl; D630013N20Rik	PSR1000016689.mm.1	-2,11	0,019053	0,471978	Alternative 3' Acce	0,19
TC1000002350.mm.1	12,76	Phyhipl; D630013N20Rik	PSR1000016703.mm.1	-3,54	0,003513	0,355749	Cassette Exon	0,05
TC1000002350.mm.1	12,76	Phyhipl; D630013N20Rik	PSR1000016702.mm.1	-5,02	0,033548	0,523131	Cassette Exon	0,26
TC1000002350.mm.1	12,76	Phyhipl; D630013N20Rik	PSR1000016710.mm.1	-5,81	0,007339	0,399128	Alternative 3' Acce	0,3
TC1000002350.mm.1	12,76	Phyhipl; D630013N20Rik	PSR1000016696.mm.1	-6,41	0,042472	0,547627	Intron Retention	0,75
TC1000002350.mm.1	12,76	Phyhipl; D630013N20Rik	PSR1000016718.mm.1	-6,49	0,007452	0,400326	Cassette Exon	0,21
TC1000002350.mm.1	12,76	Phyhipl; D630013N20Rik	PSR1000016694.mm.1	-6,75	0,009697	0,417696	Alternative 3' Acce	0,51
TC1000002350.mm.1	12,76	Phyhipl; D630013N20Rik	PSR1000016700.mm.1	-7	0,010466	0,421696	Cassette Exon	0,26
TC1000002350.mm.1	12,76	Phyhipl; D630013N20Rik	JUC1000009134.mm.1	-7,03	0,02664	0,50179		
TC1000002350.mm.1	12,76	Phyhipl; D630013N20Rik	PSR1000016719.mm.1	-7,19	0,013396	0,442454	Cassette Exon	0,21
TC1000002350.mm.1	12,76	Phyhipl; D630013N20Rik	JUC1000009127.mm.1	-8,15	0,010787	0,424006		
TC1000002350.mm.1	12,76	Phyhipl; D630013N20Rik	PSR1000016692.mm.1	-8,98	0,002046	0,341377	Alternative 3' Acce	0,48
TC1000002350.mm.1	12,76	Phyhipl; D630013N20Rik	PSR1000016699.mm.1	-9,71	0,001486	0,329081	Cassette Exon	0,26
TC1000002350.mm.1	12,76	Phyhipl; D630013N20Rik	PSR1000016716.mm.1	-10,25	0,013336	0,441906	Alternative 3' Acce	0,16
TC1000002350.mm.1	12,76	Phyhipl; D630013N20Rik	PSR1000016704.mm.1	-10,38	0,003439	0,3554	Alternative 5' Donc	0,07
TC1000002350.mm.1	12,76	Phyhipl; D630013N20Rik	JUC1000009123.mm.1	-11,11	0,010885	0,424247		
TC1000002350.mm.1	12,76	Phyhipl; D630013N20Rik	JUC1000009133.mm.1	-11,3	0,029466	0,510123		
TC1000002350.mm.1	12,76	Phyhipl; D630013N20Rik	PSR1000016721.mm.1	-13,5	0,003566	0,356223	Cassette Exon	0,21
TC1000002350.mm.1	12,76	Phyhipl; D630013N20Rik	JUC1000009122.mm.1	-14,63	0,01653	0,459611		
TC1000002350.mm.1	12,76	Phyhipl; D630013N20Rik	PSR1000016722.mm.1	-16,53	0,003864	0,358861	Cassette Exon	0,41
TC1000002350.mm.1	12,76	Phyhipl; D630013N20Rik	JUC1000009136.mm.1	-17,16	0,012732	0,436677		
TC1000002350.mm.1	12,76	Phyhipl; D630013N20Rik	PSR1000016713.mm.1	-31,39	0,006666	0,393187	Cassette Exon	0,07
TC1000002350.mm.1	12,76	Phyhipl; D630013N20Rik	PSR1000016707.mm.1	-32,34	0,011141	0,426226	Alternative 3' Acce	0,16

TC1000002350.mm.1	12,76	Phyhipl; D630013N20Rik	JUC1000009128.mm.1	-59,64	0,003744	0,357586		
TC0100000828.mm.1	11,19	Sag	JUC0100004018.mm.1	-2,11	0,029494	0,510123		
TC0100000828.mm.1	11,19	Sag	JUC0100004013.mm.1	-2,2	0,03969	0,540368		
TC0100000828.mm.1	11,19	Sag	PSR0100007058.mm.1	-2,35	0,015626	0,45509	Alternative 5' Donc	0,2
TC0100000828.mm.1	11,19	Sag	PSR0100007075.mm.1	-2,53	0,021402	0,48207	Alternative 5' Donc	0,19
TC0100000828.mm.1	11,19	Sag	PSR0100007047.mm.1	-3	0,022394	0,486603	Cassette Exon	0,32
TC0100000828.mm.1	11,19	Sag	PSR0100007059.mm.1	-4,92	0,019438	0,474053	Alternative 3' Acce	0,42
TC0100000828.mm.1	11,19	Sag	JUC0100004026.mm.1	-60,52	0,002733	0,349612		
TC1000002870.mm.1	3,02	Zdhhc17	PSR1000021695.mm.1	-2,11	0,026178	0,50003	Cassette Exon	0,07
TC1000002870.mm.1	3,02	Zdhhc17	PSR1000021707.mm.1	-2,97	0,00196	0,340561	Cassette Exon	0,21
TC1000002870.mm.1	3,02	Zdhhc17	PSR1000021704.mm.1	-3	0,015148	0,452699	Alternative 5' Donc	0,31
TC0200000608.mm.1	-1,09	C130021I20Rik	PSR0200005586.mm.1	-2,11	0,025377	0,497403	Intron Retention	0,28
TC1900001454.mm.1	1,63	Ide	PSR1900012678.mm.1	-2,11	0,024367	0,49362	Alternative 3' Acce	0,13
TC1900001454.mm.1	1,63	Ide	JUC1900006933.mm.1	-2,67	0,017602	0,464312		
TC1900001454.mm.1	1,63	Ide	PSR1900012672.mm.1	-2,69	0,016658	0,460022	Cassette Exon	0,24
TC0100000082.mm.1	2,44	Kcnb2	JUC0100000366.mm.1	-2,11	0,044472	0,551693		
TC0100000082.mm.1	2,44	Kcnb2	PSR0100000667.mm.1	-2,3	0,033089	0,521723	Alternative 5' Donc	0,17
TC0100000082.mm.1	2,44	Kcnb2	PSR0100000659.mm.1	-2,44	0,0095	0,416578	Alternative 3' Acce	0,23
TC0700003648.mm.1	-1,24	Cemip; 9930013L23Rik	JUC0700016364.mm.1	-2,11	0,016026	0,45713		
TC0700003648.mm.1	-1,24	Cemip; 9930013L23Rik	PSR0700031010.mm.1	-2,85	0,01799	0,466929	Cassette Exon	0,23
TC0500001487.mm.1	1,36	Glt1d1	PSR0500013389.mm.1	-2,11	0,041236	0,544134	Cassette Exon	0,15
TC0500001487.mm.1	1,36	Glt1d1	PSR0500013386.mm.1	-2,29	0,005849	0,383669	Alternative 3' Acce	0,21
TC0500001487.mm.1	1,36	Glt1d1	JUC0500007349.mm.1	-2,73	0,045857	0,555043		
TC1500002076.mm.1	1,98	Slc2a13	PSR1500016788.mm.1	-2,11	0,021838	0,484607	Cassette Exon	0,21
TC1500002076.mm.1	1,98	Slc2a13	JUC1500009556.mm.1	-2,52	0,010368	0,421259		
TC1400002621.mm.1	-1,04	Tbc1d4	PSR1400019565.mm.1	-2,11	0,002715	0,349612	Alternative 3' Acce	0,18
TC0500002074.mm.1	1,34	Gm15587	PSR0500018845.mm.1	-2,11	0,021388	0,482022	Alternative 5' Donc	0,17
TC1700000461.mm.1	1,43	Hmga1; Hmga1-rs1	PSR1700003875.mm.1	-2,11	0,00588	0,383835	Alternative 5' Donc	0,16
TC1700000461.mm.1	1,43	Hmga1; Hmga1-rs1	PSR1700003874.mm.1	-2,17	0,039558	0,539764	Alternative 5' Donc	0,17
TC0100001871.mm.1	-1,28	Lamb3; 1600010F14Rik	JUC0100008847.mm.1	-2,11	0,010795	0,424049		
TC0100001871.mm.1	-1,28	Lamb3; 1600010F14Rik	PSR0100015564.mm.1	-2,22	0,012293	0,434277	Cassette Exon	0,16
TC0600001094.mm.1	1,11	4930590J08Rik	JUC0600004384.mm.1	-2,11	0,004424	0,366832		
TC0600001094.mm.1	1,11	4930590J08Rik	PSR0600008609.mm.1	-2,18	0,008462	0,406624	Cassette Exon	0,16
TC1700000471.mm.1	-1,04	Scube3	JUC1700002192.mm.1	-2,11	0,048185	0,560088		
TC1700000471.mm.1	-1,04	Scube3	PSR1700004002.mm.1	-2,18	0,03322	0,522175	Alternative 3' Acce	0,16
TC1900000156.mm.1	1,58	Nxf1; Tmem223	PSR1900002073.mm.1	-2,11	0,006524	0,391502	Alternative 3' Acce	0,16
TC1900000156.mm.1	1,58	Nxf1; Tmem223	PSR1900002088.mm.1	-2,13	0,029416	0,509995	Cassette Exon	0,14
TC1400002141.mm.1	-1,05	Gjb6	PSR1400016469.mm.1	-2,11	0,022	0,485226	Alternative 3' Acce	0,15
TC1700000290.mm.1	1,36	Zfp758	JUC1700001158.mm.1	-2,11	0,036023	0,529546		
TC1700000290.mm.1	1,36	Zfp758	PSR1700002147.mm.1	-2,34	0,033657	0,523503	Cassette Exon	0,15
TC1700002185.mm.1	1,75	Klhdc3	PSR1700020614.mm.1	-2,11	0,046349	0,556272	Cassette Exon	0,15
TC1700002185.mm.1	1,75	Klhdc3	JUC1700010985.mm.1	-2,14	0,027412	0,503961		
TC1100000545.mm.1	1,6	Gm12197	PSR1100004232.mm.1	-2,11	0,027828	0,505498	Alternative 5' Donc	0,15
TC1100000545.mm.1	1,6	Gm12197	PSR1100004230.mm.1	-2,16	0,021986	0,485125	Alternative 3' Acce	0,15
TC0300000892.mm.1	1,06	Sema6c	PSR0300006935.mm.1	-2,11	0,024941	0,495859	Cassette Exon	0,13
TC1100001523.mm.1	1,37	D030028A08Rik	PSR1100014006.mm.1	-2,11	0,031632	0,517405	Cassette Exon	0,13
TC1100001523.mm.1	1,37	D030028A08Rik	PSR1100013995.mm.1	-2,36	0,000725	0,306408	Cassette Exon	0,12
TC1100001523.mm.1	1,37	D030028A08Rik	JUC1100007291.mm.1	-2,36	0,030468	0,513244		

TC1100001523.mm.1	1,37	D030028A08Rik	JUC1100007289.mm.1	-2,43	0,009507	0,416602		
TC1100001523.mm.1	1,37	D030028A08Rik	JUC1100007288.mm.1	-2,9	0,003167	0,353901		
TC1200002237.mm.1	1,27	Ccdc88c	PSR1200015395.mm.1	-2,11	0,005151	0,37646	Cassette Exon	0,08
TC1200002237.mm.1	1,27	Ccdc88c	PSR1200015422.mm.1	-2,13	0,001755	0,335996	Cassette Exon	0,13
TC1200002237.mm.1	1,27	Ccdc88c	PSR1200015402.mm.1	-2,15	0,003748	0,357586	Cassette Exon	0,11
TC1300000625.mm.1	2,01	Cplx2	PSR1300003981.mm.1	-2,11	0,010584	0,42255	Cassette Exon	0,1
TC0300001727.mm.1	1,27	Nlgn1	PSR0300013570.mm.1	-2,11	0,004618	0,369794	Cassette Exon	0,07
TC0300001727.mm.1	1,27	Nlgn1	PSR0300013561.mm.1	-2,11	0,000935	0,313363	Alternative 3' Acce	0,06
TC0300001727.mm.1	1,27	Nlgn1	JUC0300007162.mm.1	-3,63	0,015859	0,456449		
TC0200002474.mm.1	-1,14	Srsf6	JUC0200010218.mm.1	-2,11	0,048598	0,561095		
TC1400001235.mm.1	1,24	Slitrk5	JUC1400005087.mm.1	-2,11	0,048528	0,560991		
TC1200000608.mm.1	-1,41	Dact1	JUC1200002338.mm.1	-2,11	0,034833	0,526823		
TC0300000767.mm.1	-1,3	Kcnn3	JUC0300003089.mm.1	-2,11	0,047187	0,558068		
TC1300002420.mm.1	1,42	Msh3	PSR1300016106.mm.1	-2,11	0,004763	0,371039		
TC1300002420.mm.1	1,42	Msh3	JUC1300008486.mm.1	-2,29	0,00884	0,410293		
TC1300002420.mm.1	1,42	Msh3	JUC1300008499.mm.1	-2,39	0,027461	0,504045		
TC1300002420.mm.1	1,42	Msh3	JUC1300008501.mm.1	-3,77	0,047533	0,558701		
TC1700002329.mm.1	-1,08		JUC1700011625.mm.1	-2,11	0,039296	0,539125		
TC1800000581.mm.1	1,06	1700011I03Rik	JUC1800002318.mm.1	-2,11	0,003262	0,354243		
TC1700001597.mm.1	1,2	Thoc6	JUC1700007578.mm.1	-2,11	0,006022	0,386586		
TC1700001656.mm.1	1,02	Baiap3	JUC1700008171.mm.1	-2,11	0,036667	0,531675		
TC0200002349.mm.1	1,04	Rpl5-ps2; Gm14217	JUC0200009460.mm.1	-2,11	0,00928	0,414799		
TC0800000153.mm.1	-1,21	Myom2	JUC0800000824.mm.1	-2,11	0,031636	0,517405		
TC0500000985.mm.1	-1,28	Mrps18c	JUC0500004555.mm.1	-2,11	0,016972	0,461419		
TC0700000831.mm.1	-1,31	Pih1d1	JUC0700003506.mm.1	-2,11	0,039436	0,539642		
TC0700000831.mm.1	-1,31	Pih1d1	JUC0700003505.mm.1	-2,35	0,015777	0,455583		
TC0700000010.mm.1	1,35	Cacng6	JUC0700000044.mm.1	-2,11	0,037412	0,533831		
TC0300002833.mm.1	1,2	6530403H02Rik	JUC0300011680.mm.1	-2,11	0,013721	0,444758		
TC0300002833.mm.1	1,2	6530403H02Rik	JUC0300011685.mm.1	-2,51	0,04489	0,55271		
TC1000002639.mm.1	-1,14	Parpbp	JUC1000010919.mm.1	-2,11	0,007888	0,403771		
TC1100002569.mm.1	1,28	Gm12150	JUC1100012358.mm.1	-2,11	0,027364	0,503891		
TC1100003374.mm.1	1,01	Nle1	JUC1100016185.mm.1	-2,11	0,017177	0,46232		
TC0300002668.mm.1	-1,03	Kcnd3os; 1700095B22Rik	JUC0300010813.mm.1	-2,11	0,022694	0,487767		
TC1100001459.mm.1	-1,07	Fam117a	JUC1100007104.mm.1	-2,11	0,047063	0,557783		
TC0300002188.mm.1	1,17	Rapgef2	JUC0300008628.mm.1	-2,11	0,034433	0,525858		
TC0300002370.mm.1	-1,05	Ints3	JUC0300009627.mm.1	-2,11	0,007392	0,399603		
TC0X00000686.mm.1	1,22	Atp2b3	JUC0X00002212.mm.1	-2,11	0,048448	0,560744		
TC0X00000686.mm.1	1,22	Atp2b3	JUC0X00002216.mm.1	-2,17	0,013578	0,443703		
TC0400002159.mm.1	1,89	Tox	PSR0400018131.mm.1	-2,12	0,015153	0,452699	Cassette Exon	0,15
TC0400002159.mm.1	1,89	Tox	PSR0400018147.mm.1	-2,24	0,028263	0,506565	Cassette Exon	0,12
TC0400002159.mm.1	1,89	Tox	PSR0400018130.mm.1	-2,46	0,012797	0,437255	Cassette Exon	0,15
TC0400002159.mm.1	1,89	Tox	PSR0400018135.mm.1	-2,81	0,026897	0,502518	Alternative 3' Acce	0,28
TC0400002159.mm.1	1,89	Tox	PSR0400018123.mm.1	-2,99	0,015122	0,45267	Alternative 3' Acce	0,3
TC0400002159.mm.1	1,89	Tox	PSR0400018143.mm.1	-3,3	0,025553	0,497808	Alternative 5' Donc	0,36
TC1200001902.mm.1	3,09	Rtn1	PSR1200013072.mm.1	-2,12	0,045262	0,553703		
TC1200001902.mm.1	3,09	Rtn1	PSR1200013079.mm.1	-2,33	0,028785	0,508161	Cassette Exon	0,16
TC1200001902.mm.1	3,09	Rtn1	PSR1200013067.mm.1	-3,04	0,015487	0,454456	Alternative 3' Acce	0,35
TC1200001902.mm.1	3,09	Rtn1	PSR1200013082.mm.1	-3,07	0,046678	0,556812	Alternative 5' Donc	0,21

TC1200001902.mm.1	3,09	Rtn1	PSR1200013083.mm.1	-3,3	0,048479	0,560864	Alternative 5' Donc	0,21
TC1500000478.mm.1	2,03	Efr3a	JUC1500001941.mm.1	-2,12	0,010098	0,418998		
TC1500000478.mm.1	2,03	Efr3a	PSR1500003339.mm.1	-2,13	0,000872	0,311909	Alternative 5' Donc	0,21
TC1500000478.mm.1	2,03	Efr3a	PSR1500003360.mm.1	-2,35	0,04727	0,558223	Alternative 5' Donc	0,22
TC1500000478.mm.1	2,03	Efr3a	PSR1500003373.mm.1	-2,36	0,01984	0,475536	Alternative 5' Donc	0,18
TC1500000478.mm.1	2,03	Efr3a	PSR1500003332.mm.1	-2,52	0,001564	0,331357	Cassette Exon	0,08
TC1500000478.mm.1	2,03	Efr3a	PSR1500003331.mm.1	-2,68	0,000817	0,311886	Alternative 5' Donc	0,12
TC1500000478.mm.1	2,03	Efr3a	PSR1500003367.mm.1	-3,19	0,041324	0,544282	Alternative 5' Donc	0,32
TC0800000711.mm.1	2,08	Atp6v1b2	PSR0800004787.mm.1	-2,12	0,027359	0,503858	Intron Retention	0,28
TC0800000711.mm.1	2,08	Atp6v1b2	PSR0800004791.mm.1	-2,51	0,015631	0,45509	Alternative 3' Acce	0,24
TC0800000711.mm.1	2,08	Atp6v1b2	PSR0800004776.mm.1	-2,53	0,026689	0,502057	Alternative 3' Acce	0,25
TC0800000711.mm.1	2,08	Atp6v1b2	PSR0800004770.mm.1	-2,6	0,007926	0,403819	Alternative 3' Acce	0,24
TC1700001266.mm.1	1,83	Ppm1b	PSR1700011703.mm.1	-2,12	0,031468	0,516922	Cassette Exon	0,02
TC1700001266.mm.1	1,83	Ppm1b	PSR1700011700.mm.1	-2,16	0,030035	0,511774	Cassette Exon	0,17
TC1700001266.mm.1	1,83	Ppm1b	JUC1700006406.mm.1	-2,27	0,002639	0,349612		
TC1700001266.mm.1	1,83	Ppm1b	PSR1700011713.mm.1	-3,14	0,015039	0,452111	Alternative 5' Donc	0,27
TC1900000415.mm.1	1,62	Vldlr	JUC1900002022.mm.1	-2,12	0,038317	0,536178		
TC1900000415.mm.1	1,62	Vldlr	PSR1900003883.mm.1	-2,14	0,046714	0,556856	Alternative 5' Donc	0,18
TC1900000415.mm.1	1,62	Vldlr	PSR1900003847.mm.1	-2,44	0,034059	0,524583	Alternative 3' Acce	0,12
TC1900000415.mm.1	1,62	Vldlr	PSR1900003877.mm.1	-2,49	0,018456	0,468671	Cassette Exon	0,25
TC4_JH584294_random00	2,95	Ccl27a; LOC100861978; Cc	JUC4_JH584294_random	-2,12	0,020519	0,478264		
TC4_JH584294_random00	2,95	Ccl27a; LOC100861978; Cc	PSR4_JH584294_random	-2,98	0,002243	0,34578	Cassette Exon	0,24
TC4_JH584294_random00	2,95	Ccl27a; LOC100861978; Cc	JUC4_JH584294_random	-3,88	0,002135	0,343402		
TC4_JH584293_random00	3,06	Ccl27a; Ccl27b; LOC10086:	JUC4_JH584293_random	-2,12	0,032761	0,520598		
TC4_JH584293_random00	3,06	Ccl27a; Ccl27b; LOC10086:	PSR4_JH584293_random	-2,95	0,003257	0,354243	Cassette Exon	0,23
TC4_JH584293_random00	3,06	Ccl27a; Ccl27b; LOC10086:	JUC4_JH584293_random	-3,74	0,003439	0,3554		
TC0400000011.mm.1	1,15	Tgs1	PSR0400000067.mm.1	-2,12	0,007416	0,399901	Alternative 5' Donc	0,21
TC1900000745.mm.1	2,2		PSR1900006591.mm.1	-2,12	0,031656	0,517405	Alternative 3' Acce	0,03
TC1900000745.mm.1	2,2		PSR1900006590.mm.1	-3,09	0,020732	0,479166	Cassette Exon	0,19
TC1600000659.mm.1	-1,21	BC027231	PSR1600005783.mm.1	-2,12	0,01057	0,422432	Alternative 3' Acce	0,18
TC0200001799.mm.1	1,61	AV039307	PSR0200013842.mm.1	-2,12	0,006106	0,387239	Cassette Exon	0,17
TC0200001799.mm.1	1,61	AV039307	JUC0200006924.mm.1	-2,58	0,010061	0,418902		
TC0800001043.mm.1	1,27	Gm3134	PSR0800008068.mm.1	-2,12	0,02069	0,478999	Alternative 5' Donc	0,16
TC1000002496.mm.1	1,17	Pcsk4	PSR1000018414.mm.1	-2,12	0,02768	0,504734	Alternative 5' Donc	0,16
TC1300002536.mm.1	-1,24	Ocln	PSR1300017213.mm.1	-2,12	0,003337	0,354243	Alternative 3' Acce	0,16
TC0800002369.mm.1	1,17	Zfp869	PSR0800017835.mm.1	-2,12	0,032164	0,518706	Alternative 5' Donc	0,15
TC1000000165.mm.1	1,52	Hbs1l	PSR1000001191.mm.1	-2,12	0,003685	0,357266	Cassette Exon	0,14
TC0500002539.mm.1	1,69	Kctd8	PSR0500022564.mm.1	-2,12	0,035088	0,527591	Alternative 5' Donc	0,13
TC0700000057.mm.1	2,3	U2af2	PSR0700000660.mm.1	-2,12	0,047128	0,557915	Cassette Exon	0,13
TC0700000057.mm.1	2,3	U2af2	JUC0700000328.mm.1	-2,14	0,047906	0,55942		
TC1300000715.mm.1	1,15	Rmi1	PSR1300004713.mm.1	-2,12	0,04584	0,555021	Alternative 5' Donc	0,12
TC0600001867.mm.1	1,32	Thsd7a	PSR0600014696.mm.1	-2,12	0,002732	0,349612	Cassette Exon	0,11
TC0600001867.mm.1	1,32	Thsd7a	JUC0600007710.mm.1	-2,52	0,00673	0,393343		
TC0600001867.mm.1	1,32	Thsd7a	JUC0600007711.mm.1	-3,16	0,019525	0,474538		
TC1200000788.mm.1	1,4	Rbm25	PSR1200005567.mm.1	-2,12	0,04625	0,556168	Alternative 3' Acce	0,11
TC0400003423.mm.1	-1,08	Ccdc30	PSR0400027811.mm.1	-2,12	0,007746	0,402774	Cassette Exon	0,1
TC1200000007.mm.1	1,65	Kif3c	PSR1200000053.mm.1	-2,12	0,008784	0,409822	Cassette Exon	0,1
TC1500001689.mm.1	1,38	ago-02	PSR1500012713.mm.1	-2,12	0,000486	0,298999	Alternative 3' Acce	0,08

TC1400002228.mm.1	-1,06	Prss55	JUC1400009439.mm.1	-2,12	0,033554	0,523131		
TC1400001196.mm.1	-1,27	D130009I18Rik	JUC1400005036.mm.1	-2,12	0,010109	0,418998		
TC0300001181.mm.1	1,2	Dph5	JUC0300005026.mm.1	-2,12	0,036333	0,530547		
TC1300001349.mm.1	-1,2	Gm7120	JUC1300004400.mm.1	-2,12	0,00211	0,342919		
TC1300001349.mm.1	-1,2	Gm7120	JUC1300004401.mm.1	-2,12	0,00211	0,342919		
TC1300001349.mm.1	-1,2	Gm7120	JUC1300004405.mm.1	-2,12	0,00211	0,342919		
TC1300002392.mm.1	-1,29	Vcan	JUC1300008374.mm.1	-2,12	0,004299	0,364027		
TC1300002392.mm.1	-1,29	Vcan	JUC1300008364.mm.1	-2,16	0,031377	0,516345		
TC1700002344.mm.1	1,49	Khsrp	JUC1700011750.mm.1	-2,12	0,017897	0,466466		
TC0100000663.mm.1	1,09	Stk11ip	JUC0100003303.mm.1	-2,12	0,048498	0,560886		
TC0100003870.mm.1	-1	Usf1	JUC0100007435.mm.1	-2,12	0,016272	0,45836		
TC1500000248.mm.1	1,14	Vps13b	JUC1500001087.mm.1	-2,12	0,002734	0,349612		
TC0200002013.mm.1	1,62	Pank2; Gm14233	JUC0200008062.mm.1	-2,12	0,033564	0,523131		
TC1500000719.mm.1	1,26	Xpnpep3	JUC1500003116.mm.1	-2,12	0,037657	0,53428		
TC1500000719.mm.1	1,26	Xpnpep3	JUC1500003114.mm.1	-2,16	0,040799	0,543194		
TC1500000719.mm.1	1,26	Xpnpep3	JUC1500003110.mm.1	-3,25	0,023226	0,490032		
TC1600001827.mm.1	1,87	Epha3	JUC1600007602.mm.1	-2,12	0,005806	0,383104		
TC1600001827.mm.1	1,87	Epha3	JUC1600007606.mm.1	-2,34	0,006053	0,386642		
TC1600001827.mm.1	1,87	Epha3	JUC1600007601.mm.1	-2,49	0,003632	0,3566		
TC1600001827.mm.1	1,87	Epha3	JUC1600007608.mm.1	-3,84	0,018975	0,471398		
TC0500001885.mm.1	1,01	Rbm48	JUC0500009444.mm.1	-2,12	0,009625	0,417315		
TC0500002622.mm.1	1,01	Gm19590	JUC0500012604.mm.1	-2,12	0,014044	0,44654		
TC0500002622.mm.1	1,01	Gm19590	JUC0500012605.mm.1	-2,32	0,035069	0,527574		
TC0700004270.mm.1	1,11		JUC0700018774.mm.1	-2,12	0,011175	0,426409		
TC0500000372.mm.1	1,23	Gm21446	JUC0500001952.mm.1	-2,12	0,007628	0,402028		
TC0500000372.mm.1	1,23	Gm21446	JUC0500001956.mm.1	-2,61	0,002853	0,352207		
TC0900001472.mm.1	-1,17	Prss45	JUC0900006882.mm.1	-2,12	0,024163	0,493245		
TC0800002803.mm.1	1,19	Ccdc79	JUC0800011722.mm.1	-2,12	0,000659	0,304044		
TC0600001078.mm.1	-1,14	Slc41a3	JUC0600004271.mm.1	-2,12	0,027809	0,505442		
TC0600001709.mm.1	-1,06	Lrmp	JUC0600007124.mm.1	-2,12	0,025574	0,497808		
TC0600003379.mm.1	-1,78	Abcc9	JUC0600013924.mm.1	-2,12	0,009835	0,417839		
TC0700002078.mm.1	-1,42	Lsp1	JUC0700009936.mm.1	-2,12	0,036903	0,532283		
TC1000002076.mm.1	1,53	Sobp	JUC1000008051.mm.1	-2,12	0,026624	0,50179		
TC1100001530.mm.1	1,04	Osbpl7	JUC1100007342.mm.1	-2,12	0,042459	0,547627		
TC1100001543.mm.1	1,67	Mllt6	JUC1100007417.mm.1	-2,12	0,00975	0,417839		
TC1000001049.mm.1	1,09		JUC1000004238.mm.1	-2,12	0,044528	0,551885		
TC1000001049.mm.1	1,09		JUC1000004241.mm.1	-2,39	0,048794	0,561497		
TC1000000846.mm.1	1,05	Mum1	JUC1000003454.mm.1	-2,12	0,041727	0,545379		
TC0900002312.mm.1	9,36	Elmod1	PSR0900019349.mm.1	-2,13	0,000629	0,304044	Cassette Exon	0,33
TC0900002312.mm.1	9,36	Elmod1	JUC0900010910.mm.1	-3,17	0,006981	0,395722		
TC0900002312.mm.1	9,36	Elmod1	PSR0900019359.mm.1	-3,46	0,009195	0,413763	Alternative 5' Donc	0,53
TC0900002312.mm.1	9,36	Elmod1	PSR0900019355.mm.1	-6,34	0,000879	0,311909	Alternative 5' Donc	0,48
TC0900002312.mm.1	9,36	Elmod1	JUC0900010916.mm.1	-6,6	0,000081	0,25272		
TC0900002312.mm.1	9,36	Elmod1	PSR0900019342.mm.1	-6,82	0,005707	0,382524	Cassette Exon	0,3
TC0900002312.mm.1	9,36	Elmod1	PSR0900019348.mm.1	-7,72	0,004022	0,361482	Cassette Exon	0,33
TC0900002312.mm.1	9,36	Elmod1	PSR0900019360.mm.1	-11,33	0,004255	0,363521	Cassette Exon	0,41
TC0900002312.mm.1	9,36	Elmod1	PSR0900019350.mm.1	-12,72	0,001021	0,316361	Cassette Exon	0,33
TC0900002312.mm.1	9,36	Elmod1	PSR0900019343.mm.1	-14,6	0,000701	0,30566	Cassette Exon	0,3

TC1000002689.mm.1	-1,16	4930485B16Rik	PSR1000020554.mm.1	-2,13	0,040126	0,541532	Cassette Exon	0,12
TC1000002689.mm.1	-1,16	4930485B16Rik	PSR1000020570.mm.1	-2,19	0,023815	0,491938	Cassette Exon	0,05
TC1000002689.mm.1	-1,16	4930485B16Rik	JUC1000011304.mm.1	-2,21	0,002145	0,343679		
TC1000002689.mm.1	-1,16	4930485B16Rik	PSR1000020555.mm.1	-2,24	0,002065	0,341993	Cassette Exon	0,25
TC1000002689.mm.1	-1,16	4930485B16Rik	PSR1000020583.mm.1	-2,27	0,011733	0,429969	Cassette Exon	0,2
TC1000002689.mm.1	-1,16	4930485B16Rik	PSR1000020581.mm.1	-2,29	0,000252	0,28803	Cassette Exon	0,16
TC1000002689.mm.1	-1,16	4930485B16Rik	PSR1000020558.mm.1	-2,31	0,036934	0,532353	Cassette Exon	0,15
TC1000002689.mm.1	-1,16	4930485B16Rik	JUC1000011283.mm.1	-2,4	0,000296	0,288663		
TC1000002689.mm.1	-1,16	4930485B16Rik	JUC1000011305.mm.1	-2,48	0,001898	0,338712		
TC1000002689.mm.1	-1,16	4930485B16Rik	JUC1000011284.mm.1	-2,62	0,020707	0,479166		
TC1000002689.mm.1	-1,16	4930485B16Rik	PSR1000020567.mm.1	-2,68	0,028493	0,507345	Cassette Exon	0,08
TC1000002689.mm.1	-1,16	4930485B16Rik	JUC1000011298.mm.1	-2,84	0,006851	0,393893		
TC1000002689.mm.1	-1,16	4930485B16Rik	PSR1000020620.mm.1	-2,88	0,043797	0,550419	Cassette Exon	0,25
TC1000002689.mm.1	-1,16	4930485B16Rik	PSR1000020576.mm.1	-3,02	0,000685	0,304511	Cassette Exon	0,28
TC1000002689.mm.1	-1,16	4930485B16Rik	JUC1000011332.mm.1	-3,09	0,008319	0,406313		
TC1000002689.mm.1	-1,16	4930485B16Rik	JUC1000011301.mm.1	-3,12	0,000005	0,179072		
TC1000002689.mm.1	-1,16	4930485B16Rik	JUC1000011346.mm.1	-3,14	0,030517	0,51337		
TC1000002689.mm.1	-1,16	4930485B16Rik	PSR1000020580.mm.1	-3,2	0,00738	0,39943	Cassette Exon	0,19
TC1000002689.mm.1	-1,16	4930485B16Rik	PSR1000020582.mm.1	-3,23	0,001139	0,317344	Cassette Exon	0,33
TC1000002689.mm.1	-1,16	4930485B16Rik	PSR1000020577.mm.1	-3,41	0,000052	0,24627	Cassette Exon	0,35
TC1000002689.mm.1	-1,16	4930485B16Rik	JUC1000011329.mm.1	-5,49	0,040045	0,541323		
TC0400001630.mm.1	2,89	E130218I03Rik	JUC0400006727.mm.1	-2,13	0,001617	0,332831		
TC0400001630.mm.1	2,89	E130218I03Rik	PSR0400012938.mm.1	-2,29	0,026902	0,502518	Intron Retention	0,27
TC0400001630.mm.1	2,89	E130218I03Rik	PSR0400012953.mm.1	-2,3	0,005529	0,380518	Alternative 3' Acce	0,17
TC0400001630.mm.1	2,89	E130218I03Rik	PSR0400012930.mm.1	-2,45	0,021077	0,480371	Alternative 3' Acce	0,32
TC0400001630.mm.1	2,89	E130218I03Rik	JUC0400006729.mm.1	-3	0,032148	0,518586		
TC0400001630.mm.1	2,89	E130218I03Rik	PSR0400012931.mm.1	-3,54	0,025624	0,497926	Alternative 3' Acce	0,32
TC0400001630.mm.1	2,89	E130218I03Rik	JUC0400006730.mm.1	-4,47	0,022301	0,486095		
TC0900002463.mm.1	3,12	Coro2b	PSR0900020621.mm.1	-2,13	0,025591	0,497808	Cassette Exon	0,25
TC0900002463.mm.1	3,12	Coro2b	PSR0900020627.mm.1	-2,3	0,012707	0,436444	Cassette Exon	0,12
TC0900002463.mm.1	3,12	Coro2b	PSR0900020626.mm.1	-3,89	0,002279	0,345991	Cassette Exon	0,27
TC0900002463.mm.1	3,12	Coro2b	JUC0900011621.mm.1	-4,31	0,044534	0,551894		
TC1600001287.mm.1	1,42	Pi4ka	JUC1600005420.mm.1	-2,13	0,008728	0,409503		
TC1600001287.mm.1	1,42	Pi4ka	PSR1600010483.mm.1	-2,26	0,010238	0,420146	Alternative 5' Donc	0,2
TC1600001287.mm.1	1,42	Pi4ka	JUC1600005427.mm.1	-2,34	0,044343	0,551418		
TC1600001287.mm.1	1,42	Pi4ka	PSR1600010461.mm.1	-2,56	0,025763	0,498442	Alternative 3' Acce	0,17
TC1600001287.mm.1	1,42	Pi4ka	PSR1600010420.mm.1	-2,62	0,017992	0,466929	Alternative 5' Donc	0,26
TC0500000992.mm.1	3,29	Cds1	PSR0500008376.mm.1	-2,13	0,044361	0,551441	Cassette Exon	0,23
TC0500000992.mm.1	3,29	Cds1	JUC0500004590.mm.1	-2,13	0,00248	0,349135		
TC0500000992.mm.1	3,29	Cds1	JUC0500004588.mm.1	-2,95	0,027804	0,505442		
TC0500000992.mm.1	3,29	Cds1	PSR0500008375.mm.1	-3,03	0,012844	0,437766	Cassette Exon	0,2
TC0500000992.mm.1	3,29	Cds1	JUC0500004587.mm.1	-3,42	0,025995	0,499207		
TC0500000992.mm.1	3,29	Cds1	JUC0500004596.mm.1	-4,27	0,049486	0,563298		
TC1600000253.mm.1	1,16	Hira; Gm15797; RP23-357	PSR1600002351.mm.1	-2,13	0,01656	0,459612	Intron Retention	0,21
TC1700002431.mm.1	1,84	Mtcl1; Soga2	JUC1700012076.mm.1	-2,13	0,045279	0,553723		
TC1700002431.mm.1	1,84	Mtcl1; Soga2	JUC1700012072.mm.1	-2,41	0,028899	0,50858		
TC1700002431.mm.1	1,84	Mtcl1; Soga2	PSR1700022539.mm.1	-2,61	0,041198	0,544126	Alternative 5' Donc	0,21
TC1700002431.mm.1	1,84	Mtcl1; Soga2	JUC1700012059.mm.1	-2,79	0,007382	0,39943		

TC1700002431.mm.1	1,84	Mtcl1; Soga2	JUC1700012074.mm.1	-2,84	0,011218	0,426683		
TC1700002431.mm.1	1,84	Mtcl1; Soga2	JUC1700012062.mm.1	-3,4	0,003647	0,356935		
TC1700002431.mm.1	1,84	Mtcl1; Soga2	JUC1700012070.mm.1	-3,84	0,04891	0,5618		
TC0300002939.mm.1	1,01	Sec24b	PSR0300023279.mm.1	-2,13	0,012398	0,434944	Alternative 5' Donc	0,18
TC0900000714.mm.1	1,76	Edc3	PSR0900005258.mm.1	-2,13	0,011035	0,425682	Alternative 5' Donc	0,18
TC0900000714.mm.1	1,76	Edc3	PSR0900005259.mm.1	-2,6	0,00759	0,401712	Alternative 5' Donc	0,18
TC1300002170.mm.1	-1		PSR1300013935.mm.1	-2,13	0,047535	0,558701	Alternative 5' Donc	0,18
TC1100000045.mm.1	1,28	Gm11960	PSR1100000541.mm.1	-2,13	0,013356	0,442083	Alternative 3' Acce	0,18
TC0800001327.mm.1	1,42	Exosc6; Aars	PSR0800010906.mm.1	-2,13	0,019995	0,476227	Alternative 5' Donc	0,17
TC1100003622.mm.1	1,16	Snx11	PSR1100033189.mm.1	-2,13	0,020311	0,477245	Alternative 5' Donc	0,17
TC1100003622.mm.1	1,16	Snx11	JUC1100017369.mm.1	-2,15	0,002224	0,345773		
TC0200002469.mm.1	1,19	9430021M05Rik	PSR0200020095.mm.1	-2,13	0,030379	0,512923	Cassette Exon	0,16
TC0500002053.mm.1	1,67	Srpk2	PSR0500018680.mm.1	-2,13	0,010076	0,418974	Alternative 3' Acce	0,16
TC0500002053.mm.1	1,67	Srpk2	JUC0500010186.mm.1	-2,16	0,038514	0,536557		
TC0500002053.mm.1	1,67	Srpk2	JUC0500010170.mm.1	-2,29	0,030681	0,513862		
TC0500002053.mm.1	1,67	Srpk2	PSR0500018663.mm.1	-2,36	0,021382	0,482022	Alternative 3' Acce	0,09
TC0500002053.mm.1	1,67	Srpk2	PSR0500018661.mm.1	-2,39	0,026168	0,50003	Alternative 3' Acce	0,15
TC1100002598.mm.1	2,13	Cyfip2	PSR1100023966.mm.1	-2,13	0,020652	0,478862	Cassette Exon	0,16
TC1100002598.mm.1	2,13	Cyfip2	JUC1100012463.mm.1	-2,14	0,021899	0,484824		
TC0X00000164.mm.1	1,75	Rpl3-ps1	PSR0X00001051.mm.1	-2,13	0,009149	0,413447	Alternative 5' Donc	0,16
TC1100001246.mm.1	1,46	Snord7; AA465934; AI450	PSR1100011682.mm.1	-2,13	0,035541	0,528764	Intron Retention	0,15
TC1900000978.mm.1	1,04	Pcnxl3	PSR1900008741.mm.1	-2,13	0,042535	0,5479	Alternative 3' Acce	0,15
TC0900000042.mm.1	1,35		PSR0900000312.mm.1	-2,13	0,00703	0,396042	Alternative 3' Acce	0,15
TC0600001436.mm.1	1,82	Mlf2	PSR0600011599.mm.1	-2,13	0,015541	0,454617	Alternative 5' Donc	0,14
TC0900001654.mm.1	1,89	Tmem42	JUC0900007825.mm.1	-2,13	0,035426	0,528707		
TC0900001654.mm.1	1,89	Tmem42	PSR0900013897.mm.1	-2,47	0,021573	0,483165	Cassette Exon	0,14
TC0900001654.mm.1	1,89	Tmem42	JUC0900007826.mm.1	-2,83	0,006182	0,387873		
TC0200002712.mm.1	1,22	Gm14444	PSR0200022200.mm.1	-2,13	0,003471	0,355628	Cassette Exon	0,11
TC0200002712.mm.1	1,22	Gm14444	PSR0200022194.mm.1	-2,44	0,004763	0,371039	Cassette Exon	0,13
TC0600002385.mm.1	1,27	Gprin3	PSR0600018463.mm.1	-2,13	0,027973	0,505748	Cassette Exon	0,13
TC0600003603.mm.1	1,43	Grcc10; Rnu7	PSR0600027674.mm.1	-2,13	0,029237	0,509667	Cassette Exon	0,13
TC0700004628.mm.1	1,43	Prr14	PSR0700015815.mm.1	-2,13	0,030968	0,514654	Cassette Exon	0,13
TC1800000304.mm.1	1,41	LOC102639815	PSR1800002228.mm.1	-2,13	0,026074	0,499646	Cassette Exon	0,13
TC0400002782.mm.1	1,59	Zfp37	PSR0400022778.mm.1	-2,13	0,001613	0,332831	Alternative 5' Donc	0,11
TC0400002782.mm.1	1,59	Zfp37	PSR0400022775.mm.1	-2,26	0,044867	0,552613	Alternative 3' Acce	0,12
TC0400002782.mm.1	1,59	Zfp37	PSR0400022779.mm.1	-2,5	0,002079	0,342181	Alternative 5' Donc	0,11
TC1600000447.mm.1	2,57	Tfrc	JUC1600002129.mm.1	-2,13	0,009044	0,412827		
TC1600000447.mm.1	2,57	Tfrc	PSR1600004124.mm.1	-2,41	0,007441	0,399934	Alternative 3' Acce	0,12
TC0700000472.mm.1	1,23		PSR0700003821.mm.1	-2,13	0,003339	0,354243	Cassette Exon	0,1
TC0800002972.mm.1	1,51	Adamts18	PSR0800022937.mm.1	-2,13	0,0269	0,502518	Cassette Exon	0,1
TC0800002972.mm.1	1,51	Adamts18	JUC0800012559.mm.1	-2,46	0,026774	0,502153		
TC0800002972.mm.1	1,51	Adamts18	JUC0800012574.mm.1	-2,74	0,003541	0,3562		
TC1300002084.mm.1	1,48	Trpc7	PSR1300013236.mm.1	-2,13	0,036842	0,532256	Cassette Exon	0,1
TC1300002084.mm.1	1,48	Trpc7	JUC1300006817.mm.1	-3,94	0,041404	0,544627		
TC1300002084.mm.1	1,48	Trpc7	JUC1300006827.mm.1	-6,1	0,015926	0,456718		
TC1300002084.mm.1	1,48	Trpc7	JUC1300006824.mm.1	-6,76	0,028372	0,506881		
TC0X00002403.mm.1	-1,01	Gabre	PSR0X00014818.mm.1	-2,13	0,032938	0,521214	Cassette Exon	0,09
TC0200005404.mm.1	1,73	Cables2	JUC0200023723.mm.1	-2,13	0,010434	0,421595		

TC1200001567.mm.1	-1,07	Colec11	JUC1200005988.mm.1	-2,13	0,004267	0,363868		
TC0200003717.mm.1	1,01	Mettl8	JUC0200016260.mm.1	-2,13	0,047711	0,55896		
TC0200004725.mm.1	1,39	Tasp1	JUC0200020952.mm.1	-2,13	0,015526	0,454567		
TC1700002541.mm.1	1,77	Fam98a	JUC1700012489.mm.1	-2,13	0,034829	0,526823		
TC1800000509.mm.1	-1,3	Hsd17b4	JUC1800002109.mm.1	-2,13	0,029688	0,510949		
TC1700001881.mm.1	-1,14	Kifc1	JUC1700009395.mm.1	-2,13	0,002066	0,341993		
TC1700001881.mm.1	-1,14	Kifc1	JUC1700009394.mm.1	-2,32	0,001783	0,336311		
TC1900000928.mm.1	-1,04	Gm960	JUC1900004486.mm.1	-2,13	0,017715	0,465209		
TC1800001220.mm.1	1,26	Hdac3	JUC1800005016.mm.1	-2,13	0,016966	0,461392		
TC1800001220.mm.1	1,26	Hdac3	JUC1800005014.mm.1	-4	0,01086	0,424161		
TC0200002259.mm.1	2,03	Gm14148	JUC0200008880.mm.1	-2,13	0,012383	0,434939		
TC1700000637.mm.1	-1,06	Ager	JUC1700003425.mm.1	-2,13	0,029018	0,508842		
TC0200000346.mm.1	-1,15	Myo3a	JUC0200000716.mm.1	-2,13	0,006951	0,395668		
TC1600001133.mm.1	1,06	Pam16	JUC1600004686.mm.1	-2,13	0,039627	0,540106		
TC0500002130.mm.1	-1,07	Speer4b	JUC0500010561.mm.1	-2,13	0,025009	0,496061		
TC0800001162.mm.1	1,03	A330008L17Rik	JUC0800004865.mm.1	-2,13	0,042553	0,547959		
TC0800001898.mm.1	-1,4	Adam3	JUC0800008196.mm.1	-2,13	0,00043	0,297771		
TC0900001005.mm.1	1,39	Fam214a	JUC0900004223.mm.1	-2,13	0,000247	0,28803		
TC0900001005.mm.1	1,39	Fam214a	JUC0900004214.mm.1	-2,18	0,031708	0,517443		
TC0900001005.mm.1	1,39	Fam214a	JUC0900004228.mm.1	-3,44	0,020001	0,47625		
TC0600000193.mm.1	-1,06	Ptprz1	JUC0600000614.mm.1	-2,13	0,022756	0,487931		
TC1000001868.mm.1	-1,61	Eya4	JUC1000007355.mm.1	-2,13	0,01467	0,450098		
TC1000002499.mm.1	1,14	Mbd3	JUC1000010141.mm.1	-2,13	0,02888	0,50858		
TC1000000904.mm.1	1,3	Tle2	PSR1000007174.mm.1	-2,13	0,011896	0,431453		
TC1000000904.mm.1	1,3	Tle2	JUC1000003865.mm.1	-2,21	0,023839	0,491997		
TC0X00002084.mm.1	1,8	Tmem255a	JUC0X00006749.mm.1	-2,13	0,042108	0,546516		
TC0X00002084.mm.1	1,8	Tmem255a	JUC0X00006759.mm.1	-2,95	0,003727	0,357586		
TC0400002101.mm.1	1,15	Fam132a	JUC0400009280.mm.1	-2,13	0,001266	0,322251		
TC1000001813.mm.1	7,71	Tnfaip3	JUC1000007221.mm.1	-2,14	0,036317	0,530547		
TC1000001813.mm.1	7,71	Tnfaip3	PSR1000013222.mm.1	-2,39	0,036203	0,530061	Alternative 3' Acce	0,25
TC1000001813.mm.1	7,71	Tnfaip3	PSR1000013221.mm.1	-2,49	0,000385	0,294337	Cassette Exon	0,41
TC1000001813.mm.1	7,71	Tnfaip3	PSR1000013231.mm.1	-2,78	0,004548	0,368844	Alternative 5' Donc	0,23
TC1000001813.mm.1	7,71	Tnfaip3	PSR1000013234.mm.1	-3,58	0,000089	0,255472	Alternative 5' Donc	0,28
TC1000001813.mm.1	7,71	Tnfaip3	PSR1000013235.mm.1	-4,13	0,000943	0,313363	Cassette Exon	0,41
TC1000001813.mm.1	7,71	Tnfaip3	JUC1000007216.mm.1	-4,7	0,007778	0,402902		
TC1000001813.mm.1	7,71	Tnfaip3	PSR1000013225.mm.1	-5,26	0,000078	0,25272	Intron Retention	0,75
TC1000001813.mm.1	7,71	Tnfaip3	PSR1000013236.mm.1	-6,48	0,00357	0,356223	Cassette Exon	0,28
TC1000001813.mm.1	7,71	Tnfaip3	PSR1000013238.mm.1	-6,95	0,00327	0,354243	Cassette Exon	0,28
TC1000001813.mm.1	7,71	Tnfaip3	JUC1000007224.mm.1	-7,13	0,014306	0,448073		
TC1000001813.mm.1	7,71	Tnfaip3	JUC1000007215.mm.1	-11,38	0,000349	0,290082		
TC0700002413.mm.1	10,88	Crx	PSR0700021197.mm.1	-2,14	0,003707	0,35738	Alternative 3' Acce	0,12
TC0700002413.mm.1	10,88	Crx	PSR0700021202.mm.1	-5,85	0,005781	0,383104	Alternative 5' Donc	0,34
TC0700002413.mm.1	10,88	Crx	PSR0700021205.mm.1	-7,77	0,003138	0,353892	Cassette Exon	0,28
TC0700002413.mm.1	10,88	Crx	PSR0700021203.mm.1	-10,99	0,002357	0,34829	Cassette Exon	0,4
TC0700002413.mm.1	10,88	Crx	JUC0700011293.mm.1	-21,03	0,000905	0,313363		
TC0700002413.mm.1	10,88	Crx	JUC0700011296.mm.1	-22,96	0,001318	0,324348		
TC0700002413.mm.1	10,88	Crx	PSR0700021208.mm.1	-24,21	0,000634	0,304044	Alternative 5' Donc	0,48
TC0800002169.mm.1	2,06	Stox2	PSR0800016693.mm.1	-2,14	0,005937	0,385008	Alternative 3' Acce	0,12

TC0800002169.mm.1	2,06	Stox2	PSR0800016704.mm.1	-2,47	0,02582	0,498598		
TC0800002169.mm.1	2,06	Stox2	JUC0800009098.mm.1	-2,58	0,028228	0,506368		
TC0800002169.mm.1	2,06	Stox2	JUC0800009095.mm.1	-2,87	0,014535	0,449545		
TC0800002169.mm.1	2,06	Stox2	JUC0800009097.mm.1	-3,08	0,001372	0,32572		
TC0800002169.mm.1	2,06	Stox2	PSR0800016708.mm.1	-3,43	0,01691	0,460797	Cassette Exon	0,3
TC0200004540.mm.1	1,19	Gabpb1	JUC0200020063.mm.1	-2,14	0,002138	0,343494		
TC0200004540.mm.1	1,19	Gabpb1	JUC0200020065.mm.1	-2,31	0,022103	0,485486		
TC0200004540.mm.1	1,19	Gabpb1	JUC0200020067.mm.1	-2,31	0,023295	0,49004		
TC0200004540.mm.1	1,19	Gabpb1	PSR0200038752.mm.1	-2,83	0,047618	0,558743	Alternative 3' Acce	0,28
TC0300002879.mm.1	2,07	Ndst3	PSR0300022800.mm.1	-2,14	0,003345	0,354243	Cassette Exon	0,2
TC0300002879.mm.1	2,07	Ndst3	PSR0300022805.mm.1	-2,18	0,001595	0,33209	Cassette Exon	0,25
TC0300002879.mm.1	2,07	Ndst3	PSR0300022807.mm.1	-2,46	0,030755	0,514087	Cassette Exon	0,25
TC0300002879.mm.1	2,07	Ndst3	JUC0300011895.mm.1	-2,75	0,017495	0,463964		
TC0300002879.mm.1	2,07	Ndst3	PSR0300022808.mm.1	-2,96	0,009367	0,415193	Cassette Exon	0,25
TC0300002879.mm.1	2,07	Ndst3	PSR0300022806.mm.1	-3,22	0,028634	0,50782	Cassette Exon	0,25
TC0300002879.mm.1	2,07	Ndst3	JUC0300011884.mm.1	-3,53	0,043611	0,550141		
TC0300002879.mm.1	2,07	Ndst3	JUC0300011898.mm.1	-3,65	0,031916	0,518021		
TC1100001006.mm.1	1,34	Zzef1	JUC1100004725.mm.1	-2,14	0,025157	0,496555		
TC1100001006.mm.1	1,34	Zzef1	PSR1100009040.mm.1	-2,79	0,042355	0,547419	Cassette Exon	0,19
TC1200001663.mm.1	1,12	Ifrd1	PSR1200011583.mm.1	-2,14	0,021641	0,48367	Alternative 5' Donc	0,18
TC1200001663.mm.1	1,12	Ifrd1	JUC1200006421.mm.1	-2,57	0,032331	0,519155		
TC0300002002.mm.1	2,53		PSR0300015279.mm.1	-2,14	0,048847	0,561704	Alternative 5' Donc	0,18
TC0200004974.mm.1	1,06	Fam83c	PSR0200042270.mm.1	-2,14	0,018738	0,470733	Alternative 3' Acce	0,17
TC0500001656.mm.1	1,49	Zfp157	PSR0500015242.mm.1	-2,14	0,005727	0,382767	Alternative 5' Donc	0,17
TC0500001656.mm.1	1,49	Zfp157	JUC0500008287.mm.1	-2,37	0,049696	0,563531		
TC0500001656.mm.1	1,49	Zfp157	JUC0500008289.mm.1	-2,41	0,002575	0,349501		
TC0700002039.mm.1	-1,11	Ptdss2	PSR0700017994.mm.1	-2,14	0,038335	0,53622	Alternative 3' Acce	0,17
TC1700000550.mm.1	1,63	Rrp1b	PSR1700005034.mm.1	-2,14	0,018299	0,468049	Alternative 3' Acce	0,16
TC1400002081.mm.1	1,19	Jph4	PSR1400015874.mm.1	-2,14	0,004016	0,361291	Alternative 5' Donc	0,13
TC0200003263.mm.1	1,43	Zer1	PSR0200027516.mm.1	-2,14	0,017637	0,46458	Cassette Exon	0,12
TC0800001436.mm.1	1,94	6430548M08Rik	PSR0800011755.mm.1	-2,14	0,016607	0,459815	Cassette Exon	0,12
TC0800001436.mm.1	1,94	6430548M08Rik	PSR0800011756.mm.1	-2,44	0,010866	0,424174	Cassette Exon	0,12
TC0800001436.mm.1	1,94	6430548M08Rik	JUC0800006457.mm.1	-4,53	0,010857	0,424161		
TC0300000367.mm.1	1,04	Mgst2	PSR0300002629.mm.1	-2,14	0,011938	0,431717	Cassette Exon	0,11
TC0800000168.mm.1	-1,07	Mcph1	PSR0800001512.mm.1	-2,14	0,048697	0,561345	Cassette Exon	0,11
TC1100003588.mm.1	1,34	Kat7	PSR1100032927.mm.1	-2,14	0,015211	0,452773	Cassette Exon	0,09
TC1100003588.mm.1	1,34	Kat7	PSR1100032930.mm.1	-2,7	0,001267	0,322251	Alternative 5' Donc	0,11
TC1100003588.mm.1	1,34	Kat7	JUC1100017244.mm.1	-2,97	0,003056	0,353892		
TC1100003588.mm.1	1,34	Kat7	JUC1100017261.mm.1	-4,04	0,014315	0,448078		
TC1700001663.mm.1	-1,29		PSR1700015170.mm.1	-2,14	0,021364	0,481952	Cassette Exon	0,11
TC0200001847.mm.1	-1,11	Duox1	PSR0200014346.mm.1	-2,14	0,000367	0,291725	Cassette Exon	0,1
TC0500003371.mm.1	1,58	Gtf2ird1	PSR0500030400.mm.1	-2,14	0,000675	0,304338	Cassette Exon	0,1
TC0500003371.mm.1	1,58	Gtf2ird1	JUC0500016511.mm.1	-2,28	0,026527	0,501411		
TC0500003371.mm.1	1,58	Gtf2ird1	JUC0500016542.mm.1	-4,73	0,007824	0,403089		
TC0700002715.mm.1	2,25	Spred3	PSR0700023952.mm.1	-2,14	0,013993	0,446353	Cassette Exon	0,1
TC0700002715.mm.1	2,25	Spred3	PSR0700023945.mm.1	-2,18	0,000668	0,304088	Cassette Exon	0,08
TC1100000979.mm.1	1,2	Wscd1	PSR1100008783.mm.1	-2,14	0,014009	0,446353	Cassette Exon	0,1
TC1100000979.mm.1	1,2	Wscd1	JUC1100004593.mm.1	-2,29	0,018025	0,46697		

TC1100004094.mm.1	1,31	Recql5	PSR1100038532.mm.1	-2,14	0,021822	0,484598	Alternative 5' Donc	0,09
TC0300000365.mm.1	1,1		JUC0300001340.mm.1	-2,14	0,031064	0,515094		
TC1300002318.mm.1	1,62	Sdha	JUC1300008008.mm.1	-2,14	0,01441	0,448751		
TC0200004608.mm.1	1,67	Pdyn	JUC0200020447.mm.1	-2,14	0,0015	0,329714		
TC0100002365.mm.1	1,07	Satb2	JUC0100010891.mm.1	-2,14	0,006078	0,386691		
TC0100000933.mm.1	-1,03	Ano7	JUC0100004542.mm.1	-2,14	0,020916	0,479793		
TC0100000927.mm.1	-1,81	Sned1; Mir6901; mmu-mi	JUC0100004518.mm.1	-2,14	0,012773	0,437233		
TC1900001056.mm.1	-1,33	Slc22a26	JUC1900005379.mm.1	-2,14	0,009314	0,414883		
TC1800001581.mm.1	1,29	Me2	JUC1800006373.mm.1	-2,14	0,022144	0,485609		
TC1800001581.mm.1	1,29	Me2	JUC1800006378.mm.1	-3,04	0,005771	0,383104		
TC1800001581.mm.1	1,29	Me2	JUC1800006375.mm.1	-5,99	0,000344	0,289576		
TC1900000065.mm.1	1,39	AI837181	JUC1900000419.mm.1	-2,14	0,00532	0,378059		
TC1900000167.mm.1	1,56	Eml3	JUC1900001136.mm.1	-2,14	0,028434	0,507218		
TC1900000167.mm.1	1,56	Eml3	JUC1900001150.mm.1	-2,15	0,019526	0,474538		
TC0200002327.mm.1	1,08	Bpifb2	JUC0200009238.mm.1	-2,14	0,036408	0,53088		
TC0100003848.mm.1	-1,06		JUC0100017862.mm.1	-2,14	0,019786	0,475149		
TC1700000384.mm.1	-1,16	Mslnl	JUC1700001752.mm.1	-2,14	0,045403	0,554085		
TC0800001727.mm.1	1,06	Pcid2	JUC0800007601.mm.1	-2,14	0,006119	0,387322		
TC0800001727.mm.1	1,06	Pcid2	JUC0800007618.mm.1	-2,95	0,013507	0,443256		
TC0500002235.mm.1	1,01		JUC0500011144.mm.1	-2,14	0,033103	0,521762		
TC0900001638.mm.1	-1,23	Abhd5	JUC0900007724.mm.1	-2,14	0,01671	0,460199		
TC0700004620.mm.1	1,16	Sbsn	JUC0700002569.mm.1	-2,14	0,00679	0,393554		
TC0700000161.mm.1	1,13	Lig1	JUC0700000603.mm.1	-2,14	0,008608	0,40841		
TC0600001093.mm.1	1,76	Ccdc174	JUC0600004361.mm.1	-2,14	0,018882	0,471134		
TC0600003114.mm.1	-1,07	Ptpn6	JUC0600012556.mm.1	-2,14	0,017344	0,46353		
TC0500003411.mm.1	1,02	Srcrb4d	JUC0500016780.mm.1	-2,14	0,042053	0,546234		
TC0700003530.mm.1	1,04	AU020206	PSR0700029861.mm.1	-2,14	0,042379	0,547533		
TC0700001715.mm.1	-1,18	Acsm3	JUC0700007436.mm.1	-2,14	0,043498	0,549901		
TC0400004191.mm.1	1,61	Slc6a9	PSR0400009723.mm.1	-2,14	0,034287	0,525366		
TC0400004191.mm.1	1,61	Slc6a9	JUC0400005103.mm.1	-2,6	0,044141	0,551024		
TC0400004191.mm.1	1,61	Slc6a9	JUC0400005108.mm.1	-3,07	0,007611	0,401767		
TC0300002481.mm.1	-1,13	Adamtsl4	JUC0300010005.mm.1	-2,14	0,036261	0,530445		
TC1100001216.mm.1	1,54		JUC1100006003.mm.1	-2,14	0,039836	0,540922		
TC0900003310.mm.1	1,15	Lztfl1	JUC0900015705.mm.1	-2,14	0,004104	0,36228		
TC0400002792.mm.1	-1,06	Pole3	JUC0400011935.mm.1	-2,14	0,002648	0,349612		
TC0900002972.mm.1	1,63	Tmem108	JUC0900013667.mm.1	-2,14	0,011673	0,429939		
TC1000000015.mm.1	1,25	Mthfd1l	JUC1000000063.mm.1	-2,14	0,017165	0,46232		
TC0400002531.mm.1	1,69	Gba2	PSR0400020693.mm.1	-2,15	0,001635	0,332976	Alternative 5' Donc	0,1
TC0400002531.mm.1	1,69	Gba2	PSR0400020688.mm.1	-2,6	0,014542	0,449545	Intron Retention	0,36
TC0500003221.mm.1	1,23	Kdm2b	PSR0500028854.mm.1	-2,15	0,032381	0,519221	Cassette Exon	0,1
TC0500003221.mm.1	1,23	Kdm2b	PSR0500028828.mm.1	-3,49	0,043683	0,550236	Alternative 3' Acce	0,29
TC1400000858.mm.1	1,47	Sacs	JUC1400003783.mm.1	-2,15	0,006675	0,393187		
TC1400000858.mm.1	1,47	Sacs	PSR1400007012.mm.1	-2,47	0,000472	0,298999	Alternative 3' Acce	0,26
TC0300000881.mm.1	1,14	Rfx5	JUC0300003562.mm.1	-2,15	0,014609	0,449914		
TC0300000881.mm.1	1,14	Rfx5	PSR0300006839.mm.1	-2,62	0,013352	0,442045	Alternative 5' Donc	0,25
TC0500002968.mm.1	1,33		PSR0500026261.mm.1	-2,15	0,024617	0,494589	Intron Retention	0,23
TC0800003025.mm.1	1,07	Taf1c	PSR0800023278.mm.1	-2,15	0,025007	0,496061	Alternative 3' Acce	0,09
TC0800003025.mm.1	1,07	Taf1c	PSR0800023272.mm.1	-2,32	0,02093	0,479886	Alternative 3' Acce	0,2

TC0800003025.mm.1	1,07	Taf1c	PSR0800023281.mm.1	-2,5	0,013779	0,444974	Cassette Exon	0,14
TC0100001037.mm.1	1,74	Cdh7	PSR0100008629.mm.1	-2,15	0,037642	0,534212	Alternative 5' Donc	0,19
TC0100001037.mm.1	1,74	Cdh7	PSR0100008602.mm.1	-2,27	0,018218	0,467632		
TC0100001037.mm.1	1,74	Cdh7	PSR0100008627.mm.1	-2,3	0,004517	0,368648	Alternative 5' Donc	0,19
TC0100001037.mm.1	1,74	Cdh7	JUC0100004965.mm.1	-2,35	0,01496	0,452048		
TC0100001037.mm.1	1,74	Cdh7	JUC0100004983.mm.1	-2,68	0,018536	0,469204		
TC0100001037.mm.1	1,74	Cdh7	JUC0100004979.mm.1	-2,69	0,014988	0,452048		
TC0100001037.mm.1	1,74	Cdh7	JUC0100004981.mm.1	-3,84	0,04661	0,556801		
TC0100001037.mm.1	1,74	Cdh7	PSR0100008625.mm.1	-3,87	0,0078	0,403089		
TC1100001194.mm.1	1,14	Suz12	PSR1100011083.mm.1	-2,15	0,049529	0,563325	Alternative 3' Acce	0,19
TC0300002741.mm.1	-1,18	Celsr2	PSR0300021501.mm.1	-2,15	0,013322	0,441836	Alternative 3' Acce	0,18
TC1100003766.mm.1	1,59	Hap1	PSR1100034583.mm.1	-2,15	0,006474	0,390467	Alternative 5' Donc	0,18
TC1100003766.mm.1	1,59	Hap1	PSR1100034576.mm.1	-2,48	0,002454	0,349135	Cassette Exon	0,14
TC1100003766.mm.1	1,59	Hap1	JUC1100018092.mm.1	-2,76	0,048368	0,56056		
TC0700001430.mm.1	1,02	Stard10	PSR0700012195.mm.1	-2,15	0,001752	0,335996	Alternative 5' Donc	0,17
TC0700002093.mm.1	1,2		PSR0700018885.mm.1	-2,15	0,017371	0,463612	Alternative 3' Acce	0,17
TC0500003202.mm.1	1,58	Cux2	PSR0500028574.mm.1	-2,15	0,002288	0,34605	Cassette Exon	0,09
TC0500003202.mm.1	1,58	Cux2	PSR0500028552.mm.1	-2,24	0,008689	0,408815	Alternative 5' Donc	0,12
TC0500003202.mm.1	1,58	Cux2	JUC0500015541.mm.1	-2,37	0,007765	0,402774		
TC0500003202.mm.1	1,58	Cux2	PSR0500028561.mm.1	-2,51	0,002856	0,352207	Cassette Exon	0,16
TC0700003464.mm.1	-1,31	Arrdc4	PSR0700029482.mm.1	-2,15	0,016716	0,460202	Cassette Exon	0,16
TC0700003464.mm.1	-1,31	Arrdc4	PSR0700029487.mm.1	-2,16	0,008141	0,405394	Cassette Exon	0,11
TC0100003533.mm.1	-1,33	Fcer1g	PSR0100028905.mm.1	-2,15	0,025037	0,496091	Cassette Exon	0,14
TC0200004975.mm.1	1,39	Uqcc1; Uqcc	PSR0200042300.mm.1	-2,15	0,048331	0,560493	Alternative 3' Acce	0,14
TC1800001377.mm.1	-1,08	4930511M06Rik; 1700066	PSR1800010057.mm.1	-2,15	0,000381	0,293183	Cassette Exon	0,14
TC1700000805.mm.1	1,07	Esp18	PSR1700007818.mm.1	-2,15	0,005776	0,383104	Cassette Exon	0,13
TC0700000056.mm.1	1,53	Ccdc106	PSR0700000639.mm.1	-2,15	0,029471	0,510123	Alternative 3' Acce	0,12
TC0200000406.mm.1	1,23	Anapc2	PSR0200002269.mm.1	-2,15	0,007689	0,402552	Cassette Exon	0,11
TC0700000250.mm.1	1,43	Psg16	PSR0700001857.mm.1	-2,15	0,002102	0,342919	Cassette Exon	0,11
TC1100003064.mm.1	1,85	Fgf11	PSR1100028127.mm.1	-2,15	0,007868	0,403545	Cassette Exon	0,11
TC1100003064.mm.1	1,85	Fgf11	JUC1100014743.mm.1	-3,22	0,039747	0,540496		
TC1600001245.mm.1	1,24	Marf1	PSR1600009960.mm.1	-2,15	0,026904	0,502518	Alternative 3' Acce	0,1
TC0900000448.mm.1	1,23	Tmem225	PSR0900002933.mm.1	-2,15	0,00659	0,39213	Cassette Exon	0,09
TC1400001030.mm.1	-1,08		JUC1400004407.mm.1	-2,15	0,030645	0,513747		
TC1400002072.mm.1	1,05	Efs	JUC1400008548.mm.1	-2,15	0,035153	0,527974		
TC1100004220.mm.1	1,05	Pcyt2	JUC1100021134.mm.1	-2,15	0,001589	0,331726		
TC1300000009.mm.1	1,53	Asb13	JUC1300000044.mm.1	-2,15	0,048129	0,559938		
TC1300000285.mm.1	-1,07	Dcdc2a	JUC1300000909.mm.1	-2,15	0,013997	0,446353		
TC1700002293.mm.1	1,09	Slc5a7	JUC1700011428.mm.1	-2,15	0,030601	0,513461		
TC1700002293.mm.1	1,09	Slc5a7	JUC1700011431.mm.1	-2,64	0,006381	0,389522		
TC1700001484.mm.1	-1,56	Thbs2	JUC1700007221.mm.1	-2,15	0,010955	0,425094		
TC1900000563.mm.1	1	Cyp2c65	JUC1900002711.mm.1	-2,15	0,039508	0,539762		
TC1500002277.mm.1	-1,15	Krt76	JUC1500010724.mm.1	-2,15	0,047333	0,558291		
TC1600000044.mm.1	1,1	Ubn1	JUC1600000198.mm.1	-2,15	0,031976	0,518183		
TC1500000561.mm.1	1,23	Rhpn1	JUC1500002278.mm.1	-2,15	0,000568	0,304044		
TC1500000561.mm.1	1,23	Rhpn1	JUC1500002263.mm.1	-2,41	0,042692	0,548097		
TC1700000313.mm.1	-1,11	Zscan10	PSR1700002272.mm.1	-2,15	0,036725	0,531766		
TC1700000344.mm.1	-1,03	Pkd1	JUC1700001422.mm.1	-2,15	0,038223	0,535805		

TC0200000055.mm.1	-1,04	Nudt5	JUC0200000168.mm.1	-2,15	0,000379	0,293011		
TC0200000019.mm.1	1,67	Frmd4a	JUC0200000091.mm.1	-2,15	0,030682	0,513862		
TC0200000552.mm.1	-1,07	Fubp3	JUC0200002431.mm.1	-2,15	0,016557	0,459612		
TC1600001276.mm.1	-1,07	Ppil2	JUC1600005381.mm.1	-2,15	0,040946	0,543423		
TC0500001650.mm.1	1,81	6330418K02Rik	PSR0500015157.mm.1	-2,15	0,0024	0,348564		
TC0700000683.mm.1	1,04		JUC0700002849.mm.1	-2,15	0,001938	0,340349		
TC0600001944.mm.1	-1,22	Wasl	JUC0600008004.mm.1	-2,15	0,000327	0,28927		
TC1100002683.mm.1	1,53	Zfp2	JUC1100012704.mm.1	-2,15	0,014234	0,447947		
TC1100002683.mm.1	1,53	Zfp2	JUC1100012713.mm.1	-2,15	0,043542	0,549995		
TC1100002683.mm.1	1,53	Zfp2	JUC1100012705.mm.1	-2,26	0,025547	0,497808		
TC1100002683.mm.1	1,53	Zfp2	JUC1100012710.mm.1	-4,08	0,041095	0,543933		
TC1100002705.mm.1	1,45	Gm26551	JUC1100012817.mm.1	-2,15	0,035913	0,529399		
TC0400003418.mm.1	-1,15	Ermap	JUC0400014433.mm.1	-2,15	0,003987	0,360652		
TC0400003461.mm.1	6,37	Gm8359	JUC0400014629.mm.1	-2,15	0,039898	0,54093		
TC0X00001028.mm.1	-1,07	Jpx; Gm9157	JUC0X00003430.mm.1	-2,15	0,036985	0,532502		
TC0400002949.mm.1	2,29	Psip1	PSR0400023991.mm.1	-2,16	0,021475	0,482555	Cassette Exon	0,12
TC0400002949.mm.1	2,29	Psip1	PSR0400023970.mm.1	-2,2	0,000304	0,288663	Cassette Exon	0,18
TC0400002949.mm.1	2,29	Psip1	PSR0400023989.mm.1	-2,25	0,019408	0,473802	Alternative 5' Donc	0,2
TC0400002949.mm.1	2,29	Psip1	JUC0400012516.mm.1	-2,25	0,002491	0,349135		
TC0400002949.mm.1	2,29	Psip1	PSR0400023992.mm.1	-2,37	0,021954	0,485095	Cassette Exon	0,12
TC0400002949.mm.1	2,29	Psip1	PSR0400023969.mm.1	-2,39	0,004773	0,371044	Cassette Exon	0,15
TC0400002949.mm.1	2,29	Psip1	JUC0400012528.mm.1	-3,01	0,015421	0,454066		
TC0400002949.mm.1	2,29	Psip1	PSR0400023968.mm.1	-6,13	0,001508	0,330175	Alternative 5' Donc	0,34
TC0200002836.mm.1	3,66	Pcmtd2	PSR0200023531.mm.1	-2,16	0,047213	0,558132	Cassette Exon	0,19
TC0200002836.mm.1	3,66	Pcmtd2	PSR0200023543.mm.1	-2,31	0,003082	0,353892	Cassette Exon	0,07
TC0200002836.mm.1	3,66	Pcmtd2	JUC0200011930.mm.1	-2,52	0,017027	0,461729		
TC0200002836.mm.1	3,66	Pcmtd2	JUC0200011932.mm.1	-2,64	0,017545	0,464137		
TC0200002836.mm.1	3,66	Pcmtd2	PSR0200023529.mm.1	-4,76	0,004395	0,366181	Alternative 3' Acce	0,29
TC0200002836.mm.1	3,66	Pcmtd2	JUC0200011937.mm.1	-7,42	0,000267	0,28803		
TC0200002836.mm.1	3,66	Pcmtd2	JUC0200011934.mm.1	-8,09	0,008032	0,40434		
TC0500001526.mm.1	2,21	Rabgef1	PSR0500013783.mm.1	-2,16	0,012604	0,435933	Cassette Exon	0,12
TC0500001526.mm.1	2,21	Rabgef1	PSR0500013802.mm.1	-2,25	0,00797	0,403853	Alternative 3' Acce	0,17
TC0500001526.mm.1	2,21	Rabgef1	PSR0500013804.mm.1	-2,35	0,008263	0,405976	Intron Retention	0,23
TC0500001526.mm.1	2,21	Rabgef1	JUC0500007559.mm.1	-2,46	0,006181	0,387873		
TC0500001526.mm.1	2,21	Rabgef1	PSR0500013789.mm.1	-2,6	0,035069	0,527574	Alternative 5' Donc	0,28
TC1300002520.mm.1	3,58	Map1b	PSR1300016968.mm.1	-2,16	0,037314	0,533573	Cassette Exon	0,09
TC1300002520.mm.1	3,58	Map1b	JUC1300009020.mm.1	-2,42	0,002984	0,352501		
TC1300002520.mm.1	3,58	Map1b	PSR1300016970.mm.1	-3,18	0,039976	0,540992	Alternative 5' Donc	0,23
TC0900000752.mm.1	1,36	Gramd2; 4933407I18Rik	PSR0900005615.mm.1	-2,16	0,008687	0,408815	Alternative 3' Acce	0,18
TC0900000752.mm.1	1,36	Gramd2; 4933407I18Rik	PSR0900005601.mm.1	-2,27	0,035095	0,527591	Alternative 3' Acce	0,2
TC0900000752.mm.1	1,36	Gramd2; 4933407I18Rik	JUC0900002983.mm.1	-2,42	0,015322	0,453681		
TC0100003021.mm.1	-1,02	Zranb3	PSR0100024195.mm.1	-2,16	0,024727	0,494838	Alternative 3' Acce	0,19
TC1500001152.mm.1	-1,12	Card6	PSR1500009283.mm.1	-2,16	0,024923	0,4958	Alternative 5' Donc	0,18
TC1500001646.mm.1	1,39	Kcnq3	PSR1500012357.mm.1	-2,16	0,046252	0,556168	Cassette Exon	0,07
TC1500001646.mm.1	1,39	Kcnq3	PSR1500012362.mm.1	-2,75	0,00161	0,332831	Cassette Exon	0,15
TC1800000750.mm.1	1,31		PSR1800005448.mm.1	-2,16	0,042271	0,547092	Alternative 5' Donc	0,14
TC0300002355.mm.1	2,28	Chrnb2	PSR0300018163.mm.1	-2,16	0,000939	0,313363	Cassette Exon	0,12
TC0700002387.mm.1	1,42	Vmn1r90; Nlrp5-ps; Gm18	PSR0700021103.mm.1	-2,16	0,019045	0,471941	Cassette Exon	0,11

TC0700002387.mm.1	1,42	Vmn1r90; Nlrp5-ps; Gm18	PSR0700021109.mm.1	-2,24	0,035008	0,527412	Cassette Exon	0,12
TC0700002387.mm.1	1,42	Vmn1r90; Nlrp5-ps; Gm18	PSR0700021122.mm.1	-2,36	0,011267	0,426793	Cassette Exon	0,11
TC1100000725.mm.1	1,54	4933439C10Rik	PSR1100005782.mm.1	-2,16	0,031577	0,517177	Cassette Exon	0,12
TC0300002493.mm.1	1,02	Vps45	PSR0300019471.mm.1	-2,16	0,004967	0,373851	Cassette Exon	0,11
TC0900001349.mm.1	2,72	Pcbp4	PSR0900010521.mm.1	-2,16	0,004962	0,373851	Cassette Exon	0,11
TC0900001349.mm.1	2,72	Pcbp4	JUC0900005796.mm.1	-2,56	0,024244	0,493294		
TC0900001349.mm.1	2,72	Pcbp4	JUC0900005786.mm.1	-4,71	0,001528	0,330287		
TC1300001096.mm.1	1,67	Gcnt4	PSR1300006964.mm.1	-2,16	0,019019	0,471745	Cassette Exon	0,11
TC1400000720.mm.1	1,17	Trdj1; Trdj2; Trdc; A63003	PSR1400005729.mm.1	-2,16	0,016529	0,459611	Cassette Exon	0,11
TC1400000720.mm.1	1,17	Trdj1; Trdj2; Trdc; A63003	JUC1400003089.mm.1	-2,75	0,022514	0,487114		
TC0700002179.mm.1	1,1	Sbk3; Gm1078	PSR0700019920.mm.1	-2,16	0,013023	0,439354	Cassette Exon	0,1
TC0300002453.mm.1	1	Cgn	PSR0300018846.mm.1	-2,16	0,046302	0,556186	Cassette Exon	0,07
TC1200001875.mm.1	1,07	Cdkl1	JUC1200007077.mm.1	-2,16	0,005479	0,379939		
TC1100003804.mm.1	1,13	Ptges3l; Aarsd1; Gm2702	JUC1100018429.mm.1	-2,16	0,008605	0,40841		
TC1100003804.mm.1	1,13	Ptges3l; Aarsd1; Gm2702	JUC1100018417.mm.1	-4,66	0,043004	0,54841		
TC0200003601.mm.1	1,05	Ly75	JUC0200015575.mm.1	-2,16	0,001269	0,322251		
TC1300000650.mm.1	-1,01	Fgfr4	JUC1300002196.mm.1	-2,16	0,010189	0,419711		
TC1900001390.mm.1	1,03	Sgms1	JUC1900006654.mm.1	-2,16	0,005365	0,378627		
TC0100000811.mm.1	-1,15	Eif4e2	JUC0100003888.mm.1	-2,16	0,011233	0,426718		
TC1500001701.mm.1	-1,03	Mroh5	JUC1500007298.mm.1	-2,16	0,004946	0,373293		
TC1500001701.mm.1	-1,03	Mroh5	JUC1500007297.mm.1	-2,64	0,01001	0,418602		
TC1500000605.mm.1	-1,52	Gpt	JUC1500002547.mm.1	-2,16	0,007349	0,399281		
TC1700000302.mm.1	-1,22	Gm5493	JUC1700001185.mm.1	-2,16	0,04016	0,541622		
TC0100003481.mm.1	-1,17		JUC0100016270.mm.1	-2,16	0,038348	0,53622		
TC0800000754.mm.1	1,1	Ell	JUC0800002831.mm.1	-2,16	0,047548	0,558707		
TC0800000754.mm.1	1,1	Ell	JUC0800002830.mm.1	-2,67	0,016911	0,460797		
TC0500000533.mm.1	1,02	Zcchc4	JUC0500002716.mm.1	-2,16	0,014111	0,447137		
TC0900000722.mm.1	1,07	Cyp11a1	JUC0900002810.mm.1	-2,16	0,046032	0,555624		
TC0600000551.mm.1	1,12	Krba1	JUC0600002034.mm.1	-2,16	0,03307	0,521669		
TC1000002719.mm.1	1,03		JUC1000011484.mm.1	-2,16	0,025599	0,497821		
TC1100002406.mm.1	1,44	Ccdc85a	JUC1100011739.mm.1	-2,16	0,004937	0,373093		
TC1100002406.mm.1	1,44	Ccdc85a	JUC1100011744.mm.1	-2,32	0,010779	0,423955		
TC1100002406.mm.1	1,44	Ccdc85a	JUC1100011734.mm.1	-2,54	0,007458	0,400326		
TC1100001834.mm.1	1,73	Prkar1a	JUC1100008996.mm.1	-2,16	0,044414	0,551538		
TC0400003448.mm.1	1,32	Smap2	JUC0400014595.mm.1	-2,16	0,02945	0,510051		
TC0900002421.mm.1	1,2	Senp8	JUC0900011459.mm.1	-2,16	0,037542	0,534001		
TC0900002036.mm.1	-1,22	Chek1	JUC0900009535.mm.1	-2,16	0,008596	0,408294		
TC0900002036.mm.1	-1,22	Chek1	JUC0900009527.mm.1	-2,44	0,02938	0,509995		
TC0400002457.mm.1	1,26	Nol6	JUC0400010297.mm.1	-2,16	0,019381	0,473624		
TC0500000814.mm.1	2,41	Rufy3	PSR0500007156.mm.1	-2,17	0,002946	0,352501	Cassette Exon	0,1
TC0500000814.mm.1	2,41	Rufy3	PSR0500007150.mm.1	-2,99	0,035579	0,528808	Alternative 3' Acce	0,25
TC0500000814.mm.1	2,41	Rufy3	PSR0500007153.mm.1	-3,79	0,003291	0,354243	Intron Retention	0,69
TC0500000814.mm.1	2,41	Rufy3	PSR0500007149.mm.1	-4,34	0,005746	0,382993	Cassette Exon	0,41
TC0500000814.mm.1	2,41	Rufy3	JUC0500003917.mm.1	-5,18	0,00693	0,395213		
TC0700001818.mm.1	7,21	Fam57b	PSR0700015538.mm.1	-2,17	0,01747	0,463886	Cassette Exon	0,31
TC0700001818.mm.1	7,21	Fam57b	JUC0700008145.mm.1	-2,21	0,001557	0,331094		
TC0700001818.mm.1	7,21	Fam57b	PSR0700015544.mm.1	-2,74	0,005259	0,377945	Cassette Exon	0,09
TC0700001818.mm.1	7,21	Fam57b	PSR0700015536.mm.1	-2,88	0,034762	0,52661	Cassette Exon	0,31

TC0700001818.mm.1	7,21	Fam57b	PSR0700015537.mm.1	-3,74	0,010029	0,41878	Cassette Exon	0,42
TC0700001818.mm.1	7,21	Fam57b	JUC0700008142.mm.1	-4,05	0,010499	0,422057		
TC0700001818.mm.1	7,21	Fam57b	JUC0700008146.mm.1	-5,46	0,030751	0,514082		
TC0700001818.mm.1	7,21	Fam57b	PSR0700015546.mm.1	-6,4	0,042008	0,546101	Alternative 3' Acce	0,48
TC0700001818.mm.1	7,21	Fam57b	JUC0700008139.mm.1	-7,1	0,017303	0,463271		
TC0700001818.mm.1	7,21	Fam57b	JUC0700008149.mm.1	-7,24	0,017563	0,464258		
TC0700001818.mm.1	7,21	Fam57b	JUC0700008140.mm.1	-7,51	0,035964	0,529457		
TC0700001818.mm.1	7,21	Fam57b	JUC0700008141.mm.1	-7,66	0,022421	0,486764		
TC0700001818.mm.1	7,21	Fam57b	PSR0700015540.mm.1	-9,12	0,003728	0,357586	Cassette Exon	0,55
TC1800000706.mm.1	2,37	Slmo1	PSR1800005176.mm.1	-2,17	0,010691	0,423294	Intron Retention	0,29
TC1800000706.mm.1	2,37	Slmo1	PSR1800005170.mm.1	-2,18	0,00509	0,375607	Cassette Exon	0,17
TC1800000706.mm.1	2,37	Slmo1	PSR1800005178.mm.1	-2,63	0,009608	0,417256	Alternative 5' Donc	0,3
TC1800000706.mm.1	2,37	Slmo1	PSR1800005172.mm.1	-2,85	0,018174	0,467441	Alternative 5' Donc	0,29
TC1800000706.mm.1	2,37	Slmo1	JUC1800002881.mm.1	-2,92	0,025368	0,497403		
TC1800000706.mm.1	2,37	Slmo1	PSR1800005174.mm.1	-3,03	0,007688	0,402552	Intron Retention	0,51
TC1000000593.mm.1	1,69	Rufy2	PSR1000004026.mm.1	-2,17	0,012078	0,43255	Cassette Exon	0,11
TC1000000593.mm.1	1,69	Rufy2	PSR1000004029.mm.1	-2,33	0,048753	0,561437	Cassette Exon	0,12
TC1000000593.mm.1	1,69	Rufy2	JUC1000002212.mm.1	-2,76	0,019715	0,474887		
TC1000000593.mm.1	1,69	Rufy2	PSR1000004063.mm.1	-16,12	0,001055	0,316361	Alternative 5' Donc	0,46
TC1600000460.mm.1	1,41	Rpl35a; Gm14279; Gm102	JUC1600002252.mm.1	-2,17	0,031089	0,515208		
TC1600000460.mm.1	1,41	Rpl35a; Gm14279; Gm102	PSR1600004358.mm.1	-2,53	0,018247	0,467836	Intron Retention	0,36
TC1300001747.mm.1	-1,21	1700018A04Rik	PSR1300010870.mm.1	-2,17	0,02618	0,50003	Cassette Exon	0,16
TC1300001747.mm.1	-1,21	1700018A04Rik	PSR1300010864.mm.1	-2,31	0,020514	0,478264	Intron Retention	0,32
TC0600000527.mm.1	1,51	Cul1	PSR0600003920.mm.1	-2,17	0,008927	0,410972	Cassette Exon	0,09
TC0600000527.mm.1	1,51	Cul1	PSR0600003935.mm.1	-2,21	0,020772	0,479328	Alternative 3' Acce	0,13
TC0600000527.mm.1	1,51	Cul1	JUC0600001963.mm.1	-2,92	0,003342	0,354243		
TC0600000527.mm.1	1,51	Cul1	PSR0600003892.mm.1	-3,12	0,01777	0,465467	Alternative 3' Acce	0,3
TC0100002314.mm.1	1,94	Gls	PSR0100018644.mm.1	-2,17	0,031303	0,516204	Alternative 5' Donc	0,16
TC0100002314.mm.1	1,94	Gls	PSR0100018661.mm.1	-2,19	0,004678	0,370201	Cassette Exon	0,07
TC0100002314.mm.1	1,94	Gls	PSR0100018640.mm.1	-2,23	0,04495	0,55287	Alternative 5' Donc	0,15
TC0100002314.mm.1	1,94	Gls	PSR0100018633.mm.1	-2,27	0,040334	0,541859	Cassette Exon	0,08
TC0100002314.mm.1	1,94	Gls	PSR0100018625.mm.1	-2,29	0,014421	0,448786	Cassette Exon	0,14
TC0100002314.mm.1	1,94	Gls	PSR0100018648.mm.1	-2,6	0,017373	0,463612	Cassette Exon	0,28
TC0100002314.mm.1	1,94	Gls	PSR0100018647.mm.1	-2,84	0,017328	0,463416	Cassette Exon	0,15
TC0100002314.mm.1	1,94	Gls	JUC0100010557.mm.1	-4,02	0,017485	0,463964		
TC1000000358.mm.1	1,42	Wasf1	PSR1000002389.mm.1	-2,17	0,025572	0,497808	Cassette Exon	0,09
TC1000000358.mm.1	1,42	Wasf1	PSR1000002401.mm.1	-2,39	0,02851	0,507386	Alternative 5' Donc	0,22
TC1000000358.mm.1	1,42	Wasf1	PSR1000002384.mm.1	-3,49	0,017229	0,462633	Alternative 3' Acce	0,28
TC0200004521.mm.1	2,19	Myef2; Gm9833	PSR0200038431.mm.1	-2,17	0,046439	0,556547	Cassette Exon	0,17
TC0200004521.mm.1	2,19	Myef2; Gm9833	JUC0200019828.mm.1	-2,17	0,04614	0,555804		
TC0200004521.mm.1	2,19	Myef2; Gm9833	JUC0200019836.mm.1	-2,44	0,038384	0,536356		
TC0200004521.mm.1	2,19	Myef2; Gm9833	JUC0200019837.mm.1	-2,63	0,01389	0,445828		
TC0200004521.mm.1	2,19	Myef2; Gm9833	PSR0200038405.mm.1	-3,53	0,013395	0,442454	Cassette Exon	0,25
TC0200001628.mm.1	1,18	Ccdc34	PSR0200012190.mm.1	-2,17	0,037188	0,533252	Alternative 5' Donc	0,18
TC0200001628.mm.1	1,18	Ccdc34	JUC0200006076.mm.1	-2,29	0,024875	0,49558		
TC1500000064.mm.1	2,23	Nadk2; Nadkd1	PSR1500000588.mm.1	-2,17	0,026967	0,502831	Cassette Exon	0,11
TC1500000064.mm.1	2,23	Nadk2; Nadkd1	PSR1500000595.mm.1	-2,23	0,028695	0,508019	Cassette Exon	0,17
TC1500000064.mm.1	2,23	Nadk2; Nadkd1	JUC1500000389.mm.1	-2,86	0,028525	0,507436		

TC1900000471.mm.1	1,67	Pten	PSR1900004281.mm.1	-2,17	0,016432	0,459007	Alternative 3' Acce	0,16
TC1900000471.mm.1	1,67	Pten	PSR1900004291.mm.1	-2,18	0,00396	0,359922	Alternative 3' Acce	0,17
TC0X00002481.mm.1	1,47	4933407K13Rik	PSR0X00015878.mm.1	-2,17	0,048857	0,561704	Alternative 5' Donc	0,13
TC0X00002481.mm.1	1,47	4933407K13Rik	JUC0X00008084.mm.1	-2,48	0,017778	0,465534		
TC0X00002481.mm.1	1,47	4933407K13Rik	PSR0X00015873.mm.1	-2,56	0,003304	0,354243	Alternative 3' Acce	0,13
TC0X00002481.mm.1	1,47	4933407K13Rik	PSR0X00015869.mm.1	-2,66	0,012903	0,438213	Alternative 3' Acce	0,09
TC0400001708.mm.1	-1,58	Hspg2	PSR0400013742.mm.1	-2,17	0,001404	0,325997	Cassette Exon	0,12
TC1600001022.mm.1	1,31		PSR1600008018.mm.1	-2,17	0,004885	0,372195	Cassette Exon	0,11
TC1600001022.mm.1	1,31		PSR1600008016.mm.1	-2,26	0,029865	0,511132	Cassette Exon	0,12
TC0200004844.mm.1	1,35	Vsx1	PSR0200041265.mm.1	-2,17	0,009729	0,417839	Alternative 5' Donc	0,11
TC1200001474.mm.1	1,18		PSR1200010363.mm.1	-2,17	0,006462	0,390372	Cassette Exon	0,11
TC0700003996.mm.1	-1,13	Gm4070; Gvin1; Gm1775?	PSR0700033364.mm.1	-2,17	0,032949	0,52122	Alternative 3' Acce	0,09
TC1400001702.mm.1	1,22	1700024G13Rik	JUC1400007189.mm.1	-2,17	0,039593	0,539903		
TC0200003025.mm.1	1,12		JUC0200012632.mm.1	-2,17	0,039956	0,540973		
TC1400001805.mm.1	-1,11	Gm3676	JUC1400007557.mm.1	-2,17	0,004231	0,363295		
TC1400000363.mm.1	1,17	Gdf10	JUC1400001936.mm.1	-2,17	0,00878	0,409822		
TC0200005336.mm.1	1,19	Gm14442	JUC0200023416.mm.1	-2,17	0,010894	0,424403		
TC0200005410.mm.1	-1,2	B230312C02Rik	JUC0200023758.mm.1	-2,17	0,014483	0,449345		
TC1200001770.mm.1	1,12	Ralgapa1; Gm23394	JUC1200006788.mm.1	-2,17	0,00201	0,341091		
TC0100000608.mm.1	1,24	Smarcal1	JUC0100002855.mm.1	-2,17	0,048132	0,559938		
TC0100000608.mm.1	1,24	Smarcal1	JUC0100002857.mm.1	-2,2	0,010768	0,423955		
TC1800000977.mm.1	1,38	Esco1	JUC1800004021.mm.1	-2,17	0,036834	0,532221		
TC1800001151.mm.1	-1,06	4933408B17Rik	JUC1800004694.mm.1	-2,17	0,033502	0,522992		
TC1900000211.mm.1	-1,17	Slc15a3	JUC1900001381.mm.1	-2,17	0,038258	0,535953		
TC1500001782.mm.1	1,23	Cpsf1	JUC1500007824.mm.1	-2,17	0,015369	0,453849		
TC1600000038.mm.1	1,28	Mgrn1	JUC1600000158.mm.1	-2,17	0,000249	0,28803		
TC0200000550.mm.1	-1,01	Hmcn2	JUC0200002322.mm.1	-2,17	0,043488	0,549901		
TC0200000550.mm.1	-1,01	Hmcn2	JUC0200002314.mm.1	-3,56	0,014866	0,451641		
TC0800001107.mm.1	1,1		JUC0800004559.mm.1	-2,17	0,040066	0,541386		
TC0500002120.mm.1	1,08	Gm5862	JUC0500010528.mm.1	-2,17	0,015314	0,453561		
TC0800000854.mm.1	1,38	Nr3c2	JUC0800003354.mm.1	-2,17	0,004305	0,364136		
TC0900001600.mm.1	1,1	Myrip	JUC0900007543.mm.1	-2,17	0,041128	0,543971		
TC0500001342.mm.1	1,01	Ddx54	JUC0500006411.mm.1	-2,17	0,001056	0,316361		
TC0700003783.mm.1	1,52	2210018M11Rik	JUC0700016873.mm.1	-2,17	0,020327	0,477245		
TC0700003783.mm.1	1,52	2210018M11Rik	JUC0700016854.mm.1	-2,26	0,013113	0,439759		
TC0300002716.mm.1	1,5	Ahcyl1	JUC0300011027.mm.1	-2,17	0,046603	0,556781		
TC0X00003428.mm.1	-1,32	Xlr5c	JUC0X00007615.mm.1	-2,17	0,018085	0,46716		
TC0200001204.mm.1	4,58	Fam171b	JUC0200004934.mm.1	-2,18	0,002631	0,349612		
TC0200001204.mm.1	4,58	Fam171b	PSR0200009647.mm.1	-2,77	0,015477	0,45435	Cassette Exon	0,2
TC0200001204.mm.1	4,58	Fam171b	PSR0200009648.mm.1	-3,41	0,006652	0,393165	Cassette Exon	0,23
TC0200001204.mm.1	4,58	Fam171b	PSR0200009651.mm.1	-6,03	0,000769	0,308403	Alternative 3' Acce	0,49
TC0900001954.mm.1	6,57	Ntm	PSR0900016404.mm.1	-2,18	0,044616	0,552129	Cassette Exon	0,24
TC0900001954.mm.1	6,57	Ntm	PSR0900016412.mm.1	-2,71	0,012685	0,436333	Alternative 3' Acce	0,26
TC0900001954.mm.1	6,57	Ntm	PSR0900016429.mm.1	-3,3	0,026441	0,501198		
TC0900001954.mm.1	6,57	Ntm	JUC0900009288.mm.1	-3,65	0,021613	0,483425		
TC0900001954.mm.1	6,57	Ntm	PSR0900016428.mm.1	-3,94	0,037185	0,53323	Alternative 3' Acce	0,1
TC0900001954.mm.1	6,57	Ntm	PSR0900016400.mm.1	-3,98	0,03835	0,53622	Alternative 3' Acce	0,42
TC0900001954.mm.1	6,57	Ntm	PSR0900016405.mm.1	-4,02	0,025034	0,496091	Cassette Exon	0,37

TC0900001954.mm.1	6,57	Ntm	PSR0900016419.mm.1	-4,08	0,009146	0,413447	Cassette Exon	0,41
TC0900001954.mm.1	6,57	Ntm	PSR0900016420.mm.1	-4,61	0,018067	0,46716	Alternative 3' Acce	0,35
TC0900001954.mm.1	6,57	Ntm	PSR0900016427.mm.1	-4,78	0,020204	0,477145	Cassette Exon	0,14
TC0900001954.mm.1	6,57	Ntm	PSR0900016406.mm.1	-5,05	0,008653	0,408463	Cassette Exon	0,24
TC0900001954.mm.1	6,57	Ntm	PSR0900016417.mm.1	-5,07	0,008059	0,40448	Cassette Exon	0,36
TC0900001954.mm.1	6,57	Ntm	JUC0900009292.mm.1	-5,11	0,047373	0,558302		
TC0900001954.mm.1	6,57	Ntm	JUC0900009298.mm.1	-5,58	0,008575	0,40821		
TC0900001954.mm.1	6,57	Ntm	PSR0900016416.mm.1	-5,99	0,012114	0,432704	Cassette Exon	0,24
TC0900001954.mm.1	6,57	Ntm	PSR0900016433.mm.1	-6,13	0,006973	0,395722	Alternative 5' Donc	0,11
TC0900001954.mm.1	6,57	Ntm	PSR0900016426.mm.1	-6,78	0,017346	0,46353	Cassette Exon	0,28
TC0900001954.mm.1	6,57	Ntm	JUC0900009296.mm.1	-7,11	0,009985	0,418456		
TC0900001954.mm.1	6,57	Ntm	PSR0900016407.mm.1	-7,53	0,009501	0,416578	Alternative 3' Acce	0,46
TC0100000682.mm.1	2,02	Utp14b; Acsl3	PSR0100005849.mm.1	-2,18	0,042742	0,548097	Cassette Exon	0,08
TC0100000682.mm.1	2,02	Utp14b; Acsl3	PSR0100005860.mm.1	-2,18	0,008925	0,410972	Cassette Exon	0,07
TC0100000682.mm.1	2,02	Utp14b; Acsl3	PSR0100005869.mm.1	-3,7	0,014551	0,44963	Alternative 5' Donc	0,44
TC1700000064.mm.1	-1,13	Sytl3; LOC101055745; LO(	PSR1700000453.mm.1	-2,18	0,021354	0,48184	Intron Retention	0,29
TC0400004009.mm.1	1,69	Clcn6	PSR0400033140.mm.1	-2,18	0,037943	0,535302	Cassette Exon	0,11
TC0400004009.mm.1	1,69	Clcn6	PSR0400033134.mm.1	-3,27	0,009592	0,417183	Cassette Exon	0,27
TC0600003475.mm.1	1,84	Dennd5b	PSR0600027502.mm.1	-2,18	0,011384	0,427549	Alternative 3' Acce	0,24
TC0600003475.mm.1	1,84	Dennd5b	PSR0600027520.mm.1	-2,38	0,009635	0,417354	Alternative 5' Donc	0,18
TC0600003475.mm.1	1,84	Dennd5b	JUC0600014450.mm.1	-2,67	0,038306	0,536106		
TC0600003475.mm.1	1,84	Dennd5b	PSR0600027505.mm.1	-2,8	0,002771	0,350656	Alternative 5' Donc	0,26
TC0600003475.mm.1	1,84	Dennd5b	PSR0600027501.mm.1	-2,86	0,034341	0,525546	Alternative 3' Acce	0,24
TC0600003475.mm.1	1,84	Dennd5b	JUC0600014432.mm.1	-3,2	0,02232	0,486213		
TC0900002802.mm.1	2,14	9430037G07Rik; Gm2301	JUC0900013035.mm.1	-2,18	0,028191	0,506368		
TC0900002802.mm.1	2,14	9430037G07Rik; Gm2301	JUC0900013033.mm.1	-2,4	0,016769	0,460504		
TC0900002802.mm.1	2,14	9430037G07Rik; Gm2301	PSR0900023286.mm.1	-3,14	0,013283	0,441719	Cassette Exon	0,25
TC1600000193.mm.1	1,1	Pkp2	PSR1600001504.mm.1	-2,18	0,000911	0,313363	Cassette Exon	0,13
TC1600000193.mm.1	1,1	Pkp2	PSR1600001494.mm.1	-2,77	0,030027	0,511736	Alternative 5' Donc	0,23
TC0400002016.mm.1	2,26	Acot7	PSR0400016507.mm.1	-2,18	0,024484	0,493992	Cassette Exon	0,15
TC0400002016.mm.1	2,26	Acot7	PSR0400016510.mm.1	-2,2	0,028578	0,507603	Cassette Exon	0,11
TC0400002016.mm.1	2,26	Acot7	PSR0400016515.mm.1	-2,74	0,02646	0,501252		
TC0400002016.mm.1	2,26	Acot7	PSR0400016511.mm.1	-2,94	0,005852	0,383738	Alternative 3' Acce	0,21
TC0400002016.mm.1	2,26	Acot7	PSR0400016509.mm.1	-3,02	0,01486	0,451623	Cassette Exon	0,19
TC0400002016.mm.1	2,26	Acot7	PSR0400016513.mm.1	-3,6	0,017114	0,46232	Alternative 3' Acce	0,14
TC0400002016.mm.1	2,26	Acot7	PSR0400016516.mm.1	-3,82	0,006728	0,393343	Alternative 5' Donc	0,13
TC0600000627.mm.1	1,11	Evx1	PSR0600004808.mm.1	-2,18	0,022594	0,487325	Alternative 5' Donc	0,2
TC1100001250.mm.1	2,51	Rasl10b	PSR1100011750.mm.1	-2,18	0,008487	0,406895	Cassette Exon	0,17
TC1100001250.mm.1	2,51	Rasl10b	PSR1100011745.mm.1	-2,52	0,029843	0,511132	Cassette Exon	0,11
TC1100001250.mm.1	2,51	Rasl10b	PSR1100011743.mm.1	-2,68	0,007778	0,402902	Cassette Exon	0,16
TC1100001250.mm.1	2,51	Rasl10b	PSR1100011744.mm.1	-2,75	0,013459	0,442805	Cassette Exon	0,19
TC1100001250.mm.1	2,51	Rasl10b	PSR1100011746.mm.1	-3,01	0,001374	0,32572	Cassette Exon	0,14
TC1100001250.mm.1	2,51	Rasl10b	JUC1100006148.mm.1	-4,47	0,003335	0,354243		
TC1400002493.mm.1	-1,26	Lect1	PSR1400019165.mm.1	-2,18	0,041651	0,545299	Alternative 3' Acce	0,19
TC0500002037.mm.1	1,01	Orc5; Gm15421	PSR0500018559.mm.1	-2,18	0,027763	0,505135	Alternative 3' Acce	0,17
TC0700000903.mm.1	1,12	Htatip2	PSR0700008127.mm.1	-2,18	0,037427	0,533831	Alternative 3' Acce	0,17
TC0500003746.mm.1	1,37	1810059H22Rik	PSR0500033484.mm.1	-2,18	0,014783	0,45081	Cassette Exon	0,15
TC1100000918.mm.1	1,7	Fxr2	PSR1100007793.mm.1	-2,18	0,042009	0,546101	Alternative 3' Acce	0,15

TC1500001108.mm.1	1,67	Gm4544	PSR1500008943.mm.1	-2,18	0,026857	0,502454 Alternative 3' Acce	0,13
TC0800002733.mm.1	1,26	1700121C10Rik	PSR0800020935.mm.1	-2,18	0,032983	0,521351 Cassette Exon	0,12
TC1900000024.mm.1	1,21	Doc2g; Nudt8	PSR1900000278.mm.1	-2,18	0,001429	0,327447 Alternative 3' Acce	0,12
TC0600000667.mm.1	1,13	Adcyap1r1	PSR0600005174.mm.1	-2,18	0,002566	0,349501 Cassette Exon	0,11
TC1100003144.mm.1	-1,28	Smtnl2	PSR1100029155.mm.1	-2,18	0,038432	0,536405 Cassette Exon	0,11
TC0700000567.mm.1	1,47	Igflr1; U2af1l4	PSR0700005070.mm.1	-2,18	0,04795	0,559549 Cassette Exon	0,1
TC1500001987.mm.1	1,76	1810041L15Rik	PSR1500015781.mm.1	-2,18	0,025394	0,497403 Cassette Exon	0,09
TC0100003057.mm.1	-1,08	Ikbke	PSR0100024515.mm.1	-2,18	0,045925	0,555257 Cassette Exon	0,08
TC1400002242.mm.1	1,06	Hmbox1	JUC1400009484.mm.1	-2,18	0,006121	0,387322	
TC1200000874.mm.1	1,12	Samd15	JUC1200003610.mm.1	-2,18	0,047288	0,558242	
TC0200005411.mm.1	-1,1		JUC0200023764.mm.1	-2,18	0,02089	0,479646	
TC0100002306.mm.1	-1,02	Gm17767	JUC0100010492.mm.1	-2,18	0,037255	0,53345	
TC1700001783.mm.1	-1,01	Mtch1	JUC1700008873.mm.1	-2,18	0,032922	0,521149	
TC1700001783.mm.1	-1,01	Mtch1	JUC1700008875.mm.1	-2,91	0,011474	0,428022	
TC0100001109.mm.1	1,62		JUC0100005210.mm.1	-2,18	0,032953	0,52122	
TC1800001153.mm.1	1,93	Brd8	JUC1800004720.mm.1	-2,18	0,047363	0,558302	
TC0200001982.mm.1	1,33	Vps16	JUC0200007916.mm.1	-2,18	0,033684	0,523549	
TC0200001982.mm.1	1,33	Vps16	JUC0200007903.mm.1	-2,2	0,048555	0,561008	
TC0200001982.mm.1	1,33	Vps16	JUC0200007920.mm.1	-2,58	0,035619	0,528956	
TC1600002141.mm.1	1,03	Lca5l	JUC1600008518.mm.1	-2,18	0,004224	0,363295	
TC0900000865.mm.1	-1,24	Dapk2	JUC0900003573.mm.1	-2,18	0,016898	0,460797	
TC0600003001.mm.1	1,37	Zfp248	JUC0600011995.mm.1	-2,18	0,021858	0,484635	
TC0600003001.mm.1	1,37	Zfp248	JUC0600011988.mm.1	-2,2	0,044467	0,551693	
TC0600003001.mm.1	1,37	Zfp248	JUC0600011996.mm.1	-2,23	0,018818	0,470836	
TC0500003603.mm.1	-1,09	Gm5565	JUC0500018009.mm.1	-2,18	0,000569	0,304044	
TC0700001557.mm.1	1,42	Ilk	JUC0700006749.mm.1	-2,18	0,025834	0,498598	
TC1000002240.mm.1	-1,15	Adamts14	JUC1000008624.mm.1	-2,18	0,037733	0,534632	
TC1000002252.mm.1	2,2	2010107G23Rik	JUC1000008747.mm.1	-2,18	0,005739	0,382993	
TC0X00002751.mm.1	1,22	Phka1	JUC0X00008781.mm.1	-2,18	0,041121	0,543958	
TC0400004218.mm.1	8,47	Ccdc24; Gm17114	PSR0400027115.mm.1	-2,19	0,003784	0,357586 Intron Retention	0,25
TC0400004218.mm.1	8,47	Ccdc24; Gm17114	JUC0400014106.mm.1	-2,2	0,00021	0,28803	
TC0400004218.mm.1	8,47	Ccdc24; Gm17114	PSR0400027129.mm.1	-2,63	0,032103	0,518577 Cassette Exon	0,29
TC0400004218.mm.1	8,47	Ccdc24; Gm17114	PSR0400027111.mm.1	-3,58	0,005376	0,378747 Alternative 3' Acce	0,45
TC0400004218.mm.1	8,47	Ccdc24; Gm17114	PSR0400027133.mm.1	-3,67	0,026452	0,501251 Cassette Exon	0,28
TC0400004218.mm.1	8,47	Ccdc24; Gm17114	JUC0400014103.mm.1	-3,72	0,031422	0,51654	
TC0400004218.mm.1	8,47	Ccdc24; Gm17114	PSR0400027109.mm.1	-5,08	0,012502	0,435429 Alternative 3' Acce	0,45
TC0400004218.mm.1	8,47	Ccdc24; Gm17114	PSR0400027127.mm.1	-5,6	0,033033	0,521499 Alternative 5' Donc	0,16
TC0400004218.mm.1	8,47	Ccdc24; Gm17114	PSR0400027104.mm.1	-5,68	0,047298	0,558249 Alternative 3' Acce	0,45
TC0400004218.mm.1	8,47	Ccdc24; Gm17114	JUC0400014118.mm.1	-6,28	0,002291	0,346076	
TC0400004218.mm.1	8,47	Ccdc24; Gm17114	PSR0400027130.mm.1	-6,32	0,028476	0,507299 Cassette Exon	0,29
TC0400004218.mm.1	8,47	Ccdc24; Gm17114	JUC0400014120.mm.1	-6,66	0,021628	0,483548	
TC0400004218.mm.1	8,47	Ccdc24; Gm17114	PSR0400027101.mm.1	-8,66	0,022482	0,487114 Cassette Exon	0,4
TC0400004218.mm.1	8,47	Ccdc24; Gm17114	JUC0400014109.mm.1	-8,89	0,028558	0,507497	
TC0400004218.mm.1	8,47	Ccdc24; Gm17114	PSR0400027134.mm.1	-9,39	0,008002	0,403872 Cassette Exon	0,24
TC0400004218.mm.1	8,47	Ccdc24; Gm17114	PSR0400027131.mm.1	-9,59	0,00349	0,355638 Cassette Exon	0,29
TC0400004218.mm.1	8,47	Ccdc24; Gm17114	JUC0400014110.mm.1	-10,1	0,029946	0,511499	
TC0100003042.mm.1	4,1	Pfkfb2; C4bp-ps1	PSR0100024408.mm.1	-2,19	0,045636	0,554672	
TC0100003042.mm.1	4,1	Pfkfb2; C4bp-ps1	PSR0100024404.mm.1	-2,41	0,013131	0,440038 Cassette Exon	0,13

TC0100003042.mm.1	4,1	Pfkfb2; C4bp-ps1	PSR0100024409.mm.1	-2,73	0,037384	0,533687	Alternative 3' Acce	0,07
TC0100003042.mm.1	4,1	Pfkfb2; C4bp-ps1	JUC0100013854.mm.1	-3,22	0,03266	0,520431		
TC0100003042.mm.1	4,1	Pfkfb2; C4bp-ps1	PSR0100024394.mm.1	-3,41	0,035515	0,528764	Cassette Exon	0,22
TC0100003042.mm.1	4,1	Pfkfb2; C4bp-ps1	PSR0100024398.mm.1	-3,54	0,006297	0,389033	Cassette Exon	0,26
TC0100003042.mm.1	4,1	Pfkfb2; C4bp-ps1	PSR0100024429.mm.1	-3,7	0,011752	0,430017	Alternative 5' Donc	0,41
TC0100003042.mm.1	4,1	Pfkfb2; C4bp-ps1	PSR0100024395.mm.1	-3,8	0,01679	0,460623	Cassette Exon	0,22
TC0100003042.mm.1	4,1	Pfkfb2; C4bp-ps1	JUC0100013851.mm.1	-3,9	0,02726	0,503682		
TC0100003042.mm.1	4,1	Pfkfb2; C4bp-ps1	PSR0100024430.mm.1	-4,02	0,008392	0,406601	Cassette Exon	0,41
TC0100003042.mm.1	4,1	Pfkfb2; C4bp-ps1	JUC0100013842.mm.1	-4,52	0,003024	0,353502		
TC0100003042.mm.1	4,1	Pfkfb2; C4bp-ps1	JUC0100013833.mm.1	-4,65	0,03037	0,512923		
TC0100003042.mm.1	4,1	Pfkfb2; C4bp-ps1	PSR0100024399.mm.1	-4,94	0,014257	0,447947	Cassette Exon	0,26
TC0100003042.mm.1	4,1	Pfkfb2; C4bp-ps1	PSR0100024431.mm.1	-4,97	0,020591	0,478531	Cassette Exon	0,28
TC0100003042.mm.1	4,1	Pfkfb2; C4bp-ps1	PSR0100024425.mm.1	-5,09	0,04696	0,557563		
TC0500000997.mm.1	2,4	Arhgap24	JUC0500004619.mm.1	-2,19	0,037874	0,534908		
TC0500000997.mm.1	2,4	Arhgap24	PSR0500008437.mm.1	-2,22	0,000283	0,28803	Alternative 5' Donc	0,19
TC0500000997.mm.1	2,4	Arhgap24	PSR0500008417.mm.1	-2,35	0,021983	0,485125	Cassette Exon	0,01
TC0500000997.mm.1	2,4	Arhgap24	PSR0500008429.mm.1	-2,37	0,000443	0,297771	Cassette Exon	0,12
TC0500000997.mm.1	2,4	Arhgap24	PSR0500008422.mm.1	-2,64	0,013847	0,445385	Cassette Exon	0,14
TC0500000997.mm.1	2,4	Arhgap24	PSR0500008426.mm.1	-2,67	0,002309	0,346468	Cassette Exon	0,24
TC0500000997.mm.1	2,4	Arhgap24	PSR0500008419.mm.1	-2,78	0,002791	0,351449		
TC0500000997.mm.1	2,4	Arhgap24	JUC0500004609.mm.1	-2,87	0,005366	0,378627		
TC0500000997.mm.1	2,4	Arhgap24	PSR0500008425.mm.1	-2,94	0,000875	0,311909	Cassette Exon	0,03
TC0500000997.mm.1	2,4	Arhgap24	PSR0500008415.mm.1	-3,08	0,000042	0,24627	Alternative 3' Acce	0,12
TC0500000997.mm.1	2,4	Arhgap24	JUC0500004612.mm.1	-3,14	0,012303	0,434371		
TC0500000997.mm.1	2,4	Arhgap24	JUC0500004623.mm.1	-3,2	0,042447	0,547627		
TC0500000997.mm.1	2,4	Arhgap24	PSR0500008414.mm.1	-3,73	0,000259	0,28803	Cassette Exon	0,34
TC0500000997.mm.1	2,4	Arhgap24	JUC0500004620.mm.1	-3,89	0,010239	0,420146		
TC0500000997.mm.1	2,4	Arhgap24	PSR0500008423.mm.1	-4,76	0,011725	0,429969	Alternative 3' Acce	0,17
TC0500000997.mm.1	2,4	Arhgap24	JUC0500004621.mm.1	-6,89	0,002673	0,349612		
TC1000000693.mm.1	1,64	Zwint	PSR1000004828.mm.1	-2,19	0,036626	0,53155	Intron Retention	0,3
TC1000000693.mm.1	1,64	Zwint	PSR1000004838.mm.1	-2,66	0,016796	0,460623	Alternative 3' Acce	0,22
TC1000000693.mm.1	1,64	Zwint	JUC1000002616.mm.1	-2,72	0,027429	0,503961		
TC1000000693.mm.1	1,64	Zwint	PSR1000004837.mm.1	-3,84	0,001141	0,317344	Alternative 3' Acce	0,22
TC1000000693.mm.1	1,64	Zwint	JUC1000002615.mm.1	-5,4	0,037554	0,534053		
TC1100003990.mm.1	2,81	Slc16a6; Gm25540	PSR1100037341.mm.1	-2,19	0,013551	0,44358		
TC1100003990.mm.1	2,81	Slc16a6; Gm25540	JUC1100019501.mm.1	-2,26	0,008385	0,4065		
TC1100003990.mm.1	2,81	Slc16a6; Gm25540	PSR1100037339.mm.1	-2,45	0,007888	0,403771		
TC1100003990.mm.1	2,81	Slc16a6; Gm25540	PSR1100037343.mm.1	-2,54	0,022092	0,485453	Cassette Exon	0,3
TC1100003990.mm.1	2,81	Slc16a6; Gm25540	PSR1100037340.mm.1	-2,6	0,009412	0,415853		
TC1100003990.mm.1	2,81	Slc16a6; Gm25540	JUC1100019504.mm.1	-2,97	0,025107	0,496358		
TC1100003990.mm.1	2,81	Slc16a6; Gm25540	JUC1100019509.mm.1	-4,09	0,003514	0,355749		
TC0800001875.mm.1	2,62	Ap3m2	JUC0800008092.mm.1	-2,19	0,01335	0,442045		
TC0800001875.mm.1	2,62	Ap3m2	JUC0800008098.mm.1	-2,29	0,000793	0,310881		
TC0800001875.mm.1	2,62	Ap3m2	PSR0800014874.mm.1	-3,35	0,004293	0,364023	Cassette Exon	0,28
TC1400002868.mm.1	1,6	Lats2	PSR1400016564.mm.1	-2,19	0,01216	0,432923	Cassette Exon	0,08
TC1400002868.mm.1	1,6	Lats2	PSR1400016577.mm.1	-3,58	0,029947	0,511499	Cassette Exon	0,28
TC0800002802.mm.1	1,17	Dync1li2	PSR0800021425.mm.1	-2,19	0,028592	0,507685	Cassette Exon	0,27
TC0800002802.mm.1	1,17	Dync1li2	JUC0800011693.mm.1	-5,34	0,010219	0,419955		

TC0X00000709.mm.1	2,22	Gdi1	PSR0X00004823.mm.1	-2,19	0,01684	0,460729	Cassette Exon	0,17
TC0X00000709.mm.1	2,22	Gdi1	PSR0X00004826.mm.1	-2,32	0,018018	0,466939	Alternative 5' Donc	0,21
TC0X00000709.mm.1	2,22	Gdi1	PSR0X00004816.mm.1	-2,33	0,012712	0,436515	Alternative 3' Acce	0,16
TC0X00000709.mm.1	2,22	Gdi1	JUC0X00002426.mm.1	-2,89	0,045663	0,55477		
TC0X00000709.mm.1	2,22	Gdi1	PSR0X00004822.mm.1	-2,97	0,004336	0,364898	Cassette Exon	0,2
TC0700004474.mm.1	2,06	Bnip3	PSR0700037521.mm.1	-2,19	0,012012	0,432286	Alternative 3' Acce	0,07
TC0700004474.mm.1	2,06	Bnip3	JUC0700019768.mm.1	-2,23	0,017758	0,465425		
TC0700004474.mm.1	2,06	Bnip3	PSR0700037513.mm.1	-2,24	0,0262	0,50003	Alternative 3' Acce	0,2
TC0200003140.mm.1	1,29	Ndor1	PSR0200025823.mm.1	-2,19	0,04613	0,555804	Alternative 3' Acce	0,19
TC0200005127.mm.1	-1,1	Wfdc3; LOC101055924	PSR0200043714.mm.1	-2,19	0,000537	0,304044	Alternative 3' Acce	0,19
TC1800001741.mm.1	1,05	Pcdhgb1; Pcdhgb2; Pcdhgb	JUC1800001604.mm.1	-2,19	0,013826	0,445196		
TC1800001741.mm.1	1,05	Pcdhgb1; Pcdhgb2; Pcdhgb	JUC1800001592.mm.1	-2,26	0,007561	0,401384		
TC1800001741.mm.1	1,05	Pcdhgb1; Pcdhgb2; Pcdhgb	JUC1800001587.mm.1	-2,32	0,011946	0,431753		
TC1800001741.mm.1	1,05	Pcdhgb1; Pcdhgb2; Pcdhgb	JUC1800001603.mm.1	-2,42	0,017663	0,464653		
TC1800001741.mm.1	1,05	Pcdhgb1; Pcdhgb2; Pcdhgb	JUC1800001605.mm.1	-2,42	0,024244	0,493294		
TC1800001741.mm.1	1,05	Pcdhgb1; Pcdhgb2; Pcdhgb	JUC1800001595.mm.1	-2,56	0,000892	0,313363		
TC1800001741.mm.1	1,05	Pcdhgb1; Pcdhgb2; Pcdhgb	PSR1800002760.mm.1	-2,58	0,049946	0,563958	Cassette Exon	0,19
TC1800000599.mm.1	-1,23	Chsy3	PSR1800004284.mm.1	-2,19	0,038594	0,536862	Alternative 5' Donc	0,19
TC1800000599.mm.1	-1,23	Chsy3	JUC1800002402.mm.1	-2,21	0,042578	0,548047		
TC0400003266.mm.1	2,12	Gm12750	PSR0400025992.mm.1	-2,19	0,048001	0,559574	Alternative 3' Acce	0,17
TC0100000021.mm.1	-1,01	Rb1cc1	PSR0100000159.mm.1	-2,19	0,015638	0,455193	Alternative 5' Donc	0,13
TC0100003827.mm.1	-1,09	Hhat	JUC0100017753.mm.1	-2,19	0,030972	0,514659		
TC0100003827.mm.1	-1,09	Hhat	PSR0100031294.mm.1	-2,27	0,021056	0,480262	Cassette Exon	0,12
TC0700003680.mm.1	-1,48	Vmn2r74	PSR0700031191.mm.1	-2,19	0,04076	0,543082	Cassette Exon	0,12
TC0500003203.mm.1	1,23	Ccdc63	PSR0500028617.mm.1	-2,19	0,005571	0,381015	Cassette Exon	0,11
TC0500003241.mm.1	1,43	Rsrc2	PSR0500029188.mm.1	-2,19	0,005483	0,379939	Cassette Exon	0,11
TC0500003241.mm.1	1,43	Rsrc2	JUC0500015900.mm.1	-2,75	0,0441	0,550916		
TC0800003240.mm.1	1,63	6820431F20Rik; 2610005I	PSR0800014451.mm.1	-2,19	0,040935	0,543423	Cassette Exon	0,11
TC0800003240.mm.1	1,63	6820431F20Rik; 2610005I	JUC0800007880.mm.1	-4,07	0,031829	0,517841		
TC0900001168.mm.1	-1,01	Plscr5	PSR0900009186.mm.1	-2,19	0,004154	0,3623	Cassette Exon	0,11
TC0X00000643.mm.1	2,61	Mamld1	PSR0X00003968.mm.1	-2,19	0,017562	0,464258	Cassette Exon	0,11
TC1400002142.mm.1	1,07	Cryl1	JUC1400008987.mm.1	-2,19	0,038997	0,537976		
TC1400002142.mm.1	1,07	Cryl1	JUC1400008988.mm.1	-2,37	0,020676	0,478885		
TC0300003213.mm.1	1,33	Clk2	JUC0300002941.mm.1	-2,19	0,003275	0,354243		
TC1200002088.mm.1	1,05	Rps6kl1	JUC1200007961.mm.1	-2,19	0,019985	0,476227		
TC1100004067.mm.1	1,01	Hid1	JUC1100020041.mm.1	-2,19	0,044516	0,551823		
TC1200000462.mm.1	1,42	Srp54c	JUC1200001796.mm.1	-2,19	0,042612	0,548065		
TC1200000462.mm.1	1,42	Srp54c	JUC1200001799.mm.1	-3,08	0,047416	0,558302		
TC0200001038.mm.1	1,45	Metap1d	JUC0200004316.mm.1	-2,19	0,001001	0,316361		
TC0200001038.mm.1	1,45	Metap1d	JUC0200004309.mm.1	-2,57	0,017003	0,461566		
TC0200000416.mm.1	1,08	Fut7	JUC0200001112.mm.1	-2,19	0,034269	0,525304		
TC0500000709.mm.1	-1,22	Pdgfra; Mir7025; mmu-mi	JUC0500003450.mm.1	-2,19	0,021333	0,481727		
TC0600001441.mm.1	-1,11	Zfp384	JUC0600006135.mm.1	-2,19	0,041192	0,544121		
TC0700001702.mm.1	1,32	9030624J02Rik	JUC0700007337.mm.1	-2,19	0,010598	0,422764		
TC0700001749.mm.1	-1,03	Ubfd1	JUC0700007805.mm.1	-2,19	0,015936	0,45678		
TC0700002424.mm.1	1,17	Ccdc9	JUC0700011380.mm.1	-2,19	0,010136	0,419217		
TC1100002948.mm.1	1,64	Zfp286	JUC1100014013.mm.1	-2,19	0,025439	0,497549		
TC1100002948.mm.1	1,64	Zfp286	JUC1100014016.mm.1	-2,25	0,02997	0,511499		

TC0900003250.mm.1	1,05	Csrnp1	JUC0900015431.mm.1	-2,19	0,034605	0,52602		
TC0X00000976.mm.1	-1,11		JUC0X00003219.mm.1	-2,19	0,005948	0,385276		
TC1000001149.mm.1	1,04	Tmcc3; A230066D03Rik	JUC1000004706.mm.1	-2,19	0,029363	0,509995		
TC0800002145.mm.1	2,08	Fam149a	PSR0800016514.mm.1	-2,2	0,018166	0,467441	Cassette Exon	0,06
TC0800002145.mm.1	2,08	Fam149a	PSR0800016504.mm.1	-2,3	0,030191	0,512365	Cassette Exon	0,21
TC0800002145.mm.1	2,08	Fam149a	PSR0800016517.mm.1	-2,75	0,016478	0,459352	Alternative 5' Donc	0,28
TC0800002145.mm.1	2,08	Fam149a	JUC0800008992.mm.1	-2,97	0,016091	0,457532		
TC1100003265.mm.1	1,27	Ankrd13b	PSR1100030075.mm.1	-2,2	0,001577	0,331726	Intron Retention	0,28
TC0100001464.mm.1	1,22	Gas5; Snord47; Gm26224;	PSR0100012042.mm.1	-2,2	0,025299	0,497227	Alternative 5' Donc	0,2
TC1400000024.mm.1	-1	Gm3159	PSR1400000373.mm.1	-2,2	0,000483	0,298999	Alternative 5' Donc	0,19
TC1600001427.mm.1	1,84	Fgf12; B230343J05Rik	PSR1600011790.mm.1	-2,2	0,00123	0,322251	Alternative 3' Acce	0,09
TC1600001427.mm.1	1,84	Fgf12; B230343J05Rik	PSR1600011807.mm.1	-2,22	0,013815	0,445064	Alternative 3' Acce	0,19
TC1600001427.mm.1	1,84	Fgf12; B230343J05Rik	PSR1600011813.mm.1	-2,24	0,018271	0,468043	Alternative 5' Donc	0,15
TC1600001427.mm.1	1,84	Fgf12; B230343J05Rik	PSR1600011804.mm.1	-2,24	0,022575	0,487325	Cassette Exon	0,04
TC1600001427.mm.1	1,84	Fgf12; B230343J05Rik	PSR1600011802.mm.1	-2,35	0,023238	0,490032	Alternative 3' Acce	0,18
TC1600001427.mm.1	1,84	Fgf12; B230343J05Rik	JUC1600006144.mm.1	-2,56	0,033176	0,522044		
TC1600001427.mm.1	1,84	Fgf12; B230343J05Rik	PSR1600011806.mm.1	-2,63	0,005029	0,374309	Cassette Exon	0,05
TC0300001858.mm.1	1,13	Trpc3	PSR0300014399.mm.1	-2,2	0,036338	0,530547	Alternative 3' Acce	0,18
TC0900000463.mm.1	1,22	1700063D05Rik	PSR0900003081.mm.1	-2,2	0,049653	0,563521	Alternative 3' Acce	0,18
TC0100002070.mm.1	1,54	1110058L19Rik	PSR0100016837.mm.1	-2,2	0,045666	0,554786	Cassette Exon	0,17
TC0100002070.mm.1	1,54	1110058L19Rik	PSR0100016838.mm.1	-2,23	0,015219	0,452815	Cassette Exon	0,12
TC1300002594.mm.1	-1,19	Nln	PSR1300017639.mm.1	-2,2	0,004102	0,36228	Alternative 3' Acce	0,15
TC1100000037.mm.1	1,09	Sf3a1	PSR1100000465.mm.1	-2,2	0,024091	0,493095	Cassette Exon	0,14
TC1900001577.mm.1	1,95		PSR1900013966.mm.1	-2,2	0,011188	0,426444	Cassette Exon	0,14
TC0X00002524.mm.1	-1,13	Gm8787	PSR0X00016091.mm.1	-2,2	0,012417	0,435195	Cassette Exon	0,13
TC1100000651.mm.1	1,96	Gm12239	PSR1100005265.mm.1	-2,2	0,026062	0,499619	Cassette Exon	0,13
TC1200000073.mm.1	-1,25	Matn3	PSR1200000778.mm.1	-2,2	0,039152	0,538613	Cassette Exon	0,13
TC0700003526.mm.1	1,91	Sv2b	PSR0700029852.mm.1	-2,2	0,022409	0,486711	Cassette Exon	0,12
TC0X00000596.mm.1	1,79	Slitrk2	PSR0X00003788.mm.1	-2,2	0,000208	0,28803	Cassette Exon	0,12
TC1900000931.mm.1	1,52	Rbm4; Rbm14; Gm21992;	PSR1900008193.mm.1	-2,2	0,046063	0,555645	Cassette Exon	0,12
TC1900000931.mm.1	1,52	Rbm4; Rbm14; Gm21992;	PSR1900008192.mm.1	-2,66	0,015503	0,454456	Cassette Exon	0,08
TC1100001241.mm.1	1,01	Slfn4	PSR1100011646.mm.1	-2,2	0,003852	0,358861	Cassette Exon	0,11
TC1100002134.mm.1	-1,08	Znrf3	PSR1100020807.mm.1	-2,2	0,013809	0,445064	Cassette Exon	0,11
TC0800002950.mm.1	1,05	Ldhd	PSR0800022782.mm.1	-2,2	0,040846	0,543283	Alternative 3' Acce	0,06
TC0900003102.mm.1	1,29	Dhx30	PSR0900026233.mm.1	-2,2	0,00889	0,410717	Cassette Exon	0,06
TC0200002453.mm.1	1,07	Plcg1	JUC0200010162.mm.1	-2,2	0,040097	0,541437		
TC0200002453.mm.1	1,07	Plcg1	JUC0200010147.mm.1	-2,34	0,009977	0,418456		
TC0200005162.mm.1	-1,07	Gm11464	JUC0200022773.mm.1	-2,2	0,012208	0,433286		
TC1200001482.mm.1	1,17	3110053B16Rik	JUC1200005745.mm.1	-2,2	0,01146	0,427962		
TC0300000139.mm.1	1,46	Hltf	JUC0300000294.mm.1	-2,2	0,023883	0,492304		
TC1200000287.mm.1	-1,72	Atxn7l1	JUC1200001283.mm.1	-2,2	0,046946	0,557561		
TC1800000638.mm.1	-1,04	Sh3tc2	JUC1800002617.mm.1	-2,2	0,010109	0,418998		
TC1800000638.mm.1	-1,04	Sh3tc2	JUC1800002612.mm.1	-2,52	0,020086	0,4766		
TC0100002008.mm.1	1,08	Tceb1	JUC0100009305.mm.1	-2,2	0,048114	0,559875		
TC1800000459.mm.1	1,03	A930012L18Rik	PSR1800003380.mm.1	-2,2	0,016274	0,45836		
TC1500001091.mm.1	1,81	Zfp740	JUC1500004938.mm.1	-2,2	0,047527	0,558686		
TC1700000502.mm.1	-1,39	Pi16	JUC1700002398.mm.1	-2,2	0,048401	0,560658		
TC0800001614.mm.1	1,23	Fcor	JUC0800007229.mm.1	-2,2	0,030493	0,513341		

TC0600001695.mm.1	-1		JUC0600007072.mm.1	-2,2	0,009599	0,417256		
TC0700003038.mm.1	1,12	Saal1	JUC0700014832.mm.1	-2,2	0,038693	0,537169		
TC0300002977.mm.1	1,18	Arhgef38; D630013G24Ril	JUC0300012274.mm.1	-2,2	0,038485	0,536539		
TC0400004219.mm.1	1,38	B4galt2	JUC0400014127.mm.1	-2,2	0,001725	0,335996		
TC0400004047.mm.1	-1,04	Pik3cd	JUC0400017550.mm.1	-2,2	0,046458	0,556553		
TC1000000835.mm.1	1,07	Abca7	JUC1000003362.mm.1	-2,2	0,01709	0,462202		
TC1000000835.mm.1	1,07	Abca7	JUC1000003351.mm.1	-2,26	0,020463	0,478018		
TC1300001661.mm.1	3,87	Gm11344; Gm11345; Gm1	PSR1300010325.mm.1	-2,21	0,022622	0,487464	Cassette Exon	0,25
TC1300001661.mm.1	3,87	Gm11344; Gm11345; Gm1	PSR1300010329.mm.1	-2,93	0,00216	0,344238	Cassette Exon	0,17
TC1300001661.mm.1	3,87	Gm11344; Gm11345; Gm1	PSR1300010332.mm.1	-3,05	0,028978	0,508779	Cassette Exon	0,25
TC1300001661.mm.1	3,87	Gm11344; Gm11345; Gm1	JUC1300005295.mm.1	-4,13	0,009592	0,417183		
TC1300001661.mm.1	3,87	Gm11344; Gm11345; Gm1	PSR1300010333.mm.1	-4,18	0,004699	0,370633	Cassette Exon	0,28
TC1300001661.mm.1	3,87	Gm11344; Gm11345; Gm1	JUC1300005297.mm.1	-4,82	0,009339	0,415075		
TC1300001661.mm.1	3,87	Gm11344; Gm11345; Gm1	PSR1300010335.mm.1	-5,17	0,004183	0,362669	Cassette Exon	0,28
TC1300001661.mm.1	3,87	Gm11344; Gm11345; Gm1	PSR1300010334.mm.1	-5,59	0,01986	0,475704	Cassette Exon	0,25
TC1300001661.mm.1	3,87	Gm11344; Gm11345; Gm1	PSR1300010326.mm.1	-5,65	0,001056	0,316361	Cassette Exon	0,47
TC1300001661.mm.1	3,87	Gm11344; Gm11345; Gm1	PSR1300010336.mm.1	-5,8	0,001396	0,325997	Cassette Exon	0,28
TC1300001661.mm.1	3,87	Gm11344; Gm11345; Gm1	JUC1300005296.mm.1	-7,14	0,028563	0,507547		
TC1700000913.mm.1	13,97	Guca1b	PSR1700008753.mm.1	-2,21	0,010279	0,420305	Alternative 5' Donc	0,11
TC1700000913.mm.1	13,97	Guca1b	PSR1700008751.mm.1	-2,39	0,003499	0,355638	Cassette Exon	0,32
TC1700000913.mm.1	13,97	Guca1b	PSR1700008752.mm.1	-5,16	0,001218	0,322251	Intron Retention	0,42
TC1700000913.mm.1	13,97	Guca1b	JUC1700004688.mm.1	-10,04	0,003505	0,355749		
TC1200001245.mm.1	2,19	Klc1	PSR1200008902.mm.1	-2,21	0,029013	0,508842	Cassette Exon	0,26
TC1200001245.mm.1	2,19	Klc1	PSR1200008890.mm.1	-2,36	0,038279	0,536002	Alternative 5' Donc	0,19
TC1200001245.mm.1	2,19	Klc1	PSR1200008896.mm.1	-2,39	0,006486	0,390639	Cassette Exon	0,16
TC1200001245.mm.1	2,19	Klc1	JUC1200004857.mm.1	-2,42	0,009424	0,415987		
TC1200001245.mm.1	2,19	Klc1	JUC1200004866.mm.1	-2,42	0,017002	0,461553		
TC1200001245.mm.1	2,19	Klc1	PSR1200008875.mm.1	-2,43	0,021178	0,480978	Alternative 3' Acce	0,23
TC1200001245.mm.1	2,19	Klc1	JUC1200004868.mm.1	-2,6	0,004028	0,361636		
TC1200001245.mm.1	2,19	Klc1	PSR1200008901.mm.1	-2,92	0,030426	0,513134	Cassette Exon	0,16
TC1200001245.mm.1	2,19	Klc1	JUC1200004870.mm.1	-3,07	0,036964	0,532431		
TC1200001245.mm.1	2,19	Klc1	PSR1200008903.mm.1	-3,37	0,011182	0,426414	Cassette Exon	0,37
TC1200001245.mm.1	2,19	Klc1	PSR1200008904.mm.1	-3,66	0,008424	0,406611	Cassette Exon	0,34
TC1200001245.mm.1	2,19	Klc1	JUC1200004871.mm.1	-4,8	0,027998	0,505841		
TC0400000234.mm.1	2,56	Epha7	PSR0400001437.mm.1	-2,21	0,021049	0,480262	Alternative 3' Acce	0,12
TC0400000234.mm.1	2,56	Epha7	PSR0400001440.mm.1	-2,25	0,010852	0,424161	Cassette Exon	0,11
TC0400000234.mm.1	2,56	Epha7	PSR0400001463.mm.1	-2,27	0,003124	0,353892	Cassette Exon	0,11
TC0400000234.mm.1	2,56	Epha7	PSR0400001449.mm.1	-2,28	0,005114	0,376246		
TC0400000234.mm.1	2,56	Epha7	PSR0400001450.mm.1	-2,67	0,009587	0,417183	Alternative 5' Donc	0,06
TC0400000234.mm.1	2,56	Epha7	PSR0400001459.mm.1	-2,82	0,004505	0,36858	Alternative 5' Donc	0,27
TC0400000234.mm.1	2,56	Epha7	PSR0400001471.mm.1	-2,95	0,023686	0,491379	Alternative 5' Donc	0,26
TC1300002347.mm.1	1,22	Ankrd32	PSR1300015534.mm.1	-2,21	0,026497	0,501252	Alternative 5' Donc	0,16
TC1300002347.mm.1	1,22	Ankrd32	JUC1300008161.mm.1	-2,31	0,030722	0,514036		
TC1300002347.mm.1	1,22	Ankrd32	PSR1300015530.mm.1	-2,6	0,010145	0,419381	Alternative 5' Donc	0,25
TC1100004061.mm.1	1,77	Nat9	PSR1100038101.mm.1	-2,21	0,018732	0,470733		
TC1100004061.mm.1	1,77	Nat9	PSR1100038113.mm.1	-2,84	0,020322	0,477245	Intron Retention	0,24
TC1300000275.mm.1	1,66	Fam65b	PSR1300001608.mm.1	-2,21	0,030958	0,514654	Cassette Exon	0,23
TC1300000275.mm.1	1,66	Fam65b	PSR1300001639.mm.1	-2,26	0,01391	0,445962	Cassette Exon	0,11

TC1300000275.mm.1	1,66	Fam65b	JUC1300000823.mm.1	-4,1	0,046831	0,557144		
TC0500002283.mm.1	1,24		PSR0500021055.mm.1	-2,21	0,027932	0,505612	Intron Retention	0,18
TC0500002283.mm.1	1,24		JUC0500011454.mm.1	-3,76	0,013849	0,445398		
TC0900000980.mm.1	1,06	Prtg	PSR0900007588.mm.1	-2,21	0,005305	0,378059	Alternative 5' Donc	0,18
TC0X00002833.mm.1	-1,41	Fndc3c1	PSR0X00018021.mm.1	-2,21	0,033727	0,523549	Alternative 3' Acce	0,17
TC0100001671.mm.1	2,03	Zbtb18	JUC0100007842.mm.1	-2,21	0,027187	0,503342		
TC0100001671.mm.1	2,03	Zbtb18	PSR0100013845.mm.1	-2,49	0,00608	0,386691	Cassette Exon	0,16
TC1400001652.mm.1	1,1		PSR1400012416.mm.1	-2,21	0,018383	0,468484	Alternative 5' Donc	0,16
TC0300000548.mm.1	1,02	Mlf1	PSR0300003709.mm.1	-2,21	0,013606	0,443731	Cassette Exon	0,15
TC0500001584.mm.1	1,14	Por	PSR0500014291.mm.1	-2,21	0,00972	0,417839	Cassette Exon	0,15
TC1100003002.mm.1	1,07		PSR1100027233.mm.1	-2,21	0,048778	0,561474	Cassette Exon	0,15
TC0400000005.mm.1	1,05	AI838599	PSR0400000024.mm.1	-2,21	0,007606	0,401767	Intron Retention	0,12
TC0400000005.mm.1	1,05	AI838599	PSR0400000023.mm.1	-2,43	0,0289	0,50858	Cassette Exon	0,14
TC0700004285.mm.1	1,37	Cln3	JUC0700018916.mm.1	-2,21	0,033927	0,524252		
TC0700004285.mm.1	1,37	Cln3	PSR0700035768.mm.1	-2,31	0,011646	0,42985	Cassette Exon	0,13
TC0700004285.mm.1	1,37	Cln3	PSR0700035791.mm.1	-2,31	0,027063	0,502979		
TC1100001952.mm.1	2,34	Sec14l1	PSR1100018620.mm.1	-2,21	0,007238	0,39826	Cassette Exon	0,07
TC1100001952.mm.1	2,34	Sec14l1	PSR1100018597.mm.1	-2,47	0,017022	0,461729	Cassette Exon	0,13
TC1100001952.mm.1	2,34	Sec14l1	JUC1100009678.mm.1	-3,83	0,029921	0,511499		
TC1100004288.mm.1	1,35	Urgcp	PSR1100020875.mm.1	-2,21	0,000975	0,314364	Cassette Exon	0,13
TC0200000339.mm.1	1,36	Thnsl1	PSR0200001591.mm.1	-2,21	0,021131	0,480664	Alternative 3' Acce	0,12
TC0500001724.mm.1	2,38	Fscn1	PSR0500015806.mm.1	-2,21	0,010533	0,42227	Alternative 3' Acce	0,12
TC0700002119.mm.1	1,79	Gm7353	PSR0700019089.mm.1	-2,21	0,011066	0,425887	Cassette Exon	0,12
TC0X00001710.mm.1	-1,38	Mid1	PSR0X00010951.mm.1	-2,21	0,048302	0,560426	Cassette Exon	0,12
TC0X00001710.mm.1	-1,38	Mid1	PSR0X00010952.mm.1	-2,44	0,023526	0,490947	Alternative 5' Donc	0,08
TC0X00001710.mm.1	-1,38	Mid1	JUC0X00005565.mm.1	-2,55	0,038553	0,536762		
TC1900001071.mm.1	1,23	Ttc9c	PSR1900010038.mm.1	-2,21	0,017997	0,466929	Alternative 3' Acce	0,12
TC0200001012.mm.1	1,65	Klhl23	PSR0200007989.mm.1	-2,21	0,0162	0,458183	Cassette Exon	0,11
TC0900002808.mm.1	2,67	9330159M07Rik	PSR0900023304.mm.1	-2,21	0,008808	0,409934	Cassette Exon	0,11
TC0900002808.mm.1	2,67	9330159M07Rik	PSR0900023311.mm.1	-2,41	0,00324	0,354243	Cassette Exon	0,06
TC0900002808.mm.1	2,67	9330159M07Rik	JUC0900013045.mm.1	-2,49	0,004663	0,370181		
TC0900002808.mm.1	2,67	9330159M07Rik	JUC0900013046.mm.1	-2,55	0,040435	0,542058		
TC0900002808.mm.1	2,67	9330159M07Rik	JUC0900013047.mm.1	-2,78	0,024199	0,493294		
TC0900002808.mm.1	2,67	9330159M07Rik	JUC0900013043.mm.1	-2,93	0,004133	0,3623		
TC0900002808.mm.1	2,67	9330159M07Rik	JUC0900013044.mm.1	-3,13	0,019078	0,472172		
TC1200000355.mm.1	1,51	4930555J06Rik; Arl4aos	PSR1200002579.mm.1	-2,21	0,02378	0,491795	Cassette Exon	0,11
TC1800000825.mm.1	1,83	Pias2	PSR1800005993.mm.1	-2,21	0,010338	0,420962	Cassette Exon	0,05
TC1800000825.mm.1	1,83	Pias2	PSR1800005975.mm.1	-2,34	0,012654	0,43631	Cassette Exon	0,1
TC1800000825.mm.1	1,83	Pias2	JUC1800003309.mm.1	-3,77	0,011885	0,431306		
TC0700002769.mm.1	1,1	Zbtb32	PSR0700024676.mm.1	-2,21	0,000581	0,304044	Cassette Exon	0,08
TC1300002011.mm.1	1,54	Auh	PSR1300012536.mm.1	-2,21	0,031515	0,516965	Cassette Exon	0,08
TC1400002430.mm.1	-1,19	Slc25a30	JUC1400010396.mm.1	-2,21	0,04194	0,545823		
TC0300000841.mm.1	-1,13	Gm4858	JUC0300003386.mm.1	-2,21	0,034289	0,525378		
TC1700001638.mm.1	-1,04	Tbl3	JUC1700007997.mm.1	-2,21	0,018316	0,468088		
TC0100002825.mm.1	1,21	9430060I03Rik	JUC0100012963.mm.1	-2,21	0,012453	0,43528		
TC0100000417.mm.1	1,01	9130024F11Rik	JUC0100001862.mm.1	-2,21	0,01391	0,445962		
TC1900000660.mm.1	1,34	Sema4g	JUC1900003123.mm.1	-2,21	0,000874	0,311909		
TC1500002250.mm.1	-1,28	Galnt6	JUC1500010530.mm.1	-2,21	0,038783	0,537492		

TC0800001298.mm.1	-1,14	Pkd1l3	JUC0800005663.mm.1	-2,21	0,000571	0,304044		
TC0800002154.mm.1	1,74	Snx25	JUC0800009025.mm.1	-2,21	0,006053	0,386642		
TC0500002200.mm.1	-1,22	Gm7596	JUC0500010965.mm.1	-2,21	0,002625	0,349612		
TC0500002280.mm.1	2,93	Nsg1	JUC0500011434.mm.1	-2,21	0,032692	0,520439		
TC0700004011.mm.1	-1,31	Gm1966; RP23-465M17.2	JUC0700017562.mm.1	-2,21	0,006068	0,386691		
TC0400000446.mm.1	1,05	Npr2	JUC0400001498.mm.1	-2,21	0,019634	0,474876		
TC1000003150.mm.1	1,14	Zc3h10	JUC1000013029.mm.1	-2,21	0,008373	0,406495		
TC0400000532.mm.1	-1,03	Msantd3	JUC0400001985.mm.1	-2,21	0,019802	0,475245		
TC1000001239.mm.1	-1,06	Rassf9	JUC1000005027.mm.1	-2,21	0,023476	0,490659		
TC0X00000693.mm.1	-1,37	Plxnb3	JUC0X00002295.mm.1	-2,21	0,041117	0,543958		
TC0200005006.mm.1	3,18	Ndrg3	PSR0200042774.mm.1	-2,22	0,0046	0,369794	Intron Retention	0,24
TC0200005006.mm.1	3,18	Ndrg3	PSR0200042771.mm.1	-2,27	0,007758	0,402774	Alternative 3' Acce	0,2
TC0200005006.mm.1	3,18	Ndrg3	PSR0200042801.mm.1	-2,29	0,019233	0,472971		
TC0200005006.mm.1	3,18	Ndrg3	PSR0200042791.mm.1	-2,33	0,025581	0,497808	Alternative 3' Acce	0,21
TC0200005006.mm.1	3,18	Ndrg3	PSR0200042790.mm.1	-2,37	0,01334	0,441924	Alternative 3' Acce	0,21
TC0200005006.mm.1	3,18	Ndrg3	PSR0200042793.mm.1	-2,42	0,0338	0,523848	Alternative 5' Donc	0,23
TC0200005006.mm.1	3,18	Ndrg3	PSR0200042797.mm.1	-3,04	0,007832	0,403202	Cassette Exon	0,23
TC0200005006.mm.1	3,18	Ndrg3	JUC0200022072.mm.1	-3,86	0,026639	0,50179		
TC0200005006.mm.1	3,18	Ndrg3	PSR0200042798.mm.1	-3,99	0,0135	0,443238	Alternative 5' Donc	0,15
TC0200005006.mm.1	3,18	Ndrg3	PSR0200042776.mm.1	-4,27	0,000099	0,262646	Intron Retention	0,7
TC0200005006.mm.1	3,18	Ndrg3	PSR0200042789.mm.1	-4,4	0,023793	0,491876	Cassette Exon	0,31
TC0200005006.mm.1	3,18	Ndrg3	JUC0200022069.mm.1	-10,82	0,004234	0,363295		
TC0100001610.mm.1	1,85	Igsf8	PSR0100013393.mm.1	-2,22	0,030782	0,514144	Cassette Exon	0,11
TC0100001610.mm.1	1,85	Igsf8	PSR0100013374.mm.1	-2,79	0,036326	0,530547	Cassette Exon	0,17
TC0100001610.mm.1	1,85	Igsf8	PSR0100013376.mm.1	-3,19	0,024378	0,49362	Intron Retention	0,41
TC0100001610.mm.1	1,85	Igsf8	JUC0100007567.mm.1	-3,23	0,029445	0,51004		
TC0100001610.mm.1	1,85	Igsf8	JUC0100007568.mm.1	-3,98	0,037801	0,534736		
TC0100002349.mm.1	2,25	Pgap1	PSR0100018990.mm.1	-2,22	0,026348	0,50085	Alternative 5' Donc	0,22
TC0100002349.mm.1	2,25	Pgap1	JUC0100010760.mm.1	-2,58	0,026136	0,499937		
TC0100002349.mm.1	2,25	Pgap1	JUC0100010762.mm.1	-2,77	0,027073	0,502997		
TC0100002349.mm.1	2,25	Pgap1	PSR0100018996.mm.1	-2,89	0,038469	0,536428	Cassette Exon	0,31
TC0100002349.mm.1	2,25	Pgap1	PSR0100018995.mm.1	-3,14	0,034396	0,525711	Cassette Exon	0,27
TC0100002349.mm.1	2,25	Pgap1	JUC0100010781.mm.1	-3,49	0,045342	0,553891		
TC0100002349.mm.1	2,25	Pgap1	JUC0100010764.mm.1	-3,69	0,049693	0,563531		
TC0100002349.mm.1	2,25	Pgap1	PSR0100019002.mm.1	-4,16	0,022037	0,485268	Cassette Exon	0,38
TC0300000530.mm.1	1,72	Lekr1	PSR0300003632.mm.1	-2,22	0,020957	0,479982	Alternative 5' Donc	0,11
TC0300000530.mm.1	1,72	Lekr1	PSR0300003617.mm.1	-3,34	0,005	0,373851	Alternative 5' Donc	0,3
TC0500002981.mm.1	1,29	Gtpbp6	PSR0500026342.mm.1	-2,22	0,035679	0,529027	Intron Retention	0,28
TC0700003738.mm.1	1,4	Ankrd42	PSR0700031481.mm.1	-2,22	0,007103	0,396897	Cassette Exon	0,1
TC0700003738.mm.1	1,4	Ankrd42	PSR0700031498.mm.1	-2,81	0,000541	0,304044	Alternative 5' Donc	0,28
TC0X00002418.mm.1	1,63	Xlr3a	PSR0X00014910.mm.1	-2,22	0,010133	0,419217	Alternative 5' Donc	0,22
TC0X00002418.mm.1	1,63	Xlr3a	JUC0X00007598.mm.1	-2,72	0,019545	0,474538		
TC0X00002418.mm.1	1,63	Xlr3a	PSR0X00014919.mm.1	-2,73	0,024605	0,494497	Cassette Exon	0,18
TC0X00002418.mm.1	1,63	Xlr3a	PSR0X00014927.mm.1	-3,08	0,014641	0,450098	Cassette Exon	0,18
TC1300000317.mm.1	1,5	A330102I10Rik	PSR1300001953.mm.1	-2,22	0,001188	0,321162	Alternative 3' Acce	0,22
TC0100002607.mm.1	1,79	Resp18	PSR0100021234.mm.1	-2,22	0,02056	0,478336	Cassette Exon	0,2
TC0100002607.mm.1	1,79	Resp18	JUC0100012035.mm.1	-3,58	0,02012	0,47686		
TC0300002273.mm.1	1,57	Dclk2	PSR0300017066.mm.1	-2,22	0,000445	0,297771	Alternative 3' Acce	0,2

TC0300002273.mm.1	1,57	Dclk2	JUC0300008958.mm.1	-4,91	0,008776	0,40982		
TC0900002281.mm.1	2,04	Sik2	PSR0900019028.mm.1	-2,22	0,005121	0,376275	Intron Retention	0,15
TC0800000912.mm.1	2,19	Rnf150	PSR0800006730.mm.1	-2,22	0,012647	0,436303	Alternative 3' Acce	0,14
TC1500000693.mm.1	1,17	Syngr1	PSR1500005199.mm.1	-2,22	0,012074	0,43255	Cassette Exon	0,14
TC0400001785.mm.1	-1,15	Epha2	PSR0400014825.mm.1	-2,22	0,006669	0,393187	Cassette Exon	0,13
TC0700000851.mm.1	-1,19	Plekha4	PSR0700007362.mm.1	-2,22	0,017375	0,463612	Cassette Exon	0,13
TC0700004399.mm.1	1,45	Ikzf5	PSR0700037021.mm.1	-2,22	0,030212	0,512452	Cassette Exon	0,11
TC0900001789.mm.1	-1,19	Ccdc67	PSR0900015075.mm.1	-2,22	0,016544	0,459612	Cassette Exon	0,11
TC1900000809.mm.1	-1,03	Tdrd1	PSR1900007098.mm.1	-2,22	0,024359	0,49362	Cassette Exon	0,1
TC1400000530.mm.1	1,31	Tmem260	JUC1400002534.mm.1	-2,22	0,035274	0,52837		
TC0300000775.mm.1	1,31	4933434E20Rik; 1700094I	JUC0300003142.mm.1	-2,22	0,006383	0,389554		
TC0300000775.mm.1	1,31	4933434E20Rik; 1700094I	JUC0300003144.mm.1	-2,27	0,002446	0,349135		
TC0300000769.mm.1	-1,11	Adar	JUC0300003109.mm.1	-2,22	0,038084	0,535633		
TC1200000211.mm.1	1,46	Kidins220	JUC1200000984.mm.1	-2,22	0,017966	0,466781		
TC1700002671.mm.1	-1,05	2010106C02Rik	JUC1700013108.mm.1	-2,22	0,011033	0,425682		
TC0100001903.mm.1	1,98		JUC0100008926.mm.1	-2,22	0,034783	0,526754		
TC1500001174.mm.1	1,55	Wdr70	JUC1500005314.mm.1	-2,22	0,012419	0,435195		
TC1500001174.mm.1	1,55	Wdr70	JUC1500005315.mm.1	-4,25	0,041066	0,543798		
TC0800002852.mm.1	-1,06	Esrp2	JUC0800012032.mm.1	-2,22	0,034357	0,525572		
TC0600000987.mm.1	1,24	Tia1	JUC0600003904.mm.1	-2,22	0,046112	0,555792		
TC0700001709.mm.1	-1,5	Acsm5	JUC0700007380.mm.1	-2,22	0,006992	0,395722		
TC1000002927.mm.1	-1,16	9530003J23Rik	JUC1000012168.mm.1	-2,22	0,026234	0,50019		
TC0400000498.mm.1	1,44	Tdrd7	JUC0400001774.mm.1	-2,22	0,012653	0,43631		
TC0400000498.mm.1	1,44	Tdrd7	JUC0400001789.mm.1	-2,24	0,025935	0,499126		
TC1100002934.mm.1	1,13	Ncor1	JUC1100013939.mm.1	-2,22	0,025401	0,497403		
TC1100001926.mm.1	-1,07		JUC1100009561.mm.1	-2,22	0,002189	0,344463		
TC0400001333.mm.1	-1,21	Zmynd12	JUC0400005284.mm.1	-2,22	0,009878	0,417993		
TC1300001169.mm.1	4,14	Sgtb	JUC1300003850.mm.1	-2,23	0,00027	0,28803		
TC1300001169.mm.1	4,14	Sgtb	PSR1300007396.mm.1	-2,28	0,04194	0,545823	Cassette Exon	0,13
TC1300001169.mm.1	4,14	Sgtb	PSR1300007386.mm.1	-3,11	0,012751	0,436867	Cassette Exon	0,18
TC1300001169.mm.1	4,14	Sgtb	PSR1300007385.mm.1	-4,86	0,004718	0,370888	Alternative 3' Acce	0,14
TC1300001169.mm.1	4,14	Sgtb	PSR1300007401.mm.1	-5,71	0,000654	0,304044	Alternative 5' Donc	0,48
TC1300001169.mm.1	4,14	Sgtb	JUC1300003856.mm.1	-15,06	0,013367	0,442169		
TC0100001339.mm.1	12,6	Pdc	JUC0100006364.mm.1	-2,23	0,002751	0,350099		
TC0100001339.mm.1	12,6	Pdc	JUC0100006362.mm.1	-2,62	0,001854	0,337479		
TC0100001339.mm.1	12,6	Pdc	PSR0100011037.mm.1	-2,74	0,014034	0,44654	Cassette Exon	0,22
TC0100001339.mm.1	12,6	Pdc	PSR0100011039.mm.1	-3,97	0,0217	0,484104	Cassette Exon	0,39
TC1400002124.mm.1	-1,07	Gm16573	PSR1400016381.mm.1	-2,23	0,013795	0,444974	Cassette Exon	0,11
TC1400002124.mm.1	-1,07	Gm16573	PSR1400016377.mm.1	-3,01	0,006617	0,392269	Alternative 3' Acce	0,32
TC0700002594.mm.1	1,46	Zfp61	PSR0700022414.mm.1	-2,23	0,024669	0,494682	Intron Retention	0,27
TC0700002594.mm.1	1,46	Zfp61	PSR0700022412.mm.1	-2,58	0,021908	0,484824	Alternative 3' Acce	0,21
TC0200005517.mm.1	2,29	Uckl1	PSR0200046535.mm.1	-2,23	0,019717	0,474887	Cassette Exon	0,11
TC0200005517.mm.1	2,29	Uckl1	PSR0200046528.mm.1	-2,34	0,014767	0,450801	Intron Retention	0,26
TC0200005517.mm.1	2,29	Uckl1	PSR0200046500.mm.1	-3,08	0,007703	0,402552	Alternative 3' Acce	0,25
TC0200005517.mm.1	2,29	Uckl1	JUC0200023960.mm.1	-3,53	0,027978	0,505748		
TC0600002878.mm.1	1,37	Crbn	PSR0600022122.mm.1	-2,23	0,035471	0,528724	Cassette Exon	0,14
TC0800002702.mm.1	1,18	Ces1d	PSR0800020665.mm.1	-2,23	0,049443	0,563154	Alternative 5' Donc	0,13
TC0200002961.mm.1	1,01	Pfkfb3	PSR0200024359.mm.1	-2,23	0,045539	0,554382	Cassette Exon	0,11

TC1400000797.mm.1	1,08	Khnyn	JUC1400003452.mm.1	-2,23	0,028723	0,508141		
TC1100004053.mm.1	-1,09	Cd300ld	JUC1100019948.mm.1	-2,23	0,039127	0,538487		
TC1300000790.mm.1	1,53		JUC1300002726.mm.1	-2,23	0,02367	0,491379		
TC1700002227.mm.1	1	Tspo2	JUC1700011218.mm.1	-2,23	0,022656	0,487609		
TC1800000900.mm.1	1,37		JUC1800003595.mm.1	-2,23	0,017441	0,463827		
TC1900000664.mm.1	1,11	Sfxn3	JUC1900003139.mm.1	-2,23	0,021753	0,484278		
TC0100001595.mm.1	-1,13	Cd244; LOC677008	JUC0100007459.mm.1	-2,23	0,038688	0,537169		
TC1600000149.mm.1	-1,13	Ercc4	JUC1600000540.mm.1	-2,23	0,008642	0,408463		
TC0500003727.mm.1	1,27	Tbc1d14	JUC0500011310.mm.1	-2,23	0,030907	0,514574		
TC0800000530.mm.1	1,13	4933411K20Rik	JUC0800002008.mm.1	-2,23	0,019296	0,473074		
TC0500000837.mm.1	-1,34	5830473C10Rik	JUC0500004039.mm.1	-2,23	0,04876	0,561437		
TC0500000837.mm.1	-1,34	5830473C10Rik	JUC0500004051.mm.1	-2,94	0,017992	0,466929		
TC0700000290.mm.1	1,1	Eml2; Mir330	JUC0700001070.mm.1	-2,23	0,048263	0,560283		
TC0700002947.mm.1	-1,04	4931406B18Rik	JUC0700013932.mm.1	-2,23	0,026386	0,501055		
TC0700003434.mm.1	-1,36	Lrrk1	JUC0700015451.mm.1	-2,23	0,024062	0,493026		
TC0600000458.mm.1	-1,11	Clcn1	JUC0600001856.mm.1	-2,23	0,033266	0,522243		
TC1100002866.mm.1	-1,05	Pemt	JUC1100013565.mm.1	-2,23	0,043204	0,548962		
TC0300001760.mm.1	-1,19	Fndc3b	JUC0300007205.mm.1	-2,23	0,022666	0,487625		
TC0900002887.mm.1	1,3	Zbtb38; E030011O05Rik	JUC0900013292.mm.1	-2,23	0,038094	0,535633		
TC0900002887.mm.1	1,3	Zbtb38; E030011O05Rik	JUC0900013284.mm.1	-3,44	0,010201	0,419711		
TC0500000319.mm.1	1,39	Gckr	PSR0500002822.mm.1	-2,24	0,036536	0,531355	Cassette Exon	0,19
TC0500000319.mm.1	1,39	Gckr	JUC0500001541.mm.1	-2,54	0,016688	0,460085		
TC0500000319.mm.1	1,39	Gckr	PSR0500002837.mm.1	-2,77	0,011926	0,431655	Cassette Exon	0,25
TC0500000319.mm.1	1,39	Gckr	JUC0500001550.mm.1	-2,94	0,047535	0,558701		
TC0X00002154.mm.1	1,84	Smarca1	PSR0X00013591.mm.1	-2,24	0,0051	0,375753	Intron Retention	0,25
TC0X00002154.mm.1	1,84	Smarca1	PSR0X00013625.mm.1	-2,29	0,049667	0,563531	Alternative 5' Donc	0,13
TC0X00002154.mm.1	1,84	Smarca1	PSR0X00013594.mm.1	-3,01	0,015925	0,456718	Cassette Exon	0,21
TC0X00002154.mm.1	1,84	Smarca1	JUC0X00006940.mm.1	-3,27	0,041225	0,544134		
TC0X00002154.mm.1	1,84	Smarca1	PSR0X00013628.mm.1	-5,35	0,008063	0,40448	Alternative 5' Donc	0,16
TC0400002929.mm.1	-1,36	Nfib	PSR0400023771.mm.1	-2,24	0,0369	0,532283	Cassette Exon	0,21
TC0600003539.mm.1	2,57	D630045J12Rik	PSR0600016518.mm.1	-2,24	0,036693	0,531756	Alternative 5' Donc	0,21
TC0600003539.mm.1	2,57	D630045J12Rik	PSR0600016537.mm.1	-2,34	0,031518	0,516966	Cassette Exon	0,12
TC0600003539.mm.1	2,57	D630045J12Rik	PSR0600016515.mm.1	-2,53	0,021393	0,482022	Cassette Exon	0,07
TC0600003539.mm.1	2,57	D630045J12Rik	PSR0600016519.mm.1	-2,78	0,002062	0,341987	Alternative 5' Donc	0,21
TC0600003539.mm.1	2,57	D630045J12Rik	JUC0600008660.mm.1	-5,17	0,000476	0,298999		
TC0300000309.mm.1	1,47	3110057O12Rik; Gm2011	PSR0300002323.mm.1	-2,24	0,014049	0,44654	Cassette Exon	0,12
TC0300000309.mm.1	1,47	3110057O12Rik; Gm2011	PSR0300002299.mm.1	-2,28	0,007012	0,395737	Alternative 3' Acce	0,2
TC0300000309.mm.1	1,47	3110057O12Rik; Gm2011	PSR0300002284.mm.1	-2,28	0,007012	0,395737	Alternative 3' Acce	0,15
TC0100003128.mm.1	1,65	Ipo9	PSR0100025384.mm.1	-2,24	0,031237	0,51596	Alternative 5' Donc	0,19
TC0100000109.mm.1	2,38	Gdap1	PSR0100000771.mm.1	-2,24	0,020793	0,479488	Alternative 3' Acce	0,18
TC1700002286.mm.1	1,11	Sgol1	PSR1700021392.mm.1	-2,24	0,013521	0,443431	Alternative 3' Acce	0,18
TC0800002363.mm.1	2,57		PSR0800017760.mm.1	-2,24	0,000243	0,28803	Alternative 3' Acce	0,14
TC0800002363.mm.1	2,57		PSR0800017759.mm.1	-2,98	0,001371	0,32572	Cassette Exon	0,17
TC0500002774.mm.1	1,27		PSR0500024447.mm.1	-2,24	0,045422	0,554105	Alternative 5' Donc	0,16
TC0600002178.mm.1	1,92	Fam115a	JUC0600009023.mm.1	-2,24	0,034463	0,525929		
TC0600002178.mm.1	1,92	Fam115a	PSR0600017193.mm.1	-2,26	0,04404	0,550824	Cassette Exon	0,15
TC0600002178.mm.1	1,92	Fam115a	PSR0600017186.mm.1	-2,3	0,014654	0,450098	Cassette Exon	0,04
TC0600002178.mm.1	1,92	Fam115a	JUC0600009024.mm.1	-5,24	0,005842	0,383458		

TC0600001983.mm.1	1,27	Tnpo3	PSR0600015624.mm.1	-2,24	0,014647	0,450098	Alternative 3' Acce	0,14
TC0600001983.mm.1	1,27	Tnpo3	JUC0600008204.mm.1	-2,35	0,010283	0,420351		
TC0500000066.mm.1	-1,03	Gm17091	PSR0500000600.mm.1	-2,24	0,010308	0,420505	Cassette Exon	0,11
TC1700000990.mm.1	-1,19	Emr4	PSR1700009417.mm.1	-2,24	0,019408	0,473802	Cassette Exon	0,09
TC1400001012.mm.1	1,31	Gfra2	PSR1400008125.mm.1	-2,24	0,009267	0,414688	Cassette Exon	0,07
TC0200003204.mm.1	1,12	Surf1	JUC0200013613.mm.1	-2,24	0,028525	0,507436		
TC1200000061.mm.1	-1,07	Hs1bp3	JUC1200000340.mm.1	-2,24	0,043483	0,549897		
TC1300001028.mm.1	1,15	Acot12	JUC1300003430.mm.1	-2,24	0,00465	0,370181		
TC1300001028.mm.1	1,15	Acot12	JUC1300003426.mm.1	-2,98	0,037716	0,534585		
TC1700002664.mm.1	1,01		JUC1700013068.mm.1	-2,24	0,007646	0,402193		
TC1500001641.mm.1	1,28	Adcy8	JUC1500006924.mm.1	-2,24	0,028209	0,506368		
TC0800001214.mm.1	1,35	Tmem208	JUC0800005167.mm.1	-2,24	0,021773	0,48438		
TC0800001214.mm.1	1,35	Tmem208	JUC0800005169.mm.1	-2,59	0,03352	0,523109		
TC0900001018.mm.1	2,24	Lysmd2	JUC0900004375.mm.1	-2,24	0,04496	0,552881		
TC0900000749.mm.1	1,05	Celf6	JUC0900002926.mm.1	-2,24	0,024868	0,49558		
TC0900000749.mm.1	1,05	Celf6	JUC0900002927.mm.1	-2,62	0,038099	0,535633		
TC0900000863.mm.1	-1,21	Fam96a	JUC0900003567.mm.1	-2,24	0,028914	0,50858		
TC0900001680.mm.1	1,17	Gm26870	JUC0900007965.mm.1	-2,24	0,002336	0,347265		
TC0500003234.mm.1	1,32	Diablo	JUC0500015814.mm.1	-2,24	0,049549	0,563325		
TC0700003834.mm.1	1,08	Atg16l2	JUC0700017091.mm.1	-2,24	0,026439	0,501198		
TC0700003613.mm.1	-1,76	Fsd2	JUC0700016241.mm.1	-2,24	0,022985	0,488889		
TC0700001793.mm.1	-1,09	Il4ra	JUC0700007995.mm.1	-2,24	0,019855	0,475698		
TC0700002625.mm.1	1,14	Dedd2	JUC0700012041.mm.1	-2,24	0,005759	0,383101		
TC0300001354.mm.1	2,24	Ppa2	JUC0300005711.mm.1	-2,24	0,004833	0,371555		
TC0300001354.mm.1	2,24	Ppa2	JUC0300005713.mm.1	-2,29	0,010649	0,422925		
TC1100003395.mm.1	-1,15	Heatr9; Gm11435	JUC1100016262.mm.1	-2,24	0,016662	0,460023		
TC0300002489.mm.1	1,4	Ciart; Gm129	JUC0300010103.mm.1	-2,24	0,02944	0,509995		
TC0400003258.mm.1	-1,48	8030443G20Rik	JUC0400013499.mm.1	-2,24	0,048546	0,561008		
TC1000000892.mm.1	1,63	Pip5k1c	JUC1000003809.mm.1	-2,24	0,03005	0,511812		
TC0400002442.mm.1	-1,07	4933428C19Rik	JUC0400010234.mm.1	-2,24	0,005637	0,381541		
TC0400002082.mm.1	1,26	Cdk11b	JUC0400009111.mm.1	-2,24	0,027749	0,505023		
TC0600000714.mm.1	3,74	Herc3	PSR0600005492.mm.1	-2,25	0,002328	0,346856	Intron Retention	0,29
TC0600000714.mm.1	3,74	Herc3	JUC0600002773.mm.1	-2,31	0,018927	0,471339		
TC0600000714.mm.1	3,74	Herc3	PSR0600005517.mm.1	-2,35	0,030229	0,512452	Alternative 5' Donc	0,22
TC0600000714.mm.1	3,74	Herc3	PSR0600005508.mm.1	-2,37	0,020519	0,478264	Cassette Exon	0,25
TC0600000714.mm.1	3,74	Herc3	PSR0600005509.mm.1	-2,38	0,020352	0,47734	Cassette Exon	0,26
TC0600000714.mm.1	3,74	Herc3	PSR0600005476.mm.1	-2,67	0,004711	0,370888	Cassette Exon	0,05
TC0600000714.mm.1	3,74	Herc3	JUC0600002766.mm.1	-3,07	0,011994	0,432286		
TC0600000714.mm.1	3,74	Herc3	PSR0600005473.mm.1	-3,08	0,013409	0,442624	Alternative 3' Acce	0,04
TC0600000714.mm.1	3,74	Herc3	PSR0600005472.mm.1	-3,1	0,005466	0,379939	Alternative 3' Acce	0,04
TC0600000714.mm.1	3,74	Herc3	JUC0600002757.mm.1	-3,17	0,005666	0,381922		
TC0600000714.mm.1	3,74	Herc3	PSR0600005474.mm.1	-3,18	0,004229	0,363295	Alternative 3' Acce	0,04
TC0600000714.mm.1	3,74	Herc3	PSR0600005512.mm.1	-3,44	0,00145	0,328414	Cassette Exon	0,25
TC0600000714.mm.1	3,74	Herc3	PSR0600005471.mm.1	-3,59	0,002508	0,349237	Alternative 3' Acce	0,04
TC0600000714.mm.1	3,74	Herc3	PSR0600005478.mm.1	-3,72	0,007287	0,398787	Cassette Exon	0,15
TC0600000714.mm.1	3,74	Herc3	PSR0600005511.mm.1	-4,21	0,001067	0,316361	Cassette Exon	0,25
TC0600000714.mm.1	3,74	Herc3	PSR0600005489.mm.1	-4,31	0,000976	0,314364	Alternative 3' Acce	0,45
TC0600000714.mm.1	3,74	Herc3	PSR0600005494.mm.1	-4,68	0,013417	0,442624	Cassette Exon	0,32

TC0600000714.mm.1	3,74	Herc3	PSR0600005470.mm.1	-5,21	0,010224	0,419955	Alternative 3' Acce	0,07
TC0600000714.mm.1	3,74	Herc3	JUC0600002778.mm.1	-13,33	0,001521	0,330287		
TC1500001938.mm.1	1,57	AI848285	PSR1500015263.mm.1	-2,25	0,016255	0,458312	Alternative 3' Acce	0,16
TC1500001938.mm.1	1,57	AI848285	PSR1500015265.mm.1	-3,38	0,001686	0,335182	Alternative 5' Donc	0,39
TC1100002107.mm.1	1,39	Sec14l2	PSR1100020454.mm.1	-2,25	0,007607	0,401767	Intron Retention	0,3
TC1100002107.mm.1	1,39	Sec14l2	PSR1100020473.mm.1	-2,52	0,031549	0,517128	Alternative 5' Donc	0,17
TC1100002107.mm.1	1,39	Sec14l2	PSR1100020447.mm.1	-2,81	0,0237	0,491427	Alternative 3' Acce	0,29
TC1500001772.mm.1	1,52	Oplah	PSR1500013527.mm.1	-2,25	0,020233	0,477145	Intron Retention	0,28
TC0600001092.mm.1	2,41	Slc6a6	JUC0600004346.mm.1	-2,25	0,028767	0,508148		
TC0600001092.mm.1	2,41	Slc6a6	PSR0600008547.mm.1	-3,45	0,011672	0,429939	Alternative 3' Acce	0,27
TC0300001589.mm.1	1,04	Wls	PSR0300012794.mm.1	-2,25	0,005614	0,381163	Alternative 5' Donc	0,2
TC1500001133.mm.1	2,2	Pde1b	PSR1500009130.mm.1	-2,25	0,029264	0,509788	Cassette Exon	0,18
TC1500001133.mm.1	2,2	Pde1b	PSR1500009129.mm.1	-2,27	0,018523	0,469156	Cassette Exon	0,09
TC1500001133.mm.1	2,2	Pde1b	PSR1500009127.mm.1	-2,35	0,039447	0,539705	Cassette Exon	0,1
TC1500001133.mm.1	2,2	Pde1b	PSR1500009131.mm.1	-2,71	0,010028	0,41878	Cassette Exon	0,2
TC0500000675.mm.1	1,12	Nipal1	PSR0500005958.mm.1	-2,25	0,013423	0,442624	Alternative 5' Donc	0,19
TC0900001459.mm.1	1,46	Scap	PSR0900012176.mm.1	-2,25	0,029864	0,511132	Cassette Exon	0,13
TC0900001459.mm.1	1,46	Scap	PSR0900012157.mm.1	-2,29	0,046078	0,555658	Alternative 3' Acce	0,19
TC0100002228.mm.1	2	Lonrf2	PSR0100017939.mm.1	-2,25	0,009213	0,41403	Alternative 3' Acce	0,18
TC1100004237.mm.1	1,36		PSR1100040648.mm.1	-2,25	0,003598	0,356223	Alternative 5' Donc	0,18
TC0600003533.mm.1	1,21	Ttll3	PSR0600009689.mm.1	-2,25	0,021334	0,481727	Alternative 3' Acce	0,17
TC0600003533.mm.1	1,21	Ttll3	JUC0600004985.mm.1	-3,2	0,030126	0,512017		
TC0800003218.mm.1	1,37	Map2k7	PSR0800000387.mm.1	-2,25	0,0223	0,486095	Alternative 5' Donc	0,15
TC0800003218.mm.1	1,37	Map2k7	JUC0800000224.mm.1	-4,55	0,045278	0,553722		
TC0800003218.mm.1	1,37	Map2k7	JUC0800000225.mm.1	-7,08	0,042709	0,548097		
TC1000003119.mm.1	1,15	Lrp1	PSR1000023356.mm.1	-2,25	0,040391	0,541998	Cassette Exon	0,15
TC0600002496.mm.1	-2,1	Tcf7l1	PSR0600019392.mm.1	-2,25	0,005359	0,378582	Cassette Exon	0,14
TC0900003269.mm.1	1,47	Sec22c	PSR0900027569.mm.1	-2,25	0,01511	0,452474	Cassette Exon	0,14
TC0200002569.mm.1	1,71		PSR0200021082.mm.1	-2,25	0,027725	0,504939	Cassette Exon	0,13
TC1400000866.mm.1	1,58	Trim13	PSR1400007050.mm.1	-2,25	0,026997	0,502909	Cassette Exon	0,13
TC0400001008.mm.1	1,17	Efcab7	PSR0400006808.mm.1	-2,25	0,043135	0,548664	Cassette Exon	0,12
TC0400001008.mm.1	1,17	Efcab7	JUC0400003480.mm.1	-2,25	0,038667	0,537102		
TC0600001089.mm.1	1,43		PSR0600008531.mm.1	-2,25	0,002119	0,342919	Cassette Exon	0,12
TC0200002087.mm.1	-1,51	Sptlc3	PSR0200016635.mm.1	-2,25	0,020216	0,477145	Cassette Exon	0,11
TC0600002281.mm.1	1,08	Hoxa7; Mira	PSR0600017859.mm.1	-2,25	0,03319	0,522148	Alternative 3' Acce	0,08
TC0500002344.mm.1	1,23	Fam184b	JUC0500011721.mm.1	-2,25	0,015895	0,456526		
TC0500002344.mm.1	1,23	Fam184b	PSR0500021491.mm.1	-2,52	0,01261	0,435952	Alternative 5' Donc	0,02
TC1200001348.mm.1	1,84	Efr3b	JUC1200005344.mm.1	-2,25	0,036789	0,532063		
TC1200001348.mm.1	1,84	Efr3b	JUC1200005342.mm.1	-2,43	0,005599	0,381115		
TC1200001348.mm.1	1,84	Efr3b	JUC1200005331.mm.1	-3,63	0,028781	0,508148		
TC1200001348.mm.1	1,84	Efr3b	JUC1200005353.mm.1	-3,88	0,017916	0,466466		
TC1100004081.mm.1	1,53	Gga3	JUC1100020111.mm.1	-2,25	0,025621	0,497926		
TC1200000047.mm.1	1,56		JUC1200000291.mm.1	-2,25	0,023018	0,489186		
TC1300000838.mm.1	1,04	Fastkd3	JUC1300002852.mm.1	-2,25	0,012315	0,434392		
TC1700002347.mm.1	-1,21	Dennd1c	JUC1700011800.mm.1	-2,25	0,018298	0,468049		
TC0200000587.mm.1	1,55	St6galnac6; St6galnac4	JUC0200002741.mm.1	-2,25	0,039672	0,540322		
TC0800001263.mm.1	1,1	Nip7	JUC0800005549.mm.1	-2,25	0,001659	0,334751		
TC0800001263.mm.1	1,1	Nip7	JUC0800005548.mm.1	-2,27	0,00049	0,298999		

TC0800001209.mm.1	-1,14	Hsf4	JUC0800005113.mm.1	-2,25	0,02202	0,485226		
TC0800002700.mm.1	-1,27	Ces1b	JUC0800011222.mm.1	-2,25	0,032464	0,519612		
TC0700000284.mm.1	1,39	Dmwd	JUC0700001031.mm.1	-2,25	0,028598	0,507685		
TC0700000946.mm.1	-1,08	Atp10a	JUC0700004440.mm.1	-2,25	0,034416	0,525828		
TC0700000853.mm.1	-1,01	Bcat2	JUC0700003681.mm.1	-2,25	0,017605	0,464312		
TC0600003589.mm.1	1,06	Adck1; Igkv8-24	JUC0600009781.mm.1	-2,25	0,048961	0,5618		
TC0700002980.mm.1	1,22	Prmt1	JUC0700014276.mm.1	-2,25	0,00218	0,344453		
TC0700003982.mm.1	-1,03	Hpx	JUC0700017459.mm.1	-2,25	0,046114	0,555792		
TC0700002310.mm.1	-1,45	Nlrp4d	JUC0700011075.mm.1	-2,25	0,030585	0,513425		
TC1100000168.mm.1	1,73	Cnrip1	PSR1100001497.mm.1	-2,25	0,03533	0,528404		
TC1100000168.mm.1	1,73	Cnrip1	JUC1100000808.mm.1	-3,46	0,003282	0,354243		
TC0400000090.mm.1	1,35	1110037F02Rik	JUC0400000280.mm.1	-2,25	0,026285	0,50053		
TC1000002211.mm.1	-1,06	Oit3	JUC1000008424.mm.1	-2,25	0,037518	0,533968		
TC0400000702.mm.1	-1,09	Bspry	JUC0400002472.mm.1	-2,25	0,027317	0,503743		
TC1100003641.mm.1	1,08	Npepps	JUC1100017516.mm.1	-2,25	0,021043	0,480262		
TC1100003641.mm.1	1,08	Npepps	JUC1100017520.mm.1	-3,86	0,044169	0,551056		
TC1100001591.mm.1	-1,16		JUC1100007736.mm.1	-2,25	0,0026	0,349501		
TC1200002411.mm.1	3,51	Ckb	JUC1200009226.mm.1	-2,26	0,022051	0,485327		
TC1200002411.mm.1	3,51	Ckb	PSR1200016738.mm.1	-2,58	0,031032	0,515011	Intron Retention	0,37
TC1200002411.mm.1	3,51	Ckb	PSR1200016744.mm.1	-3,82	0,030731	0,514036	Alternative 5' Donc	0,32
TC1200002411.mm.1	3,51	Ckb	PSR1200016728.mm.1	-4,63	0,016679	0,460085	Intron Retention	0,67
TC1200002411.mm.1	3,51	Ckb	PSR1200016730.mm.1	-4,91	0,021948	0,485095	Intron Retention	0,67
TC0900002327.mm.1	2,12	Chrna3	PSR0900019549.mm.1	-2,26	0,031228	0,515879	Alternative 5' Donc	0,2
TC0900002327.mm.1	2,12	Chrna3	PSR0900019545.mm.1	-2,61	0,013528	0,443465	Intron Retention	0,4
TC0900002327.mm.1	2,12	Chrna3	PSR0900019547.mm.1	-3,5	0,003121	0,353892	Intron Retention	0,62
TC0900002327.mm.1	2,12	Chrna3	PSR0900019540.mm.1	-3,55	0,004722	0,370888	Alternative 3' Acce	0,39
TC1700002359.mm.1	8,81	2610034M16Rik	PSR1700022159.mm.1	-2,26	0,02125	0,481431	Cassette Exon	0,15
TC1700002359.mm.1	8,81	2610034M16Rik	PSR1700022162.mm.1	-3,38	0,009647	0,417354	Cassette Exon	0,25
TC1700002359.mm.1	8,81	2610034M16Rik	PSR1700022170.mm.1	-4,03	0,000576	0,304044	Cassette Exon	0,24
TC1700002359.mm.1	8,81	2610034M16Rik	JUC1700011892.mm.1	-5,05	0,007001	0,395737		
TC1700002359.mm.1	8,81	2610034M16Rik	PSR1700022163.mm.1	-6,44	0,000198	0,28803	Cassette Exon	0,55
TC1700002359.mm.1	8,81	2610034M16Rik	JUC1700011893.mm.1	-10,78	0,001789	0,336311		
TC1700002359.mm.1	8,81	2610034M16Rik	JUC1700011902.mm.1	-11,1	0,001146	0,317344		
TC1800000468.mm.1	1,92	Kcnn2	PSR1800003449.mm.1	-2,26	0,041882	0,545743	Cassette Exon	0,3
TC1800000468.mm.1	1,92	Kcnn2	JUC1800001932.mm.1	-3,3	0,006434	0,390027		
TC1800000468.mm.1	1,92	Kcnn2	PSR1800003444.mm.1	-3,59	0,006103	0,387177	Alternative 5' Donc	0,42
TC0800002441.mm.1	1,25	Fcho1	JUC0800010168.mm.1	-2,26	0,044976	0,552889		
TC0800002441.mm.1	1,25	Fcho1	PSR0800018710.mm.1	-2,56	0,008669	0,408667	Intron Retention	0,34
TC0600000725.mm.1	1,07	Ccser1	PSR0600005574.mm.1	-2,26	0,001301	0,323375	Alternative 5' Donc	0,22
TC1600002056.mm.1	1,44	Urb1	JUC1600008099.mm.1	-2,26	0,04531	0,553742		
TC1600002056.mm.1	1,44	Urb1	PSR1600015751.mm.1	-2,28	0,036773	0,531983	Cassette Exon	0,12
TC1600002056.mm.1	1,44	Urb1	PSR1600015735.mm.1	-2,37	0,035908	0,529399	Cassette Exon	0,22
TC1600002056.mm.1	1,44	Urb1	JUC1600008109.mm.1	-2,74	0,036572	0,531378		
TC1600002056.mm.1	1,44	Urb1	JUC1600008117.mm.1	-2,77	0,005615	0,381191		
TC1600002056.mm.1	1,44	Urb1	JUC1600008121.mm.1	-5,52	0,014527	0,449545		
TC0300000258.mm.1	1,48	Atp11b	PSR0300001635.mm.1	-2,26	0,021778	0,48438	Alternative 3' Acce	0,2
TC0700000818.mm.1	1,03	Nup62-il4i1; Il4i1; Nup62	PSR0700006831.mm.1	-2,26	0,018599	0,469657	Alternative 5' Donc	0,19
TC0700000818.mm.1	1,03	Nup62-il4i1; Il4i1; Nup62	PSR0700006821.mm.1	-2,28	0,026883	0,502518	Alternative 5' Donc	0,08

TC0400001141.mm.1	-1,16	Echdc2	PSR0400008069.mm.1	-2,26	0,034248	0,525178	Alternative 5' Donc	0,17
TC0700004166.mm.1	1,64	Smg1	PSR0700034615.mm.1	-2,26	0,0282	0,506368	Cassette Exon	0,15
TC1400002328.mm.1	1,06	Rhobtb2	PSR1400018056.mm.1	-2,26	0,005635	0,381532	Cassette Exon	0,13
TC1000000366.mm.1	1,5	Ppil6	PSR1000002523.mm.1	-2,26	0,004716	0,370888	Cassette Exon	0,12
TC1500000992.mm.1	1,23		PSR1500007743.mm.1	-2,26	0,029947	0,511499	Cassette Exon	0,12
TC0X00002393.mm.1	1,73	BC023829	PSR0X00014768.mm.1	-2,26	0,048196	0,560128	Alternative 3' Acce	0,1
TC0X00002393.mm.1	1,73	BC023829	JUC0X00007530.mm.1	-2,51	0,025639	0,498009		
TC0X00002393.mm.1	1,73	BC023829	JUC0X00007527.mm.1	-2,75	0,025444	0,497549		
TC0X00002393.mm.1	1,73	BC023829	JUC0X00007532.mm.1	-4,71	0,022549	0,487254		
TC1200001874.mm.1	1,16	L2hgdh	JUC1200007066.mm.1	-2,26	0,028886	0,50858		
TC1300002208.mm.1	-1,04	Cntnap3	JUC1300007426.mm.1	-2,26	0,011428	0,427656		
TC0100000240.mm.1	-1,41	Arid5a	JUC0100000835.mm.1	-2,26	0,042025	0,546158		
TC0200001634.mm.1	-1,13	Slc5a12	JUC0200006102.mm.1	-2,26	0,009639	0,417354		
TC0200001634.mm.1	-1,13	Slc5a12	JUC0200006101.mm.1	-2,5	0,010258	0,420147		
TC1500002144.mm.1	-1,55		JUC1500009796.mm.1	-2,26	0,030742	0,514065		
TC1500000613.mm.1	-1,25	Commd5	JUC1500002574.mm.1	-2,26	0,04289	0,548183		
TC1700000594.mm.1	-1,13	Ndufa7	JUC1700003008.mm.1	-2,26	0,000981	0,31497		
TC0800001113.mm.1	1,1	Rspry1	JUC0800004642.mm.1	-2,26	0,019972	0,476227		
TC0800002366.mm.1	2,04		JUC0800009652.mm.1	-2,26	0,030583	0,513421		
TC0800000724.mm.1	-1,05	Atp13a1	JUC0800002589.mm.1	-2,26	0,005819	0,383169		
TC0500001404.mm.1	1,35	A930024E05Rik	JUC0500006811.mm.1	-2,26	0,041645	0,545276		
TC0500001404.mm.1	1,35	A930024E05Rik	JUC0500006810.mm.1	-2,61	0,00268	0,349612		
TC0500001370.mm.1	1,05	Atxn2	JUC0500006641.mm.1	-2,26	0,020861	0,479521		
TC0500001370.mm.1	1,05	Atxn2	JUC0500006655.mm.1	-3,19	0,007738	0,402774		
TC0600000974.mm.1	1,07	Tprkb	JUC0600003847.mm.1	-2,26	0,035365	0,528536		
TC0700000978.mm.1	-1,02	Mtmr10	JUC0700004570.mm.1	-2,26	0,0329	0,521149		
TC0700002740.mm.1	1,35	Zfp14	JUC0700012955.mm.1	-2,26	0,015493	0,454456		
TC0300002298.mm.1	-1,06	Ttc24	JUC0300009141.mm.1	-2,26	0,020259	0,477245		
TC0400003393.mm.1	-1,17	Tie1	JUC0400014356.mm.1	-2,26	0,022505	0,487114		
TC0400001992.mm.1	-1,09	Tnfrsf9	JUC0400008552.mm.1	-2,26	0,027426	0,503961		
TC0100002332.mm.1	3,33	Pms1	PSR0100018782.mm.1	-2,27	0,005664	0,381922	Cassette Exon	0,14
TC0100002332.mm.1	3,33	Pms1	PSR0100018777.mm.1	-2,92	0,012985	0,439245	Cassette Exon	0,27
TC0100002332.mm.1	3,33	Pms1	PSR0100018776.mm.1	-3,23	0,007208	0,398121	Cassette Exon	0,25
TC0100002332.mm.1	3,33	Pms1	PSR0100018779.mm.1	-3,23	0,006348	0,389463	Cassette Exon	0,22
TC0100002332.mm.1	3,33	Pms1	PSR0100018796.mm.1	-3,3	0,010378	0,421341		
TC0100002332.mm.1	3,33	Pms1	PSR0100018773.mm.1	-3,32	0,001828	0,337273	Cassette Exon	0,35
TC0100002332.mm.1	3,33	Pms1	PSR0100018772.mm.1	-3,4	0,007186	0,397855	Cassette Exon	0,48
TC0100002332.mm.1	3,33	Pms1	PSR0100018795.mm.1	-3,56	0,018399	0,468484	Alternative 3' Acce	0,04
TC0100002332.mm.1	3,33	Pms1	PSR0100018798.mm.1	-3,61	0,008263	0,405976	Cassette Exon	0,24
TC0100002332.mm.1	3,33	Pms1	JUC0100010604.mm.1	-3,71	0,007142	0,397526		
TC0100002332.mm.1	3,33	Pms1	PSR0100018778.mm.1	-3,74	0,003123	0,353892	Cassette Exon	0,36
TC0100002332.mm.1	3,33	Pms1	PSR0100018797.mm.1	-4,03	0,006887	0,39446		
TC0100002332.mm.1	3,33	Pms1	PSR0100018775.mm.1	-4,1	0,004295	0,364027	Cassette Exon	0,32
TC0100002332.mm.1	3,33	Pms1	JUC0100010620.mm.1	-4,27	0,038865	0,537834		
TC0100002332.mm.1	3,33	Pms1	JUC0100010605.mm.1	-7,19	0,00173	0,335996		
TC0100002332.mm.1	3,33	Pms1	PSR0100018771.mm.1	-7,45	0,003207	0,354243	Cassette Exon	0,4
TC1300002258.mm.1	1,63	Zfp738	PSR1300014734.mm.1	-2,27	0,026913	0,502518		
TC1300002258.mm.1	1,63	Zfp738	PSR1300014740.mm.1	-2,73	0,017165	0,46232	Alternative 3' Acce	0,33

TC1300002258.mm.1	1,63	Zfp738	JUC1300007688.mm.1	-2,77	0,045769	0,554863		
TC1300002258.mm.1	1,63	Zfp738	PSR1300014741.mm.1	-4,01	0,031757	0,517489	Alternative 3' Acce	0,44
TC0200003304.mm.1	1,42	Slc25a25	PSR0200028111.mm.1	-2,27	0,002342	0,347317	Intron Retention	0,31
TC0200003304.mm.1	1,42	Slc25a25	PSR0200028122.mm.1	-2,33	0,006726	0,393343	Cassette Exon	0,16
TC0600003123.mm.1	2,5	Gpr162	PSR0600024346.mm.1	-2,27	0,033587	0,523246	Cassette Exon	0,25
TC0600003123.mm.1	2,5	Gpr162	PSR0600024345.mm.1	-2,29	0,014955	0,451968	Cassette Exon	0,29
TC0600003123.mm.1	2,5	Gpr162	JUC0600012674.mm.1	-2,44	0,006301	0,389081		
TC0600003123.mm.1	2,5	Gpr162	JUC0600012676.mm.1	-2,98	0,034575	0,52602		
TC0600003123.mm.1	2,5	Gpr162	JUC0600012677.mm.1	-5,23	0,006567	0,39213		
TC0200000474.mm.1	1,46	Gm13421	PSR0200003219.mm.1	-2,27	0,025282	0,497095	Intron Retention	0,26
TC0800002674.mm.1	2,55	Tox3	PSR0800020459.mm.1	-2,27	0,003058	0,353892	Cassette Exon	0,23
TC0800002674.mm.1	2,55	Tox3	PSR0800020452.mm.1	-2,43	0,015567	0,454866	Alternative 3' Acce	0,17
TC0800002674.mm.1	2,55	Tox3	PSR0800020471.mm.1	-2,49	0,006643	0,392838	Alternative 5' Donc	0,16
TC0800002674.mm.1	2,55	Tox3	PSR0800020468.mm.1	-2,53	0,006266	0,388719	Alternative 5' Donc	0,16
TC0800002674.mm.1	2,55	Tox3	PSR0800020454.mm.1	-2,56	0,010323	0,420714	Alternative 3' Acce	0,22
TC0800002674.mm.1	2,55	Tox3	JUC0800011114.mm.1	-3,2	0,030293	0,512642		
TC1500001395.mm.1	2,01	Ncald; Gm15941	PSR1500010788.mm.1	-2,27	0,021645	0,483726	Cassette Exon	0,17
TC1500001395.mm.1	2,01	Ncald; Gm15941	PSR1500010787.mm.1	-2,35	0,038295	0,536095	Cassette Exon	0,08
TC1500001395.mm.1	2,01	Ncald; Gm15941	PSR1500010774.mm.1	-2,75	0,043467	0,549841	Alternative 3' Acce	0,19
TC1500001395.mm.1	2,01	Ncald; Gm15941	JUC1500006040.mm.1	-3,21	0,00914	0,413447		
TC1500001395.mm.1	2,01	Ncald; Gm15941	PSR1500010799.mm.1	-3,59	0,035178	0,528119	Cassette Exon	0,06
TC1500001395.mm.1	2,01	Ncald; Gm15941	JUC1500006043.mm.1	-4,18	0,005169	0,376797		
TC1500001395.mm.1	2,01	Ncald; Gm15941	PSR1500010780.mm.1	-5,65	0,006756	0,393343	Cassette Exon	0,23
TC0800000526.mm.1	1,61	Lrp2bp	PSR0800003691.mm.1	-2,27	0,02676	0,502096	Alternative 5' Donc	0,13
TC0800000526.mm.1	1,61	Lrp2bp	PSR0800003695.mm.1	-2,51	0,00378	0,357586	Alternative 3' Acce	0,21
TC0500000648.mm.1	1,41	Slc30a9	PSR0500005762.mm.1	-2,27	0,007767	0,402774	Alternative 5' Donc	0,2
TC1200002083.mm.1	1,24	Arel1; Gm17193	PSR1200014421.mm.1	-2,27	0,017046	0,461805	Alternative 5' Donc	0,2
TC1200002083.mm.1	1,24	Arel1; Gm17193	PSR1200014385.mm.1	-2,32	0,025899	0,498978	Alternative 3' Acce	0,12
TC0700004506.mm.1	1,68	Zfp941	PSR0700037848.mm.1	-2,27	0,005208	0,377078	Alternative 5' Donc	0,19
TC1700001665.mm.1	1,64	Sox8	PSR1700015182.mm.1	-2,27	0,014705	0,45037	Alternative 3' Acce	0,18
TC0800000323.mm.1	1,31	Whsc1l1	PSR0800002422.mm.1	-2,27	0,012027	0,432286	Alternative 5' Donc	0,13
TC0400002397.mm.1	1,24	Mob3b	PSR0400019463.mm.1	-2,27	0,007041	0,396259	Cassette Exon	0,12
TC1100000763.mm.1	1,27	Smcr8	PSR1100006130.mm.1	-2,27	0,047213	0,558132	Cassette Exon	0,12
TC1400002046.mm.1	-1,24		PSR1400015392.mm.1	-2,27	0,013924	0,446193	Cassette Exon	0,12
TC1900000026.mm.1	1,02	Cdk2ap2	PSR1900000321.mm.1	-2,27	0,00951	0,416602	Alternative 3' Acce	0,06
TC0200003280.mm.1	1,22	Tor1a	JUC0200014028.mm.1	-2,27	0,044408	0,551503		
TC0200003492.mm.1	1,54	Orc4	PSR0200029825.mm.1	-2,27	0,002979	0,352501		
TC1100004257.mm.1	1,19	Gm12590	JUC1100021405.mm.1	-2,27	0,000728	0,306408		
TC1100004257.mm.1	1,19	Gm12590	PSR1100040840.mm.1	-2,29	0,016708	0,460199		
TC1200000110.mm.1	1,2	Nbas	JUC1200000595.mm.1	-2,27	0,014119	0,447137		
TC1300002596.mm.1	1,08	Trappc13; 2410002O22Rik	JUC1300009451.mm.1	-2,27	0,027685	0,504781		
TC0100000572.mm.1	-1,35	Cps1	JUC0100002747.mm.1	-2,27	0,023767	0,491731		
TC1700000670.mm.1	1,1	Bag6	JUC1700003768.mm.1	-2,27	0,029096	0,50908		
TC0800001092.mm.1	-1,26	Mt4	JUC0800004490.mm.1	-2,27	0,010923	0,424725		
TC0800000019.mm.1	1	Retn	JUC0800000158.mm.1	-2,27	0,012913	0,438297		
TC0500000316.mm.1	-1,01	Snx17	JUC0500001501.mm.1	-2,27	0,018939	0,471376		
TC0900001658.mm.1	1,15	Clec3b	JUC0900007853.mm.1	-2,27	0,025991	0,499207		
TC0500000462.mm.1	-1,17	C1qtnf7	JUC0500002470.mm.1	-2,27	0,021176	0,480978		

TC0500000170.mm.1	1,39	Gsap	JUC0500000679.mm.1	-2,27	0,03545	0,52871		
TC0500000170.mm.1	1,39	Gsap	JUC0500000685.mm.1	-3,79	0,009255	0,414559		
TC0600002315.mm.1	1,21	Fkbp14	JUC0600009465.mm.1	-2,27	0,040149	0,541609		
TC0600001519.mm.1	1,07		JUC0600006511.mm.1	-2,27	0,026009	0,499257		
TC0300003181.mm.1	-1,06	Tnni3k	JUC0300013080.mm.1	-2,27	0,039758	0,540533		
TC0X00003411.mm.1	1,12	Ppp1r3f	JUC0X00005659.mm.1	-2,27	0,022649	0,487589		
TC0400003631.mm.1	1,07	Zcchc17	JUC0400015485.mm.1	-2,27	0,003664	0,357266		
TC1100001156.mm.1	2,94	Aldoc	PSR1100010738.mm.1	-2,28	0,010441	0,421595	Cassette Exon	0,25
TC1100001156.mm.1	2,94	Aldoc	PSR1100010742.mm.1	-2,41	0,005866	0,383835	Alternative 3' Acce	0,36
TC1100001156.mm.1	2,94	Aldoc	PSR1100010744.mm.1	-2,45	0,026714	0,502096	Alternative 3' Acce	0,36
TC1100001156.mm.1	2,94	Aldoc	JUC1100005645.mm.1	-3,93	0,002343	0,347317		
TC1100001156.mm.1	2,94	Aldoc	PSR1100010754.mm.1	-3,98	0,003937	0,359818	Alternative 5' Donc	0,48
TC1100001156.mm.1	2,94	Aldoc	JUC1100005647.mm.1	-3,99	0,003341	0,354243		
TC1400000923.mm.1	19,84	Mir124a-1; Mir3078; A93	PSR1400007348.mm.1	-2,28	0,031929	0,518048	Cassette Exon	0,12
TC1400000923.mm.1	19,84	Mir124a-1; Mir3078; A93	PSR1400007338.mm.1	-4,04	0,000879	0,311909		
TC1400000923.mm.1	19,84	Mir124a-1; Mir3078; A93	PSR1400007339.mm.1	-7,26	0,000612	0,304044	Intron Retention	0,38
TC1400000923.mm.1	19,84	Mir124a-1; Mir3078; A93	PSR1400007337.mm.1	-9,75	0,000854	0,311909		
TC1400000923.mm.1	19,84	Mir124a-1; Mir3078; A93	PSR1400007343.mm.1	-10	0,000486	0,298999	Alternative 5' Donc	0,47
TC1400000923.mm.1	19,84	Mir124a-1; Mir3078; A93	JUC1400003937.mm.1	-10,82	0,017432	0,463827		
TC0300003248.mm.1	2,94	Rnpc3	PSR0300021959.mm.1	-2,28	0,025724	0,498173	Alternative 3' Acce	0,22
TC0300003248.mm.1	2,94	Rnpc3	PSR0300021964.mm.1	-2,4	0,025697	0,498173	Alternative 3' Acce	0,19
TC0300003248.mm.1	2,94	Rnpc3	PSR0300021980.mm.1	-2,86	0,013604	0,443731	Alternative 5' Donc	0,3
TC0300003248.mm.1	2,94	Rnpc3	PSR0300021982.mm.1	-2,86	0,046581	0,556761	Alternative 5' Donc	0,3
TC0300003248.mm.1	2,94	Rnpc3	PSR0300021969.mm.1	-3,97	0,010104	0,418998	Alternative 5' Donc	0,41
TC0900000002.mm.1	1,76	Gm11168	JUC0900000010.mm.1	-2,28	0,027042	0,502979		
TC0900000002.mm.1	1,76	Gm11168	JUC0900000014.mm.1	-5,11	0,033405	0,522793		
TC0900000002.mm.1	1,76	Gm11168	PSR0900000021.mm.1	-6,5	0,023609	0,491012	Alternative 5' Donc	0,41
TC1500000982.mm.1	1,68	Cacnb3	PSR1500007700.mm.1	-2,28	0,045486	0,554313	Intron Retention	0,32
TC0800000124.mm.1	6,36	2810030D12Rik	PSR0800001146.mm.1	-2,28	0,002079	0,342181	Cassette Exon	0,2
TC0800000124.mm.1	6,36	2810030D12Rik	PSR0800001145.mm.1	-7,12	0,000048	0,24627	Alternative 3' Acce	0,28
TC0800000124.mm.1	6,36	2810030D12Rik	PSR0800001147.mm.1	-8,11	0,000134	0,270905	Cassette Exon	0,28
TC0X00001534.mm.1	1,3	Smc1a	PSR0X00009728.mm.1	-2,28	0,000602	0,304044	Alternative 5' Donc	0,19
TC0X00001534.mm.1	1,3	Smc1a	PSR0X00009738.mm.1	-3,03	0,002451	0,349135	Alternative 3' Acce	0,27
TC0600002550.mm.1	2,73	Hk2	PSR0600019732.mm.1	-2,28	0,036405	0,530878	Alternative 5' Donc	0,2
TC0600002550.mm.1	2,73	Hk2	PSR0600019729.mm.1	-2,31	0,019643	0,474876	Cassette Exon	0,25
TC0600002550.mm.1	2,73	Hk2	JUC0600010250.mm.1	-2,51	0,015704	0,455416		
TC0600002550.mm.1	2,73	Hk2	JUC0600010247.mm.1	-2,7	0,00332	0,354243		
TC0600002550.mm.1	2,73	Hk2	PSR0600019730.mm.1	-2,89	0,014745	0,450641	Cassette Exon	0,25
TC0600002550.mm.1	2,73	Hk2	JUC0600010259.mm.1	-4,6	0,037991	0,535386		
TC0600002550.mm.1	2,73	Hk2	JUC0600010261.mm.1	-4,85	0,013739	0,444758		
TC0700000421.mm.1	2,24	Exosc5	PSR0700003293.mm.1	-2,28	0,039742	0,540494	Intron Retention	0,23
TC0900002680.mm.1	2,06	Tmod2	PSR0900022171.mm.1	-2,28	0,047849	0,559373	Cassette Exon	0,21
TC0900002680.mm.1	2,06	Tmod2	JUC0900012422.mm.1	-4,71	0,027588	0,504485		
TC0800001533.mm.1	2,7	Rab4a	PSR0800012594.mm.1	-2,28	0,009852	0,417839	Alternative 5' Donc	0,2
TC1400002859.mm.1	1,13	Hacl1	PSR1400013073.mm.1	-2,28	0,001073	0,316484	Alternative 5' Donc	0,2
TC0400001726.mm.1	1,28	Mul1	PSR0400014120.mm.1	-2,28	0,03442	0,525828	Alternative 5' Donc	0,17
TC0200001803.mm.1	1,32	Ccndbp1	PSR0200013906.mm.1	-2,28	0,045372	0,55404	Alternative 5' Donc	0,15
TC0400003170.mm.1	2,16	Insl5	PSR0400025173.mm.1	-2,28	0,016112	0,457756	Cassette Exon	0,14

TC1100003472.mm.1	2,69	Ppm1e	PSR1100031890.mm.1	-2,28	0,028825	0,508407	Cassette Exon	0,14
TC1100003472.mm.1	2,69	Ppm1e	PSR1100031891.mm.1	-2,64	0,006558	0,39213	Cassette Exon	0,13
TC0400000736.mm.1	-1,21	Pappa	PSR0400005279.mm.1	-2,28	0,029884	0,51129	Cassette Exon	0,13
TC1100001352.mm.1	1,4	Bzrap1	PSR1100012684.mm.1	-2,28	0,009926	0,418378	Alternative 5' Donc	0,13
TC1100001352.mm.1	1,4	Bzrap1	JUC1100006655.mm.1	-3,81	0,014884	0,451777		
TC1400000347.mm.1	1,28	Ercc6	PSR1400003072.mm.1	-2,28	0,04537	0,55404	Alternative 5' Donc	0,13
TC1400000347.mm.1	1,28	Ercc6	JUC1400001835.mm.1	-2,75	0,038196	0,535805		
TC1600001311.mm.1	2,15	Dgcr8; Mir1306; Gm2457	PSR1600010774.mm.1	-2,28	0,039667	0,540294	Alternative 3' Acce	0,13
TC1600001311.mm.1	2,15	Dgcr8; Mir1306; Gm2457	JUC1600005626.mm.1	-2,3	0,012985	0,439245		
TC0300001715.mm.1	1,45		PSR0300013507.mm.1	-2,28	0,000101	0,262646	Cassette Exon	0,12
TC0300003091.mm.1	1,07	Wdr63	PSR0300024348.mm.1	-2,28	0,010215	0,419955	Cassette Exon	0,12
TC0300003091.mm.1	1,07	Wdr63	JUC0300012766.mm.1	-3,05	0,04799	0,559574		
TC1300002285.mm.1	1,45	Ice1	PSR1300014941.mm.1	-2,28	0,018769	0,470771	Cassette Exon	0,11
TC0200003407.mm.1	1,3	Dennd1a	JUC0200014765.mm.1	-2,28	0,049108	0,562191		
TC1300000990.mm.1	1,53	Ccnh	JUC1300003361.mm.1	-2,28	0,047526	0,558686		
TC0100000918.mm.1	-1,15		JUC0100004408.mm.1	-2,28	0,009386	0,415457		
TC0100000829.mm.1	1,7	Dgkd	JUC0100004057.mm.1	-2,28	0,016134	0,457846		
TC1800000969.mm.1	1,4	Rock1	JUC1800003983.mm.1	-2,28	0,017584	0,464264		
TC1500001716.mm.1	-1,72	2300005B03Rik	JUC1500007363.mm.1	-2,28	0,04459	0,552		
TC0200001959.mm.1	1,36	Sirpa	JUC0200007779.mm.1	-2,28	0,009525	0,416602		
TC0200001959.mm.1	1,36	Sirpa	JUC0200007789.mm.1	-2,29	0,018717	0,47064		
TC0200001959.mm.1	1,36	Sirpa	JUC0200007790.mm.1	-2,46	0,005038	0,37456		
TC0100003341.mm.1	-1,03	Pappa2; 1600012P17Rik	JUC0100015659.mm.1	-2,28	0,038314	0,536169		
TC0800002392.mm.1	-1,01	Klhl26	JUC0800009838.mm.1	-2,28	0,020173	0,477086		
TC0900001329.mm.1	1,3	Pik3r4	JUC0900005719.mm.1	-2,28	0,018866	0,471054		
TC0900001329.mm.1	1,3	Pik3r4	JUC0900005710.mm.1	-2,35	0,01894	0,471388		
TC0900000259.mm.1	1,07	Igsf9b	JUC0900001067.mm.1	-2,28	0,027628	0,504625		
TC0500003143.mm.1	-1,04		JUC0500015209.mm.1	-2,28	0,041147	0,544		
TC1100002862.mm.1	1,41	Flcn	JUC1100013515.mm.1	-2,28	0,037333	0,533573		
TC1100003046.mm.1	-1,07	Efnb3	JUC1100014608.mm.1	-2,28	0,001494	0,329143		
TC0400004118.mm.1	-1,24	Arhgef16	JUC0400017933.mm.1	-2,28	0,033023	0,521478		
TC0400001741.mm.1	1,17	Capzb	PSR0400014245.mm.1	-2,28	0,019808	0,475245		
TC0400001916.mm.1	-1,07		JUC0400008190.mm.1	-2,28	0,043567	0,550033		
TC0400002755.mm.1	-1,14	Susd1	JUC0400011620.mm.1	-2,28	0,038241	0,535883		
TC1100001725.mm.1	3,14	Mapt	PSR1100016303.mm.1	-2,29	0,046377	0,556329	Alternative 3' Acce	0,13
TC1100001725.mm.1	3,14	Mapt	JUC1100008448.mm.1	-2,65	0,019798	0,475212		
TC1100001725.mm.1	3,14	Mapt	PSR1100016315.mm.1	-2,81	0,029575	0,510456	Cassette Exon	0,1
TC1100001725.mm.1	3,14	Mapt	JUC1100008433.mm.1	-3,13	0,041791	0,545586		
TC1100001725.mm.1	3,14	Mapt	PSR1100016292.mm.1	-3,21	0,026206	0,500046	Cassette Exon	0,19
TC1100001725.mm.1	3,14	Mapt	PSR1100016311.mm.1	-3,24	0,026171	0,50003	Cassette Exon	0,25
TC1100001725.mm.1	3,14	Mapt	JUC1100008437.mm.1	-3,64	0,002593	0,349501		
TC1100001725.mm.1	3,14	Mapt	PSR1100016313.mm.1	-4,21	0,031069	0,515106	Alternative 3' Acce	0,38
TC1100001725.mm.1	3,14	Mapt	JUC1100008442.mm.1	-4,39	0,012608	0,435952		
TC1100001725.mm.1	3,14	Mapt	PSR1100016306.mm.1	-4,42	0,030592	0,51344	Cassette Exon	0,44
TC1100001725.mm.1	3,14	Mapt	PSR1100016307.mm.1	-5,89	0,013007	0,439291	Cassette Exon	0,45
TC1100001725.mm.1	3,14	Mapt	JUC1100008444.mm.1	-6,88	0,045094	0,553169		
TC0400000777.mm.1	2,38	Kdm4c	JUC0400002819.mm.1	-2,29	0,002031	0,341377		
TC0400000777.mm.1	2,38	Kdm4c	PSR0400005485.mm.1	-2,34	0,006538	0,391916	Cassette Exon	0,24

TC0400000777.mm.1	2,38	Kdm4c	PSR0400005512.mm.1	-2,45	0,013284	0,441719	Alternative 5' Donc	0,16
TC0400000777.mm.1	2,38	Kdm4c	PSR0400005488.mm.1	-2,47	0,004193	0,362939	Cassette Exon	0,17
TC0400000777.mm.1	2,38	Kdm4c	PSR0400005483.mm.1	-2,98	0,020657	0,478862	Cassette Exon	0,24
TC0400000777.mm.1	2,38	Kdm4c	PSR0400005491.mm.1	-3,12	0,011863	0,430929	Cassette Exon	0,3
TC0400000777.mm.1	2,38	Kdm4c	JUC0400002831.mm.1	-3,17	0,031638	0,517405		
TC0200001564.mm.1	1,79	Cstf3	PSR0200011752.mm.1	-2,29	0,03683	0,532211	Cassette Exon	0,12
TC0200001564.mm.1	1,79	Cstf3	PSR0200011718.mm.1	-2,72	0,020348	0,47734	Alternative 3' Acce	0,27
TC0200001564.mm.1	1,79	Cstf3	PSR0200011712.mm.1	-3	0,043879	0,550542	Alternative 3' Acce	0,28
TC1200000155.mm.1	2,82	5730507C01Rik	PSR1200001309.mm.1	-2,29	0,022718	0,487767	Alternative 5' Donc	0,14
TC1200000155.mm.1	2,82	5730507C01Rik	PSR1200001306.mm.1	-3,02	0,011398	0,427549	Alternative 3' Acce	0,26
TC0400000118.mm.1	2,43	Runx1t1	JUC0400000395.mm.1	-2,29	0,016352	0,458693		
TC0400000118.mm.1	2,43	Runx1t1	PSR0400000802.mm.1	-2,35	0,014106	0,447123	Alternative 5' Donc	0,25
TC0400000118.mm.1	2,43	Runx1t1	JUC0400000398.mm.1	-2,41	0,023971	0,492725		
TC0400000118.mm.1	2,43	Runx1t1	PSR0400000790.mm.1	-2,62	0,008882	0,410619	Cassette Exon	0,12
TC0400000118.mm.1	2,43	Runx1t1	PSR0400000798.mm.1	-2,69	0,04719	0,55807	Cassette Exon	0,06
TC0400000118.mm.1	2,43	Runx1t1	JUC0400000394.mm.1	-2,93	0,048276	0,560285		
TC0400000118.mm.1	2,43	Runx1t1	PSR0400000793.mm.1	-3,14	0,003667	0,357266	Cassette Exon	0,13
TC0200005004.mm.1	2,64	Gm14278	PSR0200042759.mm.1	-2,29	0,007982	0,403853	Cassette Exon	0,24
TC0200005004.mm.1	2,64	Gm14278	PSR0200042755.mm.1	-2,8	0,015796	0,455748	Cassette Exon	0,17
TC0200005004.mm.1	2,64	Gm14278	PSR0200042752.mm.1	-3,35	0,018459	0,468671	Cassette Exon	0,24
TC0200005004.mm.1	2,64	Gm14278	JUC0200022051.mm.1	-3,77	0,027463	0,504045		
TC0200005004.mm.1	2,64	Gm14278	JUC0200022041.mm.1	-5,41	0,024248	0,493315		
TC0200001203.mm.1	-1,24	Itgav	PSR0200009631.mm.1	-2,29	0,031775	0,517574	Intron Retention	0,23
TC1600000071.mm.1	1,73		PSR1600000545.mm.1	-2,29	0,000439	0,297771	Cassette Exon	0,22
TC1600000071.mm.1	1,73		JUC1600000280.mm.1	-3,16	0,041631	0,545202		
TC0400004003.mm.1	1,43	Mfn2	PSR0400033022.mm.1	-2,29	0,035633	0,528956	Alternative 3' Acce	0,19
TC0800001242.mm.1	-1,49	1810019D21Rik	PSR0800009963.mm.1	-2,29	0,010527	0,422224	Alternative 5' Donc	0,18
TC1600000119.mm.1	1,8		PSR1600000877.mm.1	-2,29	0,040054	0,541347	Cassette Exon	0,16
TC0200003005.mm.1	-1,08	Nsun6	PSR0200024813.mm.1	-2,29	0,036699	0,531756	Cassette Exon	0,14
TC0400003828.mm.1	1,4	Ubxn10	PSR0400031564.mm.1	-2,29	0,034904	0,52699	Cassette Exon	0,14
TC0500003264.mm.1	1,81	Ccdc92	PSR0500029496.mm.1	-2,29	0,018777	0,470789	Cassette Exon	0,12
TC0500003264.mm.1	1,81	Ccdc92	JUC0500016054.mm.1	-3,04	0,013584	0,443703		
TC1300001410.mm.1	1,67	Wdr37	PSR1300008849.mm.1	-2,29	0,002528	0,349501	Alternative 3' Acce	0,12
TC1300001410.mm.1	1,67	Wdr37	JUC1300004594.mm.1	-2,34	0,006851	0,393893		
TC1300001410.mm.1	1,67	Wdr37	JUC1300004597.mm.1	-5,34	0,000952	0,313363		
TC0200002489.mm.1	-1,08	Hnf4a	JUC0200010329.mm.1	-2,29	0,028911	0,50858		
TC0200005013.mm.1	-1,44	Samhd1	JUC0200022121.mm.1	-2,29	0,016989	0,461482		
TC1300000763.mm.1	1,17	2010111I01Rik; Mir23b; M	JUC1300002652.mm.1	-2,29	0,014115	0,447137		
TC1700001456.mm.1	-1,17	Pnldc1	JUC1700007106.mm.1	-2,29	0,018868	0,471099		
TC1700001585.mm.1	1,46	Zfp40	JUC1700007526.mm.1	-2,29	0,039268	0,538976		
TC1700002797.mm.1	1,31	Traf7	JUC1700007912.mm.1	-2,29	0,007297	0,398847		
TC1800001269.mm.1	1,88	Dpysl3; LOC100862305	JUC1800005258.mm.1	-2,29	0,037846	0,534803		
TC1500000526.mm.1	1,07	Gm6569	JUC1500002163.mm.1	-2,29	0,007398	0,399712		
TC1500000526.mm.1	1,07	Gm6569	JUC1500002164.mm.1	-3,07	0,00251	0,349237		
TC0900001197.mm.1	1,2		JUC0900005108.mm.1	-2,29	0,023375	0,490143		
TC0700001000.mm.1	-1,13	Adamts17	JUC0700004728.mm.1	-2,29	0,025872	0,498858		
TC0700001000.mm.1	-1,13	Adamts17	JUC0700004708.mm.1	-3	0,037149	0,533065		
TC0600001344.mm.1	1,33	Kdm5a	JUC0600005581.mm.1	-2,29	0,002362	0,34829		

TC0600001344.mm.1	1,33	Kdm5a	JUC0600005585.mm.1	-4,33	0,004837	0,371624		
TC0700001385.mm.1	1,17	Gdpd5	JUC0700006077.mm.1	-2,29	0,046259	0,556176		
TC0900001846.mm.1	-1,11	Col5a3	JUC0900008691.mm.1	-2,29	0,005998	0,386404		
TC0900001846.mm.1	-1,11	Col5a3	JUC0900008703.mm.1	-4,3	0,04319	0,548921		
TC0300003223.mm.1	1,11	Pmp2	JUC0300006896.mm.1	-2,29	0,030985	0,514765		
TC1000000653.mm.1	1,07	Tmem26	JUC1000002423.mm.1	-2,29	0,021498	0,482708		
TC0500001260.mm.1	6,66	Msi1	JUC0500005934.mm.1	-2,3	0,007694	0,402552		
TC0500001260.mm.1	6,66	Msi1	PSR0500010876.mm.1	-2,52	0,007519	0,401141	Cassette Exon	0,27
TC0500001260.mm.1	6,66	Msi1	JUC0500005919.mm.1	-3,01	0,015405	0,453973		
TC0500001260.mm.1	6,66	Msi1	JUC0500005920.mm.1	-3,03	0,008716	0,409276		
TC0500001260.mm.1	6,66	Msi1	JUC0500005935.mm.1	-3,06	0,01072	0,423723		
TC0500001260.mm.1	6,66	Msi1	JUC0500005937.mm.1	-3,23	0,017573	0,464258		
TC0500001260.mm.1	6,66	Msi1	PSR0500010862.mm.1	-3,31	0,00742	0,399901	Cassette Exon	0,31
TC0500001260.mm.1	6,66	Msi1	PSR0500010873.mm.1	-3,46	0,018244	0,467812	Alternative 5' Donc	0,23
TC0500001260.mm.1	6,66	Msi1	PSR0500010874.mm.1	-3,76	0,004911	0,372662	Cassette Exon	0,3
TC0500001260.mm.1	6,66	Msi1	PSR0500010871.mm.1	-4,13	0,006467	0,390452	Alternative 5' Donc	0,5
TC0500001260.mm.1	6,66	Msi1	JUC0500005932.mm.1	-5,23	0,002953	0,352501		
TC0500001260.mm.1	6,66	Msi1	PSR0500010879.mm.1	-5,47	0,003825	0,358333	Cassette Exon	0,27
TC0500001260.mm.1	6,66	Msi1	PSR0500010861.mm.1	-6,72	0,006214	0,387912	Cassette Exon	0,41
TC0500001260.mm.1	6,66	Msi1	PSR0500010858.mm.1	-6,83	0,010979	0,42525	Cassette Exon	0,37
TC0500001260.mm.1	6,66	Msi1	PSR0500010865.mm.1	-6,93	0,004219	0,363236	Alternative 5' Donc	0,48
TC0500001260.mm.1	6,66	Msi1	PSR0500010850.mm.1	-7,39	0,003336	0,354243	Cassette Exon	0,37
TC0500001260.mm.1	6,66	Msi1	PSR0500010883.mm.1	-7,59	0,005073	0,37524	Alternative 5' Donc	0,45
TC0500001260.mm.1	6,66	Msi1	JUC0500005936.mm.1	-7,71	0,005399	0,378916		
TC0500001260.mm.1	6,66	Msi1	PSR0500010848.mm.1	-9,3	0,004163	0,3623	Cassette Exon	0,37
TC0600003383.mm.1	4,04	St8sia1	PSR0600026686.mm.1	-2,3	0,011112	0,426	Cassette Exon	0,26
TC0600003383.mm.1	4,04	St8sia1	JUC0600013979.mm.1	-2,91	0,019786	0,475149		
TC0600003383.mm.1	4,04	St8sia1	PSR0600026687.mm.1	-3,61	0,000645	0,304044	Cassette Exon	0,38
TC0600003383.mm.1	4,04	St8sia1	JUC0600013980.mm.1	-5,22	0,004443	0,367065		
TC0200004245.mm.1	1,36	Tcp11l1	JUC0200018277.mm.1	-2,3	0,032714	0,520439		
TC0200004245.mm.1	1,36	Tcp11l1	PSR0200035735.mm.1	-2,84	0,008828	0,410293	Cassette Exon	0,17
TC0200004245.mm.1	1,36	Tcp11l1	PSR0200035745.mm.1	-3,31	0,012365	0,434849	Alternative 3' Acce	0,37
TC0500003271.mm.1	2,45	Scarb1	JUC0500016135.mm.1	-2,3	0,002847	0,352207		
TC0500003271.mm.1	2,45	Scarb1	PSR0500029587.mm.1	-2,33	0,008925	0,410972	Cassette Exon	0,15
TC0500003271.mm.1	2,45	Scarb1	JUC0500016139.mm.1	-2,39	0,007938	0,403819		
TC0500003271.mm.1	2,45	Scarb1	PSR0500029595.mm.1	-2,46	0,025221	0,496772	Alternative 5' Donc	0,16
TC0500003271.mm.1	2,45	Scarb1	PSR0500029614.mm.1	-2,55	0,003157	0,353892	Cassette Exon	0,07
TC0500003271.mm.1	2,45	Scarb1	JUC0500016138.mm.1	-3,05	0,01311	0,439759		
TC0500003271.mm.1	2,45	Scarb1	PSR0500029613.mm.1	-3,16	0,029947	0,511499	Alternative 3' Acce	0,1
TC0500003271.mm.1	2,45	Scarb1	PSR0500029616.mm.1	-3,18	0,006086	0,386691	Cassette Exon	0,29
TC0400003360.mm.1	1,01	Btbd19	PSR0400026875.mm.1	-2,3	0,040936	0,543423	Intron Retention	0,27
TC0300002297.mm.1	-1,09	Apoa1bp	PSR0300017373.mm.1	-2,3	0,027376	0,503961	Cassette Exon	0,21
TC0300002297.mm.1	-1,09	Apoa1bp	JUC0300009136.mm.1	-2,31	0,000658	0,304044		
TC1700001017.mm.1	1,57	Dus3l	JUC1700005234.mm.1	-2,3	0,014513	0,449545		
TC1700001017.mm.1	1,57	Dus3l	PSR1700009794.mm.1	-2,37	0,015843	0,456278	Alternative 3' Acce	0,21
TC0100002777.mm.1	-1,07		PSR0100022462.mm.1	-2,3	0,039522	0,539762	Alternative 3' Acce	0,2
TC0400000934.mm.1	1,24	Gm12637	PSR0400006220.mm.1	-2,3	0,021159	0,480937	Alternative 5' Donc	0,2
TC0300001176.mm.1	1,78	Olfm3	PSR0300009652.mm.1	-2,3	0,008449	0,406611	Alternative 3' Acce	0,13

TC0300001176.mm.1	1,78	Olfm3	PSR0300009656.mm.1	-2,61	0,01223	0,433556	Cassette Exon	0,15
TC0700003767.mm.1	1,26	Gm15623; Rsf1os2	PSR0700031706.mm.1	-2,3	0,01828	0,468049	Cassette Exon	0,13
TC0400002066.mm.1	1,48	Morn1	PSR0400017073.mm.1	-2,3	0,044342	0,551418	Cassette Exon	0,12
TC1600000041.mm.1	1,08		PSR1600000365.mm.1	-2,3	0,00581	0,383104	Cassette Exon	0,12
TC1800001603.mm.1	1,94	Ctif	PSR1800011588.mm.1	-2,3	0,006031	0,386586	Cassette Exon	0,12
TC0200003561.mm.1	-1,57		PSR0200030359.mm.1	-2,3	0,046965	0,557563	Alternative 3' Acce	0,12
TC1000000598.mm.1	1,38	Herc4	PSR1000004117.mm.1	-2,3	0,003019	0,353447	Alternative 3' Acce	0,11
TC0200003424.mm.1	1,34	Nr6a1	JUC0200014823.mm.1	-2,3	0,015872	0,456526		
TC0200003137.mm.1	-1,21	Slc34a3	JUC0200013035.mm.1	-2,3	0,024899	0,495661		
TC0200003137.mm.1	-1,21	Slc34a3	JUC0200013047.mm.1	-2,75	0,032866	0,521098		
TC0200003215.mm.1	-1,28	Vav2	JUC0200013703.mm.1	-2,3	0,003826	0,358339		
TC0300000750.mm.1	1,36	Gba	JUC0300002977.mm.1	-2,3	0,002218	0,345773		
TC0200004941.mm.1	1,35	Necab3	JUC0200021634.mm.1	-2,3	0,003611	0,356223		
TC0200004941.mm.1	1,35	Necab3	JUC0200021633.mm.1	-2,59	0,02245	0,487003		
TC0200004941.mm.1	1,35	Necab3	JUC0200021635.mm.1	-3,14	0,024882	0,49558		
TC1300000418.mm.1	-1,27		JUC1300001272.mm.1	-2,3	0,022104	0,485486		
TC1700001270.mm.1	1,34	Camkmt	JUC1700006434.mm.1	-2,3	0,013861	0,445426		
TC1700001674.mm.1	1,13	Metrn	JUC1700008334.mm.1	-2,3	0,049233	0,562541		
TC0200002429.mm.1	1,12	Ralgapb	JUC0200009990.mm.1	-2,3	0,020162	0,477086		
TC1600001922.mm.1	-1,33	Nrip1	JUC1600007733.mm.1	-2,3	0,009194	0,413763		
TC0900000590.mm.1	1,17	Zw10	JUC0900002163.mm.1	-2,3	0,01272	0,43654		
TC0700000138.mm.1	1,24	Zfp606	JUC0700000517.mm.1	-2,3	0,007919	0,403819		
TC1100000011.mm.1	1,28	8430429K09Rik	JUC1100000081.mm.1	-2,3	0,005424	0,379427		
TC1100000011.mm.1	1,28	8430429K09Rik	JUC1100000083.mm.1	-2,3	0,039581	0,539856		
TC1100000011.mm.1	1,28	8430429K09Rik	JUC1100000088.mm.1	-2,78	0,003817	0,358136		
TC1100000011.mm.1	1,28	8430429K09Rik	JUC1100000086.mm.1	-3,38	0,001065	0,316361		
TC0300002580.mm.1	1,23	Wdr3	JUC0300010464.mm.1	-2,3	0,022613	0,487423		
TC0900003157.mm.1	-1,22	AU023762	JUC0900014997.mm.1	-2,3	0,044419	0,551557		
TC0X00002851.mm.1	-1,01	Brwd3	JUC0X00009195.mm.1	-2,3	0,038329	0,53622		
TC1300000148.mm.1	4,89	Elmo1	PSR1300001032.mm.1	-2,31	0,012376	0,434939	Cassette Exon	0,13
TC1300000148.mm.1	4,89	Elmo1	PSR1300001027.mm.1	-2,88	0,027236	0,503642	Cassette Exon	0,33
TC1300000148.mm.1	4,89	Elmo1	PSR1300001007.mm.1	-3,54	0,0257	0,498173	Cassette Exon	0,26
TC1300000148.mm.1	4,89	Elmo1	PSR1300001015.mm.1	-3,61	0,02088	0,479589	Cassette Exon	0,53
TC1300000148.mm.1	4,89	Elmo1	JUC1300000585.mm.1	-4,05	0,043867	0,550524		
TC1300000148.mm.1	4,89	Elmo1	PSR1300001025.mm.1	-4,29	0,035565	0,528805	Cassette Exon	0,55
TC1300000148.mm.1	4,89	Elmo1	PSR1300001019.mm.1	-4,63	0,005492	0,379939	Cassette Exon	0,55
TC1300000148.mm.1	4,89	Elmo1	PSR1300001008.mm.1	-4,66	0,003631	0,3566	Cassette Exon	0,28
TC1300000148.mm.1	4,89	Elmo1	PSR1300001005.mm.1	-4,92	0,006752	0,393343	Cassette Exon	0,26
TC1300000148.mm.1	4,89	Elmo1	PSR1300001021.mm.1	-5,07	0,00912	0,413305	Cassette Exon	0,41
TC1300000148.mm.1	4,89	Elmo1	JUC1300000593.mm.1	-5,46	0,010713	0,423598		
TC1300000148.mm.1	4,89	Elmo1	PSR1300001012.mm.1	-5,71	0,003357	0,354243	Cassette Exon	0,55
TC1300000148.mm.1	4,89	Elmo1	PSR1300001028.mm.1	-5,88	0,004938	0,373093	Alternative 3' Acce	0,48
TC1300000148.mm.1	4,89	Elmo1	JUC1300000599.mm.1	-5,97	0,003594	0,356223		
TC1300000148.mm.1	4,89	Elmo1	PSR1300001020.mm.1	-6,06	0,019811	0,475266	Cassette Exon	0,55
TC1300000148.mm.1	4,89	Elmo1	PSR1300001010.mm.1	-6,16	0,012806	0,437316	Cassette Exon	0,55
TC1300000148.mm.1	4,89	Elmo1	JUC1300000597.mm.1	-6,34	0,004508	0,368648		
TC1300000148.mm.1	4,89	Elmo1	PSR1300001013.mm.1	-6,39	0,008768	0,409781	Cassette Exon	0,55
TC1300000148.mm.1	4,89	Elmo1	JUC1300000590.mm.1	-6,45	0,003671	0,357266		

TC1300000148.mm.1	4,89	Elmo1	PSR1300001022.mm.1	-6,58	0,006144	0,387745	Cassette Exon	0,41
TC1300000148.mm.1	4,89	Elmo1	PSR1300001014.mm.1	-6,6	0,004845	0,37168	Cassette Exon	0,55
TC1300000148.mm.1	4,89	Elmo1	JUC1300000589.mm.1	-6,75	0,004226	0,363295		
TC1300000148.mm.1	4,89	Elmo1	PSR1300001018.mm.1	-7,06	0,002527	0,349501	Cassette Exon	0,55
TC1300000148.mm.1	4,89	Elmo1	JUC1300000591.mm.1	-7,46	0,003616	0,356223		
TC1300000148.mm.1	4,89	Elmo1	JUC1300000587.mm.1	-7,61	0,005667	0,381922		
TC1300000148.mm.1	4,89	Elmo1	PSR1300001009.mm.1	-7,78	0,009864	0,417945	Cassette Exon	0,41
TC1300000148.mm.1	4,89	Elmo1	PSR1300001024.mm.1	-7,81	0,002849	0,352207	Cassette Exon	0,55
TC1300000148.mm.1	4,89	Elmo1	PSR1300001026.mm.1	-7,95	0,015081	0,452418	Cassette Exon	0,43
TC1300000148.mm.1	4,89	Elmo1	JUC1300000594.mm.1	-8,78	0,010726	0,423745		
TC1300000148.mm.1	4,89	Elmo1	JUC1300000586.mm.1	-9,08	0,007932	0,403819		
TC1300000148.mm.1	4,89	Elmo1	PSR1300001023.mm.1	-9,16	0,012009	0,432286	Cassette Exon	0,41
TC1300000148.mm.1	4,89	Elmo1	PSR1300001011.mm.1	-9,21	0,010754	0,423859	Cassette Exon	0,55
TC1300000148.mm.1	4,89	Elmo1	PSR1300001017.mm.1	-9,51	0,014313	0,448078	Cassette Exon	0,55
TC1300000148.mm.1	4,89	Elmo1	JUC1300000592.mm.1	-10,83	0,007873	0,403545		
TC1300000148.mm.1	4,89	Elmo1	JUC1300000596.mm.1	-10,94	0,00251	0,349237		
TC1300000148.mm.1	4,89	Elmo1	JUC1300000588.mm.1	-12,31	0,012798	0,437255		
TC0900003112.mm.1	5,78	Klhl18	PSR0900026296.mm.1	-2,31	0,046443	0,556547	Cassette Exon	0,21
TC0900003112.mm.1	5,78	Klhl18	JUC0900014763.mm.1	-3,16	0,015214	0,45279		
TC0900003112.mm.1	5,78	Klhl18	PSR0900026303.mm.1	-5,03	0,011434	0,427663	Alternative 5' Donc	0,39
TC0900000252.mm.1	1,93	B3gat1	PSR0900001871.mm.1	-2,31	0,041322	0,544282	Intron Retention	0,32
TC0900000252.mm.1	1,93	B3gat1	JUC0900001009.mm.1	-3,51	0,0057	0,382524		
TC1600001665.mm.1	1,46	Abhd10	JUC1600007108.mm.1	-2,31	0,015725	0,455416		
TC1600001665.mm.1	1,46	Abhd10	PSR1600013633.mm.1	-3,12	0,020567	0,478392	Intron Retention	0,28
TC0800000973.mm.1	8,49	Rtbdn	PSR0800007380.mm.1	-2,31	0,038472	0,536441	Alternative 3' Acce	0,13
TC0800000973.mm.1	8,49	Rtbdn	PSR0800007384.mm.1	-2,49	0,009034	0,412736	Alternative 3' Acce	0,27
TC1200001676.mm.1	2,35	Lrrn3	PSR1200011634.mm.1	-2,31	0,024951	0,495859	Cassette Exon	0,1
TC1200001676.mm.1	2,35	Lrrn3	PSR1200011631.mm.1	-4,06	0,002933	0,352289	Cassette Exon	0,27
TC0700001989.mm.1	1,32	Lrrc27	PSR0700017428.mm.1	-2,31	0,029817	0,511132	Alternative 3' Acce	0,18
TC0700001989.mm.1	1,32	Lrrc27	JUC0700009254.mm.1	-3,53	0,037681	0,534394		
TC0700001989.mm.1	1,32	Lrrc27	PSR0700017427.mm.1	-3,6	0,003235	0,354243	Cassette Exon	0,24
TC1700002230.mm.1	-1,01		PSR1700021084.mm.1	-2,31	0,00072	0,306015	Alternative 5' Donc	0,21
TC0100003279.mm.1	1,39	Dhx9	PSR0100026681.mm.1	-2,31	0,005525	0,380518	Intron Retention	0,18
TC1800000944.mm.1	1,27	Arhgap12	PSR1800006892.mm.1	-2,31	0,021011	0,480143	Alternative 3' Acce	0,17
TC1800000944.mm.1	1,27	Arhgap12	JUC1800003819.mm.1	-2,74	0,012155	0,432923		
TC0X00002053.mm.1	1,77	sept-06	JUC0X00006649.mm.1	-2,31	0,03225	0,519011		
TC0X00002053.mm.1	1,77	sept-06	PSR0X00013038.mm.1	-2,61	0,01257	0,435818	Cassette Exon	0,15
TC0X00000061.mm.1	-1,16	Prickle3	PSR0X00000326.mm.1	-2,31	0,022947	0,488667	Cassette Exon	0,12
TC0X00000094.mm.1	-1,32	Gm6592	PSR0X00000750.mm.1	-2,31	0,007661	0,402377	Cassette Exon	0,12
TC1400001661.mm.1	-1,4	Itih3	JUC1400006812.mm.1	-2,31	0,01148	0,428022		
TC1300001680.mm.1	1,02	1700092E19Rik	JUC1300005351.mm.1	-2,31	0,020301	0,477245		
TC0100002954.mm.1	1,59	Ptpn4	JUC0100013492.mm.1	-2,31	0,044049	0,550824		
TC1900001552.mm.1	1,52	Chuk	JUC1900007601.mm.1	-2,31	0,015754	0,455437		
TC0200000719.mm.1	1,07	Arpc5l	JUC0200003207.mm.1	-2,31	0,025685	0,498173		
TC1600000664.mm.1	-1,13	Cd200r4	JUC1600003032.mm.1	-2,31	0,042743	0,548097		
TC0500001448.mm.1	1,46	Ddx55	JUC0500007118.mm.1	-2,31	0,047583	0,558743		
TC0600003496.mm.1	-1,14	Trbj1-1; Trbj1-2; Trbj1-3; T	JUC0600001725.mm.1	-2,31	0,020909	0,479756		
TC0300001475.mm.1	1,18	Odf2l	JUC0300006230.mm.1	-2,31	0,044963	0,552881		

TC0Y00000380.mm.1	-1,02 Orly	JUC0Y00001637.mm.1	-2,31	0,000806	0,311765		
TC0Y00000389.mm.1	-1,02 Orly	JUC0Y00001644.mm.1	-2,31	0,000806	0,311765		
TC1000001063.mm.1	-1,17 Pah	JUC1000004306.mm.1	-2,31	0,006889	0,394489		
TC1300001266.mm.1	1,96 Slc38a9	PSR1300008037.mm.1	-2,32	0,023272	0,49004	Alternative 5' Donc	0,21
TC1300001266.mm.1	1,96 Slc38a9	PSR1300008036.mm.1	-2,35	0,010348	0,421002	Alternative 5' Donc	0,21
TC1300001266.mm.1	1,96 Slc38a9	JUC1300004190.mm.1	-3,32	0,044093	0,550916		
TC1300001266.mm.1	1,96 Slc38a9	PSR1300008018.mm.1	-3,52	0,005376	0,378747	Alternative 3' Acce	0,4
TC0300001322.mm.1	1,89 Etnppl	PSR0300010707.mm.1	-2,32	0,035069	0,527574	Intron Retention	0,33
TC0300001322.mm.1	1,89 Etnppl	JUC0300005612.mm.1	-2,87	0,01172	0,429969		
TC0400000774.mm.1	3,42 Frmd3	PSR0400005479.mm.1	-2,32	0,007718	0,402552	Cassette Exon	0,17
TC0400000774.mm.1	3,42 Frmd3	PSR0400005478.mm.1	-2,36	0,000287	0,28803	Cassette Exon	0,15
TC0400000774.mm.1	3,42 Frmd3	PSR0400005473.mm.1	-2,45	0,023728	0,491556	Alternative 3' Acce	0,23
TC0400000774.mm.1	3,42 Frmd3	PSR0400005457.mm.1	-2,75	0,005952	0,385276	Alternative 3' Acce	0,23
TC0400000774.mm.1	3,42 Frmd3	PSR0400005456.mm.1	-3,17	0,007711	0,402552	Alternative 3' Acce	0,23
TC0400000774.mm.1	3,42 Frmd3	PSR0400005463.mm.1	-3,26	0,009221	0,41411	Alternative 3' Acce	0,31
TC0700003107.mm.1	3,7 Gabrg3	PSR0700028193.mm.1	-2,32	0,018966	0,471398	Cassette Exon	0,12
TC0700003107.mm.1	3,7 Gabrg3	PSR0700028198.mm.1	-3,9	0,03029	0,512627	Cassette Exon	0,3
TC1500001846.mm.1	2,72 Elfn2	JUC1500008211.mm.1	-2,32	0,0311	0,515257		
TC1500001846.mm.1	2,72 Elfn2	PSR1500014400.mm.1	-3,29	0,002887	0,352207	Cassette Exon	0,12
TC1500001846.mm.1	2,72 Elfn2	PSR1500014401.mm.1	-3,49	0,005476	0,379939	Cassette Exon	0,29
TC0300002711.mm.1	1,75 Slc6a17	JUC0300010980.mm.1	-2,32	0,032051	0,518459		
TC0300002711.mm.1	1,75 Slc6a17	JUC0300010968.mm.1	-2,36	0,018424	0,468579		
TC0300002711.mm.1	1,75 Slc6a17	PSR0300021083.mm.1	-3,64	0,002246	0,34578	Alternative 3' Acce	0,26
TC1100002004.mm.1	1,18 Endov	PSR1100019210.mm.1	-2,32	0,044502	0,551748	Alternative 5' Donc	0,18
TC1100002004.mm.1	1,18 Endov	PSR1100019231.mm.1	-2,51	0,007463	0,400381	Alternative 5' Donc	0,24
TC1500000297.mm.1	2,34 Baalc	JUC1500001235.mm.1	-2,32	0,01521	0,452773		
TC1500000297.mm.1	2,34 Baalc	PSR1500002133.mm.1	-2,52	0,004865	0,371692	Alternative 5' Donc	0,24
TC1500000297.mm.1	2,34 Baalc	PSR1500002125.mm.1	-2,54	0,015613	0,455082	Cassette Exon	0,2
TC0200004507.mm.1	-1,5 Duoxa1	PSR0200038300.mm.1	-2,32	0,034359	0,525572	Alternative 3' Acce	0,21
TC0700004644.mm.1	1,32 Zfp82	PSR0700024281.mm.1	-2,32	0,041853	0,545612	Alternative 5' Donc	0,21
TC0600000698.mm.1	1,25 Lancl2	PSR0600005380.mm.1	-2,32	0,013003	0,439291	Alternative 3' Acce	0,2
TC1200002037.mm.1	1,29 1700085C21Rik	PSR1200014012.mm.1	-2,32	0,039841	0,540922	Cassette Exon	0,2
TC1200002037.mm.1	1,29 1700085C21Rik	JUC1200007714.mm.1	-2,65	0,038675	0,537102		
TC1500002118.mm.1	1,94 Nell2	PSR1500017041.mm.1	-2,32	0,025848	0,498657	Cassette Exon	0,18
TC1500002118.mm.1	1,94 Nell2	JUC1500009699.mm.1	-4,6	0,003887	0,359225		
TC0800000796.mm.1	1,49 Slc27a1	PSR0800005743.mm.1	-2,32	0,002902	0,352207	Cassette Exon	0,14
TC1100004222.mm.1	1,13 Pycr1	PSR1100040373.mm.1	-2,32	0,009277	0,414799	Alternative 3' Acce	0,14
TC0900002179.mm.1	1,27 Tmem25	PSR0900018131.mm.1	-2,32	0,012777	0,437233	Cassette Exon	0,13
TC0300002219.mm.1	3,63 Gucy1b3	PSR0300016684.mm.1	-2,32	0,007117	0,397044	Cassette Exon	0,12
TC0X00001787.mm.1	1,42 Hdac6	PSR0X00011355.mm.1	-2,32	0,040945	0,543423	Alternative 3' Acce	0,12
TC1100003221.mm.1	1,09 Rpa1	PSR1100029628.mm.1	-2,32	0,024074	0,49304	Cassette Exon	0,12
TC0200003046.mm.1	1,01 Gm20539	PSR0200025054.mm.1	-2,32	0,044295	0,551351	Cassette Exon	0,1
TC0400004042.mm.1	1,36 Nmnat1	PSR0400033591.mm.1	-2,32	0,049983	0,564085	Cassette Exon	0,1
TC1400001856.mm.1	-1,09 1700001F09Rik	JUC1400007740.mm.1	-2,32	0,002578	0,349501		
TC0300001055.mm.1	1,42 Syt6	JUC0300004412.mm.1	-2,32	0,00777	0,402804		
TC1200000204.mm.1	1,06	JUC1200000930.mm.1	-2,32	0,03507	0,527574		
TC1300000382.mm.1	1,16 1110046J04Rik	JUC1300001158.mm.1	-2,32	0,009847	0,417839		
TC0100002273.mm.1	-1,03	JUC0100010384.mm.1	-2,32	0,037024	0,532502		

TC1900000612.mm.1	1,36	Zfyve27	JUC1900002931.mm.1	-2,32	0,002005	0,341091		
TC1900000612.mm.1	1,36	Zfyve27	JUC1900002933.mm.1	-2,84	0,00467	0,370181		
TC0200002261.mm.1	1,38	Nsfl1c	JUC0200008892.mm.1	-2,32	0,048997	0,561857		
TC0500003286.mm.1	-1,03	Tmem132d	JUC0500016219.mm.1	-2,32	0,028916	0,50858		
TC0700001851.mm.1	1,29	Srcap	JUC0700008339.mm.1	-2,32	0,019643	0,474876		
TC0700001851.mm.1	1,29	Srcap	JUC0700008336.mm.1	-3,19	0,041432	0,544646		
TC1000002224.mm.1	2,13	Ddit4	JUC1000008460.mm.1	-2,32	0,03158	0,517177		
TC1100003652.mm.1	2,11	Pip4k2b	JUC1100017585.mm.1	-2,32	0,036875	0,532257		
TC1100003674.mm.1	1,27	Pgap3	JUC1100017736.mm.1	-2,32	0,021068	0,480303		
TC0X00003438.mm.1	1,39	Zmat1	JUC0X00009499.mm.1	-2,32	0,012581	0,435924		
TC0500002578.mm.1	11,76	Cnga1	PSR0500022802.mm.1	-2,33	0,00509	0,375607	Cassette Exon	0,17
TC0500002578.mm.1	11,76	Cnga1	PSR0500022799.mm.1	-2,81	0,01019	0,419711	Cassette Exon	0,44
TC0500002578.mm.1	11,76	Cnga1	JUC0500012388.mm.1	-4,14	0,007219	0,39826		
TC0500002578.mm.1	11,76	Cnga1	JUC0500012391.mm.1	-7,23	0,002754	0,350151		
TC0500002578.mm.1	11,76	Cnga1	PSR0500022806.mm.1	-9,83	0,00459	0,369794	Cassette Exon	0,31
TC0500002578.mm.1	11,76	Cnga1	JUC0500012387.mm.1	-11,91	0,007948	0,403819		
TC0500002578.mm.1	11,76	Cnga1	PSR0500022800.mm.1	-15,69	0,002577	0,349501	Cassette Exon	0,41
TC0200004762.mm.1	1,81	Dzank1	PSR0200040798.mm.1	-2,33	0,013939	0,446203	Alternative 3' Acce	0,2
TC0200004762.mm.1	1,81	Dzank1	PSR0200040774.mm.1	-2,56	0,002501	0,349189	Intron Retention	0,36
TC0200004762.mm.1	1,81	Dzank1	JUC0200021138.mm.1	-2,68	0,004392	0,366156		
TC0200004762.mm.1	1,81	Dzank1	PSR0200040787.mm.1	-3,89	0,001277	0,322251	Alternative 3' Acce	0,37
TC0X00000166.mm.1	1,33	Ddx3x	PSR0X00001066.mm.1	-2,33	0,033333	0,522438	Intron Retention	0,33
TC0Y00000493.mm.1	1,42	Ssty2; Gm21257	PSR0Y00002545.mm.1	-2,33	0,028397	0,507058	Alternative 3' Acce	0,21
TC1500001051.mm.1	-1,33	Dazap2; Gm2444	PSR1500008315.mm.1	-2,33	0,020125	0,47686	Alternative 5' Donc	0,2
TC1100003775.mm.1	-1,32	Acly	PSR1100034709.mm.1	-2,33	0,023199	0,490032	Alternative 5' Donc	0,19
TC1300000662.mm.1	1,37	Prr7	PSR1300004394.mm.1	-2,33	0,012215	0,433321	Alternative 5' Donc	0,19
TC0X00003401.mm.1	2,13	Gprasp2	PSR0X00008224.mm.1	-2,33	0,017721	0,465209	Alternative 3' Acce	0,11
TC0X00003401.mm.1	2,13	Gprasp2	PSR0X00008229.mm.1	-2,45	0,013421	0,442624	Intron Retention	0,18
TC1500000766.mm.1	1	Serhl	PSR1500006034.mm.1	-2,33	0,048324	0,560488	Alternative 5' Donc	0,18
TC0100002016.mm.1	-1,47	Gm16070; LOC102634913	PSR0100016473.mm.1	-2,33	0,040332	0,541841	Cassette Exon	0,12
TC0200005462.mm.1	-1,17	Entpd2	PSR0200046644.mm.1	-2,33	0,039332	0,539324	Cassette Exon	0,11
TC1200001236.mm.1	-1,12	Exoc3l4	JUC1200004772.mm.1	-2,33	0,038693	0,537169		
TC1200000659.mm.1	1,19		JUC1200002553.mm.1	-2,33	0,019772	0,475054		
TC0300000658.mm.1	1,42	Fbxw7	JUC0300002280.mm.1	-2,33	0,012277	0,434165		
TC1300001725.mm.1	1,28	Cdkal1	JUC1300005464.mm.1	-2,33	0,032958	0,521223		
TC1200002596.mm.1	1,34	Ighv6-6	JUC1200010022.mm.1	-2,33	0,020966	0,479982		
TC1300000547.mm.1	-1,16	G630093K05Rik	JUC1300001729.mm.1	-2,33	0,02792	0,505612		
TC0100003026.mm.1	-1,83	Mcm6	JUC0100013751.mm.1	-2,33	0,036403	0,530878		
TC1700001814.mm.1	1,11	Tmprss3	JUC1700008972.mm.1	-2,33	0,006663	0,393187		
TC1500001661.mm.1	1,14	Zfat	JUC1500007065.mm.1	-2,33	0,00142	0,327268		
TC1500001202.mm.1	-1,02	Brix1	JUC1500005474.mm.1	-2,33	0,01304	0,439418		
TC1600000448.mm.1	1,24	Tnk2	JUC1600002173.mm.1	-2,33	0,044841	0,552566		
TC0200000515.mm.1	1,41	Odf2	JUC0200001931.mm.1	-2,33	0,0017	0,33549		
TC0800000441.mm.1	-1,12	D8Ertd82e	JUC0800001664.mm.1	-2,33	0,041461	0,544702		
TC0700000015.mm.1	1,17	Prpf31	JUC0700000076.mm.1	-2,33	0,035757	0,529087		
TC0500003376.mm.1	1,05	Lat2	JUC0500016566.mm.1	-2,33	0,027402	0,503961		
TC1000003209.mm.1	-1,14	Vmn2r87	JUC1000013199.mm.1	-2,33	0,045804	0,554863		
TC1100000726.mm.1	-1,45	Nlrp3	JUC1100002980.mm.1	-2,33	0,008244	0,405976		

TC0400000529.mm.1	-1,06	Nr4a3	JUC0400001938.mm.1	-2,33	0,046145	0,555804		
TC1000002437.mm.1	-1,02	Trpm2; Gm22871; Gm223	JUC1000009687.mm.1	-2,33	0,016245	0,458312		
TC1100002454.mm.1	1,01	Gm12106	JUC1100011889.mm.1	-2,33	0,044257	0,551268		
TC1100003563.mm.1	-1,01	Spata20	JUC1100017044.mm.1	-2,33	0,039722	0,540494		
TC0X00003231.mm.1	-1,34	Magea2	JUC0X00010191.mm.1	-2,33	0,039882	0,54093		
TC0100001313.mm.1	3,36	B3galt2	PSR0100010918.mm.1	-2,34	0,022974	0,48884		
TC0100001313.mm.1	3,36	B3galt2	PSR0100010910.mm.1	-3,64	0,021714	0,484104	Alternative 3' Acce	0,46
TC0100001313.mm.1	3,36	B3galt2	PSR0100010908.mm.1	-4,44	0,009557	0,416829	Alternative 3' Acce	0,46
TC0100001313.mm.1	3,36	B3galt2	PSR0100010909.mm.1	-5,53	0,009461	0,416514	Alternative 3' Acce	0,46
TC0500000188.mm.1	3,74	Lhfpl3	PSR0500001365.mm.1	-2,34	0,000761	0,308387	Cassette Exon	0,26
TC0500000188.mm.1	3,74	Lhfpl3	PSR0500001367.mm.1	-2,71	0,01286	0,437783	Cassette Exon	0,41
TC0500000188.mm.1	3,74	Lhfpl3	JUC0500000785.mm.1	-6,06	0,021763	0,484349		
TC0500000188.mm.1	3,74	Lhfpl3	JUC0500000786.mm.1	-6,07	0,008817	0,410077		
TC0500000188.mm.1	3,74	Lhfpl3	JUC0500000783.mm.1	-10,35	0,000839	0,311886		
TC0900002757.mm.1	1,32	Phip	PSR0900022794.mm.1	-2,34	0,036831	0,532211	Intron Retention	0,34
TC1600001919.mm.1	2,18	Samsn1	PSR1600014963.mm.1	-2,34	0,006357	0,389469	Cassette Exon	0,12
TC1600001919.mm.1	2,18	Samsn1	PSR1600014962.mm.1	-2,81	0,014257	0,447947	Cassette Exon	0,24
TC1600001919.mm.1	2,18	Samsn1	PSR1600014954.mm.1	-3,03	0,017767	0,46545	Alternative 3' Acce	0,31
TC1600001919.mm.1	2,18	Samsn1	PSR1600014959.mm.1	-3,15	0,044208	0,551188	Alternative 5' Donc	0,34
TC1600001919.mm.1	2,18	Samsn1	PSR1600014967.mm.1	-3,92	0,000416	0,297771	Cassette Exon	0,27
TC1600001919.mm.1	2,18	Samsn1	JUC1600007722.mm.1	-4,12	0,002394	0,348564		
TC1600001919.mm.1	2,18	Samsn1	JUC1600007721.mm.1	-5,66	0,000348	0,290082		
TC1600001919.mm.1	2,18	Samsn1	JUC1600007723.mm.1	-5,98	0,007268	0,398281		
TC1600000564.mm.1	2,37	Igsf11	JUC1600002812.mm.1	-2,34	0,04799	0,559574		
TC1600000564.mm.1	2,37	Igsf11	PSR1600005347.mm.1	-2,39	0,038737	0,537362	Cassette Exon	0,1
TC1600000564.mm.1	2,37	Igsf11	JUC1600002814.mm.1	-3,04	0,024791	0,495094		
TC1600000564.mm.1	2,37	Igsf11	PSR1600005346.mm.1	-5,27	0,015163	0,452699	Alternative 3' Acce	0,26
TC1600000564.mm.1	2,37	Igsf11	JUC1600002816.mm.1	-8,53	0,002328	0,346856		
TC0400003234.mm.1	1,24	Scp2	JUC0400013412.mm.1	-2,34	0,001216	0,322251		
TC0400003234.mm.1	1,24	Scp2	PSR0400025754.mm.1	-3,23	0,019227	0,472971	Cassette Exon	0,24
TC0100000523.mm.1	-1,22	Zdbf2; Gm11607	PSR0100004185.mm.1	-2,34	0,000328	0,28927	Alternative 3' Acce	0,21
TC1600001151.mm.1	1,14	Glyr1	PSR1600009195.mm.1	-2,34	0,034154	0,524919	Alternative 3' Acce	0,2
TC1200000069.mm.1	1,12	Sdc1	PSR1200000752.mm.1	-2,34	0,001298	0,322929	Alternative 5' Donc	0,18
TC1000002374.mm.1	1,75	Gucd1	PSR1000016909.mm.1	-2,34	0,018139	0,467372	Cassette Exon	0,17
TC1000002374.mm.1	1,75	Gucd1	JUC1000009232.mm.1	-2,58	0,003164	0,353901		
TC1000002374.mm.1	1,75	Gucd1	JUC1000009227.mm.1	-3,13	0,001205	0,322249		
TC0300002736.mm.1	-1,58	Sypl2	PSR0300021481.mm.1	-2,34	0,025962	0,499161	Cassette Exon	0,16
TC0X00002442.mm.1	-1,13	Irak1; Mir718; Mir5132	PSR0X00015367.mm.1	-2,34	0,003686	0,357266	Cassette Exon	0,12
TC0400001582.mm.1	1,12	Snord99; Snhg12; Snora6:	PSR0400012426.mm.1	-2,34	0,027846	0,505597	Intron Retention	0,1
TC1400000108.mm.1	-1,09	Gm11100; LOC102633458	JUC1400000527.mm.1	-2,34	0,024317	0,49362		
TC1200000775.mm.1	1,51	Rgs6	JUC1200003129.mm.1	-2,34	0,009902	0,418332		
TC1300001083.mm.1	-1,02		JUC1300003598.mm.1	-2,34	0,022255	0,485896		
TC1700001813.mm.1	-1,5	Tff1	JUC1700008966.mm.1	-2,34	0,03202	0,518418		
TC1900001272.mm.1	1,03		JUC1900006305.mm.1	-2,34	0,029563	0,510436		
TC0100000836.mm.1	-1,46	Ugt1a2; Ugt1a6a; Ugt1a6	JUC0100004068.mm.1	-2,34	0,019016	0,471745		
TC0100000048.mm.1	1,12	Cspp1	JUC0100000228.mm.1	-2,34	0,023641	0,491117		
TC0100000048.mm.1	1,12	Cspp1	JUC0100000218.mm.1	-2,62	0,011569	0,429011		
TC1800001628.mm.1	1,29	8030462N17Rik	JUC1800006526.mm.1	-2,34	0,02266	0,487609		

TC1800001175.mm.1	-1,24	Slc23a1	JUC1800004825.mm.1	-2,34	0,020909	0,479756		
TC0100001383.mm.1	1,07		JUC0100006606.mm.1	-2,34	0,049243	0,56256		
TC1500000515.mm.1	1,13	Chrac1	JUC1500002114.mm.1	-2,34	0,001279	0,322251		
TC1700000305.mm.1	-1,17	Vmn2r113	JUC1700001194.mm.1	-2,34	0,015253	0,452847		
TC0800002244.mm.1	1,28	Cep44	JUC0800009300.mm.1	-2,34	0,049487	0,563298		
TC0500001779.mm.1	1,08	Cdk8	JUC0500008978.mm.1	-2,34	0,008222	0,405976		
TC0900000807.mm.1	-1,01	1110036E04Rik	JUC0900003229.mm.1	-2,34	0,029784	0,511132		
TC0500000371.mm.1	-1,07		JUC0500001944.mm.1	-2,34	0,003174	0,354243		
TC0900001422.mm.1	1,52	Ip6k2	JUC0900006346.mm.1	-2,34	0,026688	0,502057		
TC0900001422.mm.1	1,52	Ip6k2	JUC0900006352.mm.1	-3,31	0,024015	0,492783		
TC0500003184.mm.1	1,03	Ptpn11	JUC0500015427.mm.1	-2,34	0,014561	0,449719		
TC0600000010.mm.1	-1	Col1a2	JUC0600000095.mm.1	-2,34	0,015446	0,454271		
TC1000000605.mm.1	-1,18	Ctnna3	JUC1000002297.mm.1	-2,34	0,021828	0,484607		
TC1000000605.mm.1	-1,18	Ctnna3	JUC1000002301.mm.1	-2,41	0,032565	0,520133		
TC0400001515.mm.1	1,08	Yars	JUC0400006101.mm.1	-2,34	0,032293	0,519116		
TC0X00003427.mm.1	-1,18	Xlr5a	JUC0X00007604.mm.1	-2,34	0,014038	0,44654		
TC0400003426.mm.1	3,26	Rimkla	PSR0400027846.mm.1	-2,35	0,024187	0,493294	Cassette Exon	0,22
TC0400003426.mm.1	3,26	Rimkla	PSR0400027849.mm.1	-2,78	0,000378	0,293011	Cassette Exon	0,22
TC0400003426.mm.1	3,26	Rimkla	JUC0400014517.mm.1	-3,59	0,003295	0,354243		
TC0400003426.mm.1	3,26	Rimkla	PSR0400027848.mm.1	-3,92	0,000905	0,313363	Cassette Exon	0,39
TC0400003426.mm.1	3,26	Rimkla	PSR0400027852.mm.1	-4,43	0,001272	0,322251	Cassette Exon	0,22
TC0400003426.mm.1	3,26	Rimkla	JUC0400014513.mm.1	-7,68	0,001994	0,341091		
TC0X00003023.mm.1	1,25	Bex1	PSR0X00018971.mm.1	-2,35	0,010103	0,418998	Intron Retention	0,32
TC0700000647.mm.1	1,66	Pepd	PSR0700005447.mm.1	-2,35	0,034641	0,526115	Alternative 5' Donc	0,21
TC0900003044.mm.1	1,47	Rbm6	PSR0900025537.mm.1	-2,35	0,002029	0,341377	Alternative 3' Acce	0,21
TC1800000052.mm.1	1,1	Wac	PSR1800000354.mm.1	-2,35	0,001454	0,328414	Alternative 5' Donc	0,21
TC0200001922.mm.1	1,06	Acoxl	PSR0200015100.mm.1	-2,35	0,000165	0,272566	Alternative 5' Donc	0,18
TC1100003090.mm.1	-1,77	Alox12	PSR1100028518.mm.1	-2,35	0,015068	0,45236	Alternative 3' Acce	0,18
TC1100000002.mm.1	1,14	Gm11399	PSR1100000059.mm.1	-2,35	0,018151	0,467399	Cassette Exon	0,17
TC1700000070.mm.1	1,9	Rps6ka2	JUC1700000306.mm.1	-2,35	0,018428	0,468598		
TC1700000070.mm.1	1,9	Rps6ka2	PSR1700000530.mm.1	-2,65	0,028498	0,507345	Cassette Exon	0,15
TC1100002571.mm.1	1,41	Gm12149	PSR1100023766.mm.1	-2,35	0,048491	0,560867	Cassette Exon	0,14
TC0400002634.mm.1	1,22	Acnat1	PSR0400021457.mm.1	-2,35	0,000113	0,267977	Cassette Exon	0,13
TC0X00002234.mm.1	1,09	Mir322; Mir351; Mir503;	PSR0X00014158.mm.1	-2,35	0,035705	0,529027	Alternative 5' Donc	0,11
TC0X00002234.mm.1	1,09	Mir322; Mir351; Mir503;	PSR0X00014157.mm.1	-2,54	0,014308	0,448073	Alternative 5' Donc	0,11
TC0X00003396.mm.1	1,92	Nlgn3	PSR0X00006439.mm.1	-2,35	0,019211	0,472971	Alternative 5' Donc	0,1
TC0200004447.mm.1	-1,24	Rpap1	JUC0200019035.mm.1	-2,35	0,005867	0,383835		
TC1900000908.mm.1	1,21	Rad9a	JUC1900004348.mm.1	-2,35	0,02107	0,480303		
TC1900000602.mm.1	1,47	Zdhhc16	JUC1900002881.mm.1	-2,35	0,026509	0,501346		
TC1900000602.mm.1	1,47	Zdhhc16	JUC1900002887.mm.1	-2,45	0,021913	0,484832		
TC0200002367.mm.1	-1,05	Acss2	JUC0200009552.mm.1	-2,35	0,029545	0,510307		
TC1600001053.mm.1	-1,43	Sim2	JUC1600004289.mm.1	-2,35	0,039509	0,539762		
TC0700000584.mm.1	1,03		JUC0700002611.mm.1	-2,35	0,016114	0,457756		
TC1100000894.mm.1	1,13	Tmem107	JUC1100003970.mm.1	-2,35	0,014781	0,45081		
TC0300001936.mm.1	-1,35		JUC0300007779.mm.1	-2,35	0,012954	0,438936		
TC0X00000313.mm.1	-1,44	Dock11	JUC0X00001055.mm.1	-2,35	0,024098	0,49316		
TC0400003522.mm.1	1,14	Zc3h12a	JUC0400014929.mm.1	-2,35	0,003417	0,355257		
TC0400003862.mm.1	1,29	Arhgef10l	JUC0400016543.mm.1	-2,35	0,038829	0,537696		

TC0400003862.mm.1	1,29	Arhgef10l	JUC0400016529.mm.1	-2,66	0,01526	0,452891		
TC0400002471.mm.1	1,69	Fam219a	JUC0400010397.mm.1	-2,35	0,001203	0,322097		
TC0300002750.mm.1	1,93	Gpsm2	PSR0300021645.mm.1	-2,36	0,011666	0,429939	Alternative 5' Donc	0,19
TC0300002750.mm.1	1,93	Gpsm2	PSR0300021646.mm.1	-2,42	0,021331	0,481727	Alternative 5' Donc	0,19
TC0300002750.mm.1	1,93	Gpsm2	PSR0300021647.mm.1	-2,57	0,018809	0,470836	Cassette Exon	0,25
TC0300002750.mm.1	1,93	Gpsm2	JUC0300011275.mm.1	-3,39	0,009396	0,415633		
TC0100001511.mm.1	1,11	Gm20743	PSR0100012460.mm.1	-2,36	0,006756	0,393343	Cassette Exon	0,13
TC0100001511.mm.1	1,11	Gm20743	PSR0100012446.mm.1	-2,76	0,04281	0,548097	Cassette Exon	0,18
TC0200003209.mm.1	1,27	Slc2a6	PSR0200027007.mm.1	-2,36	0,005863	0,383815	Intron Retention	0,18
TC0300001559.mm.1	1,17	Lrriq3	PSR0300012597.mm.1	-2,36	0,001528	0,330287	Cassette Exon	0,17
TC0200004383.mm.1	1,35	Meis2	PSR0200036647.mm.1	-2,36	0,01469	0,450253	Cassette Exon	0,14
TC1200000737.mm.1	1,33	Exd2	PSR1200005176.mm.1	-2,36	0,023527	0,490947	Alternative 3' Acce	0,14
TC0400004188.mm.1	-1,18	Cyp4a32	PSR0400009004.mm.1	-2,36	0,027051	0,502979	Cassette Exon	0,13
TC1100001264.mm.1	-1,2	Wfdc17	PSR1100011838.mm.1	-2,36	0,035872	0,529376	Cassette Exon	0,12
TC0700002257.mm.1	-1,3	Vmn2r-ps45	PSR0700020414.mm.1	-2,36	0,042129	0,546571	Cassette Exon	0,11
TC0600003489.mm.1	2,36	Glcci1	JUC0600000282.mm.1	-2,36	0,024604	0,494497		
TC0600003489.mm.1	2,36	Glcci1	PSR0600000506.mm.1	-2,39	0,003418	0,355257	Cassette Exon	0,06
TC0600003489.mm.1	2,36	Glcci1	JUC0600000284.mm.1	-3,27	0,024715	0,494838		
TC1400000120.mm.1	-1,01	Nek10	JUC1400000633.mm.1	-2,36	0,010776	0,423955		
TC0200004508.mm.1	-1	Shf	JUC0200019790.mm.1	-2,36	0,006615	0,392269		
TC1800000228.mm.1	-1,18	4930474G06Rik	JUC1800000929.mm.1	-2,36	0,025587	0,497808		
TC0100001543.mm.1	1,99		JUC0100007229.mm.1	-2,36	0,016619	0,459964		
TC0200001788.mm.1	1,25	Jmjd7; Pla2g4b; Jmjd7-pla	JUC0200006757.mm.1	-2,36	0,000024	0,22008		
TC0200001788.mm.1	1,25	Jmjd7; Pla2g4b; Jmjd7-pla	JUC0200006773.mm.1	-2,51	0,040218	0,541714		
TC1500000871.mm.1	-1,08	Ncaph2	JUC1500003869.mm.1	-2,36	0,011072	0,42589		
TC0200002005.mm.1	-1,04	Hspa12b	JUC0200007999.mm.1	-2,36	0,006429	0,389972		
TC1600000761.mm.1	-1		JUC1600003516.mm.1	-2,36	0,040013	0,541174		
TC0900001543.mm.1	-1,52		JUC0900007201.mm.1	-2,36	0,043903	0,550652		
TC0700004076.mm.1	-1,22	St5	JUC0700017689.mm.1	-2,36	0,006181	0,387873		
TC1400002831.mm.1	1,9	Bcl2l2	PSR1400005893.mm.1	-2,37	0,021545	0,483034	Intron Retention	0,33
TC1400002831.mm.1	1,9	Bcl2l2	PSR1400005890.mm.1	-2,73	0,031572	0,51716	Alternative 5' Donc	0,19
TC1000000045.mm.1	3,97	Lrp11	PSR1000000334.mm.1	-2,37	0,016945	0,461025	Cassette Exon	0,23
TC1000000045.mm.1	3,97	Lrp11	JUC1000000188.mm.1	-2,54	0,018856	0,470956		
TC1000000045.mm.1	3,97	Lrp11	PSR1000000325.mm.1	-3,51	0,001411	0,326543	Cassette Exon	0,25
TC1000000045.mm.1	3,97	Lrp11	PSR1000000324.mm.1	-3,59	0,019904	0,475879	Cassette Exon	0,24
TC1000002364.mm.1	1,17		PSR1000016853.mm.1	-2,37	0,006995	0,395737	Alternative 3' Acce	0,22
TC0100003238.mm.1	1,89	2310030A07Rik	PSR0100026187.mm.1	-2,37	0,043732	0,550282	Cassette Exon	0,2
TC0100003238.mm.1	1,89	2310030A07Rik	JUC0100014889.mm.1	-2,73	0,015199	0,452738		
TC1700002226.mm.1	-1,33	Apobec2	PSR1700021051.mm.1	-2,37	0,006418	0,389893	Cassette Exon	0,19
TC1700002226.mm.1	-1,33	Apobec2	JUC1700011211.mm.1	-2,43	0,029228	0,509609		
TC0100002215.mm.1	1,29	Aff3	PSR0100017890.mm.1	-2,37	0,002842	0,352207	Alternative 3' Acce	0,18
TC0800002059.mm.1	1,05		PSR0800015997.mm.1	-2,37	0,048844	0,561693	Cassette Exon	0,17
TC1000001590.mm.1	1,32	Gls2	PSR1000011578.mm.1	-2,37	0,01635	0,458679	Alternative 5' Donc	0,16
TC1000001590.mm.1	1,32	Gls2	JUC1000006208.mm.1	-2,47	0,034482	0,525929		
TC0300001712.mm.1	1,6	7530428D23Rik	PSR0300013489.mm.1	-2,37	0,023308	0,49004	Cassette Exon	0,15
TC0700002156.mm.1	1,35	Rdh13; Gm24605	PSR0700019551.mm.1	-2,37	0,023351	0,490143	Cassette Exon	0,12
TC0X00003265.mm.1	-1,12	Gm15188	PSR0X00020435.mm.1	-2,37	0,013674	0,444535	Cassette Exon	0,11
TC1300002606.mm.1	2,29	Rgs7bp	PSR1300017758.mm.1	-2,37	0,037828	0,534803	Cassette Exon	0,11

TC0200002667.mm.1	1,71	Cstf1	JUC0200011027.mm.1	-2,37	0,018952	0,471398		
TC0200005003.mm.1	-1,13	Sla2	JUC0200022039.mm.1	-2,37	0,039843	0,540922		
TC1300000876.mm.1	1,13	Irx2	JUC1300002931.mm.1	-2,37	0,02846	0,507299		
TC1700000646.mm.1	1,01	Dxo; Dom3z	JUC1700003540.mm.1	-2,37	0,033752	0,523605		
TC0800002779.mm.1	-1,28		JUC0800011634.mm.1	-2,37	0,034389	0,525677		
TC0600001026.mm.1	-1,2		JUC0600004087.mm.1	-2,37	0,026913	0,502518		
TC0700003731.mm.1	3,11		JUC0700016624.mm.1	-2,37	0,000755	0,308387		
TC1100001780.mm.1	1,14	Tcam1	JUC1100008754.mm.1	-2,37	0,024303	0,49362		
TC1100001780.mm.1	1,14	Tcam1	JUC1100008752.mm.1	-2,71	0,023402	0,490259		
TC0900003065.mm.1	1,65	P4htm	JUC0900014480.mm.1	-2,37	0,004027	0,361636		
TC1100003966.mm.1	3,46	Prkca	JUC1100019451.mm.1	-2,38	0,000326	0,28927		
TC1100003966.mm.1	3,46	Prkca	PSR1100037242.mm.1	-2,52	0,017305	0,463271	Cassette Exon	0,14
TC1100003966.mm.1	3,46	Prkca	PSR1100037236.mm.1	-2,63	0,007183	0,397855	Alternative 3' Acce	0,26
TC1100003966.mm.1	3,46	Prkca	PSR1100037227.mm.1	-2,77	0,010756	0,423859	Cassette Exon	0,16
TC1100003966.mm.1	3,46	Prkca	PSR1100037244.mm.1	-2,99	0,020454	0,478018	Cassette Exon	0,13
TC1100003966.mm.1	3,46	Prkca	PSR1100037232.mm.1	-3,58	0,009217	0,414099	Alternative 5' Donc	0,41
TC0100003468.mm.1	1,36	Pogk	PSR0100028432.mm.1	-2,38	0,010503	0,42211	Alternative 5' Donc	0,22
TC1000000639.mm.1	-1,06		PSR1000004368.mm.1	-2,38	0,022612	0,487423	Alternative 3' Acce	0,22
TC1200000038.mm.1	1,46	Atad2b	PSR1200000478.mm.1	-2,38	0,015694	0,455416	Alternative 5' Donc	0,2
TC1200000038.mm.1	1,46	Atad2b	JUC1200000283.mm.1	-3,14	0,009269	0,414688		
TC0300000244.mm.1	3,55	Sox2	PSR0300001584.mm.1	-2,38	0,031508	0,516965	Alternative 3' Acce	0,16
TC0300000244.mm.1	3,55	Sox2	JUC0300000793.mm.1	-3,35	0,005256	0,377945		
TC1000002229.mm.1	1,76	Chst3	PSR1000015583.mm.1	-2,38	0,003965	0,359943	Cassette Exon	0,15
TC1200000232.mm.1	-1,17		PSR1200001803.mm.1	-2,38	0,004599	0,369794	Cassette Exon	0,15
TC0X00001978.mm.1	-1,2	Gm5168	PSR0X00012552.mm.1	-2,38	0,043032	0,54841	Cassette Exon	0,13
TC1500000914.mm.1	1,03		PSR1500007331.mm.1	-2,38	0,043555	0,550033	Cassette Exon	0,11
TC1400000341.mm.1	1,23	Parg	JUC1400001788.mm.1	-2,38	0,002626	0,349612		
TC1400000341.mm.1	1,23	Parg	JUC1400001776.mm.1	-2,64	0,016046	0,457283		
TC0300000939.mm.1	1,26	Gm26689; 4930459I23Rik	JUC0300003869.mm.1	-2,38	0,027741	0,505023		
TC1300002496.mm.1	-1,13		JUC1300008863.mm.1	-2,38	0,037208	0,533262		
TC1700002595.mm.1	-1,03	Thumpd2	JUC1700012802.mm.1	-2,38	0,039326	0,539324		
TC1700001067.mm.1	-1,12	Dreh	JUC1700005450.mm.1	-2,38	0,041629	0,545202		
TC0700004489.mm.1	1,13	Tubgcp2	JUC0700019878.mm.1	-2,38	0,001846	0,337414		
TC0700001626.mm.1	1,19	Swap70	JUC0700006924.mm.1	-2,38	0,029812	0,511132		
TC1100000547.mm.1	-1,24	Adamts2	JUC1100002227.mm.1	-2,38	0,010102	0,418998		
TC1100000547.mm.1	-1,24	Adamts2	JUC1100002215.mm.1	-2,47	0,027793	0,505395		
TC1100002697.mm.1	-1,21	N4bp3	JUC1100012766.mm.1	-2,38	0,008589	0,408223		
TC0300002507.mm.1	1,26	Gm26553; 4930459I23Rik	JUC0300010169.mm.1	-2,38	0,027741	0,505023		
TC0400003253.mm.1	1,52	Zfyve9	JUC0400013470.mm.1	-2,38	0,011549	0,428821		
TC0400003896.mm.1	1,03	Fhad1	JUC0400016865.mm.1	-2,38	0,012671	0,43631		
TC0Y00000442.mm.1	-1,04	Orly	JUC0Y00001767.mm.1	-2,38	0,000692	0,305164		
TC0400003369.mm.1	2,07	Rnf220	PSR0400027017.mm.1	-2,39	0,009868	0,417961	Alternative 3' Acce	0,17
TC0400003369.mm.1	2,07	Rnf220	PSR0400027031.mm.1	-6,96	0,02226	0,485896	Alternative 3' Acce	0,44
TC0800000036.mm.1	1,83	Cers4	PSR0800000445.mm.1	-2,39	0,037991	0,535386	Cassette Exon	0,24
TC0800000036.mm.1	1,83	Cers4	PSR0800000447.mm.1	-2,39	0,004725	0,370888	Alternative 3' Acce	0,17
TC0800000036.mm.1	1,83	Cers4	JUC0800000247.mm.1	-3,39	0,00048	0,298999		
TC0600000765.mm.1	4,56	Ndnf	PSR0600005742.mm.1	-2,39	0,008097	0,405071	Alternative 5' Donc	0,18
TC0600000765.mm.1	4,56	Ndnf	PSR0600005732.mm.1	-2,84	0,004652	0,370181	Cassette Exon	0,21

TC0900002768.mm.1	1,27		PSR0900022909.mm.1	-2,39	0,000767	0,308403	Cassette Exon	0,18
TC0200004238.mm.1	1,33	D430041D05Rik	PSR0200035657.mm.1	-2,39	0,017815	0,465819	Cassette Exon	0,13
TC0200004238.mm.1	1,33	D430041D05Rik	JUC0200018219.mm.1	-2,68	0,00278	0,351017		
TC0500000321.mm.1	1,5	Zfp512	PSR0500002856.mm.1	-2,39	0,012625	0,436032	Cassette Exon	0,13
TC1200000068.mm.1	1,31	Pum2	PSR1200000693.mm.1	-2,39	0,0267	0,502057	Cassette Exon	0,11
TC1800001045.mm.1	-1,24	Dsc1	PSR1800007728.mm.1	-2,39	0,044635	0,552129	Cassette Exon	0,1
TC0200002700.mm.1	1,01	Gnas	PSR0200022062.mm.1	-2,39	0,006079	0,386691	Cassette Exon	0,07
TC0200003355.mm.1	-1,27	Traf1	PSR0200028800.mm.1	-2,39	0,012705	0,436428		
TC1800000835.mm.1	1,24	Epg5	JUC1800003411.mm.1	-2,39	0,036821	0,532174		
TC1500002108.mm.1	-1,01		JUC1500009611.mm.1	-2,39	0,030482	0,513325		
TC0200002188.mm.1	1,33	A530006G24Rik	JUC0200008739.mm.1	-2,39	0,004561	0,369273		
TC0200000577.mm.1	1,04	Ciz1	JUC0200002670.mm.1	-2,39	0,015407	0,453994		
TC0600001966.mm.1	-1,15	Gcc1	JUC0600008054.mm.1	-2,39	0,022594	0,487325		
TC0X00001636.mm.1	7,91	Rs1	JUC0X00005278.mm.1	-2,4	0,023426	0,490372		
TC0X00001636.mm.1	7,91	Rs1	JUC0X00005281.mm.1	-2,56	0,039095	0,538395		
TC0X00001636.mm.1	7,91	Rs1	PSR0X00010414.mm.1	-8,17	0,011002	0,425491	Alternative 3' Acce	0,47
TC0300002906.mm.1	1,15	Larp7	PSR0300023018.mm.1	-2,4	0,004818	0,371408	Alternative 3' Acce	0,22
TC0900002220.mm.1	1,02	4931429L15Rik	PSR0900018559.mm.1	-2,4	0,003913	0,359698	Alternative 3' Acce	0,22
TC1400002466.mm.1	-1,34	Fam216b	PSR1400018979.mm.1	-2,4	0,038088	0,535633	Alternative 3' Acce	0,22
TC0900000711.mm.1	1,22	Ulk3	PSR0900005229.mm.1	-2,4	0,011721	0,429969	Alternative 5' Donc	0,21
TC0900001665.mm.1	1,7	Limd1	PSR0900014002.mm.1	-2,4	0,029221	0,509537	Alternative 5' Donc	0,2
TC0700000223.mm.1	1,26	Meis3	JUC0700000793.mm.1	-2,4	0,019187	0,472828		
TC0700000223.mm.1	1,26	Meis3	PSR0700001608.mm.1	-3,01	0,016206	0,458282	Cassette Exon	0,19
TC1300002322.mm.1	1,24		PSR1300015287.mm.1	-2,4	0,04468	0,55216	Alternative 5' Donc	0,18
TC0600001632.mm.1	1,74	Pde6h	PSR0600012973.mm.1	-2,4	0,022344	0,486348	Cassette Exon	0,17
TC1300000147.mm.1	-2,1	Sfrp4	PSR1300000999.mm.1	-2,4	0,005689	0,382387	Cassette Exon	0,16
TC0200001523.mm.1	-1,02	Pamr1	PSR0200011427.mm.1	-2,4	0,001096	0,317328	Cassette Exon	0,14
TC0400001465.mm.1	-1,16	Clspn	PSR0400011178.mm.1	-2,4	0,011329	0,427211	Cassette Exon	0,13
TC0600001742.mm.1	1,48	Mrps35	PSR0600013897.mm.1	-2,4	0,029805	0,511132	Cassette Exon	0,13
TC1200002194.mm.1	1,2	Ptpn21	PSR1200015158.mm.1	-2,4	0,006934	0,395269	Cassette Exon	0,13
TC1800000953.mm.1	-1,1	Mkx	PSR1800007019.mm.1	-2,4	0,013051	0,439551	Cassette Exon	0,13
TC1400002260.mm.1	-1,13	Esco2	JUC1400009531.mm.1	-2,4	0,007749	0,402774		
TC0300000308.mm.1	-1,34	Plk4	JUC0300001163.mm.1	-2,4	0,015369	0,453849		
TC1200002077.mm.1	1,23	Entpd5	JUC1200007824.mm.1	-2,4	0,034627	0,526041		
TC1300001130.mm.1	-1,44		JUC1300003735.mm.1	-2,4	0,034204	0,525106		
TC1700001767.mm.1	-1,03	Slc26a8	JUC1700008785.mm.1	-2,4	0,028783	0,508148		
TC1500000214.mm.1	-1,38		JUC1500000929.mm.1	-2,4	0,002478	0,349135		
TC0800001915.mm.1	1,48	Letm2	PSR0800015279.mm.1	-2,4	0,032234	0,51899		
TC0800002722.mm.1	1,2	Amfr	JUC0800011351.mm.1	-2,4	0,038081	0,535633		
TC0700001915.mm.1	-1,13	1700029B22Rik	JUC0700008894.mm.1	-2,4	0,007538	0,401261		
TC1100000276.mm.1	1,27	Gm12081	JUC1100001218.mm.1	-2,4	0,002934	0,352289		
TC1100003487.mm.1	-1,2	Lpo	JUC1100016706.mm.1	-2,4	0,015612	0,455082		
TC1100003296.mm.1	-1,28	Slc13a2	JUC1100015893.mm.1	-2,4	0,025031	0,496091		
TC0400002822.mm.1	-1,05	Astn2	JUC0400012082.mm.1	-2,4	0,004806	0,371408		
TC0X00003425.mm.1	-1,29	Cul4b	JUC0X00006781.mm.1	-2,4	0,047287	0,558242		
TC1700001860.mm.1	2,24	Zfp763	PSR1700017232.mm.1	-2,41	0,047877	0,559408	Cassette Exon	0,1
TC1700001860.mm.1	2,24	Zfp763	PSR1700017234.mm.1	-2,92	0,048534	0,560994	Alternative 5' Donc	0,31
TC0500000172.mm.1	1,21	Fam185a	PSR0500001223.mm.1	-2,41	0,003678	0,357266	Alternative 5' Donc	0,22

TC0500000172.mm.1	1,21	Fam185a	PSR0500001217.mm.1	-2,74	0,013358	0,442089	Alternative 3' Acce	0,27
TC1800000901.mm.1	2,78	Cbln2	PSR1800006508.mm.1	-2,41	0,029734	0,511096	Cassette Exon	0,08
TC1800000901.mm.1	2,78	Cbln2	PSR1800006511.mm.1	-3,31	0,015446	0,454271	Cassette Exon	0,24
TC1800000901.mm.1	2,78	Cbln2	PSR1800006510.mm.1	-3,65	0,002422	0,34896	Cassette Exon	0,24
TC0600002314.mm.1	2,05	Scrn1	PSR0600018090.mm.1	-2,41	0,034997	0,527405	Cassette Exon	0,1
TC0600002314.mm.1	2,05	Scrn1	PSR0600018085.mm.1	-3,34	0,005752	0,383101	Cassette Exon	0,22
TC0400001006.mm.1	1,14	Alg6	PSR0400006771.mm.1	-2,41	0,001252	0,322251	Alternative 3' Acce	0,21
TC0400001006.mm.1	1,14	Alg6	JUC0400003454.mm.1	-5,99	0,04289	0,548183		
TC0200003146.mm.1	1,7	Dpp7	JUC0200013124.mm.1	-2,41	0,034964	0,52724		
TC0200003146.mm.1	1,7	Dpp7	PSR0200025935.mm.1	-2,49	0,035222	0,528366	Cassette Exon	0,16
TC1000001209.mm.1	-1,14	B530045E10Rik	PSR1000009086.mm.1	-2,41	0,003325	0,354243	Cassette Exon	0,14
TC0X00003351.mm.1	-1,5	Gm15230	PSR0X00020900.mm.1	-2,41	0,002739	0,349612	Cassette Exon	0,13
TC0900000540.mm.1	1,1	Fxyd6	PSR0900003741.mm.1	-2,41	0,006546	0,392081	Cassette Exon	0,12
TC1200000239.mm.1	1,01	Tssc1	JUC1200001051.mm.1	-2,41	0,044235	0,55119		
TC1700001100.mm.1	1,03	L3mbtl4	JUC1700005636.mm.1	-2,41	0,032006	0,518367		
TC1900000866.mm.1	1,46	Grk5	JUC1900004106.mm.1	-2,41	0,031326	0,516208		
TC1900000866.mm.1	1,46	Grk5	JUC1900004103.mm.1	-2,43	0,042826	0,548097		
TC0100003304.mm.1	1,1	Xpr1	JUC0100015377.mm.1	-2,41	0,047932	0,559506		
TC1400002515.mm.1	1,35	Diap3	JUC1400010660.mm.1	-2,41	0,031245	0,516001		
TC0800001247.mm.1	1,06	Prmt7	JUC0800005428.mm.1	-2,41	0,01139	0,427549		
TC0800000632.mm.1	1,46	AW046200	JUC0800002264.mm.1	-2,41	0,002407	0,348653		
TC0800000589.mm.1	1,16		JUC0800002200.mm.1	-2,41	0,042677	0,548097		
TC0800002818.mm.1	1,02	Lrrc29	JUC0800011818.mm.1	-2,41	0,008512	0,407441		
TC0300002095.mm.1	-1,11		JUC0300008303.mm.1	-2,41	0,000535	0,304044		
TC1100001163.mm.1	2,19	Sebox	JUC1100005684.mm.1	-2,41	0,011947	0,431753		
TC0400004147.mm.1	1,43	Mib2	JUC0400018117.mm.1	-2,41	0,000358	0,290838		
TC1700001756.mm.1	9,75	Tulp1	PSR1700016187.mm.1	-2,42	0,018039	0,467148	Alternative 5' Donc	0,51
TC1700001756.mm.1	9,75	Tulp1	PSR1700016190.mm.1	-2,79	0,016794	0,460623	Cassette Exon	0,42
TC1700001756.mm.1	9,75	Tulp1	JUC1700008718.mm.1	-3,59	0,039623	0,540083		
TC1700001756.mm.1	9,75	Tulp1	PSR1700016191.mm.1	-3,61	0,003388	0,3547	Cassette Exon	0,37
TC1700001756.mm.1	9,75	Tulp1	PSR1700016173.mm.1	-4,25	0,005173	0,376797	Intron Retention	0,38
TC1700001756.mm.1	9,75	Tulp1	PSR1700016167.mm.1	-4,79	0,009508	0,416602	Alternative 3' Acce	0,48
TC1700001756.mm.1	9,75	Tulp1	PSR1700016192.mm.1	-4,83	0,002983	0,352501	Cassette Exon	0,5
TC1700001756.mm.1	9,75	Tulp1	PSR1700016168.mm.1	-5,46	0,004599	0,369794	Alternative 3' Acce	0,48
TC1700001756.mm.1	9,75	Tulp1	PSR1700016188.mm.1	-5,62	0,011069	0,425887	Cassette Exon	0,42
TC1700001756.mm.1	9,75	Tulp1	PSR1700016181.mm.1	-5,78	0,010652	0,422928	Alternative 5' Donc	0,47
TC1700001756.mm.1	9,75	Tulp1	PSR1700016186.mm.1	-5,94	0,003027	0,353502	Alternative 5' Donc	0,51
TC1700001756.mm.1	9,75	Tulp1	PSR1700016163.mm.1	-5,94	0,001978	0,340886	Alternative 3' Acce	0,4
TC1700001756.mm.1	9,75	Tulp1	PSR1700016174.mm.1	-5,95	0,019387	0,473657		
TC1700001756.mm.1	9,75	Tulp1	PSR1700016194.mm.1	-6,27	0,001967	0,340561	Cassette Exon	0,41
TC1700001756.mm.1	9,75	Tulp1	PSR1700016193.mm.1	-6,63	0,006845	0,393893	Cassette Exon	0,37
TC1700001756.mm.1	9,75	Tulp1	PSR1700016185.mm.1	-7,27	0,035651	0,528956	Alternative 5' Donc	0,51
TC1700001756.mm.1	9,75	Tulp1	PSR1700016201.mm.1	-8,41	0,020916	0,479793	Alternative 5' Donc	0,33
TC1700001756.mm.1	9,75	Tulp1	JUC1700008729.mm.1	-9,7	0,00668	0,393187		
TC1700001756.mm.1	9,75	Tulp1	PSR1700016166.mm.1	-9,73	0,003417	0,355257	Alternative 3' Acce	0,48
TC1700001756.mm.1	9,75	Tulp1	JUC1700008719.mm.1	-11,72	0,004912	0,372662		
TC1700001756.mm.1	9,75	Tulp1	PSR1700016176.mm.1	-12,01	0,003027	0,353502	Alternative 3' Acce	0,51
TC1700001756.mm.1	9,75	Tulp1	JUC1700008725.mm.1	-12,12	0,018143	0,467399		

TC1700001756.mm.1	9,75	Tulp1	PSR1700016189.mm.1	-13,1	0,032308	0,519155	Cassette Exon	0,42
TC1700001756.mm.1	9,75	Tulp1	JUC1700008722.mm.1	-13,5	0,022707	0,487767		
TC1700001756.mm.1	9,75	Tulp1	JUC1700008728.mm.1	-13,51	0,00172	0,335996		
TC1700001756.mm.1	9,75	Tulp1	JUC1700008720.mm.1	-14,24	0,002066	0,341993		
TC1700001756.mm.1	9,75	Tulp1	PSR1700016196.mm.1	-15,94	0,004685	0,370338	Intron Retention	0,38
TC1700001756.mm.1	9,75	Tulp1	PSR1700016178.mm.1	-17,2	0,002303	0,346364	Alternative 5' Donc	0,36
TC1200002410.mm.1	1,6	2810029C07Rik	PSR1200016721.mm.1	-2,42	0,048226	0,56024	Intron Retention	0,35
TC1200002410.mm.1	1,6	2810029C07Rik	PSR1200016724.mm.1	-2,45	0,003772	0,357586	Alternative 5' Donc	0,23
TC1200002410.mm.1	1,6	2810029C07Rik	JUC1200009219.mm.1	-4,21	0,013285	0,441719		
TC1600001092.mm.1	-1,13	Mx2	JUC1600004502.mm.1	-2,42	0,036464	0,531117		
TC1600001092.mm.1	-1,13	Mx2	PSR1600008644.mm.1	-3,12	0,000099	0,262646	Cassette Exon	0,26
TC0400002389.mm.1	1,35	1700003M02Rik	PSR0400019382.mm.1	-2,42	0,011449	0,427811	Cassette Exon	0,13
TC0400002389.mm.1	1,35	1700003M02Rik	JUC0400010105.mm.1	-2,99	0,022706	0,487767		
TC0400002389.mm.1	1,35	1700003M02Rik	PSR0400019383.mm.1	-3,54	0,034088	0,524719	Cassette Exon	0,23
TC0600001750.mm.1	1,22	1700049E15Rik	PSR0600013959.mm.1	-2,42	0,019231	0,472971	Cassette Exon	0,16
TC0Y00000001.mm.1	1,47		PSR0Y00000003.mm.1	-2,42	0,001414	0,32678	Cassette Exon	0,14
TC0500000291.mm.1	-1,1	Drc1	PSR0500002281.mm.1	-2,42	0,00274	0,349612	Cassette Exon	0,13
TC0500001131.mm.1	1,81	Zfp605	PSR0500009627.mm.1	-2,42	0,02005	0,476473	Cassette Exon	0,13
TC1200002295.mm.1	1,61	Dicer1	JUC1200008816.mm.1	-2,42	0,03306	0,521637		
TC0100001195.mm.1	1,5	Rbbp5	JUC0100005616.mm.1	-2,42	0,02413	0,493245		
TC0500001692.mm.1	-1,12	Eif3b	JUC0500008440.mm.1	-2,42	0,036341	0,530559		
TC0500000176.mm.1	1,07	Pmpcb	JUC0500000743.mm.1	-2,42	0,025013	0,496061		
TC0800003047.mm.1	1,01	Mthfsd	JUC0800012859.mm.1	-2,42	0,011668	0,429939		
TC0800003047.mm.1	1,01	Mthfsd	JUC0800012864.mm.1	-4,99	0,004395	0,366181		
TC0900000182.mm.1	1,06	Ppan; Gm24067; Gm2300	JUC0900000569.mm.1	-2,42	0,042819	0,548097		
TC0700003850.mm.1	1,1	Il18bp	JUC0700017198.mm.1	-2,42	0,031645	0,517405		
TC0700001997.mm.1	-1,36	Kndc1	JUC0700009307.mm.1	-2,42	0,025851	0,498657		
TC0900001970.mm.1	1,35		JUC0900009304.mm.1	-2,42	0,041592	0,544987		
TC0900002152.mm.1	-1,09	Nlrx1	JUC0900010047.mm.1	-2,42	0,031241	0,515984		
TC0900002472.mm.1	1,27	Map2k5	JUC0900011660.mm.1	-2,42	0,000949	0,313363		
TC0200005477.mm.1	5,21	A530058N18Rik	PSR0200012552.mm.1	-2,43	0,003152	0,353892	Intron Retention	0,46
TC0200005477.mm.1	5,21	A530058N18Rik	PSR0200012547.mm.1	-3,08	0,007995	0,403853	Cassette Exon	0,21
TC0200005477.mm.1	5,21	A530058N18Rik	PSR0200012550.mm.1	-3,12	0,003228	0,354243	Intron Retention	0,46
TC0200005477.mm.1	5,21	A530058N18Rik	PSR0200012551.mm.1	-3,47	0,028956	0,508766	Intron Retention	0,46
TC0200005477.mm.1	5,21	A530058N18Rik	PSR0200012563.mm.1	-3,97	0,002318	0,346481	Alternative 5' Donc	0,46
TC0200005477.mm.1	5,21	A530058N18Rik	PSR0200046663.mm.1	-5,37	0,005378	0,378749	Cassette Exon	0,21
TC0200005477.mm.1	5,21	A530058N18Rik	PSR0200012541.mm.1	-6,21	0,002874	0,352207	Cassette Exon	0,21
TC0200005477.mm.1	5,21	A530058N18Rik	JUC0200006243.mm.1	-6,61	0,000518	0,303623		
TC0200005477.mm.1	5,21	A530058N18Rik	JUC0200006250.mm.1	-9,95	0,00699	0,395722		
TC0200005477.mm.1	5,21	A530058N18Rik	JUC0200006249.mm.1	-10,51	0,0012	0,321581		
TC1000001025.mm.1	2,9	Ric8b	JUC1000004158.mm.1	-2,43	0,015232	0,452839		
TC1000001025.mm.1	2,9	Ric8b	PSR1000007746.mm.1	-2,63	0,019566	0,474636	Alternative 3' Acce	0,23
TC1000001025.mm.1	2,9	Ric8b	PSR1000007758.mm.1	-3,85	0,005537	0,380557	Alternative 3' Acce	0,39
TC0100003627.mm.1	2,77	Akt3	PSR0100029768.mm.1	-2,43	0,012661	0,43631	Cassette Exon	0,13
TC0100003627.mm.1	2,77	Akt3	JUC0100016931.mm.1	-2,44	0,014772	0,450801		
TC0100003627.mm.1	2,77	Akt3	PSR0100029784.mm.1	-2,61	0,006716	0,393343	Alternative 5' Donc	0,26
TC0100003627.mm.1	2,77	Akt3	PSR0100029785.mm.1	-3,29	0,00389	0,359225	Cassette Exon	0,21
TC0800001085.mm.1	1,83	Gnao1	JUC0800004459.mm.1	-2,43	0,043751	0,550368		

TC0800001085.mm.1	1,83	Gnao1	PSR0800008343.mm.1	-2,58	0,001472	0,329052	Alternative 5' Donc		0,26
TC0800001085.mm.1	1,83	Gnao1	JUC0800004452.mm.1	-3,44	0,012444	0,43528			
TC1200002345.mm.1	1,13	Setd3	PSR1200016309.mm.1	-2,43	0,004734	0,370888	Alternative 5' Donc		0,2
TC0200000879.mm.1	1,44	Upp2; Gm13558	PSR0200007081.mm.1	-2,43	0,013802	0,445012	Cassette Exon		0,14
TC0200004843.mm.1	-1,04	Acss1	PSR0200041229.mm.1	-2,43	0,007591	0,401712	Cassette Exon		0,14
TC0300001950.mm.1	1,24		PSR0300014864.mm.1	-2,43	0,018648	0,470078	Cassette Exon		0,14
TC0300001281.mm.1	1,11		PSR0300010438.mm.1	-2,43	0,002151	0,343679	Cassette Exon		0,13
TC0900001243.mm.1	2,33	Faim	PSR0900009708.mm.1	-2,43	0,013372	0,442269	Cassette Exon		0,13
TC1400001915.mm.1	1,13	Rubie; Gm15219	JUC1400007941.mm.1	-2,43	0,034048	0,524583			
TC0100001089.mm.1	-1,22		JUC0100005155.mm.1	-2,43	0,034647	0,526139			
TC0100000601.mm.1	1,86	Tmem169	JUC0100002831.mm.1	-2,43	0,018285	0,468049			
TC0800001635.mm.1	1,27	Elavl1	JUC0800007350.mm.1	-2,43	0,017102	0,46232			
TC0500002650.mm.1	1,05	Noa1	JUC0500012748.mm.1	-2,43	0,028513	0,507402			
TC0700000413.mm.1	1,07	Megf8	JUC0700001600.mm.1	-2,43	0,018072	0,46716			
TC0700002153.mm.1	-1,32	Gp6	JUC0700010381.mm.1	-2,43	0,009812	0,417839			
TC1100002874.mm.1	1,35	Atpaf2	JUC1100013621.mm.1	-2,43	0,020394	0,47784			
TC1100001538.mm.1	-1,19	Arhgap23	JUC1100007369.mm.1	-2,43	0,002785	0,35133			
TC0X00003164.mm.1	1,11	Apex2	JUC0X00010026.mm.1	-2,43	0,016003	0,457051			
TC0X00003164.mm.1	1,11	Apex2	JUC0X00010024.mm.1	-2,62	0,045072	0,553115			
TC1700002660.mm.1	2,7	Prepl	PSR1700024174.mm.1	-2,44	0,031784	0,517596	Alternative 5' Donc		0,11
TC1700002660.mm.1	2,7	Prepl	PSR1700024168.mm.1	-3,18	0,028024	0,505851	Alternative 5' Donc		0,35
TC1700002660.mm.1	2,7	Prepl	JUC1700013042.mm.1	-3,58	0,011236	0,426718			
TC0200004281.mm.1	1,29	BB218582; LOC102640562	JUC0200018373.mm.1	-2,44	0,030818	0,514173			
TC0200004281.mm.1	1,29	BB218582; LOC102640562	PSR0200035971.mm.1	-2,94	0,0089	0,410728	Alternative 3' Acce		0,3
TC0900000509.mm.1	1,76	Slc37a4	PSR0900003470.mm.1	-2,44	0,018103	0,467161	Alternative 3' Acce		0,23
TC0200002876.mm.1	2,01	Camk1d	PSR0200023855.mm.1	-2,44	0,005717	0,382767	Alternative 3' Acce		0,17
TC1500001778.mm.1	2,13	Scrt1	PSR1500013684.mm.1	-2,44	0,005646	0,381706	Cassette Exon		0,15
TC1500001778.mm.1	2,13	Scrt1	PSR1500013685.mm.1	-2,85	0,002627	0,349612	Cassette Exon		0,15
TC1500001778.mm.1	2,13	Scrt1	JUC1500007797.mm.1	-2,89	0,018114	0,467161			
TC1400002281.mm.1	1,26	Bnip3l; Gm10480	JUC1400009647.mm.1	-2,44	0,035522	0,528764			
TC0300001124.mm.1	-1,05	Gstm5	JUC0300004733.mm.1	-2,44	0,000242	0,28803			
TC0100003882.mm.1	1,36	Chpf	JUC0100012067.mm.1	-2,44	0,007741	0,402774			
TC1700001822.mm.1	1,09	Cbs	JUC1700009012.mm.1	-2,44	0,006912	0,394898			
TC0800001733.mm.1	1,41	Dcun1d2	JUC0800007645.mm.1	-2,44	0,010875	0,424174			
TC0500003592.mm.1	1,05	Zkscan14	JUC0500017925.mm.1	-2,44	0,010022	0,418657			
TC0700002028.mm.1	-1,11	Psmd13	JUC0700009447.mm.1	-2,44	0,017478	0,463933			
TC0500002770.mm.1	2,33	Ccni	PSR0500024436.mm.1	-2,45	0,035261	0,52837	Cassette Exon		0,17
TC1300000157.mm.1	1,06	Aoah	PSR1300001068.mm.1	-2,45	0,012952	0,438927	Cassette Exon		0,17
TC0500001123.mm.1	1,37	Gm26779	PSR0500009558.mm.1	-2,45	0,009742	0,417839	Cassette Exon		0,16
TC0400001207.mm.1	-1,18	Skint11	PSR0400008775.mm.1	-2,45	0,021953	0,485095	Cassette Exon		0,13
TC0700004312.mm.1	1,51	Qprt	PSR0700036188.mm.1	-2,45	0,049446	0,563154	Cassette Exon		0,12
TC0200005181.mm.1	-1,11	Znfx1	JUC0200022885.mm.1	-2,45	0,01888	0,471134			
TC1500002349.mm.1	1,02	Tonsl	JUC1500007891.mm.1	-2,45	0,028883	0,50858			
TC0100003799.mm.1	1,23	Tatdn3	JUC0100017642.mm.1	-2,45	0,00263	0,349612			
TC1600001117.mm.1	-1,09	Crebbp	JUC1600004621.mm.1	-2,45	0,010579	0,422482			
TC0800000787.mm.1	1,19	Ankle1	JUC0800003013.mm.1	-2,45	0,024083	0,493056			
TC0800002861.mm.1	1,43	Chtf8	JUC0800012073.mm.1	-2,45	0,047061	0,557783			
TC0700001870.mm.1	1,5	Kat8	JUC0700008468.mm.1	-2,45	0,027002	0,502911			

TC1100000527.mm.1	1,39	Mapk9	JUC1100002079.mm.1	-2,45	0,045733	0,554855		
TC1100000903.mm.1	1,15	Alox12b	JUC1100004038.mm.1	-2,45	0,040284	0,541823		
TC1100001596.mm.1	1,05		JUC1100007758.mm.1	-2,45	0,045469	0,55427		
TC1100002062.mm.1	1,4	Narf	JUC1100010374.mm.1	-2,45	0,010893	0,424403		
TC0200001260.mm.1	-1,21	Olfr1029	PSR0200009974.mm.1	-2,46	0,034287	0,525366	Alternative 5' Donc	0,23
TC0300002459.mm.1	1,54	A730011C13Rik; Gm1526!	PSR0300018912.mm.1	-2,46	0,006961	0,395722	Alternative 3' Acce	0,22
TC0200001009.mm.1	1,03	Ppig	PSR0200007929.mm.1	-2,46	0,001779	0,336311	Alternative 3' Acce	0,21
TC0600001121.mm.1	1,07		PSR0600008778.mm.1	-2,46	0,003971	0,360082	Cassette Exon	0,19
TC0100002471.mm.1	1,36	Plekhm3	PSR0100020065.mm.1	-2,46	0,016415	0,458966	Cassette Exon	0,14
TC0600000902.mm.1	1,25	Eva1a	PSR0600006780.mm.1	-2,46	0,043894	0,550614	Cassette Exon	0,14
TC0600000902.mm.1	1,25	Eva1a	JUC0600003407.mm.1	-2,57	0,009275	0,41476		
TC0Y00000364.mm.1	1,03	Gm21396; Ssty2; Gm2079	PSR0Y00002149.mm.1	-2,46	0,025731	0,498198	Cassette Exon	0,11
TC1200000682.mm.1	-1,03	Plekhg3	JUC1200002769.mm.1	-2,46	0,004245	0,363417		
TC0300000090.mm.1	1,08	Lrrcc1; Gm17119	JUC0300000152.mm.1	-2,46	0,040469	0,542132		
TC1200000134.mm.1	-1,24	Rock2	JUC1200000654.mm.1	-2,46	0,044996	0,552975		
TC1300002539.mm.1	1,06	Rad17	JUC1300009216.mm.1	-2,46	0,00888	0,410619		
TC1200002392.mm.1	1,99	Mok; Stk30	JUC1200009145.mm.1	-2,46	0,011024	0,425635		
TC1200002392.mm.1	1,99	Mok; Stk30	JUC1200009140.mm.1	-2,98	0,018482	0,46874		
TC1200002392.mm.1	1,99	Mok; Stk30	JUC1200009149.mm.1	-3,04	0,030046	0,511812		
TC1200002392.mm.1	1,99	Mok; Stk30	JUC1200009135.mm.1	-3,63	0,013601	0,443714		
TC1200002392.mm.1	1,99	Mok; Stk30	JUC1200009136.mm.1	-4,42	0,008708	0,409126		
TC1700002030.mm.1	-1,14	H2-M9	JUC1700010341.mm.1	-2,46	0,015644	0,455298		
TC1500000837.mm.1	1,17		JUC1500003713.mm.1	-2,46	0,004177	0,362543		
TC0500002563.mm.1	-1,14	Gabra4	JUC0500012312.mm.1	-2,46	0,009802	0,417839		
TC0800000077.mm.1	1,07	Myo16	JUC0800000352.mm.1	-2,46	0,021678	0,483934		
TC0800000077.mm.1	1,07	Myo16	JUC0800000359.mm.1	-2,6	0,0106	0,422767		
TC0700004589.mm.1	-1,01	Cars	JUC0700020401.mm.1	-2,46	0,011242	0,426718		
TC0600003196.mm.1	-1,21	Tead4	JUC0600012939.mm.1	-2,46	0,003575	0,356223		
TC0700001791.mm.1	1,12	Kdm8	JUC0700007979.mm.1	-2,46	0,021055	0,480262		
TC0400003085.mm.1	-1	Cyp2j13	JUC0400012847.mm.1	-2,46	0,039141	0,538564		
TC1000000211.mm.1	1,28	Moxd1	JUC1000000796.mm.1	-2,46	0,013561	0,443675		
TC0500002355.mm.1	2,76	Kcnip4	PSR0500021562.mm.1	-2,47	0,025275	0,497033	Cassette Exon	0,1
TC0500002355.mm.1	2,76	Kcnip4	PSR0500021565.mm.1	-3,3	0,046363	0,556304	Cassette Exon	0,32
TC0500002355.mm.1	2,76	Kcnip4	JUC0500011767.mm.1	-4,37	0,01057	0,422432		
TC0500002355.mm.1	2,76	Kcnip4	PSR0500021572.mm.1	-5,59	0,006749	0,393343	Cassette Exon	0,35
TC0500002355.mm.1	2,76	Kcnip4	PSR0500021570.mm.1	-6,68	0,011045	0,425792		
TC1100002854.mm.1	1,48	Zkscan17	PSR1100025891.mm.1	-2,47	0,045524	0,554359	Alternative 5' Donc	0,16
TC1100002854.mm.1	1,48	Zkscan17	PSR1100025904.mm.1	-3,27	0,014931	0,451919	Cassette Exon	0,3
TC0200000969.mm.1	1,42	B3galt1	PSR0200007681.mm.1	-2,47	0,041152	0,544	Cassette Exon	0,27
TC0200003290.mm.1	1,61	Gm13427	JUC0200014083.mm.1	-2,47	0,015748	0,455423		
TC0200003290.mm.1	1,61	Gm13427	JUC0200014084.mm.1	-2,71	0,039593	0,539903		
TC0200003290.mm.1	1,61	Gm13427	PSR0200027918.mm.1	-2,75	0,002584	0,349501	Alternative 5' Donc	0,24
TC0500000389.mm.1	-1	Hgfac	PSR0500003861.mm.1	-2,47	0,032042	0,518459	Alternative 3' Acce	0,24
TC0300002522.mm.1	1,51	Polr3gl	PSR0300019631.mm.1	-2,47	0,019058	0,471978	Alternative 5' Donc	0,23
TC0700001756.mm.1	1,03	Prkcb	PSR0700015000.mm.1	-2,47	0,031436	0,516647	Alternative 5' Donc	0,23
TC0700001756.mm.1	1,03	Prkcb	JUC0700007854.mm.1	-2,62	0,030246	0,512493		
TC0200005089.mm.1	-1,1	Jph2	JUC0200022378.mm.1	-2,47	0,019536	0,474538		
TC0200005089.mm.1	-1,1	Jph2	PSR0200043399.mm.1	-2,67	0,014896	0,451802	Cassette Exon	0,22

TC0200005371.mm.1	1,41	Gm14406	PSR0200045694.mm.1	-2,47	0,0018	0,336311	Cassette Exon	0,16
TC0700004374.mm.1	1,26		PSR0700036773.mm.1	-2,47	0,044959	0,552881	Alternative 5' Donc	0,15
TC1200002506.mm.1	1,03	Dnah11	JUC1200010467.mm.1	-2,47	0,00049	0,298999		
TC0200004894.mm.1	1,06	Rbck1	JUC0200021482.mm.1	-2,47	0,009183	0,413665		
TC0100002175.mm.1	1,21	Uggt1	JUC0100009820.mm.1	-2,47	0,026406	0,501198		
TC0100000374.mm.1	-1,19	Stat4	JUC0100001687.mm.1	-2,47	0,035477	0,528724		
TC0100000153.mm.1	1,06		JUC0100000525.mm.1	-2,47	0,017365	0,463612		
TC1900000907.mm.1	1,08	Tbc1d10c	JUC1900004338.mm.1	-2,47	0,035828	0,529349		
TC0100003811.mm.1	-1		JUC0100017680.mm.1	-2,47	0,04971	0,563531		
TC0800000391.mm.1	1,02		JUC0800001569.mm.1	-2,47	0,010541	0,422281		
TC0900001425.mm.1	1,26	Slc26a6	PSR0900011607.mm.1	-2,47	0,038944	0,537874		
TC0900001425.mm.1	1,26	Slc26a6	JUC0900006419.mm.1	-3,21	0,02484	0,495465		
TC0500001340.mm.1	1,11	Iqcd	JUC0500006398.mm.1	-2,47	0,001956	0,340561		
TC0700000924.mm.1	1,09		JUC0700004206.mm.1	-2,47	0,012494	0,435429		
TC0600003030.mm.1	-1,54	Wnk1	JUC0600012182.mm.1	-2,47	0,038204	0,535805		
TC0500003318.mm.1	1,13	Sbds	JUC0500016363.mm.1	-2,47	0,036855	0,532257		
TC1100000253.mm.1	1,25		JUC1100001156.mm.1	-2,47	0,0126	0,435933		
TC1000002915.mm.1	-1,2	Myrfl	JUC1000012103.mm.1	-2,47	0,007551	0,401384		
TC0400001130.mm.1	-1,06	Glis1; Gm24804	JUC0400004075.mm.1	-2,47	0,027015	0,502979		
TC0400002051.mm.1	1	Gm13133	JUC0400008886.mm.1	-2,47	0,005207	0,377078		
TC1800001749.mm.1	5,7	Ppp2r2b	PSR1800009355.mm.1	-2,48	0,027205	0,503406	Cassette Exon	0,27
TC1800001749.mm.1	5,7	Ppp2r2b	PSR1800009368.mm.1	-2,66	0,018077	0,46716	Alternative 3' Acce	0,32
TC1800001749.mm.1	5,7	Ppp2r2b	PSR1800009378.mm.1	-2,93	0,018811	0,470836	Alternative 5' Donc	0,41
TC1800001749.mm.1	5,7	Ppp2r2b	PSR1800009366.mm.1	-3,57	0,008384	0,4065	Alternative 3' Acce	0,13
TC1800001749.mm.1	5,7	Ppp2r2b	JUC1800005216.mm.1	-3,65	0,006433	0,389994		
TC1800001749.mm.1	5,7	Ppp2r2b	PSR1800009369.mm.1	-3,75	0,00721	0,398126	Alternative 3' Acce	0,32
TC1800001749.mm.1	5,7	Ppp2r2b	PSR1800009393.mm.1	-3,94	0,000092	0,255472	Cassette Exon	0,27
TC1800001749.mm.1	5,7	Ppp2r2b	PSR1800009365.mm.1	-4,18	0,00147	0,329052	Alternative 3' Acce	0,13
TC1800001749.mm.1	5,7	Ppp2r2b	JUC1800005212.mm.1	-4,44	0,007332	0,399002		
TC1800001749.mm.1	5,7	Ppp2r2b	PSR1800009372.mm.1	-4,49	0,003831	0,358553	Alternative 5' Donc	0,49
TC1800001749.mm.1	5,7	Ppp2r2b	PSR1800009356.mm.1	-4,57	0,037856	0,534804	Alternative 3' Acce	0,48
TC1800001749.mm.1	5,7	Ppp2r2b	PSR1800009385.mm.1	-4,66	0,006795	0,393554	Cassette Exon	0,28
TC1800001749.mm.1	5,7	Ppp2r2b	PSR1800009374.mm.1	-5,29	0,00174	0,335996	Cassette Exon	0,29
TC1800001749.mm.1	5,7	Ppp2r2b	PSR1800009394.mm.1	-5,72	0,001356	0,325441	Cassette Exon	0,27
TC1800001749.mm.1	5,7	Ppp2r2b	PSR1800009380.mm.1	-5,76	0,004132	0,3623	Alternative 5' Donc	0,41
TC1800001749.mm.1	5,7	Ppp2r2b	JUC1800005222.mm.1	-6,07	0,000644	0,304044		
TC1800001749.mm.1	5,7	Ppp2r2b	PSR1800009383.mm.1	-6,08	0,001649	0,333988	Cassette Exon	0,28
TC1800001749.mm.1	5,7	Ppp2r2b	PSR1800009384.mm.1	-6,38	0,032916	0,521149	Cassette Exon	0,28
TC1800001749.mm.1	5,7	Ppp2r2b	PSR1800009386.mm.1	-6,42	0,001195	0,321568	Cassette Exon	0,28
TC1800001749.mm.1	5,7	Ppp2r2b	PSR1800009377.mm.1	-6,86	0,003135	0,353892	Alternative 5' Donc	0,45
TC1800001749.mm.1	5,7	Ppp2r2b	PSR1800009379.mm.1	-6,88	0,001303	0,323504	Alternative 5' Donc	0,41
TC1800001749.mm.1	5,7	Ppp2r2b	PSR1800009382.mm.1	-7,6	0,003897	0,359372		
TC1100002099.mm.1	6,48	Osbp2	PSR1100020376.mm.1	-2,48	0,000892	0,313363	Alternative 3' Acce	0,22
TC1100002099.mm.1	6,48	Osbp2	JUC1100010636.mm.1	-2,66	0,001379	0,32572		
TC1100002099.mm.1	6,48	Osbp2	PSR1100020379.mm.1	-2,76	0,009166	0,413447	Cassette Exon	0,16
TC1100002099.mm.1	6,48	Osbp2	JUC1100010644.mm.1	-3,03	0,013184	0,440686		
TC1100002099.mm.1	6,48	Osbp2	PSR1100020381.mm.1	-4,33	0,024294	0,49362	Cassette Exon	0,28
TC1100002099.mm.1	6,48	Osbp2	PSR1100020382.mm.1	-5,15	0,007797	0,403089	Cassette Exon	0,42

TC1100002099.mm.1	6,48	Osbp2	JUC1100010645.mm.1	-5,74	0,012721	0,43654		
TC1100002099.mm.1	6,48	Osbp2	PSR1100020384.mm.1	-5,94	0,011273	0,426793	Cassette Exon	0,07
TC1100002099.mm.1	6,48	Osbp2	PSR1100020383.mm.1	-6,96	0,009786	0,417839	Cassette Exon	0,07
TC1100002099.mm.1	6,48	Osbp2	PSR1100020387.mm.1	-7,82	0,004867	0,371692	Cassette Exon	0,32
TC1100002099.mm.1	6,48	Osbp2	JUC1100010641.mm.1	-8,86	0,011067	0,425887		
TC1100002099.mm.1	6,48	Osbp2	PSR1100020386.mm.1	-10,31	0,008058	0,40448	Cassette Exon	0,32
TC1200001813.mm.1	1,12	Sec23a	PSR1200012408.mm.1	-2,48	0,0025	0,349189	Intron Retention	0,37
TC0800002618.mm.1	1,76	Neto2	JUC0800010931.mm.1	-2,48	0,027468	0,504083		
TC0800002618.mm.1	1,76	Neto2	PSR0800020127.mm.1	-2,74	0,00988	0,418011	Alternative 5' Donc	0,31
TC0800002618.mm.1	1,76	Neto2	PSR0800020134.mm.1	-2,9	0,017167	0,46232	Cassette Exon	0,25
TC0800002618.mm.1	1,76	Neto2	JUC0800010933.mm.1	-3,73	0,00043	0,297771		
TC0600002354.mm.1	2,79	Vopp1	PSR0600018365.mm.1	-2,48	0,009126	0,413316	Cassette Exon	0,27
TC0600002354.mm.1	2,79	Vopp1	PSR0600018366.mm.1	-2,61	0,00982	0,417839	Cassette Exon	0,15
TC0600002354.mm.1	2,79	Vopp1	JUC0600009589.mm.1	-5,37	0,025147	0,496499		
TC0300000224.mm.1	1,46	Mfn1	PSR0300001313.mm.1	-2,48	0,022613	0,487423	Alternative 5' Donc	0,24
TC1800001340.mm.1	1,49	Cep120	PSR1800009853.mm.1	-2,48	0,008207	0,405909	Alternative 3' Acce	0,21
TC1900001683.mm.1	2,01	Bbip1	JUC1900008219.mm.1	-2,48	0,016445	0,459051		
TC1900001683.mm.1	2,01	Bbip1	PSR1900014806.mm.1	-2,73	0,01108	0,42589	Cassette Exon	0,16
TC1900001683.mm.1	2,01	Bbip1	JUC1900008215.mm.1	-2,77	0,001817	0,336896		
TC1900001683.mm.1	2,01	Bbip1	PSR1900014798.mm.1	-2,98	0,011569	0,429011	Alternative 3' Acce	0,18
TC1900001683.mm.1	2,01	Bbip1	JUC1900008220.mm.1	-8,09	0,001625	0,332831		
TC1100003407.mm.1	1,4	Tada2a	JUC1100016287.mm.1	-2,48	0,002235	0,34578		
TC1100003407.mm.1	1,4	Tada2a	PSR1100031182.mm.1	-2,49	0,042	0,546097	Cassette Exon	0,15
TC1600001320.mm.1	-1,13	Cdc45	PSR1600010896.mm.1	-2,48	0,022143	0,485609	Cassette Exon	0,15
TC0500000309.mm.1	1,16	Tcf23	PSR0500002630.mm.1	-2,48	0,0371	0,532885	Cassette Exon	0,14
TC0600001968.mm.1	1,66	Lrrc4	PSR0600015439.mm.1	-2,48	0,025221	0,496772	Alternative 5' Donc	0,14
TC1700001595.mm.1	1,31	Zfp13	PSR1700013916.mm.1	-2,48	0,011342	0,427345	Cassette Exon	0,14
TC0600001875.mm.1	1,34	B630005N14Rik	PSR0600014773.mm.1	-2,48	0,013393	0,442454	Alternative 5' Donc	0,13
TC0200002851.mm.1	1,34	Hspa14	PSR0200023660.mm.1	-2,48	0,006562	0,39213	Cassette Exon	0,12
TC0200002851.mm.1	1,34	Hspa14	JUC0200011986.mm.1	-2,57	0,008909	0,410728		
TC0500000374.mm.1	1,46	Fam193a	PSR0500003600.mm.1	-2,48	0,043722	0,550256	Cassette Exon	0,12
TC0500003157.mm.1	1,03	AW549542; Gm2301; Tbx	PSR0500027974.mm.1	-2,48	0,041938	0,545823	Alternative 5' Donc	0,11
TC0200002842.mm.1	-1,25	Gm14496	JUC0200011947.mm.1	-2,48	0,025302	0,497227		
TC0200003164.mm.1	-1,12	Tmem141	JUC0200013269.mm.1	-2,48	0,036448	0,531024		
TC1200000500.mm.1	1,8	Pnn	JUC1200001939.mm.1	-2,48	0,002898	0,352207		
TC1300002121.mm.1	1,06	Spata31d1a	JUC1300007043.mm.1	-2,48	0,014318	0,448086		
TC0800003241.mm.1	1,6	2610005L07Rik	JUC0800007907.mm.1	-2,48	0,038904	0,537874		
TC0800000378.mm.1	-1,09	Nrg1	JUC0800001550.mm.1	-2,48	0,015277	0,453131		
TC0900001065.mm.1	-1	Gm3211	JUC0900004568.mm.1	-2,48	0,003672	0,357266		
TC0800002458.mm.1	1,14	Eps15l1	JUC0800010255.mm.1	-2,48	0,024985	0,496025		
TC0600001442.mm.1	-1,02	Acrbp; Ing4	JUC0600006161.mm.1	-2,48	0,019628	0,474852		
TC1100001088.mm.1	1,34	Gm16075	JUC1100005241.mm.1	-2,48	0,020787	0,479453		
TC1000002228.mm.1	1,12		JUC1000008463.mm.1	-2,48	0,012506	0,435429		
TC0300002472.mm.1	-1,09	Prune	JUC0300009952.mm.1	-2,48	0,02562	0,497926		
TC1100001948.mm.1	1,4	Gm11731	JUC1100009663.mm.1	-2,48	0,044776	0,552442		
TC0X00002236.mm.1	-1,24	Fam122b	JUC0X00007251.mm.1	-2,48	0,02861	0,507714		
TC0X00002236.mm.1	-1,24	Fam122b	JUC0X00007250.mm.1	-5,27	0,033215	0,522175		
TC0400001639.mm.1	2,25	Stmn1; Gm23634	PSR0400013046.mm.1	-2,49	0,015352	0,453763	Intron Retention	0,35

TC1200000803.mm.1	1,64	Dnal1	PSR1200005744.mm.1	-2,49	0,017867	0,466232	Alternative 5' Donc	0,24
TC1200000803.mm.1	1,64	Dnal1	JUC1200003250.mm.1	-2,68	0,032907	0,521149		
TC1300001995.mm.1	2,1		PSR1300012428.mm.1	-2,49	0,004609	0,369794	Alternative 3' Acce	0,24
TC1500001977.mm.1	1,5	Ttll1	PSR1500015604.mm.1	-2,49	0,024575	0,4943	Alternative 3' Acce	0,18
TC0700000997.mm.1	1,16	Lins	PSR0700009143.mm.1	-2,49	0,011711	0,429969	Cassette Exon	0,15
TC0400002426.mm.1	-1,08	Gm17094	PSR0400019589.mm.1	-2,49	0,048395	0,56064	Cassette Exon	0,14
TC1700001521.mm.1	-1,03	Has1	PSR1700013567.mm.1	-2,49	0,022879	0,488524	Cassette Exon	0,14
TC1400000116.mm.1	-1,12		JUC1400000558.mm.1	-2,49	0,035332	0,528404		
TC0200002833.mm.1	1,02	Tcea2	JUC0200011870.mm.1	-2,49	0,004079	0,362111		
TC1400000786.mm.1	1,26	Rec8	JUC1400003398.mm.1	-2,49	0,002165	0,344238		
TC1400000786.mm.1	1,26	Rec8	JUC1400003396.mm.1	-2,64	0,007372	0,39943		
TC0200004440.mm.1	1,11	Oip5	JUC0200018997.mm.1	-2,49	0,006748	0,393343		
TC1300001127.mm.1	1,11		JUC1300003728.mm.1	-2,49	0,03919	0,538692		
TC0700001383.mm.1	1,18	Map6	JUC0700006060.mm.1	-2,49	0,013954	0,446239		
TC0300002551.mm.1	-1,38	Hsd3b4; Gm10681; Gm45	JUC0300010368.mm.1	-2,49	0,002469	0,349135		
TC0300002551.mm.1	-1,38	Hsd3b4; Gm10681; Gm45	JUC0300010373.mm.1	-2,74	0,018966	0,471398		
TC1800000300.mm.1	3,81	Reep2	JUC1800001306.mm.1	-2,5	0,02503	0,496091		
TC1800000300.mm.1	3,81	Reep2	PSR1800002198.mm.1	-2,54	0,002563	0,349501	Cassette Exon	0,25
TC1800000300.mm.1	3,81	Reep2	JUC1800001310.mm.1	-2,71	0,001471	0,329052		
TC1800000300.mm.1	3,81	Reep2	JUC1800001308.mm.1	-3,11	0,00878	0,409822		
TC1800000300.mm.1	3,81	Reep2	PSR1800002197.mm.1	-3,4	0,010421	0,42148	Cassette Exon	0,25
TC1800000300.mm.1	3,81	Reep2	PSR1800002203.mm.1	-5,39	0,000228	0,28803	Alternative 3' Acce	0,49
TC0200002858.mm.1	1,33	Gm13187; LOC102639987	PSR0200023722.mm.1	-2,5	0,020924	0,479862	Alternative 5' Donc	0,22
TC1200002119.mm.1	1,11	Noxred1	JUC1200008095.mm.1	-2,5	0,037212	0,533267		
TC1300000618.mm.1	1,1	Gm2762	JUC1300002019.mm.1	-2,5	0,034443	0,525886		
TC0200002436.mm.1	-1,06	Arhgap40	JUC0200010033.mm.1	-2,5	0,004775	0,371044		
TC0100002139.mm.1	1,07		JUC0100009736.mm.1	-2,5	0,002041	0,341377		
TC1800001539.mm.1	1,36	4930503L19Rik	JUC1800006288.mm.1	-2,5	0,02049	0,478019		
TC1500001791.mm.1	1,26	Arhgap39	JUC1500007951.mm.1	-2,5	0,009048	0,412827		
TC1500001791.mm.1	1,26	Arhgap39	JUC1500007952.mm.1	-2,61	0,00248	0,349135		
TC1500000650.mm.1	-1,05	1700041B01Rik	JUC1500002707.mm.1	-2,5	0,010431	0,421581		
TC0800000194.mm.1	1,38		JUC0800000923.mm.1	-2,5	0,03469	0,526294		
TC0900000335.mm.1	1,03		JUC0900001335.mm.1	-2,5	0,007401	0,399784		
TC0700000070.mm.1	1,54	Zfp444	JUC0700000368.mm.1	-2,5	0,02177	0,48438		
TC0700000070.mm.1	1,54	Zfp444	JUC0700000369.mm.1	-2,56	0,01848	0,468729		
TC0500002965.mm.1	-1,33	Vmn2r14	JUC0500014316.mm.1	-2,5	0,015741	0,455416		
TC0700003493.mm.1	1,31	Chd2; 1810026B05Rik; RP2	JUC0700015681.mm.1	-2,5	0,011799	0,430307		
TC0700001821.mm.1	1,07	Doc2a	JUC0700008165.mm.1	-2,5	0,023761	0,491689		
TC0700001821.mm.1	1,07	Doc2a	JUC0700008154.mm.1	-2,85	0,017668	0,464755		
TC1000002677.mm.1	1,46	Rmst; Gm3515	JUC1000011236.mm.1	-2,5	0,020755	0,479262		
TC0300001423.mm.1	1,34	Tspan5	JUC0300006035.mm.1	-2,5	0,019744	0,474932		
TC0900002164.mm.1	1,53	Trappc4	JUC0900010131.mm.1	-2,5	0,045415	0,554105		
TC0200003359.mm.1	1,06	AI182371	PSR0200028883.mm.1	-2,51	0,042126	0,546571	Alternative 5' Donc	0,25
TC0300001110.mm.1	3,91	AI504432	PSR0300009046.mm.1	-2,51	0,03221	0,518852	Alternative 5' Donc	0,22
TC1100000544.mm.1	2,03	Hnrnph1	PSR1100004217.mm.1	-2,51	0,016049	0,457283	Alternative 5' Donc	0,18
TC1100000544.mm.1	2,03	Hnrnph1	JUC1100002197.mm.1	-2,64	0,042675	0,548097		
TC1800001063.mm.1	1,6	Nol4	PSR1800007846.mm.1	-2,51	0,027294	0,503732	Alternative 3' Acce	0,18
TC1800001063.mm.1	1,6	Nol4	JUC1800004383.mm.1	-3,39	0,01911	0,47227		

TC0X00000723.mm.1	1,19	Xlr3d-ps	PSR0X00005013.mm.1	-2,51	0,005773	0,383104	Cassette Exon	0,15
TC0300001150.mm.1	1,43	Wdr47	PSR0300009367.mm.1	-2,51	0,005462	0,379939	Cassette Exon	0,14
TC1200000596.mm.1	-1,24	Frmd6	JUC1200002234.mm.1	-2,51	0,04374	0,550304		
TC1500000856.mm.1	1,06	Mov10l1	JUC1500003778.mm.1	-2,51	0,042814	0,548097		
TC0200000783.mm.1	1,09	Mbd5	JUC0200003325.mm.1	-2,51	0,037715	0,534585		
TC0500002879.mm.1	1,4	Klhl8	JUC0500013826.mm.1	-2,51	0,010172	0,419711		
TC1100001047.mm.1	-1,17	Cluh	JUC1100004968.mm.1	-2,51	0,007401	0,399784		
TC1100002804.mm.1	1,22	2010001A14Rik	JUC1100013199.mm.1	-2,51	0,009493	0,416578		
TC0900003138.mm.1	-1,2	Arpp21	JUC0900014965.mm.1	-2,51	0,036916	0,5323		
TC0X00002059.mm.1	1,62	Upf3b	JUC0X00006666.mm.1	-2,51	0,016774	0,460519		
TC0700003184.mm.1	8,18	Gm25088	PSR0700028373.mm.1	-2,52	0,00013	0,270905	Alternative 5' Donc	0,24
TC0700003184.mm.1	8,18	Gm25088	PSR0700028371.mm.1	-6,96	0,00004	0,245341	Alternative 3' Acce	0,49
TC1900000084.mm.1	-1,01	Gm20417	PSR1900001010.mm.1	-2,52	0,047155	0,557948	Intron Retention	0,16
TC1900000084.mm.1	-1,01	Gm20417	PSR1900001005.mm.1	-3,2	0,018813	0,470836	Alternative 3' Acce	0,33
TC1800001477.mm.1	8,9	Cplx4	JUC1800006079.mm.1	-2,52	0,012047	0,432362		
TC1800001477.mm.1	8,9	Cplx4	PSR1800010883.mm.1	-2,55	0,001601	0,332773	Alternative 5' Donc	0,24
TC1100003233.mm.1	3,43	Doc2b	PSR1100029738.mm.1	-2,52	0,009888	0,418122	Cassette Exon	0,21
TC1100003233.mm.1	3,43	Doc2b	JUC1100015562.mm.1	-2,59	0,038796	0,537586		
TC1100003233.mm.1	3,43	Doc2b	JUC1100015555.mm.1	-4,79	0,001427	0,327447		
TC0100002741.mm.1	2,28	Mir3535; Gm24148	PSR0100022176.mm.1	-2,52	0,045493	0,55435	Cassette Exon	0,1
TC0100002741.mm.1	2,28	Mir3535; Gm24148	PSR0100022175.mm.1	-2,83	0,012309	0,434392	Cassette Exon	0,17
TC0600000328.mm.1	1,41	Bpgm	PSR0600002434.mm.1	-2,52	0,013062	0,439579	Cassette Exon	0,15
TC0100002588.mm.1	1,39	Rnf25	JUC0100011848.mm.1	-2,52	0,000751	0,308185		
TC0200002380.mm.1	1,49	Cep250	JUC0200009655.mm.1	-2,52	0,005359	0,378582		
TC1600001379.mm.1	1,08	Etv5	JUC1600005956.mm.1	-2,52	0,023919	0,492324		
TC0500003713.mm.1	1,31	Ccdc62	JUC0500007014.mm.1	-2,52	0,017086	0,462201		
TC0500003713.mm.1	1,31	Ccdc62	JUC0500007007.mm.1	-3,24	0,018915	0,471305		
TC0600000948.mm.1	-1,23	Dysf	JUC0600003711.mm.1	-2,52	0,018098	0,46716		
TC1100001641.mm.1	1,21	Mlx	JUC1100007911.mm.1	-2,52	0,040882	0,543423		
TC0X00000675.mm.1	-1,09	Xlr5b	JUC0X00002160.mm.1	-2,52	0,004034	0,361636		
TC0400002120.mm.1	-1,24	Tmem68	JUC0400009376.mm.1	-2,52	0,00298	0,352501		
TC0200003759.mm.1	4,07	Chn1	JUC0200016416.mm.1	-2,53	0,012701	0,43642		
TC0200003759.mm.1	4,07	Chn1	PSR0200032168.mm.1	-2,7	0,039943	0,540973	Cassette Exon	0,02
TC0200003759.mm.1	4,07	Chn1	PSR0200032135.mm.1	-2,76	0,028682	0,508019	Alternative 3' Acce	0,29
TC0200003759.mm.1	4,07	Chn1	PSR0200032162.mm.1	-2,93	0,017691	0,465026	Cassette Exon	0,42
TC0200003759.mm.1	4,07	Chn1	JUC0200016418.mm.1	-3,39	0,038977	0,53792		
TC0200003759.mm.1	4,07	Chn1	PSR0200032167.mm.1	-3,84	0,000789	0,310805	Cassette Exon	0,4
TC0200003759.mm.1	4,07	Chn1	PSR0200032151.mm.1	-3,98	0,018936	0,471376	Cassette Exon	0,27
TC0200003759.mm.1	4,07	Chn1	PSR0200032147.mm.1	-4,2	0,019414	0,473856	Alternative 5' Donc	0,48
TC0200003759.mm.1	4,07	Chn1	PSR0200032148.mm.1	-4,63	0,016208	0,458312	Alternative 5' Donc	0,48
TC0200003759.mm.1	4,07	Chn1	JUC0200016417.mm.1	-4,85	0,014262	0,447947		
TC0200003759.mm.1	4,07	Chn1	PSR0200032169.mm.1	-5,36	0,015054	0,452223	Cassette Exon	0,37
TC0200003759.mm.1	4,07	Chn1	PSR0200032133.mm.1	-5,74	0,00629	0,388922	Alternative 3' Acce	0,28
TC0200003759.mm.1	4,07	Chn1	PSR0200032134.mm.1	-7,13	0,013322	0,441836	Alternative 3' Acce	0,29
TC0200003759.mm.1	4,07	Chn1	PSR0200032165.mm.1	-8,28	0,004948	0,373364	Cassette Exon	0,35
TC0200003759.mm.1	4,07	Chn1	JUC0200016424.mm.1	-8,74	0,020731	0,479166		
TC0200003759.mm.1	4,07	Chn1	PSR0200032157.mm.1	-9,85	0,003922	0,359818	Alternative 5' Donc	0,36
TC0200003759.mm.1	4,07	Chn1	JUC0200016426.mm.1	-10,01	0,037084	0,532836		

TC1100002118.mm.1	3,83	Gm11961	PSR1100020568.mm.1	-2,53	0,000056	0,24627	Cassette Exon	0,16
TC1100002118.mm.1	3,83	Gm11961	JUC1100010749.mm.1	-2,8	0,005529	0,380518		
TC1100002118.mm.1	3,83	Gm11961	PSR1100020569.mm.1	-4,09	0,002719	0,349612	Cassette Exon	0,28
TC1100002118.mm.1	3,83	Gm11961	PSR1100020570.mm.1	-5,14	0,008258	0,405976	Cassette Exon	0,28
TC0200002827.mm.1	1,66	Dnajc5	PSR0200023340.mm.1	-2,53	0,006581	0,39213	Cassette Exon	0,26
TC0200002827.mm.1	1,66	Dnajc5	JUC0200011825.mm.1	-3,17	0,013938	0,446202		
TC0200002827.mm.1	1,66	Dnajc5	JUC0200011830.mm.1	-3,66	0,032056	0,518469		
TC1500000045.mm.1	1,01	Lifr	PSR1500000401.mm.1	-2,53	0,009793	0,417839	Alternative 3' Acce	0,25
TC0700004575.mm.1	1,08	Th	PSR0700038595.mm.1	-2,53	0,006308	0,389234	Alternative 3' Acce	0,24
TC0900000496.mm.1	2,44	Thy1	PSR0900003239.mm.1	-2,53	0,041396	0,544608	Cassette Exon	0,15
TC0900000496.mm.1	2,44	Thy1	PSR0900003243.mm.1	-2,6	0,025604	0,497821	Cassette Exon	0,23
TC0900000496.mm.1	2,44	Thy1	PSR0900003240.mm.1	-3,13	0,020853	0,479521	Cassette Exon	0,23
TC1700002815.mm.1	-1,41	C4a	PSR1700018078.mm.1	-2,53	0,042167	0,546785	Cassette Exon	0,2
TC1600000346.mm.1	-1,23	Rtp4	PSR1600003433.mm.1	-2,53	0,021306	0,481714	Cassette Exon	0,18
TC0100003315.mm.1	1,79	Fam163a	PSR0100027074.mm.1	-2,53	0,034678	0,526294	Cassette Exon	0,14
TC0100003315.mm.1	1,79	Fam163a	PSR0100027072.mm.1	-2,82	0,026946	0,50274	Cassette Exon	0,17
TC0600000968.mm.1	1,81	Alms1-ps2	PSR0600007501.mm.1	-2,53	0,003556	0,356223	Cassette Exon	0,16
TC0600000968.mm.1	1,81	Alms1-ps2	JUC0600003832.mm.1	-3,67	0,007102	0,396897		
TC0500001011.mm.1	1,12	Aff1	PSR0500008542.mm.1	-2,53	0,027562	0,50444	Cassette Exon	0,15
TC1100003870.mm.1	1,05	Map3k14; 1700028N14Ril	JUC1100018890.mm.1	-2,53	0,01385	0,445398		
TC1300002172.mm.1	1,16	Gm5665	JUC1300007249.mm.1	-2,53	0,015914	0,456623		
TC0200004595.mm.1	1,14	Gm14029	JUC0200020399.mm.1	-2,53	0,024555	0,494116		
TC0200001781.mm.1	1,09	Tyro3	JUC0200006673.mm.1	-2,53	0,047995	0,559574		
TC0200002177.mm.1	1,76	Kiz; Plk1s1	JUC0200008687.mm.1	-2,53	0,033273	0,522325		
TC0600000576.mm.1	-1,19	Svs1	JUC0600002247.mm.1	-2,53	0,027088	0,503022		
TC0600002714.mm.1	1,17	Ccdc37	JUC0600010922.mm.1	-2,53	0,00108	0,317191		
TC0600001528.mm.1	-1,4	Klrb1-ps1	JUC0600006554.mm.1	-2,53	0,01484	0,451311		
TC0400002712.mm.1	4,25	Frrs1l	PSR0400021922.mm.1	-2,54	0,004883	0,372195	Alternative 3' Acce	0,36
TC0400002712.mm.1	4,25	Frrs1l	JUC0400011365.mm.1	-2,54	0,041189	0,544121		
TC0400002712.mm.1	4,25	Frrs1l	JUC0400011364.mm.1	-3,11	0,001726	0,335996		
TC0400002712.mm.1	4,25	Frrs1l	PSR0400021930.mm.1	-3,89	0,003588	0,356223	Cassette Exon	0,33
TC0400002712.mm.1	4,25	Frrs1l	PSR0400021923.mm.1	-4,29	0,021798	0,484444	Alternative 3' Acce	0,36
TC0400002712.mm.1	4,25	Frrs1l	PSR0400021931.mm.1	-4,58	0,010508	0,422115	Cassette Exon	0,27
TC1100004301.mm.1	-1,04	Trim47	PSR1100038674.mm.1	-2,54	0,01864	0,470078	Alternative 5' Donc	0,25
TC1700001893.mm.1	1,38	Ring1	PSR1700017631.mm.1	-2,54	0,024945	0,495859	Alternative 3' Acce	0,19
TC0900003334.mm.1	-1,05	Acad11	PSR0900010260.mm.1	-2,54	0,03135	0,516281	Alternative 5' Donc	0,16
TC1600002128.mm.1	-1,41	Erg	PSR1600016347.mm.1	-2,54	0,026745	0,502096	Cassette Exon	0,16
TC1700000980.mm.1	1,23		PSR1700009371.mm.1	-2,54	0,030693	0,513934	Cassette Exon	0,15
TC0200003873.mm.1	1,36	Tmx2	PSR0200033525.mm.1	-2,54	0,047066	0,557783	Cassette Exon	0,14
TC1700001323.mm.1	-1,21	Ston1	PSR1700012075.mm.1	-2,54	0,005001	0,373851	Cassette Exon	0,14
TC1400000833.mm.1	1,11	Rcbtb1	JUC1400003685.mm.1	-2,54	0,038697	0,537187		
TC0200004366.mm.1	-1,01	Arhgap11a	JUC0200018671.mm.1	-2,54	0,007812	0,403089		
TC1700000864.mm.1	-1,32	Nfkbie	JUC1700004428.mm.1	-2,54	0,012982	0,439245		
TC1500002181.mm.1	1,58	Adcy6	JUC1500010094.mm.1	-2,54	0,001471	0,329052		
TC1500001935.mm.1	1,02	Desi1	JUC1500008636.mm.1	-2,54	0,002531	0,349501		
TC1500002031.mm.1	1,23	Alg12	JUC1500009127.mm.1	-2,54	0,030991	0,514765		
TC1700000347.mm.1	-1,1	Zfp598	JUC1700001464.mm.1	-2,54	0,036877	0,532257		
TC0800001057.mm.1	1,28		JUC0800004374.mm.1	-2,54	0,004589	0,369794		

TC0800000701.mm.1	-1,02	1700125H03Rik	JUC0800002507.mm.1	-2,54	0,008166	0,405603		
TC0600001075.mm.1	-1,09		JUC0600004241.mm.1	-2,54	0,004596	0,369794		
TC0700001803.mm.1	1,05		JUC0700008065.mm.1	-2,54	0,016083	0,457504		
TC0900002186.mm.1	-1,04	Ube4a	JUC0900010290.mm.1	-2,54	0,02186	0,484635		
TC0600003412.mm.1	3,04	Bcat1	PSR0600026862.mm.1	-2,55	0,002205	0,345178	Cassette Exon	0,17
TC0600003412.mm.1	3,04	Bcat1	PSR0600026874.mm.1	-2,99	0,013856	0,445409	Cassette Exon	0,06
TC0600003412.mm.1	3,04	Bcat1	PSR0600026864.mm.1	-3,05	0,008384	0,4065	Cassette Exon	0,28
TC0600003412.mm.1	3,04	Bcat1	PSR0600026875.mm.1	-3,13	0,007349	0,399281	Cassette Exon	0,28
TC0600003412.mm.1	3,04	Bcat1	PSR0600026879.mm.1	-3,3	0,015755	0,455437	Alternative 5' Donc	0,31
TC0600003412.mm.1	3,04	Bcat1	PSR0600026881.mm.1	-3,3	0,008508	0,407341	Cassette Exon	0,25
TC0600003412.mm.1	3,04	Bcat1	PSR0600026872.mm.1	-3,36	0,013069	0,4396	Cassette Exon	0,25
TC0600003412.mm.1	3,04	Bcat1	JUC0600014095.mm.1	-3,6	0,010155	0,419579		
TC0600003412.mm.1	3,04	Bcat1	PSR0600026876.mm.1	-3,82	0,013201	0,440783	Cassette Exon	0,28
TC0600003412.mm.1	3,04	Bcat1	PSR0600026858.mm.1	-3,83	0,000748	0,308185	Alternative 3' Acce	0,42
TC0600003412.mm.1	3,04	Bcat1	PSR0600026880.mm.1	-3,92	0,010404	0,42148	Cassette Exon	0,27
TC0600003412.mm.1	3,04	Bcat1	JUC0600014096.mm.1	-4,01	0,006202	0,387873		
TC0600003412.mm.1	3,04	Bcat1	JUC0600014093.mm.1	-4,65	0,00262	0,349612		
TC0600003412.mm.1	3,04	Bcat1	PSR0600026882.mm.1	-5,05	0,000652	0,304044	Cassette Exon	0,25
TC1900000520.mm.1	1,3	Hectd2	PSR1900004616.mm.1	-2,55	0,034622	0,52602	Alternative 5' Donc	0,23
TC1900000520.mm.1	1,3	Hectd2	JUC1900002408.mm.1	-2,65	0,048624	0,561193		
TC0500003724.mm.1	1,26	Gtf3c2	PSR0500019855.mm.1	-2,55	0,037219	0,533311	Alternative 3' Acce	0,18
TC0500003724.mm.1	1,26	Gtf3c2	JUC0500010842.mm.1	-4,13	0,01952	0,474538		
TC1400002454.mm.1	-1,09	Lacc1	JUC1400010451.mm.1	-2,55	0,017484	0,463964		
TC1800000209.mm.1	-1,01	AW554918	JUC1800000895.mm.1	-2,55	0,028777	0,508148		
TC1700000715.mm.1	1,11	Gnl1	JUC1700004021.mm.1	-2,55	0,034157	0,524923		
TC0500002508.mm.1	1,22	Apbb2	JUC0500012186.mm.1	-2,55	0,042579	0,548047		
TC0500003268.mm.1	-1,19	Ncor2	JUC0500016071.mm.1	-2,55	0,034744	0,526574		
TC0400000833.mm.1	1,06	Gm11184	JUC0400002875.mm.1	-2,55	0,004845	0,37168		
TC0300002324.mm.1	2,55	Syt11	PSR0300017682.mm.1	-2,56	0,032136	0,518586	Alternative 5' Donc	0,15
TC0300002324.mm.1	2,55	Syt11	PSR0300017685.mm.1	-2,59	0,016928	0,460892	Cassette Exon	0,28
TC0300002324.mm.1	2,55	Syt11	JUC0300009272.mm.1	-4,38	0,010553	0,422341		
TC0200002811.mm.1	1,37	Arfgap1	JUC0200011688.mm.1	-2,56	0,026548	0,501435		
TC1300002063.mm.1	1,19	Ddx41	JUC1300006734.mm.1	-2,56	0,01848	0,468729		
TC0200004830.mm.1	-1,3		JUC0200021286.mm.1	-2,56	0,02811	0,506167		
TC1700001549.mm.1	-1,17		JUC1700007441.mm.1	-2,56	0,003138	0,353892		
TC1700000387.mm.1	-1,14	Metrn	JUC1700001782.mm.1	-2,56	0,036999	0,532502		
TC0100003335.mm.1	1,03		JUC0100015621.mm.1	-2,56	0,010988	0,425397		
TC0800001573.mm.1	-1	Slc35f3	JUC0800007047.mm.1	-2,56	0,000219	0,28803		
TC0700001555.mm.1	1,08	Timm10b; Dnhd1	JUC0700006688.mm.1	-2,56	0,017374	0,463612		
TC1000002387.mm.1	-1,07		JUC1000009324.mm.1	-2,56	0,009666	0,41754		
TC0300002541.mm.1	1,15	Chd1l	JUC0300010276.mm.1	-2,56	0,030754	0,514087		
TC0X00002440.mm.1	-1,09	Renbp	JUC0X00007807.mm.1	-2,56	0,025397	0,497403		
TC1900001316.mm.1	1,49	Rfx3	PSR1900011758.mm.1	-2,57	0,044103	0,550916	Cassette Exon	0,16
TC1900001316.mm.1	1,49	Rfx3	PSR1900011737.mm.1	-2,59	0,005608	0,381115	Intron Retention	0,39
TC1900001316.mm.1	1,49	Rfx3	JUC1900006446.mm.1	-2,89	0,011913	0,431645		
TC0100003203.mm.1	1,49	Trove2	PSR0100026012.mm.1	-2,57	0,01381	0,445064	Intron Retention	0,38
TC0100003203.mm.1	1,49	Trove2	JUC0100014813.mm.1	-3,88	0,011259	0,426757		
TC0300000096.mm.1	8,02	Car2	JUC0300000183.mm.1	-2,57	0,001169	0,318556		

TC0300000096.mm.1	8,02	Car2	PSR0300000428.mm.1	-3,02	0,006939	0,395353	Cassette Exon	0,22
TC0300000096.mm.1	8,02	Car2	PSR0300000429.mm.1	-3,76	0,000389	0,296342	Cassette Exon	0,26
TC0300002755.mm.1	-1,23	Fndc7	PSR0300021700.mm.1	-2,57	0,032448	0,519587	Alternative 3' Acce	0,25
TC0400002187.mm.1	-1,09	Gm11816	PSR0400018214.mm.1	-2,57	0,006999	0,395737	Alternative 5' Donc	0,23
TC0300003100.mm.1	2,11	Spata1	JUC0300012788.mm.1	-2,57	0,016845	0,460785		
TC0300003100.mm.1	2,11	Spata1	PSR0300024377.mm.1	-2,62	0,007072	0,39672	Cassette Exon	0,15
TC0300003100.mm.1	2,11	Spata1	PSR0300024388.mm.1	-2,8	0,023937	0,492536	Cassette Exon	0,19
TC0300003100.mm.1	2,11	Spata1	JUC0300012787.mm.1	-3,44	0,001265	0,322251		
TC0200004348.mm.1	-1,01	Nutm1	JUC0200018520.mm.1	-2,57	0,007322	0,39896		
TC0100002078.mm.1	1		JUC0100009638.mm.1	-2,57	0,016905	0,460797		
TC1700001394.mm.1	-1,17	Rsph3b; Rsph3a	JUC1700006855.mm.1	-2,57	0,035373	0,528536		
TC1900000154.mm.1	1,85	Stx5a; Mir6992; mmu-mir	JUC1900001021.mm.1	-2,57	0,008973	0,411821		
TC0500001102.mm.1	1,67	Gm10419	JUC0500005096.mm.1	-2,57	0,018755	0,470733		
TC0700000072.mm.1	-1,03	Galp	JUC0700000381.mm.1	-2,57	0,023588	0,490995		
TC0600002880.mm.1	1,07	Sumf1	JUC0600011502.mm.1	-2,57	0,046237	0,55611		
TC0600003125.mm.1	-1,14	Lag3	JUC0600012691.mm.1	-2,57	0,027426	0,503961		
TC0700002882.mm.1	-1,28	Gm4454; Gm21115; Gm21	JUC0700013753.mm.1	-2,57	0,01118	0,426414		
TC0900002121.mm.1	1,09	Grik4; Gm24420	JUC0900009915.mm.1	-2,57	0,001875	0,338405		
TC1000001014.mm.1	1,17		JUC1000004076.mm.1	-2,57	0,023304	0,49004		
TC0800003155.mm.1	1,15	4732419C18Rik	JUC0800013218.mm.1	-2,57	0,002976	0,352501		
TC0800003155.mm.1	1,15	4732419C18Rik	JUC0800013217.mm.1	-2,74	0,016805	0,460623		
TC0300000916.mm.1	2,87	Anp32e	PSR0300007312.mm.1	-2,58	0,032465	0,519612	Alternative 5' Donc	0,29
TC0300000916.mm.1	2,87	Anp32e	JUC0300003793.mm.1	-2,87	0,017465	0,463886		
TC0300000916.mm.1	2,87	Anp32e	PSR0300007304.mm.1	-3,32	0,046962	0,557563	Alternative 3' Acce	0,33
TC0300000916.mm.1	2,87	Anp32e	JUC0300003791.mm.1	-3,56	0,041624	0,545159		
TC0800002582.mm.1	1,01		PSR0800019706.mm.1	-2,58	0,015829	0,456076	Cassette Exon	0,19
TC1300002006.mm.1	3,56	Diras2	PSR1300012507.mm.1	-2,58	0,0066	0,39213	Cassette Exon	0,17
TC1300002006.mm.1	3,56	Diras2	PSR1300012508.mm.1	-3,17	0,009739	0,417839	Cassette Exon	0,17
TC0100000922.mm.1	1,14	Capn10	PSR0100007764.mm.1	-2,58	0,01125	0,426718		
TC0500000809.mm.1	-1,29	Prol1	JUC0500003886.mm.1	-2,58	0,032991	0,521365		
TC0600002195.mm.1	-1,56	Gm3120	JUC0600009072.mm.1	-2,58	0,015946	0,456831		
TC1000002258.mm.1	1,16	Supv3l1	JUC1000008826.mm.1	-2,58	0,032024	0,51843		
TC0X00001090.mm.1	1,08	Tbx22	JUC0X00003541.mm.1	-2,58	0,019577	0,474672		
TC1000000733.mm.1	1,41	Zfp280b	JUC1000002859.mm.1	-2,58	0,042927	0,548271		
TC0800000679.mm.1	2,89	March1	PSR0800004579.mm.1	-2,59	0,006253	0,388622	Cassette Exon	0,27
TC0800000679.mm.1	2,89	March1	JUC0800002465.mm.1	-3,16	0,004357	0,365487		
TC0800000679.mm.1	2,89	March1	PSR0800004572.mm.1	-3,44	0,035515	0,528764	Cassette Exon	0,21
TC0800000679.mm.1	2,89	March1	JUC0800002457.mm.1	-3,48	0,001401	0,325997		
TC0800000679.mm.1	2,89	March1	PSR0800004570.mm.1	-3,71	0,016326	0,458616	Cassette Exon	0,39
TC0800000679.mm.1	2,89	March1	PSR0800004567.mm.1	-3,77	0,010357	0,421055	Cassette Exon	0,39
TC0800000679.mm.1	2,89	March1	PSR0800004566.mm.1	-4,36	0,005113	0,376246	Alternative 3' Acce	0,04
TC0800000679.mm.1	2,89	March1	PSR0800004562.mm.1	-4,63	0,013267	0,441583	Alternative 3' Acce	0,04
TC0800000679.mm.1	2,89	March1	PSR0800004569.mm.1	-5,53	0,002045	0,341377	Cassette Exon	0,07
TC0800000679.mm.1	2,89	March1	JUC0800002453.mm.1	-9,71	0,006564	0,39213		
TC1900001276.mm.1	-1,22	Fxn	PSR1900011532.mm.1	-2,59	0,006817	0,39387	Alternative 3' Acce	0,25
TC1700001672.mm.1	1,94	Haghl	PSR1700015308.mm.1	-2,59	0,037852	0,534803	Alternative 5' Donc	0,18
TC0700001417.mm.1	1,59	Fam168a	PSR0700012089.mm.1	-2,59	0,01349	0,44319	Cassette Exon	0,17
TC0700001417.mm.1	1,59	Fam168a	JUC0700006268.mm.1	-2,98	0,031322	0,516204		

TC0100000446.mm.1	1,1		PSR0100003646.mm.1	-2,59	0,023302	0,49004	Cassette Exon	0,15
TC1700000975.mm.1	1,13	Kat2b	JUC1700004970.mm.1	-2,59	0,000235	0,28803		
TC1900000114.mm.1	1,26	Map4k2	JUC1900000747.mm.1	-2,59	0,035394	0,528596		
TC0200001849.mm.1	-1,15	Slc28a2	JUC0200007198.mm.1	-2,59	0,001017	0,316361		
TC0100003370.mm.1	1,09	Zbtb37	JUC0100015745.mm.1	-2,59	0,043581	0,550052		
TC0800001497.mm.1	1,14	Acsf3	JUC0800006650.mm.1	-2,59	0,048682	0,561341		
TC0900000174.mm.1	-1,23	Fbxl12os; 5730577I03Rik	JUC0900000528.mm.1	-2,59	0,046548	0,55676		
TC0900003052.mm.1	1,63	Bsn	JUC0900014430.mm.1	-2,59	0,005	0,373851		
TC0900003052.mm.1	1,63	Bsn	JUC0900014425.mm.1	-3,17	0,006513	0,391376		
TC0400004007.mm.1	1,18		JUC0400017269.mm.1	-2,59	0,044297	0,551351		
TC0400004007.mm.1	1,18		JUC0400017270.mm.1	-2,59	0,044297	0,551351		
TC0200000250.mm.1	5,45	Cacnb2	PSR0200001128.mm.1	-2,6	0,040491	0,542169	Alternative 5' Donc	0,23
TC0200000250.mm.1	5,45	Cacnb2	PSR0200001109.mm.1	-2,95	0,006613	0,392269	Alternative 5' Donc	0,27
TC0200000250.mm.1	5,45	Cacnb2	PSR0200001114.mm.1	-3,53	0,004812	0,371408	Cassette Exon	0,48
TC0200000250.mm.1	5,45	Cacnb2	JUC0200000493.mm.1	-4,11	0,022413	0,486711		
TC0200000250.mm.1	5,45	Cacnb2	JUC0200000488.mm.1	-4,48	0,006281	0,388863		
TC0200000250.mm.1	5,45	Cacnb2	PSR0200001100.mm.1	-4,64	0,007921	0,403819	Cassette Exon	0,21
TC0200000250.mm.1	5,45	Cacnb2	PSR0200001097.mm.1	-4,83	0,043085	0,548459	Cassette Exon	0,28
TC0200000250.mm.1	5,45	Cacnb2	PSR0200001098.mm.1	-4,95	0,010216	0,419955	Cassette Exon	0,41
TC0200000250.mm.1	5,45	Cacnb2	PSR0200001104.mm.1	-5,09	0,005113	0,376246	Cassette Exon	0,21
TC0200000250.mm.1	5,45	Cacnb2	PSR0200001102.mm.1	-5,58	0,008581	0,40821	Cassette Exon	0,41
TC0200000250.mm.1	5,45	Cacnb2	JUC0200000492.mm.1	-5,58	0,003938	0,359818		
TC0200000250.mm.1	5,45	Cacnb2	PSR0200001099.mm.1	-5,79	0,006359	0,389469	Cassette Exon	0,55
TC0200000250.mm.1	5,45	Cacnb2	PSR0200001105.mm.1	-6,01	0,01955	0,474538	Cassette Exon	0,21
TC0200000250.mm.1	5,45	Cacnb2	PSR0200001103.mm.1	-6,23	0,017247	0,462729	Cassette Exon	0,07
TC0200000250.mm.1	5,45	Cacnb2	JUC0200000497.mm.1	-7,32	0,008037	0,40434		
TC0200000250.mm.1	5,45	Cacnb2	JUC0200000481.mm.1	-7,49	0,004575	0,369794		
TC0200000250.mm.1	5,45	Cacnb2	JUC0200000480.mm.1	-9,1	0,020297	0,477245		
TC0200000250.mm.1	5,45	Cacnb2	JUC0200000476.mm.1	-9,41	0,003212	0,354243		
TC0200000250.mm.1	5,45	Cacnb2	JUC0200000490.mm.1	-10,13	0,00057	0,304044		
TC1200000074.mm.1	1,49	Wdr35	JUC1200000444.mm.1	-2,6	0,028956	0,508766		
TC1200000074.mm.1	1,49	Wdr35	JUC1200000426.mm.1	-2,94	0,032327	0,519155		
TC1300002214.mm.1	-1,02		JUC1300007475.mm.1	-2,6	0,00813	0,405267		
TC0100003243.mm.1	-1,21		JUC0100015033.mm.1	-2,6	0,000398	0,29748		
TC1600001962.mm.1	-1,28		JUC1600007840.mm.1	-2,6	0,006155	0,387873		
TC0800000736.mm.1	1,51	Hapln4	JUC0800002678.mm.1	-2,6	0,033699	0,523549		
TC0500001049.mm.1	-1,29	Zfp326	JUC0500004855.mm.1	-2,6	0,005597	0,381115		
TC0600000663.mm.1	1,19	Fam188b	JUC0600002566.mm.1	-2,6	0,017697	0,465114		
TC0600000663.mm.1	1,19	Fam188b	JUC0600002568.mm.1	-2,83	0,042429	0,547627		
TC1100003077.mm.1	-1,03		JUC1100014838.mm.1	-2,6	0,009498	0,416578		
TC0400003604.mm.1	1,11	Eif3i	JUC0400015324.mm.1	-2,6	0,027347	0,503766		
TC0100000627.mm.1	2,22	Pnkd	PSR0100005039.mm.1	-2,61	0,048133	0,559938	Alternative 5' Donc	0,25
TC0300001043.mm.1	-1,93	Ampd1	JUC0300004339.mm.1	-2,61	0,013113	0,439759		
TC0300001278.mm.1	1,22		JUC0300005442.mm.1	-2,61	0,001527	0,330287		
TC0100002913.mm.1	1,18		JUC0100013360.mm.1	-2,61	0,002339	0,347265		
TC0900000982.mm.1	1,38	Pygo1	JUC0900004119.mm.1	-2,61	0,027243	0,503642		
TC0900001692.mm.1	-1,15	Aasdhppt	JUC0900008033.mm.1	-2,61	0,026544	0,501435		
TC1100001214.mm.1	-1,12	Gm11418	JUC1100005994.mm.1	-2,61	0,044554	0,551921		

TC0300002671.mm.1	1,03		JUC0300010820.mm.1	-2,61	0,020894	0,479697		
TC0900003316.mm.1	1,04		JUC0900015733.mm.1	-2,61	0,009619	0,417315		
TC0400001527.mm.1	1,24	Tmem234	JUC0400006147.mm.1	-2,61	0,01238	0,434939		
TC0400001527.mm.1	1,24	Tmem234	JUC0400006166.mm.1	-3,9	0,043048	0,54841		
TC0700004652.mm.1	3,59	Snrpn; Snurf	JUC0700015174.mm.1	-2,62	0,040178	0,541655		
TC0700004652.mm.1	3,59	Snrpn; Snurf	PSR0700028716.mm.1	-2,93	0,029467	0,510123	Cassette Exon	0,28
TC0700004652.mm.1	3,59	Snrpn; Snurf	JUC0700015184.mm.1	-3,29	0,039542	0,539764		
TC0700004652.mm.1	3,59	Snrpn; Snurf	JUC0700015172.mm.1	-3,48	0,01226	0,433888		
TC0700004652.mm.1	3,59	Snrpn; Snurf	PSR0700028717.mm.1	-3,78	0,012455	0,43528	Cassette Exon	0,36
TC0700004652.mm.1	3,59	Snrpn; Snurf	PSR0700028715.mm.1	-4,06	0,010116	0,41912	Cassette Exon	0,28
TC0300000112.mm.1	14,82	2610100L16Rik	JUC0300000211.mm.1	-2,62	0,014907	0,451803		
TC0300000112.mm.1	14,82	2610100L16Rik	PSR0300000485.mm.1	-3,19	0,000432	0,297771	Cassette Exon	0,34
TC0300000112.mm.1	14,82	2610100L16Rik	JUC0300000210.mm.1	-6,88	0,006593	0,39213		
TC0300000112.mm.1	14,82	2610100L16Rik	JUC0300000212.mm.1	-17,01	0,004617	0,369794		
TC0300000112.mm.1	14,82	2610100L16Rik	PSR0300000482.mm.1	-24,23	0,003676	0,357266	Alternative 3' Acce	0,35
TC0200002863.mm.1	1,58	Prpf18	PSR0200023736.mm.1	-2,62	0,011314	0,427133	Alternative 3' Acce	0,19
TC0100001257.mm.1	-1,33	Tnnt2	PSR0100010377.mm.1	-2,62	0,004456	0,367394	Cassette Exon	0,17
TC0X00000729.mm.1	1,03	Gm6897	PSR0X00005041.mm.1	-2,62	0,000476	0,298999	Cassette Exon	0,15
TC1200001366.mm.1	1,26	Fkbp1b	PSR1200009792.mm.1	-2,62	0,016768	0,460504		
TC1800001409.mm.1	1,09	Tcof1	JUC1800005713.mm.1	-2,62	0,038121	0,535709		
TC1900000142.mm.1	-1,22	Hrasls5	JUC1900000947.mm.1	-2,62	0,036894	0,532257		
TC0200001724.mm.1	1,3	Fam98b	JUC0200006327.mm.1	-2,62	0,04828	0,56031		
TC1700000473.mm.1	1,01	Def6	JUC1700002220.mm.1	-2,62	0,02901	0,508842		
TC0500003645.mm.1	1,55	Katnal1; 5930430L01Rik	PSR0500033438.mm.1	-2,63	0,005322	0,378059	Alternative 3' Acce	0,23
TC0500003645.mm.1	1,55	Katnal1; 5930430L01Rik	JUC0500018198.mm.1	-3,08	0,008907	0,410728		
TC0200001709.mm.1	1,46	G630016G05Rik	PSR0200012649.mm.1	-2,63	0,00178	0,336311	Cassette Exon	0,16
TC1400000536.mm.1	1,21	Otx2os1; RP23-131O4.2	JUC1400002548.mm.1	-2,63	0,011217	0,426683		
TC1900000116.mm.1	-1,1	Sf1	JUC1900000775.mm.1	-2,63	0,025109	0,496358		
TC1700000423.mm.1	1,01	Gm20468	JUC1700001951.mm.1	-2,63	0,024515	0,493992		
TC0400000376.mm.1	-1,09	Fam219aos; Gm12404; 17	JUC0400001130.mm.1	-2,63	0,035098	0,527591		
TC0X00000855.mm.1	1	Gm26958; RP23-286G19.6	JUC0X00002792.mm.1	-2,63	0,004326	0,364646		
TC1300002418.mm.1	8,48	Rasgrf2	PSR1300016056.mm.1	-2,64	0,030214	0,512452	Cassette Exon	0,16
TC1300002418.mm.1	8,48	Rasgrf2	JUC1300008445.mm.1	-3,21	0,016261	0,458329		
TC1300002418.mm.1	8,48	Rasgrf2	PSR1300016054.mm.1	-3,28	0,025205	0,49668	Cassette Exon	0,26
TC1300002418.mm.1	8,48	Rasgrf2	PSR1300016045.mm.1	-3,7	0,045308	0,553742	Cassette Exon	0,25
TC1300002418.mm.1	8,48	Rasgrf2	JUC1300008451.mm.1	-3,72	0,011181	0,426414		
TC1300002418.mm.1	8,48	Rasgrf2	PSR1300016074.mm.1	-4,25	0,043624	0,550161	Cassette Exon	0,41
TC1300002418.mm.1	8,48	Rasgrf2	PSR1300016057.mm.1	-4,92	0,011346	0,427374	Cassette Exon	0,43
TC1300002418.mm.1	8,48	Rasgrf2	PSR1300016085.mm.1	-6,23	0,019793	0,475212	Cassette Exon	0,32
TC1300002418.mm.1	8,48	Rasgrf2	JUC1300008442.mm.1	-6,81	0,005318	0,378059		
TC1300002418.mm.1	8,48	Rasgrf2	JUC1300008463.mm.1	-9,18	0,012441	0,43528		
TC1300002418.mm.1	8,48	Rasgrf2	JUC1300008450.mm.1	-10,76	0,040204	0,541714		
TC1300002418.mm.1	8,48	Rasgrf2	JUC1300008472.mm.1	-13,69	0,00304	0,353826		
TC1300002418.mm.1	8,48	Rasgrf2	JUC1300008461.mm.1	-13,74	0,029378	0,509995		
TC1700000562.mm.1	-1,5	Cyp4f39	PSR1700005087.mm.1	-2,64	0,025579	0,497808	Alternative 5' Donc	0,26
TC1200002389.mm.1	1,97	Hsp90aa1	PSR1200016571.mm.1	-2,64	0,033564	0,523131		
TC1200002389.mm.1	1,97	Hsp90aa1	PSR1200016570.mm.1	-2,68	0,02758	0,504482		
TC1200002389.mm.1	1,97	Hsp90aa1	PSR1200016578.mm.1	-2,87	0,042319	0,547263	Cassette Exon	0,2

TC0700004318.mm.1	-1,18	sept-01	PSR0700036253.mm.1	-2,64	0,021015	0,480143	Alternative 3' Acce	0,16
TC1200001408.mm.1	1,13	1700022H16Rik	JUC1200005509.mm.1	-2,64	0,000576	0,304044		
TC1900000878.mm.1	1,08	Ighmbp2	JUC1900004140.mm.1	-2,64	0,017575	0,464258		
TC1500001657.mm.1	1,75	Ndrg1	JUC1500007041.mm.1	-2,64	0,030387	0,512923		
TC1500001657.mm.1	1,75	Ndrg1	JUC1500007036.mm.1	-2,92	0,000023	0,22008		
TC1500001657.mm.1	1,75	Ndrg1	JUC1500007044.mm.1	-3,73	0,005475	0,379939		
TC0200001514.mm.1	-1,42	Traf6	JUC0200005685.mm.1	-2,64	0,028844	0,508446		
TC1500002042.mm.1	2,26	Dennd6b	JUC1500009293.mm.1	-2,64	0,014898	0,451802		
TC1500002042.mm.1	2,26	Dennd6b	JUC1500009301.mm.1	-3,13	0,019293	0,473074		
TC1500002042.mm.1	2,26	Dennd6b	JUC1500009296.mm.1	-6,77	0,008428	0,406611		
TC1500000103.mm.1	-1,01	Mtmr12	JUC1500000585.mm.1	-2,64	0,018739	0,470733		
TC0100003595.mm.1	-1,52	Gm16340	JUC0100016783.mm.1	-2,64	0,036631	0,53158		
TC1700000611.mm.1	1,07	Rxrb	JUC1700003127.mm.1	-2,64	0,024347	0,49362		
TC0700000214.mm.1	1,42	Crxos; Crxos1	JUC0700000727.mm.1	-2,64	0,00384	0,358714		
TC0700000214.mm.1	1,42	Crxos; Crxos1	JUC0700000724.mm.1	-2,75	0,00984	0,417839		
TC1000001554.mm.1	-1,19	Tmem194; 1700012D01Ri	JUC1000005918.mm.1	-2,64	0,013138	0,440196		
TC1000002262.mm.1	1,09	2510003E04Rik	PSR1000016155.mm.1	-2,65	0,0034	0,354874	Alternative 3' Acce	0,27
TC1500000595.mm.1	2,47		PSR1500004269.mm.1	-2,65	0,001336	0,325349	Alternative 3' Acce	0,26
TC1500001965.mm.1	1,34	Rrp7a	PSR1500015464.mm.1	-2,65	0,003371	0,354303	Alternative 5' Donc	0,25
TC1500001965.mm.1	1,34	Rrp7a	JUC1500008763.mm.1	-2,71	0,009665	0,41754		
TC0X00003067.mm.1	1,48	Rbm41	PSR0X00019258.mm.1	-2,65	0,024511	0,493992	Alternative 3' Acce	0,22
TC0700004241.mm.1	1,85	Zkscan2	PSR0700035309.mm.1	-2,65	0,007027	0,395972	Cassette Exon	0,18
TC0300001061.mm.1	1,27	Ap4b1	JUC0300004444.mm.1	-2,65	0,019455	0,474165		
TC1500000488.mm.1	-1,06	Tg	JUC1500002023.mm.1	-2,65	0,008179	0,40568		
TC0500000940.mm.1	-1,03	4930467D21Rik	JUC0500004461.mm.1	-2,65	0,036984	0,532502		
TC1100000585.mm.1	1,53	Cdkl3	JUC1100002362.mm.1	-2,65	0,029026	0,508859		
TC1100000585.mm.1	1,53	Cdkl3	JUC1100002372.mm.1	-4,44	0,009097	0,412827		
TC0900003114.mm.1	-1,12	Nradd	PSR0900026308.mm.1	-2,65	0,044011	0,550779		
TC0900002332.mm.1	-1,03	Nrg4	JUC0900011048.mm.1	-2,65	0,003823	0,358315		
TC1000000231.mm.1	1,08	Samd3	JUC1000000895.mm.1	-2,65	0,003866	0,358861		
TC0900003040.mm.1	2,85	Slc38a3	PSR0900025416.mm.1	-2,66	0,029033	0,508904	Alternative 3' Acce	0,25
TC0900003040.mm.1	2,85	Slc38a3	PSR0900025398.mm.1	-3,45	0,022842	0,488353	Alternative 3' Acce	0,39
TC0900003040.mm.1	2,85	Slc38a3	PSR0900025420.mm.1	-5,48	0,006729	0,393343	Cassette Exon	0,28
TC0900003040.mm.1	2,85	Slc38a3	PSR0900025421.mm.1	-5,86	0,004361	0,365487	Cassette Exon	0,28
TC0700002744.mm.1	1,57	Zfp146	PSR0700024315.mm.1	-2,66	0,006962	0,395722	Cassette Exon	0,18
TC1000002684.mm.1	1,17	Gm20757	PSR1000020523.mm.1	-2,66	0,004889	0,372195	Cassette Exon	0,16
TC0200003866.mm.1	-1,19	Tfpi	PSR0200033381.mm.1	-2,66	0,010279	0,420305	Alternative 5' Donc	0,12
TC1400002862.mm.1	1,3	Gm10377; Gm10376	JUC1400007580.mm.1	-2,66	0,010353	0,421002		
TC0200003134.mm.1	-1,2	4933433C11Rik	JUC0200013027.mm.1	-2,66	0,007623	0,401947		
TC1600002059.mm.1	1,26	1110004E09Rik	JUC1600008135.mm.1	-2,66	0,007494	0,400809		
TC0700004526.mm.1	-1,15	Lmntd2	JUC0700020036.mm.1	-2,66	0,022058	0,485327		
TC0500002297.mm.1	-1,19	Hs3st1	JUC0500011537.mm.1	-2,66	0,010965	0,425117		
TC0500003685.mm.1	1,12	N4bp2l1	JUC0500018283.mm.1	-2,66	0,021237	0,481319		
TC1000002191.mm.1	-1,21		JUC1000008369.mm.1	-2,66	0,027969	0,505748		
TC1100003295.mm.1	-1,36	Foxn1	JUC1100015892.mm.1	-2,66	0,014705	0,45037		
TC0600001212.mm.1	1,99	Grm7	PSR0600009350.mm.1	-2,67	0,028087	0,506064	Cassette Exon	0,19
TC0600001212.mm.1	1,99	Grm7	PSR0600009347.mm.1	-2,97	0,009898	0,418332	Cassette Exon	0,09
TC0600001212.mm.1	1,99	Grm7	JUC0600004827.mm.1	-4,12	0,038998	0,537976		

TC1200001908.mm.1	2,17	Dhrs7	PSR1200013093.mm.1	-2,67	0,001702	0,335511	Cassette Exon	0,18
TC0200005143.mm.1	1,03	Cdh22	JUC0200022629.mm.1	-2,67	0,010439	0,421595		
TC1700000600.mm.1	1,03	Daxx	JUC1700003038.mm.1	-2,67	0,0069	0,394768		
TC0200000649.mm.1	1,07	Gm13429	JUC0200003056.mm.1	-2,67	0,005073	0,37524		
TC0X00002464.mm.1	-1,19	Xlr5e-ps	JUC0X00007999.mm.1	-2,67	0,013544	0,44358		
TC0600000964.mm.1	1,38	Alms1	JUC0600003806.mm.1	-2,68	0,025636	0,497989		
TC0600000964.mm.1	1,38	Alms1	PSR0600007466.mm.1	-2,79	0,026606	0,501778	Alternative 3' Acce	0,26
TC0600000964.mm.1	1,38	Alms1	PSR0600007463.mm.1	-4,42	0,029425	0,509995	Alternative 5' Donc	0,49
TC0300003247.mm.1	4,11	Amy1	PSR0300021944.mm.1	-2,68	0,003973	0,360082	Cassette Exon	0,19
TC0300003247.mm.1	4,11	Amy1	JUC0300011442.mm.1	-2,88	0,00532	0,378059		
TC0300003247.mm.1	4,11	Amy1	PSR0300021946.mm.1	-3,52	0,001968	0,340561	Cassette Exon	0,19
TC0300003247.mm.1	4,11	Amy1	PSR0300021943.mm.1	-4,01	0,036732	0,531784	Cassette Exon	0,28
TCX_GL456233_random00	2,02	Tmlhe	PSRX_GL456233_random	-2,68	0,010286	0,420355	Cassette Exon	0,24
TCX_GL456233_random00	2,02	Tmlhe	JUCX_GL456233_random	-2,98	0,012138	0,432868		
TC1300001051.mm.1	-1,23	Dmgdh	PSR1300006702.mm.1	-2,68	0,002958	0,352501	Cassette Exon	0,19
TC0X00000698.mm.1	1,03	Avpr2	PSR0X00004603.mm.1	-2,68	0,018442	0,468671	Alternative 5' Donc	0,18
TC1700001112.mm.1	1,54	A330050F15Rik	JUC1700005678.mm.1	-2,68	0,04439	0,551441		
TC1900001765.mm.1	1,05	Vps51; Tm7sf2	JUC1900005021.mm.1	-2,68	0,014354	0,448293		
TC1900001765.mm.1	1,05	Vps51; Tm7sf2	JUC1900005028.mm.1	-3,87	0,01466	0,450098		
TC1600001752.mm.1	-1,3	Tmem45a	JUC1600007408.mm.1	-2,68	0,017144	0,46232		
TC0100003316.mm.1	1,01	Tdrd5	JUC0100015469.mm.1	-2,68	0,046832	0,557144		
TC1100000074.mm.1	-1,12	Ankrd36	JUC1100000397.mm.1	-2,68	0,012038	0,432286		
TC1100000876.mm.1	-1,16	Ccdc42	JUC1100003855.mm.1	-2,68	0,000718	0,306015		
TC0900002776.mm.1	1,52	Ube2cbp	JUC0900012860.mm.1	-2,68	0,003549	0,356223		
TC0900002776.mm.1	1,52	Ube2cbp	JUC0900012858.mm.1	-2,72	0,007599	0,401712		
TC0900002776.mm.1	1,52	Ube2cbp	JUC0900012850.mm.1	-2,8	0,016483	0,459355		
TC0900002776.mm.1	1,52	Ube2cbp	JUC0900012859.mm.1	-3,05	0,005398	0,378916		
TC0200004897.mm.1	3,24	Nrsn2	PSR0200041603.mm.1	-2,69	0,03941	0,539566	Cassette Exon	0,29
TC0200004897.mm.1	3,24	Nrsn2	JUC0200021504.mm.1	-4,23	0,016575	0,459612		
TC1200000629.mm.1	3,26	Six6	PSR1200004361.mm.1	-2,69	0,005281	0,378059	Alternative 3' Acce	0,27
TC1200000629.mm.1	3,26	Six6	JUC1200002449.mm.1	-3,67	0,000088	0,255472		
TC0300001060.mm.1	-1,06	Gm15471	PSR0300008567.mm.1	-2,69	0,004998	0,373851		
TC1800001083.mm.1	-1	Slc39a6	JUC1800004441.mm.1	-2,69	0,015727	0,455416		
TC1900000168.mm.1	1,39	Mta2	JUC1900001176.mm.1	-2,69	0,028753	0,508148		
TC1600000033.mm.1	1,17	Hmox2	JUC1600000122.mm.1	-2,69	0,014774	0,450801		
TC1600000033.mm.1	1,17	Hmox2	PSR1600000271.mm.1	-3,07	0,034617	0,52602		
TC0100003421.mm.1	-1,31	Blzf1	JUC0100016065.mm.1	-2,69	0,03118	0,515739		
TC0800002579.mm.1	1,07	Mri1	JUC0800010711.mm.1	-2,69	0,002669	0,349612		
TC0500001073.mm.1	-1,3	Brdt	PSR0500009037.mm.1	-2,7	0,008352	0,406435	Cassette Exon	0,16
TC1700001190.mm.1	1,08	Sult6b1	PSR1700011251.mm.1	-2,7	0,040782	0,543175	Cassette Exon	0,16
TC1900001504.mm.1	-1,09	Tll2	JUC1900007249.mm.1	-2,7	0,046671	0,556812		
TC1900000937.mm.1	-1,09	Ccs	JUC1900004518.mm.1	-2,7	0,007673	0,402377		
TC0900000614.mm.1	1,12		JUC0900002320.mm.1	-2,7	0,005745	0,382993		
TC0700000251.mm.1	-1,16	Ceacam3	JUC0700000906.mm.1	-2,7	0,004761	0,371039		
TC0300001834.mm.1	-1,08	Gm3143	JUC0300007447.mm.1	-2,7	0,040802	0,543194		
TC0X00002721.mm.1	1,11	Zmym3	JUC0X00008708.mm.1	-2,7	0,029966	0,511499		
TC0700002617.mm.1	6,13	Atp1a3	PSR0700022556.mm.1	-2,71	0,009066	0,412827	Alternative 3' Acce	0,19
TC0700002617.mm.1	6,13	Atp1a3	JUC0700011977.mm.1	-2,75	0,028001	0,505848		

TC0700002617.mm.1	6,13	Atp1a3	PSR0700022584.mm.1	-3,56	0,000369	0,291725	Cassette Exon	0,26
TC0700002617.mm.1	6,13	Atp1a3	PSR0700022558.mm.1	-3,69	0,002263	0,34578	Intron Retention	0,54
TC0700002617.mm.1	6,13	Atp1a3	PSR0700022588.mm.1	-7,52	0,004633	0,36999	Cassette Exon	0,26
TC0300001562.mm.1	4,06	Negr1	JUC0300006684.mm.1	-2,71	0,005403	0,378967		
TC0300001562.mm.1	4,06	Negr1	PSR0300012623.mm.1	-2,95	0,03031	0,512738	Cassette Exon	0,18
TC0300001562.mm.1	4,06	Negr1	PSR0300012625.mm.1	-3,69	0,042955	0,548357	Cassette Exon	0,25
TC0300001562.mm.1	4,06	Negr1	PSR0300012611.mm.1	-5,27	0,008682	0,408815	Alternative 3' Acce	0,37
TC0300001562.mm.1	4,06	Negr1	PSR0300012610.mm.1	-7,03	0,034587	0,52602	Alternative 3' Acce	0,37
TC0500002008.mm.1	1,41	Rsbn1l	PSR0500018259.mm.1	-2,71	0,001008	0,316361	Alternative 5' Donc	0,28
TC0Y00000357.mm.1	1,1	Gm20881; Ssty2; Gm2079	PSR0Y00002131.mm.1	-2,71	0,011668	0,429939	Alternative 3' Acce	0,27
TC0Y00000419.mm.1	1,1	Gm21617; Ssty2; Gm2079	PSR0Y00002324.mm.1	-2,71	0,011668	0,429939	Alternative 3' Acce	0,27
TC0900002126.mm.1	1,58	Tmem136	PSR0900017686.mm.1	-2,71	0,032204	0,51885	Cassette Exon	0,16
TC0600000438.mm.1	1,29	Gm5771	JUC0600001762.mm.1	-2,71	0,001051	0,316361		
TC1100000609.mm.1	1,75	Kif3a	JUC1100002511.mm.1	-2,71	0,002611	0,349501		
TC0400004177.mm.1	-1,16	Akap2	JUC0400002240.mm.1	-2,71	0,014207	0,447756		
TC0900002971.mm.1	1,25	Bfsp2	JUC0900013662.mm.1	-2,71	0,0231	0,489699		
TC1300001857.mm.1	11,14	Mak	JUC1300005937.mm.1	-2,72	0,031779	0,517596		
TC1300001857.mm.1	11,14	Mak	JUC1300005938.mm.1	-3,04	0,004502	0,368473		
TC1300001857.mm.1	11,14	Mak	PSR1300011580.mm.1	-3,11	0,018965	0,471398	Cassette Exon	0,32
TC1300001857.mm.1	11,14	Mak	JUC1300005945.mm.1	-3,64	0,032443	0,519525		
TC1300001857.mm.1	11,14	Mak	PSR1300011574.mm.1	-4,02	0,016128	0,457772	Cassette Exon	0,25
TC1300001857.mm.1	11,14	Mak	JUC1300005947.mm.1	-6,26	0,016479	0,459352		
TC1300001857.mm.1	11,14	Mak	JUC1300005934.mm.1	-9,7	0,006098	0,386974		
TC1300001857.mm.1	11,14	Mak	PSR1300011590.mm.1	-12,91	0,008802	0,409933	Cassette Exon	0,41
TC1300001857.mm.1	11,14	Mak	JUC1300005949.mm.1	-15,91	0,003844	0,358723		
TC1400001115.mm.1	2,84	Pcdh17	PSR1400008917.mm.1	-2,72	0,010447	0,421652	Cassette Exon	0,19
TC1400001115.mm.1	2,84	Pcdh17	PSR1400008936.mm.1	-2,86	0,030187	0,512365	Alternative 5' Donc	0,3
TC1400001115.mm.1	2,84	Pcdh17	PSR1400008920.mm.1	-4,37	0,004927	0,373093	Cassette Exon	0,39
TC1000001137.mm.1	1,28		PSR1000008620.mm.1	-2,72	0,048042	0,559637	Cassette Exon	0,16
TC1200001989.mm.1	1,19	2310015A10Rik	JUC1200007583.mm.1	-2,72	0,019075	0,472139		
TC0600002095.mm.1	-1,06	Svopl	JUC0600008615.mm.1	-2,72	0,045588	0,554548		
TC0500001690.mm.1	1,08		JUC0500008425.mm.1	-2,72	0,007441	0,399934		
TC0600000991.mm.1	1,55	1600020E01Rik	JUC0600003946.mm.1	-2,72	0,03411	0,524882		
TC0700000776.mm.1	-1,2	Klk7	JUC0700003137.mm.1	-2,72	0,023025	0,489209		
TC0700004129.mm.1	-1,02	1700006P19Rik; Far1os	JUC0700017980.mm.1	-2,72	0,018463	0,468677		
TC0700001718.mm.1	1,24	Lyrm1	JUC0700007491.mm.1	-2,72	0,009333	0,414981		
TC1100002907.mm.1	1,31	Aldh3a2	JUC1100013730.mm.1	-2,72	0,022094	0,485453		
TC0900003244.mm.1	-1,01	Scn5a	JUC0900015361.mm.1	-2,72	0,030468	0,513244		
TC1800001107.mm.1	5,13	Rit2	PSR1800008081.mm.1	-2,73	0,004692	0,370481	Cassette Exon	0,2
TC1800001107.mm.1	5,13	Rit2	JUC1800004490.mm.1	-3,37	0,009635	0,417354		
TC1800001107.mm.1	5,13	Rit2	PSR1800008075.mm.1	-3,49	0,000665	0,304044	Alternative 3' Acce	0,35
TC1800001107.mm.1	5,13	Rit2	JUC1800004493.mm.1	-3,76	0,041101	0,543933		
TC1800001107.mm.1	5,13	Rit2	PSR1800008077.mm.1	-3,99	0,011876	0,431125	Cassette Exon	0,36
TC1800001107.mm.1	5,13	Rit2	JUC1800004494.mm.1	-5,58	0,026021	0,499406		
TC1800001107.mm.1	5,13	Rit2	PSR1800008084.mm.1	-7,92	0,003852	0,358861	Alternative 5' Donc	0,2
TC1800001107.mm.1	5,13	Rit2	PSR1800008072.mm.1	-8,91	0,007188	0,397891	Alternative 3' Acce	0,47
TC0200002052.mm.1	-1,05	Bmp2	PSR0200016318.mm.1	-2,73	0,020109	0,47686	Cassette Exon	0,16
TC0200005495.mm.1	1,11	Gm16534	JUC0200014102.mm.1	-2,73	0,007659	0,402377		

TC1200000967.mm.1	1,18	Spata7	JUC1200003751.mm.1	-2,73	0,021802	0,484487		
TC1200000680.mm.1	-1,2	Ppp1r36	JUC1200002740.mm.1	-2,73	0,032196	0,518843		
TC0800002711.mm.1	-1,1	Ces5a	JUC0800011315.mm.1	-2,73	0,04842	0,560736		
TC0400001773.mm.1	1,19	Atp13a2	JUC0400007724.mm.1	-2,73	0,001063	0,316361		
TC0400001773.mm.1	1,19	Atp13a2	JUC0400007726.mm.1	-2,77	0,002105	0,342919		
TC0700000087.mm.1	2,56	Usp29	JUC0700000452.mm.1	-2,74	0,012516	0,435429		
TC0700000087.mm.1	2,56	Usp29	JUC0700000455.mm.1	-3,06	0,035542	0,528764		
TC0700000087.mm.1	2,56	Usp29	PSR0700000912.mm.1	-3,39	0,047129	0,557915	Cassette Exon	0,42
TC0700000087.mm.1	2,56	Usp29	JUC0700000446.mm.1	-6,23	0,016523	0,459611		
TC1100000238.mm.1	1,39	Xpo1	PSR1100002000.mm.1	-2,74	0,025991	0,499207	Alternative 5' Donc	0,28
TC0400004171.mm.1	-1,18	Isg15	PSR0400035094.mm.1	-2,74	0,047334	0,558291	Cassette Exon	0,16
TC1300002329.mm.1	1,15		JUC1300008118.mm.1	-2,74	0,025145	0,496487		
TC1800000783.mm.1	-1,28	Mro	JUC1800003087.mm.1	-2,74	0,023994	0,492783		
TC1900000064.mm.1	1,26	4930481A15Rik	JUC1900000412.mm.1	-2,74	0,020244	0,477245		
TC1900000064.mm.1	1,26	4930481A15Rik	JUC1900000417.mm.1	-4,81	0,010454	0,421652		
TC1600000116.mm.1	-1,05	Ciita	JUC1600000411.mm.1	-2,74	0,003213	0,354243		
TC0300002553.mm.1	-1,38	Gm10681; Hsd3b4	JUC0300010380.mm.1	-2,74	0,018966	0,471398		
TC1100002247.mm.1	-1,13	Eldr; 2810442I21Rik	JUC1100011365.mm.1	-2,74	0,009479	0,416578		
TC0400001685.mm.1	1,97	Gm17388	JUC0400007019.mm.1	-2,74	0,020912	0,479765		
TC1000000053.mm.1	1,47	Ppil4	PSR1000000421.mm.1	-2,75	0,021009	0,480143	Intron Retention	0,44
TC0500003084.mm.1	-1,05	Hnf1a	PSR0500027430.mm.1	-2,75	0,016233	0,458312	Alternative 5' Donc	0,28
TC1100002175.mm.1	2,61	Purb; Gm22123	PSR1100021165.mm.1	-2,75	0,034385	0,525677	Alternative 5' Donc	0,28
TC1100001123.mm.1	1,02	Coro6	PSR1100010172.mm.1	-2,75	0,036556	0,531355	Alternative 3' Acce	0,27
TC0900003160.mm.1	-1,09	Pdcd6ip	PSR0900026709.mm.1	-2,75	0,016563	0,459612	Alternative 3' Acce	0,24
TC1400000763.mm.1	1,57	Cmtm5	PSR1400005943.mm.1	-2,75	0,044803	0,552497	Cassette Exon	0,23
TC0500000582.mm.1	1,42	Nwd2; 3110047P20Rik	PSR0500005167.mm.1	-2,75	0,000509	0,303579	Alternative 3' Acce	0,19
TC0900001315.mm.1	1,59	Cpne4	PSR0900010267.mm.1	-2,75	0,000008	0,179072	Cassette Exon	0,16
TC1200000165.mm.1	1,65	Gm5784	JUC1200000770.mm.1	-2,75	0,045876	0,555047		
TC0500002095.mm.1	-1,15	Prkag2	JUC0500010408.mm.1	-2,75	0,015696	0,455416		
TC0500001651.mm.1	-1,05	Stag3	JUC0500008237.mm.1	-2,75	0,000944	0,313363		
TC0100001904.mm.1	8,48	Rp1	JUC0100008928.mm.1	-2,76	0,041059	0,543798		
TC0100001904.mm.1	8,48	Rp1	PSR0100015721.mm.1	-3,05	0,01896	0,471398	Cassette Exon	0,37
TC0100001904.mm.1	8,48	Rp1	PSR0100015715.mm.1	-3,08	0,018327	0,468135	Cassette Exon	0,41
TC0100001904.mm.1	8,48	Rp1	PSR0100015716.mm.1	-3,59	0,01505	0,452177	Cassette Exon	0,45
TC0100001904.mm.1	8,48	Rp1	JUC0100008927.mm.1	-4,19	0,012866	0,437829		
TC0100001904.mm.1	8,48	Rp1	PSR0100015723.mm.1	-4,29	0,034431	0,525858	Cassette Exon	0,41
TC0100001904.mm.1	8,48	Rp1	PSR0100015725.mm.1	-4,9	0,002604	0,349501	Alternative 3' Acce	0,48
TC0100001904.mm.1	8,48	Rp1	PSR0100015717.mm.1	-5,91	0,034333	0,525546	Cassette Exon	0,46
TC0100001904.mm.1	8,48	Rp1	PSR0100015731.mm.1	-7,42	0,023948	0,4926	Cassette Exon	0,41
TC0100001904.mm.1	8,48	Rp1	JUC0100008929.mm.1	-8,82	0,009914	0,418378		
TC0100001904.mm.1	8,48	Rp1	PSR0100015718.mm.1	-9,43	0,031033	0,515011	Alternative 3' Acce	0,24
TC0100001904.mm.1	8,48	Rp1	PSR0100015720.mm.1	-9,95	0,006462	0,390372	Alternative 5' Donc	0,33
TC0100001904.mm.1	8,48	Rp1	PSR0100015722.mm.1	-16,11	0,009985	0,418456	Cassette Exon	0,41
TC0100001904.mm.1	8,48	Rp1	JUC0100008936.mm.1	-18,57	0,003509	0,355749		
TC0100001904.mm.1	8,48	Rp1	JUC0100008931.mm.1	-22,01	0,015627	0,45509		
TC0100001904.mm.1	8,48	Rp1	JUC0100008934.mm.1	-31,49	0,025834	0,498598		
TC1700002204.mm.1	15,13	Guca1a	PSR1700020809.mm.1	-2,76	0,02335	0,490143	Cassette Exon	0,32
TC1700002204.mm.1	15,13	Guca1a	PSR1700020808.mm.1	-4,56	0,009677	0,417605	Cassette Exon	0,41

TC1700002204.mm.1	15,13	Guca1a	JUC1700011100.mm.1	-4,68	0,047107	0,557863		
TC1700002204.mm.1	15,13	Guca1a	PSR1700020812.mm.1	-7,03	0,004774	0,371044	Alternative 5' Donc	0,48
TC0600003266.mm.1	1,09	Magohb	PSR0600025698.mm.1	-2,76	0,001135	0,317344	Alternative 5' Donc	0,28
TC1300002471.mm.1	-1,16	Polk	PSR1300016560.mm.1	-2,76	0,010171	0,419711	Alternative 5' Donc	0,24
TC1400001243.mm.1	-1,23	4930524C18Rik	JUC1400005095.mm.1	-2,76	0,030903	0,514574		
TC0200002742.mm.1	1,35	Gm14408	JUC0200011352.mm.1	-2,76	0,003045	0,353892		
TC1700001088.mm.1	1,09		JUC1700005538.mm.1	-2,76	0,015448	0,454271		
TC1700001929.mm.1	1,04	Skiv2l; Mir6970; mmu-mi	JUC1700009758.mm.1	-2,76	0,000406	0,297771		
TC1500000659.mm.1	1,06	Nol12	JUC1500002780.mm.1	-2,76	0,008474	0,406734		
TC0800000977.mm.1	1,32	Tnpo2	JUC0800003957.mm.1	-2,76	0,03092	0,514584		
TC0500001663.mm.1	1,31	Fam20c	JUC0500008308.mm.1	-2,76	0,021288	0,481708		
TC0400000426.mm.1	1,1	Dnajb5	JUC0400001303.mm.1	-2,76	0,031486	0,516965		
TC0300002354.mm.1	-1,6		JUC0300009501.mm.1	-2,76	0,008134	0,405267		
TC1300002772.mm.1	1,03	Zfp595	PSR1300014583.mm.1	-2,77	0,020802	0,479494	Intron Retention	0,27
TC0500000373.mm.1	1,99	Rnf4	PSR0500003540.mm.1	-2,77	0,016641	0,459985	Alternative 3' Acce	0,24
TC0500000373.mm.1	1,99	Rnf4	PSR0500003563.mm.1	-3,2	0,018168	0,467441	Cassette Exon	0,22
TC0100000381.mm.1	1,08	Hibch	PSR0100002975.mm.1	-2,77	0,040023	0,54127	Cassette Exon	0,21
TC0300002532.mm.1	-1,11	Gja8	PSR0300019759.mm.1	-2,77	0,046279	0,556186	Alternative 5' Donc	0,21
TC1200002089.mm.1	-1,03	Pgf	JUC1200007971.mm.1	-2,77	0,026862	0,502454		
TC1300000303.mm.1	-1,27	Prl8a2	JUC1300000955.mm.1	-2,77	0,00458	0,369794		
TC1500000705.mm.1	1,68	Tnrc6b; Mir5113	JUC1500003054.mm.1	-2,77	0,011089	0,425942		
TC0700004397.mm.1	1,11	2310057M21Rik	JUC0700019493.mm.1	-2,77	0,015164	0,452699		
TC0400001163.mm.1	-1,04	Calr4	JUC0400004338.mm.1	-2,77	0,002577	0,349501		
TC0100000164.mm.1	1,36	Col9a1	PSR0100001081.mm.1	-2,78	0,000813	0,311886	Alternative 5' Donc	0,29
TC0600002980.mm.1	-1,4	Tmem72	PSR0600022953.mm.1	-2,78	0,018787	0,470789	Cassette Exon	0,15
TC1400001993.mm.1	1,4	Osgep	JUC1400008198.mm.1	-2,78	0,008635	0,408463		
TC1200001035.mm.1	-1,15	Serpina4-ps1	JUC1200004140.mm.1	-2,78	0,03238	0,519221		
TC1700002000.mm.1	1,05	Gm9574	JUC1700010242.mm.1	-2,78	0,009074	0,412827		
TC1400002725.mm.1	-1,19	Oxgr1	JUC1400011179.mm.1	-2,78	0,021109	0,480525		
TC0700001106.mm.1	-1,16		JUC0700004990.mm.1	-2,78	0,022215	0,485896		
TC0900001380.mm.1	6,76		PSR0900010883.mm.1	-2,79	0,023907	0,492316	Alternative 3' Acce	0,29
TC0900001380.mm.1	6,76		JUC0900005984.mm.1	-6,13	0,008749	0,409704		
TC0X00002036.mm.1	-1,09	Gm14552; Gm3669; Gm2	PSR0X00012930.mm.1	-2,79	0,047206	0,558132	Alternative 3' Acce	0,29
TC1700000419.mm.1	1,32	Crebrf	PSR1700003581.mm.1	-2,79	0,005593	0,381047	Alternative 3' Acce	0,28
TC0200003607.mm.1	-1,05	Rbms1	PSR0200030809.mm.1	-2,79	0,01331	0,441836	Alternative 3' Acce	0,23
TC0800003099.mm.1	-1,16	Fanca	PSR0800024118.mm.1	-2,79	0,016935	0,460959	Cassette Exon	0,17
TC0800001050.mm.1	1,25	Chd9	PSR0800008086.mm.1	-2,79	0,012302	0,434371	Cassette Exon	0,12
TC1700000923.mm.1	1,02	Pgc	JUC1700004744.mm.1	-2,79	0,043834	0,550456		
TC0100000258.mm.1	1,01	Cnga3	JUC0100000987.mm.1	-2,79	0,037208	0,533262		
TC1800001595.mm.1	1,03	Lipg	JUC1800006407.mm.1	-2,79	0,0203	0,477245		
TC1000003140.mm.1	1,18	Coq10a	JUC1000012955.mm.1	-2,79	0,020305	0,477245		
TC0X00003395.mm.1	-1,08	Med12	JUC0X00003153.mm.1	-2,79	0,009178	0,41357		
TC0900000262.mm.1	4,43	Opcml	JUC0900001087.mm.1	-2,8	0,043533	0,549995		
TC0900000262.mm.1	4,43	Opcml	PSR0900002000.mm.1	-3,52	0,027091	0,503022	Alternative 3' Acce	0,35
TC0900000262.mm.1	4,43	Opcml	PSR0900001994.mm.1	-3,77	0,034144	0,524893	Cassette Exon	0,34
TC0900000262.mm.1	4,43	Opcml	PSR0900001989.mm.1	-3,85	0,046084	0,555668	Cassette Exon	0,26
TC0900000262.mm.1	4,43	Opcml	JUC0900001090.mm.1	-3,85	0,041235	0,544134		
TC0900000262.mm.1	4,43	Opcml	PSR0900001996.mm.1	-4,58	0,037508	0,533968	Alternative 5' Donc	0,16

TC0900000262.mm.1	4,43	Opcml	PSR0900002005.mm.1	-7,36	0,040538	0,542414	Cassette Exon	0,28
TC0900000262.mm.1	4,43	Opcml	PSR0900001997.mm.1	-7,85	0,032391	0,519278	Cassette Exon	0,16
TC0900000262.mm.1	4,43	Opcml	PSR0900001991.mm.1	-11,18	0,015091	0,452418	Alternative 3' Acce	0,04
TC0900000262.mm.1	4,43	Opcml	JUC0900001089.mm.1	-13,06	0,006947	0,395542		
TC0900000262.mm.1	4,43	Opcml	JUC0900001091.mm.1	-14,99	0,006423	0,389915		
TC1000002074.mm.1	1,44		PSR1000014818.mm.1	-2,8	0,004968	0,373851	Cassette Exon	0,15
TC1400000748.mm.1	1,02	Rem2	JUC1400003132.mm.1	-2,8	0,0329	0,521149		
TC0500001980.mm.1	-1,31	Speer4c	JUC0500009830.mm.1	-2,8	0,03849	0,536557		
TC0900001016.mm.1	1,13	Leo1	JUC0900004363.mm.1	-2,8	0,011134	0,426153		
TC0400000481.mm.1	-1,23	Grhpr	JUC0400001639.mm.1	-2,8	0,037135	0,533013		
TC0400003605.mm.1	1,12	Dcdc2b	JUC0400015335.mm.1	-2,8	0,007941	0,403819		
TC0900003066.mm.1	1,59	Arih2; Gm26298	PSR0900025834.mm.1	-2,81	0,024334	0,49362	Alternative 5' Donc	0,29
TC1100002064.mm.1	1,89	Fn3krp	PSR1100019932.mm.1	-2,81	0,037426	0,533831	Alternative 3' Acce	0,29
TC1400002661.mm.1	-1,3	Mir6390; mmu-mir-6390;	PSR1400019945.mm.1	-2,81	0,000524	0,304044	Intron Retention	0,14
TC1700000355.mm.1	1,08	Igfals	JUC1700001530.mm.1	-2,81	0,040131	0,541572		
TC0800001422.mm.1	-1,01	Dnaaf1	JUC0800006334.mm.1	-2,81	0,009163	0,413447		
TC0800000390.mm.1	-1,12	Tex15	JUC0800001556.mm.1	-2,81	0,03322	0,522175		
TC0700000077.mm.1	1,8	Zfp78	JUC0700000429.mm.1	-2,81	0,04542	0,554105		
TC0700000077.mm.1	1,8	Zfp78	JUC0700000427.mm.1	-3,23	0,009186	0,413756		
TC0500002994.mm.1	-1,06	Ep400; Gm26079	JUC0500014506.mm.1	-2,81	0,006202	0,387873		
TC0900002300.mm.1	2,33	AI593442	PSR0900019156.mm.1	-2,82	0,013944	0,446234	Cassette Exon	0,19
TC0600001972.mm.1	1,9	Prrt4	JUC0600008090.mm.1	-2,82	0,033973	0,524252		
TC1100000621.mm.1	1,39	Acsl6	JUC1100002598.mm.1	-2,82	0,026312	0,500687		
TC1100000621.mm.1	1,39	Acsl6	JUC1100002603.mm.1	-3,14	0,02797	0,505748		
TC1100001785.mm.1	-1,23	Milr1; Gm885	JUC1100008779.mm.1	-2,82	0,036436	0,530977		
TC1100001785.mm.1	-1,23	Milr1; Gm885	JUC1100008772.mm.1	-3,21	0,014694	0,450265		
TC0900002009.mm.1	-1,07	Foxred1	JUC0900009442.mm.1	-2,82	0,047088	0,557835		
TC0400002041.mm.1	1,13	Wrap73	JUC0400008833.mm.1	-2,82	0,020681	0,478929		
TC0X00002292.mm.1	1,52	Fgf13	JUC0X00007402.mm.1	-2,83	0,041104	0,543933		
TC0X00002292.mm.1	1,52	Fgf13	PSR0X00014505.mm.1	-2,91	0,045075	0,553115	Cassette Exon	0,26
TC0600001372.mm.1	-1,64	A2m	PSR0600010993.mm.1	-2,83	0,003499	0,355638	Cassette Exon	0,2
TC0200002584.mm.1	1,63	1110018N20Rik	PSR0200021276.mm.1	-2,83	0,00057	0,304044	Cassette Exon	0,1
TC1700002688.mm.1	1,05	4833418N02Rik	JUC1700013136.mm.1	-2,83	0,001987	0,341091		
TC0800001223.mm.1	1,06	Ctcf	JUC0800005269.mm.1	-2,83	0,041306	0,544259		
TC0600000940.mm.1	1,11		JUC0600003647.mm.1	-2,83	0,030089	0,511932		
TC0600001163.mm.1	-1,53		JUC0600004586.mm.1	-2,83	0,040578	0,542469		
TC0500003111.mm.1	1,12	Tmem233	JUC0500015089.mm.1	-2,83	0,045269	0,553703		
TC1100002195.mm.1	1,3	Igfbp3	JUC1100011129.mm.1	-2,83	0,038014	0,535454		
TC0500000036.mm.1	-1,27		PSR0500000328.mm.1	-2,83	0,005744	0,382993		
TC0700001576.mm.1	1,19	Syt9	PSR0700013197.mm.1	-2,84	0,010244	0,420147	Alternative 3' Acce	0,29
TC1900000425.mm.1	1,42		JUC1900002044.mm.1	-2,84	0,013385	0,442388		
TC1900000425.mm.1	1,42		PSR1900003934.mm.1	-3,19	0,016337	0,458616	Alternative 3' Acce	0,27
TC1000001309.mm.1	2,09	Kcnc2	PSR1000009802.mm.1	-2,84	0,032042	0,518459	Cassette Exon	0,17
TC1100004174.mm.1	2,13	Gm11754	JUC1100020855.mm.1	-2,84	0,015894	0,456526		
TC1100004174.mm.1	2,13	Gm11754	JUC1100020856.mm.1	-3,48	0,019307	0,473208		
TC1200002493.mm.1	1,34	Wdr60	JUC1200010434.mm.1	-2,84	0,019432	0,473969		
TC1300000444.mm.1	1,23		JUC1300001378.mm.1	-2,84	0,016811	0,460623		
TC0100000954.mm.1	-1,05	Neu4	JUC0100004663.mm.1	-2,84	0,018297	0,468049		

TC0800002242.mm.1	1,01		JUC0800009296.mm.1	-2,84	0,028274	0,506588		
TC0600002800.mm.1	-1,61	Lmod3	JUC0600011302.mm.1	-2,84	0,035942	0,529399		
TC0700003014.mm.1	1,73	A030001D20Rik; Gm1604	JUC0700014606.mm.1	-2,84	0,014016	0,446478		
TC1000002535.mm.1	-1,24	Tjp3	JUC1000010420.mm.1	-2,84	0,011639	0,42984		
TC0400003938.mm.1	1,03	Pramel5	JUC0400016997.mm.1	-2,84	0,03302	0,521478		
TC1000001038.mm.1	1,18	Pwp1	JUC1000004196.mm.1	-2,84	0,045459	0,554224		
TC0800001433.mm.1	3,45	Gm15898	PSR0800011749.mm.1	-2,85	0,024114	0,49322	Cassette Exon	0,17
TC0600001479.mm.1	-1,08	9330179D12Rik	PSR0600012188.mm.1	-2,85	0,00909	0,412827	Cassette Exon	0,16
TC0200005283.mm.1	1,22		JUC0200023216.mm.1	-2,85	0,030307	0,512738		
TC0200004408.mm.1	1,22	A930104D05Rik	JUC0200018830.mm.1	-2,85	0,012651	0,43631		
TC1700002588.mm.1	1,29	Map4k3	JUC1700012765.mm.1	-2,85	0,049348	0,562803		
TC1600000759.mm.1	-1,08		JUC1600003496.mm.1	-2,85	0,004649	0,370181		
TC0900001101.mm.1	-1,03	Ttk	JUC0900004693.mm.1	-2,85	0,008835	0,410293		
TC0400000916.mm.1	-1,22	Gm12609; Gm12610	JUC0400003137.mm.1	-2,85	0,031994	0,518312		
TC1100002777.mm.1	-1	Slc36a2	JUC1100013091.mm.1	-2,85	0,049749	0,563589		
TC0700001284.mm.1	2,55	Dlg2	PSR0700011110.mm.1	-2,86	0,014047	0,44654	Alternative 5' Donc	0,26
TC1900000005.mm.1	-1,12	Mrpl21	JUC1900000008.mm.1	-2,86	0,010051	0,418902		
TC0600001648.mm.1	1,01	Pik3c2g	JUC0600006877.mm.1	-2,86	0,020713	0,479166		
TC0500003175.mm.1	-2,59	Oas2	JUC0500015347.mm.1	-2,86	0,045926	0,555257		
TC1100001131.mm.1	1,21	Nufip2	JUC1100005386.mm.1	-2,86	0,007156	0,397811		
TC0300002617.mm.1	1,04	Tshb	JUC0300010631.mm.1	-2,86	0,036501	0,5313		
TC0400003738.mm.1	1,01	Extl1	JUC0400016001.mm.1	-2,86	0,002649	0,349612		
TC1100003511.mm.1	4,97	Dgke	JUC1100016812.mm.1	-2,87	0,044904	0,55271		
TC1100003511.mm.1	4,97	Dgke	PSR1100032135.mm.1	-3,1	0,00107	0,316484	Alternative 3' Acce	0,34
TC1100003511.mm.1	4,97	Dgke	PSR1100032147.mm.1	-3,15	0,014932	0,451919	Cassette Exon	0,33
TC1100003511.mm.1	4,97	Dgke	PSR1100032143.mm.1	-3,79	0,001358	0,325659	Alternative 5' Donc	0,42
TC1100003511.mm.1	4,97	Dgke	PSR1100032145.mm.1	-5,08	0,000985	0,315219	Cassette Exon	0,16
TC1100003511.mm.1	4,97	Dgke	PSR1100032146.mm.1	-5,68	0,007456	0,400326	Cassette Exon	0,32
TC1100003511.mm.1	4,97	Dgke	JUC1100016806.mm.1	-6,67	0,000274	0,28803		
TC1100003511.mm.1	4,97	Dgke	PSR1100032144.mm.1	-7,83	0,004688	0,3704	Alternative 5' Donc	0,42
TC1100003511.mm.1	4,97	Dgke	JUC1100016808.mm.1	-13,87	0,001809	0,336544		
TC0500000019.mm.1	1,52	Krit1	PSR0500000177.mm.1	-2,87	0,024291	0,49362	Intron Retention	0,33
TC0500000019.mm.1	1,52	Krit1	JUC0500000097.mm.1	-4,54	0,008487	0,406895		
TC1700000717.mm.1	1,43	A930015D03Rik	PSR1700007382.mm.1	-2,87	0,012491	0,435429	Alternative 3' Acce	0,28
TC0400000413.mm.1	2,97	Ccl27b; Ccl27a; LOC10086	PSR0400002645.mm.1	-2,87	0,005007	0,373851	Cassette Exon	0,23
TC0400000413.mm.1	2,97	Ccl27b; Ccl27a; LOC10086	JUC0400001277.mm.1	-3,71	0,005208	0,377078		
TC0100001749.mm.1	1	Capn8	PSR0100014598.mm.1	-2,87	0,040208	0,541714	Cassette Exon	0,14
TC0200002721.mm.1	1,07	Gm14436; Gm14306; Gm8	JUC0200011284.mm.1	-2,87	0,000093	0,256273		
TC1300000099.mm.1	1,1		JUC1300000456.mm.1	-2,87	0,007405	0,399876		
TC1700001034.mm.1	-1,5	Emr1	JUC1700005334.mm.1	-2,87	0,041856	0,545617		
TC1900000327.mm.1	-1,38	Trpm6	JUC1900001692.mm.1	-2,87	0,025917	0,499027		
TC0600003000.mm.1	-1,12	Bms1	JUC0600011965.mm.1	-2,87	0,021252	0,481431		
TC0700002815.mm.1	-1,07	Scgb2b23-ps; Scgb2b1	JUC0700013451.mm.1	-2,87	0,02548	0,497664		
TC0500003208.mm.1	1,44	Fam216a	JUC0500015621.mm.1	-2,87	0,044404	0,5515		
TC0700003652.mm.1	-1,09	Arnt2	JUC0700016385.mm.1	-2,87	0,031756	0,517489		
TC0400003215.mm.1	-1,14	Mrpl37	JUC0400013317.mm.1	-2,87	0,00365	0,356935		
TC0500000084.mm.1	-1,14	Sema3a	JUC0500000430.mm.1	-2,87	0,01198	0,432263		
TC0400003826.mm.1	1,17	Vwa5b1	JUC0400016382.mm.1	-2,87	0,0065	0,39101		

TC1000000506.mm.1	1,28	Pkib	JUC1000001823.mm.1	-2,87	0,038116	0,535709		
TC1200000817.mm.1	5,07	Vsx2	JUC1200003334.mm.1	-2,88	0,008155	0,40551		
TC1200000817.mm.1	5,07	Vsx2	PSR1200005901.mm.1	-5,82	0,006601	0,39213	Alternative 3' Acce	0,42
TC0400002404.mm.1	2,15	Gm12366	PSR0400019510.mm.1	-2,88	0,005819	0,383169		
TC0400002404.mm.1	2,15	Gm12366	PSR0400019509.mm.1	-7,03	0,001969	0,340561	Alternative 3' Acce	0,18
TC1400002129.mm.1	1,51	C030013D06Rik	PSR1400016422.mm.1	-2,88	0,022531	0,487133	Alternative 5' Donc	0,08
TC1400000872.mm.1	-1,19	Gm27017	JUC1400003808.mm.1	-2,88	0,008537	0,407916		
TC0900001442.mm.1	-1,25	Cdc25a	JUC0900006679.mm.1	-2,88	0,0355	0,528724		
TC0600001238.mm.1	-1,01	Thumpd3; Gm22591	JUC0600004857.mm.1	-2,88	0,000403	0,297771		
TC0600002118.mm.1	1,14		JUC0600008762.mm.1	-2,88	0,020801	0,479493		
TC0X00001071.mm.1	1,59	Pgk1	JUC0X00003501.mm.1	-2,88	0,000358	0,290838		
TC0400003942.mm.1	1,02	Pramef12	JUC0400017002.mm.1	-2,88	0,027546	0,504425		
TC0300000605.mm.1	5,01	Serpini1	JUC0300002088.mm.1	-2,89	0,024367	0,49362		
TC0300000605.mm.1	5,01	Serpini1	JUC0300002087.mm.1	-4,49	0,004142	0,3623		
TC0300000605.mm.1	5,01	Serpini1	PSR0300003999.mm.1	-4,53	0,007304	0,398847	Cassette Exon	0,41
TC0300000605.mm.1	5,01	Serpini1	PSR0300004002.mm.1	-6,17	0,003492	0,355638	Intron Retention	0,54
TC0300000605.mm.1	5,01	Serpini1	PSR0300004000.mm.1	-7,1	0,001503	0,329896	Cassette Exon	0,07
TC0900002355.mm.1	3,35	Lingo1	PSR0900019741.mm.1	-2,89	0,007869	0,403545	Cassette Exon	0,23
TC0900002355.mm.1	3,35	Lingo1	PSR0900019735.mm.1	-3,54	0,044425	0,551566	Cassette Exon	0,17
TC0900002355.mm.1	3,35	Lingo1	PSR0900019737.mm.1	-3,7	0,019004	0,471669	Cassette Exon	0,3
TC0900002355.mm.1	3,35	Lingo1	PSR0900019739.mm.1	-4,16	0,002371	0,34829	Cassette Exon	0,28
TC0900002355.mm.1	3,35	Lingo1	PSR0900019740.mm.1	-4,46	0,001454	0,328414	Cassette Exon	0,28
TC0900002355.mm.1	3,35	Lingo1	PSR0900019731.mm.1	-4,55	0,012121	0,432781	Alternative 3' Acce	0,48
TC0900002355.mm.1	3,35	Lingo1	JUC0900011141.mm.1	-5,16	0,007564	0,401384		
TC0900002355.mm.1	3,35	Lingo1	JUC0900011137.mm.1	-5,76	0,020524	0,478276		
TC0900001594.mm.1	1,58	Slc25a38	PSR0900013329.mm.1	-2,89	0,013251	0,441416	Alternative 5' Donc	0,3
TC1400002031.mm.1	1,83	Rab2b	JUC1400008367.mm.1	-2,89	0,002816	0,352207		
TC0100003058.mm.1	-1,24	Srgap2	JUC0100013952.mm.1	-2,89	0,003402	0,354888		
TC0X00000888.mm.1	1,54	Pcyt1b	JUC0X00002809.mm.1	-2,89	0,000656	0,304044		
TC0X00001977.mm.1	1,01	E330010L02Rik	JUC0X00006355.mm.1	-2,89	0,03406	0,524583		
TC1400002280.mm.1	4,03	Dpysl2	PSR1400017606.mm.1	-2,9	0,023575	0,490947	Alternative 5' Donc	0,27
TC1400002280.mm.1	4,03	Dpysl2	PSR1400017590.mm.1	-4,5	0,005797	0,383104	Alternative 3' Acce	0,39
TC0400001710.mm.1	2,34	Usp48	PSR0400013827.mm.1	-2,9	0,031159	0,515677	Cassette Exon	0,18
TC0400001710.mm.1	2,34	Usp48	PSR0400013776.mm.1	-3	0,048335	0,560493	Alternative 5' Donc	0,26
TC0400001710.mm.1	2,34	Usp48	PSR0400013818.mm.1	-3,42	0,002526	0,349501	Alternative 3' Acce	0,31
TC1100001111.mm.1	-2,53	Slc6a4	PSR1100010117.mm.1	-2,9	0,000594	0,304044	Alternative 5' Donc	0,3
TC0900000716.mm.1	1,9	Gm17231	PSR0900005262.mm.1	-2,9	0,030544	0,51337	Cassette Exon	0,18
TC0900000716.mm.1	1,9	Gm17231	PSR0900005263.mm.1	-4,19	0,035231	0,52837	Cassette Exon	0,28
TC1600002084.mm.1	1,51	Cryzl1	JUC1600008298.mm.1	-2,9	0,001387	0,325997		
TC1600002084.mm.1	1,51	Cryzl1	JUC1600008299.mm.1	-3,06	0,002607	0,349501		
TC0800001941.mm.1	-1,04		JUC0800008456.mm.1	-2,9	0,006508	0,391259		
TC0300002554.mm.1	-1,15	Hsd3b5	JUC0300010389.mm.1	-2,9	0,006206	0,387873		
TC0200003760.mm.1	2,92		PSR0200032173.mm.1	-2,91	0,036283	0,530445	Cassette Exon	0,33
TC0200003760.mm.1	2,92		PSR0200032174.mm.1	-3,16	0,032039	0,518459	Cassette Exon	0,22
TC0200003760.mm.1	2,92		JUC0200016428.mm.1	-3,89	0,001073	0,316484		
TC0200000242.mm.1	1,04	Stam	PSR0200001013.mm.1	-2,91	0,017503	0,463964	Alternative 5' Donc	0,32
TC0700004292.mm.1	1,68	Slx1b; Gm6939	PSR0700035871.mm.1	-2,91	0,041554	0,544941	Cassette Exon	0,19
TC0300001452.mm.1	1,29	9530052C20Rik	PSR0300011730.mm.1	-2,91	0,033222	0,522175	Cassette Exon	0,18

TC0700003191.mm.1	10,72		PSR0700028387.mm.1	-2,91	0,038836	0,537731	Cassette Exon	0,07
TC0700003191.mm.1	10,72		JUC0700015094.mm.1	-3,04	0,007051	0,396358		
TC0700003191.mm.1	10,72		JUC0700015092.mm.1	-21,13	0,00444	0,367041		
TC0100000652.mm.1	1,62	Fam134a	JUC0100003131.mm.1	-2,91	0,005614	0,381163		
TC0100000652.mm.1	1,62	Fam134a	JUC0100003132.mm.1	-3,24	0,043087	0,548459		
TC0700002913.mm.1	-1,03	Vmn2r-ps56	JUC0700013840.mm.1	-2,91	0,008986	0,411965		
TC1000003230.mm.1	1,06	Vmn2r85	JUC1000013187.mm.1	-2,91	0,038342	0,53622		
TC1100002486.mm.1	-1	Npm1	JUC1100012025.mm.1	-2,91	0,020331	0,477255		
TC0200005421.mm.1	5,32	Bhlhe23; Gm14343	PSR0200046167.mm.1	-2,92	0,003368	0,354278	Alternative 3' Acce	0,29
TC0200005421.mm.1	5,32	Bhlhe23; Gm14343	PSR0200046172.mm.1	-4,92	0,001291	0,322541	Alternative 5' Donc	0,48
TC0200005421.mm.1	5,32	Bhlhe23; Gm14343	PSR0200046173.mm.1	-5,13	0,004138	0,3623	Alternative 5' Donc	0,48
TC0200005421.mm.1	5,32	Bhlhe23; Gm14343	PSR0200046176.mm.1	-6,46	0,005092	0,375649	Alternative 5' Donc	0,48
TC0200005421.mm.1	5,32	Bhlhe23; Gm14343	PSR0200046166.mm.1	-8,49	0,000186	0,284485	Cassette Exon	0,28
TC0200005421.mm.1	5,32	Bhlhe23; Gm14343	PSR0200046165.mm.1	-8,59	0,001278	0,322251	Cassette Exon	0,28
TC1300000928.mm.1	-1,03	Mctp1	PSR1300006052.mm.1	-2,92	0,019262	0,473074	Alternative 5' Donc	0,28
TC1200001252.mm.1	1,1	Tdrd9	JUC1200004895.mm.1	-2,92	0,005283	0,378059		
TC0300001083.mm.1	-1,04	St7l	JUC0300004543.mm.1	-2,92	0,024337	0,49362		
TC0200004701.mm.1	1,44	Pak7	JUC0200020885.mm.1	-2,92	0,004447	0,367215		
TC1500001862.mm.1	1,03	1700088E04Rik	JUC1500008278.mm.1	-2,92	0,003816	0,35811		
TC0200000223.mm.1	1,12	Ankrd16	JUC0200000369.mm.1	-2,92	0,031857	0,517902		
TC1100001909.mm.1	-1,34	Myo15b	JUC1100009408.mm.1	-2,92	0,020736	0,479172		
TC0300001987.mm.1	1,3	Exosc8	PSR0300015143.mm.1	-2,93	0,014294	0,448073	Cassette Exon	0,18
TC0200004713.mm.1	1,33	Jag1	JUC0200020925.mm.1	-2,93	0,012798	0,437255		
TC1400002631.mm.1	-1,07		JUC1400010785.mm.1	-2,93	0,007133	0,397309		
TC0700000860.mm.1	1,37	Car11	JUC0700003738.mm.1	-2,93	0,007852	0,403345		
TC0300003103.mm.1	-1,05	Dnase2b	JUC0300012803.mm.1	-2,93	0,01466	0,450098		
TC0400000994.mm.1	1,09	Gm12705	JUC0400003430.mm.1	-2,93	0,018579	0,469618		
TC0300001512.mm.1	1,64	Gm1653	JUC0300006448.mm.1	-2,93	0,004694	0,370496		
TC1800000342.mm.1	1,05	Tmco6	JUC1800001508.mm.1	-2,94	0,034563	0,525975		
TC0500001849.mm.1	-1,03	Tex26	JUC0500009207.mm.1	-2,94	0,034845	0,526823		
TC0500000560.mm.1	-1,07	Pcdh7	JUC0500002848.mm.1	-2,94	0,0428	0,548097		
TC0900001398.mm.1	1,66	Nicn1	JUC0900006152.mm.1	-2,94	0,021376	0,481977		
TC0500002962.mm.1	1,06	Vmn2r11	JUC0500014301.mm.1	-2,94	0,006319	0,38927		
TC1100000029.mm.1	1,04	Gal3st1	JUC1100000164.mm.1	-2,94	0,023219	0,490032		
TC1000001324.mm.1	-1,07		JUC1000005338.mm.1	-2,94	0,043082	0,548459		
TC1200002364.mm.1	1,27	Begain	JUC1200009061.mm.1	-2,95	0,00682	0,39387		
TC1200002364.mm.1	1,27	Begain	JUC1200009065.mm.1	-3,17	0,012725	0,436583		
TC0100001381.mm.1	6,01	Rgs16	PSR0100011431.mm.1	-2,96	0,021778	0,48438	Cassette Exon	0,18
TC0200003275.mm.1	-1,03	Gm14488	JUC0200013996.mm.1	-2,96	0,003499	0,355638		
TC1900001105.mm.1	1,37	Tmem216	JUC1900005642.mm.1	-2,96	0,001835	0,337414		
TC0600003301.mm.1	-1,18	4933406J09Rik	JUC0600013500.mm.1	-2,96	0,037147	0,533065		
TC0400001147.mm.1	1,15	Zcchc11	PSR0400008126.mm.1	-2,97	0,02254	0,487201	Intron Retention	0,46
TC1500001320.mm.1	3,83	Ankrd33b	PSR1500010242.mm.1	-2,97	0,028209	0,506368	Cassette Exon	0,33
TC1500001320.mm.1	3,83	Ankrd33b	PSR1500010241.mm.1	-3,28	0,009501	0,416578	Cassette Exon	0,33
TC1500001320.mm.1	3,83	Ankrd33b	JUC1500005782.mm.1	-3,67	0,027349	0,503766		
TC1500001320.mm.1	3,83	Ankrd33b	PSR1500010227.mm.1	-3,76	0,001682	0,335182	Alternative 3' Acce	0,44
TC1500001320.mm.1	3,83	Ankrd33b	JUC1500005778.mm.1	-4,55	0,000448	0,297771		
TC1500001320.mm.1	3,83	Ankrd33b	PSR1500010243.mm.1	-5,15	0,001742	0,335996	Cassette Exon	0,33

TC1500001320.mm.1	3,83	Ankrd33b	JUC1500005781.mm.1	-5,51	0,037796	0,534693		
TC1500001320.mm.1	3,83	Ankrd33b	PSR1500010244.mm.1	-6,6	0,001335	0,325349	Cassette Exon	0,33
TC1500000503.mm.1	3,21	Khdrbs3	PSR1500003555.mm.1	-2,97	0,02579	0,498518	Cassette Exon	0,28
TC1500000503.mm.1	3,21	Khdrbs3	JUC1500002094.mm.1	-3,1	0,020543	0,478319		
TC1500000503.mm.1	3,21	Khdrbs3	PSR1500003541.mm.1	-3,21	0,00347	0,355628	Cassette Exon	0,24
TC1500000503.mm.1	3,21	Khdrbs3	PSR1500003540.mm.1	-4,35	0,003193	0,354243	Cassette Exon	0,24
TC1500000503.mm.1	3,21	Khdrbs3	JUC1500002093.mm.1	-5,15	0,000169	0,273067		
TC0100003140.mm.1	1,84	Gm15850	JUC0100014499.mm.1	-2,97	0,011272	0,426793		
TC0100003140.mm.1	1,84	Gm15850	PSR0100025515.mm.1	-3,06	0,043832	0,550456	Alternative 5' Donc	0,23
TC0100003140.mm.1	1,84	Gm15850	JUC0100014500.mm.1	-3,18	0,004357	0,365487		
TC0100003140.mm.1	1,84	Gm15850	JUC0100014498.mm.1	-3,52	0,042007	0,546101		
TC0500002641.mm.1	1,32	Aasdh	PSR0500023332.mm.1	-2,97	0,00998	0,418456	Cassette Exon	0,18
TC1300000980.mm.1	1,08	2810049E08Rik	JUC1300003333.mm.1	-2,97	0,036663	0,531675		
TC0800000539.mm.1	-1,02	Cenpu; Mlf1ip	JUC0800002064.mm.1	-2,97	0,034211	0,525106		
TC0600001536.mm.1	3,15	Gabarapl1	JUC0600006596.mm.1	-2,97	0,04318	0,548858		
TC0400000428.mm.1	-1,43		JUC0400001314.mm.1	-2,97	0,011242	0,426718		
TC1100000717.mm.1	1,19	Mrpl55	JUC1100002940.mm.1	-2,97	0,005365	0,378627		
TC0X00001784.mm.1	1,57	Pqbp1	PSR0X00011310.mm.1	-2,97	0,030745	0,514065		
TC0X00000060.mm.1	11,81	Syp	JUC0X00000144.mm.1	-2,98	0,000255	0,28803		
TC0X00000060.mm.1	11,81	Syp	JUC0X00000149.mm.1	-3,11	0,002172	0,344453		
TC0X00000060.mm.1	11,81	Syp	JUC0X00000147.mm.1	-3,14	0,041228	0,544134		
TC0X00000060.mm.1	11,81	Syp	PSR0X00000299.mm.1	-5,21	0,00078	0,309885	Alternative 3' Acce	0,5
TC0X00000060.mm.1	11,81	Syp	PSR0X00000289.mm.1	-9,85	0,001831	0,33731	Alternative 3' Acce	0,16
TC0X00000060.mm.1	11,81	Syp	PSR0X00000306.mm.1	-11,9	0,000187	0,284485	Alternative 5' Donc	0,46
TC0X00000060.mm.1	11,81	Syp	PSR0X00000294.mm.1	-13,38	0,001845	0,337414	Alternative 5' Donc	0,16
TC0X00000060.mm.1	11,81	Syp	PSR0X00000307.mm.1	-13,55	0,000199	0,28803	Alternative 5' Donc	0,46
TC0X00000060.mm.1	11,81	Syp	JUC0X00000150.mm.1	-14,08	0,001581	0,331726		
TC0X00000060.mm.1	11,81	Syp	JUC0X00000148.mm.1	-16,96	0,000247	0,28803		
TC0X00000060.mm.1	11,81	Syp	PSR0X00000295.mm.1	-19,23	0,000023	0,22008	Cassette Exon	0,21
TC0X00000060.mm.1	11,81	Syp	PSR0X00000291.mm.1	-28,5	0,001229	0,322251	Alternative 3' Acce	0,16
TC0X00000060.mm.1	11,81	Syp	PSR0X00000296.mm.1	-32,34	0,00048	0,298999	Alternative 3' Acce	0,29
TC0X00000060.mm.1	11,81	Syp	JUC0X00000151.mm.1	-41,03	0,000193	0,28803		
TC0600003457.mm.1	-1,11	Ergic2	PSR0600027284.mm.1	-2,98	0,043506	0,549909	Alternative 5' Donc	0,32
TC1400001494.mm.1	1,44		PSR1400011169.mm.1	-2,98	0,028091	0,50608	Alternative 5' Donc	0,32
TC0200000955.mm.1	2,1	Csrnp3	PSR0200007599.mm.1	-2,98	0,010388	0,421341	Cassette Exon	0,29
TC1700000424.mm.1	1,09	Kifc5b	JUC1700001963.mm.1	-2,98	0,00604	0,386586		
TC1000002164.mm.1	-1,18	Cep85l; 1700003O08Rik	JUC1000008248.mm.1	-2,98	0,016533	0,459611		
TC1400000965.mm.1	33,59	Nefl	PSR1400007745.mm.1	-2,99	0,0081	0,405071	Alternative 5' Donc	0,32
TC1500001863.mm.1	-1,15	Sox10	PSR1500014537.mm.1	-2,99	0,043823	0,550456	Alternative 5' Donc	0,23
TC1400000587.mm.1	1,01	Vmn2r88	JUC1400002656.mm.1	-2,99	0,009555	0,416829		
TC0100001582.mm.1	1,09	B4galt3	JUC0100007376.mm.1	-2,99	0,02141	0,482126		
TC1500000445.mm.1	1,32	4930402D18Rik	JUC1500001816.mm.1	-2,99	0,005526	0,380518		
TC1500000445.mm.1	1,32	4930402D18Rik	JUC1500001826.mm.1	-3,55	0,000908	0,313363		
TC0800001917.mm.1	1,16	Ddhd2	JUC0800008354.mm.1	-2,99	0,035662	0,528966		
TC0800002550.mm.1	1,89	Elmod2	PSR0800019357.mm.1	-2,99	0,002603	0,349501		
TC0800002550.mm.1	1,89	Elmod2	JUC0800010542.mm.1	-3,63	0,043359	0,549429		
TC1100004290.mm.1	2,14	Commd1	PSR1100022243.mm.1	-3	0,034026	0,524517	Alternative 5' Donc	0,24
TC1100004290.mm.1	2,14	Commd1	JUC1100011570.mm.1	-3,36	0,042206	0,546983		

TC1700000993.mm.1	1,4	Ccdc94	JUC1700005026.mm.1	-3	0,045039	0,553049		
TC1000002156.mm.1	1,15	Gopc	JUC1000008222.mm.1	-3	0,009658	0,417484		
TC0900002805.mm.1	1,31	Gm2382	JUC0900013038.mm.1	-3	0,02282	0,488291		
TC1500002317.mm.1	3,2	Zfp385a	PSR1500019211.mm.1	-3,01	0,039703	0,540417	Cassette Exon	0,28
TC0300000866.mm.1	1,54	Tdrkh	JUC0300003456.mm.1	-3,01	0,005516	0,380428		
TC0300000866.mm.1	1,54	Tdrkh	JUC0300003458.mm.1	-3,25	0,031833	0,517847		
TC0900001726.mm.1	1,47	AK129341	JUC0900008222.mm.1	-3,01	0,02436	0,49362		
TC0600000324.mm.1	-1,43	Akr1b8	JUC0600001198.mm.1	-3,01	0,009857	0,417907		
TC0500002927.mm.1	2,75	Glmn	PSR0500025887.mm.1	-3,02	0,01723	0,462633		
TC0500002927.mm.1	2,75	Glmn	PSR0500025888.mm.1	-3,11	0,005338	0,378059	Cassette Exon	0,19
TC0500002927.mm.1	2,75	Glmn	PSR0500025855.mm.1	-3,19	0,01814	0,467376	Alternative 3' Acce	0,26
TC0500002927.mm.1	2,75	Glmn	PSR0500025866.mm.1	-3,7	0,003329	0,354243	Cassette Exon	0,24
TC0500002927.mm.1	2,75	Glmn	PSR0500025881.mm.1	-4,22	0,009589	0,417183		
TC1200000152.mm.1	2,44	Gm4983	JUC1200000754.mm.1	-3,02	0,010114	0,419058		
TC0500001390.mm.1	1,56	Gpn3	JUC0500006748.mm.1	-3,02	0,02372	0,491521		
TC0500000027.mm.1	-1,02	Gm15750	JUC0500000167.mm.1	-3,02	0,002406	0,348653		
TC0200001911.mm.1	1,65	Stard7	PSR0200014984.mm.1	-3,03	0,000474	0,298999	Alternative 5' Donc	0,32
TC0200003273.mm.1	-1,04		PSR0200027715.mm.1	-3,03	0,00122	0,322251	Cassette Exon	0,19
TC0200003335.mm.1	-1,34		JUC0200014408.mm.1	-3,03	0,003869	0,358862		
TC1400000790.mm.1	-1,02	Gmpr2	JUC1400003408.mm.1	-3,03	0,023635	0,491089		
TC1400002102.mm.1	-1,17	Adcy4	JUC1400008825.mm.1	-3,03	0,00382	0,358311		
TC1500002252.mm.1	-1,08		JUC1500010543.mm.1	-3,03	0,032009	0,518367		
TC0700000706.mm.1	1,25		PSR0700005809.mm.1	-3,04	0,02156	0,483034	Alternative 3' Acce	0,31
TC1700001869.mm.1	1,77	Zfp81	PSR1700017278.mm.1	-3,04	0,029578	0,510459	Alternative 5' Donc	0,29
TC0800003171.mm.1	1,37	Agt	PSR0800024358.mm.1	-3,04	0,019834	0,475536	Cassette Exon	0,18
TC1400001061.mm.1	-1,11	Tpt1	JUC1400004579.mm.1	-3,04	0,002975	0,352501		
TC1200002434.mm.1	1,12	Akt1	JUC1200009314.mm.1	-3,04	0,020498	0,478123		
TC1800000623.mm.1	-1,25	Pdgfrb	JUC1800002494.mm.1	-3,04	0,03722	0,533311		
TC1700001805.mm.1	1,59	Glo1	JUC1700008945.mm.1	-3,04	0,011156	0,426295		
TC0500002239.mm.1	-1,15	Mfsd10	JUC0500011158.mm.1	-3,04	0,008054	0,404416		
TC1600000171.mm.1	6,17	2900011O08Rik	PSR1600001237.mm.1	-3,05	0,00015	0,272178	Cassette Exon	0,2
TC1600000171.mm.1	6,17	2900011O08Rik	JUC1600000630.mm.1	-3,74	0,005999	0,386404		
TC1600000171.mm.1	6,17	2900011O08Rik	JUC1600000631.mm.1	-3,91	0,032332	0,519155		
TC1600000171.mm.1	6,17	2900011O08Rik	PSR1600001245.mm.1	-5,93	0,000199	0,28803	Alternative 5' Donc	0,45
TC1600000171.mm.1	6,17	2900011O08Rik	JUC1600000632.mm.1	-6,35	0,007304	0,398847		
TC0900001109.mm.1	1,48	Gm11114	JUC0900004730.mm.1	-3,05	0,037578	0,534053		
TC0X00003038.mm.1	1,05	Morf4l2	JUC0X00009629.mm.1	-3,05	0,028731	0,508141		
TC0X00003038.mm.1	1,05	Morf4l2	JUC0X00009630.mm.1	-5,12	0,008971	0,411788		
TC0800001522.mm.1	2,93	Tubb3	PSR0800012490.mm.1	-3,06	0,00371	0,357512	Alternative 3' Acce	0,32
TC0800001522.mm.1	2,93	Tubb3	JUC0800006810.mm.1	-3,29	0,015785	0,455613		
TC0200004940.mm.1	1,62	Snta1	JUC0200021629.mm.1	-3,06	0,004497	0,36828		
TC1200002420.mm.1	1,01		JUC1200009269.mm.1	-3,06	0,004102	0,36228		
TC0100001382.mm.1	-1,64	Rnasel	JUC0100006605.mm.1	-3,06	0,038002	0,535441		
TC0800002364.mm.1	1,62	Gm7697	JUC0800009636.mm.1	-3,06	0,003787	0,357586		
TC0600000215.mm.1	1,03	Snd1	JUC0600000708.mm.1	-3,06	0,027458	0,504045		
TC1000000829.mm.1	-1,06	Arid3a	JUC1000003284.mm.1	-3,06	0,043129	0,548664		
TC0X00003435.mm.1	-1,42	Slc10a3	JUC0X00007963.mm.1	-3,06	0,02836	0,506881		
TC1200001925.mm.1	-1,41		JUC1200007348.mm.1	-3,07	0,017848	0,466223		

TC1800000902.mm.1	-1,02		JUC1800003606.mm.1	-3,07	0,025977	0,499207		
TC0500002496.mm.1	-1,21		JUC0500012128.mm.1	-3,07	0,010668	0,423012		
TC0700000298.mm.1	1,21	Ercc1	JUC0700001121.mm.1	-3,07	0,011227	0,426718		
TC1000001369.mm.1	-1		JUC1000005523.mm.1	-3,07	0,033451	0,522793		
TC1100003478.mm.1	1,43	Rad51c	PSR1100031917.mm.1	-3,08	0,0117	0,429969	Alternative 5' Donc	0,25
TC1200001460.mm.1	1,21	Lpin1	JUC1200005633.mm.1	-3,08	0,006179	0,387873		
TC0200003705.mm.1	1,03	Mettl5; Gm13625	JUC0200016206.mm.1	-3,08	0,028357	0,506875		
TC0600002374.mm.1	-1,1	Vmn1r31	JUC0600009593.mm.1	-3,08	0,014718	0,45037		
TC1000000940.mm.1	1,35	D10Wsu102e	JUC1000004030.mm.1	-3,08	0,017001	0,461553		
TC0200002980.mm.1	7,84	C1ql3	PSR0200024607.mm.1	-3,09	0,030864	0,514425	Cassette Exon	0,37
TC0200002980.mm.1	7,84	C1ql3	PSR0200024608.mm.1	-5,57	0,005889	0,383835	Cassette Exon	0,37
TC0200002980.mm.1	7,84	C1ql3	JUC0200012436.mm.1	-9,62	0,000202	0,28803		
TC0600000584.mm.1	2,81	Ccdc126	JUC0600002266.mm.1	-3,09	0,002869	0,352207		
TC0600000584.mm.1	2,81	Ccdc126	PSR0600004477.mm.1	-3,3	0,006897	0,394673	Alternative 3' Acce	0,32
TC0200001025.mm.1	1,93	Gorasp2	JUC0200004229.mm.1	-3,09	0,016377	0,458693		
TC0200001025.mm.1	1,93	Gorasp2	PSR0200008230.mm.1	-3,33	0,026827	0,502373	Alternative 5' Donc	0,23
TC1300000660.mm.1	1,05	Grk6	JUC1300002278.mm.1	-3,09	0,007198	0,39798		
TC1700001202.mm.1	-1,06		JUC1700006192.mm.1	-3,09	0,029883	0,51129		
TC0600003370.mm.1	-1,16	Slco1a5	JUC0600013866.mm.1	-3,09	0,044394	0,551441		
TC0500003112.mm.1	-1,22	Ccdc60	JUC0500015091.mm.1	-3,09	0,005363	0,378627		
TC0300000043.mm.1	3,2	Stmn2	PSR0300000150.mm.1	-3,1	0,003619	0,356223	Alternative 5' Donc	0,26
TC0300000043.mm.1	3,2	Stmn2	PSR0300000146.mm.1	-3,93	0,002509	0,349237	Alternative 3' Acce	0,45
TC0200001395.mm.1	3,45		PSR0200010929.mm.1	-3,1	0,01642	0,458966	Alternative 3' Acce	0,34
TC1100001474.mm.1	2,24	Gngt2	PSR1100013687.mm.1	-3,11	0,02322	0,490032	Cassette Exon	0,33
TC1100001474.mm.1	2,24	Gngt2	JUC1100007164.mm.1	-6,31	0,049264	0,562639		
TC0200004225.mm.1	4,72	A930006I01Rik	PSR0200035474.mm.1	-3,11	0,010393	0,421409	Cassette Exon	0,19
TC0100000116.mm.1	1,41	Tfap2b	JUC0100000454.mm.1	-3,11	0,018686	0,470362		
TC1100000134.mm.1	-1,06	Ikzf1	JUC1100000730.mm.1	-3,11	0,017504	0,463964		
TC1000001840.mm.1	-1,12	Myb	JUC1000007285.mm.1	-3,11	0,017867	0,466232		
TC0300000489.mm.1	1,4		PSR0300003386.mm.1	-3,12	0,002476	0,349135	Cassette Exon	0,2
TC0800002362.mm.1	1,47	Gm9495	JUC0800009625.mm.1	-3,12	0,003609	0,356223		
TC0700000859.mm.1	1	Ntn5	JUC0700003732.mm.1	-3,12	0,000149	0,272178		
TC0700000821.mm.1	1,42	Fuz	PSR0700006939.mm.1	-3,13	0,047845	0,559363	Alternative 5' Donc	0,33
TC1700002454.mm.1	1,13	Arhgap28	JUC1700012150.mm.1	-3,13	0,000966	0,313363		
TC0500001974.mm.1	1,02	Gm10354	JUC0500009804.mm.1	-3,13	0,014302	0,448073		
TC0500003608.mm.1	1,03	Rnf6	JUC0500018029.mm.1	-3,13	0,028696	0,508019		
TC1100001658.mm.1	1,24	Rnd2	JUC1100008063.mm.1	-3,13	0,012401	0,434961		
TC1000000369.mm.1	1,03	5730435O14Rik	JUC1000001411.mm.1	-3,13	0,011921	0,431655		
TC1100002611.mm.1	1,1	Timd2	PSR1100024044.mm.1	-3,14	0,001374	0,32572	Cassette Exon	0,19
TC1300001878.mm.1	1,44	Gfod1	PSR1300011701.mm.1	-3,14	0,014576	0,449876	Cassette Exon	0,07
TC1400001746.mm.1	1	2610528A11Rik	JUC1400007441.mm.1	-3,14	0,003222	0,354243		
TC0900000006.mm.1	1,57	Gm10717	PSR0900000056.mm.1	-3,15	0,007925	0,403819	Alternative 5' Donc	0,29
TC1200000715.mm.1	1,96	Arg2	JUC1200002919.mm.1	-3,15	0,002551	0,349501		
TC1200000715.mm.1	1,96	Arg2	PSR1200005101.mm.1	-3,49	0,00306	0,353892	Cassette Exon	0,25
TC1900000431.mm.1	1,45	Gm9895	PSR1900003991.mm.1	-3,15	0,002898	0,352207	Alternative 3' Acce	0,25
TC1500001656.mm.1	1,03	Sla	JUC1500007014.mm.1	-3,15	0,028509	0,507386		
TC0200001552.mm.1	1,14	Cd59a	JUC0200005826.mm.1	-3,15	0,032081	0,518469		
TC1700000307.mm.1	-1,2	Vmn2r-ps130	JUC1700001200.mm.1	-3,16	0,013526	0,44345		

TC0800002108.mm.1	2,15	Mtmr7	PSR0800016218.mm.1	-3,16	0,001189	0,321162		
TC0700000840.mm.1	-1,36	Hrc	JUC0700003572.mm.1	-3,16	0,040006	0,541155		
TC1000001140.mm.1	2,81	Gm15963	PSR1000008641.mm.1	-3,17	0,022267	0,485916	Cassette Exon	0,2
TC1000001140.mm.1	2,81	Gm15963	PSR1000008638.mm.1	-3,5	0,008269	0,405976	Cassette Exon	0,24
TC0300000360.mm.1	1,67	Rab33b	PSR0300002585.mm.1	-3,17	0,008542	0,408005	Alternative 5' Donc	0,18
TC1600000329.mm.1	2,16	Eif4a2; Snord2; Snora81; (	JUC1600001749.mm.1	-3,17	0,001264	0,322251		
TC0700004366.mm.1	1,12	Tial1	JUC0700019322.mm.1	-3,17	0,049813	0,563739		
TC0700004366.mm.1	1,12	Tial1	JUC0700019342.mm.1	-3,57	0,04461	0,552129		
TC0700004366.mm.1	1,12	Tial1	JUC0700019333.mm.1	-4,32	0,000149	0,272178		
TC1000002539.mm.1	1,04	4930404N11Rik	JUC1000010445.mm.1	-3,17	0,005342	0,378188		
TC0300002151.mm.1	-1,19	Sis	PSR0300016182.mm.1	-3,18	0,037781	0,534693	Cassette Exon	0,23
TC0500001510.mm.1	-1,01	Gbas	JUC0500007493.mm.1	-3,18	0,045686	0,554833		
TC0600002505.mm.1	-1,11	Dnah6	JUC0600010163.mm.1	-3,18	0,028011	0,505851		
TC0600002122.mm.1	-1,16	Mkrn1	JUC0600008794.mm.1	-3,18	0,026592	0,501731		
TC0200004364.mm.1	3,22	Scg5	PSR0200036454.mm.1	-3,19	0,005529	0,380518	Cassette Exon	0,24
TC0200004364.mm.1	3,22	Scg5	PSR0200036448.mm.1	-3,63	0,01149	0,42815	Cassette Exon	0,27
TC0200004364.mm.1	3,22	Scg5	PSR0200036456.mm.1	-5,52	0,003936	0,359818	Cassette Exon	0,24
TC0900002406.mm.1	1,06	Arih1	PSR0900020273.mm.1	-3,19	0,017959	0,466709	Cassette Exon	0,23
TC0700004085.mm.1	1,65	Dennd5a	PSR0700033764.mm.1	-3,19	0,018865	0,471054	Alternative 5' Donc	0,2
TC1500002194.mm.1	1,39	Rhebl1	JUC1500010230.mm.1	-3,19	0,02909	0,509063		
TC1700000306.mm.1	-1,33	Vmn2r-ps129	JUC1700001195.mm.1	-3,19	0,014038	0,44654		
TC0700000802.mm.1	1,24	Syt3	JUC0700003280.mm.1	-3,19	0,005175	0,376851		
TC0900002522.mm.1	1	Snx1	JUC0900011920.mm.1	-3,19	0,012522	0,43545		
TC0200005223.mm.1	-1,23	Sall4	JUC0200023074.mm.1	-3,2	0,004093	0,36228		
TC0100003025.mm.1	-1,28	Lct	JUC0100013725.mm.1	-3,2	0,004888	0,372195		
TC1900001402.mm.1	-1,02		JUC1900006683.mm.1	-3,2	0,035985	0,529491		
TC0900001411.mm.1	1,02	Qrich1	JUC0900006301.mm.1	-3,2	0,010875	0,424174		
TC0600002464.mm.1	-1,02		JUC0600009896.mm.1	-3,2	0,009869	0,417961		
TC0700003050.mm.1	1,1	Ptpn5	JUC0700014936.mm.1	-3,2	0,044859	0,552605		
TC0X00000209.mm.1	-1,01	Rgn	JUC0X00000607.mm.1	-3,2	0,030079	0,511908		
TC0600001713.mm.1	3,72	Gm15706	PSR0600013636.mm.1	-3,21	0,035944	0,529399	Alternative 3' Acce	0,32
TC0600003386.mm.1	2,76		JUC0600013983.mm.1	-3,21	0,02598	0,499207		
TC0600003386.mm.1	2,76		JUC0600013982.mm.1	-3,77	0,0197	0,474887		
TC0X00000708.mm.1	1,14	Atp6ap1	JUC0X00002408.mm.1	-3,21	0,024166	0,493245		
TC1000000512.mm.1	1,1	Gcc2	JUC1000001855.mm.1	-3,21	0,018333	0,468192		
TC0300001701.mm.1	2,69	Hps3	PSR0300013383.mm.1	-3,22	0,02503	0,496091	Alternative 3' Acce	0,3
TC1100000891.mm.1	1,44	Ctc1	PSR1100007433.mm.1	-3,22	0,007431	0,399925	Alternative 5' Donc	0,25
TC0200004410.mm.1	2,5	Gpr176	PSR0200036785.mm.1	-3,22	0,030904	0,514574	Cassette Exon	0,13
TC1600001113.mm.1	1,23	Gm15879	JUC1600004597.mm.1	-3,22	0,006821	0,39387		
TC1000000704.mm.1	3,65	Gnaz	PSR1000004941.mm.1	-3,23	0,003952	0,359922	Alternative 3' Acce	0,43
TC1000000704.mm.1	3,65	Gnaz	JUC1000002698.mm.1	-4,03	0,008643	0,408463		
TC1000000704.mm.1	3,65	Gnaz	PSR1000004940.mm.1	-8,64	0,007254	0,39826	Alternative 3' Acce	0,43
TC1000000192.mm.1	-1,38	Vnn3	PSR1000001417.mm.1	-3,23	0,014983	0,452048	Alternative 3' Acce	0,34
TC1700002195.mm.1	1,48		JUC1700011045.mm.1	-3,23	0,046699	0,556856		
TC0500002893.mm.1	-1,02	Abcg3	JUC0500013887.mm.1	-3,23	0,041696	0,545356		
TC0400004176.mm.1	1,79	Palm2	JUC0400002220.mm.1	-3,23	0,04525	0,553697		
TC0700001765.mm.1	10,36	Cacng3	JUC0700007861.mm.1	-3,24	0,007427	0,399925		
TC0700001765.mm.1	10,36	Cacng3	PSR0700015025.mm.1	-3,86	0,003348	0,354243	Cassette Exon	0,16

TC0700001765.mm.1	10,36	Cacng3	PSR0700015020.mm.1	-9,65	0,001589	0,331726	Alternative 3' Acce	0,36
TC0700001765.mm.1	10,36	Cacng3	JUC0700007864.mm.1	-28,82	0,001592	0,331726		
TC0700001765.mm.1	10,36	Cacng3	JUC0700007863.mm.1	-46,1	0,002915	0,352289		
TC1500000524.mm.1	3,13	Ptp4a3	PSR1500003658.mm.1	-3,24	0,007193	0,397947	Cassette Exon	0,35
TC1500000524.mm.1	3,13	Ptp4a3	JUC1500002161.mm.1	-3,41	0,01649	0,459391		
TC1500000524.mm.1	3,13	Ptp4a3	PSR1500003659.mm.1	-3,74	0,01239	0,434939	Cassette Exon	0,35
TC1500000524.mm.1	3,13	Ptp4a3	JUC1500002160.mm.1	-4,21	0,008432	0,406611		
TC1600001582.mm.1	1,66	Slc15a2	PSR1600012939.mm.1	-3,24	0,021161	0,480949	Alternative 5' Donc	0,27
TC1600001582.mm.1	1,66	Slc15a2	JUC1600006722.mm.1	-5,1	0,032419	0,519333		
TC1600000501.mm.1	-1,11	Kpna1	JUC1600002536.mm.1	-3,24	0,026428	0,501198		
TC0500002940.mm.1	1,15	Tmed5	JUC0500014172.mm.1	-3,24	0,002837	0,352207		
TC0500002940.mm.1	1,15	Tmed5	JUC0500014168.mm.1	-3,59	0,005162	0,376735		
TC0800002508.mm.1	1,54	Pou4f2	PSR0800019073.mm.1	-3,25	0,002124	0,342919	Cassette Exon	0,32
TC0800002508.mm.1	1,54	Pou4f2	JUC0800010390.mm.1	-3,45	0,01856	0,469537		
TC0300000209.mm.1	-1		JUC0300000577.mm.1	-3,25	0,015449	0,454271		
TC1500001832.mm.1	-1,1	Tmprss6	JUC1500008177.mm.1	-3,25	0,021738	0,484249		
TC1700000229.mm.1	-1,2	Vmn2r98	JUC1700000984.mm.1	-3,25	0,043977	0,550764		
TC0700001434.mm.1	1,38		JUC0700006402.mm.1	-3,25	0,044969	0,552889		
TC1000002172.mm.1	1,01	Fam184a	JUC1000008286.mm.1	-3,25	0,000856	0,311909		
TC1400000877.mm.1	-1,17	Rnaseh2b	JUC1400003816.mm.1	-3,26	0,035261	0,52837		
TC0200004126.mm.1	1,37	Atg13	JUC0200017669.mm.1	-3,26	0,018644	0,470078		
TC0100003068.mm.1	1,15	Mfsd4	JUC0100013976.mm.1	-3,26	0,006745	0,393343		
TC0900000668.mm.1	1,43	Chrna5	JUC0900002514.mm.1	-3,26	0,037152	0,533065		
TC0700004194.mm.1	1,59	Crym	JUC0700018442.mm.1	-3,26	0,004167	0,3623		
TC1100000864.mm.1	-1,54	Dhrs7c	JUC1100003790.mm.1	-3,26	0,008496	0,407077		
TC1000002269.mm.1	1,32	Tet1	JUC1000008924.mm.1	-3,28	0,014748	0,450662		
TC1000002269.mm.1	1,32	Tet1	PSR1000016291.mm.1	-3,61	0,02649	0,501252	Alternative 5' Donc	0,41
TC0100001398.mm.1	1,06		JUC0100006642.mm.1	-3,28	0,048545	0,561008		
TC0500003404.mm.1	-1,35	Tmem120a	JUC0500016743.mm.1	-3,28	0,049434	0,563114		
TC0500003605.mm.1	-1,09	1700001J03Rik	JUC0500018015.mm.1	-3,28	0,03866	0,537102		
TC0X00001674.mm.1	-1,09	Fancb	JUC0X00005455.mm.1	-3,28	0,029153	0,509333		
TC0700004651.mm.1	13,46		JUC0700015152.mm.1	-3,29	0,02929	0,509874		
TC0700004651.mm.1	13,46		PSR0700039182.mm.1	-5,81	0,001647	0,333988	Cassette Exon	0,23
TC0700004651.mm.1	13,46		JUC0700015153.mm.1	-7,03	0,001019	0,316361		
TC0700002162.mm.1	2,21	Syt5	PSR0700019725.mm.1	-3,29	0,004475	0,367858	Cassette Exon	0,21
TC1700000492.mm.1	1,07	Kctd20	JUC1700002344.mm.1	-3,29	0,029818	0,511132		
TC0200003063.mm.1	-1,18	4921504E06Rik	JUC0200012690.mm.1	-3,3	0,035668	0,528966		
TC1000002783.mm.1	-1,44	Lrriq1	JUC1000011656.mm.1	-3,3	0,023128	0,489756		
TC1100003043.mm.1	-1,04	Kdm6b	JUC1100014516.mm.1	-3,3	0,026194	0,50003		
TC0500002856.mm.1	1,07	Nkx6-1	PSR0500025193.mm.1	-3,31	0,012891	0,438165	Alternative 3' Acce	0,37
TC1300001812.mm.1	2,49	Nrn1	PSR1300011307.mm.1	-3,31	0,01093	0,424831	Cassette Exon	0,34
TC1300001812.mm.1	2,49	Nrn1	JUC1300005785.mm.1	-3,75	0,004512	0,368648		
TC1500001362.mm.1	1,17		JUC1500005896.mm.1	-3,31	0,01549	0,454456		
TC0800000806.mm.1	1,07	Zfp961	JUC0800003148.mm.1	-3,31	0,039938	0,540973		
TC0700003360.mm.1	-1,08		JUC0700015204.mm.1	-3,31	0,043547	0,550012		
TC0300001989.mm.1	1,36	Rfxap	JUC0300007941.mm.1	-3,31	0,015986	0,456946		
TC0200000342.mm.1	2,92	Gpr158	PSR0200001612.mm.1	-3,32	0,005309	0,378059	Alternative 5' Donc	0,39
TC0200002032.mm.1	1,47	Cds2	JUC0200008134.mm.1	-3,32	0,004519	0,368648		

TC0900000339.mm.1	1,51	Pus3	JUC0900001365.mm.1	-3,32	0,003434	0,35532		
TC0600000656.mm.1	1,61	Mturn; 2410066E13Rik	JUC0600002524.mm.1	-3,32	0,000396	0,29748		
TC0300002702.mm.1	1,15	Prok1	JUC0300010957.mm.1	-3,32	0,049637	0,563494		
TC0X00000652.mm.1	1,13	Hmgb3	JUC0X00002060.mm.1	-3,32	0,00901	0,412369		
TC1200001513.mm.1	1,03	Gm20472	JUC1200005842.mm.1	-3,33	0,020872	0,479521		
TC0200005503.mm.1	-1,05	Ell3	JUC0200019613.mm.1	-3,33	0,009762	0,417839		
TC1000001081.mm.1	1,15	Sycp3	JUC1000004392.mm.1	-3,33	0,047067	0,557783		
TC1000000843.mm.1	2,67	Cirbp	JUC1000003442.mm.1	-3,33	0,013598	0,443703		
TC1000000843.mm.1	2,67	Cirbp	JUC1000003445.mm.1	-8,23	0,047636	0,558743		
TC0500002250.mm.1	1,45	Trmt44	PSR0500020616.mm.1	-3,34	0,012661	0,43631	Alternative 3' Acce	0,31
TC1500000865.mm.1	1,24	Ppp6r2	JUC1500003828.mm.1	-3,34	0,023833	0,491992		
TC0500001678.mm.1	1,06	Uncx	JUC0500008403.mm.1	-3,34	0,031972	0,518183		
TC0600003164.mm.1	1,96	Kcna6	JUC0600012847.mm.1	-3,34	0,018356	0,468408		
TC0600003164.mm.1	1,96	Kcna6	JUC0600012846.mm.1	-3,65	0,005857	0,383747		
TC1000002385.mm.1	1,24	Gstt2	JUC1000009321.mm.1	-3,34	0,041997	0,546079		
TC0400003106.mm.1	1,68	E130114P18Rik	JUC0400012935.mm.1	-3,34	0,039619	0,540064		
TC0200002838.mm.1	2,06		JUC0200011941.mm.1	-3,35	0,007543	0,401384		
TC0200002838.mm.1	2,06		PSR0200023565.mm.1	-3,87	0,006063	0,386691	Cassette Exon	0,38
TC0200002838.mm.1	2,06		PSR0200023566.mm.1	-4,49	0,025199	0,49668	Cassette Exon	0,38
TC1100000371.mm.1	1,2		PSR1100003078.mm.1	-3,35	0,027112	0,503022	Cassette Exon	0,22
TC1200000669.mm.1	3,24		PSR1200004592.mm.1	-3,35	0,016204	0,458277	Cassette Exon	0,22
TC1500001887.mm.1	1,48	Npcd; Cbx6; Nptxr	JUC1500008469.mm.1	-3,35	0,00479	0,37131		
TC0900000340.mm.1	1,48	Pate2	JUC0900001374.mm.1	-3,35	0,027289	0,503732		
TC1100003986.mm.1	4,25	Rgs9	PSR1100037290.mm.1	-3,36	0,020489	0,478019	Cassette Exon	0,25
TC1100003986.mm.1	4,25	Rgs9	JUC1100019478.mm.1	-3,54	0,007435	0,399934		
TC1100003986.mm.1	4,25	Rgs9	PSR1100037322.mm.1	-4,28	0,026463	0,501252	Alternative 5' Donc	0,48
TC1100003986.mm.1	4,25	Rgs9	PSR1100037291.mm.1	-4,82	0,005421	0,379269	Cassette Exon	0,41
TC1100003986.mm.1	4,25	Rgs9	PSR1100037301.mm.1	-5,71	0,002116	0,342919	Cassette Exon	0,27
TC1100003986.mm.1	4,25	Rgs9	JUC1100019477.mm.1	-5,99	0,006754	0,393343		
TC1100003986.mm.1	4,25	Rgs9	PSR1100037289.mm.1	-6,44	0,008207	0,405909	Cassette Exon	0,25
TC1100003063.mm.1	-1,23	Chrnb1	JUC1100014730.mm.1	-3,36	0,02572	0,498173		
TC0700004625.mm.1	2,12	Kctd13	PSR0700039157.mm.1	-3,37	0,009128	0,413316	Alternative 3' Acce	0,39
TC1200000521.mm.1	1,34	Gm527	JUC1200001993.mm.1	-3,37	0,001131	0,317328		
TC0100000994.mm.1	1,37		JUC0100004778.mm.1	-3,37	0,006469	0,390467		
TC1700000743.mm.1	1	H2-M10.5	JUC1700004075.mm.1	-3,37	0,047629	0,558743		
TC0300002742.mm.1	1,34	Sars	PSR0300021571.mm.1	-3,38	0,039535	0,539764	Alternative 5' Donc	0,38
TC0900001399.mm.1	1,3	Amt	PSR0900011234.mm.1	-3,38	0,04706	0,557783	Alternative 5' Donc	0,38
TC1300000983.mm.1	1,25	Tmem161b	PSR1300006343.mm.1	-3,38	0,015238	0,452847	Alternative 3' Acce	0,38
TC0700001686.mm.1	2,19		PSR0700014051.mm.1	-3,38	0,021734	0,484227	Alternative 5' Donc	0,38
TC1400001394.mm.1	1,01	Gm8246	JUC1400005599.mm.1	-3,38	0,041279	0,544166		
TC0300000446.mm.1	1,02		JUC0300001604.mm.1	-3,38	0,002366	0,34829		
TC0100001223.mm.1	-1,42	Mybph	JUC0100005776.mm.1	-3,39	0,028399	0,507058		
TC0600000914.mm.1	-1,26	Loxl3	JUC0600003439.mm.1	-3,39	0,015903	0,456586		
TC1400000861.mm.1	1,35		PSR1400007029.mm.1	-3,4	0,000574	0,304044	Alternative 3' Acce	0,38
TC0900002262.mm.1	1,15		PSR0900018832.mm.1	-3,4	0,039669	0,540305	Cassette Exon	0,23
TC0X00000701.mm.1	2,11	Rpl3-ps2	PSR0X00004629.mm.1	-3,41	0,00176	0,335996	Alternative 5' Donc	0,32
TC0500002486.mm.1	-1,03	Ugdh	JUC0500012081.mm.1	-3,41	0,005799	0,383104		
TC0500002284.mm.1	-1,21	Slc2a9	PSR0500021092.mm.1	-3,42	0,007866	0,403545	Alternative 5' Donc	0,38

TC1400001401.mm.1	1,2	Gm3424	JUC1400005618.mm.1	-3,42	0,045136	0,553287		
TC0400003991.mm.1	-1,2		JUC0400017173.mm.1	-3,42	0,008556	0,408183		
TC1200000606.mm.1	1,41	2700049A03Rik	JUC1200002332.mm.1	-3,43	0,031822	0,517753		
TC1800001474.mm.1	1,07	LOC102631870	JUC1800006074.mm.1	-3,43	0,021639	0,483643		
TC0600000608.mm.1	1,69	Cbx3; Gm6917; Gm6901; (	JUC0600002358.mm.1	-3,43	0,047015	0,557766		
TC0200005326.mm.1	1,34		JUC0200023373.mm.1	-3,44	0,005636	0,381541		
TC1500000627.mm.1	-1,01	Apol10a	JUC1500002614.mm.1	-3,44	0,005725	0,382767		
TC1700000410.mm.1	1,51	Luc7l	JUC1700001893.mm.1	-3,44	0,022467	0,487042		
TC0200004613.mm.1	1,44		PSR0200039471.mm.1	-3,45	0,028286	0,506588	Cassette Exon	0,23
TC1000001691.mm.1	1,59	Ipcef1	JUC1000006699.mm.1	-3,45	0,008721	0,409318		
TC1800000257.mm.1	1,81	Map3k2	PSR1800001820.mm.1	-3,46	0,027525	0,504313	Alternative 5' Donc	0,4
TC0600002159.mm.1	-1,22	Trpv6	JUC0600008928.mm.1	-3,46	0,015634	0,455102		
TC0800001805.mm.1	1,25	Defb39	PSR0800014399.mm.1	-3,47	0,008645	0,408463	Alternative 3' Acce	0,39
TC0100003537.mm.1	1,21	Usp21	JUC0100016513.mm.1	-3,47	0,012232	0,433601		
TC0900001366.mm.1	1,33	6430571L13Rik	JUC0900005860.mm.1	-3,47	0,014375	0,448307		
TC1400000130.mm.1	1,34	Top2b	PSR1400001061.mm.1	-3,48	0,00284	0,352207	Alternative 3' Acce	0,4
TC1600001560.mm.1	-1,08		JUC1600006675.mm.1	-3,48	0,000607	0,304044		
TC1400001451.mm.1	1,65	Gm3839	JUC1400005865.mm.1	-3,49	0,000715	0,306015		
TC1700002281.mm.1	-1,22	Efhb	JUC1700011391.mm.1	-3,49	0,042239	0,547034		
TC1800001505.mm.1	1,18	Afg3l2	JUC1800006157.mm.1	-3,49	0,014204	0,447701		
TC1800001505.mm.1	1,18	Afg3l2	JUC1800006154.mm.1	-3,77	0,000255	0,28803		
TC1700000081.mm.1	-1,09	Fgfr1op	JUC1700000358.mm.1	-3,49	0,014246	0,447947		
TC0300000200.mm.1	-1,14	Lrriq4	JUC0300000522.mm.1	-3,5	0,018367	0,468484		
TC0100000244.mm.1	1,67	Cnnm4	JUC0100000908.mm.1	-3,5	0,027639	0,504658		
TC1400000392.mm.1	5,02	Lrit1	PSR1400003488.mm.1	-3,51	0,006238	0,388361	Cassette Exon	0,37
TC1400000392.mm.1	5,02	Lrit1	JUC1400002073.mm.1	-5,8	0,000935	0,313363		
TC1500000852.mm.1	1,18	Creld2	JUC1500003747.mm.1	-3,51	0,014336	0,448173		
TC1700002769.mm.1	1,52	Gm3435; 9030025P20Rik	JUC1700000763.mm.1	-3,51	0,027363	0,503888		
TC0500002823.mm.1	1,09	A830083F22	JUC0500013480.mm.1	-3,51	0,02909	0,509063		
TC0500002828.mm.1	1,51	Hnrnpdl; Hnrpdl	PSR0500024888.mm.1	-3,52	0,016422	0,45898	Alternative 5' Donc	0,36
TC0500000349.mm.1	3,4	Ywhah	JUC0500001811.mm.1	-3,52	0,019588	0,474672		
TC0300000477.mm.1	1,31	Gm16527; 4930449A18Ril	PSR0300003303.mm.1	-3,53	0,028315	0,506741	Cassette Exon	0,23
TC1900001465.mm.1	1,17	Fra10ac1	JUC1900007004.mm.1	-3,53	0,046564	0,556761		
TC0200001889.mm.1	1,19	Galk2	JUC0200007377.mm.1	-3,53	0,041124	0,543963		
TC1500001950.mm.1	1,56		JUC1500008696.mm.1	-3,54	0,026883	0,502518		
TC1500001950.mm.1	1,56		JUC1500008695.mm.1	-4,15	0,022504	0,487114		
TC1500001981.mm.1	1,18	Efcab6	JUC1500008907.mm.1	-3,54	0,013258	0,441506		
TC0500002322.mm.1	2,02		JUC0500011664.mm.1	-3,54	0,002193	0,344674		
TC1900001117.mm.1	-1,28	Zp1	JUC1900005735.mm.1	-3,55	0,008214	0,405934		
TC0300000231.mm.1	1,01	4930419G24Rik	JUC0300000716.mm.1	-3,56	0,000344	0,289576		
TC0900001663.mm.1	1,08	Lars2	PSR0900013967.mm.1	-3,57	0,007819	0,403089	Alternative 3' Acce	0,4
TC1000002866.mm.1	-1,36	1700020G17Rik	PSR1000021674.mm.1	-3,57	0,000152	0,272178	Cassette Exon	0,24
TC1700002757.mm.1	1,23	Gm1976	JUC1700013311.mm.1	-3,58	0,02499	0,496025		
TC0X00001421.mm.1	1,02	Alg13	JUC0X00004481.mm.1	-3,58	0,018267	0,467999		
TC0600001711.mm.1	-1,33	Gm15543	JUC0600007140.mm.1	-3,59	0,001978	0,340886		
TC0300000752.mm.1	1,07	Thbs3	JUC0300002995.mm.1	-3,6	0,004658	0,370181		
TC0100000628.mm.1	1,44		JUC0100002942.mm.1	-3,6	0,011712	0,429969		
TC1500000053.mm.1	1,06	Nup155	JUC1500000296.mm.1	-3,6	0,04253	0,547849		

TC0700004169.mm.1	1,11		JUC0700018242.mm.1	-3,6	0,049182	0,562402		
TC0X00001075.mm.1	-1,42	Gm5127	JUC0X00003506.mm.1	-3,61	0,047365	0,558302		
TC0200002857.mm.1	1,08	1700080N15Rik	JUC0200012000.mm.1	-3,63	0,047075	0,557792		
TC1200002120.mm.1	1,19	Vipas39	JUC1200008118.mm.1	-3,63	0,010395	0,421426		
TC1200000418.mm.1	1,51	Coch	JUC1200001657.mm.1	-3,64	0,045112	0,553206		
TC1100000686.mm.1	1,5	Zfp692	JUC1100002851.mm.1	-3,65	0,014263	0,447947		
TC1100002497.mm.1	-1,11	Dock2	PSR1100023309.mm.1	-3,67	0,013625	0,444111	Cassette Exon	0,33
TC0700004104.mm.1	1,48	Lyve1	JUC0700017862.mm.1	-3,67	0,005374	0,378731		
TC0600003201.mm.1	1,07	Rhno1	PSR0600024938.mm.1	-3,68	0,015343	0,453763	Alternative 3' Acce	0,18
TC0X00001945.mm.1	1,27		PSR0X00012372.mm.1	-3,69	0,008967	0,411742	Cassette Exon	0,25
TC1800000757.mm.1	1,15	4930448D08Rik	JUC1800003042.mm.1	-3,69	0,033738	0,523558		
TC0900000255.mm.1	1,28	Ncapd3	JUC0900001044.mm.1	-3,69	0,002209	0,345614		
TC0200005485.mm.1	1,56	Sys1	PSR0200020518.mm.1	-3,7	0,028599	0,507685	Cassette Exon	0,25
TC1400002763.mm.1	3,9		PSR1400020665.mm.1	-3,72	0,00367	0,357266	Alternative 3' Acce	0,43
TC0200002850.mm.1	1,77	Suv39h2	JUC0200011977.mm.1	-3,72	0,025487	0,497664		
TC0600000145.mm.1	-1,14	Cftr	JUC0600000506.mm.1	-3,73	0,038602	0,536862		
TC1300001037.mm.1	1,21	Mtx3	JUC1300003464.mm.1	-3,74	0,037112	0,532942		
TC1300000074.mm.1	1,51		JUC1300000319.mm.1	-3,76	0,006811	0,393827		
TC1900000904.mm.1	6,45	Cabp4	PSR1900007841.mm.1	-3,78	0,000939	0,313363	Cassette Exon	0,24
TC1900000904.mm.1	6,45	Cabp4	JUC1900004296.mm.1	-4,31	0,001687	0,335192		
TC1900000565.mm.1	-1,21	Cyp2c29; Cyp2c53-ps	JUC1900002726.mm.1	-3,78	0,022911	0,488524		
TC0X00003188.mm.1	-1,11		JUC0X00010116.mm.1	-3,79	0,015352	0,453763		
TC0700003593.mm.1	1,09	Fes	JUC0700016060.mm.1	-3,8	0,00696	0,395722		
TC0700003593.mm.1	1,09	Fes	JUC0700016074.mm.1	-3,93	0,000933	0,313363		
TC1400002025.mm.1	1,24	Supt16	JUC1400008293.mm.1	-3,81	0,016397	0,458814		
TC1500000842.mm.1	-1,04		JUC1500003724.mm.1	-3,81	0,038171	0,535805		
TC1000002571.mm.1	1,1	Glt8d2	JUC1000010583.mm.1	-3,81	0,000894	0,313363		
TC1000002274.mm.1	1,41	Hnrnph3; D10Bwg1070e	JUC1000008942.mm.1	-3,81	0,029171	0,509333		
TC0500001753.mm.1	1,2	Dmr	JUC0500008775.mm.1	-3,83	0,011404	0,427549		
TC0600001245.mm.1	1,09	Brpf1	PSR0600009626.mm.1	-3,84	0,03282	0,520896	Alternative 5' Donc	0,3
TC0500001918.mm.1	2,49	Zfp804b	JUC0500009607.mm.1	-3,84	0,004747	0,371039		
TC0900002696.mm.1	1,09	Tinag	JUC0900012466.mm.1	-3,84	0,002163	0,344238		
TC0300001026.mm.1	-1,1		PSR0300008240.mm.1	-3,85	0,0386	0,536862	Cassette Exon	0,26
TC1600000398.mm.1	1,02		JUC1600001955.mm.1	-3,85	0,023603	0,490995		
TC1100000751.mm.1	1,37	Lrrc48	PSR1100005927.mm.1	-3,86	0,028986	0,508806	Alternative 3' Acce	0,44
TC1400000135.mm.1	1,2	Thrb	JUC1400000707.mm.1	-3,86	0,026558	0,501513		
TC0800001492.mm.1	1,05		JUC0800006629.mm.1	-3,86	0,030723	0,514036		
TC0Y00000014.mm.1	1,43		JUC0Y00000121.mm.1	-3,86	0,00122	0,322251		
TC1500001105.mm.1	1,17	Tarbp2	JUC1500005052.mm.1	-3,87	0,002736	0,349612		
TC0500001618.mm.1	1,35	Vgf	JUC0500007984.mm.1	-3,88	0,005839	0,383458		
TC1700001636.mm.1	2,46	Syngr3	PSR1700014646.mm.1	-3,89	0,004109	0,3623	Cassette Exon	0,2
TC1700001636.mm.1	2,46	Syngr3	PSR1700014648.mm.1	-3,98	0,008175	0,40565	Alternative 5' Donc	0,41
TC1400000397.mm.1	1,07		JUC1400002085.mm.1	-3,89	0,01559	0,454982		
TC0X00002820.mm.1	1,16	Atrx	JUC0X00009000.mm.1	-3,9	0,047521	0,558686		
TC0100003796.mm.1	1,13	Vash2	JUC0100017621.mm.1	-3,93	0,014974	0,452048		
TC1600001702.mm.1	-1,01		JUC1600007240.mm.1	-3,95	0,018432	0,468646		
TC0700002830.mm.1	1,25	4931406P16Rik	JUC0700013536.mm.1	-3,95	0,015279	0,453159		
TC1000000216.mm.1	1,21	Med23; Gm23410	PSR1000001554.mm.1	-3,96	0,025513	0,497664	Alternative 3' Acce	0,28

TC1100003839.mm.1	1,24	Itga2b	JUC1100018731.mm.1	-3,96	0,005458	0,379939		
TC0800001710.mm.1	-1,29		JUC0800007548.mm.1	-3,96	0,032384	0,519221		
TC1100002218.mm.1	-1,35	Zpbp	JUC1100011249.mm.1	-3,97	0,012244	0,433659		
TC0900003009.mm.1	1,59	Abhd14a	JUC0900014022.mm.1	-3,97	0,00057	0,304044		
TC1600001585.mm.1	1,03		JUC1600006745.mm.1	-3,99	0,000332	0,28927		
TC0X00002669.mm.1	1,8	Zc4h2	JUC0X00008473.mm.1	-4	0,028498	0,507345		
TC0200002332.mm.1	-1,31	Bpifa2	JUC0200009311.mm.1	-4,01	0,025438	0,497549		
TC1600001647.mm.1	1,38		JUC1600007024.mm.1	-4,01	0,002264	0,34578		
TC1000001340.mm.1	1,3	5330438D12Rik	JUC1000005449.mm.1	-4,04	0,018482	0,46874		
TC1300001199.mm.1	6,87		PSR1300007627.mm.1	-4,06	0,004719	0,370888	Cassette Exon	0,41
TC1300001199.mm.1	6,87		JUC1300003981.mm.1	-4,97	0,011728	0,429969		
TC1300001199.mm.1	6,87		PSR1300007628.mm.1	-5,22	0,000904	0,313363	Cassette Exon	0,1
TC0100002560.mm.1	-1,07		JUC0100011693.mm.1	-4,06	0,003535	0,3562		
TC0100003470.mm.1	-1,05		JUC0100016231.mm.1	-4,07	0,000147	0,272178		
TC0600003563.mm.1	1,24	Igkv4-63	JUC0600009736.mm.1	-4,07	0,044564	0,551921		
TC1100001049.mm.1	1,09	Mettl16	JUC1100004987.mm.1	-4,07	0,047078	0,557794		
TC1600001539.mm.1	1,15	1700119H24Rik	JUC1600006598.mm.1	-4,08	0,044448	0,551654		
TC1500002027.mm.1	-1,05		JUC1500009100.mm.1	-4,09	0,015796	0,455748		
TC0100001611.mm.1	3,37	Kcnj10	PSR0100013399.mm.1	-4,1	0,03999	0,541013	Intron Retention	0,74
TC0100001611.mm.1	3,37	Kcnj10	PSR0100013396.mm.1	-4,64	0,049325	0,562746	Alternative 3' Acce	0,31
TC0100001611.mm.1	3,37	Kcnj10	JUC0100007572.mm.1	-5,04	0,013578	0,443703		
TC1200001998.mm.1	1,03		JUC1200007618.mm.1	-4,1	0,034743	0,526574		
TC1300001619.mm.1	-1,07	4930557F10Rik	JUC1300005156.mm.1	-4,1	0,045625	0,55464		
TC0X00000941.mm.1	1,02	Eda	JUC0X00002991.mm.1	-4,11	0,02081	0,479505		
TC1700000389.mm.1	6,04	Fbxl16	PSR1700003263.mm.1	-4,12	0,015986	0,456946	Cassette Exon	0,32
TC1700000389.mm.1	6,04	Fbxl16	JUC1700001784.mm.1	-4,2	0,018745	0,470733		
TC1700000389.mm.1	6,04	Fbxl16	PSR1700003262.mm.1	-5,89	0,008891	0,410717	Cassette Exon	0,41
TC0700003137.mm.1	7,92		JUC0700015079.mm.1	-4,12	0,010553	0,422341		
TC0700003137.mm.1	7,92		JUC0700015077.mm.1	-7,87	0,013861	0,445426		
TC1000002280.mm.1	1,27	Sirt1	PSR1000016362.mm.1	-4,13	0,040821	0,543283	Alternative 3' Acce	0,48
TC1100000113.mm.1	-1,22		JUC1100000611.mm.1	-4,14	0,011367	0,427487		
TC1100001551.mm.1	2,54	B230217C12Rik	PSR1100014320.mm.1	-4,15	0,037491	0,533968	Alternative 3' Acce	0,44
TC1700001858.mm.1	-1,02	Cyp4f41-ps	JUC1700009305.mm.1	-4,17	0,006715	0,393343		
TC0600000675.mm.1	-1,29		JUC0600002643.mm.1	-4,17	0,010173	0,419711		
TC1100002210.mm.1	-1,08	Pkd1l1	JUC1100011195.mm.1	-4,17	0,046933	0,557521		
TC0400003809.mm.1	1,28	Gm13010	JUC0400016327.mm.1	-4,21	0,025553	0,497808		
TC0200003834.mm.1	3,98	Frzb	PSR0200033232.mm.1	-4,22	0,012247	0,43367	Cassette Exon	0,28
TC0200003834.mm.1	3,98	Frzb	PSR0200033230.mm.1	-4,36	0,006336	0,389463	Cassette Exon	0,32
TC1500000784.mm.1	1,09	Samm50	JUC1500003450.mm.1	-4,22	0,007363	0,39938		
TC0800000201.mm.1	2,06		JUC0800000959.mm.1	-4,22	0,010803	0,424055		
TC0700001710.mm.1	1,06	Acsm2	JUC0700007392.mm.1	-4,24	0,007197	0,39798		
TC0900000106.mm.1	1,17		JUC0900000392.mm.1	-4,26	0,00083	0,311886		
TC1800001515.mm.1	1,38	Gm26910	JUC1800006210.mm.1	-4,28	0,018601	0,469657		
TC0800000196.mm.1	1,81	4930467E23Rik; Gm2111	JUC0800000930.mm.1	-4,32	0,002148	0,343679		
TC1500001026.mm.1	1,48	Gm17057	JUC1500004578.mm.1	-4,34	0,000174	0,278719		
TC1100001504.mm.1	1,13	Skap1	PSR1100013907.mm.1	-4,36	0,027336	0,503766	Cassette Exon	0,28
TC1100001668.mm.1	6,03	Arl4d	PSR1100015687.mm.1	-4,39	0,000001	0,179072	Cassette Exon	0,3
TC0200003763.mm.1	1,15	Atf2	PSR0200032189.mm.1	-4,4	0,0178	0,465641	Alternative 3' Acce	0,1

TC0X00001201.mm.1	1,42	3110007F17Rik	JUC0X00003724.mm.1	-4,43	0,004064	0,361956		
TC1800001149.mm.1	1,4	Fam13b	JUC1800004679.mm.1	-4,44	0,00341	0,355242		
TC0X00000527.mm.1	-1,35	Gpr112	JUC0X00001783.mm.1	-4,44	0,030179	0,512365		
TC0X00002807.mm.1	1,19	5530601H04Rik; LOC1005	PSR0X00017700.mm.1	-4,45	0,038067	0,535633	Alternative 3' Acce	0,47
TC1500000844.mm.1	1,1	Fam19a5	JUC1500003728.mm.1	-4,45	0,003659	0,357266		
TC1500001092.mm.1	3,63	Gm9918	PSR1500008709.mm.1	-4,48	0,006159	0,387873	Cassette Exon	0,28
TC1500001092.mm.1	3,63	Gm9918	JUC1500004948.mm.1	-5,04	0,029278	0,509874		
TC1500001092.mm.1	3,63	Gm9918	JUC1500004949.mm.1	-9,27	0,001782	0,336311		
TC1300001674.mm.1	9,08	Nrsn1	JUC1300005339.mm.1	-4,52	0,010479	0,421891		
TC1300001674.mm.1	9,08	Nrsn1	PSR1300010428.mm.1	-4,59	0,001412	0,326543	Alternative 3' Acce	0,46
TC1300001674.mm.1	9,08	Nrsn1	PSR1300010436.mm.1	-6,55	0,002242	0,34578	Cassette Exon	0,43
TC1300001674.mm.1	9,08	Nrsn1	PSR1300010432.mm.1	-7,39	0,006747	0,393343	Cassette Exon	0,21
TC1300001674.mm.1	9,08	Nrsn1	PSR1300010440.mm.1	-7,95	0,004313	0,364152	Cassette Exon	0,41
TC1300001674.mm.1	9,08	Nrsn1	PSR1300010437.mm.1	-8,92	0,002102	0,342919	Cassette Exon	0,43
TC1300001674.mm.1	9,08	Nrsn1	PSR1300010434.mm.1	-12,46	0,000487	0,298999	Cassette Exon	0,41
TC1300001674.mm.1	9,08	Nrsn1	JUC1300005341.mm.1	-15,58	0,000611	0,304044		
TC1300001674.mm.1	9,08	Nrsn1	JUC1300005342.mm.1	-17,13	0,001754	0,335996		
TC1800001319.mm.1	-1	Dtwd2	JUC1800005424.mm.1	-4,53	0,000868	0,311909		
TC1500001817.mm.1	-1,05	Apol11b	JUC1500008042.mm.1	-4,53	0,024581	0,494362		
TC1100004056.mm.1	-1,29	Gm11709	JUC1100019954.mm.1	-4,54	0,012557	0,435731		
TC1200001347.mm.1	-1,07	Dnmt3aos; Gm10485	JUC1200005329.mm.1	-4,55	0,019068	0,472031		
TC0800000130.mm.1	-1,06	Cdc16	JUC0800000700.mm.1	-4,57	0,000255	0,28803		
TC0X00002476.mm.1	1,4	Mtcp1	JUC0X00008073.mm.1	-4,57	0,014454	0,44909		
TC0100002331.mm.1	-1,11	1700019A02Rik	JUC0100010599.mm.1	-4,59	0,011473	0,428022		
TC1600001007.mm.1	1,28	Gm15964	PSR1600007803.mm.1	-4,62	0,048981	0,561835	Alternative 3' Acce	0,5
TC1500001198.mm.1	1,06	Spef2	JUC1500005445.mm.1	-4,63	0,019256	0,473074		
TC1500000041.mm.1	1,46	Rictor	JUC1500000207.mm.1	-4,63	0,013185	0,440686		
TC0700004431.mm.1	1,03	Adam12; A130023I24Rik;	JUC0700019643.mm.1	-4,64	0,034448	0,525886		
TC1200000927.mm.1	8,29		PSR1200006642.mm.1	-4,65	0,016603	0,459787	Alternative 5' Donc	0,48
TC0800002433.mm.1	6,08	Nxnl1	PSR0800018572.mm.1	-4,66	0,020738	0,479172	Alternative 5' Donc	0,48
TC0800002433.mm.1	6,08	Nxnl1	JUC0800010101.mm.1	-5,18	0,019369	0,473624		
TC0800002433.mm.1	6,08	Nxnl1	JUC0800010100.mm.1	-5,55	0,040354	0,541949		
TC0800002433.mm.1	6,08	Nxnl1	JUC0800010102.mm.1	-8,43	0,011804	0,430357		
TC1900001081.mm.1	1,69	Asrgl1	JUC1900005512.mm.1	-4,66	0,005306	0,378059		
TC0400000001.mm.1	-1,05		JUC0400000005.mm.1	-4,66	0,012067	0,432522		
TC0400000001.mm.1	-1,05		JUC0400000002.mm.1	-4,69	0,008441	0,406611		
TC1500001022.mm.1	1,28	Smarcd1	JUC1500004560.mm.1	-4,67	0,026906	0,502518		
TC0900000938.mm.1	1,33	Sltm	JUC0900003945.mm.1	-4,67	0,031964	0,518173		
TC0X00001679.mm.1	1,15	Gemin8	JUC0X00005468.mm.1	-4,75	0,002832	0,352207		
TC0200003697.mm.1	2,58	Spc25	PSR0200031542.mm.1	-4,76	0,038267	0,536002	Alternative 5' Donc	0,48
TC0100001967.mm.1	1,27	Slco5a1	JUC0100009145.mm.1	-4,82	0,007852	0,403345		
TC0200001101.mm.1	-1,08	Hoxd1	JUC0200004549.mm.1	-4,85	0,023313	0,490065		
TC0X00001587.mm.1	1,35	Gm15155	JUC0X00005048.mm.1	-4,86	0,044475	0,551693		
TC1300000602.mm.1	1,51	Secisbp2	JUC1300001956.mm.1	-4,94	0,042639	0,548097		
TC1300002188.mm.1	1,76	Ptch1	JUC1300007310.mm.1	-4,98	0,009346	0,415092		
TC0500001439.mm.1	10,75	Gm16338	JUC0500007068.mm.1	-4,99	0,009915	0,418378		
TC0500001439.mm.1	10,75	Gm16338	JUC0500007067.mm.1	-7,6	0,025906	0,498985		
TC0500001439.mm.1	10,75	Gm16338	PSR0500012904.mm.1	-10,05	0,007752	0,402774	Cassette Exon	0,28

TC0200002437.mm.1	5,54	Slc32a1	PSR0200019809.mm.1	-5,03	0,012653	0,43631	Cassette Exon	0,28
TC0200002437.mm.1	5,54	Slc32a1	JUC0200010038.mm.1	-9,59	0,011992	0,432286		
TC0200002295.mm.1	1,69	H13	JUC0200009013.mm.1	-5,03	0,04907	0,562033		
TC1100001998.mm.1	1,28	Ccdc40	JUC1100009882.mm.1	-5,03	0,037151	0,533065		
TC0100000654.mm.1	1,3	Ankzf1	JUC0100003155.mm.1	-5,06	0,037352	0,533573		
TC0700002768.mm.1	1,09	Kmt2b	JUC0700013160.mm.1	-5,06	0,013505	0,443238		
TC0900000212.mm.1	-1,38	Cnn1	JUC0900000863.mm.1	-5,09	0,012568	0,435803		
TC0700003163.mm.1	7,77		JUC0700015084.mm.1	-5,13	0,017202	0,462491		
TC0700003163.mm.1	7,77		JUC0700015085.mm.1	-9,29	0,015162	0,452699		
TC1900000665.mm.1	-1,36	Kazald1	PSR1900005884.mm.1	-5,22	0,019569	0,474672	Cassette Exon	0,26
TC1300002119.mm.1	-1,31	Golm1	JUC1300007026.mm.1	-5,22	0,01407	0,446841		
TC0400001039.mm.1	-1,32	Lepr	JUC0400003622.mm.1	-5,26	0,034434	0,525858		
TC1800000741.mm.1	1,13	Tcf4	JUC1800003021.mm.1	-5,32	0,022493	0,487114		
TC0200002375.mm.1	1,63	Mmp24	JUC0200009616.mm.1	-5,32	0,048784	0,561486		
TC0100003045.mm.1	-1,19		JUC0100013859.mm.1	-5,34	0,03069	0,513932		
TC1300000568.mm.1	-1,38	Omd	JUC1300001805.mm.1	-5,5	0,000948	0,313363		
TC0700001005.mm.1	-1,15	Ttc23	JUC0700004759.mm.1	-5,63	0,049096	0,562186		
TC0200004196.mm.1	1,14	Gm10800	JUC0200018012.mm.1	-5,64	0,009319	0,414883		
TC1800000829.mm.1	-1,08	Loxhd1	JUC1800003332.mm.1	-5,7	0,000673	0,304338		
TC0100003891.mm.1	-1,86	Ifi203; LOC100862473; Gm	JUC0100016811.mm.1	-5,81	0,018456	0,468671		
TC0500001971.mm.1	3,6	Gm26954	JUC0500009792.mm.1	-5,82	0,02761	0,504557		
TC0500001971.mm.1	3,6	Gm26954	JUC0500009794.mm.1	-7,69	0,000006	0,179072		
TC0300001022.mm.1	2,83	Nhlh2	PSR0300008209.mm.1	-5,87	0,011251	0,426718	Intron Retention	0,75
TC1100003138.mm.1	1,09	4933427D14Rik	JUC1100015245.mm.1	-5,87	0,00311	0,353892		
TC1500001013.mm.1	-1,07	Tmbim6	PSR1500007987.mm.1	-5,88	0,006141	0,387604	Alternative 5' Donc	0,48
TC1500000317.mm.1	1,37	Zfpm2	JUC1500001330.mm.1	-6,04	0,001519	0,330268		
TC1100003941.mm.1	2,25	Kpna2	JUC1100019349.mm.1	-6,14	0,045387	0,554078		
TC0100000093.mm.1	1,34		JUC0100000380.mm.1	-6,15	0,003047	0,353892		
TC0900000534.mm.1	1,76	Scn2b	JUC0900001886.mm.1	-6,37	0,025545	0,497801		
TC0300002672.mm.1	-1,04	Ddx20	JUC0300010829.mm.1	-6,37	0,012761	0,436997		
TC0400003584.mm.1	11,57	Hpca	JUC0400015221.mm.1	-6,53	0,000287	0,28803		
TC0400003584.mm.1	11,57	Hpca	PSR0400029172.mm.1	-6,87	0,002703	0,349612	Alternative 5' Donc	0,16
TC0400003584.mm.1	11,57	Hpca	PSR0400029169.mm.1	-7,15	0,004239	0,363295	Alternative 5' Donc	0,46
TC0400003584.mm.1	11,57	Hpca	PSR0400029173.mm.1	-7,36	0,000263	0,28803	Cassette Exon	0,21
TC0400003584.mm.1	11,57	Hpca	JUC0400015220.mm.1	-7,75	0,001199	0,321568		
TC0400003584.mm.1	11,57	Hpca	JUC0400015223.mm.1	-7,88	0,004892	0,372195		
TC0400003584.mm.1	11,57	Hpca	PSR0400029184.mm.1	-10,11	0,00328	0,354243	Cassette Exon	0,28
TC0400003584.mm.1	11,57	Hpca	PSR0400029178.mm.1	-12,23	0,003893	0,359372	Cassette Exon	0,21
TC0400003584.mm.1	11,57	Hpca	PSR0400029171.mm.1	-12,23	0,000665	0,304044	Alternative 5' Donc	0,16
TC0400003584.mm.1	11,57	Hpca	PSR0400029182.mm.1	-12,99	0,003941	0,359922	Cassette Exon	0,41
TC0400003584.mm.1	11,57	Hpca	PSR0400029183.mm.1	-13,2	0,001675	0,335182	Cassette Exon	0,28
TC0400003584.mm.1	11,57	Hpca	JUC0400015227.mm.1	-13,36	0,004757	0,371039		
TC0400003584.mm.1	11,57	Hpca	PSR0400029177.mm.1	-13,9	0,002479	0,349135	Cassette Exon	0,34
TC0400003584.mm.1	11,57	Hpca	JUC0400015225.mm.1	-16,34	0,003243	0,354243		
TC0400003584.mm.1	11,57	Hpca	PSR0400029179.mm.1	-17,45	0,001897	0,338712	Cassette Exon	0,21
TC0100000027.mm.1	-1,04	Pcmtd1	JUC0100000133.mm.1	-6,92	0,001034	0,316361		
TC1100004206.mm.1	10,54	Pde6g	JUC1100021069.mm.1	-6,94	0,016379	0,458696		
TC1100004206.mm.1	10,54	Pde6g	PSR1100040112.mm.1	-10,07	0,00522	0,377078	Cassette Exon	0,29

TC1400000084.mm.1	-1,21	4930455B14Rik	JUC1400000460.mm.1	-7	0,007319	0,39887		
TC1500001178.mm.1	-1,1	Nipbl	JUC1500005324.mm.1	-7,17	0,027983	0,505748		
TC1200001812.mm.1	12,24		PSR1200012387.mm.1	-7,23	0,012395	0,434939	Cassette Exon	0,28
TC1200000588.mm.1	1,38	Tmx1	JUC1200002208.mm.1	-7,37	0,01443	0,448873		
TC0600002815.mm.1	2,33	A930015G24Rik	JUC0600011338.mm.1	-8,04	0,001939	0,340349		
TC0800002544.mm.1	1,56	Zfp330	JUC0800010525.mm.1	-8,26	0,009954	0,41841		
TC1900001077.mm.1	5,66	Rom1	PSR1900010084.mm.1	-8,29	0,006828	0,393893	Alternative 5' Donc	0,48
TC0200002017.mm.1	4,29		PSR0200016047.mm.1	-8,36	0,028894	0,50858	Cassette Exon	0,28
TC0800000146.mm.1	1,26	Cln8	JUC0800000767.mm.1	-8,82	0,043106	0,548593		
TC0200004213.mm.1	1,49	Trim44	JUC0200018067.mm.1	-9,83	0,001458	0,328465		
TC0900001143.mm.1	2,23		JUC0900004881.mm.1	-10,55	0,01174	0,429995		
TC1100003551.mm.1	1,06	Utp18	JUC1100016901.mm.1	-12,58	0,039973	0,540973		
TC0800003224.mm.1	2,11	Zdhhc2	JUC0800001752.mm.1	-13,62	0,032174	0,518761		
TC0700004621.mm.1	1,1	Tarsl2	JUC0700004606.mm.1	-13,65	0,00269	0,349612		
TC1400000393.mm.1	8,76	Lrit2	JUC1400002077.mm.1	-15,03	0,000492	0,299146		
